# Genome-Wide Analysis and Functional Characterization of the Polyadenylation Site in Pigs Using RNAseq Data

**DOI:** 10.1038/srep36388

**Published:** 2016-11-04

**Authors:** Hongyang Wang, Rui Li, Xiang Zhou, Liyao Xue, Xuewen Xu, Bang Liu

**Affiliations:** 1 Key Laboratory of Agricultural Animal Genetics, Breeding and Reproduction of Ministry of Education & Key Laboratory of Pig Genetics and Breeding of Ministry of Agriculture; Huazhong Agricultural University, Wuhan, China; 2 The Cooperative Innovation Center for Sustainable Pig Production, Wuhan, China; 3 Department of Animal Sciences, Washington State University, Pullman, WA, United States

## Abstract

Polyadenylation, a critical step in the production of mature mRNA for translation in most eukaryotes, involves cleavage and poly(A) tail addition at the 3′ end of mRNAs at the polyadenylation site (PAS). Sometimes, one gene can have more than one PAS, which can produce the alternative polyadenylation (APA) phenomenon and affect the stability, localization and translation of the mRNA. In this study, we discovered 28,363 PASs using pig RNAseq data, with 13,033 located in 7,403 genes. Among the genes, 41% were identified to have more than one PAS. PAS distribution analysis indicated that the PAS position was highly variable in genes. Additionally, the analysis of RNAseq data from the liver and testis showed a difference in their PAS number and usage. RT-PCR and qRT-PCR were performed to confirm our findings by detecting the expression of 3′UTR isoforms for five candidate genes. The analysis of RNAseq data under a different androstenone level and salmonella inoculation indicated that the functional usage of PAS might participate in the immune response and may be related to the androstenone level in pigs. This study provides new insights into pig PAS and facilitates further functional research of PAS.

In eukaryotes, polyadenylation is an essential step for mRNA maturation and the subsequent execution of gene function. In the process, multiple enzymes first recognize poly(A) signals, such as cleavage/polyadenylation specificity factor (CPSF), cleavage stimulation factor (CstF) and cleavage factor I (CFI), and then cleavage occurs at the 3′ ends of mRNAs^1^,^2^,^3^. Subsequently, poly(A) tail addition modifies mRNA at the cleavage site (also called the polyadenylation site, PAS), catalysed by CPSF, polyadenylated polymerase (PAP), polyadenylated binding protein II (PAB II) and RNA polymerase II (RNAP II)^4^,^5^,^6^. The poly(A) tail in eukaryotic mRNA plays a key role in its stability, translation and transport^7^,^8^,^9^. A short or absent poly(A) tail might lead to the degradation of the transcript and changes in the abundance of gene expression^10^,^11^. However, more than one PAS can exist in protein coding genes and long non-coding RNAs in eukaryotes and produce various transcript isoforms with different lengths^12^ in a process, called alternative polyadenylation (APA), which depends on the PAS number and poly(A) signals^13^,^14^. APA can change the length and composition of 3′UTR and alter its ability to bind with various regulatory elements, such as miRNAs and RNA-binding proteins (RBPs); it can also change the expression of genes^15^. Not surprisingly, APA has been widely reported to be functionally important. Additionally, when PASs are located in introns or ORF regions, APA can also alter the coding sequence (CDS) and produce different proteins for a gene^16^. Recently, many studies have observed the global shortening of 3′UTRs associated with diseases, such as cancer^17^,^18^,^19^, myotonic dystrophy^20^ and oculopharyngeal muscular dystrophy^21^.

APA has been found in pig genes and associated with some important traits. The porcine *GBP1* gene is a negative regulator of the T-cell response, the gene expression of which might be associated with viremia levels and lower weight gain following porcine reproduction and respiratory syndrome virus (PRRSV)^22^. Recently, Gol *et al*. found that a *G* to *A* mutation in the porcine *GBP1* gene was associated with its expression. Interestingly, different alleles of the mutation affected the usage of two PASs in 3′UTR of *GBP1* gene. The distal PAS is more commonly used in all test samples, and the short transcript is generated by the proximal PAS when the *A* allele is present^22^. The study suggested that the APA participates in the formation of different lengths of transcripts and results in various expressions for *GBP1*. To better understand the mechanism of oocyte maturation in pigs, two porcine genes *cyclin B1* and *Cdc2*, the components of maturation/M-phase promotion factor (MPF) and key players in the maturation of mammalian oocytes, were characterized during the *in vitro* maturation (IVM) of pig oocytes^23^. The results showed that two isoforms of the *cyclin B1* gene underwent polyadenylation beginning at approximately 24 hr and continuing to 44 hr of IVM, suggesting that *cyclin B1* was regulated post-transcriptionally by polyadenylation during IVM. Zhang *et al*. also found the *cyclin B1* short isoform, *GDF9* and *C-mos* genes underwent more intensive polyadenylation modification in cyclopiazonic acid treated oocytes than control oocytes, which indicated that polyadenylation might influence Ca(2+)-modulated changes in gene expression^24^. Polyadenylation signals and the expression of the *CYP51* (cytochrome P450, family 51) gene were identified in pigs^25^. The shorter size of the 1.8-kb transcript was generated through the usage of an unusual polyadenylation signal and was only found in the testis, not in the epididymis. The aforementioned observations indicated that APA is commonly present in the pig genome and associated with porcine traits. However, to our knowledge, a genome-wide analysis of the PAS in the pig genome has not yet been conducted.

Since the completion of pig genome sequencing project, a large number of transcriptome analyses have been conducted, making possible a genome-wide analysis of PAS in pigs. In this study, we used currently published transcriptome data containing more than 12 billion reads to identify 28,363 positive PASs in pigs. We annotated pig PASs and analysed the physical position and distribution of PAS in pig genome. Additionally, the differences in PAS number and usage between the liver and testis were also analysed. Finally, using the RNAseq data under a different androstenone levels and salmonella inoculation, we concluded that the functional usage of PAS might participate in the immune response and may be related to the androstenone level in pigs. To the best of our knowledge, this is the first report to reveal pig PAS at the genome-wide level, the PAS distribution in the genome and the functional usage of PAS under these conditions. These results provide new insights into pig PAS and facilitate further functional research of PAS.

## Results

### Polyadenylation sites and annotation

**Figure 1:**
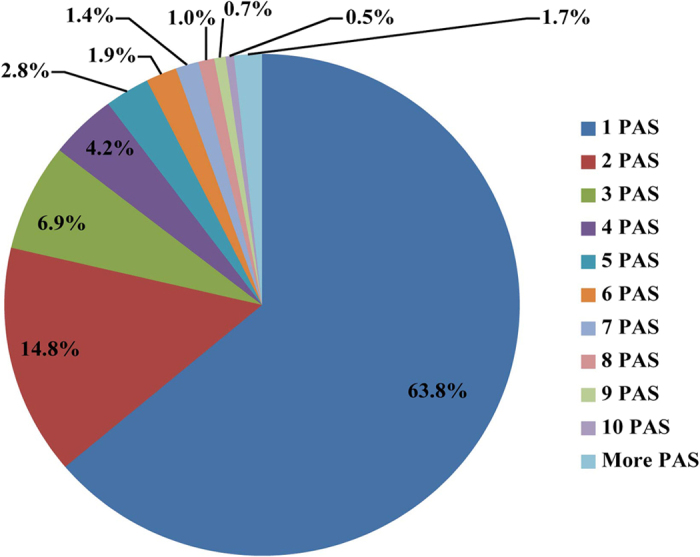
One or more PASs considered as a cluster by separating 20 or less nt.

The pig polyadenylation site (PAS) was discovered using all RNAseq data collected, with a total of 12.2 billion RNAseq reads after filtering out low-quality and short reads using Trimmomatic software^26^. The alignment of raw reads was performed using bowtie2 software, and 4.8 billion reads failed to be aligned to the pig genome (Supplementary Table S1). In the unmapped reads, 38.3 million RNAseq reads were identified as candidate polyadenylated reads starting or ending with at least 10 consecutive A or T with 10% mismatch allowed for either case as described in Materials and Methods. In total, we obtained 13.8 million polyadenylated reads that mapped to the genome when A and T residues were trimmed. After selecting the unique mapping reads and filtering out potential cases of internal priming in the genome, we obtained 1.9 million polyadenylated reads and identified a total of 69,496 putative PASs, which were classified into 28,363 clusters by considering the adjacent PASs (separated by less than 20 bp) as one. The large majority of PASs (63.8%) showed no heterogeneity (Fig. 1). Two PASs and more than two PASs were grouped in 14.8% and less than 22% of cases, respectively. Positive PASs were estimated according to the covered reads for PASs in each cluster (Supplementary Table S2).

Pig PASs were first annotated using the annotated files from the Ensembl database with a total of 30,585 gene transcripts and 21,630 protein coding genes (genes version is *Sus_scrofa.Sscrofa10.2.81*). Among the 28,363 positive PASs described above, 13,033 PASs (47%) were located in a total of 7,403 genes with different gene regions, with 34% pig genes annotated with PAS. Among the 13,033 PASs, 7,900 (61%) were located in 3′UTRs, 3,441 (26%) in introns and 2,187 (17%) in ORFs (Supplementary Table S2). The remaining 15,330 PASs were located in non-annotated gene regions. Additionally, a de novo transcriptome analysis was performed using all RNAseq data. After the intersection between 15,330 PASs and de novo transcripts, we obtained 6,806 PASs (24%) overlapping with the regions of de novo genes (Supplementary Table S2). Therefore, a total of 19,839 (70%) PASs obtained were located in gene regions and 8,524 (30%) PASs in intergenic regions.

### PAS distribution in the pig genome

**Figure 2:**
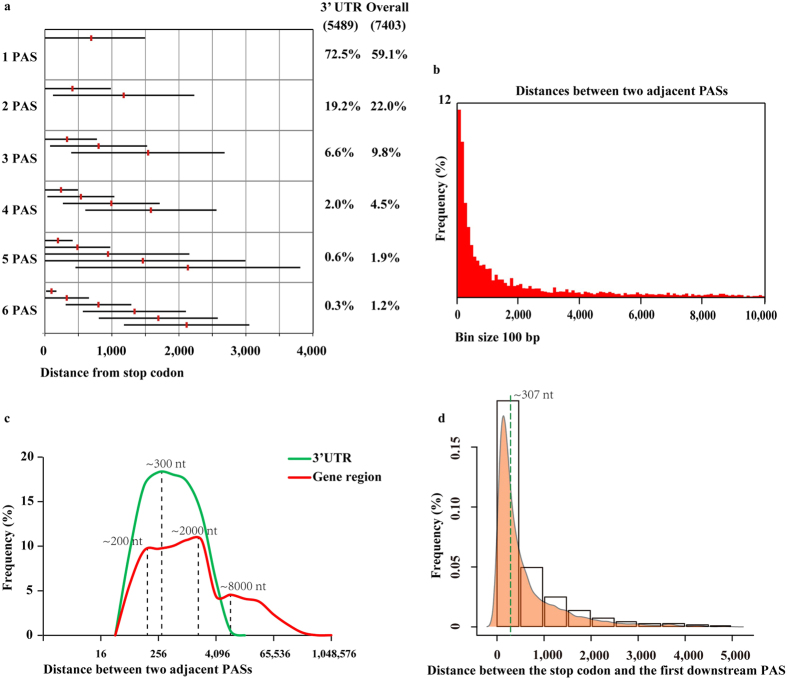
PAS distribution in 3′UTRs and genes. (**a**) The red vertical line represents the average position of PAS relative to the stop codon based on the number and order of PAS. The horizontal error bars correspond to standard deviation. The percentage of genes with a corresponding PAS number in 3′ UTRs, as well as genes are indicated. (**b**) Distribution of distances between two adjacent PASs per gene. (**c**) Frequency of the distance between two adjacent PASs in 3′UTRs (green line) and gene regions (red line). (**d**) Frequency of the distance between the stop codon and the first downstream PAS.

The distribution of PAS in genes was analysed using all PASs. PAS was observed in 3′UTRs and 5,489 (74%) associated genes, 37.5% of which showed alternative polyadenylation, leading to different lengths of 3′UTRs. A total of 40.9% of genes were also identified to have alternative PAS, and we plotted the average position of PAS following the stop codon according to the number of PASs (one to six PASs). The standard deviations displayed that the position of PAS in genes is highly variable (Fig. 2a). We also presented the distance between adjacent PASs per gene, and 45% PASs showed that the distance of two adjacent PASs was less than 1 kb, indicating the high frequency of close PAS (Fig. 2b). Additionally, the genomic distance between adjacent PASs per gene displayed two models in 3′UTRs and genes, with one peak at ~300 nt for 3′UTRs and three peaks at ~200 nt, ~2000 nt and ~8000 nt for genes (Fig. 2c). A similar distribution was observed in the left peak (200–300 nt) for 3′UTRs and genes, and the last two peaks for genes were mainly attributed to introns. When using only the first PAS located in 3′UTRs following the stop codon per gene, the distance between the stop codon and the first downstream PAS had a median value of 307 nt (Fig. 2d).

### The differences of PAS between tissues

**Figure 3:**
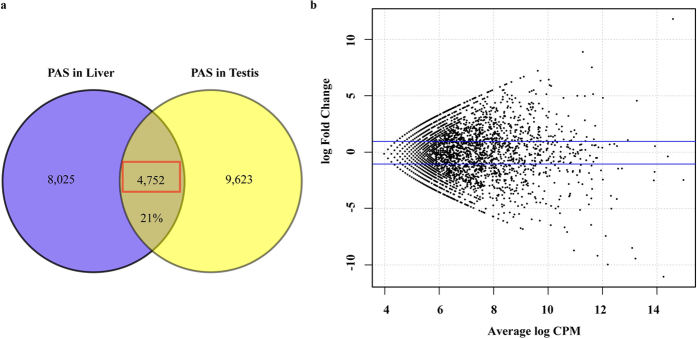
Comparison of PAS number and usage between two tissues. (**a**) The PAS number discovered from the liver and testis and the common and specific PAS numbers were shown. (**b**) The different usages of common PAS from (**a**) were analysed. CPM means counts per million reads. Y axis means higher or lower usage of PAS discovered in the liver compared to the testis. FC means fold change. The upper and lower blue lines represent log Fold Change = 1 and log Fold Change = −1, respectively.

Here, we discovered the differences of PAS in terms of PAS number and usage using the PASs identified from data sets of the liver and testis (SRP018524)^27^, resulting in 12,777 and 14,375 PASs in the liver and testis, respectively. Approximately 21% of total PASs were commonly present in the two tissues, whereas others uniquely existed in one tissue (Fig. 3a), which implied that the PAS number varied between the liver and testis. In addition, the usage of the common PASs was analysed using the edgeR program, and the results illustrated that most of the common PASs had various usages between the two tissues (Fig. 3b). Therefore, both PAS number and usage were different between the liver and testis.

**Figure 4:**
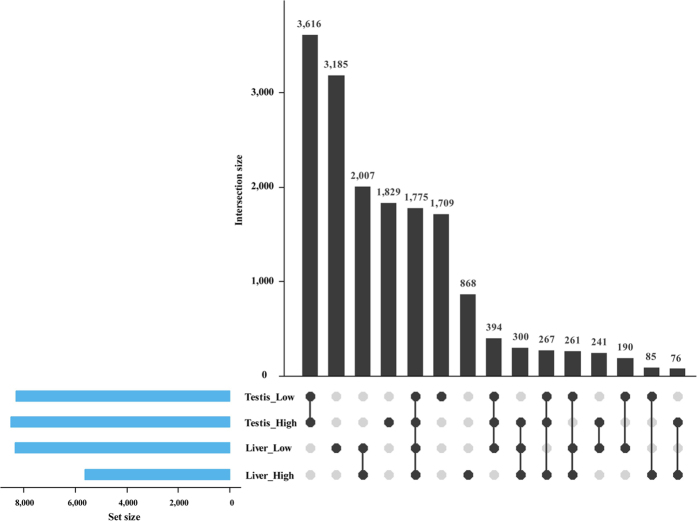
Difference in PAS number between the liver and testis with high and low androstenone. The horizontal bars (in blue) represent the total PAS number in each group. The vertical bars (in black) represent the common PAS number corresponding to the bottom intersection groups.

**Figure 5:**
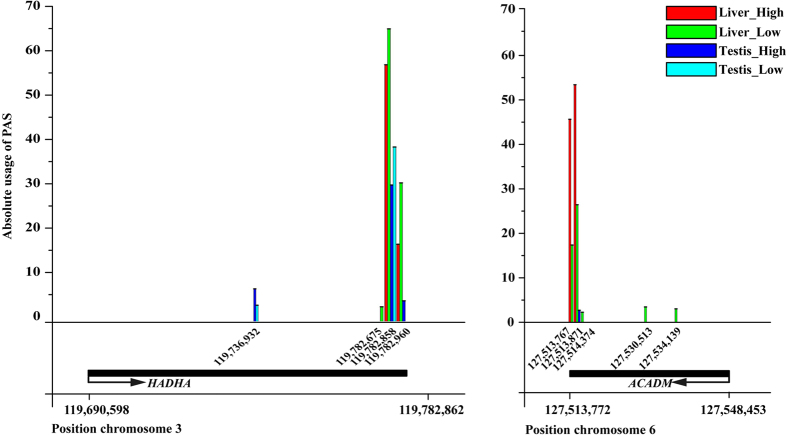
Different usage of the same PAS among groups for two genes, *HADHA* and *ACADM*. X axis, PASs and genomic positions of genes. Y axis, the number of polyadenylated reads mapping to PAS was counted and normalized to the total number of polyadenylated reads within each data set and rescaled by a factor of 10^8^.

With a further comparison among tissues, we discovered PAS in four data sets (liver of pigs with high androstenone, liver of pigs with low androstenone, testis of pigs with high androstenone and testis of pigs with high androstenone) and the PAS information was listed in Supplementary Tables S3–S6, with the commonness and difference in the PAS number among the four groups shown by an upset R program^28^. The results indicated that the number of common PAS is larger in the same tissue than in a different tissue (Fig. 4). Additionally, we compared the absolute value of PAS usage in a PAS among the four groups. Two examples from our results were used to indicate the different usage of PAS under various tissues and conditions (Fig. 5). The *HADHA* gene has been identified to have a total of four PASs, and the first PAS produces a shorter 3′UTR and only exists in the testis. The last three PASs produce relatively longer 3′UTR and show various usages among the four groups. *ACADM* is a reverse transcription gene with five PASs found along it, the usages of which are diverse between the liver and testis as well as under different conditions. The aforementioned results illustrate that PAS number and usage have sharp differences between the liver and testis.

### Relationship between the functional usage of PAS and pig traits

Two data sets, ERP004640 and SRP018524, were selected to analyse PAS function under a different androstenone level and salmonella inoculation, respectively^27^,^29^, including biological replications as well as control and treatment conditions. PAS was first discovered from the two data sets; then, PASs with different usages and their associated genes were identified for gene functional analysis in terms of corresponding conditions (see Materials and Methods).

### PAS usage related to the androstenone level of pigs

**Figure 6:**
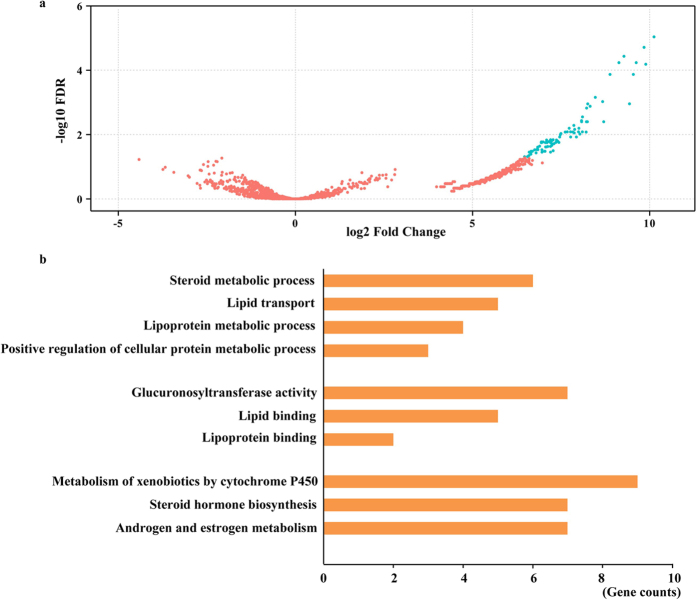
Different-usage PASs, functional groups of associated genes in the liver with high and low androstenone. (**a**) Representation of 84 different-usage PASs (in blue) with FDR < 0.05 and |log2FC| ≥1. (**b**) Gene ontology analysis of the different-usage PAS-associated genes.

The RNAseq data of the testis and liver were collected from ten pigs with two different androstenone levels (five pigs with high androstenone and five with low androstenone), enabling us to discover the different-usage PASs in the testis and liver under the two conditions. According to PASs obtained from the four data sets (Supplementary Tables S3–S6), the different-usage PASs under the two conditions were investigated. In the liver, 84 PASs showed usages that were statistically significant, with at least a 2-fold difference and a FDR less than 0.05 (FDR <0.05, |log2FC| ≥1) (Fig. 6a, Supplementary Table S7). All of them displayed high usages in the liver of pigs with low androstenone, and 36 protein-coding genes harboured them. Gene ontology analysis indicated that the significant high-usage PAS-associated genes were mainly involved in lipid binding, steroid metabolic process, steroid hormone biosynthesis, and the metabolism of xenobiotics by cytochrome P450 (P < 0.05) in terms of molecular function, biological process and KEGG pathway enrichment (Fig. 6b, Supplementary Table S8). These results indicate that the high-usage PASs have a close relationship with liver function in pigs with a low level of androstenone.

In the testis, 29 PASs showed different usages that were statistically significant (FDR <0.05, |log2FC| ≥1) between high and low androstenone conditions. (Supplementary Table S9), and 19 associated genes harboured the PASs. Gene ontology analyses displayed that some of the genes were involved in spermatogenesis, translation and gene expression (Supplementary Table S10), suggesting that the different-usage PASs might be related to the testis function of pigs at the androstenone level.

### PAS usage participates in the immune response of pigs after salmonella inoculation

**Figure 7:**
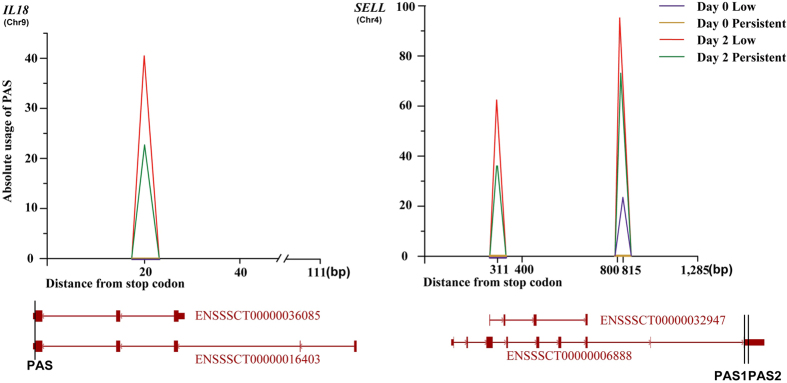
Different-usage PASs for *IL18* and SELL genes before and after salmonella inoculation. The vertical bars (in black) on gene transcripts represent the positions of PAS.

One of the RNAseq data sets we downloaded was collected from 16 pigs, including 8 pigs with low shedding and 8 pigs with persistent shedding. Peripheral blood was collected before (day 0) and two days after (day 2) salmonella inoculation. After PAS detection, we found 20 PASs with a statistically significant difference in usage between two time points (FDR <0.05, |log2FC| ≥1, Supplementary Table S11). Additionally, 16 porcine genes harboured the different-usage PASs. Gene ontology analysis of the different-usage PAS-associated genes showed the involvement of some of the genes in the inflammatory response and defence response. Two of the genes, *IL18* and *SELL*, were presented as the examples for different-usage PASs under various conditions (Fig. 7). These results show that PAS with a different usage might participate in the immune process after salmonella inoculation.

### PAS validation

**Figure 8:**
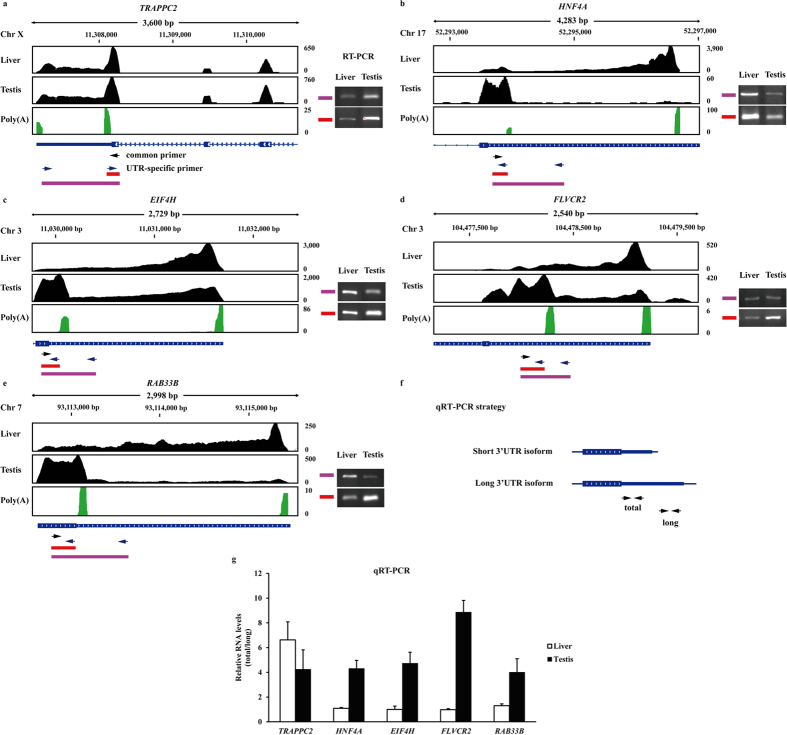
PASs validation in five genes. **(a–e)** The black regions represent the mapped RNAseq reads from the liver and testis, green indicates the poly(A)-spanning reads pooled from all RNAseq libraries. RT-PCR was performed using a common primer (black arrows) and UTR-specific primer (blue arrows). To the right of the RNAseq data are agarose gels stained with GelRed, in which lower molecular weight bands were produced from both short and long 3′UTR isoforms and higher molecular weight bands are specific to long 3′UTR isoforms. **(f)** Strategy for the quantification of alternative length 3′UTRs in the liver and testis by qRT-PCR. **(g)** Relative RNA levels are shown as the ratio of total/long isoforms with the standard error bars.

As two or more PASs could produce more than one transcript with different lengths of the 3′UTR for a gene, we detected the expression of 3′UTR isoforms to confirm corresponding PASs. We selected five genes (*TRAPPC2*, *HNF4A*, *EIF4H*, *FLVCR2* and *RAB33B*) from annotated PASs we discovered (Supplementary Table S2), which contained ten PASs in their 3′UTRs. To confirm the PASs, we performed RT-PCR with the use of a common proximal primer and two specific distal primers for each gene (Fig. 8a–e). The short PCR product corresponds to the expression of total isoforms, and the longer one specified the expression of the long isoform. These assays indicate that the proximal PASs of five genes existed in the testis, and the proximal PAS of *TRAPPC2* was also found in liver (Fig. 8a).

In addition, we performed qRT-PCR experiments for further confirmation of the PASs with the use of primers that amplify all 3′UTR isoforms for a gene (total) or only the longer 3′UTR isoform (long) (Fig. 8f). The results, displayed as a ratio of total/long isoforms, show the 4- to 9-fold increased expression of all 3′UTR isoforms in the testis and approximately 7-fold for first gene in the liver (Fig. 8g), which are consistent with the RT-PCR results. Altogether, these analyses confirmed our findings and validated the computational method.

## Discussion

In the process of PAS detection, polyadenylated reads were identified with at least ten A or T residues, a standard obviously higher than that with five A or T residues^30^. Additionally, the internal priming was eliminated in the pig genome sequence, which generated PAS with a low false positive rate. Despite the strict criteria used in the process of PAS discovery and the high positive PAS obtained, the results did not represent the comprehensive PAS in pigs due to the lower number of polyadenylated reads. We only obtained 1.92 million polyadenylated reads uniquely mapped to the pig genome from the original 12.2 billion RNAseq reads. The low discovery rate (0.016%) made the wide discovery of PAS in pigs, as well as in some rare sequencing species, difficult. Direct RNA sequencing (DRS) has been used for PAS discovery in humans and yeasts; this process does not require the conversion of RNA molecules to cDNA and eliminates the bias for ligation or amplification^31^. Additionally, poly(A) site sequencing (PAS-Seq) has also been developed for profiling RNA polyadenylation at the transcriptome level^32^,^33^. Recently, whole transcriptome termini site sequencing (WTTS-Seq) is an effective library preparation method to maximize transcriptomic investigation and validate transcriptome-wide APA^34^. Both the methods could obtain more polyadenylated reads than whole transcriptome shot gun sequencing. Therefore, PAS-Seq and other methods should be developed for the more accurate detection of pig PASs in the future.

In this study, we found that 13,033 PASs overlapped with annotated genes, 26% of which were located in intron regions, probably due to intron retention in the process of mRNA maturation from pre-mRNA^35^ and transcript production with a different number of exons. PAS was common in 3′UTRs (61%) but occurred less in ORFs (17%), leading to different exon lengths and further protein synthesis. The PAS located in 3′UTRs always produces genes with short 3′UTRs and increases gene expression by the lack of some microRNA target sequences^36^. Additionally, Bin Tian found 29,283 and 16,282 PASs in humans and mice, respectively, and 54% and 32% of the PAS-associated genes exhibited alternative polyadenylation, respectively^12^. In the present study, we found 28,363 PASs associated with 7,403 pig genes, 41% of which harboured alternative polyadenylation sites (Fig. 2a). The analysis of PAS annotation showed that 30% of total PASs were located in intergenic regions, which might contain some protein coded genes or small non-annotated RNAs. From the results, we speculated that the current imperfect annotation of pig genes made some PASs difficult to annotate, which might affect PAS annotation in gene features. Further study is needed to fill the gaps in pig gene annotation.

In our results, we found that the PAS number and usage varies between the liver and testis. More than one PAS can exist in genes, such as *HADHA* and *ACADM* genes. It is difficult to determine which PAS is more highly used than others. However, a usage comparison of the same PAS illustrates that PAS varies among different tissues, which is also supported by other reports^32^,^36^,^37^. Furthermore, the prominent differences in PAS number and usage could indicate alternative polyadenylation, which might alter the gene expression and be associated with different functions among tissues.

Alternative polyadenylation might depend on the environment^38^ and is implicated in many diseases^11^. PAS number and usage provide the possibility for alternative polyadenylation. With changing environments such as bacterial inoculation, PAS number and usage might also vary, leading to the occurrence of alternative polyadenylation. In this study, we discovered different-usage PASs and associated genes by RNAseq data before and after salmonella inoculation. Some of the PASs displayed high usage after infection and the associated genes were involved in immune responses, which implied that PAS might participate in the immune process as one of the immune mechanisms in disease development.

## Materials and Methods

### Data generation

All pig RNAseq data sets used for PAS discovery and functional analysis were obtained from SRA in NCBI (http://www.ncbi.nlm.nih.gov/sra) and one of the data sets was from our own unpublished data. The studies with our own data involving animals were conducted according to the Regulation of the Standing Committee of Hubei People’s Congress. All experimental protocols were approved by the Ethics Committee of Huazhong Agricultural University, P.R. China. The animal experiments were performed at the Laboratory Animal Center of Huazhong Agricultural University, P.R. China. Total RNAseq data sets and corresponding experiments are listed in Supplementary Table S1.

(1) Total RNA was isolated from adipose tissue (backfat) of six Iberian × Landrace pigs and sequenced in 75-bp paired-end reads (SRP031783)^39^.

(2) Hypothalamic transcriptome data of ten divergent pigs for growth and fatness traits was generated in 75-bp paired-end reads (SRP032451)^40^.

(3) Total RNA of three organs (liver, abdominal fat and longissimus dorsi) of two female pigs was sequenced in 90-bp paired-end reads (SRP006208)^41^.

(4) Total RNA from the liver of 23 pigs at four time points of the carbohydrate pre-fed state was sequenced in 50-bp paired-end reads (SRP039511)^42^.

(5) Total RNA from macrophages infected with two PRRSV strains and a mock group was sequenced in 100-bp paired-end reads (SRP030003)^43^.

(6) Liver tissues were collected from the following three groups: high fat diet-fed intact male pigs, castrated male pigs, and castrated male pigs with testosterone replacement, from which total RNA was extracted and sequenced in 100-bp paired-end reads (SRP049250)^44^.

(7) Four tissue RNA libraries (heart, liver, lung and kidney) of one Tibetan wild boar were constructed and sequenced in 100-bp paired-end reads (SRP018288)^45^.

(8) Thirty-six Duroc pigs were divided into three groups according to high, mean and low level of subcutaneous adipose tissue, from which the RNA was extracted and sequenced in 100-bp paired-end reads (SRP046752)^46^.

(9) The longissimus dorsi muscle and subcutaneous adipose tissues of nine pigs (Danbred 600× Newsham NC50) were used for total RNA extraction and subsequent sequencing in 100-bp paired-end reads (SRP055090)^47^.

(10) The frontal parts of two pig brains were used for total RNA extraction and subsequent sequencing in 50-bp paired-end reads (SRP017611)^48^.

(11) Macrophages were isolated from the lavage fluid of the lung, and then treated separately with LPS, IFNγ, IL-4, IL-10, IFNα and IFNβ, respectively. Finally, the six cell groups were infected with a PRRSV strain for five hours, and RNA was extracted and sequenced in 50-bp single-end reads (SRP033717)^49^.

(12) Small intestine tissues (duodenum, jejunum, ileum and ileal) from four Large White pigs were used for total RNA extraction. Sixteen libraries were constructed and sequenced in 110-bp single-end reads (SRP030679)^50^.

(13) Endometrial RNA samples of 12-day and 14-day pregnant pigs (eight) and corresponding nonpregnant controls (eight pigs) were sequenced in 75-bp single-end reads (SRP027378)^51^.

(14) Sixteen pigs were identified as low shedding (eight) and persistent shedding (eight). The whole blood samples were collected at day 0 and day 2 after the inoculation of pigs with salmonella. A total of 32 RNA libraries were sequenced in 50-bp single-end reads (ERP004640)^29^.

(15) Two anterior pituitary tissues came from one Bama and one Landrace pig, and then RNA was extracted and sequenced in 50-bp single-end reads (SRP057957)^52^.

(16) Longissimus dorsi muscle tissues of nine Liangshan pigs were used for RNA extraction, and nine libraries were constructed and sequenced in 50-bp single-end reads (SRP058576)^53^.

(17) Two Large White pigs were fed a standard diet supplemented with high amounts of methylating micronutrients, and two Large White pigs were fed a standard diet as a control group. The sperm cells of the four pigs were isolated and the RNA extracted was sequenced in 100-bp single-end reads (SRP027011)^54^.

(18) Longissimus dorsi muscle tissues were collected from Yorkshire pigs with high residual food intake (RFI) (three) and low RFI (three). RNA sequencing was performed in 50-bp single-end reads (ERP007043)^55^.

(19) The RNA was extracted from the testis and liver tissues from ten boars, five samples each for high and low androstenone levels and sequenced in 100-bp single-end reads (SRP018524)^27^.

(20) Based on our own unpublished data, the RNA was extracted from macrophages of three Tongcheng (a typical indigenous Chinese breed) and three Large White pig individuals infected with PRRSV, with three Tongcheng and three Large White pigs as control groups. RNA libraries were constructed and sequenced in 100-bp paired-end reads.

### Polyadenylated reads extraction and PAS discovery

The whole process of PAS detection consists of five steps (Supplementary Fig. S1). In step 1, RNAseq data were extracted from sra files downloaded using fastq-dump and the split-3 parameter was used for the extraction of pair-end reads, followed by filtering read quality and read length using Trimmomatic software^26^. After that, we aligned RNAseq reads from each data set to the *Sus scrofa* 10.2 genome (Ensembl database) by the bowtie2 program^56^. For each data set, alignment was performed with the sensitive setting (*-D 15 -R 2 -N 0 -L 22 -i S, 1, 1.15*) in the ‘end-to-end’ model. Meanwhile, *un-conc* and *un* parameters in the bowtie2 programme were used to extract unmapped reads from pair-end and single-end data sets, respectively.

In step 2, the reads that failed to be aligned to the pig genome were examined to identify polyadenylated reads^37^,^57^. Two strategies were adopted to extracted polyadenylated reads in terms of different libraries building types of RNAseq. For non-strand specific data, we extracted all reads that either started or ended with a run of at least ten consecutive As or Ts. In either case, error bases in homopolymers were allowed with a 10% mismatch. For strand-specific RNAseq data, we extracted the first reads containing at least ten consecutive T at 5′ end or second reads containing at least ten consecutive A at the 3′ end, also with a 10% mismatch allowed. The *FindTail* programme was used for the discovery of A and T residues^58^. Here, we used criteria of at least 10 consecutive A or T with a 10% mismatch allowed in each case, which were higher than those suggested by Dong *et al*.

In step 3, terminal A or T residues in candidate polyadenylated reads extracted from each data set were trimmed using a custom perl script, and the trimmed reads with a length less than 20 bp were discarded. All the trimmed reads were pooled and mapped back to *Sus scrofa* 10.2 genome by bowtie2 with one mismatch parameter (*N 1*). After that, uniquely mapped reads were selected for the next step.

In step 4, to eliminate the possibility that reads are not polyadenylated, the internal priming issue was considered due to the common presence of consecutive A or T residues in the genome sequence, which might lead to false positive PAS. The *CleanSam.pl* script was used to scan the sequence of the *Sus scrofa* 10.2 genome around PASs^58^. Briefly, we removed putative PASs where the downstream genomic regions contained at least nine consecutive A or T residues.

In step 5, the remaining reads were combined and clustered so that all reads aligning to the same strand and ending within twenty nucleotides were classified into a cluster. In each cluster, PAS covered with at least three reads were retained and the PASs covered by the most reads were determined as positive sites for further analysis.

### PAS annotation

The annotated pig gene file (Sus_scrofa.Sscrofa10.2.81.gtf) obtained from the Ensembl database was used first for PAS annotation; then, de novo transcripts were detected using all RNAseq data to fill the gap in the PAS annotation caused by the former annotated file. Alignment was performed for each RNAseq data set using Hisat2 software with –dta-cufflinks parameter^59^. Next, cufflinks and cuffmerge programmes were used to generate de novo transcripts according to the protocol as previously described^60^.

### PAS usage and functional analysis

We referred to the absolute usage of PAS when analysing the differences in PAS in the liver and testis. The absolute usage of PAS was calculated to compare PAS usages in the two tissues. The number of polyadenylated reads mapping to PAS was counted using the htseq-count program^61^ and normalized to the total number of polyadenylated reads within each data set. The absolute value of PAS usage was obtained by rescaling the number with a factor of 10^8^.

To compare PAS usages under a different androstenone level and salmonella inoculation, logFoldChange was employed and performed using the edgeR program^62^. For data with no biological replication, such as the analysis of PAS usage in the common PAS of liver and testis, the *estimateGLMCommonDisp* method was used to estimate dispersion without replicates in the edgeR programme. However, the *estimateCommonDisp* and *estimateTagwiseDisp* methods were utilized to discover different-usage PASs under conditions with multiple replicates. Different-usage PASs were filtered according to the FDR and logFoldChange obtained from the edgeR programme.

For statistically significant different-usage PASs under different conditions, the high or low usage of PAS (high-usage or low-usage PAS) associated genes were annotated and their functions were analysed. The DAVID web tool^63^,^64^ was used to identify PAS-associated genes that were homologous to human genes and to classify the genes in terms of molecular function, cell components and biological processes.

### RT-PCR and qRT-PCR

Total RNA of the liver and testis obtained from three Tongcheng pigs was extracted from 100 mg of each sample using the TRIzol Reagent (Invitrogen) following the manufacturer’s instructions. Then, the cDNA of each sample was reverse-transcribed using a First Strand cDNA Synthesis Kit (ThermoFisher). cDNA was diluted with H_2_O at the rate of 1:4 for RT-PCR and qRT-PCR. For RT-PCR, a common proximal primer (forward) and two specific distal primers (reverse) were designed for each gene (Fig. 8a–e). After agarose gel electrophoresis, we detected the proximal PAS for each gene by comparing the lower and higher molecular weight bands. In addition, the total and long primers used for qRT-PCR were designed according to the common region of short and long 3′UTR isoforms and the specified region in long 3′UTR isoform, respectively (Fig. 8f). qRT-PCR was performed on a CFX384 Real-Time System (BioRad) in triplicate and the expression levels of the total and long isoforms were quantified relative to the expression of the porcine *RPL32* gene. Relative RNA levels are shown as ratio of total/long isoforms with the standard error bars. The primers designed for putative PASs and internal control *RPL32* are shown in Supplementary Table S12.

## Additional Information

**How to cite this article**: Wang, H. *et al*. Genome-Wide Analysis and Functional Characterization of the Polyadenylation Site in Pigs Using RNAseq Data. *Sci. Rep.* **6**, 36388; doi: 10.1038/srep36388 (2016).

**Publisher’s note:** Springer Nature remains neutral with regard to jurisdictional claims in published maps and institutional affiliations.

## Supplementary Material

Supplementary Information
