# Supplementary material for: Genome-Wide Analysis and Functional Characterization of the Polyadenylation Site in Pigs Using RNAseq Data
Source: Sci Rep. 2016 Nov 4;6:36388. doi: 10.1038/srep36388 (PMC5095665; doi:10.1038/srep36388)
Supplement: Supplementary Information [file srep36388-s1.pdf]

Genome-Wide Analysis and Functional Characterization of the Polyadenylation Site in Pigs Using RNAseq Data

Hongyang Wang[1, 2], Rui Li[3], Xiang Zhou[3], Liyao Xue[1,2], Xuewen Xu[1,2], Bang Liu[1, 2*]

[1] Key Laboratory of Pig Genetics and Breeding of Ministry of Agriculture & Key Laboratory of Agricultural Animal Genetics, Breeding and Reproduction of Ministry of Education; Huazhong Agricultural University, Wuhan, China

[2] The Cooperative Innovation Center for Sustainable Pig Production, Wuhan, China

[3] Department of Animal Sciences, Washington State University, Pullman, WA, USA

[*]Corresponding author

Bang Liu (liubang@mail.hzau.edu.cn)

Supplementary Information

Supplementary Figure S1: The process of PAS detection and functional analysis.

Supplementary Table S1: Information of RNAseq data. Data provided for all RNAseq data used in PAS discovery.

Supplementary Table S2: Total PASs discovered from all RNAseq data.

Supplementary Table S3: PAS discovered from Liver of pigs with low androstenone.

Supplementary Table S4: PAS discovered from Liver of pigs with high androstenone.

Supplementary Table S5: PAS discovered from Testis of pigs with low androstenone.

Supplementary Table S6: PAS discovered from Testis of pigs with high androstenone.

Supplementary Table S7: Different-usage PAS in Liver with different conditions (Low androstenone/High androstenone).

Supplementary Table S8: Gene ontology analysis of high-usage PASs associated genes in liver of pigs with low androstenone.

Supplementary Table S9: Different-usage PAS in Testis with different conditions (Low androstenone/High androstenone).

Supplementary Table S10: Gene ontology analysis of high-usage PASs associated genes in testis of pigs.

Supplementary Table S11: Different-usage PAS after and before Salmonella inoculation (Day 2/Day 0).

Supplementary Table S12: Primers designed for PAS validation.

Supplementary Figure S1: The process of PAS detection and functional analysis.

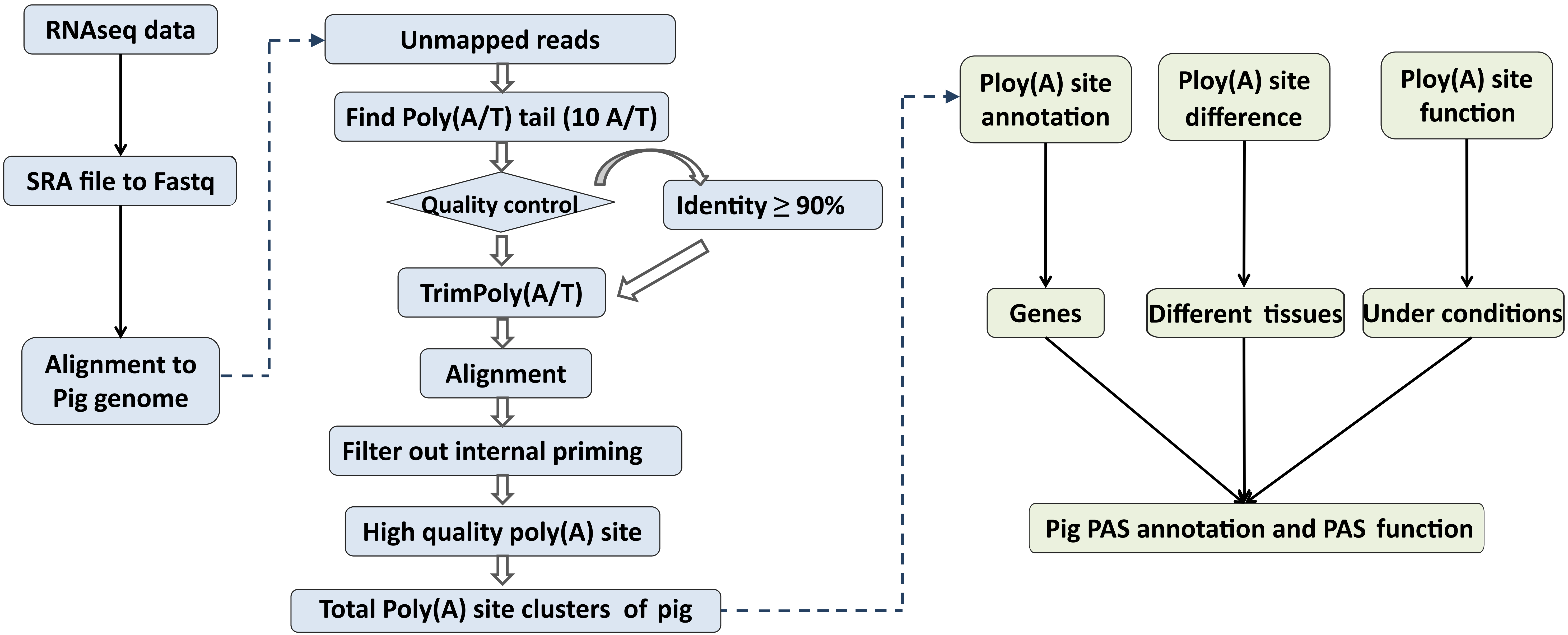

Supplementary Table S1: Information of RNAseq data. Data provided for all RNAseq data used in PAS discovery. Accession number for data download from SRA database (www.ncbi.nlm.nih.gov/sra). Unpublished data is our own data, which has not published yet. Samples number was used in each experiment. Raw reads number were counted in each data set. Unmapped reads mean the number of raw reads unmapped to pig genome after alignment performed. Tissues or cell used in each experiment under various conditions were described. The corresponding reference for each data set was listed in Reference part of manuscript. The first data generated from our lab has not published yet.

**Accession**	**Samples**	**Platform**	**Strand specific**	**Type**	**Raw Reads (Mil.)**	**Unmapped reads (Mil)**	**Tissues or Cell (conditions)**	**Reference**
Unpublished	12	HiSeq 2000	Yes	Pair(100bp×2)	1,771	583	Macrophages (PRRSV infection)	No
SRP031783	6	HiSeq 2000	No	Pair(75bp×2)	350	112	Backfat	[38]
SRP032451	10	HiSeq 2000	No	Pair(75bp×2)	1,059	460	Hypothalamus	[39]
SRP006208	6	HiSeq 2000	No	Pair(90bp×2)	237	107	Liver, Abdominal fat, Longissimus dorsi	[40]
SRP039511	92	HiSeq 2000	No	Pair(50bp×2)	2,143	913	Liver	[41]
SRP030003	8	HiSeq 2000	No	Pair(100bp×2)	234	136	Macrophages	[42]
SRP049250	9	HiSeq 2000	No	Pair(100bp×2)	125	65	Liver (Pig castration)	[43]
SRP018288	1	HiSeq 2000	No	Pair(100bp×2)	191	94	Kidney, Lung, Liver, Heart	[44]
SRP046752	36	HiSeq 2500	No	Pair(100bp×2)	1,304	716	Subcutaneous adipose	[45]
SRP055090	18	HiSeq 2000	No	Pair(100bp×2)	1,156	557	Longissimus dorsi muscle, Subcutaneous adipose	[46]
SRP017611	2	HiSeq 2000	No	Pair(50bp×2)	100	43	Brain	[47]
SRP033717	6	HiSeq 2000	No	Single(49bp)	182	47	Macrophages	[48]
SRP030679	16	HiSeq 2000	No	Single(110bp)	445	143	Small intestine	[49]
SRP027378	16	Illumina GA	No	Single(76bp)	523	175	Endometrium	[50]
ERP004640	32	HiScanSQ	No	Single(51bp)	1,612	409	Blood (Salmonella infection)	[28]
SRP057957	2	HiSeq 2000	No	Single(50bp)	15	3	Anterior pituitary	[51]
SRP058576	9	HiSeq 2000	No	Single(50bp)	42	12	Longissimus dorsi muscle	[52]
SRP027011	4	HiSeq 2000	No	Single(100bp)	144	11	Sperm	[53]
ERP007043	6	HiSeq 2000	Yes	Single(50bp)	55	16	Longissimus dorsi muscle	[54]
SRP018524	10	HiSeq 2000	No	Single(100bp)	474	192	Liver, Testis (Androstenone)	[26]

Supplementary Table S2: Total PASs discovered from all RNAseq data. Data provided for genomic positions of PASs and PASs associated gene features. PAS cluster start and end are the start and end position in genomic coordinate of one poly(A) cluster, respectively. Strand (+/-) of the original genome annotation. Positive PAS represents the PAS covered with most reads in each cluster. Positive PAS reads show the number of covered reads in each positive PAS. Cluster reads show the number of total reads in each cluster. The last four columns show genes harbored the corresponding PAS. Here --- means no genes for PAS annotation. Numbers started with "ENSSCG" and "XLOC_" inferred the PAS were annotated from Ensembl database and novo genes predicted, respectively.

Chr	PAS Cluster Start	PAS Cluster End	PAS Strand	Positive PAS	Positive PAS reads	Cluster reads	Gene ID	Gene Strand	Type	Gene Name
1	1069588	1069588	-	1069588	6	6	ENSSSCG00000004010	-	3'UTR	*ERMARD*
1	1348897	1348897	-	1348897	3	3	---	---	intergenic	---
1	1904746	1904746	-	1904746	7	7	XLOC_001890	-	predicted	---
1	2651496	2651527	+	2651516	14	44	XLOC_000024	+	predicted	---
1	2677888	2677889	-	2677888	16	20	ENSSSCG00000004016	-	intron	*KIF25*
1	2690116	2690116	-	2690116	3	3	ENSSSCG00000004016	-	intron	*KIF25*
1	2720203	2720205	-	2720205	22	27	ENSSSCG00000004018	-	3'UTR	*MLLT4*
1	2871206	2871206	+	2871206	4	4	XLOC_000028	+	predicted	---
1	2896944	2896944	-	2896944	5	5	XLOC_001901	-	predicted	---
1	3215154	3215155	-	3215154	8	12	---	---	intergenic	---
1	3215178	3215178	+	3215178	5	5	---	---	intergenic	---
1	3238597	3238644	-	3238634	14	44	ENSSSCG00000004023	-	3'UTR	*FGFR1OP*
1	3238683	3238683	-	3238683	3	3	ENSSSCG00000004023	-	3'UTR	*FGFR1OP*
1	3307690	3307698	+	3307693	37	112	XLOC_000039	+	predicted	---
1	3662173	3662179	+	3662173	4	8	ENSSSCG00000004022	+	3'UTR	*RPS6KA2*
1	3692463	3692481	+	3692474	207	370	XLOC_000043	+	predicted	---

1	3732834	3732843	+	3732835	8	26	ENSSSCG00000004020	+	3'UTR	*SFT2D1*
1	3733141	3733142	+	3733142	26	32	ENSSSCG00000004020	+	3'UTR	*SFT2D1*
1	3752042	3752042	+	3752042	10	10	XLOC_000044	+	predicted	---
1	4108136	4108138	+	4108136	5	8	---	---	intergenic	---
1	4108234	4108234	+	4108234	6	6	---	---	intergenic	---
1	4108299	4108299	-	4108299	4	4	---	---	intergenic	---
1	4108370	4108371	+	4108371	10	13	---	---	intergenic	---
1	4108481	4108491	+	4108481	6	15	---	---	intergenic	---
1	4108559	4108566	+	4108566	20	63	---	---	intergenic	---
1	4108611	4108611	+	4108611	7	7	---	---	intergenic	---
1	4298567	4298567	-	4298567	3	3	---	---	intergenic	---
1	4383515	4383515	+	4383515	3	3	---	---	intergenic	---
1	4743626	4743630	+	4743626	4	8	XLOC_000048	+	predicted	---
1	4752292	4752292	-	4752292	3	3	---	---	intergenic	---
1	4760389	4760414	+	4760390	32	72	ENSSSCG00000004028	+	intron	*C6orf118*
1	4760489	4760489	+	4760489	3	3	ENSSSCG00000004028	+	intron	*C6orf118*
1	4760580	4760580	+	4760580	3	3	ENSSSCG00000004028	+	intron	*C6orf118*
1	4776329	4776374	+	4776374	6	20	XLOC_000048	+	predicted	---
1	4886814	4886814	-	4886814	3	3	---	---	intergenic	---
1	5073463	5073463	+	5073463	5	5	---	---	intergenic	---
1	5789742	5789750	-	5789749	5	12	---	---	intergenic	---
1	6280434	6280440	-	6280436	10	18	---	---	intergenic	---
1	6336463	6336463	+	6336463	3	3	XLOC_000058	+	predicted	---
1	6341394	6341394	+	6341394	4	4	XLOC_000058	+	predicted	---
1	6374742	6374743	-	6374743	6	9	XLOC_001921	-	predicted	---
1	6374855	6374865	-	6374865	11	16	XLOC_001921	-	predicted	---

1	6377672	6377681	-	6377673	21	64	XLOC_001921	-	predicted	---
1	6381277	6381277	-	6381277	9	9	ENSSSCG00000004029	-	intron	*QKI*
1	6384569	6384569	-	6384569	4	4	ENSSSCG00000004029	-	intron	*QKI*
1	6496733	6496750	-	6496733	101	119	---	---	intergenic	---
1	6537960	6537960	+	6537960	3	3	XLOC_000059	+	predicted	---
1	6597170	6597170	-	6597170	4	4	---	---	intergenic	---
1	6609765	6609774	-	6609768	128	242	---	---	intergenic	---
1	6680433	6680433	-	6680433	5	5	XLOC_001925	-	predicted	---
1	6736265	6736271	+	6736265	3	9	---	---	intergenic	---
1	6736716	6736717	-	6736717	4	7	---	---	intergenic	---
1	6737180	6737180	+	6737180	3	3	---	---	intergenic	---
1	6883912	6883912	+	6883912	4	4	XLOC_000061	+	predicted	---
1	7065273	7065277	-	7065274	16	30	XLOC_001928	-	predicted	---
1	7663538	7663538	+	7663538	3	3	ENSSSCG00000004032	+	intron	*PARK2*
1	8266588	8266588	-	8266588	5	5	---	---	intergenic	---
1	8746436	8746436	+	8746436	4	4	ENSSSCG00000004038	+	ORF	*PLG*
1	8751317	8751317	+	8751317	5	5	ENSSSCG00000004038	+	ORF	*PLG*
1	8753028	8753028	+	8753028	3	3	ENSSSCG00000004038	+	ORF	*PLG*
1	8755004	8755012	+	8755012	33	52	ENSSSCG00000004038	+	intron	*PLG*
1	8758806	8758820	+	8758806	6	11	ENSSSCG00000004038	+	ORF	*PLG*
1	8763403	8763404	+	8763403	4	7	ENSSSCG00000004038	+	ORF	*PLG*
1	8774345	8774352	+	8774345	3	6	ENSSSCG00000004038	+	intron	*PLG*
1	8778362	8778362	+	8778362	3	3	ENSSSCG00000004038	+	ORF	*PLG*
1	8787273	8787312	+	8787276	29	67	ENSSSCG00000004038	+	ORF	*PLG*
1	8787523	8787561	+	8787544	581	1941	ENSSSCG00000004038	+	3'UTR	*PLG*
1	9127486	9127488	+	9127487	10	18	---	---	intergenic	---

1	9294494	9294494	+	9294494	3	3	XLOC_000078	+	predicted	---
1	9321701	9321706	-	9321704	8	21	---	---	intergenic	---
1	9321740	9321740	+	9321740	4	4	---	---	intergenic	---
1	9359192	9359192	-	9359192	3	3	XLOC_001944	-	predicted	---
1	9380718	9380722	-	9380720	41	89	ENSSSCG00000004047	-	3'UTR	*MRPL18*
1	9392954	9392954	+	9392954	13	13	ENSSSCG00000004048	+	intron	*TCP1*
1	9397887	9397887	+	9397887	4	4	ENSSSCG00000004048	+	ORF	*TCP1*
1	9399573	9399582	-	9399579	41	67	ENSSSCG00000004049	-	3'UTR	*ACAT2*
1	9399654	9399667	+	9399654	50	213	ENSSSCG00000004048	+	3'UTR	*TCP1*
1	9399851	9399851	-	9399851	3	3	ENSSSCG00000004049	-	3'UTR	*ACAT2*
1	9419862	9419870	-	9419862	12	19	XLOC_001946	-	predicted	---
1	9426852	9426857	-	9426856	19	31	ENSSSCG00000004050	-	intron	*WTAP*
1	9457105	9457109	+	9457108	13	39	XLOC_000086	+	predicted	---
1	9484860	9484860	+	9484860	4	4	ENSSSCG00000029876	+	3'UTR	*SOD2*
1	9484990	9485021	+	9484990	3	6	ENSSSCG00000029876	+	3'UTR	*SOD2*
1	9485261	9485266	+	9485266	11	20	ENSSSCG00000029876	+	3'UTR	*SOD2*
1	9485433	9485433	+	9485433	8	8	ENSSSCG00000029876	+	3'UTR	*SOD2*
1	9485512	9485531	+	9485525	14	35	ENSSSCG00000029876	+	3'UTR	*SOD2*
1	9486920	9486927	+	9486920	21	24	ENSSSCG00000029876	+	3'UTR	*SOD2*
1	9487629	9487634	+	9487629	71	85	ENSSSCG00000029876	+	3'UTR	*SOD2*
1	9487676	9487688	+	9487683	40	55	ENSSSCG00000029876	+	3'UTR	*SOD2*
1	9488771	9488771	+	9488771	4	4	XLOC_000087	+	predicted	---
1	9838882	9838882	-	9838882	5	5	ENSSSCG00000004052	-	3'UTR	*FNDC1*
1	9845476	9845476	-	9845476	4	4	ENSSSCG00000004052	-	intron	*FNDC1*
1	9904954	9904954	-	9904954	7	7	ENSSSCG00000004052	-	intron	*FNDC1*
1	9952480	9952481	-	9952480	6	9	XLOC_001952	-	predicted	---

1	9974348	9974349	+	9974348	14	21	---	---	intergenic	---
1	10051656	10051678	+	10051659	20	29	ENSSSCG00000004053	+	3'UTR	*TAGAP*
1	10067475	10067476	-	10067476	5	8	---	---	intergenic	---
1	10149309	10149323	+	10149317	61	155	ENSSSCG00000004055	+	3'UTR	*RSPH3*
1	10310764	10310765	-	10310765	4	7	---	---	intergenic	---
1	10310786	10310787	+	10310786	20	27	---	---	intergenic	---
1	10312267	10312267	+	10312267	4	4	---	---	intergenic	---
1	10382075	10382075	+	10382075	3	3	---	---	intergenic	---
1	10432156	10432158	+	10432157	33	44	ENSSSCG00000004058	+	3'UTR	*EZR*
1	10432695	10432695	-	10432695	25	25	ENSSSCG00000004057	-	3'UTR	*SYTL3*
1	10565871	10565891	-	10565889	5	16	---	---	intergenic	---
1	10892992	10892992	+	10892992	3	3	---	---	intergenic	---
1	10900664	10900669	-	10900669	5	9	XLOC_001966	-	predicted	---
1	10900863	10900863	-	10900863	3	3	XLOC_001966	-	predicted	---
1	11252466	11252466	-	11252466	3	3	XLOC_001972	-	predicted	---
1	11253571	11253571	-	11253571	3	3	XLOC_001972	-	predicted	---
1	11260371	11260371	-	11260371	5	5	ENSSSCG00000030535	-	intron	*SNX9*
1	11262084	11262084	-	11262084	3	3	ENSSSCG00000030535	-	intron	*SNX9*
1	11475845	11475846	-	11475845	7	10	ENSSSCG00000024528	-	3'UTR	*ZDHHC14*
1	11734100	11734107	+	11734100	22	29	ENSSSCG00000004063	+	intron	---
1	11734449	11734455	+	11734454	9	17	ENSSSCG00000004063	+	intron	---
1	11756297	11756303	+	11756303	7	11	ENSSSCG00000004063	+	3'UTR	---
1	11892259	11892259	+	11892259	4	4	ENSSSCG00000004064	+	3'UTR	*ARID1B*
1	12075577	12075577	+	12075577	4	4	---	---	intergenic	---
1	12083338	12083338	-	12083338	5	5	---	---	intergenic	---
1	12192103	12192103	+	12192103	3	3	---	---	intergenic	---

1	12642501	12642509	-	12642509	4	7	---	---	intergenic	---
1	13215597	13215597	+	13215597	4	4	---	---	intergenic	---
1	13622586	13622599	+	13622587	53	105	ENSSSCG00000024361	+	3'UTR	*TFB1M*
1	13812788	13812788	-	13812788	3	3	---	---	intergenic	---
1	13980542	13980542	+	13980542	3	3	---	---	intergenic	---
1	14012583	14012583	-	14012583	4	4	---	---	intergenic	---
1	14033904	14033904	-	14033904	18	18	ENSSSCG00000004070	-	intron	*SCAF8*
1	14083147	14083149	-	14083147	8	16	ENSSSCG00000004070	-	intron	*SCAF8*
1	14450355	14450355	+	14450355	5	5	ENSSSCG00000004071	+	3'UTR	*CNKSR3*
1	14480589	14480589	-	14480589	4	4	---	---	intergenic	---
1	15268651	15268657	-	15268657	7	15	XLOC_001995	-	predicted	---
1	15313819	15313819	+	15313819	3	3	---	---	intergenic	---
1	15512801	15512801	-	15512801	3	3	---	---	intergenic	---
1	15512835	15512835	+	15512835	3	3	---	---	intergenic	---
1	15537468	15537468	-	15537468	13	13	---	---	intergenic	---
1	15564427	15564428	+	15564427	7	10	---	---	intergenic	---
1	15566768	15566777	-	15566768	6	17	---	---	intergenic	---
1	15708416	15708416	+	15708416	3	3	ENSSSCG00000004076	+	3'UTR	*MTRF1L*
1	15750697	15750697	+	15750697	4	4	---	---	intergenic	---
1	15946509	15946509	-	15946509	5	5	XLOC_001998	-	predicted	---
1	16055725	16055725	+	16055725	3	3	---	---	intergenic	---
1	16134015	16134015	-	16134015	4	4	ENSSSCG00000004081	-	intron	*SYNE1*
1	16177718	16177718	-	16177718	3	3	ENSSSCG00000004081	-	intron	*SYNE1*
1	16185339	16185339	-	16185339	6	6	ENSSSCG00000004081	-	intron	*SYNE1*
1	16433852	16433852	+	16433852	4	4	ENSSSCG00000004082	+	intron	---
1	16508193	16508193	+	16508193	8	8	XLOC_000130	+	predicted	---

1	16546513	16546513	-	16546513	3	3	ENSSSCG00000025777	-	3'UTR	*ESR1*
1	16620198	16620198	-	16620198	4	4	---	---	intergenic	---
1	16659030	16659030	-	16659030	5	5	---	---	intergenic	---
1	17007995	17007999	+	17007995	3	6	---	---	intergenic	---
1	17012443	17012443	-	17012443	7	7	---	---	intergenic	---
1	17172097	17172097	-	17172097	8	8	---	---	intergenic	---
1	17190308	17190310	-	17190309	9	19	---	---	intergenic	---
1	17262698	17262699	+	17262698	5	10	ENSSSCG00000004089	+	3'UTR	*RMND1*
1	17285901	17285901	+	17285901	3	3	---	---	intergenic	---
1	17313169	17313169	-	17313169	7	7	XLOC_002007	-	predicted	---
1	17314973	17314978	-	17314976	19	54	XLOC_002007	-	predicted	---
1	17315671	17315683	-	17315672	16	31	XLOC_002007	-	predicted	---
1	17315722	17315724	-	17315724	20	33	XLOC_002007	-	predicted	---
1	17317574	17317574	-	17317574	3	3	ENSSSCG00000004091	-	ORF	*AKAP12*
1	17318170	17318170	-	17318170	3	3	ENSSSCG00000004091	-	ORF	*AKAP12*
1	17318463	17318463	-	17318463	3	3	ENSSSCG00000004091	-	ORF	*AKAP12*
1	17318594	17318594	-	17318594	3	3	ENSSSCG00000004091	-	ORF	*AKAP12*
1	17320193	17320193	-	17320193	3	3	ENSSSCG00000004091	-	ORF	*AKAP12*
1	17331396	17331402	+	17331396	7	10	---	---	intergenic	---
1	17550523	17550523	-	17550523	4	4	ENSSSCG00000004092	-	3'UTR	---
1	17550688	17550713	-	17550688	4	7	ENSSSCG00000004092	-	3'UTR	---
1	17607174	17607174	-	17607174	5	5	ENSSSCG00000004092	-	intron	---
1	17984509	17984509	+	17984509	3	3	---	---	intergenic	---
1	18462325	18462327	+	18462327	19	23	---	---	intergenic	---
1	18472246	18472248	+	18472247	14	24	XLOC_000153	+	predicted	---
1	18472758	18472767	+	18472765	13	39	XLOC_000153	+	predicted	---

1	18476654	18476655	-	18476654	3	6	ENSSSCG00000004101	-	3'UTR	*LRP11*
1	18478175	18478175	-	18478175	3	3	ENSSSCG00000004101	-	3'UTR	*LRP11*
1	18546907	18546907	+	18546907	3	3	---	---	intergenic	---
1	18571613	18571613	-	18571613	4	4	XLOC_002016	-	predicted	---
1	18591165	18591174	+	18591167	11	24	ENSSSCG00000004105	+	3'UTR	*NUP43*
1	18591248	18591250	+	18591248	6	11	ENSSSCG00000004105	+	3'UTR	*NUP43*
1	18591610	18591610	+	18591610	4	4	ENSSSCG00000004105	+	3'UTR	*NUP43*
1	18648120	18648120	-	18648120	3	3	XLOC_002017	-	predicted	---
1	18657447	18657453	-	18657448	10	23	ENSSSCG00000004104	-	3'UTR	*KATNA1*
1	18657533	18657534	-	18657534	14	22	ENSSSCG00000004104	-	3'UTR	*KATNA1*
1	18657877	18657877	+	18657877	3	3	ENSSSCG00000021892	+	intron	---
1	18698388	18698389	+	18698388	4	7	ENSSSCG00000004108	+	intron	---
							ENSSSCG00000021892	+	intron	---
							ENSSSCG00000028867	+	intron	*PPIL4*
1	18745998	18745998	-	18745998	5	5	ENSSSCG00000030067	-	3'UTR	*GINM1*
1	18746142	18746142	-	18746142	4	4	ENSSSCG00000030067	-	3'UTR	*GINM1*
1	18746266	18746266	+	18746266	3	3	ENSSSCG00000028867	+	intron	*PPIL4*
1	18746455	18746455	-	18746455	3	3	ENSSSCG00000030067	-	3'UTR	*GINM1*
1	18746511	18746511	-	18746511	5	5	ENSSSCG00000030067	-	3'UTR	*GINM1*
1	18763435	18763436	+	18763435	5	8	ENSSSCG00000028867	+	3'UTR	*PPIL4*
1	18777329	18777332	+	18777332	4	7	---	---	intergenic	---
1	18819625	18819631	+	18819625	3	6	ENSSSCG00000004109	+	3'UTR	*ZC3H12D*
1	18852318	18852318	-	18852318	3	3	XLOC_002020	-	predicted	---
1	18852399	18852400	-	18852399	3	6	ENSSSCG00000004110	-	3'UTR	*TAB2*
1	18878593	18878593	-	18878593	3	3	---	---	intergenic	---
1	19295053	19295053	-	19295053	3	3	ENSSSCG00000028974	-	3'UTR	*UST*

1	19306703	19306705	-	19306704	32	52	ENSSSCG00000028974	-	intron	*UST*
1	19805262	19805262	-	19805262	3	3	---	---	intergenic	---
1	19805389	19805419	-	19805389	13	17	---	---	intergenic	---
1	19994818	19994818	-	19994818	4	4	---	---	intergenic	---
1	20461006	20461006	-	20461006	4	4	ENSSSCG00000004112	-	intron	*SAMD5*
1	20492300	20492300	-	20492300	3	3	XLOC_002028	-	predicted	---
1	20492387	20492388	-	20492387	16	23	XLOC_002028	-	predicted	---
1	20528758	20528758	+	20528758	4	4	---	---	intergenic	---
1	20542906	20542906	-	20542906	5	5	ENSSSCG00000004113	-	3'UTR	---
1	20597244	20597244	-	20597244	3	3	ENSSSCG00000004113	-	intron	---
1	20819859	20819859	+	20819859	3	3	XLOC_000165	+	predicted	---
1	20874258	20874258	+	20874258	4	4	XLOC_000165	+	predicted	---
1	20954463	20954463	-	20954463	14	14	---	---	intergenic	---
1	20975181	20975181	+	20975181	3	3	---	---	intergenic	---
1	20986056	20986056	+	20986056	8	8	---	---	intergenic	---
1	21283446	21283446	+	21283446	4	4	XLOC_000166	+	predicted	---
1	21319321	21319323	-	21319322	25	38	ENSSSCG00000004117	-	3'UTR	*RAB32*
1	21319996	21320008	-	21320006	7	17	ENSSSCG00000004117	-	3'UTR	*RAB32*
1	21325202	21325202	-	21325202	3	3	ENSSSCG00000004117	-	intron	*RAB32*
1	21351408	21351408	+	21351408	3	3	---	---	intergenic	---
1	21938961	21938961	+	21938961	3	3	XLOC_000168	+	predicted	---
1	22014558	22014558	+	22014558	3	3	ENSSSCG00000004121	+	3'UTR	*FBXO30*
1	22016722	22016722	+	22016722	3	3	ENSSSCG00000004121	+	3'UTR	*FBXO30*
1	22999401	22999405	-	22999401	7	10	ENSSSCG00000004123	-	3'UTR	---
1	23567448	23567448	-	23567448	3	3	XLOC_002045	-	predicted	---
1	23569068	23569085	+	23569083	64	236	XLOC_000173	+	predicted	---

1	23710026	23710072	-	23710032	41	102	---	---	intergenic	---
1	23800734	23800735	-	23800734	10	18	ENSSSCG00000004132	-	intron	*PHACTR2*
1	23800761	23800762	+	23800761	6	10	ENSSSCG00000023897	+	intron	---
1	24149531	24149535	+	24149531	7	10	ENSSSCG00000004133	+	3'UTR	*FUCA2*
1	24150977	24150980	+	24150978	5	14	ENSSSCG00000004133	+	3'UTR	*FUCA2*
1	24154167	24154168	-	24154168	15	19	ENSSSCG00000004134	-	3'UTR	*PEX3*
1	24154772	24154781	-	24154773	10	21	ENSSSCG00000004134	-	3'UTR	*PEX3*
1	24210652	24210653	+	24210652	7	10	ENSSSCG00000004135	+	3'UTR	*ADAT2*
1	24300407	24300420	-	24300409	42	102	ENSSSCG00000004136	-	3'UTR	*AIG1*
1	24307008	24307015	-	24307010	11	25	ENSSSCG00000004136	-	intron	*AIG1*
1	24307945	24307945	-	24307945	9	9	ENSSSCG00000004136	-	intron	*AIG1*
1	24421258	24421258	+	24421258	3	3	---	---	intergenic	---
1	24496222	24496222	-	24496222	6	6	---	---	intergenic	---
1	25214235	25214235	+	25214235	7	7	---	---	intergenic	---
1	25588893	25588897	-	25588897	5	8	XLOC_002061	-	predicted	---
1	25664438	25664438	-	25664438	3	3	---	---	intergenic	---
1	26252340	26252340	-	26252340	3	3	---	---	intergenic	---
1	26511399	26511399	+	26511399	3	3	---	---	intergenic	---
1	26541905	26541905	+	26541905	5	5	---	---	intergenic	---
1	27669158	27669158	-	27669158	3	3	---	---	intergenic	---
1	28085801	28085801	-	28085801	10	10	---	---	intergenic	---
1	28297449	28297452	+	28297452	20	26	XLOC_000193	+	predicted	---
1	28427259	28427262	-	28427259	4	7	---	---	intergenic	---
1	28516823	28516823	-	28516823	3	3	ENSSSCG00000004144	-	3'UTR	*HECA*
1	28570441	28570442	-	28570441	15	18	ENSSSCG00000004145	-	3'UTR	*ABRACL*

1	28700497	28700503	+	28700503	6	9	ENSSSCG00000004146	+	3'UTR	*REPS1*
							ENSSSCG00000004146	+	ORF	---
1	28782283	28782289	-	28782283	14	20	ENSSSCG00000004148	-	3'UTR	*CCDC28A*
1	28782677	28782677	-	28782677	9	9	ENSSSCG00000004148	-	3'UTR	*CCDC28A*
1	28786779	28786779	-	28786779	3	3	ENSSSCG00000004148	-	intron	*CCDC28A*
1	28787301	28787301	-	28787301	4	4	ENSSSCG00000004148	-	intron	*CCDC28A*
1	28796077	28796077	-	28796077	3	3	ENSSSCG00000004148	-	ORF	*CCDC28A*
1	28975054	28975054	+	28975054	3	3	ENSSSCG00000004149	+	intron	*NHSL1*
1	29125402	29125402	+	29125402	3	3	ENSSSCG00000004149	+	3'UTR	*NHSL1*
1	29141350	29141350	-	29141350	4	4	ENSSSCG00000004150	-	3'UTR	*HEBP2*
1	29171050	29171052	-	29171050	23	51	---	---	intergenic	---
1	29611327	29611335	-	29611328	31	74	---	---	intergenic	---
1	29612562	29612562	-	29612562	4	4	XLOC_002079	-	predicted	---
1	29910615	29910615	+	29910615	4	4	---	---	intergenic	---
1	30278742	30278742	-	30278742	5	5	XLOC_002082	-	predicted	---
1	30409192	30409192	+	30409192	7	7	XLOC_000211	+	predicted	---
1	30441830	30441830	-	30441830	3	3	---	---	intergenic	---
1	30506332	30506332	+	30506332	3	3	---	---	intergenic	---
1	30995530	30995531	+	30995530	7	13	ENSSSCG00000026041	+	intron	---
1	31012413	31012413	+	31012413	3	3	XLOC_000216	+	predicted	---
1	31297849	31297853	-	31297853	9	15	XLOC_002089	-	predicted	---
1	31299161	31299169	-	31299161	9	23	XLOC_002089	-	predicted	---
1	31415616	31415631	+	31415624	13	78	ENSSSCG00000004165	+	intron	*PDE7B*
1	31446424	31446424	-	31446424	5	5	---	---	intergenic	---
1	31446452	31446456	+	31446452	6	16	---	---	intergenic	---
1	31470188	31470188	-	31470188	6	6	ENSSSCG00000004164	-	3'UTR	*MTFR2*

1	31490846	31490849	-	31490847	24	38	ENSSSCG00000004163	-	3'UTR	*BCLAF1*
1	31492355	31492355	-	31492355	3	3	ENSSSCG00000004163	-	3'UTR	*BCLAF1*
1	31493044	31493044	-	31493044	3	3	ENSSSCG00000004163	-	3'UTR	*BCLAF1*
1	31511903	31511903	-	31511903	4	4	ENSSSCG00000004163	-	ORF	*BCLAF1*
1	31522433	31522443	+	31522433	3	9	XLOC_000225	+	predicted	---
1	31524219	31524219	+	31524219	3	3	XLOC_000225	+	predicted	---
1	31644230	31644230	-	31644230	7	7	---	---	intergenic	---
1	31751692	31751692	-	31751692	3	3	---	---	intergenic	---
1	31765504	31765506	-	31765504	4	8	---	---	intergenic	---
1	32066902	32066902	+	32066902	4	4	ENSSSCG00000004166	+	intron	*AHI1*
1	32083963	32083969	+	32083964	87	142	ENSSSCG00000004166	+	intron	*AHI1*
1	32096050	32096050	+	32096050	3	3	ENSSSCG00000004166	+	intron	*AHI1*
1	32336689	32336693	+	32336693	62	108	---	---	intergenic	---
1	32404390	32404390	+	32404390	9	9	---	---	intergenic	---
1	32473326	32473326	+	32473326	10	10	ENSSSCG00000004169	+	ORF	*HBS1L*
1	32543595	32543598	+	32543598	9	18	XLOC_000230	+	predicted	---
1	32570483	32570483	+	32570483	3	3	---	---	intergenic	---
1	32768080	32768080	-	32768080	4	4	---	---	intergenic	---
1	32833284	32833284	-	32833284	3	3	---	---	intergenic	---
1	33256202	33256206	+	33256204	14	31	ENSSSCG00000021443	+	3'UTR	*SGK1*
1	33402500	33402500	-	33402500	4	4	ENSSSCG00000022387	-	3'UTR	*TBPL1*
1	33509017	33509017	+	33509017	3	3	---	---	intergenic	---
1	34000113	34000113	+	34000113	3	3	---	---	intergenic	---
1	34005794	34005795	-	34005795	13	19	---	---	intergenic	---
1	34065743	34065743	+	34065743	4	4	---	---	intergenic	---
1	34225296	34225299	-	34225299	13	24	---	---	intergenic	---

1	34225354	34225354	+	34225354	4	4	---	---	intergenic	---
1	34439072	34439089	-	34439084	1387	2552	ENSSSCG00000004177	-	3'UTR	*RPS12*
1	34441226	34441226	-	34441226	3	3	ENSSSCG00000004177	-	ORF	*RPS12*
1	34490383	34490383	+	34490383	4	4	ENSSSCG00000004179	+	3'UTR	*VNN2*
1	34512909	34512909	+	34512909	3	3	ENSSSCG00000004180	+	3'UTR	*VNN3*
1	34512995	34512997	+	34512995	16	27	ENSSSCG00000004180	+	3'UTR	*VNN3*
1	34544338	34544338	+	34544338	3	3	XLOC_000242	+	predicted	---
1	34545486	34545486	+	34545486	3	3	XLOC_000242	+	predicted	---
1	34757858	34757858	+	34757858	16	16	ENSSSCG00000004190	+	3'UTR	*STX7*
1	35073070	35073070	+	35073070	3	3	---	---	intergenic	---
1	35133820	35133822	-	35133822	10	16	ENSSSCG00000004192	-	3'UTR	*CTGF*
1	35134805	35134805	-	35134805	3	3	ENSSSCG00000004192	-	3'UTR	*CTGF*
1	35201835	35201835	+	35201835	3	3	---	---	intergenic	---
1	35243791	35243792	-	35243792	22	28	ENSSSCG00000004193	-	3'UTR	*ENPP1*
1	35243891	35243891	-	35243891	21	21	ENSSSCG00000004193	-	3'UTR	*ENPP1*
1	35594547	35594550	-	35594547	5	13	---	---	intergenic	---
1	35795821	35795821	-	35795821	4	4	ENSSSCG00000022533	-	3'UTR	---
1	36026325	36026325	+	36026325	3	3	ENSSSCG00000004199	+	3'UTR	---
1	36026384	36026384	+	36026384	3	3	ENSSSCG00000004199	+	3'UTR	---
1	36027414	36027416	+	36027415	19	32	ENSSSCG00000004199	+	3'UTR	---
1	36034944	36034955	-	36034949	18	55	XLOC_002136	-	predicted	---
1	36269524	36269528	+	36269527	11	38	---	---	intergenic	---
1	36632453	36632453	-	36632453	3	3	XLOC_002139	-	predicted	---
1	36974698	36974698	+	36974698	3	3	---	---	intergenic	---
1	37001645	37001650	-	37001650	26	37	---	---	intergenic	---
1	37043297	37043297	+	37043297	4	4	---	---	intergenic	---

1	37240069	37240077	-	37240070	5	20	ENSSSCG00000004207	-	intron	*LAMA2*
1	37264096	37264096	-	37264096	3	3	ENSSSCG00000004207	-	intron	*LAMA2*
1	37264145	37264155	-	37264155	12	15	ENSSSCG00000004207	-	intron	*LAMA2*
1	37361359	37361359	+	37361359	4	4	ENSSSCG00000004206	+	3'UTR	---
1	37443328	37443332	-	37443332	4	7	ENSSSCG00000025669	-	intron	---
							ENSSSCG00000004207	-	intron	*LAMA2*
1	37700691	37700691	-	37700691	4	4	---	---	intergenic	---
1	38301998	38301998	+	38301998	3	3	ENSSSCG00000004209	+	intron	*PTPRK*
1	38629224	38629224	+	38629224	3	3	ENSSSCG00000004209	+	intron	*PTPRK*
1	38690259	38690259	+	38690259	6	6	ENSSSCG00000004209	+	intron	*PTPRK*
1	38838654	38838654	+	38838654	4	4	ENSSSCG00000004209	+	3'UTR	*PTPRK*
1	38839333	38839333	+	38839333	3	3	ENSSSCG00000004209	+	3'UTR	*PTPRK*
1	39289119	39289119	-	39289119	3	3	---	---	intergenic	---
1	39568072	39568072	+	39568072	3	3	ENSSSCG00000004216	+	ORF	---
1	39592088	39592094	+	39592088	7	11	ENSSSCG00000004216	+	ORF	---
1	39592153	39592153	+	39592153	12	12	ENSSSCG00000004216	+	ORF	---
1	39592251	39592258	+	39592251	24	49	ENSSSCG00000004216	+	3'UTR	---
1	39592521	39592527	+	39592521	8	25	ENSSSCG00000004216	+	3'UTR	---
1	39593392	39593392	+	39593392	3	3	ENSSSCG00000004216	+	3'UTR	---
1	39593562	39593562	+	39593562	8	8	ENSSSCG00000004216	+	3'UTR	---
1	39594046	39594050	-	39594047	55	80	XLOC_002152	-	predicted	---
1	39594565	39594567	-	39594566	6	14	XLOC_002152	-	predicted	---
1	40025611	40025611	-	40025611	5	5	---	---	intergenic	---
1	40386561	40386561	-	40386561	5	5	---	---	intergenic	---
1	40459580	40459586	-	40459583	4	14	XLOC_002154	-	predicted	---
1	40713545	40713545	-	40713545	10	10	ENSSSCG00000004220	-	3'UTR	*TRMT11*

1	40777840	40777840	-	40777840	9	9	---	---	intergenic	---
1	40780782	40780782	-	40780782	4	4	ENSSSCG00000004221	-	3'UTR	*HINT3*
1	40782697	40782722	-	40782700	157	337	ENSSSCG00000004221	-	3'UTR	*HINT3*
1	40789375	40789375	-	40789375	5	5	ENSSSCG00000004221	-	ORF	*HINT3*
1	40815397	40815400	-	40815400	13	16	ENSSSCG00000004222	-	3'UTR	*NCOA7*
1	40891897	40891897	-	40891897	10	10	ENSSSCG00000004222	-	intron	*NCOA7*
1	40951720	40951720	-	40951720	3	3	ENSSSCG00000004222	-	intron	*NCOA7*
1	41154122	41154122	+	41154122	3	3	---	---	intergenic	---
1	41415263	41415265	+	41415265	22	29	ENSSSCG00000004224	+	3'UTR	*HDDC2*
1	41419353	41419353	+	41419353	5	5	---	---	intergenic	---
1	42100126	42100126	-	42100126	3	3	ENSSSCG00000029277	-	intron	*NKAIN2*
1	42531907	42531907	-	42531907	3	3	---	---	intergenic	---
1	42654353	42654353	-	42654353	3	3	---	---	intergenic	---
1	42894134	42894134	-	42894134	9	9	XLOC_002168	-	predicted	---
1	42902801	42902802	-	42902801	49	80	---	---	intergenic	---
1	43566219	43566219	+	43566219	4	4	XLOC_000295	+	predicted	---
1	43656576	43656576	+	43656576	6	6	---	---	intergenic	---
1	43912847	43912852	-	43912847	3	6	ENSSSCG00000004232	-	3'UTR	*CLVS2*
1	44139014	44139024	-	44139024	17	26	XLOC_002178	-	predicted	---
1	44139492	44139493	-	44139492	4	7	ENSSSCG00000004236	-	3'UTR	---
1	44139977	44139990	-	44139978	14	70	ENSSSCG00000004236	-	3'UTR	---
1	44140048	44140088	-	44140081	4	11	ENSSSCG00000004236	-	3'UTR	---
1	44145748	44145760	-	44145748	3	6	ENSSSCG00000004236	-	intron	---
1	44422944	44422944	+	44422944	3	3	---	---	intergenic	---
1	44446776	44446786	+	44446780	19	51	ENSSSCG00000004237	+	3'UTR	*SERINC1*
1	44452505	44452508	-	44452506	18	34	ENSSSCG00000004238	-	3'UTR	*HSF2*

1	44456453	44456453	-	44456453	9	9	ENSSSCG00000004238	-	intron	*HSF2*
1	44935482	44935482	+	44935482	7	7	---	---	intergenic	---
1	45031490	45031490	+	45031490	3	3	---	---	intergenic	---
1	45543887	45543896	-	45543894	15	21	ENSSSCG00000004241	-	3'UTR	*CONNEXIN*
1	45696283	45696285	+	45696285	6	12	ENSSSCG00000004242	+	intron	*TBC1D32*
1	45982977	45982977	-	45982977	9	9	---	---	intergenic	---
1	46018767	46018767	+	46018767	3	3	---	---	intergenic	---
1	46058825	46058825	+	46058825	7	7	---	---	intergenic	---
1	46179632	46179632	-	46179632	6	6	---	---	intergenic	---
1	46259146	46259146	+	46259146	7	7	---	---	intergenic	---
1	46313881	46313881	+	46313881	3	3	---	---	intergenic	---
1	46535108	46535108	-	46535108	4	4	---	---	intergenic	---
1	46818929	46818929	+	46818929	3	3	---	---	intergenic	---
1	47109225	47109227	-	47109227	5	9	---	---	intergenic	---
1	47119564	47119564	-	47119564	7	7	---	---	intergenic	---
1	47687406	47687421	-	47687416	65	128	XLOC_002190	-	predicted	---
1	47688687	47688688	-	47688687	26	37	ENSSSCG00000004243	-	ORF	---
1	47695716	47695716	-	47695716	3	3	---	---	intergenic	---
1	48059880	48059887	+	48059882	11	25	XLOC_000306	+	predicted	---
1	48258898	48258898	+	48258898	3	3	ENSSSCG00000004245	+	ORF	*FAM184A*
1	48263823	48263823	+	48263823	4	4	XLOC_000307	+	predicted	---
1	48352117	48352121	-	48352117	10	19	ENSSSCG00000004247	-	3'UTR	*ASF1A*
1	48497227	48497227	+	48497227	3	3	ENSSSCG00000030488	+	3'UTR	---
1	48566263	48566263	+	48566263	4	4	---	---	intergenic	---
1	48599210	48599210	+	48599210	5	5	---	---	intergenic	---
1	48614079	48614079	-	48614079	3	3	---	---	intergenic	---

1	48677542	48677542	-	48677542	3	3	XLOC_002195	-	predicted	---
1	48678936	48678936	-	48678936	4	4	ENSSSCG00000004248	-	3'UTR	*PLN*
1	48758448	48758448	+	48758448	8	8	ENSSSCG00000004249	+	intron	*CEP85L*
1	48780196	48780196	+	48780196	3	3	ENSSSCG00000004249	+	ORF	*CEP85L*
1	49138020	49138021	-	49138020	11	14	ENSSSCG00000004250	-	intron	*SLC35F1*
1	49203867	49203867	-	49203867	3	3	---	---	intergenic	---
1	49372811	49372811	+	49372811	3	3	---	---	intergenic	---
1	49503145	49503150	-	49503145	5	13	ENSSSCG00000020885	-	3'UTR	*NUS1*
1	49504381	49504381	-	49504381	10	10	ENSSSCG00000020885	-	3'UTR	*NUS1*
1	49627899	49627899	+	49627899	5	5	ENSSSCG00000004253	+	3'UTR	---
1	49629088	49629088	+	49629088	3	3	XLOC_000315	+	predicted	---
1	49637955	49637955	-	49637955	3	3	---	---	intergenic	---
1	49866545	49866545	-	49866545	3	3	---	---	intergenic	---
1	50234406	50234411	+	50234411	14	21	ENSSSCG00000004263	+	3'UTR	---
1	50511167	50511167	-	50511167	3	3	XLOC_002215	-	predicted	---
1	50519765	50519803	+	50519784	10	35	ENSSSCG00000025941	+	3'UTR	*PTP4A1*
1	50520189	50520189	+	50520189	11	11	ENSSSCG00000025941	+	3'UTR	*PTP4A1*
1	50520519	50520519	+	50520519	7	7	ENSSSCG00000025941	+	3'UTR	*PTP4A1*
1	50521037	50521051	+	50521037	35	133	ENSSSCG00000025941	+	3'UTR	*PTP4A1*
1	50521243	50521243	+	50521243	3	3	ENSSSCG00000025941	+	3'UTR	*PTP4A1*
1	50521597	50521603	+	50521598	23	60	ENSSSCG00000025941	+	3'UTR	*PTP4A1*
1	50522337	50522337	+	50522337	3	3	ENSSSCG00000025941	+	3'UTR	*PTP4A1*
1	50523101	50523108	+	50523101	3	6	ENSSSCG00000025941	+	3'UTR	*PTP4A1*
1	50988572	50988572	-	50988572	5	5	---	---	intergenic	---
1	51023898	51023898	+	51023898	3	3	---	---	intergenic	---
1	52573529	52573529	-	52573529	6	6	---	---	intergenic	---

1	52819937	52819937	-	52819937	3	3	---	---	intergenic	---
1	53441427	53441428	+	53441427	51	55	---	---	intergenic	---
1	53654751	53654752	+	53654752	6	10	---	---	intergenic	---
1	53676843	53676843	-	53676843	3	3	---	---	intergenic	---
1	53719457	53719457	+	53719457	3	3	---	---	intergenic	---
1	53733134	53733134	-	53733134	3	3	---	---	intergenic	---
1	53993828	53993828	+	53993828	3	3	---	---	intergenic	---
1	54116900	54116900	+	54116900	3	3	---	---	intergenic	---
1	55096485	55096486	+	55096486	5	8	---	---	intergenic	---
1	55672779	55672779	+	55672779	3	3	ENSSSCG00000004270	+	intron	*ADGRB3*
1	56034682	56034682	-	56034682	4	4	ENSSSCG00000004272	-	3'UTR	*LMBRD1*
1	56034783	56034783	-	56034783	5	5	ENSSSCG00000004272	-	3'UTR	*LMBRD1*
1	56034841	56034841	-	56034841	3	3	ENSSSCG00000004272	-	3'UTR	*LMBRD1*
1	56110350	56110350	-	56110350	4	4	ENSSSCG00000004272	-	intron	*LMBRD1*
1	56349399	56349405	+	56349399	8	13	ENSSSCG00000004273	+	intron	*COL19A1*
1	56695730	56695730	-	56695730	8	8	ENSSSCG00000004274	-	intron	*COL9A1*
1	56824080	56824080	+	56824080	3	3	---	---	intergenic	---
1	56989551	56989551	+	56989551	10	10	ENSSSCG00000004275	+	3'UTR	*FAM135A*
1	57009915	57009915	+	57009915	6	6	ENSSSCG00000026204	+	intron	*SDHAF4*
1	57044047	57044047	+	57044047	4	4	---	---	intergenic	---
1	57204304	57204304	+	57204304	4	4	ENSSSCG00000004276	+	ORF	*SMAP1*
1	57280048	57280051	+	57280048	10	25	ENSSSCG00000004276	+	3'UTR	*SMAP1*
1	57284548	57284548	-	57284548	4	4	ENSSSCG00000026246	-	intron	*B3GAT2*
1	57507477	57507478	+	57507478	4	7	---	---	intergenic	---
1	57561338	57561338	-	57561338	5	5	---	---	intergenic	---
1	57594749	57594751	+	57594749	24	32	---	---	intergenic	---

1	57665304	57665304	+	57665304	3	3	XLOC_000343	+	predicted	---
1	57670235	57670236	+	57670235	11	16	XLOC_000343	+	predicted	---
1	58630116	58630116	-	58630116	14	14	---	---	intergenic	---
1	58699213	58699216	-	58699213	4	8	---	---	intergenic	---
1	58827606	58827606	-	58827606	3	3	---	---	intergenic	---
1	58900954	58900954	+	58900954	3	3	---	---	intergenic	---
1	59004263	59004263	-	59004263	3	3	---	---	intergenic	---
1	59040708	59040708	-	59040708	8	8	---	---	intergenic	---
1	59153349	59153349	+	59153349	4	4	ENSSSCG00000004281	+	intron	*KCNQ5*
1	59281601	59281610	-	59281604	259	431	ENSSSCG00000004282	-	3'UTR	---
1	59359335	59359335	+	59359335	3	3	ENSSSCG00000004287	+	ORF	*DDX43*
1	59386958	59386958	+	59386958	4	4	ENSSSCG00000004287	+	intron	*DDX43*
1	59399637	59399637	+	59399637	4	4	ENSSSCG00000004287	+	intron	*DDX43*
1	59399777	59399779	+	59399778	8	16	ENSSSCG00000004287	+	intron	*DDX43*
1	59439607	59439609	-	59439607	6	9	---	---	intergenic	---
1	59439644	59439644	+	59439644	4	4	---	---	intergenic	---
1	59511234	59511236	+	59511236	6	12	ENSSSCG00000028526	+	intron	---
1	59662856	59662856	-	59662856	3	3	XLOC_002247	-	predicted	---
1	60031697	60031697	+	60031697	4	4	---	---	intergenic	---
1	60323508	60323508	-	60323508	5	5	---	---	intergenic	---
1	60810508	60810508	-	60810508	4	4	---	---	intergenic	---
1	60879524	60879524	+	60879524	6	6	ENSSSCG00000004291	+	3'UTR	*NT5E*
1	60881526	60881526	+	60881526	4	4	XLOC_000358	+	predicted	---
1	60888262	60888262	-	60888262	3	3	---	---	intergenic	---
1	60891437	60891446	-	60891438	31	83	XLOC_002251	-	predicted	---
1	60905611	60905611	-	60905611	6	6	ENSSSCG00000004293	-	ORF	*SNX14*

1	60989560	60989595	+	60989560	4	15	XLOC_000359	+	predicted	---
1	60993506	60993509	-	60993509	10	13	XLOC_002252	-	predicted	---
1	60996305	60996305	-	60996305	3	3	XLOC_002252	-	predicted	---
1	60996540	60996541	-	60996540	3	6	XLOC_002252	-	predicted	---
1	60996639	60996642	-	60996639	3	6	XLOC_002252	-	predicted	---
1	60997798	60997800	-	60997798	10	14	XLOC_002252	-	predicted	---
1	60997870	60997870	-	60997870	3	3	XLOC_002252	-	predicted	---
1	61007154	61007154	-	61007154	3	3	ENSSSCG00000004294	-	ORF	*SYNCRIP*
1	61013001	61013001	-	61013001	6	6	ENSSSCG00000004294	-	intron	*SYNCRIP*
1	61025469	61025469	-	61025469	3	3	ENSSSCG00000004294	-	ORF	*SYNCRIP*
1	61028358	61028358	-	61028358	28	28	---	---	intergenic	---
1	61059483	61059490	-	61059484	10	19	XLOC_002253	-	predicted	---
1	61061259	61061259	-	61061259	3	3	ENSSSCG00000019135	-	ORF	*SNORD50*
1	61115269	61115271	-	61115270	17	24	---	---	intergenic	---
1	62214073	62214073	+	62214073	10	10	---	---	intergenic	---
1	62216331	62216331	-	62216331	3	3	---	---	intergenic	---
1	62221275	62221275	+	62221275	3	3	---	---	intergenic	---
1	62245796	62245809	-	62245801	60	94	ENSSSCG00000025434	-	3'UTR	*CGA*
1	62304615	62304615	-	62304615	5	5	---	---	intergenic	---
1	62310614	62310614	-	62310614	4	4	---	---	intergenic	---
1	62455836	62455847	+	62455846	22	51	ENSSSCG00000004300	+	3'UTR	*SMIM8*
1	62455894	62455899	+	62455898	16	39	ENSSSCG00000004300	+	3'UTR	*SMIM8*
1	62456194	62456194	+	62456194	3	3	XLOC_000370	+	predicted	---
1	62459992	62459992	+	62459992	3	3	ENSSSCG00000004301	+	intron	*C6orf163*
1	62481550	62481552	+	62481550	13	16	XLOC_000370	+	predicted	---
1	62546064	62546064	+	62546064	22	22	XLOC_000371	+	predicted	---

1	62632608	62632608	+	62632608	4	4	XLOC_000373	+	predicted	---
1	62643336	62643336	-	62643336	9	9	---	---	intergenic	---
1	62680562	62680562	+	62680562	7	7	ENSSSCG00000004306	+	intron	*ORC3*
1	62786212	62786212	+	62786212	5	5	ENSSSCG00000004306	+	3'UTR	*ORC3*
1	62790463	62790463	-	62790463	3	3	ENSSSCG00000004307	-	3'UTR	*AKIRIN2*
1	62790524	62790524	-	62790524	12	12	ENSSSCG00000004307	-	3'UTR	*AKIRIN2*
1	62790860	62790913	-	62790912	40	143	ENSSSCG00000004307	-	3'UTR	*AKIRIN2*
1	62814450	62814450	+	62814450	4	4	---	---	intergenic	---
1	62852669	62852674	+	62852671	55	78	---	---	intergenic	---
1	63014278	63014278	+	63014278	3	3	XLOC_000375	+	predicted	---
1	63063359	63063359	-	63063359	3	3	---	---	intergenic	---
1	63076899	63076906	-	63076901	4	13	XLOC_002264	-	predicted	---
1	63092416	63092417	-	63092416	9	15	XLOC_002264	-	predicted	---
1	63092781	63092781	-	63092781	3	3	XLOC_002264	-	predicted	---
1	63092852	63092852	-	63092852	4	4	XLOC_002264	-	predicted	---
1	63110658	63110658	-	63110658	3	3	---	---	intergenic	---
1	63130046	63130061	+	63130061	12	21	XLOC_000376	+	predicted	---
1	63348195	63348195	+	63348195	3	3	---	---	intergenic	---
1	63364151	63364151	+	63364151	10	10	---	---	intergenic	---
1	63409051	63409051	+	63409051	6	6	ENSSSCG00000029003	+	ORF	*protein_coding*
1	63409130	63409157	+	63409157	4	13	ENSSSCG00000029003	+	ORF	*protein_coding*
1	63748553	63748553	-	63748553	3	3	ENSSSCG00000004311	-	ORF	---
1	63826234	63826241	-	63826238	66	130	XLOC_002271	-	predicted	---
1	63827852	63827852	+	63827852	3	3	XLOC_000379	+	predicted	---

1	63872006	63872006	-	63872006	5	5	ENSSSCG00000026249	-	ORF	*PNRC1*
1	63992655	63992655	+	63992655	5	5	XLOC_000380	+	predicted	---
1	63996616	63996616	+	63996616	6	6	XLOC_000380	+	predicted	---
1	64114979	64114979	-	64114979	4	4	XLOC_002277	-	predicted	---
1	64116658	64116666	-	64116658	6	21	XLOC_002277	-	predicted	---
1	64118446	64118455	-	64118447	35	82	ENSSSCG00000004317	-	3'UTR	*UBE2J1*
1	64151952	64151952	-	64151952	5	5	XLOC_002278	-	predicted	---
1	64154560	64154564	-	64154564	7	10	XLOC_002278	-	predicted	---
1	64479742	64479742	-	64479742	6	6	XLOC_002280	-	predicted	---
1	64481855	64481898	-	64481855	6	22	XLOC_002280	-	predicted	---
1	64551585	64551587	-	64551586	31	51	XLOC_002281	-	predicted	---
1	64671362	64671362	+	64671362	5	5	XLOC_000385	+	predicted	---
1	64811592	64811592	+	64811592	3	3	XLOC_000386	+	predicted	---
1	65123379	65123379	-	65123379	5	5	ENSSSCG00000004332	-	intron	*BACH2*
1	65414439	65414439	+	65414439	3	3	---	---	intergenic	---
1	65476823	65476823	-	65476823	9	9	XLOC_002290	-	predicted	---
1	65478965	65478965	-	65478965	4	4	---	---	intergenic	---
1	65479328	65479328	-	65479328	3	3	---	---	intergenic	---
1	65713904	65713904	-	65713904	10	10	---	---	intergenic	---
1	65954282	65954282	+	65954282	4	4	---	---	intergenic	---
1	65974928	65974928	+	65974928	4	4	---	---	intergenic	---
1	66072725	66072725	+	66072725	9	9	---	---	intergenic	---
1	66307098	66307098	+	66307098	8	8	---	---	intergenic	---
1	66770209	66770209	-	66770209	3	3	---	---	intergenic	---
1	67085465	67085465	-	67085465	5	5	---	---	intergenic	---
1	67111818	67111818	-	67111818	8	8	XLOC_002295	-	predicted	---

1	67568915	67568915	-	67568915	4	4	---	---	intergenic	---
1	69417080	69417080	-	69417080	3	3	---	---	intergenic	---
1	69621873	69621873	+	69621873	3	3	---	---	intergenic	---
1	69949670	69949670	+	69949670	4	4	---	---	intergenic	---
1	70264761	70264762	-	70264761	6	12	---	---	intergenic	---
1	71041626	71041626	+	71041626	4	4	ENSSSCG00000004337	+	ORF	*MANEA*
1	71082804	71082804	+	71082804	4	4	XLOC_000396	+	predicted	---
1	71084850	71084858	+	71084856	6	21	XLOC_000396	+	predicted	---
1	71338434	71338434	-	71338434	3	3	---	---	intergenic	---
1	72008963	72008963	+	72008963	3	3	ENSSSCG00000004339	+	ORF	---
1	72034176	72034176	+	72034176	4	4	ENSSSCG00000004339	+	ORF	---
1	72055339	72055339	+	72055339	5	5	ENSSSCG00000004339	+	ORF	---
1	72069311	72069320	+	72069320	17	30	ENSSSCG00000004339	+	3'UTR	---
1	72110428	72110430	+	72110430	5	11	ENSSSCG00000004340	+	intron	*FHL5*
1	72120825	72120833	+	72120826	52	108	ENSSSCG00000004340	+	3'UTR	*FHL5*
1	72300133	72300133	-	72300133	4	4	---	---	intergenic	---
1	72395399	72395399	-	72395399	5	5	---	---	intergenic	---
1	72407812	72407825	-	72407812	3	6	ENSSSCG00000004342	-	3'UTR	*NDUFAF4*
							ENSSSCG00000004342	-	intron	*NDUFAF4*
1	72446249	72446249	+	72446249	5	5	---	---	intergenic	---
1	72723036	72723036	+	72723036	7	7	ENSSSCG00000027002	+	3'UTR	---
1	72916693	72916693	-	72916693	3	3	---	---	intergenic	---
1	73287927	73287927	+	73287927	3	3	---	---	intergenic	---
1	73299162	73299162	+	73299162	4	4	---	---	intergenic	---
1	73347112	73347113	+	73347112	31	36	---	---	intergenic	---
1	74312075	74312075	+	74312075	3	3	---	---	intergenic	---

1	74422380	74422381	-	74422380	3	6	ENSSSCG00000004347	-	3'UTR	*FBXL4*
1	74500848	74500848	+	74500848	3	3	---	---	intergenic	---
1	74970420	74970421	+	74970421	18	31	ENSSSCG00000004349	+	intron	---
1	74975473	74975478	+	74975475	12	24	ENSSSCG00000004349	+	3'UTR	---
1	74975594	74975596	+	74975596	70	101	ENSSSCG00000004349	+	3'UTR	---
1	75037944	75037944	+	75037944	5	5	---	---	intergenic	---
1	75057161	75057162	+	75057161	5	8	---	---	intergenic	---
1	75063413	75063418	+	75063413	11	21	XLOC_000409	+	predicted	---
1	75141046	75141047	+	75141046	3	6	ENSSSCG00000029638	+	3'UTR	---
1	75144576	75144577	+	75144576	7	12	XLOC_000410	+	predicted	---
1	75149769	75149772	-	75149771	15	32	ENSSSCG00000004353	-	3'UTR	*CCNC*
1	75176066	75176066	+	75176066	3	3	---	---	intergenic	---
1	76107607	76107614	-	76107608	7	16	ENSSSCG00000004358	-	3'UTR	---
1	76147475	76147475	-	76147475	6	6	ENSSSCG00000004358	-	intron	---
1	76173627	76173627	+	76173627	3	3	---	---	intergenic	---
1	76386876	76386876	-	76386876	3	3	ENSSSCG00000004360	-	ORF	---
1	76393681	76393681	-	76393681	3	3	ENSSSCG00000004360	-	intron	---
1	77007481	77007481	+	77007481	3	3	---	---	intergenic	---
1	77578839	77578839	-	77578839	3	3	---	---	intergenic	---
1	77591547	77591547	+	77591547	8	8	---	---	intergenic	---
1	77955208	77955208	+	77955208	3	3	---	---	intergenic	---
1	78014453	78014453	-	78014453	15	15	---	---	intergenic	---
1	78103642	78103649	-	78103645	9	26	---	---	intergenic	---
1	78405518	78405518	+	78405518	30	30	---	---	intergenic	---
1	78507407	78507407	-	78507407	3	3	---	---	intergenic	---
1	78557878	78557878	-	78557878	5	5	---	---	intergenic	---

1	79501004	79501004	+	79501004	6	6	---	---	intergenic	---
1	80102298	80102298	+	80102298	3	3	---	---	intergenic	---
1	80233391	80233391	-	80233391	5	5	ENSSSCG00000004364	-	3'UTR	*HACE1*
1	80371501	80371501	-	80371501	9	9	ENSSSCG00000004364	-	intron	*HACE1*
1	80817008	80817011	-	80817011	7	11	ENSSSCG00000004368	-	3'UTR	*PREP*
1	80817049	80817049	-	80817049	7	7	ENSSSCG00000004368	-	3'UTR	*PREP*
1	81040963	81040963	-	81040963	4	4	---	---	intergenic	---
1	81111665	81111666	-	81111666	4	7	---	---	intergenic	---
1	81154052	81154052	+	81154052	4	4	---	---	intergenic	---
1	81343872	81343873	-	81343872	5	8	---	---	intergenic	---
1	81349979	81349984	+	81349982	111	197	---	---	intergenic	---
1	81409812	81409813	-	81409812	3	6	---	---	intergenic	---
1	81605877	81605877	-	81605877	3	3	---	---	intergenic	---
1	81609737	81609750	-	81609738	32	47	---	---	intergenic	---
1	81609791	81609793	-	81609791	3	9	---	---	intergenic	---
1	81670573	81670573	-	81670573	3	3	XLOC_002338	-	predicted	---
1	81720136	81720136	+	81720136	4	4	---	---	intergenic	---
1	82012304	82012304	+	82012304	3	3	ENSSSCG00000004371	+	intron	*AIM1*
1	82060051	82060053	+	82060053	7	12	ENSSSCG00000004371	+	3'UTR	*AIM1*
1	82062557	82062559	-	82062557	20	31	ENSSSCG00000004373	-	3'UTR	---
1	82111345	82111345	-	82111345	3	3	ENSSSCG00000004373	-	intron	---
1	82143969	82143971	+	82143969	4	8	ENSSSCG00000004374	+	3'UTR	---
1	82144032	82144032	+	82144032	6	6	ENSSSCG00000004374	+	3'UTR	---
1	82202278	82202278	-	82202278	6	6	---	---	intergenic	---
1	82273304	82273304	+	82273304	15	15	XLOC_000438	+	predicted	---
1	82314068	82314068	+	82314068	3	3	---	---	intergenic	---

1	82526150	82526150	-	82526150	10	10	XLOC_002344	-	predicted	---
1	82566114	82566116	-	82566115	48	60	---	---	intergenic	---
1	82585599	82585599	-	82585599	3	3	---	---	intergenic	---
1	83124533	83124536	+	83124535	22	50	XLOC_000443	+	predicted	---
1	83124641	83124641	+	83124641	3	3	XLOC_000443	+	predicted	---
1	83170335	83170342	-	83170339	38	79	ENSSSCG00000004381	-	intron	*SCML4*
1	83274127	83274130	-	83274129	38	67	ENSSSCG00000004382	-	3'UTR	*SEC63*
1	83274800	83274803	-	83274801	18	33	ENSSSCG00000004382	-	3'UTR	*SEC63*
1	83274972	83274980	-	83274973	20	42	ENSSSCG00000004382	-	3'UTR	*SEC63*
1	83289161	83289161	-	83289161	5	5	ENSSSCG00000004382	-	intron	*SEC63*
							ENSSSCG00000004382	-	ORF	*SEC63*
1	83291290	83291290	-	83291290	3	3	ENSSSCG00000004382	-	ORF	*SEC63*
1	83293013	83293018	-	83293018	5	11	ENSSSCG00000004382	-	intron	*SEC63*
1	83300062	83300062	-	83300062	5	5	ENSSSCG00000004382	-	ORF	*SEC63*
1	83322970	83322970	-	83322970	3	3	ENSSSCG00000004382	-	ORF	*SEC63*
1	83410032	83410036	-	83410036	16	22	ENSSSCG00000004383	-	3'UTR	*GL*
1	83436478	83436479	-	83436478	7	11	ENSSSCG00000004383	-	intron	*GL*
1	83562971	83562971	+	83562971	3	3	ENSSSCG00000004384	+	3'UTR	*NR2E1*
1	83576736	83576749	-	83576742	49	167	ENSSSCG00000004385	-	3'UTR	*SNX3*
1	83577057	83577068	-	83577058	28	115	ENSSSCG00000004385	-	3'UTR	*SNX3*
1	83577199	83577199	-	83577199	3	3	ENSSSCG00000004385	-	3'UTR	*SNX3*
1	83655224	83655226	+	83655224	38	46	ENSSSCG00000024176	+	intron	---
1	83847161	83847161	+	83847161	3	3	XLOC_000449	+	predicted	---
1	83847303	83847306	+	83847304	14	26	XLOC_000449	+	predicted	---
1	83848065	83848065	+	83848065	6	6	XLOC_000449	+	predicted	---
1	83983409	83983409	+	83983409	4	4	ENSSSCG00000004387	+	intron	*FOXO3A*

1	84000574	84000574	+	84000574	3	3	ENSSSCG00000004387	+	intron	*FOXO3A*
1	84029803	84029804	+	84029803	5	8	ENSSSCG00000004387	+	intron	*FOXO3A*
1	84070783	84070793	+	84070787	13	27	XLOC_000450	+	predicted	---
1	84258700	84258705	+	84258705	24	36	ENSSSCG00000004388	+	intron	*ARMC2*
1	84342421	84342421	+	84342421	9	9	XLOC_000451	+	predicted	---
1	84357132	84357132	-	84357132	16	16	XLOC_002358	-	predicted	---
1	84491380	84491380	-	84491380	3	3	---	---	intergenic	---
1	84556827	84556827	+	84556827	4	4	ENSSSCG00000004391	+	intron	*CEP57L1*
1	84559240	84559240	+	84559240	3	3	XLOC_000454	+	predicted	---
1	84626923	84626925	-	84626924	15	22	---	---	intergenic	---
1	84719537	84719542	-	84719540	91	193	ENSSSCG00000004414	-	3'UTR	*CD164*
1	84719595	84719595	-	84719595	6	6	ENSSSCG00000004414	-	3'UTR	*CD164*
1	84720295	84720295	-	84720295	4	4	ENSSSCG00000004414	-	3'UTR	*CD164*
1	84721353	84721368	-	84721365	18	48	ENSSSCG00000004414	-	3'UTR	*CD164*
1	84721661	84721663	-	84721662	11	20	ENSSSCG00000004414	-	3'UTR	*CD164*
1	84721814	84721814	-	84721814	19	19	ENSSSCG00000004414	-	3'UTR	*CD164*
1	84735589	84735589	-	84735589	9	9	---	---	intergenic	---
1	84743268	84743278	-	84743269	31	55	ENSSSCG00000004413	-	3'UTR	*PPIL6*
1	84743381	84743381	-	84743381	5	5	ENSSSCG00000004413	-	ORF	*PPIL6*
1	84789538	84789543	+	84789541	81	131	ENSSSCG00000004408	+	3'UTR	*SMPD2*
1	84877630	84877630	-	84877630	4	4	---	---	intergenic	---
1	85160408	85160423	-	85160420	9	69	---	---	intergenic	---
1	85452465	85452467	-	85452467	18	25	ENSSSCG00000004405	-	3'UTR	*FIG4*
1	85605938	85605938	-	85605938	4	4	ENSSSCG00000004404	-	intron	*WASF1*
1	85687257	85687257	+	85687257	6	6	ENSSSCG00000004403	+	intron	*CDC40*
1	85714990	85714990	+	85714990	3	3	ENSSSCG00000004403	+	3'UTR	*CDC40*

1	85718063	85718063	+	85718063	4	4	---	---	intergenic	---
1	85815389	85815390	+	85815390	6	9	---	---	intergenic	---
1	85867147	85867151	-	85867148	6	14	ENSSSCG00000004401	-	3'UTR	*DDO*
1	86020551	86020556	-	86020551	3	6	---	---	intergenic	---
1	86102316	86102316	-	86102316	13	13	XLOC_002378	-	predicted	---
1	86105430	86105430	-	86105430	3	3	XLOC_002378	-	predicted	---
1	86117049	86117049	+	86117049	8	8	---	---	intergenic	---
1	86304318	86304318	-	86304318	3	3	XLOC_002382	-	predicted	---
1	86305737	86305737	-	86305737	18	18	ENSSSCG00000004394	-	ORF	*RPF2*
1	86353217	86353225	-	86353225	26	63	ENSSSCG00000004393	-	3'UTR	*GTF3C6*
1	86353465	86353465	-	86353465	3	3	ENSSSCG00000004393	-	3'UTR	*GTF3C6*
1	86365684	86365684	-	86365684	3	3	---	---	intergenic	---
1	86444288	86444288	-	86444288	3	3	XLOC_002387	-	predicted	---
1	86452590	86452590	-	86452590	7	7	---	---	intergenic	---
1	86467167	86467167	+	86467167	5	5	---	---	intergenic	---
1	86670543	86670543	-	86670543	3	3	XLOC_002388	-	predicted	---
1	86679203	86679203	-	86679203	3	3	XLOC_002388	-	predicted	---
1	86679740	86679740	-	86679740	4	4	XLOC_002388	-	predicted	---
1	86694614	86694615	-	86694614	6	9	---	---	intergenic	---
1	86940516	86940527	-	86940523	30	112	ENSSSCG00000025439	-	intron	---
1	86965903	86965908	+	86965907	18	46	XLOC_000470	+	predicted	---
1	86979022	86979022	+	86979022	3	3	XLOC_000470	+	predicted	---
1	86986586	86986592	+	86986586	7	12	XLOC_000470	+	predicted	---
1	87048043	87048043	-	87048043	7	7	ENSSSCG00000004420	-	3'UTR	*TRAF3IP2*
1	87071943	87071943	-	87071943	15	15	ENSSSCG00000004420	-	intron	*TRAF3IP2*
1	87282779	87282779	-	87282779	5	5	ENSSSCG00000004421	-	intron	*FYN*

1	87389136	87389136	-	87389136	44	44	ENSSSCG00000004421	-	intron	*FYN*
1	87543005	87543005	-	87543005	13	13	---	---	intergenic	---
1	87731969	87731984	+	87731969	9	16	ENSSSCG00000004424	+	ORF	*FAM229B*
1	87732083	87732098	+	87732094	338	1103	XLOC_000474	+	predicted	---
1	87740893	87740917	-	87740917	4	7	ENSSSCG00000004425	-	3'UTR	---
1	88200936	88200936	-	88200936	12	12	---	---	intergenic	---
1	88724956	88724956	-	88724956	3	3	---	---	intergenic	---
1	88852890	88852890	-	88852890	4	4	---	---	intergenic	---
1	89322501	89322501	+	89322501	4	4	---	---	intergenic	---
1	89751557	89751557	+	89751557	3	3	---	---	intergenic	---
1	89855851	89855852	+	89855851	37	40	ENSSSCG00000023610	+	3'UTR	*MARCKS*
1	89857393	89857393	+	89857393	5	5	ENSSSCG00000023610	+	3'UTR	*MARCKS*
1	89857528	89857529	+	89857529	12	23	ENSSSCG00000023610	+	3'UTR	*MARCKS*
1	89929201	89929211	-	89929207	21	55	ENSSSCG00000004432	-	3'UTR	*HDAC2*
1	90150451	90150451	+	90150451	4	4	---	---	intergenic	---
1	90173549	90173549	+	90173549	3	3	---	---	intergenic	---
1	90199961	90199961	+	90199961	4	4	---	---	intergenic	---
1	90204590	90204590	-	90204590	3	3	---	---	intergenic	---
1	91251709	91251709	-	91251709	3	3	---	---	intergenic	---
1	91624700	91624700	-	91624700	4	4	---	---	intergenic	---
1	91624765	91624765	+	91624765	24	24	---	---	intergenic	---
1	91625387	91625389	-	91625387	6	12	---	---	intergenic	---
1	91696176	91696176	+	91696176	5	5	XLOC_000481	+	predicted	---
1	91698944	91698944	+	91698944	3	3	XLOC_000481	+	predicted	---
1	91997369	91997369	+	91997369	4	4	ENSSSCG00000004435	+	3'UTR	*NT5DC1*
1	92008063	92008063	-	92008063	4	4	ENSSSCG00000004439	-	ORF	*TSPYL4*

1	92021815	92021819	-	92021818	42	58	XLOC_002407	-	predicted	---
1	92240074	92240074	-	92240074	3	3	---	---	intergenic	---
1	92383886	92383894	-	92383888	71	151	---	---	intergenic	---
1	92403428	92403428	+	92403428	8	8	ENSSSCG00000004446	+	3'UTR	*RWDD1*
1	92500992	92500992	+	92500992	3	3	ENSSSCG00000025656	+	intron	---
1	92502570	92502570	+	92502570	3	3	XLOC_000494	+	predicted	---
1	92502650	92502657	+	92502656	71	174	XLOC_000494	+	predicted	---
1	92502804	92502805	+	92502804	3	6	XLOC_000494	+	predicted	---
1	93050522	93050522	-	93050522	3	3	XLOC_002418	-	predicted	---
1	93183863	93183863	+	93183863	7	7	ENSSSCG00000004454	+	intron	*ME1*
1	93240322	93240322	+	93240322	3	3	ENSSSCG00000004454	+	3'UTR	*ME1*
1	93249515	93249516	-	93249516	19	22	XLOC_002420	-	predicted	---
1	93251804	93251804	-	93251804	9	9	ENSSSCG00000029129	-	3'UTR	*RWDD2A*
1	93280918	93280920	+	93280919	19	28	ENSSSCG00000004456	+	3'UTR	*PGM3*
1	93281354	93281354	-	93281354	4	4	ENSSSCG00000004457	-	3'UTR	*DOPEY1*
1	93412564	93412565	-	93412565	26	40	ENSSSCG00000004457	-	intron	*DOPEY1*
1	93442711	93442712	+	93442711	10	14	XLOC_000499	+	predicted	---
1	93499565	93499565	-	93499565	6	6	---	---	intergenic	---
1	93499928	93499948	-	93499928	20	50	---	---	intergenic	---
1	94010484	94010484	-	94010484	3	3	---	---	intergenic	---
1	94609695	94609705	+	94609698	20	52	ENSSSCG00000004460	+	3'UTR	*IBTK*
1	94888437	94888437	-	94888437	3	3	---	---	intergenic	---
1	95051184	95051184	-	95051184	3	3	---	---	intergenic	---
1	95111381	95111381	+	95111381	4	4	XLOC_000502	+	predicted	---
1	96350625	96350631	-	96350627	97	137	XLOC_002429	-	predicted	---
1	96398372	96398372	+	96398372	5	5	---	---	intergenic	---

1	96728007	96728007	-	96728007	6	6	---	---	intergenic	---
1	96780830	96780831	-	96780831	6	9	XLOC_002430	-	predicted	---
1	96781968	96781972	-	96781968	7	11	XLOC_002430	-	predicted	---
1	96896053	96896053	+	96896053	3	3	ENSSSCG00000028080	+	3'UTR	---
1	97046069	97046069	-	97046069	5	5	---	---	intergenic	---
1	97113316	97113333	-	97113316	19	72	XLOC_002431	-	predicted	---
1	97114514	97114514	-	97114514	5	5	XLOC_002431	-	predicted	---
1	97175084	97175084	-	97175084	3	3	---	---	intergenic	---
1	97275586	97275586	-	97275586	3	3	XLOC_002432	-	predicted	---
1	97308197	97308198	+	97308197	5	10	---	---	intergenic	---
1	97476660	97476660	+	97476660	25	25	---	---	intergenic	---
1	97549483	97549483	-	97549483	3	3	---	---	intergenic	---
1	97662031	97662031	-	97662031	7	7	---	---	intergenic	---
1	97758647	97758647	+	97758647	3	3	ENSSSCG00000004470	+	3'UTR	*HMGN3*
1	97758718	97758719	+	97758719	10	15	ENSSSCG00000004470	+	3'UTR	*HMGN3*
1	97758925	97758951	+	97758949	126	233	ENSSSCG00000004470	+	3'UTR	*HMGN3*
1	97785274	97785276	+	97785274	68	102	---	---	intergenic	---
1	98020138	98020138	+	98020138	8	8	XLOC_000513	+	predicted	---
1	98057042	98057043	-	98057043	4	7	ENSSSCG00000004472	-	3'UTR	*IRAK1BP1*
1	98057229	98057245	-	98057244	17	45	ENSSSCG00000004472	-	3'UTR	*IRAK1BP1*
1	98057461	98057461	-	98057461	10	10	ENSSSCG00000004472	-	3'UTR	*IRAK1BP1*
1	98057635	98057661	-	98057635	7	10	ENSSSCG00000004472	-	ORF	*IRAK1BP1*
1	98107555	98107555	+	98107555	3	3	XLOC_000514	+	predicted	---
1	98453173	98453173	-	98453173	3	3	---	---	intergenic	---
1	98865802	98865802	+	98865802	6	6	---	---	intergenic	---
1	99070987	99071006	-	99070987	6	13	---	---	intergenic	---

1	99071615	99071615	+	99071615	6	6	---	---	intergenic	---
1	99071664	99071677	+	99071664	7	16	---	---	intergenic	---
1	99071747	99071748	+	99071748	12	16	---	---	intergenic	---
1	99071893	99071893	-	99071893	3	3	---	---	intergenic	---
1	99072133	99072145	+	99072136	46	110	---	---	intergenic	---
1	99074066	99074066	+	99074066	4	4	---	---	intergenic	---
1	99318859	99318859	+	99318859	3	3	---	---	intergenic	---
1	99342277	99342277	+	99342277	3	3	---	---	intergenic	---
1	99387033	99387033	+	99387033	4	4	---	---	intergenic	---
1	99416476	99416476	-	99416476	5	5	---	---	intergenic	---
1	99560835	99560835	+	99560835	4	4	---	---	intergenic	---
1	99624600	99624602	+	99624600	12	16	---	---	intergenic	---
1	99860588	99860588	-	99860588	4	4	---	---	intergenic	---
1	99913314	99913315	+	99913315	4	7	---	---	intergenic	---
1	99971673	99971673	-	99971673	3	3	---	---	intergenic	---
1	100114635	100114635	-	100114635	3	3	---	---	intergenic	---
1	100511374	100511374	-	100511374	4	4	---	---	intergenic	---
1	100519049	100519068	-	100519068	32	77	---	---	intergenic	---
1	100966082	100966087	+	100966087	7	11	XLOC_000519	+	predicted	---
1	100972531	100972531	-	100972531	3	3	ENSSSCG00000004478	-	3'UTR	*MYO6*
1	101173639	101173641	-	101173639	3	6	ENSSSCG00000020702	-	3'UTR	*SENP6*
1	101198684	101198684	-	101198684	3	3	ENSSSCG00000020702	-	intron	*SENP6*
1	101261357	101261357	+	101261357	3	3	ENSSSCG00000028780	+	intron	---
1	101658490	101658490	-	101658490	4	4	XLOC_002446	-	predicted	---
1	101659188	101659188	-	101659188	11	11	XLOC_002446	-	predicted	---
1	101758593	101758594	+	101758594	15	22	ENSSSCG00000004484	+	intron	---

1	101781734	101781734	+	101781734	3	3	ENSSSCG00000004484	+	intron	---
1	101816756	101816780	-	101816759	1448	3130	XLOC_002448	-	predicted	---
1	101823405	101823410	+	101823405	4	10	ENSSSCG00000004484	+	intron	---
1	101824282	101824287	+	101824286	17	24	ENSSSCG00000004484	+	intron	---
1	101889124	101889124	+	101889124	3	3	ENSSSCG00000004484	+	intron	---
1	101890878	101890886	+	101890886	9	12	XLOC_000524	+	predicted	---
1	102197716	102197716	+	102197716	3	3	---	---	intergenic	---
1	102254522	102254522	+	102254522	4	4	---	---	intergenic	---
1	103137007	103137007	-	103137007	3	3	ENSSSCG00000004485	-	3'UTR	*CD109*
1	103473713	103473713	-	103473713	3	3	---	---	intergenic	---
1	103475668	103475668	+	103475668	11	11	ENSSSCG00000004489	+	ORF	*EEF1A*
1	103475970	103475970	+	103475970	7	7	ENSSSCG00000004489	+	ORF	*EEF1A*
1	103476313	103476391	+	103476388	9	37	ENSSSCG00000004489	+	ORF	*EEF1A*
1	103477010	103477015	+	103477015	54	71	ENSSSCG00000004489	+	ORF	*EEF1A*
1	103477236	103477268	+	103477268	12	29	ENSSSCG00000004489	+	ORF	*EEF1A*
1	103477731	103477746	+	103477742	14	21	ENSSSCG00000004489	+	3'UTR	*EEF1A*
1	103477883	103477893	+	103477890	550	1816	ENSSSCG00000004489	+	3'UTR	*EEF1A*
1	103477954	103477954	+	103477954	3	3	XLOC_000530	+	predicted	---
1	103478706	103478718	+	103478706	6	20	XLOC_000530	+	predicted	---
1	103499999	103500002	-	103500002	4	7	XLOC_002452	-	predicted	---
1	103727513	103727513	+	103727513	5	5	---	---	intergenic	---
1	103887157	103887157	-	103887157	9	9	---	---	intergenic	---
1	104780051	104780052	+	104780051	3	6	---	---	intergenic	---
1	105304120	105304121	-	105304121	11	18	---	---	intergenic	---
1	105556016	105556016	-	105556016	5	5	ENSSSCG00000004494	-	3'UTR	---
1	105810597	105810599	-	105810597	9	12	ENSSSCG00000004497	-	3'UTR	*PSTPIP2*

1	105913277	105913280	-	105913277	9	17	---	---	intergenic	---
1	105919777	105919778	-	105919778	6	9	ENSSSCG00000004498	-	3'UTR	*HAUS1*
1	105919932	105919933	-	105919933	8	11	ENSSSCG00000004498	-	3'UTR	*HAUS1*
1	105950069	105950081	+	105950069	8	11	ENSSSCG00000004499	+	ORF	*ATP5A1*
1	105950449	105950449	+	105950449	6	6	ENSSSCG00000004499	+	intron	*ATP5A1*
1	105955784	105955796	+	105955794	296	579	ENSSSCG00000004499	+	3'UTR	*ATP5A1*
1	106076649	106076649	+	106076649	3	3	XLOC_000539	+	predicted	---
1	106255973	106256013	+	106256008	37	96	XLOC_000541	+	predicted	---
1	106612125	106612125	+	106612125	6	6	---	---	intergenic	---
1	106994911	106994911	+	106994911	15	15	XLOC_000544	+	predicted	---
1	107085250	107085250	+	107085250	4	4	---	---	intergenic	---
1	107345979	107345979	-	107345979	3	3	---	---	intergenic	---
1	107471935	107471935	-	107471935	4	4	---	---	intergenic	---
1	107547830	107547830	+	107547830	3	3	---	---	intergenic	---
1	107589674	107589674	-	107589674	14	14	---	---	intergenic	---
1	107589696	107589696	+	107589696	3	3	---	---	intergenic	---
1	107679567	107679568	+	107679568	6	11	ENSSSCG00000004506	+	intron	*CTIF*
1	107965064	107965064	+	107965064	10	10	ENSSSCG00000004506	+	intron	*CTIF*
1	108216516	108216516	-	108216516	3	3	XLOC_002473	-	predicted	---
1	108216907	108216915	-	108216913	23	85	ENSSSCG00000004508	-	3'UTR	---
1	108372657	108372657	+	108372657	3	3	---	---	intergenic	---
1	108439090	108439103	-	108439098	26	84	XLOC_002475	-	predicted	---
1	108439147	108439155	-	108439147	3	6	XLOC_002475	-	predicted	---
1	108439438	108439468	-	108439467	40	67	XLOC_002475	-	predicted	---
1	108439667	108439691	-	108439683	8	30	XLOC_002475	-	predicted	---
1	108442193	108442193	-	108442193	4	4	---	---	intergenic	---

1	108447067	108447067	-	108447067	3	3	XLOC_002476	-	predicted	---
1	108447409	108447411	-	108447410	54	84	XLOC_002476	-	predicted	---
1	108447465	108447465	-	108447465	3	3	XLOC_002476	-	predicted	---
1	108447510	108447530	-	108447511	59	178	XLOC_002476	-	predicted	---
1	108448320	108448346	-	108448345	11	20	ENSSSCG00000029642	-	ORF	*RPL17*
1	108450569	108450569	-	108450569	6	6	ENSSSCG00000029642	-	ORF	*RPL17*
1	108457568	108457570	+	108457569	10	20	---	---	intergenic	---
1	108648335	108648335	+	108648335	4	4	ENSSSCG00000004509	+	intron	*LIPG*
1	108667379	108667379	+	108667379	3	3	XLOC_000550	+	predicted	---
1	108679014	108679014	+	108679014	4	4	---	---	intergenic	---
1	108842621	108842633	-	108842627	178	389	ENSSSCG00000004510	-	3'UTR	*ACAA2*
1	108842675	108842675	-	108842675	3	3	ENSSSCG00000004510	-	3'UTR	*ACAA2*
1	108870853	108870853	+	108870853	3	3	---	---	intergenic	---
1	109021577	109021587	-	109021578	35	84	ENSSSCG00000004511	-	3'UTR	*MYO5B*
1	109431235	109431235	+	109431235	7	7	---	---	intergenic	---
1	109483879	109483880	+	109483879	3	6	---	---	intergenic	---
1	109565529	109565529	-	109565529	3	3	ENSSSCG00000027690	-	3'UTR	---
1	109589782	109589782	-	109589782	4	4	XLOC_002486	-	predicted	---
1	109589956	109589956	-	109589956	5	5	XLOC_002486	-	predicted	---
1	109591025	109591052	-	109591025	4	7	XLOC_002486	-	predicted	---
1	109623498	109623507	-	109623498	6	9	ENSSSCG00000004517	-	3'UTR	*CXXC1*
1	109625930	109625930	-	109625930	3	3	ENSSSCG00000004517	-	ORF	*CXXC1*
1	109689049	109689054	+	109689052	4	14	ENSSSCG00000004518	+	intron	*SKA1*
1	109732839	109732839	+	109732839	5	5	XLOC_000558	+	predicted	---
1	110169087	110169087	-	110169087	8	8	---	---	intergenic	---
1	110351245	110351245	+	110351245	3	3	ENSSSCG00000004522	+	ORF	*ME2*

1	110410387	110410387	+	110410387	3	3	XLOC_000562	+	predicted	---
1	110410790	110410796	+	110410796	17	20	XLOC_000562	+	predicted	---
1	110563186	110563188	+	110563187	7	15	ENSSSCG00000004524	+	3'UTR	*MADH4*
1	110568652	110568656	+	110568652	5	8	XLOC_000563	+	predicted	---
1	110611809	110611815	-	110611815	7	12	XLOC_002495	-	predicted	---
1	110613442	110613455	-	110613448	40	83	XLOC_002495	-	predicted	---
1	110613523	110613534	-	110613523	15	31	XLOC_002495	-	predicted	---
1	110614448	110614448	-	110614448	5	5	ENSSSCG00000004525	-	ORF	*MEX3C*
1	111132918	111132918	+	111132918	3	3	---	---	intergenic	---
1	111644574	111644576	-	111644575	35	52	---	---	intergenic	---
1	112590613	112590613	-	112590613	3	3	---	---	intergenic	---
1	113103000	113103012	+	113103000	5	16	---	---	intergenic	---
1	113406047	113406048	+	113406048	10	14	---	---	intergenic	---
1	113947944	113947948	-	113947946	8	16	XLOC_002497	-	predicted	---
1	113948058	113948062	-	113948062	8	12	XLOC_002497	-	predicted	---
1	114047426	114047426	-	114047426	3	3	ENSSSCG00000004528	-	intron	*MBD2*
1	114127155	114127155	+	114127155	5	5	XLOC_000570	+	predicted	---
1	114142461	114142461	-	114142461	18	18	XLOC_002498	-	predicted	---
1	114142616	114142616	-	114142616	4	4	XLOC_002498	-	predicted	---
1	114142659	114142659	-	114142659	3	3	XLOC_002498	-	predicted	---
1	114174196	114174201	+	114174199	7	17	ENSSSCG00000004531	+	ORF	*C18orf54*
1	114188681	114188683	+	114188682	8	15	ENSSSCG00000004531	+	intron	*C18orf54*
1	114189869	114189869	+	114189869	3	3	ENSSSCG00000004531	+	intron	*C18orf54*
1	114345787	114345787	-	114345787	4	4	---	---	intergenic	---
1	114759904	114759904	+	114759904	3	3	XLOC_000573	+	predicted	---
1	114925500	114925500	-	114925500	5	5	---	---	intergenic	---

1	115082221	115082221	-	115082221	3	3	---	---	intergenic	---
1	115106423	115106423	-	115106423	14	14	---	---	intergenic	---
1	115113215	115113215	-	115113215	3	3	XLOC_002501	-	predicted	---
1	115113475	115113478	+	115113477	25	37	XLOC_000576	+	predicted	---
1	115211681	115211681	-	115211681	3	3	ENSSSCG00000004535	-	intron	*TCF4*
1	115234377	115234377	+	115234377	3	3	---	---	intergenic	---
1	115454077	115454080	-	115454078	7	19	---	---	intergenic	---
1	115589219	115589219	+	115589219	3	3	---	---	intergenic	---
1	115589301	115589301	-	115589301	3	3	---	---	intergenic	---
1	115589349	115589350	+	115589350	8	11	---	---	intergenic	---
1	115589583	115589583	+	115589583	3	3	---	---	intergenic	---
1	115589600	115589602	-	115589602	33	46	---	---	intergenic	---
1	115590448	115590461	-	115590461	74	416	---	---	intergenic	---
1	115698750	115698750	-	115698750	3	3	---	---	intergenic	---
1	115866678	115866680	-	115866679	10	18	---	---	intergenic	---
1	116211398	116211398	-	116211398	5	5	---	---	intergenic	---
1	116395859	116395859	-	116395859	3	3	XLOC_002507	-	predicted	---
1	116993199	116993201	-	116993201	5	13	ENSSSCG00000029836	-	3'UTR	---
1	116993293	116993293	-	116993293	4	4	ENSSSCG00000029836	-	3'UTR	---
1	117094867	117094867	-	117094867	6	6	---	---	intergenic	---
1	117151818	117151818	-	117151818	3	3	XLOC_002511	-	predicted	---
1	117306024	117306024	-	117306024	4	4	XLOC_002513	-	predicted	---
1	117306080	117306080	-	117306080	4	4	XLOC_002513	-	predicted	---
1	117309991	117309992	-	117309991	21	27	ENSSSCG00000004539	-	ORF	---
1	117310495	117310495	-	117310495	6	6	ENSSSCG00000004539	-	ORF	---
1	117466473	117466473	-	117466473	3	3	---	---	intergenic	---

1	117498413	117498413	+	117498413	4	4	ENSSSCG00000004542	+	ORF	*NARS*
1	117510474	117510483	+	117510483	24	38	XLOC_000584	+	predicted	---
1	117587959	117587959	-	117587959	3	3	XLOC_002517	-	predicted	---
1	117590845	117590845	-	117590845	4	4	XLOC_002517	-	predicted	---
1	117627445	117627447	+	117627446	27	35	XLOC_000587	+	predicted	---
1	117627936	117627936	+	117627936	10	10	XLOC_000587	+	predicted	---
1	117822208	117822208	+	117822208	3	3	ENSSSCG00000004545	+	intron	*ANKDD1A*
1	117822917	117822917	+	117822917	12	12	ENSSSCG00000004545	+	intron	*ANKDD1A*
1	117838421	117838421	-	117838421	3	3	ENSSSCG00000004546	-	ORF	*SUMO1*
1	118016231	118016235	-	118016231	25	37	XLOC_002521	-	predicted	---
1	118107271	118107271	-	118107271	3	3	---	---	intergenic	---
1	118169154	118169154	+	118169154	5	5	ENSSSCG00000004550	+	3'UTR	*OAZ2*
1	118169202	118169232	+	118169203	60	170	ENSSSCG00000004550	+	3'UTR	*OAZ2*
1	118169406	118169407	+	118169406	10	17	ENSSSCG00000004550	+	3'UTR	*OAZ2*
1	118170081	118170089	+	118170088	13	31	ENSSSCG00000004550	+	3'UTR	*OAZ2*
1	118171295	118171299	-	118171295	3	6	XLOC_002524	-	predicted	---
1	118172720	118172720	-	118172720	3	3	ENSSSCG00000004551	-	3'UTR	*ZNF609*
1	118175454	118175454	-	118175454	4	4	ENSSSCG00000004551	-	3'UTR	*ZNF609*
1	118353756	118353756	-	118353756	3	3	ENSSSCG00000004551	-	intron	*ZNF609*
1	118414253	118414253	-	118414253	4	4	XLOC_002526	-	predicted	---
1	118495371	118495378	-	118495377	9	18	ENSSSCG00000004554	-	3'UTR	---
							ENSSSCG00000004552	-	intron	*TRIP4*
1	118496477	118496477	-	118496477	3	3	ENSSSCG00000004554	-	3'UTR	---
							ENSSSCG00000004552	-	intron	*TRIP4*
1	118782827	118782827	+	118782827	5	5	ENSSSCG00000004558	+	intron	---
1	118793465	118793465	+	118793465	4	4	XLOC_000600	+	predicted	---

1	118798196	118798196	+	118798196	11	11	---	---	intergenic	---
1	118838151	118838162	+	118838151	30	63	XLOC_000602	+	predicted	---
1	118993427	118993431	-	118993427	4	7	ENSSSCG00000021588	-	3'UTR	*DAPK2*
1	119166673	119166673	-	119166673	10	10	---	---	intergenic	---
1	119560523	119560530	+	119560523	6	15	ENSSSCG00000004561	+	3'UTR	*HERC1*
1	119564762	119564762	-	119564762	4	4	XLOC_002532	-	predicted	---
1	119566876	119566879	+	119566876	5	11	---	---	intergenic	---
1	119570743	119570744	-	119570744	6	9	XLOC_002533	-	predicted	---
1	119573461	119573461	-	119573461	5	5	ENSSSCG00000028737	-	3'UTR	---
1	119574029	119574029	-	119574029	5	5	ENSSSCG00000028737	-	3'UTR	---
1	119582993	119582995	+	119582993	7	11	---	---	intergenic	---
1	119675490	119675497	-	119675497	81	140	ENSSSCG00000029654	-	intron	---
1	119854710	119854711	+	119854711	10	13	---	---	intergenic	---
1	119885832	119885835	+	119885832	3	6	XLOC_000609	+	predicted	---
1	119998141	119998141	-	119998141	40	40	ENSSSCG00000026571	-	3'UTR	*RAB8B*
1	120001670	120001676	-	120001671	15	28	ENSSSCG00000026571	-	3'UTR	*RAB8B*
1	120104367	120104367	-	120104367	3	3	---	---	intergenic	---
1	120109486	120109489	+	120109486	28	35	ENSSSCG00000023158	+	3'UTR	*RPS27L*
1	120109576	120109583	+	120109578	106	289	ENSSSCG00000023158	+	3'UTR	*RPS27L*
1	120133392	120133419	-	120133404	6	31	ENSSSCG00000004569	-	3'UTR	*LACTB*
1	120195269	120195269	+	120195269	3	3	---	---	intergenic	---
1	120211178	120211185	-	120211178	17	26	XLOC_002541	-	predicted	---
1	120211636	120211636	-	120211636	3	3	XLOC_002541	-	predicted	---
1	120214418	120214418	+	120214418	5	5	---	---	intergenic	---
1	120217023	120217025	-	120217025	6	9	---	---	intergenic	---
1	120217106	120217111	-	120217106	51	55	ENSSSCG00000004570	-	3'UTR	*TPM1*

1	120220822	120220862	-	120220823	23	42	ENSSSCG00000004570	-	intron	*TPM1*
1	120221711	120221722	-	120221722	22	60	ENSSSCG00000004570	-	ORF	*TPM1*
1	120225886	120225888	-	120225886	5	13	ENSSSCG00000004570	-	intron	*TPM1*
1	120833196	120833196	+	120833196	4	4	---	---	intergenic	---
1	120991497	120991497	-	120991497	5	5	---	---	intergenic	---
1	121144490	121144492	-	121144491	9	20	XLOC_002548	-	predicted	---
1	121529132	121529132	+	121529132	5	5	ENSSSCG00000025747	+	3'UTR	---
1	121530405	121530405	+	121530405	6	6	ENSSSCG00000025747	+	3'UTR	---
1	121965755	121965755	-	121965755	3	3	ENSSSCG00000004575	-	ORF	---
1	122282485	122282485	+	122282485	5	5	---	---	intergenic	---
1	122291387	122291392	+	122291392	12	19	---	---	intergenic	---
1	122480879	122480879	+	122480879	3	3	---	---	intergenic	---
1	122484855	122484855	+	122484855	3	3	---	---	intergenic	---
1	122672909	122672909	+	122672909	3	3	XLOC_000623	+	predicted	---
1	123056048	123056050	+	123056048	4	7	---	---	intergenic	---
1	123056384	123056393	-	123056384	5	8	---	---	intergenic	---
1	123058730	123058733	+	123058730	4	7	XLOC_000625	+	predicted	---
1	123247970	123247975	+	123247970	5	10	XLOC_000624	+	predicted	---
1	123339352	123339352	+	123339352	3	3	ENSSSCG00000004577	+	3'UTR	*ICE2*
1	123398153	123398153	+	123398153	4	4	---	---	intergenic	---
1	123410211	123410211	+	123410211	3	3	ENSSSCG00000004578	+	intron	*ANXA2*
1	123414395	123414409	+	123414401	169	313	ENSSSCG00000004578	+	3'UTR	*ANXA2*
1	124316514	124316524	+	124316521	273	428	ENSSSCG00000004583	+	3'UTR	*GTF2A2*
1	124320748	124320748	+	124320748	3	3	XLOC_000628	+	predicted	---
1	124392744	124392744	-	124392744	7	7	XLOC_002564	-	predicted	---
1	124435323	124435323	-	124435323	7	7	---	---	intergenic	---

1	124545514	124545514	-	124545514	3	3	---	---	intergenic	---
1	124901513	124901519	+	124901514	5	15	ENSSSCG00000004587	+	3'UTR	*MYO1E*
1	124906567	124906576	-	124906576	36	100	XLOC_002569	-	predicted	---
1	124952376	124952376	+	124952376	5	5	---	---	intergenic	---
1	124962346	124962346	-	124962346	4	4	ENSSSCG00000004589	-	3'UTR	*RNF111*
1	124998279	124998281	-	124998279	8	12	ENSSSCG00000004589	-	intron	*RNF111*
1	125013121	125013121	-	125013121	3	3	ENSSSCG00000004589	-	ORF	*RNF111*
1	125013246	125013246	-	125013246	5	5	ENSSSCG00000004589	-	ORF	*RNF111*
1	125013327	125013327	-	125013327	4	4	ENSSSCG00000004589	-	ORF	*RNF111*
1	125109742	125109742	+	125109742	3	3	---	---	intergenic	---
1	125153131	125153131	+	125153131	7	7	ENSSSCG00000004592	+	intron	---
1	125212244	125212245	-	125212244	6	11	ENSSSCG00000004590	-	3'UTR	---
1	125212609	125212618	-	125212612	6	23	ENSSSCG00000004590	-	3'UTR	---
1	125352645	125352646	+	125352645	6	10	---	---	intergenic	---
1	125424502	125424517	+	125424517	10	19	---	---	intergenic	---
1	125473971	125473972	-	125473972	16	19	ENSSSCG00000004595	-	3'UTR	*ADAM10*
1	125474024	125474024	-	125474024	5	5	ENSSSCG00000004595	-	3'UTR	*ADAM10*
1	125517851	125517851	-	125517851	3	3	ENSSSCG00000004595	-	ORF	*ADAM10*
1	125736588	125736589	+	125736588	4	7	---	---	intergenic	---
1	125876175	125876175	+	125876175	3	3	---	---	intergenic	---
1	125890487	125890487	+	125890487	3	3	---	---	intergenic	---
1	125897932	125897938	-	125897933	15	55	XLOC_002579	-	predicted	---
1	125898159	125898167	-	125898165	33	62	ENSSSCG00000004597	-	3'UTR	*AQP9*
1	125955248	125955253	-	125955249	22	58	XLOC_002579	-	predicted	---
1	126105078	126105089	+	126105079	64	111	ENSSSCG00000025578	+	3'UTR	*ALDH1A2*
1	126217122	126217122	-	126217122	8	8	---	---	intergenic	---

1	126272289	126272289	-	126272289	3	3	---	---	intergenic	---
1	126383491	126383491	-	126383491	3	3	ENSSSCG00000030605	-	3'UTR	*POLR2M*
1	126385137	126385145	-	126385137	16	23	ENSSSCG00000030605	-	3'UTR	*POLR2M*
1	126390964	126390964	-	126390964	4	4	ENSSSCG00000030605	-	intron	*POLR2M*
1	126548277	126548277	-	126548277	5	5	XLOC_002583	-	predicted	---
1	126628903	126628903	-	126628903	6	6	---	---	intergenic	---
1	126719577	126719577	-	126719577	5	5	---	---	intergenic	---
1	126827429	126827429	-	126827429	3	3	---	---	intergenic	---
1	126843709	126843709	-	126843709	3	3	ENSSSCG00000004600	-	ORF	*CH242-58J12.2*
1	126845453	126845453	-	126845453	6	6	ENSSSCG00000004600	-	ORF	*CH242-58J12.2*
1	126847768	126847768	-	126847768	3	3	ENSSSCG00000004600	-	intron	*CH242-58J12.2*
1	127433111	127433116	-	127433116	4	7	ENSSSCG00000004600	-	intron	*CH242-58J12.2*
1	127621691	127621691	+	127621691	3	3	ENSSSCG00000026960	+	intron	*ZNF280D*
1	127733713	127733713	-	127733713	8	8	---	---	intergenic	---
1	127997786	127997786	-	127997786	9	9	ENSSSCG00000004602	-	intron	*TEX9*
1	128030065	128030065	-	128030065	3	3	ENSSSCG00000004602	-	intron	*TEX9*
1	128063648	128063648	+	128063648	4	4	---	---	intergenic	---
1	128288808	128288808	+	128288808	5	5	ENSSSCG00000004603	+	3'UTR	*RFX7*
1	128375834	128375834	+	128375834	3	3	ENSSSCG00000029459	+	intron	---
1	128496141	128496141	+	128496141	8	8	ENSSSCG00000029459	+	intron	---
1	128898965	128898965	-	128898965	3	3	XLOC_002598	-	predicted	---
1	128934352	128934355	+	128934355	8	12	XLOC_000663	+	predicted	---

1	129024899	129024899	+	129024899	3	3	ENSSSCG00000004610	+	ORF	*CCPG1*
1	129024956	129024956	+	129024956	3	3	ENSSSCG00000004610	+	ORF	*CCPG1*
1	129028731	129028731	+	129028731	3	3	ENSSSCG00000004610	+	intron	*CCPG1*
1	129029519	129029524	+	129029523	54	82	XLOC_000664	+	predicted	---
1	129029520	129029523	-	129029520	5	9	ENSSSCG00000024046	-	3'UTR	---
1	129059336	129059355	+	129059336	15	21	XLOC_000666	+	predicted	---
1	129165586	129165586	+	129165586	4	4	ENSSSCG00000024983	+	3'UTR	*RSL24D1*
1	129165909	129165912	+	129165909	6	9	ENSSSCG00000024983	+	3'UTR	*RSL24D1*
1	129166826	129166837	+	129166837	12	21	ENSSSCG00000024983	+	3'UTR	*RSL24D1*
1	129214593	129214593	-	129214593	4	4	---	---	intergenic	---
1	129511861	129511861	+	129511861	6	6	---	---	intergenic	---
1	129662639	129662639	+	129662639	3	3	---	---	intergenic	---
1	130177635	130177635	-	130177635	3	3	ENSSSCG00000004614	-	intron	*UNC13C*
1	130797159	130797159	+	130797159	4	4	ENSSSCG00000004615	+	intron	---
1	131668032	131668032	-	131668032	3	3	---	---	intergenic	---
1	132025585	132025585	+	132025585	5	5	XLOC_000674	+	predicted	---
1	132192312	132192312	+	132192312	3	3	XLOC_000676	+	predicted	---
1	132221111	132221128	+	132221117	85	154	ENSSSCG00000004618	+	3'UTR	*ARPP19*
1	132221840	132221850	+	132221848	49	85	ENSSSCG00000004618	+	3'UTR	*ARPP19*
1	132222358	132222359	+	132222359	5	8	XLOC_000678	+	predicted	---
1	132223814	132223814	+	132223814	4	4	XLOC_000678	+	predicted	---
1	132286908	132286908	+	132286908	4	4	---	---	intergenic	---
1	132479998	132479998	+	132479998	12	12	ENSSSCG00000004620	+	3'UTR	*MYO5A*
1	132704132	132704132	+	132704132	6	6	---	---	intergenic	---
1	132764118	132764118	-	132764118	3	3	---	---	intergenic	---
1	132815469	132815469	-	132815469	3	3	ENSSSCG00000004624	-	3'UTR	*MAPK6*

1	132815584	132815584	-	132815584	5	5	ENSSSCG00000004624	-	3'UTR	*MAPK6*
1	132816690	132816711	-	132816711	5	8	ENSSSCG00000004624	-	3'UTR	*MAPK6*
1	132932776	132932776	+	132932776	6	6	ENSSSCG00000004625	+	3'UTR	*LEO1*
1	132950845	132950845	-	132950845	3	3	XLOC_002610	-	predicted	---
1	132952682	132952690	-	132952690	10	13	XLOC_002610	-	predicted	---
1	133003746	133003746	+	133003746	3	3	---	---	intergenic	---
1	133179404	133179404	+	133179404	3	3	ENSSSCG00000004630	+	3'UTR	*SCG3*
1	133450248	133450263	+	133450248	5	9	ENSSSCG00000004631	+	3'UTR	*DMXL2*
1	133583616	133583617	-	133583616	11	15	XLOC_002614	-	predicted	---
1	133591087	133591088	-	133591087	3	6	---	---	intergenic	---
1	134212795	134212796	-	134212795	34	38	---	---	intergenic	---
1	134278020	134278022	-	134278022	4	7	XLOC_002618	-	predicted	---
1	134494981	134494981	+	134494981	7	7	XLOC_000691	+	predicted	---
1	134560681	134560681	+	134560681	3	3	ENSSSCG00000004635	+	ORF	*SPPL2A*
1	134569321	134569323	+	134569322	17	31	ENSSSCG00000004635	+	3'UTR	*SPPL2A*
1	134571043	134571056	+	134571043	3	6	ENSSSCG00000004635	+	3'UTR	*SPPL2A*
1	134571570	134571570	+	134571570	3	3	ENSSSCG00000004635	+	3'UTR	*SPPL2A*
1	134573602	134573602	+	134573602	3	3	XLOC_000692	+	predicted	---
1	134598380	134598380	-	134598380	3	3	---	---	intergenic	---
1	134645306	134645307	+	134645306	4	7	ENSSSCG00000004636	+	intron	*TRPM7*
1	134657026	134657026	+	134657026	6	6	ENSSSCG00000004636	+	ORF	*TRPM7*
1	134682849	134682849	+	134682849	3	3	ENSSSCG00000004636	+	ORF	*TRPM7*
1	134689350	134689350	+	134689350	4	4	ENSSSCG00000004636	+	3'UTR	*TRPM7*
1	134689663	134689665	+	134689663	3	6	ENSSSCG00000004636	+	3'UTR	*TRPM7*
1	134695291	134695291	+	134695291	6	6	---	---	intergenic	---
1	134719417	134719417	+	134719417	4	4	ENSSSCG00000004638	+	ORF	*USP50*

1	134746562	134746562	+	134746562	3	3	ENSSSCG00000004638	+	ORF	*USP50*
1	134746790	134746807	+	134746795	208	395	XLOC_000697	+	predicted	---
1	134748021	134748021	-	134748021	8	8	XLOC_002622	-	predicted	---
1	134787800	134787812	-	134787811	12	20	ENSSSCG00000004639	-	ORF	*USP8*
1	134787856	134787856	-	134787856	3	3	ENSSSCG00000004639	-	ORF	*USP8*
1	135129032	135129032	+	135129032	3	3	ENSSSCG00000004645	+	3'UTR	---
1	135180930	135180930	-	135180930	3	3	ENSSSCG00000004643	-	intron	*SLC27A2*
1	135181068	135181071	-	135181068	3	6	ENSSSCG00000004643	-	ORF	*SLC27A2*
1	135186321	135186321	-	135186321	6	6	ENSSSCG00000004643	-	ORF	*SLC27A2*
1	135792121	135792132	-	135792130	18	71	ENSSSCG00000004647	-	3'UTR	*DTWD1*
1	135824276	135824278	+	135824278	7	13	ENSSSCG00000004648	+	intron	---
1	136150449	136150449	-	136150449	5	5	ENSSSCG00000004651	-	3'UTR	*GALK2*
1	136420265	136420270	-	136420267	7	21	---	---	intergenic	---
1	136469083	136469083	+	136469083	5	5	ENSSSCG00000004653	+	ORF	*SECISBP2L*
1	136513288	136513288	+	136513288	8	8	ENSSSCG00000004653	+	3'UTR	*SECISBP2L*
1	136635409	136635410	-	136635409	7	12	XLOC_002631	-	predicted	---
1	136730510	136730510	+	136730510	3	3	ENSSSCG00000004657	+	intron	*CEP152*
1	136737354	136737354	+	136737354	6	6	---	---	intergenic	---
1	136802468	136802471	+	136802471	13	22	XLOC_000714	+	predicted	---
1	137056405	137056405	+	137056405	7	7	---	---	intergenic	---
1	137389625	137389625	+	137389625	3	3	XLOC_000715	+	predicted	---
1	137390173	137390175	+	137390174	16	26	XLOC_000715	+	predicted	---
1	137480903	137480910	-	137480909	47	97	ENSSSCG00000026096	-	3'UTR	*DUT*
1	137513556	137513556	-	137513556	3	3	ENSSSCG00000004659	-	intron	*SLC12A1*
1	137629918	137629918	-	137629918	5	5	---	---	intergenic	---
1	137708003	137708003	-	137708003	5	5	---	---	intergenic	---

1	137711993	137711995	-	137711995	6	12	XLOC_002640	-	predicted	---
1	138154813	138154813	-	138154813	6	6	---	---	intergenic	---
1	138577922	138577922	+	138577922	32	32	---	---	intergenic	---
1	138597456	138597456	-	138597456	3	3	---	---	intergenic	---
1	138677300	138677300	-	138677300	3	3	---	---	intergenic	---
1	138758448	138758448	+	138758448	5	5	---	---	intergenic	---
1	139654779	139654779	-	139654779	3	3	---	---	intergenic	---
1	139655062	139655062	+	139655062	4	4	---	---	intergenic	---
1	140193811	140193811	-	140193811	3	3	---	---	intergenic	---
1	140435909	140435916	+	140435913	38	89	ENSSSCG00000004665	+	3'UTR	---
1	140706368	140706370	+	140706370	13	21	ENSSSCG00000004666	+	3'UTR	*SQRDL*
1	140844956	140844971	-	140844960	64	305	ENSSSCG00000004672	-	3'UTR	*GATM*
1	140845215	140845225	-	140845225	20	23	ENSSSCG00000004672	-	3'UTR	*GATM*
1	140845436	140845436	-	140845436	4	4	ENSSSCG00000004672	-	3'UTR	*GATM*
1	140845562	140845562	-	140845562	5	5	ENSSSCG00000004672	-	3'UTR	*GATM*
1	140845664	140845667	-	140845664	8	14	ENSSSCG00000004672	-	3'UTR	*GATM*
1	140845844	140845844	-	140845844	3	3	ENSSSCG00000004672	-	3'UTR	*GATM*
1	140848806	140848806	-	140848806	5	5	ENSSSCG00000004672	-	ORF	*GATM*
1	140851106	140851106	-	140851106	3	3	ENSSSCG00000004672	-	ORF	*GATM*
1	140853771	140853783	-	140853783	7	18	ENSSSCG00000004672	-	ORF	*GATM*
1	140916438	140916438	+	140916438	6	6	ENSSSCG00000004670	+	intron	*C15orf48*
1	140919139	140919139	+	140919139	7	7	ENSSSCG00000004670	+	3'UTR	*C15orf48*
1	140919279	140919291	+	140919284	142	351	ENSSSCG00000004670	+	3'UTR	*C15orf48*
1	141072728	141072736	-	141072728	8	22	ENSSSCG00000004673	-	3'UTR	*SLC28A2*
1	141141091	141141093	-	141141091	7	14	---	---	intergenic	---
1	141141280	141141282	-	141141280	5	13	---	---	intergenic	---

1	141142956	141142956	+	141142956	3	3	---	---	intergenic	---
1	141257783	141257783	-	141257783	4	4	ENSSSCG00000004679	-	3'UTR	*SORD*
1	141257873	141257876	-	141257874	4	10	ENSSSCG00000004679	-	3'UTR	*SORD*
1	141257926	141257928	-	141257926	11	14	ENSSSCG00000004679	-	3'UTR	*SORD*
1	141258008	141258009	-	141258009	16	19	ENSSSCG00000004679	-	3'UTR	*SORD*
1	141259119	141259162	-	141259136	206	918	ENSSSCG00000004679	-	3'UTR	*SORD*
1	141259255	141259270	-	141259255	9	23	ENSSSCG00000004679	-	3'UTR	*SORD*
1	141259319	141259348	-	141259319	11	15	ENSSSCG00000004679	-	ORF	*SORD*
1	141274440	141274440	-	141274440	3	3	ENSSSCG00000004679	-	ORF	*SORD*
1	141282424	141282424	-	141282424	3	3	ENSSSCG00000004679	-	intron	*SORD*
1	141284119	141284138	-	141284119	6	18	ENSSSCG00000004679	-	ORF	*SORD*
1	141338395	141338395	-	141338395	3	3	XLOC_002652	-	predicted	---
1	141375184	141375197	-	141375193	33	62	XLOC_002653	-	predicted	---
1	141375244	141375254	-	141375252	23	48	ENSSSCG00000004681	-	ORF	*TRIM69*
1	141390734	141390734	-	141390734	12	12	ENSSSCG00000004681	-	ORF	*TRIM69*
1	141391282	141391282	-	141391282	12	12	ENSSSCG00000004681	-	intron	*TRIM69*
1	141418157	141418157	-	141418157	3	3	XLOC_002655	-	predicted	---
1	141434380	141434380	-	141434380	3	3	---	---	intergenic	---
1	141436073	141436081	-	141436074	105	137	XLOC_002654	-	predicted	---
1	141436261	141436273	-	141436262	17	52	XLOC_002654	-	predicted	---
1	141436329	141436392	-	141436333	105	458	ENSSSCG00000004687	-	3'UTR	*B2M*
1	141436629	141436642	-	141436642	5	9	ENSSSCG00000004687	-	3'UTR	*B2M*
1	141436695	141436700	-	141436698	18	44	ENSSSCG00000004687	-	3'UTR	*B2M*
1	141436833	141436850	-	141436839	302	531	ENSSSCG00000004687	-	3'UTR	*B2M*
1	141436937	141436937	-	141436937	4	4	ENSSSCG00000004687	-	3'UTR	*B2M*
1	141500816	141500816	+	141500816	3	3	ENSSSCG00000004684	+	intron	---

1	141721357	141721358	-	141721357	4	8	XLOC_002660	-	predicted	---
1	141721459	141721460	+	141721460	4	7	ENSSSCG00000028089	+	3'UTR	---
							ENSSSCG00000023996	+	intron	---
1	141722062	141722070	-	141722066	11	27	ENSSSCG00000004691	-	3'UTR	*EIF3J*
1	141722509	141722509	-	141722509	3	3	ENSSSCG00000004691	-	3'UTR	*EIF3J*
1	141722799	141722800	-	141722800	4	7	ENSSSCG00000004691	-	3'UTR	*EIF3J*
1	141722908	141722911	-	141722908	4	12	ENSSSCG00000004691	-	3'UTR	*EIF3J*
1	141727598	141727598	-	141727598	3	3	ENSSSCG00000004691	-	ORF	*EIF3J*
1	141745088	141745098	+	141745096	19	47	ENSSSCG00000023996	+	intron	---
1	141828217	141828217	+	141828217	4	4	ENSSSCG00000023996	+	intron	---
1	141893318	141893318	+	141893318	13	13	ENSSSCG00000023996	+	intron	---
1	141898733	141898733	+	141898733	4	4	ENSSSCG00000023996	+	intron	---
1	141907043	141907043	+	141907043	3	3	ENSSSCG00000023996	+	intron	---
1	142037819	142037819	+	142037819	3	3	---	---	intergenic	---
1	142229215	142229216	+	142229215	12	15	---	---	intergenic	---
1	142474930	142474930	+	142474930	4	4	ENSSSCG00000004694	+	intron	*FRMD5*
1	142482386	142482386	-	142482386	3	3	XLOC_002665	-	predicted	---
1	142483348	142483370	-	142483370	4	7	XLOC_002665	-	predicted	---
1	142576251	142576251	+	142576251	3	3	XLOC_000749	+	predicted	---
1	142577466	142577470	+	142577466	9	12	XLOC_000749	+	predicted	---
1	142577514	142577514	+	142577514	5	5	---	---	intergenic	---
1	142579448	142579468	+	142579448	4	7	XLOC_000749	+	predicted	---
1	142585500	142585512	+	142585500	6	18	XLOC_000749	+	predicted	---
1	142585553	142585561	+	142585554	30	54	XLOC_000749	+	predicted	---
1	142585608	142585611	+	142585608	15	20	XLOC_000749	+	predicted	---
1	142585688	142585688	+	142585688	3	3	XLOC_000749	+	predicted	---

1	142588041	142588072	-	142588066	77	231	ENSSSCG00000004697	-	3'UTR	*HYPK*
1	142595269	142595284	-	142595280	985	4016	ENSSSCG00000024263	-	3'UTR	*SERF2*
1	142597001	142597001	+	142597001	3	3	XLOC_000750	+	predicted	---
1	142614459	142614466	-	142614460	13	19	XLOC_002668	-	predicted	---
1	142615353	142615366	-	142615363	110	271	XLOC_002668	-	predicted	---
1	142615434	142615434	-	142615434	3	3	XLOC_002668	-	predicted	---
1	142615492	142615532	-	142615492	12	23	XLOC_002668	-	predicted	---
1	142620225	142620233	-	142620225	12	15	ENSSSCG00000004700	-	ORF	*GRP-58*
1	142621474	142621511	-	142621511	15	19	ENSSSCG00000004700	-	ORF	*GRP-58*
1	142656671	142656671	+	142656671	7	7	ENSSSCG00000004701	+	ORF	*CATSPER2*
1	142658309	142658310	+	142658310	19	29	XLOC_000752	+	predicted	---
1	142763149	142763149	-	142763149	4	4	XLOC_002672	-	predicted	---
1	142836997	142836997	+	142836997	3	3	XLOC_000759	+	predicted	---
1	142837685	142837697	-	142837688	6	15	---	---	intergenic	---
1	142951533	142951535	+	142951535	6	11	XLOC_000760	+	predicted	---
1	142984540	142984546	+	142984540	11	14	XLOC_000762	+	predicted	---
1	143106962	143106963	+	143106962	3	6	---	---	intergenic	---
1	143129858	143129864	+	143129864	4	7	XLOC_000766	+	predicted	---
1	143133256	143133277	-	143133259	29	48	XLOC_002676	-	predicted	---
1	143133316	143133316	-	143133316	3	3	XLOC_002676	-	predicted	---
1	143133630	143133631	-	143133630	3	6	XLOC_002676	-	predicted	---
1	143203325	143203325	-	143203325	3	3	---	---	intergenic	---
1	143203358	143203368	+	143203358	48	96	---	---	intergenic	---
1	143313375	143313375	+	143313375	3	3	ENSSSCG00000004717	+	intron	*UBR1*
1	143317232	143317232	+	143317232	6	6	ENSSSCG00000004717	+	3'UTR	*UBR1*
1	143462918	143462918	+	143462918	3	3	ENSSSCG00000004718	+	ORF	*TTBK2*

1	143478044	143478044	+	143478044	3	3	ENSSSCG00000004718	+	3'UTR	*TTBK2*
1	143534204	143534211	+	143534204	7	11	XLOC_000770	+	predicted	---
1	143549674	143549674	+	143549674	5	5	ENSSSCG00000004719	+	3'UTR	*CDAN1*
1	143549993	143549993	+	143549993	3	3	ENSSSCG00000004719	+	3'UTR	*CDAN1*
1	143713429	143713429	-	143713429	4	4	ENSSSCG00000004724	-	ORF	*HAUS2*
1	143738145	143738148	+	143738146	38	58	ENSSSCG00000004726	+	3'UTR	*LRRC57*
1	143738568	143738568	-	143738568	3	3	XLOC_002680	-	predicted	---
1	143742364	143742392	-	143742365	8	37	ENSSSCG00000004727	-	3'UTR	*SNAP23*
1	143742928	143742929	-	143742928	4	7	ENSSSCG00000004727	-	3'UTR	*SNAP23*
1	143743190	143743200	-	143743190	3	9	ENSSSCG00000004727	-	3'UTR	*SNAP23*
1	143743641	143743641	-	143743641	4	4	ENSSSCG00000004727	-	ORF	*SNAP23*
1	143823727	143823727	+	143823727	3	3	ENSSSCG00000004725	+	ORF	*ZNF106*
1	143831970	143831970	+	143831970	6	6	ENSSSCG00000004725	+	intron	*ZNF106*
1	143848494	143848494	+	143848494	5	5	XLOC_000773	+	predicted	---
1	143852329	143852351	-	143852337	204	916	ENSSSCG00000004728	-	3'UTR	*SKM*
1	143860563	143860563	-	143860563	26	26	---	---	intergenic	---
1	143900241	143900241	-	143900241	3	3	ENSSSCG00000004728	-	intron	*SKM*
1	143913342	143913342	-	143913342	7	7	ENSSSCG00000004729	-	3'UTR	*GANC*
1	143982203	143982203	-	143982203	3	3	ENSSSCG00000004729	-	intron	*GANC*
1	144030576	144030576	+	144030576	3	3	ENSSSCG00000004730	+	ORF	*TMEM87A*
1	144138200	144138200	+	144138200	3	3	ENSSSCG00000004731	+	3'UTR	*VPS39*
1	144138658	144138658	+	144138658	4	4	ENSSSCG00000004731	+	3'UTR	*VPS39*
1	144140108	144140109	+	144140109	6	10	ENSSSCG00000004731	+	3'UTR	*VPS39*
1	144271743	144271743	-	144271743	4	4	---	---	intergenic	---
1	144349237	144349237	+	144349237	3	3	XLOC_000779	+	predicted	---
1	144393694	144393703	+	144393694	5	12	---	---	intergenic	---

1	144546218	144546219	+	144546218	6	10	ENSSSCG00000004738	+	3'UTR	---
1	144548231	144548233	+	144548232	15	23	ENSSSCG00000004738	+	3'UTR	---
1	144867408	144867408	-	144867408	3	3	---	---	intergenic	---
1	144883365	144883373	-	144883365	5	10	ENSSSCG00000004744	-	3'UTR	*TYRO3*
1	144939084	144939084	-	144939084	9	9	ENSSSCG00000004746	-	3'UTR	*ITPKA*
1	144952497	144952497	-	144952497	6	6	---	---	intergenic	---
1	144952523	144952523	+	144952523	3	3	---	---	intergenic	---
1	144958716	144958717	-	144958716	4	7	ENSSSCG00000004747	-	3'UTR	*RTF1*
1	144959995	144959995	-	144959995	9	9	ENSSSCG00000004747	-	3'UTR	*RTF1*
1	144960215	144960215	-	144960215	3	3	ENSSSCG00000004747	-	3'UTR	*RTF1*
1	144993517	144993517	+	144993517	3	3	---	---	intergenic	---
1	144993762	144993773	-	144993762	4	8	ENSSSCG00000004747	-	intron	*RTF1*
1	145141595	145141595	+	145141595	12	12	XLOC_000794	+	predicted	---
1	145142147	145142148	-	145142147	5	8	XLOC_002699	-	predicted	---
1	145143048	145143048	-	145143048	3	3	XLOC_002699	-	predicted	---
1	145349207	145349207	+	145349207	3	3	---	---	intergenic	---
1	145401448	145401448	+	145401448	10	10	ENSSSCG00000004753	+	intron	*INO80*
1	145539051	145539051	-	145539051	3	3	ENSSSCG00000004755	-	3'UTR	*DLL4*
1	145570608	145570609	-	145570608	13	18	ENSSSCG00000004757	-	3'UTR	*VPS18*
1	145603634	145603635	+	145603635	4	7	ENSSSCG00000004758	+	3'UTR	*RHOV*
1	145654309	145654310	+	145654310	10	13	ENSSSCG00000004760	+	intron	*PPP1R14D*
1	145668921	145668921	-	145668921	8	8	XLOC_002706	-	predicted	---
1	145717600	145717607	+	145717601	16	45	ENSSSCG00000004760	+	intron	*PPP1R14D*
1	145717791	145717808	-	145717803	18	71	ENSSSCG00000004764	-	3'UTR	*GCHFR*
1	145749954	145749960	+	145749954	3	6	ENSSSCG00000004765	+	3'UTR	*RMDN3*
							ENSSSCG00000004760	+	intron	*PPP1R14D*

1	145844748	145844748	-	145844748	3	3	ENSSSCG00000004769	-	ORF	*CASC5*
1	145862891	145862891	-	145862891	5	5	XLOC_002711	-	predicted	---
1	145921546	145921553	+	145921546	5	9	XLOC_000814	+	predicted	---
1	145935050	145935061	-	145935058	5	11	ENSSSCG00000004772	-	3'UTR	*BAHD1*
1	145979036	145979038	-	145979037	8	21	ENSSSCG00000004774	-	3'UTR	*IVD*
1	145979213	145979217	-	145979214	10	18	ENSSSCG00000004774	-	3'UTR	*IVD*
1	145980598	145980608	-	145980608	24	44	ENSSSCG00000004774	-	3'UTR	*IVD*
1	145992385	145992385	-	145992385	3	3	ENSSSCG00000004774	-	intron	*IVD*
1	146002012	146002014	-	146002012	6	14	XLOC_002714	-	predicted	---
1	146002473	146002473	-	146002473	9	9	XLOC_002714	-	predicted	---
1	146002513	146002528	-	146002523	44	87	XLOC_002714	-	predicted	---
1	146003065	146003069	-	146003065	5	14	ENSSSCG00000004775	-	ORF	*KNSTRN*
1	146038779	146038779	-	146038779	21	21	XLOC_002715	-	predicted	---
1	146069182	146069182	+	146069182	5	5	XLOC_000817	+	predicted	---
1	146124951	146124956	-	146124951	3	6	XLOC_002717	-	predicted	---
1	146126678	146126678	-	146126678	3	3	---	---	intergenic	---
1	146156002	146156003	-	146156003	4	7	---	---	intergenic	---
1	146211282	146211282	-	146211282	6	6	XLOC_002721	-	predicted	---
1	146252439	146252439	-	146252439	11	11	XLOC_002724	-	predicted	---
1	146253384	146253384	-	146253384	7	7	XLOC_002724	-	predicted	---
1	146303909	146303909	+	146303909	3	3	---	---	intergenic	---
1	146312686	146312687	+	146312687	6	9	XLOC_000821	+	predicted	---
1	146436268	146436271	-	146436268	17	39	---	---	intergenic	---
1	146440298	146440298	-	146440298	3	3	---	---	intergenic	---
1	146797824	146797825	-	146797824	6	11	---	---	intergenic	---
1	146894391	146894391	+	146894391	4	4	XLOC_000827	+	predicted	---

1	146905999	146906007	-	146906007	16	27	ENSSSCG00000004789	-	3'UTR	*THBS1*
1	147024390	147024396	-	147024393	8	24	---	---	intergenic	---
1	147161060	147161060	+	147161060	4	4	---	---	intergenic	---
1	147457825	147457837	-	147457834	18	37	XLOC_002739	-	predicted	---
1	147654267	147654267	-	147654267	3	3	---	---	intergenic	---
1	147843035	147843035	-	147843035	7	7	---	---	intergenic	---
1	148274626	148274626	-	148274626	3	3	XLOC_002741	-	predicted	---
1	148293710	148293710	-	148293710	3	3	ENSSSCG00000004792	-	intron	*FAM98B*
1	148440341	148440341	-	148440341	4	4	XLOC_002742	-	predicted	---
1	148517631	148517631	+	148517631	3	3	---	---	intergenic	---
1	148558414	148558418	+	148558417	7	14	---	---	intergenic	---
1	148666591	148666591	-	148666591	3	3	---	---	intergenic	---
1	148817054	148817093	-	148817056	378	691	XLOC_002743	-	predicted	---
1	148817221	148817238	-	148817238	5	9	XLOC_002743	-	predicted	---
1	148830680	148830687	-	148830680	16	33	---	---	intergenic	---
1	148831239	148831239	-	148831239	3	3	---	---	intergenic	---
1	148833279	148833279	+	148833279	3	3	---	---	intergenic	---
1	148947094	148947095	+	148947094	5	8	---	---	intergenic	---
1	149062075	149062075	+	149062075	3	3	---	---	intergenic	---
1	150095577	150095577	+	150095577	23	23	---	---	intergenic	---
1	150270953	150270953	-	150270953	7	7	---	---	intergenic	---
1	150577013	150577013	-	150577013	4	4	---	---	intergenic	---
1	150662092	150662092	+	150662092	6	6	---	---	intergenic	---
1	150877238	150877238	+	150877238	5	5	XLOC_000847	+	predicted	---
1	151885721	151885721	+	151885721	10	10	---	---	intergenic	---
1	152088969	152088972	-	152088970	16	23	XLOC_002755	-	predicted	---

1	152189151	152189151	-	152189151	4	4	ENSSSCG00000004799	-	ORF	*ZNF770*
1	152206661	152206665	-	152206665	4	7	---	---	intergenic	---
1	152208671	152208671	+	152208671	3	3	XLOC_000853	+	predicted	---
1	152219734	152219734	+	152219734	43	43	---	---	intergenic	---
1	152358477	152358477	+	152358477	3	3	XLOC_000855	+	predicted	---
1	152417606	152417608	+	152417606	7	12	ENSSSCG00000004803	+	3'UTR	*ACTC1*
1	152711807	152711807	+	152711807	5	5	ENSSSCG00000004807	+	3'UTR	*SCG5*
1	152712167	152712171	+	152712171	11	19	ENSSSCG00000004807	+	3'UTR	*SCG5*
1	152893816	152893816	+	152893816	4	4	---	---	intergenic	---
1	152989415	152989428	+	152989425	22	45	XLOC_000861	+	predicted	---
1	152989747	152989747	+	152989747	4	4	XLOC_000861	+	predicted	---
1	153487040	153487047	+	153487046	15	49	ENSSSCG00000004811	+	intron	*PGPEP1L*
1	153511352	153511353	+	153511352	15	20	XLOC_000862	+	predicted	---
1	153773696	153773696	-	153773696	4	4	ENSSSCG00000004812	-	intron	*IGF1R*
1	153854667	153854667	-	153854667	5	5	XLOC_002769	-	predicted	---
1	154050038	154050046	-	154050046	13	37	XLOC_002771	-	predicted	---
1	154089170	154089170	-	154089170	3	3	ENSSSCG00000004813	-	intron	*TTC23*
1	154179378	154179378	-	154179378	6	6	---	---	intergenic	---
1	154362469	154362477	+	154362470	64	84	XLOC_000866	+	predicted	---
1	154364406	154364406	+	154364406	6	6	XLOC_000866	+	predicted	---
1	154420266	154420268	-	154420268	5	8	---	---	intergenic	---
1	154689911	154689911	+	154689911	4	4	ENSSSCG00000028771	+	3'UTR	*MEF2A*
1	154690108	154690108	+	154690108	4	4	ENSSSCG00000028771	+	3'UTR	*MEF2A*
1	154692174	154692179	+	154692178	9	26	ENSSSCG00000028771	+	3'UTR	*MEF2A*
1	154713646	154713646	-	154713646	6	6	---	---	intergenic	---
1	154812672	154812674	-	154812672	3	6	---	---	intergenic	---

1	155112831	155112831	+	155112831	3	3	---	---	intergenic	---
1	155115514	155115514	+	155115514	5	5	---	---	intergenic	---
1	155166105	155166106	+	155166106	7	10	---	---	intergenic	---
1	155340280	155340284	+	155340280	4	8	---	---	intergenic	---
1	155373224	155373225	-	155373225	15	18	ENSSSCG00000004820	-	ORF	*LINS*
1	155373990	155373990	-	155373990	4	4	ENSSSCG00000004820	-	ORF	*LINS*
1	155442456	155442456	+	155442456	4	4	ENSSSCG00000004821	+	3'UTR	*ASB7*
1	155444631	155444631	+	155444631	3	3	XLOC_000873	+	predicted	---
1	155549013	155549013	+	155549013	3	3	---	---	intergenic	---
1	155732335	155732342	-	155732335	4	10	ENSSSCG00000004822	-	3'UTR	---
1	155853745	155853745	+	155853745	4	4	ENSSSCG00000004823	+	3'UTR	*LRRK1*
1	155960055	155960073	-	155960068	9	15	ENSSSCG00000004825	-	3'UTR	*CHSY1*
1	156057734	156057778	-	156057777	68	103	ENSSSCG00000004826	-	3'UTR	*VIMP*
1	156059652	156059652	-	156059652	3	3	ENSSSCG00000004826	-	intron	*VIMP*
1	156062388	156062392	-	156062392	19	22	ENSSSCG00000004826	-	intron	*VIMP*
1	156106945	156106945	-	156106945	3	3	---	---	intergenic	---
1	156574235	156574235	+	156574235	3	3	---	---	intergenic	---
1	156834864	156834866	-	156834865	18	26	ENSSSCG00000004827	-	intron	*GABRA5*
1	157180251	157180251	+	157180251	4	4	---	---	intergenic	---
1	157269222	157269222	+	157269222	3	3	---	---	intergenic	---
1	157521704	157521704	+	157521704	3	3	ENSSSCG00000004830	+	3'UTR	*ATP10A*
1	157522094	157522094	+	157522094	11	11	ENSSSCG00000004830	+	3'UTR	*ATP10A*
1	157765550	157765559	-	157765558	6	18	XLOC_002797	-	predicted	---
1	157770997	157770998	-	157770997	14	17	XLOC_002797	-	predicted	---
1	157771297	157771298	-	157771298	15	18	XLOC_002797	-	predicted	---
1	157856090	157856090	+	157856090	3	3	ENSSSCG00000004832	+	ORF	*UBE3A*

1	157881626	157881633	+	157881626	4	7	XLOC_000882	+	predicted	---
1	157882827	157882828	+	157882828	4	7	XLOC_000882	+	predicted	---
1	157890997	157891003	-	157891003	4	7	XLOC_002797	-	predicted	---
1	157891507	157891507	-	157891507	4	4	XLOC_002797	-	predicted	---
1	158313894	158313895	-	158313895	10	17	---	---	intergenic	---
1	158396397	158396397	-	158396397	3	3	---	---	intergenic	---
1	158749579	158749579	+	158749579	6	6	---	---	intergenic	---
1	159065855	159065855	+	159065855	7	7	---	---	intergenic	---
1	159382865	159382865	+	159382865	3	3	---	---	intergenic	---
1	159416002	159416002	-	159416002	3	3	ENSSSCG00000026006	-	intron	*KLF13*
1	159497540	159497540	-	159497540	4	4	---	---	intergenic	---
1	159791924	159791924	+	159791924	3	3	ENSSSCG00000004843	+	intron	---
1	159808432	159808432	+	159808432	4	4	---	---	intergenic	---
1	159863316	159863319	+	159863316	10	27	ENSSSCG00000004844	+	3'UTR	*MTMR10*
1	159865134	159865136	-	159865135	8	19	ENSSSCG00000004845	-	3'UTR	*FAN1*
1	159865180	159865180	-	159865180	3	3	ENSSSCG00000004845	-	3'UTR	*FAN1*
1	159882098	159882098	-	159882098	11	11	ENSSSCG00000004845	-	intron	*FAN1*
1	159910467	159910467	+	159910467	12	12	---	---	intergenic	---
1	159918105	159918106	-	159918105	3	6	XLOC_002811	-	predicted	---
1	159925902	159925902	-	159925902	3	3	ENSSSCG00000004847	-	intron	*MPHOSPH10*
							ENSSSCG00000004847	-	ORF	*MPHOSPH10*
1	159968080	159968088	+	159968081	25	39	ENSSSCG00000004846	+	3'UTR	*MCEE*
1	160084686	160084686	-	160084686	5	5	---	---	intergenic	---
1	160894752	160894752	-	160894752	3	3	ENSSSCG00000022328	-	intron	---
1	160992326	160992326	-	160992326	10	10	---	---	intergenic	---
1	161061521	161061522	+	161061522	5	8	ENSSSCG00000004854	+	3'UTR	---

1	161062869	161062891	+	161062869	8	12	ENSSSCG00000004854	+	3'UTR	---
1	161210689	161210689	-	161210689	5	5	ENSSSCG00000030470	-	intron	---
1	161232136	161232136	-	161232136	3	3	ENSSSCG00000030470	-	intron	---
1	161290745	161290745	-	161290745	4	4	---	---	intergenic	---
1	161319840	161319840	+	161319840	7	7	---	---	intergenic	---
1	161520005	161520005	+	161520005	8	8	---	---	intergenic	---
1	161556820	161556825	-	161556823	8	14	ENSSSCG00000004856	-	intron	*NFATC1*
1	161699785	161699785	-	161699785	4	4	---	---	intergenic	---
1	161918940	161918958	-	161918953	91	251	XLOC_002821	-	predicted	---
1	162058048	162058048	+	162058048	4	4	XLOC_000906	+	predicted	---
1	162064396	162064396	-	162064396	7	7	---	---	intergenic	---
1	163179516	163179516	+	163179516	4	4	---	---	intergenic	---
1	163200173	163200173	-	163200173	25	25	XLOC_002826	-	predicted	---
1	163201191	163201192	-	163201191	5	8	XLOC_002826	-	predicted	---
1	163229970	163229979	+	163229978	9	29	XLOC_000910	+	predicted	---
1	164053355	164053355	+	164053355	4	4	---	---	intergenic	---
1	164703715	164703723	-	164703715	9	16	XLOC_002841	-	predicted	---
1	164704144	164704149	-	164704149	20	26	XLOC_002841	-	predicted	---
1	164784028	164784051	+	164784047	7	20	XLOC_000914	+	predicted	---
1	164837212	164837212	+	164837212	3	3	XLOC_000915	+	predicted	---
1	165027292	165027305	+	165027302	75	157	---	---	intergenic	---
1	165027400	165027400	+	165027400	3	3	---	---	intergenic	---
1	165027645	165027645	+	165027645	11	11	---	---	intergenic	---
1	165113357	165113357	-	165113357	3	3	XLOC_002847	-	predicted	---
1	165124946	165124946	-	165124946	48	48	---	---	intergenic	---
1	165285672	165285672	-	165285672	4	4	---	---	intergenic	---

1	165447845	165447845	-	165447845	3	3	---	---	intergenic	---
1	165447862	165447865	+	165447863	7	21	---	---	intergenic	---
1	165548502	165548510	+	165548508	177	275	ENSSSCG00000004870	+	3'UTR	---
1	165803888	165803900	-	165803892	14	44	---	---	intergenic	---
1	165803933	165803933	+	165803933	10	10	---	---	intergenic	---
1	165805909	165805909	-	165805909	3	3	---	---	intergenic	---
1	165805967	165805967	+	165805967	5	5	---	---	intergenic	---
1	165899356	165899356	-	165899356	3	3	XLOC_002851	-	predicted	---
1	165902260	165902260	+	165902260	4	4	ENSSSCG00000004875	+	ORF	*CYB5A*
1	165936346	165936385	+	165936373	13	26	ENSSSCG00000004875	+	3'UTR	*CYB5A*
1	165936458	165936495	+	165936458	6	9	ENSSSCG00000004875	+	3'UTR	*CYB5A*
1	165936546	165936547	+	165936546	6	9	ENSSSCG00000004875	+	3'UTR	*CYB5A*
1	165936654	165936654	+	165936654	3	3	ENSSSCG00000004875	+	3'UTR	*CYB5A*
1	165936726	165936728	+	165936726	7	11	ENSSSCG00000004875	+	3'UTR	*CYB5A*
1	165937060	165937060	+	165937060	5	5	ENSSSCG00000004875	+	3'UTR	*CYB5A*
1	165937569	165937611	+	165937604	750	912	ENSSSCG00000004875	+	3'UTR	*CYB5A*
1	165938913	165938920	+	165938914	10	16	XLOC_000921	+	predicted	---
1	166038551	166038551	-	166038551	3	3	ENSSSCG00000004872	-	ORF	*TIMM21*
1	166069019	166069019	+	166069019	3	3	ENSSSCG00000004873	+	intron	*FBXO15*
1	166082708	166082708	+	166082708	16	16	ENSSSCG00000004873	+	intron	*FBXO15*
1	166089516	166089518	+	166089516	3	6	---	---	intergenic	---
1	166224562	166224562	-	166224562	4	4	---	---	intergenic	---
1	166232072	166232076	+	166232073	17	34	XLOC_000922	+	predicted	---
1	166820746	166820746	-	166820746	5	5	---	---	intergenic	---
1	166862555	166862555	-	166862555	3	3	---	---	intergenic	---
1	166919190	166919190	+	166919190	7	7	---	---	intergenic	---

1	168040288	168040288	-	168040288	4	4	---	---	intergenic	---
1	168137192	168137192	+	168137192	12	12	---	---	intergenic	---
1	168682151	168682152	-	168682152	11	14	---	---	intergenic	---
1	168988005	168988005	-	168988005	6	6	XLOC_002860	-	predicted	---
1	168991772	168991772	-	168991772	3	3	XLOC_002860	-	predicted	---
1	169115091	169115091	-	169115091	4	4	---	---	intergenic	---
1	169418110	169418111	+	169418110	6	9	XLOC_000931	+	predicted	---
1	169849666	169849666	+	169849666	5	5	---	---	intergenic	---
1	170789607	170789614	+	170789607	8	14	ENSSSCG00000004884	+	3'UTR	*TMX3*
1	170881491	170881491	+	170881491	3	3	---	---	intergenic	---
1	170894575	170894576	-	170894576	9	17	---	---	intergenic	---
1	171751756	171751759	+	171751756	115	170	---	---	intergenic	---
1	171751765	171751769	-	171751769	9	25	---	---	intergenic	---
1	171751807	171751821	+	171751820	22	70	---	---	intergenic	---
1	171751936	171751936	+	171751936	3	3	---	---	intergenic	---
1	171751990	171751990	-	171751990	4	4	---	---	intergenic	---
1	171752048	171752048	-	171752048	6	6	---	---	intergenic	---
1	171752089	171752097	-	171752096	7	24	---	---	intergenic	---
1	171752093	171752129	+	171752125	6	19	---	---	intergenic	---
1	171752418	171752422	-	171752421	11	18	---	---	intergenic	---
1	171752454	171752455	+	171752454	4	7	---	---	intergenic	---
1	171761516	171761516	-	171761516	3	3	---	---	intergenic	---
1	171942059	171942059	-	171942059	3	3	---	---	intergenic	---
1	172015096	172015096	-	172015096	4	4	---	---	intergenic	---
1	172315230	172315230	-	172315230	3	3	---	---	intergenic	---
1	172587653	172587653	+	172587653	4	4	---	---	intergenic	---

1	172603089	172603089	+	172603089	3	3	XLOC_000939	+	predicted	---
1	172727057	172727057	-	172727057	3	3	---	---	intergenic	---
1	172727398	172727398	+	172727398	7	7	---	---	intergenic	---
1	172823361	172823361	-	172823361	22	22	---	---	intergenic	---
1	173133690	173133690	+	173133690	8	8	---	---	intergenic	---
1	173221708	173221708	-	173221708	3	3	---	---	intergenic	---
1	174275736	174275736	+	174275736	3	3	---	---	intergenic	---
1	174444273	174444273	+	174444273	4	4	---	---	intergenic	---
1	174675211	174675211	-	174675211	3	3	---	---	intergenic	---
1	174876428	174876428	+	174876428	6	6	---	---	intergenic	---
1	174877591	174877594	+	174877593	6	12	---	---	intergenic	---
1	174886549	174886549	-	174886549	3	3	---	---	intergenic	---
1	175328936	175328936	-	175328936	5	5	ENSSSCG00000004889	-	3'UTR	*SERPINB10*
1	175466552	175466552	+	175466552	8	8	XLOC_000947	+	predicted	---
1	175517265	175517265	+	175517265	3	3	ENSSSCG00000004890	+	3'UTR	*SERPINB2*
1	175756756	175756756	+	175756756	8	8	XLOC_000950	+	predicted	---
1	175760218	175760218	+	175760218	6	6	XLOC_000950	+	predicted	---
1	175816563	175816563	+	175816563	4	4	ENSSSCG00000025744	+	3'UTR	*KDSR*
1	176119438	176119438	-	176119438	4	4	ENSSSCG00000004896	-	3'UTR	*PHLPP1*
1	176120400	176120400	-	176120400	9	9	ENSSSCG00000004896	-	3'UTR	*PHLPP1*
1	176412876	176412879	+	176412879	7	12	XLOC_000956	+	predicted	---
1	176416402	176416402	+	176416402	6	6	XLOC_000956	+	predicted	---
1	176493757	176493758	+	176493757	5	10	---	---	intergenic	---
1	176510048	176510048	+	176510048	5	5	---	---	intergenic	---
1	176512537	176512538	-	176512538	6	10	---	---	intergenic	---
1	176594928	176594928	-	176594928	3	3	XLOC_002882	-	predicted	---

1	176654297	176654297	-	176654297	3	3	---	---	intergenic	---
1	176670503	176670503	-	176670503	5	5	XLOC_002883	-	predicted	---
1	176679066	176679066	-	176679066	3	3	ENSSSCG00000004899	-	intron	*KIAA1468*
1	176789713	176789713	+	176789713	3	3	ENSSSCG00000004901	+	ORF	*PIGN*
1	176879029	176879044	+	176879029	13	23	XLOC_000960	+	predicted	---
1	177057194	177057214	-	177057201	21	32	XLOC_002884	-	predicted	---
1	177060480	177060480	-	177060480	3	3	XLOC_002884	-	predicted	---
1	177062250	177062250	-	177062250	3	3	XLOC_002884	-	predicted	---
1	177062590	177062590	-	177062590	3	3	XLOC_002884	-	predicted	---
1	177066844	177066844	+	177066844	5	5	---	---	intergenic	---
1	177069702	177069702	+	177069702	3	3	---	---	intergenic	---
1	178398998	178398998	+	178398998	5	5	---	---	intergenic	---
1	178946548	178946548	+	178946548	9	9	ENSSSCG00000004907	+	intron	*CCBE1*
1	178946818	178946818	+	178946818	3	3	ENSSSCG00000004907	+	intron	*CCBE1*
1	179006988	179006988	+	179006988	3	3	ENSSSCG00000004907	+	intron	*CCBE1*
1	179113730	179113730	+	179113730	8	8	ENSSSCG00000004907	+	intron	*CCBE1*
1	179133622	179133622	-	179133622	4	4	---	---	intergenic	---
1	179149218	179149218	+	179149218	4	4	---	---	intergenic	---
1	179189377	179189378	+	179189378	21	24	ENSSSCG00000004908	+	ORF	*LMAN1*
1	179196348	179196353	+	179196348	3	6	ENSSSCG00000004908	+	intron	*LMAN1*
1	179206190	179206203	+	179206202	8	24	XLOC_000971	+	predicted	---
1	179207950	179207950	+	179207950	4	4	XLOC_000971	+	predicted	---
1	179209549	179209550	+	179209549	20	28	XLOC_000971	+	predicted	---
1	179286870	179286878	-	179286871	7	20	XLOC_002892	-	predicted	---
1	179366581	179366581	+	179366581	4	4	XLOC_000973	+	predicted	---
1	179397515	179397515	+	179397515	4	4	ENSSSCG00000004912	+	ORF	*SEC11C*

1	179397650	179397661	+	179397654	100	178	ENSSSCG00000004912	+	3'UTR	*SEC11C*
1	179452175	179452175	-	179452175	5	5	XLOC_002893	-	predicted	---
1	179453543	179453547	-	179453545	26	40	XLOC_002893	-	predicted	---
1	179469694	179469694	+	179469694	3	3	---	---	intergenic	---
1	179701310	179701310	+	179701310	3	3	ENSSSCG00000004917	+	3'UTR	*MALT1*
							ENSSSCG00000004917	+	ORF	*MALT1*
1	179738455	179738455	-	179738455	3	3	---	---	intergenic	---
1	179912219	179912219	-	179912219	3	3	XLOC_002898	-	predicted	---
1	179915457	179915457	-	179915457	3	3	XLOC_002898	-	predicted	---
1	179915872	179915881	-	179915879	11	29	XLOC_002898	-	predicted	---
1	179916108	179916108	-	179916108	3	3	XLOC_002898	-	predicted	---
1	179917424	179917424	-	179917424	4	4	XLOC_002898	-	predicted	---
1	179954912	179954943	-	179954912	3	6	ENSSSCG00000004919	-	3'UTR	*NEDD4L*
1	179955071	179955071	-	179955071	4	4	ENSSSCG00000004919	-	3'UTR	*NEDD4L*
1	180099392	180099392	-	180099392	5	5	---	---	intergenic	---
1	180103336	180103336	-	180103336	5	5	---	---	intergenic	---
1	180119984	180119984	-	180119984	3	3	---	---	intergenic	---
1	180310453	180310453	+	180310453	3	3	XLOC_000986	+	predicted	---
1	180330338	180330338	+	180330338	4	4	---	---	intergenic	---
1	180402597	180402597	+	180402597	3	3	---	---	intergenic	---
1	180516976	180516976	+	180516976	4	4	---	---	intergenic	---
1	180535960	180535960	+	180535960	5	5	---	---	intergenic	---
1	180698289	180698295	-	180698295	42	48	ENSSSCG00000004927	-	3'UTR	*CLPX*
1	180699563	180699563	-	180699563	5	5	ENSSSCG00000004927	-	3'UTR	*CLPX*
1	180699826	180699833	-	180699826	5	11	ENSSSCG00000004927	-	3'UTR	*CLPX*
1	180712259	180712259	-	180712259	3	3	ENSSSCG00000004927	-	ORF	*CLPX*

1	180723016	180723016	-	180723016	3	3	ENSSSCG00000004927	-	intron	*CLPX*
1	180725346	180725346	-	180725346	5	5	ENSSSCG00000004927	-	ORF	*CLPX*
1	180728236	180728236	-	180728236	6	6	ENSSSCG00000004927	-	intron	*CLPX*
1	180737285	180737285	-	180737285	3	3	ENSSSCG00000004927	-	ORF	*CLPX*
1	180817972	180817972	-	180817972	4	4	---	---	intergenic	---
1	180979242	180979242	-	180979242	6	6	ENSSSCG00000004931	-	intron	*IGDCC4*
1	181020984	181020984	-	181020984	12	12	ENSSSCG00000004934	-	3'UTR	*DPP8*
1	181023162	181023162	-	181023162	7	7	ENSSSCG00000004934	-	3'UTR	*DPP8*
1	181140166	181140166	+	181140166	3	3	ENSSSCG00000004935	+	3'UTR	*HACD3*
1	181141066	181141066	+	181141066	3	3	ENSSSCG00000004935	+	3'UTR	*HACD3*
1	181141920	181141935	+	181141920	16	39	ENSSSCG00000004935	+	3'UTR	*HACD3*
1	181170642	181170642	-	181170642	5	5	ENSSSCG00000004936	-	intron	*VWA9*
1	181304576	181304576	-	181304576	3	3	---	---	intergenic	---
1	181305700	181305700	-	181305700	6	6	---	---	intergenic	---
1	181327509	181327509	-	181327509	3	3	ENSSSCG00000004938	-	intron	*DENND4A*
1	181460735	181460735	-	181460735	5	5	ENSSSCG00000022696	-	3'UTR	*RAB11A*
1	181461688	181461696	-	181461695	26	59	ENSSSCG00000022696	-	3'UTR	*RAB11A*
1	181463070	181463072	-	181463071	12	21	ENSSSCG00000022696	-	3'UTR	*RAB11A*
1	181463144	181463144	-	181463144	4	4	ENSSSCG00000022696	-	3'UTR	*RAB11A*
1	181463231	181463237	-	181463233	48	80	ENSSSCG00000022696	-	3'UTR	*RAB11A*
1	182110328	182110328	-	182110328	10	10	ENSSSCG00000004942	-	3'UTR	*TIPIN*
1	182122481	182122481	-	182122481	11	11	ENSSSCG00000004942	-	intron	*TIPIN*
1	182184613	182184615	+	182184615	4	7	ENSSSCG00000023980	+	intron	*SNAPC5*
1	182185412	182185418	+	182185418	8	13	ENSSSCG00000023980	+	intron	*SNAPC5*
1	182189346	182189346	+	182189346	3	3	ENSSSCG00000023980	+	3'UTR	*SNAPC5*
1	182220962	182220962	-	182220962	3	3	ENSSSCG00000004943	-	intron	*MAP2K1*

1	182226366	182226391	-	182226367	449	2590	ENSSSCG00000004945	-	3'UTR	*RPL4*
1	182226478	182226527	-	182226498	7	20	ENSSSCG00000004945	-	ORF	*RPL4*
1	182226831	182226831	-	182226831	5	5	ENSSSCG00000004945	-	ORF	*RPL4*
1	182227006	182227006	-	182227006	3	3	ENSSSCG00000004945	-	intron	*RPL4*
							ENSSSCG00000004945	-	ORF	*RPL4*
1	182269606	182269607	+	182269606	70	74	---	---	intergenic	---
1	182312752	182312752	+	182312752	5	5	---	---	intergenic	---
1	182316901	182316901	+	182316901	6	6	---	---	intergenic	---
1	182453927	182453927	+	182453927	3	3	ENSSSCG00000004948	+	intron	*SMAD6*
1	182559393	182559393	+	182559393	3	3	---	---	intergenic	---
1	182650951	182650951	+	182650951	3	3	---	---	intergenic	---
1	182892612	182892612	+	182892612	8	8	ENSSSCG00000004951	+	3'UTR	*AAGAB*
1	182893163	182893165	+	182893165	7	11	ENSSSCG00000004951	+	3'UTR	*AAGAB*
1	182898816	182898816	-	182898816	5	5	ENSSSCG00000004952	-	3'UTR	*SMAD2*
1	182921723	182921723	-	182921723	3	3	ENSSSCG00000004952	-	intron	*SMAD2*
1	182996207	182996207	-	182996207	3	3	---	---	intergenic	---
1	182996238	182996248	+	182996248	10	28	---	---	intergenic	---
1	183131810	183131820	+	183131818	50	116	ENSSSCG00000004954	+	3'UTR	*C15orf61*
1	183186458	183186458	+	183186458	4	4	ENSSSCG00000004955	+	intron	---
1	183510453	183510466	+	183510466	9	22	XLOC_001018	+	predicted	---
1	183539066	183539066	+	183539066	3	3	XLOC_001020	+	predicted	---
1	183878603	183878603	-	183878603	5	5	ENSSSCG00000004957	-	3'UTR	*CLN6*
1	183929849	183929849	+	183929849	7	7	ENSSSCG00000022419	+	3'UTR	*FEM1B*
							ENSSSCG00000004958	+	intron	---
1	183932214	183932214	+	183932214	4	4	ENSSSCG00000022419	+	3'UTR	*FEM1B*
							ENSSSCG00000004958	+	intron	---

1	183934184	183934185	+	183934185	7	10	ENSSSCG00000022419	+	3'UTR	*FEM1B*
							ENSSSCG00000004958	+	intron	---
1	183948938	183948941	+	183948941	5	8	XLOC_001021	+	predicted	---
1	183952512	183952512	+	183952512	10	10	XLOC_001021	+	predicted	---
1	184102484	184102484	+	184102484	3	3	---	---	intergenic	---
1	184153759	184153764	+	184153759	3	6	---	---	intergenic	---
1	184440672	184440675	+	184440674	16	22	XLOC_001026	+	predicted	---
1	184441055	184441055	+	184441055	3	3	XLOC_001026	+	predicted	---
1	184442299	184442301	+	184442299	4	7	XLOC_001026	+	predicted	---
1	184592896	184592900	-	184592900	7	11	ENSSSCG00000004963	-	3'UTR	---
1	184898190	184898190	-	184898190	4	4	---	---	intergenic	---
1	185023468	185023468	+	185023468	3	3	ENSSSCG00000026078	+	intron	*GLCE*
1	185092462	185092464	+	185092464	15	18	XLOC_001034	+	predicted	---
1	185176372	185176372	-	185176372	7	7	---	---	intergenic	---
1	185178605	185178605	+	185178605	4	4	---	---	intergenic	---
1	185184001	185184044	+	185184015	7	25	ENSSSCG00000004968	+	intron	*PAQR5*
1	185184236	185184236	+	185184236	14	14	ENSSSCG00000004968	+	intron	*PAQR5*
1	185202976	185202988	+	185202987	144	654	ENSSSCG00000004968	+	3'UTR	*PAQR5*
1	185203088	185203089	+	185203088	5	10	ENSSSCG00000004968	+	3'UTR	*PAQR5*
1	185286222	185286222	+	185286222	6	6	ENSSSCG00000004969	+	intron	*KIF23*
1	185303337	185303337	+	185303337	4	4	ENSSSCG00000004969	+	3'UTR	*KIF23*
1	185303423	185303429	+	185303429	7	14	ENSSSCG00000004969	+	3'UTR	*KIF23*
1	185384983	185384987	-	185384985	421	514	XLOC_002951	-	predicted	---
1	185385328	185385332	-	185385328	14	29	ENSSSCG00000004970	-	intron	---
1	185391837	185391837	+	185391837	10	10	---	---	intergenic	---
1	185821264	185821264	+	185821264	4	4	---	---	intergenic	---

1	186043534	186043538	+	186043537	8	14	---	---	intergenic	---
1	186217387	186217387	+	186217387	3	3	XLOC_001043	+	predicted	---
1	186541096	186541096	-	186541096	10	10	---	---	intergenic	---
1	186559167	186559167	-	186559167	3	3	---	---	intergenic	---
1	186637638	186637641	-	186637638	4	7	ENSSSCG00000004973	-	3'UTR	*UACA*
1	186843710	186843710	+	186843710	5	5	ENSSSCG00000021937	+	intron	---
1	187068234	187068238	-	187068238	47	89	---	---	intergenic	---
1	187396902	187396923	-	187396923	47	339	---	---	intergenic	---
1	187849027	187849027	+	187849027	3	3	ENSSSCG00000004980	+	intron	*THSD4*
1	187978442	187978443	+	187978442	6	12	ENSSSCG00000020940	+	3'UTR	---
							ENSSSCG00000004980	+	intron	*THSD4*
1	187985763	187985763	+	187985763	3	3	ENSSSCG00000004980	+	intron	*THSD4*
1	188013286	188013297	+	188013289	16	81	ENSSSCG00000004980	+	intron	*THSD4*
1	188119157	188119157	-	188119157	23	23	ENSSSCG00000004979	-	intron	---
1	188143618	188143618	-	188143618	3	3	ENSSSCG00000004979	-	intron	---
1	188402797	188402808	-	188402797	5	17	ENSSSCG00000022828	-	3'UTR	*PNN*
1	188403741	188403741	-	188403741	4	4	ENSSSCG00000022828	-	3'UTR	*PNN*
1	188406087	188406087	-	188406087	4	4	ENSSSCG00000022828	-	intron	*PNN*
							ENSSSCG00000022828	-	ORF	*PNN*
1	188411749	188411749	+	188411749	3	3	---	---	intergenic	---
1	188424804	188424812	+	188424804	4	7	ENSSSCG00000004986	+	3'UTR	*TRAPPC6B*
1	188425849	188425856	+	188425849	34	43	ENSSSCG00000004986	+	3'UTR	*TRAPPC6B*
1	188436388	188436401	-	188436401	7	16	ENSSSCG00000004985	-	3'UTR	---
1	188630121	188630122	+	188630122	19	22	---	---	intergenic	---
1	188780037	188780037	-	188780037	3	3	---	---	intergenic	---
1	189474219	189474219	-	189474219	6	6	---	---	intergenic	---

1	189918838	189918838	-	189918838	3	3	---	---	intergenic	---
1	190012241	190012241	-	190012241	3	3	---	---	intergenic	---
1	190656467	190656467	+	190656467	7	7	---	---	intergenic	---
1	190683939	190683939	+	190683939	6	6	---	---	intergenic	---
1	191716582	191716582	-	191716582	3	3	---	---	intergenic	---
1	192716273	192716273	+	192716273	4	4	---	---	intergenic	---
1	193068359	193068359	-	193068359	7	7	---	---	intergenic	---
1	193087447	193087447	-	193087447	6	6	---	---	intergenic	---
1	193278754	193278755	+	193278754	5	8	---	---	intergenic	---
1	193489662	193489662	+	193489662	4	4	---	---	intergenic	---
1	194215741	194215741	-	194215741	3	3	---	---	intergenic	---
1	194276042	194276042	-	194276042	13	13	---	---	intergenic	---
1	194528270	194528270	+	194528270	4	4	ENSSSCG00000004992	+	ORF	*FSCB*
1	194530040	194530045	+	194530045	19	27	XLOC_001061	+	predicted	---
1	194672637	194672637	-	194672637	3	3	---	---	intergenic	---
1	195142183	195142183	+	195142183	3	3	ENSSSCG00000027765	+	3'UTR	*FAM179B*
1	195142628	195142628	+	195142628	7	7	ENSSSCG00000027765	+	3'UTR	*FAM179B*
1	195153731	195153736	+	195153736	12	20	ENSSSCG00000004997	+	intron	*PRPF39*
1	195185567	195185567	+	195185567	11	11	XLOC_001066	+	predicted	---
1	195185881	195185884	-	195185883	44	71	XLOC_002978	-	predicted	---
1	195186118	195186118	-	195186118	3	3	XLOC_002978	-	predicted	---
1	195187301	195187301	-	195187301	6	6	ENSSSCG00000004998	-	ORF	*FKBP3*
1	195188829	195188829	-	195188829	5	5	ENSSSCG00000004998	-	ORF	*FKBP3*
1	195189621	195189631	-	195189621	19	25	ENSSSCG00000004998	-	intron	*FKBP3*
1	195219434	195219434	+	195219434	5	5	ENSSSCG00000004999	+	3'UTR	*MIS18BP1*
1	195292028	195292032	+	195292028	6	10	ENSSSCG00000021217	+	ORF	---

1	195296928	195296928	+	195296928	3	3	---	---	intergenic	---
1	196090870	196090870	-	196090870	14	14	---	---	intergenic	---
1	196196371	196196371	-	196196371	6	6	---	---	intergenic	---
1	196293335	196293335	+	196293335	4	4	---	---	intergenic	---
1	196499451	196499454	+	196499454	17	28	---	---	intergenic	---
1	196774233	196774238	-	196774235	99	230	XLOC_002981	-	predicted	---
1	197044521	197044523	-	197044521	5	8	ENSSSCG00000005002	-	intron	*MDGA2*
1	197300012	197300013	+	197300012	23	34	---	---	intergenic	---
1	197547138	197547138	+	197547138	4	4	---	---	intergenic	---
1	197706792	197706792	+	197706792	3	3	---	---	intergenic	---
1	197861382	197861382	+	197861382	3	3	---	---	intergenic	---
1	198437072	198437072	-	198437072	8	8	---	---	intergenic	---
1	198903988	198903988	+	198903988	8	8	---	---	intergenic	---
1	198942566	198942605	-	198942573	12	90	---	---	intergenic	---
1	198942628	198942639	+	198942629	16	81	---	---	intergenic	---
1	198942731	198942731	+	198942731	9	9	---	---	intergenic	---
1	198961699	198961699	-	198961699	3	3	---	---	intergenic	---
1	198961789	198961789	+	198961789	3	3	---	---	intergenic	---
1	199307133	199307134	+	199307134	22	32	---	---	intergenic	---
1	199653101	199653101	-	199653101	3	3	---	---	intergenic	---
1	199696777	199696781	+	199696778	442	468	ENSSSCG00000005003	+	3'UTR	---
1	199863011	199863058	-	199863017	420	1072	ENSSSCG00000005005	-	3'UTR	*RPL36A*
1	199866084	199866085	+	199866084	3	6	ENSSSCG00000005006	+	ORF	---
1	199866920	199866923	+	199866922	10	26	XLOC_001073	+	predicted	---
1	199868584	199868584	-	199868584	3	3	XLOC_002988	-	predicted	---
1	199957841	199957844	+	199957841	38	53	ENSSSCG00000005009	+	3'UTR	*KLHDC2*

1	199957891	199957892	+	199957891	5	8	ENSSSCG00000005009	+	3'UTR	*KLHDC2*
1	199966223	199966223	-	199966223	6	6	ENSSSCG00000005011	-	intron	*NEMF*
1	200071304	200071308	-	200071306	28	67	XLOC_002992	-	predicted	---
1	200073548	200073555	-	200073553	40	79	XLOC_002992	-	predicted	---
1	200261252	200261252	+	200261252	5	5	XLOC_001082	+	predicted	---
1	200281365	200281365	-	200281365	6	6	ENSSSCG00000005015	-	intron	*SOS2*
1	200362055	200362055	-	200362055	5	5	ENSSSCG00000005016	-	3'UTR	*L2HGDH*
1	200418706	200418707	+	200418706	5	9	ENSSSCG00000005017	+	ORF	*ATP5S*
1	200421188	200421189	+	200421188	11	15	ENSSSCG00000005017	+	3'UTR	*ATP5S*
1	200421344	200421344	+	200421344	3	3	ENSSSCG00000005017	+	3'UTR	*ATP5S*
1	200535397	200535397	+	200535397	3	3	---	---	intergenic	---
1	200582720	200582720	+	200582720	4	4	---	---	intergenic	---
1	200766290	200766290	-	200766290	8	8	XLOC_003001	-	predicted	---
1	200948935	200948948	-	200948940	5	12	ENSSSCG00000005024	-	ORF	*PYGL*
1	200954937	200954937	-	200954937	23	23	ENSSSCG00000005024	-	intron	*PYGL*
1	200957419	200957419	-	200957419	7	7	ENSSSCG00000005024	-	intron	*PYGL*
1	201230160	201230160	+	201230160	52	52	ENSSSCG00000021213	+	ORF	*TMX1*
1	201235360	201235373	+	201235362	21	42	ENSSSCG00000021213	+	3'UTR	*TMX1*
1	201288285	201288285	-	201288285	4	4	---	---	intergenic	---
1	201407399	201407399	+	201407399	3	3	---	---	intergenic	---
1	201516848	201516848	+	201516848	5	5	---	---	intergenic	---
1	201605080	201605080	+	201605080	5	5	---	---	intergenic	---
1	201697942	201697942	+	201697942	3	3	ENSSSCG00000005027	+	3'UTR	*FRMD6*
1	201698224	201698228	+	201698224	5	8	XLOC_001092	+	predicted	---
1	201719277	201719277	-	201719277	3	3	---	---	intergenic	---
1	201891065	201891070	-	201891070	16	25	ENSSSCG00000028092	-	3'UTR	*GNG2*

1	201893526	201893526	-	201893526	5	5	ENSSSCG00000028092	-	3'UTR	*GNG2*
1	201893889	201893889	-	201893889	6	6	ENSSSCG00000028092	-	3'UTR	*GNG2*
1	201953279	201953279	+	201953279	7	7	ENSSSCG00000005029	+	ORF	*C14orf166*
1	201970939	201970945	-	201970939	7	14	XLOC_003007	-	predicted	---
1	202264243	202264249	-	202264243	7	12	---	---	intergenic	---
1	202432982	202432982	-	202432982	3	3	ENSSSCG00000027537	-	intron	---
1	202433118	202433120	-	202433118	3	6	ENSSSCG00000027537	-	intron	---
1	202534953	202534953	-	202534953	3	3	ENSSSCG00000005037	-	intron	*ERO1L*
1	202599845	202599845	+	202599845	3	3	ENSSSCG00000005038	+	intron	*PSMC6*
1	202625181	202625185	+	202625181	9	17	ENSSSCG00000005038	+	3'UTR	*PSMC6*
1	202625489	202625501	+	202625500	29	101	ENSSSCG00000005038	+	3'UTR	*PSMC6*
1	202673902	202673903	+	202673903	4	7	ENSSSCG00000005039	+	intron	*STYX*
1	202674997	202674997	+	202674997	3	3	XLOC_001099	+	predicted	---
1	202678285	202678291	+	202678291	6	10	XLOC_001099	+	predicted	---
1	202680650	202680673	-	202680651	9	36	ENSSSCG00000005040	-	3'UTR	*GNPNAT1*
1	202790226	202790226	-	202790226	5	5	ENSSSCG00000005041	-	3'UTR	*FERMT2*
1	202790804	202790804	-	202790804	3	3	ENSSSCG00000005041	-	3'UTR	*FERMT2*
1	202791225	202791231	-	202791225	7	10	ENSSSCG00000005041	-	3'UTR	*FERMT2*
1	202793858	202793858	-	202793858	3	3	ENSSSCG00000005041	-	intron	*FERMT2*
1	202934426	202934426	-	202934426	4	4	ENSSSCG00000005043	-	3'UTR	*DDHD1*
1	202935099	202935122	-	202935099	6	15	ENSSSCG00000005043	-	3'UTR	*DDHD1*
1	202975430	202975430	-	202975430	3	3	ENSSSCG00000005043	-	intron	*DDHD1*
1	203066732	203066732	+	203066732	6	6	---	---	intergenic	---
1	203170721	203170721	+	203170721	3	3	---	---	intergenic	---
1	203192271	203192271	-	203192271	4	4	---	---	intergenic	---
1	203421944	203421944	-	203421944	3	3	---	---	intergenic	---

1	203424749	203424749	-	203424749	9	9	---	---	intergenic	---
1	204213940	204213940	+	204213940	6	6	XLOC_001107	+	predicted	---
1	204214551	204214552	+	204214551	7	10	XLOC_001107	+	predicted	---
1	204217675	204217676	+	204217676	4	7	---	---	intergenic	---
1	204224615	204224620	+	204224615	5	8	XLOC_001107	+	predicted	---
1	204297681	204297681	+	204297681	3	3	ENSSSCG00000005047	+	intron	*CDKN3*
1	204304768	204304779	+	204304770	17	70	XLOC_001110	+	predicted	---
1	204306983	204306990	-	204306983	7	11	---	---	intergenic	---
1	204310634	204310647	-	204310646	10	19	---	---	intergenic	---
1	204375030	204375035	+	204375035	23	27	XLOC_001111	+	predicted	---
1	204424884	204424884	+	204424884	3	3	ENSSSCG00000021451	+	3'UTR	---
1	204425129	204425129	+	204425129	6	6	ENSSSCG00000021451	+	3'UTR	---
1	204425461	204425461	+	204425461	3	3	ENSSSCG00000021451	+	3'UTR	---
1	204538822	204538822	+	204538822	3	3	ENSSSCG00000023408	+	intron	*SAMD4A*
1	204554008	204554008	+	204554008	5	5	ENSSSCG00000023408	+	ORF	*SAMD4A*
1	204640984	204641003	+	204640995	49	165	ENSSSCG00000023408	+	3'UTR	*SAMD4A*
1	204645191	204645191	+	204645191	4	4	ENSSSCG00000023408	+	3'UTR	*SAMD4A*
1	204684913	204684913	-	204684913	9	9	---	---	intergenic	---
1	204710344	204710344	-	204710344	5	5	XLOC_003021	-	predicted	---
1	204780492	204780492	-	204780492	3	3	ENSSSCG00000005052	-	3'UTR	*WDHD1*
1	204876991	204876993	-	204876993	10	14	---	---	intergenic	---
1	204906100	204906100	+	204906100	3	3	XLOC_001118	+	predicted	---
1	204918171	204918171	+	204918171	7	7	ENSSSCG00000005054	+	ORF	*MAPK1IP1L*
1	204919936	204919936	+	204919936	7	7	ENSSSCG00000005054	+	3'UTR	*MAPK1IP1L*
1	204974106	204974122	+	204974108	51	63	ENSSSCG00000005055	+	3'UTR	*LGALS3*
							ENSSSCG00000005055	+	ORF	---

1	204978747	204978747	-	204978747	5	5	XLOC_003024	-	predicted	---
1	204979103	204979103	-	204979103	3	3	ENSSSCG00000005056	-	3'UTR	*DLGAP5*
1	204991119	204991122	-	204991121	111	178	ENSSSCG00000005056	-	intron	*DLGAP5*
1	204995621	204995621	-	204995621	3	3	ENSSSCG00000005056	-	ORF	*DLGAP5*
1	205007810	205007810	-	205007810	7	7	ENSSSCG00000005056	-	intron	*DLGAP5*
1	205011605	205011605	-	205011605	6	6	ENSSSCG00000005056	-	intron	*DLGAP5*
1	205079844	205079844	+	205079844	4	4	---	---	intergenic	---
1	205239909	205239917	+	205239916	10	19	XLOC_001121	+	predicted	---
1	205262635	205262635	-	205262635	4	4	ENSSSCG00000005058	-	intron	*ATG14*
1	205465631	205465631	+	205465631	3	3	ENSSSCG00000005060	+	ORF	*KTN1*
1	205465934	205465934	+	205465934	7	7	ENSSSCG00000005060	+	ORF	*KTN1*
1	205472186	205472186	+	205472186	10	10	ENSSSCG00000005060	+	ORF	*KTN1*
1	205479144	205479145	+	205479144	12	18	ENSSSCG00000005060	+	intron	*KTN1*
1	205481307	205481320	+	205481310	5	12	ENSSSCG00000005060	+	intron	*KTN1*
1	205496639	205496639	+	205496639	19	19	ENSSSCG00000005060	+	intron	*KTN1*
1	205503442	205503443	+	205503442	14	18	ENSSSCG00000005060	+	intron	*KTN1*
1	205505938	205505944	+	205505943	33	46	ENSSSCG00000005060	+	intron	*KTN1*
1	205509438	205509439	+	205509439	14	17	ENSSSCG00000005060	+	intron	*KTN1*
1	205529819	205529819	+	205529819	4	4	ENSSSCG00000005060	+	intron	*KTN1*
1	205540755	205540755	+	205540755	4	4	---	---	intergenic	---
1	205540884	205540886	+	205540884	3	6	---	---	intergenic	---
1	205541064	205541071	+	205541069	69	117	---	---	intergenic	---
1	205896572	205896572	+	205896572	5	5	---	---	intergenic	---
1	206184998	206184998	+	206184998	7	7	ENSSSCG00000021134	+	intron	*PELI2*
1	206214298	206214298	+	206214298	5	5	ENSSSCG00000021134	+	intron	*PELI2*
1	206220667	206220667	+	206220667	3	3	ENSSSCG00000021134	+	3'UTR	*PELI2*

1	206258589	206258589	-	206258589	4	4	---	---	intergenic	---
1	206288641	206288641	-	206288641	7	7	---	---	intergenic	---
1	206412946	206412946	-	206412946	3	3	XLOC_003030	-	predicted	---
1	206420402	206420402	-	206420402	3	3	---	---	intergenic	---
1	206503666	206503672	+	206503666	5	16	ENSSSCG00000005062	+	3'UTR	*TMEM260*
1	206503741	206503746	+	206503744	6	14	ENSSSCG00000005062	+	3'UTR	*TMEM260*
1	206505057	206505057	+	206505057	3	3	ENSSSCG00000005062	+	3'UTR	*TMEM260*
1	206634316	206634316	+	206634316	4	4	---	---	intergenic	---
1	206642526	206642526	-	206642526	6	6	ENSSSCG00000005063	-	3'UTR	*OTX2*
1	206679712	206679712	-	206679712	5	5	---	---	intergenic	---
1	206780755	206780756	-	206780756	7	10	---	---	intergenic	---
1	206792837	206792837	+	206792837	3	3	XLOC_001133	+	predicted	---
1	206823481	206823481	+	206823481	3	3	---	---	intergenic	---
1	206906597	206906597	-	206906597	3	3	XLOC_003034	-	predicted	---
1	206906816	206906816	-	206906816	3	3	XLOC_003034	-	predicted	---
1	207027555	207027555	+	207027555	3	3	ENSSSCG00000005065	+	3'UTR	*AP5M1*
1	207122082	207122093	+	207122093	7	11	XLOC_001136	+	predicted	---
1	207124914	207124914	+	207124914	3	3	XLOC_001136	+	predicted	---
1	207348141	207348143	-	207348142	25	33	---	---	intergenic	---
1	207886683	207886689	-	207886688	20	34	XLOC_003040	-	predicted	---
1	208057298	208057298	-	208057298	3	3	---	---	intergenic	---
1	208194398	208194398	+	208194398	5	5	---	---	intergenic	---
1	208238132	208238150	+	208238142	18	54	ENSSSCG00000022299	+	3'UTR	---
1	208238673	208238675	+	208238674	16	43	ENSSSCG00000022299	+	3'UTR	---
1	208271962	208271962	+	208271962	10	10	ENSSSCG00000005072	+	ORF	*PSMA3*
1	208274214	208274224	+	208274218	341	427	ENSSSCG00000005072	+	3'UTR	*PSMA3*

1	208338675	208338675	+	208338675	22	22	ENSSSCG00000005073	+	intron	*ARID4A*
1	208356174	208356180	+	208356180	6	10	ENSSSCG00000005073	+	3'UTR	*ARID4A*
1	208356693	208356693	+	208356693	3	3	---	---	intergenic	---
1	208389460	208389462	-	208389461	11	21	ENSSSCG00000027270	-	3'UTR	*TIMM9*
1	208389513	208389515	+	208389513	10	16	XLOC_001146	+	predicted	---
1	208389558	208389560	-	208389559	19	30	ENSSSCG00000027270	-	3'UTR	*TIMM9*
1	208694411	208694411	+	208694411	3	3	---	---	intergenic	---
1	209045977	209045990	+	209045977	11	21	ENSSSCG00000005077	+	intron	---
1	209062855	209062855	+	209062855	3	3	XLOC_001150	+	predicted	---
1	209072017	209072018	-	209072018	8	12	XLOC_003044	-	predicted	---
1	209097482	209097482	-	209097482	4	4	---	---	intergenic	---
1	209102238	209102238	-	209102238	6	6	XLOC_003044	-	predicted	---
1	209433166	209433166	-	209433166	3	3	---	---	intergenic	---
1	210152838	210152838	+	210152838	3	3	XLOC_001154	+	predicted	---
1	210232550	210232550	-	210232550	5	5	XLOC_003051	-	predicted	---
1	210275975	210275979	-	210275977	56	114	ENSSSCG00000005083	-	3'UTR	*DHRS7*
1	210278652	210278652	-	210278652	3	3	ENSSSCG00000005083	-	ORF	*DHRS7*
1	210330142	210330142	+	210330142	5	5	XLOC_001157	+	predicted	---
1	210364562	210364562	-	210364562	3	3	---	---	intergenic	---
1	210381722	210381730	+	210381722	3	12	XLOC_001158	+	predicted	---
1	210384605	210384605	-	210384605	3	3	---	---	intergenic	---
1	210397288	210397288	+	210397288	6	6	ENSSSCG00000005084	+	intron	*PPM1A*
1	210401330	210401346	+	210401331	5	16	XLOC_001159	+	predicted	---
1	210401962	210401962	+	210401962	4	4	XLOC_001159	+	predicted	---
1	210402276	210402277	+	210402276	5	10	XLOC_001159	+	predicted	---
1	210407062	210407062	+	210407062	3	3	XLOC_001159	+	predicted	---

1	210516815	210516815	-	210516815	3	3	XLOC_003055	-	predicted	---
1	210614621	210614621	+	210614621	3	3	XLOC_001160	+	predicted	---
1	211166857	211166857	+	211166857	3	3	---	---	intergenic	---
1	211190428	211190428	+	211190428	18	18	XLOC_001164	+	predicted	---
1	211190705	211190705	+	211190705	4	4	XLOC_001164	+	predicted	---
1	211196393	211196397	-	211196393	3	6	ENSSSCG00000024529	-	3'UTR	*TRMT5*
1	211249023	211249024	+	211249023	25	31	ENSSSCG00000005092	+	intron	*SLC38A6*
1	211468908	211468908	+	211468908	3	3	---	---	intergenic	---
1	211524142	211524145	-	211524143	19	35	XLOC_003060	-	predicted	---
1	211817807	211817807	+	211817807	9	9	ENSSSCG00000005095	+	3'UTR	*PKC*
1	212031937	212031937	+	212031937	5	5	---	---	intergenic	---
1	212057692	212057692	+	212057692	3	3	---	---	intergenic	---
1	212070784	212070784	-	212070784	3	3	---	---	intergenic	---
1	212180807	212180814	+	212180813	51	82	XLOC_001171	+	predicted	---
1	212192700	212192700	+	212192700	7	7	ENSSSCG00000005097	+	intron	*SNAPC1*
1	212203478	212203478	+	212203478	15	15	ENSSSCG00000005097	+	ORF	*SNAPC1*
1	212218508	212218516	+	212218510	49	69	ENSSSCG00000005097	+	3'UTR	*SNAPC1*
1	212274129	212274129	-	212274129	3	3	---	---	intergenic	---
1	212400805	212400806	-	212400805	20	32	---	---	intergenic	---
1	212488252	212488257	+	212488252	10	22	XLOC_001174	+	predicted	---
1	212564281	212564285	+	212564281	4	14	---	---	intergenic	---
1	212616903	212616903	-	212616903	18	18	XLOC_003067	-	predicted	---
1	212633430	212633437	-	212633437	23	34	ENSSSCG00000005102	-	3'UTR	*AEN*
1	212698196	212698196	+	212698196	5	5	ENSSSCG00000005103	+	intron	*DET1*
1	212717929	212717929	+	212717929	6	6	ENSSSCG00000005103	+	3'UTR	*DET1*
1	212743516	212743523	-	212743520	30	105	ENSSSCG00000005104	-	3'UTR	*MRPS11*

1	212764266	212764273	+	212764270	31	98	ENSSSCG00000005105	+	3'UTR	*MRPL46*
1	213047441	213047441	+	213047441	26	26	ENSSSCG00000005106	+	intron	*NTRK3*
1	213912649	213912649	+	213912649	5	5	XLOC_001183	+	predicted	---
1	215029427	215029427	-	215029427	3	3	---	---	intergenic	---
1	215499894	215499894	+	215499894	3	3	---	---	intergenic	---
1	215795840	215795842	-	215795840	4	7	ENSSSCG00000005110	-	3'UTR	*SYNE2*
1	215796387	215796387	-	215796387	3	3	ENSSSCG00000005110	-	3'UTR	*SYNE2*
1	215842275	215842275	-	215842275	3	3	---	---	intergenic	---
1	215884334	215884337	-	215884334	10	14	ENSSSCG00000005110	-	intron	*SYNE2*
1	215892943	215892943	-	215892943	5	5	---	---	intergenic	---
1	215951697	215951697	-	215951697	4	4	ENSSSCG00000005110	-	intron	*SYNE2*
1	216022332	216022332	-	216022332	3	3	ENSSSCG00000005110	-	intron	*SYNE2*
1	216143178	216143184	-	216143181	4	13	---	---	intergenic	---
1	216143832	216143832	+	216143832	6	6	---	---	intergenic	---
1	216316107	216316107	+	216316107	3	3	ENSSSCG00000028558	+	intron	*SGPP1*
1	216445623	216445623	-	216445623	3	3	XLOC_003086	-	predicted	---
1	216590828	216590828	-	216590828	3	3	ENSSSCG00000026382	-	3'UTR	---
1	216592733	216592733	-	216592733	5	5	ENSSSCG00000026382	-	3'UTR	---
1	216593552	216593552	-	216593552	3	3	ENSSSCG00000026382	-	3'UTR	---
1	216594508	216594508	-	216594508	3	3	ENSSSCG00000026382	-	3'UTR	---
1	216599486	216599488	-	216599486	7	11	ENSSSCG00000026382	-	intron	---
1	216606063	216606063	-	216606063	4	4	ENSSSCG00000026382	-	intron	---
1	216958959	216958959	-	216958959	3	3	---	---	intergenic	---
1	217483055	217483055	-	217483055	3	3	---	---	intergenic	---
1	217580173	217580173	+	217580173	3	3	---	---	intergenic	---
1	217913707	217913707	-	217913707	3	3	---	---	intergenic	---

1	218124725	218124725	+	218124725	4	4	---	---	intergenic	---
1	218224166	218224168	-	218224168	13	21	ENSSSCG00000005122	-	3'UTR	*TEK*
1	218575626	218575626	-	218575626	3	3	ENSSSCG00000005124	-	ORF	*IFT74*
1	218668145	218668148	+	218668146	12	29	ENSSSCG00000005126	+	3'UTR	*PLAA*
1	218684206	218684206	+	218684206	10	10	ENSSSCG00000005127	+	intron	*CAAP1*
1	218721486	218721486	+	218721486	5	5	ENSSSCG00000005127	+	3'UTR	*CAAP1*
1	218722412	218722412	+	218722412	4	4	ENSSSCG00000005127	+	3'UTR	*CAAP1*
1	219727394	219727398	+	219727398	4	7	XLOC_001203	+	predicted	---
1	220693034	220693034	-	220693034	4	4	---	---	intergenic	---
1	220787022	220787022	-	220787022	4	4	XLOC_003096	-	predicted	---
1	221069846	221069850	+	221069850	6	12	XLOC_001206	+	predicted	---
1	221070016	221070034	+	221070033	24	102	XLOC_001206	+	predicted	---
1	221861954	221861954	+	221861954	8	8	---	---	intergenic	---
1	221867227	221867233	-	221867227	5	9	---	---	intergenic	---
1	221867245	221867245	+	221867245	4	4	---	---	intergenic	---
1	222480379	222480379	+	222480379	12	12	---	---	intergenic	---
1	222596639	222596639	-	222596639	3	3	---	---	intergenic	---
1	222937994	222938000	+	222938000	8	23	---	---	intergenic	---
1	223574974	223574974	-	223574974	4	4	---	---	intergenic	---
1	223666125	223666125	+	223666125	7	7	---	---	intergenic	---
1	223732489	223732489	+	223732489	6	6	---	---	intergenic	---
1	223852374	223852374	+	223852374	8	8	XLOC_001216	+	predicted	---
1	223940614	223940622	-	223940615	16	30	XLOC_003105	-	predicted	---
1	223965115	223965115	-	223965115	7	7	XLOC_003105	-	predicted	---
1	224016183	224016215	+	224016215	12	26	---	---	intergenic	---
1	224016188	224016188	-	224016188	3	3	---	---	intergenic	---

1	224075795	224075796	-	224075796	6	9	ENSSSCG00000005135	-	3'UTR	---
1	224229364	224229364	-	224229364	3	3	---	---	intergenic	---
1	224307151	224307151	-	224307151	5	5	---	---	intergenic	---
1	224603534	224603534	-	224603534	4	4	ENSSSCG00000029085	-	intron	*POIFN-ALPHA*
1	224955488	224955488	-	224955488	5	5	XLOC_003124	-	predicted	---
1	225615119	225615120	+	225615120	7	10	---	---	intergenic	---
1	225787030	225787030	-	225787030	3	3	ENSSSCG00000028406	-	intron	---
1	225806170	225806170	-	225806170	4	4	ENSSSCG00000028406	-	intron	---
1	225853845	225853845	-	225853845	3	3	---	---	intergenic	---
1	225885952	225885964	-	225885961	33	85	---	---	intergenic	---
1	226408368	226408368	+	226408368	12	12	---	---	intergenic	---
1	226571900	226571903	+	226571901	63	80	---	---	intergenic	---
1	227035772	227035773	-	227035772	11	16	ENSSSCG00000026955	-	3'UTR	*ACER2*
1	227070800	227070800	+	227070800	3	3	---	---	intergenic	---
1	227097825	227097825	+	227097825	4	4	ENSSSCG00000005169	+	ORF	*RPS6*
1	227098091	227098091	+	227098091	5	5	ENSSSCG00000005169	+	ORF	*RPS6*
1	227098136	227098141	+	227098138	274	480	ENSSSCG00000005169	+	3'UTR	*RPS6*
1	227100331	227100331	-	227100331	7	7	XLOC_003141	-	predicted	---
1	227140679	227140681	-	227140679	3	6	ENSSSCG00000005170	-	intron	*DENND4C*
1	227284742	227284742	+	227284742	5	5	---	---	intergenic	---
1	227339930	227339930	+	227339930	3	3	ENSSSCG00000005171	+	ORF	*ADFP*
1	227340061	227340077	+	227340064	38	59	ENSSSCG00000005171	+	ORF	*ADFP*
1	227344571	227344578	+	227344575	53	163	ENSSSCG00000005171	+	3'UTR	*ADFP*
1	227344714	227344723	+	227344717	67	133	ENSSSCG00000005171	+	3'UTR	*ADFP*
1	227356906	227356915	+	227356908	10	17	XLOC_001268	+	predicted	---

1	227357116	227357123	+	227357116	28	79	XLOC_001268	+	predicted	---
1	227419618	227419623	-	227419618	26	32	XLOC_003142	-	predicted	---
1	227419661	227419662	-	227419661	8	15	XLOC_003142	-	predicted	---
1	227420783	227420783	-	227420783	3	3	ENSSSCG00000005174	-	ORF	*RRAGA*
1	228443318	228443319	-	228443319	27	30	---	---	intergenic	---
1	228678270	228678270	-	228678270	16	16	---	---	intergenic	---
1	229065310	229065310	+	229065310	3	3	---	---	intergenic	---
1	229235384	229235384	-	229235384	3	3	ENSSSCG00000005178	-	intron	*CNTLN*
1	229343167	229343167	-	229343167	3	3	ENSSSCG00000005178	-	intron	*CNTLN*
1	229639367	229639367	-	229639367	3	3	---	---	intergenic	---
1	229911780	229911780	+	229911780	3	3	ENSSSCG00000025613	+	intron	---
1	230040132	230040132	+	230040132	3	3	ENSSSCG00000025613	+	intron	---
1	230106397	230106397	+	230106397	7	7	ENSSSCG00000025613	+	3'UTR	---
1	230135960	230135960	+	230135960	4	4	---	---	intergenic	---
1	230183961	230183965	-	230183965	5	8	---	---	intergenic	---
1	230289187	230289187	-	230289187	4	4	XLOC_003150	-	predicted	---
1	230443790	230443790	-	230443790	9	9	---	---	intergenic	---
1	230579121	230579163	-	230579122	49	176	ENSSSCG00000005182	-	3'UTR	---
1	230608938	230608938	-	230608938	3	3	ENSSSCG00000005182	-	intron	---
1	230651579	230651579	-	230651579	3	3	ENSSSCG00000005182	-	intron	---
1	230676939	230676939	-	230676939	6	6	ENSSSCG00000005182	-	ORF	---
1	230972564	230972564	-	230972564	3	3	---	---	intergenic	---
1	231010026	231010031	+	231010031	28	37	ENSSSCG00000005183	+	intron	*PSIP1*
1	231014222	231014249	+	231014240	12	27	ENSSSCG00000005183	+	ORF	*PSIP1*
1	231015431	231015431	+	231015431	3	3	ENSSSCG00000005183	+	intron	*PSIP1*
1	231024645	231024655	+	231024648	63	103	XLOC_001280	+	predicted	---

1	231038389	231038392	-	231038389	8	13	ENSSSCG00000005184	-	intron	*SNAPC3*
1	231278192	231278192	+	231278192	3	3	ENSSSCG00000005186	+	3'UTR	*TTC39B*
1	231384321	231384321	+	231384321	3	3	---	---	intergenic	---
1	232197791	232197793	+	232197793	11	21	XLOC_001286	+	predicted	---
1	232691574	232691574	-	232691574	3	3	---	---	intergenic	---
1	232959365	232959369	-	232959365	6	11	---	---	intergenic	---
1	233215766	233215766	+	233215766	3	3	XLOC_001288	+	predicted	---
1	233364407	233364407	+	233364407	4	4	---	---	intergenic	---
1	233481333	233481335	+	233481335	14	19	---	---	intergenic	---
1	233629061	233629061	-	233629061	9	9	---	---	intergenic	---
1	234004385	234004385	+	234004385	12	12	---	---	intergenic	---
1	235990780	235990780	+	235990780	4	4	---	---	intergenic	---
1	236047754	236047756	-	236047754	5	9	---	---	intergenic	---
1	236047780	236047780	+	236047780	3	3	---	---	intergenic	---
1	236273623	236273624	+	236273623	15	19	---	---	intergenic	---
1	236927399	236927423	-	236927415	101	469	---	---	intergenic	---
1	236927473	236927473	+	236927473	6	6	---	---	intergenic	---
1	237924484	237924484	+	237924484	6	6	---	---	intergenic	---
1	237933130	237933130	-	237933130	3	3	---	---	intergenic	---
1	238155011	238155019	-	238155011	5	8	---	---	intergenic	---
1	239451656	239451656	-	239451656	5	5	---	---	intergenic	---
1	239872623	239872623	-	239872623	3	3	---	---	intergenic	---
1	240163062	240163062	-	240163062	4	4	---	---	intergenic	---
1	240664877	240664877	-	240664877	6	6	---	---	intergenic	---
1	241088650	241088650	+	241088650	3	3	---	---	intergenic	---
1	241103513	241103514	+	241103513	3	6	---	---	intergenic	---

1	241784754	241784754	+	241784754	3	3	ENSSSCG00000005204	+	ORF	*RANBP6*
1	241896943	241896943	+	241896943	4	4	ENSSSCG00000005205	+	intron	*KIAA2026*
1	241952374	241952377	+	241952376	9	15	ENSSSCG00000005205	+	3'UTR	*KIAA2026*
							ENSSSCG00000005205	+	ORF	---
1	242109062	242109089	+	242109062	5	12	XLOC_001316	+	predicted	---
1	242115142	242115142	-	242115142	3	3	ENSSSCG00000005208	-	3'UTR	*RIC1*
1	242280827	242280827	+	242280827	8	8	---	---	intergenic	---
1	242282065	242282065	-	242282065	10	10	---	---	intergenic	---
1	242387492	242387492	+	242387492	10	10	---	---	intergenic	---
1	242473398	242473410	+	242473407	12	47	XLOC_001318	+	predicted	---
1	242561673	242561675	+	242561673	16	20	ENSSSCG00000005214	+	ORF	*INSL6*
1	242561769	242561769	+	242561769	3	3	ENSSSCG00000005214	+	ORF	*INSL6*
1	242561826	242561843	+	242561836	214	358	XLOC_001320	+	predicted	---
1	242561923	242561964	+	242561923	5	13	XLOC_001320	+	predicted	---
1	242978605	242978614	-	242978607	36	68	ENSSSCG00000005217	-	3'UTR	*RCL1*
1	242979091	242979091	-	242979091	3	3	ENSSSCG00000005217	-	3'UTR	*RCL1*
1	243093925	243093934	+	243093925	11	22	ENSSSCG00000023595	+	3'UTR	*AK3*
1	243095767	243095774	+	243095774	10	23	ENSSSCG00000023595	+	3'UTR	*AK3*
1	243098788	243098788	-	243098788	3	3	ENSSSCG00000005219	-	3'UTR	*CDC37L1*
1	243099824	243099825	-	243099824	3	6	ENSSSCG00000005219	-	3'UTR	*CDC37L1*
1	243099877	243099877	-	243099877	3	3	ENSSSCG00000005219	-	3'UTR	*CDC37L1*
1	243214833	243214846	+	243214845	65	190	XLOC_001331	+	predicted	---
1	243230806	243230806	-	243230806	3	3	ENSSSCG00000005222	-	3'UTR	*SLC1A1*
1	243507274	243507274	+	243507274	3	3	---	---	intergenic	---
1	244489969	244489969	+	244489969	3	3	ENSSSCG00000024190	+	3'UTR	---
1	244577843	244577843	-	244577843	3	3	---	---	intergenic	---

1	244826703	244826719	+	244826719	7	10	ENSSSCG00000005227	+	intron	---
1	244855986	244856026	+	244855987	38	125	ENSSSCG00000005227	+	intron	---
1	244857093	244857095	+	244857093	5	8	ENSSSCG00000005227	+	intron	---
1	244909847	244909847	-	244909847	9	9	---	---	intergenic	---
1	245001226	245001234	-	245001226	11	26	ENSSSCG00000005229	-	3'UTR	*VLDLR*
1	245196236	245196236	+	245196236	6	6	---	---	intergenic	---
1	245225467	245225467	-	245225467	3	3	XLOC_003211	-	predicted	---
1	245489521	245489524	-	245489524	12	16	ENSSSCG00000005232	-	3'UTR	*SMARCA2*
1	245489567	245489577	-	245489570	28	56	ENSSSCG00000005232	-	3'UTR	*SMARCA2*
1	245489850	245489850	-	245489850	3	3	ENSSSCG00000005232	-	3'UTR	*SMARCA2*
1	245505808	245505808	-	245505808	5	5	ENSSSCG00000005232	-	ORF	*SMARCA2*
1	245565519	245565519	-	245565519	4	4	ENSSSCG00000005232	-	ORF	*SMARCA2*
1	245574927	245574933	-	245574927	3	6	ENSSSCG00000005232	-	ORF	*SMARCA2*
1	245685369	245685383	-	245685376	9	39	---	---	intergenic	---
1	245685416	245685418	+	245685417	16	26	---	---	intergenic	---
1	246343480	246343480	+	246343480	3	3	XLOC_001341	+	predicted	---
1	246377133	246377133	-	246377133	6	6	---	---	intergenic	---
1	246798948	246798948	+	246798948	13	13	---	---	intergenic	---
1	247156906	247156929	+	247156922	6	24	ENSSSCG00000005234	+	3'UTR	---
1	247231015	247231015	+	247231015	3	3	---	---	intergenic	---
1	247468114	247468117	-	247468117	8	21	ENSSSCG00000005235	-	intron	---
1	247764237	247764237	+	247764237	7	7	---	---	intergenic	---
1	247828121	247828122	+	247828122	12	23	ENSSSCG00000005241	+	3'UTR	---
1	248144451	248144457	+	248144456	4	10	ENSSSCG00000005243	+	3'UTR	*PGM5*
1	248228511	248228511	-	248228511	7	7	---	---	intergenic	---
1	248244770	248244770	+	248244770	3	3	---	---	intergenic	---

1	248860479	248860479	+	248860479	5	5	ENSSSCG00000005247	+	intron	*TJP2*
1	248883723	248883728	+	248883723	7	18	ENSSSCG00000005247	+	3'UTR	*TJP2*
1	249031690	249031690	-	249031690	3	3	ENSSSCG00000005250	-	3'UTR	---
1	249356661	249356661	-	249356661	5	5	ENSSSCG00000005251	-	intron	*C9orf135*
1	249444285	249444285	-	249444285	4	4	---	---	intergenic	---
1	249499208	249499209	+	249499208	4	7	ENSSSCG00000005252	+	intron	*PTAR1*
1	249499269	249499269	+	249499269	3	3	ENSSSCG00000005252	+	intron	*PTAR1*
1	249909946	249909946	-	249909946	3	3	ENSSSCG00000026830	-	ORF	---
1	250029431	250029431	-	250029431	3	3	---	---	intergenic	---
1	250036957	250036958	+	250036957	7	11	ENSSSCG00000021646	+	intron	*KLF9*
1	250056425	250056429	+	250056425	6	12	ENSSSCG00000021646	+	3'UTR	*KLF9*
1	250057258	250057266	+	250057258	10	15	XLOC_001366	+	predicted	---
1	250125007	250125011	-	250125011	9	12	ENSSSCG00000005255	-	3'UTR	---
1	250127231	250127231	-	250127231	3	3	ENSSSCG00000005255	-	3'UTR	---
1	250300841	250300854	-	250300841	4	14	XLOC_003235	-	predicted	---
1	250852418	250852418	+	250852418	17	17	---	---	intergenic	---
1	251231421	251231428	-	251231421	6	11	---	---	intergenic	---
1	251369965	251369965	-	251369965	6	6	---	---	intergenic	---
1	251658917	251658917	+	251658917	17	17	---	---	intergenic	---
1	251668425	251668438	-	251668425	15	32	---	---	intergenic	---
1	251717265	251717267	-	251717267	7	11	XLOC_003242	-	predicted	---
1	251827098	251827098	+	251827098	5	5	XLOC_001376	+	predicted	---
1	251913640	251913645	-	251913645	83	103	XLOC_003245	-	predicted	---
1	251914244	251914250	-	251914248	55	133	XLOC_003245	-	predicted	---
1	251942916	251942916	+	251942916	3	3	---	---	intergenic	---
1	251961294	251961294	-	251961294	7	7	---	---	intergenic	---

1	252169981	252169982	+	252169981	3	6	---	---	intergenic	---
1	252229781	252229795	-	252229783	42	72	XLOC_003247	-	predicted	---
1	252231189	252231197	-	252231190	15	45	ENSSSCG00000005264	-	3'UTR	*ZFAND5*
1	252773960	252773960	-	252773960	4	4	---	---	intergenic	---
1	252808522	252808522	+	252808522	5	5	ENSSSCG00000028996	+	3'UTR	*ALDH1A1*
1	252998393	252998393	+	252998393	5	5	---	---	intergenic	---
1	253007315	253007315	+	253007315	4	4	ENSSSCG00000005267	+	3'UTR	*ANXA1*
1	253007484	253007486	+	253007485	205	227	ENSSSCG00000005267	+	3'UTR	*ANXA1*
1	253146968	253146970	+	253146968	3	6	---	---	intergenic	---
1	254167279	254167284	-	254167281	11	20	---	---	intergenic	---
1	254167317	254167323	+	254167317	9	27	---	---	intergenic	---
1	254523476	254523477	+	254523476	7	10	---	---	intergenic	---
1	254763128	254763128	-	254763128	3	3	ENSSSCG00000005269	-	intron	*TRPM6*
1	254854997	254854997	-	254854997	5	5	---	---	intergenic	---
1	254937433	254937438	-	254937438	7	14	ENSSSCG00000025640	-	3'UTR	*C9orf40*
1	254938776	254938776	-	254938776	5	5	ENSSSCG00000025640	-	3'UTR	*C9orf40*
1	254954156	254954163	-	254954163	9	17	ENSSSCG00000005271	-	3'UTR	*C9orf41*
1	255013001	255013008	-	255013002	54	132	XLOC_003254	-	predicted	---
1	255094985	255094990	+	255094985	10	19	ENSSSCG00000005273	+	3'UTR	*OSTF1*
1	255095055	255095055	+	255095055	3	3	XLOC_001385	+	predicted	---
1	255097774	255097774	+	255097774	3	3	XLOC_001385	+	predicted	---
1	255732437	255732438	+	255732437	5	8	ENSSSCG00000023520	+	intron	*PCSK5*
1	255834584	255834584	+	255834584	16	16	---	---	intergenic	---
1	256066053	256066053	+	256066053	4	4	XLOC_001388	+	predicted	---
1	256084220	256084220	-	256084220	3	3	XLOC_003256	-	predicted	---
1	256086602	256086605	-	256086602	3	6	ENSSSCG00000005276	-	3'UTR	*RFK*

1	256086645	256086648	-	256086648	26	61	ENSSSCG00000005276	-	3'UTR	*RFK*
1	256087996	256087996	-	256087996	3	3	ENSSSCG00000005276	-	ORF	*RFK*
1	256092961	256092961	+	256092961	3	3	---	---	intergenic	---
1	256224509	256224509	+	256224509	3	3	XLOC_001390	+	predicted	---
1	256265837	256265837	-	256265837	3	3	---	---	intergenic	---
1	256315012	256315033	-	256315012	3	9	XLOC_003259	-	predicted	---
1	256424939	256424939	-	256424939	5	5	---	---	intergenic	---
1	256472343	256472343	+	256472343	3	3	---	---	intergenic	---
1	256585972	256585973	-	256585972	11	14	XLOC_003261	-	predicted	---
1	256782299	256782338	-	256782331	5	12	---	---	intergenic	---
1	256806080	256806081	+	256806081	26	46	ENSSSCG00000005282	+	intron	*VPS13A*
1	256998229	256998229	+	256998229	6	6	ENSSSCG00000005282	+	intron	*VPS13A*
1	257093763	257093764	+	257093763	3	6	ENSSSCG00000005282	+	3'UTR	*VPS13A*
1	257094951	257094951	+	257094951	3	3	ENSSSCG00000005282	+	3'UTR	*VPS13A*
1	257105700	257105700	-	257105700	4	4	XLOC_003262	-	predicted	---
1	257241167	257241167	-	257241167	4	4	ENSSSCG00000005284	-	intron	*GNA14*
1	257367754	257367756	-	257367754	7	13	XLOC_003263	-	predicted	---
1	257368813	257368813	-	257368813	5	5	XLOC_003263	-	predicted	---
1	257780244	257780252	+	257780252	7	12	ENSSSCG00000005286	+	3'UTR	*CEP78*
1	257785229	257785230	-	257785230	4	7	---	---	intergenic	---
1	257835722	257835735	+	257835732	134	234	XLOC_001399	+	predicted	---
1	258221639	258221639	-	258221639	5	5	---	---	intergenic	---
1	259260783	259260783	+	259260783	3	3	ENSSSCG00000005288	+	intron	*TLE4*
1	259286502	259286502	+	259286502	3	3	ENSSSCG00000005288	+	intron	*TLE4*
1	259398091	259398098	+	259398096	11	30	ENSSSCG00000005288	+	3'UTR	*TLE4*
1	259399352	259399352	+	259399352	3	3	ENSSSCG00000005288	+	3'UTR	*TLE4*

1	259403119	259403119	+	259403119	3	3	XLOC_001402	+	predicted	---
1	259950280	259950280	+	259950280	5	5	---	---	intergenic	---
1	260095455	260095455	-	260095455	3	3	XLOC_003269	-	predicted	---
1	260493218	260493219	+	260493218	9	13	---	---	intergenic	---
1	260575699	260575699	-	260575699	3	3	---	---	intergenic	---
1	261712047	261712047	+	261712047	3	3	---	---	intergenic	---
1	261797392	261797395	-	261797395	21	38	ENSSSCG00000005291	-	3'UTR	---
1	261843668	261843675	-	261843668	15	22	ENSSSCG00000005291	-	intron	---
1	262084306	262084306	+	262084306	3	3	ENSSSCG00000005292	+	ORF	---
1	262087425	262087425	+	262087425	4	4	XLOC_001406	+	predicted	---
1	262087480	262087486	+	262087480	13	31	XLOC_001406	+	predicted	---
1	263171697	263171704	+	263171698	27	42	XLOC_001411	+	predicted	---
1	263172721	263172721	+	263172721	33	33	---	---	intergenic	---
1	263206274	263206298	-	263206279	26	41	XLOC_003275	-	predicted	---
1	263371498	263371498	+	263371498	3	3	ENSSSCG00000005298	+	ORF	*C9orf131*
1	263374460	263374462	+	263374461	15	29	XLOC_001414	+	predicted	---
1	263379444	263379449	-	263379444	10	16	XLOC_003278	-	predicted	---
1	263379917	263379933	-	263379932	105	291	ENSSSCG00000005300	-	3'UTR	---
1	263380268	263380275	-	263380269	19	45	ENSSSCG00000005300	-	3'UTR	---
1	263384612	263384612	-	263384612	3	3	ENSSSCG00000005300	-	ORF	---
1	263405477	263405478	-	263405478	20	23	XLOC_003278	-	predicted	---
1	263420994	263420994	-	263420994	7	7	ENSSSCG00000005301	-	3'UTR	*FAM214B*
1	263421958	263421965	-	263421959	4	11	ENSSSCG00000005301	-	3'UTR	*FAM214B*
1	263451791	263451791	+	263451791	4	4	---	---	intergenic	---
1	263877075	263877078	+	263877075	3	6	ENSSSCG00000005305	+	3'UTR	*UNC13B*
1	263980857	263980861	+	263980861	11	14	XLOC_001419	+	predicted	---

1	264047988	264047989	+	264047988	36	42	ENSSSCG00000005308	+	3'UTR	*RUSC2*
1	264070983	264070983	+	264070983	4	4	ENSSSCG00000005310	+	3'UTR	*TESK1*
1	264096750	264096751	-	264096750	13	16	ENSSSCG00000005312	-	3'UTR	*SIT1*
1	264105117	264105118	-	264105117	7	12	ENSSSCG00000018700	-	ORF	*RNase_MRP*
1	264108699	264108705	+	264108700	15	33	ENSSSCG00000005313	+	ORF	*CCDC107*
1	264108957	264108960	-	264108958	27	51	XLOC_003289	-	predicted	---
1	264126008	264126027	+	264126020	316	480	ENSSSCG00000005315	+	3'UTR	*CA9*
1	264127283	264127288	-	264127285	68	97	ENSSSCG00000005316	-	3'UTR	*TPM2*
1	264128175	264128176	-	264128175	90	94	ENSSSCG00000005316	-	intron	*TPM2*
1	264129461	264129463	-	264129462	11	18	ENSSSCG00000005316	-	intron	*TPM2*
1	264129923	264129944	-	264129932	22	49	ENSSSCG00000005316	-	intron	*TPM2*
1	264141075	264141080	-	264141076	75	150	ENSSSCG00000005317	-	3'UTR	*TLN1*
1	264164182	264164185	-	264164182	220	227	ENSSSCG00000005317	-	ORF	*TLN1*
1	264166034	264166038	-	264166038	8	11	ENSSSCG00000005317	-	ORF	*TLN1*
1	264168294	264168294	-	264168294	3	3	ENSSSCG00000005317	-	ORF	*TLN1*
1	264180431	264180431	-	264180431	6	6	XLOC_003292	-	predicted	---
1	264181427	264181433	-	264181427	15	18	XLOC_003292	-	predicted	---
1	264181559	264181571	+	264181560	41	76	ENSSSCG00000005318	+	3'UTR	*CREB3*
1	264197778	264197792	+	264197779	14	23	ENSSSCG00000005320	+	3'UTR	*RGP1*
1	264254276	264254282	-	264254282	33	57	XLOC_003294	-	predicted	---
1	264254333	264254333	-	264254333	14	14	XLOC_003294	-	predicted	---
1	264257331	264257333	-	264257332	94	116	ENSSSCG00000005323	-	3'UTR	*HINT2*
1	264264273	264264278	-	264264275	57	206	XLOC_003294	-	predicted	---
1	264308658	264308658	+	264308658	7	7	ENSSSCG00000005325	+	3'UTR	*TMEM8B*
1	264387939	264387939	+	264387939	4	4	---	---	intergenic	---
1	264417647	264417647	+	264417647	3	3	---	---	intergenic	---

1	264506702	264506702	-	264506702	3	3	XLOC_003299	-	predicted	---
1	264525366	264525368	-	264525368	10	18	---	---	intergenic	---
1	264620418	264620418	-	264620418	3	3	ENSSSCG00000005340	-	ORF	*CCIN*
1	264627082	264627082	-	264627082	11	11	ENSSSCG00000005339	-	3'UTR	---
1	264627984	264627984	-	264627984	9	9	ENSSSCG00000005339	-	3'UTR	---
1	264628166	264628166	-	264628166	4	4	ENSSSCG00000005339	-	3'UTR	---
1	264874670	264874671	-	264874670	16	24	ENSSSCG00000005343	-	3'UTR	*RNF38*
1	264877847	264877861	-	264877850	55	108	ENSSSCG00000005343	-	3'UTR	*RNF38*
1	265094097	265094099	+	265094097	9	19	---	---	intergenic	---
1	265414930	265414930	-	265414930	4	4	---	---	intergenic	---
1	265414952	265414954	+	265414953	30	54	---	---	intergenic	---
1	265581322	265581323	-	265581322	12	15	ENSSSCG00000005345	-	intron	*PAX5*
1	265634903	265634903	+	265634903	8	8	XLOC_001440	+	predicted	---
1	265810053	265810053	-	265810053	7	7	---	---	intergenic	---
1	266101724	266101734	+	266101732	37	134	ENSSSCG00000005349	+	3'UTR	*GRHPR*
1	266125145	266125152	+	266125151	12	26	ENSSSCG00000005347	+	3'UTR	*POLR1E*
1	266262218	266262240	-	266262239	54	196	ENSSSCG00000005350	-	intron	---
1	266262370	266262370	-	266262370	8	8	ENSSSCG00000005350	-	intron	---
1	266262435	266262435	-	266262435	4	4	ENSSSCG00000005350	-	intron	---
1	266469786	266469786	+	266469786	3	3	ENSSSCG00000005354	+	3'UTR	*TRMT10B*
1	266471326	266471326	-	266471326	4	4	ENSSSCG00000005355	-	3'UTR	*EXOSC3*
1	266471420	266471427	-	266471421	24	45	ENSSSCG00000005355	-	3'UTR	*EXOSC3*
1	266471479	266471488	-	266471487	34	82	ENSSSCG00000005355	-	3'UTR	*EXOSC3*
1	266476691	266476692	+	266476692	10	13	---	---	intergenic	---
1	266486428	266486429	+	266486428	4	7	---	---	intergenic	---
1	266498244	266498244	+	266498244	4	4	XLOC_001448	+	predicted	---

1	266612737	266612737	+	266612737	3	3	XLOC_001449	+	predicted	---
1	266617371	266617377	+	266617371	3	6	XLOC_001449	+	predicted	---
1	266621910	266621910	+	266621910	3	3	XLOC_001450	+	predicted	---
1	266622356	266622356	-	266622356	14	14	XLOC_003319	-	predicted	---
1	266623540	266623540	-	266623540	5	5	XLOC_003319	-	predicted	---
1	266670708	266670708	+	266670708	5	5	XLOC_001451	+	predicted	---
1	266673751	266673757	+	266673751	3	6	XLOC_001451	+	predicted	---
1	266816095	266816095	+	266816095	3	3	---	---	intergenic	---
1	267168779	267168786	+	267168780	9	19	XLOC_001456	+	predicted	---
1	267258152	267258152	+	267258152	3	3	ENSSSCG00000025655	+	intron	*CCDC180*
1	267259243	267259244	+	267259244	6	11	ENSSSCG00000025655	+	ORF	*CCDC180*
1	267291523	267291523	+	267291523	3	3	ENSSSCG00000025655	+	ORF	*CCDC180*
1	267327354	267327362	+	267327355	9	21	---	---	intergenic	---
1	267335136	267335136	+	267335136	11	11	---	---	intergenic	---
1	267419705	267419705	+	267419705	12	12	ENSSSCG00000005364	+	intron	*TDRD7*
1	267421883	267421883	+	267421883	3	3	ENSSSCG00000005364	+	intron	*TDRD7*
1	267425062	267425075	+	267425067	28	46	ENSSSCG00000005364	+	3'UTR	*TDRD7*
1	267547209	267547210	-	267547209	26	29	ENSSSCG00000023010	-	3'UTR	*TMOD1*
1	267548374	267548382	+	267548374	8	20	ENSSSCG00000005367	+	3'UTR	*TSTD2*
1	267583615	267583615	+	267583615	5	5	ENSSSCG00000005366	+	intron	---
1	267651553	267651568	+	267651563	26	92	ENSSSCG00000005366	+	intron	---
1	267658969	267658971	+	267658969	24	38	XLOC_001462	+	predicted	---
1	267660229	267660229	+	267660229	3	3	XLOC_001462	+	predicted	---
1	267660927	267660927	-	267660927	5	5	ENSSSCG00000005368	-	3'UTR	*XPA*
1	267661918	267661919	-	267661918	4	8	ENSSSCG00000005368	-	3'UTR	*XPA*
1	267908446	267908446	+	267908446	6	6	ENSSSCG00000005371	+	ORF	*HEMGN*

1	267908923	267908948	+	267908948	4	7	ENSSSCG00000005371	+	ORF	*HEMGN*
1	267909032	267909032	+	267909032	5	5	ENSSSCG00000005371	+	ORF	*HEMGN*
1	267909235	267909235	+	267909235	3	3	ENSSSCG00000005371	+	ORF	*HEMGN*
1	267912370	267912396	+	267912379	85	203	ENSSSCG00000005371	+	3'UTR	*HEMGN*
1	267913313	267913313	+	267913313	3	3	ENSSSCG00000005371	+	3'UTR	*HEMGN*
1	267971764	267971773	-	267971766	15	33	XLOC_003328	-	predicted	---
1	267998351	267998363	+	267998356	24	67	XLOC_001465	+	predicted	---
1	268034374	268034374	+	268034374	3	3	ENSSSCG00000005372	+	intron	*ANP32B*
1	268036107	268036119	+	268036119	15	46	ENSSSCG00000005372	+	intron	*ANP32B*
1	268037790	268037793	+	268037792	5	12	XLOC_001465	+	predicted	---
1	268037943	268037943	+	268037943	3	3	XLOC_001465	+	predicted	---
1	268082283	268082284	+	268082283	20	34	ENSSSCG00000005373	+	3'UTR	*NANS*
1	268085338	268085338	-	268085338	3	3	XLOC_003329	-	predicted	---
1	268104398	268104398	-	268104398	9	9	ENSSSCG00000005374	-	intron	*TRIM14*
1	268119337	268119337	-	268119337	4	4	ENSSSCG00000005375	-	3'UTR	*CORO2A*
1	268119612	268119621	-	268119615	9	29	ENSSSCG00000005375	-	3'UTR	*CORO2A*
1	268174458	268174458	-	268174458	4	4	XLOC_003332	-	predicted	---
1	268175302	268175302	-	268175302	5	5	XLOC_003332	-	predicted	---
1	268881946	268881953	+	268881953	4	7	XLOC_001470	+	predicted	---
1	268992978	268992978	-	268992978	3	3	---	---	intergenic	---
1	269022429	269022429	+	269022429	6	6	ENSSSCG00000005381	+	3'UTR	---
1	269022833	269022833	+	269022833	9	9	ENSSSCG00000005381	+	3'UTR	---
1	269023118	269023121	+	269023118	4	7	ENSSSCG00000005381	+	3'UTR	---
1	269023223	269023230	+	269023226	6	21	ENSSSCG00000005381	+	3'UTR	---
1	269149042	269149043	+	269149043	9	16	ENSSSCG00000005382	+	3'UTR	*TGFBR1*
							ENSSSCG00000005382	+	ORF	---

1	269222549	269222550	+	269222550	12	15	ENSSSCG00000005384	+	3'UTR	*SEC61B*
1	269222673	269222683	+	269222678	164	300	ENSSSCG00000005384	+	3'UTR	*SEC61B*
1	269222754	269222756	+	269222756	95	107	XLOC_001475	+	predicted	---
1	269640987	269640987	-	269640987	3	3	---	---	intergenic	---
1	270002147	270002147	+	270002147	3	3	ENSSSCG00000005386	+	3'UTR	*STX17*
1	270006474	270006474	+	270006474	4	4	XLOC_001480	+	predicted	---
1	270074289	270074289	-	270074289	7	7	ENSSSCG00000005387	-	3'UTR	*ERP44*
1	270077522	270077522	-	270077522	8	8	ENSSSCG00000005387	-	3'UTR	*ERP44*
1	270077764	270077767	-	270077764	17	27	ENSSSCG00000005387	-	3'UTR	*ERP44*
1	270077853	270077921	-	270077869	19	72	ENSSSCG00000005387	-	3'UTR	*ERP44*
1	270106492	270106492	-	270106492	3	3	ENSSSCG00000005387	-	ORF	*ERP44*
1	270111828	270111828	-	270111828	6	6	ENSSSCG00000005387	-	ORF	*ERP44*
1	270214620	270214620	+	270214620	3	3	ENSSSCG00000005388	+	intron	*INVS*
1	270407977	270407977	+	270407977	4	4	ENSSSCG00000005388	+	3'UTR	*INVS*
1	270480369	270480369	-	270480369	3	3	ENSSSCG00000005389	-	intron	*TEX10*
1	270508748	270508751	-	270508748	4	7	---	---	intergenic	---
1	270549245	270549245	+	270549245	11	11	ENSSSCG00000005390	+	intron	*MSANTD3*
1	270571198	270571198	-	270571198	3	3	XLOC_003345	-	predicted	---
1	270757468	270757468	-	270757468	13	13	---	---	intergenic	---
1	271003377	271003396	-	271003377	40	104	---	---	intergenic	---
1	271003566	271003566	-	271003566	3	3	---	---	intergenic	---
1	271534732	271534732	-	271534732	3	3	XLOC_003349	-	predicted	---
1	271535061	271535069	-	271535062	12	24	XLOC_003349	-	predicted	---
1	271535238	271535238	-	271535238	3	3	XLOC_003349	-	predicted	---
1	271536143	271536147	-	271536147	14	49	XLOC_003349	-	predicted	---
1	271587035	271587040	-	271587040	10	13	ENSSSCG00000005398	-	3'UTR	*BAAT*

1	271587136	271587164	-	271587161	6	24	ENSSSCG00000005398	-	ORF	*BAAT*
1	271589142	271589142	-	271589142	3	3	ENSSSCG00000005398	-	intron	*BAAT*
1	271654135	271654135	-	271654135	3	3	ENSSSCG00000027974	-	3'UTR	*MRPL50*
1	271655690	271655690	-	271655690	4	4	ENSSSCG00000027974	-	3'UTR	*MRPL50*
1	271656127	271656129	-	271656129	20	41	ENSSSCG00000027974	-	3'UTR	*MRPL50*
1	271710335	271710349	+	271710349	9	13	ENSSSCG00000005395	+	ORF	---
1	271718967	271718973	+	271718967	4	7	XLOC_001491	+	predicted	---
1	271725715	271725715	-	271725715	8	8	XLOC_003352	-	predicted	---
1	271725866	271725866	-	271725866	3	3	XLOC_003352	-	predicted	---
1	271725916	271725921	-	271725916	3	9	XLOC_003352	-	predicted	---
1	271727110	271727110	-	271727110	3	3	ENSSSCG00000005394	-	3'UTR	*ALDOB*
1	271727537	271727538	-	271727538	6	10	ENSSSCG00000005394	-	3'UTR	*ALDOB*
1	271727645	271727653	-	271727645	20	45	ENSSSCG00000005394	-	3'UTR	*ALDOB*
1	271728480	271728480	-	271728480	5	5	ENSSSCG00000005394	-	3'UTR	*ALDOB*
1	271728809	271728817	-	271728817	8	32	ENSSSCG00000005394	-	3'UTR	*ALDOB*
1	271728924	271728944	-	271728934	606	1393	ENSSSCG00000005394	-	3'UTR	*ALDOB*
1	271728986	271729002	-	271728986	54	81	ENSSSCG00000005394	-	3'UTR	*ALDOB*
1	271729086	271729086	-	271729086	3	3	ENSSSCG00000005394	-	3'UTR	*ALDOB*
1	271729220	271729236	-	271729234	901	1963	ENSSSCG00000005394	-	3'UTR	*ALDOB*
1	271729388	271729401	-	271729388	57	95	ENSSSCG00000005394	-	3'UTR	*ALDOB*
1	271729462	271729482	-	271729481	13	37	ENSSSCG00000005394	-	3'UTR	*ALDOB*
1	271729523	271729560	-	271729534	25	72	ENSSSCG00000005394	-	3'UTR	*ALDOB*
1	271732126	271732136	-	271732136	10	14	ENSSSCG00000005394	-	ORF	*ALDOB*
1	271732708	271732708	-	271732708	9	9	ENSSSCG00000005394	-	ORF	*ALDOB*
1	271734424	271734427	-	271734424	13	26	ENSSSCG00000005394	-	ORF	*ALDOB*
1	271826284	271826284	-	271826284	3	3	---	---	intergenic	---

1	271868248	271868248	+	271868248	5	5	ENSSSCG00000005399	+	3'UTR	---
1	271868916	271868918	+	271868918	7	18	XLOC_001494	+	predicted	---
1	271898609	271898609	-	271898609	3	3	ENSSSCG00000005400	-	intron	*GRIN3A*
1	271898758	271898766	-	271898758	20	51	ENSSSCG00000005400	-	intron	*GRIN3A*
1	271898850	271898863	-	271898856	150	392	ENSSSCG00000005400	-	intron	*GRIN3A*
1	272220014	272220016	+	272220014	6	9	---	---	intergenic	---
1	272752339	272752339	+	272752339	4	4	---	---	intergenic	---
1	272847581	272847583	-	272847581	12	15	---	---	intergenic	---
1	272909027	272909027	-	272909027	40	40	---	---	intergenic	---
1	273275259	273275259	+	273275259	4	4	ENSSSCG00000005401	+	intron	*CYLC2*
1	273275821	273275821	+	273275821	5	5	ENSSSCG00000005401	+	ORF	*CYLC2*
1	273326030	273326030	+	273326030	3	3	---	---	intergenic	---
1	273371451	273371456	+	273371456	6	14	XLOC_001497	+	predicted	---
1	274154077	274154077	+	274154077	4	4	---	---	intergenic	---
1	274632082	274632082	+	274632082	11	11	ENSSSCG00000005403	+	3'UTR	---
1	274997778	274997778	-	274997778	5	5	---	---	intergenic	---
1	275329942	275329942	+	275329942	3	3	---	---	intergenic	---
1	275507037	275507037	+	275507037	3	3	ENSSSCG00000026173	+	3'UTR	*ABCA1*
1	275507906	275507907	+	275507906	12	16	ENSSSCG00000026173	+	3'UTR	*ABCA1*
1	275534268	275534268	-	275534268	8	8	XLOC_003364	-	predicted	---
1	276204007	276204007	+	276204007	4	4	ENSSSCG00000005425	+	intron	*SLC44A1*
1	276352932	276352932	+	276352932	3	3	XLOC_001516	+	predicted	---
1	276412277	276412277	+	276412277	5	5	---	---	intergenic	---
1	276515462	276515463	+	276515463	6	11	ENSSSCG00000005426	+	intron	*FSD1L*
1	276540865	276540868	+	276540868	5	8	XLOC_001516	+	predicted	---
1	276569092	276569093	-	276569092	3	6	ENSSSCG00000005428	-	intron	*HNRNPA1L2*

1	276711006	276711006	+	276711006	3	3	ENSSSCG00000005427	+	3'UTR	*FKTN*
1	276748813	276748813	-	276748813	4	4	XLOC_003374	-	predicted	---
1	276777186	276777186	+	276777186	4	4	ENSSSCG00000005429	+	intron	*TMEM38B*
1	276820047	276820047	+	276820047	5	5	XLOC_001519	+	predicted	---
1	276821941	276821941	+	276821941	3	3	XLOC_001519	+	predicted	---
1	276825961	276825961	+	276825961	4	4	---	---	intergenic	---
1	277801043	277801057	-	277801057	13	21	XLOC_003378	-	predicted	---
1	277809091	277809091	+	277809091	4	4	---	---	intergenic	---
1	278302105	278302105	+	278302105	10	10	ENSSSCG00000005432	+	ORF	*protein_coding*
1	278302305	278302306	+	278302305	6	10	ENSSSCG00000005432	+	3'UTR	---
							ENSSSCG00000005432	+	ORF	*protein_coding*
1	278547302	278547302	-	278547302	5	5	---	---	intergenic	---
1	278759651	278759666	-	278759651	3	6	XLOC_003381	-	predicted	---
1	278818137	278818137	+	278818137	4	4	---	---	intergenic	---
1	278945829	278945834	+	278945829	4	14	ENSSSCG00000005436	+	3'UTR	*RAD23B*
1	278946571	278946578	+	278946571	9	12	ENSSSCG00000005436	+	3'UTR	*RAD23B*
1	278947016	278947023	+	278947022	26	33	ENSSSCG00000005436	+	3'UTR	*RAD23B*
1	279056803	279056803	-	279056803	3	3	ENSSSCG00000005437	-	3'UTR	*KLF4*
1	279056928	279056928	-	279056928	5	5	ENSSSCG00000005437	-	3'UTR	*KLF4*
1	279057309	279057309	-	279057309	3	3	ENSSSCG00000005437	-	3'UTR	*KLF4*
1	279057500	279057500	-	279057500	4	4	ENSSSCG00000005437	-	3'UTR	*KLF4*
1	279096769	279096769	-	279096769	3	3	---	---	intergenic	---
1	279190891	279190894	+	279190894	6	14	---	---	intergenic	---
1	279236246	279236246	+	279236246	3	3	---	---	intergenic	---

1	279257626	279257626	-	279257626	9	9	XLOC_003385	-	predicted	---
1	279281456	279281457	+	279281457	6	9	---	---	intergenic	---
1	279497701	279497701	+	279497701	5	5	---	---	intergenic	---
1	279990906	279990906	+	279990906	4	4	---	---	intergenic	---
1	280022477	280022477	+	280022477	3	3	---	---	intergenic	---
1	280384461	280384471	+	280384464	16	36	XLOC_001534	+	predicted	---
1	280388608	280388609	+	280388608	3	6	---	---	intergenic	---
1	280407266	280407294	-	280407275	106	335	XLOC_003390	-	predicted	---
1	280408632	280408632	-	280408632	4	4	---	---	intergenic	---
1	280408673	280408673	+	280408673	9	9	---	---	intergenic	---
1	280415197	280415203	+	280415199	259	456	XLOC_001535	+	predicted	---
1	280429160	280429160	-	280429160	3	3	XLOC_003391	-	predicted	---
1	280429288	280429288	-	280429288	8	8	ENSSSCG00000005440	-	3'UTR	---
1	280510919	280510919	+	280510919	4	4	ENSSSCG00000005441	+	3'UTR	*IKBKAP*
1	280531326	280531326	+	280531326	3	3	ENSSSCG00000005442	+	ORF	---
1	280535895	280535898	-	280535897	10	23	ENSSSCG00000005443	-	3'UTR	*CTNNAL1*
1	280570779	280570779	-	280570779	3	3	ENSSSCG00000005443	-	intron	*CTNNAL1*
							ENSSSCG00000005443	-	ORF	*CTNNAL1*
1	280610779	280610780	-	280610780	6	11	XLOC_003395	-	predicted	---
1	280611140	280611140	+	280611140	4	4	---	---	intergenic	---
1	280616170	280616170	-	280616170	3	3	XLOC_003395	-	predicted	---
1	280795050	280795050	-	280795050	3	3	ENSSSCG00000005446	-	intron	*EPB41L4B*
1	280816661	280816661	-	280816661	3	3	ENSSSCG00000005446	-	intron	*EPB41L4B*
1	280817102	280817102	-	280817102	3	3	ENSSSCG00000005446	-	intron	*EPB41L4B*
1	281952952	281952955	+	281952955	9	12	XLOC_001540	+	predicted	---
1	282026924	282026932	-	282026927	264	422	XLOC_003404	-	predicted	---

1	282026979	282026990	-	282026989	19	50	XLOC_003404	-	predicted	---
1	282049739	282049740	-	282049739	3	6	ENSSSCG00000005453	-	intron	*TRX1*
1	282054126	282054135	-	282054127	47	172	ENSSSCG00000005453	-	intron	*TRX1*
1	282054173	282054209	-	282054187	233	703	ENSSSCG00000005453	-	intron	*TRX1*
1	282054298	282054302	-	282054299	31	67	ENSSSCG00000005453	-	intron	*TRX1*
1	282068785	282068785	-	282068785	3	3	ENSSSCG00000005453	-	intron	*TRX1*
							ENSSSCG00000005454	-	ORF	*TXNDC8*
1	282076236	282076236	-	282076236	3	3	ENSSSCG00000005453	-	intron	*TRX1*
1	282076620	282076620	+	282076620	3	3	---	---	intergenic	---
1	282087031	282087032	+	282087032	8	11	---	---	intergenic	---
1	282116152	282116152	-	282116152	3	3	XLOC_003406	-	predicted	---
1	282227261	282227261	-	282227261	6	6	ENSSSCG00000005455	-	intron	*SVEP1*
1	282381804	282381804	+	282381804	3	3	---	---	intergenic	---
1	282651600	282651600	-	282651600	8	8	---	---	intergenic	---
1	282728153	282728153	+	282728153	6	6	---	---	intergenic	---
1	282783252	282783252	-	282783252	46	46	---	---	intergenic	---
1	282867626	282867626	-	282867626	3	3	XLOC_003409	-	predicted	---
1	282867674	282867674	-	282867674	6	6	XLOC_003409	-	predicted	---
1	282963404	282963404	+	282963404	4	4	---	---	intergenic	---
1	283043128	283043128	-	283043128	3	3	---	---	intergenic	---
1	283052856	283052858	-	283052856	7	17	ENSSSCG00000005459	-	3'UTR	*KIAA0368*
1	283064481	283064481	-	283064481	3	3	ENSSSCG00000005459	-	ORF	*KIAA0368*
1	283076871	283076871	-	283076871	5	5	ENSSSCG00000005459	-	intron	*KIAA0368*
1	283206749	283206749	-	283206749	8	8	XLOC_003413	-	predicted	---
1	283250933	283250933	+	283250933	3	3	---	---	intergenic	---
1	283251663	283251665	+	283251665	9	13	---	---	intergenic	---

1	283265802	283265810	+	283265802	14	24	ENSSSCG00000005462	+	3'UTR	*GNG10*
1	283266448	283266475	+	283266472	38	140	ENSSSCG00000005462	+	3'UTR	*GNG10*
1	283291496	283291496	-	283291496	27	27	---	---	intergenic	---
1	283479531	283479531	+	283479531	6	6	ENSSSCG00000022780	+	3'UTR	*UGCG*
1	283526583	283526586	+	283526585	5	12	---	---	intergenic	---
1	283560386	283560386	-	283560386	3	3	---	---	intergenic	---
1	283772671	283772671	-	283772671	5	5	XLOC_003419	-	predicted	---
1	283853713	283853713	+	283853713	3	3	---	---	intergenic	---
1	283932071	283932071	+	283932071	10	10	ENSSSCG00000005467	+	intron	*HSDL2*
1	283945839	283945839	+	283945839	7	7	ENSSSCG00000005467	+	intron	*HSDL2*
1	283975763	283975775	+	283975774	20	42	ENSSSCG00000005467	+	3'UTR	*HSDL2*
1	283975894	283975902	+	283975896	9	19	ENSSSCG00000005467	+	3'UTR	*HSDL2*
1	283976612	283976615	+	283976614	21	43	ENSSSCG00000005467	+	3'UTR	*HSDL2*
							ENSSSCG00000005467	+	ORF	---
1	284184381	284184384	-	284184381	5	10	ENSSSCG00000026194	-	3'UTR	*INIP*
1	284359478	284359478	+	284359478	6	6	XLOC_001554	+	predicted	---
1	284364774	284364774	+	284364774	4	4	XLOC_001554	+	predicted	---
1	284368653	284368653	-	284368653	5	5	ENSSSCG00000005472	-	3'UTR	*SLC46A2*
1	284399915	284399915	+	284399915	3	3	---	---	intergenic	---
1	284443977	284443977	+	284443977	3	3	---	---	intergenic	---
1	284447109	284447112	-	284447111	15	38	ENSSSCG00000005474	-	3'UTR	---
1	284599670	284599670	+	284599670	3	3	ENSSSCG00000023714	+	3'UTR	*SLC31A2*
1	284602179	284602185	-	284602185	12	23	ENSSSCG00000005477	-	3'UTR	---
1	284648923	284648934	-	284648928	9	27	ENSSSCG00000024123	-	3'UTR	*CDC26*
1	284648973	284648981	-	284648975	109	248	ENSSSCG00000024123	-	3'UTR	*CDC26*
1	284655390	284655391	-	284655390	9	13	ENSSSCG00000024123	-	intron	*CDC26*

1	284676653	284676653	+	284676653	3	3	ENSSSCG00000024648	+	3'UTR	*PRPF4*
1	284677012	284677012	+	284677012	3	3	ENSSSCG00000024648	+	3'UTR	*PRPF4*
1	284677108	284677108	+	284677108	3	3	ENSSSCG00000024648	+	3'UTR	*PRPF4*
1	284697708	284697708	-	284697708	3	3	XLOC_003431	-	predicted	---
1	284700374	284700378	-	284700375	17	49	XLOC_003431	-	predicted	---
1	284828766	284828768	-	284828766	22	32	ENSSSCG00000005478	-	3'UTR	*POLE3*
1	284852411	284852414	+	284852411	20	33	XLOC_001564	+	predicted	---
1	284953028	284953032	+	284953028	4	7	ENSSSCG00000005480	+	intron	---
1	284993338	284993338	-	284993338	3	3	ENSSSCG00000005481	-	3'UTR	*RGS3*
1	285504575	285504575	-	285504575	3	3	---	---	intergenic	---
1	285661146	285661146	+	285661146	4	4	ENSSSCG00000005484	+	intron	*ZNF618*
1	285705501	285705501	-	285705501	3	3	XLOC_003439	-	predicted	---
1	285709023	285709023	-	285709023	4	4	ENSSSCG00000005485	-	intron	*AMBP*
1	285710959	285710975	-	285710967	136	298	ENSSSCG00000005485	-	intron	*AMBP*
1	285711012	285711012	-	285711012	3	3	ENSSSCG00000005485	-	intron	*AMBP*
1	285711154	285711167	-	285711163	2007	3895	ENSSSCG00000005485	-	3'UTR	*AMBP*
1	285712382	285712382	-	285712382	4	4	ENSSSCG00000005485	-	3'UTR	*AMBP*
1	285713932	285713946	-	285713938	56	84	---	---	intergenic	---
1	285719913	285719913	-	285719913	3	3	---	---	intergenic	---
1	285720881	285720881	-	285720881	13	13	ENSSSCG00000005485	-	intron	*AMBP*
1	285722063	285722102	-	285722063	15	31	---	---	intergenic	---
1	285723901	285723901	-	285723901	16	16	ENSSSCG00000005485	-	intron	*AMBP*
1	285723979	285723979	-	285723979	3	3	---	---	intergenic	---
1	285725229	285725229	-	285725229	8	8	ENSSSCG00000005485	-	intron	*AMBP*
1	285739013	285739013	-	285739013	3	3	XLOC_003441	-	predicted	---
1	285921038	285921038	-	285921038	3	3	ENSSSCG00000023894	-	3'UTR	---

1	286250326	286250362	-	286250327	25	36	ENSSSCG00000005491	-	3'UTR	*ATP6V1G1*
1	286250764	286250764	-	286250764	4	4	ENSSSCG00000005491	-	3'UTR	*ATP6V1G1*
1	286250834	286250839	-	286250834	21	28	ENSSSCG00000005491	-	3'UTR	*ATP6V1G1*
1	286250958	286250978	-	286250977	11	29	ENSSSCG00000005491	-	ORF	*ATP6V1G1*
1	286263357	286263360	+	286263360	10	13	XLOC_001577	+	predicted	---
1	286264796	286264796	+	286264796	5	5	XLOC_001577	+	predicted	---
1	286267969	286267976	-	286267972	38	108	XLOC_003449	-	predicted	---
1	286274750	286274757	-	286274752	124	181	XLOC_003450	-	predicted	---
1	286304552	286304552	+	286304552	6	6	XLOC_001578	+	predicted	---
1	287347230	287347230	-	287347230	8	8	---	---	intergenic	---
1	287347663	287347664	-	287347664	25	28	---	---	intergenic	---
1	287844017	287844017	-	287844017	6	6	---	---	intergenic	---
1	288222950	288222950	+	288222950	9	9	XLOC_001589	+	predicted	---
1	288462900	288462903	+	288462900	19	22	ENSSSCG00000005501	+	3'UTR	*TRIM32*
							ENSSSCG00000005501	+	ORF	---
1	288483890	288483890	-	288483890	3	3	---	---	intergenic	---
1	288953766	288953772	-	288953767	50	100	---	---	intergenic	---
1	289433934	289433934	-	289433934	3	3	---	---	intergenic	---
1	289785606	289785606	+	289785606	4	4	ENSSSCG00000005503	+	3'UTR	*TLR4*
1	289785735	289785740	+	289785740	9	15	ENSSSCG00000005503	+	3'UTR	*TLR4*
1	289785809	289785845	+	289785845	9	12	ENSSSCG00000005503	+	3'UTR	*TLR4*
1	289938993	289938993	-	289938993	5	5	---	---	intergenic	---
1	289970016	289970017	+	289970017	7	11	---	---	intergenic	---
1	290082464	290082464	-	290082464	8	8	---	---	intergenic	---
1	290195793	290195793	-	290195793	5	5	---	---	intergenic	---
1	290222409	290222412	-	290222412	10	19	---	---	intergenic	---

1	290288031	290288031	+	290288031	32	32	---	---	intergenic	---
1	292115345	292115347	-	292115346	11	19	---	---	intergenic	---
1	292373282	292373285	+	292373282	28	55	ENSSSCG00000005505	+	3'UTR	---
1	292547119	292547119	+	292547119	3	3	ENSSSCG00000028551	+	ORF	---
1	292566438	292566438	+	292566438	41	41	ENSSSCG00000028551	+	intron	---
1	292704498	292704498	-	292704498	3	3	ENSSSCG00000028117	-	intron	---
1	292879895	292879895	-	292879895	6	6	---	---	intergenic	---
1	292891505	292891526	-	292891505	4	8	ENSSSCG00000021318	-	3'UTR	*FBXW2*
1	293058891	293058891	-	293058891	5	5	---	---	intergenic	---
1	293077415	293077422	-	293077419	27	51	ENSSSCG00000005512	-	3'UTR	*C5*
1	293104680	293104684	-	293104680	3	6	ENSSSCG00000005512	-	ORF	*C5*
1	293109406	293109406	-	293109406	3	3	ENSSSCG00000005512	-	ORF	*C5*
1	293134307	293134307	-	293134307	3	3	ENSSSCG00000005512	-	ORF	*C5*
1	293136378	293136378	-	293136378	4	4	ENSSSCG00000005512	-	ORF	*C5*
1	293145447	293145473	-	293145447	4	10	ENSSSCG00000005512	-	ORF	*C5*
1	293163213	293163213	-	293163213	3	3	ENSSSCG00000005512	-	ORF	*C5*
1	293200747	293200747	+	293200747	3	3	XLOC_001607	+	predicted	---
1	293286698	293286698	+	293286698	3	3	---	---	intergenic	---
1	293358306	293358306	+	293358306	33	33	XLOC_001609	+	predicted	---
1	293462907	293462907	+	293462907	25	25	---	---	intergenic	---
1	293504309	293504309	+	293504309	3	3	ENSSSCG00000005514	+	3'UTR	*RAB14*
1	293505666	293505669	+	293505669	22	25	XLOC_001610	+	predicted	---
1	293506260	293506261	+	293506260	12	16	XLOC_001610	+	predicted	---
1	293629349	293629349	-	293629349	12	12	ENSSSCG00000005518	-	3'UTR	*GGTA1*
1	293630977	293630977	-	293630977	6	6	ENSSSCG00000005518	-	3'UTR	*GGTA1*
1	293919649	293919649	-	293919649	5	5	---	---	intergenic	---

1	294248296	294248297	-	294248297	6	10	XLOC_003495	-	predicted	---
1	294278631	294278632	-	294278631	5	8	---	---	intergenic	---
1	294517290	294517290	+	294517290	3	3	---	---	intergenic	---
1	294544828	294544835	-	294544834	206	229	XLOC_003496	-	predicted	---
1	294594845	294594857	+	294594847	57	151	XLOC_001620	+	predicted	---
1	294599065	294599075	-	294599075	7	19	ENSSSCG00000005530	-	3'UTR	*LHX6*
1	294599138	294599138	-	294599138	4	4	ENSSSCG00000005530	-	3'UTR	*LHX6*
1	294633290	294633297	-	294633296	16	36	ENSSSCG00000030089	-	3'UTR	*RBM18*
1	294713756	294713763	+	294713757	79	98	ENSSSCG00000005532	+	3'UTR	*MRRF*
1	295133921	295133923	-	295133923	10	17	---	---	intergenic	---
1	295134056	295134056	-	295134056	3	3	---	---	intergenic	---
1	295237292	295237292	+	295237292	3	3	---	---	intergenic	---
1	295650072	295650072	-	295650072	6	6	---	---	intergenic	---
1	296082871	296082872	-	296082871	14	18	---	---	intergenic	---
1	296422178	296422181	-	296422178	5	8	---	---	intergenic	---
1	297090248	297090248	-	297090248	3	3	---	---	intergenic	---
1	297212593	297212593	-	297212593	4	4	XLOC_003521	-	predicted	---
1	297236920	297236922	-	297236920	11	18	XLOC_003522	-	predicted	---
1	297288721	297288721	-	297288721	5	5	XLOC_003523	-	predicted	---
1	297315564	297315565	+	297315564	19	28	---	---	intergenic	---
1	297478577	297478577	+	297478577	4	4	ENSSSCG00000023197	+	3'UTR	---
1	297478629	297478637	+	297478636	7	15	ENSSSCG00000023197	+	3'UTR	---
1	297483094	297483103	-	297483095	76	177	XLOC_003525	-	predicted	---
1	297497270	297497277	-	297497276	20	42	XLOC_003525	-	predicted	---
1	297497775	297497775	-	297497775	10	10	ENSSSCG00000005582	-	intron	*STRBP*
1	297822785	297822785	+	297822785	3	3	ENSSSCG00000005585	+	ORF	---

1	298108754	298108754	-	298108754	35	35	ENSSSCG00000026555	-	intron	*DENND1A*
1	298120354	298120354	-	298120354	3	3	ENSSSCG00000026555	-	intron	*DENND1A*
1	298451517	298451548	+	298451530	23	57	---	---	intergenic	---
1	298451692	298451692	+	298451692	3	3	---	---	intergenic	---
1	298580714	298580714	-	298580714	3	3	---	---	intergenic	---
1	298612721	298612721	+	298612721	6	6	---	---	intergenic	---
1	298806229	298806229	+	298806229	4	4	---	---	intergenic	---
1	298942107	298942110	+	298942109	52	71	ENSSSCG00000005587	+	3'UTR	*NEK6*
1	298943399	298943413	-	298943400	23	80	ENSSSCG00000005590	-	3'UTR	*PSMB7*
1	298953126	298953127	-	298953126	5	10	ENSSSCG00000005590	-	intron	*PSMB7*
1	299055647	299055647	-	299055647	5	5	XLOC_003533	-	predicted	---
1	299461614	299461614	-	299461614	3	3	ENSSSCG00000005595	-	3'UTR	*RPL35*
1	299461700	299461700	-	299461700	3	3	ENSSSCG00000005595	-	3'UTR	*RPL35*
1	299462239	299462278	-	299462247	1682	3397	ENSSSCG00000005595	-	3'UTR	*RPL35*
1	299462245	299462247	+	299462245	52	68	ENSSSCG00000005594	+	3'UTR	*WDR38*
1	299462340	299462340	-	299462340	4	4	ENSSSCG00000005595	-	ORF	*RPL35*
1	299465461	299465493	-	299465476	6	13	ENSSSCG00000005595	-	ORF	*RPL35*
1	299479043	299479048	+	299479046	15	28	ENSSSCG00000005596	+	3'UTR	*ARPC5L*
1	299481002	299481011	-	299481011	5	8	XLOC_003539	-	predicted	---
1	299482311	299482311	-	299482311	3	3	XLOC_003539	-	predicted	---
1	299531510	299531510	+	299531510	3	3	---	---	intergenic	---
1	299623049	299623049	+	299623049	3	3	---	---	intergenic	---
1	299665525	299665525	+	299665525	6	6	---	---	intergenic	---
1	299672839	299672842	-	299672841	29	44	ENSSSCG00000005599	-	3'UTR	*RABEPK*
1	299696326	299696326	-	299696326	3	3	---	---	intergenic	---
1	299696359	299696360	+	299696359	4	7	---	---	intergenic	---

1	299727164	299727179	+	299727172	39	76	ENSSSCG00000005600	+	3'UTR	*PPP6C*
1	299728143	299728143	+	299728143	3	3	XLOC_001672	+	predicted	---
1	299739802	299739802	+	299739802	7	7	---	---	intergenic	---
1	299754230	299754234	-	299754230	23	32	ENSSSCG00000005601	-	ORF	*HSPA5*
1	299754297	299754330	-	299754297	7	10	ENSSSCG00000005601	-	ORF	*HSPA5*
1	299754425	299754432	-	299754425	17	23	ENSSSCG00000005601	-	ORF	*HSPA5*
1	299756024	299756024	-	299756024	3	3	ENSSSCG00000005601	-	ORF	*HSPA5*
1	299756354	299756359	-	299756356	7	19	ENSSSCG00000005601	-	ORF	*HSPA5*
1	299757417	299757417	-	299757417	21	21	ENSSSCG00000005601	-	ORF	*HSPA5*
1	299757880	299757883	-	299757883	7	12	ENSSSCG00000005601	-	intron	*HSPA5*
1	299758260	299758260	-	299758260	4	4	ENSSSCG00000005601	-	ORF	*HSPA5*
1	299759532	299759532	+	299759532	3	3	---	---	intergenic	---
1	299765732	299765736	-	299765736	4	10	---	---	intergenic	---
1	299781722	299781722	+	299781722	4	4	ENSSSCG00000005602	+	intron	*GAPVD1*
1	299839406	299839406	-	299839406	3	3	XLOC_003544	-	predicted	---
1	299840468	299840471	-	299840468	12	22	XLOC_003544	-	predicted	---
1	299840553	299840553	-	299840553	3	3	ENSSSCG00000029141	-	3'UTR	---
1	299868183	299868183	-	299868183	3	3	ENSSSCG00000029141	-	intron	---
1	299984610	299984610	+	299984610	6	6	XLOC_001674	+	predicted	---
1	299985043	299985043	+	299985043	4	4	XLOC_001674	+	predicted	---
1	300433682	300433685	+	300433685	11	14	ENSSSCG00000005604	+	3'UTR	*PBX3*
1	300666862	300666862	-	300666862	5	5	---	---	intergenic	---
1	301005169	301005170	+	301005169	4	7	---	---	intergenic	---
1	301050609	301050609	-	301050609	3	3	XLOC_003550	-	predicted	---
1	301149121	301149121	-	301149121	6	6	XLOC_003551	-	predicted	---
1	301193528	301193528	-	301193528	4	4	---	---	intergenic	---

1	301339023	301339023	+	301339023	3	3	---	---	intergenic	---
1	301362526	301362526	+	301362526	3	3	---	---	intergenic	---
1	301378041	301378041	+	301378041	8	8	---	---	intergenic	---
1	301511219	301511219	+	301511219	4	4	ENSSSCG00000005607	+	intron	*RALGPS1*
1	301554340	301554340	-	301554340	5	5	ENSSSCG00000005608	-	3'UTR	*ANGPTL2*
1	301608172	301608172	+	301608172	3	3	ENSSSCG00000005607	+	intron	*RALGPS1*
1	301624069	301624069	+	301624069	3	3	ENSSSCG00000005607	+	intron	*RALGPS1*
1	301679986	301679986	+	301679986	3	3	XLOC_001682	+	predicted	---
1	301680214	301680214	+	301680214	3	3	XLOC_001682	+	predicted	---
1	301683984	301683984	+	301683984	3	3	XLOC_001682	+	predicted	---
1	301811763	301811763	+	301811763	11	11	ENSSSCG00000005609	+	intron	---
1	301832606	301832615	+	301832606	7	10	ENSSSCG00000005609	+	intron	---
1	301844611	301844618	+	301844617	14	47	ENSSSCG00000005610	+	3'UTR	*SLC2A8*
1	301870273	301870278	+	301870273	5	10	XLOC_001689	+	predicted	---
1	301873277	301873277	-	301873277	3	3	ENSSSCG00000005612	-	ORF	*RPL12*
1	301920997	301920997	-	301920997	8	8	ENSSSCG00000005614	-	3'UTR	*FAM129B*
1	301992243	301992244	-	301992243	3	6	---	---	intergenic	---
1	302017169	302017169	-	302017169	3	3	---	---	intergenic	---
1	302094400	302094405	-	302094400	4	11	XLOC_003567	-	predicted	---
1	302098187	302098187	+	302098187	7	7	XLOC_001691	+	predicted	---
1	302125766	302125771	-	302125769	7	15	XLOC_003570	-	predicted	---
1	302129602	302129602	-	302129602	3	3	ENSSSCG00000005620	-	3'UTR	*SH2D3C*
1	302207378	302207382	+	302207381	78	110	ENSSSCG00000005621	+	3'UTR	*CDK9*
1	302232070	302232070	-	302232070	31	31	ENSSSCG00000005625	-	3'UTR	*ENG*
1	302278354	302278364	-	302278358	33	101	ENSSSCG00000005627	-	3'UTR	*AK1*
1	302293881	302293883	-	302293882	16	34	XLOC_003574	-	predicted	---

1	302314227	302314235	-	302314229	19	36	ENSSSCG00000005628	-	3'UTR	*ST6GALNAC2*
1	302342027	302342027	-	302342027	8	8	ENSSSCG00000005632	-	3'UTR	*DPM2*
1	302349461	302349463	-	302349461	16	31	ENSSSCG00000005631	-	3'UTR	*FAM102A*
1	302567456	302567464	+	302567456	33	66	ENSSSCG00000005636	+	3'UTR	*SLC25A25*
1	302605198	302605198	+	302605198	8	8	ENSSSCG00000005638	+	3'UTR	*LCN2*
1	302610200	302610208	+	302610200	39	67	ENSSSCG00000022295	+	3'UTR	*C9orf16*
1	302611408	302611416	-	302611414	9	22	XLOC_003582	-	predicted	---
1	302657017	302657017	+	302657017	3	3	ENSSSCG00000005641	+	intron	*DNM1*
1	302693138	302693141	-	302693139	9	30	XLOC_003585	-	predicted	---
1	302702509	302702509	-	302702509	64	64	---	---	intergenic	---
1	302703347	302703347	-	302703347	3	3	---	---	intergenic	---
1	302710754	302710754	-	302710754	3	3	---	---	intergenic	---
1	302716831	302716863	+	302716857	178	594	ENSSSCG00000005644	+	3'UTR	*SWI5*
1	302770842	302770842	-	302770842	3	3	ENSSSCG00000005645	-	intron	---
1	302778919	302778919	+	302778919	13	13	ENSSSCG00000005648	+	3'UTR	*FATP4*
1	302805040	302805048	+	302805048	21	61	ENSSSCG00000005649	+	3'UTR	*URM1*
1	302879799	302879799	-	302879799	3	3	---	---	intergenic	---
1	302902423	302902423	+	302902423	16	16	ENSSSCG00000005651	+	ORF	*ODF2*
1	302907681	302907705	+	302907681	6	9	ENSSSCG00000005651	+	ORF	*ODF2*
1	302909585	302909641	+	302909633	834	1654	ENSSSCG00000005651	+	intron	*ODF2*
1	302915440	302915440	+	302915440	4	4	ENSSSCG00000005651	+	3'UTR	*ODF2*
1	302915544	302915544	+	302915544	9	9	ENSSSCG00000005651	+	3'UTR	*ODF2*
1	302916885	302916887	+	302916886	5	11	ENSSSCG00000005651	+	3'UTR	*ODF2*
1	302932406	302932406	+	302932406	3	3	ENSSSCG00000005652	+	intron	*GLE1*
1	302944938	302944949	+	302944940	43	96	XLOC_001716	+	predicted	---

1	302946280	302946280	+	302946280	3	3	XLOC_001716	+	predicted	---
1	303004723	303004723	+	303004723	5	5	ENSSSCG00000005654	+	ORF	*SPTAN1*
1	303025982	303025995	-	303025988	17	49	ENSSSCG00000005655	-	3'UTR	*WDR34*
1	303025985	303025987	+	303025986	46	70	ENSSSCG00000005654	+	3'UTR	*SPTAN1*
1	303084441	303084441	+	303084441	4	4	ENSSSCG00000005656	+	ORF	*SET*
1	303087163	303087163	+	303087163	4	4	ENSSSCG00000005656	+	3'UTR	*SET*
1	303087360	303087365	+	303087364	12	31	ENSSSCG00000005656	+	3'UTR	*SET*
1	303088274	303088276	+	303088274	12	21	ENSSSCG00000005656	+	3'UTR	*SET*
1	303105131	303105134	-	303105131	4	8	XLOC_003592	-	predicted	---
1	303108884	303108892	-	303108885	12	28	XLOC_003593	-	predicted	---
1	303168333	303168334	+	303168333	13	17	XLOC_001721	+	predicted	---
1	303173223	303173225	-	303173223	7	12	XLOC_003596	-	predicted	---
1	303173837	303173837	+	303173837	5	5	ENSSSCG00000005661	+	3'UTR	*ENDOG*
1	303188685	303188693	-	303188692	10	19	ENSSSCG00000005663	-	3'UTR	*CCBL1*
1	303204265	303204265	-	303204265	10	10	ENSSSCG00000005663	-	intron	*CCBL1*
1	303253676	303253676	+	303253676	3	3	ENSSSCG00000005664	+	3'UTR	*LRRC8A*
1	303254626	303254626	+	303254626	3	3	ENSSSCG00000005664	+	3'UTR	*LRRC8A*
1	303270693	303270700	+	303270699	14	32	XLOC_001723	+	predicted	---
1	303273507	303273507	-	303273507	7	7	---	---	intergenic	---
1	303276716	303276716	+	303276716	10	10	ENSSSCG00000005667	+	intron	*NUP188*
1	303327198	303327200	+	303327200	4	7	ENSSSCG00000005667	+	ORF	*NUP188*
1	303328986	303328995	+	303328993	12	58	ENSSSCG00000005667	+	3'UTR	*NUP188*
1	303329918	303329919	-	303329918	10	18	ENSSSCG00000005668	-	3'UTR	*SH3GLB2*
1	303380130	303380133	+	303380132	24	36	ENSSSCG00000005669	+	3'UTR	*FAM73B*
1	303400133	303400135	+	303400133	5	11	ENSSSCG00000005670	+	3'UTR	*DOLPP1*
1	303403325	303403325	-	303403325	3	3	XLOC_003601	-	predicted	---

1	303403628	303403642	-	303403630	61	210	ENSSSCG00000005671	-	3'UTR	*CRAT*
1	303447663	303447668	+	303447666	28	73	ENSSSCG00000005672	+	3'UTR	*PPP2R4*
1	303508639	303508639	-	303508639	3	3	---	---	intergenic	---
1	303695879	303695883	+	303695881	37	61	ENSSSCG00000005678	+	3'UTR	*NTMT1*
1	303696573	303696573	-	303696573	4	4	ENSSSCG00000005679	-	3'UTR	*ASB6*
1	303779645	303779650	-	303779645	8	11	XLOC_003606	-	predicted	---
1	303780087	303780087	-	303780087	3	3	ENSSSCG00000005688	-	3'UTR	*PTGES*
1	303807617	303807629	+	303807617	5	8	XLOC_001732	+	predicted	---
1	303807666	303807678	+	303807667	99	362	XLOC_001732	+	predicted	---
1	303870969	303870976	+	303870975	7	13	XLOC_001734	+	predicted	---
1	303872845	303872865	-	303872847	10	56	ENSSSCG00000025964	-	3'UTR	*DYT1*
1	303883696	303883703	-	303883699	12	35	ENSSSCG00000030426	-	3'UTR	*C9orf78*
1	303884015	303884015	-	303884015	23	23	ENSSSCG00000030426	-	3'UTR	*C9orf78*
1	303887761	303887761	-	303887761	6	6	ENSSSCG00000030426	-	intron	*C9orf78*
1	303888785	303888785	-	303888785	3	3	ENSSSCG00000030426	-	ORF	*C9orf78*
1	303935524	303935534	+	303935531	8	31	ENSSSCG00000024341	+	3'UTR	*USP20*
1	303940418	303940421	-	303940418	5	13	---	---	intergenic	---
1	303945888	303945888	-	303945888	3	3	ENSSSCG00000005689	-	3'UTR	*FNBP1*
1	303952072	303952072	-	303952072	9	9	ENSSSCG00000005689	-	intron	*FNBP1*
1	304445871	304445871	+	304445871	3	3	XLOC_001737	+	predicted	---
1	304507774	304507775	+	304507774	3	6	ENSSSCG00000005701	+	intron	*ASS1*
1	304508980	304508990	+	304508986	168	562	ENSSSCG00000005701	+	3'UTR	*ASS1*
1	304521871	304521871	-	304521871	12	12	---	---	intergenic	---
1	304566110	304566110	-	304566110	7	7	---	---	intergenic	---
1	304573677	304573677	-	304573677	3	3	---	---	intergenic	---
1	304624686	304624686	+	304624686	3	3	ENSSSCG00000005702	+	3'UTR	*FUBP3*

1	304625554	304625560	+	304625554	30	37	ENSSSCG00000005702	+	3'UTR	*FUBP3*
1	304631104	304631109	-	304631108	24	71	---	---	intergenic	---
1	304631146	304631149	+	304631149	4	7	---	---	intergenic	---
1	304680408	304680408	+	304680408	6	6	XLOC_001742	+	predicted	---
1	304829645	304829653	+	304829645	5	15	ENSSSCG00000005706	+	3'UTR	*ABL1*
1	304954777	304954777	-	304954777	3	3	---	---	intergenic	---
1	305016357	305016357	+	305016357	3	3	ENSSSCG00000005710	+	intron	*LAMC3*
1	305098320	305098323	+	305098322	18	37	ENSSSCG00000005710	+	3'UTR	*LAMC3*
1	305129755	305129755	+	305129755	3	3	XLOC_001745	+	predicted	---
1	305139442	305139442	+	305139442	11	11	---	---	intergenic	---
1	305237536	305237539	+	305237538	17	39	ENSSSCG00000005711	+	3'UTR	*NUP214*
1	305271860	305271860	-	305271860	11	11	ENSSSCG00000028172	-	3'UTR	*FAM78A*
1	305272033	305272055	-	305272033	6	9	ENSSSCG00000028172	-	3'UTR	*FAM78A*
1	305290020	305290026	+	305290026	7	15	---	---	intergenic	---
1	305293526	305293534	+	305293526	4	8	XLOC_001748	+	predicted	---
1	305353468	305353468	-	305353468	3	3	ENSSSCG00000022130	-	ORF	---
1	305456739	305456742	+	305456740	25	48	XLOC_001752	+	predicted	---
1	305456744	305456745	-	305456744	18	21	ENSSSCG00000005717	-	3'UTR	*UCK1*
1	305502208	305502220	-	305502208	8	12	ENSSSCG00000005719	-	3'UTR	*RAPGEF1*
1	305648350	305648350	+	305648350	4	4	---	---	intergenic	---
1	305733695	305733703	-	305733700	11	31	ENSSSCG00000005720	-	3'UTR	*MED27*
1	305785065	305785066	-	305785065	41	48	ENSSSCG00000005720	-	intron	*MED27*
1	305988709	305988709	-	305988709	5	5	---	---	intergenic	---
1	306148226	306148226	+	306148226	3	3	XLOC_001758	+	predicted	---
1	306152450	306152450	-	306152450	4	4	XLOC_003630	-	predicted	---
1	306154422	306154422	-	306154422	17	17	XLOC_003630	-	predicted	---

1	306181551	306181551	-	306181551	4	4	ENSSSCG00000005724	-	ORF	*SETX*
1	306337141	306337142	+	306337141	9	12	ENSSSCG00000005727	+	intron	*C9orf171*
1	306394535	306394538	+	306394535	4	7	XLOC_001759	+	predicted	---
1	306507469	306507469	+	306507469	4	4	XLOC_001762	+	predicted	---
1	306529986	306530005	-	306530002	25	68	XLOC_003633	-	predicted	---
1	306543056	306543056	-	306543056	3	3	---	---	intergenic	---
1	306713800	306713800	+	306713800	8	8	ENSSSCG00000005738	+	3'UTR	*RALGDS*
1	306729972	306729975	-	306729972	4	8	---	---	intergenic	---
1	306789043	306789050	+	306789047	76	160	XLOC_001766	+	predicted	---
1	306789319	306789323	-	306789322	16	44	ENSSSCG00000005739	-	intron	*GTF3C5*
1	306795202	306795203	-	306795202	9	13	ENSSSCG00000005733	-	3'UTR	---
							ENSSSCG00000005739	-	intron	*GTF3C5*
1	306859045	306859045	-	306859045	4	4	ENSSSCG00000005739	-	intron	*GTF3C5*
1	306874950	306874950	-	306874950	3	3	ENSSSCG00000005739	-	intron	*GTF3C5*
1	306931669	306931680	+	306931680	4	7	XLOC_001769	+	predicted	---
1	306985546	306985546	-	306985546	5	5	ENSSSCG00000023173	-	3'UTR	*REXO4*
1	307029853	307029855	+	307029854	11	19	ENSSSCG00000021241	+	intron	*ADAMTS13*
1	307040729	307040729	+	307040729	15	15	XLOC_001770	+	predicted	---
1	307041088	307041088	-	307041088	3	3	ENSSSCG00000024166	-	3'UTR	*SLC2A6*
1	307129230	307129230	+	307129230	3	3	XLOC_001772	+	predicted	---
1	307133877	307133884	+	307133882	32	76	XLOC_001773	+	predicted	---
1	307204204	307204211	-	307204209	7	17	ENSSSCG00000005740	-	3'UTR	*SARDH*
1	307258260	307258260	-	307258260	3	3	ENSSSCG00000005740	-	intron	*SARDH*
1	307276070	307276070	-	307276070	3	3	ENSSSCG00000005743	-	3'UTR	*VAV2*
1	307473050	307473055	+	307473050	6	17	XLOC_001777	+	predicted	---
1	307531420	307531420	-	307531420	3	3	XLOC_003651	-	predicted	---

1	307549505	307549510	+	307549510	16	27	ENSSSCG00000024912	+	3'UTR	*WDR5*
1	307550805	307550805	+	307550805	4	4	ENSSSCG00000024912	+	3'UTR	*WDR5*
1	308174532	308174532	+	308174532	3	3	XLOC_001783	+	predicted	---
1	308176185	308176185	+	308176185	4	4	XLOC_001783	+	predicted	---
1	308203076	308203083	+	308203077	421	496	ENSSSCG00000023333	+	3'UTR	*FCN2*
1	308216807	308216814	-	308216807	39	64	ENSSSCG00000029414	-	3'UTR	*FCN1*
1	308337501	308337503	+	308337501	21	27	ENSSSCG00000029715	+	intron	*OLFM1*
1	308353271	308353273	+	308353271	16	19	ENSSSCG00000029715	+	3'UTR	*OLFM1*
1	308513178	308513178	-	308513178	3	3	---	---	intergenic	---
1	308630628	308630637	-	308630635	20	41	ENSSSCG00000005760	-	3'UTR	*C9orf116*
1	308638755	308638771	+	308638755	6	14	ENSSSCG00000005759	+	3'UTR	*MRPS2*
1	308669072	308669076	+	308669073	142	214	ENSSSCG00000005752	+	3'UTR	*BLG*
1	308680070	308680071	-	308680070	6	9	XLOC_003667	-	predicted	---
1	308722670	308722671	-	308722670	4	7	XLOC_003668	-	predicted	---
1	308852511	308852511	-	308852511	4	4	ENSSSCG00000005753	-	3'UTR	*CAMSAP1*
1	308956418	308956418	+	308956418	3	3	---	---	intergenic	---
1	309240038	309240038	-	309240038	4	4	---	---	intergenic	---
1	309277621	309277621	+	309277621	3	3	---	---	intergenic	---
1	309277689	309277690	+	309277689	3	6	---	---	intergenic	---
1	309490253	309490287	-	309490253	3	6	XLOC_003674	-	predicted	---
1	309577917	309577917	+	309577917	3	3	ENSSSCG00000005768	+	intron	---
1	309591293	309591295	+	309591294	9	17	ENSSSCG00000005768	+	3'UTR	---
1	309592152	309592158	+	309592157	48	80	ENSSSCG00000005768	+	3'UTR	---
1	309592286	309592298	+	309592286	3	6	ENSSSCG00000005768	+	3'UTR	---
1	309649723	309649723	+	309649723	3	3	XLOC_001804	+	predicted	---
1	309650254	309650254	+	309650254	3	3	XLOC_001804	+	predicted	---

1	309657517	309657517	-	309657517	4	4	XLOC_003677	-	predicted	---
1	309751323	309751323	+	309751323	4	4	---	---	intergenic	---
1	309781395	309781395	-	309781395	3	3	---	---	intergenic	---
1	309959065	309959065	+	309959065	3	3	---	---	intergenic	---
1	310003282	310003282	-	310003282	7	7	XLOC_003679	-	predicted	---
1	310067122	310067122	-	310067122	3	3	ENSSSCG00000005773	-	3'UTR	*RBFA*
1	310085837	310085837	-	310085837	3	3	XLOC_003681	-	predicted	---
1	310087849	310087860	-	310087860	4	7	XLOC_003681	-	predicted	---
1	310087953	310087958	-	310087955	8	26	XLOC_003681	-	predicted	---
1	310088015	310088015	-	310088015	5	5	ENSSSCG00000005776	-	ORF	---
1	310140421	310140434	+	310140427	34	76	ENSSSCG00000005777	+	3'UTR	*TXNL4A*
1	310225643	310225643	+	310225643	7	7	---	---	intergenic	---
1	310226014	310226016	-	310226016	11	14	---	---	intergenic	---
1	310226064	310226064	+	310226064	3	3	---	---	intergenic	---
1	311068026	311068026	+	311068026	3	3	ENSSSCG00000005781	+	intron	*TARSL2*
1	311068108	311068108	+	311068108	3	3	ENSSSCG00000005781	+	intron	*TARSL2*
1	311077813	311077814	+	311077813	4	7	ENSSSCG00000005781	+	3'UTR	*TARSL2*
1	311091263	311091271	+	311091263	22	70	ENSSSCG00000005782	+	3'UTR	*TM2D3*
1	311091687	311091687	+	311091687	3	3	XLOC_001815	+	predicted	---
1	311180808	311180811	+	311180809	4	10	---	---	intergenic	---
1	311582520	311582528	+	311582521	8	19	ENSSSCG00000005787	+	3'UTR	---
1	311596003	311596009	+	311596007	45	92	XLOC_001819	+	predicted	---
1	311632952	311632958	-	311632954	95	212	ENSSSCG00000005786	-	3'UTR	*SNRPA1*
1	311905978	311905979	-	311905978	5	8	---	---	intergenic	---
1	311949065	311949065	+	311949065	13	13	---	---	intergenic	---
1	312365249	312365249	-	312365249	3	3	---	---	intergenic	---

1	312365855	312365857	-	312365856	33	47	---	---	intergenic	---
1	312560655	312560691	+	312560691	5	9	ENSSSCG00000005813	+	ORF	---
1	312574574	312574576	-	312574576	17	37	---	---	intergenic	---
1	312969357	312969357	-	312969357	3	3	---	---	intergenic	---
1	313091518	313091518	-	313091518	4	4	XLOC_003716	-	predicted	---
1	313094726	313094726	-	313094726	7	7	XLOC_003716	-	predicted	---
1	313098181	313098200	-	313098200	10	13	XLOC_003716	-	predicted	---
1	313736341	313736341	+	313736341	9	9	ENSSSCG00000005828	+	3'UTR	*EGFL7*
1	313736714	313736724	-	313736721	17	48	XLOC_003720	-	predicted	---
1	313757725	313757732	-	313757728	600	1022	ENSSSCG00000005840	-	3'UTR	*C8G*
1	313764354	313764361	+	313764361	26	67	XLOC_001846	+	predicted	---
1	313771821	313771828	-	313771828	5	8	ENSSSCG00000005838	-	3'UTR	*TRAF2*
1	313786065	313786065	-	313786065	4	4	---	---	intergenic	---
1	313806923	313806923	+	313806923	4	4	ENSSSCG00000005833	+	ORF	*MBF1*
1	313807446	313807446	+	313807446	14	14	ENSSSCG00000005833	+	ORF	*MBF1*
1	313808686	313808690	+	313808688	22	56	ENSSSCG00000005833	+	3'UTR	*MBF1*
1	313809274	313809274	-	313809274	3	3	ENSSSCG00000005832	-	3'UTR	*MAMDC4*
1	313819278	313819292	-	313819280	96	327	XLOC_003726	-	predicted	---
1	313854423	313854424	-	313854423	9	15	XLOC_003727	-	predicted	---
1	313878945	313878951	+	313878949	124	231	ENSSSCG00000005842	+	3'UTR	*TMEM141*
1	313883542	313883542	+	313883542	4	4	ENSSSCG00000005830	+	intron	*CCDC183*
1	313890320	313890322	+	313890322	11	21	XLOC_001859	+	predicted	---
1	313920704	313920704	-	313920704	9	9	ENSSSCG00000005841	-	3'UTR	*RABL6*
1	313982377	313982380	+	313982378	11	23	XLOC_001860	+	predicted	---
1	314012810	314012821	+	314012811	5	26	XLOC_001862	+	predicted	---
1	314078843	314078846	+	314078844	20	37	ENSSSCG00000005855	+	3'UTR	*EXD3*

1	314112198	314112198	-	314112198	7	7	ENSSSCG00000005845	-	3'UTR	*TOR4A*
1	314117258	314117260	-	314117259	13	19	ENSSSCG00000005846	-	3'UTR	*NELFB*
1	314135345	314135371	-	314135351	703	1721	ENSSSCG00000005849	-	3'UTR	*TUBB4B*
1	314135482	314135498	+	314135482	11	33	XLOC_001865	+	predicted	---
2	93867	93867	-	93867	3	3	ENSSSCG00000012836	-	intron	*AP2A2*
2	122002	122010	+	122004	107	335	ENSSSCG00000012837	+	3'UTR	*CHID1*
2	123628	123628	-	123628	5	5	ENSSSCG00000012838	-	3'UTR	*TSPAN4*
2	142458	142458	-	142458	3	3	---	---	intergenic	---
2	143697	143736	+	143718	175	255	---	---	intergenic	---
2	144770	144773	-	144771	61	135	ENSSSCG00000012840	-	3'UTR	*CD151*
2	156380	156382	-	156382	23	43	ENSSSCG00000012841	-	3'UTR	*ATGL*
2	164511	164553	-	164531	1199	2527	ENSSSCG00000012842	-	3'UTR	---
2	164745	164752	-	164752	13	23	ENSSSCG00000012842	-	intron	---
							ENSSSCG00000012842	-	ORF	---
2	183067	183077	+	183077	9	12	ENSSSCG00000012844	+	3'UTR	*SLC25A22*
2	200073	200077	+	200073	6	9	ENSSSCG00000012846	+	3'UTR	*PDDC1*
2	201527	201534	-	201532	343	962	ENSSSCG00000012847	-	3'UTR	*TALDO1*
2	221441	221444	-	221444	8	13	ENSSSCG00000012848	-	3'UTR	*EPS8L2*
2	240546	240547	-	240547	10	13	ENSSSCG00000012849	-	3'UTR	*TMEM80*
2	240596	240596	-	240596	3	3	ENSSSCG00000012849	-	3'UTR	*TMEM80*
2	269548	269571	+	269552	9	24	ENSSSCG00000012850	+	3'UTR	*DEAF1*
2	302178	302180	+	302178	25	32	ENSSSCG00000012853	+	3'UTR	*IRF7*
2	302719	302719	-	302719	3	3	ENSSSCG00000029723	-	3'UTR	*PHRF1*
2	341916	341916	-	341916	7	7	ENSSSCG00000012854	-	3'UTR	*RASSF7*
2	350720	350721	-	350721	9	13	XLOC_049103	-	predicted	---
2	425911	425924	+	425919	33	91	XLOC_047599	+	predicted	---

2	439709	439722	-	439715	80	247	ENSSSCG00000012856	-	3'UTR	*NAP1L4*
2	440558	440559	-	440558	3	6	ENSSSCG00000012856	-	3'UTR	*NAP1L4*
2	440620	440641	-	440635	49	98	ENSSSCG00000012856	-	3'UTR	*NAP1L4*
2	443198	443198	-	443198	5	5	ENSSSCG00000012856	-	intron	*NAP1L4*
2	446541	446541	-	446541	3	3	ENSSSCG00000012856	-	ORF	*NAP1L4*
2	458929	458929	-	458929	3	3	ENSSSCG00000012856	-	ORF	*NAP1L4*
2	494736	494743	-	494737	27	65	XLOC_049108	-	predicted	---
2	725956	725975	-	725956	8	19	ENSSSCG00000012859	-	3'UTR	---
2	851379	851411	-	851406	50	93	XLOC_049119	-	predicted	---
2	1268011	1268019	+	1268011	8	16	---	---	intergenic	---
2	1466852	1466852	+	1466852	5	5	XLOC_047616	+	predicted	---
2	1467525	1467531	+	1467529	5	12	XLOC_047616	+	predicted	---
2	1467768	1467768	+	1467768	4	4	XLOC_047616	+	predicted	---
2	1543855	1543855	+	1543855	5	5	---	---	intergenic	---
2	1795079	1795079	-	1795079	3	3	XLOC_049132	-	predicted	---
2	1831039	1831039	+	1831039	3	3	---	---	intergenic	---
2	2096670	2096696	-	2096681	9	15	ENSSSCG00000012874	-	intron	*ORAOV1*
2	2768422	2768422	-	2768422	3	3	ENSSSCG00000012878	-	intron	*IGHMBP2*
2	2785356	2785361	+	2785359	26	44	ENSSSCG00000012879	+	3'UTR	*MRPL21*
2	2795595	2795595	-	2795595	3	3	---	---	intergenic	---
2	2797564	2797564	+	2797564	3	3	XLOC_047631	+	predicted	---
2	2873256	2873256	+	2873256	3	3	ENSSSCG00000012880	+	3'UTR	*CPT1A*
2	2873341	2873341	+	2873341	3	3	ENSSSCG00000012880	+	3'UTR	*CPT1A*
2	2873951	2873952	+	2873952	7	10	ENSSSCG00000012880	+	3'UTR	*CPT1A*
2	2874134	2874144	+	2874139	81	196	ENSSSCG00000012880	+	3'UTR	*CPT1A*
2	2886448	2886450	+	2886448	7	10	ENSSSCG00000012882	+	intron	*MTL5*

2	2921157	2921159	+	2921158	21	27	XLOC_047633	+	predicted	---
2	2974929	2974931	-	2974929	6	9	---	---	intergenic	---
2	2975481	2975481	-	2975481	14	14	---	---	intergenic	---
2	3124483	3124483	-	3124483	3	3	---	---	intergenic	---
2	3132440	3132440	-	3132440	6	6	---	---	intergenic	---
2	3175232	3175232	-	3175232	3	3	ENSSSCG00000012885	-	intron	---
2	3238250	3238251	-	3238251	7	10	XLOC_049144	-	predicted	---
2	3256840	3256840	+	3256840	3	3	ENSSSCG00000012886	+	3'UTR	---
2	3256952	3256955	+	3256952	3	9	ENSSSCG00000012886	+	3'UTR	---
2	3322929	3322935	+	3322931	12	23	ENSSSCG00000012887	+	intron	---
2	3332598	3332598	+	3332598	7	7	ENSSSCG00000012887	+	3'UTR	---
2	3342538	3342538	-	3342538	3	3	XLOC_049147	-	predicted	---
2	3342605	3342605	-	3342605	3	3	XLOC_049147	-	predicted	---
2	3413834	3413834	-	3413834	6	6	---	---	intergenic	---
2	3474458	3474470	+	3474470	10	14	XLOC_047641	+	predicted	---
2	3476373	3476378	-	3476374	22	40	XLOC_049150	-	predicted	---
2	3491221	3491228	-	3491224	140	258	XLOC_049151	-	predicted	---
2	3496491	3496491	-	3496491	9	9	---	---	intergenic	---
2	3536749	3536749	+	3536749	4	4	XLOC_047643	+	predicted	---
2	3550646	3550646	+	3550646	5	5	ENSSSCG00000012893	+	3'UTR	---
2	3551051	3551051	+	3551051	10	10	ENSSSCG00000012893	+	3'UTR	---
2	3592839	3592845	+	3592840	9	19	XLOC_047647	+	predicted	---
2	3598805	3598825	-	3598821	62	177	ENSSSCG00000012896	-	3'UTR	*NDUFV1*
2	3618595	3618618	-	3618603	187	401	XLOC_049153	-	predicted	---
2	3746264	3746278	+	3746276	49	77	ENSSSCG00000012902	+	3'UTR	*CDK2AP2*
2	3760856	3760858	+	3760856	30	46	XLOC_047652	+	predicted	---

2	3761438	3761441	-	3761439	39	53	XLOC_049161	-	predicted	---
2	3769627	3769627	+	3769627	6	6	---	---	intergenic	---
2	3780768	3780771	+	3780768	22	46	ENSSSCG00000012905	+	3'UTR	*TMEM134*
2	3782301	3782306	+	3782301	5	9	ENSSSCG00000012905	+	3'UTR	*TMEM134*
2	3804406	3804406	+	3804406	13	13	XLOC_047654	+	predicted	---
2	3807116	3807118	+	3807118	20	42	XLOC_047655	+	predicted	---
2	3807228	3807228	-	3807228	5	5	ENSSSCG00000012910	-	3'UTR	*RPS6KB2*
2	3815001	3815003	-	3815002	19	41	ENSSSCG00000012911	-	3'UTR	*CARNS1*
2	3830990	3830992	-	3830992	10	19	ENSSSCG00000012912	-	3'UTR	*TBC1D10C*
2	3841894	3841902	+	3841900	48	69	ENSSSCG00000012913	+	3'UTR	*PPP1CA*
2	3946381	3946392	+	3946384	80	235	ENSSSCG00000012916	+	3'UTR	---
2	3968549	3968560	-	3968560	4	7	ENSSSCG00000027956	-	3'UTR	*SSH3*
2	3992506	3992507	-	3992506	28	31	ENSSSCG00000012918	-	3'UTR	*ADRBK1*
2	3993568	3993568	-	3993568	7	7	ENSSSCG00000012918	-	3'UTR	*ADRBK1*
2	4238573	4238576	+	4238575	10	29	ENSSSCG00000022726	+	3'UTR	*RHOD*
2	4260487	4260487	-	4260487	3	3	---	---	intergenic	---
2	4594341	4594342	+	4594341	7	10	---	---	intergenic	---
2	4608962	4608962	+	4608962	5	5	ENSSSCG00000012926	+	ORF	*PC*
2	4610107	4610117	+	4610114	154	518	XLOC_047668	+	predicted	---
2	4610469	4610471	+	4610471	10	18	XLOC_047668	+	predicted	---
2	4611910	4611910	-	4611910	7	7	XLOC_049178	-	predicted	---
2	4615180	4615180	-	4615180	5	5	XLOC_049178	-	predicted	---
2	4863946	4863946	-	4863946	3	3	XLOC_049181	-	predicted	---
2	5057898	5057904	+	5057902	60	90	ENSSSCG00000012943	+	3'UTR	*MRPL11*
2	5112827	5112827	+	5112827	3	3	XLOC_047677	+	predicted	---
2	5122372	5122375	+	5122373	13	27	ENSSSCG00000012940	+	3'UTR	*B4GAT1*

2	5130220	5130231	+	5130226	42	82	ENSSSCG00000012939	+	3'UTR	*BRMS1*
2	5178233	5178243	+	5178241	138	427	ENSSSCG00000012953	+	3'UTR	*YIF1A*
2	5189050	5189050	-	5189050	3	3	XLOC_049191	-	predicted	---
2	5210249	5210253	-	5210249	7	11	ENSSSCG00000012956	-	3'UTR	*PACS1*
2	5224362	5224362	-	5224362	3	3	ENSSSCG00000012956	-	intron	*PACS1*
2	5284500	5284500	+	5284500	3	3	XLOC_047684	+	predicted	---
2	5318799	5318799	-	5318799	4	4	---	---	intergenic	---
2	5326154	5326154	-	5326154	26	26	---	---	intergenic	---
2	5351061	5351078	-	5351062	37	106	XLOC_049193	-	predicted	---
2	5351653	5351653	-	5351653	9	9	ENSSSCG00000012957	-	ORF	*SF3B2*
2	5354796	5354796	-	5354796	3	3	ENSSSCG00000012957	-	ORF	*SF3B2*
2	5360821	5360821	-	5360821	11	11	ENSSSCG00000012957	-	ORF	*SF3B2*
2	5361064	5361067	-	5361064	3	6	ENSSSCG00000012957	-	intron	*SF3B2*
2	5459382	5459384	-	5459383	18	37	ENSSSCG00000012963	-	3'UTR	*SART1*
2	5478313	5478314	-	5478314	4	7	XLOC_049199	-	predicted	---
2	5497910	5497910	+	5497910	6	6	XLOC_047692	+	predicted	---
2	5513476	5513489	-	5513480	56	93	ENSSSCG00000012965	-	3'UTR	*DRAP1*
2	5513604	5513604	-	5513604	3	3	ENSSSCG00000012965	-	ORF	*DRAP1*
2	5518124	5518138	+	5518136	17	50	ENSSSCG00000012966	+	3'UTR	*C11orf68*
							ENSSSCG00000012966	+	ORF	---
2	5540816	5540817	-	5540817	12	15	XLOC_049201	-	predicted	---
2	5549947	5549957	+	5549951	84	188	ENSSSCG00000012969	+	3'UTR	*FIBP*
2	5550130	5550134	-	5550132	15	36	ENSSSCG00000012970	-	3'UTR	*CTSW*
2	5566997	5566997	+	5566997	6	6	ENSSSCG00000012971	+	3'UTR	*EFEMP2*
2	5579043	5579068	+	5579068	39	72	XLOC_047697	+	predicted	---
2	5646929	5646929	+	5646929	3	3	---	---	intergenic	---

2	5655373	5655373	+	5655373	4	4	ENSSSCG00000012978	+	3'UTR	*AP5B1*
2	5668315	5668320	+	5668319	54	117	ENSSSCG00000012979	+	3'UTR	*RNASEH2C*
2	5668370	5668374	-	5668373	13	42	ENSSSCG00000012980	-	3'UTR	*KAT5*
2	5691572	5691572	-	5691572	15	15	---	---	intergenic	---
2	5695512	5695522	+	5695512	7	10	XLOC_047702	+	predicted	---
2	5698665	5698669	-	5698666	22	46	XLOC_049207	-	predicted	---
2	5771516	5771523	-	5771523	6	14	ENSSSCG00000012983	-	3'UTR	*PCNXL3*
2	5806745	5806787	-	5806749	6	16	XLOC_049210	-	predicted	---
2	5806831	5806849	-	5806831	4	7	XLOC_049210	-	predicted	---
2	5806852	5806852	+	5806852	4	4	XLOC_047704	+	predicted	---
2	5806928	5806945	+	5806945	21	48	XLOC_047704	+	predicted	---
2	5807006	5807033	+	5807006	15	18	XLOC_047704	+	predicted	---
2	5807074	5807083	+	5807074	18	25	XLOC_047704	+	predicted	---
2	5807186	5807199	+	5807186	5	8	XLOC_047704	+	predicted	---
2	5807311	5807392	+	5807378	23	81	XLOC_047704	+	predicted	---
2	5807455	5807455	+	5807455	10	10	XLOC_047704	+	predicted	---
2	5807839	5807840	+	5807840	8	13	XLOC_047704	+	predicted	---
2	5807894	5807894	+	5807894	3	3	XLOC_047704	+	predicted	---
2	5808914	5808914	+	5808914	4	4	XLOC_047704	+	predicted	---
2	5809706	5809706	+	5809706	4	4	XLOC_047704	+	predicted	---
2	5810072	5810072	-	5810072	4	4	XLOC_049210	-	predicted	---
2	5810356	5810356	+	5810356	6	6	XLOC_047704	+	predicted	---
2	5810482	5810484	+	5810484	26	32	XLOC_047704	+	predicted	---
2	5810555	5810556	+	5810556	19	26	XLOC_047704	+	predicted	---
2	5810957	5810957	+	5810957	3	3	XLOC_047704	+	predicted	---
2	5811061	5811061	+	5811061	8	8	XLOC_047704	+	predicted	---

2	5811131	5811136	+	5811131	11	18	XLOC_047704	+	predicted	---
2	5811305	5811305	+	5811305	6	6	XLOC_047704	+	predicted	---
2	5812005	5812009	+	5812005	4	7	XLOC_047704	+	predicted	---
2	5812305	5812312	-	5812305	5	17	XLOC_049210	-	predicted	---
2	5813348	5813348	+	5813348	5	5	XLOC_047704	+	predicted	---
2	5813406	5813406	+	5813406	3	3	XLOC_047704	+	predicted	---
2	5813453	5813458	+	5813453	4	8	XLOC_047704	+	predicted	---
2	5813972	5813972	+	5813972	3	3	XLOC_047704	+	predicted	---
2	5843802	5843802	-	5843802	3	3	ENSSSCG00000012985	-	3'UTR	*LTBP3*
2	5843902	5843902	+	5843902	10	10	ENSSSCG00000012984	+	3'UTR	*SCYL1*
2	5886225	5886232	-	5886226	15	24	XLOC_049213	-	predicted	---
2	5906056	5906068	-	5906066	26	36	ENSSSCG00000020737	-	3'UTR	---
2	5922937	5922937	+	5922937	7	7	XLOC_047709	+	predicted	---
2	5925841	5925844	+	5925841	4	11	XLOC_047709	+	predicted	---
2	5926945	5926945	+	5926945	3	3	XLOC_047709	+	predicted	---
2	5936660	5936660	-	5936660	4	4	XLOC_049215	-	predicted	---
2	5941500	5941517	-	5941511	109	196	XLOC_049215	-	predicted	---
2	5941626	5941626	-	5941626	18	18	XLOC_049215	-	predicted	---
2	5943153	5943154	-	5943154	4	7	XLOC_049215	-	predicted	---
2	5955719	5955719	-	5955719	7	7	ENSSSCG00000012992	-	3'UTR	*FRMD8*
2	5991449	5991449	+	5991449	6	6	XLOC_047710	+	predicted	---
2	6004002	6004005	-	6004005	64	130	ENSSSCG00000012995	-	3'UTR	*DPF2*
2	6030299	6030299	-	6030299	5	5	ENSSSCG00000012996	-	3'UTR	*CDC42EP2*
2	6045730	6045730	-	6045730	3	3	---	---	intergenic	---
2	6054450	6054450	-	6054450	3	3	ENSSSCG00000012997	-	3'UTR	*POLA2*
2	6129550	6129550	-	6129550	21	21	ENSSSCG00000012999	-	3'UTR	*CAPN1*

2	6160183	6160183	-	6160183	4	4	---	---	intergenic	---
2	6198549	6198559	-	6198557	19	38	XLOC_049226	-	predicted	---
2	6198634	6198655	+	6198636	32	90	ENSSSCG00000027057	+	3'UTR	*SYVN1*
2	6204517	6204517	+	6204517	11	11	ENSSSCG00000013002	+	intron	*FAU*
2	6204839	6204850	+	6204842	528	964	ENSSSCG00000013002	+	3'UTR	*FAU*
2	6208674	6208683	+	6208683	11	29	ENSSSCG00000013003	+	3'UTR	*ZNHIT2*
2	6208813	6208822	-	6208815	208	469	ENSSSCG00000013004	-	3'UTR	*TM7SF2*
2	6209735	6209735	-	6209735	7	7	ENSSSCG00000013004	-	ORF	*TM7SF2*
2	6213643	6213648	-	6213645	69	188	ENSSSCG00000013005	-	3'UTR	*VPS51*
2	6231275	6231275	-	6231275	4	4	ENSSSCG00000013007	-	3'UTR	*ZFPL1*
2	6231291	6231296	+	6231291	8	22	ENSSSCG00000013006	+	ORF	*TMEM262*
2	6251943	6251945	+	6251944	6	15	XLOC_047717	+	predicted	---
2	6270456	6270456	-	6270456	10	10	ENSSSCG00000013011	-	3'UTR	*SAC3D1*
2	6270461	6270461	+	6270461	4	4	XLOC_047718	+	predicted	---
2	6290007	6290011	-	6290010	278	452	ENSSSCG00000013013	-	3'UTR	---
2	6290111	6290112	-	6290112	10	15	ENSSSCG00000013013	-	3'UTR	---
2	6321039	6321039	+	6321039	4	4	ENSSSCG00000023178	+	3'UTR	*BATF2*
2	6368085	6368089	+	6368089	4	13	---	---	intergenic	---
2	6375538	6375538	+	6375538	3	3	XLOC_047721	+	predicted	---
2	6379457	6379458	-	6379457	5	8	ENSSSCG00000013016	-	3'UTR	*PPP2R5B*
2	6452836	6452844	+	6452837	11	27	XLOC_047724	+	predicted	---
2	6454129	6454133	+	6454132	27	53	XLOC_047724	+	predicted	---
2	6480111	6480111	+	6480111	3	3	ENSSSCG00000013018	+	3'UTR	*CDC42BPG*
2	6511572	6511572	+	6511572	7	7	ENSSSCG00000013020	+	3'UTR	*MAP4K2*
2	6518481	6518490	-	6518481	4	7	XLOC_049238	-	predicted	---
2	6537527	6537534	+	6537534	9	13	XLOC_047728	+	predicted	---

2	6548198	6548199	+	6548198	15	19	ENSSSCG00000013022	+	ORF	*PYGM*
2	6549447	6549462	+	6549460	6	25	ENSSSCG00000013022	+	intron	*PYGM*
2	6553845	6553867	+	6553847	44	72	XLOC_047729	+	predicted	---
2	6569157	6569157	+	6569157	3	3	ENSSSCG00000013023	+	intron	*RASGRP2*
2	6573066	6573066	+	6573066	3	3	ENSSSCG00000013023	+	3'UTR	*RASGRP2*
2	6846288	6846288	-	6846288	3	3	---	---	intergenic	---
2	6864479	6864497	-	6864487	918	2290	ENSSSCG00000013030	-	3'UTR	*PRDX5*
2	6869325	6869330	-	6869328	32	55	ENSSSCG00000013028	-	3'UTR	*ESRRA*
2	6869363	6869374	+	6869365	215	336	ENSSSCG00000013029	+	3'UTR	*TRMT112*
2	6879439	6879444	-	6879443	21	45	---	---	intergenic	---
2	6880221	6880229	-	6880222	6	26	---	---	intergenic	---
2	6893223	6893229	-	6893224	17	36	ENSSSCG00000013032	-	3'UTR	*GPR137*
2	6909680	6909684	+	6909684	28	49	ENSSSCG00000013033	+	3'UTR	*BAD*
2	6911756	6911756	-	6911756	7	7	ENSSSCG00000013034	-	3'UTR	*PLCB3*
2	6934693	6934693	+	6934693	10	10	ENSSSCG00000013035	+	3'UTR	*PPP1R14B*
2	6934753	6934758	+	6934754	15	24	ENSSSCG00000013035	+	3'UTR	*PPP1R14B*
2	6935110	6935120	-	6935113	187	289	XLOC_049247	-	predicted	---
2	6939764	6939786	-	6939775	20	60	ENSSSCG00000013037	-	3'UTR	*VEGFB*
2	6941513	6941513	-	6941513	10	10	ENSSSCG00000013037	-	intron	*VEGFB*
2	6943343	6943346	-	6943344	38	90	XLOC_049249	-	predicted	---
2	6947820	6947822	-	6947820	8	18	ENSSSCG00000013039	-	3'UTR	*NUDT22*
2	6953557	6953557	-	6953557	14	14	XLOC_049251	-	predicted	---
2	6953730	6953730	+	6953730	4	4	ENSSSCG00000013040	+	3'UTR	*TRPT1*
2	6954936	6954936	+	6954936	3	3	XLOC_047738	+	predicted	---
2	6978504	6978511	-	6978507	46	189	---	---	intergenic	---
2	6982422	6982422	-	6982422	3	3	ENSSSCG00000021620	-	ORF	---

2	6984576	6984576	-	6984576	7	7	ENSSSCG00000021620	-	intron	---
2	6985152	6985152	-	6985152	3	3	ENSSSCG00000021620	-	ORF	---
2	6988729	6988729	-	6988729	4	4	ENSSSCG00000021620	-	ORF	---
2	6993558	6993582	-	6993563	6	30	ENSSSCG00000021620	-	intron	---
2	6993609	6993634	+	6993619	5	24	ENSSSCG00000013042	+	intron	*CCDC88B*
2	6993873	6993889	-	6993889	4	7	ENSSSCG00000021620	-	intron	---
2	7010207	7010207	+	7010207	4	4	ENSSSCG00000013042	+	3'UTR	*CCDC88B*
2	7025875	7025875	+	7025875	3	3	ENSSSCG00000028806	+	3'UTR	---
2	7204580	7204584	+	7204582	106	177	XLOC_047741	+	predicted	---
2	7204713	7204713	-	7204713	5	5	ENSSSCG00000013045	-	3'UTR	*OTUB1*
2	7205409	7205414	-	7205412	155	225	ENSSSCG00000013045	-	3'UTR	*OTUB1*
2	7218507	7218544	-	7218519	215	661	ENSSSCG00000013046	-	3'UTR	---
2	7282388	7282395	-	7282395	6	16	ENSSSCG00000022687	-	3'UTR	*NAA40*
2	7558480	7558480	-	7558480	11	11	XLOC_049261	-	predicted	---
2	7559986	7560020	-	7559996	48	90	ENSSSCG00000013052	-	3'UTR	*RTN3*
2	7638721	7638721	+	7638721	3	3	ENSSSCG00000013054	+	intron	---
2	7645143	7645172	+	7645144	28	87	ENSSSCG00000013054	+	3'UTR	---
2	7649353	7649356	+	7649356	10	27	ENSSSCG00000013054	+	3'UTR	---
2	7665161	7665161	-	7665161	4	4	ENSSSCG00000013052	-	intron	*RTN3*
2	7681374	7681408	+	7681407	45	130	ENSSSCG00000026914	+	3'UTR	*PLA2G16*
2	7683560	7683560	-	7683560	3	3	ENSSSCG00000013052	-	intron	*RTN3*
2	7792628	7792681	+	7792648	39	131	---	---	intergenic	---
2	7804579	7804579	-	7804579	5	5	---	---	intergenic	---
2	7861180	7861182	-	7861180	4	10	---	---	intergenic	---
2	8013060	8013060	-	8013060	3	3	ENSSSCG00000029248	-	intron	---
2	8029633	8029636	-	8029634	10	21	XLOC_049273	-	predicted	---

2	8029690	8029690	-	8029690	10	10	XLOC_049273	-	predicted	---
2	8030989	8030989	-	8030989	7	7	ENSSSCG00000025413	-	intron	---
							ENSSSCG00000025413	-	ORF	---
2	8031039	8031039	-	8031039	10	10	ENSSSCG00000025413	-	ORF	---
2	8235258	8235265	-	8235258	5	8	ENSSSCG00000027405	-	ORF	*U2*
2	8241767	8241775	-	8241775	28	32	XLOC_049278	-	predicted	---
2	8242758	8242759	-	8242758	6	9	ENSSSCG00000023669	-	ORF	*SNORD30*
2	8244166	8244166	-	8244166	12	12	ENSSSCG00000028290	-	ORF	*SNORD27*
2	8273046	8273066	+	8273048	13	35	ENSSSCG00000022404	+	3'UTR	---
2	8282132	8282139	+	8282138	25	43	ENSSSCG00000028025	+	3'UTR	*WDR74*
2	8282736	8282752	+	8282743	43	86	XLOC_047762	+	predicted	---
2	8301425	8301425	+	8301425	3	3	ENSSSCG00000026293	+	intron	*STX5*
2	8313746	8313749	+	8313747	44	61	ENSSSCG00000026293	+	3'UTR	*STX5*
2	8327223	8327226	+	8327225	53	80	ENSSSCG00000022866	+	3'UTR	*NXF1*
2	8328495	8328516	+	8328515	34	143	ENSSSCG00000021431	+	3'UTR	*TMEM223*
2	8349627	8349632	-	8349631	86	147	ENSSSCG00000025571	-	3'UTR	*POLR2G*
2	8369948	8369948	-	8369948	7	7	XLOC_049280	-	predicted	---
2	8392395	8392412	+	8392408	14	36	XLOC_047764	+	predicted	---
2	8394519	8394519	+	8394519	5	5	XLOC_047764	+	predicted	---
2	8398346	8398352	+	8398352	4	7	---	---	intergenic	---
2	8398658	8398658	+	8398658	11	11	---	---	intergenic	---
2	8410622	8410631	+	8410629	25	79	ENSSSCG00000020823	+	3'UTR	*BSCL2*
2	8413379	8413379	+	8413379	5	5	XLOC_047764	+	predicted	---
2	8416535	8416540	+	8416540	7	10	---	---	intergenic	---
2	8421309	8421336	+	8421334	140	324	XLOC_047764	+	predicted	---
2	8422799	8422799	-	8422799	3	3	XLOC_049283	-	predicted	---

2	8423620	8423628	-	8423627	41	118	ENSSSCG00000024995	-	3'UTR	*UQCC3*
2	8435138	8435143	+	8435142	19	43	ENSSSCG00000023323	+	3'UTR	---
2	8435775	8435775	+	8435775	3	3	ENSSSCG00000023323	+	3'UTR	---
2	8438186	8438186	+	8438186	3	3	XLOC_047765	+	predicted	---
2	8446224	8446225	+	8446225	6	9	ENSSSCG00000028275	+	3'UTR	*INTS5*
2	8463089	8463090	+	8463089	5	10	ENSSSCG00000030653	+	3'UTR	*GANAB*
2	8463992	8463994	+	8463993	13	25	ENSSSCG00000030653	+	3'UTR	*GANAB*
2	8470704	8470706	+	8470704	19	22	ENSSSCG00000020809	+	3'UTR	*B3GAT3*
2	8483287	8483289	+	8483287	14	23	XLOC_047768	+	predicted	---
2	8492258	8492258	+	8492258	5	5	ENSSSCG00000013062	+	3'UTR	*MTA2*
2	8492561	8492562	+	8492561	24	27	ENSSSCG00000013062	+	3'UTR	*MTA2*
2	8492616	8492622	+	8492616	14	29	XLOC_047768	+	predicted	---
2	8506614	8506617	+	8506614	6	11	ENSSSCG00000013063	+	3'UTR	---
2	8514025	8514025	+	8514025	5	5	ENSSSCG00000013064	+	intron	*EEF1G*
2	8514416	8514417	+	8514416	6	12	ENSSSCG00000013064	+	ORF	*EEF1G*
2	8514573	8514573	+	8514573	3	3	ENSSSCG00000013064	+	ORF	*EEF1G*
2	8518104	8518119	+	8518112	644	1889	ENSSSCG00000013064	+	3'UTR	*EEF1G*
2	8544550	8544550	+	8544550	3	3	XLOC_047770	+	predicted	---
2	8545786	8545786	+	8545786	3	3	ENSSSCG00000027969	+	ORF	---
2	8546623	8546623	+	8546623	5	5	ENSSSCG00000027969	+	ORF	---
2	8547007	8547007	+	8547007	5	5	ENSSSCG00000027969	+	ORF	---
2	8587354	8587354	+	8587354	3	3	XLOC_047770	+	predicted	---
2	8588782	8588784	+	8588783	95	138	XLOC_047770	+	predicted	---
2	8650759	8650759	-	8650759	15	15	ENSSSCG00000013065	-	3'UTR	*ASRGL1*
2	8651524	8651527	-	8651525	6	16	ENSSSCG00000013065	-	3'UTR	*ASRGL1*
2	8651600	8651615	-	8651614	25	56	ENSSSCG00000013065	-	3'UTR	*ASRGL1*

2	8651706	8651747	-	8651725	108	240	ENSSSCG00000013065	-	3'UTR	*ASRGL1*
2	8665828	8665829	-	8665828	6	9	ENSSSCG00000013065	-	ORF	*ASRGL1*
2	8709273	8709273	-	8709273	4	4	---	---	intergenic	---
2	8740607	8740608	-	8740607	8	12	XLOC_049294	-	predicted	---
2	8975592	8975592	+	8975592	3	3	---	---	intergenic	---
2	8983386	8983387	+	8983386	4	8	ENSSSCG00000014540	+	intron	---
2	8985096	8985096	-	8985096	5	5	XLOC_049295	-	predicted	---
2	8985128	8985196	+	8985171	14	40	ENSSSCG00000014540	+	ORF	---
2	8985581	8985604	-	8985595	14	80	XLOC_049295	-	predicted	---
2	8985599	8985599	+	8985599	5	5	ENSSSCG00000014540	+	ORF	---
2	8985653	8985655	+	8985655	61	76	ENSSSCG00000014540	+	ORF	---
2	8985819	8985819	+	8985819	3	3	ENSSSCG00000014540	+	ORF	---
2	8985850	8985850	-	8985850	3	3	XLOC_049295	-	predicted	---
2	8985895	8985895	-	8985895	4	4	XLOC_049295	-	predicted	---
2	8986030	8986094	+	8986090	6104	11068	ENSSSCG00000014540	+	3'UTR	---
2	8986191	8986196	-	8986194	45	103	XLOC_049295	-	predicted	---
2	9001583	9001583	+	9001583	4	4	XLOC_047775	+	predicted	---
2	9153701	9153701	+	9153701	25	25	XLOC_047779	+	predicted	---
2	9158845	9158845	-	9158845	7	7	ENSSSCG00000013073	-	3'UTR	*FADS3*
2	9186243	9186245	-	9186244	27	37	ENSSSCG00000013074	-	3'UTR	*RAB3IL1*
2	9237628	9237638	-	9237636	839	1346	ENSSSCG00000027538	-	3'UTR	*TMEM258*
2	9245512	9245514	+	9245514	5	11	XLOC_047782	+	predicted	---
2	9247757	9247768	-	9247768	27	36	ENSSSCG00000024015	-	3'UTR	*FADS1*
2	9249974	9249982	-	9249976	48	123	ENSSSCG00000024015	-	3'UTR	*FADS1*
2	9423520	9423520	+	9423520	3	3	---	---	intergenic	---
2	9567226	9567228	-	9567228	5	9	XLOC_049302	-	predicted	---

2	9588888	9588900	-	9588890	136	286	XLOC_049303	-	predicted	---
2	9622385	9622385	-	9622385	5	5	XLOC_049304	-	predicted	---
2	9622546	9622546	-	9622546	3	3	ENSSSCG00000013082	-	3'UTR	---
2	9629381	9629381	-	9629381	3	3	ENSSSCG00000013082	-	ORF	---
2	9659638	9659647	+	9659639	17	24	XLOC_047790	+	predicted	---
2	9659711	9659712	+	9659711	3	6	XLOC_047790	+	predicted	---
2	9662386	9662388	-	9662388	4	7	ENSSSCG00000013084	-	3'UTR	*TMEM216*
2	9667209	9667211	-	9667211	5	8	ENSSSCG00000013084	-	intron	*TMEM216*
2	9682244	9682244	-	9682244	6	6	ENSSSCG00000013085	-	3'UTR	*TMEM138*
2	9704327	9704334	+	9704327	9	13	XLOC_047791	+	predicted	---
2	9705518	9705525	-	9705520	31	58	ENSSSCG00000013087	-	3'UTR	*DAK*
2	9749426	9749439	+	9749432	43	79	XLOC_047792	+	predicted	---
2	9774618	9774618	+	9774618	3	3	ENSSSCG00000013093	+	intron	*VWCE*
2	9786159	9786165	+	9786159	7	33	ENSSSCG00000013093	+	3'UTR	*VWCE*
2	9973910	9973910	-	9973910	11	11	---	---	intergenic	---
2	10074002	10074002	+	10074002	7	7	---	---	intergenic	---
2	10079704	10079704	-	10079704	4	4	---	---	intergenic	---
2	10079736	10079736	+	10079736	15	15	---	---	intergenic	---
2	10167657	10167662	-	10167662	11	18	ENSSSCG00000028762	-	3'UTR	*VPS37C*
2	10240965	10240965	-	10240965	3	3	ENSSSCG00000013111	-	3'UTR	*CD6*
2	10251314	10251340	-	10251314	7	17	ENSSSCG00000013115	-	3'UTR	*CD5*
							ENSSSCG00000013111	-	intron	*CD6*
2	10352004	10352004	-	10352004	5	5	ENSSSCG00000013114	-	3'UTR	*SLC15A3*
2	10352093	10352093	-	10352093	12	12	ENSSSCG00000013114	-	3'UTR	*SLC15A3*
2	10372980	10372992	-	10372992	9	22	ENSSSCG00000013110	-	3'UTR	*TMEM109*
2	10457016	10457016	-	10457016	4	4	ENSSSCG00000013107	-	3'UTR	*PRPF19*

2	10466281	10466283	-	10466281	3	6	XLOC_049320	-	predicted	---
2	10489169	10489173	+	10489171	11	20	ENSSSCG00000013105	+	3'UTR	*CCDC86*
2	10583948	10583961	-	10583949	65	202	XLOC_049324	-	predicted	---
2	10603796	10603796	+	10603796	4	4	---	---	intergenic	---
2	10605585	10605588	+	10605585	16	22	XLOC_047806	+	predicted	---
2	10636754	10636754	+	10636754	3	3	---	---	intergenic	---
2	10643309	10643315	+	10643313	270	554	XLOC_047807	+	predicted	---
2	10659296	10659296	+	10659296	4	4	XLOC_047807	+	predicted	---
2	10659358	10659358	+	10659358	5	5	---	---	intergenic	---
2	10700879	10700880	+	10700879	5	9	XLOC_047807	+	predicted	---
2	10774633	10774638	-	10774633	12	26	ENSSSCG00000021812	-	3'UTR	*MS4A1*
2	10792927	10792927	-	10792927	4	4	XLOC_049329	-	predicted	---
2	10793061	10793091	-	10793067	122	256	XLOC_049329	-	predicted	---
2	10805985	10805985	-	10805985	4	4	ENSSSCG00000026796	-	ORF	*MS4A5*
2	10817020	10817020	-	10817020	3	3	ENSSSCG00000026796	-	ORF	*MS4A5*
2	10828355	10828362	-	10828357	56	108	XLOC_049330	-	predicted	---
2	10829479	10829479	-	10829479	4	4	ENSSSCG00000022114	-	ORF	*MS4A14*
2	10829983	10829983	-	10829983	3	3	ENSSSCG00000022114	-	ORF	*MS4A14*
2	10830447	10830447	-	10830447	6	6	ENSSSCG00000022114	-	ORF	*MS4A14*
2	10847925	10847925	-	10847925	3	3	ENSSSCG00000022114	-	ORF	*MS4A14*
2	10854383	10854383	-	10854383	5	5	XLOC_049331	-	predicted	---
2	10855775	10855775	-	10855775	3	3	ENSSSCG00000013102	-	3'UTR	*MS4A7*
2	10927136	10927136	+	10927136	5	5	XLOC_047809	+	predicted	---
2	10942816	10942822	+	10942822	7	13	ENSSSCG00000013100	+	3'UTR	---
2	10959658	10959664	-	10959658	5	8	XLOC_049332	-	predicted	---
2	11072622	11072622	+	11072622	3	3	ENSSSCG00000013119	+	intron	*STX3*

2	11088780	11088786	-	11088781	29	55	ENSSSCG00000013118	-	3'UTR	*MRPL16*
2	11319485	11319485	+	11319485	20	20	ENSSSCG00000013124	+	intron	*PATL1*
2	11331797	11331798	+	11331798	10	13	ENSSSCG00000013124	+	3'UTR	*PATL1*
2	11333203	11333203	+	11333203	6	6	---	---	intergenic	---
2	11385588	11385597	+	11385596	16	25	ENSSSCG00000013125	+	3'UTR	*OSBP*
2	11471863	11471873	-	11471863	7	12	---	---	intergenic	---
2	11811354	11811354	+	11811354	13	13	ENSSSCG00000013144	+	3'UTR	*MPEG1*
							ENSSSCG00000013144	+	ORF	---
2	11811572	11811572	+	11811572	28	28	ENSSSCG00000013144	+	3'UTR	*MPEG1*
							ENSSSCG00000013144	+	ORF	---
2	11812238	11812238	-	11812238	6	6	ENSSSCG00000013145	-	3'UTR	*DTX4*
2	11863586	11863586	-	11863586	20	20	XLOC_049358	-	predicted	---
2	11875312	11875312	+	11875312	3	3	---	---	intergenic	---
2	11995051	11995064	-	11995058	108	317	XLOC_049362	-	predicted	---
2	11995215	11995223	-	11995223	6	9	XLOC_049362	-	predicted	---
2	11995360	11995367	-	11995360	40	43	XLOC_049362	-	predicted	---
2	11995469	11995469	-	11995469	3	3	XLOC_049362	-	predicted	---
2	12070864	12070868	+	12070867	48	140	XLOC_047829	+	predicted	---
2	12133496	12133505	-	12133496	11	19	XLOC_049364	-	predicted	---
2	12134709	12134710	-	12134709	13	24	XLOC_049364	-	predicted	---
2	12134789	12134790	-	12134790	23	28	XLOC_049364	-	predicted	---
2	12135668	12135668	-	12135668	5	5	XLOC_049364	-	predicted	---
2	12135754	12135757	-	12135757	6	11	XLOC_049364	-	predicted	---
2	12136326	12136326	-	12136326	3	3	XLOC_049364	-	predicted	---
2	12137977	12137994	-	12137994	22	39	XLOC_049364	-	predicted	---
2	12141929	12141929	-	12141929	4	4	ENSSSCG00000013155	-	intron	---

2	12143250	12143251	-	12143250	5	8	ENSSSCG00000013155	-	intron	---
2	12144978	12144987	-	12144978	9	16	ENSSSCG00000013155	-	intron	---
2	12302167	12302167	-	12302167	3	3	---	---	intergenic	---
2	12511425	12511425	+	12511425	4	4	XLOC_047845	+	predicted	---
2	12789448	12789451	-	12789450	15	35	ENSSSCG00000013174	-	3'UTR	*CTNND1*
2	12791192	12791192	-	12791192	4	4	ENSSSCG00000013174	-	3'UTR	*CTNND1*
2	12791559	12791559	-	12791559	4	4	ENSSSCG00000013174	-	3'UTR	*CTNND1*
2	12845545	12845545	+	12845545	11	11	---	---	intergenic	---
2	12863472	12863480	-	12863480	38	114	ENSSSCG00000013176	-	3'UTR	---
2	12884154	12884164	-	12884163	15	33	XLOC_049379	-	predicted	---
2	12892903	12892903	-	12892903	5	5	ENSSSCG00000013178	-	intron	*ZDHHC5*
2	12915349	12915357	-	12915350	10	27	XLOC_049380	-	predicted	---
2	12957674	12957719	-	12957677	1441	3421	XLOC_049382	-	predicted	---
2	12959302	12959302	-	12959302	4	4	ENSSSCG00000013181	-	3'UTR	*C1-INH*
2	12965485	12965521	-	12965485	5	8	ENSSSCG00000013181	-	3'UTR	*C1-INH*
2	12967701	12967701	-	12967701	18	18	ENSSSCG00000013181	-	intron	*C1-INH*
							ENSSSCG00000013181	-	ORF	*C1-INH*
2	12970444	12970448	-	12970448	12	18	---	---	intergenic	---
2	12970572	12970592	-	12970592	8	11	---	---	intergenic	---
2	13002860	13002860	+	13002860	4	4	ENSSSCG00000023379	+	3'UTR	*UBE2L6*
2	13003121	13003130	+	13003129	22	63	XLOC_047861	+	predicted	---
2	13076042	13076043	-	13076042	30	36	---	---	intergenic	---
2	13390287	13390287	+	13390287	14	14	ENSSSCG00000029059	+	intron	*SSRP1*
2	13392023	13392033	+	13392027	108	170	ENSSSCG00000029059	+	3'UTR	*SSRP1*
2	13419726	13419726	+	13419726	6	6	ENSSSCG00000028527	+	3'UTR	*TNKS1BP1*
2	14573360	14573360	-	14573360	5	5	---	---	intergenic	---

2	15254182	15254182	-	15254182	3	3	---	---	intergenic	---
2	15624152	15624152	-	15624152	3	3	---	---	intergenic	---
2	15632256	15632257	-	15632256	12	15	XLOC_049426	-	predicted	---
2	15634389	15634389	-	15634389	5	5	ENSSSCG00000023709	-	3'UTR	*PTPRJ*
2	15652984	15652984	-	15652984	4	4	ENSSSCG00000023709	-	intron	*PTPRJ*
2	15969754	15969754	-	15969754	4	4	---	---	intergenic	---
2	16009814	16009814	-	16009814	4	4	---	---	intergenic	---
2	16091745	16091745	+	16091745	3	3	ENSSSCG00000023418	+	intron	---
2	16110435	16110438	+	16110436	8	24	ENSSSCG00000030610	+	3'UTR	---
2	16120266	16120266	+	16120266	3	3	ENSSSCG00000013225	+	3'UTR	---
2	16148421	16148424	+	16148422	30	48	XLOC_047900	+	predicted	---
2	16180443	16180456	+	16180447	248	429	ENSSSCG00000013232	+	3'UTR	*MTCH2*
2	16181743	16181755	+	16181746	36	96	XLOC_047899	+	predicted	---
2	16220381	16220387	+	16220387	18	24	ENSSSCG00000013227	+	3'UTR	*KBTBD4*
2	16220999	16221004	-	16221002	14	39	ENSSSCG00000013226	-	3'UTR	*PTPMT1*
2	16221057	16221058	-	16221057	13	18	ENSSSCG00000013226	-	3'UTR	*PTPMT1*
2	16311079	16311089	-	16311087	9	26	XLOC_049434	-	predicted	---
2	16311384	16311384	-	16311384	3	3	XLOC_049434	-	predicted	---
2	16313587	16313587	-	16313587	9	9	XLOC_049434	-	predicted	---
2	16394165	16394165	+	16394165	5	5	ENSSSCG00000013239	+	intron	*PSMC3*
2	16397824	16397838	+	16397829	147	746	ENSSSCG00000013239	+	3'UTR	*PSMC3*
2	16398334	16398334	+	16398334	3	3	ENSSSCG00000013239	+	3'UTR	*PSMC3*
2	16400675	16400675	-	16400675	4	4	ENSSSCG00000013238	-	3'UTR	*SLC39A13*
2	16401716	16401724	-	16401716	3	9	ENSSSCG00000013238	-	3'UTR	*SLC39A13*
2	16452506	16452517	+	16452506	31	43	ENSSSCG00000013237	+	3'UTR	*SPI1*
2	16476407	16476407	+	16476407	5	5	ENSSSCG00000013236	+	3'UTR	*MYBPC3*

2	16565466	16565466	+	16565466	3	3	ENSSSCG00000013242	+	3'UTR	*ACP2*
2	16566095	16566102	+	16566095	25	79	ENSSSCG00000013242	+	3'UTR	*ACP2*
2	16566180	16566186	-	16566181	20	32	ENSSSCG00000013243	-	3'UTR	---
2	16624710	16624711	-	16624711	19	24	XLOC_049439	-	predicted	---
2	16624768	16624786	+	16624786	19	47	ENSSSCG00000013245	+	3'UTR	---
2	16626156	16626161	-	16626158	25	43	---	---	intergenic	---
2	16802934	16802939	-	16802936	6	14	ENSSSCG00000013246	-	intron	---
2	16803273	16803273	-	16803273	3	3	ENSSSCG00000013246	-	intron	---
2	16925826	16925830	-	16925826	10	19	ENSSSCG00000028351	-	intron	---
2	17130476	17130476	+	17130476	3	3	ENSSSCG00000013249	+	intron	---
2	17141841	17141841	+	17141841	6	6	ENSSSCG00000013249	+	ORF	---
2	17142312	17142319	+	17142312	14	29	XLOC_047914	+	predicted	---
2	17144993	17145022	-	17145004	693	1577	ENSSSCG00000013252	-	3'UTR	*F2*
2	17145194	17145194	-	17145194	7	7	ENSSSCG00000013252	-	ORF	*F2*
2	17161118	17161138	-	17161127	58	445	ENSSSCG00000013252	-	intron	*F2*
2	17164004	17164004	-	17164004	5	5	ENSSSCG00000013252	-	ORF	*F2*
2	17164711	17164711	-	17164711	6	6	ENSSSCG00000013252	-	ORF	*F2*
2	17180517	17180518	-	17180518	7	10	XLOC_049444	-	predicted	---
2	17203974	17203979	+	17203976	9	32	ENSSSCG00000013256	+	3'UTR	*ARHGAP1*
2	17205200	17205207	+	17205200	28	38	ENSSSCG00000013256	+	3'UTR	*ARHGAP1*
2	17207884	17207888	-	17207886	31	52	ENSSSCG00000013257	-	3'UTR	*ATG13*
2	17210038	17210042	-	17210041	6	18	ENSSSCG00000013257	-	3'UTR	*ATG13*
2	17214201	17214201	-	17214201	4	4	ENSSSCG00000013257	-	intron	*ATG13*
2	17259578	17259578	-	17259578	5	5	ENSSSCG00000013257	-	intron	*ATG13*
2	17326092	17326098	+	17326096	99	213	ENSSSCG00000013262	+	intron	*AMBRA1*
2	17449151	17449152	+	17449151	3	6	ENSSSCG00000013262	+	intron	*AMBRA1*

2	17465126	17465127	+	17465126	8	11	ENSSSCG00000013262	+	3'UTR	*AMBRA1*
2	17476858	17476858	-	17476858	7	7	ENSSSCG00000013260	-	3'UTR	*MDK*
2	17480092	17480098	-	17480098	13	39	ENSSSCG00000013259	-	3'UTR	*DGKZ*
2	17600166	17600166	+	17600166	3	3	---	---	intergenic	---
2	17926672	17926676	+	17926672	9	24	XLOC_047928	+	predicted	---
2	17949514	17949519	+	17949514	4	8	ENSSSCG00000013269	+	3'UTR	*MAPK8IP1*
2	17949585	17949594	-	17949592	130	348	XLOC_049454	-	predicted	---
2	17952995	17953002	-	17952996	58	137	XLOC_049455	-	predicted	---
2	17971663	17971664	-	17971663	3	6	ENSSSCG00000023974	-	3'UTR	---
2	17972842	17972842	-	17972842	4	4	ENSSSCG00000023974	-	3'UTR	---
2	17975677	17975677	-	17975677	6	6	ENSSSCG00000023974	-	intron	---
							ENSSSCG00000023974	-	ORF	---
2	18178344	18178344	-	18178344	3	3	XLOC_049459	-	predicted	---
2	18182702	18182707	+	18182703	10	26	---	---	intergenic	---
2	18253566	18253574	+	18253573	33	58	XLOC_047932	+	predicted	---
2	18456507	18456508	+	18456507	4	7	---	---	intergenic	---
2	18456608	18456608	+	18456608	4	4	---	---	intergenic	---
2	18456666	18456677	+	18456666	3	6	---	---	intergenic	---
2	18645737	18645737	-	18645737	3	3	XLOC_049461	-	predicted	---
2	18923928	18923937	-	18923928	4	7	XLOC_049465	-	predicted	---
2	19139838	19139838	-	19139838	3	3	---	---	intergenic	---
2	19194616	19194616	+	19194616	5	5	---	---	intergenic	---
2	19196579	19196579	-	19196579	6	6	---	---	intergenic	---
2	19220980	19220980	+	19220980	3	3	---	---	intergenic	---
2	19243736	19243736	-	19243736	3	3	---	---	intergenic	---
2	19243756	19243757	+	19243756	6	10	---	---	intergenic	---

2	19263923	19263932	+	19263923	24	58	ENSSSCG00000029521	+	3'UTR	*CD82*
2	19437978	19437978	+	19437978	5	5	---	---	intergenic	---
2	19572349	19572349	+	19572349	4	4	---	---	intergenic	---
2	19709058	19709074	-	19709058	4	15	ENSSSCG00000013281	-	3'UTR	*EXT2*
2	19718862	19718862	-	19718862	3	3	ENSSSCG00000013281	-	intron	*EXT2*
2	19745110	19745110	-	19745110	4	4	ENSSSCG00000013281	-	intron	*EXT2*
2	19858838	19858838	-	19858838	4	4	ENSSSCG00000013282	-	3'UTR	*ACCS*
2	19959387	19959387	-	19959387	4	4	---	---	intergenic	---
2	19963243	19963243	+	19963243	12	12	---	---	intergenic	---
2	19986093	19986101	+	19986099	19	53	---	---	intergenic	---
2	20003075	20003075	-	20003075	3	3	---	---	intergenic	---
2	20009062	20009062	-	20009062	6	6	XLOC_049473	-	predicted	---
2	20011988	20011989	-	20011988	7	10	ENSSSCG00000025731	-	3'UTR	*ALKBH3*
2	20012127	20012134	-	20012131	16	44	ENSSSCG00000025731	-	3'UTR	*ALKBH3*
2	20061560	20061573	-	20061561	16	66	XLOC_049474	-	predicted	---
2	20152321	20152321	-	20152321	6	6	ENSSSCG00000021739	-	intron	*HSD17B12*
2	20316539	20316539	+	20316539	3	3	---	---	intergenic	---
2	20401324	20401324	+	20401324	4	4	---	---	intergenic	---
2	20459973	20459977	-	20459976	8	15	ENSSSCG00000013284	-	3'UTR	*TTC17*
2	20537571	20537583	-	20537571	3	6	ENSSSCG00000013284	-	intron	*TTC17*
2	20614006	20614006	-	20614006	3	3	ENSSSCG00000013284	-	intron	*TTC17*
2	20614709	20614709	-	20614709	3	3	ENSSSCG00000013284	-	intron	*TTC17*
2	20650699	20650700	-	20650699	3	6	ENSSSCG00000013284	-	intron	*TTC17*
2	20666704	20666712	-	20666712	27	60	XLOC_049481	-	predicted	---
2	20797820	20797820	+	20797820	3	3	---	---	intergenic	---
2	22225915	22225928	-	22225926	37	131	---	---	intergenic	---

2	22225941	22225956	+	22225954	7	24	---	---	intergenic	---
2	23003629	23003630	+	23003629	6	9	---	---	intergenic	---
2	23354012	23354012	-	23354012	9	9	---	---	intergenic	---
2	23928400	23928400	+	23928400	4	4	---	---	intergenic	---
2	23931092	23931092	-	23931092	3	3	XLOC_049488	-	predicted	---
2	23937199	23937199	-	23937199	4	4	XLOC_049489	-	predicted	---
2	24176121	24176121	-	24176121	5	5	XLOC_049488	-	predicted	---
2	24226791	24226791	-	24226791	3	3	---	---	intergenic	---
2	24287990	24287992	-	24287992	11	24	---	---	intergenic	---
2	24442601	24442601	-	24442601	3	3	---	---	intergenic	---
2	24621873	24621881	-	24621881	4	7	---	---	intergenic	---
2	24641311	24641311	-	24641311	3	3	---	---	intergenic	---
2	24750577	24750577	+	24750577	3	3	XLOC_047958	+	predicted	---
2	24909198	24909198	+	24909198	8	8	---	---	intergenic	---
2	25978608	25978608	+	25978608	4	4	---	---	intergenic	---
2	26590438	26590438	+	26590438	3	3	---	---	intergenic	---
2	26789688	26789689	+	26789688	9	12	XLOC_047962	+	predicted	---
2	27138699	27138705	+	27138699	43	86	ENSSSCG00000013293	+	3'UTR	*COMMD9*
2	27175374	27175376	-	27175374	7	12	XLOC_049495	-	predicted	---
2	27379145	27379155	-	27379148	5	18	ENSSSCG00000013294	-	intron	*LDLRAD3*
2	27491214	27491215	-	27491214	42	55	---	---	intergenic	---
2	27491240	27491240	+	27491240	4	4	---	---	intergenic	---
2	27558508	27558508	-	27558508	3	3	---	---	intergenic	---
2	27558560	27558560	+	27558560	3	3	---	---	intergenic	---
2	27598315	27598315	+	27598315	3	3	---	---	intergenic	---
2	27636878	27636878	-	27636878	6	6	XLOC_049496	-	predicted	---

2	27663657	27663657	-	27663657	3	3	ENSSSCG00000013295	-	intron	*TRIM44*
2	27768137	27768139	+	27768137	36	49	---	---	intergenic	---
2	27918703	27918703	+	27918703	10	10	ENSSSCG00000028338	+	intron	*PAMR1*
2	27954880	27954881	+	27954880	3	6	ENSSSCG00000028338	+	3'UTR	*PAMR1*
2	28045749	28045749	-	28045749	4	4	---	---	intergenic	---
2	28136628	28136634	+	28136634	13	16	XLOC_047969	+	predicted	---
2	28154041	28154041	-	28154041	5	5	XLOC_049502	-	predicted	---
2	28156391	28156393	-	28156392	61	67	ENSSSCG00000013297	-	3'UTR	*CD44*
2	28156464	28156471	-	28156471	15	29	ENSSSCG00000013297	-	3'UTR	*CD44*
2	28478634	28478634	+	28478634	5	5	ENSSSCG00000013299	+	3'UTR	*APIP*
2	28482428	28482433	+	28482432	15	31	ENSSSCG00000013299	+	3'UTR	*APIP*
2	28654346	28654346	-	28654346	3	3	XLOC_049507	-	predicted	---
2	28817605	28817606	-	28817605	23	26	---	---	intergenic	---
2	28852489	28852502	+	28852490	18	50	ENSSSCG00000013301	+	3'UTR	*ELF5*
2	28852566	28852569	+	28852568	11	25	XLOC_047972	+	predicted	---
2	28860862	28860959	-	28860869	27	94	ENSSSCG00000013302	-	3'UTR	*CAT*
2	28861030	28861030	-	28861030	6	6	ENSSSCG00000013302	-	3'UTR	*CAT*
2	28861107	28861107	-	28861107	3	3	ENSSSCG00000013302	-	3'UTR	*CAT*
2	28861185	28861201	-	28861201	46	75	ENSSSCG00000013302	-	3'UTR	*CAT*
2	28861408	28861408	-	28861408	3	3	ENSSSCG00000013302	-	3'UTR	*CAT*
2	28861474	28861474	-	28861474	9	9	ENSSSCG00000013302	-	3'UTR	*CAT*
2	28862185	28862187	-	28862185	26	34	ENSSSCG00000013302	-	ORF	*CAT*
2	28869255	28869255	-	28869255	3	3	ENSSSCG00000013302	-	ORF	*CAT*
2	28876741	28876741	-	28876741	6	6	ENSSSCG00000013302	-	ORF	*CAT*
2	28883742	28883743	-	28883742	22	26	ENSSSCG00000013302	-	ORF	*CAT*
2	28885152	28885152	-	28885152	20	20	ENSSSCG00000013302	-	ORF	*CAT*

2	29004736	29004737	+	29004736	21	29	ENSSSCG00000013303	+	intron	*ABTB2*
2	29149539	29149546	+	29149539	11	18	ENSSSCG00000013303	+	3'UTR	*ABTB2*
2	29153542	29153548	-	29153546	8	14	ENSSSCG00000013304	-	3'UTR	*NAT10*
2	29197666	29197666	-	29197666	3	3	ENSSSCG00000013305	-	3'UTR	*CAPRIN1*
2	29199142	29199143	-	29199142	16	23	ENSSSCG00000013305	-	3'UTR	*CAPRIN1*
2	29199776	29199791	-	29199791	8	14	ENSSSCG00000013305	-	3'UTR	*CAPRIN1*
2	29200757	29200782	-	29200767	7	33	ENSSSCG00000013305	-	3'UTR	*CAPRIN1*
2	29213214	29213214	-	29213214	4	4	ENSSSCG00000013305	-	ORF	*CAPRIN1*
2	29224758	29224758	-	29224758	4	4	ENSSSCG00000013305	-	intron	*CAPRIN1*
2	29422797	29422797	+	29422797	15	15	ENSSSCG00000013307	+	3'UTR	*LMO2*
2	29480163	29480165	+	29480164	11	29	XLOC_047981	+	predicted	---
2	29518338	29518338	+	29518338	3	3	ENSSSCG00000025765	+	ORF	*FBXO3*
2	29768738	29768739	-	29768738	7	11	XLOC_049517	-	predicted	---
2	29768800	29768802	-	29768800	7	14	XLOC_049517	-	predicted	---
2	29778077	29778083	-	29778077	3	6	ENSSSCG00000024791	-	3'UTR	*CD59*
2	29778371	29778371	-	29778371	4	4	ENSSSCG00000024791	-	3'UTR	*CD59*
2	29800876	29800876	-	29800876	8	8	ENSSSCG00000013308	-	3'UTR	*FBXO3*
2	29801076	29801076	-	29801076	3	3	ENSSSCG00000013308	-	3'UTR	*FBXO3*
2	29954581	29954582	+	29954581	3	6	---	---	intergenic	---
2	29998385	29998385	+	29998385	7	7	---	---	intergenic	---
2	30028375	30028379	-	30028376	34	52	ENSSSCG00000013312	-	3'UTR	*HIPK3*
2	30031753	30031763	-	30031760	9	24	ENSSSCG00000013312	-	3'UTR	*HIPK3*
2	30289303	30289303	+	30289303	9	9	XLOC_047988	+	predicted	---
2	30344706	30344707	+	30344706	7	10	---	---	intergenic	---
2	30344770	30344772	+	30344772	14	17	---	---	intergenic	---
2	30395325	30395325	+	30395325	5	5	ENSSSCG00000030326	+	3'UTR	*TCP11L1*

2	30395374	30395381	+	30395374	3	6	ENSSSCG00000030326	+	3'UTR	*TCP11L1*
2	30624359	30624359	-	30624359	5	5	---	---	intergenic	---
2	30746636	30746645	-	30746636	7	13	ENSSSCG00000013315	-	3'UTR	*EIF3M*
2	30748560	30748573	-	30748567	58	122	ENSSSCG00000013315	-	3'UTR	*EIF3M*
2	30947391	30947391	-	30947391	3	3	---	---	intergenic	---
2	30976818	30976818	-	30976818	4	4	XLOC_049527	-	predicted	---
2	31194686	31194687	+	31194687	25	31	ENSSSCG00000024950	+	intron	---
2	31201516	31201527	-	31201525	41	73	ENSSSCG00000013318	-	3'UTR	*RCN1*
2	31513497	31513497	-	31513497	8	8	XLOC_049529	-	predicted	---
2	31662642	31662644	-	31662642	11	25	---	---	intergenic	---
2	31715938	31715938	-	31715938	4	4	ENSSSCG00000013321	-	intron	*ELP4*
2	31813002	31813002	-	31813002	7	7	ENSSSCG00000026083	-	3'UTR	*DNAJC24*
2	31813218	31813221	+	31813218	67	96	ENSSSCG00000013322	+	3'UTR	*IMMP1L*
2	31909690	31909690	+	31909690	3	3	ENSSSCG00000013324	+	intron	---
2	32860738	32860738	-	32860738	16	16	ENSSSCG00000013328	-	3'UTR	*FSHB*
2	32895694	32895694	-	32895694	4	4	---	---	intergenic	---
2	33512535	33512535	+	33512535	4	4	---	---	intergenic	---
2	33938339	33938339	-	33938339	7	7	---	---	intergenic	---
2	34995919	34995919	+	34995919	4	4	---	---	intergenic	---
2	35028795	35028795	+	35028795	6	6	---	---	intergenic	---
2	35125872	35125872	-	35125872	3	3	---	---	intergenic	---
2	35222627	35222627	+	35222627	3	3	---	---	intergenic	---
2	35346903	35346903	+	35346903	3	3	---	---	intergenic	---
2	35357904	35357904	-	35357904	3	3	---	---	intergenic	---
2	35357939	35357943	+	35357939	7	12	---	---	intergenic	---
2	35435234	35435235	+	35435234	3	6	ENSSSCG00000022806	+	3'UTR	*LIN7C*

2	35436493	35436493	+	35436493	3	3	ENSSSCG00000022806	+	3'UTR	*LIN7C*
2	35436735	35436735	+	35436735	3	3	ENSSSCG00000022806	+	3'UTR	*LIN7C*
2	35437801	35437801	+	35437801	4	4	ENSSSCG00000022806	+	3'UTR	*LIN7C*
2	35438345	35438352	+	35438345	11	18	ENSSSCG00000022806	+	3'UTR	*LIN7C*
2	35438855	35438855	+	35438855	5	5	ENSSSCG00000022806	+	3'UTR	*LIN7C*
2	35468266	35468266	+	35468266	3	3	ENSSSCG00000013335	+	intron	*LGR4*
2	35614276	35614282	+	35614276	4	7	XLOC_048012	+	predicted	---
2	35641798	35641798	+	35641798	3	3	---	---	intergenic	---
2	35656384	35656396	+	35656384	3	6	XLOC_048014	+	predicted	---
2	35864233	35864234	-	35864234	12	15	XLOC_049542	-	predicted	---
2	35864383	35864387	-	35864384	7	20	ENSSSCG00000013337	-	3'UTR	---
2	35864571	35864572	-	35864571	3	6	ENSSSCG00000013337	-	3'UTR	---
2	36215325	36215325	+	36215325	3	3	ENSSSCG00000013338	+	3'UTR	*SLC5A12*
2	36912603	36912603	+	36912603	18	18	---	---	intergenic	---
2	37567891	37567931	-	37567927	78	135	ENSSSCG00000013341	-	3'UTR	*LUZP2*
2	37914320	37914327	-	37914320	5	10	ENSSSCG00000013341	-	intron	*LUZP2*
2	38375209	38375210	+	38375210	4	7	---	---	intergenic	---
2	38382899	38382899	-	38382899	5	5	---	---	intergenic	---
2	38725968	38725968	-	38725968	7	7	---	---	intergenic	---
2	38903083	38903083	-	38903083	3	3	---	---	intergenic	---
2	39481634	39481634	-	39481634	3	3	---	---	intergenic	---
2	39481651	39481651	+	39481651	6	6	---	---	intergenic	---
2	39674895	39674895	-	39674895	4	4	---	---	intergenic	---
2	39932954	39932954	+	39932954	5	5	---	---	intergenic	---
2	39933018	39933018	+	39933018	4	4	---	---	intergenic	---
2	39935212	39935212	-	39935212	7	7	XLOC_049551	-	predicted	---

2	39947325	39947365	-	39947327	115	257	XLOC_049551	-	predicted	---
2	40097326	40097326	+	40097326	6	6	XLOC_048027	+	predicted	---
2	40101127	40101128	+	40101127	4	7	XLOC_048027	+	predicted	---
2	40382291	40382291	-	40382291	3	3	ENSSSCG00000013344	-	3'UTR	*ANO5*
2	40629183	40629183	-	40629183	22	22	---	---	intergenic	---
2	40899759	40899759	-	40899759	11	11	---	---	intergenic	---
2	41038238	41038238	-	41038238	3	3	---	---	intergenic	---
2	41112145	41112145	-	41112145	5	5	---	---	intergenic	---
2	41231021	41231039	-	41231038	21	114	---	---	intergenic	---
2	41611776	41611777	-	41611777	10	14	---	---	intergenic	---
2	41611821	41611821	+	41611821	3	3	---	---	intergenic	---
2	41641500	41641500	+	41641500	4	4	---	---	intergenic	---
2	41980414	41980416	+	41980416	11	14	ENSSSCG00000013349	+	3'UTR	*HTATIP2*
2	41991590	41991590	+	41991590	6	6	---	---	intergenic	---
2	42054198	42054198	-	42054198	20	20	---	---	intergenic	---
2	42072159	42072159	-	42072159	7	7	---	---	intergenic	---
2	42100808	42100808	-	42100808	4	4	---	---	intergenic	---
2	42525523	42525523	-	42525523	7	7	---	---	intergenic	---
2	42527161	42527161	-	42527161	3	3	---	---	intergenic	---
2	42838413	42838424	+	42838424	4	7	---	---	intergenic	---
2	42848390	42848390	+	42848390	3	3	---	---	intergenic	---
2	42892089	42892089	-	42892089	3	3	---	---	intergenic	---
2	43342311	43342327	+	43342327	25	31	ENSSSCG00000021688	+	intron	---
2	43342386	43342386	+	43342386	13	13	ENSSSCG00000021688	+	intron	---
2	43555006	43555009	-	43555008	34	46	ENSSSCG00000013360	-	3'UTR	*TMEM86A*
2	43577361	43577361	+	43577361	3	3	---	---	intergenic	---

2	43588695	43588695	-	43588695	5	5	---	---	intergenic	---
2	43604264	43604264	+	43604264	6	6	ENSSSCG00000026892	+	intron	---
2	43611160	43611160	+	43611160	3	3	ENSSSCG00000013361	+	3'UTR	---
2	43611640	43611641	+	43611640	6	9	ENSSSCG00000013361	+	3'UTR	---
2	43614254	43614263	+	43614255	4	13	ENSSSCG00000013361	+	3'UTR	---
2	43616650	43616715	-	43616709	463	946	---	---	intergenic	---
2	43617980	43617980	+	43617980	4	4	XLOC_048051	+	predicted	---
2	43619720	43619728	-	43619724	49	188	XLOC_049568	-	predicted	---
2	43619876	43619876	-	43619876	4	4	XLOC_049568	-	predicted	---
2	43692050	43692050	+	43692050	3	3	ENSSSCG00000013362	+	intron	*UEVLD*
							ENSSSCG00000013363	+	ORF	*LDHC*
2	43723997	43724005	+	43724004	11	23	ENSSSCG00000013363	+	intron	*LDHC*
							ENSSSCG00000013362	+	intron	*UEVLD*
2	43730834	43730844	+	43730836	58	266	ENSSSCG00000013362	+	intron	*UEVLD*
2	43759513	43759517	-	43759514	53	122	ENSSSCG00000013364	-	3'UTR	---
2	43909308	43909309	+	43909309	11	15	ENSSSCG00000013366	+	3'UTR	*LDHA*
2	43909447	43909461	+	43909455	266	420	ENSSSCG00000013366	+	3'UTR	*LDHA*
2	43921524	43921527	-	43921525	6	20	ENSSSCG00000013367	-	3'UTR	*GTF2H1*
2	43928112	43928112	-	43928112	3	3	ENSSSCG00000013367	-	ORF	*GTF2H1*
2	43945902	43945902	-	43945902	3	3	ENSSSCG00000013367	-	intron	*GTF2H1*
2	44002715	44002715	+	44002715	10	10	ENSSSCG00000013368	+	3'UTR	*HPS5*
2	44004083	44004083	+	44004083	3	3	ENSSSCG00000013368	+	3'UTR	*HPS5*
2	44039635	44039664	+	44039654	208	730	XLOC_048058	+	predicted	---
2	44046278	44046290	+	44046290	4	7	---	---	intergenic	---
2	44059261	44059268	+	44059266	241	551	XLOC_048059	+	predicted	---
2	44063246	44063246	+	44063246	3	3	XLOC_048059	+	predicted	---

2	44082938	44082950	+	44082944	177	220	ENSSSCG00000028525	+	3'UTR	---
2	44108796	44108805	+	44108797	9	38	ENSSSCG00000013371	+	3'UTR	*SAAL1*
2	44142329	44142329	+	44142329	3	3	ENSSSCG00000013372	+	intron	*TPH1*
2	44172786	44172786	+	44172786	5	5	XLOC_048061	+	predicted	---
2	44173720	44173720	-	44173720	4	4	---	---	intergenic	---
2	44301832	44301833	+	44301833	26	47	ENSSSCG00000013373	+	intron	*SERGEF*
2	44417154	44417158	+	44417157	15	40	ENSSSCG00000013373	+	3'UTR	*SERGEF*
2	44840117	44840143	-	44840143	6	10	ENSSSCG00000013380	-	3'UTR	*NUCB2*
2	44910020	44910020	+	44910020	6	6	---	---	intergenic	---
2	45079395	45079398	+	45079398	5	9	XLOC_048070	+	predicted	---
2	45086945	45086945	+	45086945	6	6	ENSSSCG00000013381	+	ORF	*RPS13*
2	45087625	45087634	+	45087633	143	251	ENSSSCG00000013381	+	3'UTR	*RPS13*
2	45291120	45291123	+	45291120	5	8	ENSSSCG00000013382	+	intron	*PLEKHA7*
2	45402821	45402821	+	45402821	3	3	ENSSSCG00000013382	+	intron	*PLEKHA7*
2	45411573	45411573	+	45411573	4	4	ENSSSCG00000013382	+	3'UTR	*PLEKHA7*
2	45426667	45426672	-	45426672	4	10	XLOC_049583	-	predicted	---
2	45426739	45426747	-	45426742	51	135	ENSSSCG00000013383	-	3'UTR	*C11orf58*
2	45426809	45426818	-	45426810	23	29	ENSSSCG00000013383	-	3'UTR	*C11orf58*
2	45426913	45426913	-	45426913	3	3	ENSSSCG00000013383	-	3'UTR	*C11orf58*
2	45427007	45427010	-	45427009	5	12	ENSSSCG00000013383	-	3'UTR	*C11orf58*
2	45427048	45427048	-	45427048	3	3	ENSSSCG00000013383	-	3'UTR	*C11orf58*
2	45445223	45445223	-	45445223	5	5	ENSSSCG00000013383	-	intron	*C11orf58*
2	45459559	45459562	-	45459559	6	12	ENSSSCG00000013383	-	ORF	*C11orf58*
2	46107808	46107809	+	46107809	8	13	ENSSSCG00000013384	+	intron	---
2	46133098	46133098	+	46133098	8	8	ENSSSCG00000013384	+	intron	---
2	46206832	46206835	+	46206832	4	8	ENSSSCG00000030418	+	3'UTR	---

2	46206922	46206922	+	46206922	3	3	XLOC_048077	+	predicted	---
2	46213214	46213214	+	46213214	7	7	XLOC_048077	+	predicted	---
2	46450098	46450103	+	46450099	67	85	---	---	intergenic	---
2	46672626	46672626	+	46672626	8	8	---	---	intergenic	---
2	46883657	46883657	-	46883657	13	13	---	---	intergenic	---
2	46957209	46957209	+	46957209	3	3	---	---	intergenic	---
2	47472792	47472815	+	47472792	3	6	ENSSSCG00000013387	+	3'UTR	*CRSP2*
2	47566771	47566771	-	47566771	3	3	---	---	intergenic	---
2	47606211	47606211	+	47606211	3	3	ENSSSCG00000013388	+	intron	---
2	47652667	47652667	+	47652667	3	3	ENSSSCG00000021819	+	3'UTR	---
2	47653512	47653512	+	47653512	8	8	ENSSSCG00000021819	+	3'UTR	---
2	47973107	47973107	+	47973107	3	3	ENSSSCG00000013391	+	ORF	---
2	47980918	47980918	+	47980918	3	3	ENSSSCG00000013391	+	3'UTR	---
2	47981197	47981204	+	47981199	65	142	ENSSSCG00000013391	+	3'UTR	---
2	47987954	47987956	+	47987955	11	21	ENSSSCG00000013392	+	ORF	*COPB1*
2	47993405	47993405	+	47993405	4	4	ENSSSCG00000013392	+	intron	*COPB1*
2	47993546	47993546	+	47993546	4	4	ENSSSCG00000013392	+	ORF	*COPB1*
2	48078671	48078718	+	48078672	29	85	ENSSSCG00000021317	+	3'UTR	---
2	48089283	48089283	-	48089283	3	3	ENSSSCG00000013393	-	3'UTR	*SPON1*
2	48090246	48090246	-	48090246	7	7	ENSSSCG00000013393	-	3'UTR	*SPON1*
2	48712164	48712164	-	48712164	3	3	---	---	intergenic	---
2	48832670	48832670	-	48832670	3	3	ENSSSCG00000021170	-	3'UTR	*FAR1*
2	48835215	48835232	-	48835225	27	89	ENSSSCG00000021170	-	3'UTR	*FAR1*
2	48859610	48859610	-	48859610	6	6	ENSSSCG00000021170	-	ORF	*FAR1*
2	49103302	49103302	+	49103302	7	7	---	---	intergenic	---
2	49163333	49163333	-	49163333	14	14	ENSSSCG00000013395	-	3'UTR	*BTBD10*

2	49305034	49305034	+	49305034	3	3	ENSSSCG00000013396	+	intron	*ARNTL*
2	49387404	49387413	-	49387408	5	15	---	---	intergenic	---
2	49820440	49820440	-	49820440	3	3	XLOC_049613	-	predicted	---
2	50265944	50265944	-	50265944	3	3	XLOC_049615	-	predicted	---
2	50266717	50266717	-	50266717	9	9	XLOC_049615	-	predicted	---
2	50268373	50268373	-	50268373	3	3	XLOC_049615	-	predicted	---
2	50511892	50511892	-	50511892	7	7	ENSSSCG00000021777	-	intron	---
2	50758469	50758472	+	50758469	9	12	ENSSSCG00000013401	+	3'UTR	*DKK3*
2	50758555	50758562	+	50758562	10	20	XLOC_048098	+	predicted	---
2	50764924	50764960	-	50764960	5	8	ENSSSCG00000013402	-	3'UTR	---
2	50765914	50765914	-	50765914	4	4	ENSSSCG00000013402	-	3'UTR	---
2	50766300	50766303	-	50766302	9	20	ENSSSCG00000013402	-	ORF	---
2	50800181	50800181	-	50800181	3	3	ENSSSCG00000013402	-	ORF	---
2	50918487	50918495	+	50918487	8	16	---	---	intergenic	---
2	50970120	50970120	+	50970120	6	6	---	---	intergenic	---
2	51205904	51205904	-	51205904	7	7	---	---	intergenic	---
2	51234701	51234701	-	51234701	3	3	---	---	intergenic	---
2	51429562	51429562	+	51429562	4	4	ENSSSCG00000013403	+	intron	*GALNT18*
2	51976891	51976893	+	51976893	8	11	XLOC_048105	+	predicted	---
2	52003452	52003460	+	52003453	77	150	XLOC_048105	+	predicted	---
2	52012681	52012683	-	52012682	5	12	---	---	intergenic	---
2	52018315	52018351	+	52018351	19	27	ENSSSCG00000013405	+	intron	*EIF4G2*
2	52023053	52023053	+	52023053	4	4	ENSSSCG00000013405	+	ORF	*EIF4G2*
2	52025973	52025973	+	52025973	4	4	XLOC_048105	+	predicted	---
2	52047108	52047114	-	52047114	33	50	ENSSSCG00000013406	-	3'UTR	*CTR9*
2	52053171	52053171	-	52053171	3	3	ENSSSCG00000013406	-	ORF	*CTR9*

2	52059209	52059210	-	52059209	4	7	ENSSSCG00000013406	-	ORF	*CTR9*
2	52238073	52238073	+	52238073	3	3	ENSSSCG00000020720	+	intron	*MRVI1*
2	52301176	52301181	+	52301181	4	7	ENSSSCG00000029886	+	3'UTR	*LYVE1*
2	52331760	52331773	-	52331770	37	81	---	---	intergenic	---
2	52373754	52373757	+	52373755	35	75	ENSSSCG00000027983	+	3'UTR	*RNF141*
2	52376207	52376212	+	52376207	5	8	ENSSSCG00000027983	+	3'UTR	*RNF141*
2	52376468	52376469	+	52376469	15	18	XLOC_048109	+	predicted	---
2	52417438	52417438	-	52417438	3	3	ENSSSCG00000022492	-	ORF	*AMPD3*
2	52534389	52534389	+	52534389	20	20	---	---	intergenic	---
2	52576216	52576216	-	52576216	3	3	ENSSSCG00000013408	-	3'UTR	*ADM*
2	52717732	52717732	+	52717732	4	4	ENSSSCG00000028260	+	intron	---
2	53041004	53041005	+	53041005	6	9	XLOC_048112	+	predicted	---
2	53070345	53070346	-	53070345	4	7	ENSSSCG00000013410	-	3'UTR	*SWAP70*
2	53176445	53176445	+	53176445	6	6	---	---	intergenic	---
2	53226463	53226463	-	53226463	4	4	---	---	intergenic	---
2	53229797	53229798	-	53229797	7	10	ENSSSCG00000013411	-	3'UTR	*WEE1*
2	53271795	53271803	-	53271796	35	83	XLOC_049636	-	predicted	---
2	53284254	53284255	-	53284255	21	28	XLOC_049636	-	predicted	---
2	53353000	53353000	-	53353000	3	3	---	---	intergenic	---
2	53398181	53398181	-	53398181	7	7	---	---	intergenic	---
2	53824579	53824593	-	53824586	11	33	ENSSSCG00000013988	-	3'UTR	*IBA57*
2	53854709	53854709	-	53854709	4	4	ENSSSCG00000013990	-	3'UTR	---
2	53873178	53873180	+	53873178	8	11	ENSSSCG00000013991	+	3'UTR	*C1orf35*
2	53874546	53874567	-	53874567	62	201	ENSSSCG00000013992	-	3'UTR	*ARF1*
2	53874645	53874651	-	53874650	36	52	ENSSSCG00000013992	-	3'UTR	*ARF1*
2	53874793	53874793	-	53874793	10	10	ENSSSCG00000013992	-	3'UTR	*ARF1*

2	54124090	54124090	-	54124090	5	5	XLOC_049649	-	predicted	---
2	54250037	54250037	+	54250037	3	3	---	---	intergenic	---
2	54307423	54307423	-	54307423	5	5	ENSSSCG00000013984	-	3'UTR	*SNAP47*
2	54307784	54307784	-	54307784	3	3	ENSSSCG00000013984	-	ORF	*SNAP47*
2	54319544	54319544	-	54319544	12	12	ENSSSCG00000013984	-	intron	*SNAP47*
2	54396547	54396547	-	54396547	3	3	ENSSSCG00000013984	-	intron	*SNAP47*
2	54420069	54420076	+	54420076	5	16	ENSSSCG00000013983	+	3'UTR	*JMJD4*
2	54871000	54871000	-	54871000	3	3	---	---	intergenic	---
2	55158593	55158594	+	55158593	11	15	---	---	intergenic	---
2	55183320	55183343	-	55183330	256	1173	XLOC_049653	-	predicted	---
2	55183430	55183439	-	55183439	4	7	XLOC_049653	-	predicted	---
2	55183480	55183517	-	55183480	18	32	XLOC_049653	-	predicted	---
2	55183599	55183599	-	55183599	4	4	XLOC_049653	-	predicted	---
2	55196951	55196957	+	55196956	5	13	ENSSSCG00000030672	+	3'UTR	*ZNF692*
2	55197472	55197475	-	55197474	20	40	ENSSSCG00000013978	-	3'UTR	*ZNF672*
2	55200755	55200761	-	55200761	21	31	ENSSSCG00000013978	-	intron	*ZNF672*
2	55207871	55207877	-	55207876	11	30	ENSSSCG00000013978	-	intron	*ZNF672*
2	55236996	55237003	+	55237002	8	19	ENSSSCG00000025900	+	3'UTR	*SH3BP5L*
2	55702733	55702733	-	55702733	3	3	---	---	intergenic	---
2	56435568	56435568	-	56435568	3	3	XLOC_049664	-	predicted	---
2	57278606	57278606	+	57278606	3	3	---	---	intergenic	---
2	57807028	57807028	-	57807028	3	3	---	---	intergenic	---
2	57893944	57893944	-	57893944	4	4	---	---	intergenic	---
2	58049613	58049613	+	58049613	3	3	---	---	intergenic	---
2	58279733	58279733	+	58279733	4	4	ENSSSCG00000013938	+	3'UTR	*GATAD2A*
2	58280233	58280239	+	58280234	25	82	ENSSSCG00000013938	+	3'UTR	*GATAD2A*

2	58280570	58280572	+	58280572	4	7	ENSSSCG00000013938	+	3'UTR	*GATAD2A*
2	58282240	58282240	+	58282240	9	9	ENSSSCG00000013938	+	3'UTR	*GATAD2A*
2	58285594	58285610	-	58285606	244	497	XLOC_049676	-	predicted	---
2	58285828	58285828	-	58285828	5	5	XLOC_049676	-	predicted	---
2	58285929	58285929	-	58285929	6	6	XLOC_049676	-	predicted	---
2	58296447	58296457	+	58296455	68	188	ENSSSCG00000013936	+	3'UTR	*NDUFA13*
2	58310933	58310933	+	58310933	8	8	ENSSSCG00000013934	+	ORF	*CILP2*
2	58314433	58314435	+	58314434	14	23	ENSSSCG00000013934	+	3'UTR	*CILP2*
2	58334088	58334089	-	58334089	31	40	ENSSSCG00000013933	-	3'UTR	*PBX4*
2	58334389	58334397	-	58334395	9	33	ENSSSCG00000013933	-	3'UTR	*PBX4*
2	58393980	58393980	-	58393980	8	8	ENSSSCG00000013932	-	3'UTR	*LPAR2*
2	58399772	58399774	-	58399772	6	9	ENSSSCG00000013931	-	3'UTR	*GMIP*
2	58410651	58410654	-	58410654	14	17	ENSSSCG00000013930	-	3'UTR	*ATP13A1*
2	58606516	58606523	-	58606523	8	18	ENSSSCG00000013927	-	3'UTR	*ARMC6*
2	58636673	58636673	+	58636673	3	3	ENSSSCG00000025839	+	ORF	*SUGP2*
2	58664570	58664577	+	58664570	3	6	XLOC_048165	+	predicted	---
2	58664665	58664678	+	58664671	46	86	XLOC_048165	+	predicted	---
2	58721277	58721277	+	58721277	4	4	ENSSSCG00000013919	+	3'UTR	*HOMER3*
2	58721788	58721794	-	58721794	16	40	ENSSSCG00000025329	-	3'UTR	*DDX49*
2	58747254	58747269	+	58747254	35	78	ENSSSCG00000027840	+	3'UTR	---
2	58823287	58823293	+	58823290	88	219	ENSSSCG00000013916	+	3'UTR	*COMP*
2	58920091	58920091	-	58920091	6	6	XLOC_049685	-	predicted	---
2	58965377	58965377	-	58965377	4	4	ENSSSCG00000013911	-	3'UTR	*TMEM59L*
2	59035637	59035641	-	59035640	15	25	ENSSSCG00000013905	-	3'UTR	*FKBP8*
2	59060490	59060504	+	59060496	59	106	ENSSSCG00000013906	+	3'UTR	*KXD1*
2	59062609	59062617	+	59062617	15	19	ENSSSCG00000013907	+	ORF	*UBA52*

2	59062972	59062972	+	59062972	8	8	ENSSSCG00000013907	+	ORF	*UBA52*
2	59063970	59064005	+	59064002	2128	6674	ENSSSCG00000013907	+	3'UTR	*UBA52*
2	59068469	59068480	+	59068469	45	86	ENSSSCG00000013908	+	3'UTR	*C19orf60*
2	59149128	59149133	-	59149128	21	36	ENSSSCG00000013896	-	3'UTR	*MPV17L2*
2	59157071	59157076	+	59157074	66	120	XLOC_048177	+	predicted	---
2	59163301	59163301	+	59163301	3	3	XLOC_048177	+	predicted	---
2	59194623	59194623	+	59194623	4	4	ENSSSCG00000029924	+	3'UTR	---
2	59217992	59218008	+	59218005	39	140	ENSSSCG00000013901	+	3'UTR	*IFI30*
2	59278534	59278534	+	59278534	3	3	ENSSSCG00000013894	+	intron	*IL12RB1*
2	59339838	59339838	-	59339838	5	5	ENSSSCG00000013893	-	3'UTR	*ARRDC2*
2	59390287	59390294	-	59390289	61	133	ENSSSCG00000013891	-	3'UTR	*CCDC124*
2	59462218	59462253	-	59462236	1438	2488	ENSSSCG00000013889	-	3'UTR	*RPL18A*
2	59495928	59495929	+	59495929	9	12	ENSSSCG00000013888	+	3'UTR	*JAK3*
2	59500529	59500529	+	59500529	4	4	ENSSSCG00000013887	+	3'UTR	*INSL3*
2	59500643	59500656	+	59500651	633	1635	ENSSSCG00000013887	+	3'UTR	*INSL3*
2	59504384	59504391	-	59504384	7	19	ENSSSCG00000013886	-	3'UTR	*B3GNT3*
2	59610013	59610015	+	59610013	21	25	ENSSSCG00000013884	+	3'UTR	*MAP1S*
2	59771658	59771658	+	59771658	4	4	XLOC_048192	+	predicted	---
2	59787011	59787020	+	59787013	46	75	ENSSSCG00000013878	+	3'UTR	*PGLS*
2	59808130	59808134	-	59808130	8	14	XLOC_049711	-	predicted	---
2	59856524	59856525	-	59856524	10	14	XLOC_049713	-	predicted	---
2	59946804	59946804	-	59946804	8	8	ENSSSCG00000013871	-	3'UTR	*HAUS8*
2	59968519	59968519	-	59968519	7	7	ENSSSCG00000013871	-	intron	*HAUS8*
2	60010852	60010852	+	60010852	4	4	ENSSSCG00000013870	+	intron	---
2	60077951	60077954	+	60077953	29	41	ENSSSCG00000013876	+	3'UTR	---
2	60083127	60083135	+	60083129	30	102	XLOC_048199	+	predicted	---

2	60091328	60091328	+	60091328	9	9	ENSSSCG00000013874	+	3'UTR	---
2	60091381	60091381	+	60091381	5	5	ENSSSCG00000013874	+	3'UTR	---
2	60095382	60095386	-	60095383	29	77	ENSSSCG00000013873	-	3'UTR	*NR2F6*
2	60181097	60181097	-	60181097	3	3	---	---	intergenic	---
2	60181124	60181125	+	60181125	5	9	---	---	intergenic	---
2	60314312	60314312	-	60314312	6	6	XLOC_049720	-	predicted	---
2	60335317	60335317	-	60335317	3	3	ENSSSCG00000013867	-	ORF	*SIN3B*
2	60406731	60406731	-	60406731	37	37	---	---	intergenic	---
2	60517876	60517886	-	60517876	5	8	ENSSSCG00000013864	-	3'UTR	*TMEM38A*
2	60556715	60556715	+	60556715	4	4	XLOC_048202	+	predicted	---
2	60556933	60556933	+	60556933	4	4	XLOC_048202	+	predicted	---
2	60557244	60557244	+	60557244	3	3	XLOC_048202	+	predicted	---
2	60557580	60557590	+	60557581	78	108	XLOC_048202	+	predicted	---
2	60569638	60569656	-	60569646	111	277	---	---	intergenic	---
2	60665993	60665993	+	60665993	19	19	XLOC_048203	+	predicted	---
2	60759090	60759091	+	60759090	6	10	ENSSSCG00000013858	+	ORF	*CALR3*
2	60767058	60767107	+	60767106	86	215	ENSSSCG00000013858	+	3'UTR	*CALR3*
2	60796432	60796433	+	60796432	24	33	---	---	intergenic	---
2	60798742	60798742	+	60798742	3	3	ENSSSCG00000013857	+	intron	*EPS15L1*
2	60870444	60870444	+	60870444	3	3	ENSSSCG00000013857	+	intron	*EPS15L1*
2	60903979	60903979	-	60903979	6	6	XLOC_049727	-	predicted	---
2	60909582	60909582	+	60909582	5	5	---	---	intergenic	---
2	60915491	60915491	+	60915491	4	4	---	---	intergenic	---
2	60988669	60988696	-	60988693	44	137	ENSSSCG00000013856	-	3'UTR	*AP1M1*
2	61020924	61020924	-	61020924	23	23	ENSSSCG00000013855	-	3'UTR	*FAM32A*
2	61022005	61022015	-	61022012	69	176	ENSSSCG00000013855	-	3'UTR	*FAM32A*

2	61026197	61026197	-	61026197	3	3	ENSSSCG00000013855	-	ORF	*FAM32A*
2	61026430	61026437	-	61026436	9	24	ENSSSCG00000013855	-	ORF	*FAM32A*
2	61046889	61046897	+	61046890	4	14	XLOC_048205	+	predicted	---
2	61050328	61050333	-	61050333	5	8	ENSSSCG00000013853	-	3'UTR	*HSH2D*
2	61165717	61165717	+	61165717	3	3	ENSSSCG00000013849	+	intron	*TPM4*
2	61173496	61173496	+	61173496	12	12	XLOC_048208	+	predicted	---
2	61173564	61173574	+	61173573	74	104	XLOC_048208	+	predicted	---
2	61195970	61195970	+	61195970	11	11	ENSSSCG00000013848	+	3'UTR	*RAB8A*
2	61196615	61196618	+	61196615	17	32	ENSSSCG00000013848	+	3'UTR	*RAB8A*
2	61197777	61197777	+	61197777	3	3	XLOC_048208	+	predicted	---
2	61344296	61344296	-	61344296	17	17	ENSSSCG00000013842	-	3'UTR	---
2	61491655	61491657	-	61491656	20	28	XLOC_049736	-	predicted	---
2	61510196	61510196	+	61510196	11	11	ENSSSCG00000027013	+	intron	---
2	61567651	61567655	-	61567652	34	64	ENSSSCG00000025560	-	3'UTR	*PGLYRP2*
2	61637029	61637029	-	61637029	5	5	---	---	intergenic	---
2	61729122	61729122	+	61729122	8	8	ENSSSCG00000024211	+	3'UTR	---
2	61757100	61757100	+	61757100	4	4	ENSSSCG00000013836	+	3'UTR	*WIZ*
2	61773987	61773987	+	61773987	7	7	ENSSSCG00000013838	+	intron	*AKAP8L*
2	61775706	61775706	+	61775706	3	3	ENSSSCG00000013838	+	ORF	*AKAP8L*
2	61797258	61797259	+	61797258	13	17	XLOC_048221	+	predicted	---
2	61817845	61817845	+	61817845	4	4	XLOC_048221	+	predicted	---
2	61832081	61832081	+	61832081	20	20	ENSSSCG00000022227	+	intron	*BRD4*
2	61889974	61889974	+	61889974	4	4	ENSSSCG00000022227	+	intron	*BRD4*
2	61912300	61912300	+	61912300	3	3	ENSSSCG00000022227	+	intron	*BRD4*
2	61912391	61912411	+	61912401	8	38	ENSSSCG00000022227	+	intron	*BRD4*
2	61923455	61923456	+	61923455	17	23	ENSSSCG00000022227	+	3'UTR	*BRD4*

2	61930727	61930728	+	61930727	6	9	XLOC_048223	+	predicted	---
2	61963141	61963141	+	61963141	8	8	ENSSSCG00000025176	+	3'UTR	---
2	62005406	62005406	+	62005406	4	4	ENSSSCG00000013830	+	ORF	*ILVBL*
2	62008030	62008056	+	62008053	48	143	ENSSSCG00000013830	+	3'UTR	*ILVBL*
2	62008056	62008056	-	62008056	3	3	ENSSSCG00000013829	-	3'UTR	*SYDE1*
2	62129932	62129943	-	62129939	15	42	---	---	intergenic	---
2	63632910	63632910	-	63632910	3	3	---	---	intergenic	---
2	64146547	64146547	+	64146547	4	4	---	---	intergenic	---
2	64299586	64299587	-	64299586	4	7	---	---	intergenic	---
2	64308028	64308031	+	64308029	161	292	---	---	intergenic	---
2	65115617	65115623	+	65115623	26	53	ENSSSCG00000013775	+	3'UTR	*CD97*
2	65115714	65115729	-	65115725	61	159	ENSSSCG00000013776	-	3'UTR	*DDX39A*
2	65115745	65115745	+	65115745	4	4	ENSSSCG00000013775	+	3'UTR	*CD97*
2	65154602	65154608	+	65154604	95	154	ENSSSCG00000013783	+	3'UTR	---
2	65166355	65166355	-	65166355	3	3	---	---	intergenic	---
2	65176571	65176574	-	65176573	78	104	XLOC_049783	-	predicted	---
2	65177569	65177585	-	65177584	155	708	ENSSSCG00000013784	-	3'UTR	*DNAJB1*
2	65178589	65178589	-	65178589	5	5	ENSSSCG00000013784	-	ORF	*DNAJB1*
2	65189568	65189568	-	65189568	6	6	---	---	intergenic	---
2	65191192	65191205	+	65191198	248	670	ENSSSCG00000013780	+	3'UTR	*TECR*
2	65191260	65191273	-	65191269	160	236	---	---	intergenic	---
2	65348211	65348218	+	65348211	15	29	ENSSSCG00000013772	+	3'UTR	*ASF1B*
2	65371225	65371238	+	65371227	22	29	ENSSSCG00000013771	+	3'UTR	*PRKACA*
2	65378330	65378336	+	65378330	10	32	XLOC_048264	+	predicted	---
2	65381882	65381912	-	65381890	66	131	ENSSSCG00000013768	-	3'UTR	---
2	65401272	65401272	+	65401272	4	4	XLOC_048265	+	predicted	---

2	65401430	65401430	-	65401430	4	4	ENSSSCG00000013766	-	3'UTR	*IL27RA*
2	65431747	65431747	-	65431747	3	3	---	---	intergenic	---
2	65442575	65442576	-	65442576	10	15	XLOC_049793	-	predicted	---
2	65447914	65447918	+	65447914	59	63	---	---	intergenic	---
2	65452417	65452417	+	65452417	3	3	---	---	intergenic	---
2	65452601	65452601	+	65452601	4	4	---	---	intergenic	---
2	65453090	65453090	-	65453090	5	5	XLOC_049794	-	predicted	---
2	65479695	65479695	+	65479695	7	7	ENSSSCG00000013764	+	3'UTR	*RFX1*
2	65479798	65479804	-	65479804	4	7	ENSSSCG00000013763	-	3'UTR	*DCAF15*
2	65483345	65483345	-	65483345	3	3	ENSSSCG00000013763	-	intron	*DCAF15*
2	65509481	65509481	-	65509481	6	6	ENSSSCG00000013761	-	3'UTR	*CC2D1A*
2	65546751	65546752	+	65546752	7	10	XLOC_048268	+	predicted	---
2	65583040	65583040	+	65583040	9	9	XLOC_048270	+	predicted	---
2	65611000	65611000	+	65611000	3	3	---	---	intergenic	---
2	65675862	65675863	-	65675862	5	10	ENSSSCG00000013756	-	3'UTR	*MRI1*
2	65683165	65683173	-	65683171	64	175	ENSSSCG00000013755	-	3'UTR	*CCDC130*
2	65687249	65687252	-	65687250	13	38	ENSSSCG00000013755	-	ORF	*CCDC130*
2	65687497	65687497	-	65687497	6	6	ENSSSCG00000013755	-	intron	*CCDC130*
2	65920820	65920820	+	65920820	3	3	XLOC_048273	+	predicted	---
2	66210159	66210159	-	66210159	22	22	XLOC_049804	-	predicted	---
2	66217578	66217583	+	66217580	12	44	ENSSSCG00000013752	+	3'UTR	*STX10*
2	66262229	66262235	+	66262229	6	18	ENSSSCG00000013751	+	3'UTR	*NACC1*
2	66270997	66270997	-	66270997	4	4	ENSSSCG00000013745	-	3'UTR	*FARSA*
2	66271545	66271547	-	66271547	71	101	ENSSSCG00000013745	-	3'UTR	*FARSA*
2	66282824	66282824	-	66282824	3	3	XLOC_049810	-	predicted	---
2	66282864	66282864	-	66282864	6	6	XLOC_049810	-	predicted	---

2	66286008	66286008	+	66286008	4	4	ENSSSCG00000013746	+	ORF	*CALR*
2	66286719	66286722	+	66286722	26	44	ENSSSCG00000013746	+	intron	*CALR*
2	66286873	66286899	+	66286873	17	23	ENSSSCG00000013746	+	ORF	*CALR*
2	66287089	66287089	+	66287089	3	3	ENSSSCG00000013746	+	3'UTR	*CALR*
2	66287155	66287155	+	66287155	6	6	ENSSSCG00000013746	+	3'UTR	*CALR*
2	66287601	66287638	+	66287604	212	433	XLOC_048278	+	predicted	---
2	66290141	66290141	+	66290141	3	3	ENSSSCG00000013747	+	intron	*RAD23A*
2	66295769	66295776	+	66295776	12	17	XLOC_048279	+	predicted	---
2	66296296	66296306	-	66296297	15	28	XLOC_049811	-	predicted	---
2	66314599	66314603	+	66314600	13	27	ENSSSCG00000026775	+	3'UTR	---
2	66320080	66320080	+	66320080	3	3	ENSSSCG00000013743	+	3'UTR	---
2	66321715	66321715	+	66321715	3	3	XLOC_048282	+	predicted	---
2	66403710	66403710	-	66403710	5	5	---	---	intergenic	---
2	66447516	66447518	-	66447516	62	74	ENSSSCG00000013731	-	3'UTR	*DNASE2*
2	66466915	66466922	-	66466916	8	23	ENSSSCG00000013740	-	intron	*SYCE2*
2	66478110	66478110	+	66478110	3	3	XLOC_048286	+	predicted	---
2	66493833	66493834	+	66493834	4	7	XLOC_048287	+	predicted	---
2	66507022	66507023	+	66507022	51	54	ENSSSCG00000013735	+	3'UTR	*JUNB*
							ENSSSCG00000013735	+	ORF	*havana*
2	66509636	66509661	-	66509638	245	602	ENSSSCG00000013736	-	3'UTR	*PRDX2*
2	66522534	66522541	+	66522536	12	36	ENSSSCG00000013737	+	3'UTR	*RNASEH2A*
2	66631986	66631986	-	66631986	3	3	ENSSSCG00000013728	-	3'UTR	*ASNA1*
2	66632153	66632161	-	66632154	17	37	ENSSSCG00000013728	-	3'UTR	*ASNA1*
2	66646023	66646027	+	66646026	42	92	ENSSSCG00000013727	+	3'UTR	*C19orf43*
2	66667946	66667947	+	66667947	17	26	ENSSSCG00000013726	+	3'UTR	*TNPO2*
2	66676398	66676406	+	66676398	12	35	ENSSSCG00000013725	+	3'UTR	*FBXW9*

2	66686816	66686817	+	66686817	15	20	ENSSSCG00000013723	+	ORF	*DHPS*
2	66686845	66686848	-	66686847	12	25	ENSSSCG00000013722	-	3'UTR	*WDR83*
2	66686956	66686966	+	66686963	37	82	ENSSSCG00000013723	+	3'UTR	*DHPS*
2	66705724	66705748	+	66705731	166	446	ENSSSCG00000013721	+	3'UTR	*WDR83OS*
2	66724770	66724789	+	66724788	18	39	XLOC_048296	+	predicted	---
2	66776376	66776376	-	66776376	3	3	XLOC_049824	-	predicted	---
2	66920355	66920356	-	66920356	4	7	---	---	intergenic	---
2	67059674	67059674	+	67059674	12	12	---	---	intergenic	---
2	67231515	67231515	+	67231515	5	5	ENSSSCG00000013714	+	intron	*MUC16*
2	67239465	67239465	+	67239465	4	4	ENSSSCG00000013714	+	3'UTR	*MUC16*
2	67651467	67651467	-	67651467	7	7	---	---	intergenic	---
2	67679932	67679932	+	67679932	3	3	ENSSSCG00000022610	+	intron	*ZNF317*
2	67690114	67690116	+	67690116	8	14	ENSSSCG00000022610	+	3'UTR	*ZNF317*
2	67806326	67806326	+	67806326	4	4	---	---	intergenic	---
2	68422729	68422729	-	68422729	3	3	---	---	intergenic	---
2	68431995	68432040	-	68432001	21	64	---	---	intergenic	---
2	68432107	68432144	-	68432131	39	187	---	---	intergenic	---
2	68432218	68432219	-	68432218	3	6	---	---	intergenic	---
2	68603685	68603690	+	68603685	46	77	XLOC_048333	+	predicted	---
2	68649840	68649840	-	68649840	4	4	ENSSSCG00000022738	-	intron	---
2	68753067	68753067	-	68753067	3	3	ENSSSCG00000023397	-	intron	---
2	68753456	68753456	-	68753456	5	5	ENSSSCG00000023397	-	ORF	---
2	68753632	68753633	-	68753633	4	7	ENSSSCG00000023397	-	ORF	---
2	68920487	68920487	-	68920487	13	13	XLOC_049857	-	predicted	---
2	68925624	68925624	-	68925624	7	7	ENSSSCG00000013670	-	intron	*FBXL12*
2	69156494	69156494	-	69156494	3	3	XLOC_049860	-	predicted	---

2	69242115	69242115	+	69242115	5	5	ENSSSCG00000013665	+	ORF	---
2	69248933	69248943	+	69248940	38	73	ENSSSCG00000013665	+	3'UTR	---
2	69275242	69275245	-	69275243	21	34	XLOC_049862	-	predicted	---
2	69275276	69275282	+	69275276	28	35	ENSSSCG00000013664	+	3'UTR	*C19orf66*
2	69290829	69290836	+	69290836	17	27	ENSSSCG00000013662	+	3'UTR	*PPAN*
2	69294399	69294410	-	69294402	217	624	ENSSSCG00000013661	-	3'UTR	*EIF3G*
2	69294404	69294405	+	69294404	7	13	ENSSSCG00000024072	+	3'UTR	*P2RY11*
2	69298536	69298536	+	69298536	6	6	---	---	intergenic	---
2	69318582	69318582	-	69318582	3	3	ENSSSCG00000013659	-	ORF	*DNMT1*
2	69344659	69344662	-	69344659	5	8	ENSSSCG00000013659	-	intron	*DNMT1*
2	69417246	69417260	+	69417246	29	39	ENSSSCG00000013656	+	3'UTR	*MRPL4*
2	69459113	69459114	-	69459113	8	14	ENSSSCG00000013651	-	3'UTR	*FDX1L*
2	69464123	69464126	-	69464126	44	51	XLOC_049867	-	predicted	---
2	69477412	69477421	-	69477412	37	45	XLOC_049867	-	predicted	---
2	69477713	69477725	+	69477724	32	123	ENSSSCG00000013655	+	intron	*ICAM-1*
2	69498278	69498278	+	69498278	3	3	ENSSSCG00000013655	+	intron	*ICAM-1*
2	69527516	69527516	+	69527516	4	4	ENSSSCG00000013655	+	intron	*ICAM-1*
2	69544289	69544289	+	69544289	5	5	ENSSSCG00000013655	+	intron	*ICAM-1*
							ENSSSCG00000013647	+	ORF	*CDC37*
2	69544386	69544390	+	69544386	52	90	ENSSSCG00000013647	+	3'UTR	*CDC37*
							ENSSSCG00000013655	+	intron	*ICAM-1*
2	69544712	69544715	+	69544712	13	25	ENSSSCG00000013647	+	3'UTR	*CDC37*
							ENSSSCG00000013655	+	intron	*ICAM-1*
2	69723118	69723128	-	69723124	103	276	ENSSSCG00000013643	-	3'UTR	*CDKN2D*
2	69723183	69723183	-	69723183	8	8	ENSSSCG00000013643	-	3'UTR	*CDKN2D*
2	69728049	69728050	-	69728050	8	12	ENSSSCG00000025838	-	intron	---

2	69783389	69783395	+	69783393	31	86	XLOC_048361	+	predicted	---
2	69798166	69798175	-	69798173	10	23	---	---	intergenic	---
2	69798362	69798374	-	69798362	11	29	---	---	intergenic	---
2	69827340	69827340	+	69827340	3	3	---	---	intergenic	---
2	69827486	69827488	+	69827488	27	37	---	---	intergenic	---
2	69855934	69855934	+	69855934	5	5	ENSSSCG00000013637	+	3'UTR	*QTRT1*
2	69858117	69858117	+	69858117	4	4	---	---	intergenic	---
2	70006127	70006142	+	70006142	44	95	XLOC_048366	+	predicted	---
2	70006713	70006716	-	70006714	6	12	XLOC_049880	-	predicted	---
2	70006958	70006958	+	70006958	5	5	XLOC_048366	+	predicted	---
2	70024463	70024467	+	70024463	27	42	ENSSSCG00000013632	+	intron	*C19orf38*
2	70035673	70035673	+	70035673	8	8	ENSSSCG00000013632	+	3'UTR	*C19orf38*
2	70078594	70078605	+	70078600	7	24	XLOC_048368	+	predicted	---
2	70078745	70078745	+	70078745	3	3	XLOC_048368	+	predicted	---
2	70079364	70079364	-	70079364	5	5	XLOC_049881	-	predicted	---
2	70079501	70079522	-	70079521	37	104	XLOC_049881	-	predicted	---
2	70079760	70079761	-	70079761	11	15	ENSSSCG00000013634	-	3'UTR	*YIPF2*
2	70079791	70079791	+	70079791	3	3	XLOC_048368	+	predicted	---
2	70085638	70085638	+	70085638	5	5	ENSSSCG00000013635	+	3'UTR	*C19orf52*
2	70130452	70130454	+	70130452	47	57	XLOC_048371	+	predicted	---
2	70139822	70139822	-	70139822	8	8	---	---	intergenic	---
2	70224907	70224907	-	70224907	13	13	ENSSSCG00000013629	-	3'UTR	*SMARCA4*
2	70332895	70332900	-	70332900	18	32	ENSSSCG00000013627	-	3'UTR	*PRKCSH*
2	70378635	70378642	+	70378640	55	89	XLOC_048375	+	predicted	---
2	70400088	70400099	-	70400094	123	194	ENSSSCG00000013622	-	3'UTR	*TMEM205*
2	70410207	70410214	+	70410211	15	62	---	---	intergenic	---

2	70418864	70418864	+	70418864	3	3	ENSSSCG00000013620	+	3'UTR	---
2	70425363	70425371	+	70425365	12	25	ENSSSCG00000013619	+	3'UTR	*SWSAP1*
2	70425424	70425424	+	70425424	3	3	---	---	intergenic	---
2	70442789	70442789	-	70442789	5	5	ENSSSCG00000030693	-	intron	*TSPAN16*
2	70507981	70508011	-	70508004	31	119	XLOC_049893	-	predicted	---
2	70544009	70544009	+	70544009	3	3	ENSSSCG00000013614	+	3'UTR	*CNN1*
2	70546986	70546996	-	70546991	70	159	ENSSSCG00000013613	-	3'UTR	*ELOF1*
2	70567397	70567398	-	70567398	11	14	---	---	intergenic	---
2	70810991	70810992	-	70810991	3	6	---	---	intergenic	---
2	70948449	70948449	-	70948449	3	3	ENSSSCG00000029035	-	3'UTR	*ZNF558*
2	70978694	70978695	-	70978694	3	6	XLOC_049902	-	predicted	---
2	70979428	70979428	-	70979428	5	5	XLOC_049902	-	predicted	---
2	71001416	71001419	+	71001418	12	20	---	---	intergenic	---
2	71026246	71026252	-	71026252	248	475	---	---	intergenic	---
2	71026324	71026324	-	71026324	5	5	ENSSSCG00000013605	-	ORF	*ACTL9*
2	71062914	71062914	+	71062914	4	4	ENSSSCG00000013607	+	ORF	---
2	71159352	71159352	+	71159352	36	36	XLOC_048390	+	predicted	---
2	71166140	71166140	+	71166140	6	6	XLOC_048391	+	predicted	---
2	71179219	71179219	+	71179219	7	7	ENSSSCG00000024810	+	3'UTR	*PRAM1*
2	71180164	71180168	-	71180166	94	148	ENSSSCG00000013602	-	3'UTR	*HNRNPM*
2	71195689	71195689	-	71195689	4	4	ENSSSCG00000013602	-	intron	*HNRNPM*
							ENSSSCG00000013602	-	ORF	*HNRNPM*
2	71201828	71201828	-	71201828	5	5	ENSSSCG00000013602	-	ORF	*HNRNPM*
2	71228595	71228595	-	71228595	3	3	ENSSSCG00000013601	-	3'UTR	*MARCH2*
2	71228819	71228823	-	71228822	31	66	ENSSSCG00000013601	-	3'UTR	*MARCH2*

2	71251154	71251158	-	71251154	12	30	ENSSSCG00000013600	-	3'UTR	*RAB11B*
							ENSSSCG00000013600	-	intron	*RAB11B*
2	71251265	71251265	-	71251265	8	8	ENSSSCG00000013600	-	3'UTR	*RAB11B*
2	71253535	71253537	-	71253537	45	53	ENSSSCG00000013600	-	ORF	*RAB11B*
2	71263235	71263237	+	71263236	6	15	XLOC_048392	+	predicted	---
2	71272457	71272466	-	71272463	92	180	ENSSSCG00000013599	-	3'UTR	*ANGPTL4*
2	71320232	71320232	+	71320232	4	4	ENSSSCG00000013598	+	3'UTR	*KANK3*
2	71320396	71320404	-	71320398	497	850	ENSSSCG00000013597	-	3'UTR	*RPS28*
2	71328402	71328408	+	71328404	196	375	XLOC_048394	+	predicted	---
2	71405235	71405269	-	71405253	26	129	ENSSSCG00000013595	-	3'UTR	*LASS4*
2	71645226	71645227	+	71645226	16	22	ENSSSCG00000013592	+	3'UTR	*ELAVL1*
2	71646398	71646402	+	71646401	28	49	ENSSSCG00000013592	+	3'UTR	*ELAVL1*
2	71649897	71649897	+	71649897	4	4	XLOC_048398	+	predicted	---
2	71688179	71688183	+	71688182	38	56	ENSSSCG00000013591	+	3'UTR	*TIMM44*
2	71691644	71691655	-	71691646	7	30	ENSSSCG00000013589	-	3'UTR	*SNAPC2*
2	71695349	71695349	-	71695349	4	4	---	---	intergenic	---
2	71698707	71698711	-	71698707	6	12	ENSSSCG00000013588	-	intron	*MAP2K7*
2	71710363	71710363	-	71710363	3	3	XLOC_049916	-	predicted	---
2	71746723	71746723	+	71746723	5	5	---	---	intergenic	---
2	71775649	71775649	+	71775649	4	4	ENSSSCG00000013585	+	3'UTR	*EVI5L*
2	71785458	71785458	-	71785458	4	4	XLOC_049919	-	predicted	---
2	71852229	71852229	+	71852229	16	16	ENSSSCG00000013576	+	3'UTR	*STXBP2*
2	71872645	71872645	+	71872645	8	8	ENSSSCG00000013575	+	3'UTR	*RETN*
2	71883025	71883025	+	71883025	3	3	XLOC_048404	+	predicted	---
2	71883436	71883443	+	71883436	26	33	XLOC_048404	+	predicted	---

2	71886381	71886387	+	71886383	50	110	ENSSSCG00000013573	+	3'UTR	*TRAPPC5*
							ENSSSCG00000013573	+	ORF	---
2	71901019	71901025	+	71901025	8	13	ENSSSCG00000013583	+	3'UTR	*CAMSAP3*
2	71902169	71902169	-	71902169	12	12	ENSSSCG00000013582	-	3'UTR	*XAB2*
2	71913231	71913250	+	71913239	49	110	ENSSSCG00000013581	+	3'UTR	*PET100*
2	71931685	71931685	+	71931685	6	6	ENSSSCG00000013579	+	3'UTR	*CD209*
2	71970820	71970834	+	71970832	33	66	ENSSSCG00000013578	+	3'UTR	*CLEC4G*
							ENSSSCG00000013578	+	ORF	---
2	72000975	72000975	+	72000975	9	9	XLOC_048410	+	predicted	---
2	72005834	72005834	-	72005834	4	4	---	---	intergenic	---
2	72031154	72031156	-	72031154	17	27	ENSSSCG00000013572	-	3'UTR	*PNPLA6*
2	72056672	72056672	-	72056672	11	11	ENSSSCG00000013570	-	3'UTR	*MCOLN1*
2	72153916	72153924	-	72153917	14	63	XLOC_049926	-	predicted	---
2	72164793	72164794	-	72164793	3	6	XLOC_049927	-	predicted	---
2	72174792	72174797	+	72174795	22	58	ENSSSCG00000013569	+	3'UTR	*PEX11G*
2	72176543	72176558	-	72176543	15	46	XLOC_049928	-	predicted	---
2	72204065	72204066	+	72204065	56	60	---	---	intergenic	---
2	72425046	72425052	+	72425049	29	38	ENSSSCG00000013564	+	3'UTR	---
2	72739446	72739447	+	72739447	51	54	ENSSSCG00000013556	+	3'UTR	*ADGRE1*
2	72756140	72756143	+	72756141	45	77	XLOC_048421	+	predicted	---
2	72766814	72766814	+	72766814	8	8	ENSSSCG00000013551	+	ORF	*C3*
2	72767270	72767270	+	72767270	10	10	ENSSSCG00000013551	+	ORF	*C3*
2	72770444	72770447	+	72770447	6	11	ENSSSCG00000013551	+	intron	*C3*
2	72775945	72775945	+	72775945	8	8	ENSSSCG00000013551	+	intron	*C3*
2	72781494	72781515	+	72781515	103	113	ENSSSCG00000013551	+	ORF	*C3*
2	72786104	72786125	+	72786125	18	29	ENSSSCG00000013551	+	ORF	*C3*

2	72827629	72827630	-	72827629	11	14	ENSSSCG00000013553	-	3'UTR	*VAV1*
2	72950559	72950600	-	72950566	1354	6124	ENSSSCG00000030385	-	3'UTR	---
2	72950688	72950703	-	72950692	11	29	ENSSSCG00000030385	-	ORF	---
2	72950932	72950935	-	72950932	3	6	ENSSSCG00000030385	-	ORF	---
2	72952080	72952082	-	72952080	14	20	ENSSSCG00000030385	-	ORF	---
2	72954048	72954052	-	72954051	54	72	ENSSSCG00000030385	-	ORF	---
2	72956232	72956232	-	72956232	6	6	ENSSSCG00000030385	-	ORF	---
2	72956599	72956613	-	72956609	212	455	ENSSSCG00000030385	-	ORF	---
2	72998669	72998669	+	72998669	4	4	XLOC_048426	+	predicted	---
2	73019114	73019114	+	73019114	5	5	---	---	intergenic	---
2	73073084	73073084	-	73073084	3	3	ENSSSCG00000013545	-	intron	*TUBB4A*
2	73166493	73166493	+	73166493	4	4	ENSSSCG00000013537	+	intron	---
2	73168525	73168529	+	73168527	15	45	XLOC_048432	+	predicted	---
2	73181924	73181924	+	73181924	8	8	XLOC_048433	+	predicted	---
2	73197703	73197727	-	73197703	3	9	---	---	intergenic	---
2	73210652	73210652	+	73210652	3	3	ENSSSCG00000013539	+	ORF	*GTF2F1*
2	73210701	73210701	+	73210701	3	3	ENSSSCG00000013539	+	ORF	*GTF2F1*
2	73211774	73211778	+	73211777	44	106	ENSSSCG00000013539	+	3'UTR	*GTF2F1*
2	73215583	73215593	-	73215586	150	486	ENSSSCG00000024254	-	3'UTR	*ALKBH7*
2	73220872	73220878	-	73220873	35	111	XLOC_049953	-	predicted	---
2	73410928	73410928	+	73410928	3	3	XLOC_048436	+	predicted	---
2	73412473	73412479	+	73412473	6	11	XLOC_048436	+	predicted	---
2	73441573	73441590	-	73441582	102	268	XLOC_049955	-	predicted	---
2	73445333	73445333	-	73445333	5	5	ENSSSCG00000013536	-	intron	*ACSBG2*
2	73447658	73447658	-	73447658	3	3	ENSSSCG00000013536	-	ORF	*ACSBG2*
2	73473579	73473579	-	73473579	3	3	ENSSSCG00000013536	-	ORF	*ACSBG2*

2	73675742	73675767	-	73675745	128	337	XLOC_049958	-	predicted	---
2	73685719	73685719	+	73685719	3	3	XLOC_048439	+	predicted	---
2	73685762	73685763	+	73685762	8	11	XLOC_048439	+	predicted	---
2	73687387	73687392	+	73687387	9	15	XLOC_048439	+	predicted	---
2	73690399	73690404	-	73690404	10	16	XLOC_049959	-	predicted	---
2	73691207	73691207	-	73691207	3	3	XLOC_049959	-	predicted	---
2	73691460	73691460	-	73691460	10	10	XLOC_049959	-	predicted	---
2	73808707	73808709	-	73808707	10	13	XLOC_049963	-	predicted	---
2	73808750	73808755	+	73808754	42	101	ENSSSCG00000013526	+	3'UTR	*LONP1*
2	73808880	73808891	-	73808889	842	1561	ENSSSCG00000030010	-	3'UTR	*RPL36*
2	73811632	73811632	-	73811632	3	3	XLOC_049963	-	predicted	---
2	73818802	73818817	+	73818811	64	216	ENSSSCG00000013524	+	3'UTR	*QIL1*
2	73822605	73822616	-	73822616	28	79	ENSSSCG00000013522	-	3'UTR	*SAFB*
2	73832976	73832976	-	73832976	6	6	ENSSSCG00000013522	-	ORF	*SAFB*
2	73833669	73833669	-	73833669	7	7	ENSSSCG00000013522	-	intron	*SAFB*
2	73833831	73833840	-	73833839	14	27	ENSSSCG00000013522	-	ORF	*SAFB*
2	73904684	73904684	-	73904684	3	3	ENSSSCG00000013522	-	intron	*SAFB*
2	73922428	73922428	+	73922428	4	4	ENSSSCG00000013523	+	intron	*SAFB2*
2	73922632	73922632	+	73922632	8	8	ENSSSCG00000013523	+	intron	*SAFB2*
2	74053031	74053031	+	74053031	3	3	---	---	intergenic	---
2	74177085	74177090	-	74177088	204	423	XLOC_049967	-	predicted	---
2	74177170	74177170	-	74177170	4	4	XLOC_049967	-	predicted	---
2	74180538	74180545	+	74180544	47	109	XLOC_048447	+	predicted	---
2	74340465	74340465	+	74340465	10	10	---	---	intergenic	---
2	74458403	74458403	+	74458403	10	10	XLOC_048450	+	predicted	---
2	74642450	74642450	+	74642450	3	3	---	---	intergenic	---

2	74678504	74678504	-	74678504	6	6	XLOC_049972	-	predicted	---
2	74794833	74794833	+	74794833	3	3	ENSSSCG00000013514	+	ORF	*LRG1*
2	74795347	74795364	+	74795351	135	378	XLOC_048453	+	predicted	---
2	74806549	74806554	+	74806552	10	31	ENSSSCG00000013513	+	3'UTR	*LSDP5*
2	74824917	74824922	-	74824922	21	24	ENSSSCG00000013511	-	3'UTR	---
2	74871185	74871206	+	74871186	82	256	ENSSSCG00000013510	+	3'UTR	*UBXN6*
2	74871540	74871549	+	74871543	14	57	ENSSSCG00000013510	+	3'UTR	*UBXN6*
2	74873654	74873657	-	74873654	10	16	ENSSSCG00000013509	-	3'UTR	*CHAF1A*
2	74873796	74873796	-	74873796	4	4	ENSSSCG00000013509	-	3'UTR	*CHAF1A*
2	74938398	74938401	+	74938400	38	54	ENSSSCG00000013508	+	3'UTR	*SH3GL1*
2	74938612	74938615	-	74938613	10	23	ENSSSCG00000013507	-	3'UTR	*MPND*
2	74964483	74964483	+	74964483	3	3	ENSSSCG00000013506	+	3'UTR	*STAP2*
2	75006225	75006227	-	75006226	6	14	XLOC_049982	-	predicted	---
2	75018491	75018491	-	75018491	3	3	ENSSSCG00000013499	-	intron	*CCDC94*
2	75025379	75025379	-	75025379	3	3	---	---	intergenic	---
2	75071640	75071640	-	75071640	4	4	XLOC_049984	-	predicted	---
2	75071641	75071641	+	75071641	8	8	ENSSSCG00000013496	+	3'UTR	*SIRT6*
							ENSSSCG00000013496	+	ORF	*SIRT6*
2	75072337	75072357	-	75072352	63	270	ENSSSCG00000013501	-	3'UTR	*CREB3L3*
2	75194246	75194248	+	75194246	7	14	ENSSSCG00000027127	+	3'UTR	*MAP2K2*
2	75195460	75195460	+	75195460	7	7	ENSSSCG00000027127	+	3'UTR	*MAP2K2*
2	75240464	75240469	+	75240464	8	13	XLOC_048466	+	predicted	---
2	75243588	75243588	+	75243588	4	4	XLOC_048466	+	predicted	---
2	75295134	75295134	+	75295134	4	4	ENSSSCG00000025675	+	ORF	*EEF2*
2	75300752	75300752	+	75300752	3	3	ENSSSCG00000025675	+	3'UTR	*EEF2*
2	75300808	75300816	+	75300808	3	6	ENSSSCG00000025675	+	3'UTR	*EEF2*

2	75300975	75300983	+	75300978	746	1344	ENSSSCG00000025675	+	3'UTR	*EEF2*
2	75320023	75320026	+	75320025	73	103	XLOC_048469	+	predicted	---
2	75516539	75516544	-	75516540	17	33	XLOC_049992	-	predicted	---
2	75523819	75523819	+	75523819	3	3	ENSSSCG00000013492	+	intron	*APBA3*
2	75529526	75529535	+	75529534	23	47	ENSSSCG00000013492	+	3'UTR	*APBA3*
2	75565700	75565700	-	75565700	3	3	---	---	intergenic	---
2	75711291	75711291	+	75711291	3	3	XLOC_048476	+	predicted	---
2	75714918	75714921	+	75714920	19	41	XLOC_048476	+	predicted	---
2	75730713	75730714	-	75730714	11	16	ENSSSCG00000013478	-	3'UTR	*DOHH*
2	75743088	75743088	-	75743088	11	11	---	---	intergenic	---
2	75776374	75776374	-	75776374	4	4	XLOC_049997	-	predicted	---
2	75776431	75776464	-	75776445	276	563	XLOC_049997	-	predicted	---
2	75776787	75776787	-	75776787	3	3	ENSSSCG00000013482	-	ORF	*C19orf71*
2	75780209	75780210	-	75780209	5	8	ENSSSCG00000013481	-	3'UTR	*FZR1*
2	75781668	75781670	-	75781668	10	13	ENSSSCG00000013481	-	3'UTR	*FZR1*
2	75783000	75783011	-	75783009	35	80	ENSSSCG00000013481	-	3'UTR	*FZR1*
2	75892640	75892648	-	75892641	52	86	XLOC_049999	-	predicted	---
2	75893915	75893924	-	75893916	10	21	ENSSSCG00000013475	-	3'UTR	*NCLN*
2	75895537	75895537	-	75895537	4	4	ENSSSCG00000013475	-	intron	*NCLN*
2	75913340	75913340	-	75913340	4	4	XLOC_050001	-	predicted	---
2	75928410	75928416	-	75928414	4	11	ENSSSCG00000013473	-	3'UTR	*GNA15*
2	76043984	76043984	+	76043984	3	3	ENSSSCG00000028995	+	intron	*TLE2*
2	76057178	76057178	+	76057178	4	4	ENSSSCG00000028995	+	3'UTR	*TLE2*
2	76088517	76088517	+	76088517	6	6	ENSSSCG00000029279	+	3'UTR	*GNA11*
2	76090607	76090610	+	76090609	23	36	ENSSSCG00000029279	+	3'UTR	*GNA11*
2	76212410	76212410	+	76212410	3	3	XLOC_048487	+	predicted	---

2	76212572	76212572	+	76212572	3	3	XLOC_048487	+	predicted	---
2	76295320	76295329	+	76295328	12	24	ENSSSCG00000023175	+	intron	---
2	76369980	76369994	-	76369993	13	32	XLOC_050009	-	predicted	---
2	76408502	76408511	+	76408509	61	137	ENSSSCG00000013467	+	3'UTR	*SGTA*
2	76426346	76426349	+	76426347	101	161	ENSSSCG00000022754	+	3'UTR	*SLC39A3*
2	76480683	76480683	+	76480683	11	11	---	---	intergenic	---
2	76541808	76541808	-	76541808	3	3	---	---	intergenic	---
2	76546781	76546783	+	76546783	10	17	---	---	intergenic	---
2	76634452	76634453	+	76634453	8	11	XLOC_048492	+	predicted	---
2	76659395	76659395	-	76659395	13	13	ENSSSCG00000022689	-	3'UTR	*GADD45B*
2	76695319	76695368	+	76695319	21	84	ENSSSCG00000026784	+	3'UTR	*LMNB2*
2	76695607	76695607	+	76695607	4	4	ENSSSCG00000026784	+	3'UTR	*LMNB2*
2	76698029	76698030	+	76698029	7	10	ENSSSCG00000026784	+	3'UTR	*LMNB2*
2	76699096	76699160	+	76699105	87	300	ENSSSCG00000022757	+	3'UTR	*TIMM13*
2	76699624	76699634	+	76699632	37	123	ENSSSCG00000022757	+	3'UTR	*TIMM13*
2	76781962	76781962	-	76781962	6	6	ENSSSCG00000013464	-	3'UTR	*SPPL2B*
2	76782832	76782833	-	76782833	9	15	ENSSSCG00000013464	-	3'UTR	*SPPL2B*
2	76813609	76813627	+	76813616	121	203	ENSSSCG00000013463	+	3'UTR	*LSM7*
2	76854942	76854947	-	76854945	277	445	ENSSSCG00000013460	-	3'UTR	*OAZ1*
2	76855103	76855111	-	76855111	30	86	ENSSSCG00000013460	-	3'UTR	*OAZ1*
2	76855185	76855215	-	76855198	429	644	ENSSSCG00000013460	-	3'UTR	*OAZ1*
2	76856157	76856157	-	76856157	5	5	ENSSSCG00000013460	-	ORF	*OAZ1*
2	76982095	76982098	+	76982096	17	30	XLOC_048500	+	predicted	---
2	77006972	77007006	-	77006982	268	739	XLOC_050022	-	predicted	---
2	77026049	77026052	-	77026052	15	44	XLOC_050025	-	predicted	---
2	77052725	77052754	+	77052728	22	103	XLOC_048501	+	predicted	---

2	77148852	77148873	-	77148864	28	51	ENSSSCG00000013442	-	3'UTR	*ABHD17A*
2	77167350	77167350	+	77167350	5	5	XLOC_048504	+	predicted	---
2	77245674	77245684	-	77245682	17	25	XLOC_050031	-	predicted	---
2	77261815	77261815	+	77261815	3	3	ENSSSCG00000013444	+	intron	*BTBD2*
2	77264704	77264711	+	77264704	8	12	ENSSSCG00000013444	+	3'UTR	*BTBD2*
2	77279216	77279222	-	77279216	3	6	---	---	intergenic	---
2	77279750	77279753	-	77279752	42	88	ENSSSCG00000013445	-	3'UTR	*CSNK1G2*
2	77279901	77279926	-	77279926	8	12	ENSSSCG00000013445	-	3'UTR	*CSNK1G2*
2	77320043	77320057	-	77320054	23	42	ENSSSCG00000013446	-	3'UTR	*SCAMP4*
2	77402720	77402737	-	77402721	56	234	XLOC_050035	-	predicted	---
2	77468896	77468896	-	77468896	3	3	XLOC_050038	-	predicted	---
2	77487840	77487848	+	77487842	102	193	ENSSSCG00000013418	+	3'UTR	*DF*
2	77491097	77491097	-	77491097	5	5	XLOC_050038	-	predicted	---
2	77508534	77508541	-	77508538	17	46	ENSSSCG00000013419	-	3'UTR	*R3HDM4*
2	77569028	77569028	+	77569028	4	4	ENSSSCG00000013414	+	3'UTR	*ARID3A*
2	77580112	77580113	+	77580113	24	27	ENSSSCG00000013423	+	3'UTR	*WDR18*
2	77595382	77595389	-	77595385	46	88	ENSSSCG00000027779	-	3'UTR	*TMEM259*
2	77595450	77595450	+	77595450	3	3	ENSSSCG00000029635	+	intron	*GRIN3B*
2	77621620	77621629	+	77621623	89	106	ENSSSCG00000030270	+	3'UTR	*CNN2*
2	77640440	77640440	+	77640440	4	4	ENSSSCG00000023121	+	3'UTR	*ABCA7*
2	77655495	77655497	+	77655497	47	50	ENSSSCG00000027565	+	3'UTR	*HMHA1*
2	77655747	77655778	-	77655750	57	105	ENSSSCG00000021472	-	3'UTR	*POLR2E*
2	77676020	77676085	+	77676082	1247	4951	XLOC_048522	+	predicted	---
2	77677281	77677281	-	77677281	15	15	ENSSSCG00000030042	-	3'UTR	*SBNO2*
2	77784837	77784844	+	77784842	270	586	ENSSSCG00000013431	+	3'UTR	*ATP5D*
2	77790526	77790528	-	77790527	24	33	ENSSSCG00000013432	-	3'UTR	*MIDN*

2	77792156	77792156	-	77792156	5	5	ENSSSCG00000013432	-	3'UTR	*MIDN*
2	77820037	77820044	+	77820040	22	78	ENSSSCG00000013429	+	intron	*STK11*
2	77823167	77823171	+	77823170	23	43	XLOC_048526	+	predicted	---
2	77823638	77823653	+	77823645	15	27	XLOC_048526	+	predicted	---
2	77838492	77838498	+	77838493	101	203	ENSSSCG00000013427	+	3'UTR	*CIRBP*
2	77840158	77840168	+	77840159	22	38	XLOC_048527	+	predicted	---
2	77844372	77844395	+	77844374	54	122	ENSSSCG00000028040	+	3'UTR	*C19orf24*
2	77876317	77876324	-	77876323	27	100	XLOC_050048	-	predicted	---
2	77888783	77888790	+	77888784	6	19	XLOC_048529	+	predicted	---
2	77895610	77895615	+	77895614	12	32	ENSSSCG00000022265	+	3'UTR	*C19orf25*
2	77935611	77935623	-	77935621	897	1855	ENSSSCG00000023248	-	3'UTR	*RPS15*
2	77941177	77941183	-	77941178	15	32	XLOC_050051	-	predicted	---
2	77941543	77941543	-	77941543	3	3	XLOC_050051	-	predicted	---
2	77941694	77941705	-	77941705	4	7	ENSSSCG00000022528	-	3'UTR	*DAZAP1*
2	77972019	77972053	+	77972035	292	1440	ENSSSCG00000023142	+	3'UTR	*GAMT*
2	77972662	77972675	-	77972669	81	193	ENSSSCG00000024144	-	3'UTR	*NDUFS7*
2	77982198	77982201	-	77982200	35	73	XLOC_050054	-	predicted	---
2	77987121	77987123	-	77987122	9	15	ENSSSCG00000013426	-	intron	*MUM1*
2	78018092	78018092	-	78018092	3	3	---	---	intergenic	---
2	78141937	78141937	-	78141937	4	4	ENSSSCG00000021750	-	3'UTR	*MEX3D*
2	78142528	78142530	-	78142529	9	20	ENSSSCG00000021750	-	3'UTR	*MEX3D*
2	78151916	78151916	+	78151916	4	4	XLOC_048535	+	predicted	---
2	78156273	78156282	-	78156280	59	103	XLOC_050058	-	predicted	---
2	78157111	78157111	-	78157111	3	3	ENSSSCG00000013435	-	3'UTR	*MBD3*
2	78173283	78173283	-	78173283	3	3	---	---	intergenic	---
2	78173434	78173445	-	78173438	194	414	ENSSSCG00000013436	-	3'UTR	---

2	78181361	78181361	-	78181361	4	4	XLOC_050060	-	predicted	---
2	78182724	78182724	-	78182724	5	5	XLOC_050060	-	predicted	---
2	78182791	78182791	-	78182791	3	3	XLOC_050060	-	predicted	---
2	78324192	78324193	-	78324192	4	8	XLOC_050062	-	predicted	---
2	78360157	78360165	-	78360162	67	129	XLOC_050063	-	predicted	---
2	78360254	78360264	-	78360264	36	60	ENSSSCG00000013421	-	3'UTR	*PTBP1*
2	78360936	78360965	-	78360965	5	8	ENSSSCG00000013421	-	3'UTR	*PTBP1*
2	78361694	78361694	-	78361694	3	3	ENSSSCG00000013421	-	3'UTR	*PTBP1*
2	78366343	78366343	-	78366343	3	3	ENSSSCG00000013421	-	intron	*PTBP1*
							ENSSSCG00000013421	-	ORF	*PTBP1*
2	78416085	78416085	-	78416085	3	3	XLOC_050065	-	predicted	---
2	78832862	78832862	+	78832862	5	5	XLOC_048546	+	predicted	---
2	78920236	78920237	+	78920236	13	17	XLOC_048549	+	predicted	---
2	78921826	78921828	+	78921828	8	12	XLOC_048549	+	predicted	---
2	79110994	79110994	+	79110994	4	4	---	---	intergenic	---
2	79241619	79241625	+	79241625	5	12	ENSSSCG00000014002	+	intron	*THEG*
2	79244912	79244927	+	79244919	33	76	ENSSSCG00000014002	+	intron	*THEG*
2	79245214	79245220	+	79245216	31	74	XLOC_048550	+	predicted	---
2	79277765	79277765	+	79277765	5	5	ENSSSCG00000014003	+	3'UTR	*MIER2*
2	79384633	79384633	+	79384633	5	5	---	---	intergenic	---
2	79504045	79504045	+	79504045	4	4	XLOC_048554	+	predicted	---
2	79566448	79566448	-	79566448	3	3	ENSSSCG00000014009	-	3'UTR	*CNOT6*
2	79566788	79566788	-	79566788	7	7	ENSSSCG00000014009	-	3'UTR	*CNOT6*
2	79670464	79670521	-	79670464	8	17	XLOC_050077	-	predicted	---
2	79743193	79743193	-	79743193	6	6	---	---	intergenic	---
2	79813318	79813318	+	79813318	4	4	---	---	intergenic	---

2	79884336	79884342	+	79884336	3	6	XLOC_048559	+	predicted	---
2	80144143	80144143	+	80144143	4	4	ENSSSCG00000014013	+	intron	*RNF130*
2	80183037	80183046	+	80183046	11	17	ENSSSCG00000014013	+	3'UTR	*RNF130*
							ENSSSCG00000014013	+	intron	*RNF130*
2	80199821	80199821	+	80199821	4	4	ENSSSCG00000014013	+	3'UTR	*RNF130*
2	80236072	80236072	+	80236072	3	3	XLOC_048562	+	predicted	---
2	80291586	80291586	+	80291586	3	3	ENSSSCG00000014014	+	3'UTR	*TBC1D9B*
2	80292429	80292438	+	80292429	3	6	ENSSSCG00000014014	+	3'UTR	*TBC1D9B*
2	80318817	80318853	-	80318828	49	189	XLOC_050080	-	predicted	---
2	80318878	80318887	+	80318878	3	6	ENSSSCG00000014015	+	3'UTR	*C5orf45*
2	80319637	80319637	-	80319637	4	4	ENSSSCG00000014016	-	ORF	*SQSTM1*
2	80364459	80364478	+	80364466	14	27	ENSSSCG00000014017	+	3'UTR	*MGAT4B*
2	80365497	80365505	-	80365499	199	456	ENSSSCG00000028488	-	3'UTR	*LTC4S*
2	80371421	80371424	-	80371424	4	10	---	---	intergenic	---
2	80371457	80371466	+	80371461	8	21	---	---	intergenic	---
2	80371595	80371599	-	80371598	10	20	---	---	intergenic	---
2	80384982	80384982	-	80384982	7	7	ENSSSCG00000014019	-	3'UTR	*MAML1*
2	80432174	80432174	-	80432174	3	3	ENSSSCG00000014019	-	intron	*MAML1*
2	80445615	80445615	-	80445615	16	16	ENSSSCG00000014020	-	3'UTR	*CANX*
2	80445683	80445683	-	80445683	3	3	ENSSSCG00000014020	-	3'UTR	*CANX*
2	80446082	80446091	-	80446083	55	96	ENSSSCG00000014020	-	3'UTR	*CANX*
2	80447675	80447675	-	80447675	4	4	ENSSSCG00000014020	-	3'UTR	*CANX*
2	80451229	80451229	-	80451229	3	3	ENSSSCG00000014020	-	ORF	*CANX*
2	80451453	80451481	-	80451453	3	9	ENSSSCG00000014020	-	intron	*CANX*
							ENSSSCG00000014020	-	ORF	*CANX*
2	80459813	80459813	-	80459813	3	3	ENSSSCG00000014020	-	ORF	*CANX*

2	80461844	80461855	-	80461844	4	7	ENSSSCG00000014020	-	intron	*CANX*
							ENSSSCG00000014020	-	ORF	*CANX*
2	80483961	80483961	-	80483961	4	4	---	---	intergenic	---
2	80502338	80502339	+	80502338	17	21	XLOC_048570	+	predicted	---
2	80527689	80527689	-	80527689	3	3	---	---	intergenic	---
2	80529669	80529669	+	80529669	4	4	ENSSSCG00000014022	+	intron	*HNRNPH1*
2	80530971	80530971	+	80530971	3	3	---	---	intergenic	---
2	80531447	80531447	+	80531447	3	3	---	---	intergenic	---
2	80531915	80531915	+	80531915	3	3	ENSSSCG00000014022	+	intron	*HNRNPH1*
2	80533078	80533078	+	80533078	3	3	---	---	intergenic	---
2	80537830	80537830	+	80537830	3	3	XLOC_048571	+	predicted	---
2	80538169	80538170	+	80538169	6	9	XLOC_048571	+	predicted	---
2	80538246	80538246	+	80538246	3	3	XLOC_048571	+	predicted	---
2	80538395	80538406	+	80538395	82	199	XLOC_048571	+	predicted	---
2	80540988	80540988	-	80540988	10	10	ENSSSCG00000014023	-	3'UTR	*RUFY1*
2	80561201	80561201	-	80561201	4	4	ENSSSCG00000014023	-	intron	*RUFY1*
2	81010539	81010539	+	81010539	6	6	XLOC_048572	+	predicted	---
2	81117523	81117523	-	81117523	3	3	XLOC_050090	-	predicted	---
2	81121990	81121993	-	81121990	9	14	ENSSSCG00000029380	-	3'UTR	*ZFP2*
2	81134221	81134231	-	81134221	8	30	ENSSSCG00000029380	-	intron	*ZFP2*
2	81175014	81175014	+	81175014	5	5	---	---	intergenic	---
2	81176606	81176606	+	81176606	5	5	---	---	intergenic	---
2	81295634	81295634	-	81295634	3	3	---	---	intergenic	---
2	81323213	81323214	-	81323213	27	33	---	---	intergenic	---
2	81366052	81366054	+	81366054	7	15	ENSSSCG00000014028	+	ORF	*CLK4*
2	81367330	81367330	+	81367330	3	3	ENSSSCG00000014028	+	ORF	*CLK4*

2	81367398	81367399	+	81367398	18	22	ENSSSCG00000014028	+	3'UTR	*CLK4*
2	81367505	81367511	+	81367505	5	8	ENSSSCG00000014028	+	3'UTR	*CLK4*
2	81367593	81367593	+	81367593	3	3	ENSSSCG00000014028	+	3'UTR	*CLK4*
2	81375189	81375189	-	81375189	5	5	---	---	intergenic	---
2	81430006	81430006	+	81430006	3	3	---	---	intergenic	---
2	81483989	81483989	+	81483989	3	3	---	---	intergenic	---
2	81489783	81489783	+	81489783	3	3	---	---	intergenic	---
2	81579724	81579726	-	81579724	3	6	---	---	intergenic	---
2	81581565	81581565	+	81581565	3	3	---	---	intergenic	---
2	81799222	81799223	-	81799223	42	58	XLOC_050096	-	predicted	---
2	81799425	81799425	-	81799425	3	3	XLOC_050096	-	predicted	---
2	81799623	81799625	-	81799624	18	24	XLOC_050096	-	predicted	---
2	81799667	81799669	-	81799668	35	54	XLOC_050096	-	predicted	---
2	81838672	81838672	-	81838672	3	3	---	---	intergenic	---
2	81844893	81844894	-	81844893	12	17	ENSSSCG00000014033	-	3'UTR	*RMND5B*
2	81919012	81919012	+	81919012	3	3	ENSSSCG00000014036	+	ORF	*TMED9*
2	81922747	81922759	+	81922749	115	228	ENSSSCG00000014036	+	3'UTR	*TMED9*
2	81937554	81937554	+	81937554	4	4	ENSSSCG00000014035	+	3'UTR	*B4GALT7*
2	81937784	81937784	+	81937784	3	3	ENSSSCG00000014035	+	3'UTR	*B4GALT7*
2	81937849	81937849	+	81937849	5	5	ENSSSCG00000014035	+	3'UTR	*B4GALT7*
2	81955993	81955993	-	81955993	3	3	ENSSSCG00000014033	-	intron	*RMND5B*
2	82136032	82136032	+	82136032	4	4	ENSSSCG00000014044	+	ORF	*NSD1*
2	82207432	82207432	+	82207432	5	5	ENSSSCG00000014044	+	intron	*NSD1*
2	82214463	82214467	+	82214463	6	11	XLOC_048591	+	predicted	---
2	82220165	82220171	-	82220171	11	19	ENSSSCG00000014043	-	3'UTR	*RAB24*
2	82226985	82226997	+	82226995	115	304	ENSSSCG00000014042	+	3'UTR	*PRELID1*

2	82227501	82227502	-	82227501	13	19	ENSSSCG00000014041	-	3'UTR	*MXD3*
2	82260350	82260358	-	82260352	119	177	XLOC_050107	-	predicted	---
2	82260727	82260764	-	82260728	19	42	ENSSSCG00000014040	-	3'UTR	*LMAN2*
2	82294430	82294430	+	82294430	10	10	ENSSSCG00000014039	+	3'UTR	*RGS14*
2	82415004	82415004	-	82415004	8	8	ENSSSCG00000014047	-	3'UTR	*FGFR4*
2	82445059	82445059	-	82445059	8	8	ENSSSCG00000014046	-	3'UTR	*ZNF346*
2	82494689	82494707	+	82494701	150	343	ENSSSCG00000022508	+	intron	*RAP80*
2	82502892	82502892	+	82502892	20	20	ENSSSCG00000022508	+	intron	*RAP80*
2	82516484	82516487	+	82516486	10	19	ENSSSCG00000022508	+	ORF	*RAP80*
2	82643922	82643954	+	82643941	16	46	ENSSSCG00000022508	+	3'UTR	*RAP80*
2	82669056	82669062	+	82669060	22	42	ENSSSCG00000014048	+	3'UTR	*HK3*
2	82869085	82869092	-	82869092	6	10	ENSSSCG00000014051	-	3'UTR	*TSPAN17*
2	82920873	82920873	-	82920873	4	4	XLOC_050121	-	predicted	---
2	82991312	82991312	+	82991312	3	3	ENSSSCG00000014056	+	3'UTR	*RNF44*
2	82991880	82991880	+	82991880	4	4	ENSSSCG00000014056	+	3'UTR	*RNF44*
2	83003517	83003517	-	83003517	5	5	ENSSSCG00000014057	-	3'UTR	*FAF2*
2	83005913	83005916	-	83005914	5	15	ENSSSCG00000014057	-	3'UTR	*FAF2*
2	83136281	83136369	+	83136320	27	91	XLOC_048601	+	predicted	---
2	83138545	83138568	-	83138547	414	843	ENSSSCG00000029179	-	3'UTR	*HIGD2A*
2	83146419	83146426	+	83146420	28	62	XLOC_048601	+	predicted	---
2	83191021	83191025	+	83191023	49	75	ENSSSCG00000014060	+	3'UTR	*KIAA1191*
2	83191121	83191121	-	83191121	7	7	ENSSSCG00000014062	-	3'UTR	*SIMC1*
2	83647645	83647645	-	83647645	5	5	ENSSSCG00000014063	-	3'UTR	*SFXN1*
2	83648098	83648098	-	83648098	3	3	ENSSSCG00000014063	-	3'UTR	*SFXN1*
2	83648493	83648497	-	83648497	5	8	ENSSSCG00000014063	-	3'UTR	*SFXN1*
2	84006352	84006353	+	84006352	5	8	ENSSSCG00000014066	+	3'UTR	*TMEM171*

2	84070867	84070871	+	84070870	445	701	---	---	intergenic	---
2	84071048	84071048	+	84071048	5	5	---	---	intergenic	---
2	84116940	84116941	-	84116941	4	7	---	---	intergenic	---
2	84221732	84221732	-	84221732	3	3	ENSSSCG00000014068	-	intron	*UTP15*
2	84240201	84240202	+	84240202	6	11	ENSSSCG00000014070	+	3'UTR	*ANKRA2*
							ENSSSCG00000014070	+	ORF	---
2	84261919	84261920	-	84261920	35	38	XLOC_050134	-	predicted	---
2	84262051	84262057	-	84262053	311	495	ENSSSCG00000014071	-	3'UTR	*BTF3*
2	84262181	84262181	-	84262181	3	3	ENSSSCG00000014071	-	3'UTR	*BTF3*
2	84269695	84269695	-	84269695	5	5	ENSSSCG00000014071	-	ORF	*BTF3*
2	84910266	84910266	-	84910266	3	3	---	---	intergenic	---
2	85266422	85266422	-	85266422	3	3	---	---	intergenic	---
2	85271292	85271292	-	85271292	3	3	XLOC_050137	-	predicted	---
2	85273832	85273832	-	85273832	4	4	ENSSSCG00000014072	-	3'UTR	*ENC1*
2	85371586	85371589	+	85371589	39	46	ENSSSCG00000014073	+	3'UTR	*HEXB*
2	85371667	85371674	-	85371667	5	13	XLOC_050138	-	predicted	---
2	85431247	85431247	+	85431247	6	6	ENSSSCG00000014075	+	ORF	*NSA2*
2	85431326	85431326	+	85431326	3	3	ENSSSCG00000014075	+	intron	*NSA2*
2	85436659	85436659	+	85436659	16	16	ENSSSCG00000014075	+	3'UTR	*NSA2*
2	85436707	85436717	+	85436710	66	146	ENSSSCG00000014075	+	3'UTR	*NSA2*
2	85696058	85696060	-	85696060	7	13	ENSSSCG00000029710	-	3'UTR	*GCNT4*
2	85720311	85720311	-	85720311	9	9	ENSSSCG00000029710	-	intron	*GCNT4*
2	85736790	85736792	-	85736792	8	14	---	---	intergenic	---
2	85888924	85888924	-	85888924	3	3	---	---	intergenic	---
2	85928641	85928641	-	85928641	4	4	---	---	intergenic	---
2	85988241	85988242	+	85988242	6	10	ENSSSCG00000014080	+	3'UTR	*HMGCR*

2	85989710	85989710	+	85989710	4	4	ENSSSCG00000014080	+	3'UTR	*HMGCR*
2	85990064	85990082	+	85990076	8	32	ENSSSCG00000014080	+	3'UTR	*HMGCR*
2	85992326	85992326	+	85992326	4	4	XLOC_048621	+	predicted	---
2	85997592	85997593	-	85997593	11	16	XLOC_050145	-	predicted	---
2	86094828	86094830	-	86094828	3	6	---	---	intergenic	---
2	86213780	86213781	+	86213780	3	6	ENSSSCG00000014082	+	intron	*POLK*
2	86214392	86214399	+	86214395	26	32	ENSSSCG00000014082	+	intron	*POLK*
2	86291595	86291595	+	86291595	3	3	ENSSSCG00000014083	+	intron	*ANKDD1B*
2	86326168	86326168	-	86326168	12	12	ENSSSCG00000014084	-	ORF	*POC5*
2	86464165	86464165	+	86464165	3	3	---	---	intergenic	---
2	86612494	86612494	-	86612494	5	5	---	---	intergenic	---
2	86890633	86890634	-	86890633	3	6	---	---	intergenic	---
2	87135490	87135490	-	87135490	3	3	ENSSSCG00000014088	-	ORF	*IQGAP2*
2	87155366	87155366	-	87155366	3	3	ENSSSCG00000014088	-	ORF	*IQGAP2*
2	87170935	87170935	-	87170935	4	4	ENSSSCG00000014088	-	ORF	*IQGAP2*
2	87207741	87207741	-	87207741	4	4	ENSSSCG00000014088	-	intron	*IQGAP2*
2	87279785	87279789	-	87279786	26	46	ENSSSCG00000020672	-	3'UTR	*F2R*
2	87305248	87305258	-	87305252	49	115	ENSSSCG00000025645	-	3'UTR	---
2	87305305	87305305	-	87305305	3	3	ENSSSCG00000025645	-	3'UTR	---
2	87305985	87305998	-	87305986	16	44	ENSSSCG00000025645	-	3'UTR	---
2	87316208	87316208	-	87316208	5	5	ENSSSCG00000025645	-	ORF	---
2	87318596	87318596	-	87318596	5	5	ENSSSCG00000025645	-	intron	---
2	87332962	87332962	-	87332962	4	4	ENSSSCG00000025645	-	intron	---
2	87542382	87542382	+	87542382	4	4	ENSSSCG00000014094	+	intron	*AGGF1*
2	87550865	87550865	+	87550865	4	4	ENSSSCG00000014094	+	3'UTR	*AGGF1*
2	87562638	87562638	-	87562638	3	3	XLOC_050154	-	predicted	---

2	87829431	87829432	+	87829431	7	12	ENSSSCG00000014097	+	intron	*PDE8B*
2	87933807	87933816	-	87933807	8	13	ENSSSCG00000014098	-	3'UTR	*WDR41*
2	88189129	88189174	-	88189156	178	568	ENSSSCG00000014100	-	3'UTR	*TBCA*
2	88192598	88192604	-	88192598	11	19	ENSSSCG00000014100	-	intron	*TBCA*
2	88273083	88273087	-	88273087	5	9	---	---	intergenic	---
2	88273665	88273669	+	88273665	5	10	---	---	intergenic	---
2	88288934	88288934	+	88288934	4	4	---	---	intergenic	---
2	88534416	88534420	-	88534416	10	16	XLOC_050163	-	predicted	---
2	88552246	88552246	-	88552246	4	4	ENSSSCG00000014101	-	ORF	---
2	88658614	88658614	-	88658614	3	3	ENSSSCG00000014101	-	intron	---
2	88746476	88746476	+	88746476	3	3	ENSSSCG00000027810	+	ORF	*AP3B1*
2	88972811	88972811	+	88972811	21	21	ENSSSCG00000028536	+	3'UTR	*LHFPL2*
2	88980417	88980418	-	88980417	5	10	XLOC_050165	-	predicted	---
2	88982235	88982235	-	88982235	3	3	XLOC_050165	-	predicted	---
2	88983058	88983059	-	88983058	8	11	XLOC_050165	-	predicted	---
2	89406700	89406723	-	89406703	392	854	---	---	intergenic	---
2	89406796	89406796	-	89406796	3	3	XLOC_050168	-	predicted	---
2	89407076	89407076	-	89407076	3	3	XLOC_050168	-	predicted	---
2	89407293	89407294	-	89407293	3	6	XLOC_050168	-	predicted	---
2	89407389	89407389	-	89407389	3	3	XLOC_050168	-	predicted	---
2	89407752	89407772	-	89407764	410	887	ENSSSCG00000014108	-	3'UTR	*BHMT*
2	89407916	89407916	-	89407916	7	7	ENSSSCG00000014108	-	ORF	*BHMT*
2	89417680	89417680	-	89417680	19	19	ENSSSCG00000014108	-	ORF	*BHMT*
2	89419142	89419144	-	89419144	26	33	ENSSSCG00000014108	-	ORF	*BHMT*
2	89424940	89424940	-	89424940	3	3	ENSSSCG00000014108	-	intron	*BHMT*
2	89431051	89431051	-	89431051	3	3	---	---	intergenic	---

2	89459602	89459606	-	89459603	11	35	ENSSSCG00000014111	-	3'UTR	*BHMT-2*
2	89459758	89459758	-	89459758	3	3	ENSSSCG00000014111	-	3'UTR	*BHMT-2*
2	89468621	89468621	-	89468621	8	8	ENSSSCG00000014111	-	ORF	*BHMT-2*
2	89468679	89468679	-	89468679	3	3	ENSSSCG00000014111	-	ORF	*BHMT-2*
2	89513221	89513221	+	89513221	3	3	ENSSSCG00000014110	+	ORF	*DMGDH*
2	89547061	89547066	+	89547066	8	11	ENSSSCG00000014110	+	intron	*DMGDH*
2	89547231	89547243	+	89547242	25	72	ENSSSCG00000014110	+	intron	*DMGDH*
2	89547359	89547366	+	89547365	11	22	ENSSSCG00000014110	+	intron	*DMGDH*
2	89593305	89593305	+	89593305	4	4	ENSSSCG00000014110	+	ORF	*DMGDH*
2	89593593	89593593	+	89593593	5	5	ENSSSCG00000014110	+	3'UTR	*DMGDH*
2	89792746	89792748	+	89792748	10	13	ENSSSCG00000014112	+	ORF	*JMY*
2	89813724	89813724	-	89813724	4	4	XLOC_050171	-	predicted	---
2	89819884	89819884	+	89819884	3	3	XLOC_048656	+	predicted	---
2	90091155	90091156	+	90091156	15	20	---	---	intergenic	---
2	90102059	90102059	-	90102059	6	6	---	---	intergenic	---
2	90181078	90181084	+	90181084	8	11	ENSSSCG00000014114	+	3'UTR	*PAPD4*
2	90182365	90182399	+	90182398	13	20	XLOC_048657	+	predicted	---
2	90215308	90215308	-	90215308	3	3	ENSSSCG00000026098	-	intron	*CMYA5*
2	90275202	90275202	-	90275202	5	5	---	---	intergenic	---
2	90350762	90350762	-	90350762	5	5	---	---	intergenic	---
2	90401136	90401136	-	90401136	3	3	XLOC_050176	-	predicted	---
2	90403601	90403601	+	90403601	3	3	---	---	intergenic	---
2	90496961	90496962	+	90496962	8	12	---	---	intergenic	---
2	90694033	90694033	+	90694033	14	14	ENSSSCG00000014119	+	3'UTR	---
2	90794179	90794179	-	90794179	4	4	---	---	intergenic	---
2	90883376	90883376	-	90883376	3	3	ENSSSCG00000014122	-	ORF	*SPZ1*

2	90885788	90885788	+	90885788	8	8	XLOC_048662	+	predicted	---
2	90954770	90954770	+	90954770	3	3	XLOC_048663	+	predicted	---
2	90954884	90954884	+	90954884	4	4	XLOC_048663	+	predicted	---
2	90977833	90977833	+	90977833	3	3	ENSSSCG00000014123	+	intron	*FAM151B*
2	90992170	90992173	+	90992173	5	9	ENSSSCG00000014123	+	3'UTR	*FAM151B*
							ENSSSCG00000014123	+	ORF	*FAM151B*
2	91078637	91078645	-	91078638	5	21	ENSSSCG00000014125	-	3'UTR	*DHFR*
2	91235849	91235850	+	91235849	15	20	ENSSSCG00000014126	+	intron	*MSH3*
2	91266476	91266476	+	91266476	7	7	ENSSSCG00000014126	+	intron	*MSH3*
2	91309659	91309666	-	91309665	47	94	---	---	intergenic	---
2	91391878	91391878	+	91391878	7	7	---	---	intergenic	---
2	91507668	91507680	-	91507672	6	19	---	---	intergenic	---
2	91507701	91507701	+	91507701	4	4	---	---	intergenic	---
2	91623198	91623198	+	91623198	11	11	ENSSSCG00000014127	+	intron	*RASGRF2*
2	91748507	91748507	+	91748507	5	5	---	---	intergenic	---
2	91754198	91754204	-	91754198	20	40	---	---	intergenic	---
2	91865498	91865498	-	91865498	3	3	ENSSSCG00000014131	-	3'UTR	*SSBP2*
2	91865626	91865626	-	91865626	5	5	ENSSSCG00000014131	-	3'UTR	*SSBP2*
2	91932428	91932429	-	91932428	32	61	ENSSSCG00000014131	-	intron	*SSBP2*
2	91992899	91992899	+	91992899	9	9	---	---	intergenic	---
2	92454437	92454437	+	92454437	4	4	ENSSSCG00000025210	+	intron	*ATG10*
2	92719907	92719917	+	92719907	4	8	XLOC_048675	+	predicted	---
2	92720181	92720194	+	92720181	3	6	XLOC_048675	+	predicted	---
2	92796172	92796172	+	92796172	8	8	---	---	intergenic	---
2	93366436	93366436	-	93366436	5	5	---	---	intergenic	---
2	93410337	93410338	-	93410338	11	15	XLOC_050198	-	predicted	---

2	93410440	93410440	-	93410440	12	12	XLOC_050198	-	predicted	---
2	93410595	93410601	-	93410595	3	6	XLOC_050198	-	predicted	---
2	93410648	93410649	-	93410648	7	10	XLOC_050198	-	predicted	---
2	93410875	93410877	-	93410875	4	11	XLOC_050198	-	predicted	---
2	93411000	93411001	-	93411001	6	10	XLOC_050198	-	predicted	---
2	93411125	93411125	-	93411125	4	4	XLOC_050198	-	predicted	---
2	93894997	93894997	+	93894997	3	3	ENSSSCG00000014136	+	3'UTR	*VCAN*
2	93920613	93920613	+	93920613	12	12	---	---	intergenic	---
2	93926083	93926084	-	93926083	4	7	---	---	intergenic	---
2	93989914	93989914	-	93989914	3	3	---	---	intergenic	---
2	94916950	94916950	+	94916950	5	5	---	---	intergenic	---
2	95317865	95317865	-	95317865	3	3	---	---	intergenic	---
2	95530711	95530711	+	95530711	3	3	---	---	intergenic	---
2	95696833	95696837	+	95696833	21	28	ENSSSCG00000014141	+	3'UTR	*RFESD*
2	95733960	95733967	-	95733962	11	32	XLOC_050205	-	predicted	---
2	95842418	95842418	+	95842418	7	7	---	---	intergenic	---
2	95860361	95860361	-	95860361	44	44	---	---	intergenic	---
2	96264504	96264528	-	96264522	55	67	XLOC_050206	-	predicted	---
2	96265052	96265052	-	96265052	27	27	XLOC_050206	-	predicted	---
2	96265674	96265675	-	96265674	19	28	---	---	intergenic	---
2	96266942	96266942	-	96266942	12	12	---	---	intergenic	---
2	96267200	96267200	-	96267200	4	4	---	---	intergenic	---
2	96267979	96267980	-	96267979	9	13	---	---	intergenic	---
2	96268677	96268686	-	96268677	17	26	---	---	intergenic	---
2	96444833	96444834	-	96444834	4	7	---	---	intergenic	---
2	96465854	96465854	+	96465854	7	7	---	---	intergenic	---

2	96728238	96728238	-	96728238	5	5	---	---	intergenic	---
2	96866614	96866657	+	96866625	547	1114	ENSSSCG00000014145	+	3'UTR	*COX7C*
2	96867000	96867000	+	96867000	3	3	ENSSSCG00000014145	+	3'UTR	*COX7C*
2	97031356	97031356	-	97031356	5	5	---	---	intergenic	---
2	97409820	97409822	+	97409821	15	31	ENSSSCG00000014146	+	intron	*RASA1*
2	97480525	97480525	+	97480525	9	9	ENSSSCG00000014146	+	intron	*RASA1*
2	97511538	97511538	+	97511538	15	15	ENSSSCG00000014146	+	intron	*RASA1*
2	97517168	97517168	-	97517168	3	3	ENSSSCG00000014147	-	intron	*CCNH*
							ENSSSCG00000014147	-	ORF	*CCNH*
2	97701802	97701802	-	97701802	3	3	---	---	intergenic	---
2	97826862	97826862	+	97826862	6	6	---	---	intergenic	---
2	98210181	98210181	+	98210181	3	3	---	---	intergenic	---
2	98618431	98618431	-	98618431	4	4	---	---	intergenic	---
2	98790617	98790618	-	98790617	3	6	XLOC_050212	-	predicted	---
2	98909237	98909239	+	98909238	9	16	---	---	intergenic	---
2	99188821	99188821	-	99188821	3	3	---	---	intergenic	---
2	100250982	100250982	+	100250982	3	3	---	---	intergenic	---
2	100266110	100266110	-	100266110	3	3	XLOC_050214	-	predicted	---
2	100398602	100398602	+	100398602	4	4	---	---	intergenic	---
2	100411595	100411596	-	100411595	8	13	---	---	intergenic	---
2	100651983	100651990	-	100651985	46	78	ENSSSCG00000014151	-	3'UTR	*CETN3*
2	100652097	100652097	-	100652097	5	5	ENSSSCG00000014151	-	3'UTR	*CETN3*
2	100767464	100767464	-	100767464	3	3	XLOC_050217	-	predicted	---
2	100770331	100770331	-	100770331	3	3	ENSSSCG00000024193	-	3'UTR	*LYSMD3*
2	100855036	100855036	-	100855036	3	3	---	---	intergenic	---
2	100856168	100856168	+	100856168	3	3	---	---	intergenic	---

2	101151609	101151609	+	101151609	4	4	---	---	intergenic	---
2	101475413	101475413	+	101475413	7	7	---	---	intergenic	---
2	101569573	101569575	-	101569574	23	33	ENSSSCG00000014156	-	3'UTR	*ARRDC3*
2	101686338	101686338	-	101686338	4	4	---	---	intergenic	---
2	102421875	102421875	+	102421875	5	5	---	---	intergenic	---
2	102741491	102741491	+	102741491	3	3	---	---	intergenic	---
2	103220756	103220756	-	103220756	4	4	---	---	intergenic	---
2	103235133	103235133	-	103235133	3	3	---	---	intergenic	---
2	103687069	103687077	-	103687069	3	9	XLOC_050232	-	predicted	---
2	103919131	103919131	-	103919131	3	3	XLOC_050235	-	predicted	---
2	104290349	104290349	-	104290349	7	7	---	---	intergenic	---
2	104424180	104424180	-	104424180	7	7	---	---	intergenic	---
2	104536729	104536733	-	104536733	13	21	ENSSSCG00000014159	-	intron	---
2	104873918	104873918	+	104873918	3	3	ENSSSCG00000014163	+	ORF	*ANKRD32*
2	105071172	105071172	+	105071172	3	3	XLOC_048724	+	predicted	---
2	105096878	105096878	+	105096878	4	4	---	---	intergenic	---
2	105631840	105631840	-	105631840	4	4	---	---	intergenic	---
2	105816984	105816984	+	105816984	5	5	ENSSSCG00000026175	+	intron	*FAM81B*
2	105820833	105820833	+	105820833	6	6	ENSSSCG00000026175	+	3'UTR	*FAM81B*
2	105820921	105820934	+	105820931	9	35	XLOC_048727	+	predicted	---
2	105833573	105833574	-	105833573	7	10	XLOC_050242	-	predicted	---
2	105838181	105838181	-	105838181	4	4	ENSSSCG00000014165	-	intron	---
2	106279273	106279273	+	106279273	3	3	XLOC_048729	+	predicted	---
2	106280138	106280138	+	106280138	3	3	XLOC_048729	+	predicted	---
2	106310499	106310504	-	106310501	193	258	XLOC_050244	-	predicted	---
2	106310583	106310583	-	106310583	11	11	ENSSSCG00000014167	-	3'UTR	*GRX1*

2	106393479	106393479	-	106393479	3	3	---	---	intergenic	---
2	106402543	106402543	-	106402543	6	6	XLOC_050245	-	predicted	---
2	106403514	106403514	-	106403514	4	4	XLOC_050245	-	predicted	---
2	106541412	106541415	+	106541414	4	10	---	---	intergenic	---
2	106650069	106650069	+	106650069	6	6	---	---	intergenic	---
2	106851279	106851279	-	106851279	3	3	---	---	intergenic	---
2	106851307	106851310	+	106851307	3	6	---	---	intergenic	---
2	107030915	107030915	+	107030915	5	5	---	---	intergenic	---
2	107039355	107039355	+	107039355	3	3	---	---	intergenic	---
2	107041078	107041085	+	107041085	5	9	ENSSSCG00000014170	+	intron	*CAST*
2	107042123	107042134	+	107042123	9	16	ENSSSCG00000014170	+	intron	*CAST*
2	107099569	107099569	+	107099569	11	11	ENSSSCG00000014170	+	intron	*CAST*
2	107107204	107107204	+	107107204	3	3	---	---	intergenic	---
2	107112860	107112860	+	107112860	3	3	---	---	intergenic	---
2	107117564	107117564	+	107117564	4	4	ENSSSCG00000014170	+	intron	*CAST*
2	107128518	107128518	+	107128518	3	3	---	---	intergenic	---
2	107131780	107131780	-	107131780	5	5	XLOC_050247	-	predicted	---
2	107294865	107294865	+	107294865	3	3	---	---	intergenic	---
2	107363268	107363268	+	107363268	7	7	ENSSSCG00000027860	+	3'UTR	---
2	107363740	107363740	+	107363740	3	3	ENSSSCG00000027860	+	3'UTR	---
2	107415929	107415929	+	107415929	3	3	ENSSSCG00000014173	+	intron	*LNPEP*
2	107467452	107467452	+	107467452	3	3	XLOC_048738	+	predicted	---
2	107780027	107780027	-	107780027	3	3	---	---	intergenic	---
2	107984615	107984615	-	107984615	4	4	---	---	intergenic	---
2	109023195	109023195	-	109023195	4	4	ENSSSCG00000014177	-	intron	*CHD1*
2	109023856	109023856	-	109023856	3	3	ENSSSCG00000014177	-	intron	*CHD1*

2	109038428	109038428	-	109038428	3	3	ENSSSCG00000014177	-	intron	*CHD1*
2	109065089	109065089	+	109065089	5	5	XLOC_048742	+	predicted	---
2	109707973	109707973	-	109707973	6	6	---	---	intergenic	---
2	109876026	109876029	+	109876026	3	6	---	---	intergenic	---
2	109961816	109961816	-	109961816	4	4	---	---	intergenic	---
2	110471015	110471015	-	110471015	3	3	---	---	intergenic	---
2	110562476	110562476	+	110562476	7	7	ENSSSCG00000014179	+	3'UTR	*FAM174A*
2	110562904	110562904	+	110562904	5	5	ENSSSCG00000014179	+	3'UTR	*FAM174A*
2	110733502	110733502	-	110733502	5	5	XLOC_050260	-	predicted	---
2	110900968	110900968	-	110900968	3	3	---	---	intergenic	---
2	111188403	111188403	+	111188403	4	4	---	---	intergenic	---
2	111209584	111209584	+	111209584	4	4	---	---	intergenic	---
2	111766840	111766840	+	111766840	3	3	---	---	intergenic	---
2	112015152	112015158	-	112015158	21	33	XLOC_050263	-	predicted	---
2	112497366	112497366	+	112497366	5	5	ENSSSCG00000014184	+	ORF	*PAM*
2	112553938	112553945	+	112553941	15	36	ENSSSCG00000014184	+	3'UTR	*PAM*
2	112554237	112554243	+	112554237	4	7	XLOC_048748	+	predicted	---
2	112579165	112579165	-	112579165	3	3	XLOC_050265	-	predicted	---
2	112823977	112823978	+	112823977	4	8	XLOC_048751	+	predicted	---
2	113204432	113204432	-	113204432	5	5	---	---	intergenic	---
2	113473877	113473877	-	113473877	3	3	---	---	intergenic	---
2	115100148	115100148	+	115100148	9	9	---	---	intergenic	---
2	115100154	115100154	-	115100154	10	10	---	---	intergenic	---
2	115100241	115100275	-	115100243	13	31	---	---	intergenic	---
2	115100298	115100298	+	115100298	11	11	---	---	intergenic	---
2	115100412	115100412	+	115100412	3	3	---	---	intergenic	---

2	115100572	115100583	-	115100583	12	26	---	---	intergenic	---
2	115503261	115503268	+	115503263	9	30	---	---	intergenic	---
2	115681827	115681827	-	115681827	3	3	XLOC_050269	-	predicted	---
2	116442090	116442093	-	116442093	4	7	---	---	intergenic	---
2	116800359	116800359	+	116800359	3	3	---	---	intergenic	---
2	116864068	116864068	+	116864068	3	3	---	---	intergenic	---
2	117048474	117048474	-	117048474	3	3	XLOC_050272	-	predicted	---
2	117048558	117048558	-	117048558	4	4	XLOC_050272	-	predicted	---
2	117187882	117187883	-	117187882	32	38	---	---	intergenic	---
2	117556835	117556836	-	117556835	8	13	---	---	intergenic	---
2	118048399	118048406	+	118048401	7	20	XLOC_048760	+	predicted	---
2	118240822	118240822	+	118240822	5	5	ENSSSCG00000014191	+	intron	---
2	118846504	118846504	-	118846504	5	5	ENSSSCG00000014194	-	ORF	---
2	118848587	118848587	-	118848587	3	3	ENSSSCG00000014194	-	ORF	---
2	118859577	118859577	-	118859577	3	3	ENSSSCG00000014194	-	intron	---
2	118923916	118923916	-	118923916	4	4	ENSSSCG00000022845	-	3'UTR	*PJA2*
2	118926114	118926114	-	118926114	4	4	ENSSSCG00000022845	-	3'UTR	*PJA2*
2	118926483	118926485	-	118926484	12	18	ENSSSCG00000022845	-	3'UTR	*PJA2*
2	119030102	119030104	-	119030104	13	16	---	---	intergenic	---
2	119134528	119134528	+	119134528	6	6	---	---	intergenic	---
2	119212746	119212746	+	119212746	3	3	ENSSSCG00000014195	+	intron	*MAN2A1*
2	119310265	119310266	+	119310265	5	8	ENSSSCG00000014195	+	3'UTR	*MAN2A1*
2	119741349	119741349	+	119741349	4	4	---	---	intergenic	---
2	119783660	119783661	-	119783660	3	6	XLOC_050282	-	predicted	---
2	120006444	120006444	+	120006444	34	34	---	---	intergenic	---
2	120043048	120043048	+	120043048	10	10	ENSSSCG00000014197	+	3'UTR	*SLC25A46*

2	120051058	120051058	-	120051058	3	3	---	---	intergenic	---
2	120051087	120051120	+	120051118	5	12	---	---	intergenic	---
2	120051292	120051302	-	120051292	4	8	---	---	intergenic	---
2	120052381	120052382	-	120052382	5	8	---	---	intergenic	---
2	120052454	120052485	+	120052485	22	47	---	---	intergenic	---
2	120052705	120052705	+	120052705	6	6	---	---	intergenic	---
2	120052937	120052937	+	120052937	6	6	---	---	intergenic	---
2	120053268	120053274	-	120053272	7	13	---	---	intergenic	---
2	120053357	120053357	-	120053357	3	3	---	---	intergenic	---
2	120053392	120053429	+	120053395	17	50	---	---	intergenic	---
2	120053404	120053442	-	120053439	7	22	---	---	intergenic	---
2	120053446	120053490	+	120053474	98	176	---	---	intergenic	---
2	120053532	120053569	+	120053565	9741	17756	---	---	intergenic	---
2	120053559	120053559	-	120053559	3	3	---	---	intergenic	---
2	120053641	120053664	-	120053660	38	223	---	---	intergenic	---
2	120053678	120053678	+	120053678	40	40	---	---	intergenic	---
2	120053803	120053807	-	120053803	4	11	---	---	intergenic	---
2	120053997	120053997	-	120053997	4	4	---	---	intergenic	---
2	120054313	120054322	+	120054321	383	447	---	---	intergenic	---
2	120054621	120054629	+	120054629	11	14	---	---	intergenic	---
2	120054729	120054736	+	120054729	3	6	---	---	intergenic	---
2	120055072	120055072	-	120055072	3	3	---	---	intergenic	---
2	120056010	120056012	+	120056011	8	14	---	---	intergenic	---
2	120056784	120056784	-	120056784	3	3	---	---	intergenic	---
2	120056818	120056842	+	120056825	16	62	---	---	intergenic	---
2	120057493	120057493	+	120057493	3	3	---	---	intergenic	---

2	120058033	120058050	+	120058036	8	14	---	---	intergenic	---
2	120058158	120058162	+	120058161	347	390	---	---	intergenic	---
2	120059084	120059086	-	120059084	4	8	---	---	intergenic	---
2	120059712	120059712	-	120059712	6	6	---	---	intergenic	---
2	120059765	120059797	+	120059791	517	597	---	---	intergenic	---
2	120060265	120060321	+	120060278	18	41	---	---	intergenic	---
2	120060493	120060493	+	120060493	12	12	---	---	intergenic	---
2	120061737	120061737	+	120061737	4	4	---	---	intergenic	---
2	120071960	120071960	+	120071960	4	4	---	---	intergenic	---
2	120105915	120105920	+	120105915	12	31	---	---	intergenic	---
2	120180349	120180349	-	120180349	3	3	---	---	intergenic	---
2	120386125	120386139	-	120386126	32	64	XLOC_050283	-	predicted	---
2	120814651	120814664	+	120814658	45	86	ENSSSCG00000023497	+	3'UTR	*CAMK4*
2	120826729	120826730	-	120826729	17	20	XLOC_050286	-	predicted	---
2	120883539	120883539	-	120883539	4	4	---	---	intergenic	---
2	120971746	120971746	-	120971746	14	14	---	---	intergenic	---
2	121364618	121364618	-	121364618	7	7	XLOC_050291	-	predicted	---
2	121371838	121371839	-	121371838	6	10	XLOC_050291	-	predicted	---
2	121467788	121467788	-	121467788	3	3	---	---	intergenic	---
2	121483140	121483140	+	121483140	4	4	ENSSSCG00000022048	+	intron	*REEP5*
2	121505515	121505519	+	121505516	45	68	ENSSSCG00000022048	+	3'UTR	*REEP5*
2	121505656	121505656	+	121505656	3	3	ENSSSCG00000022048	+	3'UTR	*REEP5*
2	121507937	121507942	+	121507937	30	61	ENSSSCG00000022048	+	3'UTR	*REEP5*
2	121515470	121515484	-	121515477	89	177	ENSSSCG00000026230	-	3'UTR	---
2	121515635	121515674	-	121515672	6	17	ENSSSCG00000026230	-	3'UTR	---
2	121515911	121515923	-	121515911	3	6	ENSSSCG00000026230	-	ORF	---

2	121518158	121518158	-	121518158	3	3	ENSSSCG00000026230	-	intron	---
2	121523276	121523278	+	121523277	5	12	---	---	intergenic	---
2	121577828	121577828	-	121577828	3	3	XLOC_050294	-	predicted	---
2	121577940	121577940	-	121577940	6	6	XLOC_050294	-	predicted	---
2	121578101	121578101	-	121578101	3	3	XLOC_050294	-	predicted	---
2	122146383	122146384	-	122146383	7	10	XLOC_050298	-	predicted	---
2	122392623	122392623	+	122392623	4	4	ENSSSCG00000025831	+	3'UTR	*NREP*
2	122393178	122393187	+	122393185	56	113	ENSSSCG00000025831	+	3'UTR	*NREP*
2	122480616	122480616	+	122480616	3	3	---	---	intergenic	---
2	123098572	123098572	-	123098572	9	9	---	---	intergenic	---
2	123612810	123612810	+	123612810	8	8	ENSSSCG00000014213	+	3'UTR	*KCNN2*
2	124053891	124053891	-	124053891	3	3	---	---	intergenic	---
2	124121855	124121875	-	124121858	86	385	ENSSSCG00000014214	-	3'UTR	*TRIM36*
2	124121919	124121923	-	124121920	27	50	ENSSSCG00000014214	-	3'UTR	*TRIM36*
2	124128077	124128077	-	124128077	3	3	---	---	intergenic	---
2	124141312	124141312	-	124141312	3	3	---	---	intergenic	---
2	124154125	124154125	-	124154125	5	5	ENSSSCG00000014214	-	intron	*TRIM36*
2	124203728	124203728	-	124203728	6	6	ENSSSCG00000014215	-	3'UTR	*PGGT1B*
2	124337488	124337491	-	124337489	4	14	ENSSSCG00000029866	-	3'UTR	*CCDC112*
2	124427077	124427077	-	124427077	3	3	---	---	intergenic	---
2	124461484	124461510	+	124461498	11	59	---	---	intergenic	---
2	124490541	124490541	-	124490541	3	3	---	---	intergenic	---
2	124605090	124605094	-	124605090	4	7	XLOC_050310	-	predicted	---
2	124606006	124606006	-	124606006	3	3	XLOC_050310	-	predicted	---
2	124606073	124606077	-	124606074	26	47	XLOC_050310	-	predicted	---
2	124613027	124613027	+	124613027	3	3	ENSSSCG00000014216	+	3'UTR	*TICAM2*

2	124653529	124653534	-	124653531	32	57	---	---	intergenic	---
2	124679726	124679735	-	124679735	20	28	XLOC_050312	-	predicted	---
2	124682038	124682041	-	124682041	15	21	XLOC_050312	-	predicted	---
2	124682233	124682233	-	124682233	3	3	ENSSSCG00000014218	-	3'UTR	*TMED7*
2	124815048	124815090	-	124815055	398	1134	ENSSSCG00000014219	-	3'UTR	*CDO1*
2	124815384	124815426	-	124815391	6	15	ENSSSCG00000014219	-	3'UTR	*CDO1*
2	124817435	124817435	-	124817435	3	3	ENSSSCG00000014219	-	ORF	*CDO1*
2	124829196	124829197	-	124829196	5	8	XLOC_050314	-	predicted	---
2	124834160	124834160	-	124834160	3	3	XLOC_050314	-	predicted	---
2	124834871	124834874	-	124834871	18	25	ENSSSCG00000014220	-	3'UTR	*ATG12*
2	125022612	125022612	+	125022612	3	3	---	---	intergenic	---
2	125366081	125366081	-	125366081	3	3	---	---	intergenic	---
2	125429032	125429032	-	125429032	9	9	---	---	intergenic	---
2	125466105	125466107	-	125466107	8	11	ENSSSCG00000014224	-	3'UTR	*SEMA6A*
2	125468025	125468025	-	125468025	5	5	ENSSSCG00000014224	-	3'UTR	*SEMA6A*
2	125540837	125540837	+	125540837	4	4	---	---	intergenic	---
2	125615931	125615931	+	125615931	3	3	---	---	intergenic	---
2	125960434	125960438	+	125960434	20	30	---	---	intergenic	---
2	126455486	126455486	+	126455486	4	4	---	---	intergenic	---
2	127234611	127234611	+	127234611	4	4	---	---	intergenic	---
2	127531644	127531645	+	127531644	32	36	---	---	intergenic	---
2	127769843	127769845	-	127769844	17	23	XLOC_050320	-	predicted	---
2	127831619	127831619	+	127831619	17	17	---	---	intergenic	---
2	127880220	127880221	-	127880220	10	20	---	---	intergenic	---
2	127982834	127982839	-	127982838	9	26	ENSSSCG00000021549	-	3'UTR	---
2	128099204	128099205	+	128099204	30	33	ENSSSCG00000014225	+	intron	---

2	128239750	128239751	-	128239751	5	9	---	---	intergenic	---
2	128543772	128543775	+	128543773	12	22	ENSSSCG00000027857	+	3'UTR	*DMXL1*
2	128723906	128723906	+	128723906	3	3	XLOC_048808	+	predicted	---
2	128766046	128766046	+	128766046	4	4	ENSSSCG00000028431	+	intron	*HSD17B4*
2	128770556	128770556	+	128770556	3	3	ENSSSCG00000028431	+	intron	*HSD17B4*
2	128783691	128783691	+	128783691	6	6	ENSSSCG00000028431	+	ORF	*HSD17B4*
2	128791956	128791956	+	128791956	3	3	ENSSSCG00000028431	+	ORF	*HSD17B4*
2	128795793	128795802	+	128795802	8	12	ENSSSCG00000028431	+	ORF	*HSD17B4*
2	128816143	128816143	+	128816143	4	4	XLOC_048809	+	predicted	---
2	128840743	128840743	+	128840743	4	4	XLOC_048809	+	predicted	---
2	128840801	128840815	+	128840801	7	11	XLOC_048809	+	predicted	---
2	128841051	128841079	+	128841058	29	141	XLOC_048809	+	predicted	---
2	128841214	128841215	+	128841214	3	6	XLOC_048809	+	predicted	---
2	128841297	128841345	+	128841337	51	147	XLOC_048809	+	predicted	---
2	128841411	128841433	+	128841431	24	43	XLOC_048809	+	predicted	---
2	128848244	128848244	+	128848244	3	3	XLOC_048809	+	predicted	---
2	128905654	128905655	+	128905654	3	6	XLOC_048810	+	predicted	---
2	128905693	128905693	+	128905693	5	5	XLOC_048810	+	predicted	---
2	129246638	129246638	-	129246638	3	3	---	---	intergenic	---
2	129798094	129798094	+	129798094	3	3	---	---	intergenic	---
2	129919166	129919166	-	129919166	3	3	---	---	intergenic	---
2	129944826	129944826	-	129944826	3	3	---	---	intergenic	---
2	130037867	130037869	+	130037869	7	12	---	---	intergenic	---
2	130051011	130051011	-	130051011	3	3	---	---	intergenic	---
2	130401355	130401355	-	130401355	3	3	---	---	intergenic	---
2	130470746	130470746	+	130470746	3	3	---	---	intergenic	---

2	130472221	130472221	-	130472221	5	5	---	---	intergenic	---
2	130685205	130685205	-	130685205	6	6	---	---	intergenic	---
2	130686554	130686580	+	130686573	7	25	XLOC_048818	+	predicted	---
2	130686756	130686763	+	130686761	55	91	XLOC_048818	+	predicted	---
2	130686965	130686965	+	130686965	4	4	XLOC_048818	+	predicted	---
2	130884265	130884266	-	130884266	10	14	XLOC_050334	-	predicted	---
2	130985836	130985836	+	130985836	3	3	ENSSSCG00000014233	+	intron	*ZNF474*
2	130985890	130985898	+	130985897	13	34	ENSSSCG00000014233	+	intron	*ZNF474*
2	131012673	131012686	+	131012675	282	967	ENSSSCG00000014233	+	3'UTR	*ZNF474*
2	131013021	131013021	+	131013021	5	5	ENSSSCG00000014233	+	3'UTR	*ZNF474*
2	131135093	131135093	+	131135093	3	3	XLOC_048823	+	predicted	---
2	131668150	131668155	+	131668155	11	22	ENSSSCG00000025977	+	3'UTR	*SNX24*
2	132182338	132182339	-	132182339	26	36	ENSSSCG00000014239	-	3'UTR	*CEP120*
2	132183372	132183372	-	132183372	3	3	ENSSSCG00000014239	-	3'UTR	*CEP120*
2	132188861	132188861	-	132188861	5	5	ENSSSCG00000014239	-	intron	*CEP120*
2	132330513	132330513	-	132330513	4	4	---	---	intergenic	---
2	132564429	132564429	-	132564429	4	4	---	---	intergenic	---
2	132824570	132824570	+	132824570	3	3	---	---	intergenic	---
2	132900078	132900078	-	132900078	5	5	---	---	intergenic	---
2	133035226	133035226	+	133035226	5	5	---	---	intergenic	---
2	133123041	133123041	+	133123041	4	4	---	---	intergenic	---
2	133203959	133203959	+	133203959	3	3	---	---	intergenic	---
2	133258437	133258437	-	133258437	3	3	ENSSSCG00000014242	-	intron	*ZNF608*
2	133295028	133295038	-	133295029	9	20	ENSSSCG00000014242	-	intron	*ZNF608*
2	133425533	133425534	-	133425534	6	9	---	---	intergenic	---
2	133843949	133843949	+	133843949	4	4	---	---	intergenic	---

2	133866797	133866797	-	133866797	6	6	---	---	intergenic	---
2	134098722	134098722	-	134098722	4	4	---	---	intergenic	---
2	134387151	134387151	-	134387151	3	3	---	---	intergenic	---
2	134530257	134530257	+	134530257	4	4	---	---	intergenic	---
2	134570124	134570124	+	134570124	4	4	---	---	intergenic	---
2	134934784	134934797	+	134934784	7	11	---	---	intergenic	---
2	134982783	134982783	+	134982783	3	3	ENSSSCG00000014247	+	intron	*ALDH7A1*
2	134986907	134986907	+	134986907	3	3	ENSSSCG00000014247	+	3'UTR	*ALDH7A1*
2	134988059	134988060	+	134988059	4	7	XLOC_048837	+	predicted	---
2	134988283	134988288	+	134988286	7	20	XLOC_048837	+	predicted	---
2	134990495	134990495	+	134990495	3	3	XLOC_048837	+	predicted	---
2	135022596	135022596	+	135022596	12	12	ENSSSCG00000014244	+	3'UTR	*PHAX*
2	135023061	135023065	+	135023065	16	24	ENSSSCG00000014244	+	3'UTR	*PHAX*
2	135029884	135029939	+	135029892	107	185	ENSSSCG00000027018	+	3'UTR	*TEX43*
2	135158938	135158938	+	135158938	5	5	ENSSSCG00000014248	+	3'UTR	*LMNB1*
2	135176985	135176985	-	135176985	7	7	ENSSSCG00000014249	-	3'UTR	*MARCH3*
2	135180692	135180694	-	135180694	7	12	ENSSSCG00000014249	-	intron	*MARCH3*
2	135452607	135452607	+	135452607	5	5	---	---	intergenic	---
2	135857250	135857252	+	135857250	13	25	---	---	intergenic	---
2	136010833	136010838	+	136010834	12	25	XLOC_048846	+	predicted	---
2	136031787	136031787	+	136031787	3	3	XLOC_048846	+	predicted	---
2	136209461	136209461	-	136209461	5	5	---	---	intergenic	---
2	136209500	136209500	+	136209500	3	3	---	---	intergenic	---
2	136291452	136291452	-	136291452	4	4	XLOC_050354	-	predicted	---
2	136356169	136356169	+	136356169	8	8	---	---	intergenic	---
2	136367671	136367671	+	136367671	4	4	XLOC_048847	+	predicted	---

2	136867829	136867850	-	136867850	8	11	---	---	intergenic	---
2	136867882	136867910	+	136867910	5	11	---	---	intergenic	---
2	136868542	136868544	+	136868542	7	14	---	---	intergenic	---
2	136868738	136868738	+	136868738	8	8	---	---	intergenic	---
2	136868788	136868799	-	136868788	3	6	---	---	intergenic	---
2	136868810	136868863	+	136868860	7042	11433	---	---	intergenic	---
2	136868869	136868869	-	136868869	7	7	---	---	intergenic	---
2	136868948	136868959	-	136868955	7	22	---	---	intergenic	---
2	136869005	136869005	+	136869005	4	4	---	---	intergenic	---
2	137219593	137219593	+	137219593	17	17	ENSSSCG00000014258	+	3'UTR	*ISOC1*
2	137219687	137219687	+	137219687	5	5	ENSSSCG00000014258	+	3'UTR	*ISOC1*
2	137498990	137498990	+	137498990	3	3	ENSSSCG00000014259	+	intron	---
2	138414257	138414257	-	138414257	6	6	XLOC_050364	-	predicted	---
2	138418098	138418100	-	138418098	4	8	---	---	intergenic	---
2	138581167	138581167	+	138581167	14	14	---	---	intergenic	---
2	138602897	138602897	-	138602897	3	3	---	---	intergenic	---
2	138632429	138632429	-	138632429	3	3	---	---	intergenic	---
2	138703604	138703604	+	138703604	7	7	ENSSSCG00000014264	+	ORF	*HINT1*
2	138705612	138705620	+	138705612	5	13	ENSSSCG00000014264	+	3'UTR	*HINT1*
2	138705699	138705710	+	138705708	657	1619	ENSSSCG00000014264	+	3'UTR	*HINT1*
2	138706949	138706952	+	138706950	12	34	ENSSSCG00000014264	+	3'UTR	*HINT1*
2	138794861	138794861	-	138794861	10	10	---	---	intergenic	---
2	138821276	138821276	-	138821276	3	3	---	---	intergenic	---
2	138829372	138829372	+	138829372	3	3	ENSSSCG00000014265	+	intron	*LYRM7*
2	138848251	138848251	+	138848251	3	3	ENSSSCG00000014265	+	intron	*LYRM7*
2	138848370	138848373	+	138848373	11	20	ENSSSCG00000014265	+	intron	*LYRM7*

2	138848505	138848512	+	138848505	3	12	ENSSSCG00000014265	+	intron	*LYRM7*
2	138857797	138857797	+	138857797	3	3	ENSSSCG00000014265	+	intron	*LYRM7*
2	138928201	138928202	+	138928201	12	15	---	---	intergenic	---
2	139002730	139002732	+	139002731	34	44	XLOC_048862	+	predicted	---
2	139027117	139027128	-	139027128	4	10	ENSSSCG00000014267	-	3'UTR	---
2	139165052	139165056	+	139165054	24	43	---	---	intergenic	---
2	139207761	139207762	+	139207762	9	14	---	---	intergenic	---
2	139228539	139228539	+	139228539	3	3	ENSSSCG00000014268	+	intron	---
2	139275934	139275942	-	139275936	25	47	ENSSSCG00000014269	-	3'UTR	---
2	139278550	139278550	-	139278550	5	5	ENSSSCG00000014269	-	3'UTR	---
2	139333552	139333552	-	139333552	3	3	ENSSSCG00000014269	-	intron	---
2	139483691	139483691	-	139483691	3	3	---	---	intergenic	---
2	139569527	139569538	-	139569538	18	34	ENSSSCG00000014272	-	3'UTR	---
2	139690391	139690391	-	139690391	7	7	---	---	intergenic	---
2	139696494	139696494	-	139696494	3	3	---	---	intergenic	---
2	139853794	139853803	-	139853801	22	51	ENSSSCG00000020915	-	3'UTR	*P4HA2*
2	139973934	139973934	+	139973934	3	3	ENSSSCG00000014274	+	3'UTR	*PDLIM4*
2	140218888	140218889	+	140218889	10	13	XLOC_048872	+	predicted	---
2	140229752	140229752	-	140229752	11	11	XLOC_050377	-	predicted	---
2	140229799	140229800	-	140229799	4	8	ENSSSCG00000014277	-	3'UTR	*IRF1*
2	140376085	140376102	+	140376102	6	9	ENSSSCG00000014280	+	intron	*RAD50*
2	140407464	140407468	+	140407466	15	28	ENSSSCG00000014280	+	3'UTR	*RAD50*
2	140471001	140471001	-	140471001	3	3	ENSSSCG00000014283	-	3'UTR	*KIF3A*
2	140550672	140550672	-	140550672	7	7	XLOC_050381	-	predicted	---
2	140552850	140552853	-	140552851	4	15	XLOC_050381	-	predicted	---
2	140610393	140610393	+	140610393	3	3	XLOC_048877	+	predicted	---

2	140616425	140616425	-	140616425	3	3	XLOC_050383	-	predicted	---
2	140616475	140616482	-	140616475	4	7	XLOC_050383	-	predicted	---
2	140649599	140649601	-	140649599	7	15	ENSSSCG00000014288	-	3'UTR	*GDF9*
2	140656478	140656486	+	140656484	37	97	ENSSSCG00000014289	+	3'UTR	*UQCRQ*
2	140656737	140656750	+	140656748	166	252	ENSSSCG00000014289	+	3'UTR	*UQCRQ*
2	140663865	140663868	+	140663868	11	19	ENSSSCG00000014290	+	3'UTR	*LEAP2*
2	140664070	140664070	+	140664070	4	4	ENSSSCG00000014290	+	3'UTR	*LEAP2*
2	140664341	140664342	+	140664341	7	14	XLOC_048880	+	predicted	---
2	140664612	140664616	+	140664612	9	24	XLOC_048880	+	predicted	---
2	140664761	140664764	+	140664762	30	49	XLOC_048880	+	predicted	---
2	140664973	140664973	+	140664973	5	5	XLOC_048880	+	predicted	---
2	140670097	140670130	-	140670130	9	21	XLOC_050385	-	predicted	---
2	140782899	140782899	-	140782899	3	3	ENSSSCG00000014291	-	intron	*AFF4*
2	140806197	140806204	+	140806204	17	34	XLOC_048881	+	predicted	---
2	140849057	140849057	+	140849057	4	4	ENSSSCG00000023344	+	3'UTR	*ZCCHC10*
2	140864868	140864868	+	140864868	25	25	---	---	intergenic	---
2	140888984	140888984	+	140888984	3	3	ENSSSCG00000014292	+	ORF	*HSPA4*
2	140894087	140894087	+	140894087	6	6	ENSSSCG00000014292	+	intron	*HSPA4*
2	140895094	140895096	+	140895094	6	13	ENSSSCG00000014292	+	intron	*HSPA4*
2	140898700	140898700	+	140898700	3	3	ENSSSCG00000014292	+	ORF	*HSPA4*
2	140903803	140903803	+	140903803	6	6	ENSSSCG00000014292	+	ORF	*HSPA4*
2	140909524	140909524	+	140909524	5	5	ENSSSCG00000014292	+	ORF	*HSPA4*
2	140909713	140909726	+	140909718	37	88	ENSSSCG00000014292	+	3'UTR	*HSPA4*
2	140911629	140911634	+	140911633	31	37	ENSSSCG00000014292	+	3'UTR	*HSPA4*
2	141029707	141029709	-	141029707	4	8	ENSSSCG00000014293	-	intron	*FSTL4*
2	141861116	141861118	-	141861117	24	39	ENSSSCG00000014295	-	3'UTR	*C5orf15*

2	141876797	141876805	-	141876805	30	67	ENSSSCG00000014296	-	3'UTR	*VDAC1*
2	141893165	141893175	-	141893166	7	19	ENSSSCG00000014296	-	ORF	*VDAC1*
2	142096654	142096654	-	142096654	3	3	---	---	intergenic	---
2	142103585	142103585	-	142103585	4	4	---	---	intergenic	---
2	142151765	142151769	+	142151765	6	9	XLOC_048892	+	predicted	---
2	142216298	142216301	-	142216299	153	222	ENSSSCG00000014302	-	intron	*CDKL3*
2	142218076	142218087	-	142218076	3	9	ENSSSCG00000014302	-	intron	*CDKL3*
2	142248355	142248355	-	142248355	9	9	ENSSSCG00000014302	-	intron	*CDKL3*
2	142249336	142249357	-	142249351	40	81	ENSSSCG00000014302	-	intron	*CDKL3*
2	142446231	142446231	+	142446231	9	9	ENSSSCG00000014303	+	3'UTR	*JADE2*
2	142458801	142458801	-	142458801	23	23	XLOC_050403	-	predicted	---
2	142463680	142463699	-	142463693	217	718	ENSSSCG00000014305	-	3'UTR	*SAR1B*
2	142565794	142565805	+	142565800	6	13	XLOC_048899	+	predicted	---
2	142566325	142566325	+	142566325	3	3	XLOC_048899	+	predicted	---
2	142568256	142568265	+	142568256	26	53	XLOC_048899	+	predicted	---
2	142579657	142579657	-	142579657	11	11	---	---	intergenic	---
2	142584025	142584026	+	142584025	16	21	XLOC_048900	+	predicted	---
2	142596918	142596944	+	142596944	14	17	ENSSSCG00000026644	+	intron	*DDX46*
2	142597425	142597437	+	142597425	7	10	ENSSSCG00000026644	+	ORF	*DDX46*
2	142600559	142600559	+	142600559	3	3	ENSSSCG00000026644	+	ORF	*DDX46*
2	142644667	142644667	+	142644667	3	3	ENSSSCG00000026644	+	3'UTR	*DDX46*
2	142646408	142646408	+	142646408	7	7	ENSSSCG00000026644	+	3'UTR	*DDX46*
2	142652413	142652413	-	142652413	9	9	---	---	intergenic	---
2	142665521	142665528	+	142665521	3	6	XLOC_048901	+	predicted	---
2	142675565	142675573	-	142675569	52	95	XLOC_050407	-	predicted	---
2	142695360	142695369	+	142695368	14	53	ENSSSCG00000026106	+	3'UTR	*TXNDC15*

2	142735477	142735478	-	142735477	3	6	---	---	intergenic	---
2	143054433	143054444	-	143054436	36	94	ENSSSCG00000014307	-	3'UTR	*H2AFY*
2	143054513	143054514	-	143054514	7	11	ENSSSCG00000014307	-	3'UTR	*H2AFY*
2	143186460	143186460	+	143186460	10	10	---	---	intergenic	---
2	143452489	143452498	+	143452492	5	11	ENSSSCG00000014310	+	3'UTR	*CXCL14*
2	143453540	143453543	+	143453540	3	6	ENSSSCG00000014310	+	3'UTR	*CXCL14*
2	143576235	143576238	+	143576238	5	8	---	---	intergenic	---
2	143594721	143594721	+	143594721	3	3	ENSSSCG00000014311	+	intron	*SLC25A48*
2	143634933	143634934	+	143634934	9	13	ENSSSCG00000014311	+	3'UTR	*SLC25A48*
2	143652209	143652211	-	143652209	24	27	---	---	intergenic	---
2	143773797	143773805	-	143773797	3	6	XLOC_050417	-	predicted	---
2	143773979	143773992	-	143773987	161	273	ENSSSCG00000014315	-	3'UTR	*LECT2*
2	143827400	143827400	-	143827400	3	3	---	---	intergenic	---
2	143911335	143911338	+	143911335	5	10	---	---	intergenic	---
2	144011089	144011089	-	144011089	3	3	ENSSSCG00000014318	-	intron	*TRPM2*
2	144016321	144016322	-	144016321	4	7	ENSSSCG00000014319	-	3'UTR	*SMAD5*
							ENSSSCG00000014318	-	intron	*TRPM2*
2	144718320	144718320	-	144718320	3	3	XLOC_050424	-	predicted	---
2	145353777	145353786	-	145353785	19	30	XLOC_050426	-	predicted	---
2	145501462	145501464	-	145501464	16	24	XLOC_050426	-	predicted	---
2	145502517	145502519	-	145502517	9	19	XLOC_050426	-	predicted	---
2	145560252	145560252	-	145560252	5	5	---	---	intergenic	---
2	145632298	145632300	+	145632299	17	37	ENSSSCG00000014324	+	intron	*MYOT*
2	145637695	145637697	+	145637696	6	13	ENSSSCG00000014324	+	intron	*MYOT*
2	145642081	145642088	+	145642081	18	21	ENSSSCG00000014324	+	3'UTR	*MYOT*
2	145668056	145668056	+	145668056	7	7	---	---	intergenic	---

2	145681669	145681693	+	145681669	38	103	ENSSSCG00000021604	+	intron	---
2	145681934	145681939	+	145681939	16	25	ENSSSCG00000021604	+	3'UTR	---
2	145682035	145682035	+	145682035	5	5	XLOC_048917	+	predicted	---
2	145682641	145682641	-	145682641	4	4	---	---	intergenic	---
2	145682858	145682858	+	145682858	3	3	XLOC_048917	+	predicted	---
2	145692736	145692736	-	145692736	7	7	ENSSSCG00000029125	-	intron	*FAM13B*
2	145718180	145718180	-	145718180	3	3	ENSSSCG00000029125	-	intron	*FAM13B*
2	145776064	145776064	-	145776064	3	3	---	---	intergenic	---
2	145800463	145800470	-	145800468	66	153	---	---	intergenic	---
2	145800486	145800486	+	145800486	3	3	---	---	intergenic	---
2	145845293	145845293	-	145845293	4	4	ENSSSCG00000027997	-	intron	*NME5*
2	145866441	145866447	-	145866442	20	37	XLOC_050430	-	predicted	---
2	145893411	145893411	-	145893411	28	28	ENSSSCG00000014327	-	ORF	*BRD8*
2	145910491	145910491	-	145910491	3	3	ENSSSCG00000014325	-	3'UTR	*CDC23*
2	145962147	145962147	-	145962147	5	5	ENSSSCG00000014328	-	3'UTR	*GFRA3*
2	146029054	146029054	+	146029054	3	3	XLOC_048920	+	predicted	---
2	146107159	146107159	+	146107159	4	4	ENSSSCG00000014331	+	intron	*KDM3B*
2	146139729	146139739	-	146139739	7	14	XLOC_050433	-	predicted	---
2	146139817	146139819	-	146139817	4	7	XLOC_050433	-	predicted	---
2	146141786	146141789	+	146141787	103	141	---	---	intergenic	---
2	146159946	146159947	+	146159946	4	7	ENSSSCG00000014336	+	3'UTR	*EGR1*
							ENSSSCG00000014336	+	ORF	---
2	146181227	146181231	-	146181227	9	12	---	---	intergenic	---
2	146186592	146186595	-	146186592	8	15	ENSSSCG00000014337	-	3'UTR	*ETF1*
2	146188285	146188291	-	146188289	24	41	ENSSSCG00000014337	-	3'UTR	*ETF1*
2	146188409	146188411	-	146188409	9	19	ENSSSCG00000014337	-	3'UTR	*ETF1*

2	146230068	146230088	-	146230088	112	192	ENSSSCG00000014338	-	3'UTR	*HSPA9*
2	146230521	146230539	-	146230535	13	52	ENSSSCG00000014338	-	3'UTR	*HSPA9*
2	146230760	146230760	-	146230760	4	4	ENSSSCG00000014338	-	ORF	*HSPA9*
2	146231425	146231425	-	146231425	13	13	ENSSSCG00000014338	-	intron	*HSPA9*
2	146426635	146426635	+	146426635	3	3	---	---	intergenic	---
2	146453111	146453116	+	146453116	6	13	ENSSSCG00000014339	+	intron	*CTNNA1*
2	146624707	146624722	+	146624718	46	130	ENSSSCG00000014339	+	3'UTR	*CTNNA1*
2	146630014	146630028	+	146630018	196	364	XLOC_048927	+	predicted	---
2	146967489	146967489	+	146967489	6	6	---	---	intergenic	---
2	147041283	147041283	+	147041283	3	3	ENSSSCG00000014343	+	ORF	---
2	147054015	147054015	+	147054015	3	3	ENSSSCG00000014343	+	ORF	---
2	147054241	147054242	+	147054242	15	24	ENSSSCG00000014343	+	ORF	---
2	147059906	147059906	+	147059906	3	3	XLOC_048930	+	predicted	---
2	147060195	147060204	+	147060195	10	27	XLOC_048930	+	predicted	---
2	147060827	147060860	+	147060855	14	60	XLOC_048930	+	predicted	---
2	147060973	147060973	+	147060973	3	3	XLOC_048930	+	predicted	---
2	147085174	147085180	+	147085174	3	6	ENSSSCG00000026606	+	ORF	*PAIP2*
2	147088928	147088932	-	147088929	5	11	ENSSSCG00000026423	-	3'UTR	*SLC23A1*
2	147089986	147089986	+	147089986	7	7	ENSSSCG00000026606	+	3'UTR	*PAIP2*
2	147090031	147090040	+	147090035	89	154	ENSSSCG00000026606	+	3'UTR	*PAIP2*
2	147090805	147090813	+	147090812	45	71	ENSSSCG00000026606	+	3'UTR	*PAIP2*
2	147108146	147108149	-	147108148	56	65	ENSSSCG00000029239	-	3'UTR	*MZB1*
2	147117483	147117489	-	147117483	8	18	XLOC_050446	-	predicted	---
2	147117879	147117902	-	147117896	72	216	ENSSSCG00000014348	-	3'UTR	*SPATA24*
2	147117980	147117982	-	147117980	4	8	ENSSSCG00000014348	-	ORF	*SPATA24*
2	147121151	147121184	-	147121174	62	163	ENSSSCG00000014348	-	intron	*SPATA24*

2	147153637	147153638	-	147153638	8	11	ENSSSCG00000014351	-	3'UTR	---
2	147169471	147169471	-	147169471	4	4	ENSSSCG00000014349	-	3'UTR	*TMEM173*
2	147196056	147196069	-	147196065	173	286	XLOC_050450	-	predicted	---
2	147212090	147212090	-	147212090	3	3	ENSSSCG00000014356	-	ORF	*DNAJC18*
2	147320700	147320700	+	147320700	3	3	ENSSSCG00000014357	+	3'UTR	*CXXC5*
2	147321654	147321658	+	147321657	29	46	ENSSSCG00000014357	+	3'UTR	*CXXC5*
2	147425442	147425452	-	147425443	13	35	ENSSSCG00000026374	-	3'UTR	*UBE2D2*
2	147425895	147425916	-	147425904	16	28	ENSSSCG00000026374	-	3'UTR	*UBE2D2*
2	147426267	147426279	-	147426279	29	44	ENSSSCG00000026374	-	3'UTR	*UBE2D2*
2	147442231	147442231	+	147442231	6	6	---	---	intergenic	---
2	147763225	147763225	+	147763225	3	3	---	---	intergenic	---
2	147802781	147802781	+	147802781	3	3	XLOC_048947	+	predicted	---
2	147905746	147905746	+	147905746	6	6	ENSSSCG00000014361	+	intron	*CYSTM1*
2	147935433	147935439	+	147935433	10	18	ENSSSCG00000014361	+	intron	*CYSTM1*
2	147935580	147935585	+	147935584	23	60	ENSSSCG00000014361	+	intron	*CYSTM1*
2	147953343	147953348	+	147953345	195	261	ENSSSCG00000014361	+	3'UTR	*CYSTM1*
2	147990662	147990662	-	147990662	3	3	ENSSSCG00000030422	-	ORF	*PFDN1*
2	147994221	147994221	-	147994221	3	3	ENSSSCG00000030422	-	intron	*PFDN1*
2	148177404	148177404	+	148177404	8	8	ENSSSCG00000014364	+	intron	*ANKHD1*
2	148241941	148241946	+	148241941	4	11	XLOC_048951	+	predicted	---
2	148249556	148249557	+	148249556	27	31	ENSSSCG00000020835	+	3'UTR	*EIF4EBP3*
2	148250848	148250892	-	148250873	20	114	XLOC_050468	-	predicted	---
2	148257917	148257917	-	148257917	16	16	XLOC_050468	-	predicted	---
2	148269170	148269178	+	148269177	18	54	XLOC_048951	+	predicted	---
2	148269544	148269544	-	148269544	6	6	XLOC_050468	-	predicted	---
2	148270492	148270492	-	148270492	3	3	XLOC_050468	-	predicted	---

2	148291969	148291986	-	148291974	558	832	ENSSSCG00000014369	-	3'UTR	*CD14*
							ENSSSCG00000014369	-	ORF	*CD14*
2	148293909	148293909	+	148293909	4	4	---	---	intergenic	---
2	148302949	148302950	-	148302950	26	29	ENSSSCG00000014371	-	3'UTR	---
2	148302982	148302982	+	148302982	5	5	ENSSSCG00000014370	+	3'UTR	*TMCO6*
2	148303052	148303063	-	148303061	140	295	ENSSSCG00000014371	-	3'UTR	---
2	148311527	148311527	+	148311527	4	4	ENSSSCG00000014372	+	intron	*WDR55*
2	148320506	148320517	+	148320513	103	262	ENSSSCG00000014372	+	3'UTR	*WDR55*
							ENSSSCG00000014372	+	intron	*WDR55*
2	148328709	148328709	-	148328709	3	3	XLOC_050471	-	predicted	---
2	148331743	148331753	-	148331744	39	98	ENSSSCG00000014375	-	3'UTR	*HARS*
2	148332013	148332013	-	148332013	3	3	ENSSSCG00000014375	-	3'UTR	*HARS*
2	148360951	148360968	+	148360967	12	22	ENSSSCG00000029158	+	3'UTR	*ZMAT2*
2	148361340	148361348	+	148361347	10	18	ENSSSCG00000029158	+	3'UTR	*ZMAT2*
2	148361578	148361606	+	148361598	7	31	XLOC_048953	+	predicted	---
2	148364729	148364729	+	148364729	3	3	ENSSSCG00000023841	+	ORF	*Vault*
2	148530891	148530891	-	148530891	3	3	---	---	intergenic	---
2	148811576	148811576	-	148811576	4	4	---	---	intergenic	---
2	149090501	149090501	-	149090501	3	3	XLOC_050482	-	predicted	---
2	149119741	149119755	+	149119755	6	12	---	---	intergenic	---
2	149149391	149149397	-	149149392	25	34	XLOC_050488	-	predicted	---
2	149149979	149149979	-	149149979	3	3	ENSSSCG00000028168	-	ORF	*TAF7*
2	149150541	149150541	-	149150541	3	3	ENSSSCG00000028168	-	ORF	*TAF7*
2	149197493	149197500	-	149197500	4	7	---	---	intergenic	---
2	149209936	149209939	-	149209937	15	37	XLOC_050491	-	predicted	---
2	149236536	149236536	-	149236536	3	3	---	---	intergenic	---

2	149335853	149335853	+	149335853	3	3	XLOC_048968	+	predicted	---
2	149475764	149475767	-	149475766	35	43	ENSSSCG00000014388	-	3'UTR	*HDAC3*
2	149506700	149506700	-	149506700	4	4	ENSSSCG00000014391	-	3'UTR	*ARAP3*
2	149651501	149651501	-	149651501	3	3	---	---	intergenic	---
2	149679041	149679041	-	149679041	3	3	XLOC_050501	-	predicted	---
2	149747347	149747355	+	149747354	25	71	ENSSSCG00000014396	+	3'UTR	*KIAA0141*
2	149752325	149752325	-	149752325	3	3	ENSSSCG00000014395	-	3'UTR	*PCDH12*
2	149781639	149781641	+	149781639	20	26	ENSSSCG00000014394	+	intron	*RNF14*
2	149795996	149795998	+	149795997	26	66	ENSSSCG00000014394	+	3'UTR	*RNF14*
2	149797094	149797106	+	149797100	17	38	XLOC_048979	+	predicted	---
2	149805874	149805874	-	149805874	5	5	ENSSSCG00000014393	-	3'UTR	*GNPDA1*
2	149807065	149807076	-	149807066	26	66	ENSSSCG00000014393	-	3'UTR	*GNPDA1*
2	149997968	149997968	-	149997968	4	4	---	---	intergenic	---
2	150159654	150159673	+	150159664	15	81	---	---	intergenic	---
2	150206761	150206761	-	150206761	6	6	---	---	intergenic	---
2	150206794	150206794	+	150206794	7	7	---	---	intergenic	---
2	150274575	150274575	-	150274575	3	3	---	---	intergenic	---
2	150469982	150469982	-	150469982	3	3	---	---	intergenic	---
2	150955355	150955355	+	150955355	4	4	XLOC_048985	+	predicted	---
2	151032190	151032190	-	151032190	10	10	---	---	intergenic	---
2	151051816	151051817	-	151051817	9	12	XLOC_050514	-	predicted	---
2	151053543	151053543	-	151053543	3	3	XLOC_050514	-	predicted	---
2	151055192	151055192	-	151055192	4	4	ENSSSCG00000014401	-	3'UTR	*NR3C1*
2	151079057	151079058	-	151079057	4	7	---	---	intergenic	---
2	151195573	151195574	-	151195573	6	9	---	---	intergenic	---
2	151347949	151347957	+	151347957	13	17	XLOC_048989	+	predicted	---

2	151609774	151609774	+	151609774	13	13	---	---	intergenic	---
2	151998702	151998702	+	151998702	3	3	---	---	intergenic	---
2	152318283	152318283	+	152318283	3	3	---	---	intergenic	---
2	152409261	152409261	+	152409261	3	3	---	---	intergenic	---
2	152529970	152529970	-	152529970	3	3	---	---	intergenic	---
2	152931296	152931296	-	152931296	12	12	---	---	intergenic	---
2	153045891	153045891	-	153045891	3	3	---	---	intergenic	---
2	153350976	153351001	-	153350994	76	693	---	---	intergenic	---
2	153519344	153519344	-	153519344	5	5	XLOC_050518	-	predicted	---
2	153671507	153671507	-	153671507	6	6	---	---	intergenic	---
2	153827031	153827031	+	153827031	5	5	ENSSSCG00000014410	+	intron	---
2	153830762	153830769	-	153830768	26	34	ENSSSCG00000014411	-	3'UTR	---
2	153957808	153957816	-	153957810	50	118	XLOC_050525	-	predicted	---
2	154072785	154072785	+	154072785	3	3	ENSSSCG00000014409	+	3'UTR	---
2	154073069	154073081	+	154073069	3	9	ENSSSCG00000014409	+	3'UTR	---
2	154075885	154075885	+	154075885	12	12	ENSSSCG00000014409	+	3'UTR	---
2	154235713	154235714	+	154235713	12	19	---	---	intergenic	---
2	154298920	154298924	+	154298924	8	21	ENSSSCG00000014416	+	intron	*TCERG1*
2	154299012	154299026	+	154299012	6	15	ENSSSCG00000014416	+	intron	*TCERG1*
2	154341434	154341434	+	154341434	3	3	ENSSSCG00000014416	+	ORF	*TCERG1*
2	154345070	154345080	+	154345079	12	27	ENSSSCG00000014416	+	3'UTR	*TCERG1*
2	154429929	154429929	-	154429929	11	11	XLOC_050530	-	predicted	---
2	154430314	154430315	-	154430315	5	9	XLOC_050530	-	predicted	---
2	154440882	154440882	-	154440882	4	4	ENSSSCG00000028976	-	intron	*PPP2R2B*
2	155000595	155000595	+	155000595	3	3	---	---	intergenic	---
2	155540636	155540636	-	155540636	3	3	---	---	intergenic	---

2	155635045	155635045	-	155635045	6	6	XLOC_050537	-	predicted	---
2	155749563	155749563	-	155749563	3	3	---	---	intergenic	---
2	156102003	156102003	+	156102003	3	3	---	---	intergenic	---
2	156299474	156299474	+	156299474	3	3	---	---	intergenic	---
2	156351720	156351738	+	156351726	12	25	XLOC_049013	+	predicted	---
2	156406036	156406036	+	156406036	3	3	XLOC_049014	+	predicted	---
2	156409223	156409223	-	156409223	3	3	XLOC_050548	-	predicted	---
2	156773897	156773897	-	156773897	7	7	---	---	intergenic	---
2	157186528	157186531	+	157186529	11	34	XLOC_049017	+	predicted	---
2	157283111	157283111	+	157283111	3	3	XLOC_049019	+	predicted	---
2	157285900	157285900	+	157285900	7	7	XLOC_049019	+	predicted	---
2	157378848	157378848	-	157378848	9	9	XLOC_050558	-	predicted	---
2	157379066	157379068	-	157379067	11	19	XLOC_050558	-	predicted	---
2	157379477	157379479	-	157379477	4	7	XLOC_050558	-	predicted	---
2	157380374	157380381	-	157380374	7	12	XLOC_050558	-	predicted	---
2	157380458	157380459	-	157380459	5	9	XLOC_050558	-	predicted	---
2	157381009	157381017	-	157381017	7	17	XLOC_050558	-	predicted	---
2	157386700	157386735	-	157386701	61	107	XLOC_050558	-	predicted	---
2	157393711	157393712	-	157393711	39	47	ENSSSCG00000014435	-	intron	*CSNK1A1*
2	157504225	157504225	+	157504225	3	3	XLOC_049023	+	predicted	---
2	157662537	157662537	-	157662537	5	5	---	---	intergenic	---
2	157954696	157954699	-	157954698	20	34	ENSSSCG00000014442	-	intron	*PDGFRB*
2	158335777	158335777	-	158335777	3	3	XLOC_050580	-	predicted	---
2	158453424	158453430	-	158453427	107	145	ENSSSCG00000014450	-	intron	*TCOF1*
2	158453549	158453562	-	158453562	4	10	ENSSSCG00000014450	-	ORF	*TCOF1*
2	158546123	158546124	-	158546123	5	10	---	---	intergenic	---

2	158565101	158565102	+	158565102	23	26	---	---	intergenic	---
2	158624787	158624788	-	158624788	32	46	---	---	intergenic	---
2	158672462	158672462	+	158672462	10	10	---	---	intergenic	---
2	158939986	158939986	+	158939986	4	4	---	---	intergenic	---
2	158945080	158945080	-	158945080	4	4	---	---	intergenic	---
2	158972655	158972655	+	158972655	3	3	---	---	intergenic	---
2	159092570	159092570	-	159092570	10	10	---	---	intergenic	---
2	159753275	159753278	+	159753277	30	48	ENSSSCG00000014538	+	3'UTR	*MGAT1*
							ENSSSCG00000014538	+	ORF	---
2	160011693	160011693	+	160011693	5	5	---	---	intergenic	---
2	160441278	160441278	-	160441278	3	3	XLOC_050631	-	predicted	---
2	161512710	161512714	-	161512710	3	6	---	---	intergenic	---
2	162076670	162076670	-	162076670	5	5	---	---	intergenic	---
2	162099159	162099160	-	162099159	8	12	ENSSSCG00000014564	-	3'UTR	*IFITM1*
2	162155476	162155476	+	162155476	12	12	ENSSSCG00000014560	+	3'UTR	*COX8H*
2	162155821	162155825	-	162155823	51	90	ENSSSCG00000014559	-	3'UTR	*PSMD13*
							ENSSSCG00000014564	-	intron	*IFITM1*
2	162186201	162186214	+	162186214	16	23	ENSSSCG00000014558	+	3'UTR	*SIRT3*
2	162186997	162187006	-	162187006	11	21	ENSSSCG00000014557	-	3'UTR	*RIC8A*
							ENSSSCG00000014564	-	intron	*IFITM1*
2	162187082	162187082	-	162187082	3	3	ENSSSCG00000014557	-	3'UTR	*RIC8A*
							ENSSSCG00000014564	-	intron	*IFITM1*
2	162199364	162199371	-	162199368	22	76	ENSSSCG00000014564	-	intron	*IFITM1*
2	162285395	162285396	-	162285396	18	26	ENSSSCG00000024389	-	3'UTR	---
2	162339712	162339712	-	162339712	22	22	---	---	intergenic	---
3	94197	94207	+	94203	28	84	XLOC_054569	+	predicted	---

3	190916	190916	-	190916	3	3	ENSSSCG00000007542	-	3'UTR	---
3	191879	191879	-	191879	3	3	ENSSSCG00000007542	-	3'UTR	---
3	209785	209785	-	209785	5	5	ENSSSCG00000007542	-	intron	---
3	727189	727197	+	727189	27	30	ENSSSCG00000007546	+	intron	*C7orf50*
3	743054	743077	+	743075	33	153	ENSSSCG00000007546	+	3'UTR	*C7orf50*
3	757758	757758	+	757758	3	3	XLOC_054581	+	predicted	---
3	763222	763222	+	763222	6	6	XLOC_054581	+	predicted	---
3	810687	810694	+	810687	5	11	XLOC_054581	+	predicted	---
3	873586	873592	-	873587	128	142	ENSSSCG00000007554	-	3'UTR	*ZFAND2A*
3	1066653	1066653	-	1066653	4	4	XLOC_055844	-	predicted	---
3	1094039	1094048	-	1094039	3	9	ENSSSCG00000007558	-	3'UTR	---
3	1149710	1149710	+	1149710	5	5	XLOC_054586	+	predicted	---
3	1167043	1167056	-	1167044	14	55	XLOC_055848	-	predicted	---
3	1374029	1374029	+	1374029	4	4	XLOC_054591	+	predicted	---
3	1466074	1466074	+	1466074	3	3	XLOC_054592	+	predicted	---
3	2012022	2012024	+	2012022	3	6	ENSSSCG00000023433	+	intron	*GNA12*
3	2012091	2012091	+	2012091	4	4	ENSSSCG00000023433	+	intron	*GNA12*
3	2020481	2020481	+	2020481	3	3	ENSSSCG00000023433	+	3'UTR	*GNA12*
3	2124280	2124286	-	2124280	6	9	---	---	intergenic	---
3	2147251	2147251	+	2147251	3	3	ENSSSCG00000007568	+	ORF	*IQCE*
3	2162720	2162721	+	2162720	15	19	XLOC_054597	+	predicted	---
3	2163250	2163250	+	2163250	5	5	XLOC_054597	+	predicted	---
3	2165603	2165608	+	2165607	4	10	XLOC_054597	+	predicted	---
3	2201171	2201171	+	2201171	18	18	XLOC_054598	+	predicted	---
3	2208310	2208312	-	2208312	19	26	ENSSSCG00000007572	-	3'UTR	*LFNG*
3	2218149	2218149	-	2218149	3	3	XLOC_055854	-	predicted	---

3	2284919	2284919	-	2284919	4	4	XLOC_055855	-	predicted	---
3	2964743	2964743	+	2964743	3	3	---	---	intergenic	---
3	3407483	3407483	-	3407483	3	3	---	---	intergenic	---
3	3526726	3526726	-	3526726	8	8	---	---	intergenic	---
3	3695759	3695759	+	3695759	5	5	ENSSSCG00000007574	+	intron	*SDK1*
3	4007130	4007130	-	4007130	3	3	---	---	intergenic	---
3	4068104	4068104	-	4068104	10	10	---	---	intergenic	---
3	4126436	4126437	+	4126437	5	8	---	---	intergenic	---
3	4191087	4191087	-	4191087	5	5	---	---	intergenic	---
3	4191362	4191362	-	4191362	52	52	---	---	intergenic	---
3	4289831	4289843	+	4289842	7	23	ENSSSCG00000007577	+	3'UTR	*AP5Z1*
3	4292993	4292993	+	4292993	3	3	ENSSSCG00000007577	+	3'UTR	*AP5Z1*
3	4347042	4347064	-	4347042	3	12	ENSSSCG00000007575	-	intron	*RADIL*
3	4348700	4348713	-	4348700	3	6	ENSSSCG00000007575	-	intron	*RADIL*
3	4349085	4349086	-	4349085	7	12	ENSSSCG00000007575	-	intron	*RADIL*
3	4517666	4517666	+	4517666	3	3	ENSSSCG00000007580	+	3'UTR	*WIPI2*
3	4517982	4517989	+	4517989	24	34	ENSSSCG00000007580	+	3'UTR	*WIPI2*
3	4534761	4534761	+	4534761	3	3	---	---	intergenic	---
3	4686635	4686635	-	4686635	3	3	XLOC_055871	-	predicted	---
3	4729273	4729290	-	4729285	2616	3087	ENSSSCG00000007585	-	3'UTR	*BETA-ACTIN*
3	4729415	4729426	-	4729424	13	26	ENSSSCG00000007585	-	3'UTR	*BETA-ACTIN*
3	4729709	4729709	-	4729709	41	41	ENSSSCG00000007585	-	3'UTR	*BETA-ACTIN*
3	4729803	4729803	-	4729803	16	16	ENSSSCG00000007585	-	3'UTR	*BETA-ACTIN*
3	4729848	4729885	-	4729869	18	31	ENSSSCG00000007585	-	3'UTR	*BETA-ACTIN*
3	4729932	4729932	-	4729932	5	5	ENSSSCG00000007585	-	ORF	*BETA-ACTIN*
3	4729988	4729988	-	4729988	3	3	ENSSSCG00000007585	-	ORF	*BETA-ACTIN*

3	4731387	4731387	-	4731387	13	13	ENSSSCG00000007585	-	ORF	*BETA-ACTIN*
3	5130820	5130820	+	5130820	4	4	XLOC_054633	+	predicted	---
3	5170735	5170741	+	5170741	11	29	XLOC_054634	+	predicted	---
3	5170739	5170742	-	5170741	35	61	ENSSSCG00000024245	-	3'UTR	*AIMP2*
3	5206750	5206750	+	5206750	7	7	ENSSSCG00000007590	+	3'UTR	*PMS2*
3	5252673	5252675	+	5252674	33	40	ENSSSCG00000007589	+	ORF	---
3	5260116	5260119	+	5260116	7	23	XLOC_054635	+	predicted	---
3	5262068	5262069	-	5262069	18	23	ENSSSCG00000025940	-	3'UTR	---
3	5295458	5295458	+	5295458	3	3	---	---	intergenic	---
3	5334102	5334103	+	5334102	5	8	---	---	intergenic	---
3	5382122	5382122	-	5382122	12	12	---	---	intergenic	---
3	5439914	5439915	-	5439915	8	11	---	---	intergenic	---
3	5473365	5473365	-	5473365	5	5	ENSSSCG00000007602	-	intron	*BAIAP2L1*
3	5510055	5510055	-	5510055	4	4	ENSSSCG00000007602	-	intron	*BAIAP2L1*
3	5510283	5510283	-	5510283	6	6	ENSSSCG00000007602	-	intron	*BAIAP2L1*
3	5518356	5518356	-	5518356	3	3	ENSSSCG00000007602	-	intron	*BAIAP2L1*
3	5527687	5527692	-	5527687	6	9	ENSSSCG00000007602	-	intron	*BAIAP2L1*
3	5528156	5528156	-	5528156	4	4	ENSSSCG00000007602	-	3'UTR	*BAIAP2L1*
3	5675905	5675905	+	5675905	8	8	XLOC_054641	+	predicted	---
3	5740843	5740856	+	5740843	9	22	XLOC_054643	+	predicted	---
3	5741746	5741752	+	5741752	6	11	XLOC_054643	+	predicted	---
3	5754512	5754513	-	5754513	8	13	---	---	intergenic	---
3	5922497	5922497	+	5922497	5	5	---	---	intergenic	---
3	6082942	6082942	+	6082942	5	5	ENSSSCG00000007606	+	intron	*TRRAP*
3	6087736	6087740	+	6087740	4	7	ENSSSCG00000007606	+	3'UTR	*TRRAP*
3	6098691	6098691	-	6098691	3	3	ENSSSCG00000030153	-	3'UTR	---

3	6532594	6532601	-	6532595	10	21	XLOC_055908	-	predicted	---
3	6543819	6543826	-	6543819	4	11	ENSSSCG00000007617	-	intron	*ZNF655*
3	6582290	6582297	+	6582290	4	8	ENSSSCG00000007618	+	intron	---
3	6588208	6588208	+	6588208	3	3	ENSSSCG00000007618	+	intron	---
3	6588828	6588833	+	6588828	16	19	ENSSSCG00000007618	+	intron	---
3	6589061	6589064	-	6589064	5	8	ENSSSCG00000022023	-	3'UTR	*ZKSCAN5*
3	6589795	6589795	-	6589795	3	3	ENSSSCG00000022023	-	3'UTR	*ZKSCAN5*
3	6623659	6623660	+	6623659	11	18	XLOC_054657	+	predicted	---
3	6693611	6693622	-	6693614	96	250	ENSSSCG00000007623	-	3'UTR	*BUD31*
3	6694106	6694106	+	6694106	3	3	ENSSSCG00000007622	+	3'UTR	---
3	6704149	6704149	+	6704149	3	3	ENSSSCG00000023257	+	intron	*PDAP1*
3	6708327	6708333	+	6708329	21	67	ENSSSCG00000023257	+	ORF	*PDAP1*
3	6710995	6710997	+	6710997	28	36	ENSSSCG00000023257	+	3'UTR	*PDAP1*
3	7095616	7095616	+	7095616	3	3	XLOC_054666	+	predicted	---
3	7129548	7129600	+	7129587	189	881	XLOC_054668	+	predicted	---
3	7131112	7131112	+	7131112	3	3	XLOC_054668	+	predicted	---
3	7144795	7144795	+	7144795	3	3	---	---	intergenic	---
3	7249306	7249308	+	7249307	16	23	XLOC_054670	+	predicted	---
3	7346257	7346257	-	7346257	3	3	---	---	intergenic	---
3	7364011	7364013	+	7364011	13	19	ENSSSCG00000007647	+	3'UTR	*C7orf43*
3	7364231	7364241	-	7364234	26	116	ENSSSCG00000007646	-	3'UTR	*LAMTOR4*
3	7414243	7414243	+	7414243	4	4	---	---	intergenic	---
3	7431174	7431182	+	7431174	8	29	XLOC_054675	+	predicted	---
3	7576674	7576674	-	7576674	9	9	XLOC_055931	-	predicted	---
3	7665751	7665766	+	7665756	12	33	ENSSSCG00000026653	+	3'UTR	---

3	7666297	7666301	-	7666297	12	16	ENSSSCG00000007659	-	3'UTR	*ZCWPW1*
							ENSSSCG00000025164	-	intron	---
3	7842381	7842385	-	7842385	11	15	XLOC_055937	-	predicted	---
3	7843113	7843114	-	7843114	6	11	XLOC_055937	-	predicted	---
3	7909264	7909267	+	7909264	6	9	ENSSSCG00000025096	+	intron	*FBXO24*
3	7917192	7917197	+	7917192	21	29	XLOC_054684	+	predicted	---
3	7923555	7923588	+	7923575	27	63	ENSSSCG00000027466	+	3'UTR	*PCOLCE*
3	7928770	7928775	+	7928770	14	31	ENSSSCG00000023255	+	3'UTR	*MOSPD3*
3	7934261	7934277	-	7934262	54	215	XLOC_055940	-	predicted	---
3	8003718	8003745	+	8003737	9	75	XLOC_054689	+	predicted	---
3	8036569	8036569	+	8036569	4	4	ENSSSCG00000007674	+	ORF	*ZAN*
3	8036634	8036634	+	8036634	3	3	ENSSSCG00000007674	+	ORF	*ZAN*
3	8036716	8036716	+	8036716	3	3	ENSSSCG00000007674	+	ORF	*ZAN*
3	8087272	8087272	+	8087272	3	3	ENSSSCG00000007674	+	intron	*ZAN*
3	8095772	8095773	+	8095772	11	16	ENSSSCG00000007674	+	3'UTR	*ZAN*
3	8097953	8097953	-	8097953	3	3	ENSSSCG00000007675	-	3'UTR	*EPHB4*
3	8137658	8137658	+	8137658	4	4	ENSSSCG00000023619	+	intron	*SLC12A9*
3	8139821	8139829	+	8139829	5	8	XLOC_054693	+	predicted	---
3	8143783	8143783	+	8143783	6	6	XLOC_054693	+	predicted	---
3	8158068	8158070	+	8158069	10	19	ENSSSCG00000024789	+	3'UTR	*SRRT*
3	8158124	8158129	+	8158127	64	108	ENSSSCG00000024789	+	3'UTR	*SRRT*
3	8158196	8158197	-	8158197	4	7	XLOC_055945	-	predicted	---
3	8165259	8165259	+	8165259	7	7	---	---	intergenic	---
3	8495606	8495607	+	8495607	9	16	ENSSSCG00000007679	+	3'UTR	*IFT22*
3	8495857	8495857	+	8495857	3	3	ENSSSCG00000007679	+	3'UTR	*IFT22*
3	8495909	8495929	+	8495917	5	13	ENSSSCG00000007679	+	3'UTR	*IFT22*

3	8501630	8501630	-	8501630	3	3	XLOC_055946	-	predicted	---
3	8656596	8656597	+	8656596	4	8	ENSSSCG00000007678	+	intron	*COL26A1*
3	8797665	8797665	+	8797665	3	3	---	---	intergenic	---
3	8934199	8934199	+	8934199	3	3	---	---	intergenic	---
3	8947756	8947756	-	8947756	4	4	---	---	intergenic	---
3	9328446	9328447	+	9328447	12	15	ENSSSCG00000007681	+	ORF	---
3	9333531	9333531	+	9333531	6	6	ENSSSCG00000007681	+	intron	---
3	9439042	9439042	+	9439042	8	8	---	---	intergenic	---
3	9448971	9448972	-	9448972	4	7	---	---	intergenic	---
3	9480182	9480186	-	9480185	27	64	ENSSSCG00000007683	-	3'UTR	---
3	9525412	9525412	+	9525412	3	3	ENSSSCG00000007684	+	intron	*PRKRIP1*
3	9548048	9548049	+	9548049	10	13	ENSSSCG00000007684	+	3'UTR	*PRKRIP1*
3	9621271	9621271	+	9621271	6	6	ENSSSCG00000024397	+	3'UTR	*DTX2*
3	9690878	9690880	+	9690879	9	17	XLOC_054702	+	predicted	---
3	9953240	9953292	-	9953241	32	138	ENSSSCG00000025486	-	3'UTR	*MDH2*
3	10041087	10041111	-	10041096	27	250	---	---	intergenic	---
3	10041129	10041163	+	10041159	101	582	---	---	intergenic	---
3	10144437	10144438	+	10144437	21	29	---	---	intergenic	---
3	10263424	10263425	+	10263424	17	29	ENSSSCG00000007700	+	intron	*HIP1*
3	10282560	10282560	+	10282560	5	5	ENSSSCG00000007700	+	3'UTR	*HIP1*
3	10287567	10287567	-	10287567	3	3	---	---	intergenic	---
3	10407823	10407831	+	10407829	44	82	ENSSSCG00000007702	+	3'UTR	*FKBP6*
3	10456457	10456457	+	10456457	3	3	XLOC_054715	+	predicted	---
3	10501139	10501139	-	10501139	7	7	ENSSSCG00000027342	-	ORF	*BAZ1B*
3	10528811	10528811	-	10528811	3	3	ENSSSCG00000027342	-	intron	*BAZ1B*
3	10550010	10550011	-	10550011	26	36	XLOC_055977	-	predicted	---

3	10584279	10584280	-	10584280	4	7	XLOC_055977	-	predicted	---
3	10586453	10586481	-	10586460	21	50	XLOC_055977	-	predicted	---
3	10586952	10586958	-	10586956	46	102	XLOC_055977	-	predicted	---
3	10587000	10587004	-	10587002	39	81	XLOC_055977	-	predicted	---
3	10654892	10654892	-	10654892	5	5	XLOC_055980	-	predicted	---
3	10668643	10668643	+	10668643	4	4	ENSSSCG00000007713	+	ORF	*WBSCR22*
3	10669009	10669009	+	10669009	4	4	ENSSSCG00000007713	+	3'UTR	*WBSCR22*
3	10669249	10669259	+	10669251	208	431	ENSSSCG00000007713	+	3'UTR	*WBSCR22*
3	10697165	10697169	-	10697169	46	63	ENSSSCG00000007715	-	3'UTR	*ABHD11*
3	10715260	10715261	-	10715261	14	20	XLOC_055983	-	predicted	---
3	10752477	10752481	-	10752477	8	21	XLOC_055984	-	predicted	---
3	10757530	10757530	+	10757530	14	14	XLOC_054724	+	predicted	---
3	10775996	10776002	+	10775996	15	19	ENSSSCG00000007717	+	3'UTR	*WBSCR27*
3	10776895	10776906	-	10776901	92	145	ENSSSCG00000007718	-	3'UTR	*CLDN4*
3	10879782	10879782	+	10879782	3	3	XLOC_054726	+	predicted	---
3	10908513	10908517	+	10908515	41	71	XLOC_054727	+	predicted	---
3	10961581	10961581	+	10961581	3	3	ENSSSCG00000030378	+	3'UTR	*LIMK1*
3	11029713	11029713	+	11029713	3	3	ENSSSCG00000025966	+	intron	*EIF4H*
3	11030115	11030135	+	11030131	23	82	ENSSSCG00000025966	+	3'UTR	*EIF4H*
3	11031691	11031700	+	11031693	86	165	ENSSSCG00000025966	+	3'UTR	*EIF4H*
3	11054826	11054829	+	11054829	4	10	ENSSSCG00000027487	+	3'UTR	*LAT2*
3	11055019	11055031	+	11055022	4	10	ENSSSCG00000027487	+	3'UTR	*LAT2*
3	11055312	11055322	+	11055312	57	94	ENSSSCG00000027487	+	3'UTR	*LAT2*
3	11056710	11056740	-	11056716	50	120	ENSSSCG00000028504	-	3'UTR	*RFC2*
3	11125876	11125878	+	11125876	5	11	ENSSSCG00000007719	+	intron	*CLIP2*
3	11423476	11423486	-	11423486	6	9	XLOC_055993	-	predicted	---

3	11450003	11450003	+	11450003	6	6	ENSSSCG00000007721	+	intron	*GTF2I*
3	11492966	11492967	+	11492967	12	23	ENSSSCG00000007721	+	intron	*GTF2I*
3	11529002	11529005	+	11529004	19	38	ENSSSCG00000007721	+	3'UTR	*GTF2I*
3	11529337	11529337	+	11529337	9	9	ENSSSCG00000007721	+	3'UTR	*GTF2I*
3	11529975	11529983	+	11529982	14	24	ENSSSCG00000007721	+	3'UTR	*GTF2I*
3	11553096	11553096	+	11553096	59	59	ENSSSCG00000007722	+	3'UTR	*NCF1*
3	11560515	11560516	-	11560515	14	18	ENSSSCG00000028635	-	3'UTR	---
3	11569095	11569103	-	11569095	3	6	ENSSSCG00000028635	-	intron	---
3	11668500	11668510	+	11668501	17	42	ENSSSCG00000007723	+	3'UTR	*WBSCR16*
3	11676548	11676548	-	11676548	4	4	XLOC_055996	-	predicted	---
3	11762227	11762236	+	11762232	12	35	XLOC_054741	+	predicted	---
3	12113546	12113546	-	12113546	10	10	XLOC_055999	-	predicted	---
3	12152703	12152703	-	12152703	4	4	XLOC_056000	-	predicted	---
3	12152907	12152913	-	12152912	21	48	---	---	intergenic	---
3	12743636	12743636	-	12743636	3	3	---	---	intergenic	---
3	13288459	13288459	-	13288459	3	3	---	---	intergenic	---
3	13418518	13418518	-	13418518	4	4	---	---	intergenic	---
3	13775097	13775097	-	13775097	27	27	---	---	intergenic	---
3	14231319	14231320	-	14231320	97	105	---	---	intergenic	---
3	14312488	14312488	+	14312488	3	3	---	---	intergenic	---
3	14331697	14331697	+	14331697	5	5	---	---	intergenic	---
3	14482789	14482789	-	14482789	4	4	---	---	intergenic	---
3	14946225	14946230	+	14946225	12	25	ENSSSCG00000007727	+	3'UTR	*AUTS2*
3	15106486	15106487	-	15106486	6	10	---	---	intergenic	---
3	15324327	15324327	-	15324327	3	3	---	---	intergenic	---
3	15341197	15341197	+	15341197	4	4	---	---	intergenic	---

3	15698677	15698677	-	15698677	3	3	---	---	intergenic	---
3	16268153	16268154	-	16268154	4	7	XLOC_056012	-	predicted	---
3	16381541	16381541	-	16381541	3	3	ENSSSCG00000007733	-	intron	---
3	16409195	16409195	+	16409195	6	6	ENSSSCG00000007732	+	ORF	*SBDS*
3	16413174	16413174	+	16413174	3	3	ENSSSCG00000007732	+	ORF	*SBDS*
3	16414388	16414390	+	16414389	15	29	ENSSSCG00000007732	+	3'UTR	*SBDS*
3	16414644	16414644	+	16414644	3	3	ENSSSCG00000007732	+	3'UTR	*SBDS*
3	16414737	16414737	+	16414737	3	3	ENSSSCG00000007732	+	3'UTR	*SBDS*
3	16414976	16414980	+	16414978	27	44	ENSSSCG00000007732	+	3'UTR	*SBDS*
3	16416062	16416066	+	16416063	28	46	ENSSSCG00000007732	+	3'UTR	*SBDS*
3	16504999	16505006	-	16505000	31	47	ENSSSCG00000007736	-	3'UTR	*TMEM248*
3	16507538	16507546	-	16507544	16	33	ENSSSCG00000007736	-	3'UTR	*TMEM248*
3	16560383	16560384	-	16560384	8	12	ENSSSCG00000007735	-	3'UTR	*RABGEF1*
3	16561927	16561927	-	16561927	3	3	ENSSSCG00000007735	-	3'UTR	*RABGEF1*
3	16593670	16593671	-	16593671	8	12	ENSSSCG00000007735	-	intron	*RABGEF1*
3	16660055	16660063	-	16660062	40	62	ENSSSCG00000007737	-	3'UTR	*TPST1*
3	16800688	16800706	+	16800693	15	27	XLOC_054762	+	predicted	---
3	16862210	16862210	-	16862210	38	38	---	---	intergenic	---
3	16899550	16899558	+	16899556	96	243	ENSSSCG00000007738	+	3'UTR	---
3	16937256	16937256	+	16937256	4	4	ENSSSCG00000007739	+	ORF	*GUSB*
3	16939380	16939392	+	16939386	40	115	ENSSSCG00000007739	+	3'UTR	*GUSB*
3	16939436	16939445	+	16939443	29	69	ENSSSCG00000007739	+	3'UTR	*GUSB*
3	16941580	16941582	-	16941580	15	27	ENSSSCG00000028513	-	3'UTR	*VKORC1L1*
3	16943918	16943930	-	16943918	7	10	ENSSSCG00000028513	-	3'UTR	*VKORC1L1*
3	16944341	16944341	-	16944341	3	3	ENSSSCG00000028513	-	3'UTR	*VKORC1L1*
3	16944664	16944669	-	16944665	7	16	ENSSSCG00000028513	-	3'UTR	*VKORC1L1*

3	16944763	16944763	-	16944763	4	4	ENSSSCG00000028513	-	3'UTR	*VKORC1L1*
3	17009124	17009124	-	17009124	4	4	ENSSSCG00000028513	-	intron	*VKORC1L1*
3	17028951	17028951	+	17028951	11	11	---	---	intergenic	---
3	17060430	17060449	+	17060437	41	137	XLOC_054766	+	predicted	---
3	17069940	17069940	+	17069940	3	3	ENSSSCG00000007743	+	ORF	*CHCHD2*
3	17071870	17071874	+	17071872	86	110	ENSSSCG00000007743	+	3'UTR	*CHCHD2*
3	17082861	17082861	-	17082861	4	4	ENSSSCG00000007745	-	3'UTR	*SUMF2*
3	17082929	17082929	-	17082929	8	8	ENSSSCG00000007745	-	3'UTR	*SUMF2*
3	17095570	17095585	-	17095579	41	98	ENSSSCG00000020808	-	3'UTR	---
3	17096104	17096110	-	17096104	7	13	ENSSSCG00000020808	-	3'UTR	---
3	17096287	17096297	-	17096287	6	15	ENSSSCG00000020808	-	3'UTR	---
3	17127603	17127603	+	17127603	6	6	XLOC_054768	+	predicted	---
3	17128530	17128535	+	17128532	10	23	XLOC_054768	+	predicted	---
3	17136060	17136060	-	17136060	5	5	ENSSSCG00000007748	-	3'UTR	*PSPH*
3	17236801	17236811	-	17236808	18	49	XLOC_056029	-	predicted	---
3	17236910	17236910	-	17236910	4	4	ENSSSCG00000007749	-	3'UTR	---
3	17270269	17270269	-	17270269	15	15	ENSSSCG00000029029	-	intron	*ZNF713*
3	17386339	17386341	-	17386341	15	23	ENSSSCG00000025688	-	3'UTR	*COX6A2*
3	17387626	17387629	-	17387629	7	13	---	---	intergenic	---
3	17392386	17392386	+	17392386	7	7	---	---	intergenic	---
3	17496663	17496675	+	17496670	39	139	ENSSSCG00000007763	+	3'UTR	*BCKDK*
3	17510034	17510104	+	17510035	12	89	ENSSSCG00000007762	+	3'UTR	*KAT8*
3	17554604	17554604	+	17554604	3	3	ENSSSCG00000030798	+	3'UTR	*FUS*
3	17557336	17557341	+	17557340	30	56	ENSSSCG00000030798	+	3'UTR	*FUS*
3	17564759	17564773	-	17564759	9	20	ENSSSCG00000007758	-	3'UTR	---
3	17615680	17615680	-	17615680	3	3	XLOC_056041	-	predicted	---

3	17651479	17651479	-	17651479	3	3	XLOC_056042	-	predicted	---
3	17719647	17719662	+	17719649	195	291	XLOC_054788	+	predicted	---
3	17727268	17727269	-	17727268	17	20	XLOC_056043	-	predicted	---
3	17807043	17807043	+	17807043	3	3	ENSSSCG00000028186	+	3'UTR	*FBXL19*
3	17814002	17814006	+	17814005	31	46	ENSSSCG00000007770	+	3'UTR	*ORAI3*
3	17840379	17840380	+	17840379	6	10	ENSSSCG00000007782	+	3'UTR	*SETD1A*
3	17844521	17844524	+	17844522	77	137	ENSSSCG00000021238	+	3'UTR	*HSD3B7*
3	17949315	17949317	-	17949316	32	42	ENSSSCG00000007776	-	3'UTR	*BCL7C*
3	18063283	18063283	+	18063283	4	4	ENSSSCG00000007780	+	3'UTR	*ZNF629*
3	18065309	18065311	+	18065310	15	24	ENSSSCG00000007780	+	3'UTR	*ZNF629*
3	18068619	18068627	-	18068626	30	47	XLOC_056052	-	predicted	---
3	18087209	18087216	+	18087210	49	93	ENSSSCG00000007778	+	3'UTR	*C16orf93*
3	18087273	18087287	-	18087282	217	566	ENSSSCG00000007788	-	3'UTR	*PHKG2*
3	18110424	18110427	-	18110427	11	16	XLOC_056053	-	predicted	---
3	18113115	18113115	-	18113115	3	3	ENSSSCG00000007789	-	ORF	---
3	18120458	18120458	-	18120458	7	7	ENSSSCG00000007789	-	intron	---
3	18187492	18187499	-	18187492	11	24	ENSSSCG00000025794	-	3'UTR	*FBRS*
3	18188128	18188135	-	18188134	6	12	ENSSSCG00000025794	-	3'UTR	*FBRS*
3	18202126	18202132	-	18202126	10	37	ENSSSCG00000007790	-	3'UTR	*PRR14*
3	18228889	18228896	+	18228896	6	14	XLOC_054801	+	predicted	---
3	18231497	18231497	-	18231497	3	3	---	---	intergenic	---
3	18242522	18242522	+	18242522	4	4	XLOC_054801	+	predicted	---
3	18298946	18298946	+	18298946	4	4	ENSSSCG00000007797	+	3'UTR	*ITGAL*
							ENSSSCG00000007797	+	ORF	---
3	18299831	18299831	+	18299831	17	17	XLOC_054804	+	predicted	---
3	18303234	18303234	-	18303234	3	3	ENSSSCG00000023349	-	3'UTR	*ZNF768*

3	18310657	18310657	-	18310657	3	3	---	---	intergenic	---
3	18323328	18323330	-	18323329	83	104	XLOC_056061	-	predicted	---
3	18343063	18343063	-	18343063	3	3	XLOC_056062	-	predicted	---
3	18344316	18344317	-	18344317	5	8	XLOC_056062	-	predicted	---
3	18359646	18359647	-	18359647	10	15	ENSSSCG00000007801	-	3'UTR	*ZNF48*
3	18376613	18376613	+	18376613	3	3	ENSSSCG00000007800	+	3'UTR	*SEPT1*
3	18376796	18376802	-	18376796	164	189	XLOC_056064	-	predicted	---
3	18394731	18394736	+	18394735	33	94	ENSSSCG00000007798	+	3'UTR	*TBC1D10B*
3	18399152	18399157	+	18399153	13	36	ENSSSCG00000007791	+	3'UTR	*CD2BP2*
3	18400485	18400485	+	18400485	3	3	ENSSSCG00000007791	+	3'UTR	*CD2BP2*
3	18496667	18496667	+	18496667	3	3	---	---	intergenic	---
3	18502764	18502768	+	18502767	45	69	ENSSSCG00000027629	+	3'UTR	*CDIPT*
3	18503136	18503136	+	18503136	3	3	ENSSSCG00000027629	+	3'UTR	*CDIPT*
3	18509115	18509119	-	18509115	21	24	ENSSSCG00000027946	-	3'UTR	*MVP*
3	18543782	18543796	-	18543783	21	33	ENSSSCG00000025321	-	3'UTR	*MAZ*
3	18548869	18548869	-	18548869	3	3	ENSSSCG00000029509	-	3'UTR	*KIF22*
3	18579338	18579342	+	18579342	38	55	---	---	intergenic	---
3	18588985	18588985	-	18588985	3	3	XLOC_056072	-	predicted	---
3	18589032	18589036	-	18589035	8	17	XLOC_056072	-	predicted	---
3	18633345	18633345	+	18633345	3	3	ENSSSCG00000022075	+	3'UTR	*KCTD13*
3	18704355	18704355	+	18704355	4	4	XLOC_054817	+	predicted	---
3	18706413	18706426	-	18706424	168	244	ENSSSCG00000007803	-	3'UTR	*TUFM*
3	18709383	18709383	-	18709383	3	3	ENSSSCG00000007803	-	ORF	*TUFM*
3	18728401	18728404	+	18728404	7	10	ENSSSCG00000007804	+	3'UTR	*SH2B1*
3	18753525	18753525	-	18753525	6	6	ENSSSCG00000007806	-	3'UTR	*RABEP2*
3	18753569	18753571	+	18753569	43	75	ENSSSCG00000007805	+	3'UTR	*ATP2A1*

3	18766077	18766078	-	18766077	15	21	ENSSSCG00000007806	-	intron	*RABEP2*
3	18810700	18810703	+	18810700	6	9	ENSSSCG00000007808	+	3'UTR	*NFATC2IP*
3	18825009	18825011	+	18825011	18	32	ENSSSCG00000007809	+	3'UTR	*SPNS1*
3	18829965	18829969	+	18829965	6	9	ENSSSCG00000007811	+	3'UTR	*LAT*
3	18830934	18830934	+	18830934	4	4	XLOC_054823	+	predicted	---
3	18861178	18861178	-	18861178	3	3	XLOC_056079	-	predicted	---
3	19112476	19112480	+	19112480	9	16	XLOC_054826	+	predicted	---
3	19436725	19436725	-	19436725	5	5	---	---	intergenic	---
3	19598930	19598930	-	19598930	3	3	---	---	intergenic	---
3	19672865	19672865	+	19672865	5	5	ENSSSCG00000007815	+	3'UTR	*GTF3C1*
3	19701329	19701329	-	19701329	4	4	ENSSSCG00000007816	-	intron	*IL21R*
3	19843482	19843482	-	19843482	3	3	---	---	intergenic	---
3	19846362	19846397	-	19846365	11	30	ENSSSCG00000007817	-	3'UTR	*IL4R*
3	19871956	19871961	-	19871958	4	11	ENSSSCG00000007818	-	3'UTR	*KDM8*
3	20021456	20021465	-	20021458	418	1042	XLOC_056091	-	predicted	---
3	20021510	20021511	-	20021511	31	34	XLOC_056091	-	predicted	---
3	20022109	20022111	-	20022109	4	8	XLOC_056091	-	predicted	---
3	20022193	20022194	-	20022193	3	6	XLOC_056091	-	predicted	---
3	20022285	20022285	-	20022285	3	3	XLOC_056091	-	predicted	---
3	20022602	20022629	-	20022602	7	12	XLOC_056091	-	predicted	---
3	20027051	20027057	+	20027056	8	14	XLOC_054836	+	predicted	---
3	20226473	20226473	-	20226473	5	5	---	---	intergenic	---
3	20426749	20426749	+	20426749	3	3	---	---	intergenic	---
3	20696755	20696755	-	20696755	8	8	---	---	intergenic	---
3	20744820	20744820	-	20744820	4	4	---	---	intergenic	---
3	21321319	21321319	-	21321319	22	22	---	---	intergenic	---

3	21370541	21370541	+	21370541	3	3	---	---	intergenic	---
3	21374237	21374237	-	21374237	3	3	---	---	intergenic	---
3	21396086	21396086	+	21396086	7	7	---	---	intergenic	---
3	21570287	21570287	+	21570287	3	3	---	---	intergenic	---
3	21586437	21586437	+	21586437	3	3	---	---	intergenic	---
3	21636778	21636778	+	21636778	3	3	---	---	intergenic	---
3	21640556	21640556	+	21640556	3	3	---	---	intergenic	---
3	21929207	21929208	+	21929207	13	16	---	---	intergenic	---
3	22219520	22219537	+	22219536	20	59	ENSSSCG00000026077	+	3'UTR	---
3	22228427	22228427	-	22228427	6	6	XLOC_056100	-	predicted	---
3	22327926	22327930	-	22327930	5	8	XLOC_056101	-	predicted	---
3	22536847	22536849	-	22536848	9	20	---	---	intergenic	---
3	22539406	22539406	-	22539406	5	5	ENSSSCG00000007830	-	ORF	*RBBP6*
3	22539825	22539825	-	22539825	4	4	ENSSSCG00000007830	-	ORF	*RBBP6*
3	22542152	22542152	-	22542152	4	4	ENSSSCG00000007830	-	intron	*RBBP6*
3	22552057	22552057	-	22552057	3	3	ENSSSCG00000007830	-	intron	*RBBP6*
3	22555400	22555406	-	22555400	3	9	ENSSSCG00000007830	-	intron	*RBBP6*
3	22562300	22562300	-	22562300	4	4	ENSSSCG00000007830	-	intron	*RBBP6*
3	22570082	22570082	-	22570082	9	9	XLOC_056102	-	predicted	---
3	22591106	22591106	-	22591106	4	4	---	---	intergenic	---
3	22591132	22591132	+	22591132	3	3	---	---	intergenic	---
3	22614922	22614922	-	22614922	3	3	---	---	intergenic	---
3	22949686	22949686	-	22949686	4	4	---	---	intergenic	---
3	22955875	22955875	+	22955875	3	3	XLOC_054851	+	predicted	---
3	23073885	23073885	-	23073885	3	3	ENSSSCG00000007833	-	intron	---
3	23206859	23206860	-	23206860	10	17	---	---	intergenic	---

3	23206884	23206886	+	23206886	14	31	---	---	intergenic	---
3	23434105	23434109	-	23434105	3	9	---	---	intergenic	---
3	23439110	23439110	-	23439110	4	4	---	---	intergenic	---
3	23545429	23545429	+	23545429	6	6	XLOC_054853	+	predicted	---
3	23822182	23822182	+	23822182	3	3	---	---	intergenic	---
3	24076506	24076506	-	24076506	8	8	---	---	intergenic	---
3	24157678	24157683	-	24157681	13	35	ENSSSCG00000027967	-	3'UTR	*IGSF6*
3	24161745	24161745	-	24161745	6	6	ENSSSCG00000027967	-	ORF	*IGSF6*
3	24170870	24170871	+	24170870	16	21	ENSSSCG00000030373	+	3'UTR	*METTL9*
3	24287296	24287297	-	24287297	6	11	XLOC_056115	-	predicted	---
3	24287532	24287532	-	24287532	3	3	ENSSSCG00000007839	-	3'UTR	*EEF2K*
3	24288299	24288299	-	24288299	3	3	ENSSSCG00000007839	-	3'UTR	*EEF2K*
3	24333493	24333493	-	24333493	4	4	ENSSSCG00000007839	-	intron	*EEF2K*
3	24335290	24335290	-	24335290	3	3	ENSSSCG00000007840	-	3'UTR	*POLR3E*
							ENSSSCG00000007839	-	intron	*EEF2K*
3	24475486	24475486	-	24475486	3	3	XLOC_056120	-	predicted	---
3	24533827	24533831	+	24533831	10	16	XLOC_054860	+	predicted	---
3	24533883	24533883	+	24533883	4	4	XLOC_054860	+	predicted	---
3	24553668	24553668	+	24553668	4	4	ENSSSCG00000025266	+	intron	---
3	24624438	24624439	+	24624439	16	21	ENSSSCG00000007841	+	3'UTR	---
3	24746038	24746038	-	24746038	4	4	XLOC_056122	-	predicted	---
3	24966359	24966359	+	24966359	5	5	---	---	intergenic	---
3	25106845	25106852	-	25106845	16	21	ENSSSCG00000007847	-	intron	---
3	25189336	25189336	-	25189336	3	3	ENSSSCG00000007847	-	intron	---
3	25255755	25255755	+	25255755	5	5	XLOC_054870	+	predicted	---
3	25320256	25320264	+	25320256	20	31	---	---	intergenic	---

3	25569528	25569528	+	25569528	5	5	ENSSSCG00000007849	+	3'UTR	*CRYM*
3	25569583	25569588	+	25569584	85	151	ENSSSCG00000007849	+	3'UTR	*CRYM*
3	25684930	25684933	+	25684930	3	6	XLOC_054872	+	predicted	---
3	25694394	25694406	-	25694401	21	38	---	---	intergenic	---
3	25694505	25694505	-	25694505	5	5	XLOC_056128	-	predicted	---
3	25870035	25870035	+	25870035	5	5	---	---	intergenic	---
3	25913508	25913508	+	25913508	3	3	XLOC_054877	+	predicted	---
3	25917676	25917685	-	25917683	22	41	ENSSSCG00000007853	-	3'UTR	*LYRM1*
3	25917772	25917772	-	25917772	3	3	ENSSSCG00000007853	-	3'UTR	*LYRM1*
3	25971290	25971300	+	25971299	15	35	ENSSSCG00000007854	+	3'UTR	*DCUN1D3*
3	25971361	25971363	+	25971363	11	24	ENSSSCG00000007854	+	3'UTR	*DCUN1D3*
3	25972870	25972870	+	25972870	3	3	XLOC_054878	+	predicted	---
3	25976506	25976506	+	25976506	5	5	XLOC_054878	+	predicted	---
3	25982518	25982542	-	25982534	273	458	XLOC_056131	-	predicted	---
3	25987216	25987217	-	25987216	5	8	ENSSSCG00000007855	-	ORF	---
3	26031987	26031992	-	26031988	7	19	ENSSSCG00000007857	-	3'UTR	*ACSM3*
3	26079740	26079741	+	26079740	11	15	ENSSSCG00000024748	+	3'UTR	*THUMPD1*
3	26080602	26080609	-	26080608	95	158	ENSSSCG00000030033	-	3'UTR	*ACSM4*
3	26080770	26080770	-	26080770	6	6	ENSSSCG00000030033	-	3'UTR	*ACSM4*
3	26092287	26092287	-	26092287	4	4	ENSSSCG00000030033	-	intron	*ACSM4*
3	26095203	26095203	-	26095203	3	3	ENSSSCG00000030033	-	ORF	*ACSM4*
3	26136697	26136704	-	26136704	17	43	XLOC_056135	-	predicted	---
3	26137017	26137017	-	26137017	4	4	XLOC_056135	-	predicted	---
3	26138156	26138166	-	26138166	5	9	XLOC_056135	-	predicted	---
3	26212802	26212810	-	26212805	75	153	ENSSSCG00000026453	-	intron	*ACSM5*

3	26221684	26221684	-	26221684	6	6	ENSSSCG00000026453	-	intron	*ACSM5*
							ENSSSCG00000007860	-	ORF	*PDILT*
3	26292578	26292578	+	26292578	4	4	ENSSSCG00000007861	+	3'UTR	*GP2*
3	26527087	26527087	-	26527087	4	4	---	---	intergenic	---
3	26562546	26562547	+	26562546	4	7	---	---	intergenic	---
3	26736185	26736189	-	26736188	6	16	ENSSSCG00000007864	-	3'UTR	*GPRC5B*
3	26876455	26876456	-	26876455	11	14	XLOC_056145	-	predicted	---
3	26904662	26904663	+	26904663	9	13	---	---	intergenic	---
3	26904671	26904671	-	26904671	3	3	XLOC_056145	-	predicted	---
3	26922604	26922623	+	26922618	23	48	ENSSSCG00000029212	+	3'UTR	*GDE1*
3	26923805	26923805	+	26923805	65	65	XLOC_054886	+	predicted	---
3	26928297	26928320	-	26928319	21	44	---	---	intergenic	---
3	26952710	26952713	-	26952710	3	9	ENSSSCG00000007868	-	intron	*TMC5*
3	26955212	26955212	+	26955212	4	4	ENSSSCG00000029976	+	intron	---
3	26964520	26964520	-	26964520	3	3	ENSSSCG00000007868	-	intron	*TMC5*
3	26967115	26967117	-	26967116	33	51	ENSSSCG00000007866	-	3'UTR	*TMC7*
							ENSSSCG00000007868	-	intron	*TMC5*
3	27066362	27066363	-	27066362	3	6	ENSSSCG00000007868	-	intron	*TMC5*
3	27693093	27693093	+	27693093	3	3	---	---	intergenic	---
3	27733890	27733890	-	27733890	4	4	XLOC_056152	-	predicted	---
3	27747694	27747694	-	27747694	6	6	XLOC_056152	-	predicted	---
3	27773974	27773974	-	27773974	3	3	ENSSSCG00000007872	-	intron	---
3	27964085	27964085	-	27964085	3	3	---	---	intergenic	---
3	27973630	27973630	+	27973630	3	3	---	---	intergenic	---
3	28157531	28157531	-	28157531	7	7	---	---	intergenic	---
3	28552345	28552345	-	28552345	6	6	---	---	intergenic	---

3	28576845	28576845	+	28576845	3	3	---	---	intergenic	---
3	28886614	28886614	-	28886614	3	3	---	---	intergenic	---
3	29063405	29063405	+	29063405	9	9	XLOC_054890	+	predicted	---
3	29067573	29067583	-	29067574	18	43	XLOC_056159	-	predicted	---
3	29181488	29181489	+	29181489	35	39	---	---	intergenic	---
3	29428702	29428702	+	29428702	4	4	---	---	intergenic	---
3	29554349	29554362	+	29554352	18	37	ENSSSCG00000024206	+	3'UTR	*CPPED1*
3	29554486	29554487	+	29554486	7	11	ENSSSCG00000024206	+	3'UTR	*CPPED1*
3	29702611	29702611	+	29702611	3	3	ENSSSCG00000007878	+	intron	*PARN*
3	29877401	29877402	+	29877402	6	10	ENSSSCG00000007878	+	3'UTR	*PARN*
3	30116487	30116488	-	30116488	10	13	ENSSSCG00000023626	-	intron	---
3	30286848	30286848	-	30286848	3	3	ENSSSCG00000007881	-	3'UTR	*ERCC4*
3	30288973	30288973	-	30288973	3	3	ENSSSCG00000007881	-	3'UTR	*ERCC4*
3	30356814	30356814	-	30356814	4	4	ENSSSCG00000007881	-	intron	*ERCC4*
3	30369702	30369702	-	30369702	6	6	ENSSSCG00000007881	-	intron	*ERCC4*
3	30456138	30456138	+	30456138	3	3	---	---	intergenic	---
3	30766450	30766450	+	30766450	3	3	---	---	intergenic	---
3	31045263	31045263	+	31045263	3	3	---	---	intergenic	---
3	31265179	31265179	+	31265179	3	3	---	---	intergenic	---
3	31285944	31285944	+	31285944	4	4	---	---	intergenic	---
3	31366175	31366175	-	31366175	3	3	---	---	intergenic	---
3	31488697	31488699	-	31488697	5	13	ENSSSCG00000007885	-	intron	*SNX29*
3	31538473	31538473	-	31538473	3	3	ENSSSCG00000007885	-	intron	*SNX29*
3	31566045	31566046	-	31566045	4	7	ENSSSCG00000007885	-	intron	*SNX29*
3	31802235	31802236	+	31802235	11	16	---	---	intergenic	---
3	31893485	31893487	+	31893485	14	31	---	---	intergenic	---

3	31893641	31893641	-	31893641	3	3	---	---	intergenic	---
3	32033538	32033544	+	32033538	11	20	XLOC_054918	+	predicted	---
3	32046274	32046274	-	32046274	5	5	---	---	intergenic	---
3	32319439	32319467	-	32319449	40	161	ENSSSCG00000024982	-	3'UTR	*LITAF*
3	32346265	32346265	-	32346265	3	3	ENSSSCG00000024982	-	intron	*LITAF*
3	32358240	32358240	-	32358240	8	8	---	---	intergenic	---
3	32449478	32449478	-	32449478	4	4	ENSSSCG00000028743	-	3'UTR	---
3	32476640	32476640	-	32476640	4	4	---	---	intergenic	---
3	32607804	32607804	+	32607804	3	3	---	---	intergenic	---
3	32650535	32650555	-	32650551	90	258	---	---	intergenic	---
3	32650675	32650676	+	32650676	5	9	ENSSSCG00000021337	+	ORF	*PRM1*
3	32650915	32650916	+	32650916	5	9	XLOC_054928	+	predicted	---
3	32650971	32650971	+	32650971	31	31	XLOC_054928	+	predicted	---
3	32651012	32651044	+	32651035	7078	16492	XLOC_054928	+	predicted	---
3	32654215	32654217	+	32654217	8	14	ENSSSCG00000024307	+	ORF	*PRM2*
3	32654506	32654506	+	32654506	4	4	ENSSSCG00000024307	+	3'UTR	*PRM2*
3	32656212	32656212	+	32656212	5	5	XLOC_054929	+	predicted	---
3	32656357	32656371	+	32656358	557	803	XLOC_054929	+	predicted	---
3	32659703	32659710	-	32659709	14	47	---	---	intergenic	---
3	32660148	32660149	+	32660149	13	16	ENSSSCG00000027300	+	intron	*TNP2*
3	32661155	32661186	+	32661171	1827	6054	ENSSSCG00000027300	+	3'UTR	*TNP2*
3	32672830	32672830	+	32672830	3	3	XLOC_054931	+	predicted	---
3	32683557	32683557	-	32683557	4	4	---	---	intergenic	---
3	32726163	32726163	+	32726163	3	3	---	---	intergenic	---
3	32726813	32726817	-	32726817	8	22	---	---	intergenic	---
3	32726839	32726839	+	32726839	3	3	---	---	intergenic	---

3	32726978	32726978	+	32726978	4	4	---	---	intergenic	---
3	32727232	32727232	-	32727232	4	4	---	---	intergenic	---
3	32876190	32876191	-	32876190	3	6	---	---	intergenic	---
3	32905693	32905693	-	32905693	3	3	---	---	intergenic	---
3	33045135	33045153	-	33045135	7	10	ENSSSCG00000024488	-	3'UTR	---
3	33079523	33079534	-	33079531	13	30	---	---	intergenic	---
3	33194091	33194094	+	33194091	11	18	ENSSSCG00000007904	+	3'UTR	---
3	33194154	33194162	+	33194161	16	38	ENSSSCG00000007904	+	3'UTR	---
3	33274249	33274249	-	33274249	3	3	XLOC_056197	-	predicted	---
3	33274808	33274811	-	33274808	6	16	XLOC_056197	-	predicted	---
3	33294315	33294316	+	33294316	10	13	XLOC_054940	+	predicted	---
3	33294821	33294821	+	33294821	5	5	XLOC_054940	+	predicted	---
3	33324469	33324472	-	33324469	7	10	XLOC_056199	-	predicted	---
3	33325727	33325727	-	33325727	11	11	ENSSSCG00000020675	-	ORF	*ATF7IP2*
3	33370867	33370867	-	33370867	3	3	ENSSSCG00000020675	-	intron	*ATF7IP2*
3	33493677	33493677	+	33493677	3	3	XLOC_054943	+	predicted	---
3	33493805	33493812	+	33493812	10	14	XLOC_054943	+	predicted	---
3	34067405	34067405	-	34067405	3	3	---	---	intergenic	---
3	34419378	34419387	-	34419378	11	19	ENSSSCG00000028454	-	3'UTR	---
3	34420901	34420901	-	34420901	8	8	ENSSSCG00000028454	-	3'UTR	---
3	34421435	34421444	-	34421436	16	48	ENSSSCG00000028454	-	3'UTR	---
3	34421530	34421530	-	34421530	6	6	ENSSSCG00000028454	-	3'UTR	---
3	34421614	34421614	-	34421614	3	3	ENSSSCG00000028454	-	ORF	---
3	34424794	34424801	-	34424801	9	13	ENSSSCG00000028454	-	intron	---
3	34425858	34425866	-	34425861	14	28	ENSSSCG00000028454	-	intron	---
3	34426060	34426060	-	34426060	3	3	ENSSSCG00000028454	-	intron	---

3	34430625	34430625	-	34430625	4	4	ENSSSCG00000028454	-	intron	---
3	34437016	34437016	-	34437016	3	3	ENSSSCG00000028454	-	intron	---
3	34512253	34512254	-	34512254	4	7	---	---	intergenic	---
3	34623833	34623841	+	34623839	8	18	XLOC_054947	+	predicted	---
3	34636951	34636951	+	34636951	5	5	---	---	intergenic	---
3	34638344	34638352	-	34638352	10	14	XLOC_056209	-	predicted	---
3	34639512	34639512	-	34639512	3	3	XLOC_056209	-	predicted	---
3	34686117	34686126	-	34686117	10	16	XLOC_056211	-	predicted	---
3	34686829	34686829	-	34686829	4	4	ENSSSCG00000007907	-	3'UTR	*USP7*
3	34687073	34687098	-	34687079	31	51	ENSSSCG00000007907	-	3'UTR	*USP7*
3	34690412	34690419	-	34690419	7	14	ENSSSCG00000007907	-	ORF	*USP7*
3	34692693	34692693	-	34692693	3	3	ENSSSCG00000007907	-	intron	*USP7*
3	34729278	34729278	-	34729278	4	4	ENSSSCG00000007907	-	intron	*USP7*
3	34747908	34747915	-	34747908	6	16	XLOC_056212	-	predicted	---
3	34771037	34771040	-	34771040	19	28	---	---	intergenic	---
3	34778654	34778687	+	34778678	292	464	ENSSSCG00000026710	+	3'UTR	*CARHSP1*
3	34780840	34780846	+	34780843	35	65	ENSSSCG00000026710	+	3'UTR	*CARHSP1*
3	34789963	34789971	-	34789970	6	19	XLOC_056214	-	predicted	---
3	34802794	34802796	-	34802794	49	58	ENSSSCG00000021236	-	ORF	---
3	34806597	34806597	+	34806597	44	44	---	---	intergenic	---
3	34919546	34919554	-	34919552	18	37	ENSSSCG00000007914	-	3'UTR	---
3	35027920	35027920	+	35027920	3	3	---	---	intergenic	---
3	35362414	35362414	-	35362414	12	12	---	---	intergenic	---
3	35600030	35600030	-	35600030	4	4	---	---	intergenic	---
3	35742079	35742079	-	35742079	3	3	---	---	intergenic	---
3	35827305	35827306	+	35827305	3	6	---	---	intergenic	---

3	35922502	35922508	-	35922508	7	14	---	---	intergenic	---
3	35927968	35927968	-	35927968	3	3	---	---	intergenic	---
3	36173890	36173890	+	36173890	3	3	XLOC_054961	+	predicted	---
3	36297230	36297230	+	36297230	9	9	---	---	intergenic	---
3	36579522	36579522	+	36579522	4	4	---	---	intergenic	---
3	37092194	37092194	-	37092194	3	3	---	---	intergenic	---
3	37216883	37216883	+	37216883	9	9	---	---	intergenic	---
3	37350070	37350070	-	37350070	4	4	---	---	intergenic	---
3	37417959	37417959	+	37417959	3	3	---	---	intergenic	---
3	37450582	37450585	-	37450582	3	6	---	---	intergenic	---
3	37484802	37484802	+	37484802	3	3	---	---	intergenic	---
3	37645376	37645377	-	37645377	9	16	---	---	intergenic	---
3	37708429	37708429	+	37708429	4	4	---	---	intergenic	---
3	38022437	38022437	+	38022437	4	4	---	---	intergenic	---
3	38329048	38329048	-	38329048	4	4	XLOC_056228	-	predicted	---
3	38394592	38394592	+	38394592	5	5	ENSSSCG00000007925	+	3'UTR	*NAGPA*
3	38517778	38517781	+	38517779	13	28	XLOC_054969	+	predicted	---
3	38638299	38638299	+	38638299	6	6	ENSSSCG00000007931	+	ORF	*GLYR1*
3	38646405	38646405	+	38646405	4	4	---	---	intergenic	---
3	38651765	38651765	+	38651765	5	5	ENSSSCG00000007931	+	intron	*GLYR1*
3	38664615	38664622	+	38664617	23	45	XLOC_054972	+	predicted	---
3	38671059	38671064	+	38671064	17	28	XLOC_054972	+	predicted	---
3	38688201	38688222	+	38688216	21	36	XLOC_054973	+	predicted	---
3	38713439	38713445	-	38713439	26	41	XLOC_056235	-	predicted	---
3	38713536	38713574	-	38713567	20	46	XLOC_056235	-	predicted	---
3	38748317	38748331	-	38748330	24	64	XLOC_056236	-	predicted	---

3	38753410	38753418	-	38753418	12	23	XLOC_056236	-	predicted	---
3	38817353	38817353	+	38817353	3	3	ENSSSCG00000023603	+	3'UTR	*UBALD1*
3	38817692	38817700	+	38817692	4	14	ENSSSCG00000023603	+	3'UTR	*UBALD1*
3	38823033	38823041	-	38823033	5	15	XLOC_056238	-	predicted	---
3	38823994	38823994	-	38823994	3	3	ENSSSCG00000007937	-	3'UTR	---
3	38882381	38882381	-	38882381	6	6	XLOC_056240	-	predicted	---
3	38885191	38885191	+	38885191	3	3	---	---	intergenic	---
3	38894107	38894111	+	38894108	20	59	ENSSSCG00000007941	+	3'UTR	*CDIP1*
3	38895255	38895274	-	38895257	74	350	ENSSSCG00000007942	-	3'UTR	*HMOX2*
3	38895709	38895712	-	38895711	46	69	ENSSSCG00000007942	-	3'UTR	*HMOX2*
3	38896121	38896121	-	38896121	4	4	ENSSSCG00000007942	-	ORF	*HMOX2*
3	38896991	38896991	-	38896991	10	10	ENSSSCG00000007942	-	ORF	*HMOX2*
3	38934135	38934149	+	38934137	33	88	ENSSSCG00000007943	+	3'UTR	*NMRAL1*
3	38938178	38938185	-	38938178	7	10	XLOC_056241	-	predicted	---
3	38938250	38938250	-	38938250	4	4	XLOC_056241	-	predicted	---
3	38938989	38938991	-	38938989	10	13	XLOC_056241	-	predicted	---
3	39040261	39040261	+	39040261	16	16	ENSSSCG00000007944	+	3'UTR	---
3	39072056	39072128	-	39072120	13	66	XLOC_056244	-	predicted	---
3	39072996	39073002	-	39073001	16	62	---	---	intergenic	---
3	39131340	39131340	-	39131340	3	3	---	---	intergenic	---
3	39222901	39222901	+	39222901	3	3	---	---	intergenic	---
3	39240793	39240793	-	39240793	4	4	XLOC_056246	-	predicted	---
3	39474120	39474120	-	39474120	3	3	XLOC_056249	-	predicted	---
3	39479886	39479886	+	39479886	7	7	---	---	intergenic	---
3	39628855	39628855	+	39628855	7	7	ENSSSCG00000007951	+	intron	*CREBBP*
3	39669954	39669955	+	39669954	6	11	ENSSSCG00000007951	+	intron	*CREBBP*

3	39862835	39862840	+	39862837	40	79	---	---	intergenic	---
3	39893035	39893035	-	39893035	3	3	---	---	intergenic	---
3	40023347	40023350	+	40023349	6	16	XLOC_054993	+	predicted	---
3	40145552	40145552	-	40145552	3	3	XLOC_056257	-	predicted	---
3	40149713	40149713	+	40149713	3	3	---	---	intergenic	---
3	40162419	40162419	+	40162419	3	3	XLOC_054998	+	predicted	---
3	40293877	40293884	-	40293877	6	12	ENSSSCG00000007963	-	3'UTR	*ZNF263*
3	40336489	40336489	+	40336489	4	4	ENSSSCG00000007964	+	3'UTR	*MEFV*
3	40346241	40346241	+	40346241	3	3	ENSSSCG00000007965	+	intron	---
3	40357408	40357408	+	40357408	15	15	XLOC_055004	+	predicted	---
3	40357932	40357932	+	40357932	4	4	XLOC_055004	+	predicted	---
3	40417383	40417383	+	40417383	5	5	---	---	intergenic	---
3	40451697	40451697	-	40451697	3	3	---	---	intergenic	---
3	40451760	40451760	-	40451760	7	7	---	---	intergenic	---
3	40490459	40490461	-	40490459	6	11	XLOC_056267	-	predicted	---
3	40514862	40514863	+	40514862	15	20	XLOC_055009	+	predicted	---
3	40533678	40533678	+	40533678	6	6	XLOC_055011	+	predicted	---
3	40533926	40533926	-	40533926	9	9	ENSSSCG00000027252	-	3'UTR	*HCFC1R1*
3	40538524	40538540	+	40538528	5	14	XLOC_055011	+	predicted	---
3	40560217	40560220	+	40560217	6	18	ENSSSCG00000021569	+	intron	*MMP25*
3	40578639	40578639	+	40578639	14	14	XLOC_055013	+	predicted	---
3	40604787	40604788	-	40604788	5	8	---	---	intergenic	---
3	40612054	40612057	-	40612055	24	51	---	---	intergenic	---
3	40619798	40619798	+	40619798	3	3	ENSSSCG00000023304	+	intron	*SRRM2*
3	40623309	40623309	+	40623309	4	4	ENSSSCG00000023304	+	ORF	*SRRM2*
3	40625636	40625636	+	40625636	3	3	ENSSSCG00000023304	+	ORF	*SRRM2*

3	40631938	40631941	+	40631941	31	50	ENSSSCG00000023304	+	3'UTR	*SRRM2*
3	40631945	40631946	-	40631946	17	20	XLOC_056275	-	predicted	---
3	40632295	40632329	-	40632309	350	1349	ENSSSCG00000008061	-	3'UTR	*TCEB2*
3	40682764	40682770	+	40682765	15	39	XLOC_055016	+	predicted	---
3	40693538	40693538	+	40693538	3	3	ENSSSCG00000022630	+	3'UTR	*FLYWCH2*
3	40699349	40699350	-	40699350	11	17	XLOC_056277	-	predicted	---
3	40832692	40832692	+	40832692	5	5	---	---	intergenic	---
3	40861851	40861857	-	40861851	3	6	ENSSSCG00000007970	-	3'UTR	*POLR3K*
3	40861896	40861901	-	40861897	32	45	ENSSSCG00000007970	-	3'UTR	*POLR3K*
3	40869000	40869008	+	40869001	51	83	ENSSSCG00000007962	+	3'UTR	---
3	40869446	40869452	-	40869446	12	23	ENSSSCG00000007968	-	3'UTR	---
3	40897522	40897541	-	40897526	32	88	XLOC_056283	-	predicted	---
3	40897586	40897590	+	40897587	35	91	ENSSSCG00000007982	+	3'UTR	*MPG*
3	40947480	40947480	+	40947480	4	4	ENSSSCG00000007975	+	3'UTR	*HBZ*
3	40948917	40948935	+	40948919	379	892	XLOC_055020	+	predicted	---
3	40950761	40950761	+	40950761	7	7	ENSSSCG00000007978	+	ORF	---
3	40951413	40951421	+	40951413	35	38	ENSSSCG00000007978	+	3'UTR	*HBA*
3	40951504	40951558	+	40951532	9224	18911	ENSSSCG00000007978	+	3'UTR	*HBA*
3	40954471	40954471	+	40954471	7	7	XLOC_055020	+	predicted	---
3	40960782	40960782	-	40960782	4	4	ENSSSCG00000007979	-	3'UTR	*LUC7L*
3	40962914	40962923	-	40962919	22	86	ENSSSCG00000007979	-	3'UTR	*LUC7L*
3	40972539	40972539	-	40972539	3	3	ENSSSCG00000007979	-	ORF	*LUC7L*
3	41022110	41022111	+	41022111	14	18	ENSSSCG00000007980	+	3'UTR	---
3	41033961	41033961	-	41033961	6	6	ENSSSCG00000023204	-	3'UTR	*AXIN1*
3	41094503	41094510	-	41094506	160	345	ENSSSCG00000007984	-	3'UTR	*MRPL28*
3	41097593	41097593	-	41097593	9	9	XLOC_056288	-	predicted	---

3	41098632	41098632	-	41098632	5	5	XLOC_056288	-	predicted	---
3	41121917	41121925	+	41121917	6	9	ENSSSCG00000007986	+	3'UTR	*NME4*
3	41137039	41137049	+	41137043	30	64	XLOC_055027	+	predicted	---
3	41137275	41137278	+	41137275	4	7	XLOC_055027	+	predicted	---
3	41296830	41296830	+	41296830	3	3	ENSSSCG00000007993	+	3'UTR	*CAPN15*
3	41344719	41344720	-	41344720	7	12	---	---	intergenic	---
3	41353417	41353431	+	41353425	20	52	ENSSSCG00000007995	+	3'UTR	*FAM195A*
3	41369617	41369617	+	41369617	4	4	ENSSSCG00000007991	+	3'UTR	*WDR90*
3	41377030	41377030	+	41377030	3	3	ENSSSCG00000007998	+	intron	*RHBDL1*
3	41380723	41380723	+	41380723	3	3	XLOC_055035	+	predicted	---
3	41380913	41380915	-	41380915	10	18	ENSSSCG00000007999	-	3'UTR	---
3	41383996	41384006	-	41384003	7	30	XLOC_056295	-	predicted	---
3	41385100	41385101	+	41385100	20	25	XLOC_055035	+	predicted	---
3	41387150	41387151	-	41387150	7	13	ENSSSCG00000008007	-	3'UTR	*WDR24*
3	41394129	41394129	-	41394129	6	6	ENSSSCG00000008006	-	3'UTR	*FBXL16*
3	41402094	41402094	-	41402094	3	3	ENSSSCG00000008006	-	intron	*FBXL16*
3	41420184	41420187	+	41420185	47	69	ENSSSCG00000008004	+	3'UTR	*FAM173A*
3	41427865	41427865	+	41427865	4	4	ENSSSCG00000008003	+	3'UTR	*HAGHL*
							ENSSSCG00000008003	+	intron	*HAGHL*
3	41427901	41427903	-	41427903	5	8	XLOC_056297	-	predicted	---
3	41452323	41452323	-	41452323	3	3	---	---	intergenic	---
3	41464901	41464908	-	41464901	11	20	XLOC_056299	-	predicted	---
3	41548551	41548562	-	41548552	15	60	ENSSSCG00000008009	-	3'UTR	*LMF1*
3	41633569	41633572	+	41633571	8	24	ENSSSCG00000008014	+	3'UTR	*NUBP2*
3	41634232	41634235	-	41634235	8	12	ENSSSCG00000008013	-	3'UTR	*ALS*
3	41648914	41648991	-	41648942	356	913	ENSSSCG00000008012	-	3'UTR	*HAGH*

3	41659255	41659267	+	41659262	187	418	ENSSSCG00000008016	+	3'UTR	*MRPS34*
3	41660780	41660821	+	41660817	48	238	ENSSSCG00000008017	+	3'UTR	*NME3*
3	41660822	41660822	-	41660822	6	6	XLOC_056307	-	predicted	---
3	41681372	41681373	-	41681373	21	24	ENSSSCG00000008018	-	intron	*MAPK8IP3*
3	41708349	41708350	-	41708349	6	10	ENSSSCG00000008019	-	3'UTR	*HN1L*
3	41709019	41709030	-	41709029	30	72	ENSSSCG00000008019	-	3'UTR	*HN1L*
3	41721844	41721844	-	41721844	5	5	XLOC_056308	-	predicted	---
3	41806130	41806133	-	41806130	4	7	ENSSSCG00000008021	-	3'UTR	*TMEM204*
3	41835311	41835311	+	41835311	7	7	XLOC_055043	+	predicted	---
3	41835390	41835390	-	41835390	3	3	XLOC_056311	-	predicted	---
3	41966990	41966997	+	41966990	20	36	ENSSSCG00000021560	+	3'UTR	*UBE2I*
3	41986786	41986795	-	41986790	9	22	ENSSSCG00000008030	-	3'UTR	*TSR3*
3	41986995	41986995	+	41986995	3	3	XLOC_055048	+	predicted	---
3	42010609	42010609	+	42010609	6	6	ENSSSCG00000008031	+	3'UTR	*GNPTG*
3	42011395	42011395	+	42011395	3	3	ENSSSCG00000008031	+	3'UTR	*GNPTG*
3	42082445	42082456	-	42082449	23	42	ENSSSCG00000024405	-	3'UTR	*C16orf91*
3	42198145	42198145	+	42198145	5	5	ENSSSCG00000008035	+	ORF	*GFER*
3	42199294	42199315	+	42199312	242	747	ENSSSCG00000008035	+	3'UTR	*GFER*
3	42199765	42199775	+	42199774	19	49	ENSSSCG00000008035	+	3'UTR	*GFER*
3	42209054	42209064	-	42209054	4	12	ENSSSCG00000023400	-	3'UTR	*ZNF598*
3	42238711	42238711	+	42238711	8	8	ENSSSCG00000008038	+	3'UTR	*SLC9A3R2*
3	42239172	42239178	+	42239172	5	11	ENSSSCG00000008038	+	3'UTR	*SLC9A3R2*
3	42239827	42239831	-	42239831	5	8	ENSSSCG00000008039	-	3'UTR	*NTHL1*
3	42282085	42282105	+	42282085	7	14	ENSSSCG00000008040	+	3'UTR	*TSC2*
3	42282624	42282624	+	42282624	4	4	ENSSSCG00000008040	+	3'UTR	*TSC2*
3	42398652	42398654	+	42398653	9	15	ENSSSCG00000008044	+	3'UTR	*MLST8*

3	42402236	42402238	-	42402237	10	16	XLOC_056332	-	predicted	---
3	42402998	42402998	-	42402998	3	3	XLOC_056332	-	predicted	---
3	42418797	42418797	+	42418797	3	3	XLOC_055061	+	predicted	---
3	42422449	42422454	-	42422453	108	192	ENSSSCG00000024587	-	3'UTR	*ECI1*
3	42422688	42422693	-	42422692	17	34	ENSSSCG00000024587	-	3'UTR	*ECI1*
3	42422814	42422814	-	42422814	3	3	ENSSSCG00000024587	-	ORF	*ECI1*
3	42433970	42433976	-	42433970	9	20	ENSSSCG00000008051	-	3'UTR	---
3	42513096	42513096	+	42513096	7	7	ENSSSCG00000008048	+	ORF	*RNPS1*
3	42520028	42520064	+	42520049	109	229	XLOC_055065	+	predicted	---
3	42521064	42521065	-	42521064	3	6	---	---	intergenic	---
3	42560671	42560699	+	42560671	4	8	XLOC_055066	+	predicted	---
3	42700740	42700740	-	42700740	9	9	XLOC_056343	-	predicted	---
3	42706484	42706516	+	42706484	12	16	ENSSSCG00000008056	+	3'UTR	*ATP6V0C*
3	42706547	42706547	+	42706547	6	6	ENSSSCG00000008056	+	3'UTR	*ATP6V0C*
3	42706827	42706840	+	42706833	288	657	ENSSSCG00000008056	+	3'UTR	*ATP6V0C*
3	42716473	42716474	+	42716474	21	26	ENSSSCG00000008057	+	3'UTR	---
3	42777239	42777244	+	42777239	8	12	XLOC_055078	+	predicted	---
3	42974914	42974918	-	42974914	5	9	ENSSSCG00000008068	-	intron	---
3	43030230	43030234	-	43030234	14	20	ENSSSCG00000024292	-	3'UTR	---
3	43111468	43111468	+	43111468	3	3	XLOC_055082	+	predicted	---
3	43259172	43259178	+	43259172	5	10	ENSSSCG00000030143	+	3'UTR	*OGN*
3	43350297	43350297	-	43350297	6	6	ENSSSCG00000008072	-	3'UTR	*ASPN*
3	43389935	43389935	-	43389935	4	4	ENSSSCG00000028244	-	3'UTR	---
3	43389981	43389986	-	43389981	7	24	ENSSSCG00000028244	-	3'UTR	---
3	43492146	43492146	-	43492146	5	5	ENSSSCG00000030204	-	3'UTR	---
3	43492711	43492712	+	43492711	9	13	XLOC_055086	+	predicted	---

3	43493723	43493723	-	43493723	3	3	ENSSSCG00000030204	-	3'UTR	---
3	43627510	43627510	-	43627510	3	3	XLOC_056359	-	predicted	---
3	43688242	43688242	-	43688242	3	3	---	---	intergenic	---
3	43981109	43981117	-	43981117	26	82	ENSSSCG00000008077	-	3'UTR	*C9orf89*
3	43999829	43999829	+	43999829	3	3	---	---	intergenic	---
3	44044520	44044525	-	44044522	61	154	XLOC_056364	-	predicted	---
3	44278691	44278691	+	44278691	8	8	ENSSSCG00000008079	+	intron	*FAM120A*
3	44330360	44330360	+	44330360	7	7	ENSSSCG00000008079	+	3'UTR	*FAM120A*
3	44330541	44330543	+	44330541	3	6	ENSSSCG00000008079	+	3'UTR	*FAM120A*
3	44330937	44330940	+	44330939	18	28	ENSSSCG00000008079	+	3'UTR	*FAM120A*
3	44430584	44430586	+	44430584	3	6	ENSSSCG00000008080	+	intron	*PHF2*
3	44434167	44434171	+	44434171	5	8	ENSSSCG00000008080	+	3'UTR	*PHF2*
3	44480711	44480711	-	44480711	7	7	---	---	intergenic	---
3	44632922	44632922	-	44632922	3	3	---	---	intergenic	---
3	44640029	44640029	-	44640029	3	3	---	---	intergenic	---
3	44924278	44924278	+	44924278	3	3	---	---	intergenic	---
3	45067381	45067381	-	45067381	3	3	---	---	intergenic	---
3	45293109	45293110	+	45293109	20	27	---	---	intergenic	---
3	45452997	45452997	-	45452997	4	4	XLOC_056382	-	predicted	---
3	45681452	45681452	-	45681452	4	4	XLOC_056386	-	predicted	---
3	45716185	45716185	-	45716185	3	3	ENSSSCG00000008096	-	ORF	*ZC3H6*
3	45728109	45728111	-	45728109	5	13	---	---	intergenic	---
3	45750608	45750615	+	45750611	21	42	---	---	intergenic	---
3	45768928	45768928	+	45768928	17	17	ENSSSCG00000008099	+	intron	*ZC3H8*
3	46168423	46168426	-	46168423	9	19	---	---	intergenic	---
3	46303999	46304000	+	46304000	11	17	ENSSSCG00000008105	+	intron	---

3	46326361	46326362	+	46326361	7	13	ENSSSCG00000008105	+	3'UTR	---
3	46366984	46366984	-	46366984	10	10	ENSSSCG00000008103	-	intron	---
3	46640043	46640043	-	46640043	3	3	---	---	intergenic	---
3	46831241	46831241	+	46831241	4	4	---	---	intergenic	---
3	46910346	46910346	+	46910346	5	5	---	---	intergenic	---
3	47005271	47005271	-	47005271	4	4	---	---	intergenic	---
3	47006952	47006952	+	47006952	3	3	XLOC_055126	+	predicted	---
3	47456337	47456355	-	47456346	29	103	ENSSSCG00000022937	-	3'UTR	---
							ENSSSCG00000008109	-	intron	---
3	47456433	47456449	-	47456447	27	54	ENSSSCG00000022937	-	3'UTR	---
							ENSSSCG00000008109	-	intron	---
3	47580856	47580856	+	47580856	6	6	ENSSSCG00000030469	+	ORF	*BUB1*
3	47685110	47685110	-	47685110	7	7	---	---	intergenic	---
3	47754731	47754731	+	47754731	4	4	---	---	intergenic	---
3	47765032	47765032	-	47765032	4	4	XLOC_056395	-	predicted	---
3	47766794	47766795	-	47766794	4	7	XLOC_056395	-	predicted	---
3	47791249	47791249	+	47791249	14	14	ENSSSCG00000023050	+	ORF	*SEPT10*
3	47830642	47830645	+	47830645	13	19	XLOC_055131	+	predicted	---
3	47831287	47831288	+	47831288	9	12	XLOC_055131	+	predicted	---
3	47844241	47844241	-	47844241	4	4	---	---	intergenic	---
3	47876112	47876112	+	47876112	9	9	---	---	intergenic	---
3	47883614	47883614	-	47883614	3	3	---	---	intergenic	---
3	47912798	47912799	+	47912798	13	16	XLOC_055133	+	predicted	---
3	47952789	47952802	-	47952802	10	14	ENSSSCG00000008111	-	3'UTR	*NPHP1*
3	47952898	47952917	-	47952899	12	27	ENSSSCG00000008111	-	3'UTR	*NPHP1*
3	47978985	47978987	-	47978987	79	159	ENSSSCG00000008111	-	intron	*NPHP1*

3	47997040	47997040	-	47997040	3	3	---	---	intergenic	---
3	48016400	48016418	-	48016408	14	43	XLOC_056398	-	predicted	---
3	48137536	48137536	-	48137536	3	3	XLOC_056402	-	predicted	---
3	48235308	48235318	+	48235308	8	28	XLOC_055136	+	predicted	---
3	48439216	48439224	+	48439224	6	10	ENSSSCG00000008119	+	3'UTR	*KCNIP3*
3	48487130	48487137	+	48487132	90	207	ENSSSCG00000008120	+	3'UTR	---
3	48487221	48487225	-	48487222	15	24	XLOC_056411	-	predicted	---
3	48607659	48607659	-	48607659	7	7	---	---	intergenic	---
3	48632481	48632481	+	48632481	3	3	---	---	intergenic	---
3	48727395	48727400	-	48727398	11	37	---	---	intergenic	---
3	48848384	48848385	-	48848385	7	10	ENSSSCG00000008123	-	3'UTR	*ARID5A*
3	48902933	48902933	-	48902933	3	3	---	---	intergenic	---
3	48999156	48999158	-	48999156	20	27	XLOC_056417	-	predicted	---
3	48999408	48999408	-	48999408	4	4	XLOC_056417	-	predicted	---
3	49088234	49088234	-	49088234	3	3	---	---	intergenic	---
3	49102258	49102269	+	49102260	85	118	ENSSSCG00000008129	+	3'UTR	---
3	49102307	49102308	+	49102308	11	15	XLOC_055150	+	predicted	---
3	49121830	49121830	+	49121830	11	11	XLOC_055151	+	predicted	---
3	49159346	49159346	+	49159346	3	3	ENSSSCG00000008132	+	3'UTR	*STARD7*
3	49159683	49159688	+	49159687	31	60	ENSSSCG00000008132	+	3'UTR	*STARD7*
3	49161234	49161234	+	49161234	3	3	XLOC_055152	+	predicted	---
3	49194988	49194991	+	49194988	13	21	ENSSSCG00000008128	+	3'UTR	*DUSP2*
3	49435090	49435090	-	49435090	3	3	---	---	intergenic	---
3	49461762	49461763	+	49461763	18	23	---	---	intergenic	---
3	49685958	49685958	+	49685958	3	3	XLOC_055162	+	predicted	---
3	49693974	49693984	-	49693974	6	12	ENSSSCG00000008136	-	3'UTR	*RANBP2*

3	49694447	49694461	-	49694447	3	6	ENSSSCG00000008136	-	3'UTR	*RANBP2*
3	49694651	49694653	-	49694652	36	48	ENSSSCG00000008136	-	3'UTR	*RANBP2*
3	49717346	49717346	-	49717346	5	5	ENSSSCG00000008136	-	ORF	*RANBP2*
3	49811883	49811883	+	49811883	12	12	ENSSSCG00000008137	+	3'UTR	*LIMS1*
3	49813537	49813537	+	49813537	4	4	XLOC_055163	+	predicted	---
3	49824430	49824431	+	49824430	9	14	ENSSSCG00000023884	+	intron	---
3	49887514	49887514	-	49887514	3	3	ENSSSCG00000008138	-	3'UTR	*GCC2*
3	49887599	49887599	-	49887599	3	3	ENSSSCG00000008138	-	3'UTR	*GCC2*
3	49887708	49887710	-	49887708	6	10	ENSSSCG00000008138	-	3'UTR	*GCC2*
3	49901285	49901285	-	49901285	3	3	ENSSSCG00000008138	-	ORF	*GCC2*
3	49901333	49901333	-	49901333	3	3	ENSSSCG00000008138	-	ORF	*GCC2*
3	49903796	49903796	-	49903796	3	3	ENSSSCG00000008138	-	intron	*GCC2*
							ENSSSCG00000008138	-	ORF	*GCC2*
3	49919408	49919408	-	49919408	3	3	ENSSSCG00000008138	-	ORF	*GCC2*
3	49920230	49920231	-	49920230	11	14	ENSSSCG00000008138	-	ORF	*GCC2*
3	49934287	49934296	-	49934294	24	48	ENSSSCG00000025836	-	3'UTR	*SULT1C4*
3	49934491	49934491	-	49934491	3	3	ENSSSCG00000025836	-	3'UTR	*SULT1C4*
3	49939410	49939410	-	49939410	3	3	ENSSSCG00000025836	-	intron	*SULT1C4*
3	49977269	49977269	-	49977269	4	4	XLOC_056434	-	predicted	---
3	50008062	50008070	-	50008069	25	36	XLOC_056435	-	predicted	---
3	50008168	50008190	-	50008188	78	167	ENSSSCG00000028691	-	3'UTR	*SULT1C3*
3	50009424	50009424	-	50009424	3	3	ENSSSCG00000028691	-	ORF	*SULT1C3*
3	50009635	50009635	-	50009635	5	5	ENSSSCG00000028691	-	ORF	*SULT1C3*
3	50033258	50033258	-	50033258	5	5	ENSSSCG00000028691	-	intron	*SULT1C3*
3	50114428	50114428	-	50114428	6	6	---	---	intergenic	---
3	50308541	50308541	-	50308541	4	4	---	---	intergenic	---

3	50719515	50719517	-	50719515	3	6	XLOC_056439	-	predicted	---
3	50797855	50797858	+	50797858	5	13	---	---	intergenic	---
3	50918068	50918068	-	50918068	5	5	---	---	intergenic	---
3	50977039	50977039	-	50977039	5	5	---	---	intergenic	---
3	51062803	51062803	+	51062803	3	3	ENSSSCG00000008142	+	3'UTR	*C2orf40*
3	51082490	51082502	-	51082502	11	20	ENSSSCG00000008143	-	3'UTR	*UXS1*
3	51082844	51082844	-	51082844	7	7	ENSSSCG00000008143	-	3'UTR	*UXS1*
3	51141370	51141370	-	51141370	4	4	---	---	intergenic	---
3	51358801	51358803	-	51358801	14	17	XLOC_056443	-	predicted	---
3	51439309	51439309	-	51439309	7	7	---	---	intergenic	---
3	51476738	51476738	+	51476738	3	3	---	---	intergenic	---
3	51608805	51608806	-	51608806	12	16	XLOC_056446	-	predicted	---
3	51743638	51743639	+	51743638	11	14	---	---	intergenic	---
3	51798841	51798843	-	51798841	4	7	ENSSSCG00000024636	-	3'UTR	*TGFBRAP1*
3	51883485	51883491	+	51883485	5	14	ENSSSCG00000028020	+	ORF	*MRPS9*
3	51887400	51887400	+	51887400	10	10	ENSSSCG00000028020	+	intron	*MRPS9*
3	51891400	51891408	+	51891406	36	81	ENSSSCG00000028020	+	3'UTR	*MRPS9*
3	51891871	51891876	+	51891876	13	21	ENSSSCG00000028020	+	3'UTR	*MRPS9*
3	51892021	51892028	+	51892028	5	8	ENSSSCG00000028020	+	3'UTR	*MRPS9*
3	52210662	52210662	-	52210662	3	3	---	---	intergenic	---
3	52276463	52276469	-	52276463	8	14	---	---	intergenic	---
3	52386666	52386674	+	52386674	5	9	---	---	intergenic	---
3	52520784	52520784	+	52520784	4	4	---	---	intergenic	---
3	52629957	52629957	+	52629957	3	3	---	---	intergenic	---
3	52664502	52664502	-	52664502	3	3	XLOC_056454	-	predicted	---
3	52828706	52828706	-	52828706	3	3	---	---	intergenic	---

3	53088913	53088916	-	53088916	4	7	---	---	intergenic	---
3	53290126	53290127	+	53290126	9	14	---	---	intergenic	---
3	53368418	53368418	+	53368418	3	3	---	---	intergenic	---
3	53420920	53420920	-	53420920	5	5	---	---	intergenic	---
3	53870962	53870962	-	53870962	3	3	ENSSSCG00000008150	-	intron	*TMEM182*
3	53925890	53925890	-	53925890	8	8	---	---	intergenic	---
3	53960466	53960466	-	53960466	3	3	XLOC_056457	-	predicted	---
3	54368666	54368666	+	54368666	10	10	---	---	intergenic	---
3	54703318	54703319	+	54703318	6	12	---	---	intergenic	---
3	54729117	54729131	-	54729121	61	220	ENSSSCG00000008162	-	3'UTR	*IL1R1*
3	54729759	54729759	-	54729759	7	7	ENSSSCG00000008162	-	3'UTR	*IL1R1*
3	54730579	54730579	-	54730579	4	4	ENSSSCG00000008162	-	3'UTR	*IL1R1*
3	54730619	54730653	-	54730619	3	6	ENSSSCG00000008162	-	3'UTR	*IL1R1*
3	54745462	54745462	-	54745462	3	3	---	---	intergenic	---
3	54757438	54757438	-	54757438	3	3	---	---	intergenic	---
3	54901383	54901383	-	54901383	3	3	ENSSSCG00000028331	-	3'UTR	*IL1R2*
3	55031487	55031487	-	55031487	6	6	ENSSSCG00000008164	-	3'UTR	*MAP4K4*
3	55033530	55033540	-	55033540	7	14	ENSSSCG00000008164	-	3'UTR	*MAP4K4*
3	55034872	55034888	-	55034872	5	10	ENSSSCG00000008164	-	3'UTR	*MAP4K4*
3	55042685	55042685	-	55042685	3	3	ENSSSCG00000008164	-	intron	*MAP4K4*
							ENSSSCG00000008164	-	ORF	*MAP4K4*
3	55086816	55086816	-	55086816	3	3	ENSSSCG00000008164	-	intron	*MAP4K4*
3	55481712	55481712	+	55481712	5	5	ENSSSCG00000008166	+	intron	*CREG2*
3	55605564	55605564	+	55605564	3	3	ENSSSCG00000008168	+	3'UTR	*RNF149*
3	55605718	55605725	+	55605724	19	31	ENSSSCG00000008168	+	3'UTR	*RNF149*
3	55610359	55610361	-	55610360	26	34	ENSSSCG00000008167	-	3'UTR	*CNOT11*

3	55611007	55611007	-	55611007	3	3	ENSSSCG00000008167	-	3'UTR	*CNOT11*
3	55704440	55704445	-	55704440	5	11	ENSSSCG00000008170	-	intron	*RPL31*
3	55835476	55835476	+	55835476	7	7	XLOC_055205	+	predicted	---
3	55836223	55836235	-	55836227	204	528	ENSSSCG00000008170	-	intron	*RPL31*
3	55836586	55836586	-	55836586	3	3	ENSSSCG00000008170	-	ORF	*RPL31*
3	55836632	55836632	-	55836632	3	3	ENSSSCG00000008170	-	ORF	*RPL31*
3	55837856	55837856	-	55837856	4	4	ENSSSCG00000008170	-	ORF	*RPL31*
3	56095372	56095372	+	56095372	3	3	---	---	intergenic	---
3	56255383	56255384	+	56255383	7	10	XLOC_055209	+	predicted	---
3	56256182	56256182	+	56256182	3	3	XLOC_055209	+	predicted	---
3	56427936	56427937	-	56427936	6	9	---	---	intergenic	---
3	56484853	56484860	+	56484859	20	26	ENSSSCG00000008175	+	3'UTR	*CHST10*
3	56665733	56665733	+	56665733	4	4	---	---	intergenic	---
3	56750890	56750890	-	56750890	3	3	---	---	intergenic	---
3	57083081	57083087	+	57083087	5	12	ENSSSCG00000008177	+	intron	*AFF3*
3	57389367	57389367	+	57389367	3	3	ENSSSCG00000008179	+	intron	*REV1*
3	57497493	57497493	-	57497493	3	3	ENSSSCG00000008180	-	3'UTR	*EIF5B*
3	57497866	57497866	+	57497866	4	4	ENSSSCG00000008179	+	3'UTR	*REV1*
3	57498029	57498029	-	57498029	5	5	ENSSSCG00000008180	-	3'UTR	*EIF5B*
3	57499009	57499012	-	57499009	11	15	ENSSSCG00000008180	-	3'UTR	*EIF5B*
3	57537115	57537140	-	57537115	14	20	ENSSSCG00000008180	-	intron	*EIF5B*
3	57540167	57540167	-	57540167	7	7	ENSSSCG00000008180	-	intron	*EIF5B*
3	57540405	57540405	-	57540405	3	3	---	---	intergenic	---
3	57578822	57578840	+	57578837	9	19	ENSSSCG00000008181	+	3'UTR	*TXNDC9*
3	57579235	57579242	+	57579242	6	15	ENSSSCG00000008181	+	3'UTR	*TXNDC9*
3	57676957	57676976	-	57676970	63	133	ENSSSCG00000008183	-	3'UTR	---

3	57703818	57703818	+	57703818	3	3	ENSSSCG00000008185	+	3'UTR	*MITD1*
3	57782381	57782382	+	57782382	6	10	ENSSSCG00000008186	+	ORF	*TSGA10*
3	57786651	57786652	+	57786652	4	7	ENSSSCG00000008186	+	intron	*TSGA10*
3	57812435	57812459	+	57812435	3	6	ENSSSCG00000008186	+	ORF	*TSGA10*
3	57815538	57815539	+	57815539	6	9	ENSSSCG00000008186	+	ORF	*TSGA10*
3	57815616	57815616	+	57815616	4	4	ENSSSCG00000008186	+	intron	*TSGA10*
3	57867725	57867725	-	57867725	3	3	---	---	intergenic	---
3	57871715	57871715	+	57871715	3	3	XLOC_055224	+	predicted	---
3	57871823	57871826	+	57871823	6	10	XLOC_055224	+	predicted	---
3	57872191	57872192	+	57872191	3	6	XLOC_055224	+	predicted	---
3	57873443	57873443	+	57873443	3	3	XLOC_055224	+	predicted	---
3	58076442	58076442	-	58076442	7	7	---	---	intergenic	---
3	58217699	58217707	+	58217707	6	9	XLOC_055227	+	predicted	---
3	58220281	58220281	-	58220281	13	13	XLOC_056481	-	predicted	---
3	58220339	58220339	-	58220339	3	3	ENSSSCG00000025010	-	3'UTR	*UNC50*
3	58220391	58220391	-	58220391	3	3	ENSSSCG00000025010	-	3'UTR	*UNC50*
3	58227938	58227938	-	58227938	3	3	ENSSSCG00000025010	-	intron	*UNC50*
3	58239464	58239464	+	58239464	3	3	XLOC_055228	+	predicted	---
3	58239550	58239559	+	58239553	44	132	XLOC_055228	+	predicted	---
3	58261022	58261022	-	58261022	16	16	---	---	intergenic	---
3	58263620	58263620	-	58263620	7	7	ENSSSCG00000008190	-	3'UTR	*INPP4A*
3	58266073	58266074	-	58266073	3	6	ENSSSCG00000008190	-	3'UTR	*INPP4A*
3	58448665	58448665	-	58448665	3	3	---	---	intergenic	---
3	58565678	58565678	+	58565678	3	3	---	---	intergenic	---
3	58676951	58676956	-	58676951	18	32	---	---	intergenic	---
3	58977963	58977963	+	58977963	4	4	ENSSSCG00000008192	+	intron	*TMEM131*

3	59021949	59021949	+	59021949	73	73	ENSSSCG00000008192	+	intron	*TMEM131*
3	59024943	59024943	+	59024943	4	4	ENSSSCG00000008192	+	intron	*TMEM131*
3	59040062	59040069	+	59040062	16	22	ENSSSCG00000008192	+	3'UTR	*TMEM131*
3	59135457	59135458	-	59135457	9	12	ENSSSCG00000008193	-	3'UTR	*ZAP70*
3	59140416	59140416	-	59140416	3	3	ENSSSCG00000008193	-	intron	*ZAP70*
3	59225527	59225528	-	59225528	16	19	ENSSSCG00000008193	-	intron	*ZAP70*
3	59233939	59233940	-	59233940	12	16	ENSSSCG00000008198	-	3'UTR	*ANKRD39*
							ENSSSCG00000008193	-	intron	*ZAP70*
3	59261809	59261813	-	59261810	35	62	ENSSSCG00000008193	-	intron	*ZAP70*
3	59322054	59322054	-	59322054	3	3	ENSSSCG00000008196	-	intron	*FAM178B*
3	59327556	59327556	-	59327556	3	3	ENSSSCG00000008196	-	intron	*FAM178B*
3	59377455	59377455	+	59377455	3	3	ENSSSCG00000008195	+	intron	*COX5B*
3	59378243	59378267	+	59378262	376	1204	ENSSSCG00000008195	+	3'UTR	*COX5B*
3	59388417	59388425	-	59388417	14	36	ENSSSCG00000008194	-	3'UTR	*ACTR1B*
3	59447568	59447576	-	59447569	29	52	ENSSSCG00000008201	-	3'UTR	*CNNM3*
3	59720703	59720703	+	59720703	3	3	XLOC_055241	+	predicted	---
3	59834040	59834046	-	59834040	18	26	XLOC_056497	-	predicted	---
3	59880798	59880824	-	59880798	53	92	ENSSSCG00000008203	-	3'UTR	*IGKC*
3	60020081	60020084	+	60020081	3	6	---	---	intergenic	---
3	60030054	60030062	-	60030054	3	6	XLOC_056505	-	predicted	---
3	60132820	60132831	-	60132824	21	81	---	---	intergenic	---
3	60418994	60418995	-	60418995	4	7	---	---	intergenic	---
3	60473244	60473244	+	60473244	4	4	---	---	intergenic	---
3	60494663	60494669	-	60494664	31	92	ENSSSCG00000025106	-	3'UTR	---
3	60554510	60554512	+	60554510	6	11	XLOC_055248	+	predicted	---
3	60611510	60611510	+	60611510	4	4	ENSSSCG00000008213	+	intron	*CD8B*

3	60622643	60622668	-	60622646	524	800	ENSSSCG00000008214	-	3'UTR	*FABP1*
3	60627099	60627105	+	60627103	8	18	ENSSSCG00000008213	+	intron	*CD8B*
3	60679557	60679557	+	60679557	4	4	ENSSSCG00000008213	+	3'UTR	*CD8B*
3	60727464	60727464	+	60727464	4	4	---	---	intergenic	---
3	60840617	60840617	+	60840617	3	3	ENSSSCG00000008216	+	3'UTR	*RMND5A*
3	60844227	60844234	+	60844234	6	9	ENSSSCG00000008216	+	3'UTR	*RMND5A*
3	60844462	60844470	+	60844463	13	35	ENSSSCG00000008216	+	3'UTR	*RMND5A*
3	60941732	60941733	+	60941732	11	16	ENSSSCG00000008218	+	3'UTR	---
3	60964160	60964160	-	60964160	4	4	XLOC_056517	-	predicted	---
3	61035533	61035533	+	61035533	4	4	---	---	intergenic	---
3	61037027	61037027	+	61037027	10	10	ENSSSCG00000008222	+	3'UTR	---
3	61037173	61037191	+	61037176	49	153	ENSSSCG00000008222	+	3'UTR	---
3	61060330	61060330	-	61060330	6	6	ENSSSCG00000008221	-	3'UTR	*KDM3A*
3	61066205	61066205	-	61066205	3	3	ENSSSCG00000008221	-	intron	*KDM3A*
3	61126365	61126365	+	61126365	3	3	---	---	intergenic	---
3	61258369	61258369	+	61258369	3	3	ENSSSCG00000021746	+	intron	*MRPL35*
3	61261276	61261292	+	61261284	25	53	ENSSSCG00000021746	+	3'UTR	*MRPL35*
3	61263065	61263065	+	61263065	6	6	ENSSSCG00000021746	+	3'UTR	*MRPL35*
3	61264886	61264886	-	61264886	8	8	ENSSSCG00000023710	-	3'UTR	*REEP1*
3	61264975	61264975	-	61264975	3	3	ENSSSCG00000023710	-	3'UTR	*REEP1*
3	61351785	61351786	+	61351786	4	7	---	---	intergenic	---
3	61492655	61492655	-	61492655	13	13	ENSSSCG00000008225	-	3'UTR	*PTCD3*
3	61493759	61493759	-	61493759	5	5	ENSSSCG00000008225	-	3'UTR	*PTCD3*
3	61609801	61609812	+	61609801	8	16	ENSSSCG00000008226	+	3'UTR	*POLR1A*
3	61645744	61645746	+	61645745	108	166	---	---	intergenic	---
3	61650144	61650144	-	61650144	4	4	---	---	intergenic	---

3	61650941	61650941	+	61650941	32	32	---	---	intergenic	---
3	61832922	61832929	-	61832922	5	12	XLOC_056527	-	predicted	---
3	61924835	61924836	-	61924836	19	27	ENSSSCG00000027683	-	3'UTR	*USP39*
3	61931658	61931658	-	61931658	4	4	ENSSSCG00000027683	-	intron	*USP39*
3	61957915	61957924	+	61957919	220	628	XLOC_055267	+	predicted	---
3	62131760	62131760	-	62131760	3	3	ENSSSCG00000022300	-	3'UTR	*MATA2*
3	62132025	62132025	-	62132025	7	7	ENSSSCG00000022300	-	3'UTR	*MATA2*
3	62132571	62132571	-	62132571	3	3	ENSSSCG00000022300	-	intron	*MATA2*
3	62137694	62137694	-	62137694	3	3	XLOC_056531	-	predicted	---
3	62154035	62154035	-	62154035	3	3	---	---	intergenic	---
3	62165718	62165720	+	62165720	6	14	---	---	intergenic	---
3	62249015	62249027	-	62249018	208	491	ENSSSCG00000022820	-	3'UTR	*VAMP8*
3	62303464	62303476	+	62303466	17	41	XLOC_055278	+	predicted	---
3	62304348	62304355	+	62304348	3	6	XLOC_055278	+	predicted	---
3	62307508	62307508	-	62307508	3	3	XLOC_056535	-	predicted	---
3	62307572	62307572	-	62307572	15	15	XLOC_056535	-	predicted	---
3	62307661	62307678	-	62307661	4	11	XLOC_056535	-	predicted	---
3	62354553	62354558	+	62354558	14	28	ENSSSCG00000008238	+	3'UTR	*ELMOD3*
3	62357515	62357534	-	62357531	66	185	ENSSSCG00000008239	-	3'UTR	*CAPG*
3	62405069	62405069	+	62405069	11	11	ENSSSCG00000008240	+	3'UTR	*SH2D6*
3	62472928	62472929	-	62472928	6	10	---	---	intergenic	---
3	62618539	62618539	-	62618539	5	5	XLOC_056540	-	predicted	---
3	62620033	62620042	-	62620034	41	98	ENSSSCG00000008242	-	3'UTR	*KCMF1*
3	62620338	62620338	-	62620338	4	4	ENSSSCG00000008242	-	3'UTR	*KCMF1*
3	62620411	62620411	-	62620411	14	14	ENSSSCG00000008242	-	ORF	*KCMF1*
3	62821352	62821372	-	62821352	7	12	ENSSSCG00000008245	-	ORF	*TMSB10*

3	62862013	62862013	+	62862013	7	7	XLOC_055284	+	predicted	---
3	62896612	62896612	+	62896612	6	6	---	---	intergenic	---
3	62904413	62904415	-	62904413	4	10	XLOC_056542	-	predicted	---
3	63171645	63171645	+	63171645	4	4	ENSSSCG00000008247	+	intron	*SUCLG1*
3	63174801	63174813	+	63174811	228	428	ENSSSCG00000008247	+	3'UTR	*SUCLG1*
3	63178037	63178046	-	63178044	49	156	---	---	intergenic	---
3	63252015	63252015	-	63252015	3	3	---	---	intergenic	---
3	65037359	65037359	+	65037359	6	6	---	---	intergenic	---
3	65141553	65141553	-	65141553	3	3	---	---	intergenic	---
3	65333994	65333994	+	65333994	3	3	---	---	intergenic	---
3	66285686	66285695	-	66285693	12	28	ENSSSCG00000008250	-	3'UTR	*CTNNA2*
3	66943634	66943634	+	66943634	6	6	---	---	intergenic	---
3	66969833	66969833	+	66969833	9	9	---	---	intergenic	---
3	67087853	67087853	+	67087853	8	8	---	---	intergenic	---
3	67509481	67509481	-	67509481	3	3	---	---	intergenic	---
3	67590217	67590217	+	67590217	5	5	XLOC_055298	+	predicted	---
3	67766469	67766469	+	67766469	3	3	---	---	intergenic	---
3	68096426	68096426	+	68096426	5	5	---	---	intergenic	---
3	68134432	68134449	-	68134433	55	81	ENSSSCG00000022842	-	3'UTR	*RPL7A*
3	68134981	68134981	-	68134981	3	3	ENSSSCG00000022842	-	ORF	---
3	68217144	68217144	+	68217144	4	4	---	---	intergenic	---
3	68333229	68333229	+	68333229	5	5	---	---	intergenic	---
3	68409191	68409192	-	68409192	5	9	---	---	intergenic	---
3	68501468	68501468	-	68501468	3	3	---	---	intergenic	---
3	68570819	68570819	-	68570819	3	3	---	---	intergenic	---
3	68656707	68656707	-	68656707	7	7	---	---	intergenic	---

3	68842663	68842663	-	68842663	3	3	---	---	intergenic	---
3	70228562	70228562	-	70228562	3	3	---	---	intergenic	---
3	71103598	71103605	+	71103604	19	65	ENSSSCG00000021007	+	3'UTR	*MRPL19*
3	71103674	71103674	+	71103674	6	6	ENSSSCG00000021007	+	3'UTR	*MRPL19*
3	71151006	71151006	+	71151006	3	3	XLOC_055313	+	predicted	---
3	71280588	71280597	-	71280596	47	72	XLOC_056560	-	predicted	---
3	71329949	71329951	-	71329949	4	7	XLOC_056561	-	predicted	---
3	71666761	71666761	-	71666761	11	11	ENSSSCG00000008265	-	3'UTR	*DOK1*
3	71667089	71667089	-	71667089	4	4	ENSSSCG00000008265	-	3'UTR	*DOK1*
3	71688452	71688452	-	71688452	4	4	ENSSSCG00000008267	-	3'UTR	---
3	71751229	71751238	-	71751233	68	128	ENSSSCG00000008274	-	3'UTR	*DCTN1*
3	71759822	71759822	-	71759822	4	4	ENSSSCG00000008274	-	intron	*DCTN1*
3	71792137	71792158	-	71792139	102	195	---	---	intergenic	---
3	71799670	71799675	-	71799670	26	41	ENSSSCG00000023156	-	3'UTR	---
3	71809067	71809074	+	71809071	83	132	ENSSSCG00000008282	+	3'UTR	---
							ENSSSCG00000008268	+	intron	*AUP1*
3	71809459	71809466	-	71809466	8	14	ENSSSCG00000008281	-	3'UTR	---
3	71837268	71837273	+	71837273	7	18	ENSSSCG00000008268	+	intron	*AUP1*
3	71840154	71840161	+	71840154	14	28	ENSSSCG00000008273	+	3'UTR	---
							ENSSSCG00000008268	+	intron	*AUP1*
3	71840319	71840331	-	71840323	19	47	XLOC_056570	-	predicted	---
3	71848173	71848173	-	71848173	3	3	ENSSSCG00000008270	-	3'UTR	---
3	71848726	71848737	-	71848734	33	125	ENSSSCG00000008270	-	3'UTR	---
3	71849257	71849257	-	71849257	10	10	ENSSSCG00000008270	-	3'UTR	---
3	71850607	71850607	+	71850607	3	3	ENSSSCG00000008268	+	ORF	*AUP1*
3	71850882	71850889	+	71850886	163	237	ENSSSCG00000008268	+	3'UTR	*AUP1*

3	71880252	71880253	-	71880253	8	12	ENSSSCG00000008275	-	3'UTR	*TTC31*
3	71977395	71977395	-	71977395	6	6	XLOC_056577	-	predicted	---
3	71977514	71977515	+	71977515	9	12	XLOC_055329	+	predicted	---
3	71978932	71978932	-	71978932	3	3	XLOC_056577	-	predicted	---
3	71984941	71984941	+	71984941	3	3	ENSSSCG00000008290	+	ORF	---
3	71994143	71994143	+	71994143	3	3	XLOC_055330	+	predicted	---
3	71995486	71995486	+	71995486	3	3	XLOC_055330	+	predicted	---
3	71996331	71996332	+	71996331	6	12	XLOC_055330	+	predicted	---
3	72008416	72008416	+	72008416	5	5	ENSSSCG00000008291	+	intron	*BOLA3*
3	72015254	72015257	+	72015256	14	25	XLOC_055331	+	predicted	---
3	72015348	72015388	+	72015348	8	22	XLOC_055331	+	predicted	---
3	72074724	72074725	-	72074725	4	7	XLOC_056581	-	predicted	---
3	72209551	72209560	-	72209559	14	32	ENSSSCG00000008293	-	3'UTR	*DGUOK*
3	72212463	72212463	-	72212463	8	8	ENSSSCG00000008293	-	intron	*DGUOK*
3	72251873	72251880	-	72251873	16	32	ENSSSCG00000008294	-	3'UTR	*ACTG2*
3	72359734	72359734	-	72359734	3	3	ENSSSCG00000008300	-	3'UTR	*STAMBP*
3	72394692	72394692	-	72394692	3	3	XLOC_056585	-	predicted	---
3	72418163	72418163	+	72418163	3	3	ENSSSCG00000008298	+	intron	*DUSP11*
3	72427553	72427569	+	72427557	25	35	XLOC_055335	+	predicted	---
3	72438000	72438000	+	72438000	3	3	---	---	intergenic	---
3	72445181	72445201	+	72445198	17	52	ENSSSCG00000008297	+	3'UTR	*TPRKB*
3	72453589	72453596	+	72453596	16	53	XLOC_055337	+	predicted	---
3	72453690	72453690	+	72453690	9	9	XLOC_055337	+	predicted	---
3	72454989	72454989	+	72454989	3	3	XLOC_055337	+	predicted	---
3	72454992	72454993	-	72454993	6	10	ENSSSCG00000021579	-	3'UTR	*ALMS1*
3	72455193	72455200	-	72455194	37	81	ENSSSCG00000021579	-	3'UTR	*ALMS1*

3	72491147	72491157	-	72491151	10	34	ENSSSCG00000021579	-	intron	*ALMS1*
3	72559493	72559493	-	72559493	3	3	XLOC_056587	-	predicted	---
3	72656549	72656552	-	72656549	3	6	---	---	intergenic	---
3	72730396	72730396	-	72730396	3	3	XLOC_056588	-	predicted	---
3	72730503	72730529	-	72730520	123	525	ENSSSCG00000008303	-	3'UTR	*CCT7*
3	72755295	72755323	+	72755298	34	117	ENSSSCG00000008304	+	3'UTR	*PRADC1*
3	72756042	72756048	-	72756048	5	8	ENSSSCG00000008305	-	3'UTR	*SMYD5*
3	72902102	72902104	+	72902102	3	6	ENSSSCG00000022162	+	3'UTR	---
3	73035658	73035666	+	73035658	4	7	XLOC_055342	+	predicted	---
3	73090290	73090319	-	73090292	54	199	XLOC_056596	-	predicted	---
3	73265350	73265350	-	73265350	4	4	---	---	intergenic	---
3	73444907	73444907	+	73444907	3	3	---	---	intergenic	---
3	73934213	73934213	+	73934213	7	7	XLOC_055348	+	predicted	---
3	73992020	73992027	-	73992027	7	10	XLOC_056601	-	predicted	---
3	74406199	74406200	+	74406200	16	24	ENSSSCG00000008313	+	3'UTR	---
3	74422610	74422610	-	74422610	4	4	XLOC_056604	-	predicted	---
3	74679477	74679477	+	74679477	3	3	---	---	intergenic	---
3	74712145	74712145	-	74712145	14	14	XLOC_056606	-	predicted	---
3	74740865	74740865	-	74740865	3	3	XLOC_056606	-	predicted	---
3	74938472	74938472	+	74938472	4	4	ENSSSCG00000008315	+	intron	*PAIP2B*
3	74945600	74945610	+	74945610	5	11	XLOC_055352	+	predicted	---
3	74946443	74946443	-	74946443	3	3	XLOC_056607	-	predicted	---
3	74949395	74949402	-	74949400	24	70	ENSSSCG00000008316	-	3'UTR	*NAGK*
3	74960926	74960926	-	74960926	4	4	XLOC_056607	-	predicted	---
3	74998159	74998180	+	74998180	19	50	XLOC_055353	+	predicted	---
3	75000179	75000184	+	75000179	17	34	XLOC_055353	+	predicted	---

3	75000803	75000803	-	75000803	4	4	XLOC_056609	-	predicted	---
3	75273077	75273077	+	75273077	3	3	---	---	intergenic	---
3	75400417	75400419	+	75400417	8	13	---	---	intergenic	---
3	75439493	75439493	-	75439493	4	4	---	---	intergenic	---
3	75440185	75440187	-	75440185	6	9	XLOC_056614	-	predicted	---
3	75441383	75441384	+	75441383	4	7	XLOC_055358	+	predicted	---
3	75466623	75466626	+	75466623	7	10	ENSSSCG00000026446	+	3'UTR	*FAM136A*
3	75467829	75467835	+	75467835	7	10	ENSSSCG00000026446	+	3'UTR	*FAM136A*
3	75474775	75474775	+	75474775	3	3	ENSSSCG00000024776	+	intron	*SNRPG*
3	75477672	75477677	+	75477677	18	36	ENSSSCG00000024776	+	3'UTR	*SNRPG*
3	75477802	75477803	+	75477803	7	11	ENSSSCG00000024776	+	3'UTR	*SNRPG*
3	75478451	75478458	-	75478453	35	48	ENSSSCG00000029790	-	3'UTR	*PCYOX1*
3	75479634	75479634	-	75479634	5	5	ENSSSCG00000029790	-	3'UTR	*PCYOX1*
3	75479703	75479704	-	75479703	10	14	ENSSSCG00000029790	-	3'UTR	*PCYOX1*
3	75480557	75480557	-	75480557	5	5	ENSSSCG00000029790	-	ORF	*PCYOX1*
3	75499866	75499866	+	75499866	12	12	---	---	intergenic	---
3	75704297	75704298	-	75704297	11	14	XLOC_056618	-	predicted	---
3	75712208	75712208	-	75712208	6	6	ENSSSCG00000023409	-	3'UTR	*TIA1*
3	75713939	75713939	-	75713939	3	3	ENSSSCG00000023409	-	3'UTR	*TIA1*
3	75723328	75723330	-	75723329	20	35	---	---	intergenic	---
3	75732869	75732870	+	75732870	8	13	XLOC_055363	+	predicted	---
3	75762587	75762587	+	75762587	9	9	ENSSSCG00000008332	+	3'UTR	*C2orf42*
3	75762896	75762897	+	75762896	13	20	ENSSSCG00000008332	+	3'UTR	*C2orf42*
3	75763649	75763649	+	75763649	3	3	XLOC_055364	+	predicted	---
3	75813905	75813986	-	75813948	100	290	---	---	intergenic	---
3	75814496	75814512	-	75814496	3	6	ENSSSCG00000027224	-	ORF	*PCBP1*

3	75852988	75852988	+	75852988	6	6	XLOC_055365	+	predicted	---
3	75912357	75912357	-	75912357	10	10	XLOC_056621	-	predicted	---
3	75914570	75914570	-	75914570	7	7	XLOC_056621	-	predicted	---
3	75950669	75950676	-	75950669	8	25	XLOC_056621	-	predicted	---
3	75965024	75965038	-	75965038	5	13	XLOC_056622	-	predicted	---
3	75965991	75965991	-	75965991	4	4	XLOC_056622	-	predicted	---
3	75966774	75966774	-	75966774	3	3	XLOC_056622	-	predicted	---
3	75966813	75966828	-	75966813	6	9	XLOC_056622	-	predicted	---
3	76019247	76019248	-	76019247	8	11	ENSSSCG00000008335	-	intron	---
3	76056863	76056863	-	76056863	7	7	ENSSSCG00000008335	-	ORF	---
3	76060041	76060042	-	76060042	12	16	ENSSSCG00000008336	-	3'UTR	*ANXA4*
3	76060806	76060812	-	76060808	47	66	ENSSSCG00000008336	-	3'UTR	*ANXA4*
3	76060939	76060945	-	76060939	3	6	ENSSSCG00000008336	-	3'UTR	*ANXA4*
3	76146139	76146139	+	76146139	3	3	---	---	intergenic	---
3	76173812	76173812	+	76173812	3	3	---	---	intergenic	---
3	76229281	76229281	-	76229281	3	3	---	---	intergenic	---
3	76338082	76338082	+	76338082	4	4	ENSSSCG00000008337	+	intron	*AAK1*
3	76365826	76365826	+	76365826	7	7	ENSSSCG00000008337	+	intron	*AAK1*
3	76403240	76403240	+	76403240	3	3	XLOC_055367	+	predicted	---
3	76444225	76444255	+	76444230	23	72	XLOC_055369	+	predicted	---
3	76454141	76454149	-	76454142	9	18	XLOC_056629	-	predicted	---
3	76456216	76456216	+	76456216	4	4	XLOC_055369	+	predicted	---
3	76506218	76506218	+	76506218	4	4	---	---	intergenic	---
3	76509214	76509222	+	76509222	29	39	ENSSSCG00000008339	+	3'UTR	*GFAT1*
3	76509566	76509566	+	76509566	3	3	ENSSSCG00000008339	+	3'UTR	*GFAT1*
3	76510035	76510035	+	76510035	3	3	XLOC_055370	+	predicted	---

3	76513242	76513242	+	76513242	7	7	XLOC_055370	+	predicted	---
3	76513284	76513284	+	76513284	4	4	XLOC_055370	+	predicted	---
3	76514410	76514412	+	76514412	19	25	XLOC_055370	+	predicted	---
3	76514530	76514530	+	76514530	4	4	XLOC_055370	+	predicted	---
3	76515275	76515275	+	76515275	9	9	XLOC_055370	+	predicted	---
3	76586489	76586489	-	76586489	6	6	---	---	intergenic	---
3	76676886	76676886	-	76676886	3	3	ENSSSCG00000008340	-	intron	*ANTXR1*
3	77289330	77289330	-	77289330	3	3	ENSSSCG00000008346	-	3'UTR	*APLF*
3	77408772	77408777	-	77408777	14	24	ENSSSCG00000008348	-	3'UTR	*PLEK*
3	77600077	77600082	+	77600082	30	34	ENSSSCG00000008353	+	intron	---
3	77603531	77603538	-	77603537	10	18	ENSSSCG00000008352	-	3'UTR	*PNO1*
3	77640822	77640830	+	77640822	9	14	ENSSSCG00000008353	+	3'UTR	---
3	77678669	77678669	+	77678669	3	3	ENSSSCG00000008354	+	intron	*C1D*
3	77684501	77684501	+	77684501	4	4	XLOC_055384	+	predicted	---
3	77684944	77684945	+	77684944	6	9	XLOC_055384	+	predicted	---
3	77887536	77887536	-	77887536	4	4	---	---	intergenic	---
3	77945168	77945168	+	77945168	5	5	---	---	intergenic	---
3	78113509	78113509	-	78113509	3	3	---	---	intergenic	---
3	78385503	78385503	+	78385503	3	3	ENSSSCG00000023908	+	intron	*ETAA1*
3	78540982	78540982	-	78540982	4	4	---	---	intergenic	---
3	78844305	78844306	+	78844305	7	12	---	---	intergenic	---
3	79255296	79255297	-	79255297	7	10	XLOC_056650	-	predicted	---
3	80065282	80065283	-	80065283	4	7	---	---	intergenic	---
3	80104602	80104602	-	80104602	3	3	XLOC_056652	-	predicted	---
3	80149924	80149924	+	80149924	6	6	---	---	intergenic	---
3	80339273	80339274	+	80339273	5	8	XLOC_055393	+	predicted	---

3	80487355	80487355	-	80487355	33	33	ENSSSCG00000008358	-	3'UTR	*ACTR2*
3	80487503	80487503	-	80487503	3	3	ENSSSCG00000008358	-	3'UTR	*ACTR2*
3	80489645	80489645	-	80489645	3	3	ENSSSCG00000008358	-	3'UTR	*ACTR2*
3	80489792	80489801	-	80489801	9	12	ENSSSCG00000008358	-	3'UTR	*ACTR2*
3	80525703	80525703	+	80525703	4	4	---	---	intergenic	---
3	80664283	80664283	-	80664283	3	3	---	---	intergenic	---
3	80706167	80706168	+	80706168	13	18	ENSSSCG00000008359	+	3'UTR	*RAB1B*
3	80706533	80706545	+	80706534	26	54	ENSSSCG00000008359	+	3'UTR	*RAB1B*
3	80706797	80706797	+	80706797	4	4	XLOC_055396	+	predicted	---
3	80746112	80746112	-	80746112	4	4	---	---	intergenic	---
3	80748023	80748023	-	80748023	4	4	ENSSSCG00000027527	-	3'UTR	---
3	80748641	80748641	-	80748641	4	4	ENSSSCG00000027527	-	3'UTR	---
3	80809557	80809557	-	80809557	3	3	---	---	intergenic	---
3	80931831	80931832	-	80931831	3	6	---	---	intergenic	---
3	81032810	81032817	+	81032810	3	9	XLOC_055401	+	predicted	---
3	81155706	81155706	+	81155706	4	4	XLOC_055401	+	predicted	---
3	81192698	81192698	-	81192698	3	3	ENSSSCG00000008363	-	3'UTR	*AFTPH*
3	81258923	81258923	+	81258923	6	6	---	---	intergenic	---
3	81264604	81264612	+	81264610	47	76	XLOC_055403	+	predicted	---
3	81337706	81337706	-	81337706	3	3	---	---	intergenic	---
3	81357505	81357505	+	81357505	3	3	---	---	intergenic	---
3	81433370	81433370	-	81433370	3	3	---	---	intergenic	---
3	81506459	81506459	+	81506459	3	3	---	---	intergenic	---
3	81523797	81523797	-	81523797	3	3	---	---	intergenic	---
3	81750467	81750467	+	81750467	3	3	ENSSSCG00000027509	+	ORF	---
3	82173298	82173298	-	82173298	3	3	---	---	intergenic	---

3	82182547	82182549	-	82182548	13	26	---	---	intergenic	---
3	82549932	82549932	-	82549932	4	4	XLOC_056670	-	predicted	---
3	82792999	82792999	+	82792999	3	3	XLOC_055413	+	predicted	---
3	82972986	82972986	-	82972986	6	6	XLOC_056673	-	predicted	---
3	82973796	82973796	-	82973796	10	10	ENSSSCG00000008372	-	3'UTR	*EHBP1*
3	82982210	82982210	-	82982210	3	3	ENSSSCG00000008372	-	intron	*EHBP1*
3	83313179	83313179	+	83313179	6	6	---	---	intergenic	---
3	83438582	83438583	+	83438583	57	60	---	---	intergenic	---
3	83605345	83605354	-	83605347	114	188	XLOC_056676	-	predicted	---
3	83756567	83756567	-	83756567	21	21	---	---	intergenic	---
3	83777096	83777112	+	83777100	82	148	ENSSSCG00000008377	+	3'UTR	*CCT4*
3	83777449	83777475	+	83777462	9	62	ENSSSCG00000008377	+	3'UTR	*CCT4*
3	83788543	83788543	+	83788543	3	3	ENSSSCG00000008378	+	ORF	*FAM161A*
3	83801552	83801552	+	83801552	3	3	ENSSSCG00000008378	+	ORF	*FAM161A*
3	83802440	83802451	+	83802450	6	15	ENSSSCG00000008378	+	intron	*FAM161A*
3	83805484	83805485	+	83805484	8	13	ENSSSCG00000008378	+	intron	*FAM161A*
3	83812728	83812728	+	83812728	5	5	ENSSSCG00000008378	+	intron	*FAM161A*
3	83814249	83814249	+	83814249	3	3	ENSSSCG00000008378	+	3'UTR	*FAM161A*
3	83814325	83814331	+	83814330	19	35	ENSSSCG00000008378	+	3'UTR	*FAM161A*
3	83827763	83827763	-	83827763	3	3	XLOC_056678	-	predicted	---
3	83828434	83828436	-	83828435	6	12	XLOC_056678	-	predicted	---
3	83828774	83828778	-	83828778	5	12	XLOC_056678	-	predicted	---
3	83853019	83853032	-	83853031	37	124	---	---	intergenic	---
3	83853070	83853073	-	83853073	7	14	---	---	intergenic	---
3	83889581	83889581	-	83889581	4	4	---	---	intergenic	---
3	83895905	83895905	-	83895905	3	3	---	---	intergenic	---

3	83933414	83933414	+	83933414	3	3	---	---	intergenic	---
3	84002184	84002184	+	84002184	4	4	---	---	intergenic	---
3	84039705	84039705	-	84039705	4	4	---	---	intergenic	---
3	84045249	84045249	-	84045249	4	4	---	---	intergenic	---
3	84139241	84139241	-	84139241	6	6	ENSSSCG00000028100	-	ORF	---
3	84176430	84176430	+	84176430	7	7	ENSSSCG00000028228	+	intron	*XPO1*
3	84181358	84181371	+	84181359	6	12	ENSSSCG00000028228	+	3'UTR	*XPO1*
3	84182139	84182140	+	84182139	25	30	ENSSSCG00000028228	+	3'UTR	*XPO1*
3	84222240	84222240	-	84222240	3	3	ENSSSCG00000028100	-	intron	---
3	84267402	84267403	-	84267402	10	13	ENSSSCG00000028100	-	intron	---
3	84283738	84283738	+	84283738	3	3	ENSSSCG00000008382	+	intron	*USP34*
3	84515559	84515559	+	84515559	3	3	---	---	intergenic	---
3	84515773	84515782	+	84515782	9	15	---	---	intergenic	---
3	84517847	84517849	+	84517847	20	28	ENSSSCG00000008384	+	3'UTR	*C2orf74*
3	84517943	84517947	+	84517945	68	145	ENSSSCG00000008384	+	3'UTR	*C2orf74*
3	84541700	84541700	+	84541700	3	3	ENSSSCG00000008383	+	intron	*AHSA2*
3	84555489	84555504	+	84555490	10	23	ENSSSCG00000008383	+	intron	*AHSA2*
3	84574398	84574398	+	84574398	6	6	XLOC_055423	+	predicted	---
3	84590539	84590539	-	84590539	6	6	---	---	intergenic	---
3	84852672	84852674	-	84852674	4	7	XLOC_056686	-	predicted	---
3	84852992	84852996	-	84852992	4	7	XLOC_056686	-	predicted	---
3	84953235	84953235	+	84953235	6	6	XLOC_055424	+	predicted	---
3	84963805	84963805	-	84963805	4	4	XLOC_056687	-	predicted	---
3	85078002	85078002	-	85078002	5	5	XLOC_056688	-	predicted	---
3	85081490	85081491	-	85081490	5	8	XLOC_056688	-	predicted	---
3	85161438	85161445	-	85161438	5	12	XLOC_056689	-	predicted	---

3	85208581	85208581	-	85208581	6	6	XLOC_056689	-	predicted	---
3	85722808	85722808	-	85722808	4	4	---	---	intergenic	---
3	86770784	86770784	+	86770784	5	5	XLOC_055433	+	predicted	---
3	87581857	87581857	+	87581857	3	3	---	---	intergenic	---
3	87860507	87860507	+	87860507	3	3	---	---	intergenic	---
3	88018618	88018618	-	88018618	4	4	---	---	intergenic	---
3	88020679	88020679	-	88020679	6	6	ENSSSCG00000008395	-	3'UTR	*VRK2*
3	88021144	88021144	+	88021144	6	6	XLOC_055437	+	predicted	---
3	88022383	88022383	+	88022383	3	3	XLOC_055437	+	predicted	---
3	88477160	88477160	-	88477160	3	3	---	---	intergenic	---
3	88848934	88848934	-	88848934	3	3	---	---	intergenic	---
3	89266012	89266013	+	89266012	5	8	---	---	intergenic	---
3	89402814	89402814	+	89402814	3	3	---	---	intergenic	---
3	89968091	89968091	-	89968091	3	3	---	---	intergenic	---
3	90084076	90084076	-	90084076	3	3	---	---	intergenic	---
3	90160450	90160450	+	90160450	3	3	ENSSSCG00000008397	+	intron	*EFEMP1*
3	90334903	90334903	+	90334903	8	8	ENSSSCG00000025233	+	ORF	---
3	90370828	90370833	-	90370828	3	6	ENSSSCG00000022880	-	ORF	---
3	90425355	90425355	+	90425355	3	3	XLOC_055448	+	predicted	---
3	90532218	90532249	+	90532249	11	14	ENSSSCG00000008400	+	ORF	*CFAP36*
3	90542782	90542782	+	90542782	9	9	ENSSSCG00000008400	+	intron	*CFAP36*
3	90543446	90543455	+	90543448	56	91	ENSSSCG00000008400	+	3'UTR	*CFAP36*
3	90544705	90544705	-	90544705	4	4	ENSSSCG00000008398	-	3'UTR	*SMEK2*
3	90545595	90545600	-	90545597	52	101	ENSSSCG00000008398	-	3'UTR	*SMEK2*
3	90545950	90545950	-	90545950	6	6	ENSSSCG00000008398	-	3'UTR	*SMEK2*
3	90546197	90546197	-	90546197	3	3	ENSSSCG00000008398	-	3'UTR	*SMEK2*

3	90555371	90555386	-	90555371	9	19	ENSSSCG00000008398	-	ORF	*SMEK2*
3	90592228	90592228	-	90592228	4	4	ENSSSCG00000008398	-	ORF	*SMEK2*
3	90612383	90612383	+	90612383	3	3	---	---	intergenic	---
3	90615735	90615735	-	90615735	3	3	---	---	intergenic	---
3	90660784	90660784	+	90660784	3	3	ENSSSCG00000008406	+	intron	*CCDC88A*
3	90663327	90663333	+	90663333	14	21	ENSSSCG00000008406	+	intron	*CCDC88A*
3	90674725	90674725	+	90674725	7	7	---	---	intergenic	---
3	90681601	90681610	+	90681601	4	10	---	---	intergenic	---
3	90692273	90692277	+	90692273	3	6	---	---	intergenic	---
3	90701393	90701393	+	90701393	3	3	ENSSSCG00000008406	+	intron	*CCDC88A*
3	90709547	90709547	+	90709547	4	4	ENSSSCG00000008406	+	intron	*CCDC88A*
3	90722848	90722849	+	90722848	29	33	ENSSSCG00000008406	+	3'UTR	*CCDC88A*
3	90723574	90723575	+	90723574	4	7	ENSSSCG00000008406	+	3'UTR	*CCDC88A*
3	90725510	90725510	+	90725510	3	3	ENSSSCG00000008406	+	3'UTR	*CCDC88A*
3	90729092	90729092	-	90729092	3	3	ENSSSCG00000029514	-	3'UTR	---
3	90729139	90729146	-	90729146	8	21	ENSSSCG00000029514	-	3'UTR	---
3	90748332	90748332	+	90748332	6	6	ENSSSCG00000008404	+	intron	*MTIF2*
3	90753991	90753991	+	90753991	8	8	ENSSSCG00000008404	+	intron	*MTIF2*
3	90773762	90773770	-	90773763	11	23	XLOC_056713	-	predicted	---
3	90773810	90773819	-	90773812	143	196	XLOC_056713	-	predicted	---
3	90773921	90773921	-	90773921	3	3	ENSSSCG00000022357	-	ORF	*RPS27A*
3	90776200	90776202	-	90776200	3	6	ENSSSCG00000022357	-	intron	*RPS27A*
3	90831107	90831108	+	90831107	9	12	XLOC_055452	+	predicted	---
3	91190296	91190296	-	91190296	3	3	ENSSSCG00000026559	-	intron	*EML6*
3	91491910	91491910	-	91491910	3	3	---	---	intergenic	---
3	91491971	91491971	+	91491971	3	3	---	---	intergenic	---

3	91492101	91492101	+	91492101	3	3	---	---	intergenic	---
3	91522195	91522195	-	91522195	4	4	ENSSSCG00000024602	-	intron	---
3	91633802	91633802	+	91633802	3	3	---	---	intergenic	---
3	91646973	91646973	-	91646973	4	4	---	---	intergenic	---
3	91670110	91670118	-	91670111	36	69	XLOC_056717	-	predicted	---
3	91670298	91670298	-	91670298	4	4	ENSSSCG00000008410	-	ORF	*C2orf73*
3	91712470	91712470	+	91712470	4	4	---	---	intergenic	---
3	91728775	91728778	-	91728778	17	22	XLOC_056718	-	predicted	---
3	91990573	91990573	+	91990573	3	3	ENSSSCG00000030197	+	3'UTR	---
3	92132152	92132153	-	92132152	10	18	---	---	intergenic	---
3	92204802	92204802	-	92204802	8	8	ENSSSCG00000026974	-	intron	---
3	92219097	92219097	+	92219097	6	6	---	---	intergenic	---
3	92299680	92299680	+	92299680	3	3	---	---	intergenic	---
3	92489019	92489020	+	92489019	16	21	ENSSSCG00000008412	+	3'UTR	*PSME4*
3	92521491	92521509	-	92521509	19	59	ENSSSCG00000008414	-	3'UTR	*ERLEC1*
3	92522320	92522320	-	92522320	5	5	ENSSSCG00000008414	-	3'UTR	*ERLEC1*
3	92539061	92539062	-	92539061	19	24	ENSSSCG00000008414	-	ORF	*ERLEC1*
3	92541635	92541635	-	92541635	3	3	ENSSSCG00000008414	-	intron	*ERLEC1*
							ENSSSCG00000008414	-	ORF	*ERLEC1*
3	92656077	92656077	+	92656077	6	6	ENSSSCG00000008415	+	3'UTR	---
3	92657489	92657489	+	92657489	3	3	XLOC_055464	+	predicted	---
3	92788221	92788228	-	92788222	14	24	---	---	intergenic	---
3	93009853	93009853	+	93009853	3	3	---	---	intergenic	---
3	93967160	93967161	+	93967161	12	19	---	---	intergenic	---
3	93995861	93995861	-	93995861	3	3	---	---	intergenic	---
3	93995892	93995892	+	93995892	3	3	---	---	intergenic	---

3	94021103	94021103	+	94021103	3	3	---	---	intergenic	---
3	95028260	95028260	-	95028260	7	7	---	---	intergenic	---
3	95226032	95226032	+	95226032	10	10	---	---	intergenic	---
3	95772071	95772071	-	95772071	12	12	XLOC_056727	-	predicted	---
3	95776031	95776035	-	95776035	11	18	XLOC_056727	-	predicted	---
3	96053292	96053309	+	96053292	7	20	---	---	intergenic	---
3	96231308	96231308	+	96231308	4	4	---	---	intergenic	---
3	96266312	96266313	+	96266313	7	12	---	---	intergenic	---
3	96391833	96391833	-	96391833	3	3	---	---	intergenic	---
3	96661601	96661603	+	96661601	3	6	ENSSSCG00000026180	+	3'UTR	---
3	96697248	96697248	-	96697248	3	3	---	---	intergenic	---
3	96697272	96697273	+	96697272	8	13	---	---	intergenic	---
3	96741418	96741418	-	96741418	4	4	---	---	intergenic	---
3	97497931	97497932	-	97497931	16	19	---	---	intergenic	---
3	97497961	97497961	+	97497961	5	5	---	---	intergenic	---
3	97644132	97644132	-	97644132	5	5	ENSSSCG00000022243	-	ORF	---
3	97851443	97851443	+	97851443	5	5	ENSSSCG00000008422	+	3'UTR	---
3	97865983	97865983	-	97865983	12	12	XLOC_056734	-	predicted	---
3	97952594	97952607	+	97952606	20	60	---	---	intergenic	---
3	97965694	97965694	-	97965694	9	9	ENSSSCG00000008423	-	3'UTR	*PPP1R21*
3	97965898	97965898	-	97965898	3	3	ENSSSCG00000008423	-	3'UTR	*PPP1R21*
3	98001115	98001115	-	98001115	3	3	ENSSSCG00000008423	-	intron	*PPP1R21*
3	98147143	98147143	+	98147143	29	29	ENSSSCG00000029593	+	3'UTR	*FOXN2*
3	98462936	98462936	-	98462936	3	3	---	---	intergenic	---
3	98506407	98506407	+	98506407	3	3	---	---	intergenic	---
3	98674067	98674067	+	98674067	3	3	ENSSSCG00000029485	+	intron	*FBXO11*

3	98708607	98708609	+	98708609	9	13	ENSSSCG00000029485	+	ORF	*FBXO11*
3	98710234	98710235	+	98710234	12	16	XLOC_055478	+	predicted	---
3	99312706	99312722	-	99312722	15	22	ENSSSCG00000008429	-	3'UTR	*TACSTD1*
3	99496835	99496842	+	99496840	6	16	---	---	intergenic	---
3	99516457	99516471	-	99516465	76	252	---	---	intergenic	---
3	99516527	99516527	-	99516527	3	3	ENSSSCG00000008432	-	3'UTR	*C2orf61*
3	99516655	99516658	-	99516657	39	62	ENSSSCG00000008432	-	3'UTR	*C2orf61*
3	99519717	99519717	-	99519717	5	5	ENSSSCG00000008432	-	intron	*C2orf61*
3	99520595	99520595	-	99520595	4	4	ENSSSCG00000008432	-	intron	*C2orf61*
3	99567547	99567555	-	99567554	130	205	ENSSSCG00000008433	-	3'UTR	*CALM1*
3	99568055	99568064	-	99568055	8	14	ENSSSCG00000008433	-	3'UTR	*CALM1*
3	99569261	99569289	-	99569265	113	225	ENSSSCG00000008433	-	ORF	*CALM1*
3	99569783	99569783	-	99569783	4	4	ENSSSCG00000008433	-	ORF	*CALM1*
3	99596006	99596006	-	99596006	3	3	---	---	intergenic	---
3	99653450	99653458	-	99653452	6	24	XLOC_056749	-	predicted	---
3	99719246	99719246	+	99719246	3	3	ENSSSCG00000021325	+	3'UTR	*MCFD2*
3	99719370	99719380	+	99719378	63	125	ENSSSCG00000021325	+	3'UTR	*MCFD2*
3	99870763	99870763	-	99870763	5	5	XLOC_056754	-	predicted	---
3	99999281	99999281	-	99999281	3	3	XLOC_056756	-	predicted	---
3	100002162	100002162	-	100002162	12	12	XLOC_056756	-	predicted	---
3	100054940	100054947	-	100054947	5	9	XLOC_056757	-	predicted	---
3	100117303	100117303	+	100117303	3	3	ENSSSCG00000008441	+	ORF	*ATP6V1E2*
3	100118008	100118012	+	100118010	29	58	XLOC_055496	+	predicted	---
3	100118241	100118241	+	100118241	3	3	XLOC_055496	+	predicted	---
3	100146886	100146887	-	100146887	10	17	ENSSSCG00000008442	-	ORF	*TMEM247*
3	100149818	100149818	-	100149818	3	3	ENSSSCG00000008442	-	intron	*TMEM247*

3	100211420	100211421	+	100211421	8	12	ENSSSCG00000008443	+	3'UTR	*EPAS1*
3	100213295	100213298	+	100213296	52	100	ENSSSCG00000008443	+	3'UTR	*EPAS1*
3	100225946	100225949	-	100225946	3	9	---	---	intergenic	---
3	100785360	100785361	+	100785360	8	12	---	---	intergenic	---
3	100906531	100906531	+	100906531	3	3	---	---	intergenic	---
3	100961031	100961031	-	100961031	6	6	---	---	intergenic	---
3	100975400	100975400	-	100975400	12	12	XLOC_056765	-	predicted	---
3	100975627	100975627	-	100975627	3	3	XLOC_056765	-	predicted	---
3	100975751	100975751	-	100975751	3	3	XLOC_056765	-	predicted	---
3	100981688	100981688	+	100981688	3	3	---	---	intergenic	---
3	101097728	101097728	+	101097728	3	3	ENSSSCG00000008445	+	intron	*SRBD1*
3	101295795	101295795	+	101295795	6	6	XLOC_055504	+	predicted	---
3	101420366	101420366	+	101420366	5	5	XLOC_055504	+	predicted	---
3	101489718	101489718	+	101489718	3	3	---	---	intergenic	---
3	101737806	101737806	+	101737806	7	7	---	---	intergenic	---
3	101778046	101778046	-	101778046	4	4	XLOC_056768	-	predicted	---
3	101875845	101875845	+	101875845	8	8	---	---	intergenic	---
3	101931181	101931181	-	101931181	10	10	---	---	intergenic	---
3	102261396	102261397	+	102261397	18	21	ENSSSCG00000008448	+	intron	*PREPL*
3	102312690	102312690	-	102312690	4	4	XLOC_056770	-	predicted	---
3	102314755	102314755	+	102314755	3	3	ENSSSCG00000008448	+	3'UTR	*PREPL*
3	102374521	102374521	+	102374521	14	14	---	---	intergenic	---
3	102389508	102389517	-	102389517	14	17	XLOC_056771	-	predicted	---
3	102392142	102392142	-	102392142	3	3	XLOC_056771	-	predicted	---
3	102497133	102497133	+	102497133	11	11	---	---	intergenic	---
3	102537794	102537794	-	102537794	3	3	---	---	intergenic	---

3	102742808	102742808	+	102742808	23	23	XLOC_055510	+	predicted	---
3	102744873	102744873	+	102744873	7	7	XLOC_055510	+	predicted	---
3	102752063	102752069	-	102752063	4	8	XLOC_056772	-	predicted	---
3	102769288	102769289	+	102769288	3	6	---	---	intergenic	---
3	103107104	103107104	+	103107104	6	6	ENSSSCG00000028599	+	intron	---
3	103255695	103255695	+	103255695	8	8	XLOC_055514	+	predicted	---
3	103681172	103681172	-	103681172	8	8	---	---	intergenic	---
3	103872742	103872745	-	103872744	14	20	ENSSSCG00000027800	-	3'UTR	---
3	103904305	103904308	+	103904308	4	7	---	---	intergenic	---
3	103960919	103960919	-	103960919	3	3	---	---	intergenic	---
3	104009015	104009015	+	104009015	3	3	ENSSSCG00000008465	+	intron	*KCNG3*
3	104141154	104141157	-	104141156	18	28	XLOC_056786	-	predicted	---
3	104209516	104209516	-	104209516	4	4	ENSSSCG00000008467	-	ORF	*EML4*
3	104213050	104213051	-	104213050	4	7	ENSSSCG00000008467	-	intron	*EML4*
3	104309207	104309207	-	104309207	8	8	---	---	intergenic	---
3	104316183	104316183	+	104316183	4	4	---	---	intergenic	---
3	104399334	104399334	-	104399334	3	3	ENSSSCG00000008468	-	3'UTR	*PKDCC*
3	104502880	104502880	-	104502880	4	4	XLOC_056789	-	predicted	---
3	104919537	104919537	-	104919537	3	3	---	---	intergenic	---
3	105445969	105445973	-	105445969	3	6	---	---	intergenic	---
3	105622632	105622632	-	105622632	3	3	---	---	intergenic	---
3	105807793	105807794	-	105807794	6	11	---	---	intergenic	---
3	106200191	106200191	-	106200191	3	3	---	---	intergenic	---
3	106677791	106677791	+	106677791	3	3	ENSSSCG00000024293	+	3'UTR	*TMEM178A*
3	106694190	106694192	-	106694191	11	21	ENSSSCG00000008470	-	3'UTR	*THUMPD2*
3	106713010	106713010	-	106713010	4	4	ENSSSCG00000008470	-	intron	*THUMPD2*

3	106858028	106858028	+	106858028	3	3	---	---	intergenic	---
3	107050196	107050214	-	107050196	5	13	XLOC_056798	-	predicted	---
3	107209215	107209216	+	107209216	116	120	ENSSSCG00000008477	+	intron	*CDKL4*
3	107247044	107247054	+	107247046	32	88	ENSSSCG00000008477	+	intron	*CDKL4*
3	107275936	107275936	-	107275936	3	3	---	---	intergenic	---
3	107409799	107409799	+	107409799	5	5	ENSSSCG00000008478	+	intron	---
3	107466092	107466094	+	107466094	6	9	XLOC_055539	+	predicted	---
3	107519419	107519419	+	107519419	3	3	---	---	intergenic	---
3	107607710	107607734	-	107607734	16	23	XLOC_056801	-	predicted	---
3	107607787	107607811	-	107607791	353	655	ENSSSCG00000008480	-	3'UTR	*MORN2*
3	107609044	107609045	-	107609044	6	9	ENSSSCG00000008480	-	ORF	*MORN2*
3	107640026	107640026	-	107640026	3	3	---	---	intergenic	---
3	107713211	107713218	-	107713211	9	15	ENSSSCG00000008482	-	3'UTR	*GEMIN6*
3	107722734	107722734	-	107722734	5	5	ENSSSCG00000008483	-	intron	---
3	107722803	107722804	-	107722804	12	15	ENSSSCG00000008483	-	intron	---
3	107753388	107753395	+	107753388	11	35	ENSSSCG00000008484	+	3'UTR	*SRSF7*
3	107762131	107762132	-	107762132	45	64	ENSSSCG00000008486	-	3'UTR	*GALM*
3	107763065	107763078	-	107763065	6	38	ENSSSCG00000008486	-	3'UTR	*GALM*
3	107835744	107835744	+	107835744	9	9	ENSSSCG00000008485	+	ORF	---
3	107847250	107847251	-	107847251	11	18	---	---	intergenic	---
3	107955885	107955885	-	107955885	3	3	---	---	intergenic	---
3	108088673	108088673	-	108088673	6	6	---	---	intergenic	---
3	108136734	108136734	+	108136734	3	3	ENSSSCG00000008487	+	intron	*ATL2*
3	108146062	108146064	+	108146064	5	8	ENSSSCG00000008487	+	3'UTR	*ATL2*
3	108187800	108187800	-	108187800	3	3	XLOC_056808	-	predicted	---
3	108361153	108361153	-	108361153	3	3	---	---	intergenic	---

3	108496203	108496203	-	108496203	3	3	ENSSSCG00000008488	-	ORF	---
3	108512194	108512219	-	108512194	4	7	ENSSSCG00000008488	-	intron	---
							ENSSSCG00000008488	-	ORF	---
3	108773870	108773871	+	108773871	9	17	---	---	intergenic	---
3	108902974	108902974	+	108902974	3	3	---	---	intergenic	---
3	109282463	109282463	+	109282463	3	3	XLOC_055559	+	predicted	---
3	109332654	109332656	-	109332655	6	15	ENSSSCG00000008491	-	3'UTR	---
3	109397930	109397934	-	109397931	11	29	ENSSSCG00000008493	-	3'UTR	*CEBPZ*
3	109398190	109398190	-	109398190	3	3	ENSSSCG00000008493	-	ORF	*CEBPZ*
3	109399068	109399068	+	109399068	4	4	ENSSSCG00000020689	+	intron	*CEBPZOS*
3	109400032	109400033	-	109400033	37	46	ENSSSCG00000008493	-	intron	*CEBPZ*
3	109400734	109400734	-	109400734	3	3	ENSSSCG00000008493	-	ORF	*CEBPZ*
3	109404705	109404705	-	109404705	4	4	ENSSSCG00000008493	-	ORF	*CEBPZ*
3	109404938	109404940	+	109404940	6	9	ENSSSCG00000020689	+	3'UTR	*CEBPZOS*
3	109419731	109419731	-	109419731	8	8	ENSSSCG00000008493	-	ORF	*CEBPZ*
3	109442739	109442739	-	109442739	6	6	XLOC_056816	-	predicted	---
3	109445071	109445103	-	109445102	56	114	ENSSSCG00000008495	-	3'UTR	*PRKD3*
3	109445365	109445368	+	109445365	3	6	ENSSSCG00000008494	+	3'UTR	*NDUFAF7*
3	109629071	109629071	+	109629071	3	3	ENSSSCG00000008496	+	intron	*EIF2AK2*
3	109679717	109679717	-	109679717	4	4	ENSSSCG00000008497	-	3'UTR	*GPATCH11*
3	109679792	109679801	-	109679799	12	33	ENSSSCG00000008497	-	3'UTR	*GPATCH11*
3	109886848	109886848	+	109886848	3	3	ENSSSCG00000008499	+	intron	*STRN*
3	109930753	109930753	+	109930753	4	4	ENSSSCG00000008499	+	3'UTR	*STRN*
3	109944482	109944482	+	109944482	3	3	XLOC_055564	+	predicted	---
3	110222284	110222284	+	110222284	4	4	ENSSSCG00000008503	+	intron	*FEZ2*
3	110257182	110257182	+	110257182	5	5	ENSSSCG00000008503	+	3'UTR	*FEZ2*

3	110257605	110257605	-	110257605	3	3	ENSSSCG00000008504	-	3'UTR	*CRIM1*
3	110726590	110726590	-	110726590	3	3	---	---	intergenic	---
3	110735492	110735492	+	110735492	4	4	---	---	intergenic	---
3	111319617	111319617	-	111319617	3	3	---	---	intergenic	---
3	111928996	111928997	+	111928996	8	12	---	---	intergenic	---
3	112676561	112676561	+	112676561	4	4	---	---	intergenic	---
3	112751383	112751383	-	112751383	3	3	---	---	intergenic	---
3	112949325	112949329	+	112949326	101	149	ENSSSCG00000008508	+	3'UTR	*FAM98A*
3	112951532	112951532	+	112951532	3	3	XLOC_055573	+	predicted	---
3	112971457	112971457	-	112971457	3	3	ENSSSCG00000008509	-	3'UTR	*RASGRP3*
3	113199433	113199433	+	113199433	3	3	---	---	intergenic	---
3	113230087	113230088	-	113230087	10	13	ENSSSCG00000008510	-	3'UTR	*LTBP1*
3	113534586	113534586	+	113534586	3	3	---	---	intergenic	---
3	113776181	113776183	+	113776182	9	20	ENSSSCG00000021320	+	3'UTR	---
3	113955615	113955615	-	113955615	3	3	ENSSSCG00000008513	-	3'UTR	*BIRC6*
3	113955904	113955904	-	113955904	3	3	ENSSSCG00000008513	-	3'UTR	*BIRC6*
3	113956078	113956080	-	113956080	6	16	ENSSSCG00000008513	-	3'UTR	*BIRC6*
3	113956419	113956419	-	113956419	3	3	ENSSSCG00000008513	-	3'UTR	*BIRC6*
3	113956464	113956464	-	113956464	3	3	ENSSSCG00000008513	-	3'UTR	*BIRC6*
3	113999400	113999400	-	113999400	4	4	ENSSSCG00000008513	-	ORF	*BIRC6*
3	114091035	114091038	-	114091037	12	21	ENSSSCG00000008513	-	intron	*BIRC6*
3	114271130	114271130	-	114271130	3	3	---	---	intergenic	---
3	114306305	114306305	-	114306305	13	13	ENSSSCG00000008515	-	3'UTR	*MEMO1*
3	114420750	114420750	+	114420750	5	5	---	---	intergenic	---
3	114427338	114427348	-	114427339	51	85	ENSSSCG00000008516	-	3'UTR	*DPY30*
3	114492189	114492189	+	114492189	6	6	ENSSSCG00000008517	+	intron	*SPAST*

3	114521793	114521793	-	114521793	4	4	XLOC_056834	-	predicted	---
3	114524173	114524173	-	114524173	4	4	XLOC_056834	-	predicted	---
3	114549528	114549528	-	114549528	6	6	---	---	intergenic	---
3	114579449	114579449	-	114579449	3	3	---	---	intergenic	---
3	114796786	114796800	+	114796786	5	8	ENSSSCG00000008522	+	3'UTR	*XDH*
3	114797152	114797153	+	114797152	6	9	ENSSSCG00000008522	+	3'UTR	*XDH*
3	114941115	114941115	-	114941115	3	3	XLOC_056839	-	predicted	---
3	115256087	115256087	-	115256087	4	4	XLOC_056840	-	predicted	---
3	115453392	115453392	+	115453392	6	6	ENSSSCG00000025901	+	intron	*LCLAT1*
3	115724978	115724980	+	115724979	85	109	---	---	intergenic	---
3	115764424	115764425	-	115764424	13	17	XLOC_056845	-	predicted	---
3	115810786	115810786	+	115810786	3	3	---	---	intergenic	---
3	115863173	115863178	-	115863174	32	58	ENSSSCG00000008529	-	3'UTR	*YPEL5*
3	115864123	115864130	-	115864128	34	66	ENSSSCG00000008529	-	3'UTR	*YPEL5*
3	115884635	115884636	+	115884636	9	12	ENSSSCG00000030917	+	ORF	*YWHAE*
3	115945774	115945774	+	115945774	3	3	---	---	intergenic	---
3	116018076	116018076	+	116018076	6	6	---	---	intergenic	---
3	116525624	116525627	+	116525625	7	26	XLOC_055609	+	predicted	---
3	116526011	116526012	+	116526011	7	11	XLOC_055609	+	predicted	---
3	116526455	116526455	+	116526455	3	3	XLOC_055609	+	predicted	---
3	116931706	116931706	-	116931706	3	3	XLOC_056848	-	predicted	---
3	116936942	116936942	-	116936942	3	3	ENSSSCG00000008535	-	intron	*CLIP4*
3	116938366	116938366	-	116938366	4	4	ENSSSCG00000008535	-	intron	*CLIP4*
3	116938429	116938434	-	116938430	48	76	ENSSSCG00000008535	-	intron	*CLIP4*
3	116966529	116966529	-	116966529	5	5	ENSSSCG00000008535	-	ORF	*CLIP4*
3	117131906	117131906	+	117131906	3	3	ENSSSCG00000026017	+	intron	---

3	117165163	117165167	-	117165167	5	9	XLOC_056850	-	predicted	---
3	117166368	117166368	-	117166368	4	4	ENSSSCG00000008536	-	3'UTR	---
3	117166651	117166651	-	117166651	4	4	ENSSSCG00000008536	-	ORF	---
3	117219740	117219740	-	117219740	6	6	ENSSSCG00000008538	-	3'UTR	*RINGO*
3	117220187	117220251	-	117220204	26	152	ENSSSCG00000008538	-	3'UTR	*RINGO*
3	117220332	117220332	-	117220332	5	5	ENSSSCG00000008538	-	3'UTR	*RINGO*
3	117228488	117228492	-	117228492	9	12	ENSSSCG00000008538	-	intron	*RINGO*
3	117228830	117228830	-	117228830	7	7	ENSSSCG00000008538	-	intron	*RINGO*
3	117275118	117275130	+	117275124	55	125	ENSSSCG00000008540	+	3'UTR	*PPP1CB*
3	117279678	117279678	+	117279678	3	3	ENSSSCG00000008539	+	intron	---
3	117360471	117360471	-	117360471	3	3	XLOC_056859	-	predicted	---
3	117407646	117407698	+	117407653	14	74	XLOC_055621	+	predicted	---
3	117474820	117474821	-	117474820	5	8	---	---	intergenic	---
3	117476310	117476310	+	117476310	3	3	XLOC_055620	+	predicted	---
3	117491052	117491052	+	117491052	6	6	XLOC_055622	+	predicted	---
3	117494844	117494844	+	117494844	5	5	XLOC_055622	+	predicted	---
3	117700101	117700102	-	117700101	3	6	---	---	intergenic	---
3	117700200	117700215	-	117700207	17	67	XLOC_056866	-	predicted	---
3	117803644	117803644	-	117803644	3	3	---	---	intergenic	---
3	117985608	117985608	-	117985608	4	4	---	---	intergenic	---
3	118308189	118308189	+	118308189	5	5	---	---	intergenic	---
3	118337534	118337534	+	118337534	6	6	---	---	intergenic	---
3	118462143	118462148	+	118462144	26	63	XLOC_055631	+	predicted	---
3	118465402	118465402	-	118465402	3	3	XLOC_056874	-	predicted	---
3	118493276	118493276	-	118493276	3	3	ENSSSCG00000008549	-	ORF	*SLC4A1AP*
3	118507939	118507939	-	118507939	13	13	ENSSSCG00000008546	-	3'UTR	*GPN1*

3	118507959	118507964	+	118507959	3	6	ENSSSCG00000008548	+	3'UTR	*SUPT7L*
3	118535158	118535164	+	118535159	7	19	---	---	intergenic	---
3	118538976	118538980	-	118538976	8	16	ENSSSCG00000008545	-	3'UTR	*ZNF512*
3	118573218	118573226	-	118573218	7	12	XLOC_056876	-	predicted	---
3	118590767	118590776	-	118590776	45	62	XLOC_056877	-	predicted	---
3	118597605	118597605	-	118597605	10	10	---	---	intergenic	---
3	118698629	118698634	+	118698632	76	141	ENSSSCG00000025363	+	3'UTR	*SNX17*
3	118698828	118698828	+	118698828	3	3	ENSSSCG00000025363	+	3'UTR	*SNX17*
3	118701618	118701618	-	118701618	4	4	XLOC_056879	-	predicted	---
3	118703499	118703515	-	118703507	66	187	ENSSSCG00000027135	-	3'UTR	*PPM1G*
3	118706079	118706079	-	118706079	3	3	ENSSSCG00000027135	-	ORF	*PPM1G*
3	118739758	118739758	-	118739758	3	3	---	---	intergenic	---
3	118754392	118754404	-	118754404	19	60	ENSSSCG00000026367	-	3'UTR	*IFT172*
3	118791034	118791041	-	118791041	5	12	ENSSSCG00000029305	-	3'UTR	*FNDC4*
3	118823336	118823338	+	118823336	18	25	ENSSSCG00000028672	+	3'UTR	*GCKR*
3	118845658	118845658	+	118845658	4	4	---	---	intergenic	---
3	118849040	118849040	+	118849040	3	3	XLOC_055638	+	predicted	---
3	118912960	118912969	-	118912966	103	229	ENSSSCG00000022058	-	3'UTR	*ATRAID*
3	118929189	118929198	+	118929192	163	298	ENSSSCG00000008550	+	3'UTR	*SLC5A6*
3	118987919	118987920	+	118987920	9	13	ENSSSCG00000008552	+	ORF	*PRR30*
3	118989287	118989298	+	118989290	38	83	XLOC_055641	+	predicted	---
3	118994624	118994624	-	118994624	4	4	ENSSSCG00000008554	-	3'UTR	*ABHD1*
3	118994655	118994674	+	118994662	12	29	ENSSSCG00000008553	+	3'UTR	*PREB*
3	119018198	119018207	+	119018206	7	17	ENSSSCG00000008555	+	3'UTR	*CGREF1*
3	119019653	119019662	-	119019659	10	34	XLOC_056886	-	predicted	---
3	119019712	119019743	-	119019719	267	1229	XLOC_056886	-	predicted	---

3	119045890	119045899	-	119045898	23	33	---	---	intergenic	---
3	119054613	119054615	-	119054614	4	10	ENSSSCG00000008558	-	intron	*AGBL5*
3	119057044	119057052	-	119057045	23	72	ENSSSCG00000008558	-	intron	*AGBL5*
3	119060159	119060159	-	119060159	3	3	---	---	intergenic	---
3	119070712	119070716	-	119070714	56	76	ENSSSCG00000008559	-	3'UTR	*TMEM214*
3	119083234	119083234	-	119083234	4	4	XLOC_056889	-	predicted	---
3	119084018	119084026	-	119084023	52	117	XLOC_056889	-	predicted	---
3	119279995	119279995	-	119279995	3	3	ENSSSCG00000008562	-	3'UTR	*SLC35F6*
3	119430893	119430899	+	119430893	3	6	ENSSSCG00000008564	+	intron	*CIB4*
3	119456531	119456546	+	119456544	55	92	XLOC_055651	+	predicted	---
3	119458323	119458326	-	119458325	58	97	XLOC_056896	-	predicted	---
3	119564020	119564020	-	119564020	7	7	XLOC_056899	-	predicted	---
3	119566233	119566235	-	119566234	20	30	XLOC_056899	-	predicted	---
3	119568391	119568391	-	119568391	4	4	XLOC_056899	-	predicted	---
3	119570241	119570241	-	119570241	3	3	XLOC_056899	-	predicted	---
3	119650381	119650390	-	119650382	16	32	ENSSSCG00000008569	-	3'UTR	*LCTHIO*
3	119650683	119650697	-	119650688	36	143	ENSSSCG00000008569	-	3'UTR	*LCTHIO*
3	119660244	119660244	-	119660244	3	3	ENSSSCG00000008569	-	ORF	*LCTHIO*
3	119717735	119717735	-	119717735	5	5	---	---	intergenic	---
3	119729831	119729831	+	119729831	3	3	ENSSSCG00000008571	+	intron	*HADHA*
3	119736923	119736933	-	119736933	7	18	ENSSSCG00000008568	-	intron	*DRC1*
3	119782674	119782675	+	119782675	6	9	ENSSSCG00000008571	+	3'UTR	*HADHA*
3	119782858	119782866	+	119782862	107	258	ENSSSCG00000008571	+	3'UTR	*HADHA*
3	119782960	119782974	+	119782962	41	94	XLOC_055653	+	predicted	---
3	119827430	119827431	-	119827430	6	11	ENSSSCG00000008573	-	3'UTR	*RAB10*
3	119827471	119827473	-	119827473	17	20	ENSSSCG00000008573	-	3'UTR	*RAB10*

3	119829517	119829517	-	119829517	3	3	ENSSSCG00000008573	-	3'UTR	*RAB10*
3	119829777	119829783	-	119829777	8	11	ENSSSCG00000008573	-	3'UTR	*RAB10*
3	119945105	119945105	+	119945105	5	5	---	---	intergenic	---
3	120772977	120772984	+	120772977	80	160	ENSSSCG00000021155	+	3'UTR	*POMC*
3	120775184	120775184	-	120775184	4	4	XLOC_056910	-	predicted	---
3	120823754	120823754	+	120823754	3	3	ENSSSCG00000026767	+	intron	---
3	120867319	120867322	-	120867321	6	19	---	---	intergenic	---
3	120867936	120867936	-	120867936	4	4	---	---	intergenic	---
3	120868594	120868594	-	120868594	5	5	---	---	intergenic	---
3	120879386	120879386	-	120879386	3	3	---	---	intergenic	---
3	121045807	121045807	+	121045807	8	8	XLOC_055667	+	predicted	---
3	121199866	121199866	-	121199866	3	3	ENSSSCG00000008579	-	3'UTR	*CENPO*
3	121199907	121199907	+	121199907	4	4	ENSSSCG00000008578	+	3'UTR	*ADCY3*
3	121213391	121213399	+	121213391	34	48	ENSSSCG00000008580	+	3'UTR	*PTRHD1*
3	121213798	121213799	+	121213798	7	10	---	---	intergenic	---
3	121228098	121228098	-	121228098	3	3	ENSSSCG00000008581	-	3'UTR	*NCOA1*
3	121453876	121453876	-	121453876	3	3	---	---	intergenic	---
3	121686925	121686925	+	121686925	12	12	ENSSSCG00000008583	+	intron	*ITSN2*
3	121688672	121688672	+	121688672	3	3	ENSSSCG00000008583	+	ORF	*ITSN2*
3	121697535	121697535	+	121697535	4	4	ENSSSCG00000008583	+	ORF	*ITSN2*
3	121707477	121707483	+	121707477	9	13	ENSSSCG00000008583	+	intron	*ITSN2*
3	121772528	121772530	+	121772529	5	12	ENSSSCG00000008583	+	3'UTR	*ITSN2*
3	121772833	121772833	+	121772833	6	6	ENSSSCG00000008583	+	3'UTR	*ITSN2*
3	121773088	121773088	+	121773088	6	6	ENSSSCG00000008583	+	3'UTR	*ITSN2*
3	121775454	121775456	-	121775454	14	20	---	---	intergenic	---
3	121799966	121799966	-	121799966	3	3	ENSSSCG00000008582	-	intron	*FAM228A*

3	121801032	121801032	-	121801032	8	8	---	---	intergenic	---
3	121802237	121802239	-	121802238	10	22	XLOC_056919	-	predicted	---
3	121809703	121809703	+	121809703	4	4	XLOC_055671	+	predicted	---
3	121849261	121849262	+	121849261	3	6	XLOC_055672	+	predicted	---
3	121849314	121849320	+	121849316	9	18	XLOC_055672	+	predicted	---
3	121849600	121849602	+	121849601	15	35	---	---	intergenic	---
3	121882892	121882900	+	121882899	66	109	ENSSSCG00000008587	+	3'UTR	*SF3B6*
3	121888311	121888312	-	121888312	14	17	XLOC_056920	-	predicted	---
3	121917777	121917777	+	121917777	3	3	XLOC_055674	+	predicted	---
3	121937990	121937990	-	121937990	3	3	XLOC_056921	-	predicted	---
3	121938609	121938621	-	121938621	8	11	XLOC_056921	-	predicted	---
3	122061581	122061581	+	122061581	5	5	---	---	intergenic	---
3	122112006	122112006	+	122112006	3	3	XLOC_055678	+	predicted	---
3	122720259	122720259	+	122720259	6	6	XLOC_055682	+	predicted	---
3	122801264	122801265	+	122801264	10	13	---	---	intergenic	---
3	122903642	122903642	-	122903642	3	3	---	---	intergenic	---
3	123494029	123494029	-	123494029	5	5	---	---	intergenic	---
3	123594956	123594956	-	123594956	3	3	---	---	intergenic	---
3	123609558	123609558	+	123609558	3	3	---	---	intergenic	---
3	123683220	123683220	-	123683220	3	3	XLOC_056925	-	predicted	---
3	123799453	123799455	-	123799454	10	21	XLOC_056926	-	predicted	---
3	124019770	124019770	-	124019770	4	4	---	---	intergenic	---
3	125052747	125052747	-	125052747	4	4	---	---	intergenic	---
3	125233403	125233403	+	125233403	4	4	---	---	intergenic	---
3	125234412	125234426	+	125234426	14	32	---	---	intergenic	---
3	125238206	125238206	+	125238206	3	3	---	---	intergenic	---

3	125240305	125240306	+	125240306	4	7	---	---	intergenic	---
3	125240404	125240435	+	125240404	21	29	---	---	intergenic	---
3	125241072	125241072	+	125241072	4	4	---	---	intergenic	---
3	125252008	125252008	+	125252008	3	3	ENSSSCG00000008595	+	ORF	*APOB*
3	125252053	125252064	+	125252064	11	17	ENSSSCG00000008595	+	ORF	*APOB*
3	125252148	125252149	+	125252149	24	28	ENSSSCG00000008595	+	intron	*APOB*
3	125252899	125252905	+	125252905	67	175	ENSSSCG00000008595	+	intron	*APOB*
3	125253062	125253062	+	125253062	3	3	ENSSSCG00000008595	+	intron	*APOB*
3	125253188	125253188	+	125253188	4	4	ENSSSCG00000008595	+	intron	*APOB*
3	125253764	125253795	+	125253764	7	14	XLOC_055686	+	predicted	---
3	125297456	125297459	+	125297459	14	18	XLOC_055687	+	predicted	---
3	125297701	125297701	+	125297701	4	4	XLOC_055687	+	predicted	---
3	125341173	125341198	+	125341173	18	28	---	---	intergenic	---
3	125341261	125341278	+	125341278	19	24	---	---	intergenic	---
3	125341347	125341382	+	125341347	4	8	---	---	intergenic	---
3	125341735	125341735	+	125341735	5	5	---	---	intergenic	---
3	125341958	125341958	+	125341958	13	13	---	---	intergenic	---
3	125342033	125342057	+	125342033	8	12	---	---	intergenic	---
3	125342261	125342261	-	125342261	3	3	---	---	intergenic	---
3	125342372	125342372	+	125342372	6	6	---	---	intergenic	---
3	125342491	125342505	+	125342491	7	12	---	---	intergenic	---
3	125342726	125342726	+	125342726	4	4	---	---	intergenic	---
3	125342891	125342916	+	125342897	6	14	ENSSSCG00000008596	+	ORF	---
3	125343159	125343159	+	125343159	4	4	ENSSSCG00000008596	+	ORF	---
3	125343377	125343413	+	125343377	12	25	ENSSSCG00000008596	+	ORF	---
3	125343478	125343478	+	125343478	7	7	ENSSSCG00000008596	+	ORF	---

3	125343642	125343659	+	125343642	35	47	ENSSSCG00000008596	+	ORF	---
3	125343797	125343797	+	125343797	8	8	ENSSSCG00000008596	+	ORF	---
3	125344071	125344071	+	125344071	78	78	ENSSSCG00000008596	+	ORF	---
3	125344128	125344128	+	125344128	4	4	ENSSSCG00000008596	+	ORF	---
3	125344191	125344191	+	125344191	3	3	ENSSSCG00000008596	+	ORF	---
3	125344308	125344308	+	125344308	6	6	ENSSSCG00000008596	+	ORF	---
3	125344457	125344457	+	125344457	7	7	ENSSSCG00000008596	+	ORF	---
3	125344701	125344701	+	125344701	4	4	ENSSSCG00000008596	+	ORF	---
3	125344952	125344990	+	125344953	125	148	ENSSSCG00000008596	+	ORF	---
3	125345068	125345068	+	125345068	4	4	ENSSSCG00000008596	+	intron	---
3	125345181	125345215	+	125345181	42	60	ENSSSCG00000008596	+	ORF	---
3	125345303	125345303	+	125345303	43	43	ENSSSCG00000008596	+	ORF	---
3	125346096	125346096	+	125346096	6	6	ENSSSCG00000008596	+	ORF	---
3	125346613	125346613	+	125346613	8	8	ENSSSCG00000008596	+	ORF	---
3	125346668	125346668	+	125346668	90	90	ENSSSCG00000008596	+	intron	---
3	125347999	125347999	+	125347999	10	10	ENSSSCG00000008596	+	ORF	---
3	125348064	125348064	+	125348064	3	3	ENSSSCG00000008596	+	ORF	---
3	125348305	125348305	+	125348305	4	4	ENSSSCG00000008596	+	ORF	---
3	125348366	125348383	+	125348383	31	46	ENSSSCG00000008596	+	ORF	---
3	125348594	125348594	+	125348594	5	5	ENSSSCG00000008596	+	ORF	---
3	125348831	125348831	+	125348831	5	5	ENSSSCG00000008596	+	ORF	---
3	125348906	125348906	+	125348906	3	3	ENSSSCG00000008596	+	ORF	---
3	125349009	125349023	+	125349009	3	6	ENSSSCG00000008596	+	ORF	---
3	125349213	125349213	+	125349213	8	8	ENSSSCG00000008596	+	ORF	---
3	125349462	125349462	+	125349462	3	3	---	---	intergenic	---
3	125532639	125532639	+	125532639	3	3	ENSSSCG00000025357	+	3'UTR	*LDAH*

3	125534848	125534848	+	125534848	4	4	ENSSSCG00000025357	+	3'UTR	*LDAH*
3	125606990	125606990	+	125606990	4	4	ENSSSCG00000030115	+	3'UTR	---
3	125607911	125607916	+	125607911	6	11	ENSSSCG00000030115	+	3'UTR	---
3	125662662	125662662	-	125662662	8	8	XLOC_056935	-	predicted	---
3	125745804	125745811	-	125745807	20	38	ENSSSCG00000008598	-	3'UTR	*RHOB*
3	125745900	125745901	-	125745901	22	28	ENSSSCG00000008598	-	3'UTR	*RHOB*
3	125827716	125827716	+	125827716	10	10	ENSSSCG00000008600	+	intron	*PUM2*
3	125984666	125984686	+	125984686	9	21	---	---	intergenic	---
3	125988756	125988764	-	125988762	24	73	ENSSSCG00000008601	-	3'UTR	*SDC1*
3	125989441	125989447	-	125989441	3	6	ENSSSCG00000008601	-	3'UTR	*SDC1*
							ENSSSCG00000008601	-	intron	*SDC1*
3	126081759	126081760	+	126081759	8	12	XLOC_055698	+	predicted	---
3	126200614	126200654	+	126200652	227	483	ENSSSCG00000008602	+	3'UTR	*LAPTM4A*
3	126200771	126200774	+	126200774	4	7	ENSSSCG00000008602	+	3'UTR	*LAPTM4A*
3	126201147	126201158	+	126201156	162	291	ENSSSCG00000008602	+	3'UTR	*LAPTM4A*
3	126246675	126246675	+	126246675	3	3	---	---	intergenic	---
3	126296750	126296753	+	126296750	7	12	XLOC_055700	+	predicted	---
3	126296928	126296929	+	126296928	3	6	XLOC_055700	+	predicted	---
3	126299254	126299254	+	126299254	3	3	XLOC_055700	+	predicted	---
3	126309194	126309194	+	126309194	6	6	XLOC_055700	+	predicted	---
3	126460115	126460115	-	126460115	3	3	---	---	intergenic	---
3	126466121	126466122	+	126466122	6	11	---	---	intergenic	---
3	126497500	126497500	+	126497500	3	3	---	---	intergenic	---
3	126508240	126508244	+	126508242	10	20	XLOC_055700	+	predicted	---
3	126720285	126720312	-	126720292	27	66	XLOC_056943	-	predicted	---
3	126722525	126722525	-	126722525	4	4	XLOC_056943	-	predicted	---

3	126846784	126846793	-	126846785	21	46	XLOC_056944	-	predicted	---
3	126981618	126981618	-	126981618	3	3	---	---	intergenic	---
3	127522935	127522935	+	127522935	12	12	ENSSSCG00000008607	+	ORF	---
3	127539942	127539942	+	127539942	6	6	ENSSSCG00000008607	+	ORF	---
3	127540050	127540065	+	127540053	241	568	ENSSSCG00000008607	+	3'UTR	---
3	127540573	127540573	+	127540573	3	3	XLOC_055707	+	predicted	---
3	127547393	127547394	+	127547393	6	10	ENSSSCG00000008608	+	3'UTR	*RDH14*
3	128141958	128141958	-	128141958	3	3	ENSSSCG00000023060	-	ORF	---
3	128273508	128273508	+	128273508	4	4	ENSSSCG00000008614	+	intron	*SMC6*
3	128317393	128317401	+	128317401	9	23	ENSSSCG00000008614	+	3'UTR	*SMC6*
3	128318838	128318838	+	128318838	3	3	XLOC_055711	+	predicted	---
3	128464744	128464750	+	128464745	14	39	---	---	intergenic	---
3	128956746	128956746	-	128956746	3	3	---	---	intergenic	---
3	129103181	129103182	-	129103182	22	29	---	---	intergenic	---
3	129177554	129177554	-	129177554	3	3	---	---	intergenic	---
3	129319021	129319021	+	129319021	4	4	XLOC_055718	+	predicted	---
3	130457630	130457630	+	130457630	4	4	ENSSSCG00000008619	+	ORF	---
3	130887173	130887175	-	130887174	6	13	ENSSSCG00000008621	-	intron	---
3	131113409	131113409	-	131113409	14	14	---	---	intergenic	---
3	131214848	131214850	-	131214849	11	20	---	---	intergenic	---
3	131837036	131837038	-	131837036	4	7	---	---	intergenic	---
3	132473005	132473005	+	132473005	4	4	---	---	intergenic	---
3	132641815	132641815	-	132641815	3	3	XLOC_056968	-	predicted	---
3	132790608	132790612	-	132790612	8	15	XLOC_056970	-	predicted	---
3	133118943	133118943	+	133118943	8	8	---	---	intergenic	---
3	133322123	133322126	+	133322124	30	43	XLOC_055734	+	predicted	---

3	133440597	133440597	-	133440597	4	4	---	---	intergenic	---
3	133441788	133441788	-	133441788	3	3	XLOC_056976	-	predicted	---
3	133589351	133589351	-	133589351	4	4	---	---	intergenic	---
3	133589378	133589378	+	133589378	4	4	---	---	intergenic	---
3	133660297	133660297	-	133660297	3	3	ENSSSCG00000008624	-	3'UTR	---
3	133662951	133662977	-	133662975	58	97	ENSSSCG00000008624	-	3'UTR	---
3	133758024	133758024	-	133758024	3	3	---	---	intergenic	---
3	133761517	133761517	-	133761517	6	6	---	---	intergenic	---
3	133898923	133898923	-	133898923	3	3	---	---	intergenic	---
3	133906998	133907002	-	133907001	5	13	---	---	intergenic	---
3	133912902	133912902	+	133912902	5	5	XLOC_055737	+	predicted	---
3	133976917	133976917	+	133976917	3	3	---	---	intergenic	---
3	134581175	134581182	-	134581177	28	54	XLOC_056987	-	predicted	---
3	134592275	134592276	-	134592275	6	10	ENSSSCG00000008633	-	ORF	*PDI-P5*
3	134620755	134620755	-	134620755	10	10	---	---	intergenic	---
3	134632261	134632261	+	134632261	3	3	ENSSSCG00000008635	+	intron	*NOL10*
3	134723596	134723598	+	134723597	12	19	XLOC_055747	+	predicted	---
3	134748614	134748614	+	134748614	24	24	---	---	intergenic	---
3	134932412	134932412	-	134932412	4	4	---	---	intergenic	---
3	134976202	134976202	+	134976202	5	5	ENSSSCG00000008637	+	intron	*TAF1B*
3	135003158	135003159	+	135003158	9	14	ENSSSCG00000008637	+	3'UTR	*TAF1B*
3	135035109	135035110	-	135035110	10	13	---	---	intergenic	---
3	135122408	135122408	-	135122408	5	5	ENSSSCG00000008640	-	3'UTR	*IAH1*
3	135122446	135122446	+	135122446	9	9	ENSSSCG00000008641	+	3'UTR	---
							ENSSSCG00000008641	+	ORF	---
3	135122586	135122590	-	135122586	45	82	ENSSSCG00000008640	-	3'UTR	*IAH1*

3	135137423	135137426	-	135137426	11	29	ENSSSCG00000020799	-	3'UTR	*CPSF3*
3	135245475	135245487	-	135245487	53	99	XLOC_056992	-	predicted	---
3	135245557	135245557	-	135245557	11	11	XLOC_056992	-	predicted	---
3	135245820	135245847	-	135245824	49	99	XLOC_056992	-	predicted	---
3	135246224	135246224	-	135246224	5	5	XLOC_056992	-	predicted	---
3	135280651	135280651	+	135280651	4	4	ENSSSCG00000029313	+	intron	*ITGB1BP1*
3	135295230	135295231	+	135295230	6	12	ENSSSCG00000029313	+	3'UTR	*ITGB1BP1*
3	135497052	135497052	-	135497052	3	3	---	---	intergenic	---
3	135597470	135597470	+	135597470	3	3	XLOC_055756	+	predicted	---
3	135606295	135606295	+	135606295	8	8	XLOC_055756	+	predicted	---
3	135614994	135614994	+	135614994	8	8	XLOC_055756	+	predicted	---
3	135615271	135615272	+	135615271	4	8	XLOC_055756	+	predicted	---
3	135775248	135775255	+	135775249	13	29	ENSSSCG00000008644	+	3'UTR	---
3	135792911	135792911	-	135792911	5	5	---	---	intergenic	---
3	135801816	135801816	-	135801816	5	5	ENSSSCG00000008645	-	3'UTR	*ID2*
3	135801947	135801951	-	135801947	17	20	ENSSSCG00000008645	-	3'UTR	*ID2*
3	135825586	135825586	+	135825586	3	3	XLOC_055759	+	predicted	---
3	136060808	136060814	+	136060811	20	49	XLOC_055763	+	predicted	---
3	136062636	136062645	+	136062638	19	43	XLOC_055763	+	predicted	---
3	136094063	136094063	+	136094063	6	6	---	---	intergenic	---
3	137499598	137499605	-	137499604	10	21	---	---	intergenic	---
3	137618444	137618444	-	137618444	37	37	---	---	intergenic	---
3	137665476	137665476	-	137665476	3	3	---	---	intergenic	---
3	137703871	137703872	+	137703871	4	7	ENSSSCG00000008647	+	3'UTR	*CMPK2*
3	137704123	137704129	+	137704123	33	98	ENSSSCG00000008647	+	3'UTR	*CMPK2*
3	137791614	137791614	-	137791614	3	3	---	---	intergenic	---

3	137962455	137962455	+	137962455	3	3	---	---	intergenic	---
3	138417226	138417226	-	138417226	3	3	---	---	intergenic	---
3	139072198	139072198	+	139072198	3	3	---	---	intergenic	---
3	139313791	139313791	+	139313791	4	4	---	---	intergenic	---
3	139503834	139503834	+	139503834	3	3	XLOC_055774	+	predicted	---
3	139811189	139811192	-	139811190	9	19	ENSSSCG00000024926	-	3'UTR	*ASGR1*
3	139857141	139857148	+	139857142	97	161	ENSSSCG00000020972	+	3'UTR	*ACADVL*
3	139857220	139857220	-	139857220	6	6	XLOC_057016	-	predicted	---
3	139866342	139866344	-	139866342	5	8	ENSSSCG00000029345	-	3'UTR	*PHF23*
3	139934252	139934257	-	139934255	8	19	---	---	intergenic	---
3	139934366	139934366	-	139934366	3	3	XLOC_057021	-	predicted	---
3	139934442	139934442	-	139934442	3	3	XLOC_057021	-	predicted	---
3	139940011	139940011	+	139940011	11	11	ENSSSCG00000017939	+	3'UTR	---
3	139940429	139940478	+	139940468	259	450	ENSSSCG00000017939	+	3'UTR	---
3	139940519	139940519	+	139940519	3	3	ENSSSCG00000017939	+	3'UTR	---
3	139940659	139940670	-	139940664	78	227	ENSSSCG00000017940	-	3'UTR	*GPS2*
3	139942329	139942329	-	139942329	5	5	ENSSSCG00000017940	-	ORF	*GPS2*
3	140305771	140305771	-	140305771	3	3	XLOC_057025	-	predicted	---
3	140404714	140404718	-	140404715	4	10	XLOC_057026	-	predicted	---
3	140421207	140421213	-	140421207	9	20	ENSSSCG00000008650	-	intron	*ALLC*
3	140442314	140442321	+	140442314	18	30	ENSSSCG00000025305	+	3'UTR	---
3	140442665	140442665	+	140442665	10	10	ENSSSCG00000025305	+	3'UTR	---
3	140444633	140444634	+	140444633	4	7	ENSSSCG00000025305	+	3'UTR	---
3	140454533	140454533	+	140454533	7	7	XLOC_055784	+	predicted	---
3	140469259	140469268	+	140469268	26	71	ENSSSCG00000029161	+	3'UTR	*ADI1*
3	140477383	140477386	-	140477383	23	60	ENSSSCG00000024665	-	3'UTR	*TRAPPC12*

3	140478387	140478387	-	140478387	3	3	ENSSSCG00000024665	-	intron	*TRAPPC12*
3	140517630	140517630	+	140517630	3	3	ENSSSCG00000027353	+	ORF	---
3	140521044	140521044	+	140521044	25	25	ENSSSCG00000027353	+	ORF	---
3	140521638	140521644	+	140521642	219	667	ENSSSCG00000027353	+	3'UTR	---
3	140533703	140533703	+	140533703	3	3	ENSSSCG00000025629	+	ORF	*COLEC11*
3	140547557	140547557	+	140547557	7	7	ENSSSCG00000025629	+	intron	*COLEC11*
3	140553398	140553407	+	140553400	36	95	ENSSSCG00000025629	+	3'UTR	*COLEC11*
3	140555919	140555926	-	140555926	5	8	ENSSSCG00000024665	-	intron	*TRAPPC12*
3	140627434	140627434	+	140627434	3	3	ENSSSCG00000028755	+	intron	*TSSC1*
3	140709674	140709674	+	140709674	4	4	XLOC_055788	+	predicted	---
3	142395999	142396024	+	142396017	159	554	ENSSSCG00000008659	+	3'UTR	*ACP1*
3	142396736	142396737	+	142396737	7	11	ENSSSCG00000008659	+	3'UTR	*ACP1*
3	143731632	143731632	+	143731632	4	4	---	---	intergenic	---
3	143756265	143756266	-	143756266	57	63	---	---	intergenic	---
3	143821222	143821223	-	143821222	6	11	---	---	intergenic	---
3	143905448	143905448	-	143905448	12	12	XLOC_057051	-	predicted	---
3	143960019	143960019	-	143960019	4	4	---	---	intergenic	---
3	144233862	144233862	-	144233862	9	9	XLOC_057057	-	predicted	---
3	144234838	144234838	-	144234838	3	3	XLOC_057057	-	predicted	---
3	144252188	144252194	+	144252193	9	18	---	---	intergenic	---
3	144541917	144541917	-	144541917	14	14	---	---	intergenic	---
4	64700	64701	+	64700	4	7	XLOC_060902	+	predicted	---
4	101119	101119	-	101119	3	3	ENSSSCG00000027957	-	3'UTR	*ZNF250*
4	117607	117613	-	117613	37	53	XLOC_061975	-	predicted	---
4	207988	207989	+	207988	4	7	ENSSSCG00000029830	+	intron	*RPL8*
4	209469	209469	+	209469	21	21	ENSSSCG00000029830	+	3'UTR	*RPL8*

4	209514	209524	+	209517	1026	2257	ENSSSCG00000029830	+	3'UTR	*RPL8*
4	279035	279037	+	279035	4	7	---	---	intergenic	---
4	369547	369550	+	369549	27	55	ENSSSCG00000005896	+	intron	*LRRC24*
4	373972	374001	-	373972	5	14	ENSSSCG00000030451	-	intron	*LRRC14*
4	384711	384714	-	384713	28	61	---	---	intergenic	---
4	388747	388747	-	388747	10	10	ENSSSCG00000005900	-	3'UTR	*GPT*
4	393622	393624	-	393622	13	21	ENSSSCG00000005901	-	3'UTR	*PPP1R16A*
4	425164	425164	+	425164	3	3	ENSSSCG00000027076	+	intron	*CYHR1*
4	435984	435986	+	435984	3	6	ENSSSCG00000027076	+	intron	*CYHR1*
4	452348	452348	+	452348	3	3	ENSSSCG00000025116	+	3'UTR	*TONSL*
4	458609	458640	+	458617	43	211	ENSSSCG00000005904	+	3'UTR	*VPS28*
4	469119	469124	+	469122	28	64	ENSSSCG00000005905	+	3'UTR	*ZIP4*
4	482140	482162	+	482149	49	140	---	---	intergenic	---
4	482154	482160	-	482160	13	16	ENSSSCG00000005907	-	3'UTR	*ADCK5*
4	518687	518689	+	518688	58	73	---	---	intergenic	---
4	586100	586104	+	586104	10	30	XLOC_060921	+	predicted	---
4	590368	590378	-	590376	8	24	XLOC_061985	-	predicted	---
4	609269	609270	+	609270	13	17	ENSSSCG00000005918	+	3'UTR	*DGAT1*
4	610322	610325	-	610323	31	52	ENSSSCG00000005917	-	3'UTR	*HSF1*
4	681111	681114	+	681111	7	14	ENSSSCG00000005921	+	3'UTR	---
4	681205	681207	-	681206	9	16	ENSSSCG00000005920	-	3'UTR	*BOP1*
4	696782	696782	-	696782	4	4	---	---	intergenic	---
4	699066	699066	-	699066	3	3	XLOC_061990	-	predicted	---
4	750536	750544	-	750542	37	80	ENSSSCG00000005915	-	3'UTR	*MAF1*
4	790225	790241	-	790240	4	11	ENSSSCG00000005926	-	3'UTR	*PARP10*
4	815891	815892	+	815891	6	12	XLOC_060928	+	predicted	---

4	828840	828842	-	828842	34	42	XLOC_061997	-	predicted	---
4	1012577	1012581	-	1012579	14	30	---	---	intergenic	---
4	1013997	1014005	+	1014001	7	24	XLOC_060938	+	predicted	---
4	1014540	1014540	-	1014540	3	3	---	---	intergenic	---
4	1031569	1031569	+	1031569	9	9	---	---	intergenic	---
4	1034960	1034962	+	1034962	6	9	XLOC_060937	+	predicted	---
4	1035532	1035533	+	1035532	3	6	---	---	intergenic	---
4	1047352	1047378	+	1047370	233	336	ENSSSCG00000006954	+	3'UTR	---
4	1054286	1054289	-	1054286	24	36	ENSSSCG00000006955	-	3'UTR	*GSDMD*
4	1121843	1121846	-	1121845	28	54	---	---	intergenic	---
4	1121895	1121895	-	1121895	4	4	---	---	intergenic	---
4	1182154	1182154	-	1182154	5	5	ENSSSCG00000027691	-	3'UTR	*ZC3H3*
4	1302567	1302567	-	1302567	3	3	XLOC_062008	-	predicted	---
4	1305096	1305096	-	1305096	3	3	XLOC_062008	-	predicted	---
4	1543961	1543961	+	1543961	5	5	---	---	intergenic	---
4	2034073	2034073	+	2034073	4	4	XLOC_060953	+	predicted	---
4	2041675	2041681	-	2041681	12	21	ENSSSCG00000025283	-	3'UTR	*DENND3*
4	2151891	2151891	+	2151891	3	3	XLOC_060954	+	predicted	---
4	2424745	2424745	+	2424745	8	8	---	---	intergenic	---
4	2635742	2635742	+	2635742	5	5	ENSSSCG00000005934	+	intron	*TRAPPC9*
4	2946657	2946667	+	2946660	35	84	XLOC_060967	+	predicted	---
4	3021008	3021011	+	3021011	7	15	ENSSSCG00000005934	+	3'UTR	*TRAPPC9*
4	3243340	3243340	-	3243340	3	3	---	---	intergenic	---
4	3307859	3307859	+	3307859	4	4	XLOC_060969	+	predicted	---
4	3391611	3391611	+	3391611	3	3	---	---	intergenic	---
4	3767671	3767672	+	3767672	28	34	---	---	intergenic	---

4	3793616	3793616	-	3793616	3	3	---	---	intergenic	---
4	3841127	3841134	-	3841133	10	16	XLOC_062042	-	predicted	---
4	3841948	3841948	-	3841948	3	3	XLOC_062042	-	predicted	---
4	3933296	3933296	+	3933296	6	6	ENSSSCG00000005938	+	intron	---
4	4339885	4339885	+	4339885	3	3	ENSSSCG00000005939	+	intron	*FAM135B*
4	4340707	4340707	+	4340707	3	3	XLOC_060973	+	predicted	---
4	4376424	4376424	+	4376424	11	11	---	---	intergenic	---
4	4563745	4563745	-	4563745	6	6	---	---	intergenic	---
4	5498807	5498807	-	5498807	3	3	---	---	intergenic	---
4	5504424	5504424	-	5504424	3	3	---	---	intergenic	---
4	5635367	5635367	-	5635367	3	3	---	---	intergenic	---
4	6127960	6127966	-	6127966	8	11	XLOC_062053	-	predicted	---
4	6135481	6135482	-	6135481	20	23	ENSSSCG00000005941	-	3'UTR	*havana*
							ENSSSCG00000005941	-	intron	*havana*
4	6135884	6135893	-	6135885	11	18	ENSSSCG00000005941	-	3'UTR	*havana*
							ENSSSCG00000005941	-	intron	*havana*
4	6288074	6288100	+	6288074	5	11	---	---	intergenic	---
4	6288337	6288337	+	6288337	4	4	---	---	intergenic	---
4	6290963	6290963	+	6290963	3	3	---	---	intergenic	---
4	6298529	6298532	+	6298529	4	10	---	---	intergenic	---
4	6457092	6457092	-	6457092	3	3	---	---	intergenic	---
4	6515730	6515730	+	6515730	6	6	---	---	intergenic	---
4	6725654	6725654	-	6725654	3	3	XLOC_062055	-	predicted	---
4	6989074	6989074	+	6989074	3	3	ENSSSCG00000030947	+	3'UTR	*CU571095.1*
4	7649823	7649823	-	7649823	3	3	---	---	intergenic	---
4	7704891	7704891	+	7704891	7	7	ENSSSCG00000005943	+	intron	*ST3GAL1*

4	7744536	7744538	+	7744538	37	55	ENSSSCG00000005943	+	intron	*ST3GAL1*
4	7773457	7773457	+	7773457	4	4	ENSSSCG00000005943	+	3'UTR	*ST3GAL1*
4	7774201	7774201	+	7774201	3	3	ENSSSCG00000005943	+	3'UTR	*ST3GAL1*
4	7776987	7776990	+	7776987	29	39	ENSSSCG00000005943	+	3'UTR	*ST3GAL1*
							ENSSSCG00000005943	+	ORF	---
4	7859000	7859000	-	7859000	3	3	XLOC_062066	-	predicted	---
4	7979396	7979399	-	7979398	15	24	XLOC_062068	-	predicted	---
4	8049569	8049569	+	8049569	6	6	---	---	intergenic	---
4	8203477	8203479	-	8203478	10	19	ENSSSCG00000005948	-	intron	*TG*
4	8437442	8437442	-	8437442	5	5	XLOC_062071	-	predicted	---
4	8446329	8446329	-	8446329	3	3	ENSSSCG00000005950	-	ORF	*PHF20L1*
4	8469588	8469595	-	8469592	5	15	ENSSSCG00000005950	-	intron	*PHF20L1*
4	8556577	8556577	+	8556577	3	3	ENSSSCG00000023825	+	3'UTR	---
4	8572619	8572619	+	8572619	3	3	ENSSSCG00000005952	+	intron	*LRRC6*
4	8583683	8583683	+	8583683	3	3	---	---	intergenic	---
4	8597438	8597449	+	8597448	7	17	ENSSSCG00000005952	+	intron	*LRRC6*
4	8608550	8608566	+	8608550	9	16	---	---	intergenic	---
4	8643994	8644011	+	8643995	67	143	ENSSSCG00000005952	+	3'UTR	*LRRC6*
							ENSSSCG00000005952	+	ORF	---
4	9089984	9089997	-	9089984	18	52	ENSSSCG00000005955	-	3'UTR	*EFR3A*
4	9090997	9090997	-	9090997	5	5	ENSSSCG00000005955	-	3'UTR	*EFR3A*
4	10215362	10215364	+	10215362	4	7	---	---	intergenic	---
4	10241240	10241240	+	10241240	21	21	---	---	intergenic	---
4	10446963	10446963	+	10446963	4	4	---	---	intergenic	---
4	10667093	10667095	+	10667095	7	14	ENSSSCG00000028759	+	3'UTR	---
4	10668855	10668855	+	10668855	8	8	ENSSSCG00000028759	+	3'UTR	---

4	10752186	10752186	+	10752186	18	18	---	---	intergenic	---
4	10859540	10859543	+	10859543	18	27	ENSSSCG00000005959	+	3'UTR	*FAM49B*
4	11049049	11049049	+	11049049	12	12	---	---	intergenic	---
4	11173977	11173977	+	11173977	3	3	XLOC_061023	+	predicted	---
4	12471499	12471499	-	12471499	7	7	---	---	intergenic	---
4	12556522	12556522	-	12556522	3	3	---	---	intergenic	---
4	12771375	12771376	-	12771375	7	13	---	---	intergenic	---
4	12771409	12771409	+	12771409	6	6	---	---	intergenic	---
4	12778432	12778432	-	12778432	3	3	---	---	intergenic	---
4	13156340	13156340	+	13156340	17	17	---	---	intergenic	---
4	13520700	13520700	+	13520700	3	3	XLOC_061029	+	predicted	---
4	14478098	14478098	+	14478098	3	3	XLOC_061032	+	predicted	---
4	14902636	14902637	-	14902636	32	44	---	---	intergenic	---
4	14904540	14904550	+	14904540	10	16	---	---	intergenic	---
4	14904735	14904736	+	14904736	4	7	---	---	intergenic	---
4	14904911	14904911	+	14904911	3	3	---	---	intergenic	---
4	14948774	14948775	-	14948775	16	22	---	---	intergenic	---
4	14948826	14948835	-	14948826	6	14	---	---	intergenic	---
4	15022518	15022518	-	15022518	3	3	ENSSSCG00000005968	-	intron	*CU463271.1*
4	15039952	15039952	-	15039952	4	4	ENSSSCG00000005968	-	intron	*CU463271.1*
4	15042699	15042699	-	15042699	5	5	ENSSSCG00000005968	-	intron	*CU463271.1*
4	15145251	15145251	+	15145251	3	3	---	---	intergenic	---
4	15230034	15230034	+	15230034	3	3	---	---	intergenic	---
4	15246602	15246606	+	15246602	9	13	ENSSSCG00000005969	+	3'UTR	*CU407238.7*
							ENSSSCG00000005969	+	ORF	---
4	15248862	15248864	-	15248864	60	71	ENSSSCG00000005970	-	3'UTR	*SQLE*

4	15253349	15253349	-	15253349	4	4	ENSSSCG00000005970	-	intron	*SQLE*
4	15278474	15278474	-	15278474	3	3	---	---	intergenic	---
4	15293527	15293527	-	15293527	3	3	XLOC_062105	-	predicted	---
4	15333555	15333577	+	15333555	11	23	---	---	intergenic	---
4	15364349	15364349	-	15364349	3	3	XLOC_062107	-	predicted	---
4	15391809	15391812	-	15391809	11	22	---	---	intergenic	---
4	15734029	15734029	+	15734029	4	4	ENSSSCG00000005975	+	intron	*MTSS1*
							ENSSSCG00000005977	+	intron	*TMEM65*
4	15761856	15761868	-	15761859	290	762	ENSSSCG00000005976	-	3'UTR	*NDUFB9*
4	15766810	15766810	-	15766810	4	4	---	---	intergenic	---
4	16359638	16359652	-	16359649	23	46	ENSSSCG00000024424	-	3'UTR	*FAM91A1*
4	16392045	16392045	-	16392045	3	3	ENSSSCG00000024424	-	ORF	*FAM91A1*
4	16468158	16468158	+	16468158	4	4	ENSSSCG00000005979	+	intron	*ANXA13*
4	16484867	16484867	+	16484867	3	3	ENSSSCG00000005979	+	3'UTR	*ANXA13*
4	16485072	16485076	+	16485073	29	78	ENSSSCG00000005979	+	3'UTR	*ANXA13*
4	16485266	16485269	+	16485266	3	6	ENSSSCG00000005979	+	3'UTR	*ANXA13*
4	16486057	16486057	+	16486057	3	3	XLOC_061049	+	predicted	---
4	16674237	16674237	+	16674237	3	3	XLOC_061053	+	predicted	---
4	16678000	16678000	+	16678000	5	5	---	---	intergenic	---
4	16724014	16724022	-	16724021	26	37	ENSSSCG00000005982	-	3'UTR	*WDYHV1*
4	16767314	16767314	+	16767314	3	3	ENSSSCG00000005983	+	ORF	*ATAD2*
4	16785856	16785856	+	16785856	3	3	ENSSSCG00000005983	+	ORF	*ATAD2*
4	16822752	16822758	+	16822756	7	22	ENSSSCG00000005983	+	3'UTR	*ATAD2*
4	16863200	16863200	-	16863200	4	4	XLOC_062126	-	predicted	---
4	16870496	16870496	+	16870496	4	4	XLOC_061055	+	predicted	---
4	16870788	16870795	+	16870795	11	17	XLOC_061055	+	predicted	---

4	16880686	16880686	-	16880686	4	4	---	---	intergenic	---
4	16902380	16902382	+	16902380	4	7	XLOC_061056	+	predicted	---
4	17113852	17113852	+	17113852	3	3	---	---	intergenic	---
4	17124931	17124931	+	17124931	4	4	---	---	intergenic	---
4	17130365	17130365	-	17130365	3	3	---	---	intergenic	---
4	17156478	17156478	-	17156478	6	6	---	---	intergenic	---
4	17158115	17158115	+	17158115	3	3	---	---	intergenic	---
4	17193645	17193645	-	17193645	6	6	---	---	intergenic	---
4	17229619	17229619	+	17229619	4	4	---	---	intergenic	---
4	17234752	17234759	-	17234753	31	53	XLOC_062131	-	predicted	---
4	17235181	17235181	-	17235181	4	4	XLOC_062131	-	predicted	---
4	17236666	17236668	-	17236668	11	25	ENSSSCG00000021004	-	3'UTR	*DERL1*
4	17236723	17236729	-	17236727	39	57	ENSSSCG00000021004	-	3'UTR	*DERL1*
4	18104291	18104291	-	18104291	3	3	---	---	intergenic	---
4	18218724	18218724	+	18218724	4	4	---	---	intergenic	---
4	18489934	18489936	+	18489935	5	13	---	---	intergenic	---
4	18560443	18560443	-	18560443	4	4	---	---	intergenic	---
4	18946638	18946641	-	18946641	13	29	---	---	intergenic	---
4	19221637	19221637	+	19221637	3	3	---	---	intergenic	---
4	19262867	19262867	+	19262867	3	3	XLOC_061071	+	predicted	---
4	19548296	19548296	+	19548296	3	3	ENSSSCG00000005994	+	3'UTR	*SNTB1*
4	19548394	19548394	+	19548394	3	3	ENSSSCG00000005994	+	3'UTR	*SNTB1*
4	19549001	19549016	+	19549001	10	16	ENSSSCG00000005994	+	3'UTR	*SNTB1*
4	19696722	19696733	+	19696724	9	24	ENSSSCG00000005996	+	3'UTR	*MRPL13*
4	19696794	19696799	+	19696795	12	30	ENSSSCG00000005996	+	3'UTR	*MRPL13*
4	19917681	19917702	-	19917681	5	13	ENSSSCG00000005997	-	intron	*COL14A1*

4	19989168	19989168	-	19989168	3	3	---	---	intergenic	---
4	20001489	20001492	-	20001489	15	18	ENSSSCG00000005998	-	intron	*DEPTOR*
4	20105980	20105980	-	20105980	3	3	ENSSSCG00000005998	-	intron	*DEPTOR*
4	20234185	20234185	+	20234185	5	5	---	---	intergenic	---
4	20284747	20284747	+	20284747	5	5	ENSSSCG00000006000	+	3'UTR	*TAF2*
4	20453081	20453088	+	20453081	17	57	ENSSSCG00000006001	+	3'UTR	*ENPP2*
4	20528345	20528345	-	20528345	3	3	ENSSSCG00000006002	-	intron	*NOV*
4	20579271	20579271	+	20579271	3	3	---	---	intergenic	---
4	20613478	20613485	-	20613483	8	18	ENSSSCG00000006003	-	3'UTR	*MAL2*
4	20763019	20763023	-	20763021	30	48	XLOC_062152	-	predicted	---
4	20785141	20785141	-	20785141	3	3	ENSSSCG00000029324	-	intron	---
4	21199465	21199465	-	21199465	3	3	---	---	intergenic	---
4	21288221	21288221	+	21288221	3	3	---	---	intergenic	---
4	21397531	21397531	+	21397531	4	4	---	---	intergenic	---
4	21893286	21893286	+	21893286	3	3	---	---	intergenic	---
4	21972247	21972247	+	21972247	5	5	---	---	intergenic	---
4	22128056	22128056	+	22128056	3	3	ENSSSCG00000006009	+	3'UTR	*EXT1*
4	22128124	22128129	+	22128124	17	24	ENSSSCG00000006009	+	3'UTR	*EXT1*
4	22223007	22223008	-	22223007	3	6	---	---	intergenic	---
4	22441045	22441045	-	22441045	3	3	XLOC_062158	-	predicted	---
4	22779673	22779673	+	22779673	8	8	---	---	intergenic	---
4	22786204	22786204	-	22786204	3	3	XLOC_062160	-	predicted	---
4	23059568	23059578	-	23059573	21	58	ENSSSCG00000006014	-	3'UTR	*RAD21*
4	23059981	23059981	-	23059981	3	3	ENSSSCG00000006014	-	3'UTR	*RAD21*
4	23060972	23060975	-	23060973	19	37	ENSSSCG00000006014	-	3'UTR	*RAD21*
4	23063997	23063997	-	23063997	5	5	---	---	intergenic	---

4	23072024	23072024	-	23072024	5	5	ENSSSCG00000006014	-	intron	*RAD21*
4	23434298	23434298	+	23434298	4	4	ENSSSCG00000006016	+	intron	*EIF3H*
4	23448891	23448893	+	23448892	14	21	XLOC_061091	+	predicted	---
4	24293023	24293023	+	24293023	3	3	---	---	intergenic	---
4	24652777	24652777	-	24652777	11	11	---	---	intergenic	---
4	24652820	24652821	+	24652821	7	10	---	---	intergenic	---
4	24656845	24656846	-	24656846	11	17	---	---	intergenic	---
4	24656964	24656974	-	24656973	16	77	---	---	intergenic	---
4	24657014	24657018	+	24657016	10	31	XLOC_061097	+	predicted	---
4	25053532	25053532	-	25053532	3	3	---	---	intergenic	---
4	25070672	25070672	-	25070672	10	10	---	---	intergenic	---
4	25453576	25453576	-	25453576	3	3	---	---	intergenic	---
4	25646910	25646910	+	25646910	15	15	---	---	intergenic	---
4	26008134	26008134	-	26008134	3	3	---	---	intergenic	---
4	26171046	26171049	+	26171049	7	11	---	---	intergenic	---
4	26866302	26866302	+	26866302	3	3	---	---	intergenic	---
4	26964586	26964586	+	26964586	9	9	---	---	intergenic	---
4	26996427	26996427	-	26996427	7	7	---	---	intergenic	---
4	27568150	27568150	-	27568150	3	3	---	---	intergenic	---
4	27876167	27876167	-	27876167	5	5	---	---	intergenic	---
4	28124971	28124971	-	28124971	3	3	---	---	intergenic	---
4	28271645	28271649	+	28271645	3	6	---	---	intergenic	---
4	28346637	28346637	+	28346637	8	8	---	---	intergenic	---
4	28898710	28898710	-	28898710	3	3	---	---	intergenic	---
4	28962248	28962257	+	28962249	29	43	---	---	intergenic	---
4	29010303	29010304	+	29010304	11	15	---	---	intergenic	---

4	29035447	29035451	+	29035448	14	40	---	---	intergenic	---
4	29671625	29671625	+	29671625	4	4	---	---	intergenic	---
4	29866423	29866423	-	29866423	6	6	---	---	intergenic	---
4	30217056	30217056	+	30217056	4	4	XLOC_061108	+	predicted	---
4	30228551	30228552	-	30228551	11	15	ENSSSCG00000006024	-	3'UTR	*EBAG9*
4	30228816	30228816	-	30228816	4	4	ENSSSCG00000006024	-	3'UTR	*EBAG9*
4	30229078	30229081	-	30229079	8	15	ENSSSCG00000006024	-	3'UTR	*EBAG9*
4	30266240	30266241	-	30266240	5	9	XLOC_062181	-	predicted	---
4	30891150	30891156	-	30891155	8	17	---	---	intergenic	---
4	30891246	30891246	-	30891246	3	3	---	---	intergenic	---
4	31049977	31049977	+	31049977	3	3	---	---	intergenic	---
4	31202173	31202173	-	31202173	8	8	---	---	intergenic	---
4	31379851	31379853	-	31379851	7	10	ENSSSCG00000006032	-	3'UTR	*EMC2*
4	31382051	31382056	-	31382051	9	16	ENSSSCG00000006032	-	intron	*EMC2*
4	31473453	31473453	-	31473453	3	3	ENSSSCG00000006032	-	intron	*EMC2*
4	31681568	31681568	-	31681568	3	3	---	---	intergenic	---
4	31682332	31682332	+	31682332	3	3	ENSSSCG00000006033	+	ORF	---
4	31731543	31731606	+	31731603	183	238	ENSSSCG00000006033	+	3'UTR	*EIF3E*
4	31736588	31736589	-	31736588	5	8	XLOC_062188	-	predicted	---
4	31753559	31753559	-	31753559	3	3	---	---	intergenic	---
4	31811701	31811701	+	31811701	5	5	ENSSSCG00000006034	+	intron	*RSPO2*
4	32673068	32673068	+	32673068	3	3	ENSSSCG00000006035	+	intron	*ANGPT1*
4	32839772	32839772	-	32839772	4	4	---	---	intergenic	---
4	33145364	33145364	+	33145364	3	3	XLOC_061123	+	predicted	---
4	33378076	33378076	-	33378076	5	5	ENSSSCG00000006037	-	3'UTR	---
4	33378195	33378196	-	33378195	3	6	ENSSSCG00000006037	-	3'UTR	---

4	33379682	33379682	-	33379682	3	3	ENSSSCG00000006037	-	3'UTR	---
4	33379722	33379742	-	33379723	36	118	ENSSSCG00000006037	-	3'UTR	---
4	33426810	33426824	-	33426816	9	19	ENSSSCG00000006037	-	ORF	---
4	33426987	33426987	-	33426987	4	4	ENSSSCG00000006037	-	ORF	---
4	33482756	33482756	-	33482756	5	5	---	---	intergenic	---
4	35011567	35011567	-	35011567	4	4	---	---	intergenic	---
4	35011595	35011598	+	35011595	4	10	---	---	intergenic	---
4	35359869	35359869	+	35359869	20	20	---	---	intergenic	---
4	35517012	35517012	+	35517012	4	4	ENSSSCG00000006039	+	3'UTR	*LRP12*
4	35520214	35520217	+	35520217	5	9	XLOC_061129	+	predicted	---
4	35544180	35544180	+	35544180	3	3	ENSSSCG00000006040	+	intron	*DPYS*
4	35548380	35548380	+	35548380	4	4	ENSSSCG00000006040	+	ORF	*DPYS*
4	35615955	35615955	+	35615955	4	4	ENSSSCG00000006040	+	3'UTR	*DPYS*
4	35616160	35616167	+	35616165	51	78	XLOC_061130	+	predicted	---
4	35669976	35669976	-	35669976	3	3	---	---	intergenic	---
4	35670118	35670118	-	35670118	3	3	---	---	intergenic	---
4	35705619	35705619	-	35705619	4	4	---	---	intergenic	---
4	35756703	35756703	-	35756703	3	3	XLOC_062196	-	predicted	---
4	36060043	36060043	-	36060043	3	3	---	---	intergenic	---
4	36277974	36277974	-	36277974	10	10	---	---	intergenic	---
4	36346268	36346270	-	36346270	4	7	---	---	intergenic	---
4	36427347	36427347	-	36427347	4	4	---	---	intergenic	---
4	36445545	36445545	-	36445545	9	9	ENSSSCG00000006048	-	3'UTR	*DCAF13*
4	36446415	36446424	-	36446416	5	11	ENSSSCG00000006048	-	3'UTR	*DCAF13*
4	36499710	36499710	+	36499710	13	13	ENSSSCG00000006049	+	3'UTR	*SLC25A32*
4	36499789	36499789	+	36499789	3	3	XLOC_061134	+	predicted	---

4	36653111	36653111	-	36653111	3	3	---	---	intergenic	---
4	36702599	36702600	+	36702599	4	7	---	---	intergenic	---
4	36882557	36882557	-	36882557	4	4	XLOC_062204	-	predicted	---
4	36886999	36886999	-	36886999	3	3	XLOC_062204	-	predicted	---
4	36887066	36887073	-	36887066	10	27	ENSSSCG00000006054	-	3'UTR	*ATP6V1C1*
4	36887156	36887156	-	36887156	4	4	ENSSSCG00000006054	-	3'UTR	*ATP6V1C1*
4	36887575	36887587	-	36887583	13	78	ENSSSCG00000006054	-	3'UTR	*ATP6V1C1*
4	36904410	36904410	-	36904410	3	3	ENSSSCG00000006054	-	ORF	*ATP6V1C1*
4	36934235	36934239	-	36934235	5	14	---	---	intergenic	---
4	36937897	36937901	+	36937901	22	29	---	---	intergenic	---
4	36995463	36995463	-	36995463	3	3	XLOC_062205	-	predicted	---
4	37095518	37095518	+	37095518	9	9	ENSSSCG00000023873	+	intron	*AZIN1*
4	37107495	37107495	+	37107495	4	4	ENSSSCG00000023873	+	3'UTR	*AZIN1*
4	37107927	37107927	+	37107927	8	8	ENSSSCG00000023873	+	3'UTR	*AZIN1*
4	37109555	37109568	+	37109568	21	37	ENSSSCG00000023873	+	3'UTR	*AZIN1*
4	37109698	37109698	+	37109698	3	3	ENSSSCG00000023873	+	3'UTR	*AZIN1*
4	37117793	37117793	-	37117793	3	3	---	---	intergenic	---
4	37183807	37183807	-	37183807	3	3	---	---	intergenic	---
4	37237258	37237258	+	37237258	3	3	---	---	intergenic	---
4	37305963	37305963	+	37305963	3	3	---	---	intergenic	---
4	37307577	37307577	+	37307577	3	3	---	---	intergenic	---
4	37337137	37337157	-	37337139	55	131	XLOC_062208	-	predicted	---
4	37443344	37443349	-	37443349	11	28	ENSSSCG00000027480	-	3'UTR	*KLF10*
4	37679205	37679205	-	37679205	4	4	XLOC_062211	-	predicted	---
4	37745021	37745038	-	37745038	14	27	ENSSSCG00000022479	-	3'UTR	*UBR5*
4	38264849	38264851	+	38264849	4	7	ENSSSCG00000006059	+	3'UTR	*NCALD*

4	38546151	38546151	+	38546151	3	3	---	---	intergenic	---
4	38605654	38605654	-	38605654	3	3	---	---	intergenic	---
4	38670485	38670485	-	38670485	5	5	---	---	intergenic	---
4	38724216	38724225	+	38724224	7	17	ENSSSCG00000006061	+	3'UTR	*ZNF706*
4	38724296	38724303	+	38724297	11	18	ENSSSCG00000006061	+	3'UTR	*ZNF706*
4	38724407	38724422	+	38724409	29	115	ENSSSCG00000006061	+	3'UTR	*ZNF706*
4	38738069	38738069	+	38738069	5	5	---	---	intergenic	---
4	38781850	38781850	+	38781850	3	3	XLOC_061145	+	predicted	---
4	38894790	38894790	+	38894790	4	4	---	---	intergenic	---
4	38896596	38896596	-	38896596	3	3	---	---	intergenic	---
4	38940624	38940663	+	38940628	35	66	ENSSSCG00000006062	+	3'UTR	*YWHAZ*
4	38941574	38941587	+	38941579	39	73	XLOC_061149	+	predicted	---
4	38942543	38942544	+	38942544	30	35	XLOC_061149	+	predicted	---
4	38942586	38942586	+	38942586	8	8	XLOC_061149	+	predicted	---
4	38942688	38942696	+	38942695	42	65	XLOC_061149	+	predicted	---
4	38942903	38942903	+	38942903	12	12	XLOC_061149	+	predicted	---
4	39059009	39059009	+	39059009	3	3	---	---	intergenic	---
4	39133738	39133738	-	39133738	14	14	---	---	intergenic	---
4	39137549	39137549	+	39137549	3	3	---	---	intergenic	---
4	39140877	39140877	+	39140877	4	4	---	---	intergenic	---
4	39143381	39143382	+	39143382	18	21	---	---	intergenic	---
4	39148536	39148536	+	39148536	6	6	ENSSSCG00000006063	+	intron	---
4	39152242	39152242	+	39152242	3	3	ENSSSCG00000006063	+	3'UTR	---
4	39152363	39152363	+	39152363	3	3	XLOC_061150	+	predicted	---
4	39152534	39152544	+	39152542	237	343	XLOC_061150	+	predicted	---
4	39368004	39368004	+	39368004	3	3	XLOC_061152	+	predicted	---

4	39368106	39368108	+	39368108	12	17	XLOC_061152	+	predicted	---
4	39432149	39432149	+	39432149	3	3	---	---	intergenic	---
4	39466441	39466441	-	39466441	3	3	---	---	intergenic	---
4	39553718	39553718	+	39553718	6	6	---	---	intergenic	---
4	39632327	39632327	+	39632327	3	3	ENSSSCG00000006066	+	intron	*RNF19*
4	39645959	39645959	+	39645959	5	5	ENSSSCG00000006066	+	ORF	*RNF19*
4	39645999	39645999	+	39645999	4	4	ENSSSCG00000006066	+	3'UTR	*RNF19*
4	39647463	39647479	+	39647468	16	35	ENSSSCG00000006066	+	3'UTR	*RNF19*
4	39668768	39668772	-	39668768	10	23	ENSSSCG00000006067	-	3'UTR	---
4	39669578	39669582	-	39669578	13	22	ENSSSCG00000006067	-	3'UTR	---
4	39670168	39670168	-	39670168	3	3	ENSSSCG00000006067	-	ORF	---
4	39723908	39723908	-	39723908	6	6	ENSSSCG00000027587	-	intron	---
4	39736287	39736287	-	39736287	7	7	ENSSSCG00000027587	-	ORF	---
4	39765499	39765515	-	39765505	100	170	ENSSSCG00000021180	-	3'UTR	*POLR2K*
4	39787295	39787295	+	39787295	4	4	XLOC_061158	+	predicted	---
4	39799741	39799742	-	39799741	6	10	XLOC_062221	-	predicted	---
4	39958658	39958658	+	39958658	3	3	---	---	intergenic	---
4	39958924	39958924	+	39958924	4	4	XLOC_061159	+	predicted	---
4	40005755	40005769	+	40005756	95	230	ENSSSCG00000006070	+	3'UTR	*COX6C*
4	40006552	40006552	-	40006552	3	3	ENSSSCG00000006071	-	3'UTR	*VPS13B*
4	40072158	40072159	-	40072159	12	16	ENSSSCG00000006071	-	intron	*VPS13B*
4	40244546	40244546	-	40244546	8	8	---	---	intergenic	---
4	40283332	40283332	-	40283332	4	4	---	---	intergenic	---
4	40294303	40294303	+	40294303	3	3	---	---	intergenic	---
4	40735538	40735538	+	40735538	8	8	---	---	intergenic	---
4	40845137	40845138	-	40845137	41	65	---	---	intergenic	---

4	40862187	40862187	-	40862187	7	7	XLOC_062225	-	predicted	---
4	40934393	40934393	+	40934393	3	3	XLOC_061167	+	predicted	---
4	40946354	40946354	-	40946354	3	3	---	---	intergenic	---
4	41021903	41021903	+	41021903	3	3	ENSSSCG00000006074	+	intron	*STK3*
4	41310037	41310038	-	41310038	11	15	---	---	intergenic	---
4	41330058	41330059	+	41330058	13	16	XLOC_061169	+	predicted	---
4	41451456	41451458	+	41451457	17	26	XLOC_061172	+	predicted	---
4	41668276	41668276	-	41668276	3	3	XLOC_062233	-	predicted	---
4	41706221	41706221	+	41706221	4	4	ENSSSCG00000006081	+	ORF	*RPL30*
4	41708318	41708325	+	41708325	13	29	ENSSSCG00000006081	+	ORF	*RPL30*
4	41709149	41709166	+	41709154	1108	1257	ENSSSCG00000006081	+	3'UTR	*RPL30*
4	41732770	41732779	-	41732779	30	33	ENSSSCG00000006078	-	intron	---
4	41765314	41765314	-	41765314	3	3	ENSSSCG00000006078	-	intron	---
4	41817591	41817608	+	41817601	135	339	ENSSSCG00000006079	+	3'UTR	*HRSP12*
4	41821308	41821308	-	41821308	3	3	XLOC_062235	-	predicted	---
4	41821484	41821484	+	41821484	6	6	XLOC_061176	+	predicted	---
4	41843435	41843435	+	41843435	3	3	---	---	intergenic	---
4	41892444	41892446	-	41892445	7	14	---	---	intergenic	---
4	41976819	41976819	-	41976819	3	3	XLOC_062238	-	predicted	---
4	41977260	41977276	-	41977269	108	266	ENSSSCG00000006083	-	3'UTR	*LAPTM4B*
4	41977879	41977882	-	41977879	3	6	ENSSSCG00000006083	-	3'UTR	*LAPTM4B*
4	42022611	42022611	-	42022611	6	6	ENSSSCG00000006083	-	intron	*LAPTM4B*
4	42097773	42097773	-	42097773	6	6	XLOC_062239	-	predicted	---
4	42099143	42099154	-	42099151	26	66	XLOC_062239	-	predicted	---
4	42100272	42100277	-	42100277	16	20	XLOC_062239	-	predicted	---
4	42102083	42102083	-	42102083	3	3	ENSSSCG00000006084	-	ORF	*MTDH*

4	42152660	42152660	-	42152660	7	7	ENSSSCG00000006084	-	intron	*MTDH*
4	42169540	42169542	+	42169540	3	6	XLOC_061177	+	predicted	---
4	42170008	42170012	-	42170008	4	7	---	---	intergenic	---
4	42173440	42173440	+	42173440	3	3	---	---	intergenic	---
4	42283961	42283961	+	42283961	3	3	---	---	intergenic	---
4	42296387	42296387	+	42296387	6	6	---	---	intergenic	---
4	42456334	42456334	+	42456334	5	5	---	---	intergenic	---
4	42482419	42482423	-	42482420	11	17	---	---	intergenic	---
4	43097819	43097819	-	43097819	3	3	---	---	intergenic	---
4	43121678	43121678	-	43121678	3	3	---	---	intergenic	---
4	43122876	43122886	-	43122884	47	152	ENSSSCG00000006088	-	3'UTR	*SDC2*
4	43123915	43123944	-	43123923	29	112	ENSSSCG00000006088	-	3'UTR	*SDC2*
4	43148481	43148481	+	43148481	3	3	---	---	intergenic	---
4	43357317	43357317	-	43357317	4	4	---	---	intergenic	---
4	43428465	43428466	-	43428466	4	7	---	---	intergenic	---
4	43478298	43478309	-	43478299	32	51	XLOC_062243	-	predicted	---
4	43567842	43567842	+	43567842	4	4	ENSSSCG00000006090	+	intron	*MTERF3*
4	43578507	43578509	+	43578507	14	17	XLOC_061192	+	predicted	---
4	43584375	43584375	+	43584375	4	4	---	---	intergenic	---
4	43627549	43627549	-	43627549	4	4	XLOC_062245	-	predicted	---
4	43631690	43631716	-	43631709	61	115	XLOC_062245	-	predicted	---
4	43631778	43631778	-	43631778	4	4	XLOC_062245	-	predicted	---
4	43754583	43754583	-	43754583	3	3	---	---	intergenic	---
4	43799988	43799988	+	43799988	3	3	---	---	intergenic	---
4	44266273	44266273	+	44266273	5	5	---	---	intergenic	---
4	44322079	44322079	+	44322079	9	9	---	---	intergenic	---

4	44339794	44339794	-	44339794	5	5	---	---	intergenic	---
4	44709160	44709161	-	44709161	19	29	ENSSSCG00000006092	-	3'UTR	*C8orf37*
4	44722434	44722434	-	44722434	7	7	ENSSSCG00000006092	-	intron	*C8orf37*
4	44759394	44759397	-	44759394	4	7	---	---	intergenic	---
4	44875410	44875416	-	44875412	10	25	ENSSSCG00000006093	-	3'UTR	*NDUFAF6*
4	44915825	44915825	+	44915825	3	3	---	---	intergenic	---
4	45015762	45015762	+	45015762	4	4	ENSSSCG00000023609	+	3'UTR	*TP53INP1*
4	45017322	45017329	+	45017329	6	12	ENSSSCG00000023609	+	3'UTR	*TP53INP1*
4	45019064	45019068	+	45019065	35	56	ENSSSCG00000023609	+	3'UTR	*TP53INP1*
4	45123598	45123598	+	45123598	7	7	ENSSSCG00000006095	+	intron	*CCNE2*
4	45139306	45139306	-	45139306	7	7	ENSSSCG00000006096	-	3'UTR	*INTS8*
4	45464490	45464490	-	45464490	3	3	---	---	intergenic	---
4	45557936	45557936	-	45557936	3	3	ENSSSCG00000023492	-	intron	---
4	45573276	45573276	-	45573276	8	8	ENSSSCG00000023492	-	intron	---
4	45602666	45602666	+	45602666	3	3	ENSSSCG00000006100	+	intron	*KIAA1429*
4	45685648	45685652	-	45685650	46	79	---	---	intergenic	---
4	45839539	45839539	+	45839539	3	3	---	---	intergenic	---
4	45944236	45944236	-	45944236	8	8	---	---	intergenic	---
4	46038432	46038432	-	46038432	3	3	---	---	intergenic	---
4	46108488	46108488	-	46108488	3	3	---	---	intergenic	---
4	46192764	46192764	-	46192764	7	7	XLOC_062261	-	predicted	---
4	46257594	46257594	+	46257594	4	4	XLOC_061212	+	predicted	---
4	46282159	46282159	-	46282159	3	3	---	---	intergenic	---
4	46297658	46297658	-	46297658	4	4	ENSSSCG00000006108	-	intron	---
4	46382623	46382624	+	46382623	4	8	ENSSSCG00000006109	+	intron	*FAM92A1*
4	46409024	46409024	+	46409024	3	3	XLOC_061213	+	predicted	---

4	46705027	46705029	+	46705028	30	62	---	---	intergenic	---
4	46805940	46805940	+	46805940	4	4	---	---	intergenic	---
4	46823406	46823406	-	46823406	3	3	XLOC_062267	-	predicted	---
4	46823459	46823465	-	46823465	5	8	XLOC_062267	-	predicted	---
4	47008563	47008563	+	47008563	3	3	---	---	intergenic	---
4	47056411	47056416	-	47056411	4	7	XLOC_062269	-	predicted	---
4	47140143	47140143	+	47140143	3	3	---	---	intergenic	---
4	47779372	47779376	-	47779375	16	38	---	---	intergenic	---
4	48096950	48096950	-	48096950	5	5	---	---	intergenic	---
4	48172693	48172693	+	48172693	5	5	XLOC_061216	+	predicted	---
4	48203372	48203372	+	48203372	3	3	---	---	intergenic	---
4	48563686	48563686	+	48563686	13	13	---	---	intergenic	---
4	48805597	48805597	+	48805597	5	5	---	---	intergenic	---
4	49400118	49400118	-	49400118	7	7	ENSSSCG00000006120	-	3'UTR	*OTUD6B*
4	49406857	49406857	-	49406857	4	4	ENSSSCG00000006120	-	intron	*OTUD6B*
4	49410879	49410881	-	49410880	4	10	ENSSSCG00000006120	-	ORF	*OTUD6B*
4	49415181	49415181	+	49415181	6	6	XLOC_061220	+	predicted	---
4	49415302	49415302	+	49415302	3	3	XLOC_061220	+	predicted	---
4	49416073	49416073	+	49416073	3	3	XLOC_061220	+	predicted	---
4	49416841	49416841	+	49416841	10	10	XLOC_061220	+	predicted	---
4	49549165	49549165	+	49549165	3	3	---	---	intergenic	---
4	49879609	49879609	+	49879609	3	3	---	---	intergenic	---
4	50019953	50019953	+	50019953	3	3	---	---	intergenic	---
4	50604818	50604818	+	50604818	3	3	---	---	intergenic	---
4	50876029	50876030	-	50876029	4	8	---	---	intergenic	---
4	51207727	51207728	-	51207727	10	18	ENSSSCG00000006126	-	3'UTR	*DECR1*

4	51207825	51207825	-	51207825	17	17	ENSSSCG00000006126	-	3'UTR	*DECR1*
4	51208444	51208446	-	51208444	22	25	ENSSSCG00000006126	-	3'UTR	*DECR1*
4	51288815	51288815	+	51288815	4	4	---	---	intergenic	---
4	51316078	51316078	+	51316078	7	7	XLOC_061224	+	predicted	---
4	51350651	51350659	-	51350651	4	7	ENSSSCG00000021184	-	3'UTR	*OSGIN2*
4	51352056	51352060	-	51352060	6	10	ENSSSCG00000021184	-	ORF	---
4	51491235	51491235	-	51491235	3	3	ENSSSCG00000024096	-	3'UTR	*RIPK2*
4	51963468	51963468	+	51963468	3	3	---	---	intergenic	---
4	53396170	53396170	-	53396170	7	7	---	---	intergenic	---
4	53987758	53987758	+	53987758	10	10	---	---	intergenic	---
4	54034228	54034229	-	54034229	15	21	---	---	intergenic	---
4	54092796	54092796	+	54092796	4	4	---	---	intergenic	---
4	54132174	54132174	-	54132174	3	3	---	---	intergenic	---
4	54422875	54422878	-	54422875	5	9	---	---	intergenic	---
4	54570939	54570939	-	54570939	3	3	XLOC_062291	-	predicted	---
4	54639735	54639735	+	54639735	6	6	---	---	intergenic	---
4	54693390	54693390	+	54693390	9	9	---	---	intergenic	---
4	54973101	54973102	+	54973102	5	8	---	---	intergenic	---
4	55170153	55170153	+	55170153	5	5	ENSSSCG00000006134	+	intron	*CNGB3*
4	55192150	55192156	-	55192156	6	17	ENSSSCG00000006135	-	3'UTR	*CPNE3*
4	55192231	55192236	-	55192235	6	14	ENSSSCG00000006135	-	3'UTR	*CPNE3*
4	55193484	55193484	-	55193484	3	3	ENSSSCG00000006135	-	3'UTR	*CPNE3*
4	55298660	55298661	+	55298660	16	28	ENSSSCG00000006136	+	3'UTR	*RMDN1*
4	55304277	55304283	-	55304283	6	9	XLOC_062294	-	predicted	---
4	55304553	55304553	-	55304553	5	5	XLOC_062294	-	predicted	---
4	55313918	55313918	-	55313918	3	3	ENSSSCG00000006137	-	intron	*WWP1*

4	55364524	55364524	-	55364524	5	5	ENSSSCG00000006137	-	ORF	*WWP1*
4	55660768	55660768	-	55660768	3	3	ENSSSCG00000006138	-	3'UTR	*ATP6V0D2*
4	55807471	55807471	+	55807471	4	4	---	---	intergenic	---
4	55909047	55909047	+	55909047	3	3	---	---	intergenic	---
4	56145309	56145309	+	56145309	4	4	---	---	intergenic	---
4	56157607	56157607	-	56157607	4	4	ENSSSCG00000006140	-	3'UTR	*CA2*
4	56158113	56158113	-	56158113	3	3	ENSSSCG00000006140	-	3'UTR	*CA2*
4	56190401	56190410	-	56190404	87	115	ENSSSCG00000006141	-	3'UTR	*CA3*
4	56190527	56190527	-	56190527	3	3	ENSSSCG00000006141	-	3'UTR	*CA3*
4	56191079	56191113	-	56191079	5	8	ENSSSCG00000006141	-	3'UTR	*CA3*
4	56493464	56493472	+	56493466	23	43	ENSSSCG00000006144	+	3'UTR	*C8orf59*
4	56554064	56554064	-	56554064	5	5	ENSSSCG00000020729	-	intron	---
							ENSSSCG00000006146	-	ORF	---
4	56769034	56769035	+	56769034	13	17	ENSSSCG00000006148	+	3'UTR	---
4	57216707	57216708	+	57216707	3	6	---	---	intergenic	---
4	57242267	57242267	+	57242267	3	3	---	---	intergenic	---
4	57322170	57322170	-	57322170	4	4	---	---	intergenic	---
4	57626754	57626754	-	57626754	5	5	ENSSSCG00000027870	-	intron	---
4	57741715	57741715	+	57741715	3	3	---	---	intergenic	---
4	57820818	57820818	+	57820818	4	4	---	---	intergenic	---
4	57876677	57876677	+	57876677	5	5	---	---	intergenic	---
4	58828885	58828885	-	58828885	7	7	---	---	intergenic	---
4	59238683	59238683	-	59238683	4	4	XLOC_062306	-	predicted	---
4	59238694	59238855	+	59238854	187	915	---	---	intergenic	---
4	59238742	59238742	-	59238742	3	3	XLOC_062306	-	predicted	---
4	59238852	59238878	-	59238878	5	12	XLOC_062306	-	predicted	---

4	59238873	59238953	+	59238893	291	624	---	---	intergenic	---
4	59239000	59239094	+	59239047	92	186	---	---	intergenic	---
4	59239144	59239150	+	59239149	71	124	---	---	intergenic	---
4	59239197	59239216	+	59239199	7	14	---	---	intergenic	---
4	59239260	59239305	-	59239267	16	175	XLOC_062306	-	predicted	---
4	59239322	59239339	+	59239339	206	240	---	---	intergenic	---
4	59239380	59239585	-	59239566	82	539	XLOC_062306	-	predicted	---
4	59239406	59239520	+	59239460	267	463	---	---	intergenic	---
4	59239550	59239619	+	59239615	1640	3179	---	---	intergenic	---
4	59239595	59239622	-	59239611	344	995	XLOC_062306	-	predicted	---
4	59239660	59239710	-	59239673	28	206	XLOC_062306	-	predicted	---
4	59239785	59239798	-	59239798	67	115	---	---	intergenic	---
4	59239829	59239864	+	59239863	696	836	---	---	intergenic	---
4	59239838	59239951	-	59239878	30	152	XLOC_062306	-	predicted	---
4	59239906	59240009	+	59239949	534	794	---	---	intergenic	---
4	59239981	59240007	-	59240007	6	23	XLOC_062306	-	predicted	---
4	59240018	59240070	+	59240052	1408	1633	---	---	intergenic	---
4	59267693	59267693	+	59267693	4	4	---	---	intergenic	---
4	59437815	59437815	+	59437815	3	3	---	---	intergenic	---
4	59522086	59522086	+	59522086	3	3	---	---	intergenic	---
4	59595540	59595545	-	59595544	24	44	---	---	intergenic	---
4	59784324	59784324	+	59784324	6	6	---	---	intergenic	---
4	59833817	59833817	+	59833817	4	4	---	---	intergenic	---
4	60217978	60217978	-	60217978	3	3	---	---	intergenic	---
4	60306425	60306429	-	60306426	51	125	ENSSSCG00000006153	-	3'UTR	*FABP5*
4	60634310	60634310	+	60634310	3	3	XLOC_061247	+	predicted	---

4	60870947	60870948	+	60870948	9	13	---	---	intergenic	---
4	61300320	61300320	+	61300320	3	3	ENSSSCG00000006155	+	3'UTR	*ZBTB10*
4	61304766	61304769	+	61304768	4	10	ENSSSCG00000006155	+	3'UTR	*ZBTB10*
4	61581854	61581854	+	61581854	3	3	---	---	intergenic	---
4	61600177	61600178	+	61600177	3	6	---	---	intergenic	---
4	61718911	61718911	-	61718911	7	7	---	---	intergenic	---
4	61813276	61813277	-	61813277	7	11	XLOC_062315	-	predicted	---
4	61813380	61813380	-	61813380	4	4	---	---	intergenic	---
4	61912454	61912454	-	61912454	3	3	XLOC_062316	-	predicted	---
4	61913584	61913584	-	61913584	18	18	ENSSSCG00000006156	-	3'UTR	*TPD52*
4	61914521	61914521	-	61914521	10	10	ENSSSCG00000006156	-	3'UTR	*TPD52*
4	62042021	62042021	+	62042021	4	4	---	---	intergenic	---
4	62204717	62204717	-	62204717	4	4	ENSSSCG00000006160	-	intron	*STMN2*
4	62258942	62258942	+	62258942	3	3	---	---	intergenic	---
4	62613491	62613491	-	62613491	4	4	XLOC_062320	-	predicted	---
4	62892138	62892138	+	62892138	12	12	ENSSSCG00000006161	+	intron	*IL7*
4	62949082	62949083	+	62949083	8	13	ENSSSCG00000006161	+	3'UTR	*IL7*
4	62965626	62965626	-	62965626	3	3	XLOC_062322	-	predicted	---
4	63073141	63073141	-	63073141	3	3	---	---	intergenic	---
4	63078480	63078480	-	63078480	5	5	ENSSSCG00000006163	-	3'UTR	*PKIA*
4	63916422	63916434	+	63916426	65	191	ENSSSCG00000006166	+	3'UTR	*NDUFB8*
4	63951401	63951409	+	63951401	3	6	XLOC_061263	+	predicted	---
4	64164801	64164801	+	64164801	13	13	---	---	intergenic	---
4	64683976	64683976	+	64683976	6	6	---	---	intergenic	---
4	64718523	64718528	+	64718528	7	15	---	---	intergenic	---
4	64728538	64728538	+	64728538	3	3	ENSSSCG00000006168	+	intron	*PEX2*

4	64817750	64817750	-	64817750	4	4	---	---	intergenic	---
4	65405673	65405673	+	65405673	7	7	---	---	intergenic	---
4	65424919	65424919	-	65424919	3	3	---	---	intergenic	---
4	65574711	65574711	-	65574711	3	3	---	---	intergenic	---
4	65729152	65729152	-	65729152	3	3	---	---	intergenic	---
4	65834483	65834484	+	65834484	5	9	---	---	intergenic	---
4	66075620	66075620	+	66075620	3	3	---	---	intergenic	---
4	66110071	66110071	+	66110071	3	3	---	---	intergenic	---
4	66612163	66612163	-	66612163	7	7	XLOC_062335	-	predicted	---
4	66886606	66886606	+	66886606	3	3	XLOC_061271	+	predicted	---
4	67179221	67179221	+	67179221	3	3	---	---	intergenic	---
4	67385827	67385827	+	67385827	3	3	ENSSSCG00000006174	+	intron	*JPH1*
4	67631663	67631669	-	67631669	18	29	ENSSSCG00000006175	-	intron	*LY96*
4	67693423	67693423	+	67693423	4	4	ENSSSCG00000006176	+	3'UTR	*TCEB1*
4	67693636	67693643	+	67693636	10	27	ENSSSCG00000006176	+	3'UTR	*TCEB1*
4	67693975	67693975	+	67693975	6	6	ENSSSCG00000006176	+	3'UTR	*TCEB1*
4	67694189	67694189	+	67694189	3	3	ENSSSCG00000006176	+	3'UTR	*TCEB1*
4	67695095	67695095	+	67695095	7	7	ENSSSCG00000006176	+	3'UTR	*TCEB1*
4	67789846	67789848	+	67789847	9	15	ENSSSCG00000006177	+	3'UTR	*UBE2W*
4	67790290	67790290	+	67790290	5	5	ENSSSCG00000006177	+	3'UTR	*UBE2W*
4	67790424	67790425	+	67790425	6	10	ENSSSCG00000006177	+	3'UTR	*UBE2W*
4	67790689	67790689	+	67790689	3	3	XLOC_061274	+	predicted	---
4	67795355	67795355	+	67795355	3	3	XLOC_061274	+	predicted	---
4	67917641	67917641	-	67917641	4	4	---	---	intergenic	---
4	68023292	68023292	+	68023292	4	4	XLOC_061275	+	predicted	---
4	68341837	68341839	-	68341838	5	12	XLOC_062346	-	predicted	---

4	68353625	68353633	-	68353625	53	70	ENSSSCG00000006184	-	intron	*TERF1*
4	68360193	68360193	-	68360193	3	3	ENSSSCG00000006184	-	intron	*TERF1*
4	68367670	68367670	-	68367670	3	3	ENSSSCG00000006184	-	ORF	*TERF1*
4	68403441	68403441	+	68403441	3	3	---	---	intergenic	---
4	69137435	69137435	-	69137435	15	15	---	---	intergenic	---
4	69392008	69392008	+	69392008	5	5	---	---	intergenic	---
4	69458333	69458333	-	69458333	3	3	---	---	intergenic	---
4	69513759	69513759	-	69513759	3	3	---	---	intergenic	---
4	69523616	69523616	+	69523616	3	3	---	---	intergenic	---
4	69866606	69866623	-	69866606	6	10	---	---	intergenic	---
4	69866639	69866645	+	69866644	18	31	---	---	intergenic	---
4	70309650	70309650	-	70309650	7	7	XLOC_062353	-	predicted	---
4	70355496	70355496	+	70355496	3	3	ENSSSCG00000006192	+	ORF	*LACTB2*
4	70363690	70363697	+	70363697	13	20	ENSSSCG00000006192	+	intron	*LACTB2*
4	70371527	70371527	+	70371527	8	8	ENSSSCG00000006192	+	ORF	*LACTB2*
4	70371645	70371651	+	70371646	6	21	ENSSSCG00000006192	+	ORF	*LACTB2*
4	70378586	70378592	+	70378590	63	93	ENSSSCG00000006192	+	3'UTR	*LACTB2*
4	70378853	70378853	+	70378853	4	4	ENSSSCG00000006192	+	3'UTR	*LACTB2*
4	70381647	70381648	-	70381647	6	10	---	---	intergenic	---
4	70381671	70381671	+	70381671	4	4	---	---	intergenic	---
4	70429534	70429554	+	70429534	8	11	ENSSSCG00000006193	+	3'UTR	*TRAM1*
4	70429612	70429622	+	70429613	36	108	ENSSSCG00000006193	+	3'UTR	*TRAM1*
4	70430074	70430074	+	70430074	3	3	XLOC_061287	+	predicted	---
4	70430998	70431008	+	70430999	53	101	XLOC_061287	+	predicted	---
4	70771839	70771839	+	70771839	4	4	---	---	intergenic	---
4	71005885	71005885	+	71005885	3	3	---	---	intergenic	---

4	71013729	71013729	+	71013729	4	4	XLOC_061293	+	predicted	---
4	71249641	71249641	+	71249641	4	4	XLOC_061293	+	predicted	---
4	71262319	71262326	-	71262319	4	7	XLOC_062360	-	predicted	---
4	71263118	71263118	+	71263118	3	3	---	---	intergenic	---
4	71563115	71563115	+	71563115	5	5	---	---	intergenic	---
4	71565872	71565878	-	71565878	5	8	XLOC_062361	-	predicted	---
4	71905812	71905812	-	71905812	3	3	---	---	intergenic	---
4	71993736	71993736	+	71993736	4	4	---	---	intergenic	---
4	72031058	72031058	-	72031058	3	3	XLOC_062362	-	predicted	---
4	72302189	72302193	-	72302189	3	6	---	---	intergenic	---
4	72393863	72393863	-	72393863	3	3	XLOC_062363	-	predicted	---
4	72402069	72402069	-	72402069	3	3	XLOC_062363	-	predicted	---
4	72946146	72946146	+	72946146	4	4	---	---	intergenic	---
4	73134481	73134481	+	73134481	5	5	ENSSSCG00000006200	+	intron	*CPA6*
4	73614072	73614072	+	73614072	6	6	ENSSSCG00000006201	+	intron	*ARFGEF1*
4	73673164	73673166	+	73673166	6	9	ENSSSCG00000006201	+	3'UTR	*ARFGEF1*
4	73674451	73674464	+	73674459	12	22	ENSSSCG00000006201	+	3'UTR	*ARFGEF1*
4	73708279	73708281	-	73708281	6	9	---	---	intergenic	---
4	73718211	73718212	-	73718211	4	7	ENSSSCG00000006202	-	intron	*CSPP1*
4	73731874	73731874	-	73731874	3	3	ENSSSCG00000006202	-	intron	*CSPP1*
4	73788428	73788428	+	73788428	3	3	---	---	intergenic	---
4	73818928	73818934	+	73818928	30	41	ENSSSCG00000006203	+	3'UTR	*COPS5*
4	73844931	73844931	+	73844931	3	3	ENSSSCG00000006204	+	ORF	*PPP1R42*
4	73859951	73859951	+	73859951	7	7	ENSSSCG00000006204	+	intron	*PPP1R42*
4	73875350	73875362	+	73875357	19	44	ENSSSCG00000006204	+	intron	*PPP1R42*
4	73875552	73875554	+	73875554	8	15	XLOC_061299	+	predicted	---

4	73908145	73908145	+	73908145	8	8	XLOC_061301	+	predicted	---
4	73959628	73959628	-	73959628	3	3	ENSSSCG00000006206	-	3'UTR	---
4	74272981	74272981	+	74272981	3	3	ENSSSCG00000024133	+	3'UTR	*VCPIP1*
4	74273042	74273042	+	74273042	3	3	ENSSSCG00000024133	+	3'UTR	*VCPIP1*
4	74274268	74274268	+	74274268	4	4	ENSSSCG00000024133	+	3'UTR	*VCPIP1*
4	74277037	74277038	+	74277037	3	6	---	---	intergenic	---
4	74326966	74326973	+	74326966	6	19	XLOC_061308	+	predicted	---
4	74419494	74419499	-	74419499	11	23	ENSSSCG00000006213	-	3'UTR	*ADHFE1*
4	74419849	74419849	-	74419849	3	3	ENSSSCG00000006213	-	3'UTR	*ADHFE1*
							ENSSSCG00000006213	-	intron	*ADHFE1*
4	74453949	74453949	-	74453949	3	3	XLOC_062372	-	predicted	---
4	74453996	74454000	-	74453996	14	18	XLOC_062372	-	predicted	---
4	74716348	74716348	+	74716348	3	3	---	---	intergenic	---
4	74724227	74724228	-	74724228	16	23	XLOC_062374	-	predicted	---
4	74724315	74724315	-	74724315	5	5	XLOC_062374	-	predicted	---
4	74725050	74725058	-	74725058	8	15	XLOC_062374	-	predicted	---
4	74725215	74725231	-	74725222	148	372	ENSSSCG00000006217	-	3'UTR	*DNAJC5B*
4	74725352	74725361	-	74725354	21	68	ENSSSCG00000006217	-	3'UTR	*DNAJC5B*
4	74738729	74738729	-	74738729	6	6	ENSSSCG00000006217	-	intron	*DNAJC5B*
4	74885202	74885202	-	74885202	3	3	---	---	intergenic	---
4	74922021	74922021	+	74922021	3	3	---	---	intergenic	---
4	74978970	74978970	-	74978970	3	3	---	---	intergenic	---
4	75010120	75010120	+	75010120	3	3	---	---	intergenic	---
4	75055302	75055310	+	75055305	11	29	ENSSSCG00000006218	+	3'UTR	*PDE7A*
4	75061579	75061589	-	75061582	33	104	ENSSSCG00000006219	-	3'UTR	*MTFR1*
4	75062666	75062666	-	75062666	4	4	ENSSSCG00000006219	-	3'UTR	*MTFR1*

4	75062960	75062972	-	75062960	3	6	ENSSSCG00000006219	-	3'UTR	*MTFR1*
4	75082726	75082726	-	75082726	6	6	ENSSSCG00000006219	-	intron	*MTFR1*
4	75159257	75159257	+	75159257	3	3	ENSSSCG00000006220	+	intron	*ARMC1*
4	75448494	75448494	-	75448494	4	4	---	---	intergenic	---
4	75977543	75977543	+	75977543	4	4	XLOC_061316	+	predicted	---
4	75978021	75978021	+	75978021	4	4	XLOC_061316	+	predicted	---
4	75978522	75978535	+	75978522	12	42	XLOC_061316	+	predicted	---
4	76001242	76001242	+	76001242	3	3	---	---	intergenic	---
4	76095869	76095869	-	76095869	4	4	---	---	intergenic	---
4	76335149	76335149	-	76335149	5	5	---	---	intergenic	---
4	76541009	76541009	+	76541009	4	4	---	---	intergenic	---
4	76615804	76615804	-	76615804	3	3	---	---	intergenic	---
4	76670116	76670116	-	76670116	5	5	---	---	intergenic	---
4	76719255	76719256	-	76719256	24	29	---	---	intergenic	---
4	77044674	77044674	-	77044674	3	3	---	---	intergenic	---
4	77180121	77180121	+	77180121	3	3	---	---	intergenic	---
4	77193837	77193840	-	77193839	31	51	---	---	intergenic	---
4	77260258	77260258	+	77260258	4	4	XLOC_061321	+	predicted	---
4	77262462	77262469	+	77262463	19	35	---	---	intergenic	---
4	77303101	77303126	+	77303125	8	17	ENSSSCG00000006227	+	3'UTR	*GGH*
4	77303260	77303269	+	77303261	22	35	ENSSSCG00000006227	+	3'UTR	*GGH*
4	77303317	77303318	+	77303317	5	8	ENSSSCG00000006227	+	3'UTR	*GGH*
4	77502133	77502133	-	77502133	3	3	---	---	intergenic	---
4	77589000	77589000	+	77589000	3	3	---	---	intergenic	---
4	77872931	77872938	-	77872936	9	35	---	---	intergenic	---
4	77872954	77872966	+	77872963	43	113	---	---	intergenic	---

4	78115579	78115582	+	78115582	10	19	XLOC_061327	+	predicted	---
4	78146145	78146145	+	78146145	3	3	---	---	intergenic	---
4	78296529	78296535	-	78296535	4	10	---	---	intergenic	---
4	78361273	78361276	+	78361273	4	10	---	---	intergenic	---
4	78384351	78384352	+	78384351	3	6	ENSSSCG00000025087	+	intron	---
4	78488197	78488197	+	78488197	6	6	ENSSSCG00000025087	+	ORF	---
4	78505767	78505768	+	78505767	10	14	ENSSSCG00000025087	+	3'UTR	---
4	78507762	78507762	-	78507762	4	4	ENSSSCG00000006230	-	intron	*CLVS1*
4	78793104	78793105	-	78793104	4	7	---	---	intergenic	---
4	79115499	79115499	-	79115499	4	4	ENSSSCG00000006231	-	ORF	*CHD7*
4	79199860	79199861	-	79199861	8	12	---	---	intergenic	---
4	79257582	79257596	-	79257589	17	57	ENSSSCG00000006232	-	3'UTR	---
4	79258170	79258170	-	79258170	6	6	ENSSSCG00000006232	-	3'UTR	---
4	79258515	79258522	-	79258516	11	20	ENSSSCG00000006232	-	3'UTR	---
4	79258642	79258652	-	79258650	44	71	ENSSSCG00000006232	-	3'UTR	---
4	79289246	79289251	-	79289251	8	13	---	---	intergenic	---
4	79457289	79457290	-	79457290	4	7	---	---	intergenic	---
4	79570156	79570156	+	79570156	3	3	ENSSSCG00000006233	+	intron	*CA8*
4	79578674	79578674	+	79578674	3	3	ENSSSCG00000006233	+	intron	*CA8*
4	79690731	79690731	+	79690731	6	6	---	---	intergenic	---
4	80148151	80148151	-	80148151	4	4	XLOC_062399	-	predicted	---
4	80154983	80154991	+	80154983	6	12	XLOC_061339	+	predicted	---
4	80272288	80272289	-	80272289	27	40	---	---	intergenic	---
4	81090100	81090113	-	81090106	75	92	ENSSSCG00000006237	-	3'UTR	*SDCBP*
4	81090482	81090482	+	81090482	3	3	XLOC_061343	+	predicted	---
4	81096565	81096565	-	81096565	3	3	ENSSSCG00000006237	-	ORF	*SDCBP*

4	81159480	81159480	+	81159480	3	3	ENSSSCG00000006238	+	ORF	*CYP7A1*
4	81166785	81166785	+	81166785	4	4	ENSSSCG00000006238	+	3'UTR	*CYP7A1*
4	81192971	81192971	+	81192971	3	3	XLOC_061345	+	predicted	---
4	81196512	81196512	+	81196512	3	3	XLOC_061345	+	predicted	---
4	81815000	81815000	+	81815000	22	22	---	---	intergenic	---
4	81944363	81944363	+	81944363	3	3	XLOC_061348	+	predicted	---
4	81946562	81946568	+	81946568	5	8	---	---	intergenic	---
4	81953900	81953901	+	81953900	4	8	XLOC_061348	+	predicted	---
4	82060148	82060168	-	82060163	6	27	---	---	intergenic	---
4	82060184	82060196	+	82060184	21	117	---	---	intergenic	---
4	82076229	82076235	-	82076235	9	16	XLOC_062405	-	predicted	---
4	82111831	82111831	+	82111831	3	3	ENSSSCG00000006242	+	intron	*IMPAD1*
4	82129989	82129998	+	82129996	34	72	ENSSSCG00000006242	+	3'UTR	*IMPAD1*
4	82130174	82130180	+	82130175	6	14	ENSSSCG00000006242	+	3'UTR	*IMPAD1*
4	82130273	82130275	+	82130275	7	12	XLOC_061350	+	predicted	---
4	82134078	82134082	+	82134079	5	16	XLOC_061350	+	predicted	---
4	82135191	82135194	+	82135193	44	81	XLOC_061350	+	predicted	---
4	82142262	82142262	-	82142262	6	6	XLOC_062406	-	predicted	---
4	82213495	82213505	-	82213505	29	32	XLOC_062407	-	predicted	---
4	82429681	82429681	+	82429681	3	3	XLOC_061352	+	predicted	---
4	82449426	82449426	+	82449426	4	4	XLOC_061353	+	predicted	---
4	82449744	82449744	+	82449744	6	6	XLOC_061353	+	predicted	---
4	82560252	82560277	-	82560255	83	369	XLOC_062413	-	predicted	---
4	82679916	82679916	-	82679916	3	3	---	---	intergenic	---
4	82711088	82711091	-	82711091	21	24	ENSSSCG00000006250	-	3'UTR	*LYN*
4	82711304	82711310	-	82711304	4	8	ENSSSCG00000006250	-	3'UTR	*LYN*

4	82713490	82713493	-	82713490	20	29	ENSSSCG00000006250	-	3'UTR	*LYN*
4	82833464	82833464	+	82833464	4	4	---	---	intergenic	---
4	82896506	82896506	-	82896506	3	3	ENSSSCG00000006251	-	intron	---
4	83105128	83105128	+	83105128	4	4	---	---	intergenic	---
4	83961301	83961303	-	83961301	5	8	XLOC_062420	-	predicted	---
4	83970606	83970606	+	83970606	3	3	ENSSSCG00000006256	+	3'UTR	*SOX17*
4	84142548	84142563	-	84142557	100	270	ENSSSCG00000030578	-	3'UTR	*MRPL15*
4	84168656	84168657	+	84168656	12	19	ENSSSCG00000006260	+	intron	---
4	84171094	84171129	+	84171113	43	241	ENSSSCG00000006260	+	3'UTR	---
4	84171210	84171250	+	84171248	92	221	ENSSSCG00000006260	+	3'UTR	---
4	84171528	84171529	+	84171528	19	26	ENSSSCG00000006260	+	3'UTR	---
4	84172650	84172662	+	84172658	37	139	ENSSSCG00000006260	+	3'UTR	---
4	84194830	84194853	+	84194830	3	6	ENSSSCG00000006261	+	ORF	*TCEA1*
4	84200816	84200816	+	84200816	3	3	ENSSSCG00000006261	+	intron	*TCEA1*
4	84207749	84207776	+	84207770	31	53	ENSSSCG00000006261	+	3'UTR	*TCEA1*
4	84208585	84208585	+	84208585	13	13	ENSSSCG00000006261	+	3'UTR	*TCEA1*
4	84290023	84290023	+	84290023	4	4	ENSSSCG00000006263	+	intron	*ATP6V1H*
4	84340039	84340042	+	84340039	43	69	ENSSSCG00000006263	+	3'UTR	*ATP6V1H*
4	84388491	84388491	+	84388491	3	3	---	---	intergenic	---
4	84395231	84395231	+	84395231	7	7	---	---	intergenic	---
4	84509321	84509321	+	84509321	3	3	---	---	intergenic	---
4	84518873	84518873	-	84518873	3	3	---	---	intergenic	---
4	84536148	84536148	+	84536148	3	3	---	---	intergenic	---
4	84629512	84629512	+	84629512	5	5	---	---	intergenic	---
4	84630348	84630348	-	84630348	3	3	---	---	intergenic	---
4	84723959	84723959	+	84723959	3	3	---	---	intergenic	---

4	84951629	84951629	+	84951629	12	12	---	---	intergenic	---
4	84970621	84970623	-	84970621	8	14	---	---	intergenic	---
4	85143042	85143043	+	85143043	5	8	ENSSSCG00000006266	+	intron	*ST18*
4	85159224	85159224	+	85159224	4	4	ENSSSCG00000006266	+	intron	*ST18*
4	85222861	85222861	-	85222861	3	3	---	---	intergenic	---
4	85378816	85378816	+	85378816	3	3	ENSSSCG00000006267	+	intron	*PCMTD1*
4	85390555	85390557	-	85390557	18	26	---	---	intergenic	---
4	85391027	85391028	+	85391027	13	17	XLOC_061373	+	predicted	---
4	85391470	85391470	+	85391470	3	3	XLOC_061373	+	predicted	---
4	85428297	85428298	-	85428297	11	15	---	---	intergenic	---
4	85564287	85564287	-	85564287	3	3	---	---	intergenic	---
4	85838890	85838890	-	85838890	3	3	---	---	intergenic	---
4	86075606	86075606	+	86075606	3	3	---	---	intergenic	---
4	86302859	86302859	+	86302859	7	7	XLOC_061374	+	predicted	---
4	86302938	86302952	+	86302938	34	94	XLOC_061374	+	predicted	---
4	86303061	86303067	+	86303061	13	17	XLOC_061374	+	predicted	---
4	86303504	86303505	+	86303504	6	9	XLOC_061374	+	predicted	---
4	86318081	86318081	+	86318081	5	5	---	---	intergenic	---
4	86325148	86325148	-	86325148	5	5	XLOC_062433	-	predicted	---
4	86355527	86355527	-	86355527	7	7	---	---	intergenic	---
4	86694844	86694844	-	86694844	10	10	---	---	intergenic	---
4	86738415	86738426	-	86738426	10	15	XLOC_062435	-	predicted	---
4	86744679	86744679	+	86744679	4	4	---	---	intergenic	---
4	86774169	86774170	+	86774169	3	6	---	---	intergenic	---
4	87012627	87012628	+	87012627	3	6	---	---	intergenic	---
4	87191994	87191994	+	87191994	5	5	ENSSSCG00000006274	+	intron	*PRKDC*

4	87343684	87343684	+	87343684	5	5	XLOC_061384	+	predicted	---
4	87348603	87348603	+	87348603	3	3	XLOC_061384	+	predicted	---
4	87370111	87370119	+	87370112	62	123	ENSSSCG00000006276	+	3'UTR	*CEBPD*
							ENSSSCG00000006276	+	ORF	---
4	87370564	87370611	+	87370585	78	206	ENSSSCG00000006276	+	3'UTR	*CEBPD*
							ENSSSCG00000006276	+	ORF	---
4	87371243	87371243	-	87371243	3	3	ENSSSCG00000006277	-	3'UTR	*SPIDR*
4	87445093	87445097	-	87445097	6	10	ENSSSCG00000006277	-	intron	*SPIDR*
4	87732692	87732693	-	87732692	8	12	---	---	intergenic	---
4	87767525	87767525	+	87767525	3	3	---	---	intergenic	---
4	87890283	87890283	-	87890283	3	3	XLOC_062446	-	predicted	---
4	88080095	88080095	+	88080095	4	4	---	---	intergenic	---
4	88352079	88352079	+	88352079	3	3	---	---	intergenic	---
4	88588468	88588468	+	88588468	6	6	ENSSSCG00000006282	+	ORF	*KIFAP3*
4	88589471	88589471	+	88589471	3	3	ENSSSCG00000006282	+	intron	*KIFAP3*
4	88605485	88605485	+	88605485	3	3	ENSSSCG00000006282	+	intron	*KIFAP3*
4	88636544	88636544	+	88636544	3	3	ENSSSCG00000006282	+	intron	*KIFAP3*
4	88734081	88734086	+	88734083	13	23	ENSSSCG00000006282	+	3'UTR	*KIFAP3*
4	88760581	88760581	-	88760581	3	3	---	---	intergenic	---
4	88802082	88802082	+	88802082	6	6	ENSSSCG00000006283	+	3'UTR	*SCYL3*
4	88802373	88802373	-	88802373	6	6	ENSSSCG00000006284	-	3'UTR	*C1orf112*
4	88845175	88845175	+	88845175	3	3	XLOC_061395	+	predicted	---
4	88950568	88950596	+	88950589	12	33	ENSSSCG00000006287	+	3'UTR	*SELL*
4	88951093	88951095	+	88951095	29	42	ENSSSCG00000006287	+	3'UTR	*SELL*
4	89032699	89032699	+	89032699	3	3	ENSSSCG00000006289	+	ORF	*F5*
4	89059456	89059464	+	89059456	4	7	ENSSSCG00000006289	+	ORF	*F5*

4	89069549	89069549	+	89069549	3	3	ENSSSCG00000006289	+	intron	*F5*
4	89083072	89083072	+	89083072	3	3	ENSSSCG00000006289	+	ORF	*F5*
4	89087996	89087998	+	89087997	11	19	ENSSSCG00000006289	+	ORF	*F5*
4	89109517	89109518	+	89109518	13	16	ENSSSCG00000006289	+	3'UTR	*F5*
4	89109652	89109652	+	89109652	7	7	XLOC_061399	+	predicted	---
4	89109729	89109735	+	89109729	8	12	XLOC_061399	+	predicted	---
4	89145726	89145726	+	89145726	3	3	ENSSSCG00000006290	+	intron	*SLC19A2*
4	89197574	89197585	+	89197585	30	49	ENSSSCG00000028804	+	intron	*CCDC181*
4	89218434	89218436	+	89218435	11	21	ENSSSCG00000028804	+	3'UTR	*CCDC181*
4	89218594	89218594	+	89218594	3	3	ENSSSCG00000028804	+	3'UTR	*CCDC181*
4	89221978	89221988	-	89221988	14	45	ENSSSCG00000022396	-	3'UTR	*BLZF1*
4	89222260	89222260	-	89222260	3	3	ENSSSCG00000022396	-	3'UTR	*BLZF1*
4	89254059	89254082	-	89254062	53	109	---	---	intergenic	---
4	89254113	89254119	+	89254119	4	10	---	---	intergenic	---
4	89254154	89254154	-	89254154	4	4	---	---	intergenic	---
4	89254172	89254174	+	89254173	13	27	---	---	intergenic	---
4	89254302	89254308	-	89254302	3	6	---	---	intergenic	---
4	89254327	89254327	+	89254327	3	3	---	---	intergenic	---
4	89375756	89375756	+	89375756	4	4	---	---	intergenic	---
4	89434816	89434816	-	89434816	5	5	---	---	intergenic	---
4	89529586	89529598	-	89529598	17	39	ENSSSCG00000006296	-	3'UTR	*ATP1B1*
4	89530313	89530319	-	89530319	8	17	ENSSSCG00000006296	-	3'UTR	*ATP1B1*
4	89530604	89530604	-	89530604	5	5	ENSSSCG00000006296	-	3'UTR	*ATP1B1*
4	89548974	89548974	-	89548974	3	3	ENSSSCG00000006296	-	intron	*ATP1B1*
4	89698371	89698371	+	89698371	3	3	---	---	intergenic	---
4	90000240	90000241	+	90000240	6	10	ENSSSCG00000006297	+	3'UTR	*DPT*

4	90000844	90000844	+	90000844	4	4	ENSSSCG00000006297	+	3'UTR	*DPT*
4	90001106	90001106	+	90001106	4	4	ENSSSCG00000006297	+	3'UTR	*DPT*
4	90387006	90387006	-	90387006	8	8	XLOC_062466	-	predicted	---
4	90392454	90392454	-	90392454	11	11	ENSSSCG00000006300	-	3'UTR	*SFT2D2*
4	90433424	90433424	-	90433424	6	6	ENSSSCG00000006301	-	3'UTR	*TIPRL*
4	90435266	90435290	-	90435279	18	29	ENSSSCG00000006301	-	3'UTR	*TIPRL*
4	90436668	90436668	-	90436668	18	18	ENSSSCG00000006301	-	intron	*TIPRL*
4	90497439	90497439	-	90497439	8	8	---	---	intergenic	---
4	90576892	90576922	+	90576909	24	97	XLOC_061410	+	predicted	---
4	90576997	90577016	+	90577012	199	517	XLOC_061410	+	predicted	---
4	90577276	90577289	+	90577276	3	6	XLOC_061410	+	predicted	---
4	90577406	90577409	+	90577406	7	22	XLOC_061410	+	predicted	---
4	90577476	90577479	+	90577476	3	9	XLOC_061410	+	predicted	---
4	90584900	90584906	-	90584906	24	56	XLOC_062468	-	predicted	---
4	90584950	90584951	-	90584950	12	23	XLOC_062468	-	predicted	---
4	90637941	90637945	-	90637942	8	21	ENSSSCG00000006303	-	intron	---
4	90764461	90764461	+	90764461	4	4	ENSSSCG00000006304	+	intron	*MPC2*
4	90767232	90767250	+	90767236	76	152	ENSSSCG00000006304	+	3'UTR	*MPC2*
4	90767329	90767337	+	90767334	17	81	ENSSSCG00000006304	+	3'UTR	*MPC2*
4	90767565	90767565	+	90767565	3	3	ENSSSCG00000006304	+	3'UTR	*MPC2*
4	90880582	90880582	+	90880582	5	5	XLOC_061414	+	predicted	---
4	90911480	90911486	-	90911482	27	62	XLOC_062474	-	predicted	---
4	90984364	90984364	-	90984364	5	5	ENSSSCG00000006306	-	intron	*MPZL1*
4	90999952	90999953	-	90999952	5	8	ENSSSCG00000006307	-	3'UTR	*RCSD1*
4	91145666	91145710	+	91145709	73	182	ENSSSCG00000006308	+	3'UTR	*CREG1*
4	91165209	91165209	-	91165209	3	3	---	---	intergenic	---

4	91215088	91215088	+	91215088	9	9	ENSSSCG00000006309	+	intron	*CD3Z*
4	91259363	91259363	+	91259363	4	4	ENSSSCG00000006309	+	3'UTR	*CD3Z*
4	91259693	91259693	+	91259693	3	3	XLOC_061417	+	predicted	---
4	91260186	91260198	+	91260191	12	25	XLOC_061417	+	predicted	---
4	91260271	91260282	+	91260281	30	76	XLOC_061417	+	predicted	---
4	91335880	91335880	-	91335880	12	12	ENSSSCG00000006310	-	intron	*POU2F1*
4	91658101	91658101	+	91658101	7	7	---	---	intergenic	---
4	91722598	91722598	+	91722598	3	3	ENSSSCG00000006316	+	ORF	---
4	91722706	91722706	+	91722706	10	10	ENSSSCG00000006316	+	3'UTR	---
4	91722766	91722768	+	91722767	34	44	ENSSSCG00000006316	+	3'UTR	---
4	91722859	91722880	+	91722879	9	38	ENSSSCG00000006316	+	3'UTR	---
4	91830465	91830472	+	91830465	12	15	---	---	intergenic	---
4	91833988	91833988	+	91833988	3	3	---	---	intergenic	---
4	91894253	91894253	+	91894253	3	3	ENSSSCG00000006318	+	intron	*TADA1*
4	91895643	91895643	+	91895643	3	3	ENSSSCG00000006318	+	intron	*TADA1*
4	91900970	91900989	+	91900989	13	16	ENSSSCG00000006318	+	3'UTR	*TADA1*
4	92244331	92244332	-	92244332	6	9	---	---	intergenic	---
4	92619231	92619241	+	92619241	19	34	ENSSSCG00000006323	+	intron	*TMCO1*
4	92668082	92668082	+	92668082	3	3	ENSSSCG00000006323	+	3'UTR	*TMCO1*
4	92668172	92668196	+	92668184	24	108	ENSSSCG00000006323	+	3'UTR	*TMCO1*
4	92674192	92674192	+	92674192	3	3	XLOC_061427	+	predicted	---
4	92674588	92674588	+	92674588	4	4	XLOC_061427	+	predicted	---
4	92701253	92701261	+	92701253	24	86	ENSSSCG00000006324	+	intron	*ALDH9A1*
4	92707891	92707891	+	92707891	4	4	ENSSSCG00000006324	+	intron	*ALDH9A1*
4	92713101	92713101	+	92713101	6	6	ENSSSCG00000006324	+	3'UTR	*ALDH9A1*
4	92713561	92713571	+	92713564	242	436	ENSSSCG00000006324	+	3'UTR	*ALDH9A1*

4	92725514	92725528	-	92725522	185	332	ENSSSCG00000006325	-	3'UTR	*MGST3*
4	92844633	92844639	-	92844638	13	30	XLOC_062492	-	predicted	---
4	93063350	93063354	+	93063350	5	12	ENSSSCG00000006328	+	3'UTR	*RXRG*
4	93071386	93071386	+	93071386	5	5	---	---	intergenic	---
4	93132968	93132968	+	93132968	7	7	---	---	intergenic	---
4	93221127	93221127	-	93221127	3	3	---	---	intergenic	---
4	93571748	93571748	-	93571748	3	3	XLOC_062496	-	predicted	---
4	93658195	93658195	-	93658195	5	5	---	---	intergenic	---
4	93763379	93763379	+	93763379	3	3	---	---	intergenic	---
4	93903211	93903211	-	93903211	3	3	---	---	intergenic	---
4	94212781	94212781	+	94212781	3	3	---	---	intergenic	---
4	94875756	94875760	-	94875760	14	31	ENSSSCG00000006333	-	3'UTR	*NUF2*
4	94959646	94959646	+	94959646	3	3	---	---	intergenic	---
4	95053087	95053087	+	95053087	4	4	ENSSSCG00000006334	+	3'UTR	*RGS5*
4	95053684	95053690	+	95053684	6	9	XLOC_061436	+	predicted	---
4	95057120	95057120	+	95057120	5	5	XLOC_061436	+	predicted	---
4	95096423	95096423	-	95096423	3	3	ENSSSCG00000006335	-	3'UTR	*RGS4*
4	95283104	95283107	+	95283104	9	17	---	---	intergenic	---
4	95564960	95564960	+	95564960	3	3	XLOC_061438	+	predicted	---
4	95738941	95738968	-	95738947	42	88	ENSSSCG00000006340	-	3'UTR	*UAP1*
4	95755691	95755691	-	95755691	3	3	ENSSSCG00000006340	-	ORF	*UAP1*
4	95900077	95900091	-	95900088	81	241	XLOC_062507	-	predicted	---
4	95900466	95900466	-	95900466	3	3	ENSSSCG00000006343	-	ORF	*C1orf111*
4	95909454	95909455	+	95909454	5	8	XLOC_061440	+	predicted	---
4	96123163	96123180	-	96123179	98	633	---	---	intergenic	---
4	96125307	96125307	-	96125307	3	3	---	---	intergenic	---

4	96500754	96500754	+	96500754	3	3	ENSSSCG00000006345	+	3'UTR	*OLFML2B*
4	96531659	96531667	-	96531659	8	15	XLOC_062511	-	predicted	---
4	96534598	96534603	-	96534598	13	37	XLOC_062511	-	predicted	---
4	96536756	96536758	-	96536756	6	12	XLOC_062511	-	predicted	---
4	96700469	96700469	-	96700469	4	4	ENSSSCG00000006346	-	intron	*ATF6*
4	96813268	96813268	-	96813268	4	4	ENSSSCG00000006346	-	ORF	*ATF6*
4	96827142	96827142	-	96827142	5	5	ENSSSCG00000006347	-	3'UTR	---
4	96889011	96889011	-	96889011	3	3	ENSSSCG00000006350	-	3'UTR	*CD32*
4	96889095	96889095	-	96889095	4	4	ENSSSCG00000006350	-	3'UTR	*CD32*
4	96990444	96990446	+	96990446	49	102	ENSSSCG00000023957	+	3'UTR	*CFAP126*
4	96992638	96992646	-	96992640	101	199	ENSSSCG00000030318	-	3'UTR	*SDHC*
4	97044312	97044312	-	97044312	3	3	XLOC_062519	-	predicted	---
4	97080398	97080398	-	97080398	3	3	---	---	intergenic	---
4	97087411	97087411	-	97087411	3	3	ENSSSCG00000006354	-	3'UTR	*TOMM40L*
4	97088465	97088465	+	97088465	3	3	ENSSSCG00000006353	+	3'UTR	*CAR*
4	97095790	97095808	+	97095806	486	923	ENSSSCG00000006355	+	3'UTR	*APOA2*
4	97099150	97099152	-	97099150	119	162	ENSSSCG00000006357	-	3'UTR	*FCER1G*
4	97103641	97103650	-	97103642	77	207	ENSSSCG00000006358	-	3'UTR	*NDUFS2*
4	97134713	97134713	-	97134713	3	3	XLOC_062522	-	predicted	---
4	97141128	97141134	+	97141129	13	39	ENSSSCG00000006360	+	3'UTR	*B4GALT3*
4	97141226	97141228	-	97141227	24	35	XLOC_062522	-	predicted	---
4	97152858	97152858	-	97152858	3	3	---	---	intergenic	---
4	97153008	97153014	-	97153013	102	178	ENSSSCG00000006363	-	3'UTR	*UFC1*
4	97153257	97153257	-	97153257	3	3	ENSSSCG00000006363	-	3'UTR	*UFC1*
4	97179925	97179925	+	97179925	23	23	XLOC_061452	+	predicted	---
4	97180251	97180252	+	97180251	10	16	XLOC_061452	+	predicted	---

4	97180532	97180540	-	97180540	39	60	ENSSSCG00000006365	-	3'UTR	*NIT1*
4	97180618	97180645	+	97180645	8	27	XLOC_061452	+	predicted	---
4	97198376	97198394	+	97198378	110	342	XLOC_061452	+	predicted	---
4	97198588	97198593	-	97198589	47	85	XLOC_062526	-	predicted	---
4	97198636	97198639	-	97198637	10	27	XLOC_062526	-	predicted	---
4	97323386	97323393	-	97323387	34	56	ENSSSCG00000006369	-	3'UTR	*F11R*
4	97344016	97344016	-	97344016	3	3	ENSSSCG00000006369	-	intron	*F11R*
4	97371107	97371117	-	97371117	22	33	ENSSSCG00000006370	-	3'UTR	*TSTD1*
4	97372799	97372799	-	97372799	4	4	ENSSSCG00000006371	-	3'UTR	*USF1*
4	97379665	97379665	-	97379665	19	19	ENSSSCG00000006372	-	3'UTR	*ARHGAP30*
4	97546649	97546649	-	97546649	3	3	ENSSSCG00000006374	-	3'UTR	*CD244*
4	97610138	97610138	-	97610138	3	3	ENSSSCG00000028374	-	3'UTR	*ITLN2*
4	97736600	97736618	+	97736601	33	70	ENSSSCG00000006379	+	3'UTR	*CD48*
4	97827922	97827922	+	97827922	4	4	ENSSSCG00000006381	+	intron	*CD84*
4	97854549	97854549	+	97854549	3	3	XLOC_061464	+	predicted	---
4	97856610	97856610	+	97856610	3	3	XLOC_061464	+	predicted	---
4	97893026	97893026	+	97893026	3	3	XLOC_061465	+	predicted	---
4	98011643	98011645	-	98011643	6	12	ENSSSCG00000006385	-	3'UTR	*NCSTN*
4	98012210	98012211	-	98012210	6	12	ENSSSCG00000006385	-	3'UTR	*NCSTN*
4	98037132	98037132	+	98037132	3	3	ENSSSCG00000006386	+	intron	*COPA*
4	98044809	98044809	+	98044809	64	64	ENSSSCG00000006386	+	intron	*COPA*
4	98082164	98082176	+	98082166	53	106	ENSSSCG00000006386	+	3'UTR	*COPA*
4	98092647	98092648	+	98092647	7	11	ENSSSCG00000023091	+	3'UTR	*PEX19*
							ENSSSCG00000023091	+	intron	*PEX19*
							ENSSSCG00000023091	+	ORF	*PEX19*

4	98093476	98093485	+	98093480	13	42	ENSSSCG00000023091	+	3'UTR	*PEX19*
							ENSSSCG00000023091	+	intron	*PEX19*
							ENSSSCG00000023091	+	ORF	*PEX19*
4	98093734	98093736	+	98093735	17	23	ENSSSCG00000023091	+	3'UTR	*PEX19*
							ENSSSCG00000023091	+	intron	*PEX19*
							ENSSSCG00000023091	+	ORF	*PEX19*
4	98095650	98095650	+	98095650	3	3	ENSSSCG00000023091	+	3'UTR	*PEX19*
							ENSSSCG00000023091	+	intron	*PEX19*
							ENSSSCG00000023091	+	ORF	*PEX19*
4	98102691	98102691	+	98102691	3	3	XLOC_061468	+	predicted	---
4	98106115	98106115	+	98106115	3	3	ENSSSCG00000006388	+	intron	---
4	98134228	98134228	+	98134228	3	3	ENSSSCG00000006388	+	intron	---
4	98203701	98203701	+	98203701	4	4	XLOC_061469	+	predicted	---
4	98204590	98204590	-	98204590	6	6	XLOC_062542	-	predicted	---
4	98204655	98204658	+	98204656	7	16	XLOC_061469	+	predicted	---
4	98204998	98205001	-	98204999	48	66	XLOC_062543	-	predicted	---
4	98218151	98218151	-	98218151	41	41	XLOC_062544	-	predicted	---
4	98232515	98232528	-	98232518	132	218	XLOC_062544	-	predicted	---
4	98270681	98270681	-	98270681	8	8	ENSSSCG00000020953	-	ORF	*ATP1A4*
4	98282717	98282718	-	98282718	20	24	XLOC_062546	-	predicted	---
4	98342769	98342770	+	98342769	5	10	ENSSSCG00000006392	+	3'UTR	*IGSF8*
4	98356839	98356842	+	98356842	7	14	XLOC_061473	+	predicted	---
4	98364748	98364748	+	98364748	3	3	---	---	intergenic	---
4	98545748	98545748	+	98545748	4	4	ENSSSCG00000006400	+	3'UTR	*SLAMF9*
							ENSSSCG00000006400	+	ORF	---
4	98555979	98555979	-	98555979	3	3	---	---	intergenic	---

4	98581377	98581377	-	98581377	3	3	ENSSSCG00000006394	-	ORF	*CFAP45*
4	98615635	98615643	-	98615641	145	185	ENSSSCG00000006395	-	3'UTR	*TAGLN2*
4	98626411	98626420	-	98626420	5	8	ENSSSCG00000006396	-	3'UTR	*IGSF9*
4	98692568	98692585	-	98692568	5	11	XLOC_062556	-	predicted	---
4	98755800	98755800	+	98755800	3	3	ENSSSCG00000021186	+	3'UTR	*CRP*
4	98756650	98756659	+	98756655	173	265	ENSSSCG00000021186	+	3'UTR	*CRP*
4	98759334	98759334	+	98759334	3	3	XLOC_061479	+	predicted	---
4	98771755	98771772	+	98771762	86	410	ENSSSCG00000006403	+	3'UTR	*CRP*
4	98772650	98772650	+	98772650	3	3	ENSSSCG00000006403	+	3'UTR	*CRP*
							ENSSSCG00000006403	+	ORF	*CRP*
4	98772719	98772750	+	98772719	67	93	ENSSSCG00000006403	+	3'UTR	*CRP*
							ENSSSCG00000006403	+	ORF	*CRP*
4	98823161	98823161	-	98823161	6	6	XLOC_062558	-	predicted	---
4	98899315	98899317	-	98899316	31	43	---	---	intergenic	---
4	98899778	98899793	-	98899792	33	74	---	---	intergenic	---
4	98900790	98900790	-	98900790	9	9	XLOC_062561	-	predicted	---
4	98900943	98900949	-	98900944	14	34	XLOC_062561	-	predicted	---
4	98901016	98901017	-	98901017	19	24	XLOC_062561	-	predicted	---
4	98954814	98954814	+	98954814	3	3	---	---	intergenic	---
4	99064776	99064776	+	99064776	3	3	---	---	intergenic	---
4	99276586	99276586	-	99276586	5	5	---	---	intergenic	---
4	99372516	99372516	-	99372516	3	3	ENSSSCG00000006418	-	3'UTR	*MNDA*
4	99386875	99386882	-	99386875	25	29	ENSSSCG00000006418	-	intron	*MNDA*
4	99388907	99388907	-	99388907	9	9	ENSSSCG00000006418	-	ORF	*MNDA*
4	99421724	99421726	-	99421724	5	9	ENSSSCG00000006418	-	ORF	*MNDA*
4	99909242	99909242	+	99909242	6	6	ENSSSCG00000025644	+	3'UTR	*PCD1A*

4	100027717	100027717	+	100027717	12	12	---	---	intergenic	---
4	100450062	100450062	-	100450062	5	5	ENSSSCG00000006452	-	intron	*CD1D*
4	100471087	100471087	+	100471087	5	5	---	---	intergenic	---
4	100476675	100476675	+	100476675	3	3	---	---	intergenic	---
4	100625374	100625374	-	100625374	14	14	ENSSSCG00000006453	-	intron	*KIRREL*
4	100739920	100739920	+	100739920	4	4	ENSSSCG00000006455	+	ORF	*CD5L*
4	100745588	100745589	+	100745589	10	15	ENSSSCG00000006455	+	intron	*CD5L*
4	100750101	100750101	+	100750101	3	3	ENSSSCG00000006455	+	3'UTR	*CD5L*
4	100750187	100750198	+	100750191	220	391	ENSSSCG00000006455	+	3'UTR	*CD5L*
4	100750863	100750863	+	100750863	18	18	---	---	intergenic	---
4	100896087	100896087	+	100896087	3	3	ENSSSCG00000006458	+	intron	*FCRL4*
4	100969762	100969762	+	100969762	3	3	XLOC_061516	+	predicted	---
4	101230766	101230769	-	101230768	27	36	XLOC_062593	-	predicted	---
4	101354606	101354606	-	101354606	4	4	XLOC_062593	-	predicted	---
4	101616557	101616558	+	101616558	9	13	ENSSSCG00000006461	+	intron	*ARHGEF11*
4	101734452	101734462	-	101734454	24	60	XLOC_062596	-	predicted	---
4	101805886	101805886	-	101805886	3	3	ENSSSCG00000006464	-	intron	*NTRK1*
4	101869506	101869512	-	101869509	13	43	ENSSSCG00000006467	-	3'UTR	*PRCC*
4	101874745	101874745	-	101874745	5	5	ENSSSCG00000006467	-	ORF	*PRCC*
4	101922963	101922963	+	101922963	3	3	ENSSSCG00000006468	+	3'UTR	*HDGF*
4	101923443	101923448	+	101923446	56	77	ENSSSCG00000006468	+	3'UTR	*HDGF*
4	101929433	101929447	+	101929436	87	196	ENSSSCG00000006469	+	3'UTR	*MRPL24*
4	101929781	101929782	-	101929781	3	6	ENSSSCG00000006470	-	3'UTR	*RRNAD1*
4	101938940	101938941	+	101938941	9	14	ENSSSCG00000006471	+	ORF	*ISG20L2*
4	101943390	101943405	+	101943402	66	153	ENSSSCG00000006471	+	3'UTR	*ISG20L2*
4	102095834	102095834	-	102095834	6	6	ENSSSCG00000006477	-	3'UTR	*BCAN*

4	102151612	102151612	+	102151612	3	3	ENSSSCG00000006475	+	intron	*IQGAP3*
4	102178070	102178070	+	102178070	4	4	XLOC_061535	+	predicted	---
4	102233691	102233691	+	102233691	3	3	ENSSSCG00000006482	+	3'UTR	*MEF2D*
4	102236157	102236159	+	102236157	3	6	ENSSSCG00000006482	+	3'UTR	*MEF2D*
4	102274581	102274582	+	102274581	7	10	---	---	intergenic	---
4	102291830	102291833	+	102291832	64	144	ENSSSCG00000006480	+	3'UTR	*APOA1BP*
4	102291983	102291991	+	102291991	10	13	ENSSSCG00000006480	+	3'UTR	*APOA1BP*
4	102292222	102292229	-	102292229	8	11	ENSSSCG00000006481	-	3'UTR	*GPATCH4*
4	102427700	102427711	-	102427709	4	15	ENSSSCG00000006486	-	3'UTR	*TSACC*
4	102427767	102427774	-	102427773	270	413	ENSSSCG00000006486	-	3'UTR	*TSACC*
4	102427896	102427896	-	102427896	3	3	ENSSSCG00000006486	-	ORF	*TSACC*
4	102445141	102445141	+	102445141	4	4	ENSSSCG00000006487	+	ORF	*CCT3*
4	102450892	102450892	+	102450892	3	3	ENSSSCG00000006487	+	ORF	*CCT3*
4	102453325	102453325	+	102453325	3	3	ENSSSCG00000006487	+	3'UTR	*CCT3*
4	102453389	102453413	+	102453405	311	704	ENSSSCG00000006487	+	3'UTR	*CCT3*
4	102461972	102461978	+	102461978	44	76	XLOC_061542	+	predicted	---
4	102501867	102501869	+	102501867	30	41	XLOC_061543	+	predicted	---
4	102511010	102511015	+	102511011	12	20	XLOC_061544	+	predicted	---
4	102529127	102529127	-	102529127	7	7	XLOC_062615	-	predicted	---
4	102552994	102553002	-	102552995	25	52	ENSSSCG00000006493	-	3'UTR	*PMF1*
4	102607464	102607464	-	102607464	6	6	XLOC_062616	-	predicted	---
4	102641459	102641467	-	102641466	28	39	XLOC_062617	-	predicted	---
4	102643592	102643593	-	102643592	44	48	ENSSSCG00000006496	-	intron	*LMNA*
4	102714888	102714895	-	102714890	115	211	ENSSSCG00000006499	-	3'UTR	*LAMTOR2*
4	102737607	102737607	+	102737607	15	15	ENSSSCG00000006500	+	3'UTR	*UBQLN4*
4	102744492	102744502	-	102744493	11	34	XLOC_062621	-	predicted	---

4	102764517	102764517	+	102764517	7	7	ENSSSCG00000006501	+	3'UTR	*SSR2*
4	102764962	102764986	+	102764967	550	1076	ENSSSCG00000006501	+	3'UTR	*SSR2*
4	102823421	102823430	+	102823427	36	54	XLOC_061550	+	predicted	---
4	102859676	102859676	+	102859676	3	3	ENSSSCG00000006504	+	3'UTR	*KIAA0907*
4	102867915	102867916	+	102867915	3	6	ENSSSCG00000023991	+	3'UTR	*RIT1*
4	102870885	102870885	+	102870885	10	10	ENSSSCG00000023991	+	3'UTR	*RIT1*
4	102907621	102907621	-	102907621	3	3	---	---	intergenic	---
4	102939579	102939579	+	102939579	5	5	ENSSSCG00000027966	+	intron	---
4	103042583	103042583	+	103042583	5	5	ENSSSCG00000006507	+	intron	---
4	103042681	103042681	+	103042681	3	3	ENSSSCG00000006507	+	intron	---
4	103047026	103047027	+	103047027	13	18	XLOC_061556	+	predicted	---
4	103060583	103060583	-	103060583	11	11	ENSSSCG00000006510	-	3'UTR	---
4	103276648	103276649	+	103276648	4	7	ENSSSCG00000006508	+	3'UTR	*ASH1L*
4	103280861	103280862	-	103280862	12	16	XLOC_062632	-	predicted	---
4	103291795	103291805	-	103291798	130	301	ENSSSCG00000006512	-	3'UTR	*FDPS*
4	103352214	103352217	+	103352216	7	20	ENSSSCG00000006519	+	3'UTR	*CLK2*
4	103357392	103357397	+	103357397	9	20	ENSSSCG00000006520	+	3'UTR	*SCAMP3*
4	103388663	103388666	+	103388664	30	40	ENSSSCG00000006522	+	3'UTR	*GBA*
4	103389137	103389143	+	103389137	3	6	ENSSSCG00000006522	+	3'UTR	*GBA*
4	103389152	103389166	-	103389153	22	79	ENSSSCG00000006523	-	3'UTR	*MTX1*
4	103389270	103389273	+	103389270	4	10	ENSSSCG00000006522	+	3'UTR	*GBA*
4	103389541	103389544	+	103389541	8	12	XLOC_061563	+	predicted	---
4	103392659	103392659	-	103392659	13	13	ENSSSCG00000006523	-	ORF	*MTX1*
4	103406719	103406719	+	103406719	6	6	ENSSSCG00000006524	+	3'UTR	*THBS3*
4	103429080	103429100	+	103429082	265	429	ENSSSCG00000006527	+	3'UTR	*KRTCAP2*

4	103444867	103444869	+	103444868	26	47	ENSSSCG00000006528	+	3'UTR	*DPM3*
							ENSSSCG00000006528	+	ORF	---
4	103445702	103445706	-	103445705	17	43	ENSSSCG00000006529	-	3'UTR	*SLC50A1*
4	103449103	103449111	-	103449109	58	191	ENSSSCG00000006530	-	3'UTR	*EFNA1*
4	103512404	103512404	-	103512404	15	15	ENSSSCG00000006532	-	3'UTR	---
4	103518256	103518256	-	103518256	3	3	ENSSSCG00000006533	-	3'UTR	---
4	103530043	103530045	-	103530043	5	8	XLOC_062647	-	predicted	---
4	103538836	103538836	-	103538836	7	7	---	---	intergenic	---
4	103563681	103563691	-	103563690	55	115	ENSSSCG00000006538	-	3'UTR	*FLAD1*
4	103572167	103572173	-	103572173	7	16	ENSSSCG00000029737	-	3'UTR	*CKS1B*
4	103588160	103588163	+	103588161	46	65	XLOC_061573	+	predicted	---
4	103591657	103591660	+	103591659	15	31	ENSSSCG00000006540	+	3'UTR	*PYGO2*
4	103592938	103592945	+	103592945	10	16	ENSSSCG00000006540	+	3'UTR	*PYGO2*
4	103620076	103620077	+	103620077	21	28	ENSSSCG00000006536	+	3'UTR	*PBXIP1*
4	103646116	103646127	+	103646124	22	117	ENSSSCG00000006535	+	3'UTR	*PMVK*
4	103797084	103797084	+	103797084	3	3	ENSSSCG00000006542	+	intron	*KCNN3*
4	103804711	103804711	+	103804711	4	4	ENSSSCG00000006542	+	intron	*KCNN3*
4	103882544	103882544	-	103882544	5	5	---	---	intergenic	---
4	104023305	104023305	+	104023305	5	5	XLOC_061582	+	predicted	---
4	104055536	104055538	+	104055537	40	59	ENSSSCG00000006544	+	3'UTR	*UBE2Q1*
4	104057254	104057257	-	104057257	15	28	XLOC_062658	-	predicted	---
4	104198427	104198437	-	104198436	144	151	ENSSSCG00000027628	-	3'UTR	---
4	104234813	104234814	+	104234813	3	6	ENSSSCG00000006549	+	intron	*IL6R*
4	104266352	104266355	-	104266353	33	54	ENSSSCG00000006552	-	3'UTR	---
4	104361923	104361923	+	104361923	3	3	XLOC_061590	+	predicted	---
4	104374633	104374633	+	104374633	3	3	---	---	intergenic	---

4	104447482	104447482	+	104447482	3	3	ENSSSCG00000006554	+	3'UTR	*C4H1ORF43*
4	104447530	104447544	+	104447533	40	158	ENSSSCG00000006554	+	3'UTR	*C4H1ORF43*
4	104451702	104451702	+	104451702	6	6	ENSSSCG00000006555	+	3'UTR	*C1orf189*
4	104451823	104451833	+	104451831	58	93	---	---	intergenic	---
4	104476835	104476836	+	104476835	5	9	ENSSSCG00000006556	+	intron	*TPM3*
4	104480044	104480047	+	104480047	10	14	ENSSSCG00000006556	+	intron	*TPM3*
4	104489133	104489163	+	104489162	17	39	ENSSSCG00000006556	+	3'UTR	*TPM3*
4	104489210	104489210	+	104489210	3	3	ENSSSCG00000006556	+	3'UTR	*TPM3*
4	104490144	104490151	+	104490144	51	60	XLOC_061594	+	predicted	---
4	104518686	104518686	+	104518686	3	3	---	---	intergenic	---
4	104519414	104519414	+	104519414	7	7	XLOC_061595	+	predicted	---
4	104524005	104524007	-	104524006	15	24	---	---	intergenic	---
4	104524077	104524077	-	104524077	9	9	---	---	intergenic	---
4	104597117	104597123	-	104597123	21	32	XLOC_062667	-	predicted	---
4	104597253	104597253	-	104597253	4	4	XLOC_062667	-	predicted	---
4	104599171	104599197	-	104599179	749	1818	XLOC_062667	-	predicted	---
4	104599642	104599642	-	104599642	6	6	ENSSSCG00000006558	-	ORF	*RPS27*
4	104609567	104609567	+	104609567	4	4	ENSSSCG00000006564	+	intron	*RAB13*
4	104610953	104610965	+	104610953	4	7	ENSSSCG00000006564	+	3'UTR	*RAB13*
4	104611128	104611128	+	104611128	4	4	ENSSSCG00000006564	+	3'UTR	*RAB13*
4	104611241	104611250	+	104611244	103	257	ENSSSCG00000006564	+	3'UTR	*RAB13*
4	104611317	104611317	+	104611317	4	4	XLOC_061599	+	predicted	---
4	104613848	104613848	+	104613848	3	3	XLOC_061599	+	predicted	---
4	104617450	104617457	+	104617450	44	51	ENSSSCG00000006559	+	3'UTR	*JTB*
4	104617834	104617840	-	104617834	5	8	XLOC_062668	-	predicted	---
4	104635126	104635130	+	104635128	16	30	ENSSSCG00000006561	+	3'UTR	*SLC39A1*

4	104647817	104647817	+	104647817	3	3	XLOC_061602	+	predicted	---
4	104664338	104664338	+	104664338	3	3	ENSSSCG00000006568	+	intron	*DENND4B*
4	104764821	104764821	+	104764821	3	3	XLOC_061603	+	predicted	---
4	104797203	104797229	-	104797203	3	6	ENSSSCG00000006569	-	3'UTR	*SLC27A3*
4	104802345	104802351	-	104802351	9	12	XLOC_062670	-	predicted	---
4	104802919	104802919	-	104802919	3	3	XLOC_062670	-	predicted	---
4	104803032	104803043	-	104803043	8	16	XLOC_062670	-	predicted	---
4	104855578	104855584	-	104855578	3	6	ENSSSCG00000006572	-	3'UTR	*NPR1*
4	104880458	104880458	+	104880458	3	3	ENSSSCG00000028553	+	3'UTR	*ILF2*
4	104880520	104880532	+	104880527	35	179	ENSSSCG00000028553	+	3'UTR	*ILF2*
4	104880681	104880712	-	104880703	63	151	ENSSSCG00000027049	-	3'UTR	*SNAPIN*
							ENSSSCG00000027049	-	intron	*SNAPIN*
4	104889209	104889217	-	104889210	14	38	ENSSSCG00000022107	-	3'UTR	*CHTOP*
4	104890062	104890065	-	104890063	36	77	ENSSSCG00000022107	-	3'UTR	*CHTOP*
4	104900201	104900213	-	104900210	160	565	ENSSSCG00000031053	-	3'UTR	*S100A1*
4	104908140	104908142	-	104908142	19	27	ENSSSCG00000006578	-	3'UTR	*S100A4*
4	104922474	104922481	-	104922479	25	41	ENSSSCG00000006580	-	3'UTR	*S100A2*
4	104961771	104961774	-	104961773	13	23	ENSSSCG00000006581	-	3'UTR	*S100A16*
4	104974130	104974139	-	104974132	71	147	---	---	intergenic	---
4	105066291	105066300	+	105066297	253	467	ENSSSCG00000006588	+	3'UTR	*S100A9*
4	105075279	105075292	-	105075282	393	585	ENSSSCG00000006589	-	3'UTR	*S100A12*
4	105075980	105075981	-	105075980	5	8	ENSSSCG00000006589	-	ORF	*S100A12*
4	105091813	105091818	-	105091817	154	168	ENSSSCG00000006590	-	3'UTR	*S100A8*
4	105232072	105232089	-	105232085	33	85	---	---	intergenic	---
4	105232116	105232119	+	105232118	8	19	---	---	intergenic	---
4	105259814	105259814	-	105259814	3	3	---	---	intergenic	---

4	105265114	105265125	-	105265120	689	1440	XLOC_062685	-	predicted	---
4	105265403	105265403	-	105265403	5	5	XLOC_062685	-	predicted	---
4	105265465	105265465	-	105265465	6	6	XLOC_062685	-	predicted	---
4	105265516	105265516	-	105265516	8	8	XLOC_062685	-	predicted	---
4	105266923	105266923	-	105266923	5	5	---	---	intergenic	---
4	105267005	105267021	+	105267006	7	23	---	---	intergenic	---
4	105314304	105314304	+	105314304	37	37	---	---	intergenic	---
4	105441993	105441993	-	105441993	3	3	---	---	intergenic	---
4	105442019	105442022	+	105442019	3	6	---	---	intergenic	---
4	105607718	105607731	-	105607720	190	423	---	---	intergenic	---
4	105607815	105607815	-	105607815	6	6	ENSSSCG00000006594	-	3'UTR	*SMCP*
4	105608040	105608040	-	105608040	4	4	ENSSSCG00000006594	-	ORF	*SMCP*
4	105608204	105608204	-	105608204	4	4	ENSSSCG00000006594	-	ORF	*SMCP*
4	105612144	105612144	-	105612144	6	6	---	---	intergenic	---
4	105815609	105815609	-	105815609	3	3	XLOC_062696	-	predicted	---
4	105819153	105819158	+	105819153	3	6	XLOC_061618	+	predicted	---
4	105886185	105886185	+	105886185	3	3	XLOC_061622	+	predicted	---
4	105909771	105909772	-	105909771	6	12	XLOC_062698	-	predicted	---
4	105909810	105909811	-	105909810	6	10	XLOC_062698	-	predicted	---
4	105911908	105911908	-	105911908	3	3	---	---	intergenic	---
4	105935327	105935327	+	105935327	3	3	XLOC_061623	+	predicted	---
4	106092529	106092529	-	106092529	3	3	---	---	intergenic	---
4	106194391	106194391	+	106194391	4	4	ENSSSCG00000006610	+	intron	*S100A11*
4	106199594	106199602	+	106199594	87	226	ENSSSCG00000006610	+	3'UTR	*S100A11*
							ENSSSCG00000006610	+	ORF	---
4	106279749	106279749	+	106279749	3	3	ENSSSCG00000006612	+	ORF	---

4	106282998	106283039	+	106283032	107	325	ENSSSCG00000006612	+	3'UTR	*S100A10*
4	106421977	106421980	+	106421977	9	17	ENSSSCG00000026427	+	3'UTR	*RORC*
4	106461261	106461264	+	106461264	7	11	XLOC_061638	+	predicted	---
4	106462993	106463021	-	106462999	1717	3208	XLOC_062703	-	predicted	---
4	106466918	106466922	+	106466918	9	23	XLOC_061639	+	predicted	---
4	106485479	106485479	+	106485479	5	5	ENSSSCG00000028343	+	3'UTR	*MRPL9*
4	106485522	106485531	+	106485526	23	56	ENSSSCG00000028343	+	3'UTR	*MRPL9*
4	106612965	106612965	+	106612965	4	4	ENSSSCG00000006620	+	intron	*TUFT1*
4	106637136	106637137	+	106637137	6	9	ENSSSCG00000006620	+	3'UTR	*TUFT1*
4	106645236	106645236	+	106645236	3	3	---	---	intergenic	---
4	106752314	106752321	+	106752315	27	37	XLOC_061642	+	predicted	---
4	106853592	106853598	-	106853592	3	9	XLOC_062712	-	predicted	---
4	107036321	107036333	-	107036330	145	180	ENSSSCG00000006623	-	3'UTR	*PSMB4*
4	107094776	107094786	+	107094777	151	363	ENSSSCG00000006624	+	3'UTR	*SELENBP1*
4	107095080	107095081	+	107095080	6	9	ENSSSCG00000006624	+	3'UTR	*SELENBP1*
4	107150936	107150946	-	107150943	11	20	XLOC_062715	-	predicted	---
4	107246844	107246869	-	107246862	27	77	XLOC_062716	-	predicted	---
4	107265724	107265725	-	107265724	12	22	XLOC_062717	-	predicted	---
4	107311576	107311576	+	107311576	9	9	---	---	intergenic	---
4	107314282	107314282	+	107314282	5	5	---	---	intergenic	---
4	107326839	107326845	+	107326844	10	53	XLOC_061651	+	predicted	---
4	107332125	107332129	+	107332129	11	15	ENSSSCG00000006631	+	3'UTR	*TMOD4*
4	107346210	107346210	-	107346210	29	29	ENSSSCG00000006634	-	3'UTR	*TNFAIP8L2*
4	107373125	107373125	+	107373125	3	3	XLOC_061653	+	predicted	---
4	107419906	107419914	-	107419913	19	30	ENSSSCG00000030278	-	3'UTR	*MLLT11*
4	107431951	107431957	+	107431957	5	9	XLOC_061654	+	predicted	---

4	107433547	107433551	+	107433551	12	17	XLOC_061654	+	predicted	---
4	107434047	107434084	-	107434047	28	76	XLOC_062724	-	predicted	---
4	107434245	107434281	-	107434246	31	50	XLOC_062724	-	predicted	---
4	107435061	107435061	-	107435061	3	3	ENSSSCG00000006639	-	intron	*C1orf56*
4	107435244	107435244	-	107435244	23	23	ENSSSCG00000006639	-	ORF	*C1orf56*
4	107447181	107447183	-	107447181	7	15	ENSSSCG00000006641	-	3'UTR	*PRUNE*
4	107448393	107448401	-	107448394	8	20	ENSSSCG00000006641	-	3'UTR	*PRUNE*
4	107448549	107448552	-	107448550	11	18	ENSSSCG00000006641	-	3'UTR	*PRUNE*
4	107485221	107485224	+	107485223	30	47	ENSSSCG00000006642	+	3'UTR	*FAM63A*
4	107514621	107514628	-	107514628	11	25	ENSSSCG00000006645	-	3'UTR	*SETDB1*
4	107514665	107514677	+	107514675	40	120	XLOC_061657	+	predicted	---
4	107514742	107514753	+	107514745	8	22	XLOC_061657	+	predicted	---
4	107514915	107514921	-	107514915	7	11	ENSSSCG00000006645	-	3'UTR	*SETDB1*
4	107539743	107539745	-	107539744	8	17	ENSSSCG00000006645	-	intron	*SETDB1*
4	107636310	107636310	+	107636310	3	3	ENSSSCG00000006646	+	3'UTR	*ARNT*
							ENSSSCG00000006646	+	ORF	---
4	107638149	107638149	+	107638149	11	11	ENSSSCG00000006646	+	3'UTR	*ARNT*
							ENSSSCG00000006646	+	ORF	---
4	107653098	107653101	+	107653098	9	14	ENSSSCG00000006647	+	3'UTR	*CTSK*
4	107658253	107658253	-	107658253	4	4	---	---	intergenic	---
4	107691543	107691577	+	107691562	79	195	ENSSSCG00000006648	+	3'UTR	*CTSS*
4	107707104	107707104	+	107707104	3	3	ENSSSCG00000006649	+	ORF	*HORMAD1*
4	107709751	107709751	+	107709751	4	4	ENSSSCG00000006649	+	ORF	*HORMAD1*
4	107718268	107718274	+	107718272	10	37	ENSSSCG00000006649	+	3'UTR	*HORMAD1*
4	107756767	107756767	+	107756767	5	5	XLOC_061659	+	predicted	---
4	107756820	107756820	+	107756820	3	3	XLOC_061659	+	predicted	---

4	107757446	107757446	+	107757446	4	4	XLOC_061659	+	predicted	---
4	107758539	107758543	+	107758541	20	41	XLOC_061659	+	predicted	---
4	107783581	107783598	+	107783588	30	59	XLOC_061659	+	predicted	---
4	107783672	107783674	+	107783672	7	10	XLOC_061659	+	predicted	---
4	107810533	107810533	+	107810533	41	41	ENSSSCG00000006652	+	ORF	*MCL1*
4	107811475	107811475	+	107811475	7	7	ENSSSCG00000006652	+	3'UTR	*MCL1*
4	107812720	107812722	+	107812722	19	31	XLOC_061660	+	predicted	---
4	107814032	107814040	+	107814033	58	121	XLOC_061660	+	predicted	---
4	107824843	107824849	-	107824849	31	40	ENSSSCG00000006651	-	3'UTR	*ADAMTSL4*
4	107928709	107928710	-	107928709	3	6	XLOC_062734	-	predicted	---
4	107931799	107931799	-	107931799	7	7	XLOC_062734	-	predicted	---
4	107996169	107996175	-	107996173	7	15	ENSSSCG00000030291	-	intron	*RPRD2*
4	108032985	108032986	-	108032986	30	33	ENSSSCG00000030119	-	3'UTR	---
4	108047280	108047280	-	108047280	4	4	ENSSSCG00000030119	-	ORF	---
4	108068052	108068069	-	108068054	22	76	XLOC_062737	-	predicted	---
4	108068123	108068133	-	108068125	28	120	XLOC_062737	-	predicted	---
4	108068205	108068205	-	108068205	3	3	ENSSSCG00000027168	-	ORF	---
4	108088650	108088650	-	108088650	4	4	XLOC_062737	-	predicted	---
4	108092103	108092105	+	108092105	7	10	XLOC_061665	+	predicted	---
4	108197945	108197954	-	108197952	10	23	ENSSSCG00000022784	-	3'UTR	*PLEKHO1*
4	108198394	108198406	-	108198395	11	39	ENSSSCG00000022784	-	3'UTR	*PLEKHO1*
4	108207048	108207049	-	108207048	3	6	ENSSSCG00000022784	-	ORF	*PLEKHO1*
4	108213429	108213429	-	108213429	10	10	ENSSSCG00000006661	-	3'UTR	*VPS45*
4	108563151	108563151	+	108563151	3	3	ENSSSCG00000006667	+	3'UTR	*BOLA1*
							ENSSSCG00000006667	+	ORF	---

4	108563379	108563389	+	108563381	30	73	ENSSSCG00000006667	+	3'UTR	*BOLA1*
							ENSSSCG00000006667	+	ORF	---
4	108590397	108590407	-	108590401	57	165	ENSSSCG00000006665	-	3'UTR	*SF3B4*
4	108608246	108608246	-	108608246	3	3	ENSSSCG00000006663	-	3'UTR	*OTUD7B*
4	108609715	108609715	-	108609715	4	4	ENSSSCG00000006663	-	3'UTR	*OTUD7B*
4	108738624	108738667	-	108738635	36	90	XLOC_062751	-	predicted	---
4	108738941	108738941	-	108738941	3	3	ENSSSCG00000006679	-	intron	---
4	108741528	108741528	-	108741528	10	10	ENSSSCG00000006679	-	intron	---
4	108758872	108758872	-	108758872	5	5	XLOC_062751	-	predicted	---
4	108782546	108782567	+	108782551	28	66	ENSSSCG00000006678	+	3'UTR	*FCGR1A*
4	108786046	108786047	+	108786046	3	6	XLOC_061674	+	predicted	---
4	108791392	108791392	-	108791392	6	6	XLOC_062752	-	predicted	---
4	108795287	108795296	-	108795296	5	12	---	---	intergenic	---
4	108797786	108797796	-	108797788	52	91	ENSSSCG00000022644	-	intron	*HIST2H2BF*
4	108941162	108941162	-	108941162	3	3	---	---	intergenic	---
4	108975319	108975319	+	108975319	13	13	XLOC_061683	+	predicted	---
4	108975444	108975447	+	108975445	14	24	XLOC_061683	+	predicted	---
4	108994543	108994544	+	108994543	5	8	ENSSSCG00000006681	+	3'UTR	*TXNIP*
4	108994947	108994980	+	108994980	15	23	ENSSSCG00000006681	+	3'UTR	*TXNIP*
4	108995557	108995562	+	108995561	66	86	ENSSSCG00000006681	+	3'UTR	*TXNIP*
4	109005756	109005756	-	109005756	5	5	ENSSSCG00000006682	-	3'UTR	*POLR3GL*
4	109046206	109046206	+	109046206	8	8	ENSSSCG00000026502	+	3'UTR	*LIX1L*
4	109052223	109052223	-	109052223	4	4	XLOC_062758	-	predicted	---
4	109052781	109052791	+	109052784	64	144	ENSSSCG00000028509	+	3'UTR	*RBM8A*
4	109054613	109054613	+	109054613	4	4	ENSSSCG00000028509	+	3'UTR	*RBM8A*
4	109064830	109064835	+	109064834	26	77	XLOC_061688	+	predicted	---

4	109092990	109093004	+	109092999	23	30	ENSSSCG00000006688	+	ORF	*ANKRD35*
4	109095020	109095023	+	109095021	24	45	ENSSSCG00000006688	+	ORF	*ANKRD35*
4	109097112	109097144	+	109097112	3	6	ENSSSCG00000006688	+	ORF	*ANKRD35*
4	109097458	109097462	+	109097458	4	7	ENSSSCG00000006688	+	ORF	*ANKRD35*
4	109097686	109097689	+	109097687	7	14	ENSSSCG00000006688	+	ORF	*ANKRD35*
4	109097827	109097837	+	109097828	14	26	ENSSSCG00000006688	+	ORF	*ANKRD35*
4	109097910	109097919	+	109097919	25	44	ENSSSCG00000006688	+	ORF	*ANKRD35*
4	109098439	109098439	+	109098439	3	3	ENSSSCG00000006688	+	ORF	*ANKRD35*
4	109098579	109098587	+	109098585	5	11	ENSSSCG00000006688	+	ORF	*ANKRD35*
4	109098685	109098688	+	109098685	3	6	ENSSSCG00000006688	+	ORF	*ANKRD35*
4	109098966	109098972	+	109098972	7	10	ENSSSCG00000006688	+	intron	*ANKRD35*
4	109102912	109102912	+	109102912	3	3	ENSSSCG00000006688	+	3'UTR	*ANKRD35*
4	109102960	109102971	+	109102964	321	943	ENSSSCG00000006688	+	3'UTR	*ANKRD35*
4	109114441	109114441	-	109114441	7	7	ENSSSCG00000006690	-	3'UTR	*NUDT17*
4	109117228	109117229	+	109117228	3	6	ENSSSCG00000006689	+	3'UTR	*PIAS3*
4	109285803	109285806	+	109285803	17	22	ENSSSCG00000021304	+	3'UTR	---
4	109286290	109286303	-	109286300	17	53	ENSSSCG00000006693	-	3'UTR	*PDZK1*
4	109704698	109704698	+	109704698	3	3	ENSSSCG00000006696	+	3'UTR	*GJA5*
							ENSSSCG00000006696	+	ORF	---
4	109781465	109781467	+	109781466	13	29	ENSSSCG00000006697	+	3'UTR	*ACP6*
4	109804861	109804861	-	109804861	5	5	ENSSSCG00000006698	-	3'UTR	*BCL9*
4	109808895	109808895	-	109808895	4	4	ENSSSCG00000006698	-	intron	*BCL9*
4	109847925	109847925	+	109847925	3	3	---	---	intergenic	---
4	110094569	110094569	-	110094569	3	3	ENSSSCG00000006701	-	3'UTR	*CHD1L*
4	110096644	110096644	-	110096644	3	3	ENSSSCG00000006701	-	3'UTR	*CHD1L*
4	110167006	110167006	+	110167006	3	3	ENSSSCG00000006702	+	ORF	*FMO5*

4	110177886	110177886	+	110177886	10	10	ENSSSCG00000006702	+	ORF	*FMO5*
4	110201294	110201294	+	110201294	4	4	ENSSSCG00000006702	+	3'UTR	*FMO5*
4	110202319	110202320	+	110202319	85	90	ENSSSCG00000006702	+	3'UTR	*FMO5*
4	110202478	110202478	+	110202478	8	8	ENSSSCG00000006702	+	3'UTR	*FMO5*
4	110223921	110223924	+	110223924	5	12	XLOC_061699	+	predicted	---
4	110270420	110270420	-	110270420	3	3	XLOC_062771	-	predicted	---
4	110276537	110276537	-	110276537	9	9	XLOC_062771	-	predicted	---
4	110455912	110455914	+	110455913	17	23	---	---	intergenic	---
4	110733218	110733219	+	110733219	23	26	---	---	intergenic	---
4	110848658	110848666	-	110848660	10	29	ENSSSCG00000020964	-	3'UTR	---
4	110848901	110848901	-	110848901	4	4	ENSSSCG00000020964	-	3'UTR	---
4	110849372	110849372	-	110849372	3	3	ENSSSCG00000020964	-	3'UTR	---
4	110849765	110849774	-	110849766	10	20	ENSSSCG00000020964	-	3'UTR	---
4	111037574	111037580	+	111037574	15	18	XLOC_061705	+	predicted	---
4	111218580	111218580	+	111218580	5	5	---	---	intergenic	---
4	111221806	111221811	+	111221806	3	6	---	---	intergenic	---
4	111256557	111256557	+	111256557	4	4	ENSSSCG00000006712	+	3'UTR	*REG4*
4	111259047	111259047	+	111259047	3	3	XLOC_061710	+	predicted	---
4	111279010	111279010	+	111279010	3	3	---	---	intergenic	---
4	111436316	111436324	-	111436324	49	140	ENSSSCG00000006717	-	3'UTR	*PHGDH*
4	111494841	111494842	+	111494841	7	10	---	---	intergenic	---
4	111542610	111542610	+	111542610	3	3	XLOC_061712	+	predicted	---
4	111555577	111555578	-	111555577	7	11	ENSSSCG00000006719	-	3'UTR	*HSD3B*
4	111579209	111579211	-	111579209	28	39	ENSSSCG00000006720	-	3'UTR	*HAO2*
4	111694152	111694152	-	111694152	6	6	---	---	intergenic	---
4	111922544	111922544	-	111922544	3	3	ENSSSCG00000027153	-	3'UTR	---

4	112055059	112055059	+	112055059	6	6	---	---	intergenic	---
4	112790944	112790948	-	112790944	3	6	XLOC_062793	-	predicted	---
4	112905920	112905920	+	112905920	5	5	ENSSSCG00000006728	+	3'UTR	*GDAP2*
4	113095389	113095390	-	113095389	545	557	XLOC_062796	-	predicted	---
4	113095681	113095681	-	113095681	3	3	XLOC_062796	-	predicted	---
4	113095760	113095760	-	113095760	3	3	XLOC_062796	-	predicted	---
4	113097474	113097482	-	113097476	38	115	XLOC_062796	-	predicted	---
4	113099009	113099009	-	113099009	4	4	XLOC_062796	-	predicted	---
4	113099169	113099169	-	113099169	3	3	ENSSSCG00000006729	-	3'UTR	*FAM46C*
4	113099666	113099671	-	113099667	30	73	ENSSSCG00000006729	-	3'UTR	*FAM46C*
4	113155330	113155330	+	113155330	5	5	---	---	intergenic	---
4	113178798	113178798	-	113178798	7	7	XLOC_062798	-	predicted	---
4	113238438	113238438	-	113238438	3	3	ENSSSCG00000006730	-	intron	*MAN1A2*
4	113396408	113396408	-	113396408	4	4	---	---	intergenic	---
4	113461495	113461495	+	113461495	3	3	---	---	intergenic	---
4	113588006	113588008	+	113588008	12	25	ENSSSCG00000006732	+	3'UTR	*CH242-366F1 0.1*
4	113588522	113588522	+	113588522	4	4	ENSSSCG00000006732	+	3'UTR	*CH242-366F1 0.1*
4	113596175	113596180	-	113596180	4	7	ENSSSCG00000006733	-	3'UTR	*TTF2*
4	113708889	113708889	-	113708889	4	4	XLOC_062802	-	predicted	---
4	113722200	113722200	-	113722200	4	4	ENSSSCG00000006735	-	intron	*PTGFRN*
4	113882856	113882857	+	113882856	12	16	---	---	intergenic	---
4	113928065	113928065	-	113928065	25	25	ENSSSCG00000006736	-	3'UTR	*CD2*
4	114331359	114331359	-	114331359	5	5	---	---	intergenic	---
4	114336430	114336430	-	114336430	4	4	XLOC_062804	-	predicted	---

4	114340610	114340641	-	114340639	34	110	ENSSSCG00000006738	-	3'UTR	*CD58*
4	114405085	114405085	+	114405085	7	7	XLOC_061735	+	predicted	---
4	114431991	114432003	-	114431992	57	109	ENSSSCG00000006740	-	3'UTR	*ATP1A1*
4	114432047	114432047	-	114432047	6	6	ENSSSCG00000006740	-	3'UTR	*ATP1A1*
4	114687809	114687811	-	114687809	3	6	XLOC_062807	-	predicted	---
4	114755036	114755036	-	114755036	3	3	ENSSSCG00000006743	-	3'UTR	*SLC22A15*
4	114795078	114795078	-	114795078	3	3	ENSSSCG00000006743	-	intron	*SLC22A15*
4	115194885	115194885	+	115194885	3	3	---	---	intergenic	---
4	115668230	115668230	+	115668230	4	4	ENSSSCG00000006748	+	intron	*TSPAN2*
4	115679745	115679745	+	115679745	3	3	ENSSSCG00000006748	+	3'UTR	*TSPAN2*
4	115680738	115680738	+	115680738	3	3	XLOC_061745	+	predicted	---
4	115691695	115691695	-	115691695	5	5	ENSSSCG00000006749	-	3'UTR	*TSHB*
4	115731022	115731022	-	115731022	3	3	XLOC_062812	-	predicted	---
4	115837796	115837796	-	115837796	3	3	ENSSSCG00000006750	-	intron	*SYCP1*
4	115848429	115848429	-	115848429	3	3	ENSSSCG00000006750	-	intron	*SYCP1*
							ENSSSCG00000006750	-	ORF	*SYCP1*
4	115872790	115872790	+	115872790	3	3	---	---	intergenic	---
4	115877764	115877764	+	115877764	6	6	XLOC_061746	+	predicted	---
4	115975048	115975048	+	115975048	3	3	XLOC_061747	+	predicted	---
4	115998340	115998340	+	115998340	12	12	---	---	intergenic	---
4	116033362	116033364	+	116033363	4	11	ENSSSCG00000006752	+	3'UTR	*CSDE1*
4	116033407	116033413	+	116033411	40	59	ENSSSCG00000006752	+	3'UTR	*CSDE1*
4	116034104	116034108	+	116034107	41	62	ENSSSCG00000006752	+	3'UTR	*CSDE1*
4	116034479	116034491	+	116034487	50	200	ENSSSCG00000006752	+	3'UTR	*CSDE1*
4	116042665	116042677	+	116042677	4	7	XLOC_061748	+	predicted	---
4	116042951	116042951	+	116042951	9	9	XLOC_061748	+	predicted	---

4	116045974	116045988	+	116045987	16	51	XLOC_061748	+	predicted	---
4	116179375	116179376	+	116179375	5	9	XLOC_061750	+	predicted	---
4	116183914	116183914	+	116183914	3	3	ENSSSCG00000006756	+	ORF	*BCAS2*
4	116185013	116185013	+	116185013	5	5	ENSSSCG00000006756	+	ORF	*BCAS2*
4	116190982	116190992	+	116190983	93	173	ENSSSCG00000006756	+	3'UTR	*BCAS2*
4	116335604	116335604	+	116335604	3	3	ENSSSCG00000006757	+	intron	---
4	116341428	116341428	+	116341428	3	3	ENSSSCG00000006757	+	intron	---
4	116704498	116704520	-	116704502	65	246	ENSSSCG00000006759	-	3'UTR	*OLFML3*
4	116706175	116706175	-	116706175	10	10	ENSSSCG00000006759	-	ORF	*OLFML3*
4	116708470	116708472	-	116708470	5	13	XLOC_062817	-	predicted	---
4	116708931	116708935	-	116708931	17	33	ENSSSCG00000006760	-	3'UTR	*HIPK1*
4	116714683	116714686	-	116714685	16	44	ENSSSCG00000006760	-	intron	*HIPK1*
4	116873390	116873396	+	116873396	11	14	ENSSSCG00000006762	+	3'UTR	*AP4B1*
4	116886107	116886107	+	116886107	5	5	ENSSSCG00000006763	+	3'UTR	*BCL2L15*
4	116950053	116950053	+	116950053	5	5	ENSSSCG00000030154	+	3'UTR	*RSBN1*
4	116953538	116953538	+	116953538	3	3	ENSSSCG00000030154	+	3'UTR	*RSBN1*
4	117022336	117022340	+	117022337	4	17	XLOC_061763	+	predicted	---
4	117166792	117166792	-	117166792	4	4	ENSSSCG00000006767	-	intron	*MAGI3*
4	117496258	117496258	+	117496258	13	13	---	---	intergenic	---
4	117496596	117496596	-	117496596	5	5	XLOC_062823	-	predicted	---
4	117577130	117577131	-	117577130	3	6	XLOC_062825	-	predicted	---
4	117577297	117577300	-	117577300	11	35	XLOC_062825	-	predicted	---
4	117577487	117577499	-	117577497	27	78	XLOC_062825	-	predicted	---
4	117581284	117581285	+	117581284	5	10	---	---	intergenic	---
4	117591444	117591444	-	117591444	3	3	---	---	intergenic	---
4	117616437	117616440	+	117616440	5	9	XLOC_061767	+	predicted	---

4	117616549	117616551	+	117616550	16	23	XLOC_061767	+	predicted	---
4	117616734	117616734	+	117616734	7	7	XLOC_061767	+	predicted	---
4	117816577	117816577	+	117816577	7	7	ENSSSCG00000006775	+	ORF	*CAPZA1*
4	117816667	117816667	+	117816667	4	4	ENSSSCG00000006775	+	3'UTR	*CAPZA1*
4	117818047	117818054	+	117818047	14	25	ENSSSCG00000006775	+	3'UTR	*CAPZA1*
4	117818126	117818130	+	117818128	95	158	ENSSSCG00000006775	+	3'UTR	*CAPZA1*
4	117848405	117848405	+	117848405	3	3	XLOC_061771	+	predicted	---
4	117848790	117848800	-	117848796	134	214	ENSSSCG00000006777	-	3'UTR	*RHOC*
4	117857580	117857598	-	117857581	20	38	---	---	intergenic	---
4	117894203	117894203	+	117894203	8	8	ENSSSCG00000006779	+	intron	*ST7L*
4	117903144	117903145	+	117903144	8	13	ENSSSCG00000006779	+	intron	*ST7L*
4	117957445	117957468	+	117957463	31	87	XLOC_061772	+	predicted	---
4	118026555	118026562	-	118026562	5	8	ENSSSCG00000006781	-	3'UTR	*CTTNBP2NL*
4	118026722	118026723	-	118026722	3	6	ENSSSCG00000006781	-	3'UTR	*CTTNBP2NL*
4	118029286	118029286	-	118029286	3	3	ENSSSCG00000006781	-	ORF	*CTTNBP2NL*
4	118063552	118063552	-	118063552	9	9	ENSSSCG00000006781	-	intron	*CTTNBP2NL*
4	118322623	118322623	-	118322623	3	3	---	---	intergenic	---
4	118423857	118423861	-	118423861	31	60	XLOC_062838	-	predicted	---
4	118449505	118449505	-	118449505	20	20	---	---	intergenic	---
4	118772056	118772059	-	118772058	43	83	ENSSSCG00000006783	-	3'UTR	*DDX20*
4	118773250	118773250	-	118773250	3	3	ENSSSCG00000006783	-	ORF	*DDX20*
4	118819673	118819677	-	118819677	7	10	XLOC_062842	-	predicted	---
4	118823006	118823010	-	118823008	37	59	XLOC_062842	-	predicted	---
4	118823081	118823092	-	118823092	7	11	XLOC_062842	-	predicted	---
4	118823178	118823178	-	118823178	3	3	XLOC_062842	-	predicted	---
4	118887308	118887309	-	118887308	10	16	ENSSSCG00000006785	-	intron	*RAP1A*

4	119039688	119039695	+	119039688	11	23	ENSSSCG00000006788	+	3'UTR	*ADORA3*
							ENSSSCG00000006787	+	intron	*ADORA3*
4	119053002	119053029	+	119053005	138	627	ENSSSCG00000006787	+	3'UTR	*ADORA3*
4	119053280	119053286	+	119053281	8	14	XLOC_061778	+	predicted	---
4	119078634	119078668	-	119078649	558	695	XLOC_062846	-	predicted	---
4	119078811	119078816	-	119078812	48	87	ENSSSCG00000006789	-	3'UTR	*ATP5F1*
4	119085312	119085312	-	119085312	13	13	ENSSSCG00000006789	-	intron	*ATP5F1*
							ENSSSCG00000006789	-	ORF	*ATP5F1*
4	119098506	119098522	+	119098512	20	94	XLOC_061780	+	predicted	---
4	119124269	119124269	+	119124269	7	7	ENSSSCG00000006791	+	3'UTR	*OVGP1*
4	119320217	119320221	-	119320217	8	26	ENSSSCG00000029918	-	3'UTR	*CEPT1*
4	119432659	119432659	-	119432659	3	3	ENSSSCG00000029918	-	intron	*CEPT1*
4	119456994	119456994	-	119456994	6	6	ENSSSCG00000029918	-	intron	*CEPT1*
4	119571469	119571469	+	119571469	10	10	ENSSSCG00000026922	+	3'UTR	*DRAM2*
4	119572074	119572074	+	119572074	3	3	ENSSSCG00000026922	+	3'UTR	*DRAM2*
4	119653870	119653870	+	119653870	3	3	ENSSSCG00000006799	+	ORF	*LRIF1*
4	119656129	119656131	+	119656131	18	22	ENSSSCG00000006799	+	intron	*LRIF1*
4	119657684	119657691	+	119657690	12	21	ENSSSCG00000006799	+	3'UTR	*LRIF1*
4	119657756	119657756	+	119657756	4	4	ENSSSCG00000006799	+	3'UTR	*LRIF1*
4	119659816	119659816	+	119659816	3	3	ENSSSCG00000006799	+	3'UTR	*LRIF1*
4	119674098	119674108	-	119674098	62	96	ENSSSCG00000006800	-	3'UTR	*CD53*
4	119863975	119863975	+	119863975	4	4	XLOC_061794	+	predicted	---
4	120114668	120114668	-	120114668	5	5	---	---	intergenic	---
4	120123239	120123256	+	120123247	109	156	XLOC_061798	+	predicted	---
4	120174544	120174544	-	120174544	17	17	XLOC_062860	-	predicted	---
4	120476237	120476255	-	120476245	81	305	---	---	intergenic	---

4	120477275	120477278	+	120477275	4	7	---	---	intergenic	---
4	120531385	120531385	-	120531385	7	7	XLOC_062865	-	predicted	---
4	120531436	120531445	-	120531445	7	10	XLOC_062865	-	predicted	---
4	120750530	120750543	-	120750531	43	168	XLOC_062868	-	predicted	---
4	120750982	120750994	-	120750992	5	20	XLOC_062868	-	predicted	---
4	120751185	120751185	-	120751185	4	4	XLOC_062868	-	predicted	---
4	120827555	120827586	-	120827566	812	3892	ENSSSCG00000006821	-	3'UTR	*GSTM3*
4	120828051	120828051	-	120828051	6	6	ENSSSCG00000006821	-	intron	*GSTM3*
4	120828671	120828672	-	120828671	12	17	ENSSSCG00000006821	-	intron	*GSTM3*
							ENSSSCG00000006821	-	ORF	*GSTM3*
4	120828848	120828852	-	120828852	8	14	ENSSSCG00000006821	-	intron	*GSTM3*
4	120833586	120833586	-	120833586	3	3	XLOC_062871	-	predicted	---
4	120990184	120990193	-	120990193	8	12	XLOC_062874	-	predicted	---
4	120990901	120990931	-	120990920	23	74	XLOC_062874	-	predicted	---
4	120991447	120991450	-	120991450	4	7	XLOC_062874	-	predicted	---
4	120991566	120991566	-	120991566	3	3	XLOC_062874	-	predicted	---
4	120992182	120992182	-	120992182	3	3	---	---	intergenic	---
4	121041078	121041078	-	121041078	3	3	XLOC_062875	-	predicted	---
4	121085254	121085254	-	121085254	3	3	XLOC_062876	-	predicted	---
4	121091915	121091915	-	121091915	3	3	ENSSSCG00000006827	-	3'UTR	*CYB561D1*
							ENSSSCG00000006827	-	intron	*CYB561D1*
4	121095595	121095595	-	121095595	4	4	ENSSSCG00000006828	-	3'UTR	*ATXN7L2*
4	121226063	121226079	+	121226064	74	85	ENSSSCG00000006830	+	3'UTR	*PSMA5*
4	121295427	121295429	+	121295429	9	19	XLOC_061812	+	predicted	---
4	121297737	121297755	-	121297755	4	7	XLOC_062880	-	predicted	---
4	121322307	121322316	-	121322308	6	15	XLOC_062882	-	predicted	---

4	121326561	121326564	-	121326561	5	8	XLOC_062882	-	predicted	---
4	121326664	121326664	-	121326664	3	3	XLOC_062882	-	predicted	---
4	121326787	121326801	-	121326791	88	183	XLOC_062882	-	predicted	---
4	121350908	121350930	-	121350926	86	135	ENSSSCG00000006835	-	3'UTR	*SARS*
4	121363069	121363074	-	121363069	3	6	ENSSSCG00000006835	-	ORF	*SARS*
4	121375544	121375551	-	121375551	7	10	XLOC_062885	-	predicted	---
4	121378122	121378130	-	121378123	50	64	ENSSSCG00000006836	-	3'UTR	*KIAA1324*
4	121407386	121407386	-	121407386	3	3	ENSSSCG00000006836	-	ORF	*KIAA1324*
4	121456353	121456361	+	121456360	53	140	XLOC_061817	+	predicted	---
4	121460287	121460290	-	121460290	7	19	XLOC_062887	-	predicted	---
4	121500727	121500727	-	121500727	7	7	---	---	intergenic	---
4	121535228	121535228	+	121535228	3	3	ENSSSCG00000006840	+	ORF	*WDR47*
4	121569039	121569039	+	121569039	8	8	ENSSSCG00000006840	+	3'UTR	*WDR47*
4	121576716	121576716	+	121576716	3	3	ENSSSCG00000006841	+	ORF	---
4	121592908	121592908	+	121592908	14	14	ENSSSCG00000006841	+	3'UTR	---
4	121594275	121594275	-	121594275	4	4	XLOC_062889	-	predicted	---
4	121720755	121720755	+	121720755	3	3	ENSSSCG00000006842	+	intron	---
4	121722406	121722413	+	121722412	11	33	ENSSSCG00000006842	+	intron	---
4	121729803	121729804	-	121729804	5	9	ENSSSCG00000006843	-	3'UTR	*STXBP3*
4	121730707	121730707	-	121730707	6	6	ENSSSCG00000006843	-	3'UTR	*STXBP3*
4	121745181	121745181	-	121745181	3	3	ENSSSCG00000006843	-	intron	*STXBP3*
4	121789277	121789277	-	121789277	3	3	ENSSSCG00000006843	-	intron	*STXBP3*
4	121836907	121836910	-	121836907	8	12	ENSSSCG00000006845	-	3'UTR	*PRPF38B*
4	121837545	121837546	-	121837545	3	6	ENSSSCG00000006845	-	3'UTR	*PRPF38B*
4	121838364	121838364	-	121838364	6	6	ENSSSCG00000006845	-	3'UTR	*PRPF38B*
4	121838645	121838645	-	121838645	8	8	ENSSSCG00000006845	-	3'UTR	*PRPF38B*

4	121839240	121839240	-	121839240	3	3	ENSSSCG00000006845	-	ORF	*PRPF38B*
4	121839318	121839318	-	121839318	4	4	ENSSSCG00000006845	-	ORF	*PRPF38B*
4	121848867	121848883	+	121848877	17	81	XLOC_061826	+	predicted	---
4	121859798	121859798	-	121859798	11	11	XLOC_062894	-	predicted	---
4	121883970	121883970	+	121883970	10	10	---	---	intergenic	---
4	122075048	122075049	+	122075048	51	65	---	---	intergenic	---
4	122094593	122094593	+	122094593	4	4	ENSSSCG00000006848	+	ORF	*SCAMC-1*
4	122967692	122967692	+	122967692	5	5	ENSSSCG00000023187	+	3'UTR	*VAV3*
4	123123799	123123799	-	123123799	3	3	ENSSSCG00000006852	-	intron	*NTNG1*
4	123412561	123412561	+	123412561	3	3	XLOC_061836	+	predicted	---
4	123419650	123419650	+	123419650	5	5	XLOC_061836	+	predicted	---
4	123525153	123525153	+	123525153	3	3	---	---	intergenic	---
4	123555703	123555703	-	123555703	3	3	---	---	intergenic	---
4	124295938	124295939	-	124295938	9	16	---	---	intergenic	---
4	124956119	124956125	+	124956119	6	12	---	---	intergenic	---
4	125489488	125489488	-	125489488	3	3	---	---	intergenic	---
4	125646917	125646917	-	125646917	3	3	---	---	intergenic	---
4	126092351	126092357	-	126092357	7	14	---	---	intergenic	---
4	126343465	126343465	-	126343465	4	4	---	---	intergenic	---
4	126477650	126477650	+	126477650	3	3	---	---	intergenic	---
4	126571349	126571349	-	126571349	4	4	---	---	intergenic	---
4	127183163	127183163	+	127183163	4	4	---	---	intergenic	---
4	127447887	127447887	+	127447887	4	4	---	---	intergenic	---
4	128432684	128432684	-	128432684	7	7	---	---	intergenic	---
4	128478773	128478774	+	128478773	39	55	---	---	intergenic	---
4	128705122	128705122	-	128705122	5	5	XLOC_062916	-	predicted	---

4	128846958	128846964	+	128846959	10	34	ENSSSCG00000006859	+	3'UTR	*DPH5*
4	128858092	128858102	-	128858092	4	7	XLOC_062918	-	predicted	---
4	128860268	128860275	+	128860268	3	6	XLOC_061850	+	predicted	---
4	128862821	128862821	-	128862821	4	4	XLOC_062918	-	predicted	---
4	128961871	128961885	+	128961879	18	58	XLOC_061852	+	predicted	---
4	129040265	129040271	-	129040265	8	15	ENSSSCG00000006862	-	3'UTR	*VCAM1*
4	129230516	129230516	-	129230516	5	5	XLOC_062922	-	predicted	---
4	129243030	129243030	-	129243030	3	3	---	---	intergenic	---
4	129251379	129251380	-	129251379	8	11	---	---	intergenic	---
4	129473156	129473156	-	129473156	6	6	ENSSSCG00000006864	-	intron	*CDC14A*
4	129473818	129473820	-	129473819	76	112	ENSSSCG00000006865	-	intron	*RTCA*
							ENSSSCG00000006864	-	intron	*CDC14A*
4	129507309	129507309	-	129507309	6	6	---	---	intergenic	---
4	129617939	129617939	-	129617939	3	3	---	---	intergenic	---
4	129621944	129621944	-	129621944	3	3	---	---	intergenic	---
4	129663116	129663116	+	129663116	3	3	XLOC_061858	+	predicted	---
4	129665335	129665340	+	129665335	12	19	XLOC_061858	+	predicted	---
4	129688587	129688587	-	129688587	3	3	---	---	intergenic	---
4	129713745	129713745	+	129713745	3	3	---	---	intergenic	---
4	129747132	129747132	+	129747132	3	3	XLOC_061859	+	predicted	---
4	129747196	129747196	+	129747196	4	4	XLOC_061859	+	predicted	---
4	129747471	129747471	+	129747471	5	5	XLOC_061859	+	predicted	---
4	129940638	129940638	-	129940638	14	14	ENSSSCG00000006874	-	3'UTR	*PALMD*
4	129990756	129990756	-	129990756	16	16	ENSSSCG00000006874	-	intron	*PALMD*
4	129995196	129995196	+	129995196	3	3	---	---	intergenic	---
4	130138320	130138320	-	130138320	5	5	---	---	intergenic	---

4	130248164	130248164	-	130248164	3	3	---	---	intergenic	---
4	130380606	130380606	+	130380606	3	3	---	---	intergenic	---
4	131340710	131340711	-	131340710	9	16	---	---	intergenic	---
4	131748484	131748484	+	131748484	5	5	---	---	intergenic	---
4	132526637	132526637	+	132526637	3	3	ENSSSCG00000006878	+	ORF	*DPYD*
4	132549941	132549941	+	132549941	4	4	ENSSSCG00000006878	+	3'UTR	*DPYD*
4	132550017	132550017	+	132550017	30	30	ENSSSCG00000006878	+	3'UTR	*DPYD*
4	132566115	132566116	+	132566115	11	17	---	---	intergenic	---
4	132566685	132566685	+	132566685	4	4	---	---	intergenic	---
4	132578800	132578800	-	132578800	7	7	---	---	intergenic	---
4	132641441	132641441	+	132641441	3	3	---	---	intergenic	---
4	132682905	132682905	+	132682905	3	3	---	---	intergenic	---
4	132885519	132885519	+	132885519	3	3	XLOC_061870	+	predicted	---
4	133191626	133191626	-	133191626	4	4	---	---	intergenic	---
4	133421141	133421141	-	133421141	3	3	XLOC_062947	-	predicted	---
4	133428220	133428220	+	133428220	4	4	---	---	intergenic	---
4	133680871	133680871	-	133680871	3	3	XLOC_062948	-	predicted	---
4	133683846	133683846	-	133683846	3	3	XLOC_062948	-	predicted	---
4	133873126	133873126	-	133873126	3	3	---	---	intergenic	---
4	133917218	133917220	-	133917219	61	82	---	---	intergenic	---
4	133997938	133997945	-	133997945	4	7	ENSSSCG00000006883	-	intron	*RWDD3*
4	134020572	134020572	+	134020572	5	5	---	---	intergenic	---
4	134021348	134021350	+	134021349	20	36	---	---	intergenic	---
4	134027568	134027568	-	134027568	3	3	XLOC_062950	-	predicted	---
4	134059352	134059353	-	134059352	7	11	---	---	intergenic	---
4	134103513	134103514	+	134103514	12	16	ENSSSCG00000006885	+	intron	*ALG14*

4	134191815	134191836	+	134191816	5	17	ENSSSCG00000006885	+	intron	*ALG14*
4	134245269	134245270	-	134245269	7	14	XLOC_062951	-	predicted	---
4	134245517	134245520	-	134245519	10	26	ENSSSCG00000006887	-	3'UTR	*CNN3*
4	134245798	134245798	+	134245798	11	11	ENSSSCG00000021743	+	intron	---
4	134294595	134294595	-	134294595	5	5	---	---	intergenic	---
4	134476650	134476655	+	134476655	4	7	ENSSSCG00000022447	+	ORF	*F3*
4	134479706	134479711	+	134479707	11	23	ENSSSCG00000022447	+	3'UTR	*F3*
4	134489526	134489527	-	134489527	6	9	---	---	intergenic	---
4	134489964	134489968	-	134489965	17	41	ENSSSCG00000028620	-	3'UTR	*ABCD3*
4	134490266	134490266	-	134490266	8	8	ENSSSCG00000028620	-	3'UTR	*ABCD3*
4	134490976	134491010	-	134491005	17	42	ENSSSCG00000028620	-	3'UTR	*ABCD3*
4	134491048	134491105	-	134491054	21	139	ENSSSCG00000028620	-	3'UTR	*ABCD3*
4	134802813	134802821	+	134802820	17	28	ENSSSCG00000006889	+	3'UTR	---
4	134978712	134978712	+	134978712	3	3	XLOC_061884	+	predicted	---
4	135032508	135032508	+	135032508	3	3	ENSSSCG00000006891	+	3'UTR	*GCLM*
4	135035882	135035886	+	135035886	16	20	XLOC_061885	+	predicted	---
4	135053868	135053870	+	135053869	8	16	ENSSSCG00000006892	+	3'UTR	*DNTTIP2*
4	135054042	135054042	+	135054042	5	5	ENSSSCG00000006892	+	3'UTR	*DNTTIP2*
4	135055496	135055500	+	135055496	6	11	ENSSSCG00000006892	+	3'UTR	*DNTTIP2*
4	135057797	135057797	-	135057797	3	3	XLOC_062960	-	predicted	---
4	135058112	135058119	-	135058118	8	16	XLOC_062960	-	predicted	---
4	135068462	135068464	-	135068462	5	14	XLOC_062960	-	predicted	---
4	135068651	135068651	-	135068651	3	3	XLOC_062960	-	predicted	---
4	135068924	135068924	-	135068924	3	3	XLOC_062960	-	predicted	---
4	135321936	135321936	+	135321936	5	5	XLOC_061888	+	predicted	---
4	135322612	135322612	+	135322612	3	3	XLOC_061888	+	predicted	---

4	135331074	135331079	-	135331075	7	16	ENSSSCG00000006894	-	3'UTR	*FNBP1L*
4	135471127	135471140	-	135471127	6	9	ENSSSCG00000028310	-	3'UTR	*DR1*
4	135471498	135471500	-	135471498	14	21	ENSSSCG00000028310	-	3'UTR	*DR1*
4	135542391	135542419	-	135542391	12	32	---	---	intergenic	---
4	135544485	135544486	-	135544485	3	6	ENSSSCG00000021670	-	intron	---
4	135581597	135581597	+	135581597	4	4	ENSSSCG00000006896	+	3'UTR	*TMED5*
4	135583690	135583690	+	135583690	3	3	XLOC_061889	+	predicted	---
4	135584655	135584655	+	135584655	4	4	XLOC_061889	+	predicted	---
4	135584919	135584927	+	135584927	26	43	XLOC_061889	+	predicted	---
4	135587189	135587189	-	135587189	4	4	---	---	intergenic	---
4	135644718	135644718	-	135644718	3	3	ENSSSCG00000006897	-	intron	---
							ENSSSCG00000006898	-	ORF	---
4	135675156	135675169	-	135675168	5	11	ENSSSCG00000006898	-	intron	*MTF2*
							ENSSSCG00000006897	-	ORF	---
4	135707029	135707029	-	135707029	3	3	---	---	intergenic	---
4	135753150	135753150	+	135753150	3	3	---	---	intergenic	---
4	135808824	135808824	-	135808824	32	32	---	---	intergenic	---
4	135842041	135842041	+	135842041	3	3	---	---	intergenic	---
4	135981645	135981651	+	135981645	4	8	XLOC_061892	+	predicted	---
4	135982694	135982694	-	135982694	7	7	XLOC_062971	-	predicted	---
4	135982735	135982758	-	135982746	632	1224	ENSSSCG00000006899	-	3'UTR	*RPL5*
4	135982805	135982805	-	135982805	5	5	ENSSSCG00000006899	-	ORF	*RPL5*
4	135983138	135983139	+	135983138	10	13	XLOC_061892	+	predicted	---
4	135983489	135983489	-	135983489	18	18	ENSSSCG00000006899	-	ORF	*RPL5*
4	135984298	135984339	-	135984298	13	21	ENSSSCG00000006899	-	ORF	*RPL5*
4	135984389	135984389	-	135984389	10	10	ENSSSCG00000006899	-	ORF	*RPL5*

4	135986619	135986619	-	135986619	3	3	ENSSSCG00000006899	-	ORF	*RPL5*
4	135986707	135986707	-	135986707	4	4	ENSSSCG00000006899	-	ORF	*RPL5*
4	135989170	135989194	-	135989194	34	37	ENSSSCG00000006899	-	ORF	*RPL5*
4	135990568	135990587	+	135990587	17	46	---	---	intergenic	---
4	136025838	136025838	-	136025838	3	3	---	---	intergenic	---
4	136112914	136112919	+	136112917	7	14	ENSSSCG00000006901	+	intron	---
4	136159145	136159145	+	136159145	4	4	---	---	intergenic	---
4	136224917	136224917	+	136224917	3	3	XLOC_061893	+	predicted	---
4	136229025	136229027	+	136229027	7	12	XLOC_061893	+	predicted	---
4	136360273	136360273	-	136360273	13	13	ENSSSCG00000006903	-	3'UTR	*RPAP2*
4	136428445	136428445	-	136428445	4	4	ENSSSCG00000006903	-	intron	*RPAP2*
4	136542563	136542565	+	136542564	152	245	---	---	intergenic	---
4	136543622	136543622	+	136543622	4	4	XLOC_061897	+	predicted	---
4	136603399	136603399	-	136603399	7	7	ENSSSCG00000023654	-	3'UTR	---
4	136603475	136603475	-	136603475	4	4	ENSSSCG00000023654	-	ORF	---
4	136625644	136625651	-	136625650	93	158	XLOC_062978	-	predicted	---
4	136640097	136640097	+	136640097	3	3	ENSSSCG00000006908	+	intron	---
4	136644513	136644513	-	136644513	3	3	---	---	intergenic	---
4	136891960	136891960	+	136891960	4	4	XLOC_061898	+	predicted	---
4	136893906	136893906	+	136893906	6	6	XLOC_061898	+	predicted	---
4	136993912	136993914	+	136993914	5	11	XLOC_061905	+	predicted	---
4	136994507	136994507	+	136994507	3	3	XLOC_061905	+	predicted	---
4	136997188	136997188	+	136997188	3	3	XLOC_061905	+	predicted	---
4	137187995	137187995	+	137187995	5	5	XLOC_061908	+	predicted	---
4	137853750	137853750	-	137853750	6	6	---	---	intergenic	---
4	138017480	138017492	-	138017480	12	15	---	---	intergenic	---

4	138397288	138397288	-	138397288	3	3	---	---	intergenic	---
4	138450364	138450364	+	138450364	3	3	---	---	intergenic	---
4	138546489	138546489	+	138546489	5	5	---	---	intergenic	---
4	138558555	138558555	-	138558555	4	4	ENSSSCG00000029402	-	intron	*ZNF326*
4	138629127	138629132	+	138629127	8	12	XLOC_061918	+	predicted	---
4	138752527	138752527	+	138752527	3	3	---	---	intergenic	---
4	139001105	139001105	-	139001105	4	4	ENSSSCG00000006917	-	3'UTR	*LRRC8C*
4	139025725	139025725	-	139025725	4	4	ENSSSCG00000006917	-	intron	*LRRC8C*
4	139111052	139111052	-	139111052	5	5	ENSSSCG00000022798	-	3'UTR	*LRRC8B*
4	139111470	139111470	-	139111470	10	10	ENSSSCG00000022798	-	3'UTR	*LRRC8B*
4	139111715	139111722	-	139111722	20	26	ENSSSCG00000022798	-	3'UTR	*LRRC8B*
4	139489863	139489863	+	139489863	3	3	XLOC_061927	+	predicted	---
4	139601152	139601159	+	139601152	5	10	ENSSSCG00000006919	+	3'UTR	*GBP4*
4	139601979	139601987	+	139601987	10	17	XLOC_061925	+	predicted	---
4	139612354	139612364	-	139612358	5	12	ENSSSCG00000006923	-	3'UTR	*GBP2*
4	139659706	139659706	+	139659706	3	3	ENSSSCG00000006924	+	intron	*GBP1*
4	139666840	139666840	+	139666840	5	5	ENSSSCG00000006924	+	3'UTR	*GBP1*
4	139667113	139667113	+	139667113	3	3	ENSSSCG00000006924	+	3'UTR	*GBP1*
4	139667264	139667273	+	139667265	37	98	ENSSSCG00000006924	+	3'UTR	*GBP1*
4	139677542	139677543	+	139677542	3	6	---	---	intergenic	---
4	139732899	139732906	+	139732901	21	36	XLOC_061929	+	predicted	---
4	139777213	139777213	+	139777213	4	4	ENSSSCG00000006926	+	ORF	*GTF2B*
4	139784740	139784760	+	139784740	56	65	XLOC_061930	+	predicted	---
4	139805896	139805896	-	139805896	3	3	ENSSSCG00000006927	-	3'UTR	*PKN2*
4	140081984	140081984	+	140081984	4	4	---	---	intergenic	---
4	140123146	140123146	+	140123146	3	3	XLOC_061935	+	predicted	---

4	140131048	140131048	-	140131048	3	3	XLOC_063014	-	predicted	---
4	140171411	140171411	+	140171411	4	4	---	---	intergenic	---
4	141166417	141166417	+	141166417	4	4	XLOC_061943	+	predicted	---
4	141351450	141351450	-	141351450	4	4	ENSSSCG00000006928	-	intron	*LMO4*
4	141444224	141444233	+	141444232	51	125	XLOC_061944	+	predicted	---
4	141551558	141551558	-	141551558	6	6	ENSSSCG00000022032	-	3'UTR	---
4	141553771	141553773	-	141553773	4	7	ENSSSCG00000022032	-	3'UTR	---
4	141735547	141735556	+	141735549	40	90	ENSSSCG00000030125	+	3'UTR	*SEPT15*
4	141799691	141799691	+	141799691	3	3	XLOC_061946	+	predicted	---
4	141805473	141805480	+	141805475	22	50	XLOC_061946	+	predicted	---
4	141805593	141805593	-	141805593	3	3	XLOC_063024	-	predicted	---
4	141809639	141809648	-	141809642	155	322	ENSSSCG00000006930	-	3'UTR	*SH3GLB1*
4	141816340	141816340	-	141816340	6	6	ENSSSCG00000006930	-	intron	*SH3GLB1*
4	141827838	141827839	-	141827838	13	23	ENSSSCG00000006930	-	ORF	*SH3GLB1*
4	142042252	142042252	+	142042252	3	3	---	---	intergenic	---
4	142576208	142576208	+	142576208	3	3	---	---	intergenic	---
4	142666707	142666707	+	142666707	3	3	ENSSSCG00000006939	+	ORF	*ZNHIT6*
4	142739802	142739803	+	142739802	13	24	ENSSSCG00000006939	+	intron	*ZNHIT6*
4	142749247	142749247	+	142749247	3	3	ENSSSCG00000006939	+	intron	*ZNHIT6*
4	142767862	142767864	+	142767864	8	15	ENSSSCG00000006939	+	intron	*ZNHIT6*
4	142855501	142855501	+	142855501	5	5	ENSSSCG00000006939	+	intron	*ZNHIT6*
4	142875038	142875041	+	142875038	18	32	ENSSSCG00000006939	+	3'UTR	*ZNHIT6*
4	142933001	142933001	+	142933001	5	5	XLOC_061956	+	predicted	---
4	142933701	142933701	+	142933701	5	5	XLOC_061956	+	predicted	---
4	142937677	142937711	+	142937677	4	7	XLOC_061956	+	predicted	---
4	143206481	143206481	+	143206481	7	7	XLOC_061963	+	predicted	---

4	143209096	143209099	+	143209099	50	89	XLOC_061963	+	predicted	---
5	46019	46019	+	46019	3	3	XLOC_066458	+	predicted	---
5	47279	47279	+	47279	3	3	XLOC_066458	+	predicted	---
5	335213	335213	-	335213	3	3	---	---	intergenic	---
5	339579	339579	+	339579	5	5	XLOC_066459	+	predicted	---
5	490495	490495	+	490495	8	8	XLOC_066461	+	predicted	---
5	496547	496553	-	496547	7	10	ENSSSCG00000000005	-	3'UTR	---
5	508146	508154	-	508152	52	171	XLOC_067424	-	predicted	---
5	510874	510874	-	510874	4	4	ENSSSCG00000000004	-	ORF	*PKDREJ*
5	543407	543412	+	543411	42	114	XLOC_066463	+	predicted	---
5	809349	809358	-	809356	35	69	---	---	intergenic	---
5	1071068	1071071	-	1071070	30	61	XLOC_067431	-	predicted	---
5	1071504	1071504	-	1071504	3	3	XLOC_067431	-	predicted	---
5	1075884	1075884	-	1075884	3	3	ENSSSCG00000000010	-	intron	---
5	1105989	1106008	-	1105994	45	133	ENSSSCG00000000010	-	intron	---
5	1200139	1200153	-	1200151	6	25	---	---	intergenic	---
5	1201492	1201492	-	1201492	3	3	ENSSSCG00000024718	-	ORF	---
5	1267693	1267694	+	1267694	4	7	ENSSSCG00000000013	+	intron	---
5	1280731	1280731	+	1280731	7	7	XLOC_066470	+	predicted	---
5	1280774	1280777	+	1280775	6	13	XLOC_066470	+	predicted	---
5	1284077	1284079	-	1284078	17	27	XLOC_067434	-	predicted	---
5	1298072	1298072	-	1298072	3	3	ENSSSCG00000000014	-	intron	*FAM118A*
5	1475030	1475030	+	1475030	4	4	---	---	intergenic	---
5	1503183	1503190	+	1503188	62	112	ENSSSCG00000000018	+	3'UTR	---
5	1507421	1507421	+	1507421	3	3	XLOC_066472	+	predicted	---
5	1509347	1509355	-	1509353	12	21	XLOC_067438	-	predicted	---

5	1512764	1512792	-	1512767	168	318	XLOC_067438	-	predicted	---
5	1522515	1522516	-	1522515	3	6	ENSSSCG00000000019	-	intron	---
5	1531527	1531535	+	1531535	10	17	XLOC_066474	+	predicted	---
5	1656877	1656877	-	1656877	3	3	---	---	intergenic	---
5	1845324	1845347	+	1845324	6	32	XLOC_066476	+	predicted	---
5	1846416	1846423	+	1846417	7	24	XLOC_066476	+	predicted	---
5	1875036	1875036	+	1875036	4	4	---	---	intergenic	---
5	2009984	2009984	+	2009984	3	3	XLOC_066480	+	predicted	---
5	2010102	2010102	-	2010102	3	3	---	---	intergenic	---
5	2158153	2158153	+	2158153	7	7	XLOC_066482	+	predicted	---
5	2198761	2198761	+	2198761	3	3	---	---	intergenic	---
5	2292232	2292232	+	2292232	8	8	---	---	intergenic	---
5	2403053	2403053	-	2403053	3	3	---	---	intergenic	---
5	3141342	3141342	+	3141342	4	4	XLOC_066490	+	predicted	---
5	3141727	3141730	+	3141729	28	47	XLOC_066490	+	predicted	---
5	3145786	3145792	-	3145791	81	192	ENSSSCG00000000033	-	3'UTR	*TSPO*
5	3172812	3172818	+	3172812	10	13	ENSSSCG00000000031	+	3'UTR	*MCAT*
							ENSSSCG00000000031	+	ORF	---
5	3175298	3175302	-	3175302	19	45	XLOC_067452	-	predicted	---
5	3175366	3175366	-	3175366	3	3	XLOC_067452	-	predicted	---
5	3215740	3215740	-	3215740	3	3	---	---	intergenic	---
5	3250362	3250371	+	3250369	26	44	ENSSSCG00000000035	+	3'UTR	*TTLL1*
5	3358648	3358651	+	3358648	26	40	ENSSSCG00000000036	+	3'UTR	*PACSIN2*
5	3477479	3477484	+	3477479	7	13	ENSSSCG00000000037	+	intron	*ARFGAP3*
5	3497452	3497453	+	3497452	22	29	ENSSSCG00000000037	+	3'UTR	*ARFGAP3*
5	3498029	3498037	+	3498034	33	92	ENSSSCG00000000037	+	3'UTR	*ARFGAP3*

5	3514186	3514186	+	3514186	3	3	---	---	intergenic	---
5	3563072	3563082	+	3563075	7	18	XLOC_066500	+	predicted	---
5	3628125	3628142	+	3628131	80	166	ENSSSCG00000000038	+	3'UTR	*CYB5R3*
5	3752007	3752011	+	3752009	65	111	---	---	intergenic	---
5	3810424	3810450	-	3810427	703	1115	ENSSSCG00000000046	-	3'UTR	*CYP2D6*
5	3812030	3812030	-	3812030	6	6	ENSSSCG00000000046	-	ORF	*CYP2D6*
5	3819139	3819139	-	3819139	4	4	XLOC_067461	-	predicted	---
5	3821269	3821276	+	3821269	4	7	---	---	intergenic	---
5	4177919	4177919	-	4177919	13	13	XLOC_067466	-	predicted	---
5	4248429	4248443	-	4248429	5	15	XLOC_067468	-	predicted	---
5	4251164	4251164	-	4251164	4	4	ENSSSCG00000000056	-	ORF	---
5	4270375	4270376	+	4270375	20	23	ENSSSCG00000000058	+	3'UTR	*NHP2L1*
5	4271097	4271097	+	4271097	6	6	ENSSSCG00000000058	+	3'UTR	*NHP2L1*
5	4271189	4271203	+	4271199	13	34	ENSSSCG00000000058	+	3'UTR	*NHP2L1*
5	4274584	4274593	-	4274586	17	39	ENSSSCG00000000059	-	3'UTR	*XRCC6*
5	4323373	4323379	+	4323379	4	10	ENSSSCG00000000060	+	3'UTR	*DESI1*
5	4323531	4323539	+	4323532	10	38	ENSSSCG00000000060	+	3'UTR	*DESI1*
5	4325773	4325795	+	4325775	16	75	XLOC_066512	+	predicted	---
5	4326121	4326121	-	4326121	6	6	---	---	intergenic	---
5	4347669	4347675	+	4347669	4	12	ENSSSCG00000000061	+	3'UTR	*PMM1*
5	4347916	4347920	-	4347916	10	17	XLOC_067471	-	predicted	---
5	4365653	4365653	-	4365653	4	4	---	---	intergenic	---
5	4384906	4384911	+	4384910	18	42	ENSSSCG00000000063	+	3'UTR	*POLR3H*
5	4385842	4385860	-	4385856	32	150	ENSSSCG00000000064	-	3'UTR	*ACO2*
5	4385944	4385952	-	4385951	9	20	ENSSSCG00000000064	-	3'UTR	*ACO2*
5	4385989	4385990	+	4385990	14	18	ENSSSCG00000000063	+	3'UTR	*POLR3H*

5	4393310	4393310	-	4393310	8	8	ENSSSCG00000000064	-	intron	*ACO2*
5	4395928	4395928	-	4395928	3	3	ENSSSCG00000000064	-	intron	*ACO2*
5	4501763	4501763	-	4501763	3	3	---	---	intergenic	---
5	4529150	4529150	+	4529150	3	3	ENSSSCG00000000066	+	3'UTR	*L3MBTL2*
5	4545684	4545700	-	4545697	26	54	ENSSSCG00000028202	-	3'UTR	*RANGAP1*
5	4546347	4546351	-	4546349	55	126	ENSSSCG00000028202	-	3'UTR	*RANGAP1*
5	4574026	4574029	-	4574028	11	24	ENSSSCG00000000068	-	3'UTR	*EP300*
5	4763908	4763908	+	4763908	3	3	ENSSSCG00000000071	+	ORF	*ST13*
5	4775158	4775161	+	4775161	4	7	ENSSSCG00000000071	+	ORF	*ST13*
5	4787595	4787595	+	4787595	3	3	ENSSSCG00000000071	+	intron	*ST13*
5	4788991	4788998	+	4788991	9	12	ENSSSCG00000000071	+	ORF	*ST13*
5	4795860	4795860	+	4795860	4	4	ENSSSCG00000000071	+	ORF	*ST13*
5	4795961	4795972	+	4795961	19	35	ENSSSCG00000000071	+	3'UTR	*ST13*
5	4796221	4796224	+	4796222	51	98	ENSSSCG00000000071	+	3'UTR	*ST13*
5	4796340	4796388	+	4796340	12	26	ENSSSCG00000000071	+	3'UTR	*ST13*
5	4796681	4796699	+	4796681	26	88	XLOC_066519	+	predicted	---
5	4798055	4798065	+	4798063	37	126	XLOC_066519	+	predicted	---
5	4849893	4849899	+	4849893	10	16	ENSSSCG00000000072	+	3'UTR	*SLC25A17*
5	4850123	4850127	+	4850123	4	10	ENSSSCG00000000072	+	3'UTR	*SLC25A17*
5	4850167	4850187	+	4850171	56	113	ENSSSCG00000000072	+	3'UTR	*SLC25A17*
5	4850235	4850250	+	4850238	10	22	XLOC_066520	+	predicted	---
5	5005376	5005384	-	5005384	4	7	---	---	intergenic	---
5	5012064	5012064	-	5012064	6	6	---	---	intergenic	---
5	5024280	5024281	-	5024280	4	8	---	---	intergenic	---
5	5040255	5040255	-	5040255	3	3	---	---	intergenic	---
5	5040283	5040283	+	5040283	3	3	---	---	intergenic	---

5	5141979	5141979	+	5141979	3	3	---	---	intergenic	---
5	5208382	5208382	+	5208382	5	5	XLOC_066524	+	predicted	---
5	5208554	5208556	-	5208554	5	8	XLOC_067487	-	predicted	---
5	5260209	5260253	-	5260251	23	53	ENSSSCG00000000077	-	3'UTR	*ADSL*
5	5284974	5284974	-	5284974	9	9	XLOC_067489	-	predicted	---
5	5287585	5287585	+	5287585	3	3	XLOC_066528	+	predicted	---
5	5373256	5373256	-	5373256	4	4	ENSSSCG00000000078	-	intron	---
5	5484862	5484862	-	5484862	3	3	---	---	intergenic	---
5	5493603	5493604	-	5493603	4	7	---	---	intergenic	---
5	5606940	5606943	+	5606940	9	17	---	---	intergenic	---
5	5607373	5607373	+	5607373	3	3	---	---	intergenic	---
5	5608010	5608010	+	5608010	3	3	---	---	intergenic	---
5	5617724	5617724	-	5617724	3	3	XLOC_067491	-	predicted	---
5	5618960	5618960	-	5618960	3	3	XLOC_067491	-	predicted	---
5	5668664	5668664	-	5668664	3	3	ENSSSCG00000000080	-	3'UTR	*GRAP2*
5	5852782	5852782	+	5852782	5	5	XLOC_066531	+	predicted	---
5	5853314	5853314	+	5853314	16	16	XLOC_066531	+	predicted	---
5	6038397	6038419	+	6038413	30	129	ENSSSCG00000000083	+	3'UTR	*RPS19BP1*
5	6042577	6042588	-	6042584	165	339	ENSSSCG00000000084	-	3'UTR	*ATF4*
5	6047785	6047785	-	6047785	3	3	XLOC_067498	-	predicted	---
5	6048714	6048714	-	6048714	3	3	ENSSSCG00000024216	-	3'UTR	*MIEF1*
5	6049360	6049361	-	6049360	4	8	ENSSSCG00000024216	-	3'UTR	*MIEF1*
5	6050540	6050540	-	6050540	5	5	ENSSSCG00000024216	-	3'UTR	*MIEF1*
5	6050670	6050675	-	6050675	9	12	ENSSSCG00000024216	-	ORF	*MIEF1*
5	6080308	6080308	-	6080308	3	3	XLOC_067498	-	predicted	---
5	6122720	6122728	-	6122728	5	9	ENSSSCG00000000087	-	3'UTR	*TAB1*

5	6165107	6165107	-	6165107	4	4	ENSSSCG00000020873	-	3'UTR	*SYNGR1*
5	6171864	6171870	-	6171867	29	64	ENSSSCG00000020873	-	intron	*SYNGR1*
5	6197603	6197604	-	6197603	11	18	---	---	intergenic	---
5	6210816	6210826	-	6210818	38	116	---	---	intergenic	---
5	6219654	6219654	+	6219654	3	3	ENSSSCG00000000089	+	intron	*RPL3*
5	6222250	6222250	+	6222250	3	3	ENSSSCG00000000089	+	ORF	*RPL3*
5	6223988	6224001	+	6223992	492	1191	ENSSSCG00000000089	+	3'UTR	*RPL3*
5	6290920	6290920	+	6290920	3	3	---	---	intergenic	---
5	6372419	6372419	+	6372419	4	4	XLOC_066540	+	predicted	---
5	6517499	6517499	+	6517499	3	3	XLOC_066544	+	predicted	---
5	6535536	6535536	+	6535536	3	3	XLOC_066544	+	predicted	---
5	6536710	6536717	+	6536716	91	181	XLOC_066544	+	predicted	---
5	6537794	6537794	-	6537794	3	3	XLOC_067510	-	predicted	---
5	6538855	6538861	-	6538861	15	29	ENSSSCG00000000095	-	3'UTR	*GTPBP1*
5	6564631	6564631	+	6564631	3	3	---	---	intergenic	---
5	6579628	6579634	-	6579634	48	80	ENSSSCG00000000097	-	3'UTR	*TOMM22*
5	6580208	6580214	-	6580208	4	7	ENSSSCG00000000097	-	3'UTR	*TOMM22*
5	6591180	6591186	-	6591180	4	10	XLOC_067512	-	predicted	---
5	6605569	6605569	+	6605569	3	3	---	---	intergenic	---
5	6697173	6697173	+	6697173	3	3	XLOC_066546	+	predicted	---
5	6700634	6700634	+	6700634	3	3	XLOC_066546	+	predicted	---
5	6749488	6749488	+	6749488	3	3	ENSSSCG00000000103	+	intron	*DMC1*
5	6751165	6751166	+	6751165	4	8	XLOC_066547	+	predicted	---
5	6765419	6765419	+	6765419	6	6	---	---	intergenic	---
5	6786253	6786259	-	6786259	9	12	XLOC_067515	-	predicted	---
5	6786306	6786306	-	6786306	4	4	XLOC_067515	-	predicted	---

5	6848638	6848638	-	6848638	4	4	---	---	intergenic	---
5	6891901	6891901	+	6891901	10	10	ENSSSCG00000000107	+	intron	*CSNK1E*
5	6898200	6898200	+	6898200	4	4	ENSSSCG00000000107	+	intron	*CSNK1E*
5	6902918	6902918	+	6902918	5	5	ENSSSCG00000000107	+	3'UTR	*CSNK1E*
5	6966183	6966183	-	6966183	5	5	ENSSSCG00000030165	-	3'UTR	*MAFF*
5	6975390	6975390	-	6975390	3	3	ENSSSCG00000030165	-	intron	*MAFF*
5	7060415	7060421	+	7060415	10	19	XLOC_066553	+	predicted	---
5	7208318	7208318	-	7208318	7	7	ENSSSCG00000000114	-	3'UTR	*PICK1*
5	7225818	7225819	+	7225818	5	9	---	---	intergenic	---
5	7406632	7406632	-	7406632	4	4	ENSSSCG00000000118	-	intron	*MICALL1*
5	7465989	7465993	-	7465991	180	277	ENSSSCG00000000119	-	3'UTR	*EIF3L*
5	7466032	7466032	-	7466032	5	5	ENSSSCG00000000119	-	3'UTR	*EIF3L*
5	7476337	7476337	-	7476337	3	3	ENSSSCG00000000119	-	ORF	*EIF3L*
5	7561823	7561827	-	7561824	35	48	XLOC_067529	-	predicted	---
5	7576979	7576979	-	7576979	4	4	ENSSSCG00000020777	-	intron	---
5	7630614	7630616	-	7630614	5	12	ENSSSCG00000021663	-	3'UTR	---
5	7642312	7642322	-	7642319	475	662	ENSSSCG00000000125	-	3'UTR	*LGALS1*
5	7768041	7768044	-	7768041	36	64	XLOC_067539	-	predicted	---
5	7802924	7802926	-	7802924	3	6	ENSSSCG00000026108	-	3'UTR	*CDC42EP1*
5	7849133	7849135	+	7849133	3	6	---	---	intergenic	---
5	7869528	7869528	+	7869528	11	11	ENSSSCG00000030209	+	3'UTR	*MFNG*
5	8021331	8021333	+	8021332	5	12	XLOC_066571	+	predicted	---
5	8072177	8072177	-	8072177	7	7	ENSSSCG00000000130	-	3'UTR	*CYTH4*
5	8165047	8165047	+	8165047	18	18	ENSSSCG00000021944	+	intron	*RAC1*
5	8166384	8166392	+	8166392	44	64	ENSSSCG00000021944	+	3'UTR	*RAC1*
5	8203779	8203781	+	8203781	10	14	ENSSSCG00000030325	+	3'UTR	*C1QTNF6*

5	8304968	8304968	+	8304968	6	6	XLOC_066579	+	predicted	---
5	8338528	8338528	-	8338528	8	8	ENSSSCG00000000134	-	3'UTR	*MPST*
5	8358939	8358976	+	8358973	358	976	ENSSSCG00000000133	+	3'UTR	*TST*
5	8375590	8375594	+	8375593	41	85	XLOC_066582	+	predicted	---
5	8403543	8403550	+	8403547	20	61	XLOC_066583	+	predicted	---
5	8416684	8416684	-	8416684	5	5	ENSSSCG00000000136	-	3'UTR	*CSF2RB*
5	8477879	8477886	-	8477886	10	29	ENSSSCG00000000137	-	3'UTR	*NCF4*
5	8607737	8607739	+	8607737	4	7	ENSSSCG00000000139	+	intron	*IFT27*
5	8616163	8616172	+	8616165	30	61	ENSSSCG00000000139	+	3'UTR	*IFT27*
5	8911614	8911625	+	8911616	93	329	ENSSSCG00000000141	+	3'UTR	*EIF3D*
5	8936988	8936988	+	8936988	3	3	ENSSSCG00000000142	+	3'UTR	*FOXRED2*
5	9010262	9010273	+	9010271	131	275	ENSSSCG00000000144	+	3'UTR	*TXN2*
5	9010522	9010527	+	9010525	66	147	ENSSSCG00000000144	+	3'UTR	*TXN2*
5	9081479	9081489	-	9081483	6	21	ENSSSCG00000000145	-	ORF	*MYH9*
5	9088225	9088225	-	9088225	5	5	ENSSSCG00000000145	-	ORF	*MYH9*
5	9092396	9092396	-	9092396	4	4	ENSSSCG00000000145	-	ORF	*MYH9*
5	9181891	9181891	-	9181891	3	3	---	---	intergenic	---
5	9181915	9181915	+	9181915	3	3	---	---	intergenic	---
5	9276526	9276527	+	9276526	4	8	XLOC_066591	+	predicted	---
5	9323813	9323817	+	9323814	236	346	ENSSSCG00000020882	+	intron	---
5	9368816	9368818	+	9368818	57	61	---	---	intergenic	---
5	9744660	9744660	-	9744660	14	14	---	---	intergenic	---
5	9984703	9984703	+	9984703	3	3	---	---	intergenic	---
5	9993249	9993249	+	9993249	9	9	---	---	intergenic	---
5	10212766	10212766	+	10212766	9	9	---	---	intergenic	---
5	10505569	10505569	+	10505569	19	19	---	---	intergenic	---

5	10641530	10641530	+	10641530	3	3	---	---	intergenic	---
5	10735014	10735021	-	10735017	13	40	XLOC_067563	-	predicted	---
5	10956063	10956063	+	10956063	7	7	---	---	intergenic	---
5	11144478	11144478	+	11144478	3	3	---	---	intergenic	---
5	11603910	11603910	-	11603910	3	3	---	---	intergenic	---
5	11962119	11962124	-	11962122	39	104	XLOC_067570	-	predicted	---
5	11962591	11962591	-	11962591	7	7	XLOC_067570	-	predicted	---
5	12291448	12291449	-	12291448	26	34	ENSSSCG00000000156	-	3'UTR	*FBXO7*
5	12364482	12364495	+	12364495	12	25	ENSSSCG00000000157	+	3'UTR	*BPIFC*
5	12380421	12380421	+	12380421	3	3	ENSSSCG00000000158	+	ORF	*RTCB*
5	12392471	12392479	+	12392477	13	29	ENSSSCG00000000158	+	3'UTR	*RTCB*
5	12432981	12432981	+	12432981	11	11	ENSSSCG00000000160	+	intron	*PRDM4*
5	12463092	12463092	-	12463092	3	3	---	---	intergenic	---
5	12488890	12488897	-	12488897	15	18	ENSSSCG00000000161	-	3'UTR	*PWP1*
5	12541469	12541469	-	12541469	6	6	ENSSSCG00000000162	-	3'UTR	*BTBD11*
5	12843008	12843008	-	12843008	4	4	ENSSSCG00000000162	-	intron	*BTBD11*
5	12979260	12979260	+	12979260	3	3	---	---	intergenic	---
5	13217199	13217200	+	13217200	18	26	XLOC_066616	+	predicted	---
5	13229228	13229228	+	13229228	4	4	XLOC_066616	+	predicted	---
5	13232270	13232270	-	13232270	6	6	XLOC_067580	-	predicted	---
5	13232369	13232369	-	13232369	7	7	XLOC_067580	-	predicted	---
5	13234057	13234058	-	13234058	12	15	ENSSSCG00000000166	-	3'UTR	*TMEM263*
5	13234470	13234474	-	13234471	7	22	ENSSSCG00000000166	-	3'UTR	*TMEM263*
5	13234579	13234579	-	13234579	5	5	ENSSSCG00000000166	-	3'UTR	*TMEM263*
5	13234935	13234935	-	13234935	4	4	ENSSSCG00000000166	-	ORF	*TMEM263*
5	13283113	13283113	+	13283113	3	3	---	---	intergenic	---

5	13442393	13442399	-	13442399	18	21	XLOC_067582	-	predicted	---
5	13442499	13442499	-	13442499	6	6	XLOC_067582	-	predicted	---
5	13442543	13442543	-	13442543	3	3	XLOC_067582	-	predicted	---
5	13442701	13442703	-	13442701	7	15	XLOC_067582	-	predicted	---
5	13581387	13581387	-	13581387	6	6	---	---	intergenic	---
5	13688003	13688003	-	13688003	5	5	ENSSSCG00000026193	-	3'UTR	---
5	13688064	13688064	-	13688064	6	6	ENSSSCG00000026193	-	3'UTR	---
5	14038429	14038434	-	14038429	10	18	XLOC_067584	-	predicted	---
5	14107439	14107445	+	14107443	27	63	XLOC_066625	+	predicted	---
5	14139572	14139572	-	14139572	4	4	XLOC_067585	-	predicted	---
5	14290615	14290621	+	14290615	3	6	ENSSSCG00000027055	+	3'UTR	*NUAK1*
5	14580256	14580266	-	14580266	22	36	XLOC_067588	-	predicted	---
5	14875922	14875926	-	14875923	20	44	XLOC_067589	-	predicted	---
5	14955673	14955673	-	14955673	3	3	ENSSSCG00000028703	-	intron	*CCNT1*
5	15128520	15128527	+	15128520	15	24	XLOC_066633	+	predicted	---
5	15128974	15128974	-	15128974	7	7	XLOC_067591	-	predicted	---
5	15365290	15365290	+	15365290	3	3	ENSSSCG00000000179	+	intron	*CCDC65*
5	15368728	15368758	+	15368747	50	145	ENSSSCG00000000179	+	3'UTR	*CCDC65*
5	15369089	15369092	-	15369091	71	101	XLOC_067596	-	predicted	---
5	15369146	15369146	-	15369146	3	3	ENSSSCG00000000180	-	ORF	*FKBP11*
5	15383222	15383229	+	15383223	34	75	XLOC_066637	+	predicted	---
5	15443377	15443386	-	15443378	21	63	ENSSSCG00000000185	-	3'UTR	*PRKAG1*
5	15480566	15480587	-	15480567	13	31	ENSSSCG00000000189	-	3'UTR	*LMBR1L*
							ENSSSCG00000000193	-	intron	---
							ENSSSCG00000000185	-	intron	*PRKAG1*

5	15520271	15520271	-	15520271	3	3	ENSSSCG00000000185	-	intron	*PRKAG1*
							ENSSSCG00000000193	-	intron	---
5	15522684	15522684	-	15522684	4	4	ENSSSCG00000000191	-	3'UTR	*KMT2D*
							ENSSSCG00000000193	-	intron	---
5	15522941	15522941	-	15522941	3	3	ENSSSCG00000000191	-	3'UTR	*KMT2D*
							ENSSSCG00000000193	-	intron	---
5	15541036	15541036	-	15541036	3	3	ENSSSCG00000000193	-	intron	---
5	15556621	15556640	-	15556624	136	263	ENSSSCG00000000190	-	3'UTR	*TUBA1B*
							ENSSSCG00000000193	-	intron	---
5	15592446	15592455	-	15592452	137	188	ENSSSCG00000000193	-	3'UTR	---
5	15676195	15676216	+	15676215	104	154	ENSSSCG00000000194	+	3'UTR	---
5	15714113	15714116	+	15714113	4	7	XLOC_066644	+	predicted	---
5	15714740	15714740	-	15714740	3	3	XLOC_067607	-	predicted	---
5	15932631	15932631	+	15932631	11	11	XLOC_066652	+	predicted	---
5	15941391	15941391	-	15941391	4	4	ENSSSCG00000000199	-	intron	*SPATS2*
5	16176375	16176380	+	16176377	156	231	ENSSSCG00000000206	+	3'UTR	*FAIM2*
5	16183497	16183499	-	16183497	3	6	XLOC_067618	-	predicted	---
5	16183583	16183583	-	16183583	18	18	ENSSSCG00000000207	-	ORF	---
5	16183673	16183698	-	16183698	4	7	ENSSSCG00000000207	-	ORF	---
5	16184168	16184169	+	16184168	7	11	---	---	intergenic	---
5	16202663	16202666	+	16202663	4	7	ENSSSCG00000026576	+	3'UTR	*BCDIN3D*
5	16202962	16202962	+	16202962	5	5	ENSSSCG00000026576	+	3'UTR	*BCDIN3D*
5	16203650	16203650	+	16203650	3	3	ENSSSCG00000026576	+	3'UTR	*BCDIN3D*
5	16247766	16247766	+	16247766	6	6	ENSSSCG00000000209	+	3'UTR	*NCKAP5L*
5	16329792	16329794	-	16329792	4	7	XLOC_067620	-	predicted	---
5	16330845	16330847	-	16330847	148	153	ENSSSCG00000000214	-	3'UTR	*GPD1*

5	16335787	16335793	+	16335791	37	98	XLOC_066660	+	predicted	---
5	16336963	16336963	-	16336963	4	4	XLOC_067621	-	predicted	---
5	16487727	16487729	-	16487728	5	13	XLOC_067624	-	predicted	---
5	16501574	16501574	-	16501574	3	3	---	---	intergenic	---
5	16570757	16570757	+	16570757	4	4	ENSSSCG00000028272	+	intron	---
5	16675853	16675869	-	16675853	4	7	ENSSSCG00000028516	-	intron	*LARP4*
5	16882575	16882575	-	16882575	3	3	ENSSSCG00000028516	-	intron	*LARP4*
5	17157471	17157471	+	17157471	3	3	ENSSSCG00000029196	+	intron	*DIP2B*
5	17178684	17178684	+	17178684	3	3	ENSSSCG00000029196	+	intron	*DIP2B*
5	17183771	17183772	+	17183771	5	8	ENSSSCG00000029196	+	3'UTR	*DIP2B*
5	17183851	17183851	+	17183851	3	3	ENSSSCG00000029196	+	3'UTR	*DIP2B*
5	17187438	17187444	+	17187438	16	27	ENSSSCG00000029196	+	3'UTR	*DIP2B*
5	17277149	17277149	-	17277149	7	7	ENSSSCG00000026890	-	3'UTR	---
5	17307603	17307621	+	17307614	12	41	ENSSSCG00000022674	+	intron	*DAZAP2*
5	17308510	17308541	+	17308533	47	230	ENSSSCG00000022674	+	intron	*DAZAP2*
5	17309512	17309519	-	17309519	6	19	ENSSSCG00000000224	-	3'UTR	*SMAGP*
							ENSSSCG00000026890	-	intron	---
5	17315117	17315122	-	17315118	31	57	ENSSSCG00000000223	-	3'UTR	---
							ENSSSCG00000026890	-	intron	---
5	17366632	17366634	-	17366632	4	7	ENSSSCG00000026890	-	intron	---
5	17422737	17422742	-	17422739	26	62	ENSSSCG00000026890	-	intron	---
5	17430795	17430795	-	17430795	7	7	ENSSSCG00000026890	-	intron	---
5	17871392	17871394	+	17871394	15	23	ENSSSCG00000000232	+	3'UTR	*ACVRL1*
							ENSSSCG00000000232	+	ORF	*ACVRL1*
5	17938185	17938187	+	17938187	6	9	ENSSSCG00000000233	+	3'UTR	*ACVR1B*
5	18121712	18121713	-	18121712	21	31	XLOC_067646	-	predicted	---

5	18123157	18123157	-	18123157	6	6	---	---	intergenic	---
5	18663342	18663352	-	18663345	416	747	ENSSSCG00000000252	-	3'UTR	*KRT8*
5	18674655	18674655	-	18674655	3	3	XLOC_067662	-	predicted	---
5	18690368	18690368	-	18690368	3	3	XLOC_067663	-	predicted	---
5	18710884	18710884	-	18710884	3	3	XLOC_067664	-	predicted	---
5	18729749	18729760	+	18729754	187	345	ENSSSCG00000000253	+	3'UTR	*KRT18*
5	18736465	18736465	+	18736465	3	3	XLOC_066690	+	predicted	---
5	18737340	18737346	+	18737344	58	150	XLOC_066690	+	predicted	---
5	18737687	18737696	+	18737695	8	22	XLOC_066690	+	predicted	---
5	18737886	18737886	+	18737886	5	5	XLOC_066690	+	predicted	---
5	18739012	18739012	+	18739012	3	3	XLOC_066690	+	predicted	---
5	18739216	18739242	+	18739241	66	105	XLOC_066690	+	predicted	---
5	18772052	18772057	+	18772057	6	13	ENSSSCG00000000261	+	3'UTR	*IGFBP6*
5	18786094	18786094	+	18786094	5	5	XLOC_066694	+	predicted	---
5	18793689	18793696	-	18793689	5	14	XLOC_067667	-	predicted	---
5	18829994	18829999	-	18829997	40	53	XLOC_067668	-	predicted	---
5	18846939	18846939	-	18846939	3	3	ENSSSCG00000000258	-	3'UTR	*RARG*
5	18891859	18891859	+	18891859	3	3	ENSSSCG00000000262	+	intron	*SPRYD3*
5	18901690	18901703	+	18901702	13	34	ENSSSCG00000000262	+	3'UTR	*SPRYD3*
5	18901929	18901934	-	18901929	4	8	ENSSSCG00000000263	-	3'UTR	*TNS2*
5	18914464	18914471	+	18914464	14	34	XLOC_066699	+	predicted	---
5	18945644	18945651	+	18945651	6	9	---	---	intergenic	---
5	18952640	18952669	+	18952664	263	581	XLOC_066701	+	predicted	---
5	18961605	18961606	+	18961605	30	36	ENSSSCG00000000267	+	3'UTR	*C12orf10*
5	18961800	18961800	-	18961800	4	4	ENSSSCG00000000268	-	3'UTR	*AAAS*
5	18962017	18962038	-	18962032	4	17	ENSSSCG00000000268	-	3'UTR	*AAAS*

5	19003432	19003432	+	19003432	4	4	---	---	intergenic	---
5	19062757	19062757	+	19062757	4	4	ENSSSCG00000000272	+	intron	*SP1*
5	19066231	19066231	+	19066231	6	6	XLOC_066702	+	predicted	---
5	19067759	19067759	+	19067759	3	3	XLOC_066702	+	predicted	---
5	19070996	19070999	+	19070996	3	6	XLOC_066702	+	predicted	---
5	19101386	19101404	+	19101404	11	17	ENSSSCG00000000273	+	3'UTR	*PRR13*
5	19101470	19101495	+	19101474	539	960	ENSSSCG00000000273	+	3'UTR	*PRR13*
5	19101643	19101646	+	19101644	22	36	ENSSSCG00000000273	+	3'UTR	*PRR13*
5	19134748	19134748	+	19134748	3	3	ENSSSCG00000000274	+	3'UTR	*PCBP2*
5	19134788	19134806	+	19134791	75	230	ENSSSCG00000000274	+	3'UTR	*PCBP2*
5	19134969	19135031	+	19135014	67	452	XLOC_066705	+	predicted	---
5	19135119	19135124	+	19135123	22	42	XLOC_066705	+	predicted	---
5	19135712	19135718	-	19135712	5	9	XLOC_067677	-	predicted	---
5	19159468	19159469	+	19159469	12	17	ENSSSCG00000000276	+	3'UTR	*TARBP2*
5	19164390	19164398	-	19164398	4	7	XLOC_067678	-	predicted	---
5	19375988	19375988	+	19375988	6	6	---	---	intergenic	---
5	19575800	19575800	+	19575800	4	4	---	---	intergenic	---
5	19677255	19677256	-	19677255	12	17	---	---	intergenic	---
5	19677431	19677431	-	19677431	4	4	---	---	intergenic	---
5	19700771	19700771	+	19700771	5	5	XLOC_066718	+	predicted	---
5	20048969	20048970	+	20048969	6	9	ENSSSCG00000024221	+	3'UTR	*SMUG1*
5	20050155	20050155	+	20050155	3	3	ENSSSCG00000024221	+	3'UTR	*SMUG1*
5	20091149	20091150	-	20091149	21	32	XLOC_067692	-	predicted	---
5	20102191	20102193	+	20102193	9	16	XLOC_066723	+	predicted	---
5	20128021	20128021	-	20128021	8	8	---	---	intergenic	---
5	20329568	20329568	-	20329568	6	6	XLOC_067694	-	predicted	---

5	20331011	20331011	+	20331011	4	4	ENSSSCG00000000288	+	ORF	*HNRNPA1*
5	20332786	20332786	+	20332786	3	3	ENSSSCG00000000288	+	ORF	*HNRNPA1*
5	20333673	20333673	+	20333673	3	3	ENSSSCG00000000288	+	3'UTR	*HNRNPA1*
5	20334138	20334144	+	20334143	73	148	XLOC_066726	+	predicted	---
5	20353741	20353743	-	20353741	17	24	XLOC_067695	-	predicted	---
5	20399555	20399563	+	20399556	58	91	ENSSSCG00000000290	+	3'UTR	*COPZ1*
5	20417581	20417581	-	20417581	4	4	ENSSSCG00000000292	-	3'UTR	*ZNF385A*
5	20442149	20442152	-	20442150	32	53	ENSSSCG00000000293	-	3'UTR	*ITGA5*
5	20485107	20485107	+	20485107	3	3	---	---	intergenic	---
5	20494160	20494166	-	20494162	20	48	ENSSSCG00000026333	-	3'UTR	*GTSF1*
5	20496529	20496529	-	20496529	5	5	ENSSSCG00000026333	-	3'UTR	*GTSF1*
5	20496783	20496784	-	20496784	9	12	ENSSSCG00000026333	-	3'UTR	*GTSF1*
5	20497274	20497286	-	20497276	42	139	ENSSSCG00000026333	-	3'UTR	*GTSF1*
5	20497379	20497414	-	20497388	451	770	ENSSSCG00000026333	-	3'UTR	*GTSF1*
5	20499831	20499837	-	20499837	7	11	ENSSSCG00000026333	-	intron	*GTSF1*
5	20501073	20501085	-	20501085	14	17	ENSSSCG00000026333	-	ORF	*GTSF1*
5	20509847	20509848	-	20509848	6	9	---	---	intergenic	---
5	20578569	20578570	+	20578569	22	25	XLOC_066731	+	predicted	---
5	20578705	20578705	+	20578705	5	5	XLOC_066731	+	predicted	---
5	20579606	20579606	+	20579606	4	4	XLOC_066731	+	predicted	---
5	20612210	20612210	+	20612210	3	3	ENSSSCG00000000297	+	3'UTR	---
5	20659796	20659805	-	20659796	10	15	XLOC_067703	-	predicted	---
5	20793847	20793847	+	20793847	6	6	XLOC_066733	+	predicted	---
5	20814594	20814594	+	20814594	7	7	---	---	intergenic	---
5	21107050	21107050	+	21107050	4	4	---	---	intergenic	---
5	21419160	21419160	-	21419160	3	3	---	---	intergenic	---

5	21419207	21419207	+	21419207	5	5	---	---	intergenic	---
5	21450621	21450621	+	21450621	3	3	---	---	intergenic	---
5	21824996	21825008	+	21824996	15	23	---	---	intergenic	---
5	22441552	22441552	+	22441552	3	3	---	---	intergenic	---
5	22640575	22640579	+	22640576	22	32	ENSSSCG00000000359	+	3'UTR	*BLOC1S1*
5	22643878	22643878	-	22643878	3	3	---	---	intergenic	---
5	22652129	22652148	-	22652131	492	944	ENSSSCG00000000361	-	3'UTR	*CD63*
5	22733159	22733159	+	22733159	3	3	---	---	intergenic	---
5	22734893	22734893	-	22734893	3	3	---	---	intergenic	---
5	22744869	22744873	+	22744869	47	120	XLOC_066774	+	predicted	---
5	22745106	22745113	-	22745107	10	27	ENSSSCG00000000366	-	3'UTR	*DNAJC14*
5	22745732	22745739	-	22745733	12	21	ENSSSCG00000000366	-	3'UTR	*DNAJC14*
5	22745785	22745797	-	22745794	20	61	ENSSSCG00000000366	-	3'UTR	*DNAJC14*
5	22759688	22759706	-	22759702	58	161	ENSSSCG00000000368	-	3'UTR	*MMP19*
5	22788969	22788969	-	22788969	5	5	ENSSSCG00000000369	-	3'UTR	*WIBG*
5	22789080	22789091	-	22789083	22	45	ENSSSCG00000000369	-	3'UTR	*WIBG*
5	22829891	22829891	+	22829891	3	3	ENSSSCG00000000370	+	intron	*DGKA*
5	22835784	22835785	+	22835784	13	16	ENSSSCG00000000370	+	3'UTR	*DGKA*
5	22912558	22912570	-	22912566	75	113	XLOC_067741	-	predicted	---
5	22936562	22936562	-	22936562	3	3	XLOC_067742	-	predicted	---
5	22947586	22947589	+	22947586	8	19	XLOC_066778	+	predicted	---
5	23003128	23003128	+	23003128	12	12	ENSSSCG00000000379	+	3'UTR	*ESYT1*
							ENSSSCG00000020725	+	intron	*ERBB3*
5	23003181	23003181	+	23003181	4	4	ENSSSCG00000000379	+	3'UTR	*ESYT1*
							ENSSSCG00000020725	+	intron	*ERBB3*
5	23029590	23029590	-	23029590	3	3	XLOC_067747	-	predicted	---

5	23036417	23036424	-	23036422	23	39	XLOC_067747	-	predicted	---
5	23103722	23103726	-	23103722	34	67	ENSSSCG00000000377	-	3'UTR	---
5	23104512	23104532	-	23104528	61	88	ENSSSCG00000000377	-	3'UTR	---
5	23104634	23104634	-	23104634	3	3	ENSSSCG00000000377	-	3'UTR	---
5	23128007	23128007	-	23128007	5	5	ENSSSCG00000022002	-	3'UTR	---
5	23172680	23172682	+	23172680	5	8	XLOC_066790	+	predicted	---
5	23209314	23209316	+	23209315	8	17	ENSSSCG00000000394	+	3'UTR	---
5	23290953	23290953	-	23290953	3	3	ENSSSCG00000000396	-	ORF	*STAT2*
5	23294815	23294870	-	23294817	156	328	XLOC_067758	-	predicted	---
5	23295450	23295450	-	23295450	5	5	XLOC_067758	-	predicted	---
5	23304872	23304889	-	23304884	109	357	ENSSSCG00000000398	-	3'UTR	*APON*
5	23305583	23305587	-	23305583	4	8	ENSSSCG00000000398	-	ORF	*APON*
5	23306976	23306980	-	23306977	4	11	ENSSSCG00000000399	-	3'UTR	*TIMELESS*
5	23308003	23308003	-	23308003	3	3	ENSSSCG00000000399	-	3'UTR	*TIMELESS*
5	23602936	23602936	+	23602936	6	6	XLOC_066798	+	predicted	---
5	23608571	23608572	-	23608571	5	10	ENSSSCG00000000403	-	3'UTR	*BAZ2A*
5	23659079	23659086	-	23659085	127	211	ENSSSCG00000000405	-	3'UTR	*ATP5B*
5	23659183	23659183	-	23659183	9	9	ENSSSCG00000000405	-	3'UTR	*ATP5B*
5	23659229	23659229	-	23659229	3	3	ENSSSCG00000000405	-	3'UTR	*ATP5B*
5	23663547	23663547	-	23663547	3	3	---	---	intergenic	---
5	23665599	23665599	-	23665599	12	12	---	---	intergenic	---
5	23674708	23674711	-	23674710	63	88	ENSSSCG00000000406	-	3'UTR	*PTGES3*
5	23675552	23675556	-	23675554	43	86	ENSSSCG00000000406	-	3'UTR	*PTGES3*
5	23683214	23683214	-	23683214	7	7	ENSSSCG00000000406	-	ORF	*PTGES3*
5	23683795	23683795	-	23683795	3	3	ENSSSCG00000000406	-	ORF	*PTGES3*
5	23697275	23697276	+	23697275	24	28	---	---	intergenic	---

5	23713042	23713044	+	23713043	73	103	---	---	intergenic	---
5	23718959	23718970	-	23718962	1230	2320	ENSSSCG00000020830	-	3'UTR	*NACA*
5	23720211	23720211	-	23720211	4	4	ENSSSCG00000020830	-	3'UTR	*NACA*
							ENSSSCG00000020830	-	ORF	*NACA*
5	23733788	23733792	-	23733792	117	156	ENSSSCG00000020830	-	intron	*NACA*
5	23775872	23775878	-	23775873	27	95	XLOC_067768	-	predicted	---
5	23775931	23775934	-	23775933	7	20	ENSSSCG00000027854	-	3'UTR	*HSD17B6*
5	23776366	23776387	-	23776372	98	230	ENSSSCG00000027854	-	3'UTR	*HSD17B6*
5	23776439	23776496	-	23776496	10	37	ENSSSCG00000027854	-	3'UTR	*HSD17B6*
5	23776738	23776738	-	23776738	3	3	ENSSSCG00000027854	-	ORF	*HSD17B6*
5	23824231	23824231	+	23824231	5	5	XLOC_066801	+	predicted	---
5	23884804	23884804	-	23884804	9	9	---	---	intergenic	---
5	24056417	24056417	+	24056417	4	4	ENSSSCG00000000418	+	3'UTR	*TAC3*
5	24072685	24072685	+	24072685	6	6	XLOC_066806	+	predicted	---
5	24074722	24074723	-	24074723	4	7	XLOC_067772	-	predicted	---
5	24133012	24133041	-	24133015	151	616	XLOC_067776	-	predicted	---
5	24133447	24133460	-	24133450	10	22	ENSSSCG00000024919	-	3'UTR	*RDH16*
5	24152298	24152298	+	24152298	3	3	---	---	intergenic	---
5	24303406	24303406	-	24303406	3	3	ENSSSCG00000023150	-	intron	---
5	24392778	24392779	-	24392779	8	15	ENSSSCG00000026498	-	3'UTR	---
5	24408001	24408001	-	24408001	3	3	ENSSSCG00000026498	-	intron	---
5	24531018	24531018	-	24531018	4	4	ENSSSCG00000026498	-	intron	---
5	24688036	24688038	+	24688037	18	36	XLOC_066819	+	predicted	---
5	24717500	24717504	+	24717501	70	141	ENSSSCG00000000439	+	intron	*KIF5A*
5	24717849	24717850	+	24717849	6	9	ENSSSCG00000000439	+	intron	*KIF5A*
5	24726684	24726684	-	24726684	6	6	ENSSSCG00000023318	-	intron	---

5	24734099	24734100	-	24734099	10	13	ENSSSCG00000000449	-	3'UTR	---
							ENSSSCG00000023318	-	intron	---
5	24753306	24753306	-	24753306	3	3	ENSSSCG00000000449	-	intron	---
							ENSSSCG00000023318	-	intron	---
5	24761366	24761375	+	24761374	9	20	ENSSSCG00000000439	+	intron	*KIF5A*
5	24761528	24761531	-	24761530	205	351	ENSSSCG00000000444	-	3'UTR	---
							ENSSSCG00000000449	-	intron	---
							ENSSSCG00000023318	-	intron	---
5	24808818	24808818	-	24808818	3	3	ENSSSCG00000000449	-	intron	---
5	24821714	24821714	-	24821714	4	4	ENSSSCG00000000436	-	3'UTR	---
5	24821894	24821894	-	24821894	5	5	ENSSSCG00000000436	-	3'UTR	---
5	24824453	24824453	-	24824453	5	5	ENSSSCG00000000436	-	intron	---
5	24835151	24835154	+	24835153	20	38	ENSSSCG00000000437	+	3'UTR	---
5	24927221	24927221	-	24927221	4	4	---	---	intergenic	---
5	24977423	24977423	+	24977423	7	7	ENSSSCG00000000453	+	3'UTR	*XRCC6BP1*
5	25166262	25166262	-	25166262	3	3	---	---	intergenic	---
5	25205266	25205266	+	25205266	3	3	---	---	intergenic	---
5	25623682	25623682	+	25623682	9	9	XLOC_066832	+	predicted	---
5	26634986	26634986	+	26634986	3	3	---	---	intergenic	---
5	26700461	26700473	-	26700465	15	23	XLOC_067806	-	predicted	---
5	26864727	26864756	+	26864743	7	47	ENSSSCG00000000456	+	3'UTR	*SLC16A7*
5	27014802	27014803	-	27014802	5	8	---	---	intergenic	---
5	27363056	27363056	-	27363056	6	6	---	---	intergenic	---
5	27922750	27922750	-	27922750	4	4	---	---	intergenic	---
5	28164886	28164888	-	28164888	5	8	---	---	intergenic	---
5	28495612	28495618	+	28495617	45	76	---	---	intergenic	---

5	28600226	28600226	-	28600226	3	3	---	---	intergenic	---
5	28751546	28751546	+	28751546	23	23	---	---	intergenic	---
5	28833104	28833104	+	28833104	3	3	---	---	intergenic	---
5	29184919	29184919	-	29184919	4	4	---	---	intergenic	---
5	29262651	29262651	-	29262651	10	10	---	---	intergenic	---
5	29735081	29735081	+	29735081	4	4	ENSSSCG00000000457	+	intron	---
5	29799380	29799389	+	29799386	15	35	ENSSSCG00000000457	+	3'UTR	---
5	29864161	29864161	-	29864161	4	4	---	---	intergenic	---
5	29906922	29906922	+	29906922	4	4	ENSSSCG00000000458	+	intron	*MON2*
5	30009469	30009472	+	30009471	6	13	ENSSSCG00000000458	+	3'UTR	*MON2*
5	30018734	30018734	-	30018734	3	3	---	---	intergenic	---
5	30021919	30021919	+	30021919	5	5	XLOC_066845	+	predicted	---
5	30281524	30281524	-	30281524	5	5	---	---	intergenic	---
5	30387549	30387551	+	30387549	9	13	---	---	intergenic	---
5	31159391	31159404	-	31159392	8	22	XLOC_067815	-	predicted	---
5	31262425	31262430	+	31262427	16	34	---	---	intergenic	---
5	31462199	31462207	+	31462206	20	52	ENSSSCG00000000462	+	3'UTR	*TMEM5*
5	31462387	31462387	+	31462387	3	3	---	---	intergenic	---
5	31485591	31485591	+	31485591	3	3	---	---	intergenic	---
5	31725935	31725938	+	31725936	24	36	---	---	intergenic	---
5	31983877	31983877	+	31983877	3	3	---	---	intergenic	---
5	32031106	32031113	+	32031106	3	6	ENSSSCG00000030352	+	ORF	*C12orf66*
5	32033565	32033565	+	32033565	3	3	---	---	intergenic	---
5	32038863	32038873	-	32038871	10	51	---	---	intergenic	---
5	32041384	32041389	-	32041384	10	15	---	---	intergenic	---
5	32173100	32173106	-	32173100	6	14	ENSSSCG00000023715	-	intron	---

5	32851831	32851831	-	32851831	18	18	---	---	intergenic	---
5	32896620	32896620	+	32896620	3	3	XLOC_066862	+	predicted	---
5	33088174	33088174	+	33088174	3	3	ENSSSCG00000026004	+	3'UTR	*MSRB3*
5	33720262	33720263	-	33720262	15	18	ENSSSCG00000000472	-	3'UTR	*LLPH*
5	33729169	33729170	-	33729170	7	11	XLOC_067831	-	predicted	---
5	33729283	33729284	-	33729284	14	19	XLOC_067831	-	predicted	---
5	33731379	33731384	-	33731384	15	20	XLOC_067831	-	predicted	---
5	33731424	33731432	-	33731432	33	57	XLOC_067831	-	predicted	---
5	33798975	33798975	-	33798975	5	5	XLOC_067832	-	predicted	---
5	33883469	33883469	+	33883469	5	5	XLOC_066869	+	predicted	---
5	33970781	33970781	+	33970781	3	3	XLOC_066870	+	predicted	---
5	33972538	33972538	-	33972538	9	9	XLOC_067834	-	predicted	---
5	33994885	33994885	-	33994885	3	3	ENSSSCG00000000478	-	intron	*GRIP1*
5	33999525	33999525	-	33999525	6	6	ENSSSCG00000000478	-	ORF	*GRIP1*
5	33999619	33999619	-	33999619	5	5	ENSSSCG00000000478	-	ORF	*GRIP1*
5	34005726	34005726	-	34005726	3	3	ENSSSCG00000000478	-	intron	*GRIP1*
5	34252423	34252424	-	34252424	4	7	ENSSSCG00000000478	-	intron	*GRIP1*
5	34631026	34631026	-	34631026	3	3	---	---	intergenic	---
5	34840672	34840672	+	34840672	3	3	---	---	intergenic	---
5	34871471	34871471	-	34871471	3	3	---	---	intergenic	---
5	34912566	34912566	-	34912566	4	4	---	---	intergenic	---
5	34937728	34937728	-	34937728	6	6	---	---	intergenic	---
5	35335570	35335570	+	35335570	3	3	---	---	intergenic	---
5	35386481	35386481	+	35386481	3	3	---	---	intergenic	---
5	35453851	35453851	-	35453851	3	3	XLOC_067841	-	predicted	---
5	35732717	35732728	+	35732728	9	12	ENSSSCG00000030014	+	3'UTR	---

5	35733569	35733586	+	35733585	26	69	ENSSSCG00000030014	+	3'UTR	---
5	35733657	35733667	+	35733657	3	6	ENSSSCG00000030014	+	3'UTR	---
5	35734754	35734754	+	35734754	5	5	ENSSSCG00000030014	+	3'UTR	---
5	35736568	35736568	+	35736568	3	3	ENSSSCG00000030014	+	3'UTR	---
5	35843760	35843760	-	35843760	3	3	---	---	intergenic	---
5	35851538	35851538	+	35851538	5	5	XLOC_066876	+	predicted	---
5	35851960	35851966	+	35851960	7	11	XLOC_066876	+	predicted	---
5	35901615	35901615	+	35901615	7	7	ENSSSCG00000000488	+	intron	*MDM2*
5	35911704	35911707	+	35911704	12	23	ENSSSCG00000000488	+	intron	*MDM2*
5	35921529	35921529	+	35921529	5	5	ENSSSCG00000000488	+	intron	*MDM2*
5	36065996	36066004	+	36066002	24	117	---	---	intergenic	---
5	36179189	36179197	-	36179191	400	450	ENSSSCG00000000492	-	3'UTR	*LYZ*
5	36189903	36189903	+	36189903	7	7	XLOC_066882	+	predicted	---
5	36214457	36214458	-	36214457	6	9	---	---	intergenic	---
5	36225114	36225116	+	36225116	4	7	XLOC_066884	+	predicted	---
5	36342481	36342481	-	36342481	3	3	XLOC_067853	-	predicted	---
5	36343096	36343096	-	36343096	9	9	XLOC_067853	-	predicted	---
5	36347390	36347390	-	36347390	3	3	ENSSSCG00000000493	-	ORF	*FRS2*
5	36443108	36443108	-	36443108	4	4	ENSSSCG00000000493	-	intron	*FRS2*
5	36462678	36462678	+	36462678	5	5	ENSSSCG00000000496	+	ORF	*CCT2*
5	36471826	36471852	+	36471827	86	241	ENSSSCG00000000496	+	3'UTR	*CCT2*
5	36555538	36555538	+	36555538	6	6	---	---	intergenic	---
5	36652149	36652151	+	36652149	5	11	ENSSSCG00000000500	+	3'UTR	---
5	36851377	36851377	+	36851377	4	4	ENSSSCG00000000501	+	intron	---
5	37207483	37207494	+	37207483	3	6	XLOC_066896	+	predicted	---
5	37208124	37208130	+	37208127	41	60	XLOC_066896	+	predicted	---

5	37225743	37225743	+	37225743	3	3	---	---	intergenic	---
5	37228340	37228344	-	37228340	4	7	XLOC_067861	-	predicted	---
5	37233718	37233718	-	37233718	4	4	XLOC_067861	-	predicted	---
5	37236597	37236597	-	37236597	4	4	ENSSSCG00000000504	-	ORF	*PTPRB*
5	37731954	37731954	+	37731954	5	5	---	---	intergenic	---
5	37797704	37797704	-	37797704	3	3	---	---	intergenic	---
5	37801371	37801371	-	37801371	4	4	---	---	intergenic	---
5	37839245	37839249	-	37839245	23	37	XLOC_067863	-	predicted	---
5	38052730	38052730	+	38052730	4	4	---	---	intergenic	---
5	38210087	38210087	+	38210087	11	11	ENSSSCG00000030511	+	intron	*LGR5*
5	38316709	38316709	+	38316709	6	6	---	---	intergenic	---
5	38388502	38388502	+	38388502	4	4	XLOC_066900	+	predicted	---
5	38488938	38488941	+	38488939	9	22	XLOC_066902	+	predicted	---
5	38490040	38490040	+	38490040	7	7	XLOC_066902	+	predicted	---
5	38576043	38576043	+	38576043	3	3	ENSSSCG00000000512	+	ORF	*TBC1D15*
5	38581769	38581769	+	38581769	3	3	ENSSSCG00000000512	+	ORF	*TBC1D15*
5	38607848	38607848	+	38607848	10	10	ENSSSCG00000000512	+	3'UTR	*TBC1D15*
5	38608793	38608798	+	38608798	9	12	ENSSSCG00000000512	+	3'UTR	*TBC1D15*
5	39009293	39009295	+	39009293	3	9	---	---	intergenic	---
5	39617274	39617274	+	39617274	5	5	---	---	intergenic	---
5	41233317	41233317	-	41233317	8	8	---	---	intergenic	---
5	41541152	41541157	+	41541154	19	29	---	---	intergenic	---
5	41695026	41695026	-	41695026	6	6	---	---	intergenic	---
5	42200873	42200873	-	42200873	3	3	---	---	intergenic	---
5	42222802	42222815	-	42222809	32	148	XLOC_067870	-	predicted	---
5	42373199	42373199	-	42373199	4	4	ENSSSCG00000000520	-	3'UTR	*KRR1*

5	42567907	42567911	-	42567910	8	20	---	---	intergenic	---
5	42567939	42567939	+	42567939	4	4	---	---	intergenic	---
5	42858299	42858299	+	42858299	3	3	XLOC_066916	+	predicted	---
5	42892569	42892569	-	42892569	8	8	ENSSSCG00000000522	-	3'UTR	*NAP1L1*
5	42892678	42892678	-	42892678	3	3	ENSSSCG00000000522	-	3'UTR	*NAP1L1*
5	42894014	42894027	-	42894020	22	60	ENSSSCG00000000522	-	3'UTR	*NAP1L1*
5	42894216	42894216	-	42894216	5	5	ENSSSCG00000000522	-	intron	*NAP1L1*
5	42896093	42896093	-	42896093	3	3	ENSSSCG00000000522	-	intron	*NAP1L1*
5	42896617	42896620	-	42896618	16	28	ENSSSCG00000000522	-	intron	*NAP1L1*
5	42896751	42896759	-	42896752	19	26	ENSSSCG00000000522	-	intron	*NAP1L1*
5	42900219	42900219	-	42900219	3	3	ENSSSCG00000000522	-	intron	*NAP1L1*
5	42903750	42903750	-	42903750	6	6	ENSSSCG00000000522	-	intron	*NAP1L1*
5	42963823	42963831	-	42963823	10	21	XLOC_067879	-	predicted	---
5	43262619	43262625	-	43262619	3	6	ENSSSCG00000027503	-	ORF	*OSBPL8*
5	43317365	43317365	-	43317365	3	3	---	---	intergenic	---
5	43317389	43317389	+	43317389	3	3	---	---	intergenic	---
5	43574587	43574587	-	43574587	3	3	ENSSSCG00000026719	-	3'UTR	---
5	43577476	43577476	-	43577476	3	3	ENSSSCG00000026719	-	ORF	---
5	44595343	44595343	+	44595343	3	3	ENSSSCG00000000528	+	intron	*PKP2*
5	44713954	44713954	-	44713954	3	3	XLOC_067886	-	predicted	---
5	44785735	44785735	+	44785735	4	4	ENSSSCG00000000529	+	3'UTR	---
5	44787266	44787271	+	44787271	6	9	ENSSSCG00000000529	+	3'UTR	---
5	45201959	45201960	-	45201960	6	9	ENSSSCG00000000531	-	intron	---
5	45429382	45429382	-	45429382	7	7	---	---	intergenic	---
5	45447016	45447016	-	45447016	3	3	---	---	intergenic	---
5	45447043	45447044	+	45447044	6	10	---	---	intergenic	---

5	45447277	45447277	-	45447277	4	4	---	---	intergenic	---
5	45447309	45447318	+	45447314	7	19	---	---	intergenic	---
5	45552764	45552764	-	45552764	3	3	ENSSSCG00000030616	-	ORF	*KIAA1551*
5	45597071	45597071	+	45597071	11	11	XLOC_066930	+	predicted	---
5	45978839	45978846	-	45978843	10	20	---	---	intergenic	---
5	46003481	46003482	-	46003481	17	25	ENSSSCG00000026593	-	intron	---
5	46467795	46467795	-	46467795	6	6	---	---	intergenic	---
5	46503495	46503495	-	46503495	4	4	---	---	intergenic	---
5	46710481	46710481	+	46710481	3	3	---	---	intergenic	---
5	46786266	46786275	-	46786272	10	26	---	---	intergenic	---
5	46881431	46881432	-	46881432	4	7	XLOC_067901	-	predicted	---
5	47362476	47362476	+	47362476	3	3	ENSSSCG00000027447	+	intron	---
5	47699918	47699918	+	47699918	3	3	ENSSSCG00000000536	+	3'UTR	*ERGIC2*
5	47700006	47700006	+	47700006	4	4	ENSSSCG00000000536	+	3'UTR	*ERGIC2*
5	47700246	47700268	+	47700246	6	21	ENSSSCG00000000536	+	3'UTR	*ERGIC2*
5	47707192	47707208	-	47707192	7	12	ENSSSCG00000000541	-	3'UTR	*FAR2*
5	47985149	47985149	+	47985149	10	10	---	---	intergenic	---
5	48191520	48191520	-	48191520	6	6	---	---	intergenic	---
5	48191573	48191573	+	48191573	3	3	---	---	intergenic	---
5	48219505	48219505	-	48219505	3	3	---	---	intergenic	---
5	48277217	48277217	-	48277217	4	4	---	---	intergenic	---
5	48560915	48560918	+	48560916	10	20	---	---	intergenic	---
5	48627586	48627586	-	48627586	5	5	ENSSSCG00000000543	-	intron	*CCDC91*
5	48879190	48879198	+	48879190	5	8	ENSSSCG00000024232	+	3'UTR	*CCDC91*
5	49127246	49127246	+	49127246	6	6	---	---	intergenic	---
5	49220406	49220406	-	49220406	5	5	---	---	intergenic	---

5	49310174	49310174	+	49310174	8	8	---	---	intergenic	---
5	49421687	49421687	+	49421687	21	21	---	---	intergenic	---
5	49448877	49448877	-	49448877	17	17	---	---	intergenic	---
5	49528207	49528208	+	49528207	12	18	---	---	intergenic	---
5	49542549	49542549	-	49542549	3	3	ENSSSCG00000000547	-	3'UTR	*MRPS35*
5	49542642	49542642	-	49542642	3	3	ENSSSCG00000000547	-	3'UTR	*MRPS35*
5	49566730	49566730	-	49566730	3	3	ENSSSCG00000000547	-	ORF	*MRPS35*
5	49597378	49597397	-	49597384	6	14	XLOC_067910	-	predicted	---
5	49599485	49599487	-	49599487	14	24	ENSSSCG00000000549	-	3'UTR	*PPFIBP1*
5	49624943	49624943	-	49624943	3	3	ENSSSCG00000000549	-	intron	*PPFIBP1*
5	49639674	49639674	-	49639674	3	3	ENSSSCG00000000549	-	intron	*PPFIBP1*
5	49639733	49639733	-	49639733	4	4	ENSSSCG00000000549	-	intron	*PPFIBP1*
5	49711655	49711661	+	49711655	29	71	---	---	intergenic	---
5	49711752	49711764	+	49711752	4	8	---	---	intergenic	---
5	49738241	49738241	+	49738241	5	5	---	---	intergenic	---
5	49744443	49744443	+	49744443	4	4	---	---	intergenic	---
5	49775557	49775557	+	49775557	3	3	---	---	intergenic	---
5	49785853	49785856	+	49785853	5	8	---	---	intergenic	---
5	49802520	49802524	-	49802522	50	79	---	---	intergenic	---
5	49905122	49905122	-	49905122	8	8	ENSSSCG00000000551	-	intron	*ARNTL2*
5	50042303	50042303	-	50042303	4	4	ENSSSCG00000022288	-	3'UTR	*STK38L*
5	50043184	50043184	-	50043184	3	3	ENSSSCG00000022288	-	3'UTR	*STK38L*
5	50095098	50095098	+	50095098	5	5	---	---	intergenic	---
5	50281840	50281845	-	50281840	6	13	ENSSSCG00000000552	-	3'UTR	*MED21*
5	50345626	50345637	-	50345626	7	14	ENSSSCG00000000554	-	3'UTR	*FGFR1OP2*
5	50349434	50349441	-	50349434	5	13	ENSSSCG00000000554	-	intron	*FGFR1OP2*

5	50352693	50352693	-	50352693	8	8	ENSSSCG00000000554	-	intron	*FGFR1OP2*
5	50649268	50649268	+	50649268	3	3	ENSSSCG00000000555	+	ORF	---
5	50696846	50696846	+	50696846	3	3	ENSSSCG00000000555	+	ORF	---
5	50895145	50895145	+	50895145	3	3	ENSSSCG00000000555	+	intron	---
5	51214259	51214259	+	51214259	3	3	ENSSSCG00000025531	+	3'UTR	*SSPN*
5	51474603	51474609	-	51474608	22	47	XLOC_067922	-	predicted	---
5	51478418	51478418	-	51478418	4	4	ENSSSCG00000000559	-	ORF	*RASSF8*
5	51624785	51624789	-	51624786	17	24	XLOC_067924	-	predicted	---
5	52270152	52270152	+	52270152	12	12	ENSSSCG00000026969	+	3'UTR	---
5	52270379	52270381	-	52270381	15	33	XLOC_067928	-	predicted	---
5	52270831	52270838	-	52270838	11	24	XLOC_067928	-	predicted	---
5	52401883	52401883	-	52401883	5	5	---	---	intergenic	---
5	52629737	52629737	+	52629737	6	6	---	---	intergenic	---
5	52781347	52781348	+	52781348	5	8	XLOC_066974	+	predicted	---
5	52841103	52841103	-	52841103	3	3	---	---	intergenic	---
5	52842365	52842366	+	52842366	8	11	XLOC_066974	+	predicted	---
5	52885838	52885838	-	52885838	4	4	XLOC_067934	-	predicted	---
5	52886153	52886153	-	52886153	3	3	XLOC_067934	-	predicted	---
5	53319682	53319683	-	53319682	5	8	---	---	intergenic	---
5	53319712	53319714	+	53319712	3	6	---	---	intergenic	---
5	53333539	53333539	-	53333539	3	3	---	---	intergenic	---
5	53363747	53363747	+	53363747	3	3	---	---	intergenic	---
5	53722357	53722357	-	53722357	3	3	XLOC_067938	-	predicted	---
5	53723416	53723420	-	53723419	23	57	XLOC_067938	-	predicted	---
5	53752707	53752707	-	53752707	3	3	ENSSSCG00000000567	-	ORF	*SOX5*
5	54398698	54398698	-	54398698	3	3	---	---	intergenic	---

5	54645708	54645708	+	54645708	4	4	XLOC_066979	+	predicted	---
5	54719897	54719909	-	54719907	8	35	ENSSSCG00000000572	-	3'UTR	*CMAS*
5	54736286	54736286	-	54736286	10	10	ENSSSCG00000000572	-	intron	*CMAS*
5	54912942	54912942	+	54912942	3	3	ENSSSCG00000000571	+	intron	---
5	55097018	55097018	+	55097018	5	5	ENSSSCG00000000576	+	ORF	---
5	55107295	55107296	+	55107296	28	33	ENSSSCG00000000576	+	intron	---
5	55191550	55191550	+	55191550	4	4	ENSSSCG00000000577	+	3'UTR	*GYS2*
5	55192260	55192298	+	55192266	51	139	XLOC_066985	+	predicted	---
5	55196920	55196923	+	55196923	16	36	XLOC_066985	+	predicted	---
5	55215655	55215655	-	55215655	20	20	ENSSSCG00000000579	-	3'UTR	*GOLT1B*
5	55217627	55217634	-	55217627	4	8	ENSSSCG00000000579	-	3'UTR	*GOLT1B*
5	55271565	55271565	+	55271565	3	3	ENSSSCG00000000580	+	3'UTR	*RECQL*
5	55272743	55272748	+	55272748	6	9	XLOC_066986	+	predicted	---
5	55272753	55272753	-	55272753	9	9	ENSSSCG00000000583	-	3'UTR	*PYROXD1*
5	55403715	55403720	+	55403719	43	74	ENSSSCG00000000584	+	3'UTR	*SLCO1A2*
5	55411447	55411451	-	55411449	29	40	ENSSSCG00000021998	-	3'UTR	---
5	55411601	55411601	-	55411601	3	3	ENSSSCG00000021998	-	3'UTR	---
5	55412320	55412320	-	55412320	7	7	ENSSSCG00000021998	-	3'UTR	---
5	55412384	55412393	-	55412384	9	13	ENSSSCG00000021998	-	3'UTR	---
5	55412966	55412973	-	55412967	7	21	ENSSSCG00000021998	-	3'UTR	---
5	55413110	55413132	-	55413111	13	19	ENSSSCG00000021998	-	3'UTR	---
5	55413702	55413702	-	55413702	4	4	ENSSSCG00000021998	-	3'UTR	---
5	55414274	55414274	-	55414274	3	3	ENSSSCG00000021998	-	3'UTR	---
5	55414360	55414398	-	55414398	5	9	ENSSSCG00000021998	-	3'UTR	---
5	55414536	55414536	-	55414536	16	16	ENSSSCG00000021998	-	3'UTR	---
5	55414667	55414667	-	55414667	3	3	ENSSSCG00000021998	-	ORF	---

5	55414715	55414715	-	55414715	3	3	ENSSSCG00000021998	-	ORF	---
5	55433398	55433399	-	55433398	12	18	ENSSSCG00000021998	-	ORF	---
5	55438810	55438810	-	55438810	11	11	ENSSSCG00000021998	-	ORF	---
5	55442971	55442971	-	55442971	3	3	ENSSSCG00000021998	-	intron	---
5	55943466	55943466	-	55943466	3	3	ENSSSCG00000028662	-	intron	*PDE3A*
5	55956399	55956399	-	55956399	4	4	ENSSSCG00000028662	-	intron	*PDE3A*
5	56638217	56638217	+	56638217	4	4	---	---	intergenic	---
5	56815892	56815892	+	56815892	3	3	---	---	intergenic	---
5	56828300	56828302	+	56828302	4	7	---	---	intergenic	---
5	56914018	56914018	-	56914018	3	3	ENSSSCG00000000587	-	3'UTR	*AEBP2*
5	57133482	57133482	-	57133482	10	10	ENSSSCG00000000588	-	intron	*PLEKHA5*
5	57190668	57190668	+	57190668	3	3	---	---	intergenic	---
5	57296992	57296992	+	57296992	3	3	---	---	intergenic	---
5	57679457	57679475	-	57679471	601	1130	---	---	intergenic	---
5	57679616	57679616	-	57679616	8	8	ENSSSCG00000000589	-	ORF	*CAPZA3*
5	57680124	57680124	-	57680124	6	6	ENSSSCG00000000589	-	ORF	*CAPZA3*
5	57680214	57680214	-	57680214	34	34	ENSSSCG00000000589	-	ORF	*CAPZA3*
5	57680262	57680262	-	57680262	3	3	ENSSSCG00000000589	-	ORF	*CAPZA3*
5	57680391	57680391	-	57680391	3	3	XLOC_067953	-	predicted	---
5	57680473	57680480	+	57680473	8	12	---	---	intergenic	---
5	57680639	57680639	-	57680639	3	3	XLOC_067953	-	predicted	---
5	57724911	57724926	+	57724918	32	140	XLOC_066999	+	predicted	---
5	57747738	57747740	-	57747738	3	6	XLOC_067954	-	predicted	---
5	58250299	58250299	+	58250299	3	3	---	---	intergenic	---
5	58254745	58254745	-	58254745	3	3	---	---	intergenic	---
5	58389270	58389270	+	58389270	3	3	---	---	intergenic	---

5	58537381	58537381	-	58537381	4	4	---	---	intergenic	---
5	58598613	58598613	+	58598613	3	3	---	---	intergenic	---
5	58649900	58649900	+	58649900	4	4	---	---	intergenic	---
5	59158464	59158464	+	59158464	3	3	---	---	intergenic	---
5	59189303	59189303	+	59189303	3	3	---	---	intergenic	---
5	59218542	59218542	-	59218542	4	4	---	---	intergenic	---
5	59316017	59316017	+	59316017	7	7	---	---	intergenic	---
5	59752162	59752162	-	59752162	7	7	---	---	intergenic	---
5	59878548	59878564	-	59878548	5	8	ENSSSCG00000000599	-	3'UTR	*DERA*
5	59990578	59990578	+	59990578	3	3	---	---	intergenic	---
5	60006585	60006586	-	60006585	56	60	ENSSSCG00000021410	-	3'UTR	*STRAP*
5	60006661	60006668	-	60006668	11	16	ENSSSCG00000021410	-	3'UTR	*STRAP*
5	60041789	60041789	-	60041789	3	3	---	---	intergenic	---
5	60286287	60286287	+	60286287	3	3	ENSSSCG00000000601	+	3'UTR	*PTPRO*
5	60303026	60303028	-	60303027	12	24	ENSSSCG00000000600	-	3'UTR	*EPS8*
5	60987343	60987351	+	60987345	59	149	ENSSSCG00000000604	+	3'UTR	*ARHGDIB*
5	61058309	61058319	+	61058316	175	335	ENSSSCG00000000606	+	3'UTR	*MGP*
5	61058724	61058724	+	61058724	12	12	XLOC_067015	+	predicted	---
5	61094196	61094196	+	61094196	6	6	---	---	intergenic	---
5	61115619	61115620	+	61115619	20	30	ENSSSCG00000000607	+	intron	*ART4*
5	61117274	61117274	+	61117274	3	3	ENSSSCG00000000607	+	intron	*ART4*
5	61123760	61123769	-	61123766	19	50	XLOC_067969	-	predicted	---
5	61123807	61123807	-	61123807	4	4	XLOC_067969	-	predicted	---
5	61123947	61123947	-	61123947	3	3	XLOC_067969	-	predicted	---
5	61141271	61141271	+	61141271	6	6	ENSSSCG00000000611	+	ORF	*WBP11*
5	61205050	61205054	+	61205054	11	15	ENSSSCG00000000611	+	3'UTR	*WBP11*

5	61205093	61205105	+	61205099	59	120	ENSSSCG00000000611	+	3'UTR	*WBP11*
5	61265952	61265952	-	61265952	4	4	ENSSSCG00000026724	-	intron	---
5	61296823	61296823	+	61296823	5	5	ENSSSCG00000000613	+	intron	*GUCY2C*
5	61372090	61372090	-	61372090	5	5	ENSSSCG00000027602	-	intron	*PLBD1*
5	61387973	61387973	+	61387973	7	7	XLOC_067022	+	predicted	---
5	61390432	61390432	+	61390432	3	3	---	---	intergenic	---
5	61425814	61425821	-	61425814	4	7	---	---	intergenic	---
5	61444261	61444261	-	61444261	6	6	ENSSSCG00000000612	-	3'UTR	*ATF7IP*
5	61448225	61448237	-	61448225	5	9	ENSSSCG00000000612	-	3'UTR	*ATF7IP*
5	61472062	61472072	-	61472072	4	10	---	---	intergenic	---
5	61502813	61502813	-	61502813	3	3	---	---	intergenic	---
5	62486575	62486575	+	62486575	3	3	---	---	intergenic	---
5	62512779	62512779	-	62512779	3	3	---	---	intergenic	---
5	62605815	62605815	-	62605815	3	3	---	---	intergenic	---
5	62662711	62662734	-	62662714	6	29	---	---	intergenic	---
5	62694001	62694001	+	62694001	3	3	---	---	intergenic	---
5	62756412	62756412	+	62756412	3	3	---	---	intergenic	---
5	62757700	62757701	+	62757700	4	7	---	---	intergenic	---
5	62801513	62801514	+	62801513	9	12	XLOC_067029	+	predicted	---
5	62824654	62824656	-	62824656	8	11	ENSSSCG00000000619	-	intron	*KIAA1467*
5	62857893	62857895	+	62857893	5	10	---	---	intergenic	---
5	63132921	63132921	+	63132921	3	3	ENSSSCG00000024152	+	intron	*ETV6*
5	63316016	63316016	+	63316016	7	7	ENSSSCG00000024152	+	ORF	*ETV6*
5	63462263	63462267	+	63462263	6	9	---	---	intergenic	---
5	63690607	63690632	-	63690608	35	99	XLOC_067981	-	predicted	---
5	63722499	63722499	+	63722499	8	8	---	---	intergenic	---

5	63725980	63725980	+	63725980	3	3	---	---	intergenic	---
5	63864857	63864857	-	63864857	4	4	---	---	intergenic	---
5	63883754	63883756	-	63883755	14	23	XLOC_067984	-	predicted	---
5	63883795	63883795	-	63883795	6	6	XLOC_067984	-	predicted	---
5	63885834	63885834	-	63885834	5	5	XLOC_067984	-	predicted	---
5	64021737	64021737	+	64021737	6	6	---	---	intergenic	---
5	64052878	64052879	-	64052878	3	6	XLOC_067987	-	predicted	---
5	64081369	64081369	+	64081369	4	4	ENSSSCG00000000633	+	intron	*YBX3*
5	64082062	64082067	+	64082066	29	51	ENSSSCG00000000633	+	intron	*YBX3*
5	64083770	64083771	+	64083770	127	136	ENSSSCG00000000633	+	3'UTR	*YBX3*
5	64084248	64084248	+	64084248	4	4	ENSSSCG00000000633	+	3'UTR	*YBX3*
5	64084400	64084400	+	64084400	4	4	ENSSSCG00000000633	+	3'UTR	*YBX3*
5	64091037	64091041	-	64091037	8	13	---	---	intergenic	---
5	64158167	64158167	+	64158167	3	3	ENSSSCG00000000635	+	3'UTR	*MAGOHB*
							ENSSSCG00000000635	+	ORF	---
5	64158555	64158556	+	64158555	6	9	ENSSSCG00000000635	+	3'UTR	*MAGOHB*
							ENSSSCG00000000635	+	ORF	---
5	64378321	64378321	+	64378321	5	5	ENSSSCG00000000641	+	3'UTR	*KLRK1*
5	64542875	64542875	-	64542875	3	3	ENSSSCG00000000648	-	3'UTR	*CLEC7A*
5	64543746	64543746	-	64543746	3	3	ENSSSCG00000000648	-	3'UTR	*CLEC7A*
5	64579162	64579163	-	64579162	6	10	ENSSSCG00000000647	-	3'UTR	*OLR1*
5	64747869	64747879	+	64747878	58	135	XLOC_067063	+	predicted	---
5	64769540	64769540	+	64769540	3	3	ENSSSCG00000000652	+	3'UTR	*CLEC12A*
5	64797157	64797157	+	64797157	5	5	XLOC_067065	+	predicted	---
5	64797907	64797907	+	64797907	4	4	XLOC_067065	+	predicted	---
5	64823704	64823718	+	64823716	96	191	---	---	intergenic	---

5	64856555	64856556	+	64856555	5	8	ENSSSCG00000000656	+	3'UTR	*CLEC2B*
5	64857027	64857027	+	64857027	13	13	ENSSSCG00000000656	+	3'UTR	*CLEC2B*
5	64956614	64956628	+	64956614	25	37	ENSSSCG00000000654	+	3'UTR	*CLECL1*
5	65023646	65023652	-	65023646	15	22	XLOC_068005	-	predicted	---
5	65023786	65023794	-	65023793	13	23	ENSSSCG00000000657	-	3'UTR	*CLEC2D*
5	65191858	65191860	+	65191858	4	7	ENSSSCG00000024402	+	ORF	---
5	65195553	65195553	+	65195553	3	3	ENSSSCG00000024402	+	ORF	---
5	65195599	65195599	+	65195599	3	3	ENSSSCG00000024402	+	ORF	---
5	65199898	65199898	+	65199898	10	10	ENSSSCG00000024402	+	ORF	---
5	65201700	65201700	+	65201700	4	4	ENSSSCG00000024402	+	ORF	---
5	65202805	65202805	+	65202805	51	51	ENSSSCG00000024402	+	ORF	---
5	65202860	65202860	+	65202860	4	4	ENSSSCG00000024402	+	ORF	---
5	65212374	65212374	+	65212374	3	3	ENSSSCG00000024402	+	ORF	---
5	65220168	65220168	+	65220168	7	7	ENSSSCG00000024402	+	intron	---
5	65226478	65226478	+	65226478	3	3	ENSSSCG00000024402	+	ORF	---
5	65233473	65233503	+	65233473	9	12	ENSSSCG00000024402	+	3'UTR	---
5	65233536	65233554	+	65233547	276	793	ENSSSCG00000024402	+	3'UTR	---
5	65234671	65234671	+	65234671	6	6	---	---	intergenic	---
5	65275305	65275305	+	65275305	6	6	ENSSSCG00000000660	+	intron	*A2M*
							ENSSSCG00000000660	+	ORF	---
5	65279660	65279669	+	65279661	17	38	---	---	intergenic	---
5	65283049	65283049	+	65283049	3	3	---	---	intergenic	---
5	65286219	65286225	+	65286219	3	6	---	---	intergenic	---
5	65288622	65288622	+	65288622	7	7	---	---	intergenic	---
5	65292325	65292325	+	65292325	3	3	---	---	intergenic	---
5	65295762	65295762	+	65295762	7	7	---	---	intergenic	---

5	65295823	65295836	+	65295836	35	38	---	---	intergenic	---
5	65296357	65296357	+	65296357	18	18	---	---	intergenic	---
5	65301232	65301232	+	65301232	6	6	---	---	intergenic	---
5	65302065	65302090	+	65302090	73	78	---	---	intergenic	---
5	65302950	65302958	+	65302951	7	13	---	---	intergenic	---
5	65305766	65305766	+	65305766	4	4	---	---	intergenic	---
5	65314855	65314861	+	65314861	6	10	---	---	intergenic	---
5	65315157	65315157	+	65315157	4	4	---	---	intergenic	---
5	65316462	65316462	+	65316462	9	9	---	---	intergenic	---
5	65319853	65319854	+	65319854	6	9	---	---	intergenic	---
5	65320276	65320278	+	65320276	4	7	ENSSSCG00000000660	+	3'UTR	*A2M*
							ENSSSCG00000000660	+	ORF	---
5	65320328	65320345	+	65320332	298	1222	ENSSSCG00000000660	+	3'UTR	*A2M*
							ENSSSCG00000000660	+	ORF	---
5	65320383	65320396	+	65320385	56	126	ENSSSCG00000000660	+	3'UTR	*A2M*
							ENSSSCG00000000660	+	ORF	---
5	65331959	65331959	+	65331959	4	4	---	---	intergenic	---
5	65378379	65378385	+	65378382	12	36	XLOC_067078	+	predicted	---
5	65431919	65431919	+	65431919	4	4	XLOC_067079	+	predicted	---
5	65548935	65548935	-	65548935	3	3	---	---	intergenic	---
5	65564409	65564410	+	65564410	16	20	XLOC_067080	+	predicted	---
5	65587528	65587528	+	65587528	3	3	ENSSSCG00000000666	+	intron	*MFAP5*
5	65666055	65666055	-	65666055	3	3	XLOC_068013	-	predicted	---
5	65676308	65676309	-	65676308	3	6	---	---	intergenic	---
5	65715415	65715423	+	65715423	11	16	ENSSSCG00000000670	+	ORF	---
5	65779002	65779002	+	65779002	12	12	---	---	intergenic	---

5	65810631	65810642	+	65810642	11	22	ENSSSCG00000022925	+	3'UTR	*SLC2A3*
5	65810870	65810870	+	65810870	4	4	ENSSSCG00000022925	+	3'UTR	*SLC2A3*
5	65901856	65901858	+	65901856	6	11	ENSSSCG00000000672	+	intron	---
5	65956835	65956837	+	65956835	4	7	ENSSSCG00000025252	+	3'UTR	*PEX5*
5	66030450	66030457	-	66030450	9	15	XLOC_068019	-	predicted	---
5	66080078	66080078	-	66080078	5	5	XLOC_068021	-	predicted	---
5	66082489	66082489	-	66082489	3	3	XLOC_068021	-	predicted	---
5	66088244	66088249	-	66088245	4	10	ENSSSCG00000030876	-	3'UTR	*RBP5*
5	66088370	66088376	-	66088371	23	33	ENSSSCG00000030876	-	3'UTR	*RBP5*
5	66089449	66089476	-	66089454	8	20	---	---	intergenic	---
5	66154719	66154719	-	66154719	8	8	---	---	intergenic	---
5	66170058	66170058	-	66170058	25	25	---	---	intergenic	---
5	66182235	66182247	+	66182246	19	42	ENSSSCG00000027205	+	3'UTR	*LPCAT3*
5	66182468	66182472	-	66182470	57	101	ENSSSCG00000026812	-	3'UTR	---
5	66188647	66188647	+	66188647	3	3	ENSSSCG00000024655	+	ORF	*PHB2*
5	66192613	66192621	+	66192619	76	125	XLOC_067090	+	predicted	---
5	66198250	66198255	-	66198252	68	79	ENSSSCG00000025537	-	3'UTR	*PTPN6*
5	66216249	66216272	-	66216268	96	257	ENSSSCG00000026877	-	3'UTR	*C12orf57*
5	66218918	66218921	-	66218918	16	19	XLOC_068025	-	predicted	---
5	66236691	66236691	-	66236691	4	4	XLOC_068026	-	predicted	---
5	66246065	66246082	-	66246079	16	78	---	---	intergenic	---
5	66274755	66274774	-	66274771	22	60	XLOC_068027	-	predicted	---
5	66274758	66274759	+	66274759	15	21	ENSSSCG00000025460	+	3'UTR	*SPSB2*
5	66274854	66274878	-	66274874	237	432	XLOC_068027	-	predicted	---
5	66275357	66275357	-	66275357	4	4	ENSSSCG00000000685	-	ORF	*TPI1*
5	66279249	66279271	-	66279251	42	116	ENSSSCG00000000684	-	3'UTR	*USP5*

5	66297400	66297415	+	66297402	58	81	XLOC_067094	+	predicted	---
5	66326573	66326573	-	66326573	4	4	ENSSSCG00000000687	-	3'UTR	*CD4*
5	66371524	66371535	-	66371524	40	84	XLOC_068033	-	predicted	---
5	66372430	66372442	-	66372442	11	20	XLOC_068033	-	predicted	---
5	66391589	66391589	+	66391589	5	5	ENSSSCG00000000690	+	3'UTR	*MLF2*
5	66405480	66405482	-	66405480	8	11	ENSSSCG00000000691	-	3'UTR	---
5	66406104	66406106	-	66406105	23	33	ENSSSCG00000000691	-	3'UTR	---
5	66406801	66406801	-	66406801	5	5	ENSSSCG00000000691	-	intron	---
5	66443816	66443816	+	66443816	5	5	ENSSSCG00000000693	+	3'UTR	*NCAPD2*
							ENSSSCG00000000692	+	intron	*PIANP*
5	66448766	66448775	+	66448773	42	57	ENSSSCG00000000694	+	intron	*GAPDH*
							ENSSSCG00000000692	+	intron	*PIANP*
5	66449127	66449127	+	66449127	3	3	ENSSSCG00000000694	+	intron	*GAPDH*
							ENSSSCG00000000692	+	intron	*PIANP*
5	66449358	66449372	-	66449369	22	66	XLOC_068036	-	predicted	---
5	66449490	66449516	+	66449511	1434	4648	ENSSSCG00000000694	+	3'UTR	*GAPDH*
							ENSSSCG00000000692	+	intron	*PIANP*
5	66450170	66450174	-	66450170	3	6	XLOC_068036	-	predicted	---
5	66450765	66450765	-	66450765	6	6	XLOC_068036	-	predicted	---
5	66463609	66463618	-	66463612	15	45	ENSSSCG00000000696	-	3'UTR	*NOP2*
5	66473918	66473923	-	66473918	30	49	XLOC_068036	-	predicted	---
5	66473942	66473955	+	66473942	5	8	ENSSSCG00000000692	+	intron	*PIANP*
5	66474184	66474187	-	66474184	13	16	XLOC_068036	-	predicted	---
5	66480308	66480309	-	66480308	8	12	ENSSSCG00000000697	-	ORF	*CHD4*
5	66480697	66480709	-	66480704	45	65	ENSSSCG00000000697	-	ORF	*CHD4*
5	66488315	66488315	-	66488315	6	6	ENSSSCG00000000697	-	ORF	*CHD4*

5	66509648	66509650	+	66509648	12	15	ENSSSCG00000000692	+	intron	*PIANP*
5	66513836	66513836	+	66513836	3	3	ENSSSCG00000000692	+	intron	*PIANP*
5	66530444	66530453	-	66530452	266	462	ENSSSCG00000000700	-	3'UTR	*ACRBP*
5	66541630	66541630	-	66541630	3	3	XLOC_068039	-	predicted	---
5	66541924	66541929	-	66541927	13	41	XLOC_068039	-	predicted	---
5	66543688	66543693	+	66543688	9	14	ENSSSCG00000000692	+	intron	*PIANP*
5	66544239	66544241	+	66544239	3	6	ENSSSCG00000000692	+	intron	*PIANP*
5	66630284	66630284	+	66630284	3	3	---	---	intergenic	---
5	66630490	66630522	+	66630511	210	453	---	---	intergenic	---
5	66649708	66649719	-	66649712	11	37	ENSSSCG00000000704	-	3'UTR	*TAPBPL*
5	66658753	66658753	-	66658753	8	8	ENSSSCG00000000705	-	3'UTR	*CD27*
5	66732886	66732894	-	66732888	17	46	ENSSSCG00000000706	-	3'UTR	*LTBR*
5	66793285	66793290	+	66793289	38	55	XLOC_067111	+	predicted	---
5	67468223	67468223	-	67468223	3	3	---	---	intergenic	---
5	67647152	67647152	-	67647152	5	5	---	---	intergenic	---
5	67652616	67652616	-	67652616	3	3	XLOC_068047	-	predicted	---
5	67965978	67965985	-	67965980	137	254	ENSSSCG00000000719	-	3'UTR	---
5	67995953	67995953	+	67995953	3	3	XLOC_067119	+	predicted	---
5	68011772	68011772	+	68011772	3	3	ENSSSCG00000000720	+	ORF	*AKAP3*
5	68020681	68020686	+	68020681	36	63	XLOC_067119	+	predicted	---
5	68022247	68022250	-	68022250	7	10	XLOC_068055	-	predicted	---
5	68023255	68023255	-	68023255	4	4	ENSSSCG00000000721	-	ORF	*DYRK4*
5	68047527	68047527	+	68047527	5	5	---	---	intergenic	---
5	68078517	68078517	-	68078517	4	4	XLOC_068056	-	predicted	---
5	68123287	68123289	+	68123288	33	77	ENSSSCG00000000723	+	intron	*C12orf4*
5	68145464	68145464	+	68145464	4	4	XLOC_067120	+	predicted	---

5	68261696	68261696	+	68261696	3	3	ENSSSCG00000029028	+	3'UTR	*FGF23*
5	68266611	68266612	-	68266611	16	19	ENSSSCG00000024219	-	3'UTR	*TIGAR*
5	68654954	68654954	+	68654954	3	3	---	---	intergenic	---
5	68740289	68740289	+	68740289	6	6	---	---	intergenic	---
5	68777318	68777318	+	68777318	3	3	ENSSSCG00000000728	+	intron	*PARP11*
5	68788832	68788833	+	68788832	4	8	ENSSSCG00000000728	+	3'UTR	*PARP11*
5	68791232	68791232	+	68791232	3	3	ENSSSCG00000000728	+	3'UTR	*PARP11*
5	69067832	69067832	-	69067832	3	3	XLOC_068068	-	predicted	---
5	69072497	69072497	-	69072497	3	3	XLOC_068068	-	predicted	---
5	69074084	69074084	-	69074084	5	5	XLOC_068068	-	predicted	---
5	69112955	69112955	-	69112955	6	6	---	---	intergenic	---
5	69216055	69216055	+	69216055	3	3	XLOC_067131	+	predicted	---
5	69216963	69216963	+	69216963	5	5	XLOC_067131	+	predicted	---
5	69338597	69338597	-	69338597	3	3	ENSSSCG00000000735	-	intron	*TSPAN9*
5	69488556	69488556	-	69488556	4	4	ENSSSCG00000000737	-	3'UTR	*TULP3*
5	69526779	69526788	-	69526780	6	16	ENSSSCG00000000738	-	3'UTR	*RHNO1*
5	69549900	69549910	+	69549908	12	39	ENSSSCG00000000739	+	3'UTR	*FOXM1*
5	69556827	69556827	+	69556827	3	3	XLOC_067134	+	predicted	---
5	69579205	69579205	-	69579205	9	9	ENSSSCG00000000742	-	3'UTR	*ITFG2*
5	69599791	69599806	-	69599805	130	153	ENSSSCG00000000743	-	3'UTR	*FKBP4*
5	69600183	69600183	-	69600183	3	3	ENSSSCG00000000743	-	3'UTR	*FKBP4*
5	69600328	69600364	-	69600350	228	667	ENSSSCG00000000743	-	3'UTR	*FKBP4*
5	69603681	69603681	-	69603681	5	5	ENSSSCG00000000743	-	ORF	*FKBP4*
5	69630613	69630628	-	69630619	8	21	ENSSSCG00000000746	-	3'UTR	*WASH1*
5	69630616	69630619	+	69630616	5	8	XLOC_067136	+	predicted	---
5	69696019	69696020	-	69696019	6	9	---	---	intergenic	---

5	69729915	69729915	+	69729915	4	4	---	---	intergenic	---
5	69865019	69865019	-	69865019	5	5	XLOC_068080	-	predicted	---
5	69922505	69922510	+	69922505	12	16	XLOC_067140	+	predicted	---
5	70053793	70053793	-	70053793	7	7	ENSSSCG00000027540	-	3'UTR	---
5	70066323	70066323	-	70066323	3	3	ENSSSCG00000027540	-	intron	---
5	70155212	70155212	-	70155212	9	9	---	---	intergenic	---
5	70165263	70165263	-	70165263	3	3	XLOC_068085	-	predicted	---
5	70179945	70179962	+	70179962	9	12	XLOC_067144	+	predicted	---
5	70182271	70182290	+	70182290	9	16	XLOC_067144	+	predicted	---
5	70217028	70217028	-	70217028	3	3	XLOC_068086	-	predicted	---
5	70226876	70226879	-	70226876	3	6	XLOC_068086	-	predicted	---
5	70261538	70261539	-	70261538	4	8	XLOC_068086	-	predicted	---
5	70347354	70347356	+	70347355	40	56	ENSSSCG00000000753	+	intron	*WNK1*
5	70361282	70361282	+	70361282	5	5	ENSSSCG00000000753	+	ORF	*WNK1*
5	70367602	70367602	+	70367602	3	3	ENSSSCG00000000753	+	intron	*WNK1*
5	70406740	70406743	+	70406741	10	18	ENSSSCG00000000753	+	3'UTR	*WNK1*
5	70408316	70408322	+	70408316	4	7	ENSSSCG00000000753	+	3'UTR	*WNK1*
5	70408383	70408394	+	70408383	12	30	ENSSSCG00000000753	+	3'UTR	*WNK1*
5	70408516	70408518	+	70408518	4	10	ENSSSCG00000000753	+	3'UTR	*WNK1*
5	70409640	70409640	-	70409640	4	4	XLOC_068087	-	predicted	---
5	70655533	70655533	+	70655533	4	4	ENSSSCG00000000755	+	intron	*RAB6IP2*
5	70804651	70804651	+	70804651	3	3	---	---	intergenic	---
5	70873474	70873483	+	70873474	7	15	XLOC_067147	+	predicted	---
5	70984260	70984260	-	70984260	3	3	XLOC_068090	-	predicted	---
5	70996345	70996345	+	70996345	3	3	---	---	intergenic	---
5	70999361	70999373	-	70999373	28	31	XLOC_068092	-	predicted	---

5	71011565	71011565	-	71011565	4	4	ENSSSCG00000000757	-	ORF	*ADIPOR2*
5	71086585	71086585	-	71086585	3	3	XLOC_068093	-	predicted	---
5	71087279	71087279	-	71087279	3	3	XLOC_068093	-	predicted	---
5	71462305	71462305	-	71462305	8	8	XLOC_068096	-	predicted	---
5	71464667	71464679	+	71464671	23	52	---	---	intergenic	---
5	71604968	71604968	+	71604968	3	3	ENSSSCG00000000763	+	intron	---
5	71738913	71738913	-	71738913	4	4	XLOC_068099	-	predicted	---
5	71779578	71779586	+	71779586	9	12	ENSSSCG00000000765	+	3'UTR	*IL17RA*
5	71898948	71898950	+	71898948	46	58	---	---	intergenic	---
5	72017164	72017164	+	72017164	4	4	XLOC_067160	+	predicted	---
5	72024967	72024967	-	72024967	5	5	XLOC_068102	-	predicted	---
5	72025379	72025386	-	72025382	34	106	ENSSSCG00000000767	-	3'UTR	*ATP6V1E1*
5	72025524	72025546	-	72025527	122	292	ENSSSCG00000000767	-	3'UTR	*ATP6V1E1*
5	72035785	72035793	-	72035785	3	6	ENSSSCG00000000767	-	ORF	*ATP6V1E1*
5	72113206	72113209	+	72113206	12	19	ENSSSCG00000023043	+	3'UTR	*BCL2L13*
5	72116337	72116338	+	72116337	5	8	ENSSSCG00000023043	+	3'UTR	*BCL2L13*
5	72118659	72118659	-	72118659	3	3	---	---	intergenic	---
5	72156764	72156764	-	72156764	12	12	XLOC_068104	-	predicted	---
5	72495841	72495841	+	72495841	11	11	ENSSSCG00000027190	+	3'UTR	---
5	72527220	72527227	+	72527225	299	529	ENSSSCG00000000773	+	3'UTR	---
5	72533191	72533191	+	72533191	3	3	XLOC_067165	+	predicted	---
5	72595953	72595953	+	72595953	16	16	ENSSSCG00000000774	+	3'UTR	*USP18*
5	72806924	72806924	-	72806924	4	4	ENSSSCG00000000778	-	intron	*CPNE8*
5	73369324	73369324	+	73369324	3	3	XLOC_067168	+	predicted	---
5	73379320	73379322	-	73379320	8	14	XLOC_068108	-	predicted	---
5	73379812	73379822	-	73379821	8	14	XLOC_068108	-	predicted	---

5	73481992	73481992	-	73481992	3	3	ENSSSCG00000000779	-	ORF	*KIF21A*
5	73488290	73488290	-	73488290	5	5	ENSSSCG00000000779	-	ORF	*KIF21A*
5	73745154	73745154	+	73745154	4	4	---	---	intergenic	---
5	73866292	73866293	+	73866292	5	10	---	---	intergenic	---
5	74238778	74238778	-	74238778	7	7	ENSSSCG00000000783	-	intron	---
5	74325924	74325924	-	74325924	3	3	ENSSSCG00000000783	-	intron	---
5	74443424	74443424	-	74443424	8	8	---	---	intergenic	---
5	74449929	74449929	+	74449929	3	3	---	---	intergenic	---
5	74499038	74499038	+	74499038	3	3	---	---	intergenic	---
5	74666467	74666467	+	74666467	4	4	---	---	intergenic	---
5	75191685	75191685	+	75191685	5	5	ENSSSCG00000000789	+	intron	*CNTN1*
5	75561963	75561963	-	75561963	3	3	---	---	intergenic	---
5	76141138	76141138	-	76141138	11	11	---	---	intergenic	---
5	76383060	76383060	+	76383060	4	4	ENSSSCG00000000793	+	intron	*PPHLN1*
5	76432643	76432644	+	76432644	10	14	ENSSSCG00000000793	+	3'UTR	*PPHLN1*
5	76434661	76434661	+	76434661	15	15	ENSSSCG00000000793	+	3'UTR	*PPHLN1*
5	76437128	76437128	-	76437128	3	3	---	---	intergenic	---
5	76447298	76447302	-	76447298	22	44	XLOC_068120	-	predicted	---
5	76589135	76589135	-	76589135	4	4	---	---	intergenic	---
5	76605335	76605336	-	76605336	6	11	---	---	intergenic	---
5	76605360	76605360	+	76605360	3	3	---	---	intergenic	---
5	76673569	76673569	+	76673569	9	9	---	---	intergenic	---
5	76917719	76917719	-	76917719	3	3	---	---	intergenic	---
5	77200546	77200547	+	77200547	18	28	---	---	intergenic	---
5	77311392	77311396	-	77311395	15	33	---	---	intergenic	---
5	77589497	77589499	+	77589497	11	14	---	---	intergenic	---

5	77594951	77594951	+	77594951	4	4	---	---	intergenic	---
5	77606008	77606008	+	77606008	4	4	---	---	intergenic	---
5	77618052	77618052	-	77618052	5	5	ENSSSCG00000000800	-	3'UTR	*PUS7L*
5	77672326	77672326	+	77672326	3	3	XLOC_067182	+	predicted	---
5	77681094	77681094	-	77681094	11	11	ENSSSCG00000000798	-	3'UTR	*TWF1*
5	77683140	77683140	-	77683140	3	3	ENSSSCG00000000798	-	ORF	*TWF1*
5	77685593	77685593	-	77685593	3	3	ENSSSCG00000000798	-	intron	*TWF1*
							ENSSSCG00000000798	-	ORF	*TWF1*
5	78092588	78092588	-	78092588	3	3	---	---	intergenic	---
5	78417213	78417213	-	78417213	5	5	XLOC_068127	-	predicted	---
5	79217138	79217138	+	79217138	3	3	ENSSSCG00000000804	+	3'UTR	*ANO6*
5	79219971	79219971	+	79219971	4	4	XLOC_067185	+	predicted	---
5	79478485	79478485	+	79478485	5	5	---	---	intergenic	---
5	79620488	79620488	+	79620488	3	3	ENSSSCG00000030685	+	intron	*ARID2*
5	79644822	79644822	+	79644822	5	5	ENSSSCG00000030685	+	intron	*ARID2*
5	79708909	79708909	+	79708909	5	5	ENSSSCG00000030685	+	intron	*ARID2*
5	79725978	79725986	+	79725985	12	25	ENSSSCG00000030685	+	3'UTR	*ARID2*
5	79736669	79736671	-	79736669	10	15	ENSSSCG00000000806	-	3'UTR	*SCAF11*
5	79738636	79738637	-	79738636	6	10	ENSSSCG00000000806	-	3'UTR	*SCAF11*
5	79745160	79745160	-	79745160	3	3	---	---	intergenic	---
5	79762505	79762505	-	79762505	4	4	ENSSSCG00000000806	-	intron	*SCAF11*
5	79899893	79899894	+	79899893	6	12	---	---	intergenic	---
5	79915289	79915289	-	79915289	7	7	---	---	intergenic	---
5	79931197	79931197	-	79931197	3	3	XLOC_068136	-	predicted	---
5	80002388	80002388	-	80002388	7	7	XLOC_068136	-	predicted	---
5	80129574	80129574	+	80129574	3	3	---	---	intergenic	---

5	80385640	80385640	+	80385640	13	13	---	---	intergenic	---
5	80391345	80391353	-	80391353	69	136	XLOC_068139	-	predicted	---
5	80392193	80392193	-	80392193	3	3	ENSSSCG00000026597	-	3'UTR	*SLC38A4*
5	80393155	80393155	-	80393155	8	8	ENSSSCG00000026597	-	3'UTR	*SLC38A4*
5	80393467	80393467	-	80393467	3	3	ENSSSCG00000026597	-	3'UTR	*SLC38A4*
5	80393518	80393518	-	80393518	9	9	ENSSSCG00000026597	-	3'UTR	*SLC38A4*
5	80489036	80489036	-	80489036	7	7	---	---	intergenic	---
5	80650589	80650589	-	80650589	3	3	XLOC_068141	-	predicted	---
5	80757452	80757452	+	80757452	9	9	---	---	intergenic	---
5	80944334	80944334	+	80944334	4	4	---	---	intergenic	---
5	81196799	81196801	-	81196801	38	53	---	---	intergenic	---
5	81404535	81404535	+	81404535	4	4	ENSSSCG00000028397	+	intron	---
5	81538762	81538762	-	81538762	8	8	---	---	intergenic	---
5	81596985	81596985	-	81596985	3	3	XLOC_068159	-	predicted	---
5	81597606	81597606	-	81597606	3	3	XLOC_068159	-	predicted	---
5	81598181	81598181	-	81598181	3	3	XLOC_068159	-	predicted	---
5	81598834	81598834	-	81598834	3	3	ENSSSCG00000000816	-	3'UTR	*SENP1*
5	81695160	81695167	+	81695166	71	100	---	---	intergenic	---
5	81696227	81696227	-	81696227	4	4	ENSSSCG00000021024	-	3'UTR	*ASB8*
5	81696305	81696306	-	81696305	4	7	ENSSSCG00000021024	-	3'UTR	*ASB8*
5	81697211	81697225	-	81697218	7	19	ENSSSCG00000021024	-	3'UTR	*ASB8*
5	81697321	81697335	-	81697323	11	30	ENSSSCG00000021024	-	3'UTR	*ASB8*
5	81767807	81767807	-	81767807	3	3	---	---	intergenic	---
5	81892925	81892944	+	81892927	41	146	XLOC_067209	+	predicted	---
5	81900564	81900565	-	81900564	4	7	ENSSSCG00000021654	-	3'UTR	*ZNF641*
5	81992705	81992706	+	81992706	21	24	---	---	intergenic	---

5	82065173	82065173	-	82065173	3	3	---	---	intergenic	---
5	82163227	82163233	-	82163230	20	69	XLOC_068169	-	predicted	---
5	82297760	82297776	+	82297761	65	291	XLOC_067212	+	predicted	---
5	82542274	82542274	+	82542274	6	6	---	---	intergenic	---
5	82645878	82645878	+	82645878	6	6	ENSSSCG00000000840	+	3'UTR	*KIAA1033*
5	82650763	82650767	-	82650766	20	59	ENSSSCG00000000841	-	3'UTR	*APPL2*
5	82744381	82744381	-	82744381	4	4	---	---	intergenic	---
5	82744988	82744996	-	82744988	4	8	XLOC_068174	-	predicted	---
5	82779982	82779982	+	82779982	7	7	ENSSSCG00000000842	+	3'UTR	*KANSL2*
5	82958192	82958192	+	82958192	5	5	ENSSSCG00000000839	+	3'UTR	*ALDH1L2*
5	82962767	82962767	-	82962767	4	4	XLOC_068177	-	predicted	---
5	82962965	82962967	-	82962966	30	48	XLOC_068177	-	predicted	---
5	83118124	83118128	+	83118128	8	14	XLOC_067223	+	predicted	---
5	83118201	83118204	+	83118202	15	38	XLOC_067223	+	predicted	---
5	83121373	83121375	+	83121375	8	15	XLOC_067223	+	predicted	---
5	83121500	83121503	+	83121502	13	21	XLOC_067223	+	predicted	---
5	83186190	83186190	+	83186190	3	3	---	---	intergenic	---
5	83315609	83315609	-	83315609	9	9	ENSSSCG00000000837	-	intron	*CHST11*
5	83684835	83684835	+	83684835	4	4	ENSSSCG00000000843	+	ORF	*TXNRD1*
5	83700531	83700540	+	83700535	16	38	ENSSSCG00000000843	+	3'UTR	*TXNRD1*
5	83700708	83700708	+	83700708	3	3	ENSSSCG00000000843	+	3'UTR	*TXNRD1*
5	83701650	83701656	+	83701653	42	98	ENSSSCG00000000843	+	3'UTR	*TXNRD1*
5	83703469	83703471	+	83703469	3	6	XLOC_067226	+	predicted	---
5	83921438	83921438	+	83921438	8	8	ENSSSCG00000000844	+	3'UTR	*NFYB*
5	83921614	83921615	+	83921614	13	16	ENSSSCG00000000844	+	3'UTR	*NFYB*
5	83922952	83922952	+	83922952	3	3	ENSSSCG00000000844	+	3'UTR	*NFYB*

5	83941007	83941007	-	83941007	3	3	ENSSSCG00000000846	-	3'UTR	*HCFC2*
5	84013544	84013544	+	84013544	6	6	ENSSSCG00000000848	+	intron	*GLT8D2*
5	84014049	84014053	+	84014052	16	37	ENSSSCG00000000848	+	intron	*GLT8D2*
5	84014383	84014383	+	84014383	6	6	ENSSSCG00000000848	+	intron	*GLT8D2*
5	84016165	84016179	-	84016175	191	488	XLOC_068185	-	predicted	---
5	84017008	84017041	-	84017008	9	18	ENSSSCG00000000849	-	ORF	*HSP90B1*
5	84017401	84017407	-	84017401	7	10	ENSSSCG00000000849	-	ORF	*HSP90B1*
5	84018918	84018918	-	84018918	4	4	ENSSSCG00000000849	-	intron	*HSP90B1*
5	84019803	84019803	-	84019803	3	3	ENSSSCG00000000849	-	ORF	*HSP90B1*
5	84019914	84019914	-	84019914	3	3	ENSSSCG00000000849	-	ORF	*HSP90B1*
5	84048001	84048007	-	84048001	5	13	ENSSSCG00000000847	-	3'UTR	*TDG*
							ENSSSCG00000000849	-	intron	*HSP90B1*
5	84079510	84079515	-	84079510	21	36	ENSSSCG00000000849	-	intron	*HSP90B1*
5	84079581	84079581	-	84079581	6	6	ENSSSCG00000000849	-	ORF	*HSP90B1*
5	84081250	84081250	-	84081250	3	3	ENSSSCG00000000849	-	ORF	*HSP90B1*
5	84082781	84082798	-	84082790	6	13	ENSSSCG00000000849	-	ORF	*HSP90B1*
5	84219524	84219524	+	84219524	3	3	ENSSSCG00000000853	+	intron	---
5	84224473	84224473	+	84224473	4	4	XLOC_067233	+	predicted	---
5	84227619	84227619	-	84227619	3	3	---	---	intergenic	---
5	84227627	84227627	+	84227627	3	3	XLOC_067233	+	predicted	---
5	84228008	84228012	-	84228012	5	12	XLOC_068188	-	predicted	---
5	84229411	84229411	-	84229411	4	4	XLOC_068188	-	predicted	---
5	84331760	84331761	+	84331761	5	9	---	---	intergenic	---
5	84387631	84387631	+	84387631	3	3	ENSSSCG00000000854	+	intron	---
5	84593454	84593454	+	84593454	6	6	---	---	intergenic	---
5	84662973	84662973	+	84662973	5	5	---	---	intergenic	---

5	84859858	84859858	+	84859858	3	3	---	---	intergenic	---
5	85046166	85046166	-	85046166	3	3	---	---	intergenic	---
5	85208226	85208226	+	85208226	3	3	ENSSSCG00000000856	+	ORF	---
5	85228438	85228438	+	85228438	3	3	XLOC_067238	+	predicted	---
5	85673696	85673696	+	85673696	4	4	XLOC_067242	+	predicted	---
5	85783782	85783782	+	85783782	3	3	XLOC_067243	+	predicted	---
5	85955443	85955443	-	85955443	3	3	ENSSSCG00000000859	-	intron	*PARPBP*
5	86118872	86118873	+	86118872	17	21	XLOC_067248	+	predicted	---
5	86207839	86207839	+	86207839	3	3	XLOC_067250	+	predicted	---
5	86207926	86207934	+	86207928	30	42	XLOC_067250	+	predicted	---
5	86272196	86272204	-	86272204	6	10	ENSSSCG00000023972	-	3'UTR	*DRAM1*
5	86277353	86277353	-	86277353	3	3	ENSSSCG00000023972	-	intron	*DRAM1*
5	86413179	86413180	+	86413179	4	7	ENSSSCG00000000862	+	3'UTR	---
5	86422098	86422098	+	86422098	3	3	---	---	intergenic	---
5	86447387	86447387	+	86447387	5	5	ENSSSCG00000000863	+	intron	*SYCP3*
5	86450208	86450209	+	86450208	5	9	ENSSSCG00000000863	+	ORF	*SYCP3*
5	86453388	86453388	+	86453388	8	8	ENSSSCG00000000863	+	intron	*SYCP3*
5	86453793	86453818	+	86453816	92	121	ENSSSCG00000000863	+	3'UTR	*SYCP3*
5	86510405	86510405	-	86510405	5	5	XLOC_068206	-	predicted	---
5	86510779	86510791	-	86510791	17	21	XLOC_068206	-	predicted	---
5	86554421	86554423	-	86554423	10	26	ENSSSCG00000000866	-	intron	*MYBPC1*
5	86642166	86642166	-	86642166	3	3	XLOC_068206	-	predicted	---
5	86660009	86660011	+	86660009	3	6	---	---	intergenic	---
5	86678998	86679000	+	86678998	3	6	---	---	intergenic	---
5	86686581	86686582	-	86686581	4	8	---	---	intergenic	---
5	86686620	86686620	+	86686620	10	10	---	---	intergenic	---

5	86727381	86727381	-	86727381	3	3	---	---	intergenic	---
5	86749502	86749511	-	86749510	41	117	---	---	intergenic	---
5	86794495	86794495	+	86794495	4	4	ENSSSCG00000000870	+	ORF	*ARL1*
5	86804688	86804711	+	86804696	11	47	ENSSSCG00000000870	+	3'UTR	*ARL1*
5	86804855	86804874	+	86804855	10	17	ENSSSCG00000000870	+	3'UTR	*ARL1*
5	86804927	86804927	+	86804927	4	4	ENSSSCG00000000870	+	3'UTR	*ARL1*
5	86806930	86806935	+	86806934	26	34	ENSSSCG00000000870	+	3'UTR	*ARL1*
5	87828516	87828516	+	87828516	7	7	XLOC_067264	+	predicted	---
5	87829066	87829073	+	87829068	13	30	XLOC_067264	+	predicted	---
5	88006349	88006350	-	88006349	15	18	ENSSSCG00000000877	-	3'UTR	---
5	88006403	88006403	-	88006403	4	4	ENSSSCG00000000877	-	3'UTR	---
5	88094129	88094132	-	88094129	24	27	ENSSSCG00000025682	-	3'UTR	---
5	88223560	88223564	+	88223561	32	52	ENSSSCG00000026697	+	3'UTR	*UHRF1BP1L*
5	88223901	88223908	+	88223901	6	12	ENSSSCG00000026697	+	3'UTR	*UHRF1BP1L*
5	88223993	88223993	+	88223993	3	3	ENSSSCG00000026697	+	3'UTR	*UHRF1BP1L*
5	88224159	88224159	+	88224159	3	3	ENSSSCG00000026697	+	3'UTR	*UHRF1BP1L*
5	88371280	88371301	+	88371281	9	29	---	---	intergenic	---
5	88590544	88590544	+	88590544	3	3	ENSSSCG00000000880	+	intron	---
5	89472425	89472425	-	89472425	3	3	ENSSSCG00000000884	-	intron	---
5	89602338	89602338	+	89602338	3	3	---	---	intergenic	---
5	89607263	89607269	-	89607263	4	7	XLOC_068226	-	predicted	---
5	89607356	89607371	-	89607364	100	230	ENSSSCG00000000886	-	3'UTR	*SLC25A3*
5	89607482	89607482	-	89607482	9	9	ENSSSCG00000000886	-	3'UTR	*SLC25A3*
5	89640645	89640683	-	89640682	7	13	XLOC_068228	-	predicted	---
5	89860964	89860964	+	89860964	3	3	---	---	intergenic	---
5	89934749	89934749	-	89934749	6	6	XLOC_068229	-	predicted	---

5	90127861	90127861	-	90127861	3	3	---	---	intergenic	---
5	90228064	90228065	-	90228065	7	12	---	---	intergenic	---
5	90408110	90408115	+	90408110	7	14	XLOC_067282	+	predicted	---
5	90527758	90527758	+	90527758	3	3	---	---	intergenic	---
5	91146837	91146837	-	91146837	6	6	---	---	intergenic	---
5	91151130	91151130	+	91151130	4	4	---	---	intergenic	---
5	91180105	91180105	-	91180105	3	3	XLOC_068239	-	predicted	---
5	91196192	91196192	+	91196192	11	11	---	---	intergenic	---
5	91270797	91270797	-	91270797	5	5	---	---	intergenic	---
5	91281459	91281459	-	91281459	10	10	---	---	intergenic	---
5	91315451	91315451	+	91315451	3	3	ENSSSCG00000000890	+	ORF	*NEDD1*
5	91394645	91394645	+	91394645	3	3	XLOC_067288	+	predicted	---
5	91406312	91406312	-	91406312	5	5	---	---	intergenic	---
5	91506602	91506602	-	91506602	9	9	ENSSSCG00000023803	-	3'UTR	*ELK3*
5	91720979	91720982	+	91720981	25	49	ENSSSCG00000000891	+	3'UTR	*LTA4H*
5	91749987	91749991	+	91749990	23	57	ENSSSCG00000000892	+	intron	*HAL*
5	91754816	91754840	-	91754817	28	84	XLOC_068243	-	predicted	---
5	91765639	91765639	-	91765639	5	5	ENSSSCG00000000893	-	ORF	*AMDHD1*
5	91782838	91782838	+	91782838	12	12	ENSSSCG00000000894	+	ORF	*CCDC38*
5	91792343	91792344	+	91792344	9	13	ENSSSCG00000000894	+	ORF	*CCDC38*
5	91799706	91799724	+	91799724	8	11	ENSSSCG00000000894	+	intron	*CCDC38*
5	91814232	91814234	+	91814233	13	31	ENSSSCG00000000894	+	intron	*CCDC38*
5	91817370	91817370	+	91817370	3	3	XLOC_067293	+	predicted	---
5	91818389	91818396	-	91818396	33	58	ENSSSCG00000000895	-	3'UTR	*SNRPF*
5	91818652	91818654	+	91818654	6	9	XLOC_067293	+	predicted	---
5	91822000	91822000	-	91822000	4	4	ENSSSCG00000000895	-	intron	*SNRPF*

5	91823996	91823996	-	91823996	3	3	ENSSSCG00000000895	-	intron	*SNRPF*
5	91980642	91980642	+	91980642	5	5	ENSSSCG00000000896	+	intron	*NTN4*
5	91985584	91985584	+	91985584	3	3	XLOC_067295	+	predicted	---
5	92089554	92089555	+	92089554	3	6	ENSSSCG00000021307	+	3'UTR	*USP44*
5	92089613	92089618	+	92089615	35	58	ENSSSCG00000021307	+	3'UTR	*USP44*
5	92091185	92091188	+	92091185	8	17	ENSSSCG00000021307	+	3'UTR	*USP44*
5	92091626	92091631	-	92091631	14	21	ENSSSCG00000030608	-	3'UTR	*METAP2*
5	92093066	92093076	-	92093076	23	69	ENSSSCG00000030608	-	3'UTR	*METAP2*
5	92094616	92094616	-	92094616	3	3	ENSSSCG00000030608	-	ORF	*METAP2*
5	92101452	92101452	-	92101452	3	3	ENSSSCG00000030608	-	intron	*METAP2*
5	92211132	92211132	+	92211132	3	3	---	---	intergenic	---
5	92232278	92232279	-	92232278	7	12	ENSSSCG00000000900	-	3'UTR	---
5	92525854	92525854	+	92525854	4	4	ENSSSCG00000000902	+	3'UTR	*NR2C1*
5	92527230	92527230	+	92527230	5	5	ENSSSCG00000000902	+	3'UTR	*NR2C1*
5	92664017	92664025	+	92664020	68	112	ENSSSCG00000000905	+	3'UTR	*NDUFA12*
5	93165125	93165132	-	93165128	5	14	---	---	intergenic	---
5	93336395	93336395	+	93336395	4	4	ENSSSCG00000000907	+	intron	*PLXNC1*
5	93455305	93455305	+	93455305	3	3	ENSSSCG00000000907	+	intron	*PLXNC1*
5	93457517	93457517	+	93457517	4	4	ENSSSCG00000000907	+	3'UTR	*PLXNC1*
5	93459843	93459843	+	93459843	19	19	ENSSSCG00000000907	+	3'UTR	*PLXNC1*
5	93460476	93460476	-	93460476	5	5	ENSSSCG00000024563	-	3'UTR	*CEP83*
							ENSSSCG00000024563	-	intron	*CEP83*
5	93461068	93461073	-	93461073	10	15	ENSSSCG00000024563	-	3'UTR	*CEP83*
							ENSSSCG00000024563	-	intron	*CEP83*
5	93471271	93471271	-	93471271	5	5	ENSSSCG00000024563	-	intron	*CEP83*
5	93507210	93507210	-	93507210	3	3	ENSSSCG00000024563	-	intron	*CEP83*

5	93636883	93636919	+	93636883	25	53	ENSSSCG00000000908	+	3'UTR	*UBE2N*
5	93638448	93638448	+	93638448	3	3	XLOC_067304	+	predicted	---
5	93643050	93643059	-	93643052	29	79	ENSSSCG00000020903	-	3'UTR	*NUDT4*
5	93643730	93643730	-	93643730	3	3	ENSSSCG00000020903	-	3'UTR	*NUDT4*
5	93645459	93645469	-	93645461	26	44	ENSSSCG00000020903	-	3'UTR	*NUDT4*
5	93712447	93712447	-	93712447	11	11	XLOC_068265	-	predicted	---
5	93763022	93763029	-	93763027	24	99	XLOC_068262	-	predicted	---
5	93819824	93819827	-	93819827	7	11	XLOC_068266	-	predicted	---
5	94002687	94002689	+	94002689	5	13	XLOC_067309	+	predicted	---
5	94482978	94482979	+	94482979	8	15	ENSSSCG00000000912	+	intron	*EEA1*
5	94489190	94489195	+	94489192	9	22	ENSSSCG00000000912	+	3'UTR	*EEA1*
5	94492338	94492341	+	94492341	5	13	XLOC_067313	+	predicted	---
5	94894168	94894168	-	94894168	3	3	---	---	intergenic	---
5	94976237	94976237	+	94976237	4	4	---	---	intergenic	---
5	95087685	95087693	-	95087685	14	38	XLOC_068283	-	predicted	---
5	95091179	95091187	+	95091185	89	151	ENSSSCG00000000914	+	3'UTR	*BTG1*
5	95141663	95141663	-	95141663	4	4	---	---	intergenic	---
5	95181826	95181826	-	95181826	4	4	---	---	intergenic	---
5	95227507	95227507	+	95227507	4	4	---	---	intergenic	---
5	95894312	95894316	+	95894316	369	738	---	---	intergenic	---
5	95925076	95925076	+	95925076	3	3	XLOC_067323	+	predicted	---
5	96017386	96017386	-	96017386	4	4	---	---	intergenic	---
5	96125723	96125723	+	96125723	4	4	ENSSSCG00000000915	+	intron	*DCN*
5	96130346	96130346	+	96130346	5	5	ENSSSCG00000000915	+	ORF	*DCN*
5	96143718	96143720	+	96143719	74	96	ENSSSCG00000000915	+	3'UTR	*DCN*
5	96143978	96143978	+	96143978	3	3	XLOC_067323	+	predicted	---

5	96144023	96144023	+	96144023	13	13	XLOC_067323	+	predicted	---
5	96183381	96183382	+	96183381	32	38	ENSSSCG00000000916	+	3'UTR	*LUM*
5	96333278	96333278	+	96333278	3	3	ENSSSCG00000000918	+	intron	*EPYC*
5	96363465	96363467	+	96363467	4	10	XLOC_067328	+	predicted	---
5	96364965	96365009	+	96365000	24	80	XLOC_067328	+	predicted	---
5	96481918	96481918	+	96481918	3	3	---	---	intergenic	---
5	96791512	96791512	-	96791512	5	5	---	---	intergenic	---
5	97418674	97418674	+	97418674	4	4	XLOC_067331	+	predicted	---
5	97418713	97418713	+	97418713	3	3	XLOC_067331	+	predicted	---
5	97568079	97568079	-	97568079	3	3	---	---	intergenic	---
5	97568105	97568105	+	97568105	3	3	---	---	intergenic	---
5	97778043	97778043	-	97778043	4	4	ENSSSCG00000000921	-	3'UTR	---
5	97886168	97886168	-	97886168	3	3	ENSSSCG00000000920	-	intron	---
5	98118428	98118428	+	98118428	8	8	XLOC_067334	+	predicted	---
5	98243443	98243447	+	98243443	5	9	---	---	intergenic	---
5	99126190	99126190	+	99126190	4	4	ENSSSCG00000000926	+	ORF	*CEP290*
5	99159960	99159960	-	99159960	9	9	ENSSSCG00000000925	-	3'UTR	*C12orf29*
5	99161954	99161957	-	99161954	8	20	ENSSSCG00000000925	-	3'UTR	*C12orf29*
5	99162085	99162085	-	99162085	4	4	ENSSSCG00000000925	-	ORF	*C12orf29*
5	99203757	99203757	+	99203757	3	3	ENSSSCG00000000924	+	intron	*C12orf50*
5	99207119	99207119	+	99207119	8	8	ENSSSCG00000000924	+	3'UTR	*C12orf50*
5	99207202	99207202	+	99207202	29	29	XLOC_067337	+	predicted	---
5	99345886	99345887	-	99345886	3	6	---	---	intergenic	---
5	99472600	99472600	-	99472600	8	8	---	---	intergenic	---
5	99568349	99568349	-	99568349	3	3	---	---	intergenic	---
5	99910080	99910080	-	99910080	3	3	---	---	intergenic	---

5	100352150	100352155	-	100352151	7	15	XLOC_068303	-	predicted	---
5	100368446	100368465	-	100368459	13	56	XLOC_068303	-	predicted	---
5	101554037	101554037	-	101554037	7	7	---	---	intergenic	---
5	101870521	101870521	+	101870521	4	4	ENSSSCG00000000934	+	intron	*LRRIQ1*
5	101873401	101873401	+	101873401	3	3	ENSSSCG00000000934	+	ORF	*LRRIQ1*
5	102136288	102136288	+	102136288	4	4	XLOC_067352	+	predicted	---
5	102307838	102307838	+	102307838	3	3	---	---	intergenic	---
5	103050678	103050679	-	103050678	3	6	---	---	intergenic	---
5	103257836	103257836	+	103257836	3	3	---	---	intergenic	---
5	103357484	103357484	+	103357484	3	3	---	---	intergenic	---
5	103441474	103441474	-	103441474	3	3	---	---	intergenic	---
5	103908126	103908126	-	103908126	7	7	ENSSSCG00000024065	-	intron	---
5	104096082	104096082	+	104096082	4	4	---	---	intergenic	---
5	104173563	104173570	-	104173569	17	38	ENSSSCG00000000942	-	3'UTR	*CCDC59*
5	104173763	104173763	-	104173763	5	5	ENSSSCG00000000942	-	ORF	*CCDC59*
5	104176085	104176085	-	104176085	3	3	ENSSSCG00000000942	-	intron	*CCDC59*
5	104208814	104208815	+	104208814	8	13	ENSSSCG00000000941	+	intron	*METTL25*
5	104293893	104293893	-	104293893	3	3	---	---	intergenic	---
5	105095888	105095888	+	105095888	5	5	ENSSSCG00000000940	+	intron	---
5	105196296	105196296	-	105196296	4	4	XLOC_068318	-	predicted	---
5	105441689	105441689	+	105441689	3	3	---	---	intergenic	---
5	105629106	105629116	+	105629114	66	138	XLOC_067367	+	predicted	---
5	105629309	105629310	+	105629309	3	6	XLOC_067367	+	predicted	---
5	105634402	105634402	+	105634402	3	3	XLOC_067367	+	predicted	---
5	105706281	105706281	+	105706281	7	7	---	---	intergenic	---
5	106177137	106177137	-	106177137	12	12	---	---	intergenic	---

5	106209174	106209174	-	106209174	3	3	---	---	intergenic	---
5	106660172	106660175	+	106660172	3	6	---	---	intergenic	---
5	106735988	106735995	-	106735988	8	18	---	---	intergenic	---
5	106736444	106736444	-	106736444	3	3	---	---	intergenic	---
5	106736626	106736626	-	106736626	3	3	---	---	intergenic	---
5	106753710	106753711	+	106753711	16	24	---	---	intergenic	---
5	106866157	106866157	-	106866157	6	6	ENSSSCG00000000948	-	intron	*SYT1*
5	107029169	107029169	-	107029169	3	3	---	---	intergenic	---
5	107061628	107061628	+	107061628	7	7	---	---	intergenic	---
5	107228205	107228207	-	107228205	85	89	XLOC_068332	-	predicted	---
5	107320255	107320255	-	107320255	3	3	---	---	intergenic	---
5	108182128	108182128	+	108182128	3	3	ENSSSCG00000029249	+	intron	---
5	108582554	108582554	-	108582554	3	3	---	---	intergenic	---
5	108813828	108813828	+	108813828	3	3	XLOC_067385	+	predicted	---
5	109267960	109267960	+	109267960	47	47	ENSSSCG00000000950	+	intron	*E2F7*
5	109400933	109400933	-	109400933	3	3	---	---	intergenic	---
5	109475936	109475944	+	109475943	21	53	ENSSSCG00000000951	+	3'UTR	*CSRP2*
5	109585128	109585128	+	109585128	6	6	XLOC_067390	+	predicted	---
5	109585791	109585791	+	109585791	3	3	XLOC_067390	+	predicted	---
5	109586035	109586042	+	109586035	3	9	XLOC_067390	+	predicted	---
5	109787664	109787664	-	109787664	5	5	---	---	intergenic	---
5	110024268	110024268	-	110024268	6	6	---	---	intergenic	---
5	110092962	110092962	-	110092962	5	5	XLOC_068340	-	predicted	---
5	110348770	110348770	+	110348770	3	3	ENSSSCG00000000959	+	3'UTR	---
5	110349653	110349654	+	110349654	18	22	ENSSSCG00000000959	+	3'UTR	---
5	110445176	110445176	+	110445176	7	7	ENSSSCG00000000970	+	intron	*PPP6R2*

5	110479521	110479521	+	110479521	4	4	ENSSSCG00000000970	+	ORF	*PPP6R2*
5	110496850	110496856	+	110496850	5	9	ENSSSCG00000000970	+	3'UTR	*PPP6R2*
5	110497379	110497379	+	110497379	5	5	ENSSSCG00000000970	+	3'UTR	*PPP6R2*
5	110532355	110532364	+	110532356	56	113	XLOC_067404	+	predicted	---
5	110542034	110542038	-	110542034	25	48	ENSSSCG00000000964	-	3'UTR	*LMF2*
5	110557497	110557499	+	110557497	25	50	ENSSSCG00000000965	+	3'UTR	*NCAPH2*
5	110557638	110557641	-	110557640	45	84	ENSSSCG00000000966	-	3'UTR	*SCO2*
5	110562325	110562328	-	110562328	8	11	ENSSSCG00000000967	-	3'UTR	*ODF3B*
5	110579825	110579833	-	110579830	70	152	XLOC_068354	-	predicted	---
5	110660796	110660798	+	110660798	6	14	XLOC_067407	+	predicted	---
5	110668159	110668160	+	110668160	10	14	ENSSSCG00000000971	+	3'UTR	---
5	110690315	110690316	+	110690316	7	12	XLOC_067407	+	predicted	---
5	110690390	110690392	-	110690390	20	34	ENSSSCG00000000973	-	3'UTR	*SELO*
5	110704453	110704453	+	110704453	3	3	---	---	intergenic	---
5	110708334	110708334	-	110708334	4	4	ENSSSCG00000000974	-	3'UTR	*TRABD*
5	110709281	110709281	-	110709281	3	3	ENSSSCG00000000974	-	3'UTR	*TRABD*
5	110720302	110720302	-	110720302	3	3	ENSSSCG00000000975	-	3'UTR	*PANX2*
5	110732805	110732805	-	110732805	6	6	ENSSSCG00000000976	-	intron	*MOV10L1*
5	110788014	110788024	-	110788024	4	7	ENSSSCG00000000976	-	intron	*MOV10L1*
5	110947037	110947039	+	110947037	4	8	---	---	intergenic	---
5	110955693	110955697	-	110955693	3	12	ENSSSCG00000000980	-	3'UTR	*ALG12*
5	110973490	110973492	+	110973492	11	17	ENSSSCG00000000981	+	3'UTR	*CRELD2*
5	111128199	111128199	-	111128199	7	7	---	---	intergenic	---
6	102150	102150	-	102150	4	4	ENSSSCG00000002637	-	intron	*DBNDD1*
6	125967	125967	-	125967	4	4	ENSSSCG00000002637	-	intron	*DBNDD1*
6	181790	181806	+	181804	100	291	ENSSSCG00000029761	+	3'UTR	---

6	215309	215309	-	215309	4	4	ENSSSCG00000002637	-	3'UTR	*DBNDD1*
6	220784	220785	-	220785	5	9	ENSSSCG00000002637	-	intron	*DBNDD1*
6	226414	226414	+	226414	9	9	---	---	intergenic	---
6	251721	251730	+	251727	17	55	XLOC_071227	+	predicted	---
6	268732	268734	+	268733	6	12	ENSSSCG00000002639	+	3'UTR	*TUBB3*
6	425690	425690	+	425690	3	3	XLOC_071232	+	predicted	---
6	450587	450587	+	450587	9	9	ENSSSCG00000022310	+	3'UTR	*VPS9D1*
6	458803	458807	-	458806	8	28	ENSSSCG00000025401	-	3'UTR	*CDK10*
6	467846	467868	-	467862	45	117	XLOC_072858	-	predicted	---
6	485466	485469	+	485467	29	47	ENSSSCG00000023677	+	3'UTR	*CHMP1A*
6	517782	517817	-	517791	90	214	XLOC_072860	-	predicted	---
6	518041	518059	-	518049	137	296	ENSSSCG00000024974	-	3'UTR	*RPL13*
6	518097	518097	-	518097	3	3	ENSSSCG00000024974	-	ORF	*RPL13*
6	866485	866522	-	866513	65	185	XLOC_072868	-	predicted	---
6	866648	866648	-	866648	5	5	ENSSSCG00000021356	-	ORF	---
6	866747	866747	-	866747	3	3	ENSSSCG00000025162	-	ORF	---
6	866804	866808	-	866816	77	162	XLOC_072868	-	predicted	---
6	866857	866883	-	866874	23	59	XLOC_072868	-	predicted	---
6	866907	866976	+	866956	6	19	---	---	intergenic	---
6	866928	866934	-	866933	16	28	XLOC_072868	-	predicted	---
6	867021	867066	-	867065	103	183	XLOC_072868	-	predicted	---
6	867084	867097	+	867084	7	14	---	---	intergenic	---
6	867124	867163	-	867160	50	63	XLOC_072868	-	predicted	---
6	867195	867294	-	867259	50	141	---	---	intergenic	---
6	867246	867273	+	867246	8	27	---	---	intergenic	---
6	867330	867370	+	867346	6	25	---	---	intergenic	---

6	867584	867686	-	867686	432	941	---	---	intergenic	---
6	867724	867763	-	867763	169	191	---	---	intergenic	---
6	867829	867837	-	867829	392	718	---	---	intergenic	---
6	867896	867927	+	867899	7	14	---	---	intergenic	---
6	867900	867918	-	867900	5	12	---	---	intergenic	---
6	867941	867998	+	867955	10	56	---	---	intergenic	---
6	868273	868273	-	868273	3	3	XLOC_072868	-	predicted	---
6	868326	868373	-	868373	739	1102	XLOC_072868	-	predicted	---
6	868446	868511	-	868510	132	262	XLOC_072868	-	predicted	---
6	868584	868624	-	868607	8	30	XLOC_072868	-	predicted	---
6	868694	868733	-	868694	81	127	XLOC_072868	-	predicted	---
6	868777	868845	-	868810	1019	2587	XLOC_072868	-	predicted	---
6	869288	869373	-	869301	841	1226	XLOC_072868	-	predicted	---
6	869447	869512	-	869504	234	592	XLOC_072868	-	predicted	---
6	869552	869625	-	869593	348	566	XLOC_072868	-	predicted	---
6	869700	869701	-	869701	181	290	XLOC_072868	-	predicted	---
6	869838	869855	-	869838	42	85	XLOC_072868	-	predicted	---
6	869892	869902	-	869892	5	8	XLOC_072868	-	predicted	---
6	869956	869957	-	869956	11	14	XLOC_072868	-	predicted	---
6	870664	870835	-	870759	108	873	XLOC_072868	-	predicted	---
6	870900	870964	-	870961	62	116	XLOC_072868	-	predicted	---
6	870987	871017	-	871010	12	15	XLOC_072868	-	predicted	---
6	872415	872433	-	872433	9	35	XLOC_072868	-	predicted	---
6	872536	872545	-	872542	1037	1862	XLOC_072868	-	predicted	---
6	872868	872869	-	872868	9	13	XLOC_072868	-	predicted	---
6	872930	872967	-	872931	24	72	XLOC_072868	-	predicted	---

6	874161	874161	-	874161	4	4	XLOC_072868	-	predicted	---
6	874209	874228	-	874222	1168	1425	XLOC_072868	-	predicted	---
6	874321	874423	-	874400	173	619	XLOC_072868	-	predicted	---
6	874548	874607	-	874557	723	825	XLOC_072868	-	predicted	---
6	874604	874641	+	874604	5	11	XLOC_071239	+	predicted	---
6	874632	874687	-	874664	135	411	XLOC_072868	-	predicted	---
6	874659	874730	+	874723	7	16	XLOC_071239	+	predicted	---
6	874749	874859	-	874859	5882	11705	XLOC_072868	-	predicted	---
6	874772	874806	+	874772	4	8	XLOC_071239	+	predicted	---
6	874878	874977	-	874960	354	774	XLOC_072868	-	predicted	---
6	874974	875056	+	875020	21	67	XLOC_071239	+	predicted	---
6	874995	875038	-	875032	1067	2688	XLOC_072868	-	predicted	---
6	875074	875095	+	875092	12	28	XLOC_071239	+	predicted	---
6	875108	875179	-	875179	1726	1881	XLOC_072868	-	predicted	---
6	875128	875197	+	875163	13	102	XLOC_071239	+	predicted	---
6	875205	875246	-	875232	16705	19745	XLOC_072868	-	predicted	---
6	875214	875237	+	875232	35	71	XLOC_071239	+	predicted	---
6	875271	875322	+	875308	15	69	XLOC_071239	+	predicted	---
6	875347	875467	-	875400	243	949	XLOC_072868	-	predicted	---
6	875370	875620	+	875595	12	271	XLOC_071239	+	predicted	---
6	875501	875626	-	875556	1106	1524	XLOC_072868	-	predicted	---
6	875632	875677	+	875670	25	164	XLOC_071239	+	predicted	---
6	875669	875677	-	875669	6	10	XLOC_072868	-	predicted	---
6	875692	875734	+	875733	29	186	XLOC_071239	+	predicted	---
6	875761	875800	+	875788	15	80	XLOC_071239	+	predicted	---
6	875812	875818	-	875812	54	95	XLOC_072868	-	predicted	---

6	922873	922881	-	922874	15	33	XLOC_072869	-	predicted	---
6	956685	956693	+	956690	30	59	XLOC_071242	+	predicted	---
6	1054028	1054028	-	1054028	5	5	XLOC_072872	-	predicted	---
6	1055726	1055737	-	1055730	189	468	ENSSSCG00000002646	-	3'UTR	*TRAPPC2L*
6	1560880	1560881	-	1560881	6	10	XLOC_072878	-	predicted	---
6	1668232	1668232	+	1668232	7	7	---	---	intergenic	---
6	1760927	1760927	+	1760927	5	5	XLOC_071250	+	predicted	---
6	1936757	1936757	-	1936757	6	6	XLOC_072882	-	predicted	---
6	2003339	2003339	+	2003339	7	7	XLOC_071253	+	predicted	---
6	2004632	2004632	+	2004632	6	6	XLOC_071253	+	predicted	---
6	2005530	2005536	+	2005530	5	8	XLOC_071253	+	predicted	---
6	2007315	2007331	-	2007317	6	30	XLOC_072883	-	predicted	---
6	2007810	2007817	-	2007816	6	14	ENSSSCG00000002655	-	3'UTR	*MAP1LC3B*
6	2008634	2008646	-	2008645	36	56	ENSSSCG00000002655	-	3'UTR	*MAP1LC3B*
6	2008843	2008853	-	2008849	54	145	ENSSSCG00000002655	-	3'UTR	*MAP1LC3B*
6	2081390	2081397	+	2081390	11	23	XLOC_071254	+	predicted	---
6	2101628	2101634	+	2101634	7	12	XLOC_071255	+	predicted	---
6	2723547	2723547	-	2723547	3	3	---	---	intergenic	---
6	2776463	2776463	+	2776463	3	3	ENSSSCG00000002660	+	intron	*MTHFSD*
6	2898659	2898671	-	2898670	8	23	XLOC_072894	-	predicted	---
6	2906753	2906755	+	2906754	9	19	---	---	intergenic	---
6	2997212	2997212	-	2997212	3	3	---	---	intergenic	---
6	3341262	3341262	-	3341262	39	39	---	---	intergenic	---
6	3357724	3357740	+	3357740	8	12	---	---	intergenic	---
6	3375560	3375562	+	3375560	18	28	---	---	intergenic	---
6	3440951	3440951	+	3440951	4	4	---	---	intergenic	---

6	3453560	3453565	-	3453562	869	1821	ENSSSCG00000029034	-	3'UTR	*COX4I1*
6	3520504	3520509	+	3520507	6	15	XLOC_071266	+	predicted	---
6	3552395	3552397	+	3552395	18	22	XLOC_071268	+	predicted	---
6	4255208	4255209	+	4255208	3	6	XLOC_071274	+	predicted	---
6	4473364	4473370	-	4473370	41	49	ENSSSCG00000002670	-	3'UTR	---
6	4495689	4495689	-	4495689	3	3	---	---	intergenic	---
6	4496624	4496624	+	4496624	6	6	---	---	intergenic	---
6	4507821	4507823	-	4507823	14	22	XLOC_072907	-	predicted	---
6	4662122	4662122	+	4662122	3	3	---	---	intergenic	---
6	4757933	4757949	-	4757939	64	126	ENSSSCG00000023837	-	3'UTR	*WFDC1*
6	5055373	5055373	-	5055373	3	3	ENSSSCG00000002675	-	ORF	*DNAAF1*
6	5061228	5061229	-	5061228	11	16	ENSSSCG00000002675	-	ORF	*DNAAF1*
6	5061443	5061443	-	5061443	4	4	---	---	intergenic	---
6	5082902	5082902	+	5082902	7	7	ENSSSCG00000002681	+	3'UTR	*HSDL1*
6	5100392	5100392	+	5100392	6	6	ENSSSCG00000002682	+	intron	*MBTPS1*
6	5140257	5140277	+	5140265	10	31	ENSSSCG00000002682	+	3'UTR	*MBTPS1*
6	6079076	6079083	+	6079076	3	6	---	---	intergenic	---
6	6313972	6313972	-	6313972	3	3	---	---	intergenic	---
6	7107391	7107391	+	7107391	21	21	---	---	intergenic	---
6	7148961	7148961	-	7148961	10	10	---	---	intergenic	---
6	7183439	7183439	-	7183439	4	4	ENSSSCG00000002689	-	3'UTR	*CMIP*
6	7379883	7379883	-	7379883	51	51	---	---	intergenic	---
6	7424812	7424812	-	7424812	6	6	---	---	intergenic	---
6	7456610	7456610	-	7456610	4	4	XLOC_072927	-	predicted	---
6	7620009	7620009	+	7620009	3	3	XLOC_071307	+	predicted	---
6	7640242	7640242	+	7640242	3	3	---	---	intergenic	---

6	7669960	7669998	-	7669980	120	243	---	---	intergenic	---
6	8048807	8048807	+	8048807	3	3	---	---	intergenic	---
6	8256364	8256381	+	8256370	9	46	XLOC_071309	+	predicted	---
6	8568594	8568595	-	8568595	10	16	XLOC_072934	-	predicted	---
6	8862173	8862173	+	8862173	4	4	---	---	intergenic	---
6	8987086	8987088	-	8987087	12	29	---	---	intergenic	---
6	9009379	9009382	-	9009380	8	15	---	---	intergenic	---
6	9156599	9156599	+	9156599	4	4	---	---	intergenic	---
6	9216457	9216457	-	9216457	4	4	---	---	intergenic	---
6	9722536	9722536	+	9722536	3	3	ENSSSCG00000027415	+	intron	*WWOX*
6	10476143	10476143	-	10476143	4	4	---	---	intergenic	---
6	10572935	10572935	-	10572935	5	5	XLOC_072944	-	predicted	---
6	10734178	10734179	+	10734178	9	14	XLOC_071325	+	predicted	---
6	10742527	10742527	+	10742527	4	4	---	---	intergenic	---
6	10747264	10747264	+	10747264	3	3	XLOC_071325	+	predicted	---
6	11691758	11691758	+	11691758	3	3	---	---	intergenic	---
6	11888295	11888307	-	11888302	16	37	ENSSSCG00000002703	-	3'UTR	*TERF2IP*
6	11889275	11889275	-	11889275	4	4	ENSSSCG00000002703	-	ORF	*TERF2IP*
6	11889354	11889355	-	11889355	4	7	ENSSSCG00000002703	-	ORF	*TERF2IP*
6	11889579	11889579	-	11889579	4	4	ENSSSCG00000002703	-	ORF	*TERF2IP*
6	11903223	11903223	+	11903223	7	7	ENSSSCG00000002704	+	ORF	*KARS*
6	11907439	11907445	+	11907439	4	7	ENSSSCG00000002704	+	intron	*KARS*
6	11911741	11911741	+	11911741	5	5	ENSSSCG00000002704	+	ORF	*KARS*
6	11914382	11914382	-	11914382	4	4	ENSSSCG00000002705	-	intron	---
6	11914644	11914648	+	11914645	248	396	ENSSSCG00000002704	+	3'UTR	*KARS*
6	11923216	11923216	+	11923216	12	12	ENSSSCG00000002706	+	intron	*ADAT1*

6	11944594	11944594	+	11944594	3	3	XLOC_071330	+	predicted	---
6	11962964	11962964	-	11962964	8	8	ENSSSCG00000002707	-	3'UTR	*GABARAPL2*
6	11963183	11963196	-	11963188	59	86	ENSSSCG00000002707	-	3'UTR	*GABARAPL2*
6	11963321	11963321	-	11963321	4	4	ENSSSCG00000002707	-	3'UTR	*GABARAPL2*
6	11963518	11963531	-	11963519	17	66	ENSSSCG00000002707	-	3'UTR	*GABARAPL2*
6	11993116	11993116	+	11993116	4	4	ENSSSCG00000002708	+	intron	---
6	12003845	12003846	+	12003846	6	10	ENSSSCG00000002708	+	3'UTR	---
6	12003898	12003898	+	12003898	4	4	ENSSSCG00000002708	+	3'UTR	---
6	12005170	12005170	+	12005170	17	17	ENSSSCG00000002708	+	3'UTR	---
6	12005453	12005455	+	12005454	14	28	ENSSSCG00000002708	+	3'UTR	---
6	12030809	12030809	+	12030809	18	18	XLOC_071333	+	predicted	---
6	12053795	12053800	+	12053795	6	11	ENSSSCG00000028464	+	3'UTR	---
6	12057019	12057022	+	12057022	8	16	ENSSSCG00000028464	+	3'UTR	---
6	12077109	12077109	+	12077109	3	3	XLOC_071334	+	predicted	---
6	12077183	12077187	+	12077183	6	9	XLOC_071334	+	predicted	---
6	12078922	12078925	+	12078925	15	26	XLOC_071334	+	predicted	---
6	12079028	12079034	+	12079034	17	29	XLOC_071334	+	predicted	---
6	12079674	12079674	+	12079674	3	3	XLOC_071334	+	predicted	---
6	12192620	12192620	+	12192620	5	5	XLOC_071334	+	predicted	---
6	12192694	12192725	+	12192725	13	79	XLOC_071334	+	predicted	---
6	12196905	12196907	+	12196905	7	17	XLOC_071334	+	predicted	---
6	12248295	12248295	-	12248295	6	6	XLOC_072961	-	predicted	---
6	12340297	12340297	+	12340297	6	6	---	---	intergenic	---
6	12883190	12883195	-	12883191	7	20	ENSSSCG00000002715	-	3'UTR	*RFWD3*
6	12885152	12885152	-	12885152	3	3	ENSSSCG00000002715	-	3'UTR	*RFWD3*
6	13045741	13045741	+	13045741	3	3	ENSSSCG00000030420	+	intron	*GLG1*

6	13056539	13056539	+	13056539	4	4	ENSSSCG00000030420	+	intron	*GLG1*
6	13131101	13131112	+	13131102	10	37	XLOC_071345	+	predicted	---
6	13239713	13239713	-	13239713	6	6	XLOC_072970	-	predicted	---
6	13239805	13239805	-	13239805	3	3	XLOC_072970	-	predicted	---
6	13240213	13240213	-	13240213	3	3	XLOC_072970	-	predicted	---
6	13241827	13241827	-	13241827	7	7	ENSSSCG00000002722	-	3'UTR	---
6	13293751	13293758	+	13293757	14	31	ENSSSCG00000002721	+	3'UTR	---
6	13360329	13360330	-	13360329	20	25	XLOC_072974	-	predicted	---
6	13363108	13363126	-	13363124	45	151	XLOC_072975	-	predicted	---
6	13398575	13398575	+	13398575	3	3	ENSSSCG00000002724	+	intron	---
6	13493973	13493981	-	13493973	19	66	XLOC_072978	-	predicted	---
6	13507386	13507386	-	13507386	4	4	ENSSSCG00000002726	-	3'UTR	*MTSS1L*
6	13692892	13692892	+	13692892	4	4	XLOC_071359	+	predicted	---
6	13876330	13876330	+	13876330	3	3	---	---	intergenic	---
6	13877175	13877175	+	13877175	3	3	---	---	intergenic	---
6	13881846	13881846	-	13881846	3	3	---	---	intergenic	---
6	14064562	14064575	-	14064571	91	285	ENSSSCG00000002736	-	3'UTR	*TAT*
6	14064867	14064904	-	14064881	12	36	ENSSSCG00000002736	-	3'UTR	*TAT*
6	14086062	14086063	+	14086062	3	6	---	---	intergenic	---
6	14123644	14123644	+	14123644	3	3	XLOC_071367	+	predicted	---
6	14296253	14296254	+	14296254	40	62	---	---	intergenic	---
6	14303780	14303780	+	14303780	6	6	---	---	intergenic	---
6	14338267	14338276	-	14338268	12	25	XLOC_072995	-	predicted	---
6	14341111	14341112	-	14341111	19	22	ENSSSCG00000002740	-	3'UTR	*AP1G1*
6	14393275	14393275	-	14393275	5	5	ENSSSCG00000002740	-	intron	*AP1G1*
6	14507672	14507672	+	14507672	5	5	---	---	intergenic	---

6	14549021	14549021	+	14549021	5	5	ENSSSCG00000002747	+	ORF	*PMFBP1*
6	14554901	14554906	+	14554901	3	6	ENSSSCG00000002747	+	intron	*PMFBP1*
6	14562945	14562945	+	14562945	3	3	ENSSSCG00000002747	+	intron	*PMFBP1*
6	14602051	14602051	+	14602051	3	3	ENSSSCG00000002747	+	ORF	*PMFBP1*
6	14602098	14602099	+	14602099	7	13	ENSSSCG00000002747	+	ORF	*PMFBP1*
6	14602151	14602151	+	14602151	3	3	ENSSSCG00000002747	+	intron	*PMFBP1*
6	14608742	14608742	+	14608742	5	5	ENSSSCG00000002747	+	intron	*PMFBP1*
6	14612502	14612515	+	14612507	35	75	XLOC_071378	+	predicted	---
6	14616069	14616088	-	14616069	7	10	XLOC_073001	-	predicted	---
6	14645352	14645372	+	14645361	4	11	---	---	intergenic	---
6	14646109	14646109	+	14646109	3	3	---	---	intergenic	---
6	14647375	14647387	-	14647375	3	6	XLOC_073002	-	predicted	---
6	14647380	14647381	+	14647380	4	7	ENSSSCG00000002749	+	intron	*HP*
6	14647430	14647433	-	14647433	6	9	XLOC_073002	-	predicted	---
6	14647449	14647512	+	14647476	5	22	ENSSSCG00000002749	+	3'UTR	*HP*
6	14647569	14647660	-	14647641	6	34	XLOC_073002	-	predicted	---
6	14647629	14647629	+	14647629	3	3	ENSSSCG00000002749	+	3'UTR	*HP*
6	14647669	14647688	-	14647679	4	13	XLOC_073002	-	predicted	---
6	14647729	14647753	+	14647730	23	30	---	---	intergenic	---
6	14647750	14647750	-	14647750	10	10	XLOC_073002	-	predicted	---
6	14647786	14647829	+	14647788	39	138	---	---	intergenic	---
6	14647850	14647863	-	14647863	4	7	XLOC_073002	-	predicted	---
6	14647875	14647912	+	14647905	4	10	---	---	intergenic	---
6	14647954	14647990	-	14647988	5	15	XLOC_073002	-	predicted	---
6	14648049	14648084	-	14648066	10	38	XLOC_073002	-	predicted	---
6	14648067	14648071	+	14648067	3	6	---	---	intergenic	---

6	14648093	14648117	-	14648103	6	24	XLOC_073002	-	predicted	---
6	14648111	14648148	+	14648125	91	270	ENSSSCG00000002749	+	3'UTR	*HP*
6	14648200	14648247	+	14648219	4297	22226	ENSSSCG00000002749	+	3'UTR	*HP*
6	14648947	14648958	+	14648947	6	13	XLOC_071380	+	predicted	---
6	14649036	14649063	+	14649056	17	29	XLOC_071380	+	predicted	---
6	14649146	14649147	+	14649146	10	13	XLOC_071380	+	predicted	---
6	14649201	14649201	+	14649201	4	4	XLOC_071380	+	predicted	---
6	14649784	14649784	+	14649784	5	5	XLOC_071380	+	predicted	---
6	14649867	14649868	-	14649868	6	9	ENSSSCG00000025218	-	3'UTR	---
6	14651353	14651353	-	14651353	7	7	ENSSSCG00000025218	-	3'UTR	---
6	14788830	14788831	-	14788831	5	8	---	---	intergenic	---
6	15031180	15031180	+	15031180	9	9	---	---	intergenic	---
6	15056165	15056165	-	15056165	3	3	XLOC_073003	-	predicted	---
6	16010657	16010658	+	16010657	30	45	---	---	intergenic	---
6	16181506	16181515	-	16181508	41	67	ENSSSCG00000002753	-	3'UTR	*NOB1*
6	16248802	16248810	+	16248803	44	78	ENSSSCG00000002754	+	3'UTR	*NQO1*
6	16248850	16248850	+	16248850	3	3	---	---	intergenic	---
6	16252168	16252168	-	16252168	6	6	XLOC_073007	-	predicted	---
6	16258217	16258217	-	16258217	3	3	XLOC_073007	-	predicted	---
6	16263514	16263514	-	16263514	3	3	ENSSSCG00000002755	-	ORF	---
6	16366382	16366383	-	16366383	24	28	ENSSSCG00000002755	-	intron	*NFAT5*
6	16466497	16466497	+	16466497	4	4	---	---	intergenic	---
6	16466519	16466525	-	16466521	15	30	XLOC_073009	-	predicted	---
6	16468972	16468972	-	16468972	3	3	XLOC_073009	-	predicted	---
6	16469804	16469811	-	16469811	10	18	ENSSSCG00000002756	-	3'UTR	*CYB5B*
6	16493512	16493513	-	16493512	5	10	ENSSSCG00000002756	-	intron	*CYB5B*

6	16569660	16569662	+	16569662	11	17	ENSSSCG00000002762	+	intron	*TERF2*
6	16578505	16578505	+	16578505	25	25	ENSSSCG00000002762	+	3'UTR	*TERF2*
6	16589621	16589621	-	16589621	3	3	ENSSSCG00000002760	-	3'UTR	*NIP7*
6	16599135	16599143	+	16599143	10	25	ENSSSCG00000002759	+	3'UTR	*COG8*
6	16604521	16604525	-	16604523	48	98	ENSSSCG00000025151	-	3'UTR	---
6	16604959	16604960	-	16604959	5	9	ENSSSCG00000025151	-	3'UTR	---
6	16620889	16620891	-	16620889	3	6	XLOC_073012	-	predicted	---
6	16628582	16628584	-	16628583	17	29	ENSSSCG00000028109	-	3'UTR	*SNTB2*
6	16732610	16732613	-	16732611	9	15	ENSSSCG00000028638	-	3'UTR	*CIRH1A*
6	16747962	16747962	-	16747962	3	3	ENSSSCG00000028638	-	ORF	*CIRH1A*
6	16847309	16847312	+	16847312	9	13	ENSSSCG00000023162	+	intron	*CDH3*
6	16858780	16858780	-	16858780	3	3	XLOC_073015	-	predicted	---
6	17204685	17204696	+	17204693	95	248	ENSSSCG00000002819	+	3'UTR	*ARL2BP*
6	17205964	17205964	+	17205964	3	3	ENSSSCG00000002819	+	3'UTR	*ARL2BP*
6	17294032	17294056	+	17294055	45	109	XLOC_071398	+	predicted	---
6	17316245	17316246	+	17316245	4	8	XLOC_071397	+	predicted	---
6	17331067	17331068	+	17331068	16	20	ENSSSCG00000029519	+	ORF	*CIAPIN1*
6	17335321	17335344	+	17335332	41	236	ENSSSCG00000029519	+	3'UTR	*CIAPIN1*
6	17369829	17369829	-	17369829	3	3	ENSSSCG00000024387	-	ORF	*Metazoa_SRP*
6	17381048	17381053	+	17381048	37	68	ENSSSCG00000025284	+	3'UTR	*COQ9*
6	17391408	17391434	+	17391425	32	125	ENSSSCG00000025454	+	3'UTR	*POLR2C*
6	17671444	17671456	+	17671444	3	6	XLOC_071404	+	predicted	---
6	17709281	17709281	+	17709281	48	48	---	---	intergenic	---
6	17719161	17719165	+	17719163	28	66	ENSSSCG00000002813	+	3'UTR	*KATNB1*
6	17773093	17773107	-	17773096	39	68	XLOC_073032	-	predicted	---
6	17797261	17797262	+	17797261	3	6	ENSSSCG00000002811	+	intron	*CNGB1*

6	17884314	17884314	+	17884314	17	17	---	---	intergenic	---
6	17935316	17935316	-	17935316	8	8	---	---	intergenic	---
6	17958848	17958848	+	17958848	4	4	ENSSSCG00000002806	+	3'UTR	*MMP15*
6	17978592	17978595	-	17978594	39	63	ENSSSCG00000002805	-	3'UTR	*CFAP20*
6	18000544	18000551	-	18000544	4	8	ENSSSCG00000002804	-	intron	*CSNK2A2*
6	18000839	18000840	-	18000840	10	13	ENSSSCG00000002804	-	intron	*CSNK2A2*
6	18012436	18012436	-	18012436	4	4	ENSSSCG00000002804	-	intron	*CSNK2A2*
6	18073964	18073964	-	18073964	5	5	ENSSSCG00000002804	-	intron	*CSNK2A2*
6	18126623	18126623	+	18126623	3	3	ENSSSCG00000002803	+	ORF	---
6	18143537	18143554	-	18143553	15	21	XLOC_073039	-	predicted	---
6	18144195	18144204	-	18144203	85	154	---	---	intergenic	---
6	18144275	18144275	-	18144275	4	4	XLOC_073039	-	predicted	---
6	18144993	18145013	+	18145010	14	27	ENSSSCG00000002803	+	3'UTR	---
6	18145150	18145168	-	18145158	15	38	XLOC_073039	-	predicted	---
6	18145433	18145433	-	18145433	6	6	ENSSSCG00000028587	-	ORF	*PRSS54*
6	18161522	18161522	+	18161522	4	4	---	---	intergenic	---
6	18179631	18179631	-	18179631	3	3	---	---	intergenic	---
6	18230103	18230104	+	18230103	3	6	ENSSSCG00000002802	+	3'UTR	*GINS3*
6	18473850	18473870	+	18473851	49	70	ENSSSCG00000002799	+	3'UTR	*CNOT1*
6	18581030	18581030	+	18581030	8	8	---	---	intergenic	---
6	18604864	18604865	+	18604864	7	12	---	---	intergenic	---
6	18635503	18635503	+	18635503	4	4	---	---	intergenic	---
6	18753000	18753000	+	18753000	3	3	---	---	intergenic	---
6	19430235	19430235	+	19430235	3	3	---	---	intergenic	---
6	19516081	19516081	-	19516081	3	3	---	---	intergenic	---
6	19691796	19691796	+	19691796	4	4	---	---	intergenic	---

6	20435086	20435086	+	20435086	5	5	---	---	intergenic	---
6	20764569	20764569	+	20764569	3	3	---	---	intergenic	---
6	21055014	21055014	-	21055014	3	3	XLOC_073050	-	predicted	---
6	21292490	21292491	-	21292490	4	7	---	---	intergenic	---
6	21494304	21494304	-	21494304	3	3	---	---	intergenic	---
6	21696466	21696466	+	21696466	3	3	---	---	intergenic	---
6	22011780	22011780	-	22011780	3	3	---	---	intergenic	---
6	22429237	22429237	-	22429237	7	7	---	---	intergenic	---
6	22454646	22454646	-	22454646	3	3	---	---	intergenic	---
6	22553510	22553510	-	22553510	4	4	---	---	intergenic	---
6	22659734	22659734	-	22659734	3	3	---	---	intergenic	---
6	23676446	23676446	+	23676446	3	3	---	---	intergenic	---
6	23710764	23710764	-	23710764	3	3	ENSSSCG00000002795	-	3'UTR	*CDH11*
6	24134051	24134051	-	24134051	3	3	---	---	intergenic	---
6	24502323	24502323	+	24502323	3	3	---	---	intergenic	---
6	24575561	24575561	+	24575561	3	3	---	---	intergenic	---
6	24721238	24721238	-	24721238	5	5	---	---	intergenic	---
6	24824636	24824636	+	24824636	3	3	---	---	intergenic	---
6	24876889	24876889	+	24876889	3	3	---	---	intergenic	---
6	24963518	24963518	+	24963518	9	9	---	---	intergenic	---
6	25078845	25078855	-	25078853	24	59	ENSSSCG00000022457	-	3'UTR	*NOL3*
6	25083307	25083309	-	25083307	16	30	ENSSSCG00000002792	-	3'UTR	*HSF4*
6	25088910	25088910	-	25088910	3	3	XLOC_073059	-	predicted	---
6	25097400	25097400	+	25097400	6	6	XLOC_071436	+	predicted	---
6	25107150	25107156	+	25107154	49	136	ENSSSCG00000002790	+	3'UTR	*TMEM208*
6	25107428	25107433	-	25107428	6	9	ENSSSCG00000026116	-	3'UTR	*FHOD1*

6	25116716	25116716	-	25116716	5	5	XLOC_073062	-	predicted	---
6	25130792	25130793	+	25130793	7	11	XLOC_071437	+	predicted	---
6	25135706	25135707	+	25135706	3	6	ENSSSCG00000002786	+	3'UTR	*ELMO3*
6	25137900	25137900	-	25137900	4	4	XLOC_073063	-	predicted	---
6	25200075	25200084	-	25200077	36	72	XLOC_073066	-	predicted	---
6	25213769	25213770	-	25213769	5	8	ENSSSCG00000002781	-	ORF	*KCTD19*
6	25231408	25231408	+	25231408	10	10	---	---	intergenic	---
6	25299372	25299372	+	25299372	3	3	ENSSSCG00000002779	+	intron	*LRRC36*
6	25304975	25304975	+	25304975	4	4	---	---	intergenic	---
6	25305249	25305250	+	25305249	8	12	XLOC_071441	+	predicted	---
6	25312517	25312518	-	25312518	14	18	ENSSSCG00000002780	-	3'UTR	*TPPP3*
6	25317030	25317035	-	25317032	4	11	ENSSSCG00000002778	-	3'UTR	*ZDHHC1*
6	25357266	25357274	-	25357272	76	169	ENSSSCG00000002776	-	3'UTR	*ATP6V0D1*
6	25461228	25461228	-	25461228	5	5	XLOC_073071	-	predicted	---
6	25462413	25462413	-	25462413	6	6	---	---	intergenic	---
6	25467990	25468004	-	25467990	6	22	ENSSSCG00000024253	-	3'UTR	*CTCF*
6	25630785	25630814	-	25630812	49	75	XLOC_073075	-	predicted	---
6	25643308	25643312	-	25643309	32	43	XLOC_073075	-	predicted	---
6	25644554	25644575	-	25644563	54	155	ENSSSCG00000030295	-	3'UTR	---
6	25665727	25665729	-	25665728	28	40	---	---	intergenic	---
6	25673793	25673793	-	25673793	6	6	---	---	intergenic	---
6	25682383	25682397	+	25682383	5	12	ENSSSCG00000002768	+	3'UTR	*CENPT*
6	25682768	25682775	-	25682773	28	72	XLOC_073077	-	predicted	---
6	25712844	25712854	+	25712853	7	35	ENSSSCG00000021803	+	intron	---
6	25780632	25780632	+	25780632	3	3	ENSSSCG00000021803	+	3'UTR	---
6	25780687	25780694	+	25780690	15	39	ENSSSCG00000021803	+	3'UTR	---

6	25901120	25901120	-	25901120	3	3	---	---	intergenic	---
6	25947950	25947950	-	25947950	3	3	---	---	intergenic	---
6	25957782	25957826	-	25957824	24	64	ENSSSCG00000022098	-	3'UTR	*DUS2*
6	26003945	26003955	+	26003952	19	55	XLOC_071452	+	predicted	---
6	26009681	26009681	+	26009681	4	4	XLOC_071453	+	predicted	---
6	26036654	26036657	+	26036657	14	39	XLOC_071454	+	predicted	---
6	26116926	26116932	+	26116927	21	70	XLOC_071456	+	predicted	---
6	26117007	26117010	+	26117008	5	12	---	---	intergenic	---
6	26369657	26369666	-	26369664	199	340	XLOC_073081	-	predicted	---
6	26369784	26369784	-	26369784	3	3	XLOC_073081	-	predicted	---
6	26374362	26374364	-	26374362	9	18	ENSSSCG00000024911	-	3'UTR	---
6	26380286	26380286	+	26380286	10	10	ENSSSCG00000024305	+	intron	*MT3*
6	26385342	26385356	+	26385349	1610	2250	ENSSSCG00000023684	+	3'UTR	*MT1A*
							ENSSSCG00000024305	+	intron	*MT3*
6	26411795	26411796	+	26411795	6	12	ENSSSCG00000024305	+	3'UTR	*MT3*
6	26421234	26421234	+	26421234	3	3	ENSSSCG00000030300	+	ORF	*MT2A*
6	26421586	26421610	+	26421586	9	27	ENSSSCG00000030300	+	3'UTR	*MT2A*
6	26465958	26465958	+	26465958	3	3	ENSSSCG00000025417	+	intron	*BBS2*
6	26473872	26473874	+	26473872	7	18	ENSSSCG00000025417	+	3'UTR	*BBS2*
6	26752244	26752284	+	26752284	12	20	ENSSSCG00000002822	+	3'UTR	*AMFR*
6	26752340	26752340	+	26752340	5	5	ENSSSCG00000002822	+	3'UTR	*AMFR*
6	26752483	26752483	+	26752483	5	5	ENSSSCG00000002822	+	3'UTR	*AMFR*
6	26753506	26753521	+	26753521	4	10	ENSSSCG00000002822	+	3'UTR	*AMFR*
6	26753637	26753640	+	26753637	27	50	ENSSSCG00000002822	+	3'UTR	*AMFR*
6	26756613	26756613	+	26756613	3	3	XLOC_071462	+	predicted	---
6	27174111	27174111	+	27174111	3	3	---	---	intergenic	---

6	27289400	27289400	-	27289400	5	5	ENSSSCG00000002826	-	intron	---
6	27304124	27304124	+	27304124	6	6	ENSSSCG00000002824	+	intron	---
6	27335532	27335532	+	27335532	4	4	ENSSSCG00000002825	+	ORF	*CES1*
6	27349692	27349700	-	27349696	17	37	ENSSSCG00000002826	-	intron	---
6	27350418	27350422	+	27350419	509	970	XLOC_071466	+	predicted	---
6	27399456	27399456	+	27399456	3	3	XLOC_071463	+	predicted	---
6	27466829	27466829	-	27466829	4	4	ENSSSCG00000002828	-	3'UTR	*LPCAT2*
6	27468126	27468126	-	27468126	7	7	ENSSSCG00000002828	-	3'UTR	*LPCAT2*
6	27526387	27526398	-	27526396	13	22	XLOC_073089	-	predicted	---
6	27637663	27637681	-	27637663	9	22	---	---	intergenic	---
6	27638719	27638719	+	27638719	5	5	---	---	intergenic	---
6	28009944	28009944	-	28009944	5	5	---	---	intergenic	---
6	28352625	28352625	+	28352625	4	4	XLOC_071473	+	predicted	---
6	28386258	28386258	-	28386258	4	4	---	---	intergenic	---
6	28728407	28728407	-	28728407	3	3	---	---	intergenic	---
6	28878030	28878030	+	28878030	8	8	---	---	intergenic	---
6	29197363	29197363	+	29197363	4	4	---	---	intergenic	---
6	29251624	29251628	+	29251628	7	16	---	---	intergenic	---
6	29432045	29432045	+	29432045	5	5	---	---	intergenic	---
6	29588448	29588448	-	29588448	3	3	---	---	intergenic	---
6	29907881	29907881	+	29907881	5	5	---	---	intergenic	---
6	30238471	30238471	+	30238471	5	5	ENSSSCG00000002836	+	3'UTR	*SALL1*
6	30416166	30416166	+	30416166	13	13	XLOC_071487	+	predicted	---
6	30416344	30416351	+	30416348	7	22	XLOC_071487	+	predicted	---
6	30421125	30421126	+	30421125	4	7	---	---	intergenic	---
6	30440374	30440381	+	30440377	45	176	---	---	intergenic	---

6	31005151	31005151	+	31005151	3	3	XLOC_071489	+	predicted	---
6	31099975	31099982	-	31099978	53	158	---	---	intergenic	---
6	31299216	31299216	-	31299216	3	3	---	---	intergenic	---
6	31703220	31703224	-	31703224	12	16	---	---	intergenic	---
6	31813638	31813638	-	31813638	5	5	XLOC_073109	-	predicted	---
6	31860724	31860737	-	31860730	51	125	ENSSSCG00000002843	-	3'UTR	---
6	31883853	31883853	+	31883853	3	3	ENSSSCG00000028289	+	ORF	*LONP2*
6	32141372	32141376	-	32141376	6	11	XLOC_073113	-	predicted	---
6	32455642	32455642	+	32455642	4	4	---	---	intergenic	---
6	32672634	32672634	+	32672634	4	4	XLOC_071496	+	predicted	---
6	32673824	32673824	+	32673824	16	16	XLOC_071496	+	predicted	---
6	32807253	32807253	-	32807253	4	4	---	---	intergenic	---
6	32833504	32833504	+	32833504	7	7	---	---	intergenic	---
6	32852180	32852180	+	32852180	8	8	XLOC_071499	+	predicted	---
6	32908029	32908030	+	32908030	6	9	XLOC_071500	+	predicted	---
6	32909944	32909955	+	32909946	40	73	XLOC_071500	+	predicted	---
6	32929750	32929763	-	32929751	10	25	XLOC_073124	-	predicted	---
6	32930173	32930173	-	32930173	6	6	XLOC_073124	-	predicted	---
6	32930451	32930452	-	32930451	15	20	ENSSSCG00000002846	-	3'UTR	*DNAJA2*
6	33079308	33079308	+	33079308	6	6	XLOC_071503	+	predicted	---
6	33099714	33099714	-	33099714	14	14	ENSSSCG00000022333	-	3'UTR	*C16orf87*
6	33100611	33100611	-	33100611	7	7	ENSSSCG00000022333	-	ORF	*C16orf87*
6	33106998	33106998	-	33106998	9	9	ENSSSCG00000022333	-	ORF	*C16orf87*
6	33284553	33284553	-	33284553	3	3	ENSSSCG00000028141	-	3'UTR	---
6	33285333	33285335	-	33285334	18	30	ENSSSCG00000028141	-	3'UTR	---
6	33314854	33314854	+	33314854	12	12	---	---	intergenic	---

6	33642800	33642800	+	33642800	3	3	---	---	intergenic	---
6	34521770	34521776	-	34521774	6	16	ENSSSCG00000027085	-	3'UTR	*UQCRFS1*
6	34523646	34523646	-	34523646	8	8	ENSSSCG00000027085	-	3'UTR	*UQCRFS1*
6	34525749	34525756	-	34525756	30	57	ENSSSCG00000027085	-	3'UTR	*UQCRFS1*
6	34525810	34525829	-	34525813	48	138	ENSSSCG00000027085	-	3'UTR	*UQCRFS1*
6	34580788	34580788	+	34580788	5	5	---	---	intergenic	---
6	35037517	35037526	+	35037524	15	26	ENSSSCG00000002854	+	3'UTR	*POP4*
6	35125329	35125350	+	35125330	29	62	---	---	intergenic	---
6	35157112	35157113	+	35157113	13	19	XLOC_071516	+	predicted	---
6	35255296	35255296	+	35255296	5	5	XLOC_071518	+	predicted	---
6	35431622	35431622	+	35431622	4	4	ENSSSCG00000025912	+	intron	*URI1*
6	35476419	35476420	+	35476420	9	12	ENSSSCG00000025912	+	3'UTR	*URI1*
6	35871262	35871262	+	35871262	4	4	---	---	intergenic	---
6	35957921	35957922	-	35957921	5	9	XLOC_073136	-	predicted	---
6	36093626	36093626	+	36093626	4	4	---	---	intergenic	---
6	36342817	36342817	+	36342817	5	5	---	---	intergenic	---
6	36367983	36367984	+	36367984	26	35	---	---	intergenic	---
6	36427368	36427368	+	36427368	3	3	---	---	intergenic	---
6	36566772	36566772	-	36566772	4	4	---	---	intergenic	---
6	36827974	36827974	-	36827974	3	3	---	---	intergenic	---
6	37084936	37084937	+	37084936	64	80	---	---	intergenic	---
6	37252728	37252736	-	37252728	30	37	---	---	intergenic	---
6	37252814	37252817	-	37252814	4	8	---	---	intergenic	---
6	37252862	37252870	+	37252865	7	15	---	---	intergenic	---
6	37676391	37676391	+	37676391	3	3	---	---	intergenic	---
6	37769359	37769359	+	37769359	4	4	XLOC_071525	+	predicted	---

6	37924101	37924101	+	37924101	5	5	ENSSSCG00000022521	+	3'UTR	*DPY19L3*
6	38191729	38191729	+	38191729	5	5	ENSSSCG00000002861	+	3'UTR	*NUDT19*
6	38242356	38242357	-	38242357	4	7	XLOC_073150	-	predicted	---
6	38277633	38277633	-	38277633	3	3	ENSSSCG00000025152	-	intron	*CEP89*
							ENSSSCG00000025152	-	ORF	*CEP89*
6	38326593	38326593	+	38326593	3	3	ENSSSCG00000023878	+	3'UTR	*FAAP24*
6	38328043	38328044	-	38328043	5	8	ENSSSCG00000022312	-	3'UTR	*RHPN2*
6	38545479	38545482	+	38545481	21	34	XLOC_071537	+	predicted	---
6	38632196	38632196	-	38632196	10	10	XLOC_073153	-	predicted	---
6	38659815	38659819	-	38659819	9	18	XLOC_073155	-	predicted	---
6	38711420	38711429	+	38711421	56	85	XLOC_071541	+	predicted	---
6	38719004	38719004	+	38719004	17	17	ENSSSCG00000002867	+	ORF	---
6	38719594	38719594	+	38719594	6	6	ENSSSCG00000002867	+	3'UTR	*CEBPG*
							ENSSSCG00000002867	+	ORF	---
6	38722002	38722002	+	38722002	4	4	XLOC_071542	+	predicted	---
6	38724257	38724262	-	38724259	34	71	ENSSSCG00000002868	-	3'UTR	---
6	38973628	38973628	+	38973628	3	3	---	---	intergenic	---
6	39150491	39150491	-	39150491	4	4	---	---	intergenic	---
6	39208102	39208102	-	39208102	5	5	---	---	intergenic	---
6	39276670	39276670	-	39276670	3	3	---	---	intergenic	---
6	39489060	39489061	-	39489061	11	14	XLOC_073161	-	predicted	---
6	39516819	39516830	-	39516820	64	181	XLOC_073162	-	predicted	---
6	39517069	39517070	-	39517069	6	12	XLOC_073162	-	predicted	---
6	39627944	39627944	+	39627944	4	4	---	---	intergenic	---
6	39747409	39747409	-	39747409	3	3	---	---	intergenic	---
6	39775307	39775310	-	39775310	5	8	XLOC_073166	-	predicted	---

6	39775388	39775390	-	39775390	4	7	XLOC_073166	-	predicted	---
6	39775763	39775763	-	39775763	5	5	XLOC_073166	-	predicted	---
6	39785647	39785647	+	39785647	3	3	ENSSSCG00000002877	+	3'UTR	*ZNF181*
6	39871756	39871756	-	39871756	7	7	XLOC_073169	-	predicted	---
6	39885662	39885662	+	39885662	4	4	---	---	intergenic	---
6	39933315	39933324	-	39933316	5	14	XLOC_073170	-	predicted	---
6	39933726	39933726	-	39933726	5	5	XLOC_073170	-	predicted	---
6	40007995	40007995	+	40007995	3	3	ENSSSCG00000002879	+	intron	*GRAMD1A*
6	40019486	40019493	+	40019491	16	24	ENSSSCG00000002879	+	3'UTR	*GRAMD1A*
6	40032359	40032360	+	40032359	4	7	XLOC_071556	+	predicted	---
6	40071668	40071698	+	40071692	218	526	XLOC_071557	+	predicted	---
6	40101139	40101150	+	40101143	83	156	ENSSSCG00000021867	+	3'UTR	*HPN*
6	40110224	40110224	+	40110224	3	3	ENSSSCG00000025126	+	3'UTR	*LGI4*
6	40145606	40145610	+	40145606	22	49	ENSSSCG00000025499	+	3'UTR	*FXYD5*
6	40166043	40166086	-	40166063	89	521	XLOC_073176	-	predicted	---
6	40203575	40203590	+	40203588	25	156	ENSSSCG00000002885	+	3'UTR	*USF2*
6	40207302	40207302	+	40207302	9	9	ENSSSCG00000002886	+	3'UTR	*HAMP*
							ENSSSCG00000002886	+	ORF	*HAMP*
6	40224865	40224865	+	40224865	3	3	XLOC_071564	+	predicted	---
6	40281286	40281286	-	40281286	8	8	XLOC_073178	-	predicted	---
6	40344665	40344666	-	40344666	4	7	ENSSSCG00000002893	-	3'UTR	*CD22*
6	40461829	40461849	-	40461848	13	55	XLOC_073185	-	predicted	---
6	40461979	40461979	-	40461979	3	3	XLOC_073185	-	predicted	---
6	40475377	40475377	+	40475377	14	14	---	---	intergenic	---
6	40475440	40475459	+	40475459	5	12	---	---	intergenic	---
6	40475568	40475627	+	40475620	423	992	---	---	intergenic	---

6	40485776	40485781	+	40485781	9	16	---	---	intergenic	---
6	40493162	40493164	+	40493162	7	10	XLOC_071571	+	predicted	---
6	40500048	40500050	+	40500049	10	19	XLOC_071570	+	predicted	---
6	40506679	40506682	+	40506679	7	17	---	---	intergenic	---
6	40508181	40508181	-	40508181	3	3	---	---	intergenic	---
6	40526975	40526975	+	40526975	4	4	ENSSSCG00000002904	+	3'UTR	*HAUS5*
6	40540416	40540420	+	40540417	46	79	ENSSSCG00000002905	+	3'UTR	*RBM42*
6	40546419	40546419	+	40546419	8	8	XLOC_071573	+	predicted	---
6	40559033	40559046	+	40559044	127	255	ENSSSCG00000002907	+	3'UTR	---
6	40559158	40559160	+	40559158	54	74	ENSSSCG00000002907	+	3'UTR	---
6	40577942	40577942	+	40577942	7	7	---	---	intergenic	---
6	40586531	40586541	-	40586541	12	40	XLOC_073187	-	predicted	---
6	40589162	40589179	-	40589168	28	51	XLOC_073187	-	predicted	---
6	40601084	40601092	+	40601092	4	7	ENSSSCG00000002908	+	3'UTR	*ZBTB32*
6	40601310	40601347	+	40601346	57	201	XLOC_071575	+	predicted	---
6	40622304	40622306	+	40622305	12	25	ENSSSCG00000002895	+	3'UTR	*KMT2B*
6	40625563	40625568	-	40625565	16	35	ENSSSCG00000002896	-	3'UTR	*U2AF1*
6	40629556	40629565	+	40629557	178	328	XLOC_071577	+	predicted	---
6	40629614	40629616	+	40629614	32	41	XLOC_071577	+	predicted	---
6	40636834	40636842	+	40636834	52	91	ENSSSCG00000002898	+	3'UTR	---
6	40636892	40636893	-	40636893	21	34	ENSSSCG00000023498	-	3'UTR	---
6	40645955	40645965	-	40645957	18	41	XLOC_073191	-	predicted	---
6	40673971	40673990	-	40673985	28	107	ENSSSCG00000002913	-	3'UTR	*PRODH2*
6	40684835	40684835	-	40684835	3	3	ENSSSCG00000002913	-	intron	*PRODH2*
6	40700969	40700969	-	40700969	6	6	ENSSSCG00000002917	-	3'UTR	*NFKBID*
6	40711714	40711722	+	40711722	21	31	ENSSSCG00000002918	+	3'UTR	*HCST*

6	40711872	40711876	-	40711875	252	290	ENSSSCG00000002919	-	3'UTR	*TYROBP*
6	40779000	40779022	+	40779021	19	41	ENSSSCG00000024907	+	3'UTR	*SDHAF1*
							ENSSSCG00000024907	+	ORF	---
6	40787631	40787641	-	40787632	49	65	ENSSSCG00000002910	-	3'UTR	*ALKBH6*
6	40793340	40793340	-	40793340	3	3	ENSSSCG00000002921	-	3'UTR	*CLIPR-59*
6	40876250	40876261	+	40876254	18	42	XLOC_071587	+	predicted	---
6	40882983	40882993	-	40882985	74	218	ENSSSCG00000002925	-	3'UTR	*POLR2I*
6	40894815	40894825	+	40894823	45	101	ENSSSCG00000002926	+	3'UTR	*CKAP1*
6	40898029	40898029	+	40898029	6	6	---	---	intergenic	---
6	40920795	40920811	+	40920805	283	716	XLOC_071589	+	predicted	---
6	40921379	40921407	-	40921402	90	203	XLOC_073201	-	predicted	---
6	40961755	40961759	-	40961755	13	25	---	---	intergenic	---
6	40999146	40999148	-	40999148	4	10	XLOC_073203	-	predicted	---
6	40999375	40999382	-	40999379	7	13	XLOC_073203	-	predicted	---
6	41000637	41000637	-	41000637	3	3	ENSSSCG00000025355	-	3'UTR	*ZNF146*
6	41000716	41000716	-	41000716	3	3	ENSSSCG00000025355	-	3'UTR	*ZNF146*
6	41001711	41001711	-	41001711	3	3	ENSSSCG00000025355	-	ORF	*ZNF146*
6	41113848	41113848	-	41113848	5	5	XLOC_073206	-	predicted	---
6	41141604	41141604	-	41141604	3	3	---	---	intergenic	---
6	41156998	41156998	-	41156998	3	3	ENSSSCG00000002932	-	3'UTR	*ZNF567*
6	41324299	41324299	+	41324299	3	3	XLOC_071595	+	predicted	---
6	41503026	41503026	-	41503026	3	3	XLOC_073212	-	predicted	---
6	41551887	41551887	-	41551887	3	3	ENSSSCG00000002935	-	ORF	*ZNF568*
6	41552604	41552604	-	41552604	5	5	ENSSSCG00000002935	-	ORF	*ZNF568*
6	41589994	41589994	+	41589994	3	3	ENSSSCG00000002936	+	ORF	*ZNF829*
6	41620986	41620986	+	41620986	3	3	---	---	intergenic	---

6	41690497	41690497	-	41690497	3	3	---	---	intergenic	---
6	41785114	41785114	+	41785114	3	3	ENSSSCG00000021741	+	3'UTR	*ZNF527*
6	42027677	42027678	-	42027677	4	8	ENSSSCG00000029600	-	intron	*ZNF470*
6	42116863	42116870	-	42116865	48	70	XLOC_073220	-	predicted	---
6	42121382	42121382	-	42121382	4	4	---	---	intergenic	---
6	42121456	42121456	-	42121456	3	3	XLOC_073220	-	predicted	---
6	42406123	42406123	-	42406123	3	3	---	---	intergenic	---
6	42463274	42463274	-	42463274	3	3	---	---	intergenic	---
6	42492558	42492564	-	42492560	49	80	---	---	intergenic	---
6	42514465	42514471	+	42514465	3	6	---	---	intergenic	---
6	42669967	42669967	-	42669967	5	5	ENSSSCG00000023212	-	3'UTR	*CPI17*
6	42710519	42710551	+	42710539	73	127	XLOC_071621	+	predicted	---
6	42710823	42710823	+	42710823	3	3	XLOC_071621	+	predicted	---
6	42710933	42710944	+	42710942	13	65	XLOC_071621	+	predicted	---
6	42724614	42724620	+	42724617	21	84	XLOC_071622	+	predicted	---
6	42724736	42724742	-	42724737	10	17	ENSSSCG00000002952	-	3'UTR	*YIF1B*
6	42769718	42769718	+	42769718	8	8	ENSSSCG00000002955	+	intron	*CATSPERG*
6	42776545	42776545	+	42776545	3	3	ENSSSCG00000002955	+	intron	*CATSPERG*
6	42783272	42783280	+	42783273	9	25	XLOC_071624	+	predicted	---
6	42795993	42795993	+	42795993	10	10	ENSSSCG00000002956	+	ORF	*PSMD8*
6	42797167	42797183	+	42797181	59	192	ENSSSCG00000002956	+	3'UTR	*PSMD8*
6	42797494	42797495	+	42797494	18	21	ENSSSCG00000002956	+	3'UTR	*PSMD8*
6	42798008	42798016	-	42798009	7	31	XLOC_073235	-	predicted	---
6	42819929	42819930	-	42819930	8	11	XLOC_073237	-	predicted	---
6	42819952	42819952	+	42819952	3	3	XLOC_071627	+	predicted	---
6	42960107	42960107	+	42960107	24	24	XLOC_071627	+	predicted	---

6	42960191	42960194	-	42960192	4	10	XLOC_073239	-	predicted	---
6	43040547	43040553	+	43040550	151	342	ENSSSCG00000002963	+	3'UTR	*EIF3K*
6	43126150	43126154	+	43126154	44	57	XLOC_071629	+	predicted	---
6	43141698	43141699	-	43141698	3	6	---	---	intergenic	---
6	43180630	43180647	-	43180635	28	58	ENSSSCG00000028626	-	3'UTR	---
6	43218290	43218299	-	43218297	7	14	XLOC_073245	-	predicted	---
6	43241117	43241129	-	43241125	31	74	ENSSSCG00000026904	-	3'UTR	*NFKBIB*
6	43266350	43266350	-	43266350	3	3	XLOC_073248	-	predicted	---
6	43351287	43351287	+	43351287	3	3	---	---	intergenic	---
6	43413251	43413251	-	43413251	3	3	XLOC_073252	-	predicted	---
6	43728931	43728931	-	43728931	4	4	ENSSSCG00000002981	-	3'UTR	*DMBT1*
6	43825768	43825776	-	43825771	67	188	ENSSSCG00000026612	-	3'UTR	*TIMM50*
6	43834853	43834855	-	43834854	44	56	ENSSSCG00000023556	-	3'UTR	*SUPT5H*
6	43986161	43986180	-	43986162	32	89	ENSSSCG00000002986	-	3'UTR	*SELV*
6	44045010	44045017	-	44045010	7	10	XLOC_073268	-	predicted	---
6	44171967	44171971	+	44171968	39	70	ENSSSCG00000030510	+	3'UTR	*FBL*
6	44180904	44180904	+	44180904	8	8	ENSSSCG00000027197	+	3'UTR	*DYRK1B*
6	44317043	44317043	+	44317043	4	4	XLOC_071658	+	predicted	---
6	44331694	44331703	-	44331695	14	42	XLOC_073272	-	predicted	---
6	44333205	44333210	-	44333207	7	27	ENSSSCG00000002989	-	3'UTR	*AKT2*
6	44396799	44396799	-	44396799	3	3	XLOC_073274	-	predicted	---
6	44413578	44413584	-	44413581	11	21	XLOC_073275	-	predicted	---
6	44444900	44444900	+	44444900	3	3	---	---	intergenic	---
6	44447207	44447209	-	44447208	39	55	XLOC_073277	-	predicted	---
6	44457026	44457027	-	44457026	16	21	ENSSSCG00000025448	-	3'UTR	*SERTAD3*
6	44463497	44463530	-	44463511	137	360	XLOC_073278	-	predicted	---

6	44609173	44609182	+	44609181	29	56	XLOC_071668	+	predicted	---
6	44621637	44621637	-	44621637	7	7	ENSSSCG00000022438	-	intron	---
6	44646052	44646062	+	44646052	8	18	ENSSSCG00000003000	+	3'UTR	*ITPKC*
6	44667219	44667219	+	44667219	7	7	ENSSSCG00000002998	+	3'UTR	*SNRPA*
6	44684425	44684426	+	44684426	7	12	ENSSSCG00000002996	+	3'UTR	---
6	44694865	44694870	+	44694867	79	154	XLOC_071672	+	predicted	---
6	44720009	44720010	+	44720009	10	14	ENSSSCG00000026331	+	3'UTR	*ADCK4*
6	44738194	44738194	+	44738194	3	3	ENSSSCG00000023228	+	intron	*NUMBL*
6	44782655	44782658	+	44782655	9	12	XLOC_071676	+	predicted	---
6	44877474	44877478	+	44877477	40	96	XLOC_071678	+	predicted	---
6	44878366	44878366	+	44878366	3	3	XLOC_071678	+	predicted	---
6	44978565	44978565	+	44978565	4	4	ENSSSCG00000003006	+	3'UTR	*CYP2B22*
6	44978785	44978787	+	44978785	8	14	ENSSSCG00000003006	+	3'UTR	*CYP2B22*
6	44978869	44978871	+	44978869	6	10	XLOC_071679	+	predicted	---
6	44979817	44979817	-	44979817	3	3	XLOC_073285	-	predicted	---
6	44996688	44996688	+	44996688	10	10	---	---	intergenic	---
6	45072491	45072492	+	45072492	19	22	XLOC_071685	+	predicted	---
6	45073024	45073025	+	45073025	18	21	XLOC_071685	+	predicted	---
6	45110796	45110798	+	45110797	27	37	ENSSSCG00000030049	+	3'UTR	*HNRNPUL1*
6	45111025	45111025	+	45111025	6	6	ENSSSCG00000030049	+	3'UTR	*HNRNPUL1*
6	45111086	45111115	+	45111086	14	50	ENSSSCG00000030049	+	3'UTR	*HNRNPUL1*
6	45111201	45111201	+	45111201	4	4	XLOC_071685	+	predicted	---
6	45127030	45127030	-	45127030	16	16	ENSSSCG00000003017	-	3'UTR	*TGFB1*
6	45128038	45128038	-	45128038	6	6	ENSSSCG00000003017	-	3'UTR	*TGFB1*
6	45142563	45142568	-	45142568	27	43	XLOC_073289	-	predicted	---

6	45166382	45166383	-	45166382	21	29	ENSSSCG00000003015	-	3'UTR	*EXOSC5*
							ENSSSCG00000003013	-	intron	*B9D2*
6	45166555	45166555	-	45166555	4	4	ENSSSCG00000003015	-	3'UTR	*EXOSC5*
							ENSSSCG00000003013	-	intron	*B9D2*
6	45314172	45314176	-	45314176	7	16	---	---	intergenic	---
6	45399836	45399836	-	45399836	4	4	XLOC_073296	-	predicted	---
6	45426379	45426379	+	45426379	9	9	---	---	intergenic	---
6	45436030	45436030	+	45436030	4	4	XLOC_071694	+	predicted	---
6	45436206	45436206	+	45436206	4	4	XLOC_071694	+	predicted	---
6	45454285	45454290	+	45454285	9	17	ENSSSCG00000003018	+	3'UTR	*HSL*
6	45454619	45454619	+	45454619	8	8	ENSSSCG00000003018	+	3'UTR	*HSL*
6	45471902	45471902	-	45471902	3	3	ENSSSCG00000003021	-	3'UTR	*MEGF8*
6	45525227	45525227	-	45525227	8	8	XLOC_073300	-	predicted	---
6	45589110	45589110	+	45589110	3	3	---	---	intergenic	---
6	45618423	45618430	+	45618423	9	12	XLOC_071702	+	predicted	---
6	45620535	45620535	-	45620535	8	8	ENSSSCG00000028054	-	3'UTR	*ZNF526*
6	45621125	45621125	-	45621125	3	3	ENSSSCG00000028054	-	3'UTR	*ZNF526*
6	45646516	45646518	+	45646517	10	17	ENSSSCG00000030182	+	3'UTR	*DEDD2*
6	45657297	45657297	+	45657297	3	3	---	---	intergenic	---
6	45803149	45803149	-	45803149	3	3	ENSSSCG00000003038	-	intron	---
6	45839205	45839205	+	45839205	12	12	ENSSSCG00000003037	+	ORF	---
6	45954731	45954760	+	45954758	432	823	ENSSSCG00000003046	+	3'UTR	*RABAC1*
6	46049093	46049094	-	46049094	14	17	ENSSSCG00000003041	-	3'UTR	*CD79-ALPHA*
							ENSSSCG00000003048	-	intron	*CXCL17*

6	46056525	46056534	-	46056531	410	623	ENSSSCG00000003042	-	3'UTR	---
							ENSSSCG00000003041	-	intron	*CD79-ALPHA*
							ENSSSCG00000003048	-	intron	*CXCL17*
6	46058116	46058116	-	46058116	3	3	ENSSSCG00000003041	-	intron	*CD79-ALPHA*
							ENSSSCG00000003048	-	intron	*CXCL17*
							ENSSSCG00000003042	-	ORF	---
6	46065161	46065165	-	46065162	33	48	ENSSSCG00000003048	-	intron	*CXCL17*
6	46076892	46076902	+	46076899	79	274	XLOC_071713	+	predicted	---
6	46134070	46134082	+	46134072	60	233	XLOC_071717	+	predicted	---
6	46140691	46140691	+	46140691	4	4	XLOC_071718	+	predicted	---
6	46158743	46158750	-	46158743	3	6	ENSSSCG00000003055	-	3'UTR	*XRCC1*
6	46188730	46188731	-	46188731	4	7	ENSSSCG00000030637	-	3'UTR	*IRGQ*
6	46206037	46206039	-	46206039	6	11	XLOC_073318	-	predicted	---
6	46211903	46211913	+	46211903	4	16	XLOC_071720	+	predicted	---
6	46233017	46233017	+	46233017	7	7	XLOC_071721	+	predicted	---
6	46258712	46258712	+	46258712	3	3	ENSSSCG00000003063	+	3'UTR	*PHLDB3*
6	46349487	46349487	-	46349487	3	3	ENSSSCG00000003065	-	3'UTR	*PLAUR*
6	46384219	46384226	-	46384219	6	15	XLOC_073320	-	predicted	---
6	46397363	46397364	-	46397364	6	10	---	---	intergenic	---
6	46399612	46399612	-	46399612	16	16	---	---	intergenic	---
6	46403338	46403360	+	46403343	59	138	XLOC_071724	+	predicted	---
6	46406882	46406882	+	46406882	4	4	---	---	intergenic	---
6	46409265	46409275	+	46409273	29	68	XLOC_071724	+	predicted	---
6	46411315	46411316	-	46411315	5	8	ENSSSCG00000003068	-	3'UTR	*SMG9*
6	46417910	46417910	-	46417910	64	64	ENSSSCG00000003068	-	intron	*SMG9*
6	46490983	46490983	+	46490983	4	4	XLOC_071726	+	predicted	---

6	46516367	46516367	-	46516367	3	3	ENSSSCG00000003071	-	intron	*ZNF404*
6	46604018	46604018	+	46604018	3	3	---	---	intergenic	---
6	46623831	46623831	+	46623831	3	3	ENSSSCG00000003070	+	intron	*ZNF45*
6	46628466	46628466	+	46628466	3	3	XLOC_071727	+	predicted	---
6	46644279	46644279	+	46644279	3	3	---	---	intergenic	---
6	47153740	47153741	-	47153740	3	6	---	---	intergenic	---
6	47153762	47153762	+	47153762	6	6	---	---	intergenic	---
6	47164823	47164826	+	47164825	9	17	ENSSSCG00000027426	+	3'UTR	*BCL3*
6	47192011	47192018	+	47192017	14	34	XLOC_071737	+	predicted	---
6	47209651	47209651	+	47209651	3	3	ENSSSCG00000020657	+	3'UTR	*BCAM*
6	47210365	47210365	+	47210365	17	17	ENSSSCG00000020657	+	3'UTR	*BCAM*
6	47248406	47248406	+	47248406	12	12	ENSSSCG00000003090	+	3'UTR	*PRR2*
							ENSSSCG00000003090	+	intron	*PRR2*
6	47270974	47270988	+	47270988	12	20	ENSSSCG00000003088	+	intron	*APOE*
							ENSSSCG00000003090	+	intron	*PRR2*
6	47272189	47272199	+	47272195	3928	5391	ENSSSCG00000003088	+	3'UTR	*APOE*
							ENSSSCG00000003090	+	intron	*PRR2*
6	47285448	47285473	+	47285470	937	2057	ENSSSCG00000003087	+	3'UTR	*APOC4*
							ENSSSCG00000003090	+	intron	*PRR2*
6	47287487	47287487	+	47287487	4	4	ENSSSCG00000003090	+	intron	*PRR2*
							ENSSSCG00000003086	+	ORF	*APOC2*
6	47288017	47288063	+	47288053	434	1895	ENSSSCG00000003090	+	intron	*PRR2*
6	47288201	47288208	+	47288201	5	13	ENSSSCG00000003090	+	intron	*PRR2*
6	47323661	47323676	+	47323668	11	23	ENSSSCG00000003090	+	intron	*PRR2*
6	47335261	47335265	+	47335264	20	40	XLOC_071739	+	predicted	---
6	47402855	47402855	-	47402855	3	3	ENSSSCG00000003093	-	3'UTR	*SYMPK*

6	47452401	47452405	+	47452403	29	65	XLOC_071746	+	predicted	---
6	47460340	47460341	-	47460340	7	10	ENSSSCG00000003091	-	3'UTR	*IRF2BP1*
6	47566084	47566084	+	47566084	4	4	ENSSSCG00000003100	+	intron	*CCDC61*
6	47600675	47600675	-	47600675	4	4	---	---	intergenic	---
6	47600712	47600712	+	47600712	6	6	---	---	intergenic	---
6	47660515	47660515	+	47660515	6	6	ENSSSCG00000003098	+	3'UTR	*PPP5C*
6	47715268	47715269	+	47715269	4	7	ENSSSCG00000026220	+	intron	*CCDC8*
6	47742038	47742038	+	47742038	12	12	ENSSSCG00000026220	+	intron	*CCDC8*
6	47832647	47832699	+	47832691	1279	2096	ENSSSCG00000022545	+	3'UTR	*CALM1*
6	47834178	47834182	+	47834180	112	180	ENSSSCG00000022545	+	3'UTR	*CALM1*
6	47864373	47864374	-	47864373	5	8	ENSSSCG00000022280	-	intron	*DACT3*
6	47922600	47922603	-	47922603	21	31	XLOC_073361	-	predicted	---
6	47968133	47968137	-	47968133	25	28	XLOC_073362	-	predicted	---
6	48066409	48066421	-	48066415	22	98	ENSSSCG00000003106	-	3'UTR	*AP2S1*
6	48112926	48112927	-	48112927	11	15	---	---	intergenic	---
6	48134572	48134572	+	48134572	5	5	ENSSSCG00000003107	+	intron	*ARHGAP35*
6	48212494	48212494	+	48212494	3	3	ENSSSCG00000003107	+	3'UTR	*ARHGAP35*
6	48214799	48214809	+	48214808	8	19	ENSSSCG00000003107	+	3'UTR	*ARHGAP35*
6	48226856	48226863	-	48226863	5	8	---	---	intergenic	---
6	48248396	48248397	-	48248396	16	19	ENSSSCG00000003109	-	3'UTR	*TMEM160*
6	48268532	48268533	-	48268533	15	18	ENSSSCG00000022958	-	3'UTR	*ZC3H4*
6	48303568	48303577	+	48303568	49	85	---	---	intergenic	---
6	48401056	48401062	+	48401059	16	54	ENSSSCG00000003111	+	3'UTR	*SAE1*
6	48450825	48450825	+	48450825	3	3	---	---	intergenic	---
6	48454947	48454948	+	48454947	12	17	XLOC_071765	+	predicted	---
6	48537507	48537524	+	48537524	16	29	ENSSSCG00000029371	+	3'UTR	*C5AR1*

6	48685696	48685698	-	48685696	26	42	---	---	intergenic	---
6	48707775	48707775	+	48707775	5	5	XLOC_071768	+	predicted	---
6	48738194	48738200	+	48738194	52	78	ENSSSCG00000003119	+	3'UTR	*ZNF541*
6	48768917	48768922	+	48768921	19	54	XLOC_071769	+	predicted	---
6	48972125	48972126	+	48972125	9	14	ENSSSCG00000023371	+	3'UTR	*EHD2*
6	48982143	48982146	+	48982146	51	54	ENSSSCG00000022774	+	3'UTR	*GLTSCR2*
6	49004977	49004985	+	49004979	125	234	ENSSSCG00000030302	+	3'UTR	*SEPW1*
6	49088159	49088159	+	49088159	3	3	---	---	intergenic	---
6	49108182	49108192	-	49108182	4	10	XLOC_073377	-	predicted	---
6	49108561	49108579	-	49108568	667	1949	ENSSSCG00000003130	-	3'UTR	*SULT2A1*
6	49108659	49108659	-	49108659	4	4	ENSSSCG00000003130	-	3'UTR	*SULT2A1*
6	49108734	49108734	-	49108734	4	4	ENSSSCG00000003130	-	ORF	*SULT2A1*
6	49128882	49128885	-	49128884	35	59	XLOC_073378	-	predicted	---
6	49142521	49142521	-	49142521	4	4	ENSSSCG00000003123	-	intron	*BSPH1*
6	49204975	49204975	-	49204975	4	4	ENSSSCG00000003128	-	3'UTR	*LIG1*
6	49366399	49366400	-	49366399	4	8	ENSSSCG00000026689	-	3'UTR	*CCDC114*
6	49398346	49398349	+	49398348	40	59	ENSSSCG00000023377	+	3'UTR	*EMP3*
6	49539910	49539918	-	49539911	28	71	ENSSSCG00000003136	-	3'UTR	---
6	49562569	49562577	-	49562575	5	21	ENSSSCG00000003134	-	3'UTR	*GRWD1*
6	49623181	49623185	+	49623184	27	43	ENSSSCG00000003132	+	3'UTR	*KDELR1*
6	49623651	49623682	+	49623676	65	190	ENSSSCG00000003132	+	3'UTR	*KDELR1*
6	49625355	49625362	-	49625362	19	39	XLOC_073388	-	predicted	---
6	49716287	49716295	+	49716289	36	72	XLOC_071792	+	predicted	---
6	49716541	49716547	+	49716543	11	23	XLOC_071792	+	predicted	---
6	49721675	49721704	-	49721682	196	935	---	---	intergenic	---
6	49722284	49722284	-	49722284	3	3	ENSSSCG00000025928	-	ORF	---

6	49806095	49806102	-	49806095	4	8	XLOC_073399	-	predicted	---
6	49819985	49819988	-	49819986	18	32	XLOC_073399	-	predicted	---
6	49824782	49824783	-	49824782	5	10	ENSSSCG00000003141	-	3'UTR	*FUT1*
6	49831964	49831964	+	49831964	3	3	XLOC_071798	+	predicted	---
6	49855036	49855043	-	49855038	17	36	ENSSSCG00000003139	-	3'UTR	*BCAT2*
6	49856002	49856010	-	49856002	12	29	ENSSSCG00000003139	-	3'UTR	*BCAT2*
6	49872601	49872605	-	49872603	54	101	XLOC_073400	-	predicted	---
6	49891384	49891384	-	49891384	5	5	---	---	intergenic	---
6	49922831	49922831	+	49922831	4	4	ENSSSCG00000003155	+	3'UTR	*PPP1R15A*
6	49991062	49991064	-	49991064	5	9	XLOC_073403	-	predicted	---
6	50015310	50015310	-	50015310	8	8	ENSSSCG00000003157	-	3'UTR	*LIN7B*
6	50024183	50024192	-	50024191	23	47	XLOC_073404	-	predicted	---
6	50037548	50037548	-	50037548	4	4	ENSSSCG00000023792	-	intron	*SNRNP70*
6	50065071	50065071	+	50065071	87	87	XLOC_071805	+	predicted	---
6	50065154	50065163	-	50065155	36	122	ENSSSCG00000003152	-	3'UTR	*RUVBL2*
6	50094054	50094054	+	50094054	5	5	ENSSSCG00000003154	+	3'UTR	*GYS1*
6	50095613	50095613	+	50095613	3	3	ENSSSCG00000003154	+	3'UTR	*GYS1*
6	50095902	50095915	-	50095910	4576	6871	ENSSSCG00000003153	-	3'UTR	*FTL*
6	50095996	50096017	+	50095996	3	9	ENSSSCG00000003154	+	3'UTR	*GYS1*
6	50096063	50096072	+	50096063	3	12	ENSSSCG00000003154	+	3'UTR	*GYS1*
6	50096064	50096070	-	50096064	6	9	ENSSSCG00000003153	-	ORF	*FTL*
6	50096439	50096439	+	50096439	10	10	ENSSSCG00000003154	+	3'UTR	*GYS1*
6	50096878	50096878	-	50096878	3	3	ENSSSCG00000003153	-	ORF	*FTL*
6	50096919	50096924	-	50096919	9	12	ENSSSCG00000003153	-	ORF	*FTL*
6	50097303	50097314	+	50097303	4	7	ENSSSCG00000003154	+	3'UTR	*GYS1*
6	50097328	50097334	-	50097334	5	9	---	---	intergenic	---

6	50097465	50097482	+	50097474	66	281	ENSSSCG00000003154	+	3'UTR	*GYS1*
6	50100515	50100515	-	50100515	9	9	XLOC_073409	-	predicted	---
6	50111386	50111393	-	50111391	45	155	ENSSSCG00000003158	-	3'UTR	*DHDH*
6	50119226	50119232	-	50119230	148	272	ENSSSCG00000003150	-	3'UTR	*NUCB1*
6	50119862	50119866	-	50119863	17	39	ENSSSCG00000003150	-	3'UTR	*NUCB1*
6	50150763	50150763	+	50150763	3	3	ENSSSCG00000003149	+	ORF	*TULP2*
6	50226380	50226380	+	50226380	3	3	ENSSSCG00000003172	+	intron	*CCDC155*
6	50229929	50229931	+	50229931	7	10	XLOC_071808	+	predicted	---
6	50233848	50233848	-	50233848	3	3	---	---	intergenic	---
6	50253125	50253131	-	50253128	27	71	ENSSSCG00000003169	-	3'UTR	*PIH1D1*
6	50275499	50275499	+	50275499	11	11	XLOC_071811	+	predicted	---
6	50275800	50275804	+	50275800	5	8	XLOC_071811	+	predicted	---
6	50292776	50292776	+	50292776	15	15	XLOC_071811	+	predicted	---
6	50296366	50296372	+	50296372	35	56	ENSSSCG00000003166	+	ORF	*RPL13A*
6	50296753	50296753	+	50296753	4	4	ENSSSCG00000003166	+	intron	*RPL13A*
							ENSSSCG00000027829	+	ORF	*SNORD35*
6	50296984	50296984	+	50296984	9	9	ENSSSCG00000003166	+	ORF	*RPL13A*
6	50297285	50297315	+	50297311	407	671	ENSSSCG00000003166	+	3'UTR	*RPL13A*
6	50297698	50297723	+	50297721	173	318	ENSSSCG00000003166	+	intron	*RPL13A*
6	50308049	50308051	-	50308051	4	7	---	---	intergenic	---
6	50308738	50308767	+	50308757	1126	2642	ENSSSCG00000003165	+	3'UTR	*RPS11*
							ENSSSCG00000003166	+	intron	*RPL13A*
6	50308920	50308920	+	50308920	7	7	ENSSSCG00000003165	+	3'UTR	*RPS11*
							ENSSSCG00000003166	+	intron	*RPL13A*
6	50327992	50327994	+	50327993	51	74	ENSSSCG00000003187	+	3'UTR	*FCRN*
							ENSSSCG00000003166	+	intron	*RPL13A*

6	50328154	50328173	+	50328166	93	183	ENSSSCG00000003187	+	3'UTR	*FCRN*
							ENSSSCG00000003166	+	intron	*RPL13A*
6	50340269	50340269	+	50340269	13	13	ENSSSCG00000003166	+	intron	*RPL13A*
6	50348617	50348619	-	50348618	17	29	ENSSSCG00000029154	-	3'UTR	*NOSIP*
6	50363010	50363010	+	50363010	12	12	ENSSSCG00000003166	+	intron	*RPL13A*
6	50404159	50404159	+	50404159	3	3	XLOC_071811	+	predicted	---
6	50409621	50409622	-	50409621	14	20	ENSSSCG00000003183	-	3'UTR	*RRAS*
6	50429414	50429422	+	50429422	6	14	ENSSSCG00000003182	+	3'UTR	*SCAF1*
6	50430689	50430694	-	50430693	18	46	ENSSSCG00000003178	-	3'UTR	*IRF3*
6	50443167	50443171	+	50443167	9	19	ENSSSCG00000003177	+	3'UTR	*BCL2L12*
6	50459461	50459472	+	50459465	50	99	XLOC_071821	+	predicted	---
6	50461632	50461632	+	50461632	6	6	XLOC_071821	+	predicted	---
6	50476782	50476782	+	50476782	8	8	ENSSSCG00000024872	+	3'UTR	*CPT1C*
6	50491252	50491258	-	50491253	23	71	XLOC_073421	-	predicted	---
6	50553003	50553003	+	50553003	8	8	ENSSSCG00000003199	+	3'UTR	*AP2A1*
6	50578646	50578649	+	50578646	3	9	XLOC_071824	+	predicted	---
6	50602853	50602861	+	50602853	21	36	XLOC_071824	+	predicted	---
6	50603437	50603440	-	50603439	28	41	ENSSSCG00000003195	-	3'UTR	*PNKP*
6	50608292	50608292	-	50608292	3	3	ENSSSCG00000003195	-	intron	*PNKP*
6	50610713	50610716	-	50610715	5	14	ENSSSCG00000003194	-	3'UTR	*AKT1S1*
6	50635287	50635289	+	50635288	18	27	ENSSSCG00000003193	+	3'UTR	*TBC1D17*
6	50635543	50635551	-	50635550	110	130	ENSSSCG00000003192	-	3'UTR	*IL4I1*
6	50701723	50701725	-	50701724	18	33	XLOC_073425	-	predicted	---
6	50731004	50731004	+	50731004	4	4	ENSSSCG00000003201	+	3'UTR	*ATF5*
6	50731279	50731282	+	50731280	44	87	ENSSSCG00000003201	+	3'UTR	*ATF5*
6	50748430	50748430	+	50748430	3	3	ENSSSCG00000003202	+	intron	---

6	50754925	50754937	-	50754935	15	82	ENSSSCG00000003204	-	3'UTR	*VRK3*
6	50803314	50803314	+	50803314	3	3	XLOC_071828	+	predicted	---
6	50804306	50804308	+	50804308	10	17	XLOC_071828	+	predicted	---
6	50836777	50836788	-	50836780	59	101	XLOC_073429	-	predicted	---
6	50843317	50843317	-	50843317	3	3	ENSSSCG00000003207	-	intron	*IZUMO2*
6	50845823	50845827	-	50845827	24	27	ENSSSCG00000003207	-	intron	*IZUMO2*
6	50850025	50850029	-	50850028	5	15	ENSSSCG00000003207	-	intron	*IZUMO2*
6	50965613	50965613	+	50965613	10	10	ENSSSCG00000027407	+	intron	---
							ENSSSCG00000029377	+	intron	---
6	51094311	51094318	+	51094318	22	29	ENSSSCG00000003211	+	3'UTR	*NR1H2*
6	51120962	51120966	+	51120964	37	66	ENSSSCG00000003216	+	3'UTR	*POLD1*
6	51151646	51151646	+	51151646	3	3	---	---	intergenic	---
6	51159082	51159089	+	51159082	17	44	ENSSSCG00000003218	+	3'UTR	*MYBPC2*
6	51159516	51159531	-	51159530	220	335	ENSSSCG00000003219	-	3'UTR	*FAM71E1*
6	51171181	51171183	+	51171182	33	43	ENSSSCG00000024391	+	3'UTR	*EMC10*
6	51185526	51185529	-	51185528	15	23	ENSSSCG00000003217	-	3'UTR	*JOSD2*
6	51190316	51190318	-	51190317	21	39	XLOC_073436	-	predicted	---
6	51404644	51404644	+	51404644	3	3	ENSSSCG00000026561	+	3'UTR	*CLEC11A*
6	51404975	51404975	+	51404975	4	4	XLOC_071845	+	predicted	---
6	51432648	51432655	-	51432655	21	24	XLOC_073440	-	predicted	---
6	51433284	51433296	-	51433286	121	430	XLOC_073440	-	predicted	---
6	51440733	51440736	+	51440734	21	38	ENSSSCG00000024736	+	3'UTR	*ACPT*
6	51441880	51441882	-	51441881	7	16	XLOC_073441	-	predicted	---
6	51457475	51457497	-	51457497	32	40	XLOC_073443	-	predicted	---
6	51587975	51587988	+	51587983	13	40	ENSSSCG00000003226	+	3'UTR	*CD33*
							ENSSSCG00000003226	+	intron	*CD33*

6	51672758	51672763	-	51672760	402	769	ENSSSCG00000003229	-	3'UTR	*ETFB*
6	51674209	51674219	-	51674211	48	108	ENSSSCG00000003229	-	intron	*ETFB*
6	51689155	51689162	-	51689157	32	82	XLOC_073451	-	predicted	---
6	51692521	51692524	-	51692522	26	41	ENSSSCG00000003231	-	3'UTR	*NKG7*
6	51715383	51715389	-	51715384	5	14	ENSSSCG00000022906	-	3'UTR	*CH242-204P3.4*
6	51783600	51783604	-	51783602	162	255	XLOC_073457	-	predicted	---
6	51805510	51805510	-	51805510	7	7	ENSSSCG00000003236	-	3'UTR	*SIGLEC5*
6	51830939	51830941	-	51830939	11	14	XLOC_073458	-	predicted	---
6	51909405	51909405	-	51909405	3	3	ENSSSCG00000003240	-	intron	---
6	51958773	51958773	-	51958773	3	3	XLOC_073462	-	predicted	---
6	52023947	52023948	+	52023947	7	13	ENSSSCG00000003243	+	intron	---
6	52050025	52050025	+	52050025	5	5	ENSSSCG00000003243	+	intron	---
6	52051530	52051530	+	52051530	4	4	XLOC_071862	+	predicted	---
6	52052206	52052206	+	52052206	5	5	XLOC_071862	+	predicted	---
6	52053350	52053368	+	52053350	16	85	XLOC_071862	+	predicted	---
6	52131738	52131738	+	52131738	3	3	XLOC_071862	+	predicted	---
6	52188187	52188187	+	52188187	6	6	---	---	intergenic	---
6	52233180	52233190	-	52233188	71	93	ENSSSCG00000024413	-	3'UTR	*PPP2R1A*
6	52324225	52324257	-	52324257	6	17	ENSSSCG00000027056	-	intron	*ZNF41*
6	52346573	52346573	-	52346573	4	4	ENSSSCG00000027056	-	intron	*ZNF41*
6	52469155	52469155	-	52469155	17	17	XLOC_073469	-	predicted	---
6	52519653	52519653	-	52519653	5	5	---	---	intergenic	---
6	52530209	52530209	-	52530209	3	3	XLOC_073469	-	predicted	---
6	53184662	53184662	-	53184662	3	3	---	---	intergenic	---
6	53199439	53199448	+	53199439	94	141	ENSSSCG00000003261	+	3'UTR	---

6	53199501	53199503	-	53199501	4	11	ENSSSCG00000003262	-	3'UTR	*TFPT*
6	53223678	53223697	+	53223695	26	48	ENSSSCG00000003263	+	3'UTR	*PRPF31*
6	53243150	53243150	-	53243150	6	6	XLOC_073488	-	predicted	---
6	53243170	53243170	+	53243170	17	17	ENSSSCG00000003264	+	3'UTR	*CNOT3*
6	53246749	53246749	-	53246749	3	3	ENSSSCG00000003265	-	ORF	*LENG1*
6	53248536	53248536	-	53248536	11	11	ENSSSCG00000003266	-	3'UTR	*TMC4*
6	53263436	53263439	-	53263437	64	79	ENSSSCG00000003267	-	3'UTR	*MBOAT7*
6	53284992	53284993	+	53284992	25	48	ENSSSCG00000003268	+	intron	*TSEN34*
6	53288499	53288501	+	53288499	14	28	ENSSSCG00000003268	+	3'UTR	*TSEN34*
6	53298284	53298316	+	53298310	729	1504	XLOC_071907	+	predicted	---
6	53412023	53412025	-	53412025	17	32	ENSSSCG00000030857	-	3'UTR	*LENG8*
6	53412293	53412293	+	53412293	9	9	XLOC_071910	+	predicted	---
6	53427494	53427494	-	53427494	4	4	XLOC_073494	-	predicted	---
6	53550019	53550019	+	53550019	4	4	ENSSSCG00000003275	+	3'UTR	---
6	53559082	53559082	-	53559082	5	5	XLOC_073499	-	predicted	---
6	53586708	53586708	+	53586708	7	7	XLOC_071915	+	predicted	---
6	53766609	53766609	+	53766609	6	6	XLOC_071921	+	predicted	---
6	53773980	53773987	+	53773980	22	30	ENSSSCG00000003278	+	3'UTR	---
6	53909819	53909819	+	53909819	5	5	XLOC_071923	+	predicted	---
6	54294172	54294172	+	54294172	6	6	XLOC_071930	+	predicted	---
6	54298571	54298578	+	54298578	14	31	ENSSSCG00000024505	+	3'UTR	*TNNI3*
							ENSSSCG00000024505	+	ORF	---
6	54314539	54314540	+	54314540	28	32	XLOC_071931	+	predicted	---
6	54340456	54340456	+	54340456	11	11	ENSSSCG00000027189	+	3'UTR	*PPP1R12C*
6	54392753	54392753	+	54392753	8	8	ENSSSCG00000003288	+	3'UTR	*HSPBP1*
6	54392894	54392895	+	54392894	13	18	ENSSSCG00000003288	+	3'UTR	*HSPBP1*

6	54418928	54418935	+	54418931	14	32	XLOC_071935	+	predicted	---
6	54421701	54421710	+	54421704	41	76	ENSSSCG00000003302	+	3'UTR	*TMEM86B*
6	54486371	54486371	+	54486371	4	4	---	---	intergenic	---
6	54509360	54509360	-	54509360	25	25	XLOC_073513	-	predicted	---
6	54560691	54560691	-	54560691	6	6	---	---	intergenic	---
6	54951892	54951892	+	54951892	3	3	---	---	intergenic	---
6	54967647	54967647	+	54967647	5	5	---	---	intergenic	---
6	55523287	55523287	-	55523287	6	6	---	---	intergenic	---
6	55766754	55766754	-	55766754	3	3	ENSSSCG00000003320	-	intron	---
6	55950581	55950581	+	55950581	3	3	---	---	intergenic	---
6	55981877	55981877	-	55981877	6	6	XLOC_073543	-	predicted	---
6	56025848	56025856	-	56025854	29	68	---	---	intergenic	---
6	56025897	56025897	-	56025897	3	3	---	---	intergenic	---
6	56033591	56033591	-	56033591	3	3	---	---	intergenic	---
6	56149406	56149406	-	56149406	4	4	ENSSSCG00000026141	-	3'UTR	*ZNF71*
6	56426585	56426585	-	56426585	7	7	---	---	intergenic	---
6	56588280	56588280	-	56588280	4	4	XLOC_073550	-	predicted	---
6	56720047	56720052	+	56720047	5	9	XLOC_071973	+	predicted	---
6	56802991	56802991	-	56802991	4	4	XLOC_073553	-	predicted	---
6	56806854	56806860	-	56806854	7	13	XLOC_073553	-	predicted	---
6	56809996	56809996	-	56809996	3	3	XLOC_073553	-	predicted	---
6	56866879	56866888	-	56866881	21	47	ENSSSCG00000003986	-	intron	---
6	56867000	56867000	-	56867000	6	6	ENSSSCG00000003986	-	intron	---
6	56993293	56993293	+	56993293	3	3	XLOC_071974	+	predicted	---
6	57047421	57047421	-	57047421	4	4	---	---	intergenic	---
6	57187275	57187294	-	57187285	10	41	XLOC_073560	-	predicted	---

6	57260476	57260476	+	57260476	6	6	---	---	intergenic	---
6	57272005	57272005	-	57272005	12	12	XLOC_073562	-	predicted	---
6	57273430	57273432	-	57273432	9	16	ENSSSCG00000003994	-	intron	*C19orf18*
6	57308975	57308975	-	57308975	9	9	ENSSSCG00000003995	-	intron	*ZNF606*
6	57317996	57318015	-	57318015	4	10	XLOC_073563	-	predicted	---
6	57321946	57321946	-	57321946	4	4	---	---	intergenic	---
6	57396916	57396916	-	57396916	5	5	XLOC_073565	-	predicted	---
6	57502282	57502282	-	57502282	10	10	XLOC_073569	-	predicted	---
6	57653278	57653315	-	57653281	383	764	ENSSSCG00000004001	-	3'UTR	*A1BG*
6	57692178	57692215	+	57692198	981	3999	ENSSSCG00000004002	+	3'UTR	*RPS5*
6	57722700	57722700	+	57722700	4	4	XLOC_071996	+	predicted	---
6	57736505	57736505	-	57736505	8	8	XLOC_073575	-	predicted	---
6	57741040	57741045	-	57741045	4	10	ENSSSCG00000027987	-	intron	*ZNF132*
6	57837801	57837801	-	57837801	3	3	XLOC_073577	-	predicted	---
6	57851241	57851243	-	57851242	10	19	ENSSSCG00000022204	-	3'UTR	---
6	57980363	57980363	+	57980363	5	5	ENSSSCG00000003334	+	ORF	*UBE2J2*
6	57982126	57982128	+	57982126	37	52	ENSSSCG00000003334	+	3'UTR	*UBE2J2*
6	57996310	57996310	-	57996310	3	3	XLOC_073584	-	predicted	---
6	58002061	58002061	+	58002061	5	5	ENSSSCG00000003332	+	ORF	*SDF4*
6	58008407	58008414	+	58008412	179	303	ENSSSCG00000003332	+	3'UTR	*SDF4*
6	58047437	58047448	+	58047442	57	95	ENSSSCG00000027991	+	3'UTR	*SCNN1D*
6	58094882	58094901	-	58094883	15	37	ENSSSCG00000003339	-	3'UTR	*CPSF3L*
6	58094993	58094993	+	58094993	3	3	XLOC_072017	+	predicted	---
6	58110400	58110400	+	58110400	4	4	XLOC_072018	+	predicted	---
6	58115864	58115869	-	58115864	13	17	XLOC_073591	-	predicted	---
6	58127551	58127554	-	58127551	7	17	---	---	intergenic	---

6	58129312	58129312	-	58129312	15	15	ENSSSCG00000021880	-	3'UTR	*MXRA8*
6	58158515	58158541	-	58158539	19	102	ENSSSCG00000003348	-	3'UTR	*SSU72*
6	58158720	58158720	-	58158720	3	3	ENSSSCG00000003348	-	3'UTR	*SSU72*
6	58193846	58193846	-	58193846	3	3	---	---	intergenic	---
6	58211126	58211126	+	58211126	3	3	---	---	intergenic	---
6	58229983	58229983	+	58229983	10	10	ENSSSCG00000003350	+	3'UTR	*MIB2*
6	58233834	58233834	+	58233834	7	7	ENSSSCG00000003351	+	3'UTR	*MMP23B*
6	58234559	58234565	-	58234564	9	18	ENSSSCG00000003352	-	3'UTR	---
6	58250369	58250385	-	58250377	35	77	XLOC_073595	-	predicted	---
6	58274564	58274571	-	58274567	432	693	ENSSSCG00000003347	-	3'UTR	*MRPL20*
6	58274611	58274637	-	58274613	15	46	ENSSSCG00000003347	-	3'UTR	*MRPL20*
6	58304133	58304134	+	58304133	7	10	ENSSSCG00000003344	+	3'UTR	*VWA1*
6	58325277	58325279	+	58325277	21	33	XLOC_072030	+	predicted	---
6	58334580	58334585	+	58334582	20	31	XLOC_072031	+	predicted	---
6	58548672	58548672	+	58548672	9	9	ENSSSCG00000028530	+	intron	*TMEM52*
6	58711179	58711186	-	58711186	5	12	ENSSSCG00000026626	-	3'UTR	---
6	58855538	58855538	+	58855538	4	4	---	---	intergenic	---
6	58856675	58856678	-	58856676	4	10	XLOC_073607	-	predicted	---
6	58933136	58933148	-	58933143	12	33	ENSSSCG00000029147	-	3'UTR	---
6	59032233	59032238	-	59032233	10	19	XLOC_073611	-	predicted	---
6	59255122	59255139	+	59255134	218	822	XLOC_072045	+	predicted	---
6	59731834	59731834	-	59731834	5	5	---	---	intergenic	---
6	59883943	59883951	+	59883947	33	54	ENSSSCG00000003360	+	3'UTR	*WRAP73*
6	59884902	59884902	-	59884902	3	3	ENSSSCG00000003359	-	3'UTR	*TPRG1L*
6	59885013	59885023	-	59885023	5	8	ENSSSCG00000003359	-	3'UTR	*TPRG1L*
6	59885105	59885112	-	59885107	26	53	ENSSSCG00000003359	-	3'UTR	*TPRG1L*

6	59885255	59885255	-	59885255	4	4	ENSSSCG00000003359	-	3'UTR	*TPRG1L*
6	59889538	59889549	+	59889545	6	17	---	---	intergenic	---
6	59953230	59953230	+	59953230	3	3	---	---	intergenic	---
6	59958162	59958166	+	59958162	6	9	XLOC_072052	+	predicted	---
6	59996805	59996811	-	59996810	11	21	XLOC_073622	-	predicted	---
6	59996962	59996962	-	59996962	4	4	XLOC_073622	-	predicted	---
6	59997119	59997127	-	59997120	9	18	XLOC_073622	-	predicted	---
6	59997942	59997942	-	59997942	3	3	XLOC_073622	-	predicted	---
6	60037302	60037314	+	60037303	28	40	ENSSSCG00000003363	+	3'UTR	*C1orf174*
6	60739883	60739883	-	60739883	7	7	---	---	intergenic	---
6	60761264	60761264	-	60761264	4	4	---	---	intergenic	---
6	60964568	60964568	-	60964568	3	3	---	---	intergenic	---
6	61222699	61222699	-	61222699	4	4	---	---	intergenic	---
6	62004350	62004350	-	62004350	9	9	ENSSSCG00000003367	-	ORF	*RPL22*
6	62023967	62023974	-	62023968	18	29	ENSSSCG00000003369	-	3'UTR	*ICMT*
6	62026029	62026029	-	62026029	6	6	ENSSSCG00000003369	-	3'UTR	*ICMT*
6	62044009	62044016	-	62044015	7	17	XLOC_073637	-	predicted	---
6	62103744	62103749	-	62103746	77	195	ENSSSCG00000003377	-	3'UTR	*ACOT7*
6	62229152	62229152	-	62229152	5	5	XLOC_073640	-	predicted	---
6	62328395	62328401	-	62328395	15	22	ENSSSCG00000030253	-	3'UTR	*NOL9*
6	62378633	62378633	+	62378633	4	4	ENSSSCG00000003383	+	intron	*ZBTB48*
6	62379122	62379125	+	62379125	14	24	ENSSSCG00000003383	+	intron	*ZBTB48*
6	62425361	62425368	+	62425362	6	16	ENSSSCG00000003383	+	3'UTR	*ZBTB48*
							ENSSSCG00000021518	+	intron	---
6	62654893	62654894	-	62654894	5	8	XLOC_073648	-	predicted	---
6	62663924	62663924	+	62663924	5	5	---	---	intergenic	---

6	62800448	62800450	-	62800450	8	12	---	---	intergenic	---
6	62829361	62829361	-	62829361	7	7	---	---	intergenic	---
6	62858811	62858813	-	62858813	6	9	---	---	intergenic	---
6	62859038	62859040	+	62859038	12	15	---	---	intergenic	---
6	62859077	62859077	-	62859077	3	3	---	---	intergenic	---
6	62989091	62989117	+	62989101	13	129	---	---	intergenic	---
6	63030586	63030586	-	63030586	3	3	ENSSSCG00000003385	-	ORF	*PARK7*
6	63077027	63077027	+	63077027	5	5	---	---	intergenic	---
6	63118705	63118720	-	63118713	11	44	XLOC_073651	-	predicted	---
6	63119187	63119217	-	63119217	5	11	XLOC_073651	-	predicted	---
6	63119599	63119599	-	63119599	3	3	XLOC_073651	-	predicted	---
6	63276874	63276874	+	63276874	3	3	---	---	intergenic	---
6	63314451	63314457	+	63314457	9	22	XLOC_072081	+	predicted	---
6	63714816	63714817	-	63714816	6	9	---	---	intergenic	---
6	63746809	63746809	+	63746809	5	5	ENSSSCG00000022343	+	ORF	*ENO1*
6	63955239	63955239	+	63955239	8	8	XLOC_072089	+	predicted	---
6	64030221	64030221	+	64030221	3	3	XLOC_072090	+	predicted	---
6	64031143	64031148	+	64031144	44	71	XLOC_072090	+	predicted	---
6	64031609	64031609	-	64031609	5	5	ENSSSCG00000003395	-	3'UTR	*CH242-402I1 1.1*
6	64130350	64130350	-	64130350	3	3	ENSSSCG00000003393	-	intron	---
6	64148522	64148528	-	64148528	17	25	ENSSSCG00000003392	-	3'UTR	*SLC25A33*
6	64148759	64148765	-	64148762	8	23	ENSSSCG00000003392	-	3'UTR	*SLC25A33*
6	64332340	64332368	+	64332340	5	9	ENSSSCG00000003400	+	intron	---
6	64334146	64334148	+	64334148	6	9	ENSSSCG00000003400	+	3'UTR	---
6	64354961	64354961	+	64354961	4	4	---	---	intergenic	---

6	64395132	64395135	+	64395132	5	11	ENSSSCG00000027687	+	3'UTR	---
6	64395787	64395787	+	64395787	5	5	ENSSSCG00000027687	+	3'UTR	---
6	64531020	64531020	+	64531020	10	10	ENSSSCG00000003401	+	intron	*KIF1B*
6	64592142	64592142	+	64592142	6	6	ENSSSCG00000003401	+	intron	*KIF1B*
6	64592349	64592349	+	64592349	3	3	ENSSSCG00000003401	+	intron	*KIF1B*
6	64596274	64596278	+	64596275	17	36	XLOC_072095	+	predicted	---
6	64603570	64603570	+	64603570	8	8	XLOC_072095	+	predicted	---
6	64644539	64644544	+	64644539	30	41	XLOC_072098	+	predicted	---
6	64658504	64658510	-	64658504	13	25	ENSSSCG00000003404	-	3'UTR	*DFFA*
6	64658997	64658997	-	64658997	4	4	ENSSSCG00000003404	-	3'UTR	*DFFA*
6	64660020	64660020	-	64660020	5	5	ENSSSCG00000003404	-	intron	*DFFA*
6	64805057	64805057	+	64805057	8	8	ENSSSCG00000003405	+	3'UTR	*PEX14*
6	65068793	65068793	+	65068793	6	6	---	---	intergenic	---
6	65134834	65134834	+	65134834	3	3	---	---	intergenic	---
6	65136074	65136076	+	65136074	11	16	ENSSSCG00000023126	+	ORF	---
6	65142287	65142287	+	65142287	26	26	XLOC_072102	+	predicted	---
6	65142426	65142443	+	65142433	8	29	XLOC_072102	+	predicted	---
6	65144358	65144358	-	65144358	3	3	XLOC_073681	-	predicted	---
6	65153832	65153832	-	65153832	4	4	---	---	intergenic	---
6	65158859	65158862	+	65158859	48	57	ENSSSCG00000027689	+	3'UTR	*SRM*
6	65163746	65163750	-	65163749	19	33	ENSSSCG00000003412	-	3'UTR	*EXOSC10*
6	65183053	65183053	-	65183053	5	5	ENSSSCG00000003412	-	intron	*EXOSC10*
6	65191749	65191749	-	65191749	11	11	ENSSSCG00000003413	-	3'UTR	*FRAP1*
6	65335890	65335890	+	65335890	9	9	ENSSSCG00000025200	+	3'UTR	*UBIAD1*
6	65491708	65491709	+	65491709	9	12	---	---	intergenic	---
6	65508181	65508188	+	65508181	4	7	XLOC_072106	+	predicted	---

6	65660596	65660596	-	65660596	9	9	ENSSSCG00000025667	-	3'UTR	*FBXO2*
6	65673465	65673465	+	65673465	3	3	ENSSSCG00000003421	+	3'UTR	*FBXO44*
6	65685234	65685234	+	65685234	5	5	ENSSSCG00000003420	+	intron	---
6	65692515	65692515	-	65692515	4	4	XLOC_073685	-	predicted	---
6	65692529	65692532	+	65692529	4	7	ENSSSCG00000003420	+	3'UTR	---
6	65692629	65692654	-	65692634	213	444	ENSSSCG00000003419	-	3'UTR	---
6	65692630	65692630	+	65692630	4	4	ENSSSCG00000003420	+	3'UTR	---
6	65693200	65693215	+	65693215	5	11	ENSSSCG00000003420	+	3'UTR	---
6	65696817	65696817	-	65696817	3	3	ENSSSCG00000003419	-	intron	---
6	65705212	65705216	-	65705214	61	105	ENSSSCG00000022401	-	3'UTR	*AGTRAP*
6	65746356	65746359	+	65746356	4	7	---	---	intergenic	---
6	65776385	65776385	+	65776385	3	3	XLOC_072111	+	predicted	---
6	65776802	65776819	-	65776819	10	18	ENSSSCG00000003428	-	3'UTR	*MTHFR*
6	65885839	65885841	-	65885839	38	64	ENSSSCG00000003432	-	3'UTR	*KIAA2013*
6	65928881	65928890	+	65928884	37	77	ENSSSCG00000003433	+	3'UTR	*PLOD1*
6	65933513	65933513	+	65933513	5	5	XLOC_072115	+	predicted	---
6	66016338	66016346	+	66016346	21	36	ENSSSCG00000003434	+	3'UTR	*MFN2*
6	66034870	66034888	+	66034871	36	134	ENSSSCG00000003435	+	3'UTR	*MIIP*
6	66416858	66416858	+	66416858	4	4	ENSSSCG00000003438	+	intron	*VPS13D*
6	66641891	66641891	+	66641891	11	11	XLOC_072121	+	predicted	---
6	66685221	66685237	-	66685234	144	243	ENSSSCG00000003439	-	3'UTR	*DHRS3*
6	66738598	66738598	+	66738598	11	11	---	---	intergenic	---
6	66828836	66828837	-	66828837	9	12	---	---	intergenic	---
6	66844912	66844917	+	66844912	7	38	ENSSSCG00000003442	+	ORF	*C1orf158*
6	66846513	66846535	+	66846534	38	78	XLOC_072127	+	predicted	---
6	67435837	67435837	-	67435837	6	6	---	---	intergenic	---

6	67478309	67478311	+	67478309	10	18	XLOC_072134	+	predicted	---
6	68076522	68076522	+	68076522	3	3	---	---	intergenic	---
6	68165879	68165879	+	68165879	3	3	---	---	intergenic	---
6	68919494	68919494	-	68919494	3	3	---	---	intergenic	---
6	68941543	68941564	-	68941564	8	32	ENSSSCG00000003455	-	3'UTR	---
6	68941895	68941895	-	68941895	4	4	ENSSSCG00000003455	-	3'UTR	---
6	69049427	69049436	+	69049432	5	12	ENSSSCG00000003458	+	3'UTR	*EFHD2*
6	69049559	69049567	+	69049559	30	35	ENSSSCG00000003458	+	3'UTR	*EFHD2*
6	69081328	69081328	+	69081328	5	5	ENSSSCG00000003459	+	intron	*CELA2A*
6	69088195	69088204	+	69088197	28	55	ENSSSCG00000003459	+	3'UTR	*CELA2A*
6	69117506	69117507	-	69117506	7	14	---	---	intergenic	---
6	69134556	69134556	-	69134556	3	3	XLOC_073715	-	predicted	---
6	69211311	69211356	+	69211319	76	146	ENSSSCG00000003463	+	3'UTR	*AGMAT*
6	69217763	69217763	-	69217763	10	10	XLOC_073716	-	predicted	---
6	69328322	69328322	-	69328322	3	3	---	---	intergenic	---
6	69342257	69342257	-	69342257	20	20	ENSSSCG00000003465	-	3'UTR	*FBLIM1*
6	69342887	69342887	-	69342887	9	9	ENSSSCG00000003465	-	3'UTR	*FBLIM1*
6	69389610	69389610	-	69389610	3	3	XLOC_073718	-	predicted	---
6	69396274	69396275	-	69396275	14	17	ENSSSCG00000003468	-	3'UTR	*PLEKHM2*
6	69404852	69404852	-	69404852	3	3	ENSSSCG00000003468	-	intron	*PLEKHM2*
6	69416399	69416399	+	69416399	4	4	---	---	intergenic	---
6	69567148	69567153	-	69567153	5	11	ENSSSCG00000003471	-	3'UTR	*EPHA2*
6	69680297	69680298	-	69680298	6	11	ENSSSCG00000024794	-	3'UTR	*FBXO42*
6	69759696	69759698	-	69759696	3	6	ENSSSCG00000024794	-	intron	*FBXO42*
6	69766371	69766371	-	69766371	4	4	XLOC_073727	-	predicted	---
6	69797848	69797849	+	69797848	13	17	XLOC_072151	+	predicted	---

6	69801019	69801021	-	69801021	24	27	XLOC_073729	-	predicted	---
6	69839885	69839887	-	69839885	5	13	---	---	intergenic	---
6	69846522	69846537	+	69846531	15	34	ENSSSCG00000003477	+	ORF	*NECAP2*
6	69855205	69855223	+	69855218	28	67	ENSSSCG00000003477	+	3'UTR	*NECAP2*
6	69855995	69855995	+	69855995	3	3	ENSSSCG00000003477	+	3'UTR	*NECAP2*
6	69856064	69856069	+	69856064	25	40	ENSSSCG00000003477	+	3'UTR	*NECAP2*
6	69864055	69864055	-	69864055	4	4	---	---	intergenic	---
6	69990915	69990933	-	69990915	57	60	ENSSSCG00000020926	-	3'UTR	*SDHB*
6	70039610	70039610	-	70039610	3	3	ENSSSCG00000026478	-	3'UTR	*PADI2*
6	70166009	70166009	+	70166009	3	3	---	---	intergenic	---
6	70310366	70310372	+	70310372	9	12	XLOC_072159	+	predicted	---
6	70334871	70334881	-	70334873	11	40	ENSSSCG00000003485	-	3'UTR	*RCC2*
6	70336961	70336964	-	70336961	9	21	ENSSSCG00000003485	-	3'UTR	*RCC2*
6	70578374	70578414	+	70578414	22	36	ENSSSCG00000022946	+	3'UTR	---
6	70650491	70650491	-	70650491	8	8	---	---	intergenic	---
6	70842835	70842835	-	70842835	3	3	---	---	intergenic	---
6	70909603	70909603	+	70909603	3	3	---	---	intergenic	---
6	70937622	70937622	+	70937622	3	3	---	---	intergenic	---
6	71000413	71000413	-	71000413	8	8	---	---	intergenic	---
6	71078233	71078233	+	71078233	3	3	---	---	intergenic	---
6	71470901	71470902	-	71470901	4	7	ENSSSCG00000022839	-	3'UTR	---
6	71512628	71512635	+	71512630	61	104	ENSSSCG00000024752	+	3'UTR	*ALDH4A1*
6	71574279	71574279	-	71574279	3	3	ENSSSCG00000003488	-	ORF	---
6	71815721	71815740	+	71815739	20	36	ENSSSCG00000003490	+	3'UTR	*MRTO4*
6	71816019	71816027	+	71816020	17	29	ENSSSCG00000003490	+	3'UTR	*MRTO4*
6	71816266	71816277	-	71816269	91	145	ENSSSCG00000003491	-	3'UTR	*AKR7A2*

6	71840700	71840709	+	71840703	6	18	XLOC_072172	+	predicted	---
6	71842814	71842820	-	71842816	123	142	ENSSSCG00000003493	-	3'UTR	*CAPZB*
6	71843192	71843192	-	71843192	3	3	ENSSSCG00000003493	-	3'UTR	*CAPZB*
6	71843391	71843393	-	71843393	36	82	ENSSSCG00000003493	-	3'UTR	*CAPZB*
6	71848053	71848054	-	71848053	6	9	ENSSSCG00000003493	-	intron	*CAPZB*
6	72077845	72077848	+	72077845	3	9	---	---	intergenic	---
6	72080065	72080065	-	72080065	3	3	XLOC_073749	-	predicted	---
6	72116279	72116279	+	72116279	3	3	ENSSSCG00000028414	+	ORF	*MINOS1*
6	72118801	72118801	+	72118801	13	13	ENSSSCG00000028414	+	3'UTR	*MINOS1*
6	72118887	72118898	+	72118888	151	361	ENSSSCG00000028414	+	3'UTR	*MINOS1*
6	72142019	72142022	+	72142020	11	29	XLOC_072173	+	predicted	---
6	72371378	72371378	+	72371378	4	4	---	---	intergenic	---
6	72519528	72519528	-	72519528	3	3	ENSSSCG00000003494	-	intron	*PLA2G2A*
6	72523769	72523769	+	72523769	3	3	---	---	intergenic	---
6	72554981	72554982	-	72554982	6	9	---	---	intergenic	---
6	72555009	72555011	+	72555009	9	21	---	---	intergenic	---
6	72580442	72580442	-	72580442	11	11	ENSSSCG00000003497	-	3'UTR	*PLA2G2D*
6	72654029	72654029	+	72654029	5	5	ENSSSCG00000003498	+	ORF	---
6	72654807	72654808	+	72654808	5	9	ENSSSCG00000003498	+	3'UTR	*UBXN10*
							ENSSSCG00000003498	+	ORF	---
6	72654857	72654877	+	72654877	25	111	XLOC_072179	+	predicted	---
6	72656509	72656509	+	72656509	3	3	XLOC_072179	+	predicted	---
6	72662376	72662376	-	72662376	8	8	---	---	intergenic	---
6	72738890	72738892	-	72738890	4	8	XLOC_073760	-	predicted	---
6	72919020	72919021	-	72919021	5	9	ENSSSCG00000024009	-	3'UTR	*CAMK2N1*
6	72940826	72940831	-	72940827	25	52	ENSSSCG00000028045	-	3'UTR	*MUL1*

6	72983927	72983927	+	72983927	4	4	XLOC_072182	+	predicted	---
6	73042846	73042853	+	73042853	12	17	---	---	intergenic	---
6	73050213	73050214	+	73050213	3	6	---	---	intergenic	---
6	73127138	73127142	+	73127140	25	42	ENSSSCG00000003506	+	3'UTR	*PINK1*
6	73128310	73128342	+	73128318	49	160	ENSSSCG00000003506	+	3'UTR	*PINK1*
6	73128590	73128590	-	73128590	4	4	ENSSSCG00000003505	-	3'UTR	*OST48*
6	73128938	73128946	-	73128945	173	276	ENSSSCG00000003505	-	3'UTR	*OST48*
6	73141558	73141565	-	73141560	65	196	XLOC_073764	-	predicted	---
6	73219225	73219233	-	73219233	14	34	XLOC_073767	-	predicted	---
6	73220561	73220561	-	73220561	7	7	XLOC_073767	-	predicted	---
6	73232878	73232878	-	73232878	3	3	ENSSSCG00000003511	-	intron	*HP1BP3*
6	73237546	73237546	-	73237546	6	6	ENSSSCG00000003511	-	intron	*HP1BP3*
6	73324696	73324707	+	73324701	7	18	XLOC_072187	+	predicted	---
6	73419094	73419100	-	73419095	13	26	ENSSSCG00000026340	-	intron	---
6	73482828	73482828	-	73482828	3	3	---	---	intergenic	---
6	73553182	73553182	-	73553182	4	4	---	---	intergenic	---
6	73558984	73558984	-	73558984	3	3	---	---	intergenic	---
6	73559757	73559757	-	73559757	3	3	---	---	intergenic	---
6	73659669	73659679	-	73659675	32	108	XLOC_073770	-	predicted	---
6	73858998	73859005	+	73858999	31	47	XLOC_072190	+	predicted	---
6	73869208	73869208	-	73869208	5	5	ENSSSCG00000003516	-	intron	---
6	73951535	73951538	+	73951535	20	54	XLOC_072192	+	predicted	---
6	73951656	73951657	+	73951657	6	9	XLOC_072192	+	predicted	---
6	73951719	73951719	+	73951719	10	10	XLOC_072192	+	predicted	---
6	74018220	74018233	-	74018223	169	594	XLOC_073778	-	predicted	---

6	74039555	74039555	+	74039555	4	4	ENSSSCG00000030015	+	3'UTR	---
							ENSSSCG00000030015	+	ORF	*protein_coding*
6	74114006	74114006	+	74114006	3	3	ENSSSCG00000003520	+	3'UTR	*CDC42*
							ENSSSCG00000003520	+	intron	*CDC42*
							ENSSSCG00000003520	+	ORF	---
6	74115501	74115510	+	74115501	14	17	ENSSSCG00000003520	+	3'UTR	*CDC42*
6	74116279	74116282	+	74116281	11	20	ENSSSCG00000003520	+	3'UTR	*CDC42*
6	74116710	74116710	+	74116710	3	3	ENSSSCG00000003520	+	3'UTR	*CDC42*
6	74116819	74116827	+	74116820	27	66	ENSSSCG00000003520	+	3'UTR	*CDC42*
6	74252985	74252985	-	74252985	4	4	---	---	intergenic	---
6	74347760	74347760	+	74347760	3	3	---	---	intergenic	---
6	74612969	74612984	+	74612974	121	278	ENSSSCG00000003524	+	3'UTR	*C1QA*
6	74621205	74621231	+	74621224	84	108	ENSSSCG00000003525	+	3'UTR	*C1QC*
6	74655525	74655525	+	74655525	5	5	XLOC_072204	+	predicted	---
6	74932046	74932059	-	74932046	4	7	XLOC_073788	-	predicted	---
6	74932430	74932433	-	74932431	38	48	XLOC_073788	-	predicted	---
6	75073951	75073960	+	75073953	14	20	ENSSSCG00000003531	+	3'UTR	*KDM1A*
6	75075341	75075348	-	75075341	5	8	ENSSSCG00000003532	-	3'UTR	*LUZP1*
6	75078896	75078896	-	75078896	3	3	ENSSSCG00000003532	-	3'UTR	*LUZP1*
6	75214320	75214321	-	75214320	7	13	XLOC_073791	-	predicted	---
6	75261853	75261861	+	75261853	26	31	ENSSSCG00000023761	+	3'UTR	---
6	75366950	75366950	+	75366950	6	6	XLOC_072212	+	predicted	---
6	75388374	75388383	+	75388374	4	8	ENSSSCG00000027659	+	3'UTR	*FUCA1*
6	75388860	75388862	+	75388861	8	19	ENSSSCG00000027659	+	3'UTR	*FUCA1*
6	75415335	75415345	+	75415339	75	212	ENSSSCG00000026025	+	3'UTR	*HMGCL*

6	75415434	75415436	+	75415434	5	10	ENSSSCG00000026025	+	3'UTR	*HMGCL*
6	75420888	75420892	+	75420889	27	71	ENSSSCG00000027827	+	3'UTR	*GALE*
6	75420946	75420953	-	75420946	10	26	ENSSSCG00000023191	-	3'UTR	*LYPLA2*
6	75427988	75427991	-	75427989	51	73	ENSSSCG00000023495	-	3'UTR	*PITHD1*
6	75446955	75446965	-	75446964	4	10	ENSSSCG00000025440	-	3'UTR	*TCEB3*
6	75463625	75463625	-	75463625	4	4	---	---	intergenic	---
6	75499938	75499965	-	75499941	498	1166	ENSSSCG00000024260	-	3'UTR	*RPL11*
6	75500476	75500491	-	75500476	15	21	ENSSSCG00000024260	-	ORF	*RPL11*
6	75502242	75502274	-	75502274	9	12	ENSSSCG00000024260	-	ORF	*RPL11*
6	75538793	75538794	-	75538794	39	42	---	---	intergenic	---
6	75703419	75703419	+	75703419	3	3	XLOC_072219	+	predicted	---
6	75855195	75855195	+	75855195	3	3	XLOC_072221	+	predicted	---
6	75856358	75856358	+	75856358	3	3	XLOC_072221	+	predicted	---
6	75927971	75927971	+	75927971	7	7	ENSSSCG00000024818	+	3'UTR	*RCAN3*
6	76013401	76013401	+	76013401	13	13	---	---	intergenic	---
6	76014328	76014338	+	76014328	5	9	XLOC_072226	+	predicted	---
6	76020680	76020680	+	76020680	13	13	XLOC_072227	+	predicted	---
6	76032307	76032307	-	76032307	3	3	---	---	intergenic	---
6	76038586	76038586	+	76038586	6	6	XLOC_072227	+	predicted	---
6	76241507	76241507	+	76241507	3	3	XLOC_072229	+	predicted	---
6	76244795	76244799	+	76244798	33	49	XLOC_072229	+	predicted	---
6	76244844	76244849	+	76244848	14	25	XLOC_072229	+	predicted	---
6	76246362	76246362	-	76246362	3	3	---	---	intergenic	---
6	76252036	76252036	+	76252036	4	4	---	---	intergenic	---
6	76287653	76287653	+	76287653	3	3	XLOC_072231	+	predicted	---
6	76338546	76338546	-	76338546	3	3	---	---	intergenic	---

6	76566363	76566368	+	76566367	13	27	---	---	intergenic	---
6	76625124	76625124	+	76625124	4	4	ENSSSCG00000003549	+	3'UTR	*LDLRAP1*
6	76643860	76643861	+	76643861	13	22	---	---	intergenic	---
6	76797556	76797563	+	76797557	34	56	ENSSSCG00000003553	+	intron	*MTFR1L*
6	76832592	76832592	+	76832592	3	3	ENSSSCG00000003553	+	3'UTR	*MTFR1L*
6	76833006	76833017	+	76833011	11	41	ENSSSCG00000003553	+	intron	*MTFR1L*
6	76926307	76926307	-	76926307	4	4	---	---	intergenic	---
6	76927411	76927412	-	76927412	12	15	---	---	intergenic	---
6	76930801	76930801	+	76930801	5	5	ENSSSCG00000026257	+	ORF	*STMN1*
6	76933155	76933155	+	76933155	3	3	ENSSSCG00000026257	+	intron	*STMN1*
6	76933699	76933711	+	76933709	121	243	ENSSSCG00000026257	+	3'UTR	*STMN1*
6	76933966	76933966	+	76933966	6	6	ENSSSCG00000026257	+	3'UTR	*STMN1*
6	76934031	76934042	+	76934042	14	21	ENSSSCG00000026257	+	3'UTR	*STMN1*
6	76934092	76934109	+	76934103	208	1478	ENSSSCG00000026257	+	3'UTR	*STMN1*
6	77034675	77034679	-	77034679	6	9	---	---	intergenic	---
6	77049137	77049137	-	77049137	3	3	ENSSSCG00000024172	-	3'UTR	*PAFAH2*
6	77049953	77049953	-	77049953	3	3	ENSSSCG00000024172	-	3'UTR	*PAFAH2*
6	77050599	77050602	-	77050602	13	26	ENSSSCG00000024172	-	3'UTR	*PAFAH2*
6	77051057	77051061	-	77051057	3	6	ENSSSCG00000024172	-	3'UTR	*PAFAH2*
6	77051890	77051890	-	77051890	3	3	ENSSSCG00000024172	-	3'UTR	*PAFAH2*
6	77102697	77102697	+	77102697	5	5	ENSSSCG00000029558	+	intron	*EXTL1*
6	77112252	77112252	+	77112252	12	12	XLOC_072244	+	predicted	---
6	77113413	77113413	-	77113413	4	4	ENSSSCG00000026386	-	3'UTR	*SLC30A2*
6	77175751	77175751	+	77175751	3	3	---	---	intergenic	---
6	77219636	77219643	+	77219642	24	39	ENSSSCG00000026526	+	intron	*CATSPER4*
6	77320027	77320027	-	77320027	8	8	XLOC_073818	-	predicted	---

6	77399630	77399638	+	77399632	6	16	XLOC_072249	+	predicted	---
6	77401633	77401634	+	77401634	4	7	XLOC_072249	+	predicted	---
6	77405460	77405473	+	77405473	14	31	ENSSSCG00000003555	+	3'UTR	*HMGN2*
6	77406188	77406199	+	77406197	359	1102	ENSSSCG00000003555	+	3'UTR	*HMGN2*
6	77406878	77406878	+	77406878	7	7	XLOC_072249	+	predicted	---
6	77480445	77480450	+	77480446	23	36	---	---	intergenic	---
6	77617115	77617115	+	77617115	3	3	XLOC_072254	+	predicted	---
6	77750874	77750874	+	77750874	12	12	XLOC_072256	+	predicted	---
6	77752521	77752524	+	77752521	12	18	XLOC_072256	+	predicted	---
6	77770930	77770930	-	77770930	5	5	XLOC_073830	-	predicted	---
6	77780078	77780079	-	77780078	8	11	ENSSSCG00000003564	-	3'UTR	*GPATCH3*
6	77783522	77783522	-	77783522	18	18	ENSSSCG00000003564	-	intron	*GPATCH3*
6	77798348	77798358	+	77798354	33	99	ENSSSCG00000003565	+	3'UTR	*NR0B2*
6	77818470	77818482	+	77818480	24	63	ENSSSCG00000003566	+	ORF	*NUDC*
6	77818646	77818646	+	77818646	4	4	ENSSSCG00000003566	+	intron	*NUDC*
6	77822239	77822241	+	77822239	184	294	ENSSSCG00000003566	+	3'UTR	*NUDC*
6	77824703	77824703	-	77824703	3	3	ENSSSCG00000003567	-	3'UTR	*KDF1*
6	77851114	77851114	+	77851114	13	13	---	---	intergenic	---
6	78071463	78071463	+	78071463	4	4	ENSSSCG00000003570	+	intron	*WDTC1*
6	78105856	78105856	+	78105856	3	3	XLOC_072263	+	predicted	---
6	78107200	78107206	+	78107203	33	55	XLOC_072263	+	predicted	---
6	78130578	78130582	+	78130580	39	67	ENSSSCG00000023636	+	3'UTR	*TMEM222*
6	78144409	78144409	+	78144409	6	6	ENSSSCG00000003572	+	3'UTR	*SYTL1*
6	78169610	78169623	-	78169620	26	74	XLOC_073837	-	predicted	---
6	78192955	78192965	-	78192955	5	8	XLOC_073838	-	predicted	---
6	78196198	78196202	-	78196198	3	6	XLOC_073838	-	predicted	---

6	78196509	78196518	-	78196509	3	9	XLOC_073838	-	predicted	---
6	78203752	78203752	-	78203752	9	9	---	---	intergenic	---
6	78416235	78416235	-	78416235	10	10	---	---	intergenic	---
6	78417892	78417892	-	78417892	5	5	---	---	intergenic	---
6	78418333	78418333	-	78418333	3	3	---	---	intergenic	---
6	78422327	78422327	-	78422327	6	6	ENSSSCG00000027894	-	intron	*FAM76A*
6	78449540	78449541	+	78449540	3	6	---	---	intergenic	---
6	78454463	78454464	+	78454463	5	8	XLOC_072270	+	predicted	---
6	78485450	78485450	+	78485450	3	3	ENSSSCG00000024927	+	intron	*STX12*
6	78500567	78500568	+	78500567	9	14	ENSSSCG00000024927	+	3'UTR	*STX12*
6	78501691	78501698	+	78501692	10	16	ENSSSCG00000024927	+	3'UTR	*STX12*
6	78501973	78501980	+	78501980	5	8	ENSSSCG00000024927	+	3'UTR	*STX12*
6	78532135	78532139	+	78532136	19	28	XLOC_072272	+	predicted	---
6	78568254	78568254	+	78568254	6	6	ENSSSCG00000003584	+	3'UTR	*THEMIS2*
6	78572939	78572942	-	78572940	20	30	ENSSSCG00000003583	-	3'UTR	*RPA2*
6	78572988	78572988	-	78572988	3	3	ENSSSCG00000003583	-	3'UTR	*RPA2*
6	78573411	78573411	-	78573411	4	4	ENSSSCG00000003583	-	3'UTR	*RPA2*
6	78625032	78625041	+	78625033	49	61	XLOC_072275	+	predicted	---
6	78633738	78633738	+	78633738	4	4	ENSSSCG00000030623	+	3'UTR	*XKR8*
6	78636359	78636359	-	78636359	7	7	ENSSSCG00000003580	-	3'UTR	---
6	79043668	79043668	-	79043668	3	3	ENSSSCG00000028809	-	intron	---
6	79047616	79047636	+	79047616	4	7	ENSSSCG00000027072	+	ORF	*ATPIF1*
6	79047795	79047809	+	79047806	285	687	ENSSSCG00000027072	+	3'UTR	*ATPIF1*
6	79087495	79087495	+	79087495	15	15	ENSSSCG00000029438	+	3'UTR	---
6	79156753	79156754	+	79156753	9	18	ENSSSCG00000028973	+	3'UTR	*MED18*
6	79232672	79232672	+	79232672	5	5	---	---	intergenic	---

6	79343714	79343714	+	79343714	4	4	XLOC_072280	+	predicted	---
6	79347355	79347355	-	79347355	3	3	---	---	intergenic	---
6	79350388	79350390	+	79350388	3	6	XLOC_072281	+	predicted	---
6	79350429	79350429	+	79350429	3	3	XLOC_072281	+	predicted	---
6	79377519	79377530	+	79377520	39	74	ENSSSCG00000029097	+	3'UTR	*RCC1*
6	79377627	79377627	+	79377627	3	3	ENSSSCG00000029097	+	3'UTR	*RCC1*
6	79388560	79388560	+	79388560	5	5	ENSSSCG00000030284	+	intron	*TRNAU1AP*
6	79406912	79406919	+	79406919	15	26	ENSSSCG00000030284	+	3'UTR	*TRNAU1AP*
6	79408019	79408019	-	79408019	11	11	XLOC_073853	-	predicted	---
6	79408230	79408232	-	79408232	22	58	ENSSSCG00000027280	-	ORF	*SNORD99*
6	79410573	79410573	-	79410573	4	4	ENSSSCG00000028807	-	ORF	*SNORA16*
6	79419316	79419319	+	79419316	16	31	ENSSSCG00000030432	+	3'UTR	*RAB42*
6	79432411	79432419	-	79432418	27	52	ENSSSCG00000027530	-	3'UTR	*TAF12*
6	79432669	79432670	-	79432670	13	16	ENSSSCG00000027530	-	3'UTR	*TAF12*
6	79440876	79440876	-	79440876	12	12	ENSSSCG00000027530	-	intron	*TAF12*
6	79583192	79583192	+	79583192	4	4	XLOC_072285	+	predicted	---
6	79583682	79583689	+	79583688	44	61	XLOC_072285	+	predicted	---
6	79913946	79913946	+	79913946	3	3	XLOC_072287	+	predicted	---
6	79916241	79916248	-	79916248	7	20	XLOC_073861	-	predicted	---
6	79952092	79952094	-	79952094	6	10	XLOC_073862	-	predicted	---
6	80009697	80009697	-	80009697	4	4	ENSSSCG00000003589	-	intron	*MECR*
6	80050685	80050685	+	80050685	5	5	ENSSSCG00000003590	+	intron	*PTPRU*
6	80077406	80077416	+	80077411	13	26	ENSSSCG00000003590	+	intron	*PTPRU*
6	80106250	80106250	+	80106250	3	3	ENSSSCG00000003590	+	3'UTR	*PTPRU*
6	80370642	80370642	+	80370642	9	9	---	---	intergenic	---
6	81064610	81064610	-	81064610	3	3	---	---	intergenic	---

6	81068824	81068835	-	81068832	93	225	XLOC_073869	-	predicted	---
6	81069620	81069620	-	81069620	5	5	XLOC_073869	-	predicted	---
6	81069788	81069812	-	81069803	31	50	XLOC_073869	-	predicted	---
6	81090189	81090189	-	81090189	3	3	ENSSSCG00000025034	-	intron	*LAPTM5*
6	81197513	81197513	+	81197513	3	3	ENSSSCG00000003591	+	intron	---
6	81236285	81236287	+	81236286	32	46	ENSSSCG00000003591	+	3'UTR	---
6	81287281	81287288	+	81287286	46	111	ENSSSCG00000003592	+	3'UTR	*SDC3*
6	81456290	81456294	-	81456294	5	8	---	---	intergenic	---
6	81545690	81545695	-	81545695	7	18	ENSSSCG00000003596	-	3'UTR	*SNRNP40*
6	81545801	81545801	-	81545801	3	3	ENSSSCG00000003596	-	3'UTR	*SNRNP40*
6	81603141	81603141	+	81603141	3	3	ENSSSCG00000028826	+	intron	---
6	81620324	81620325	+	81620324	8	15	---	---	intergenic	---
6	81631958	81631990	+	81631962	26	68	XLOC_072301	+	predicted	---
6	81656657	81656657	+	81656657	3	3	XLOC_072301	+	predicted	---
6	81728944	81728950	-	81728948	9	28	ENSSSCG00000003595	-	3'UTR	---
6	81821186	81821189	+	81821188	19	25	ENSSSCG00000003600	+	3'UTR	*TINAGL1*
6	81867264	81867274	-	81867272	24	78	ENSSSCG00000003602	-	intron	*PEF1*
6	81986673	81986673	+	81986673	3	3	---	---	intergenic	---
6	82206158	82206158	-	82206158	5	5	ENSSSCG00000003606	-	intron	*SPOCD1*
6	82335477	82335484	-	82335478	23	60	XLOC_073889	-	predicted	---
6	82337706	82337706	-	82337706	3	3	XLOC_073889	-	predicted	---
6	82349981	82349982	-	82349982	5	9	---	---	intergenic	---
6	82414420	82414420	+	82414420	3	3	ENSSSCG00000023122	+	intron	---
6	82414860	82414860	+	82414860	5	5	ENSSSCG00000023122	+	intron	---
6	82435655	82435655	+	82435655	3	3	XLOC_072314	+	predicted	---
6	82459084	82459084	-	82459084	3	3	XLOC_073890	-	predicted	---

6	82562506	82562508	-	82562507	31	55	ENSSSCG00000027436	-	intron	---
6	82606453	82606453	-	82606453	3	3	---	---	intergenic	---
6	82622084	82622084	+	82622084	3	3	---	---	intergenic	---
6	82690613	82690614	-	82690614	5	8	ENSSSCG00000003616	-	3'UTR	*FAM167B*
6	82715157	82715157	+	82715157	5	5	XLOC_072321	+	predicted	---
6	82718140	82718142	+	82718141	16	27	ENSSSCG00000003610	+	3'UTR	*IQCC*
6	82724214	82724215	-	82724214	5	9	ENSSSCG00000003611	-	3'UTR	*TMEM234*
6	82737474	82737504	+	82737476	173	231	ENSSSCG00000003612	+	3'UTR	*EIF3I*
6	82739484	82739492	+	82739490	10	18	---	---	intergenic	---
6	82741138	82741138	-	82741138	7	7	XLOC_073897	-	predicted	---
6	82758565	82758580	+	82758567	27	59	ENSSSCG00000003617	+	ORF	*TXLNA*
6	82780888	82780907	+	82780893	41	74	XLOC_072327	+	predicted	---
6	82781203	82781210	+	82781208	7	21	XLOC_072327	+	predicted	---
6	82781435	82781460	-	82781436	13	65	XLOC_073899	-	predicted	---
6	82782734	82782756	-	82782742	62	125	XLOC_073899	-	predicted	---
6	82907510	82907510	-	82907510	3	3	ENSSSCG00000029450	-	ORF	*HDAC1*
6	82942507	82942507	+	82942507	3	3	ENSSSCG00000028423	+	ORF	*RBBP4*
6	83010431	83010433	-	83010431	5	8	ENSSSCG00000029799	-	3'UTR	*ZBTB8OS*
6	83032271	83032274	+	83032272	10	31	ENSSSCG00000028423	+	3'UTR	*RBBP4*
6	83032944	83032946	+	83032946	6	9	ENSSSCG00000028423	+	3'UTR	*RBBP4*
6	83033093	83033103	+	83033097	53	190	ENSSSCG00000028423	+	3'UTR	*RBBP4*
6	83114732	83114732	+	83114732	5	5	ENSSSCG00000030369	+	3'UTR	*KIAA1522*
6	83115016	83115022	-	83115022	23	44	ENSSSCG00000029457	-	3'UTR	*YARS*
6	83115436	83115437	-	83115437	18	21	ENSSSCG00000029457	-	3'UTR	*YARS*
6	83184939	83184952	+	83184945	73	150	ENSSSCG00000022801	+	3'UTR	*S100PBP*
6	83185380	83185392	+	83185388	8	15	XLOC_072335	+	predicted	---

6	83187303	83187307	+	83187304	10	17	XLOC_072335	+	predicted	---
6	83246480	83246480	-	83246480	3	3	XLOC_073907	-	predicted	---
6	83251436	83251456	-	83251447	30	108	XLOC_073907	-	predicted	---
6	83251516	83251531	-	83251517	7	16	XLOC_073907	-	predicted	---
6	83252751	83252758	-	83252752	24	52	XLOC_073907	-	predicted	---
6	83263456	83263456	-	83263456	8	8	ENSSSCG00000025206	-	intron	*RNF19B*
6	83289839	83289839	+	83289839	4	4	---	---	intergenic	---
6	83470334	83470334	+	83470334	3	3	ENSSSCG00000003620	+	3'UTR	*TRIM62*
6	83490641	83490644	-	83490643	44	63	XLOC_073908	-	predicted	---
6	83568814	83568814	+	83568814	6	6	XLOC_072341	+	predicted	---
6	83598932	83598932	+	83598932	3	3	ENSSSCG00000025344	+	3'UTR	---
6	83599958	83599960	+	83599959	13	22	ENSSSCG00000025344	+	3'UTR	---
6	83656261	83656261	+	83656261	3	3	XLOC_072344	+	predicted	---
6	83781472	83781473	-	83781473	12	18	---	---	intergenic	---
6	83827726	83827727	-	83827727	6	9	XLOC_073913	-	predicted	---
6	84249129	84249129	+	84249129	3	3	---	---	intergenic	---
6	84312897	84312909	-	84312903	98	292	XLOC_073915	-	predicted	---
6	84361365	84361373	+	84361367	19	45	XLOC_072348	+	predicted	---
6	84615099	84615099	+	84615099	38	38	---	---	intergenic	---
6	84659569	84659569	+	84659569	3	3	---	---	intergenic	---
6	85300620	85300620	+	85300620	3	3	ENSSSCG00000003629	+	3'UTR	*EIF2C4*
6	85364219	85364219	+	85364219	6	6	XLOC_072357	+	predicted	---
6	85365452	85365452	+	85365452	4	4	XLOC_072357	+	predicted	---
6	85515547	85515547	+	85515547	3	3	XLOC_072360	+	predicted	---
6	85519632	85519632	-	85519632	3	3	XLOC_073928	-	predicted	---
6	85527445	85527445	+	85527445	4	4	ENSSSCG00000003633	+	ORF	*TEKT2*

6	85527635	85527644	+	85527636	148	482	ENSSSCG00000003633	+	3'UTR	*TEKT2*
6	85534266	85534274	+	85534274	5	11	ENSSSCG00000003634	+	3'UTR	*ADPRHL2*
6	85630049	85630052	-	85630050	34	60	ENSSSCG00000003637	-	3'UTR	---
6	85630456	85630462	-	85630462	5	9	ENSSSCG00000003637	-	3'UTR	---
6	85634086	85634088	-	85634088	6	10	ENSSSCG00000003637	-	intron	---
6	85668589	85668591	+	85668589	10	26	ENSSSCG00000003638	+	3'UTR	---
6	85669081	85669091	+	85669082	74	219	ENSSSCG00000003638	+	3'UTR	---
6	85806872	85806872	-	85806872	3	3	---	---	intergenic	---
6	85933102	85933102	-	85933102	9	9	---	---	intergenic	---
6	86062851	86062851	+	86062851	4	4	---	---	intergenic	---
6	86578365	86578365	+	86578365	6	6	---	---	intergenic	---
6	86656189	86656196	-	86656189	4	7	XLOC_073936	-	predicted	---
6	86722298	86722302	-	86722300	46	86	ENSSSCG00000003643	-	3'UTR	*SF3A3*
6	86777227	86777227	+	86777227	3	3	ENSSSCG00000003645	+	3'UTR	*UTP11L*
6	86777319	86777325	+	86777320	12	25	ENSSSCG00000003645	+	3'UTR	*UTP11L*
6	86777598	86777608	+	86777607	14	31	---	---	intergenic	---
6	86848901	86848901	+	86848901	4	4	---	---	intergenic	---
6	86985980	86985989	+	86985988	24	92	---	---	intergenic	---
6	87601317	87601317	+	87601317	5	5	---	---	intergenic	---
6	87672716	87672716	+	87672716	12	12	---	---	intergenic	---
6	87834980	87834982	-	87834982	7	12	XLOC_073944	-	predicted	---
6	87854721	87854729	-	87854729	7	14	XLOC_073944	-	predicted	---
6	87855743	87855745	-	87855744	11	19	ENSSSCG00000003649	-	3'UTR	---
6	87856753	87856775	-	87856763	28	102	ENSSSCG00000003649	-	3'UTR	---
6	87856898	87856899	-	87856898	3	6	ENSSSCG00000003649	-	3'UTR	---
6	87865784	87865784	-	87865784	3	3	ENSSSCG00000003650	-	ORF	*GJA9*

6	87866364	87866364	+	87866364	7	7	XLOC_072374	+	predicted	---
6	87872455	87872455	-	87872455	6	6	ENSSSCG00000003651	-	3'UTR	*RHBDL2*
6	87872758	87872758	-	87872758	3	3	ENSSSCG00000003651	-	3'UTR	*RHBDL2*
6	87916218	87916218	+	87916218	7	7	---	---	intergenic	---
6	87951299	87951305	+	87951305	22	63	ENSSSCG00000003652	+	3'UTR	*AKIRIN1*
6	87952581	87952602	+	87952581	8	38	ENSSSCG00000003652	+	3'UTR	*AKIRIN1*
6	87968992	87968999	+	87968997	114	328	ENSSSCG00000003653	+	3'UTR	*NDUFS5*
6	88349209	88349209	+	88349209	11	11	ENSSSCG00000003654	+	intron	*MACF1*
6	88358193	88358193	+	88358193	3	3	ENSSSCG00000003654	+	ORF	*MACF1*
6	88402384	88402384	+	88402384	3	3	ENSSSCG00000003654	+	3'UTR	*MACF1*
6	88403053	88403058	+	88403053	15	30	ENSSSCG00000003654	+	3'UTR	*MACF1*
6	88403215	88403215	+	88403215	3	3	XLOC_072377	+	predicted	---
6	88468840	88468842	-	88468840	3	6	ENSSSCG00000003660	-	3'UTR	*PABPC4*
6	88478450	88478450	-	88478450	3	3	ENSSSCG00000003660	-	intron	*PABPC4*
6	88485912	88485912	+	88485912	3	3	---	---	intergenic	---
6	88652057	88652065	+	88652058	25	53	ENSSSCG00000003664	+	3'UTR	---
6	88777779	88777779	-	88777779	3	3	ENSSSCG00000003667	-	3'UTR	*TRIT1*
6	88871585	88871585	+	88871585	3	3	---	---	intergenic	---
6	88878883	88878883	-	88878883	6	6	---	---	intergenic	---
6	88887891	88887898	-	88887898	7	10	XLOC_073962	-	predicted	---
6	88901739	88901739	+	88901739	4	4	---	---	intergenic	---
6	88945368	88945389	+	88945384	25	95	ENSSSCG00000003669	+	3'UTR	*MFSD2A*
6	89003487	89003497	-	89003496	13	20	---	---	intergenic	---
6	89123148	89123148	-	89123148	3	3	XLOC_073963	-	predicted	---
6	89134934	89134934	-	89134934	8	8	---	---	intergenic	---
6	89137696	89137705	-	89137703	143	222	XLOC_073965	-	predicted	---

6	89147724	89147724	-	89147724	7	7	ENSSSCG00000003670	-	3'UTR	*RLF*
6	89239003	89239003	-	89239003	5	5	ENSSSCG00000003670	-	intron	*RLF*
6	89434722	89434722	+	89434722	3	3	ENSSSCG00000023322	+	3'UTR	*COL9A2*
6	89663313	89663313	+	89663313	3	3	ENSSSCG00000027352	+	intron	---
6	89664040	89664067	+	89664042	5	28	ENSSSCG00000027352	+	3'UTR	---
6	89699147	89699148	+	89699147	6	10	ENSSSCG00000030438	+	3'UTR	---
6	89700371	89700373	+	89700372	15	29	ENSSSCG00000030438	+	3'UTR	---
6	89705949	89705954	-	89705949	3	6	---	---	intergenic	---
6	89708023	89708023	-	89708023	4	4	XLOC_073972	-	predicted	---
6	89712737	89712744	-	89712744	7	11	ENSSSCG00000027417	-	3'UTR	*LDLRAD4*
6	89728961	89728961	-	89728961	5	5	ENSSSCG00000027417	-	intron	*LDLRAD4*
6	89917876	89917876	-	89917876	3	3	ENSSSCG00000003673	-	3'UTR	*CEP192*
6	90270429	90270429	+	90270429	4	4	XLOC_072400	+	predicted	---
6	90272216	90272222	+	90272222	7	10	XLOC_072400	+	predicted	---
6	90304114	90304121	-	90304120	29	66	ENSSSCG00000021827	-	3'UTR	---
6	90304305	90304305	-	90304305	4	4	ENSSSCG00000021827	-	3'UTR	*ÿ*--
6	90355828	90355828	+	90355828	4	4	XLOC_072401	+	predicted	---
6	90559718	90559718	-	90559718	3	3	XLOC_073978	-	predicted	---
6	90689584	90689584	+	90689584	5	5	ENSSSCG00000022163	+	intron	*GNAL*
6	90691138	90691138	+	90691138	7	7	ENSSSCG00000022163	+	intron	*GNAL*
6	90691834	90691834	+	90691834	3	3	ENSSSCG00000022163	+	intron	*GNAL*
6	90692129	90692131	+	90692130	16	31	ENSSSCG00000022163	+	intron	*GNAL*
6	90795873	90795884	+	90795883	19	47	ENSSSCG00000024644	+	3'UTR	*CIDEA*
6	90814360	90814365	+	90814360	8	22	ENSSSCG00000026022	+	3'UTR	---
6	90816078	90816097	-	90816097	5	12	ENSSSCG00000022947	-	3'UTR	*AFG3L2*
6	90816255	90816263	-	90816255	6	9	ENSSSCG00000022947	-	3'UTR	*AFG3L2*

6	90961329	90961330	+	90961329	3	6	---	---	intergenic	---
6	91056185	91056185	+	91056185	4	4	---	---	intergenic	---
6	91125107	91125113	+	91125113	11	17	XLOC_072411	+	predicted	---
6	91162341	91162341	+	91162341	4	4	---	---	intergenic	---
6	91184112	91184112	-	91184112	15	15	---	---	intergenic	---
6	91263262	91263262	-	91263262	3	3	---	---	intergenic	---
6	91659689	91659697	-	91659691	43	58	XLOC_073984	-	predicted	---
6	91676209	91676209	+	91676209	4	4	---	---	intergenic	---
6	91705546	91705546	-	91705546	3	3	---	---	intergenic	---
6	91705573	91705574	+	91705574	8	13	---	---	intergenic	---
6	91829899	91829899	+	91829899	4	4	ENSSSCG00000003679	+	intron	---
6	91847949	91847959	+	91847959	7	18	ENSSSCG00000022041	+	3'UTR	---
							ENSSSCG00000003679	+	intron	---
6	91848197	91848206	+	91848204	26	49	ENSSSCG00000022041	+	3'UTR	---
							ENSSSCG00000003679	+	intron	---
6	91848272	91848277	+	91848275	23	69	ENSSSCG00000022041	+	3'UTR	---
							ENSSSCG00000003679	+	intron	---
6	91859557	91859557	-	91859557	19	19	XLOC_073987	-	predicted	---
6	91859761	91859761	-	91859761	3	3	XLOC_073987	-	predicted	---
6	91859871	91859871	-	91859871	3	3	XLOC_073987	-	predicted	---
6	91965863	91965863	-	91965863	9	9	XLOC_073988	-	predicted	---
6	91967444	91967444	-	91967444	5	5	XLOC_073988	-	predicted	---
6	92035073	92035073	-	92035073	3	3	XLOC_073989	-	predicted	---
6	92035123	92035123	-	92035123	3	3	XLOC_073989	-	predicted	---
6	92036922	92036922	-	92036922	4	4	ENSSSCG00000003682	-	intron	*ANKRD12*
6	92154654	92154654	-	92154654	5	5	XLOC_073990	-	predicted	---

6	92155771	92155778	-	92155771	11	49	ENSSSCG00000003683	-	3'UTR	*NDUFV2*
6	92172017	92172017	-	92172017	4	4	ENSSSCG00000003683	-	ORF	*NDUFV2*
6	92433647	92433647	-	92433647	4	4	ENSSSCG00000003684	-	intron	*MTCL1*
6	92550303	92550303	+	92550303	5	5	XLOC_072424	+	predicted	---
6	92550398	92550398	+	92550398	5	5	XLOC_072424	+	predicted	---
6	92980855	92980855	-	92980855	3	3	---	---	intergenic	---
6	92990993	92990993	-	92990993	26	26	---	---	intergenic	---
6	93135709	93135709	-	93135709	3	3	---	---	intergenic	---
6	93149390	93149392	+	93149390	20	39	---	---	intergenic	---
6	93315830	93315830	-	93315830	3	3	---	---	intergenic	---
6	93648467	93648477	+	93648474	225	419	XLOC_072428	+	predicted	---
6	93648842	93648842	+	93648842	3	3	XLOC_072428	+	predicted	---
6	94269954	94269954	+	94269954	4	4	---	---	intergenic	---
6	94880510	94880510	+	94880510	4	4	---	---	intergenic	---
6	95058920	95058921	-	95058920	13	17	---	---	intergenic	---
6	95234607	95234607	+	95234607	3	3	---	---	intergenic	---
6	95309749	95309749	-	95309749	7	7	---	---	intergenic	---
6	95761054	95761054	+	95761054	8	8	---	---	intergenic	---
6	95797277	95797277	-	95797277	4	4	---	---	intergenic	---
6	96252971	96252971	-	96252971	8	8	XLOC_074015	-	predicted	---
6	96253035	96253035	-	96253035	4	4	ENSSSCG00000024174	-	3'UTR	*TGIF1*
6	96418184	96418184	-	96418184	7	7	XLOC_074016	-	predicted	---
6	96418230	96418232	-	96418231	64	84	XLOC_074016	-	predicted	---
6	96418298	96418309	-	96418298	31	78	ENSSSCG00000003691	-	3'UTR	---
6	96418547	96418547	-	96418547	3	3	ENSSSCG00000003691	-	ORF	---
6	96440975	96440989	-	96440984	130	292	ENSSSCG00000003692	-	3'UTR	*MYL12B*

6	96441073	96441073	-	96441073	4	4	ENSSSCG00000003692	-	3'UTR	*MYL12B*
6	96620100	96620100	+	96620100	5	5	ENSSSCG00000003693	+	3'UTR	*MYOM1*
6	96819821	96819821	-	96819821	7	7	XLOC_074019	-	predicted	---
6	97060469	97060477	+	97060472	9	22	ENSSSCG00000003697	+	3'UTR	---
6	97070239	97070239	+	97070239	8	8	---	---	intergenic	---
6	97090905	97090905	+	97090905	4	4	ENSSSCG00000030096	+	ORF	---
6	97131628	97131628	-	97131628	3	3	---	---	intergenic	---
6	97458065	97458065	+	97458065	6	6	---	---	intergenic	---
6	97636125	97636125	-	97636125	3	3	XLOC_074022	-	predicted	---
6	97642390	97642391	+	97642390	10	13	---	---	intergenic	---
6	97748808	97748808	+	97748808	5	5	---	---	intergenic	---
6	98014590	98014590	-	98014590	34	34	---	---	intergenic	---
6	98292130	98292130	-	98292130	17	17	---	---	intergenic	---
6	98435131	98435131	+	98435131	3	3	XLOC_072457	+	predicted	---
6	98458666	98458666	+	98458666	4	4	---	---	intergenic	---
6	98670498	98670499	-	98670498	4	8	ENSSSCG00000027498	-	intron	---
6	98710283	98710285	-	98710283	14	18	XLOC_074026	-	predicted	---
6	98710341	98710347	-	98710347	4	7	XLOC_074026	-	predicted	---
6	98752943	98752943	-	98752943	3	3	---	---	intergenic	---
6	99204546	99204555	-	99204546	4	7	XLOC_074033	-	predicted	---
6	99257703	99257703	-	99257703	4	4	ENSSSCG00000003700	-	ORF	*ESCO1*
6	99258431	99258431	-	99258431	3	3	ENSSSCG00000003700	-	ORF	*ESCO1*
6	99283385	99283386	-	99283385	3	6	ENSSSCG00000003701	-	3'UTR	*SNRPD1*
							ENSSSCG00000003700	-	intron	*ESCO1*
6	99283575	99283575	-	99283575	4	4	ENSSSCG00000003701	-	3'UTR	*SNRPD1*
							ENSSSCG00000003700	-	intron	*ESCO1*

6	99283742	99283779	-	99283764	298	1024	ENSSSCG00000003701	-	3'UTR	*SNRPD1*
							ENSSSCG00000003700	-	intron	*ESCO1*
6	99355419	99355419	-	99355419	13	13	---	---	intergenic	---
6	99439754	99439764	-	99439754	6	18	XLOC_074035	-	predicted	---
6	99445956	99445956	-	99445956	5	5	XLOC_074035	-	predicted	---
6	99446560	99446560	-	99446560	8	8	XLOC_074035	-	predicted	---
6	99544365	99544365	-	99544365	3	3	ENSSSCG00000025478	-	intron	*MIB1*
6	99790609	99790609	+	99790609	4	4	ENSSSCG00000003702	+	3'UTR	*GATA6*
6	99790902	99790902	+	99790902	3	3	---	---	intergenic	---
6	99851118	99851118	+	99851118	3	3	---	---	intergenic	---
6	99950811	99950811	-	99950811	4	4	---	---	intergenic	---
6	100099722	100099722	-	100099722	18	18	---	---	intergenic	---
6	100242311	100242311	+	100242311	5	5	---	---	intergenic	---
6	100484606	100484606	+	100484606	3	3	ENSSSCG00000024419	+	intron	---
6	100513995	100513995	+	100513995	5	5	ENSSSCG00000024419	+	intron	---
6	100534609	100534610	+	100534609	16	19	ENSSSCG00000024419	+	3'UTR	---
6	100794324	100794324	+	100794324	5	5	ENSSSCG00000003705	+	3'UTR	*CABLES1*
6	100891984	100891984	+	100891984	4	4	ENSSSCG00000029518	+	intron	*RIOK3*
6	100895084	100895084	+	100895084	3	3	ENSSSCG00000029518	+	ORF	*RIOK3*
6	100921056	100921056	-	100921056	3	3	---	---	intergenic	---
6	100927292	100927294	-	100927294	12	22	XLOC_074047	-	predicted	---
6	100927392	100927392	-	100927392	4	4	XLOC_074047	-	predicted	---
6	101033015	101033015	-	101033015	3	3	ENSSSCG00000003707	-	intron	*NPC1*
6	101060164	101060164	-	101060164	3	3	ENSSSCG00000003708	-	3'UTR	*ANKRD29*
6	101440113	101440113	-	101440113	4	4	ENSSSCG00000003709	-	intron	---
6	101632259	101632260	+	101632259	4	8	ENSSSCG00000003710	+	3'UTR	*TTC39C*

6	101658506	101658506	+	101658506	4	4	ENSSSCG00000003711	+	ORF	*CABYR*
6	101662203	101662203	+	101662203	4	4	ENSSSCG00000003711	+	ORF	*CABYR*
6	101663498	101663523	+	101663518	74	341	XLOC_072478	+	predicted	---
6	101663956	101663964	-	101663957	22	45	ENSSSCG00000003712	-	3'UTR	*OSBPL1A*
6	101665040	101665071	-	101665071	11	36	ENSSSCG00000003712	-	3'UTR	*OSBPL1A*
6	101670138	101670163	-	101670138	5	12	ENSSSCG00000003712	-	intron	*OSBPL1A*
6	101696569	101696569	-	101696569	8	8	ENSSSCG00000003712	-	intron	*OSBPL1A*
6	101724201	101724201	-	101724201	3	3	ENSSSCG00000003712	-	ORF	*OSBPL1A*
6	102037007	102037010	+	102037008	19	28	ENSSSCG00000003713	+	3'UTR	*IMPACT*
6	102037576	102037576	+	102037576	7	7	ENSSSCG00000003713	+	3'UTR	*IMPACT*
6	102039536	102039538	+	102039538	9	16	XLOC_072482	+	predicted	---
6	102374274	102374274	+	102374274	8	8	XLOC_072485	+	predicted	---
6	102791847	102791847	+	102791847	4	4	---	---	intergenic	---
6	102976170	102976172	-	102976170	6	14	---	---	intergenic	---
6	103179628	103179628	+	103179628	3	3	---	---	intergenic	---
6	103427240	103427240	-	103427240	5	5	XLOC_074061	-	predicted	---
6	103673478	103673478	-	103673478	17	17	XLOC_074062	-	predicted	---
6	103674798	103674799	-	103674799	9	12	XLOC_074062	-	predicted	---
6	103674952	103674953	-	103674952	5	8	XLOC_074062	-	predicted	---
6	103675023	103675023	-	103675023	12	12	XLOC_074062	-	predicted	---
6	103748742	103748742	-	103748742	4	4	ENSSSCG00000003716	-	intron	*SS18*
6	103748906	103748906	-	103748906	3	3	ENSSSCG00000003716	-	ORF	*SS18*
6	103790109	103790109	+	103790109	4	4	ENSSSCG00000003717	+	intron	*PSMA8*
6	103799062	103799065	+	103799062	13	18	XLOC_072490	+	predicted	---
6	103799135	103799136	+	103799136	4	7	XLOC_072490	+	predicted	---
6	104007087	104007087	-	104007087	6	6	ENSSSCG00000003719	-	3'UTR	*KCTD1*

6	105041192	105041192	-	105041192	7	7	---	---	intergenic	---
6	105041211	105041212	+	105041212	7	10	---	---	intergenic	---
6	105083735	105083735	-	105083735	3	3	---	---	intergenic	---
6	105111475	105111476	+	105111476	8	14	---	---	intergenic	---
6	105222662	105222671	-	105222664	10	24	XLOC_074068	-	predicted	---
6	105349132	105349144	-	105349132	3	12	ENSSSCG00000003722	-	intron	*CDH2*
6	105532905	105532910	-	105532907	52	111	---	---	intergenic	---
6	105532935	105532937	+	105532936	5	13	---	---	intergenic	---
6	106157227	106157227	-	106157227	3	3	---	---	intergenic	---
6	106284703	106284703	-	106284703	38	38	---	---	intergenic	---
6	106888343	106888343	-	106888343	3	3	---	---	intergenic	---
6	107318422	107318422	-	107318422	9	9	---	---	intergenic	---
6	107358133	107358133	+	107358133	6	6	---	---	intergenic	---
6	107467759	107467759	+	107467759	4	4	---	---	intergenic	---
6	107491762	107491769	+	107491769	4	7	---	---	intergenic	---
6	107655457	107655457	+	107655457	3	3	---	---	intergenic	---
6	107662089	107662089	-	107662089	4	4	XLOC_074078	-	predicted	---
6	107968272	107968272	-	107968272	3	3	---	---	intergenic	---
6	108081926	108081930	+	108081929	19	32	ENSSSCG00000022739	+	3'UTR	*DSG2*
6	108088375	108088375	+	108088375	3	3	XLOC_072505	+	predicted	---
6	108125197	108125206	+	108125197	9	15	ENSSSCG00000023686	+	ORF	*TTR*
6	108125247	108125248	+	108125248	31	37	ENSSSCG00000023686	+	intron	*TTR*
6	108127672	108127721	+	108127672	10	18	ENSSSCG00000023686	+	ORF	*TTR*
6	108131471	108131471	+	108131471	5	5	ENSSSCG00000023686	+	3'UTR	*TTR*
6	108131525	108131572	+	108131565	2318	9575	ENSSSCG00000023686	+	3'UTR	*TTR*
6	108158448	108158448	-	108158448	6	6	ENSSSCG00000025005	-	intron	*B4GALT6*

6	108175613	108175613	-	108175613	5	5	ENSSSCG00000025005	-	intron	*B4GALT6*
6	108310015	108310015	+	108310015	3	3	ENSSSCG00000003727	+	3'UTR	---
6	108497563	108497563	+	108497563	9	9	---	---	intergenic	---
6	108560572	108560572	+	108560572	3	3	ENSSSCG00000003729	+	intron	*RNF125*
6	108562100	108562127	+	108562103	15	21	XLOC_072509	+	predicted	---
6	108562177	108562177	+	108562177	10	10	XLOC_072509	+	predicted	---
6	108572373	108572373	-	108572373	5	5	---	---	intergenic	---
6	108630916	108630920	+	108630918	79	164	ENSSSCG00000003730	+	3'UTR	*RNF138*
6	108630959	108630969	+	108630960	8	21	ENSSSCG00000003730	+	3'UTR	*RNF138*
6	108632319	108632325	+	108632319	3	6	ENSSSCG00000003730	+	3'UTR	*RNF138*
6	108633052	108633053	+	108633052	3	6	ENSSSCG00000003730	+	3'UTR	*RNF138*
6	108659752	108659752	-	108659752	5	5	---	---	intergenic	---
6	108762791	108762791	-	108762791	3	3	---	---	intergenic	---
6	109197187	109197187	-	109197187	3	3	---	---	intergenic	---
6	109294633	109294633	-	109294633	6	6	ENSSSCG00000003733	-	intron	*KLHL14*
6	109360435	109360435	-	109360435	5	5	---	---	intergenic	---
6	109411359	109411360	-	109411359	8	12	---	---	intergenic	---
6	109474484	109474484	-	109474484	4	4	---	---	intergenic	---
6	109747226	109747226	+	109747226	12	12	---	---	intergenic	---
6	109865860	109865860	+	109865860	3	3	ENSSSCG00000003736	+	ORF	*ASXL3*
6	110242824	110242824	-	110242824	7	7	---	---	intergenic	---
6	110852870	110852870	-	110852870	5	5	ENSSSCG00000028047	-	intron	*DTNA*
6	110868339	110868342	-	110868342	8	11	---	---	intergenic	---
6	111016020	111016020	-	111016020	6	6	---	---	intergenic	---
6	111039665	111039665	-	111039665	6	6	---	---	intergenic	---
6	111114005	111114005	-	111114005	5	5	---	---	intergenic	---

6	111158957	111158957	-	111158957	3	3	---	---	intergenic	---
6	111263246	111263246	+	111263246	4	4	---	---	intergenic	---
6	111357726	111357726	+	111357726	3	3	ENSSSCG00000003738	+	intron	*MAPRE2*
6	111392122	111392124	-	111392122	7	13	---	---	intergenic	---
6	111395215	111395215	-	111395215	3	3	ENSSSCG00000003741	-	ORF	---
6	111535463	111535465	-	111535465	10	17	XLOC_074098	-	predicted	---
6	111615237	111615242	-	111615242	7	18	XLOC_074099	-	predicted	---
6	111615368	111615368	+	111615368	3	3	---	---	intergenic	---
6	111619589	111619589	-	111619589	3	3	XLOC_074099	-	predicted	---
6	111619662	111619662	-	111619662	3	3	XLOC_074099	-	predicted	---
6	111626014	111626014	+	111626014	6	6	---	---	intergenic	---
6	111646316	111646317	-	111646316	6	9	ENSSSCG00000030018	-	3'UTR	*ZNF396*
6	111721283	111721284	-	111721284	7	13	ENSSSCG00000003742	-	3'UTR	*INO80C*
6	112008740	112008740	+	112008740	7	7	ENSSSCG00000003743	+	3'UTR	*GALNT1*
							ENSSSCG00000003743	+	ORF	*GALNT1*
6	112008952	112008955	+	112008952	13	17	XLOC_072528	+	predicted	---
6	112115534	112115534	+	112115534	3	3	XLOC_072529	+	predicted	---
6	112202406	112202408	+	112202407	9	15	---	---	intergenic	---
6	112271303	112271347	+	112271345	55	209	ENSSSCG00000030002	+	3'UTR	---
6	112271512	112271512	+	112271512	3	3	---	---	intergenic	---
6	112288594	112288594	-	112288594	3	3	ENSSSCG00000027700	-	3'UTR	*RPRD1A*
6	112290643	112290643	-	112290643	4	4	ENSSSCG00000027700	-	3'UTR	*RPRD1A*
6	112302929	112302929	-	112302929	4	4	ENSSSCG00000027700	-	intron	*RPRD1A*
6	112320290	112320290	-	112320290	3	3	ENSSSCG00000027700	-	intron	*RPRD1A*
6	112352185	112352185	-	112352185	3	3	ENSSSCG00000027700	-	intron	*RPRD1A*
6	112366953	112366953	-	112366953	6	6	---	---	intergenic	---

6	112458300	112458302	+	112458300	4	8	ENSSSCG00000020898	+	3'UTR	*ELP2*
6	113055101	113055101	+	113055101	3	3	ENSSSCG00000003746	+	intron	*RPSA*
							ENSSSCG00000027935	+	intron	*FHOD3*
6	113225108	113225109	+	113225108	4	7	ENSSSCG00000027935	+	3'UTR	*FHOD3*
6	113245020	113245025	-	113245023	4	14	ENSSSCG00000026953	-	3'UTR	*TPGS2*
6	113806698	113806698	+	113806698	8	8	ENSSSCG00000003747	+	3'UTR	---
6	113838455	113838455	-	113838455	3	3	XLOC_074113	-	predicted	---
6	113933489	113933490	-	113933489	21	31	---	---	intergenic	---
6	114295354	114295354	-	114295354	7	7	---	---	intergenic	---
6	114577898	114577899	-	114577898	22	30	---	---	intergenic	---
6	114917562	114917562	-	114917562	3	3	---	---	intergenic	---
6	115495896	115495896	+	115495896	9	9	---	---	intergenic	---
6	115512556	115512556	-	115512556	3	3	---	---	intergenic	---
6	115619675	115619675	-	115619675	4	4	---	---	intergenic	---
6	115647202	115647202	+	115647202	3	3	---	---	intergenic	---
6	115847188	115847188	+	115847188	5	5	---	---	intergenic	---
6	116038483	116038483	+	116038483	4	4	---	---	intergenic	---
6	116516323	116516324	-	116516323	9	13	---	---	intergenic	---
6	116544613	116544613	+	116544613	15	15	---	---	intergenic	---
6	116860917	116860917	-	116860917	5	5	XLOC_074116	-	predicted	---
6	117161547	117161547	-	117161547	6	6	---	---	intergenic	---
6	117211758	117211758	-	117211758	8	8	---	---	intergenic	---
6	117755086	117755086	-	117755086	151	151	---	---	intergenic	---
6	117755123	117755123	+	117755123	3	3	---	---	intergenic	---
6	117779431	117779431	-	117779431	3	3	---	---	intergenic	---
6	117779462	117779470	+	117779465	17	79	---	---	intergenic	---

6	118056720	118056720	+	118056720	5	5	ENSSSCG00000003749	+	intron	*PIK3C3*
6	118097707	118097708	+	118097707	12	16	ENSSSCG00000003749	+	3'UTR	*PIK3C3*
6	118855465	118855465	-	118855465	3	3	---	---	intergenic	---
6	118855535	118855536	+	118855535	7	10	---	---	intergenic	---
6	118856530	118856530	-	118856530	7	7	---	---	intergenic	---
6	119079430	119079473	+	119079438	19	66	ENSSSCG00000003753	+	3'UTR	---
6	119204700	119204700	+	119204700	3	3	XLOC_072551	+	predicted	---
6	119273132	119273133	+	119273132	6	11	ENSSSCG00000003755	+	3'UTR	*MCOLN2*
6	119534527	119534527	-	119534527	8	8	XLOC_074120	-	predicted	---
6	119537634	119537643	-	119537642	30	50	XLOC_074120	-	predicted	---
6	119557452	119557452	-	119557452	3	3	ENSSSCG00000003757	-	ORF	*SSX2IP*
6	119559784	119559784	-	119559784	7	7	ENSSSCG00000003757	-	intron	*SSX2IP*
6	119566461	119566463	-	119566461	6	10	ENSSSCG00000003757	-	ORF	*SSX2IP*
6	119738262	119738262	-	119738262	5	5	---	---	intergenic	---
6	119810702	119810702	+	119810702	3	3	---	---	intergenic	---
6	119812983	119812983	+	119812983	8	8	---	---	intergenic	---
6	120009225	120009225	-	120009225	4	4	ENSSSCG00000003760	-	intron	*TTLL7*
6	120778471	120778472	+	120778471	8	12	---	---	intergenic	---
6	121087546	121087546	-	121087546	3	3	---	---	intergenic	---
6	121415842	121415850	+	121415842	20	31	---	---	intergenic	---
6	121454888	121454896	-	121454896	7	11	---	---	intergenic	---
6	121948549	121948550	-	121948549	3	6	---	---	intergenic	---
6	121953590	121953594	-	121953594	11	15	ENSSSCG00000003761	-	3'UTR	*ADGRL2*
6	121954198	121954198	-	121954198	3	3	ENSSSCG00000003761	-	3'UTR	*ADGRL2*
6	122156611	122156611	+	122156611	3	3	---	---	intergenic	---
6	122453067	122453067	+	122453067	4	4	---	---	intergenic	---

6	122952293	122952293	-	122952293	4	4	---	---	intergenic	---
6	123448192	123448192	-	123448192	3	3	---	---	intergenic	---
6	123448228	123448229	+	123448228	3	6	---	---	intergenic	---
6	123747444	123747444	+	123747444	3	3	---	---	intergenic	---
6	124088279	124088281	-	124088280	4	10	---	---	intergenic	---
6	124225565	124225565	+	124225565	4	4	---	---	intergenic	---
6	124326563	124326563	-	124326563	3	3	---	---	intergenic	---
6	124326587	124326601	+	124326593	38	136	---	---	intergenic	---
6	124620440	124620446	+	124620440	14	22	XLOC_072569	+	predicted	---
6	124790066	124790066	-	124790066	10	10	ENSSSCG00000003763	-	3'UTR	*IFI44*
6	124897344	124897344	-	124897344	5	5	ENSSSCG00000003764	-	intron	*PTGFR*
6	125291386	125291386	-	125291386	8	8	---	---	intergenic	---
6	125425679	125425679	-	125425679	10	10	ENSSSCG00000027873	-	intron	*GIPC2*
6	125425781	125425781	-	125425781	7	7	ENSSSCG00000003766	-	3'UTR	*DNAJB4*
							ENSSSCG00000027873	-	intron	*GIPC2*
6	125426683	125426688	-	125426685	22	48	ENSSSCG00000003766	-	3'UTR	*DNAJB4*
							ENSSSCG00000027873	-	intron	*GIPC2*
6	125495431	125495433	+	125495431	11	18	ENSSSCG00000003771	+	intron	*FUBP1*
6	125496155	125496157	+	125496155	15	26	XLOC_072573	+	predicted	---
6	125693789	125693792	-	125693790	47	56	ENSSSCG00000003768	-	intron	*NEXN*
6	125799609	125799619	+	125799619	4	10	XLOC_072574	+	predicted	---
6	125945357	125945357	+	125945357	6	6	ENSSSCG00000003772	+	3'UTR	*ZZZ3*
6	126369626	126369626	+	126369626	4	4	---	---	intergenic	---
6	126397174	126397174	+	126397174	3	3	---	---	intergenic	---
6	126482300	126482300	-	126482300	3	3	---	---	intergenic	---
6	126998382	126998382	-	126998382	3	3	---	---	intergenic	---

6	127105613	127105614	-	127105614	9	14	XLOC_074145	-	predicted	---
6	127370050	127370051	+	127370051	4	7	XLOC_072584	+	predicted	---
6	127373017	127373017	+	127373017	5	5	XLOC_072584	+	predicted	---
6	127373104	127373107	+	127373105	125	150	XLOC_072584	+	predicted	---
6	127452479	127452479	-	127452479	3	3	ENSSSCG00000003774	-	intron	*MSH4*
6	127497453	127497467	-	127497454	19	34	XLOC_074148	-	predicted	---
6	127504092	127504092	-	127504092	16	16	ENSSSCG00000003775	-	intron	*RABGGTB*
							ENSSSCG00000019699	-	ORF	*SNORD45*
6	127513767	127513781	-	127513779	31	98	ENSSSCG00000003776	-	3'UTR	*ACADM*
6	127513871	127513884	-	127513882	72	135	ENSSSCG00000003776	-	3'UTR	*ACADM*
6	127514209	127514228	-	127514228	5	8	ENSSSCG00000003776	-	3'UTR	*ACADM*
6	127514374	127514374	-	127514374	5	5	ENSSSCG00000003776	-	3'UTR	*ACADM*
6	127528162	127528162	-	127528162	3	3	ENSSSCG00000003776	-	ORF	*ACADM*
6	127528842	127528842	-	127528842	4	4	ENSSSCG00000003776	-	ORF	*ACADM*
6	127530513	127530513	-	127530513	7	7	ENSSSCG00000003776	-	ORF	*ACADM*
6	127534139	127534139	-	127534139	8	8	ENSSSCG00000003776	-	ORF	*ACADM*
6	127548587	127548587	+	127548587	5	5	---	---	intergenic	---
6	127693598	127693602	+	127693598	11	15	ENSSSCG00000003777	+	intron	*SLC44A5*
6	127973701	127973701	+	127973701	6	6	XLOC_072586	+	predicted	---
6	128090054	128090054	-	128090054	3	3	---	---	intergenic	---
6	128262574	128262574	+	128262574	3	3	---	---	intergenic	---
6	128286037	128286037	-	128286037	3	3	XLOC_074153	-	predicted	---
6	128326628	128326628	+	128326628	4	4	ENSSSCG00000003780	+	ORF	*CRYZ*
6	128327058	128327058	+	128327058	11	11	ENSSSCG00000003780	+	3'UTR	*CRYZ*
6	128327110	128327110	+	128327110	8	8	ENSSSCG00000003780	+	3'UTR	*CRYZ*
6	128327348	128327358	+	128327351	83	138	ENSSSCG00000003780	+	3'UTR	*CRYZ*

6	128327405	128327420	+	128327411	34	69	ENSSSCG00000003780	+	3'UTR	*CRYZ*
6	128460819	128460821	+	128460820	10	25	ENSSSCG00000003781	+	intron	*ERICH3*
6	128714262	128714262	-	128714262	6	6	XLOC_074159	-	predicted	---
6	128766238	128766241	+	128766240	10	20	XLOC_072593	+	predicted	---
6	128898512	128898512	+	128898512	3	3	XLOC_072593	+	predicted	---
6	129260918	129260918	-	129260918	4	4	---	---	intergenic	---
6	129898795	129898813	-	129898804	11	37	---	---	intergenic	---
6	129943475	129943497	+	129943487	154	323	---	---	intergenic	---
6	131062597	131062598	+	131062597	21	24	---	---	intergenic	---
6	131153948	131153948	-	131153948	6	6	---	---	intergenic	---
6	131232945	131232945	-	131232945	3	3	---	---	intergenic	---
6	131398995	131398995	-	131398995	4	4	---	---	intergenic	---
6	131528945	131528945	+	131528945	4	4	XLOC_072597	+	predicted	---
6	131529346	131529348	+	131529347	7	13	XLOC_072597	+	predicted	---
6	131533112	131533124	+	131533113	8	19	XLOC_072597	+	predicted	---
6	131554565	131554569	+	131554569	73	94	ENSSSCG00000003787	+	intron	*ZRANB2*
6	131562692	131562692	+	131562692	4	4	ENSSSCG00000003787	+	3'UTR	*ZRANB2*
6	131562905	131562905	+	131562905	3	3	ENSSSCG00000003787	+	3'UTR	*ZRANB2*
6	131563359	131563407	+	131563407	10	27	ENSSSCG00000003787	+	3'UTR	*ZRANB2*
6	131722271	131722271	-	131722271	3	3	---	---	intergenic	---
6	132021090	132021090	+	132021090	19	19	---	---	intergenic	---
6	132032328	132032328	-	132032328	3	3	ENSSSCG00000003789	-	ORF	*CTH*
6	132034260	132034260	-	132034260	4	4	ENSSSCG00000003789	-	ORF	*CTH*
6	132041290	132041290	-	132041290	3	3	ENSSSCG00000003789	-	intron	*CTH*
6	132050087	132050087	-	132050087	9	9	ENSSSCG00000003789	-	ORF	*CTH*
6	132187043	132187043	+	132187043	3	3	ENSSSCG00000003790	+	3'UTR	*ANKRD13C*

6	132192645	132192645	-	132192645	5	5	XLOC_074168	-	predicted	---
6	132193671	132193693	-	132193687	13	36	ENSSSCG00000003791	-	3'UTR	*SFRS11*
6	132194430	132194430	-	132194430	5	5	ENSSSCG00000003791	-	3'UTR	*SFRS11*
6	132537758	132537759	+	132537758	3	6	---	---	intergenic	---
6	132619329	132619329	+	132619329	3	3	---	---	intergenic	---
6	132862588	132862588	-	132862588	3	3	---	---	intergenic	---
6	133667572	133667572	-	133667572	4	4	---	---	intergenic	---
6	133750727	133750728	-	133750727	4	7	---	---	intergenic	---
6	133818850	133818850	+	133818850	6	6	ENSSSCG00000003795	+	3'UTR	*GPR177*
6	133867484	133867484	+	133867484	10	10	ENSSSCG00000003797	+	3'UTR	*DIRAS3*
							ENSSSCG00000003797	+	ORF	---
6	134060356	134060360	+	134060356	5	13	---	---	intergenic	---
6	134069420	134069420	+	134069420	4	4	ENSSSCG00000003798	+	intron	*SERBP1*
6	134073968	134073968	+	134073968	3	3	ENSSSCG00000003798	+	ORF	*SERBP1*
6	134074652	134074652	+	134074652	3	3	ENSSSCG00000003798	+	ORF	*SERBP1*
6	134074692	134074702	+	134074702	15	18	ENSSSCG00000003798	+	intron	*SERBP1*
6	134082225	134082237	+	134082236	19	70	XLOC_072608	+	predicted	---
6	134082423	134082423	+	134082423	14	14	XLOC_072608	+	predicted	---
6	134082610	134082610	+	134082610	3	3	XLOC_072608	+	predicted	---
6	134084044	134084046	+	134084044	4	8	XLOC_072608	+	predicted	---
6	134246586	134246586	-	134246586	22	22	ENSSSCG00000003801	-	intron	*IL23RA*
6	134390569	134390569	-	134390569	3	3	---	---	intergenic	---
6	134394099	134394111	-	134394099	7	16	---	---	intergenic	---
6	134394151	134394151	+	134394151	3	3	---	---	intergenic	---
6	134402064	134402064	-	134402064	3	3	---	---	intergenic	---
6	134409979	134409988	-	134409980	6	19	XLOC_074178	-	predicted	---

6	134410700	134410700	-	134410700	7	7	---	---	intergenic	---
6	134464756	134464764	-	134464756	7	10	ENSSSCG00000003803	-	ORF	*C1orf141*
6	134468473	134468475	+	134468475	7	11	XLOC_072611	+	predicted	---
6	134473041	134473042	+	134473041	17	21	XLOC_072611	+	predicted	---
6	134486898	134486898	-	134486898	4	4	ENSSSCG00000030423	-	3'UTR	*MIER1*
6	134553381	134553381	-	134553381	5	5	---	---	intergenic	---
6	134722544	134722544	-	134722544	3	3	---	---	intergenic	---
6	134913462	134913463	+	134913462	10	14	ENSSSCG00000003805	+	3'UTR	*PDE4B*
6	134915008	134915008	+	134915008	7	7	XLOC_072614	+	predicted	---
6	135379848	135379854	-	135379848	11	35	ENSSSCG00000003806	-	3'UTR	*LEPROT*
6	135582861	135582862	-	135582861	6	9	XLOC_074190	-	predicted	---
6	135607758	135607772	-	135607768	12	55	ENSSSCG00000003808	-	intron	*AK3L1*
6	135652246	135652246	-	135652246	5	5	ENSSSCG00000003808	-	intron	*AK3L1*
6	135731686	135731686	-	135731686	3	3	XLOC_074197	-	predicted	---
6	135736668	135736668	+	135736668	5	5	XLOC_072620	+	predicted	---
6	135799798	135799799	+	135799798	5	9	XLOC_072620	+	predicted	---
6	135899424	135899424	-	135899424	7	7	ENSSSCG00000003809	-	ORF	---
6	136109385	136109385	-	136109385	3	3	---	---	intergenic	---
6	136570393	136570393	+	136570393	3	3	---	---	intergenic	---
6	136685263	136685265	-	136685265	4	7	XLOC_074203	-	predicted	---
6	136685350	136685357	-	136685350	28	54	XLOC_074203	-	predicted	---
6	137171267	137171274	-	137171271	198	335	ENSSSCG00000003812	-	3'UTR	*PGM1*
6	137230530	137230533	-	137230533	4	7	ENSSSCG00000003812	-	intron	*PGM1*
6	137320243	137320243	-	137320243	5	5	---	---	intergenic	---
6	137325225	137325225	+	137325225	9	9	XLOC_072629	+	predicted	---
6	137326107	137326107	+	137326107	13	13	---	---	intergenic	---

6	137326170	137326170	+	137326170	3	3	---	---	intergenic	---
6	137326284	137326294	+	137326292	36	115	---	---	intergenic	---
6	137327089	137327089	+	137327089	6	6	---	---	intergenic	---
6	137354139	137354139	+	137354139	3	3	---	---	intergenic	---
6	137377949	137377949	-	137377949	6	6	ENSSSCG00000003815	-	3'UTR	*ALG6*
6	137531517	137531517	-	137531517	3	3	---	---	intergenic	---
6	137704914	137704914	-	137704914	3	3	---	---	intergenic	---
6	138006959	138006964	+	138006964	4	7	---	---	intergenic	---
6	138010881	138010882	+	138010881	28	32	---	---	intergenic	---
6	138100825	138100841	-	138100840	37	59	XLOC_074215	-	predicted	---
6	138101163	138101163	-	138101163	3	3	XLOC_074215	-	predicted	---
6	138101373	138101373	-	138101373	5	5	XLOC_074215	-	predicted	---
6	138101479	138101479	-	138101479	10	10	XLOC_074215	-	predicted	---
6	138101734	138101741	-	138101740	23	44	ENSSSCG00000003819	-	3'UTR	*ANGPTL3*
6	138101783	138101783	-	138101783	3	3	ENSSSCG00000003819	-	ORF	*ANGPTL3*
6	138110916	138110916	-	138110916	3	3	ENSSSCG00000003819	-	ORF	*ANGPTL3*
6	138314405	138314405	+	138314405	4	4	ENSSSCG00000003821	+	intron	*KANK4*
6	138477216	138477216	+	138477216	3	3	---	---	intergenic	---
6	138484757	138484757	-	138484757	7	7	---	---	intergenic	---
6	138600904	138600907	-	138600906	67	115	XLOC_074218	-	predicted	---
6	138764560	138764565	-	138764564	12	29	ENSSSCG00000023243	-	3'UTR	---
6	138769234	138769234	-	138769234	8	8	ENSSSCG00000023243	-	intron	---
6	138769449	138769449	-	138769449	3	3	ENSSSCG00000023243	-	intron	---
6	138770088	138770088	-	138770088	3	3	ENSSSCG00000023243	-	intron	---
6	138770515	138770515	-	138770515	3	3	ENSSSCG00000023243	-	intron	---
6	138770983	138770983	-	138770983	5	5	ENSSSCG00000023243	-	intron	---

6	138995010	138995022	+	138995012	7	27	XLOC_072639	+	predicted	---
6	138995173	138995173	+	138995173	3	3	XLOC_072639	+	predicted	---
6	139129401	139129401	-	139129401	3	3	---	---	intergenic	---
6	139131725	139131732	-	139131731	4	10	---	---	intergenic	---
6	139157706	139157706	-	139157706	5	5	---	---	intergenic	---
6	139176378	139176378	+	139176378	5	5	---	---	intergenic	---
6	139663734	139663734	-	139663734	3	3	XLOC_074220	-	predicted	---
6	139769871	139769902	-	139769900	10	36	XLOC_074220	-	predicted	---
6	139902195	139902195	-	139902195	6	6	ENSSSCG00000021940	-	3'UTR	*CYP2J34*
6	139936095	139936095	-	139936095	3	3	ENSSSCG00000003825	-	3'UTR	*CYP2J2*
6	140188764	140188780	+	140188765	7	26	ENSSSCG00000003826	+	3'UTR	*HOOK1*
6	140188972	140188972	+	140188972	3	3	ENSSSCG00000003826	+	3'UTR	*HOOK1*
6	140189011	140189011	+	140189011	4	4	ENSSSCG00000003826	+	3'UTR	*HOOK1*
6	140190892	140190892	+	140190892	3	3	ENSSSCG00000003826	+	3'UTR	*HOOK1*
6	140192069	140192071	+	140192070	6	12	XLOC_072644	+	predicted	---
6	140336197	140336199	+	140336198	11	17	---	---	intergenic	---
6	141232389	141232389	+	141232389	3	3	ENSSSCG00000030839	+	3'UTR	*C-JUN*
							ENSSSCG00000030839	+	ORF	*havana*
6	141232895	141232895	+	141232895	6	6	ENSSSCG00000030839	+	3'UTR	*C-JUN*
							ENSSSCG00000030839	+	ORF	*havana*
6	141233061	141233069	+	141233069	4	7	ENSSSCG00000030839	+	3'UTR	*C-JUN*
							ENSSSCG00000030839	+	ORF	*havana*
6	141233592	141233592	+	141233592	6	6	ENSSSCG00000030839	+	3'UTR	*C-JUN*
							ENSSSCG00000030839	+	ORF	*havana*
6	141389544	141389544	+	141389544	7	7	ENSSSCG00000003830	+	3'UTR	*MYSM1*
6	141409459	141409459	+	141409459	3	3	---	---	intergenic	---

6	141544092	141544092	+	141544092	3	3	ENSSSCG00000003831	+	intron	*OMA1*
6	142456339	142456344	+	142456339	3	9	---	---	intergenic	---
6	142497155	142497155	+	142497155	5	5	XLOC_072655	+	predicted	---
6	142604141	142604142	+	142604141	5	8	---	---	intergenic	---
6	142981953	142981953	+	142981953	4	4	ENSSSCG00000003833	+	intron	*DAB1*
6	143127212	143127215	+	143127212	5	8	ENSSSCG00000003833	+	3'UTR	*DAB1*
6	143172531	143172531	+	143172531	4	4	ENSSSCG00000003834	+	ORF	*C8B*
6	143182166	143182191	+	143182187	76	82	ENSSSCG00000003834	+	intron	*C8B*
6	143185960	143185960	+	143185960	6	6	ENSSSCG00000003834	+	ORF	*C8B*
6	143202838	143202844	+	143202838	4	11	ENSSSCG00000003834	+	ORF	*C8B*
6	143205411	143205439	+	143205437	80	243	ENSSSCG00000003834	+	3'UTR	*C8B*
6	143205848	143205861	+	143205852	99	241	ENSSSCG00000003834	+	3'UTR	*C8B*
6	143206160	143206160	+	143206160	6	6	XLOC_072658	+	predicted	---
6	143207851	143207851	+	143207851	3	3	XLOC_072658	+	predicted	---
6	143217677	143217692	-	143217689	71	251	ENSSSCG00000003835	-	3'UTR	*C8A*
6	143251432	143251440	-	143251438	4	11	ENSSSCG00000003835	-	intron	*C8A*
6	143251644	143251644	-	143251644	7	7	ENSSSCG00000003835	-	ORF	*C8A*
6	143251792	143251792	-	143251792	4	4	ENSSSCG00000003835	-	ORF	*C8A*
6	143732348	143732348	+	143732348	3	3	ENSSSCG00000003839	+	3'UTR	*PPAP2B*
6	143734077	143734081	+	143734078	28	42	XLOC_072663	+	predicted	---
6	143766189	143766192	+	143766190	9	23	XLOC_072664	+	predicted	---
6	143806864	143806864	+	143806864	11	11	---	---	intergenic	---
6	143830863	143830868	+	143830863	20	26	XLOC_072666	+	predicted	---
6	144991780	144991780	-	144991780	3	3	---	---	intergenic	---
6	145016814	145016821	-	145016814	8	23	---	---	intergenic	---
6	145016837	145016837	+	145016837	18	18	---	---	intergenic	---

6	145213315	145213315	-	145213315	12	12	---	---	intergenic	---
6	145448929	145448930	+	145448930	10	13	ENSSSCG00000029633	+	3'UTR	*USP24*
6	145450652	145450652	+	145450652	6	6	XLOC_072670	+	predicted	---
6	145451983	145451987	-	145451984	37	73	ENSSSCG00000025020	-	3'UTR	*PCSK9*
6	145580393	145580407	+	145580407	7	10	ENSSSCG00000027525	+	3'UTR	*DHCR24*
6	145582371	145582371	+	145582371	5	5	ENSSSCG00000027525	+	3'UTR	*DHCR24*
6	145582941	145582951	+	145582944	78	219	ENSSSCG00000027525	+	3'UTR	*DHCR24*
6	145658867	145658867	+	145658867	3	3	XLOC_072676	+	predicted	---
6	145680006	145680013	-	145680006	12	19	ENSSSCG00000003844	-	3'UTR	*TTC4*
6	145684751	145684751	-	145684751	4	4	ENSSSCG00000003844	-	intron	*TTC4*
6	145713721	145713721	-	145713721	13	13	ENSSSCG00000003844	-	intron	*TTC4*
6	145718926	145718926	+	145718926	3	3	---	---	intergenic	---
6	145721069	145721075	-	145721071	46	71	XLOC_074248	-	predicted	---
6	145757408	145757414	-	145757408	6	9	ENSSSCG00000003845	-	ORF	---
6	145879057	145879059	+	145879057	10	18	ENSSSCG00000029698	+	3'UTR	*LRRC42*
6	145895427	145895427	-	145895427	3	3	ENSSSCG00000027641	-	intron	*LDLRAD1*
6	145904806	145904817	-	145904816	21	46	XLOC_074250	-	predicted	---
6	145909227	145909246	-	145909243	212	377	ENSSSCG00000022494	-	3'UTR	*TMEM59*
6	145909576	145909607	-	145909584	60	209	ENSSSCG00000022494	-	3'UTR	*TMEM59*
6	145921065	145921065	-	145921065	12	12	ENSSSCG00000022494	-	ORF	*TMEM59*
6	145941930	145941930	+	145941930	3	3	ENSSSCG00000021216	+	intron	*TCEANC2*
6	145973615	145973615	+	145973615	4	4	ENSSSCG00000021216	+	3'UTR	*TCEANC2*
6	145974306	145974306	+	145974306	3	3	ENSSSCG00000021216	+	3'UTR	*TCEANC2*
6	146028239	146028239	-	146028239	5	5	---	---	intergenic	---
6	146066632	146066636	+	146066635	13	21	XLOC_072682	+	predicted	---
6	146076208	146076212	-	146076210	19	38	ENSSSCG00000028896	-	3'UTR	*DIO1*

6	146116818	146116818	+	146116818	5	5	ENSSSCG00000028506	+	intron	*YIPF1*
6	146147113	146147123	+	146147121	16	53	ENSSSCG00000028506	+	3'UTR	*YIPF1*
6	146214096	146214098	+	146214097	11	17	ENSSSCG00000028748	+	3'UTR	*NDC1*
6	146309922	146309922	-	146309922	3	3	---	---	intergenic	---
6	146523209	146523210	+	146523210	10	14	XLOC_072688	+	predicted	---
6	146603532	146603536	+	146603533	10	26	ENSSSCG00000003848	+	intron	*APOER2*
6	146662881	146662892	+	146662892	8	17	ENSSSCG00000003849	+	3'UTR	*MAGOH*
6	146682165	146682173	+	146682168	279	680	ENSSSCG00000003850	+	3'UTR	*C1orf123*
6	146701781	146701782	-	146701782	4	7	XLOC_074260	-	predicted	---
6	146702080	146702085	-	146702082	28	69	ENSSSCG00000003851	-	3'UTR	*CPT2*
6	146761671	146761671	-	146761671	6	6	ENSSSCG00000003851	-	intron	*CPT2*
							ENSSSCG00000003851	-	ORF	*CPT2*
6	146853429	146853429	+	146853429	9	9	ENSSSCG00000003852	+	intron	*SLC1A7*
6	146867395	146867395	-	146867395	4	4	ENSSSCG00000027114	-	3'UTR	*PODN*
6	146902350	146902357	-	146902356	174	263	ENSSSCG00000027114	-	intron	*PODN*
6	146902409	146902410	-	146902410	10	18	ENSSSCG00000027114	-	intron	*PODN*
6	146902506	146902506	-	146902506	4	4	ENSSSCG00000027114	-	intron	*PODN*
6	146902545	146902554	-	146902552	15	51	ENSSSCG00000027114	-	intron	*PODN*
6	146902803	146902803	-	146902803	3	3	ENSSSCG00000027114	-	intron	*PODN*
6	146941410	146941410	-	146941410	7	7	---	---	intergenic	---
6	146991290	146991290	-	146991290	4	4	---	---	intergenic	---
6	147045887	147045887	+	147045887	6	6	ENSSSCG00000003853	+	ORF	*SCP2*
6	147313541	147313549	+	147313542	9	31	XLOC_072699	+	predicted	---
6	147329126	147329131	-	147329126	5	9	ENSSSCG00000003859	-	intron	*ZCCHC11*
6	147432746	147432747	-	147432747	17	23	ENSSSCG00000003859	-	intron	*ZCCHC11*
6	147433788	147433788	-	147433788	3	3	ENSSSCG00000003859	-	intron	*ZCCHC11*

6	147485081	147485081	+	147485081	4	4	ENSSSCG00000003861	+	ORF	---
6	147512140	147512145	+	147512143	19	69	ENSSSCG00000003861	+	3'UTR	---
6	147581555	147581555	+	147581555	3	3	ENSSSCG00000003863	+	ORF	*ZFYVE9*
6	147697959	147697959	-	147697959	10	10	ENSSSCG00000022425	-	3'UTR	---
6	147698356	147698358	-	147698358	5	8	ENSSSCG00000022425	-	3'UTR	---
6	147754751	147754757	+	147754756	9	15	XLOC_072706	+	predicted	---
6	147754861	147754861	+	147754861	4	4	XLOC_072706	+	predicted	---
6	147755017	147755026	+	147755023	17	57	XLOC_072706	+	predicted	---
6	147756264	147756264	+	147756264	3	3	XLOC_072706	+	predicted	---
6	147757637	147757640	+	147757640	5	12	XLOC_072706	+	predicted	---
6	147860339	147860342	+	147860339	8	24	ENSSSCG00000003867	+	intron	*TXNDC12*
6	147870957	147870967	+	147870959	67	112	ENSSSCG00000003867	+	3'UTR	*TXNDC12*
6	148001406	148001406	-	148001406	5	5	---	---	intergenic	---
6	148091115	148091120	+	148091118	121	228	ENSSSCG00000003870	+	3'UTR	*NRD1*
6	148091486	148091495	+	148091486	10	16	ENSSSCG00000003870	+	3'UTR	*NRD1*
6	148091597	148091600	+	148091600	9	13	ENSSSCG00000003870	+	3'UTR	*NRD1*
6	148091809	148091813	-	148091810	12	20	ENSSSCG00000021292	-	3'UTR	*OSBPL9*
6	148091874	148091874	-	148091874	3	3	ENSSSCG00000021292	-	3'UTR	*OSBPL9*
6	148092132	148092132	-	148092132	3	3	ENSSSCG00000021292	-	3'UTR	*OSBPL9*
6	148092311	148092313	-	148092312	17	26	ENSSSCG00000021292	-	3'UTR	*OSBPL9*
6	148095782	148095782	-	148095782	3	3	ENSSSCG00000021292	-	intron	*OSBPL9*
							ENSSSCG00000021292	-	ORF	*OSBPL9*
6	148422838	148422838	+	148422838	4	4	ENSSSCG00000003872	+	intron	*EPS15*
6	148454723	148454723	+	148454723	3	3	ENSSSCG00000003872	+	intron	*EPS15*
6	148492597	148492598	+	148492597	7	10	ENSSSCG00000003872	+	3'UTR	*EPS15*
6	148494389	148494389	+	148494389	8	8	ENSSSCG00000003872	+	3'UTR	*EPS15*

6	148578439	148578450	+	148578443	29	48	XLOC_072716	+	predicted	---
6	148578757	148578785	+	148578757	11	35	XLOC_072716	+	predicted	---
6	148609467	148609512	-	148609498	15	42	XLOC_074277	-	predicted	---
6	148609568	148609574	-	148609574	12	35	XLOC_074277	-	predicted	---
6	148749841	148749850	+	148749842	14	30	ENSSSCG00000003876	+	3'UTR	*CDKN2C*
6	148750264	148750264	+	148750264	3	3	ENSSSCG00000003876	+	3'UTR	*CDKN2C*
6	149146975	149146975	+	149146975	5	5	ENSSSCG00000003877	+	intron	*FAF1*
6	149304982	149304983	+	149304983	8	12	ENSSSCG00000003877	+	3'UTR	*FAF1*
6	149306841	149306842	+	149306842	5	9	ENSSSCG00000003877	+	3'UTR	*FAF1*
6	149321129	149321129	+	149321129	3	3	ENSSSCG00000030552	+	intron	*DMRTA2*
6	150031167	150031167	-	150031167	4	4	---	---	intergenic	---
6	150144066	150144066	+	150144066	8	8	---	---	intergenic	---
6	150290382	150290382	+	150290382	4	4	---	---	intergenic	---
6	150417921	150417921	+	150417921	7	7	---	---	intergenic	---
6	150796945	150796945	-	150796945	3	3	XLOC_074292	-	predicted	---
6	150965717	150965717	-	150965717	3	3	---	---	intergenic	---
6	150968838	150968846	+	150968843	132	248	XLOC_072728	+	predicted	---
6	151121832	151121838	+	151121833	20	58	---	---	intergenic	---
6	151404618	151404621	+	151404621	11	15	---	---	intergenic	---
6	151642099	151642099	+	151642099	3	3	---	---	intergenic	---
6	151666956	151666957	-	151666957	12	17	---	---	intergenic	---
6	151712203	151712209	-	151712208	15	38	ENSSSCG00000003885	-	3'UTR	*CMPK1*
6	151713283	151713287	-	151713285	15	27	ENSSSCG00000003885	-	3'UTR	*CMPK1*
6	151714197	151714245	-	151714208	80	175	ENSSSCG00000003885	-	3'UTR	*CMPK1*
6	151715974	151715974	-	151715974	3	3	ENSSSCG00000003885	-	intron	*CMPK1*
6	151716401	151716401	-	151716401	4	4	ENSSSCG00000003885	-	ORF	*CMPK1*

6	151718043	151718043	-	151718043	4	4	ENSSSCG00000003885	-	intron	*CMPK1*
6	151813755	151813759	+	151813759	4	7	XLOC_072734	+	predicted	---
6	152187407	152187414	+	152187413	16	67	ENSSSCG00000003891	+	3'UTR	---
6	152207239	152207245	+	152207239	19	56	XLOC_072739	+	predicted	---
6	152210035	152210036	-	152210036	13	19	ENSSSCG00000003894	-	3'UTR	*CYP4B1*
6	152284911	152284911	-	152284911	5	5	XLOC_074313	-	predicted	---
6	152285270	152285270	-	152285270	3	3	XLOC_074313	-	predicted	---
6	152286751	152286751	-	152286751	6	6	XLOC_074313	-	predicted	---
6	152286892	152286894	-	152286894	4	7	XLOC_074313	-	predicted	---
6	152317572	152317601	+	152317597	151	418	XLOC_072744	+	predicted	---
6	152321771	152321774	-	152321772	4	13	ENSSSCG00000003895	-	intron	*EFCAB14*
6	152323279	152323279	-	152323279	7	7	ENSSSCG00000003895	-	intron	*EFCAB14*
6	152373155	152373155	+	152373155	3	3	XLOC_072748	+	predicted	---
6	152400937	152400937	+	152400937	5	5	ENSSSCG00000003898	+	intron	*MKNK1*
6	152423379	152423382	+	152423379	20	37	ENSSSCG00000003898	+	3'UTR	*MKNK1*
6	152539044	152539049	-	152539049	21	31	ENSSSCG00000003901	-	3'UTR	*FAAH*
6	152539317	152539317	-	152539317	4	4	ENSSSCG00000003901	-	3'UTR	*FAAH*
6	152572652	152572659	-	152572653	15	45	ENSSSCG00000003902	-	3'UTR	*NSUN4*
6	152573495	152573500	-	152573500	18	33	ENSSSCG00000003902	-	3'UTR	*NSUN4*
6	152579842	152579846	-	152579846	6	13	ENSSSCG00000003902	-	intron	*NSUN4*
6	152598386	152598388	+	152598386	7	14	---	---	intergenic	---
6	152603249	152603250	-	152603250	61	67	XLOC_074321	-	predicted	---
6	152620771	152620771	+	152620771	3	3	ENSSSCG00000003904	+	intron	*LRRC41*
6	152636271	152636271	-	152636271	4	4	ENSSSCG00000003905	-	3'UTR	*RAD54L*
6	152636343	152636355	+	152636351	28	62	ENSSSCG00000003904	+	3'UTR	*LRRC41*
6	152636354	152636357	-	152636354	6	12	ENSSSCG00000003905	-	3'UTR	*RAD54L*

6	152667589	152667589	-	152667589	3	3	---	---	intergenic	---
6	152701863	152701875	+	152701871	30	64	ENSSSCG00000029145	+	3'UTR	*POMGNT1*
6	152712983	152712997	+	152712986	212	415	XLOC_072756	+	predicted	---
6	152851951	152851951	+	152851951	3	3	ENSSSCG00000003909	+	intron	---
6	152889389	152889389	+	152889389	4	4	ENSSSCG00000003909	+	ORF	---
6	152912109	152912120	+	152912109	3	9	ENSSSCG00000003909	+	3'UTR	---
6	152914706	152914710	+	152914706	21	30	XLOC_072757	+	predicted	---
6	152991349	152991349	-	152991349	19	19	---	---	intergenic	---
6	153070045	153070045	-	153070045	4	4	---	---	intergenic	---
6	153138573	153138575	+	153138573	16	30	---	---	intergenic	---
6	153254617	153254617	+	153254617	3	3	ENSSSCG00000003914	+	intron	*PRDX1*
6	153265932	153265949	+	153265946	495	1504	ENSSSCG00000003914	+	3'UTR	*PRDX1*
6	153266914	153266927	-	153266918	8	18	ENSSSCG00000025848	-	3'UTR	*MMACHC*
6	153267388	153267388	-	153267388	4	4	ENSSSCG00000025848	-	3'UTR	*MMACHC*
6	153267463	153267464	+	153267463	4	8	XLOC_072760	+	predicted	---
6	153276766	153276769	+	153276767	40	59	XLOC_072761	+	predicted	---
6	153335270	153335270	+	153335270	4	4	ENSSSCG00000003917	+	intron	*TESK2*
6	153408251	153408251	-	153408251	8	8	ENSSSCG00000003918	-	3'UTR	*TOE1*
6	153408344	153408352	+	153408351	14	30	XLOC_072762	+	predicted	---
6	153420248	153420248	+	153420248	4	4	ENSSSCG00000003919	+	3'UTR	*MUTYH*
6	153679218	153679218	-	153679218	4	4	---	---	intergenic	---
6	153900671	153900673	+	153900671	20	40	ENSSSCG00000029808	+	3'UTR	*EIF2B3*
6	153911652	153911662	-	153911654	50	112	ENSSSCG00000003922	-	3'UTR	*UROD*
6	154001408	154001410	-	154001410	5	8	ENSSSCG00000027423	-	intron	*TMEM53*
6	154006826	154006826	+	154006826	3	3	ENSSSCG00000003931	+	ORF	*KIF2C*
6	154022494	154022500	+	154022497	58	105	ENSSSCG00000003931	+	3'UTR	*KIF2C*

6	154023033	154023033	+	154023033	4	4	XLOC_072770	+	predicted	---
6	154027001	154027013	-	154027007	17	73	ENSSSCG00000027423	-	intron	*TMEM53*
6	154027468	154027468	+	154027468	9	9	ENSSSCG00000003930	+	intron	*RPS8*
6	154028620	154028652	+	154028652	22	34	ENSSSCG00000003930	+	ORF	*RPS8*
6	154029222	154029240	+	154029235	866	4635	XLOC_072771	+	predicted	---
6	154051066	154051067	+	154051067	7	10	ENSSSCG00000003928	+	3'UTR	*PLK3*
6	154098390	154098390	-	154098390	3	3	ENSSSCG00000027423	-	intron	*TMEM53*
6	154131665	154131665	+	154131665	5	5	XLOC_072779	+	predicted	---
6	154131717	154131717	+	154131717	3	3	XLOC_072779	+	predicted	---
6	154149133	154149135	-	154149134	6	13	---	---	intergenic	---
6	154367294	154367294	-	154367294	7	7	---	---	intergenic	---
6	154369371	154369371	+	154369371	20	20	---	---	intergenic	---
6	154455088	154455088	+	154455088	3	3	---	---	intergenic	---
6	154501492	154501500	-	154501492	4	7	XLOC_074341	-	predicted	---
6	154722306	154722306	-	154722306	3	3	---	---	intergenic	---
6	154785603	154785603	+	154785603	3	3	XLOC_072788	+	predicted	---
6	154792401	154792409	+	154792402	10	16	XLOC_072789	+	predicted	---
6	155173769	155173789	-	155173772	15	29	XLOC_074355	-	predicted	---
6	155180453	155180460	+	155180457	107	236	ENSSSCG00000003940	+	3'UTR	*HYI*
6	155180462	155180462	-	155180462	3	3	ENSSSCG00000003941	-	3'UTR	*SZT2*
6	155217281	155217283	+	155217283	8	11	XLOC_072798	+	predicted	---
6	155309332	155309339	-	155309333	6	15	XLOC_074358	-	predicted	---
6	155322119	155322124	+	155322119	20	25	XLOC_072800	+	predicted	---
6	155332350	155332350	-	155332350	10	10	XLOC_074359	-	predicted	---
6	155340893	155340902	+	155340901	19	51	ENSSSCG00000023937	+	3'UTR	*ELOVL1*
6	155341044	155341055	-	155341046	40	128	ENSSSCG00000003949	-	3'UTR	*CDC20*

6	155358635	155358635	-	155358635	3	3	ENSSSCG00000003943	-	intron	*MED8*
6	155359482	155359482	-	155359482	4	4	ENSSSCG00000003943	-	intron	*MED8*
6	155359525	155359540	-	155359526	29	88	ENSSSCG00000003943	-	intron	*MED8*
6	155399502	155399502	+	155399502	3	3	ENSSSCG00000003951	+	3'UTR	*C1orf210*
6	155424129	155424130	-	155424130	8	15	XLOC_074366	-	predicted	---
6	155494095	155494099	+	155494096	19	27	ENSSSCG00000030177	+	3'UTR	*EBNA1BP2*
6	155494175	155494176	+	155494175	4	7	ENSSSCG00000030177	+	3'UTR	*EBNA1BP2*
6	155494351	155494351	+	155494351	5	5	ENSSSCG00000030177	+	3'UTR	*EBNA1BP2*
6	155499080	155499084	-	155499080	13	20	XLOC_074367	-	predicted	---
6	155499161	155499187	-	155499163	51	111	XLOC_074367	-	predicted	---
6	155502117	155502121	-	155502121	9	20	ENSSSCG00000003954	-	intron	*FAM183A*
6	155984610	155984615	+	155984610	13	20	ENSSSCG00000026635	+	3'UTR	---
6	156002962	156002962	+	156002962	4	4	XLOC_072818	+	predicted	---
6	156140025	156140025	-	156140025	4	4	XLOC_074375	-	predicted	---
6	156159090	156159094	+	156159091	19	32	ENSSSCG00000023920	+	3'UTR	*P3H1*
6	156178362	156178382	-	156178362	4	11	ENSSSCG00000027374	-	3'UTR	*C1orf50*
6	156189770	156189789	-	156189788	192	333	ENSSSCG00000028485	-	3'UTR	*YBX1*
6	156189984	156189984	-	156189984	5	5	ENSSSCG00000028485	-	3'UTR	*YBX1*
6	156190677	156190678	-	156190677	4	7	ENSSSCG00000028485	-	intron	*YBX1*
6	156190815	156190825	-	156190815	6	10	ENSSSCG00000028485	-	ORF	*YBX1*
6	156190907	156190923	-	156190907	3	6	ENSSSCG00000028485	-	ORF	*YBX1*
6	156194365	156194365	-	156194365	7	7	ENSSSCG00000028485	-	ORF	*YBX1*
6	156219666	156219666	-	156219666	6	6	---	---	intergenic	---
6	156223014	156223019	-	156223016	32	81	ENSSSCG00000003964	-	3'UTR	*PPIH*
6	156227548	156227548	-	156227548	3	3	ENSSSCG00000003964	-	intron	*PPIH*
6	156246570	156246583	-	156246581	146	266	XLOC_074380	-	predicted	---

6	156253406	156253408	-	156253408	24	34	ENSSSCG00000003965	-	intron	*CCDC30*
6	156274480	156274482	-	156274480	6	9	ENSSSCG00000003965	-	ORF	*CCDC30*
6	156287381	156287383	-	156287383	6	9	ENSSSCG00000003965	-	ORF	*CCDC30*
6	156295223	156295223	-	156295223	4	4	ENSSSCG00000003965	-	intron	*CCDC30*
6	156297270	156297270	-	156297270	4	4	ENSSSCG00000003965	-	intron	*CCDC30*
6	156310082	156310082	-	156310082	3	3	ENSSSCG00000003965	-	intron	*CCDC30*
6	156366595	156366602	-	156366599	25	60	ENSSSCG00000003967	-	3'UTR	*ZMYND12*
6	156370144	156370144	-	156370144	3	3	ENSSSCG00000003967	-	intron	*ZMYND12*
6	156396904	156396905	+	156396904	7	10	ENSSSCG00000003966	+	3'UTR	---
6	156541870	156541871	-	156541870	5	8	---	---	intergenic	---
6	156770135	156770135	+	156770135	3	3	---	---	intergenic	---
6	156875123	156875123	+	156875123	3	3	---	---	intergenic	---
6	157033644	157033644	+	157033644	12	12	---	---	intergenic	---
6	157236956	157236956	+	157236956	4	4	ENSSSCG00000003971	+	3'UTR	---
6	157246728	157246732	+	157246730	6	16	XLOC_072837	+	predicted	---
6	157249355	157249355	-	157249355	10	10	ENSSSCG00000003973	-	3'UTR	*CTPS1*
6	157337679	157337679	-	157337679	3	3	XLOC_074392	-	predicted	---
6	157460448	157460448	-	157460448	7	7	ENSSSCG00000003976	-	3'UTR	*NFYC*
6	157460896	157460896	-	157460896	7	7	ENSSSCG00000003976	-	3'UTR	*NFYC*
6	157549414	157549414	-	157549414	5	5	ENSSSCG00000003983	-	intron	*SMAP1L*
6	157609337	157609340	-	157609338	8	20	ENSSSCG00000003980	-	3'UTR	---
							ENSSSCG00000003983	-	intron	*SMAP1L*
6	157673260	157673265	-	157673265	79	158	ENSSSCG00000003983	-	intron	*SMAP1L*
7	48090	48093	-	48093	42	60	ENSSSCG00000000985	-	3'UTR	*EXOC2*
7	1010263	1010264	-	1010263	14	18	---	---	intergenic	---
7	1047469	1047484	-	1047482	22	61	ENSSSCG00000000994	-	3'UTR	*GMDS*

7	1105654	1105654	-	1105654	4	4	ENSSSCG00000000994	-	intron	*GMDS*
7	1410008	1410009	+	1410008	10	14	---	---	intergenic	---
7	1762958	1762958	+	1762958	3	3	ENSSSCG00000001010	+	intron	*WRNIP1*
7	1766911	1766918	+	1766911	16	23	ENSSSCG00000001010	+	3'UTR	*WRNIP1*
7	1783255	1783257	-	1783256	74	91	XLOC_079984	-	predicted	---
7	1784113	1784113	-	1784113	6	6	XLOC_079984	-	predicted	---
7	1823570	1823570	-	1823570	3	3	XLOC_079985	-	predicted	---
7	1825221	1825221	+	1825221	4	4	XLOC_078787	+	predicted	---
7	1929395	1929395	+	1929395	4	4	ENSSSCG00000001009	+	intron	*RIPK1*
7	1945185	1945191	+	1945185	10	16	ENSSSCG00000001009	+	3'UTR	*RIPK1*
7	1974341	1974352	+	1974347	35	50	XLOC_078790	+	predicted	---
7	2013513	2013513	-	2013513	7	7	XLOC_079987	-	predicted	---
7	2048757	2048775	+	2048763	11	53	XLOC_078794	+	predicted	---
7	2201402	2201405	+	2201405	8	11	---	---	intergenic	---
7	2293808	2293819	-	2293818	8	16	XLOC_079991	-	predicted	---
7	2454112	2454120	-	2454120	9	26	XLOC_079995	-	predicted	---
7	2485723	2485723	+	2485723	4	4	---	---	intergenic	---
7	2491563	2491564	+	2491564	7	11	XLOC_078801	+	predicted	---
7	2491719	2491719	+	2491719	4	4	XLOC_078801	+	predicted	---
7	2493227	2493227	+	2493227	4	4	XLOC_078801	+	predicted	---
7	2506392	2506392	-	2506392	3	3	ENSSSCG00000001000	-	3'UTR	*ECI2*
7	2506903	2506903	-	2506903	3	3	ENSSSCG00000001000	-	3'UTR	*ECI2*
7	2506949	2506985	-	2506955	26	70	ENSSSCG00000001000	-	3'UTR	*ECI2*
7	2507385	2507385	+	2507385	4	4	ENSSSCG00000001001	+	intron	*C6orf201*
7	2595054	2595054	-	2595054	4	4	XLOC_079999	-	predicted	---
7	2798041	2798042	-	2798041	6	9	XLOC_080002	-	predicted	---

7	3069360	3069360	+	3069360	9	9	ENSSSCG00000000999	+	3'UTR	---
7	3070656	3070656	+	3070656	5	5	ENSSSCG00000000999	+	3'UTR	---
7	3096156	3096162	-	3096158	9	22	ENSSSCG00000000998	-	3'UTR	*RPP40*
7	3174593	3174619	-	3174594	32	52	XLOC_080005	-	predicted	---
7	3548454	3548465	+	3548457	63	133	XLOC_078808	+	predicted	---
7	3554550	3554553	+	3554550	4	7	XLOC_078809	+	predicted	---
7	3577046	3577046	+	3577046	5	5	---	---	intergenic	---
7	3594163	3594163	-	3594163	4	4	---	---	intergenic	---
7	3594183	3594183	+	3594183	3	3	---	---	intergenic	---
7	3759607	3759607	-	3759607	63	63	---	---	intergenic	---
7	3800823	3800823	+	3800823	4	4	ENSSSCG00000026199	+	intron	---
7	3915738	3915739	+	3915738	34	51	---	---	intergenic	---
7	4241695	4241695	-	4241695	3	3	---	---	intergenic	---
7	4460432	4460433	+	4460433	46	50	---	---	intergenic	---
7	4587315	4587315	+	4587315	3	3	XLOC_078816	+	predicted	---
7	4623672	4623672	+	4623672	5	5	ENSSSCG00000001021	+	intron	*RREB1*
7	4652445	4652466	+	4652466	11	14	ENSSSCG00000001021	+	3'UTR	*RREB1*
7	4671191	4671198	-	4671192	19	26	XLOC_080018	-	predicted	---
7	4671884	4671884	-	4671884	7	7	XLOC_080018	-	predicted	---
7	4676449	4676450	-	4676449	12	16	XLOC_080018	-	predicted	---
7	4677207	4677216	-	4677208	11	28	XLOC_080018	-	predicted	---
7	4677860	4677864	-	4677863	55	98	XLOC_080018	-	predicted	---
7	4695305	4695305	-	4695305	5	5	ENSSSCG00000001022	-	intron	*SSR1*
7	4751294	4751300	+	4751300	4	7	XLOC_078817	+	predicted	---
7	4770116	4770116	-	4770116	3	3	---	---	intergenic	---
7	4880937	4880939	+	4880937	12	19	ENSSSCG00000001024	+	ORF	*RIOK1*

7	4882349	4882349	+	4882349	5	5	ENSSSCG00000001024	+	3'UTR	*RIOK1*
7	4893100	4893101	-	4893100	4	7	---	---	intergenic	---
7	4898460	4898463	+	4898460	10	16	---	---	intergenic	---
7	4922251	4922251	+	4922251	5	5	---	---	intergenic	---
7	5006227	5006227	+	5006227	5	5	ENSSSCG00000001025	+	ORF	*DSP*
7	5019191	5019191	+	5019191	3	3	ENSSSCG00000001025	+	ORF	*DSP*
7	5020983	5020983	+	5020983	6	6	ENSSSCG00000001025	+	3'UTR	*DSP*
7	5021220	5021221	+	5021221	15	20	ENSSSCG00000001025	+	3'UTR	*DSP*
7	5040421	5040423	+	5040423	15	25	XLOC_078822	+	predicted	---
7	5058634	5058634	-	5058634	5	5	---	---	intergenic	---
7	5092850	5092850	-	5092850	3	3	---	---	intergenic	---
7	5118417	5118417	+	5118417	3	3	---	---	intergenic	---
7	5314628	5314639	+	5314639	5	13	ENSSSCG00000001027	+	3'UTR	*BMP6*
7	5421434	5421437	+	5421436	18	40	ENSSSCG00000001030	+	3'UTR	---
7	5433992	5433992	+	5433992	3	3	ENSSSCG00000026619	+	intron	*BLOC1S5*
7	5469290	5469295	+	5469295	5	11	ENSSSCG00000026619	+	3'UTR	*BLOC1S5*
7	5499888	5499888	+	5499888	8	8	---	---	intergenic	---
7	5743333	5743333	-	5743333	4	4	---	---	intergenic	---
7	6048849	6048849	-	6048849	5	5	---	---	intergenic	---
7	6213066	6213066	+	6213066	3	3	---	---	intergenic	---
7	6328705	6328708	+	6328705	15	36	---	---	intergenic	---
7	6792275	6792276	-	6792275	6	10	---	---	intergenic	---
7	7070414	7070414	+	7070414	3	3	---	---	intergenic	---
7	7097704	7097704	-	7097704	3	3	XLOC_080029	-	predicted	---
7	7298142	7298142	+	7298142	3	3	---	---	intergenic	---
7	7601308	7601310	+	7601309	37	43	---	---	intergenic	---

7	7734490	7734493	+	7734490	4	11	ENSSSCG00000001039	+	3'UTR	*GCNT2*
7	7761461	7761461	+	7761461	3	3	ENSSSCG00000001041	+	intron	*PAK1IP1*
7	7769027	7769027	+	7769027	6	6	XLOC_078841	+	predicted	---
7	7780203	7780203	+	7780203	3	3	ENSSSCG00000001038	+	ORF	---
7	7781639	7781647	+	7781645	64	165	XLOC_078842	+	predicted	---
7	7793736	7793736	-	7793736	10	10	XLOC_080035	-	predicted	---
7	7793801	7793806	-	7793801	14	20	XLOC_080035	-	predicted	---
7	7809599	7809600	-	7809599	11	16	ENSSSCG00000001042	-	intron	*MAK*
7	7976858	7976858	-	7976858	4	4	XLOC_080036	-	predicted	---
7	7976967	7976968	-	7976968	8	12	XLOC_080036	-	predicted	---
7	7979448	7979448	-	7979448	9	9	XLOC_080036	-	predicted	---
7	8171037	8171055	-	8171055	9	12	XLOC_080037	-	predicted	---
7	8188471	8188471	-	8188471	5	5	---	---	intergenic	---
7	8315144	8315144	+	8315144	3	3	---	---	intergenic	---
7	8554663	8554663	-	8554663	3	3	---	---	intergenic	---
7	8586805	8586805	+	8586805	6	6	ENSSSCG00000001048	+	intron	---
7	8595328	8595328	+	8595328	4	4	ENSSSCG00000001048	+	intron	---
7	8644465	8644475	+	8644475	20	68	XLOC_078846	+	predicted	---
7	8645085	8645085	+	8645085	3	3	XLOC_078846	+	predicted	---
7	8645232	8645232	+	8645232	4	4	XLOC_078846	+	predicted	---
7	8708892	8708892	-	8708892	3	3	XLOC_080041	-	predicted	---
7	9063409	9063409	+	9063409	5	5	ENSSSCG00000001049	+	3'UTR	*HIVEP1*
7	9333605	9333606	+	9333605	3	6	---	---	intergenic	---
7	9698469	9698469	+	9698469	3	3	ENSSSCG00000001052	+	intron	*PHACTR1*
7	9955008	9955017	+	9955017	5	12	XLOC_078857	+	predicted	---
7	9955698	9955708	+	9955700	63	163	XLOC_078857	+	predicted	---

7	9976373	9976388	-	9976387	7	16	ENSSSCG00000001053	-	3'UTR	*TBC1D7*
7	9976758	9976769	-	9976758	6	17	ENSSSCG00000001053	-	3'UTR	*TBC1D7*
7	10287654	10287659	-	10287657	17	35	ENSSSCG00000001056	-	3'UTR	*RANBP9*
7	10287776	10287778	-	10287777	22	33	ENSSSCG00000001056	-	3'UTR	*RANBP9*
7	10287825	10287825	-	10287825	6	6	ENSSSCG00000001056	-	3'UTR	*RANBP9*
7	10339232	10339232	+	10339232	6	6	---	---	intergenic	---
7	10351400	10351400	-	10351400	3	3	---	---	intergenic	---
7	10356996	10356996	-	10356996	4	4	---	---	intergenic	---
7	10533328	10533328	+	10533328	3	3	---	---	intergenic	---
7	10548744	10548744	-	10548744	3	3	ENSSSCG00000001057	-	3'UTR	*MCUR1*
7	10548943	10548943	-	10548943	3	3	ENSSSCG00000001057	-	3'UTR	*MCUR1*
7	10552293	10552293	-	10552293	3	3	ENSSSCG00000001057	-	3'UTR	*MCUR1*
7	10576602	10576613	-	10576605	4	17	---	---	intergenic	---
7	10629413	10629413	-	10629413	3	3	---	---	intergenic	---
7	10818888	10818889	+	10818889	17	23	XLOC_078860	+	predicted	---
7	11067340	11067340	-	11067340	3	3	---	---	intergenic	---
7	11138571	11138572	-	11138571	4	7	---	---	intergenic	---
7	11467166	11467167	+	11467167	4	7	XLOC_078864	+	predicted	---
7	11467680	11467682	+	11467681	13	20	XLOC_078864	+	predicted	---
7	11713403	11713403	-	11713403	3	3	---	---	intergenic	---
7	12190504	12190504	+	12190504	3	3	XLOC_078871	+	predicted	---
7	12191164	12191164	+	12191164	4	4	XLOC_078871	+	predicted	---
7	12191458	12191463	-	12191458	7	10	XLOC_080060	-	predicted	---
7	12262757	12262757	-	12262757	3	3	ENSSSCG00000001062	-	ORF	*DTNBP1*
7	12520268	12520268	+	12520268	3	3	---	---	intergenic	---
7	12658704	12658708	-	12658708	11	31	XLOC_080063	-	predicted	---

7	12659300	12659300	-	12659300	7	7	XLOC_080063	-	predicted	---
7	12680851	12680852	+	12680852	16	20	---	---	intergenic	---
7	12839692	12839694	-	12839692	4	8	---	---	intergenic	---
7	12884833	12884833	+	12884833	6	6	ENSSSCG00000001064	+	intron	*GMPR*
7	12893441	12893449	+	12893448	9	31	ENSSSCG00000001064	+	3'UTR	*GMPR*
7	12896241	12896247	-	12896241	8	11	XLOC_080065	-	predicted	---
7	13062836	13062837	+	13062837	9	16	---	---	intergenic	---
7	13116060	13116061	-	13116060	7	13	---	---	intergenic	---
7	13148442	13148442	+	13148442	6	6	---	---	intergenic	---
7	13236160	13236160	+	13236160	5	5	---	---	intergenic	---
7	13381390	13381390	-	13381390	3	3	---	---	intergenic	---
7	13462874	13462874	-	13462874	5	5	---	---	intergenic	---
7	13596644	13596644	+	13596644	8	8	---	---	intergenic	---
7	14051924	14051924	+	14051924	3	3	ENSSSCG00000001068	+	3'UTR	---
7	14053038	14053038	+	14053038	4	4	---	---	intergenic	---
7	14090024	14090024	+	14090024	3	3	XLOC_078887	+	predicted	---
7	14090079	14090090	+	14090081	11	37	XLOC_078887	+	predicted	---
7	14091865	14091866	+	14091865	10	13	XLOC_078887	+	predicted	---
7	14092967	14092972	+	14092968	16	32	XLOC_078887	+	predicted	---
7	14095619	14095619	-	14095619	6	6	XLOC_080070	-	predicted	---
7	14095854	14095854	-	14095854	23	23	ENSSSCG00000026034	-	3'UTR	*NUP153*
7	14096860	14096870	-	14096866	11	18	ENSSSCG00000026034	-	3'UTR	*NUP153*
7	14103731	14103731	-	14103731	3	3	ENSSSCG00000026034	-	intron	*NUP153*
7	14112997	14113001	-	14113000	13	39	ENSSSCG00000026034	-	intron	*NUP153*
7	14118180	14118180	-	14118180	3	3	ENSSSCG00000026034	-	ORF	*NUP153*
7	14139793	14139793	-	14139793	5	5	ENSSSCG00000026034	-	intron	*NUP153*

7	14196562	14196562	+	14196562	7	7	---	---	intergenic	---
7	14197817	14197817	+	14197817	6	6	---	---	intergenic	---
7	14201283	14201285	+	14201283	16	26	XLOC_078888	+	predicted	---
7	14201478	14201478	+	14201478	4	4	XLOC_078888	+	predicted	---
7	14201612	14201637	+	14201612	29	82	XLOC_078888	+	predicted	---
7	14254779	14254779	-	14254779	3	3	---	---	intergenic	---
7	14562567	14562576	-	14562567	6	10	ENSSSCG00000001073	-	3'UTR	*TPMT*
7	14563472	14563472	-	14563472	3	3	ENSSSCG00000001073	-	ORF	*TPMT*
7	14564325	14564325	-	14564325	3	3	ENSSSCG00000001073	-	intron	*TPMT*
7	14618116	14618116	+	14618116	3	3	ENSSSCG00000001074	+	intron	*KDM1B*
7	14642972	14642972	+	14642972	4	4	ENSSSCG00000001074	+	3'UTR	*KDM1B*
7	14643282	14643282	-	14643282	6	6	XLOC_080078	-	predicted	---
7	14643349	14643349	-	14643349	11	11	XLOC_080078	-	predicted	---
7	14643533	14643536	-	14643533	3	6	XLOC_080078	-	predicted	---
7	14643971	14643971	-	14643971	3	3	XLOC_080078	-	predicted	---
7	14649635	14649635	-	14649635	5	5	ENSSSCG00000001075	-	ORF	*DEK*
7	14805054	14805054	+	14805054	3	3	ENSSSCG00000001076	+	intron	*RNF144B*
7	14860468	14860469	+	14860469	10	13	ENSSSCG00000001076	+	3'UTR	*RNF144B*
7	14974332	14974332	-	14974332	4	4	---	---	intergenic	---
7	15080547	15080549	-	15080548	41	89	---	---	intergenic	---
7	15670686	15670686	+	15670686	6	6	---	---	intergenic	---
7	15675942	15675942	-	15675942	3	3	---	---	intergenic	---
7	16061519	16061522	-	16061519	12	15	---	---	intergenic	---
7	16061577	16061578	+	16061578	35	38	---	---	intergenic	---
7	16411823	16411824	+	16411823	11	20	---	---	intergenic	---
7	16779067	16779067	+	16779067	4	4	ENSSSCG00000001079	+	3'UTR	*E2F3*

7	16891453	16891453	+	16891453	3	3	ENSSSCG00000001080	+	intron	*CDKAL1*
7	17015775	17015775	+	17015775	4	4	ENSSSCG00000001080	+	intron	*CDKAL1*
7	17125154	17125154	+	17125154	11	11	ENSSSCG00000001080	+	intron	*CDKAL1*
7	17265836	17265842	+	17265837	11	21	ENSSSCG00000001080	+	intron	*CDKAL1*
7	17290974	17290974	+	17290974	3	3	ENSSSCG00000001080	+	intron	*CDKAL1*
7	17480991	17480991	+	17480991	3	3	ENSSSCG00000001080	+	3'UTR	*CDKAL1*
7	17856546	17856546	+	17856546	3	3	XLOC_078904	+	predicted	---
7	17857158	17857159	+	17857158	9	16	XLOC_078904	+	predicted	---
7	18195489	18195489	+	18195489	7	7	---	---	intergenic	---
7	18232473	18232473	-	18232473	4	4	XLOC_080086	-	predicted	---
7	18412508	18412509	-	18412508	89	92	ENSSSCG00000001082	-	3'UTR	*PRL*
7	18447692	18447692	+	18447692	3	3	---	---	intergenic	---
7	18495135	18495135	+	18495135	4	4	---	---	intergenic	---
7	18658632	18658636	+	18658633	26	40	---	---	intergenic	---
7	18685344	18685344	-	18685344	4	4	---	---	intergenic	---
7	18685381	18685381	+	18685381	7	7	---	---	intergenic	---
7	19008869	19008869	-	19008869	3	3	---	---	intergenic	---
7	19509839	19509841	+	19509840	17	24	---	---	intergenic	---
7	19660457	19660457	-	19660457	4	4	---	---	intergenic	---
7	19763202	19763202	+	19763202	4	4	---	---	intergenic	---
7	19979713	19979713	-	19979713	3	3	---	---	intergenic	---
7	20139866	20139866	+	20139866	3	3	---	---	intergenic	---
7	20194227	20194227	-	20194227	3	3	XLOC_080091	-	predicted	---
7	20390557	20390558	+	20390557	9	12	XLOC_078913	+	predicted	---
7	20442103	20442103	+	20442103	3	3	ENSSSCG00000001087	+	3'UTR	*MRS2*
7	20515559	20515559	+	20515559	3	3	ENSSSCG00000001090	+	ORF	*ALDH5A1*

7	20521802	20521802	+	20521802	8	8	ENSSSCG00000001090	+	ORF	*ALDH5A1*
7	20525990	20525992	+	20525990	3	6	ENSSSCG00000001090	+	3'UTR	*ALDH5A1*
7	20526043	20526043	+	20526043	3	3	XLOC_078915	+	predicted	---
7	20526503	20526503	+	20526503	12	12	XLOC_078915	+	predicted	---
7	20528485	20528492	+	20528492	9	16	XLOC_078915	+	predicted	---
7	20529571	20529581	+	20529571	4	11	XLOC_078915	+	predicted	---
7	20543143	20543143	-	20543143	3	3	ENSSSCG00000001091	-	intron	*KIAA0319*
7	20642149	20642153	-	20642149	7	16	ENSSSCG00000001092	-	3'UTR	*TDP2*
7	20643159	20643159	-	20643159	5	5	ENSSSCG00000001092	-	3'UTR	*TDP2*
7	20658618	20658618	+	20658618	3	3	XLOC_078918	+	predicted	---
7	20659470	20659472	+	20659471	8	15	XLOC_078918	+	predicted	---
7	20662442	20662479	-	20662442	8	17	ENSSSCG00000001094	-	3'UTR	*C6orf62*
7	20663194	20663194	-	20663194	5	5	ENSSSCG00000001094	-	3'UTR	*C6orf62*
7	20663415	20663428	-	20663428	6	9	ENSSSCG00000001094	-	3'UTR	*C6orf62*
7	20668878	20668878	-	20668878	7	7	ENSSSCG00000001094	-	intron	*C6orf62*
7	20675272	20675278	-	20675274	9	19	ENSSSCG00000001094	-	ORF	*C6orf62*
7	20675495	20675495	-	20675495	3	3	XLOC_080098	-	predicted	---
7	20691483	20691483	+	20691483	4	4	---	---	intergenic	---
7	20691678	20691678	+	20691678	4	4	---	---	intergenic	---
7	20724516	20724516	+	20724516	3	3	---	---	intergenic	---
7	20748705	20748719	+	20748709	51	118	ENSSSCG00000001095	+	3'UTR	*GMNN*
7	20752373	20752385	-	20752380	43	91	---	---	intergenic	---
7	20752789	20752789	-	20752789	3	3	ENSSSCG00000001096	-	ORF	*C6orf229*
7	20762726	20762726	-	20762726	3	3	XLOC_080100	-	predicted	---
7	20764412	20764431	-	20764430	26	88	XLOC_080100	-	predicted	---
7	20783199	20783199	-	20783199	4	4	ENSSSCG00000001097	-	intron	*FAM65B*

7	20915547	20915547	-	20915547	3	3	---	---	intergenic	---
7	20988379	20988379	+	20988379	6	6	---	---	intergenic	---
7	21403796	21403796	+	21403796	4	4	---	---	intergenic	---
7	21424034	21424034	-	21424034	8	8	---	---	intergenic	---
7	21447415	21447415	-	21447415	9	9	---	---	intergenic	---
7	21635823	21635826	+	21635826	11	26	ENSSSCG00000001100	+	intron	*LRRC16A*
7	21652560	21652560	+	21652560	3	3	ENSSSCG00000001100	+	3'UTR	*LRRC16A*
7	21652621	21652649	+	21652628	14	40	ENSSSCG00000001100	+	3'UTR	*LRRC16A*
7	21653300	21653300	+	21653300	3	3	ENSSSCG00000001100	+	3'UTR	*LRRC16A*
7	21653377	21653378	+	21653378	9	13	ENSSSCG00000001100	+	3'UTR	*LRRC16A*
7	21732924	21732924	+	21732924	4	4	ENSSSCG00000001101	+	3'UTR	*SCGN*
7	21764399	21764399	-	21764399	4	4	ENSSSCG00000001102	-	ORF	*HIST1H2AA*
7	21764815	21764816	+	21764816	149	152	---	---	intergenic	---
7	21765206	21765206	+	21765206	3	3	ENSSSCG00000023900	+	ORF	---
7	21765532	21765532	+	21765532	8	8	---	---	intergenic	---
7	21765871	21765871	+	21765871	3	3	---	---	intergenic	---
7	21770180	21770180	-	21770180	3	3	XLOC_080108	-	predicted	---
7	21776795	21776795	+	21776795	6	6	XLOC_078930	+	predicted	---
7	21818210	21818210	+	21818210	5	5	XLOC_078931	+	predicted	---
7	21877793	21877793	+	21877793	5	5	ENSSSCG00000001107	+	ORF	*OATV1*
7	22044568	22044577	-	22044569	23	50	ENSSSCG00000021411	-	3'UTR	---
7	22053919	22053919	-	22053919	16	16	ENSSSCG00000001138	-	ORF	---
7	22054078	22054078	-	22054078	3	3	ENSSSCG00000001138	-	ORF	---
7	22073103	22073103	+	22073103	3	3	ENSSSCG00000001137	+	ORF	*HIST1H1D*
7	22074355	22074356	+	22074356	21	24	---	---	intergenic	---
7	22075155	22075156	+	22075156	7	10	---	---	intergenic	---

7	22080852	22080853	-	22080853	9	13	---	---	intergenic	---
7	22089861	22089861	+	22089861	13	13	XLOC_078939	+	predicted	---
7	22164285	22164285	+	22164285	3	3	ENSSSCG00000020696	+	3'UTR	*HMGN4*
							ENSSSCG00000020696	+	ORF	---
7	22165568	22165571	+	22165569	7	14	ENSSSCG00000020696	+	3'UTR	*HMGN4*
							ENSSSCG00000020696	+	ORF	---
7	22417585	22417590	+	22417585	25	34	XLOC_078947	+	predicted	---
7	22513631	22513631	+	22513631	3	3	XLOC_078949	+	predicted	---
7	22520165	22520166	+	22520166	7	12	ENSSSCG00000001156	+	3'UTR	*ZNF322*
							ENSSSCG00000001156	+	ORF	---
7	22522067	22522067	+	22522067	3	3	XLOC_078949	+	predicted	---
7	22808959	22808959	-	22808959	3	3	---	---	intergenic	---
7	22938772	22938772	+	22938772	3	3	---	---	intergenic	---
7	23068178	23068193	+	23068193	5	11	XLOC_078957	+	predicted	---
7	23085816	23085826	-	23085816	5	8	XLOC_080135	-	predicted	---
7	23096767	23096767	-	23096767	3	3	XLOC_080138	-	predicted	---
7	23111036	23111036	+	23111036	4	4	ENSSSCG00000026930	+	ORF	*HIST1H2BN*
7	23164037	23164037	+	23164037	4	4	ENSSSCG00000026930	+	3'UTR	*HIST1H2BN*
7	23176626	23176626	-	23176626	3	3	ENSSSCG00000030023	-	ORF	*HIST1H1B*
7	23188138	23188138	-	23188138	7	7	---	---	intergenic	---
7	23950004	23950004	+	23950004	3	3	ENSSSCG00000001202	+	3'UTR	---
							ENSSSCG00000001202	+	ORF	*protein_coding*
7	24053850	24053879	+	24053871	7	21	ENSSSCG00000001205	+	3'UTR	*NKAPL*
							ENSSSCG00000001205	+	ORF	---

7	24054034	24054034	+	24054034	4	4	ENSSSCG00000001205	+	3'UTR	*NKAPL*
							ENSSSCG00000001205	+	ORF	---
7	24054150	24054156	+	24054156	6	9	XLOC_078980	+	predicted	---
7	24124174	24124174	-	24124174	3	3	---	---	intergenic	---
7	24161053	24161053	+	24161053	3	3	---	---	intergenic	---
7	24204587	24204588	+	24204588	9	16	---	---	intergenic	---
7	24205015	24205015	-	24205015	3	3	---	---	intergenic	---
7	24432799	24432799	-	24432799	8	8	ENSSSCG00000001219	-	3'UTR	*TRIM27*
7	24433620	24433620	-	24433620	7	7	ENSSSCG00000001219	-	3'UTR	*TRIM27*
7	24604956	24604979	+	24604963	2143	3611	ENSSSCG00000030731	+	3'UTR	*TMP-SLA-2*
7	24605024	24605074	+	24605024	8	25	XLOC_078995	+	predicted	---
7	24627382	24627384	+	24627384	4	7	---	---	intergenic	---
7	24632510	24632511	+	24632510	27	35	---	---	intergenic	---
7	24640824	24640834	-	24640833	16	26	XLOC_080161	-	predicted	---
7	24641609	24641621	-	24641617	165	304	---	---	intergenic	---
7	24683605	24683615	-	24683608	447	613	ENSSSCG00000001229	-	3'UTR	*SLA-3*
7	24712830	24712830	+	24712830	3	3	---	---	intergenic	---
7	24720644	24720645	-	24720645	57	61	XLOC_080161	-	predicted	---
7	24748095	24748112	-	24748097	494	638	---	---	intergenic	---
7	24770331	24770336	-	24770333	73	122	XLOC_080165	-	predicted	---
7	24770424	24770424	-	24770424	3	3	XLOC_080165	-	predicted	---
7	24781910	24781910	-	24781910	6	6	XLOC_080165	-	predicted	---
7	24857757	24857772	+	24857770	39	102	XLOC_079001	+	predicted	---
7	24857814	24857814	+	24857814	4	4	XLOC_079001	+	predicted	---
7	24857905	24857910	+	24857905	7	10	XLOC_079001	+	predicted	---
7	25006082	25006096	+	25006094	8	28	XLOC_079004	+	predicted	---

7	25126768	25126771	-	25126768	3	6	ENSSSCG00000001248	-	intron	*MOG*
7	25156363	25156363	-	25156363	9	9	ENSSSCG00000001245	-	3'UTR	*ZNRD1*
							ENSSSCG00000001248	-	intron	*MOG*
7	25165177	25165177	+	25165177	3	3	ENSSSCG00000028784	+	ORF	*C7H6orf12*
7	25165762	25165777	+	25165762	26	59	ENSSSCG00000028784	+	3'UTR	*C7H6orf12*
7	25332775	25332782	+	25332781	9	16	ENSSSCG00000001252	+	3'UTR	*UBD*
							ENSSSCG00000001252	+	ORF	---
7	25431875	25431875	+	25431875	4	4	---	---	intergenic	---
7	25728630	25728630	-	25728630	4	4	---	---	intergenic	---
7	26038316	26038316	-	26038316	5	5	---	---	intergenic	---
7	26480086	26480086	-	26480086	3	3	---	---	intergenic	---
7	26673751	26673759	-	26673752	49	81	XLOC_080217	-	predicted	---
7	26674114	26674119	-	26674119	16	23	XLOC_080217	-	predicted	---
7	26674204	26674235	-	26674212	14	44	XLOC_080217	-	predicted	---
7	26732458	26732463	+	26732463	12	18	XLOC_079036	+	predicted	---
7	26735756	26735785	+	26735777	33	59	ENSSSCG00000021130	+	3'UTR	*FLJ22638*
7	26772313	26772315	-	26772314	21	31	ENSSSCG00000001372	-	3'UTR	*GNL1*
7	26789686	26789686	+	26789686	13	13	ENSSSCG00000021714	+	3'UTR	*PRR3*
							ENSSSCG00000021714	+	ORF	*PRR3*
7	26801875	26801879	+	26801878	35	53	ENSSSCG00000001346	+	intron	*ABCF1*
7	26803196	26803196	+	26803196	5	5	ENSSSCG00000001346	+	intron	*ABCF1*
7	26813597	26813635	+	26813599	67	188	ENSSSCG00000001346	+	3'UTR	*ABCF1*
7	26818444	26818444	-	26818444	5	5	ENSSSCG00000001347	-	3'UTR	*FB19*
7	26833611	26833611	-	26833611	3	3	ENSSSCG00000001347	-	intron	*FB19*
7	26834623	26834659	-	26834659	5	11	---	---	intergenic	---

7	26912218	26912218	+	26912218	7	7	ENSSSCG00000025943	+	3'UTR	*C6orf136*
							ENSSSCG00000025943	+	ORF	---
7	26912272	26912272	-	26912272	9	9	ENSSSCG00000001376	-	3'UTR	*DHX16*
7	26933736	26933739	-	26933737	17	37	ENSSSCG00000001377	-	ORF	*PPP1R18*
7	26944256	26944262	-	26944260	6	14	ENSSSCG00000023639	-	3'UTR	*NRM*
7	26951469	26951472	-	26951469	7	10	ENSSSCG00000001362	-	3'UTR	*MDC1*
7	26973424	26973428	+	26973427	43	81	ENSSSCG00000001379	+	3'UTR	*TUBB*
7	26974194	26974201	+	26974200	236	269	ENSSSCG00000001379	+	3'UTR	*TUBB*
7	26975126	26975136	-	26975133	16	52	ENSSSCG00000022725	-	3'UTR	*FLOT1*
7	26987533	26987533	-	26987533	19	19	ENSSSCG00000027607	-	3'UTR	*IER3*
7	26989825	26989825	-	26989825	5	5	XLOC_080227	-	predicted	---
7	26990922	26990922	+	26990922	9	9	XLOC_079045	+	predicted	---
7	27113808	27113808	+	27113808	5	5	ENSSSCG00000001382	+	3'UTR	*DDR*
7	27126501	27126501	+	27126501	8	8	ENSSSCG00000001383	+	3'UTR	*TFIIH*
7	27138694	27138694	+	27138694	6	6	ENSSSCG00000001384	+	3'UTR	*VARS2*
7	27221802	27221802	-	27221802	3	3	XLOC_080232	-	predicted	---
7	27289647	27289650	+	27289647	3	6	ENSSSCG00000001391	+	intron	*CCHCR1*
7	27289698	27289702	+	27289701	46	89	ENSSSCG00000001391	+	ORF	*CCHCR1*
7	27311813	27311813	+	27311813	3	3	ENSSSCG00000001389	+	ORF	*SBAB-499E6.10*
7	27451016	27451019	+	27451017	46	65	ENSSSCG00000001417	+	3'UTR	*BAT5*
7	27454257	27454257	-	27454257	3	3	XLOC_080238	-	predicted	---
7	27466073	27466087	-	27466076	94	245	ENSSSCG00000001414	-	3'UTR	*CSNK2B*
7	27474834	27474838	+	27474836	44	87	ENSSSCG00000001413	+	3'UTR	*BAT4*
7	27478503	27478509	+	27478503	3	6	---	---	intergenic	---
7	27478636	27478640	-	27478638	142	186	ENSSSCG00000001411	-	3'UTR	*APOM*

7	27485861	27485861	+	27485861	3	3	ENSSSCG00000001410	+	intron	*BAT3*
7	27486960	27486960	+	27486960	3	3	ENSSSCG00000001410	+	intron	*BAT3*
7	27495039	27495079	+	27495076	180	422	ENSSSCG00000001410	+	3'UTR	*BAT3*
7	27495901	27495901	-	27495901	7	7	XLOC_080241	-	predicted	---
7	27520799	27520804	-	27520802	81	169	ENSSSCG00000001408	-	3'UTR	*AIF1*
7	27539011	27539014	-	27539014	15	30	ENSSSCG00000030882	-	3'UTR	*LST1*
							ENSSSCG00000030882	-	ORF	*LST1*
7	27547158	27547162	+	27547160	20	32	ENSSSCG00000001405	+	3'UTR	*LTB*
7	27549601	27549601	-	27549601	11	11	ENSSSCG00000001404	-	3'UTR	*TNFA*
7	27566955	27566957	-	27566957	13	16	ENSSSCG00000001402	-	3'UTR	*NFKBIL1*
7	27581068	27581068	+	27581068	4	4	ENSSSCG00000001401	+	3'UTR	*ATP6V1G2*
							ENSSSCG00000001401	+	ORF	*ATP6V1G2*
7	27593951	27593978	+	27593972	16	36	ENSSSCG00000001400	+	3'UTR	*BAT1*
7	27618074	27618078	+	27618074	3	6	XLOC_079068	+	predicted	---
7	27618176	27618176	+	27618176	3	3	---	---	intergenic	---
7	27626980	27627001	-	27626995	14	43	ENSSSCG00000001396	-	ORF	*SLA-8*
7	27723252	27723266	+	27723257	11	25	ENSSSCG00000023611	+	3'UTR	*TNXB*
							ENSSSCG00000023611	+	ORF	---
7	27729071	27729082	-	27729079	487	1230	ENSSSCG00000001427	-	3'UTR	*C4A*
7	27729528	27729529	-	27729528	3	6	ENSSSCG00000001427	-	ORF	*C4A*
7	27734927	27734927	-	27734927	3	3	ENSSSCG00000001427	-	ORF	*C4A*
7	27741019	27741019	-	27741019	10	10	ENSSSCG00000001427	-	ORF	*C4A*
7	27744803	27744817	-	27744814	32	51	ENSSSCG00000001426	-	3'UTR	*STK19*
7	27754522	27754530	+	27754522	12	35	ENSSSCG00000001425	+	3'UTR	*DOM3Z*
7	27754582	27754596	-	27754582	13	41	ENSSSCG00000001424	-	3'UTR	*SKIV2L*
7	27765890	27765894	+	27765894	30	48	ENSSSCG00000001423	+	ORF	*NELFE*

7	27771019	27771023	-	27771020	35	68	---	---	intergenic	---
7	27771531	27771534	+	27771532	43	63	ENSSSCG00000001423	+	3'UTR	*NELFE*
7	27771669	27771675	+	27771674	20	53	ENSSSCG00000001423	+	3'UTR	*NELFE*
7	27771676	27771717	-	27771682	1675	3003	XLOC_080254	-	predicted	---
7	27772392	27772392	-	27772392	3	3	ENSSSCG00000024914	-	ORF	*BF*
7	27772556	27772556	-	27772556	3	3	ENSSSCG00000024914	-	ORF	*BF*
7	27772868	27772868	-	27772868	3	3	ENSSSCG00000024914	-	ORF	*BF*
7	27772903	27772903	+	27772903	3	3	---	---	intergenic	---
7	27773076	27773077	-	27773076	14	21	ENSSSCG00000024914	-	ORF	*BF*
7	27773099	27773099	+	27773099	3	3	---	---	intergenic	---
7	27773128	27773128	-	27773128	24	24	ENSSSCG00000024914	-	ORF	*BF*
7	27774513	27774513	-	27774513	24	24	---	---	intergenic	---
7	27775381	27775381	-	27775381	7	7	---	---	intergenic	---
7	27776014	27776014	-	27776014	3	3	ENSSSCG00000024914	-	intron	*BF*
7	27777249	27777249	-	27777249	5	5	---	---	intergenic	---
7	27778160	27778195	-	27778163	185	348	ENSSSCG00000001422	-	3'UTR	*C2*
7	27778556	27778556	-	27778556	4	4	ENSSSCG00000001422	-	ORF	*C2*
7	27779718	27779718	-	27779718	3	3	---	---	intergenic	---
7	27784771	27784771	-	27784771	4	4	---	---	intergenic	---
7	27785575	27785575	-	27785575	7	7	---	---	intergenic	---
7	27834338	27834342	+	27834339	12	21	ENSSSCG00000001420	+	3'UTR	*EHMT2*
7	27858548	27858573	+	27858573	7	19	XLOC_079076	+	predicted	---
7	27859434	27859435	+	27859435	7	11	XLOC_079076	+	predicted	---
7	27981855	27981856	-	27981855	12	15	XLOC_080256	-	predicted	---
7	27993037	27993042	-	27993041	9	25	ENSSSCG00000001431	-	3'UTR	*FKBPL*
7	27994052	27994052	-	27994052	3	3	ENSSSCG00000001431	-	ORF	*FKBPL*

7	27996013	27996013	+	27996013	3	3	XLOC_079078	+	predicted	---
7	27996125	27996125	+	27996125	4	4	XLOC_079078	+	predicted	---
7	28018187	28018187	+	28018187	3	3	ENSSSCG00000001433	+	intron	*PPT2*
7	28025727	28025736	-	28025728	13	21	XLOC_080259	-	predicted	---
7	28025799	28025799	+	28025799	4	4	ENSSSCG00000001434	+	3'UTR	*EGFL8*
							ENSSSCG00000001434	+	ORF	---
7	28038809	28038814	+	28038810	35	53	ENSSSCG00000001436	+	3'UTR	*RNF5*
7	28042407	28042413	-	28042407	5	9	XLOC_080261	-	predicted	---
7	28043228	28043228	-	28043228	3	3	XLOC_080261	-	predicted	---
7	28052186	28052187	-	28052186	9	13	ENSSSCG00000001440	-	3'UTR	*NOTCH4*
7	28132584	28132584	+	28132584	4	4	---	---	intergenic	---
7	28250588	28250588	+	28250588	3	3	---	---	intergenic	---
7	29042136	29042148	+	29042146	291	580	ENSSSCG00000001453	+	3'UTR	*SLA-DRA1*
7	29046222	29046222	-	29046222	4	4	---	---	intergenic	---
7	29108720	29108720	-	29108720	6	6	XLOC_080279	-	predicted	---
7	29117864	29117864	-	29117864	12	12	---	---	intergenic	---
7	29123731	29123731	-	29123731	8	8	ENSSSCG00000001455	-	intron	*SLA-DRB*
7	29163626	29163626	-	29163626	5	5	---	---	intergenic	---
7	29170645	29170661	+	29170659	212	520	ENSSSCG00000001456	+	3'UTR	*SLA-DQA1*
7	29170807	29170811	+	29170807	32	45	XLOC_079093	+	predicted	---
7	29180529	29180536	-	29180534	264	369	XLOC_080280	-	predicted	---
7	29180588	29180596	-	29180588	12	24	XLOC_080280	-	predicted	---
7	29188763	29188763	+	29188763	5	5	XLOC_079093	+	predicted	---
7	29248472	29248474	-	29248472	10	23	ENSSSCG00000001459	-	intron	---
7	29485884	29485894	-	29485893	117	179	ENSSSCG00000001469	-	3'UTR	*SLA-DMB*
7	29500103	29500109	-	29500109	51	72	ENSSSCG00000001470	-	3'UTR	*SLA-DMA*

7	29523473	29523473	+	29523473	5	5	ENSSSCG00000001471	+	intron	*BRD2*
7	29525648	29525663	+	29525655	35	77	ENSSSCG00000001471	+	intron	*BRD2*
7	29526591	29526591	+	29526591	3	3	---	---	intergenic	---
7	29526806	29526806	+	29526806	15	15	---	---	intergenic	---
7	29526932	29526932	+	29526932	3	3	---	---	intergenic	---
7	29527953	29527953	+	29527953	6	6	---	---	intergenic	---
7	29528322	29528322	+	29528322	4	4	---	---	intergenic	---
7	29529195	29529196	+	29529195	49	61	ENSSSCG00000001471	+	3'UTR	*BRD2*
7	29546334	29546334	-	29546334	4	4	ENSSSCG00000001472	-	3'UTR	*SLA*
7	29671465	29671465	-	29671465	20	20	---	---	intergenic	---
7	29727203	29727204	-	29727204	28	32	---	---	intergenic	---
7	29748099	29748099	-	29748099	18	18	ENSSSCG00000001477	-	ORF	*RING1*
7	29753836	29753844	-	29753838	26	56	ENSSSCG00000001476	-	3'UTR	*HSD17B8*
7	29756213	29756225	-	29756219	27	100	XLOC_080297	-	predicted	---
7	29767593	29767617	+	29767617	14	30	ENSSSCG00000001474	+	3'UTR	*RXRB*
7	29904223	29904223	+	29904223	3	3	ENSSSCG00000001478	+	intron	*BMP5*
7	30414105	30414105	-	30414105	3	3	---	---	intergenic	---
7	30560503	30560504	-	30560504	25	30	ENSSSCG00000001483	-	intron	*FAM83B*
7	30926161	30926161	+	30926161	16	16	XLOC_079106	+	predicted	---
7	30953529	30953529	-	30953529	4	4	---	---	intergenic	---
7	30981565	30981565	+	30981565	3	3	ENSSSCG00000001484	+	intron	---
7	31302544	31302545	-	31302545	13	22	ENSSSCG00000001486	-	3'UTR	*LRRC1*
7	31302827	31302827	-	31302827	6	6	ENSSSCG00000001486	-	3'UTR	*LRRC1*
7	31303485	31303485	-	31303485	8	8	ENSSSCG00000001486	-	3'UTR	*LRRC1*
7	31303658	31303667	-	31303667	8	18	ENSSSCG00000001486	-	3'UTR	*LRRC1*
7	31326176	31326176	-	31326176	3	3	---	---	intergenic	---

7	31329613	31329613	-	31329613	3	3	ENSSSCG00000001486	-	intron	*LRRC1*
7	31599285	31599293	+	31599285	5	8	XLOC_079108	+	predicted	---
7	31747276	31747285	+	31747276	4	7	ENSSSCG00000001488	+	3'UTR	*GCLC*
7	31747427	31747433	+	31747427	13	23	ENSSSCG00000001488	+	3'UTR	*GCLC*
7	31757209	31757209	-	31757209	4	4	XLOC_080309	-	predicted	---
7	31758463	31758463	-	31758463	4	4	---	---	intergenic	---
7	31786201	31786201	-	31786201	4	4	---	---	intergenic	---
7	31800635	31800635	+	31800635	6	6	XLOC_079111	+	predicted	---
7	32506789	32506789	+	32506789	3	3	---	---	intergenic	---
7	32581933	32581934	-	32581934	18	35	XLOC_080315	-	predicted	---
7	32750199	32750214	-	32750199	6	14	ENSSSCG00000001493	-	3'UTR	---
7	33024428	33024428	-	33024428	3	3	---	---	intergenic	---
7	33129074	33129085	-	33129078	7	33	XLOC_080318	-	predicted	---
7	33142451	33142451	-	33142451	6	6	XLOC_080318	-	predicted	---
7	33144352	33144352	-	33144352	6	6	XLOC_080318	-	predicted	---
7	33165339	33165345	-	33165343	16	45	ENSSSCG00000001497	-	intron	*ZNF451*
7	33227220	33227243	+	33227242	22	37	---	---	intergenic	---
7	33399762	33399762	-	33399762	3	3	---	---	intergenic	---
7	33615996	33615996	+	33615996	3	3	ENSSSCG00000001499	+	ORF	*DST*
7	33677565	33677567	+	33677565	5	8	ENSSSCG00000001499	+	ORF	*DST*
7	33690377	33690377	+	33690377	5	5	ENSSSCG00000001499	+	ORF	*DST*
7	33751046	33751057	+	33751054	11	39	ENSSSCG00000001499	+	3'UTR	*DST*
7	33751652	33751658	+	33751652	7	22	ENSSSCG00000001499	+	3'UTR	*DST*
7	33751906	33751906	+	33751906	3	3	XLOC_079116	+	predicted	---
7	33899501	33899501	-	33899501	4	4	---	---	intergenic	---
7	34112805	34112805	+	34112805	3	3	XLOC_079117	+	predicted	---

7	34127994	34127996	-	34127995	5	12	XLOC_080322	-	predicted	---
7	34146669	34146681	+	34146677	461	738	ENSSSCG00000001502	+	3'UTR	*RPS18*
7	34148607	34148607	+	34148607	3	3	ENSSSCG00000001503	+	3'UTR	*B3GALT4*
							ENSSSCG00000001503	+	ORF	---
7	34148894	34148894	-	34148894	4	4	ENSSSCG00000001504	-	3'UTR	*WDR46*
7	34158688	34158708	+	34158688	7	27	ENSSSCG00000001505	+	3'UTR	*KE2*
7	34159503	34159514	-	34159509	11	29	ENSSSCG00000001506	-	3'UTR	*RGL2*
7	34160627	34160627	+	34160627	7	7	XLOC_079119	+	predicted	---
7	34167260	34167263	-	34167262	49	72	XLOC_080323	-	predicted	---
7	34167908	34167908	-	34167908	3	3	ENSSSCG00000001507	-	intron	*TAPBP*
7	34170591	34170591	-	34170591	3	3	ENSSSCG00000001507	-	intron	*TAPBP*
7	34180625	34180629	-	34180627	69	104	ENSSSCG00000001509	-	3'UTR	*DAXX*
7	34186146	34186151	+	34186148	4	10	XLOC_079121	+	predicted	---
7	34196856	34196861	-	34196861	8	11	XLOC_080325	-	predicted	---
7	34197086	34197086	-	34197086	3	3	XLOC_080325	-	predicted	---
7	34273782	34273788	-	34273785	25	62	ENSSSCG00000001517	-	3'UTR	---
7	34286286	34286286	+	34286286	15	15	ENSSSCG00000001516	+	3'UTR	*BAK1*
7	34326208	34326212	-	34326210	69	109	---	---	intergenic	---
7	34326240	34326242	+	34326242	6	9	---	---	intergenic	---
7	34337576	34337576	-	34337576	6	6	XLOC_080327	-	predicted	---
7	34340879	34340879	+	34340879	3	3	---	---	intergenic	---
7	34375036	34375041	+	34375039	102	288	ENSSSCG00000001512	+	3'UTR	*CUTA*
7	34375144	34375147	-	34375147	15	22	ENSSSCG00000001511	-	3'UTR	*PHF1*
7	34381421	34381421	-	34381421	3	3	ENSSSCG00000028301	-	3'UTR	*KIFC1*
7	34489851	34489851	+	34489851	3	3	ENSSSCG00000001518	+	intron	*ITPR3*
7	34509040	34509043	+	34509040	5	8	ENSSSCG00000001518	+	3'UTR	*ITPR3*

7	34509090	34509094	+	34509090	5	8	ENSSSCG00000001518	+	3'UTR	*ITPR3*
7	34509195	34509195	-	34509195	3	3	ENSSSCG00000030668	-	3'UTR	*UQCC2*
7	34509859	34509877	-	34509876	695	1170	ENSSSCG00000030668	-	3'UTR	*UQCC2*
7	34640190	34640190	-	34640190	7	7	XLOC_080333	-	predicted	---
7	34640362	34640362	-	34640362	5	5	XLOC_080333	-	predicted	---
7	34652059	34652062	-	34652062	6	16	ENSSSCG00000001520	-	intron	*LEMD2*
7	34713499	34713501	-	34713499	5	8	XLOC_080335	-	predicted	---
7	34765963	34765963	+	34765963	4	4	---	---	intergenic	---
7	34887690	34887691	-	34887690	13	22	ENSSSCG00000001523	-	intron	*GRM4*
7	34990086	34990088	+	34990088	18	27	ENSSSCG00000001526	+	3'UTR	*HMGA1*
7	34990227	34990227	-	34990227	6	6	ENSSSCG00000023160	-	3'UTR	---
7	35003932	35003934	-	35003932	3	6	XLOC_080340	-	predicted	---
7	35011553	35011562	-	35011562	5	9	XLOC_080340	-	predicted	---
7	35110344	35110364	-	35110347	393	1729	ENSSSCG00000001528	-	3'UTR	*RPS10*
7	35188210	35188227	+	35188225	57	150	XLOC_079135	+	predicted	---
7	35505300	35505300	+	35505300	3	3	ENSSSCG00000001532	+	intron	*UHRF1BP1*
7	35538913	35538913	+	35538913	4	4	XLOC_079139	+	predicted	---
7	35543719	35543723	-	35543720	19	52	ENSSSCG00000001533	-	3'UTR	*TAF11*
7	35555484	35555489	+	35555488	9	23	---	---	intergenic	---
7	35555534	35555534	+	35555534	3	3	---	---	intergenic	---
7	35769383	35769383	+	35769383	3	3	XLOC_079140	+	predicted	---
7	35769625	35769633	+	35769625	6	9	XLOC_079140	+	predicted	---
7	35780242	35780243	-	35780242	35	38	---	---	intergenic	---
7	35783075	35783086	-	35783080	126	201	XLOC_080350	-	predicted	---
7	35941174	35941174	-	35941174	5	5	ENSSSCG00000001536	-	intron	---
7	35949601	35949601	-	35949601	3	3	ENSSSCG00000001536	-	intron	---

7	35955514	35955514	-	35955514	3	3	ENSSSCG00000001536	-	intron	---
7	36106591	36106626	-	36106603	515	1417	ENSSSCG00000001543	-	3'UTR	*RPL10A*
7	36108334	36108334	-	36108334	4	4	ENSSSCG00000001543	-	ORF	*RPL10A*
7	36110485	36110485	-	36110485	3	3	XLOC_080355	-	predicted	---
7	36141643	36141643	-	36141643	5	5	ENSSSCG00000001539	-	3'UTR	*PPARD*
7	36144791	36144791	-	36144791	3	3	ENSSSCG00000001539	-	ORF	*PPARD*
7	36220770	36220791	-	36220791	6	14	ENSSSCG00000001538	-	3'UTR	*DEF6*
7	36244885	36244886	-	36244886	8	14	XLOC_080358	-	predicted	---
7	36370206	36370206	-	36370206	3	3	XLOC_080360	-	predicted	---
7	36370564	36370581	-	36370570	24	53	XLOC_080360	-	predicted	---
7	36370910	36370910	-	36370910	3	3	XLOC_080360	-	predicted	---
7	36372042	36372042	-	36372042	5	5	XLOC_080360	-	predicted	---
7	36372103	36372103	-	36372103	3	3	XLOC_080360	-	predicted	---
7	36372143	36372143	-	36372143	6	6	XLOC_080360	-	predicted	---
7	36501614	36501615	-	36501614	19	25	XLOC_080361	-	predicted	---
7	36529658	36529675	+	36529663	54	136	XLOC_079152	+	predicted	---
7	36573641	36573649	-	36573649	5	9	XLOC_080363	-	predicted	---
7	36573713	36573713	-	36573713	5	5	XLOC_080363	-	predicted	---
7	36575410	36575420	-	36575418	10	28	XLOC_080363	-	predicted	---
7	36575575	36575575	-	36575575	4	4	XLOC_080363	-	predicted	---
7	36660010	36660034	-	36660015	43	139	XLOC_080364	-	predicted	---
7	36662242	36662242	-	36662242	3	3	ENSSSCG00000001555	-	ORF	*SLC26A8*
7	36713573	36713584	-	36713580	14	51	ENSSSCG00000001555	-	intron	*SLC26A8*
7	36794356	36794359	+	36794359	14	17	ENSSSCG00000001556	+	3'UTR	*MAPK14*
7	36795307	36795314	+	36795313	27	44	XLOC_079154	+	predicted	---
7	37095584	37095586	+	37095586	5	12	XLOC_079158	+	predicted	---

7	37107045	37107045	-	37107045	8	8	ENSSSCG00000001563	-	3'UTR	*STK38*
7	37198098	37198098	+	37198098	3	3	ENSSSCG00000001564	+	3'UTR	*SRSF3*
7	37198256	37198257	+	37198257	8	15	ENSSSCG00000001564	+	3'UTR	*SRSF3*
7	37198500	37198500	+	37198500	6	6	ENSSSCG00000001564	+	3'UTR	*SRSF3*
7	37198736	37198758	+	37198740	30	97	ENSSSCG00000001564	+	3'UTR	*SRSF3*
7	37198890	37198900	+	37198891	9	18	XLOC_079159	+	predicted	---
7	37199364	37199367	+	37199364	5	10	XLOC_079159	+	predicted	---
7	37200705	37200705	+	37200705	4	4	XLOC_079159	+	predicted	---
7	37231655	37231655	-	37231655	3	3	---	---	intergenic	---
7	37250725	37250731	+	37250726	18	56	---	---	intergenic	---
7	37267149	37267155	+	37267149	28	32	ENSSSCG00000022111	+	3'UTR	---
7	37337850	37337850	-	37337850	6	6	ENSSSCG00000001567	-	intron	*CPNE5*
7	37426213	37426216	-	37426213	21	38	ENSSSCG00000030390	-	3'UTR	*PPIL1*
7	37482711	37482711	+	37482711	5	5	ENSSSCG00000001569	+	3'UTR	*C6orf89*
7	37483606	37483634	+	37483634	9	13	XLOC_079162	+	predicted	---
7	37487777	37487783	+	37487780	25	59	XLOC_079162	+	predicted	---
7	37516633	37516633	+	37516633	3	3	XLOC_079163	+	predicted	---
7	37517196	37517196	+	37517196	5	5	XLOC_079163	+	predicted	---
7	37520861	37520887	-	37520867	131	246	ENSSSCG00000001571	-	3'UTR	*MTCH1*
7	37551388	37551388	-	37551388	4	4	---	---	intergenic	---
7	37577984	37577984	+	37577984	8	8	ENSSSCG00000001572	+	3'UTR	*FGD2*
7	37602294	37602294	+	37602294	3	3	---	---	intergenic	---
7	37637703	37637704	-	37637704	15	19	---	---	intergenic	---
7	37659009	37659033	+	37659009	6	9	XLOC_079165	+	predicted	---
7	37659259	37659259	+	37659259	6	6	XLOC_079165	+	predicted	---
7	37696712	37696724	+	37696724	8	17	ENSSSCG00000001573	+	3'UTR	*PIM1*

7	37697468	37697470	+	37697469	23	43	XLOC_079166	+	predicted	---
7	37705254	37705254	-	37705254	4	4	---	---	intergenic	---
7	37705288	37705288	+	37705288	14	14	---	---	intergenic	---
7	37721325	37721326	-	37721325	10	15	XLOC_080379	-	predicted	---
7	37728391	37728400	-	37728400	29	55	XLOC_080379	-	predicted	---
7	38429936	38429937	-	38429936	6	10	---	---	intergenic	---
7	38687086	38687086	+	38687086	3	3	---	---	intergenic	---
7	38689441	38689441	+	38689441	4	4	---	---	intergenic	---
7	38836389	38836389	+	38836389	3	3	ENSSSCG00000001584	+	intron	*ZFAND3*
7	38921731	38921731	+	38921731	3	3	---	---	intergenic	---
7	38940830	38940836	+	38940830	15	25	XLOC_079171	+	predicted	---
7	38942679	38942680	+	38942679	9	12	XLOC_079171	+	predicted	---
7	38948116	38948116	-	38948116	17	17	ENSSSCG00000030073	-	3'UTR	*BTBD9*
7	39055231	39055231	-	39055231	5	5	ENSSSCG00000030073	-	intron	*BTBD9*
7	39218712	39218712	+	39218712	3	3	XLOC_079173	+	predicted	---
7	39244140	39244149	-	39244149	27	56	ENSSSCG00000027778	-	3'UTR	*GLO1*
7	39245205	39245207	-	39245207	5	12	ENSSSCG00000027778	-	3'UTR	*GLO1*
7	39245249	39245251	-	39245251	14	23	ENSSSCG00000027778	-	3'UTR	*GLO1*
7	39569419	39569423	+	39569423	9	24	XLOC_079174	+	predicted	---
7	39704469	39704469	-	39704469	3	3	ENSSSCG00000025423	-	3'UTR	*KCNK5*
7	39951134	39951134	+	39951134	3	3	---	---	intergenic	---
7	40344508	40344508	-	40344508	7	7	---	---	intergenic	---
7	40385740	40385740	+	40385740	12	12	ENSSSCG00000001595	+	intron	*DAAM2*
7	40416839	40416840	-	40416839	6	10	ENSSSCG00000001596	-	3'UTR	*MOCS1*
7	40682554	40682555	-	40682554	7	10	---	---	intergenic	---
7	40742902	40742902	-	40742902	3	3	XLOC_080401	-	predicted	---

7	41353660	41353666	-	41353663	20	53	---	---	intergenic	---
7	41747032	41747040	+	41747033	20	49	---	---	intergenic	---
7	41752086	41752086	-	41752086	8	8	ENSSSCG00000001613	-	3'UTR	*TREML1*
7	41777879	41777879	-	41777879	7	7	XLOC_080414	-	predicted	---
7	41804121	41804125	-	41804122	9	21	ENSSSCG00000001617	-	3'UTR	*TREM-1*
7	41830762	41830762	+	41830762	8	8	---	---	intergenic	---
7	41874574	41874576	-	41874575	48	57	XLOC_080416	-	predicted	---
7	41929004	41929004	+	41929004	3	3	---	---	intergenic	---
7	42088628	42088633	+	42088633	6	9	ENSSSCG00000001619	+	3'UTR	*FOXP4*
7	42286644	42286644	+	42286644	3	3	XLOC_079199	+	predicted	---
7	42294089	42294089	+	42294089	3	3	---	---	intergenic	---
7	42467417	42467422	-	42467421	4	11	XLOC_080425	-	predicted	---
7	42477108	42477108	+	42477108	3	3	ENSSSCG00000001631	+	intron	*BYSL*
7	42486726	42486735	+	42486727	21	31	XLOC_079205	+	predicted	---
7	42488135	42488141	-	42488138	37	74	ENSSSCG00000026752	-	3'UTR	*CCND3*
7	42500786	42500787	-	42500786	12	16	---	---	intergenic	---
7	42506510	42506510	-	42506510	3	3	---	---	intergenic	---
7	42506544	42506544	+	42506544	3	3	---	---	intergenic	---
7	42522649	42522651	+	42522649	8	15	---	---	intergenic	---
7	42558702	42558713	-	42558710	74	164	XLOC_080427	-	predicted	---
7	42697752	42697754	-	42697752	3	6	---	---	intergenic	---
7	42757835	42757835	+	42757835	6	6	ENSSSCG00000001636	+	intron	*GUCA1A*
7	42761827	42761827	+	42761827	5	5	---	---	intergenic	---
7	42774441	42774458	-	42774454	26	71	ENSSSCG00000001638	-	3'UTR	*MRPS10*
7	42774947	42774947	-	42774947	5	5	ENSSSCG00000001638	-	3'UTR	*MRPS10*
7	42774987	42774989	-	42774987	3	9	ENSSSCG00000001638	-	3'UTR	*MRPS10*

7	43073958	43073958	-	43073958	8	8	---	---	intergenic	---
7	43102150	43102150	-	43102150	5	5	---	---	intergenic	---
7	43238865	43238866	-	43238865	4	8	---	---	intergenic	---
7	43248559	43248566	-	43248561	15	39	XLOC_080436	-	predicted	---
7	43255488	43255488	-	43255488	8	8	XLOC_080436	-	predicted	---
7	43255526	43255530	+	43255526	4	7	---	---	intergenic	---
7	43275205	43275206	-	43275206	24	29	XLOC_080437	-	predicted	---
7	43278564	43278564	-	43278564	19	19	ENSSSCG00000023960	-	intron	---
7	43293818	43293818	-	43293818	6	6	---	---	intergenic	---
7	43381962	43381962	+	43381962	5	5	ENSSSCG00000001646	+	3'UTR	*GLTSCR1L*
7	43395607	43395607	+	43395607	3	3	ENSSSCG00000026472	+	3'UTR	*RPL7L1*
7	43395669	43395672	+	43395671	38	68	ENSSSCG00000026472	+	3'UTR	*RPL7L1*
7	43435110	43435117	-	43435110	6	21	XLOC_080442	-	predicted	---
7	43472343	43472354	+	43472348	19	82	ENSSSCG00000001651	+	3'UTR	*CNPY3*
7	43488887	43488890	+	43488890	9	13	ENSSSCG00000001652	+	ORF	*GNMT*
7	43489573	43489586	+	43489584	117	471	ENSSSCG00000001652	+	3'UTR	*GNMT*
7	43526674	43526704	-	43526677	476	1758	ENSSSCG00000001655	-	3'UTR	*MEA1*
7	43526783	43526786	+	43526784	12	22	ENSSSCG00000001654	+	3'UTR	*PPP2R5D*
7	43526801	43526801	-	43526801	5	5	ENSSSCG00000001655	-	3'UTR	*MEA1*
7	43535534	43535537	+	43535535	22	36	XLOC_079222	+	predicted	---
7	43541322	43541325	+	43541323	66	84	ENSSSCG00000001656	+	3'UTR	*C7H6ORF153*
7	43541413	43541413	+	43541413	3	3	ENSSSCG00000001656	+	3'UTR	*C7H6ORF153*
7	43616541	43616545	-	43616543	64	95	ENSSSCG00000001658	-	3'UTR	*MRPL2*
7	43620511	43620512	-	43620512	4	7	ENSSSCG00000001658	-	intron	*MRPL2*
7	43634893	43634906	+	43634893	12	16	ENSSSCG00000001659	+	3'UTR	*KLC4*
7	43667396	43667396	-	43667396	17	17	---	---	intergenic	---

7	43724047	43724048	+	43724047	3	6	ENSSSCG00000001661	+	3'UTR	*SRF*
7	43764179	43764181	-	43764180	48	61	ENSSSCG00000026568	-	3'UTR	*DNPH1*
7	43771490	43771490	-	43771490	5	5	XLOC_080449	-	predicted	---
7	43835218	43835228	+	43835222	93	127	ENSSSCG00000027796	+	3'UTR	*SLC22A7*
7	43859099	43859099	-	43859099	4	4	ENSSSCG00000001667	-	3'UTR	*ZNF318*
7	43859873	43859876	-	43859873	8	12	ENSSSCG00000001667	-	3'UTR	*ZNF318*
7	44082574	44082583	+	44082574	3	6	ENSSSCG00000001688	+	3'UTR	---
7	44083022	44083022	-	44083022	11	11	ENSSSCG00000001676	-	3'UTR	*LRRC73*
7	44087698	44087723	-	44087699	13	33	ENSSSCG00000001686	-	3'UTR	*YIPF3*
7	44087955	44087959	-	44087958	19	57	ENSSSCG00000001686	-	3'UTR	*YIPF3*
7	44111759	44111759	-	44111759	3	3	XLOC_080457	-	predicted	---
7	44111871	44111873	+	44111872	22	33	ENSSSCG00000028007	+	3'UTR	*POLR1C*
7	44112958	44112963	-	44112960	10	23	XLOC_080457	-	predicted	---
7	44113905	44113905	-	44113905	4	4	ENSSSCG00000001679	-	3'UTR	---
7	44122603	44122608	-	44122608	9	16	ENSSSCG00000001679	-	intron	---
7	44163111	44163111	+	44163111	4	4	ENSSSCG00000001683	+	intron	*POLH*
7	44204813	44204814	+	44204813	11	14	ENSSSCG00000029217	+	3'UTR	---
7	44475703	44475708	+	44475703	7	10	XLOC_079235	+	predicted	---
7	44942293	44942299	-	44942299	7	11	ENSSSCG00000001696	-	3'UTR	*MRPL14*
7	44942467	44942477	-	44942474	110	218	ENSSSCG00000001696	-	3'UTR	*MRPL14*
7	45008830	45008839	+	45008838	13	51	XLOC_079238	+	predicted	---
7	45102580	45102580	+	45102580	3	3	ENSSSCG00000001700	+	3'UTR	*SLC29A1*
7	45115993	45115994	+	45115993	3	6	ENSSSCG00000001701	+	ORF	*HSPCB*
7	45116175	45116175	+	45116175	3	3	ENSSSCG00000001701	+	ORF	*HSPCB*
7	45116226	45116254	+	45116226	6	11	ENSSSCG00000001701	+	ORF	*HSPCB*
7	45116374	45116374	+	45116374	3	3	ENSSSCG00000001701	+	ORF	*HSPCB*

7	45118473	45118476	+	45118473	8	17	ENSSSCG00000001701	+	ORF	*HSPCB*
7	45119929	45119937	+	45119933	108	303	ENSSSCG00000001701	+	3'UTR	*HSPCB*
7	45120581	45120582	-	45120582	8	12	ENSSSCG00000001702	-	3'UTR	*SLC35B2*
7	45124531	45124531	-	45124531	3	3	ENSSSCG00000001703	-	3'UTR	*NFKBIE*
7	45124714	45124714	-	45124714	9	9	ENSSSCG00000001703	-	3'UTR	*NFKBIE*
7	45145063	45145065	-	45145065	12	15	XLOC_080465	-	predicted	---
7	45303520	45303520	+	45303520	3	3	XLOC_079244	+	predicted	---
7	45317507	45317509	+	45317508	9	17	ENSSSCG00000001708	+	intron	*CDC5L*
7	45319128	45319128	+	45319128	4	4	ENSSSCG00000001708	+	ORF	*CDC5L*
7	45319242	45319244	+	45319244	10	22	ENSSSCG00000001708	+	intron	*CDC5L*
7	45405240	45405240	+	45405240	3	3	---	---	intergenic	---
7	45471724	45471724	-	45471724	4	4	---	---	intergenic	---
7	45485875	45485875	-	45485875	3	3	---	---	intergenic	---
7	45633232	45633236	-	45633236	45	69	---	---	intergenic	---
7	46061921	46061921	+	46061921	3	3	---	---	intergenic	---
7	46110772	46110774	-	46110774	8	14	---	---	intergenic	---
7	46412670	46412670	+	46412670	13	13	ENSSSCG00000025551	+	intron	---
7	46895609	46895611	-	46895609	9	16	---	---	intergenic	---
7	47190335	47190335	+	47190335	6	6	---	---	intergenic	---
7	47237447	47237447	+	47237447	3	3	ENSSSCG00000001715	+	ORF	*ENPP5*
7	47249971	47249979	+	47249978	11	23	ENSSSCG00000001715	+	3'UTR	*ENPP5*
7	47760146	47760146	-	47760146	5	5	---	---	intergenic	---
7	48120888	48120888	+	48120888	4	4	ENSSSCG00000001720	+	3'UTR	---
7	48122698	48122698	+	48122698	3	3	---	---	intergenic	---
7	48145806	48145813	+	48145812	12	25	XLOC_079263	+	predicted	---
7	48149039	48149052	-	48149047	10	42	ENSSSCG00000001723	-	3'UTR	*PAF-AH*

7	48149120	48149121	-	48149121	7	12	ENSSSCG00000001723	-	3'UTR	*PAF-AH*
7	48443689	48443689	+	48443689	3	3	---	---	intergenic	---
7	48504753	48504753	+	48504753	3	3	---	---	intergenic	---
7	48598270	48598273	-	48598270	4	10	---	---	intergenic	---
7	48679152	48679158	-	48679158	11	20	---	---	intergenic	---
7	48680219	48680219	-	48680219	5	5	---	---	intergenic	---
7	48680341	48680341	-	48680341	3	3	---	---	intergenic	---
7	48787415	48787415	+	48787415	3	3	XLOC_079268	+	predicted	---
7	48861445	48861445	+	48861445	3	3	---	---	intergenic	---
7	49057775	49057779	+	49057778	13	29	---	---	intergenic	---
7	49252294	49252294	-	49252294	3	3	---	---	intergenic	---
7	49657211	49657211	+	49657211	5	5	---	---	intergenic	---
7	49851124	49851125	-	49851124	5	9	---	---	intergenic	---
7	50018561	50018561	-	50018561	4	4	XLOC_080490	-	predicted	---
7	50065182	50065195	-	50065191	495	797	ENSSSCG00000001736	-	3'UTR	*CRISP2*
7	50065262	50065317	-	50065264	207	512	ENSSSCG00000001736	-	3'UTR	*CRISP2*
7	50065375	50065384	-	50065384	5	8	ENSSSCG00000001736	-	3'UTR	*CRISP2*
7	50065502	50065508	-	50065508	7	10	ENSSSCG00000001736	-	3'UTR	*CRISP2*
7	50065659	50065670	-	50065670	5	9	ENSSSCG00000001736	-	ORF	*CRISP2*
7	50065706	50065706	-	50065706	3	3	ENSSSCG00000001736	-	ORF	*CRISP2*
7	50068236	50068236	-	50068236	5	5	ENSSSCG00000001736	-	intron	*CRISP2*
7	50071033	50071047	-	50071047	25	40	ENSSSCG00000001736	-	ORF	*CRISP2*
7	50076279	50076288	-	50076279	39	56	ENSSSCG00000001736	-	ORF	*CRISP2*
7	50077204	50077204	-	50077204	3	3	ENSSSCG00000001736	-	ORF	*CRISP2*
7	50077272	50077272	-	50077272	3	3	ENSSSCG00000001736	-	intron	*CRISP2*
7	50083892	50083892	-	50083892	3	3	ENSSSCG00000001736	-	intron	*CRISP2*

7	50085005	50085013	+	50085008	7	24	---	---	intergenic	---
7	50107485	50107492	-	50107492	7	11	ENSSSCG00000001732	-	3'UTR	*MUT*
7	50108002	50108006	-	50108003	12	37	ENSSSCG00000001732	-	3'UTR	*MUT*
7	50128625	50128625	-	50128625	3	3	ENSSSCG00000001732	-	intron	*MUT*
7	50146196	50146196	-	50146196	3	3	XLOC_080493	-	predicted	---
7	50199172	50199172	+	50199172	4	4	XLOC_079276	+	predicted	---
7	50231411	50231433	-	50231413	206	478	---	---	intergenic	---
7	50231982	50231982	-	50231982	5	5	ENSSSCG00000001738	-	ORF	---
7	50278645	50278645	-	50278645	3	3	ENSSSCG00000001739	-	intron	*CRISP1*
7	50318836	50318836	+	50318836	10	10	---	---	intergenic	---
7	50379817	50379817	-	50379817	12	12	ENSSSCG00000001741	-	intron	*BD114*
7	50401130	50401130	-	50401130	3	3	---	---	intergenic	---
7	51181415	51181415	-	51181415	4	4	---	---	intergenic	---
7	51502694	51502695	+	51502694	14	19	---	---	intergenic	---
7	51837219	51837219	-	51837219	9	9	---	---	intergenic	---
7	51848298	51848298	-	51848298	3	3	---	---	intergenic	---
7	51910617	51910617	-	51910617	3	3	---	---	intergenic	---
7	51916462	51916463	+	51916462	3	6	---	---	intergenic	---
7	52281561	52281561	-	52281561	3	3	ENSSSCG00000001746	-	intron	---
7	52410346	52410349	+	52410347	34	65	---	---	intergenic	---
7	52411731	52411732	-	52411732	6	10	---	---	intergenic	---
7	52411743	52411752	+	52411749	8	41	---	---	intergenic	---
7	52477003	52477003	-	52477003	3	3	---	---	intergenic	---
7	52930875	52930875	-	52930875	4	4	ENSSSCG00000001754	-	3'UTR	*PSMA4*
7	52931014	52931024	-	52931014	9	16	ENSSSCG00000001754	-	3'UTR	*PSMA4*
7	52943003	52943003	-	52943003	6	6	ENSSSCG00000001755	-	3'UTR	*HYKK*

7	53156327	53156338	-	53156334	89	366	ENSSSCG00000021873	-	3'UTR	*CRABP1*
7	53206230	53206231	+	53206231	9	12	ENSSSCG00000001757	+	ORF	*WDR61*
7	53213195	53213195	+	53213195	5	5	ENSSSCG00000001757	+	3'UTR	*WDR61*
7	53213298	53213363	+	53213361	141	292	ENSSSCG00000001757	+	3'UTR	*WDR61*
7	53214366	53214369	-	53214367	5	12	ENSSSCG00000001759	-	3'UTR	*DNAJA4*
7	53215889	53215889	-	53215889	4	4	ENSSSCG00000001759	-	3'UTR	*DNAJA4*
7	53216013	53216029	-	53216020	15	45	ENSSSCG00000001759	-	3'UTR	*DNAJA4*
7	53236265	53236265	-	53236265	3	3	---	---	intergenic	---
7	53311937	53311981	+	53311980	17	35	ENSSSCG00000001760	+	3'UTR	*ACSBG1*
7	53312282	53312290	-	53312284	15	28	ENSSSCG00000001761	-	3'UTR	*IDH3A*
7	53313077	53313080	-	53313077	6	9	ENSSSCG00000001761	-	3'UTR	*IDH3A*
7	53313306	53313312	-	53313312	11	18	ENSSSCG00000001761	-	3'UTR	*IDH3A*
7	53365123	53365125	+	53365123	5	8	ENSSSCG00000001762	+	3'UTR	*CIB2*
7	53469688	53469688	-	53469688	5	5	ENSSSCG00000027477	-	3'UTR	---
7	53552518	53552518	-	53552518	4	4	---	---	intergenic	---
7	53603338	53603362	+	53603338	4	7	---	---	intergenic	---
7	53604760	53604765	+	53604765	68	94	ENSSSCG00000001769	+	intron	*MORF4L1*
7	53606474	53606483	+	53606483	8	14	ENSSSCG00000001769	+	intron	*MORF4L1*
7	53607086	53607086	+	53607086	3	3	ENSSSCG00000001769	+	intron	*MORF4L1*
7	53608587	53608587	+	53608587	4	4	---	---	intergenic	---
7	53613445	53613463	+	53613450	21	54	ENSSSCG00000001769	+	3'UTR	*MORF4L1*
7	53613637	53613637	+	53613637	4	4	ENSSSCG00000001769	+	3'UTR	*MORF4L1*
7	53613967	53613975	+	53613974	104	139	ENSSSCG00000001769	+	3'UTR	*MORF4L1*
7	53621750	53621750	-	53621750	6	6	XLOC_080528	-	predicted	---
7	53622226	53622226	-	53622226	3	3	XLOC_080528	-	predicted	---
7	53623061	53623069	-	53623063	174	303	ENSSSCG00000001770	-	3'UTR	*CTSH*

7	53623167	53623171	-	53623171	5	8	ENSSSCG00000001770	-	3'UTR	*CTSH*
7	53648207	53648207	+	53648207	3	3	---	---	intergenic	---
7	53679953	53679953	-	53679953	6	6	ENSSSCG00000001771	-	intron	*RASGRF1*
7	53801652	53801652	+	53801652	3	3	---	---	intergenic	---
7	53975130	53975138	-	53975132	28	75	XLOC_080531	-	predicted	---
7	54269370	54269370	+	54269370	4	4	---	---	intergenic	---
7	54353909	54353910	-	54353909	3	6	XLOC_080534	-	predicted	---
7	54357285	54357292	-	54357291	40	111	XLOC_080534	-	predicted	---
7	54357471	54357471	-	54357471	3	3	XLOC_080534	-	predicted	---
7	54395263	54395273	-	54395263	8	24	ENSSSCG00000001778	-	3'UTR	*BCL2A1*
7	54441297	54441300	+	54441297	9	26	---	---	intergenic	---
7	54528972	54528972	+	54528972	3	3	ENSSSCG00000001779	+	intron	*ZFAND6*
7	54542339	54542340	+	54542339	8	16	ENSSSCG00000001779	+	3'UTR	*ZFAND6*
7	54542953	54542953	+	54542953	3	3	ENSSSCG00000001779	+	3'UTR	*ZFAND6*
7	54554101	54554101	+	54554101	3	3	---	---	intergenic	---
7	54596317	54596341	+	54596333	65	281	ENSSSCG00000001780	+	3'UTR	*FAH*
7	54608383	54608389	-	54608384	10	28	XLOC_080537	-	predicted	---
7	54611443	54611443	-	54611443	4	4	XLOC_080537	-	predicted	---
7	54719726	54719726	-	54719726	4	4	XLOC_080538	-	predicted	---
7	54817087	54817088	+	54817087	34	41	ENSSSCG00000001781	+	intron	*ARNT2*
7	54959558	54959558	+	54959558	5	5	XLOC_079308	+	predicted	---
7	54995167	54995167	+	54995167	9	9	---	---	intergenic	---
7	55044113	55044113	-	55044113	4	4	---	---	intergenic	---
7	55108567	55108569	+	55108569	11	15	ENSSSCG00000001782	+	3'UTR	*ABHD17C*
7	55111207	55111207	+	55111207	3	3	---	---	intergenic	---
7	55297036	55297041	-	55297037	8	19	---	---	intergenic	---

7	55391917	55391929	+	55391927	28	112	ENSSSCG00000021587	+	3'UTR	*MESDC2*
7	55435542	55435544	+	55435542	6	10	XLOC_079315	+	predicted	---
7	55476989	55476989	+	55476989	3	3	---	---	intergenic	---
7	55540091	55540091	+	55540091	3	3	ENSSSCG00000001786	+	3'UTR	*C15orf26*
7	55702246	55702246	+	55702246	3	3	ENSSSCG00000001787	+	3'UTR	*IL-16*
							ENSSSCG00000001787	+	ORF	*IL-16*
7	55814254	55814258	-	55814258	33	76	---	---	intergenic	---
7	55957022	55957022	+	55957022	5	5	---	---	intergenic	---
7	56022466	56022466	-	56022466	3	3	---	---	intergenic	---
7	56207411	56207411	+	56207411	3	3	---	---	intergenic	---
7	56316564	56316564	-	56316564	5	5	---	---	intergenic	---
7	56317506	56317508	+	56317507	51	75	---	---	intergenic	---
7	56398851	56398855	-	56398853	31	46	ENSSSCG00000001791	-	intron	---
7	56431870	56431870	+	56431870	4	4	ENSSSCG00000001792	+	intron	---
7	56434437	56434447	+	56434437	4	7	ENSSSCG00000001792	+	ORF	---
7	56734736	56734737	-	56734737	8	13	---	---	intergenic	---
7	56871738	56871746	-	56871746	9	12	ENSSSCG00000001795	-	3'UTR	*SH3GL3*
7	57145818	57145826	+	57145825	22	74	---	---	intergenic	---
7	57189186	57189186	-	57189186	3	3	---	---	intergenic	---
7	57304906	57304915	-	57304915	6	10	XLOC_080556	-	predicted	---
7	57338263	57338263	-	57338263	3	3	---	---	intergenic	---
7	57365306	57365311	-	57365306	4	7	---	---	intergenic	---
7	57412684	57412684	+	57412684	3	3	ENSSSCG00000001801	+	ORF	*BTBD1*
7	57413685	57413685	+	57413685	6	6	XLOC_079325	+	predicted	---
7	57414363	57414368	+	57414364	20	30	XLOC_079325	+	predicted	---
7	57422952	57422952	+	57422952	16	16	ENSSSCG00000030131	+	3'UTR	*C15orf40*

7	57423481	57423481	+	57423481	5	5	ENSSSCG00000030131	+	3'UTR	*C15orf40*
7	57424205	57424205	+	57424205	3	3	ENSSSCG00000030131	+	3'UTR	*C15orf40*
7	57435588	57435595	-	57435588	3	9	ENSSSCG00000025905	-	3'UTR	*FAM103A1*
7	57435732	57435732	-	57435732	6	6	ENSSSCG00000025905	-	3'UTR	*FAM103A1*
7	57596473	57596473	-	57596473	5	5	ENSSSCG00000001805	-	3'UTR	*WHAMM*
7	57682225	57682225	-	57682225	3	3	XLOC_080561	-	predicted	---
7	57702652	57702652	-	57702652	4	4	---	---	intergenic	---
7	57702979	57702979	-	57702979	3	3	---	---	intergenic	---
7	57742341	57742342	+	57742342	6	9	---	---	intergenic	---
7	57869293	57869294	+	57869293	3	6	---	---	intergenic	---
7	57874551	57874564	+	57874555	632	1425	ENSSSCG00000027358	+	3'UTR	*RPS17*
7	58161240	58161244	-	58161240	3	6	---	---	intergenic	---
7	58162011	58162014	-	58162011	28	42	ENSSSCG00000001814	-	3'UTR	---
7	58162150	58162166	-	58162150	8	11	ENSSSCG00000001814	-	3'UTR	---
7	58163287	58163294	-	58163293	14	25	ENSSSCG00000001814	-	3'UTR	---
7	58189964	58189964	-	58189964	6	6	ENSSSCG00000001814	-	intron	---
7	58232666	58232666	+	58232666	3	3	---	---	intergenic	---
7	58282371	58282372	-	58282372	9	13	---	---	intergenic	---
7	58449540	58449542	+	58449540	11	15	---	---	intergenic	---
7	58498137	58498137	+	58498137	3	3	ENSSSCG00000001817	+	intron	*FURIN*
7	58503970	58503979	+	58503971	28	83	XLOC_079336	+	predicted	---
7	58516270	58516270	+	58516270	17	17	ENSSSCG00000001818	+	3'UTR	*FES*
7	58550678	58550681	+	58550680	11	22	ENSSSCG00000001819	+	3'UTR	*MAN2A2*
7	58557180	58557182	-	58557181	9	17	ENSSSCG00000001820	-	3'UTR	*HDDC3*
7	58577148	58577154	+	58577153	25	32	ENSSSCG00000001821	+	3'UTR	*UNC45A*
7	58588911	58588911	+	58588911	3	3	XLOC_079341	+	predicted	---

7	58665405	58665419	+	58665417	46	170	XLOC_079342	+	predicted	---
7	58703337	58703344	-	58703339	10	26	XLOC_080573	-	predicted	---
7	58703784	58703788	+	58703785	8	22	ENSSSCG00000001824	+	3'UTR	*ZXDC*
7	58820964	58820964	+	58820964	4	4	XLOC_079343	+	predicted	---
7	59485583	59485583	-	59485583	4	4	ENSSSCG00000030597	-	3'UTR	*HAPLN3*
7	59486447	59486447	-	59486447	3	3	ENSSSCG00000030597	-	3'UTR	*HAPLN3*
7	59487309	59487328	-	59487326	24	58	ENSSSCG00000030597	-	3'UTR	*HAPLN3*
7	59504871	59504874	-	59504872	36	64	ENSSSCG00000001834	-	3'UTR	*MFGE8*
7	59629404	59629405	-	59629405	4	7	XLOC_080583	-	predicted	---
7	59629522	59629526	-	59629523	15	22	XLOC_080583	-	predicted	---
7	59661865	59661865	+	59661865	6	6	ENSSSCG00000001835	+	ORF	*ABHD2*
7	59739443	59739443	+	59739443	3	3	ENSSSCG00000001835	+	intron	*ABHD2*
7	59745659	59745679	+	59745662	33	107	XLOC_079351	+	predicted	---
7	59745716	59745741	+	59745716	6	16	XLOC_079351	+	predicted	---
7	59751510	59751524	+	59751510	10	38	XLOC_079351	+	predicted	---
7	59864727	59864730	-	59864730	18	31	XLOC_080587	-	predicted	---
7	59865517	59865517	+	59865517	3	3	XLOC_079353	+	predicted	---
7	59943866	59943868	-	59943868	5	12	XLOC_080588	-	predicted	---
7	60125380	60125380	-	60125380	4	4	XLOC_080591	-	predicted	---
7	60145338	60145339	-	60145338	5	9	ENSSSCG00000027052	-	3'UTR	*PEX11A*
7	60152667	60152667	-	60152667	13	13	ENSSSCG00000027052	-	intron	*PEX11A*
7	60156909	60156909	+	60156909	3	3	XLOC_079358	+	predicted	---
7	60205456	60205461	+	60205461	10	21	XLOC_079358	+	predicted	---
7	60211077	60211083	-	60211079	16	47	ENSSSCG00000001847	-	3'UTR	*MESP1*
7	60226952	60226952	-	60226952	3	3	---	---	intergenic	---
7	60240143	60240151	-	60240146	87	148	ENSSSCG00000001849	-	3'UTR	*APN*

7	60284951	60284967	-	60284963	18	33	ENSSSCG00000030174	-	3'UTR	*AP3S2*
7	60353268	60353282	-	60353269	11	20	---	---	intergenic	---
7	60537504	60537504	+	60537504	3	3	XLOC_079365	+	predicted	---
7	60539334	60539338	-	60539336	51	105	ENSSSCG00000001852	-	3'UTR	*IDH2*
7	60539416	60539420	-	60539416	7	15	ENSSSCG00000001852	-	3'UTR	*IDH2*
7	60589950	60589954	-	60589954	5	8	XLOC_080600	-	predicted	---
7	60618452	60618458	+	60618458	6	19	---	---	intergenic	---
7	60673872	60673872	+	60673872	6	6	ENSSSCG00000022523	+	ORF	---
7	60775349	60775349	-	60775349	4	4	---	---	intergenic	---
7	60775377	60775386	+	60775380	40	153	---	---	intergenic	---
7	60879187	60879187	+	60879187	3	3	XLOC_079369	+	predicted	---
7	60887298	60887307	+	60887298	6	9	XLOC_079369	+	predicted	---
7	60983797	60983797	+	60983797	3	3	ENSSSCG00000001862	+	intron	*ETFA*
7	61729781	61729781	-	61729781	4	4	ENSSSCG00000001865	-	intron	*SCAPER*
7	61754276	61754276	+	61754276	5	5	---	---	intergenic	---
7	61763224	61763224	+	61763224	7	7	---	---	intergenic	---
7	61793088	61793114	+	61793104	12	37	ENSSSCG00000001866	+	3'UTR	*RCN2*
7	61880668	61880668	+	61880668	8	8	ENSSSCG00000001867	+	3'UTR	*PSTPIP1*
7	61880730	61880733	+	61880730	3	6	XLOC_079374	+	predicted	---
7	61897536	61897541	-	61897539	232	341	ENSSSCG00000001868	-	3'UTR	*TSPAN3*
7	61897611	61897611	-	61897611	3	3	ENSSSCG00000001868	-	3'UTR	*TSPAN3*
7	61908586	61908586	-	61908586	6	6	ENSSSCG00000001868	-	ORF	*TSPAN3*
7	61954967	61954967	-	61954967	11	11	XLOC_080613	-	predicted	---
7	61958303	61958303	-	61958303	3	3	XLOC_080613	-	predicted	---
7	61969984	61969984	-	61969984	3	3	ENSSSCG00000001869	-	intron	*PEAK1*
7	62154610	62154610	-	62154610	3	3	ENSSSCG00000001869	-	intron	*PEAK1*

7	62178178	62178178	-	62178178	5	5	ENSSSCG00000001869	-	intron	*PEAK1*
7	62261705	62261715	+	62261705	8	21	ENSSSCG00000001871	+	3'UTR	---
7	62627383	62627383	-	62627383	10	10	---	---	intergenic	---
7	62713320	62713321	+	62713320	9	12	ENSSSCG00000001876	+	3'UTR	*IMP3*
							ENSSSCG00000001876	+	ORF	---
7	62713387	62713391	+	62713388	30	67	ENSSSCG00000001876	+	3'UTR	*IMP3*
							ENSSSCG00000001876	+	ORF	---
7	62736398	62736398	+	62736398	4	4	ENSSSCG00000001877	+	ORF	*SNUPN*
7	62755125	62755129	+	62755126	46	60	ENSSSCG00000001877	+	3'UTR	*SNUPN*
7	62852643	62852643	+	62852643	3	3	ENSSSCG00000001878	+	3'UTR	*PTPN9*
7	62933955	62933966	+	62933965	10	21	ENSSSCG00000001880	+	3'UTR	*SIN3A*
7	62936155	62936155	+	62936155	3	3	---	---	intergenic	---
7	62949399	62949409	+	62949402	24	76	ENSSSCG00000001881	+	3'UTR	*MAN2C1*
7	62949966	62949968	-	62949967	13	23	ENSSSCG00000001882	-	3'UTR	*NEIL1*
7	62965491	62965496	-	62965493	38	61	ENSSSCG00000001884	-	3'UTR	*COMMD4*
7	63070087	63070087	-	63070087	5	5	ENSSSCG00000001885	-	3'UTR	*C15orf39*
7	63103478	63103479	-	63103478	9	14	---	---	intergenic	---
7	63109742	63109742	-	63109742	3	3	---	---	intergenic	---
7	63214353	63214353	-	63214353	4	4	---	---	intergenic	---
7	63214721	63214725	-	63214721	4	8	ENSSSCG00000027478	-	3'UTR	*PPCDC*
7	63239400	63239407	-	63239402	6	14	ENSSSCG00000001887	-	3'UTR	*SCAMP5*
7	63332446	63332454	+	63332452	236	585	ENSSSCG00000024316	+	3'UTR	*COX5A*
7	63344660	63344660	+	63344660	7	7	ENSSSCG00000029776	+	3'UTR	*FAM219B*
7	63346385	63346408	-	63346408	8	20	ENSSSCG00000024990	-	3'UTR	*MPI*
7	63386593	63386595	+	63386593	5	11	ENSSSCG00000001892	+	3'UTR	*SCAMP2*
7	63387675	63387675	+	63387675	4	4	ENSSSCG00000001892	+	3'UTR	*SCAMP2*

7	63387730	63387735	+	63387734	17	45	ENSSSCG00000001892	+	3'UTR	*SCAMP2*
7	63395783	63395784	+	63395783	13	16	ENSSSCG00000001898	+	3'UTR	*ULK3*
7	63425120	63425128	-	63425128	11	15	ENSSSCG00000001900	-	3'UTR	*CSK*
7	63425390	63425395	-	63425393	28	42	ENSSSCG00000001900	-	3'UTR	*CSK*
7	63468128	63468149	-	63468130	184	312	ENSSSCG00000001901	-	3'UTR	*CYP1A2*
7	63468389	63468389	-	63468389	4	4	ENSSSCG00000001901	-	3'UTR	*CYP1A2*
7	63497472	63497472	+	63497472	10	10	ENSSSCG00000001906	+	3'UTR	*CYP1A1*
7	63504277	63504278	+	63504277	55	67	---	---	intergenic	---
7	63575912	63575912	+	63575912	9	9	ENSSSCG00000001903	+	intron	*EDC3*
7	63583527	63583530	+	63583528	15	24	ENSSSCG00000001903	+	3'UTR	*EDC3*
7	63584005	63584009	-	63584007	48	70	ENSSSCG00000001904	-	3'UTR	*CLK3*
7	63622588	63622588	-	63622588	3	3	ENSSSCG00000022812	-	3'UTR	*ARID3B*
7	63686691	63686701	-	63686693	132	313	ENSSSCG00000001907	-	3'UTR	---
7	63859644	63859646	-	63859644	16	37	---	---	intergenic	---
7	63967908	63967911	-	63967908	8	25	ENSSSCG00000001910	-	3'UTR	*ISLR*
7	64035529	64035529	-	64035529	15	15	ENSSSCG00000001912	-	intron	*PML*
7	64042785	64042785	-	64042785	3	3	ENSSSCG00000001912	-	intron	*PML*
7	64103017	64103017	-	64103017	7	7	ENSSSCG00000001914	-	3'UTR	---
7	64148311	64148311	-	64148311	3	3	---	---	intergenic	---
7	64157215	64157229	-	64157218	57	139	---	---	intergenic	---
7	64330961	64330961	+	64330961	4	4	---	---	intergenic	---
7	64461643	64461651	+	64461643	9	19	ENSSSCG00000001918	+	3'UTR	*NPTN*
7	64463767	64463767	+	64463767	4	4	XLOC_079405	+	predicted	---
7	64688215	64688220	-	64688220	7	11	ENSSSCG00000001921	-	3'UTR	*NEO1*
7	64689970	64689970	-	64689970	4	4	ENSSSCG00000001921	-	3'UTR	*NEO1*
7	64690015	64690015	-	64690015	5	5	ENSSSCG00000001921	-	3'UTR	*NEO1*

7	64881343	64881344	+	64881344	5	8	---	---	intergenic	---
7	65206013	65206013	+	65206013	3	3	ENSSSCG00000001924	+	intron	*BBS4*
7	65206968	65206968	+	65206968	4	4	ENSSSCG00000001924	+	intron	*BBS4*
7	65209103	65209111	+	65209103	4	7	ENSSSCG00000001924	+	intron	*BBS4*
7	65216497	65216497	+	65216497	3	3	ENSSSCG00000001924	+	intron	*BBS4*
7	65267532	65267532	+	65267532	11	11	ENSSSCG00000001924	+	3'UTR	*BBS4*
7	65275555	65275557	-	65275557	7	10	XLOC_080653	-	predicted	---
7	65275648	65275648	-	65275648	4	4	XLOC_080653	-	predicted	---
7	65396340	65396352	-	65396342	324	533	XLOC_080656	-	predicted	---
7	65453892	65453909	+	65453894	113	203	ENSSSCG00000001927	+	3'UTR	*HEXA*
7	65542463	65542468	+	65542468	5	8	XLOC_079413	+	predicted	---
7	65566397	65566397	+	65566397	4	4	ENSSSCG00000001930	+	ORF	*PKM*
7	65575383	65575393	+	65575384	134	349	ENSSSCG00000001930	+	3'UTR	*PKM*
7	65662550	65662565	+	65662565	25	41	ENSSSCG00000001934	+	intron	---
7	65725500	65725501	+	65725501	5	9	ENSSSCG00000001934	+	3'UTR	---
7	65725759	65725763	+	65725761	21	70	ENSSSCG00000001934	+	3'UTR	---
7	65734482	65734482	+	65734482	3	3	---	---	intergenic	---
7	65821638	65821640	+	65821640	7	14	---	---	intergenic	---
7	65879092	65879103	-	65879103	9	19	---	---	intergenic	---
7	65879117	65879129	+	65879129	6	19	---	---	intergenic	---
7	66531013	66531020	+	66531013	8	22	ENSSSCG00000023760	+	3'UTR	*CLEC14A*
							ENSSSCG00000023760	+	ORF	---
7	66593627	66593627	-	66593627	5	5	XLOC_080663	-	predicted	---
7	66635279	66635279	-	66635279	4	4	XLOC_080664	-	predicted	---
7	66680994	66680994	-	66680994	8	8	---	---	intergenic	---
7	66686270	66686270	-	66686270	3	3	---	---	intergenic	---

7	66713608	66713608	+	66713608	3	3	---	---	intergenic	---
7	66717442	66717443	-	66717443	7	11	XLOC_080663	-	predicted	---
7	66749931	66749936	-	66749935	48	87	XLOC_080663	-	predicted	---
7	67102683	67102686	-	67102683	3	6	---	---	intergenic	---
7	67219956	67219956	-	67219956	3	3	---	---	intergenic	---
7	67318586	67318586	-	67318586	3	3	---	---	intergenic	---
7	67600710	67600710	-	67600710	3	3	---	---	intergenic	---
7	67656071	67656071	+	67656071	4	4	---	---	intergenic	---
7	68079262	68079262	-	68079262	3	3	---	---	intergenic	---
7	68669773	68669773	-	68669773	3	3	ENSSSCG00000001946	-	3'UTR	*MBIP*
7	69057766	69057766	-	69057766	3	3	ENSSSCG00000001947	-	3'UTR	*BRMS1L*
7	69068768	69068768	-	69068768	6	6	ENSSSCG00000001947	-	intron	*BRMS1L*
7	69378442	69378442	+	69378442	3	3	---	---	intergenic	---
7	69420841	69420850	+	69420843	39	70	ENSSSCG00000001952	+	3'UTR	*NFKBIA*
7	69487814	69487827	-	69487821	523	884	ENSSSCG00000001951	-	3'UTR	*PSMA6*
7	69487895	69487896	-	69487895	7	10	ENSSSCG00000001951	-	3'UTR	*PSMA6*
7	69493898	69493898	-	69493898	3	3	ENSSSCG00000001951	-	ORF	*PSMA6*
7	69618809	69618809	-	69618809	3	3	ENSSSCG00000029428	-	3'UTR	*FAM177A1*
7	69620662	69620662	-	69620662	5	5	ENSSSCG00000029428	-	3'UTR	*FAM177A1*
7	69622530	69622548	-	69622543	24	64	ENSSSCG00000029428	-	ORF	*FAM177A1*
7	69658013	69658018	-	69658018	7	11	ENSSSCG00000001955	-	3'UTR	*SRP54*
7	69658225	69658225	-	69658225	4	4	ENSSSCG00000001955	-	3'UTR	*SRP54*
7	69658380	69658396	-	69658381	20	42	ENSSSCG00000001955	-	3'UTR	*SRP54*
7	69659069	69659069	-	69659069	7	7	ENSSSCG00000001955	-	intron	*SRP54*
7	69671824	69671824	-	69671824	5	5	ENSSSCG00000001955	-	intron	*SRP54*
7	69712439	69712439	+	69712439	3	3	ENSSSCG00000001956	+	intron	---

7	69736574	69736574	+	69736574	5	5	ENSSSCG00000001956	+	3'UTR	---
							ENSSSCG00000001956	+	ORF	*protein_coding*
7	69798343	69798343	-	69798343	8	8	---	---	intergenic	---
7	69799022	69799022	-	69799022	4	4	---	---	intergenic	---
7	69842937	69842945	+	69842937	4	7	ENSSSCG00000001958	+	ORF	*BAZ1A*
7	69867461	69867461	+	69867461	3	3	ENSSSCG00000001958	+	intron	*BAZ1A*
7	69874191	69874191	+	69874191	3	3	ENSSSCG00000001958	+	intron	*BAZ1A*
7	69892795	69892795	+	69892795	3	3	ENSSSCG00000001958	+	ORF	*BAZ1A*
7	69898779	69898779	+	69898779	5	5	ENSSSCG00000001958	+	3'UTR	*BAZ1A*
7	69899357	69899361	+	69899357	7	15	ENSSSCG00000001958	+	3'UTR	*BAZ1A*
7	69899454	69899456	+	69899455	10	20	ENSSSCG00000001958	+	3'UTR	*BAZ1A*
7	69922609	69922617	+	69922614	43	67	ENSSSCG00000001959	+	3'UTR	*CFL2B*
7	69993623	69993632	+	69993632	7	19	XLOC_079439	+	predicted	---
7	69993887	69993888	+	69993888	11	14	XLOC_079439	+	predicted	---
7	70050898	70050925	+	70050924	13	31	ENSSSCG00000001960	+	3'UTR	*EAPP*
7	70113596	70113604	+	70113604	5	12	XLOC_079444	+	predicted	---
7	71160048	71160048	+	71160048	7	7	XLOC_079447	+	predicted	---
7	71160311	71160311	+	71160311	7	7	XLOC_079447	+	predicted	---
7	71501343	71501343	-	71501343	3	3	ENSSSCG00000024473	-	intron	---
7	71612968	71612968	-	71612968	3	3	ENSSSCG00000024473	-	intron	---
7	71614997	71614997	-	71614997	10	10	ENSSSCG00000024473	-	intron	---
7	72080176	72080176	-	72080176	10	10	---	---	intergenic	---
7	72080213	72080215	+	72080213	12	17	---	---	intergenic	---
7	72191863	72191863	-	72191863	3	3	---	---	intergenic	---
7	72196733	72196733	-	72196733	3	3	---	---	intergenic	---

7	72233918	72233918	-	72233918	3	3	ENSSSCG00000001966	-	ORF	---
7	72235113	72235113	-	72235113	6	6	ENSSSCG00000001966	-	ORF	---
7	72235466	72235466	-	72235466	3	3	ENSSSCG00000001966	-	ORF	---
7	72236476	72236476	-	72236476	3	3	ENSSSCG00000001966	-	ORF	---
7	72356323	72356324	-	72356323	3	6	ENSSSCG00000027799	-	3'UTR	---
7	72357369	72357370	-	72357370	13	16	ENSSSCG00000027799	-	3'UTR	---
7	72357435	72357435	-	72357435	4	4	ENSSSCG00000027799	-	3'UTR	---
7	72535965	72535965	-	72535965	6	6	ENSSSCG00000001968	-	intron	*NUBPL*
7	72704646	72704649	+	72704646	8	13	ENSSSCG00000026310	+	intron	---
7	72847022	72847022	-	72847022	3	3	---	---	intergenic	---
7	72996118	72996120	+	72996119	9	17	ENSSSCG00000026663	+	3'UTR	---
7	72997041	72997041	+	72997041	3	3	ENSSSCG00000026663	+	3'UTR	---
7	72997909	72997909	+	72997909	4	4	ENSSSCG00000026663	+	3'UTR	---
7	73139477	73139477	+	73139477	3	3	ENSSSCG00000001970	+	ORF	*HEATR5A*
7	73140658	73140661	+	73140658	3	6	ENSSSCG00000001970	+	3'UTR	*HEATR5A*
7	73303541	73303544	+	73303542	24	42	XLOC_079457	+	predicted	---
7	73481328	73481338	-	73481338	19	46	ENSSSCG00000001974	-	3'UTR	*G2E3*
7	73481416	73481416	-	73481416	3	3	ENSSSCG00000001974	-	3'UTR	*G2E3*
7	73501785	73501826	-	73501785	4	10	ENSSSCG00000001974	-	intron	*G2E3*
7	74045953	74045953	-	74045953	11	11	---	---	intergenic	---
7	74294547	74294547	-	74294547	3	3	---	---	intergenic	---
7	75141094	75141094	+	75141094	3	3	---	---	intergenic	---
7	75481398	75481398	+	75481398	3	3	---	---	intergenic	---
7	75616669	75616669	+	75616669	3	3	XLOC_079464	+	predicted	---
7	75894921	75894921	-	75894921	3	3	---	---	intergenic	---
7	76715861	76715861	+	76715861	35	35	---	---	intergenic	---

7	76804625	76804625	+	76804625	3	3	---	---	intergenic	---
7	76834170	76834170	+	76834170	3	3	---	---	intergenic	---
7	77311471	77311472	+	77311472	69	73	---	---	intergenic	---
7	77542051	77542051	+	77542051	3	3	---	---	intergenic	---
7	77587869	77587870	-	77587870	4	7	---	---	intergenic	---
7	77602655	77602655	-	77602655	3	3	---	---	intergenic	---
7	77712465	77712465	-	77712465	3	3	---	---	intergenic	---
7	78039046	78039046	+	78039046	3	3	ENSSSCG00000024396	+	3'UTR	*NOVA1*
7	79400205	79400205	+	79400205	3	3	---	---	intergenic	---
7	79434928	79434928	+	79434928	4	4	---	---	intergenic	---
7	79475675	79475675	+	79475675	16	16	---	---	intergenic	---
7	79714892	79714893	-	79714893	10	15	XLOC_080707	-	predicted	---
7	79715498	79715498	+	79715498	3	3	XLOC_079472	+	predicted	---
7	79723610	79723610	-	79723610	3	3	---	---	intergenic	---
7	79831192	79831198	-	79831192	12	39	XLOC_080711	-	predicted	---
7	79879233	79879240	+	79879240	11	30	XLOC_079475	+	predicted	---
7	79903681	79903681	+	79903681	3	3	---	---	intergenic	---
7	80125107	80125119	+	80125108	13	27	XLOC_079479	+	predicted	---
7	80125128	80125128	-	80125128	3	3	ENSSSCG00000001984	-	3'UTR	*KHNYN*
7	80189894	80189894	-	80189894	3	3	ENSSSCG00000001986	-	3'UTR	*NFATC4*
7	80226180	80226187	+	80226185	27	47	ENSSSCG00000001987	+	3'UTR	*RIPK3*
7	80256672	80256719	+	80256715	65	258	ENSSSCG00000001989	+	3'UTR	*CIDE-B*
7	80256691	80256691	-	80256691	3	3	ENSSSCG00000001990	-	3'UTR	*NOP9*
7	80273089	80273096	+	80273093	92	305	ENSSSCG00000001991	+	3'UTR	*DHRS1*
7	80274878	80274878	+	80274878	5	5	XLOC_079483	+	predicted	---
7	80298868	80298869	+	80298868	8	12	XLOC_079484	+	predicted	---

7	80316074	80316079	+	80316078	17	38	ENSSSCG00000001993	+	3'UTR	*TGM1*
							ENSSSCG00000001993	+	ORF	---
7	80331600	80331608	-	80331606	7	14	ENSSSCG00000026947	-	3'UTR	*GMPR2*
7	80331659	80331662	-	80331659	5	18	ENSSSCG00000026947	-	3'UTR	*GMPR2*
7	80332852	80332852	-	80332852	10	10	ENSSSCG00000026947	-	intron	*GMPR2*
7	80353284	80353293	+	80353290	122	283	XLOC_079486	+	predicted	---
7	80356327	80356327	+	80356327	10	10	XLOC_079487	+	predicted	---
7	80361142	80361181	+	80361180	24	54	ENSSSCG00000001999	+	intron	---
7	80362309	80362313	-	80362313	49	100	XLOC_080722	-	predicted	---
7	80377976	80377976	+	80377976	5	5	ENSSSCG00000001999	+	ORF	---
7	80378161	80378161	+	80378161	4	4	ENSSSCG00000001999	+	ORF	---
7	80381317	80381317	+	80381317	7	7	ENSSSCG00000001999	+	3'UTR	---
7	80390034	80390039	+	80390037	58	79	XLOC_079487	+	predicted	---
7	80390041	80390044	-	80390043	22	38	ENSSSCG00000002001	-	3'UTR	*REC8*
7	80404990	80404991	-	80404990	17	23	ENSSSCG00000002002	-	3'UTR	*IRF9*
7	80411498	80411505	-	80411499	6	21	XLOC_080723	-	predicted	---
7	80429158	80429167	+	80429163	106	317	ENSSSCG00000002004	+	3'UTR	*PSME2*
7	80433118	80433130	-	80433126	202	636	ENSSSCG00000002006	-	3'UTR	*INF-GAMMA*
7	80433124	80433127	+	80433125	38	63	ENSSSCG00000002005	+	3'UTR	*EMC9*
7	80434636	80434678	-	80434638	8	23	ENSSSCG00000002006	-	ORF	*INF-GAMMA*
7	80440286	80440286	-	80440286	3	3	XLOC_080725	-	predicted	---
7	80442509	80442518	-	80442510	18	63	ENSSSCG00000002008	-	3'UTR	---
7	80458498	80458500	-	80458500	17	31	ENSSSCG00000002009	-	3'UTR	*PCK2*
7	80512360	80512417	-	80512365	231	803	ENSSSCG00000002013	-	3'UTR	*DHRS4*
7	80639459	80639459	-	80639459	4	4	---	---	intergenic	---
7	80848406	80848406	+	80848406	6	6	ENSSSCG00000002014	+	3'UTR	*JPH4*

7	80848475	80848475	+	80848475	4	4	ENSSSCG00000002014	+	3'UTR	*JPH4*
7	80862679	80862679	-	80862679	3	3	XLOC_080730	-	predicted	---
7	80944630	80944631	-	80944631	12	16	ENSSSCG00000028190	-	3'UTR	*HOMEZ*
7	80951386	80951386	-	80951386	4	4	ENSSSCG00000028190	-	intron	*HOMEZ*
7	80960919	80960964	-	80960920	16	59	XLOC_080733	-	predicted	---
7	80961031	80961031	-	80961031	6	6	XLOC_080733	-	predicted	---
7	80975120	80975120	+	80975120	4	4	XLOC_079500	+	predicted	---
7	80989125	80989126	+	80989125	3	6	XLOC_079500	+	predicted	---
7	80999766	80999784	-	80999771	7	22	ENSSSCG00000002025	-	3'UTR	*SLC22A17*
7	81066281	81066281	-	81066281	59	59	ENSSSCG00000002029	-	3'UTR	*MYH7*
7	81075479	81075479	-	81075479	228	228	---	---	intergenic	---
7	81115181	81115184	+	81115184	20	23	ENSSSCG00000002020	+	3'UTR	*NGDN*
7	81258330	81258330	+	81258330	3	3	ENSSSCG00000002032	+	3'UTR	*SLC7A8*
7	81283564	81283566	-	81283564	15	19	XLOC_080741	-	predicted	---
7	81292345	81292345	+	81292345	7	7	ENSSSCG00000002034	+	ORF	*ACIN1*
7	81325191	81325196	+	81325194	28	38	ENSSSCG00000002034	+	3'UTR	*ACIN1*
7	81337077	81337084	+	81337081	365	489	ENSSSCG00000002036	+	3'UTR	---
							ENSSSCG00000002037	+	intron	*CDH24*
7	81413753	81413759	-	81413755	15	34	ENSSSCG00000002039	-	3'UTR	*MMP14*
7	81413919	81413929	-	81413919	4	7	ENSSSCG00000002039	-	3'UTR	*MMP14*
7	81427093	81427101	-	81427099	52	140	ENSSSCG00000002040	-	3'UTR	*MRPL52*
7	81427194	81427194	-	81427194	3	3	ENSSSCG00000002040	-	ORF	*MRPL52*
7	81474658	81474660	+	81474659	33	45	ENSSSCG00000002041	+	3'UTR	*SLC7A7*
7	81475119	81475152	-	81475120	17	43	XLOC_080745	-	predicted	---
7	81545239	81545239	-	81545239	4	4	XLOC_080746	-	predicted	---
7	81578890	81578890	-	81578890	7	7	XLOC_080750	-	predicted	---

7	81581194	81581194	-	81581194	10	10	---	---	intergenic	---
7	81641059	81641069	-	81641068	62	137	ENSSSCG00000002051	-	3'UTR	*ABHD4*
7	81684720	81684722	+	81684720	10	16	ENSSSCG00000002050	+	intron	*DAD1*
7	81693967	81693974	+	81693971	301	549	ENSSSCG00000002050	+	3'UTR	*DAD1*
7	81701815	81701815	-	81701815	4	4	XLOC_080754	-	predicted	---
7	81760132	81760132	+	81760132	3	3	---	---	intergenic	---
7	81787550	81787556	-	81787550	39	62	XLOC_080757	-	predicted	---
7	82086619	82086619	+	82086619	12	12	---	---	intergenic	---
7	82754541	82754541	+	82754541	4	4	---	---	intergenic	---
7	82870637	82870637	+	82870637	8	8	---	---	intergenic	---
7	82889821	82889821	+	82889821	5	5	---	---	intergenic	---
7	82994864	82994864	+	82994864	4	4	XLOC_079549	+	predicted	---
7	83020949	83020956	+	83020950	12	25	ENSSSCG00000024445	+	3'UTR	*METTL3*
7	83021651	83021659	-	83021651	8	18	XLOC_080806	-	predicted	---
7	83021721	83021727	-	83021721	11	18	XLOC_080806	-	predicted	---
7	83022085	83022085	-	83022085	4	4	XLOC_080806	-	predicted	---
7	83025065	83025065	-	83025065	3	3	ENSSSCG00000029203	-	ORF	*TOX4*
7	83025521	83025521	-	83025521	3	3	ENSSSCG00000029203	-	ORF	*TOX4*
7	83030669	83030670	-	83030669	7	10	ENSSSCG00000029203	-	intron	*TOX4*
7	83057148	83057159	+	83057156	9	30	ENSSSCG00000029544	+	3'UTR	*RAB2B*
7	83057226	83057226	+	83057226	7	7	ENSSSCG00000029544	+	3'UTR	*RAB2B*
7	83057301	83057308	+	83057307	11	34	ENSSSCG00000029544	+	3'UTR	*RAB2B*
7	83058571	83058582	+	83058571	3	9	ENSSSCG00000029544	+	3'UTR	*RAB2B*
7	83118667	83118667	+	83118667	3	3	ENSSSCG00000002127	+	ORF	*CHD8*
7	83171941	83171944	+	83171942	9	19	XLOC_079555	+	predicted	---
7	83173283	83173283	+	83173283	3	3	ENSSSCG00000022900	+	ORF	*SUPT16H*

7	83205980	83206007	+	83205988	8	37	ENSSSCG00000022900	+	3'UTR	*SUPT16H*
7	83206182	83206207	-	83206183	82	262	ENSSSCG00000002129	-	3'UTR	*RPGRIP1*
7	83367901	83367901	+	83367901	8	8	---	---	intergenic	---
7	83479598	83479600	+	83479599	28	38	ENSSSCG00000002136	+	3'UTR	*TMEM55B*
7	83479683	83479721	-	83479684	30	67	ENSSSCG00000002137	-	3'UTR	*APEX1*
7	83490099	83490099	+	83490099	14	14	XLOC_079558	+	predicted	---
7	83558526	83558527	+	83558527	7	10	ENSSSCG00000002142	+	3'UTR	---
7	83563415	83563418	-	83563418	6	14	ENSSSCG00000002143	-	3'UTR	*PARP2*
7	83568372	83568373	-	83568373	8	11	ENSSSCG00000002143	-	intron	*PARP2*
7	83590421	83590421	-	83590421	7	7	---	---	intergenic	---
7	83592440	83592440	+	83592440	3	3	XLOC_079562	+	predicted	---
7	83594441	83594441	-	83594441	5	5	---	---	intergenic	---
7	83603699	83603699	+	83603699	3	3	XLOC_079562	+	predicted	---
7	83948074	83948080	-	83948074	3	6	XLOC_080828	-	predicted	---
7	84264379	84264379	-	84264379	3	3	---	---	intergenic	---
7	84327396	84327396	+	84327396	4	4	---	---	intergenic	---
7	84719210	84719210	+	84719210	4	4	---	---	intergenic	---
7	85472425	85472427	+	85472425	6	10	---	---	intergenic	---
7	85902805	85902807	+	85902805	5	9	---	---	intergenic	---
7	86145999	86145999	+	86145999	4	4	ENSSSCG00000002235	+	3'UTR	*LPCAT4*
7	86146942	86146944	-	86146942	5	12	XLOC_080856	-	predicted	---
7	86159176	86159190	+	86159179	185	406	XLOC_079604	+	predicted	---
7	86263162	86263165	-	86263165	11	34	ENSSSCG00000026925	-	3'UTR	*TMEM85*
7	86263335	86263349	-	86263349	8	16	ENSSSCG00000026925	-	3'UTR	*TMEM85*
7	86279134	86279157	+	86279135	23	70	---	---	intergenic	---
7	86341954	86341954	+	86341954	5	5	ENSSSCG00000002245	+	3'UTR	*KATNBL1*

7	86401273	86401273	+	86401273	4	4	ENSSSCG00000027051	+	ORF	*C7H15orf24*
7	86401420	86401423	+	86401421	8	22	ENSSSCG00000027051	+	3'UTR	*C7H15orf24*
7	86401606	86401618	+	86401613	41	102	ENSSSCG00000027051	+	3'UTR	*C7H15orf24*
7	86403969	86403972	+	86403972	9	12	ENSSSCG00000027051	+	3'UTR	*C7H15orf24*
7	86446978	86446978	+	86446978	3	3	ENSSSCG00000002247	+	intron	---
7	86507532	86507532	+	86507532	3	3	---	---	intergenic	---
7	86904204	86904204	-	86904204	24	24	---	---	intergenic	---
7	86905687	86905687	+	86905687	3	3	---	---	intergenic	---
7	86998243	86998243	+	86998243	3	3	---	---	intergenic	---
7	87112880	87112886	+	87112885	56	130	ENSSSCG00000002251	+	3'UTR	---
7	87586100	87586100	-	87586100	8	8	ENSSSCG00000002252	-	3'UTR	*ARRDC4*
7	87587946	87587946	-	87587946	3	3	ENSSSCG00000002252	-	3'UTR	*ARRDC4*
7	87588654	87588654	-	87588654	12	12	ENSSSCG00000002252	-	3'UTR	*ARRDC4*
7	87693013	87693013	-	87693013	4	4	---	---	intergenic	---
7	87994116	87994116	-	87994116	3	3	XLOC_080863	-	predicted	---
7	88110460	88110460	+	88110460	3	3	---	---	intergenic	---
7	88234458	88234458	+	88234458	6	6	---	---	intergenic	---
7	88286212	88286212	+	88286212	3	3	---	---	intergenic	---
7	88296205	88296205	-	88296205	3	3	---	---	intergenic	---
7	88368288	88368307	-	88368300	8	16	---	---	intergenic	---
7	88601114	88601114	-	88601114	3	3	---	---	intergenic	---
7	88613209	88613209	+	88613209	3	3	---	---	intergenic	---
7	88634958	88634958	+	88634958	8	8	---	---	intergenic	---
7	88961580	88961580	-	88961580	8	8	ENSSSCG00000002254	-	3'UTR	*NR2F2*
7	88962662	88962662	-	88962662	3	3	ENSSSCG00000002254	-	3'UTR	*NR2F2*
7	89014843	89014846	+	89014844	7	13	---	---	intergenic	---

7	89423494	89423494	-	89423494	3	3	---	---	intergenic	---
7	89940953	89940953	+	89940953	3	3	---	---	intergenic	---
7	90033119	90033119	+	90033119	3	3	---	---	intergenic	---
7	90100336	90100336	+	90100336	4	4	---	---	intergenic	---
7	90115742	90115743	-	90115742	3	6	---	---	intergenic	---
7	90236133	90236133	+	90236133	29	29	---	---	intergenic	---
7	90257371	90257371	-	90257371	6	6	---	---	intergenic	---
7	90269648	90269653	+	90269648	3	9	---	---	intergenic	---
7	90675206	90675206	+	90675206	3	3	ENSSSCG00000022551	+	3'UTR	*MCTP2*
7	91420570	91420570	+	91420570	4	4	---	---	intergenic	---
7	91466741	91466741	-	91466741	5	5	---	---	intergenic	---
7	91479270	91479270	-	91479270	3	3	XLOC_080875	-	predicted	---
7	91657422	91657422	-	91657422	6	6	---	---	intergenic	---
7	91703816	91703816	+	91703816	3	3	---	---	intergenic	---
7	91874323	91874323	-	91874323	3	3	XLOC_080877	-	predicted	---
7	91874368	91874369	-	91874368	9	12	XLOC_080877	-	predicted	---
7	91987611	91987611	-	91987611	3	3	ENSSSCG00000002266	-	intron	*CHD2*
7	92004447	92004447	-	92004447	7	7	XLOC_080877	-	predicted	---
7	92004541	92004541	-	92004541	3	3	XLOC_080877	-	predicted	---
7	92152984	92152984	+	92152984	5	5	---	---	intergenic	---
7	92247919	92247919	+	92247919	9	9	ENSSSCG00000002265	+	3'UTR	*FAM174B*
7	92283662	92283662	-	92283662	3	3	XLOC_080879	-	predicted	---
7	92299997	92300022	+	92300003	13	25	XLOC_079628	+	predicted	---
7	92719394	92719394	-	92719394	3	3	ENSSSCG00000002263	-	3'UTR	*SLCO3A1*
7	92727281	92727281	-	92727281	3	3	ENSSSCG00000002263	-	intron	*SLCO3A1*
7	92728581	92728581	-	92728581	3	3	ENSSSCG00000002263	-	intron	*SLCO3A1*

7	93761019	93761019	-	93761019	10	10	ENSSSCG00000002262	-	intron	*SV2B*
7	94134884	94134884	-	94134884	4	4	---	---	intergenic	---
7	94525924	94525928	+	94525924	12	15	ENSSSCG00000022453	+	ORF	---
7	94571140	94571140	+	94571140	3	3	ENSSSCG00000002259	+	intron	*AKAP13*
7	94580067	94580067	+	94580067	3	3	XLOC_079635	+	predicted	---
7	94582867	94582867	+	94582867	4	4	XLOC_079635	+	predicted	---
7	94583012	94583012	+	94583012	14	14	XLOC_079635	+	predicted	---
7	94609179	94609182	-	94609180	7	20	XLOC_080891	-	predicted	---
7	94776390	94776404	-	94776390	12	76	XLOC_080893	-	predicted	---
7	94894975	94894980	+	94894976	53	92	ENSSSCG00000002269	+	3'UTR	*MTHFD1*
7	94974321	94974321	+	94974321	4	4	XLOC_079642	+	predicted	---
7	94980048	94980048	-	94980048	4	4	XLOC_080894	-	predicted	---
7	94980607	94980607	-	94980607	4	4	XLOC_080894	-	predicted	---
7	94989401	94989407	+	94989407	6	9	ENSSSCG00000002274	+	ORF	---
7	94990000	94990002	+	94990002	23	53	ENSSSCG00000002274	+	3'UTR	*HSPA2*
							ENSSSCG00000002274	+	ORF	---
7	94990121	94990124	+	94990122	57	74	ENSSSCG00000002274	+	3'UTR	*HSPA2*
							ENSSSCG00000002274	+	ORF	---
7	95016534	95016535	+	95016535	4	7	---	---	intergenic	---
7	95032293	95032294	+	95032294	15	20	ENSSSCG00000002275	+	3'UTR	*PPP1R36*
7	95032389	95032401	+	95032391	24	75	ENSSSCG00000002275	+	3'UTR	*PPP1R36*
7	95032636	95032636	+	95032636	3	3	XLOC_079643	+	predicted	---
7	95191592	95191596	+	95191592	11	22	ENSSSCG00000002276	+	3'UTR	*PLEKHG3*
7	95381502	95381502	+	95381502	15	15	ENSSSCG00000022147	+	intron	*CHURC1*
7	95383064	95383066	+	95383065	6	14	ENSSSCG00000022147	+	3'UTR	*CHURC1*
7	95384727	95384727	+	95384727	6	6	ENSSSCG00000022147	+	3'UTR	*CHURC1*

7	95384798	95384804	+	95384802	22	40	ENSSSCG00000022147	+	3'UTR	*CHURC1*
7	95443429	95443432	-	95443429	3	6	---	---	intergenic	---
7	95506171	95506171	+	95506171	3	3	ENSSSCG00000002281	+	3'UTR	*FNTB*
7	95519254	95519259	-	95519256	54	88	XLOC_080905	-	predicted	---
7	95519327	95519327	-	95519327	4	4	XLOC_080905	-	predicted	---
7	95519673	95519674	-	95519673	4	7	XLOC_080905	-	predicted	---
7	95520556	95520558	-	95520558	19	30	XLOC_080905	-	predicted	---
7	95608311	95608312	+	95608311	7	11	XLOC_079652	+	predicted	---
7	95940802	95940802	+	95940802	3	3	ENSSSCG00000002283	+	intron	*FUT8*
7	96007790	96007791	+	96007791	7	10	ENSSSCG00000002283	+	intron	*FUT8*
7	96100614	96100614	+	96100614	9	9	ENSSSCG00000002283	+	3'UTR	*FUT8*
7	96101008	96101017	+	96101017	9	15	ENSSSCG00000002283	+	3'UTR	*FUT8*
7	96166494	96166495	+	96166494	8	11	---	---	intergenic	---
7	96209275	96209275	+	96209275	3	3	---	---	intergenic	---
7	96334248	96334248	+	96334248	3	3	---	---	intergenic	---
7	96574807	96574814	+	96574810	7	13	---	---	intergenic	---
7	96614869	96614869	+	96614869	3	3	---	---	intergenic	---
7	96687720	96687720	+	96687720	4	4	---	---	intergenic	---
7	96734997	96734997	+	96734997	3	3	ENSSSCG00000002284	+	intron	---
7	96747888	96747898	+	96747895	123	222	XLOC_079655	+	predicted	---
7	97222777	97222777	+	97222777	4	4	ENSSSCG00000002285	+	3'UTR	*GPHN*
7	97247253	97247253	+	97247253	10	10	ENSSSCG00000002286	+	ORF	*FAM71D*
7	97255408	97255421	+	97255418	9	17	ENSSSCG00000002286	+	intron	*FAM71D*
7	97262560	97262582	+	97262575	58	216	XLOC_079659	+	predicted	---
7	97301939	97301939	+	97301939	10	10	ENSSSCG00000002287	+	intron	*MPP5*
7	97375521	97375529	-	97375525	25	59	ENSSSCG00000002288	-	3'UTR	*ATP6V1D*

7	97375743	97375768	-	97375751	29	44	ENSSSCG00000002288	-	3'UTR	*ATP6V1D*
7	97376232	97376232	-	97376232	3	3	ENSSSCG00000002288	-	3'UTR	*ATP6V1D*
7	97377338	97377340	-	97377339	12	27	ENSSSCG00000002288	-	intron	*ATP6V1D*
7	97405502	97405502	+	97405502	3	3	ENSSSCG00000027990	+	ORF	*EIF2S1*
7	97416892	97416892	+	97416892	3	3	ENSSSCG00000027990	+	3'UTR	*EIF2S1*
7	97417159	97417168	+	97417166	50	68	ENSSSCG00000027990	+	3'UTR	*EIF2S1*
7	97419468	97419468	+	97419468	3	3	ENSSSCG00000027990	+	3'UTR	*EIF2S1*
7	97419963	97419963	-	97419963	3	3	ENSSSCG00000002290	-	3'UTR	*PLEK2*
7	97491468	97491473	-	97491471	58	116	XLOC_080914	-	predicted	---
7	97493024	97493027	-	97493024	3	6	XLOC_080914	-	predicted	---
7	97548838	97548838	-	97548838	4	4	---	---	intergenic	---
7	97612378	97612378	-	97612378	9	9	ENSSSCG00000022069	-	3'UTR	*PIGH*
7	97613245	97613245	+	97613245	3	3	ENSSSCG00000002292	+	3'UTR	*PLEKHH1*
7	97625637	97625637	-	97625637	11	11	XLOC_080917	-	predicted	---
7	97693014	97693052	-	97693025	132	316	ENSSSCG00000002295	-	3'UTR	*NFS*
7	97693054	97693059	+	97693054	17	32	ENSSSCG00000002294	+	3'UTR	*ARG2*
							ENSSSCG00000002294	+	ORF	---
7	97693101	97693101	-	97693101	4	4	ENSSSCG00000002295	-	3'UTR	*NFS*
7	97720671	97720674	-	97720671	6	11	ENSSSCG00000002295	-	ORF	*NFS*
7	97722350	97722358	-	97722352	135	272	ENSSSCG00000002296	-	3'UTR	*RDH11*
7	97800588	97800588	+	97800588	3	3	ENSSSCG00000002297	+	3'UTR	*RDH12*
7	97802302	97802307	+	97802307	5	9	ENSSSCG00000002297	+	3'UTR	*RDH12*
7	97825811	97825811	-	97825811	5	5	---	---	intergenic	---
7	97863945	97863953	-	97863947	17	51	ENSSSCG00000002298	-	intron	*ZFYVE26*
7	97995925	97995925	+	97995925	3	3	XLOC_079667	+	predicted	---
7	98045826	98045826	-	98045826	3	3	---	---	intergenic	---

7	98104597	98104597	+	98104597	6	6	---	---	intergenic	---
7	98109712	98109713	+	98109713	10	17	---	---	intergenic	---
7	98179502	98179502	-	98179502	4	4	---	---	intergenic	---
7	98222656	98222657	+	98222656	4	8	---	---	intergenic	---
7	98417359	98417359	+	98417359	4	4	ENSSSCG00000028877	+	intron	---
7	98740582	98740582	-	98740582	3	3	---	---	intergenic	---
7	98803346	98803352	-	98803352	84	152	ENSSSCG00000028304	-	3'UTR	*ZFP36L1*
7	98803798	98803798	-	98803798	3	3	ENSSSCG00000028304	-	3'UTR	*ZFP36L1*
7	98804032	98804032	-	98804032	3	3	ENSSSCG00000028304	-	3'UTR	*ZFP36L1*
7	98810632	98810632	-	98810632	3	3	---	---	intergenic	---
7	98863741	98863741	-	98863741	7	7	ENSSSCG00000020703	-	3'UTR	*ACTN1*
7	98965875	98965875	+	98965875	6	6	---	---	intergenic	---
7	99026873	99026873	-	99026873	3	3	ENSSSCG00000002303	-	intron	---
7	99130803	99130806	+	99130803	21	43	ENSSSCG00000002305	+	3'UTR	*EXD2*
7	99263626	99263626	+	99263626	7	7	ENSSSCG00000002306	+	3'UTR	*GALNT16*
7	99291063	99291067	-	99291064	38	72	ENSSSCG00000002307	-	3'UTR	*ERH*
7	99291264	99291264	-	99291264	5	5	ENSSSCG00000002307	-	3'UTR	*ERH*
7	99419864	99419866	+	99419864	19	39	ENSSSCG00000028600	+	3'UTR	*SLC39A9*
7	99667000	99667000	+	99667000	4	4	---	---	intergenic	---
7	99675815	99675822	+	99675815	8	21	XLOC_079682	+	predicted	---
7	99676019	99676032	+	99676019	8	15	XLOC_079682	+	predicted	---
7	99676345	99676354	+	99676353	13	22	XLOC_079682	+	predicted	---
7	99677881	99677883	-	99677881	3	6	XLOC_080938	-	predicted	---
7	99677946	99677956	-	99677956	5	16	XLOC_080938	-	predicted	---
7	99679040	99679040	-	99679040	3	3	XLOC_080938	-	predicted	---
7	99679543	99679579	-	99679577	13	33	XLOC_080938	-	predicted	---

7	99679630	99679635	-	99679631	31	39	ENSSSCG00000002313	-	3'UTR	---
7	99682166	99682166	-	99682166	7	7	ENSSSCG00000002313	-	ORF	---
7	99755373	99755374	-	99755373	4	8	XLOC_080939	-	predicted	---
7	100173172	100173172	-	100173172	12	12	ENSSSCG00000002316	-	3'UTR	*COX16*
7	100247012	100247019	+	100247012	12	28	ENSSSCG00000026389	+	3'UTR	*SYNJ2BP*
7	100251544	100251551	+	100251551	5	9	XLOC_079684	+	predicted	---
7	100629460	100629471	+	100629461	14	29	XLOC_079688	+	predicted	---
7	100772532	100772532	+	100772532	3	3	ENSSSCG00000002330	+	intron	*PCNX*
7	101026026	101026026	-	101026026	29	29	---	---	intergenic	---
7	101041766	101041766	-	101041766	3	3	---	---	intergenic	---
7	101168760	101168760	+	101168760	3	3	---	---	intergenic	---
7	101689081	101689083	+	101689081	34	38	---	---	intergenic	---
7	101695590	101695590	-	101695590	3	3	---	---	intergenic	---
7	101894905	101894905	-	101894905	5	5	ENSSSCG00000002333	-	intron	---
7	102014558	102014558	-	102014558	3	3	ENSSSCG00000002333	-	intron	---
7	102020519	102020519	-	102020519	3	3	ENSSSCG00000002333	-	intron	---
7	102106557	102106568	+	102106567	19	36	ENSSSCG00000028159	+	3'UTR	---
7	102110550	102110551	-	102110550	6	9	ENSSSCG00000002337	-	3'UTR	*ZFYVE1*
7	102111977	102111983	-	102111977	9	14	ENSSSCG00000002337	-	3'UTR	*ZFYVE1*
7	102233711	102233721	+	102233720	11	23	ENSSSCG00000028663	+	intron	*RBM25*
7	102254601	102254614	+	102254601	7	13	ENSSSCG00000028663	+	3'UTR	*RBM25*
7	102350200	102350200	+	102350200	10	10	ENSSSCG00000002340	+	intron	*PSEN1*
7	102359249	102359249	+	102359249	6	6	ENSSSCG00000002340	+	3'UTR	*PSEN1*
7	102359654	102359655	+	102359654	12	19	ENSSSCG00000002340	+	3'UTR	*PSEN1*
7	102360291	102360301	+	102360296	13	23	ENSSSCG00000002340	+	3'UTR	*PSEN1*
7	102362531	102362567	+	102362567	6	9	XLOC_079699	+	predicted	---

7	102390644	102390644	+	102390644	4	4	ENSSSCG00000002341	+	intron	---
7	102419458	102419475	-	102419473	11	24	ENSSSCG00000022178	-	3'UTR	---
7	102504552	102504552	-	102504552	3	3	ENSSSCG00000022178	-	intron	---
7	102525787	102525787	-	102525787	3	3	---	---	intergenic	---
7	102561888	102561888	+	102561888	3	3	---	---	intergenic	---
7	102577405	102577405	+	102577405	3	3	---	---	intergenic	---
7	102606234	102606238	-	102606238	12	23	XLOC_080957	-	predicted	---
7	102612604	102612604	+	102612604	4	4	XLOC_079703	+	predicted	---
7	102680783	102680783	+	102680783	4	4	ENSSSCG00000002345	+	3'UTR	---
7	102683651	102683652	+	102683651	7	12	XLOC_079704	+	predicted	---
7	102685374	102685374	+	102685374	3	3	XLOC_079704	+	predicted	---
7	102689119	102689119	+	102689119	3	3	XLOC_079704	+	predicted	---
7	102691102	102691106	-	102691102	6	16	XLOC_080958	-	predicted	---
7	102691156	102691166	-	102691157	20	26	XLOC_080958	-	predicted	---
7	102696370	102696370	-	102696370	6	6	ENSSSCG00000002347	-	intron	*DNAL1*
7	102742806	102742814	-	102742806	6	10	ENSSSCG00000002348	-	3'UTR	*ACOT6*
7	102779844	102779844	-	102779844	6	6	---	---	intergenic	---
7	102934823	102934823	+	102934823	4	4	ENSSSCG00000002351	+	intron	---
7	102941883	102941883	+	102941883	3	3	ENSSSCG00000002351	+	ORF	---
7	102950178	102950201	+	102950199	28	163	ENSSSCG00000002351	+	3'UTR	---
7	102997753	102997754	+	102997753	7	11	ENSSSCG00000002352	+	3'UTR	*ZNF410*
7	102997936	102997936	+	102997936	5	5	ENSSSCG00000002352	+	3'UTR	*ZNF410*
7	102999954	102999954	-	102999954	3	3	ENSSSCG00000002353	-	ORF	*FAM161B*
7	103000853	103000853	+	103000853	3	3	ENSSSCG00000002352	+	3'UTR	*ZNF410*
7	103024481	103024492	+	103024484	21	59	ENSSSCG00000002354	+	3'UTR	*COQ6*
7	103028259	103028267	-	103028260	21	69	XLOC_080966	-	predicted	---

7	103028428	103028429	-	103028429	6	9	XLOC_080966	-	predicted	---
7	103117150	103117157	-	103117150	16	30	XLOC_080965	-	predicted	---
7	103124086	103124086	-	103124086	3	3	ENSSSCG00000002357	-	intron	*ALDH6A1*
7	103129342	103129345	-	103129345	9	12	ENSSSCG00000002357	-	ORF	*ALDH6A1*
7	103274977	103274977	+	103274977	3	3	ENSSSCG00000027564	+	intron	*LIN52*
7	103337799	103337799	+	103337799	5	5	ENSSSCG00000027564	+	3'UTR	*LIN52*
7	103368789	103368789	-	103368789	3	3	---	---	intergenic	---
7	103573086	103573104	-	103573101	47	122	ENSSSCG00000002366	-	3'UTR	*NPC2*
7	103573197	103573202	-	103573198	19	40	ENSSSCG00000002366	-	3'UTR	*NPC2*
7	103584642	103584642	+	103584642	11	11	XLOC_079717	+	predicted	---
7	103605255	103605255	-	103605255	6	6	ENSSSCG00000002368	-	intron	*LTBP2*
7	103736301	103736301	-	103736301	13	13	ENSSSCG00000002370	-	3'UTR	*AREL1*
7	103800849	103800874	+	103800873	22	36	ENSSSCG00000021442	+	3'UTR	*FCF1*
7	103886111	103886144	+	103886141	10	29	ENSSSCG00000002372	+	3'UTR	---
7	103939620	103939622	+	103939621	14	30	ENSSSCG00000002374	+	intron	*DLST*
7	103955477	103955487	+	103955479	47	93	ENSSSCG00000002374	+	3'UTR	*DLST*
7	103956655	103956655	-	103956655	3	3	XLOC_080974	-	predicted	---
7	104020760	104020762	-	104020760	8	12	XLOC_080976	-	predicted	---
7	104054495	104054507	+	104054502	20	71	ENSSSCG00000002377	+	3'UTR	*EIF2B2*
7	104057894	104057905	-	104057896	7	21	XLOC_080977	-	predicted	---
7	104064431	104064438	-	104064431	11	14	XLOC_080978	-	predicted	---
7	104093822	104093843	-	104093827	57	242	ENSSSCG00000002379	-	3'UTR	*ACYP1*
7	104094010	104094016	-	104094015	8	19	ENSSSCG00000002379	-	3'UTR	*ACYP1*
7	104110661	104110693	+	104110661	11	14	ENSSSCG00000002380	+	ORF	*ZC2HC1C*
7	104117474	104117484	+	104117475	50	111	ENSSSCG00000002380	+	3'UTR	*ZC2HC1C*
7	104120097	104120100	-	104120100	7	10	ENSSSCG00000002381	-	3'UTR	*NEK9*

7	104120392	104120404	-	104120393	16	24	ENSSSCG00000002381	-	3'UTR	*NEK9*
7	104120969	104120969	-	104120969	19	19	ENSSSCG00000002381	-	3'UTR	*NEK9*
7	104171511	104171522	-	104171519	74	173	XLOC_080980	-	predicted	---
7	104171770	104171770	-	104171770	5	5	XLOC_080980	-	predicted	---
7	104172004	104172004	-	104172004	3	3	XLOC_080980	-	predicted	---
7	104172956	104172956	-	104172956	3	3	XLOC_080980	-	predicted	---
7	104173901	104173908	-	104173908	30	33	ENSSSCG00000002382	-	3'UTR	*TMED10*
7	104175409	104175409	-	104175409	3	3	ENSSSCG00000002382	-	ORF	*TMED10*
7	104185876	104185876	-	104185876	4	4	ENSSSCG00000002382	-	intron	*TMED10*
7	104207434	104207434	+	104207434	9	9	---	---	intergenic	---
7	104297091	104297100	+	104297097	12	25	ENSSSCG00000002383	+	3'UTR	*FOS*
7	104314758	104314758	-	104314758	3	3	---	---	intergenic	---
7	104324205	104324205	-	104324205	3	3	---	---	intergenic	---
7	104424133	104424133	-	104424133	13	13	---	---	intergenic	---
7	104448957	104448957	+	104448957	4	4	ENSSSCG00000021606	+	intron	*FLVCR2*
7	104478313	104478318	+	104478318	6	9	ENSSSCG00000021606	+	3'UTR	*FLVCR2*
7	104479241	104479248	+	104479241	4	11	ENSSSCG00000021606	+	3'UTR	*FLVCR2*
7	104484563	104484563	+	104484563	13	13	---	---	intergenic	---
7	104545174	104545174	+	104545174	3	3	---	---	intergenic	---
7	104578141	104578145	-	104578145	24	31	XLOC_080987	-	predicted	---
7	104604479	104604486	+	104604485	11	20	---	---	intergenic	---
7	105175420	105175427	+	105175427	10	18	ENSSSCG00000025984	+	3'UTR	*TTLL5*
7	105175672	105175672	+	105175672	5	5	XLOC_079730	+	predicted	---
7	105178642	105178642	-	105178642	12	12	ENSSSCG00000002385	-	3'UTR	*TGFB3*
7	105199125	105199125	-	105199125	4	4	ENSSSCG00000002385	-	ORF	*TGFB3*
7	105221520	105221520	+	105221520	3	3	---	---	intergenic	---

7	105242450	105242450	+	105242450	6	6	---	---	intergenic	---
7	105299432	105299441	+	105299432	39	184	XLOC_079733	+	predicted	---
7	105356812	105356812	-	105356812	3	3	---	---	intergenic	---
7	105571794	105571794	-	105571794	21	21	---	---	intergenic	---
7	105579084	105579091	-	105579084	12	16	---	---	intergenic	---
7	105978028	105978035	+	105978035	4	7	ENSSSCG00000002389	+	3'UTR	*VASH1*
7	105981453	105981455	-	105981454	18	29	XLOC_080999	-	predicted	---
7	105983116	105983131	-	105983129	9	19	XLOC_080999	-	predicted	---
7	106036234	106036234	+	106036234	5	5	XLOC_079739	+	predicted	---
7	106150947	106150947	-	106150947	24	24	---	---	intergenic	---
7	106167138	106167138	+	106167138	4	4	XLOC_079743	+	predicted	---
7	106226181	106226181	+	106226181	4	4	ENSSSCG00000029760	+	ORF	*CIPC*
7	106229521	106229524	+	106229523	27	48	ENSSSCG00000029760	+	3'UTR	*CIPC*
7	106472502	106472508	+	106472506	24	45	XLOC_079750	+	predicted	---
7	106479434	106479434	+	106479434	4	4	ENSSSCG00000030069	+	intron	*NOXRED1*
7	106492005	106492005	-	106492005	12	12	XLOC_081008	-	predicted	---
7	106492721	106492721	+	106492721	4	4	XLOC_079750	+	predicted	---
7	106492875	106492876	-	106492876	7	10	XLOC_081008	-	predicted	---
7	106493256	106493256	-	106493256	5	5	XLOC_081008	-	predicted	---
7	106506802	106506802	-	106506802	3	3	ENSSSCG00000021242	-	ORF	---
7	106545910	106545911	+	106545910	4	8	XLOC_079751	+	predicted	---
7	106553157	106553168	-	106553165	33	54	ENSSSCG00000002396	-	3'UTR	*GSTZ1*
7	106554244	106554248	-	106554247	76	166	ENSSSCG00000002396	-	3'UTR	*GSTZ1*
							ENSSSCG00000002396	-	intron	*GSTZ1*
7	106554339	106554363	-	106554350	34	133	ENSSSCG00000002396	-	3'UTR	*GSTZ1*
							ENSSSCG00000002396	-	intron	*GSTZ1*

7	106604418	106604419	+	106604419	22	31	XLOC_079752	+	predicted	---
7	106618507	106618507	+	106618507	4	4	---	---	intergenic	---
7	106699096	106699096	+	106699096	6	6	ENSSSCG00000002404	+	intron	*SPTLC2*
7	106729560	106729560	+	106729560	3	3	ENSSSCG00000002404	+	3'UTR	*SPTLC2*
7	106734155	106734162	+	106734162	8	14	ENSSSCG00000002404	+	3'UTR	*SPTLC2*
7	106792529	106792529	-	106792529	4	4	---	---	intergenic	---
7	106966030	106966030	-	106966030	4	4	---	---	intergenic	---
7	107026394	107026396	+	107026394	6	10	---	---	intergenic	---
7	107445711	107445711	-	107445711	19	19	---	---	intergenic	---
7	108521251	108521251	+	108521251	3	3	ENSSSCG00000028718	+	intron	---
7	108772276	108772277	+	108772276	9	12	---	---	intergenic	---
7	109628258	109628258	+	109628258	3	3	---	---	intergenic	---
7	109983341	109983342	-	109983341	5	8	ENSSSCG00000002411	-	intron	*GTF2A1*
7	110049117	110049117	-	110049117	4	4	ENSSSCG00000002412	-	intron	*STON2*
7	110129304	110129305	-	110129304	278	431	ENSSSCG00000002412	-	intron	*STON2*
7	110382521	110382526	-	110382522	32	64	XLOC_081022	-	predicted	---
7	110385770	110385770	-	110385770	5	5	XLOC_081022	-	predicted	---
7	110438699	110438700	-	110438699	8	11	ENSSSCG00000002414	-	ORF	*SEL1L*
7	110846178	110846178	-	110846178	3	3	---	---	intergenic	---
7	110924208	110924208	+	110924208	4	4	---	---	intergenic	---
7	111239114	111239114	+	111239114	3	3	---	---	intergenic	---
7	112080354	112080354	+	112080354	6	6	---	---	intergenic	---
7	112444524	112444524	-	112444524	6	6	---	---	intergenic	---
7	112495761	112495761	-	112495761	3	3	---	---	intergenic	---
7	112570123	112570126	+	112570125	6	14	---	---	intergenic	---
7	112666144	112666145	+	112666145	9	13	---	---	intergenic	---

7	113383652	113383652	+	113383652	3	3	---	---	intergenic	---
7	114363112	114363112	+	114363112	3	3	XLOC_079775	+	predicted	---
7	115304759	115304760	-	115304760	12	18	---	---	intergenic	---
7	115304946	115304946	-	115304946	5	5	---	---	intergenic	---
7	115363290	115363290	-	115363290	4	4	---	---	intergenic	---
7	115363347	115363357	+	115363352	23	87	---	---	intergenic	---
7	115411228	115411228	-	115411228	11	11	---	---	intergenic	---
7	115579771	115579771	+	115579771	4	4	---	---	intergenic	---
7	115952401	115952401	+	115952401	3	3	---	---	intergenic	---
7	116142058	116142076	+	116142076	19	22	---	---	intergenic	---
7	116255755	116255755	+	116255755	4	4	---	---	intergenic	---
7	116348933	116348934	-	116348933	29	32	ENSSSCG00000027865	-	3'UTR	*GALC*
7	116567039	116567039	-	116567039	4	4	XLOC_081029	-	predicted	---
7	116748895	116748895	+	116748895	13	13	---	---	intergenic	---
7	116771046	116771046	+	116771046	5	5	---	---	intergenic	---
7	116846522	116846522	+	116846522	3	3	ENSSSCG00000002424	+	ORF	*SPATA7*
7	116846840	116846840	+	116846840	4	4	ENSSSCG00000002424	+	intron	*SPATA7*
7	116857662	116857664	+	116857664	6	9	ENSSSCG00000002424	+	intron	*SPATA7*
7	116861829	116861829	+	116861829	3	3	ENSSSCG00000002424	+	ORF	*SPATA7*
7	116862316	116862333	+	116862324	104	421	ENSSSCG00000002424	+	3'UTR	*SPATA7*
7	116873345	116873346	-	116873345	8	12	XLOC_081031	-	predicted	---
7	116952951	116952951	-	116952951	4	4	---	---	intergenic	---
7	117008373	117008399	+	117008381	80	231	XLOC_079785	+	predicted	---
7	117008522	117008531	+	117008529	49	85	XLOC_079785	+	predicted	---
7	117012099	117012099	-	117012099	7	7	ENSSSCG00000002427	-	3'UTR	*EML5*
7	117151810	117151819	-	117151812	7	41	ENSSSCG00000002427	-	intron	*EML5*

7	117274076	117274087	+	117274084	6	15	ENSSSCG00000002428	+	3'UTR	*TTC8*
7	117328181	117328182	+	117328182	8	11	---	---	intergenic	---
7	117526000	117526002	-	117526001	33	42	XLOC_081036	-	predicted	---
7	117531238	117531238	-	117531238	3	3	ENSSSCG00000002429	-	3'UTR	*FOXN3*
7	117532284	117532285	-	117532284	4	7	ENSSSCG00000002429	-	3'UTR	*FOXN3*
7	117544221	117544221	-	117544221	9	9	ENSSSCG00000002429	-	intron	*FOXN3*
7	117922484	117922484	+	117922484	4	4	---	---	intergenic	---
7	118105208	118105211	-	118105210	16	27	---	---	intergenic	---
7	118106804	118106805	+	118106804	4	7	---	---	intergenic	---
7	118181821	118181836	-	118181831	27	137	ENSSSCG00000002430	-	intron	*EFCAB11*
7	118204206	118204206	-	118204206	4	4	ENSSSCG00000002430	-	intron	*EFCAB11*
7	118250493	118250493	-	118250493	4	4	ENSSSCG00000002430	-	intron	*EFCAB11*
7	118331696	118331696	-	118331696	3	3	---	---	intergenic	---
7	118546539	118546539	+	118546539	3	3	ENSSSCG00000002432	+	3'UTR	*KCNK13*
7	118607527	118607527	+	118607527	3	3	ENSSSCG00000002433	+	ORF	*PSMC1*
7	118607769	118607776	+	118607771	231	357	ENSSSCG00000002433	+	3'UTR	*PSMC1*
7	118715051	118715051	+	118715051	4	4	---	---	intergenic	---
7	118735210	118735238	+	118735238	15	18	ENSSSCG00000025768	+	3'UTR	*CALM1*
7	118736021	118736029	+	118736025	25	92	ENSSSCG00000025768	+	3'UTR	*CALM1*
7	118738722	118738722	+	118738722	3	3	ENSSSCG00000025768	+	3'UTR	*CALM1*
7	118738909	118738939	+	118738938	30	41	ENSSSCG00000025768	+	3'UTR	*CALM1*
7	118821204	118821205	+	118821205	100	105	---	---	intergenic	---
7	118837347	118837347	-	118837347	3	3	ENSSSCG00000002436	-	3'UTR	---
7	118912199	118912199	-	118912199	3	3	ENSSSCG00000002436	-	intron	---
7	119663860	119663861	-	119663860	5	8	ENSSSCG00000002441	-	3'UTR	*SMEK1*
7	119664258	119664259	-	119664259	4	7	ENSSSCG00000002441	-	3'UTR	*SMEK1*

7	119666600	119666600	-	119666600	3	3	ENSSSCG00000002441	-	ORF	*SMEK1*
7	119701442	119701442	+	119701442	3	3	---	---	intergenic	---
7	119753812	119753813	-	119753813	10	17	XLOC_081050	-	predicted	---
7	119753900	119753900	-	119753900	3	3	ENSSSCG00000002442	-	3'UTR	*CATSPERB*
7	120031698	120031713	-	120031704	14	34	ENSSSCG00000002444	-	3'UTR	*FBLN5*
7	120135372	120135373	-	120135373	6	9	XLOC_081055	-	predicted	---
7	120176945	120176945	-	120176945	4	4	ENSSSCG00000002445	-	ORF	*TRIP11*
7	120181368	120181368	-	120181368	12	12	ENSSSCG00000002445	-	ORF	*TRIP11*
7	120187922	120187922	-	120187922	3	3	ENSSSCG00000002445	-	ORF	*TRIP11*
7	120216381	120216381	-	120216381	7	7	XLOC_081056	-	predicted	---
7	120220365	120220367	-	120220367	4	7	ENSSSCG00000002446	-	3'UTR	*ATXN3*
7	120232898	120232907	-	120232900	17	36	ENSSSCG00000002446	-	intron	*ATXN3*
7	120289175	120289185	-	120289182	8	19	ENSSSCG00000002448	-	3'UTR	---
7	120289431	120289431	-	120289431	6	6	ENSSSCG00000002448	-	3'UTR	---
7	120296369	120296376	-	120296370	47	113	ENSSSCG00000002449	-	3'UTR	*NDUFB1*
7	120389300	120389300	+	120389300	3	3	XLOC_079811	+	predicted	---
7	120403364	120403370	-	120403370	13	16	---	---	intergenic	---
7	120771247	120771254	+	120771247	13	19	ENSSSCG00000002451	+	3'UTR	*RIN3*
7	120778059	120778059	+	120778059	6	6	---	---	intergenic	---
7	120780926	120780941	-	120780928	192	415	---	---	intergenic	---
7	120781027	120781034	-	120781033	8	16	---	---	intergenic	---
7	120787144	120787147	-	120787144	5	13	---	---	intergenic	---
7	120882775	120882777	+	120882777	11	14	XLOC_079815	+	predicted	---
7	120970446	120970456	-	120970449	6	18	XLOC_081067	-	predicted	---
7	120970529	120970542	-	120970542	15	25	ENSSSCG00000002457	-	3'UTR	*ITPK1*
7	121159245	121159245	-	121159245	9	9	XLOC_081068	-	predicted	---

7	121161712	121161712	+	121161712	3	3	ENSSSCG00000002458	+	3'UTR	*TMEM251*
7	121161887	121161887	+	121161887	5	5	ENSSSCG00000002458	+	3'UTR	*TMEM251*
7	121172007	121172007	-	121172007	4	4	ENSSSCG00000002459	-	3'UTR	*C14orf142*
7	121172073	121172081	-	121172079	77	100	ENSSSCG00000002459	-	3'UTR	*C14orf142*
7	121172133	121172133	-	121172133	3	3	ENSSSCG00000002459	-	3'UTR	*C14orf142*
7	121192969	121192970	+	121192969	10	15	ENSSSCG00000002460	+	3'UTR	---
7	121193216	121193223	+	121193221	10	32	ENSSSCG00000002460	+	3'UTR	---
7	121195055	121195055	+	121195055	8	8	ENSSSCG00000002460	+	3'UTR	---
7	121202697	121202704	-	121202702	8	19	ENSSSCG00000002461	-	3'UTR	*BTBD7*
7	121314175	121314175	+	121314175	3	3	ENSSSCG00000002462	+	3'UTR	*COX8C*
7	121314308	121314325	+	121314311	57	186	ENSSSCG00000002462	+	3'UTR	*COX8C*
7	121367522	121367522	+	121367522	3	3	---	---	intergenic	---
7	121501905	121501905	-	121501905	6	6	---	---	intergenic	---
7	121627970	121627972	-	121627972	6	9	---	---	intergenic	---
7	121655541	121655541	+	121655541	3	3	XLOC_079825	+	predicted	---
7	121887164	121887171	+	121887171	7	13	XLOC_079826	+	predicted	---
7	121891391	121891394	-	121891392	11	30	XLOC_081076	-	predicted	---
7	121981570	121981570	+	121981570	3	3	---	---	intergenic	---
7	122048261	122048261	-	122048261	3	3	---	---	intergenic	---
7	122139524	122139539	-	122139531	11	31	XLOC_081077	-	predicted	---
7	122146708	122146716	+	122146709	14	37	XLOC_079829	+	predicted	---
7	122146931	122146931	+	122146931	3	3	---	---	intergenic	---
7	122151972	122151979	+	122151973	26	85	XLOC_079830	+	predicted	---
7	122181861	122181973	+	122181906	20	160	ENSSSCG00000002469	+	3'UTR	*OTUB2*
7	122182027	122182057	+	122182048	10	50	ENSSSCG00000002469	+	3'UTR	*OTUB2*
7	122182349	122182350	+	122182349	11	16	ENSSSCG00000002469	+	3'UTR	*OTUB2*

7	122184445	122184445	+	122184445	3	3	XLOC_079831	+	predicted	---
7	122205527	122205527	-	122205527	4	4	ENSSSCG00000002470	-	ORF	---
7	122220518	122220526	+	122220520	170	264	ENSSSCG00000002471	+	3'UTR	*ISG12(A)*
7	122228145	122228147	-	122228146	21	28	XLOC_081079	-	predicted	---
7	122232914	122232914	+	122232914	5	5	---	---	intergenic	---
7	122374845	122374855	-	122374852	71	217	---	---	intergenic	---
7	122544750	122544750	-	122544750	5	5	XLOC_081081	-	predicted	---
7	122544998	122545019	-	122545019	12	25	ENSSSCG00000002475	-	3'UTR	*SERPINA6*
7	122545084	122545091	-	122545086	89	201	ENSSSCG00000002475	-	3'UTR	*SERPINA6*
7	122616175	122616176	-	122616176	313	316	ENSSSCG00000002476	-	ORF	*SERPINA1*
7	122616199	122616268	-	122616223	28	40	ENSSSCG00000002476	-	ORF	*SERPINA1*
7	122620868	122620868	-	122620868	4	4	ENSSSCG00000002476	-	intron	*SERPINA1*
7	122620944	122620945	-	122620944	11	22	ENSSSCG00000002476	-	ORF	*SERPINA1*
7	122620985	122620985	-	122620985	4	4	ENSSSCG00000002476	-	ORF	*SERPINA1*
7	122785168	122785200	-	122785175	255	469	---	---	intergenic	---
7	122909004	122909007	+	122909004	4	7	XLOC_079838	+	predicted	---
7	122909065	122909065	+	122909065	9	9	XLOC_079838	+	predicted	---
7	122922446	122922446	+	122922446	3	3	ENSSSCG00000002481	+	ORF	*SERPINA5*
7	122926711	122926719	+	122926718	5	11	ENSSSCG00000002481	+	3'UTR	*SERPINA5*
7	122927234	122927235	+	122927235	14	19	ENSSSCG00000002481	+	3'UTR	*SERPINA5*
7	122927414	122927426	+	122927416	1387	1702	ENSSSCG00000002481	+	3'UTR	*SERPINA5*
7	122934056	122934057	+	122934056	6	11	XLOC_079840	+	predicted	---
7	122938444	122938469	+	122938466	513	1608	XLOC_079841	+	predicted	---
7	122968425	122968426	+	122968425	6	9	ENSSSCG00000030371	+	ORF	---
7	122969594	122969626	+	122969596	28	44	ENSSSCG00000030371	+	ORF	---
7	122969824	122969856	+	122969847	1429	5750	ENSSSCG00000030371	+	3'UTR	---

7	122999960	122999961	+	122999961	5	8	XLOC_079840	+	predicted	---
7	123002480	123002480	+	123002480	3	3	XLOC_079840	+	predicted	---
7	123002744	123002769	+	123002761	627	1573	XLOC_079840	+	predicted	---
7	123016236	123016262	-	123016244	727	2208	ENSSSCG00000002483	-	3'UTR	*SERPINA3-3*
7	123022522	123022522	-	123022522	4	4	ENSSSCG00000002483	-	ORF	*SERPINA3-3*
7	123094659	123094670	-	123094661	511	1630	ENSSSCG00000002483	-	intron	*SERPINA3-3*
7	123096112	123096112	-	123096112	6	6	ENSSSCG00000002483	-	intron	*SERPINA3-3*
							ENSSSCG00000002486	-	ORF	---
7	123098289	123098289	-	123098289	3	3	ENSSSCG00000002486	-	intron	---
							ENSSSCG00000002483	-	intron	*SERPINA3-3*
7	123110534	123110562	-	123110534	60	229	ENSSSCG00000002487	-	3'UTR	*SERPINA3-2*
7	123110784	123110784	-	123110784	11	11	ENSSSCG00000002487	-	ORF	*SERPINA3-2*
7	123112277	123112277	-	123112277	7	7	ENSSSCG00000002487	-	ORF	*SERPINA3-2*
7	123116844	123116844	-	123116844	6	6	ENSSSCG00000002487	-	ORF	*SERPINA3-2*
7	123303125	123303131	+	123303125	6	9	---	---	intergenic	---
7	123314094	123314094	+	123314094	4	4	---	---	intergenic	---
7	123581786	123581797	-	123581797	4	7	XLOC_081096	-	predicted	---
7	123656191	123656191	+	123656191	4	4	---	---	intergenic	---
7	123657925	123657925	+	123657925	3	3	---	---	intergenic	---
7	123684760	123684769	-	123684769	6	14	XLOC_081097	-	predicted	---
7	123694660	123694667	-	123694666	24	55	ENSSSCG00000002494	-	3'UTR	*CLMN*
7	123697501	123697503	-	123697501	3	6	ENSSSCG00000002494	-	intron	*CLMN*
7	123714273	123714273	-	123714273	3	3	ENSSSCG00000002494	-	intron	*CLMN*
7	123808326	123808330	-	123808328	15	37	XLOC_081100	-	predicted	---
7	123880275	123880275	-	123880275	8	8	XLOC_081101	-	predicted	---
7	123887288	123887288	-	123887288	4	4	XLOC_081101	-	predicted	---

7	123995393	123995393	+	123995393	4	4	---	---	intergenic	---
7	123999278	123999282	-	123999280	56	105	XLOC_081102	-	predicted	---
7	124011541	124011541	+	124011541	6	6	ENSSSCG00000002496	+	3'UTR	*GLRX5*
7	124011609	124011626	+	124011625	13	27	ENSSSCG00000002496	+	3'UTR	*GLRX5*
7	124011736	124011737	+	124011737	4	7	ENSSSCG00000002496	+	3'UTR	*GLRX5*
7	124148802	124148803	-	124148802	3	6	XLOC_081103	-	predicted	---
7	124474138	124474138	+	124474138	3	3	---	---	intergenic	---
7	124564102	124564102	+	124564102	14	14	---	---	intergenic	---
7	124690853	124690853	+	124690853	4	4	---	---	intergenic	---
7	124723493	124723493	-	124723493	3	3	---	---	intergenic	---
7	124747970	124747971	+	124747970	6	12	---	---	intergenic	---
7	124764158	124764158	-	124764158	5	5	XLOC_081107	-	predicted	---
7	124863865	124863867	+	124863866	11	18	ENSSSCG00000022930	+	3'UTR	*GSKIP*
7	124865534	124865534	+	124865534	5	5	ENSSSCG00000022930	+	3'UTR	*GSKIP*
7	124865661	124865661	+	124865661	3	3	XLOC_079861	+	predicted	---
7	124878141	124878141	+	124878141	7	7	ENSSSCG00000002504	+	intron	*AK7*
7	124897077	124897077	+	124897077	3	3	ENSSSCG00000002504	+	intron	*AK7*
7	124919961	124919961	+	124919961	5	5	ENSSSCG00000002504	+	ORF	*AK7*
7	124925579	124925584	+	124925579	10	30	XLOC_079862	+	predicted	---
7	124963499	124963508	+	124963507	27	61	ENSSSCG00000002505	+	intron	*PAPOLA*
7	124968362	124968362	+	124968362	3	3	ENSSSCG00000002505	+	intron	*PAPOLA*
7	124997272	124997272	+	124997272	7	7	---	---	intergenic	---
7	124999346	124999354	+	124999354	9	23	ENSSSCG00000002505	+	3'UTR	*PAPOLA*
							ENSSSCG00000002505	+	ORF	*PAPOLA*
7	125140573	125140573	+	125140573	7	7	---	---	intergenic	---
7	125289003	125289003	+	125289003	4	4	ENSSSCG00000002506	+	ORF	*VRK1*

7	125392454	125392454	+	125392454	3	3	---	---	intergenic	---
7	125665191	125665191	-	125665191	3	3	---	---	intergenic	---
7	125723128	125723128	-	125723128	9	9	XLOC_081114	-	predicted	---
7	125819306	125819306	+	125819306	3	3	---	---	intergenic	---
7	125821624	125821630	-	125821626	5	11	---	---	intergenic	---
7	126016876	126016876	-	126016876	3	3	---	---	intergenic	---
7	126229022	126229022	+	126229022	4	4	---	---	intergenic	---
7	126286908	126286915	+	126286914	8	20	---	---	intergenic	---
7	126617728	126617728	+	126617728	3	3	XLOC_079871	+	predicted	---
7	127593560	127593560	-	127593560	5	5	---	---	intergenic	---
7	127593583	127593586	+	127593583	18	41	---	---	intergenic	---
7	127596269	127596269	+	127596269	5	5	---	---	intergenic	---
7	127707826	127707826	-	127707826	6	6	---	---	intergenic	---
7	127923423	127923423	-	127923423	5	5	XLOC_081122	-	predicted	---
7	127972349	127972353	-	127972352	32	45	XLOC_081123	-	predicted	---
7	128123850	128123858	+	128123857	28	67	ENSSSCG00000002509	+	3'UTR	*CCNK*
7	128124057	128124065	+	128124058	34	49	ENSSSCG00000002509	+	3'UTR	*CCNK*
7	128302547	128302547	+	128302547	3	3	ENSSSCG00000002510	+	3'UTR	*CYP46A1*
7	128384306	128384306	-	128384306	3	3	ENSSSCG00000020788	-	intron	---
7	128588361	128588361	+	128588361	7	7	XLOC_079881	+	predicted	---
7	128588613	128588614	+	128588613	3	6	XLOC_079881	+	predicted	---
7	128631519	128631521	+	128631521	5	9	XLOC_079882	+	predicted	---
7	128631627	128631630	+	128631629	12	20	XLOC_079882	+	predicted	---
7	128635269	128635276	+	128635269	8	11	XLOC_079882	+	predicted	---
7	128642793	128642793	-	128642793	3	3	ENSSSCG00000002520	-	3'UTR	*SLC25A29*
7	128674943	128674964	+	128674961	209	326	XLOC_079884	+	predicted	---

7	128677568	128677570	-	128677570	16	35	XLOC_081134	-	predicted	---
7	128678192	128678199	-	128678192	7	11	ENSSSCG00000002516	-	intron	*WARS*
7	128824319	128824319	-	128824319	4	4	ENSSSCG00000002523	-	3'UTR	*CDC42BPB*
7	128907891	128907891	-	128907891	7	7	ENSSSCG00000002525	-	3'UTR	*TRAF3_TV1*
7	129064898	129064898	-	129064898	4	4	XLOC_081140	-	predicted	---
7	129067374	129067374	-	129067374	3	3	XLOC_081140	-	predicted	---
7	129089897	129089897	-	129089897	11	11	ENSSSCG00000002526	-	intron	*RCOR1*
7	129170468	129170468	-	129170468	4	4	ENSSSCG00000002526	-	intron	*RCOR1*
7	129231357	129231360	-	129231358	17	34	XLOC_081143	-	predicted	---
7	129271606	129271647	+	129271641	25	65	ENSSSCG00000002527	+	3'UTR	*ANKRD9*
							ENSSSCG00000002527	+	ORF	---
7	129275120	129275120	-	129275120	3	3	ENSSSCG00000024055	-	3'UTR	*TECPR2*
7	129278049	129278055	-	129278049	7	11	ENSSSCG00000024055	-	3'UTR	*TECPR2*
7	129322142	129322177	-	129322177	49	59	ENSSSCG00000002529	-	ORF	---
7	129324532	129324532	+	129324532	3	3	---	---	intergenic	---
7	129374238	129374238	-	129374238	3	3	---	---	intergenic	---
7	129472755	129472755	+	129472755	3	3	ENSSSCG00000002532	+	3'UTR	*ZNF839*
7	129476636	129476642	-	129476642	12	42	---	---	intergenic	---
7	129582755	129582755	+	129582755	4	4	ENSSSCG00000002531	+	3'UTR	---
7	129596923	129596923	-	129596923	13	13	ENSSSCG00000002533	-	intron	*WDR20*
7	129597210	129597216	-	129597210	6	13	ENSSSCG00000002533	-	intron	*WDR20*
7	129696955	129696955	-	129696955	5	5	ENSSSCG00000002533	-	intron	*WDR20*
7	129723009	129723009	-	129723009	6	6	XLOC_081151	-	predicted	---
7	129747646	129747674	+	129747647	5	12	XLOC_079897	+	predicted	---
7	129759203	129759236	+	129759205	15	26	ENSSSCG00000002535	+	ORF	*HSP90AA1*
7	129759269	129759297	+	129759269	4	11	ENSSSCG00000002535	+	ORF	*HSP90AA1*

7	129759646	129759646	+	129759646	3	3	ENSSSCG00000002535	+	ORF	*HSP90AA1*
7	129759711	129759712	+	129759712	6	9	ENSSSCG00000002535	+	ORF	*HSP90AA1*
7	129760367	129760367	+	129760367	3	3	ENSSSCG00000002535	+	3'UTR	*HSP90AA1*
7	129760437	129760465	+	129760459	37	81	ENSSSCG00000002535	+	3'UTR	*HSP90AA1*
7	129760503	129760511	+	129760509	170	342	ENSSSCG00000002535	+	3'UTR	*HSP90AA1*
7	129760663	129760663	+	129760663	7	7	ENSSSCG00000002535	+	3'UTR	*HSP90AA1*
7	129760810	129760810	+	129760810	15	15	ENSSSCG00000002535	+	3'UTR	*HSP90AA1*
7	129760956	129760977	+	129760957	253	585	ENSSSCG00000002535	+	3'UTR	*HSP90AA1*
7	129787811	129787811	+	129787811	4	4	ENSSSCG00000002536	+	ORF	---
7	129788143	129788169	+	129788167	19	150	XLOC_079898	+	predicted	---
7	129791251	129791263	-	129791256	84	150	XLOC_081155	-	predicted	---
7	129829954	129829954	+	129829954	3	3	---	---	intergenic	---
7	129836885	129836885	+	129836885	3	3	---	---	intergenic	---
7	129850587	129850588	-	129850588	21	24	XLOC_081156	-	predicted	---
7	129850642	129850642	-	129850642	15	15	XLOC_081156	-	predicted	---
7	129850810	129850810	-	129850810	5	5	XLOC_081156	-	predicted	---
7	129864472	129864480	-	129864474	19	37	ENSSSCG00000002540	-	intron	*PPP2R5C*
7	129865920	129865928	-	129865928	4	7	ENSSSCG00000002540	-	ORF	*PPP2R5C*
7	129921364	129921364	-	129921364	4	4	ENSSSCG00000002540	-	intron	*PPP2R5C*
7	129958853	129958853	-	129958853	3	3	ENSSSCG00000002540	-	intron	*PPP2R5C*
7	129978379	129978379	-	129978379	3	3	---	---	intergenic	---
7	129995508	129995508	-	129995508	3	3	ENSSSCG00000002540	-	intron	*PPP2R5C*
7	130054078	130054078	-	130054078	3	3	---	---	intergenic	---
7	130054113	130054113	+	130054113	7	7	---	---	intergenic	---
7	130214632	130214633	+	130214632	3	6	XLOC_079903	+	predicted	---
7	130286338	130286338	+	130286338	3	3	---	---	intergenic	---

7	130397600	130397600	+	130397600	8	8	---	---	intergenic	---
7	130821145	130821149	+	130821146	8	18	ENSSSCG00000002544	+	3'UTR	*PPP1R13B*
7	130821390	130821390	-	130821390	20	20	XLOC_081168	-	predicted	---
7	130935934	130935946	-	130935943	18	44	XLOC_081170	-	predicted	---
7	130945624	130945624	-	130945624	9	9	---	---	intergenic	---
7	130952647	130952647	+	130952647	5	5	---	---	intergenic	---
7	130967899	130967899	+	130967899	4	4	---	---	intergenic	---
7	131028034	131028042	-	131028036	222	470	XLOC_081173	-	predicted	---
7	131028180	131028180	-	131028180	3	3	ENSSSCG00000002550	-	3'UTR	*C14orf2*
7	131035634	131035634	+	131035634	4	4	---	---	intergenic	---
7	131119915	131119915	+	131119915	12	12	XLOC_079913	+	predicted	---
7	131141555	131141555	-	131141555	3	3	XLOC_081177	-	predicted	---
7	131203584	131203584	-	131203584	3	3	---	---	intergenic	---
7	131238638	131238638	-	131238638	3	3	ENSSSCG00000002555	-	intron	*JAG2*
7	131249503	131249503	-	131249503	8	8	ENSSSCG00000002557	-	3'UTR	*UGPP*
7	131262408	131262408	-	131262408	3	3	XLOC_081181	-	predicted	---
7	131334783	131334783	+	131334783	3	3	---	---	intergenic	---
7	131347957	131347957	+	131347957	11	11	ENSSSCG00000002554	+	3'UTR	*PLD4*
7	131360857	131360857	-	131360857	3	3	---	---	intergenic	---
7	131386135	131386140	-	131386136	5	12	XLOC_081185	-	predicted	---
7	133276754	133276755	+	133276754	6	11	---	---	intergenic	---
7	134041676	134041684	+	134041679	50	147	XLOC_079963	+	predicted	---
7	134044496	134044501	-	134044501	10	15	---	---	intergenic	---
7	134069904	134069904	-	134069904	5	5	---	---	intergenic	---
7	134127362	134127362	+	134127362	19	19	---	---	intergenic	---
7	134192023	134192023	+	134192023	4	4	---	---	intergenic	---

7	134211578	134211578	-	134211578	7	7	ENSSSCG00000002622	-	3'UTR	*TMEM14A*
7	134268608	134268609	+	134268608	6	9	---	---	intergenic	---
7	134270044	134270078	-	134270078	8	11	XLOC_081232	-	predicted	---
7	134289612	134289632	-	134289623	993	1931	ENSSSCG00000002623	-	3'UTR	---
7	134289709	134289751	-	134289744	127	328	ENSSSCG00000002623	-	3'UTR	---
7	134289822	134289822	-	134289822	3	3	ENSSSCG00000002623	-	3'UTR	---
7	134289886	134289886	-	134289886	6	6	ENSSSCG00000002623	-	ORF	---
7	134300498	134300512	-	134300498	3	6	ENSSSCG00000002623	-	intron	---
7	134315816	134315821	-	134315818	35	51	XLOC_081233	-	predicted	---
7	134354555	134354569	-	134354564	80	145	XLOC_081235	-	predicted	---
7	134357802	134357802	-	134357802	4	4	ENSSSCG00000023467	-	ORF	---
7	134379986	134379996	-	134379994	118	184	ENSSSCG00000002627	-	3'UTR	*GSTA4*
7	134399311	134399312	-	134399311	5	8	ENSSSCG00000002627	-	ORF	*GSTA4*
7	134402670	134402670	-	134402670	6	6	ENSSSCG00000002628	-	3'UTR	*ICK*
7	134490738	134490741	+	134490741	30	48	XLOC_079969	+	predicted	---
7	134545483	134545493	-	134545490	40	130	XLOC_081241	-	predicted	---
7	134546210	134546210	-	134546210	6	6	XLOC_081241	-	predicted	---
7	134558496	134558496	+	134558496	4	4	---	---	intergenic	---
8	54348	54348	-	54348	3	3	---	---	intergenic	---
8	76979	76979	-	76979	4	4	XLOC_085271	-	predicted	---
8	80088	80100	-	80088	4	11	XLOC_085271	-	predicted	---
8	150067	150082	-	150080	167	405	ENSSSCG00000023903	-	3'UTR	*ATP5I*
8	150422	150422	-	150422	5	5	ENSSSCG00000023903	-	intron	*ATP5I*
8	158069	158077	-	158075	16	25	ENSSSCG00000026084	-	3'UTR	*MFSD7*
8	296948	296957	-	296956	28	69	ENSSSCG00000008677	-	3'UTR	*TACC3*
8	297260	297268	-	297268	6	9	ENSSSCG00000008677	-	ORF	*TACC3*

8	312535	312535	+	312535	3	3	ENSSSCG00000008678	+	3'UTR	*TMEM129*
8	312611	312622	+	312618	21	45	ENSSSCG00000008678	+	3'UTR	*TMEM129*
8	321601	321607	-	321602	33	51	ENSSSCG00000008676	-	3'UTR	*SLBP*
8	342574	342577	-	342575	6	15	---	---	intergenic	---
8	344315	344319	+	344316	27	68	XLOC_084460	+	predicted	---
8	347535	347537	-	347536	20	27	XLOC_085278	-	predicted	---
8	347790	347792	-	347792	5	8	XLOC_085278	-	predicted	---
8	349642	349642	-	349642	12	12	ENSSSCG00000008675	-	3'UTR	*LETM1*
8	386310	386310	-	386310	3	3	---	---	intergenic	---
8	441789	441789	+	441789	3	3	ENSSSCG00000008682	+	intron	*WHSC1*
8	512680	512680	+	512680	4	4	XLOC_084462	+	predicted	---
8	621836	621836	-	621836	3	3	ENSSSCG00000008684	-	intron	*POLN*
							ENSSSCG00000008681	-	intron	*NELFA*
8	645874	645881	+	645881	8	41	ENSSSCG00000008686	+	3'UTR	*C4orf48*
8	679724	679740	-	679730	11	20	XLOC_085283	-	predicted	---
8	695003	695003	-	695003	5	5	XLOC_085285	-	predicted	---
8	697675	697675	-	697675	8	8	XLOC_085285	-	predicted	---
8	917478	917479	-	917478	8	11	---	---	intergenic	---
8	943637	943637	+	943637	3	3	ENSSSCG00000008690	+	3'UTR	*RNF4*
8	944200	944200	+	944200	3	3	ENSSSCG00000008690	+	3'UTR	*RNF4*
8	945478	945481	+	945481	48	63	XLOC_084470	+	predicted	---
8	947253	947257	-	947257	8	13	XLOC_085290	-	predicted	---
8	1091335	1091335	+	1091335	3	3	ENSSSCG00000029745	+	intron	*FAM193A*
8	1102202	1102202	+	1102202	6	6	ENSSSCG00000029745	+	intron	*FAM193A*
8	1106801	1106801	+	1106801	7	7	XLOC_084471	+	predicted	---
8	1115216	1115220	-	1115216	6	12	ENSSSCG00000008691	-	3'UTR	*TNIP2*

8	1201282	1201283	+	1201282	5	9	ENSSSCG00000022004	+	3'UTR	*SH3BP2*
8	1292445	1292445	-	1292445	3	3	XLOC_085295	-	predicted	---
8	1292565	1292568	+	1292568	13	22	XLOC_084473	+	predicted	---
8	1293022	1293022	-	1293022	5	5	XLOC_085295	-	predicted	---
8	1298945	1298945	-	1298945	13	13	ENSSSCG00000008693	-	3'UTR	*NOP14*
8	1387160	1387160	+	1387160	3	3	ENSSSCG00000008694	+	intron	*GRK4*
8	1444418	1444424	+	1444422	94	211	ENSSSCG00000008694	+	3'UTR	*GRK4*
8	1457556	1457556	-	1457556	4	4	XLOC_085298	-	predicted	---
8	1583524	1583524	+	1583524	6	6	ENSSSCG00000008697	+	3'UTR	*HUNTINGTIN*
8	1586204	1586204	+	1586204	4	4	ENSSSCG00000008697	+	3'UTR	*HUNTINGTIN*
8	1611717	1611717	-	1611717	3	3	XLOC_085299	-	predicted	---
8	1614515	1614515	+	1614515	3	3	XLOC_084477	+	predicted	---
8	1757617	1757630	-	1757629	149	327	ENSSSCG00000008700	-	3'UTR	*HGFAC*
8	1832080	1832080	+	1832080	3	3	ENSSSCG00000008701	+	ORF	*LREAP1*
8	1842652	1842661	+	1842659	215	438	ENSSSCG00000008701	+	3'UTR	*LREAP1*
8	1859749	1859749	-	1859749	3	3	XLOC_085300	-	predicted	---
8	2486631	2486631	+	2486631	3	3	XLOC_084487	+	predicted	---
8	2486873	2486873	+	2486873	4	4	XLOC_084487	+	predicted	---
8	2851286	2851312	+	2851312	8	11	---	---	intergenic	---
8	3118981	3118981	+	3118981	5	5	ENSSSCG00000027746	+	3'UTR	*MRFAP1*
8	3119381	3119411	+	3119411	24	28	ENSSSCG00000027746	+	3'UTR	*MRFAP1*
8	3146221	3146221	+	3146221	5	5	XLOC_084493	+	predicted	---
8	3149041	3149041	+	3149041	3	3	XLOC_084493	+	predicted	---
8	3265310	3265319	+	3265319	10	24	XLOC_084494	+	predicted	---

8	3373746	3373746	+	3373746	8	8	ENSSSCG00000027349	+	3'UTR	*TBC1D14*
8	3375800	3375801	+	3375801	9	13	ENSSSCG00000027349	+	3'UTR	*TBC1D14*
8	3377818	3377818	-	3377818	3	3	XLOC_085326	-	predicted	---
8	3378651	3378651	-	3378651	3	3	XLOC_085326	-	predicted	---
8	3397258	3397267	-	3397259	24	47	ENSSSCG00000022867	-	3'UTR	*GRPEL1*
8	3397697	3397708	-	3397704	132	299	ENSSSCG00000022867	-	3'UTR	*GRPEL1*
8	3410660	3410660	-	3410660	11	11	ENSSSCG00000022867	-	intron	*GRPEL1*
							ENSSSCG00000022867	-	ORF	*GRPEL1*
8	3456758	3456759	-	3456758	10	15	---	---	intergenic	---
8	3566993	3566993	-	3566993	5	5	---	---	intergenic	---
8	3657929	3657929	-	3657929	4	4	---	---	intergenic	---
8	4154826	4154826	-	4154826	3	3	---	---	intergenic	---
8	4315587	4315587	-	4315587	5	5	---	---	intergenic	---
8	4342692	4342692	-	4342692	3	3	ENSSSCG00000008722	-	intron	---
8	4418719	4418721	+	4418720	14	24	ENSSSCG00000008724	+	3'UTR	*ACOX3*
8	4509900	4509902	-	4509900	8	14	ENSSSCG00000024014	-	3'UTR	---
8	4625589	4625589	+	4625589	3	3	---	---	intergenic	---
8	5450897	5450897	-	5450897	3	3	---	---	intergenic	---
8	5535542	5535545	-	5535543	17	53	---	---	intergenic	---
8	5613077	5613077	+	5613077	3	3	ENSSSCG00000025991	+	intron	---
8	5723529	5723529	-	5723529	4	4	ENSSSCG00000008731	-	intron	*OTOP1*
8	5745032	5745032	-	5745032	3	3	ENSSSCG00000008731	-	intron	*OTOP1*
8	5751463	5751469	-	5751469	8	13	ENSSSCG00000008732	-	3'UTR	*TMEM128*
8	5776295	5776300	-	5776296	24	58	XLOC_085351	-	predicted	---
8	5780907	5780907	-	5780907	5	5	ENSSSCG00000008729	-	ORF	*LYAR*
8	5784873	5784882	-	5784873	3	6	ENSSSCG00000008729	-	ORF	*LYAR*

8	5788216	5788216	-	5788216	8	8	ENSSSCG00000008729	-	ORF	*LYAR*
8	5788258	5788258	-	5788258	14	14	ENSSSCG00000008729	-	ORF	*LYAR*
8	6339882	6339888	+	6339883	17	32	XLOC_084522	+	predicted	---
8	6343579	6343586	+	6343579	5	11	XLOC_084522	+	predicted	---
8	6415893	6415893	-	6415893	4	4	---	---	intergenic	---
8	6487951	6487954	-	6487953	13	28	---	---	intergenic	---
8	7086798	7086798	-	7086798	4	4	---	---	intergenic	---
8	7234618	7234618	-	7234618	6	6	---	---	intergenic	---
8	7671370	7671370	-	7671370	3	3	---	---	intergenic	---
8	7684528	7684528	+	7684528	3	3	---	---	intergenic	---
8	7812246	7812246	-	7812246	4	4	---	---	intergenic	---
8	8072312	8072312	+	8072312	3	3	---	---	intergenic	---
8	8354788	8354788	-	8354788	5	5	---	---	intergenic	---
8	8407180	8407180	+	8407180	4	4	---	---	intergenic	---
8	8453604	8453605	-	8453604	3	6	---	---	intergenic	---
8	8481916	8481916	+	8481916	4	4	---	---	intergenic	---
8	8525669	8525669	+	8525669	3	3	ENSSSCG00000008733	+	3'UTR	*RAB28*
8	8525942	8525942	+	8525942	3	3	ENSSSCG00000008733	+	3'UTR	*RAB28*
8	8526248	8526248	+	8526248	11	11	ENSSSCG00000008733	+	3'UTR	*RAB28*
8	8571210	8571210	+	8571210	3	3	---	---	intergenic	---
8	8734470	8734470	-	8734470	3	3	ENSSSCG00000008735	-	3'UTR	*BOD1L1*
8	8748916	8748916	-	8748916	3	3	ENSSSCG00000008735	-	intron	*BOD1L1*
8	8824854	8824854	-	8824854	3	3	---	---	intergenic	---
8	9063940	9063940	+	9063940	3	3	---	---	intergenic	---
8	9093353	9093353	+	9093353	4	4	XLOC_084535	+	predicted	---
8	10006107	10006117	+	10006108	21	46	ENSSSCG00000008736	+	3'UTR	*CPEB2*

8	10009658	10009661	+	10009658	7	17	ENSSSCG00000008736	+	3'UTR	*CPEB2*
8	10093478	10093478	+	10093478	3	3	---	---	intergenic	---
8	10202995	10202996	-	10202996	9	15	---	---	intergenic	---
8	10479793	10479793	+	10479793	4	4	ENSSSCG00000008738	+	intron	*CC2D2A*
8	10563432	10563444	-	10563439	30	87	ENSSSCG00000008740	-	3'UTR	*FBXL5*
8	10593092	10593095	-	10593095	7	10	ENSSSCG00000008740	-	ORF	*FBXL5*
8	10720771	10720771	-	10720771	7	7	---	---	intergenic	---
8	10779912	10779912	+	10779912	3	3	ENSSSCG00000008742	+	3'UTR	*CD38*
8	10781268	10781268	+	10781268	3	3	XLOC_084542	+	predicted	---
8	10783078	10783078	+	10783078	18	18	XLOC_084542	+	predicted	---
8	10866627	10866630	-	10866627	9	28	ENSSSCG00000008745	-	3'UTR	*PROM1*
8	10884412	10884412	-	10884412	3	3	ENSSSCG00000008745	-	intron	*PROM1*
8	10902678	10902679	-	10902678	7	12	ENSSSCG00000008745	-	intron	*PROM1*
8	10911939	10911941	-	10911939	3	6	ENSSSCG00000008745	-	intron	*PROM1*
8	10932919	10932926	-	10932926	10	17	ENSSSCG00000008745	-	intron	*PROM1*
8	10936998	10937008	-	10937008	4	10	ENSSSCG00000008745	-	intron	*PROM1*
8	11016806	11016806	-	11016806	8	8	XLOC_085382	-	predicted	---
8	11018316	11018319	-	11018317	21	47	XLOC_085382	-	predicted	---
8	11074228	11074228	-	11074228	4	4	---	---	intergenic	---
8	11205000	11205000	+	11205000	3	3	---	---	intergenic	---
8	11224897	11224897	-	11224897	4	4	---	---	intergenic	---
8	11289470	11289470	-	11289470	11	11	XLOC_085383	-	predicted	---
8	11315778	11315781	-	11315781	5	8	ENSSSCG00000029227	-	intron	*LDB2*
8	11358685	11358686	-	11358686	4	7	ENSSSCG00000029227	-	intron	*LDB2*
8	11469444	11469444	-	11469444	9	9	ENSSSCG00000029227	-	intron	*LDB2*
8	11734910	11734910	-	11734910	3	3	---	---	intergenic	---

8	11763587	11763587	+	11763587	3	3	---	---	intergenic	---
8	12290112	12290112	+	12290112	3	3	---	---	intergenic	---
8	12332253	12332265	-	12332258	96	250	ENSSSCG00000029508	-	3'UTR	*QDPR*
8	12381097	12381097	-	12381097	3	3	ENSSSCG00000023604	-	ORF	---
8	12417227	12417227	+	12417227	3	3	ENSSSCG00000028448	+	3'UTR	---
8	12417485	12417491	+	12417486	130	409	ENSSSCG00000028448	+	3'UTR	---
8	12430967	12430974	+	12430972	14	53	ENSSSCG00000029989	+	3'UTR	*MED28*
8	12431633	12431633	+	12431633	5	5	ENSSSCG00000029989	+	3'UTR	*MED28*
8	12460934	12460934	-	12460934	4	4	ENSSSCG00000026232	-	intron	---
8	12472135	12472137	-	12472136	44	54	ENSSSCG00000026232	-	intron	---
8	12553964	12553965	-	12553964	3	6	XLOC_085393	-	predicted	---
8	12578811	12578811	+	12578811	6	6	ENSSSCG00000008747	+	intron	*NCAPG*
8	12630854	12630854	-	12630854	3	3	XLOC_085394	-	predicted	---
8	12936723	12936726	+	12936724	39	54	---	---	intergenic	---
8	12952210	12952210	-	12952210	29	29	---	---	intergenic	---
8	13133021	13133021	+	13133021	4	4	XLOC_084556	+	predicted	---
8	13152655	13152658	+	13152655	7	10	---	---	intergenic	---
8	13421828	13421828	+	13421828	3	3	---	---	intergenic	---
8	14702660	14702660	+	14702660	3	3	---	---	intergenic	---
8	14702709	14702709	-	14702709	3	3	---	---	intergenic	---
8	14702791	14702794	+	14702794	5	11	---	---	intergenic	---
8	14910646	14910646	-	14910646	3	3	---	---	intergenic	---
8	15354620	15354620	-	15354620	3	3	XLOC_085397	-	predicted	---
8	15450179	15450182	-	15450182	4	7	---	---	intergenic	---
8	15469988	15469998	+	15469988	5	13	ENSSSCG00000008750	+	3'UTR	*PACRGL*
8	15612706	15612706	+	15612706	13	13	---	---	intergenic	---

8	16892963	16892963	+	16892963	7	7	---	---	intergenic	---
8	16905598	16905601	-	16905598	3	9	ENSSSCG00000008753	-	3'UTR	*ADGRA3*
8	17191908	17191914	+	17191908	18	40	---	---	intergenic	---
8	17342132	17342132	+	17342132	3	3	---	---	intergenic	---
8	17399993	17399993	+	17399993	4	4	XLOC_084567	+	predicted	---
8	17909905	17909906	+	17909906	32	40	---	---	intergenic	---
8	17917432	17917432	+	17917432	8	8	---	---	intergenic	---
8	17932270	17932270	-	17932270	11	11	---	---	intergenic	---
8	18397936	18397936	+	18397936	3	3	---	---	intergenic	---
8	18437800	18437800	-	18437800	3	3	---	---	intergenic	---
8	18740333	18740337	-	18740336	4	10	---	---	intergenic	---
8	18834364	18834364	-	18834364	3	3	---	---	intergenic	---
8	18853663	18853664	-	18853664	21	30	ENSSSCG00000008756	-	3'UTR	*DHX15*
8	18981183	18981184	-	18981183	7	11	---	---	intergenic	---
8	19159310	19159310	-	19159310	3	3	---	---	intergenic	---
8	19166064	19166064	-	19166064	4	4	---	---	intergenic	---
8	19270015	19270022	+	19270022	14	42	ENSSSCG00000023001	+	intron	*CCDC149*
8	19300726	19300727	+	19300726	3	6	ENSSSCG00000023001	+	ORF	*CCDC149*
8	19300931	19300939	+	19300932	96	168	ENSSSCG00000023001	+	3'UTR	*CCDC149*
8	19303259	19303266	+	19303259	5	8	ENSSSCG00000023001	+	3'UTR	*CCDC149*
8	19456168	19456168	-	19456168	3	3	---	---	intergenic	---
8	19626498	19626498	-	19626498	5	5	ENSSSCG00000027340	-	3'UTR	---
8	19626808	19626808	-	19626808	3	3	ENSSSCG00000027340	-	3'UTR	---
8	19629026	19629030	-	19629026	5	9	ENSSSCG00000027340	-	3'UTR	---
8	19629126	19629126	-	19629126	4	4	ENSSSCG00000027340	-	3'UTR	---
8	19635242	19635242	-	19635242	6	6	ENSSSCG00000027340	-	intron	---

8	19709797	19709797	-	19709797	3	3	---	---	intergenic	---
8	19766356	19766356	+	19766356	3	3	---	---	intergenic	---
8	19788874	19788874	-	19788874	4	4	XLOC_085419	-	predicted	---
8	19789052	19789052	-	19789052	3	3	XLOC_085419	-	predicted	---
8	19800713	19800718	+	19800718	24	44	XLOC_084580	+	predicted	---
8	19817394	19817399	+	19817398	10	27	XLOC_084576	+	predicted	---
8	19824680	19824680	+	19824680	3	3	---	---	intergenic	---
8	19915562	19915562	-	19915562	3	3	---	---	intergenic	---
8	19915590	19915591	+	19915591	4	7	---	---	intergenic	---
8	19988214	19988214	+	19988214	3	3	---	---	intergenic	---
8	20089897	20089897	-	20089897	3	3	---	---	intergenic	---
8	20100353	20100353	+	20100353	3	3	XLOC_084582	+	predicted	---
8	20105889	20105892	+	20105890	14	21	XLOC_084582	+	predicted	---
8	20503134	20503136	+	20503134	3	6	ENSSSCG00000022155	+	intron	*RBPJ*
8	20540524	20540524	+	20540524	5	5	ENSSSCG00000022155	+	3'UTR	*RBPJ*
8	20546645	20546645	+	20546645	4	4	---	---	intergenic	---
8	20757064	20757064	+	20757064	3	3	---	---	intergenic	---
8	21104449	21104449	+	21104449	3	3	ENSSSCG00000008762	+	3'UTR	*STIM2*
8	21281771	21281771	+	21281771	4	4	---	---	intergenic	---
8	21718704	21718704	+	21718704	3	3	---	---	intergenic	---
8	22734377	22734382	+	22734378	8	30	---	---	intergenic	---
8	23841505	23841505	+	23841505	3	3	---	---	intergenic	---
8	24256155	24256155	-	24256155	16	16	---	---	intergenic	---
8	25462602	25462604	-	25462603	11	25	---	---	intergenic	---
8	26018690	26018690	-	26018690	3	3	---	---	intergenic	---
8	26257934	26257934	+	26257934	4	4	---	---	intergenic	---

8	26708300	26708300	+	26708300	7	7	---	---	intergenic	---
8	27111510	27111510	+	27111510	6	6	---	---	intergenic	---
8	28015989	28015989	+	28015989	3	3	ENSSSCG00000008767	+	ORF	---
8	28026921	28026922	+	28026921	33	38	---	---	intergenic	---
8	29324081	29324081	-	29324081	149	149	ENSSSCG00000008768	-	intron	*ARAP2*
8	29354821	29354821	+	29354821	3	3	---	---	intergenic	---
8	29367757	29367757	+	29367757	4	4	ENSSSCG00000028471	+	intron	*DTHD1*
8	29584216	29584218	-	29584217	220	260	---	---	intergenic	---
8	29674536	29674538	+	29674538	20	30	ENSSSCG00000008769	+	3'UTR	*CD68*
							ENSSSCG00000008769	+	ORF	---
8	29674594	29674594	+	29674594	9	9	ENSSSCG00000008769	+	3'UTR	*CD68*
							ENSSSCG00000008769	+	ORF	---
8	30013688	30013688	+	30013688	7	7	---	---	intergenic	---
8	30334173	30334173	-	30334173	6	6	---	---	intergenic	---
8	30371342	30371342	-	30371342	11	11	---	---	intergenic	---
8	30516351	30516354	+	30516354	10	14	XLOC_084600	+	predicted	---
8	30531414	30531418	-	30531414	5	10	XLOC_085450	-	predicted	---
8	30719326	30719326	+	30719326	3	3	ENSSSCG00000008773	+	intron	*PGM2*
8	30753491	30753491	+	30753491	7	7	ENSSSCG00000008773	+	3'UTR	*PGM2*
8	30904443	30904443	+	30904443	3	3	XLOC_084605	+	predicted	---
8	31052624	31052629	+	31052628	17	60	ENSSSCG00000008774	+	3'UTR	*TBC1D1*
8	31053963	31053966	+	31053966	7	10	ENSSSCG00000008774	+	3'UTR	*TBC1D1*
8	31061135	31061135	-	31061135	8	8	XLOC_085455	-	predicted	---
8	31292158	31292159	-	31292158	6	10	---	---	intergenic	---
8	31397138	31397138	+	31397138	3	3	---	---	intergenic	---
8	31536056	31536056	+	31536056	4	4	ENSSSCG00000022270	+	3'UTR	*KLF3*

8	31539472	31539472	+	31539472	15	15	XLOC_084609	+	predicted	---
8	31627794	31627794	-	31627794	3	3	ENSSSCG00000026583	-	3'UTR	*TLR1*
8	31750565	31750565	+	31750565	4	4	ENSSSCG00000008785	+	3'UTR	*FAM114A1*
8	31754424	31754426	+	31754426	10	19	ENSSSCG00000008785	+	3'UTR	*FAM114A1*
8	31848631	31848631	+	31848631	6	6	---	---	intergenic	---
8	31886514	31886514	+	31886514	3	3	ENSSSCG00000008787	+	intron	---
8	31970086	31970086	+	31970086	3	3	ENSSSCG00000024991	+	3'UTR	---
8	32168578	32168583	+	32168578	7	21	ENSSSCG00000008789	+	3'UTR	*WDR19*
8	32169923	32169923	-	32169923	3	3	XLOC_085463	-	predicted	---
8	32169965	32169965	-	32169965	6	6	XLOC_085463	-	predicted	---
8	32187709	32187709	-	32187709	3	3	ENSSSCG00000008788	-	ORF	---
8	32187881	32187881	-	32187881	7	7	ENSSSCG00000008788	-	ORF	---
8	32190313	32190313	-	32190313	15	15	ENSSSCG00000008788	-	intron	---
8	32243891	32243893	-	32243893	16	19	ENSSSCG00000008788	-	intron	---
8	32308342	32308342	-	32308342	7	7	ENSSSCG00000025192	-	3'UTR	*RPL9*
8	32309013	32309025	-	32309018	144	232	ENSSSCG00000025192	-	3'UTR	*RPL9*
8	32331937	32331962	+	32331937	3	6	ENSSSCG00000029385	+	3'UTR	*LIAS*
8	32332036	32332043	+	32332036	11	15	ENSSSCG00000029385	+	3'UTR	*LIAS*
8	32340312	32340321	-	32340319	37	58	ENSSSCG00000030150	-	3'UTR	*UGDH*
8	32380442	32380451	-	32380451	46	115	ENSSSCG00000021793	-	3'UTR	*SMIM14*
8	32381101	32381101	-	32381101	5	5	ENSSSCG00000021793	-	3'UTR	*SMIM14*
8	32382136	32382148	-	32382138	18	78	ENSSSCG00000021793	-	3'UTR	*SMIM14*
8	32382808	32382808	-	32382808	4	4	ENSSSCG00000021793	-	3'UTR	*SMIM14*
8	32383318	32383355	-	32383342	22	63	ENSSSCG00000021793	-	3'UTR	*SMIM14*
8	32383521	32383521	-	32383521	5	5	ENSSSCG00000021793	-	3'UTR	*SMIM14*
8	32466302	32466302	+	32466302	3	3	---	---	intergenic	---

8	32492653	32492653	+	32492653	4	4	---	---	intergenic	---
8	32552228	32552229	+	32552229	5	8	XLOC_084622	+	predicted	---
8	32553713	32553716	+	32553716	11	15	XLOC_084622	+	predicted	---
8	32580803	32580803	-	32580803	3	3	ENSSSCG00000025039	-	3'UTR	*UBE2K*
8	32580963	32580963	-	32580963	4	4	ENSSSCG00000025039	-	3'UTR	*UBE2K*
8	32582322	32582323	-	32582322	5	9	ENSSSCG00000025039	-	3'UTR	*UBE2K*
8	32605485	32605485	-	32605485	3	3	ENSSSCG00000025039	-	intron	*UBE2K*
8	32632457	32632458	-	32632457	4	8	ENSSSCG00000025039	-	intron	*UBE2K*
8	32653851	32653851	-	32653851	11	11	ENSSSCG00000025039	-	intron	*UBE2K*
8	32955397	32955398	+	32955398	4	7	ENSSSCG00000008792	+	3'UTR	*N4BP2*
8	32984927	32984927	-	32984927	4	4	---	---	intergenic	---
8	32984955	32984955	+	32984955	3	3	---	---	intergenic	---
8	33037042	33037042	+	33037042	3	3	XLOC_084626	+	predicted	---
8	33039744	33039744	+	33039744	12	12	XLOC_084626	+	predicted	---
8	33123280	33123280	+	33123280	5	5	ENSSSCG00000024428	+	3'UTR	*CHRNA9*
8	33174222	33174241	-	33174241	14	24	XLOC_085480	-	predicted	---
8	33426848	33426848	+	33426848	6	6	ENSSSCG00000022168	+	3'UTR	*APBB2*
8	33428451	33428452	-	33428452	6	9	XLOC_085483	-	predicted	---
8	33429638	33429644	-	33429644	8	15	XLOC_085483	-	predicted	---
8	33472486	33472492	-	33472488	25	51	ENSSSCG00000024496	-	ORF	*NSUN7*
8	33535905	33535905	-	33535905	3	3	ENSSSCG00000024496	-	ORF	*NSUN7*
8	33604507	33604507	+	33604507	3	3	---	---	intergenic	---
8	33982435	33982439	+	33982435	17	24	XLOC_084635	+	predicted	---
8	34034130	34034130	-	34034130	4	4	---	---	intergenic	---
8	34034204	34034204	+	34034204	14	14	---	---	intergenic	---
8	34034289	34034298	+	34034289	4	7	---	---	intergenic	---

8	34035064	34035078	-	34035078	13	18	---	---	intergenic	---
8	34035135	34035135	+	34035135	10	10	---	---	intergenic	---
8	34035560	34035568	+	34035566	230	386	---	---	intergenic	---
8	34108640	34108640	+	34108640	4	4	ENSSSCG00000008799	+	intron	---
8	34281388	34281388	+	34281388	4	4	ENSSSCG00000008799	+	intron	---
8	34338330	34338338	+	34338330	5	14	ENSSSCG00000008799	+	intron	---
8	34338400	34338400	+	34338400	3	3	ENSSSCG00000008799	+	intron	---
8	34359485	34359485	+	34359485	3	3	ENSSSCG00000008799	+	intron	---
8	34408104	34408104	+	34408104	5	5	XLOC_084636	+	predicted	---
8	34686978	34686981	+	34686981	8	11	ENSSSCG00000008800	+	3'UTR	*TMEM33*
8	34691040	34691047	+	34691047	17	34	XLOC_084640	+	predicted	---
8	34718094	34718094	+	34718094	4	4	ENSSSCG00000008801	+	intron	*SLC30A9*
8	34768039	34768047	+	34768047	8	15	XLOC_084641	+	predicted	---
8	34937006	34937006	-	34937006	12	12	---	---	intergenic	---
8	34974190	34974191	-	34974190	3	6	---	---	intergenic	---
8	35030108	35030108	-	35030108	3	3	---	---	intergenic	---
8	35030126	35030127	+	35030126	10	14	---	---	intergenic	---
8	35086316	35086316	+	35086316	3	3	---	---	intergenic	---
8	35414773	35414776	-	35414773	6	14	XLOC_085495	-	predicted	---
8	35531690	35531690	+	35531690	3	3	---	---	intergenic	---
8	35605603	35605603	-	35605603	4	4	---	---	intergenic	---
8	35774884	35774884	+	35774884	4	4	---	---	intergenic	---
8	35861152	35861153	-	35861152	6	9	---	---	intergenic	---
8	36093685	36093685	-	36093685	4	4	---	---	intergenic	---
8	36259191	36259194	-	36259191	4	8	---	---	intergenic	---
8	36259221	36259221	+	36259221	3	3	---	---	intergenic	---

8	36263733	36263733	+	36263733	3	3	---	---	intergenic	---
8	36340779	36340780	+	36340779	8	11	---	---	intergenic	---
8	36900368	36900376	-	36900368	5	18	ENSSSCG00000008808	-	3'UTR	*GNPDA2*
8	36906431	36906434	-	36906431	3	6	ENSSSCG00000008808	-	intron	*GNPDA2*
8	36955239	36955240	+	36955240	7	10	---	---	intergenic	---
8	36987920	36987920	-	36987920	4	4	---	---	intergenic	---
8	37070259	37070259	-	37070259	20	20	---	---	intergenic	---
8	37221526	37221528	-	37221527	31	47	---	---	intergenic	---
8	37230494	37230494	+	37230494	3	3	---	---	intergenic	---
8	37237943	37237943	+	37237943	4	4	---	---	intergenic	---
8	37660956	37660956	-	37660956	3	3	---	---	intergenic	---
8	37660986	37660988	+	37660986	7	13	---	---	intergenic	---
8	37684715	37684715	-	37684715	3	3	---	---	intergenic	---
8	37704197	37704197	+	37704197	3	3	---	---	intergenic	---
8	37965643	37965643	-	37965643	4	4	---	---	intergenic	---
8	38182835	38182835	+	38182835	3	3	---	---	intergenic	---
8	38233155	38233155	-	38233155	3	3	ENSSSCG00000028922	-	ORF	*GABRA2*
8	38650490	38650490	-	38650490	7	7	---	---	intergenic	---
8	38661986	38661986	-	38661986	4	4	---	---	intergenic	---
8	38828944	38828944	-	38828944	13	13	---	---	intergenic	---
8	38964155	38964155	+	38964155	13	13	---	---	intergenic	---
8	39292373	39292373	-	39292373	7	7	ENSSSCG00000027912	-	3'UTR	*COMMD8*
8	39789194	39789194	-	39789194	3	3	XLOC_085506	-	predicted	---
8	39804529	39804529	-	39804529	4	4	ENSSSCG00000008816	-	intron	*NFXL1*
8	40014675	40014675	-	40014675	3	3	---	---	intergenic	---
8	40028645	40028645	-	40028645	3	3	ENSSSCG00000026803	-	3'UTR	---

8	40094010	40094010	-	40094010	3	3	ENSSSCG00000026803	-	intron	---
8	40365750	40365755	+	40365750	16	26	XLOC_084658	+	predicted	---
8	40425074	40425074	+	40425074	3	3	XLOC_084660	+	predicted	---
8	40429325	40429327	-	40429326	6	15	ENSSSCG00000008826	-	3'UTR	---
8	40520711	40520711	-	40520711	9	9	ENSSSCG00000008826	-	intron	---
8	40627299	40627299	-	40627299	3	3	ENSSSCG00000008826	-	intron	---
							ENSSSCG00000008827	-	intron	---
8	40763660	40763660	-	40763660	3	3	---	---	intergenic	---
8	40851440	40851440	+	40851440	7	7	---	---	intergenic	---
8	40872558	40872558	+	40872558	3	3	ENSSSCG00000008828	+	intron	*OCIAD1*
8	40875889	40875925	+	40875925	6	13	ENSSSCG00000008828	+	3'UTR	*OCIAD1*
8	40876075	40876076	+	40876075	3	6	ENSSSCG00000008828	+	3'UTR	*OCIAD1*
8	40876222	40876229	+	40876223	36	74	ENSSSCG00000008828	+	3'UTR	*OCIAD1*
8	40876439	40876447	+	40876446	23	35	ENSSSCG00000008828	+	3'UTR	*OCIAD1*
8	40879997	40880011	-	40879997	6	9	ENSSSCG00000008829	-	3'UTR	*OCIAD2*
8	40880436	40880436	-	40880436	3	3	ENSSSCG00000008829	-	3'UTR	*OCIAD2*
8	40880539	40880539	-	40880539	3	3	ENSSSCG00000008829	-	3'UTR	*OCIAD2*
8	41120604	41120604	+	41120604	5	5	XLOC_084664	+	predicted	---
8	41123338	41123339	-	41123338	3	6	---	---	intergenic	---
8	41216058	41216058	+	41216058	6	6	ENSSSCG00000008831	+	ORF	*DCUN1D4*
8	41233048	41233053	+	41233052	61	97	ENSSSCG00000008831	+	3'UTR	*DCUN1D4*
8	41233318	41233318	+	41233318	4	4	ENSSSCG00000008831	+	3'UTR	*DCUN1D4*
8	41234741	41234741	+	41234741	4	4	ENSSSCG00000008831	+	3'UTR	*DCUN1D4*
8	41236198	41236202	+	41236199	13	37	ENSSSCG00000008831	+	3'UTR	*DCUN1D4*
8	41239676	41239676	-	41239676	45	45	---	---	intergenic	---
8	41285898	41285898	-	41285898	5	5	ENSSSCG00000008832	-	intron	*LRRC66*

8	41302937	41302945	-	41302945	5	11	XLOC_085523	-	predicted	---
8	41337821	41337828	-	41337828	22	58	---	---	intergenic	---
8	41338217	41338227	-	41338224	46	75	---	---	intergenic	---
8	41338409	41338409	-	41338409	3	3	---	---	intergenic	---
8	41617634	41617634	-	41617634	4	4	---	---	intergenic	---
8	41639409	41639409	+	41639409	6	6	---	---	intergenic	---
8	41645646	41645648	-	41645646	3	6	ENSSSCG00000028733	-	3'UTR	*USP46*
8	41730671	41730671	-	41730671	3	3	---	---	intergenic	---
8	41833741	41833742	+	41833742	4	7	ENSSSCG00000008835	+	3'UTR	*RASL11B*
8	41834397	41834404	+	41834397	7	18	ENSSSCG00000008835	+	3'UTR	*RASL11B*
8	41841768	41841772	-	41841768	6	11	ENSSSCG00000030621	-	3'UTR	---
8	41841817	41841817	-	41841817	3	3	ENSSSCG00000030621	-	3'UTR	---
8	42043224	42043224	-	42043224	3	3	---	---	intergenic	---
8	42075391	42075391	+	42075391	3	3	---	---	intergenic	---
8	42169349	42169349	-	42169349	3	3	ENSSSCG00000008836	-	intron	*SCFD2*
8	42289133	42289138	+	42289138	7	15	---	---	intergenic	---
8	42835431	42835431	-	42835431	3	3	ENSSSCG00000008839	-	3'UTR	*CHIC2*
8	42951285	42951285	-	42951285	3	3	---	---	intergenic	---
8	42998544	42998547	+	42998544	22	29	---	---	intergenic	---
8	43024212	43024212	+	43024212	3	3	ENSSSCG00000008841	+	intron	*PDGFRA*
8	43065431	43065431	-	43065431	4	4	---	---	intergenic	---
8	43067785	43067785	-	43067785	6	6	XLOC_085531	-	predicted	---
8	43657366	43657366	+	43657366	3	3	---	---	intergenic	---
8	43661049	43661049	+	43661049	4	4	---	---	intergenic	---
8	43746555	43746556	+	43746556	9	12	---	---	intergenic	---
8	43774318	43774318	+	43774318	3	3	---	---	intergenic	---

8	43919327	43919327	-	43919327	4	4	XLOC_085539	-	predicted	---
8	44057961	44057961	-	44057961	5	5	XLOC_085541	-	predicted	---
8	44065321	44065321	-	44065321	3	3	XLOC_085542	-	predicted	---
8	44071898	44071905	+	44071898	6	9	---	---	intergenic	---
8	44139285	44139288	-	44139285	3	6	ENSSSCG00000008845	-	3'UTR	*SRD5A3*
8	44221643	44221643	-	44221643	5	5	---	---	intergenic	---
8	44403244	44403246	-	44403244	4	7	---	---	intergenic	---
8	45120320	45120320	-	45120320	4	4	---	---	intergenic	---
8	45491591	45491591	-	45491591	3	3	ENSSSCG00000008854	-	intron	*CPE*
8	45557615	45557658	-	45557634	145	392	ENSSSCG00000008857	-	3'UTR	*SC4MOL*
							ENSSSCG00000008854	-	intron	*CPE*
8	45622342	45622342	-	45622342	3	3	XLOC_085552	-	predicted	---
8	45681721	45681721	-	45681721	3	3	ENSSSCG00000008858	-	intron	*KLHL2*
8	45701604	45701604	-	45701604	6	6	ENSSSCG00000008858	-	intron	*KLHL2*
8	45919224	45919225	+	45919224	19	32	---	---	intergenic	---
8	45967098	45967098	-	45967098	3	3	---	---	intergenic	---
8	46493710	46493710	+	46493710	5	5	ENSSSCG00000008866	+	intron	*GUCY1A3*
8	46685616	46685616	-	46685616	3	3	---	---	intergenic	---
8	46704653	46704670	+	46704670	89	137	ENSSSCG00000008868	+	intron	*TDO2*
8	46705429	46705430	+	46705429	5	9	ENSSSCG00000008868	+	intron	*TDO2*
8	46705556	46705556	+	46705556	7	7	ENSSSCG00000008868	+	intron	*TDO2*
8	46705895	46705895	+	46705895	6	6	ENSSSCG00000008868	+	ORF	*TDO2*
8	46708719	46708720	+	46708719	9	12	ENSSSCG00000008868	+	intron	*TDO2*
8	46710189	46710189	+	46710189	4	4	ENSSSCG00000008868	+	intron	*TDO2*
8	46711223	46711223	+	46711223	4	4	ENSSSCG00000008868	+	ORF	*TDO2*
8	46712702	46712703	+	46712703	5	8	ENSSSCG00000008868	+	intron	*TDO2*

8	46721032	46721042	+	46721032	6	10	ENSSSCG00000008868	+	3'UTR	*TDO2*
8	46721285	46721285	+	46721285	6	6	ENSSSCG00000008868	+	3'UTR	*TDO2*
8	46721365	46721466	+	46721455	115	485	ENSSSCG00000008868	+	3'UTR	*TDO2*
8	47156507	47156507	+	47156507	3	3	---	---	intergenic	---
8	47676708	47676708	-	47676708	6	6	ENSSSCG00000024960	-	3'UTR	*PDGFC*
8	47915918	47915918	-	47915918	3	3	---	---	intergenic	---
8	47997308	47997308	-	47997308	6	6	---	---	intergenic	---
8	48008780	48008780	-	48008780	5	5	---	---	intergenic	---
8	48008809	48008810	+	48008809	6	9	---	---	intergenic	---
8	48070976	48070976	+	48070976	5	5	ENSSSCG00000021506	+	3'UTR	*GLRB*
8	48274881	48274881	+	48274881	4	4	ENSSSCG00000024685	+	intron	*GRIA2*
8	49352456	49352456	-	49352456	5	5	XLOC_085570	-	predicted	---
8	49353404	49353415	-	49353405	8	14	---	---	intergenic	---
8	49353458	49353458	-	49353458	3	3	---	---	intergenic	---
8	49425901	49425901	+	49425901	3	3	ENSSSCG00000008874	+	intron	*TMEM144*
8	49429813	49429813	+	49429813	4	4	ENSSSCG00000008874	+	3'UTR	*TMEM144*
8	50009520	50009526	-	50009520	6	9	ENSSSCG00000028492	-	3'UTR	*C4orf46*
8	50011286	50011320	-	50011286	5	9	ENSSSCG00000028492	-	3'UTR	*C4orf46*
8	50060547	50060556	+	50060552	34	88	ENSSSCG00000008877	+	3'UTR	*ETFDH*
8	50060938	50060945	-	50060938	19	31	ENSSSCG00000008878	-	3'UTR	*PPID*
8	50061055	50061068	-	50061063	20	30	ENSSSCG00000008878	-	3'UTR	*PPID*
8	50061171	50061171	-	50061171	4	4	ENSSSCG00000008878	-	3'UTR	*PPID*
8	50067040	50067040	-	50067040	3	3	ENSSSCG00000008878	-	ORF	*PPID*
8	50121151	50121151	+	50121151	8	8	---	---	intergenic	---
8	50224268	50224268	-	50224268	3	3	XLOC_085574	-	predicted	---
8	50228423	50228453	-	50228446	11	21	---	---	intergenic	---

8	50296295	50296298	-	50296298	43	53	---	---	intergenic	---
8	50472882	50472882	+	50472882	3	3	ENSSSCG00000008881	+	intron	*RAPGEF2*
8	50684099	50684099	+	50684099	5	5	ENSSSCG00000008881	+	3'UTR	*RAPGEF2*
8	51465938	51465938	-	51465938	3	3	---	---	intergenic	---
8	51550615	51550615	+	51550615	3	3	---	---	intergenic	---
8	52224848	52224849	-	52224849	18	23	---	---	intergenic	---
8	52857541	52857541	+	52857541	3	3	ENSSSCG00000027183	+	intron	---
8	53078211	53078211	-	53078211	6	6	---	---	intergenic	---
8	53661284	53661284	+	53661284	3	3	---	---	intergenic	---
8	53839118	53839118	-	53839118	7	7	ENSSSCG00000026906	-	intron	---
8	53936740	53936740	-	53936740	4	4	---	---	intergenic	---
8	54081875	54081875	-	54081875	54	54	---	---	intergenic	---
8	54433185	54433185	-	54433185	4	4	---	---	intergenic	---
8	54446701	54446701	+	54446701	6	6	---	---	intergenic	---
8	54648743	54648743	+	54648743	3	3	XLOC_084739	+	predicted	---
8	54683236	54683236	+	54683236	5	5	---	---	intergenic	---
8	55346201	55346201	+	55346201	12	12	---	---	intergenic	---
8	55453379	55453379	-	55453379	3	3	XLOC_085584	-	predicted	---
8	55498953	55498959	+	55498959	11	15	XLOC_084743	+	predicted	---
8	55503109	55503109	-	55503109	6	6	XLOC_085585	-	predicted	---
8	56032527	56032527	-	56032527	4	4	---	---	intergenic	---
8	57357720	57357720	+	57357720	4	4	XLOC_084750	+	predicted	---
8	57367377	57367377	+	57367377	4	4	XLOC_084750	+	predicted	---
8	57394424	57394435	-	57394432	25	82	XLOC_085594	-	predicted	---
8	57440581	57440581	-	57440581	7	7	ENSSSCG00000008893	-	3'UTR	*REST*
8	57463467	57463468	-	57463467	5	10	ENSSSCG00000008893	-	intron	*REST*

8	57518480	57518491	+	57518483	335	769	XLOC_084752	+	predicted	---
8	57564093	57564093	+	57564093	3	3	---	---	intergenic	---
8	57600102	57600104	-	57600102	55	77	XLOC_085596	-	predicted	---
8	57634380	57634388	+	57634381	76	92	ENSSSCG00000008898	+	3'UTR	*HOPX*
8	57718705	57718705	-	57718705	15	15	XLOC_085597	-	predicted	---
8	57774016	57774077	-	57774067	175	717	ENSSSCG00000008899	-	3'UTR	*ARL9*
8	57774820	57774820	-	57774820	3	3	ENSSSCG00000008899	-	intron	*ARL9*
8	57788062	57788076	-	57788076	42	122	---	---	intergenic	---
8	57789534	57789534	-	57789534	4	4	---	---	intergenic	---
8	57827125	57827129	-	57827129	20	37	XLOC_085600	-	predicted	---
8	57829568	57829573	-	57829568	3	6	XLOC_085600	-	predicted	---
8	57829632	57829632	-	57829632	9	9	XLOC_085600	-	predicted	---
8	57845170	57845190	-	57845190	5	8	ENSSSCG00000008901	-	ORF	*PAICS*
8	57888273	57888273	+	57888273	4	4	---	---	intergenic	---
8	57911864	57911864	-	57911864	3	3	ENSSSCG00000027403	-	intron	*AASDH*
8	57986981	57986981	+	57986981	3	3	---	---	intergenic	---
8	58014584	58014584	-	58014584	3	3	XLOC_085604	-	predicted	---
8	58158216	58158216	+	58158216	3	3	---	---	intergenic	---
8	58258704	58258704	-	58258704	4	4	ENSSSCG00000022283	-	intron	*CEP135*
							ENSSSCG00000022283	-	ORF	*CEP135*
8	58484992	58484993	-	58484993	9	13	---	---	intergenic	---
8	58591478	58591478	-	58591478	3	3	XLOC_085612	-	predicted	---
8	58592090	58592090	-	58592090	5	5	XLOC_085612	-	predicted	---
8	58601453	58601453	+	58601453	4	4	XLOC_084765	+	predicted	---
8	58603929	58603929	+	58603929	6	6	---	---	intergenic	---
8	58607888	58607888	+	58607888	4	4	ENSSSCG00000008908	+	ORF	*PDCL2*

8	58609623	58609623	+	58609623	3	3	ENSSSCG00000008908	+	intron	*PDCL2*
8	58621974	58621974	+	58621974	4	4	ENSSSCG00000008908	+	ORF	*PDCL2*
8	58634849	58634866	+	58634857	58	248	ENSSSCG00000008908	+	3'UTR	*PDCL2*
8	58769732	58769732	+	58769732	3	3	XLOC_084768	+	predicted	---
8	58773133	58773134	-	58773133	4	7	XLOC_085615	-	predicted	---
8	59035311	59035311	+	59035311	4	4	ENSSSCG00000022955	+	ORF	*POLR2B*
8	59063779	59063782	-	59063779	26	32	XLOC_085618	-	predicted	---
8	59063890	59063890	-	59063890	3	3	XLOC_085618	-	predicted	---
8	59064678	59064683	-	59064681	181	279	ENSSSCG00000008913	-	3'UTR	*IGFBP7*
8	59064746	59064746	-	59064746	4	4	ENSSSCG00000008913	-	3'UTR	*IGFBP7*
8	59064749	59064756	+	59064755	22	56	ENSSSCG00000022955	+	3'UTR	*POLR2B*
8	59065953	59065956	-	59065953	28	36	ENSSSCG00000008913	-	ORF	*IGFBP7*
8	59379980	59379980	-	59379980	3	3	---	---	intergenic	---
8	59994487	59994487	-	59994487	3	3	---	---	intergenic	---
8	60454372	60454373	+	60454372	35	38	---	---	intergenic	---
8	61042723	61042723	+	61042723	4	4	---	---	intergenic	---
8	61093900	61093900	-	61093900	8	8	---	---	intergenic	---
8	61276541	61276546	+	61276541	3	6	---	---	intergenic	---
8	61950644	61950647	+	61950644	3	6	---	---	intergenic	---
8	63345622	63345622	+	63345622	3	3	---	---	intergenic	---
8	63381378	63381378	-	63381378	3	3	XLOC_085625	-	predicted	---
8	64765154	64765155	-	64765154	20	23	---	---	intergenic	---
8	64999264	64999264	+	64999264	9	9	---	---	intergenic	---
8	65388488	65388489	-	65388489	35	39	---	---	intergenic	---
8	66646417	66646417	-	66646417	4	4	---	---	intergenic	---
8	66784773	66784773	+	66784773	3	3	---	---	intergenic	---

8	68635243	68635243	-	68635243	5	5	---	---	intergenic	---
8	68769270	68769270	+	68769270	7	7	---	---	intergenic	---
8	68780353	68780353	+	68780353	4	4	---	---	intergenic	---
8	68811545	68811546	-	68811545	5	10	---	---	intergenic	---
8	68850171	68850171	-	68850171	5	5	---	---	intergenic	---
8	68926970	68926970	+	68926970	3	3	---	---	intergenic	---
8	69069236	69069236	+	69069236	4	4	---	---	intergenic	---
8	69211168	69211168	-	69211168	5	5	---	---	intergenic	---
8	69368240	69368240	-	69368240	3	3	---	---	intergenic	---
8	69659189	69659190	-	69659189	8	14	ENSSSCG00000008921	-	3'UTR	---
8	69711265	69711265	-	69711265	4	4	ENSSSCG00000008921	-	ORF	---
8	69751550	69751550	+	69751550	3	3	---	---	intergenic	---
8	69776877	69776877	+	69776877	17	17	ENSSSCG00000008923	+	intron	*STAP1*
8	69796590	69796590	+	69796590	8	8	---	---	intergenic	---
8	69796922	69796922	-	69796922	3	3	XLOC_085639	-	predicted	---
8	70302948	70302948	+	70302948	5	5	---	---	intergenic	---
8	70329049	70329054	-	70329054	12	26	XLOC_085650	-	predicted	---
8	70337309	70337309	-	70337309	4	4	ENSSSCG00000024213	-	3'UTR	---
8	70337352	70337366	-	70337365	9	30	ENSSSCG00000024213	-	3'UTR	---
8	70337488	70337499	-	70337497	17	49	ENSSSCG00000024213	-	3'UTR	---
8	70337585	70337592	-	70337592	17	35	ENSSSCG00000024213	-	3'UTR	---
8	70337682	70337689	-	70337685	18	54	ENSSSCG00000024213	-	3'UTR	---
8	70338042	70338072	-	70338042	3	6	ENSSSCG00000024213	-	3'UTR	---
8	70338177	70338177	-	70338177	3	3	ENSSSCG00000024213	-	3'UTR	---
8	70338759	70338759	-	70338759	4	4	ENSSSCG00000024213	-	3'UTR	---
8	70338857	70338857	-	70338857	8	8	ENSSSCG00000024213	-	3'UTR	---

8	70338989	70339008	-	70338989	41	55	ENSSSCG00000024213	-	3'UTR	---
8	70339150	70339150	-	70339150	5	5	ENSSSCG00000024213	-	ORF	---
8	70357422	70357456	-	70357450	82	105	ENSSSCG00000024213	-	ORF	---
8	70463558	70463558	+	70463558	3	3	---	---	intergenic	---
8	70570422	70570435	+	70570427	58	146	XLOC_084800	+	predicted	---
8	70571520	70571529	+	70571520	8	34	XLOC_084800	+	predicted	---
8	70573256	70573290	+	70573289	7	19	XLOC_084800	+	predicted	---
8	70573655	70573661	+	70573660	25	36	XLOC_084800	+	predicted	---
8	70585598	70585598	+	70585598	3	3	ENSSSCG00000008935	+	ORF	---
8	70586493	70586493	+	70586493	3	3	ENSSSCG00000008935	+	ORF	---
8	70588817	70588817	+	70588817	9	9	ENSSSCG00000008935	+	intron	---
8	70702814	70702859	+	70702850	7	37	ENSSSCG00000030074	+	3'UTR	---
8	70755198	70755198	-	70755198	5	5	ENSSSCG00000025786	-	3'UTR	---
8	70910571	70910572	-	70910572	10	13	XLOC_085657	-	predicted	---
8	70982399	70982399	-	70982399	4	4	XLOC_085657	-	predicted	---
8	70984983	70985000	-	70984984	50	79	XLOC_085657	-	predicted	---
8	70986374	70986374	-	70986374	6	6	ENSSSCG00000025226	-	ORF	*UGT2A3*
8	71105223	71105223	+	71105223	3	3	XLOC_084804	+	predicted	---
8	71111393	71111398	+	71111394	28	46	ENSSSCG00000026944	+	3'UTR	---
8	71111893	71111893	+	71111893	4	4	ENSSSCG00000026944	+	3'UTR	---
8	71301761	71301761	+	71301761	8	8	ENSSSCG00000008937	+	3'UTR	*AMBN*
8	71347079	71347079	-	71347079	11	11	XLOC_085659	-	predicted	---
8	71347198	71347198	-	71347198	24	24	XLOC_085659	-	predicted	---
8	71347282	71347285	-	71347283	203	295	ENSSSCG00000008939	-	3'UTR	*JCHAIN*
8	71356165	71356169	-	71356169	5	8	ENSSSCG00000008939	-	intron	*JCHAIN*
8	71384569	71384569	+	71384569	3	3	ENSSSCG00000028466	+	ORF	---

8	71385439	71385439	+	71385439	3	3	ENSSSCG00000028466	+	ORF	---
8	71458584	71458584	+	71458584	3	3	ENSSSCG00000025514	+	ORF	*RUFY3*
8	71489159	71489169	+	71489159	6	16	XLOC_084809	+	predicted	---
8	71489236	71489257	+	71489237	11	28	XLOC_084809	+	predicted	---
8	71490371	71490372	+	71490371	5	8	XLOC_084809	+	predicted	---
8	71499221	71499232	-	71499231	8	21	ENSSSCG00000008940	-	3'UTR	*GRSF1*
8	71500078	71500078	-	71500078	4	4	ENSSSCG00000008940	-	3'UTR	*GRSF1*
8	71610537	71610537	+	71610537	9	9	XLOC_084811	+	predicted	---
8	71645121	71645121	+	71645121	15	15	---	---	intergenic	---
8	72050149	72050150	+	72050149	6	9	ENSSSCG00000008943	+	3'UTR	*SLC4A4*
8	72227887	72227906	-	72227905	716	1795	XLOC_085663	-	predicted	---
8	72228044	72228046	+	72228046	5	8	---	---	intergenic	---
8	72240286	72240293	-	72240286	17	39	ENSSSCG00000027609	-	ORF	*GC*
8	72241094	72241125	-	72241094	10	15	ENSSSCG00000027609	-	ORF	*GC*
8	72241210	72241210	-	72241210	3	3	ENSSSCG00000027609	-	ORF	*GC*
8	72242307	72242309	-	72242307	23	61	ENSSSCG00000027609	-	intron	*GC*
8	72242356	72242356	-	72242356	5	5	ENSSSCG00000027609	-	ORF	*GC*
8	72246736	72246736	-	72246736	3	3	ENSSSCG00000027609	-	ORF	*GC*
8	72248057	72248088	-	72248057	13	20	ENSSSCG00000027609	-	ORF	*GC*
8	72248152	72248165	-	72248152	12	24	ENSSSCG00000027609	-	ORF	*GC*
8	72251106	72251107	-	72251106	12	19	ENSSSCG00000027609	-	ORF	*GC*
8	72251956	72251956	-	72251956	3	3	ENSSSCG00000027609	-	intron	*GC*
8	72261707	72261723	+	72261723	172	184	---	---	intergenic	---
8	72528150	72528150	-	72528150	4	4	XLOC_085664	-	predicted	---
8	72606651	72606651	-	72606651	4	4	XLOC_085667	-	predicted	---
8	72644399	72644399	-	72644399	3	3	---	---	intergenic	---

8	73235196	73235196	+	73235196	3	3	---	---	intergenic	---
8	73270372	73270372	+	73270372	5	5	---	---	intergenic	---
8	73362869	73362878	-	73362869	5	8	ENSSSCG00000024592	-	3'UTR	*ANKRD17*
8	73399977	73399977	-	73399977	3	3	ENSSSCG00000024592	-	intron	*ANKRD17*
8	73417310	73417310	-	73417310	3	3	ENSSSCG00000024592	-	intron	*ANKRD17*
8	73504028	73504028	-	73504028	3	3	---	---	intergenic	---
8	73599883	73599883	+	73599883	4	4	---	---	intergenic	---
8	73609669	73609669	-	73609669	3	3	---	---	intergenic	---
8	73610310	73610314	-	73610313	7	14	---	---	intergenic	---
8	73612808	73612850	-	73612846	87	289	ENSSSCG00000008948	-	ORF	*ALB*
8	73613516	73613518	-	73613516	76	91	ENSSSCG00000008948	-	intron	*ALB*
8	73613569	73613623	-	73613611	8	20	ENSSSCG00000008948	-	ORF	*ALB*
8	73614844	73614869	-	73614869	45	65	ENSSSCG00000008948	-	ORF	*ALB*
8	73614896	73614947	-	73614923	1215	1303	ENSSSCG00000008948	-	ORF	*ALB*
8	73614998	73615030	-	73614998	56	122	ENSSSCG00000008948	-	ORF	*ALB*
8	73616485	73616511	-	73616509	146	227	ENSSSCG00000008948	-	ORF	*ALB*
8	73616550	73616555	-	73616553	95	124	ENSSSCG00000008948	-	ORF	*ALB*
8	73616597	73616597	-	73616597	3	3	ENSSSCG00000008948	-	ORF	*ALB*
8	73617658	73617705	-	73617658	207	297	ENSSSCG00000008948	-	ORF	*ALB*
8	73619392	73619419	-	73619419	149	152	ENSSSCG00000008948	-	ORF	*ALB*
8	73619452	73619464	-	73619455	135	171	ENSSSCG00000008948	-	ORF	*ALB*
8	73620846	73620896	-	73620896	195	234	ENSSSCG00000008948	-	ORF	*ALB*
8	73620935	73621011	-	73620974	346	421	ENSSSCG00000008948	-	ORF	*ALB*
8	73622163	73622185	-	73622185	5	9	ENSSSCG00000008948	-	ORF	*ALB*
8	73622220	73622250	-	73622230	28	45	ENSSSCG00000008948	-	ORF	*ALB*
8	73624154	73624195	-	73624167	1404	1521	ENSSSCG00000008948	-	ORF	*ALB*

8	73625575	73625576	-	73625576	770	773	ENSSSCG00000008948	-	ORF	*ALB*
8	73625608	73625660	-	73625641	91	116	ENSSSCG00000008948	-	ORF	*ALB*
8	73626235	73626311	-	73626311	1553	2062	ENSSSCG00000008948	-	ORF	*ALB*
8	73626343	73626406	-	73626376	745	1270	ENSSSCG00000008948	-	ORF	*ALB*
8	73626583	73626583	-	73626583	3	3	ENSSSCG00000008948	-	intron	*ALB*
8	73628272	73628352	-	73628312	680	814	ENSSSCG00000008948	-	ORF	*ALB*
8	73629238	73629257	-	73629257	6	16	ENSSSCG00000008948	-	ORF	*ALB*
8	73629993	73630010	-	73630006	7	32	ENSSSCG00000008948	-	ORF	*ALB*
8	73812225	73812225	+	73812225	3	3	ENSSSCG00000008950	+	ORF	*AFM*
8	73819575	73819575	+	73819575	4	4	ENSSSCG00000008950	+	ORF	*AFM*
8	73828342	73828357	+	73828355	28	57	XLOC_084825	+	predicted	---
8	73870619	73870621	+	73870621	10	22	ENSSSCG00000023426	+	3'UTR	---
8	74086565	74086565	+	74086565	3	3	ENSSSCG00000008953	+	3'UTR	*CXCL8*
8	74086742	74086749	+	74086749	92	95	ENSSSCG00000008953	+	3'UTR	*CXCL8*
8	74162169	74162169	+	74162169	65	65	ENSSSCG00000008957	+	3'UTR	*AMCF-II*
8	74311983	74311983	+	74311983	49	49	ENSSSCG00000008959	+	3'UTR	*CXCL2*
8	74523409	74523409	+	74523409	4	4	XLOC_084836	+	predicted	---
8	75040221	75040221	+	75040221	13	13	ENSSSCG00000008966	+	3'UTR	*PARM1*
8	75044131	75044131	+	75044131	3	3	XLOC_084840	+	predicted	---
8	75197166	75197166	-	75197166	8	8	---	---	intergenic	---
8	75268683	75268683	-	75268683	4	4	XLOC_085691	-	predicted	---
8	75276729	75276730	+	75276729	12	16	---	---	intergenic	---
8	75305761	75305761	-	75305761	4	4	XLOC_085692	-	predicted	---
8	75308160	75308164	-	75308164	10	23	ENSSSCG00000029348	-	3'UTR	*THAP6*
8	75321777	75321777	-	75321777	7	7	ENSSSCG00000029348	-	intron	*THAP6*
8	75321893	75321893	-	75321893	3	3	ENSSSCG00000029348	-	ORF	*THAP6*

8	75355287	75355287	-	75355287	21	21	ENSSSCG00000008969	-	3'UTR	*RCHY1*
8	75355650	75355653	-	75355653	9	19	ENSSSCG00000008969	-	3'UTR	*RCHY1*
8	75358225	75358225	-	75358225	3	3	ENSSSCG00000008969	-	intron	*RCHY1*
8	75398473	75398473	-	75398473	5	5	---	---	intergenic	---
8	75411009	75411015	-	75411012	21	30	ENSSSCG00000022940	-	3'UTR	*G3BP2*
8	75413374	75413374	-	75413374	4	4	ENSSSCG00000022940	-	3'UTR	*G3BP2*
8	75413633	75413633	-	75413633	3	3	ENSSSCG00000022940	-	3'UTR	*G3BP2*
8	75423936	75423945	-	75423936	4	8	ENSSSCG00000022940	-	intron	*G3BP2*
8	75464543	75464543	-	75464543	3	3	---	---	intergenic	---
8	75587275	75587275	+	75587275	9	9	XLOC_084848	+	predicted	---
8	75603658	75603658	-	75603658	4	4	XLOC_085697	-	predicted	---
8	75709385	75709386	-	75709386	37	41	ENSSSCG00000008973	-	3'UTR	*NAAA*
8	75751426	75751427	-	75751426	8	12	ENSSSCG00000008974	-	3'UTR	*SDAD1*
8	75767614	75767617	-	75767617	22	34	ENSSSCG00000008974	-	ORF	*SDAD1*
8	75801403	75801403	-	75801403	4	4	ENSSSCG00000023489	-	3'UTR	*CXCL9*
8	75809911	75809914	+	75809914	13	25	---	---	intergenic	---
8	75829905	75829914	-	75829906	112	154	ENSSSCG00000008977	-	3'UTR	*CXCL10*
8	75830295	75830295	-	75830295	9	9	ENSSSCG00000008977	-	3'UTR	*CXCL10*
8	75831155	75831155	-	75831155	8	8	---	---	intergenic	---
8	75909587	75909587	-	75909587	3	3	ENSSSCG00000008978	-	3'UTR	*CXCL11*
8	75953931	75953931	+	75953931	3	3	ENSSSCG00000008976	+	intron	*ART3*
8	75990588	75990588	+	75990588	3	3	---	---	intergenic	---
8	75990828	75990840	+	75990840	7	22	ENSSSCG00000008976	+	3'UTR	*ART3*
8	75990971	75990972	+	75990971	13	17	ENSSSCG00000008976	+	3'UTR	*ART3*
8	75991022	75991030	+	75991028	15	35	XLOC_084852	+	predicted	---
8	75992618	75992618	-	75992618	4	4	XLOC_085705	-	predicted	---

8	76034114	76034122	-	76034122	14	34	ENSSSCG00000008980	-	3'UTR	*SCARB2*
8	76036909	76036910	-	76036910	56	66	ENSSSCG00000008980	-	3'UTR	*SCARB2*
8	76182536	76182545	+	76182545	31	48	XLOC_084855	+	predicted	---
8	76185070	76185070	-	76185070	5	5	XLOC_085707	-	predicted	---
8	76185114	76185117	-	76185114	11	20	XLOC_085707	-	predicted	---
8	76256874	76256878	-	76256874	5	8	ENSSSCG00000008983	-	intron	*CCDC158*
8	76421470	76421471	-	76421471	6	10	---	---	intergenic	---
8	76562104	76562104	+	76562104	12	12	ENSSSCG00000008984	+	intron	*SHROOM3*
8	76617943	76617943	+	76617943	3	3	ENSSSCG00000008984	+	intron	*SHROOM3*
8	76717782	76717782	+	76717782	3	3	---	---	intergenic	---
8	76748568	76748569	-	76748568	3	6	---	---	intergenic	---
8	76749889	76749891	-	76749890	18	26	---	---	intergenic	---
8	76779055	76779057	+	76779056	9	20	---	---	intergenic	---
8	76838189	76838189	-	76838189	3	3	---	---	intergenic	---
8	76878670	76878670	+	76878670	10	10	XLOC_084859	+	predicted	---
8	76885273	76885273	-	76885273	3	3	XLOC_085717	-	predicted	---
8	76887177	76887188	-	76887186	97	180	ENSSSCG00000008987	-	3'UTR	*CCNI*
8	77037951	77037951	+	77037951	3	3	---	---	intergenic	---
8	77177615	77177615	+	77177615	3	3	---	---	intergenic	---
8	77334189	77334190	-	77334189	8	16	---	---	intergenic	---
8	77388872	77388872	-	77388872	3	3	XLOC_085718	-	predicted	---
8	77393794	77393795	-	77393794	8	11	XLOC_085718	-	predicted	---
8	77422318	77422318	-	77422318	4	4	ENSSSCG00000008989	-	ORF	*CNOT6L*
8	77592568	77592568	-	77592568	3	3	---	---	intergenic	---
8	77596418	77596418	-	77596418	4	4	---	---	intergenic	---
8	77608437	77608438	-	77608437	4	7	XLOC_085719	-	predicted	---

8	77608559	77608559	-	77608559	10	10	XLOC_085719	-	predicted	---
8	77656616	77656617	-	77656616	9	14	ENSSSCG00000008990	-	ORF	*MRPL1*
8	78635270	78635272	-	78635271	11	19	XLOC_085722	-	predicted	---
8	78666806	78666806	-	78666806	3	3	ENSSSCG00000008994	-	ORF	*RBM46*
8	78687097	78687102	+	78687102	6	11	---	---	intergenic	---
8	78711568	78711569	-	78711568	8	11	ENSSSCG00000008995	-	3'UTR	*LRAT*
8	78712049	78712049	-	78712049	8	8	ENSSSCG00000008995	-	3'UTR	*LRAT*
8	78762343	78762343	-	78762343	4	4	---	---	intergenic	---
8	78851484	78851484	+	78851484	3	3	ENSSSCG00000024314	+	ORF	---
8	78851608	78851609	+	78851609	14	18	ENSSSCG00000024314	+	ORF	---
8	78851758	78851834	+	78851789	53	150	ENSSSCG00000024314	+	intron	---
8	78852753	78852780	+	78852754	29	54	ENSSSCG00000024314	+	ORF	---
8	78852831	78852831	+	78852831	6	6	ENSSSCG00000024314	+	ORF	---
8	78853493	78853493	+	78853493	3	3	ENSSSCG00000024314	+	ORF	---
8	78855103	78855103	+	78855103	3	3	ENSSSCG00000024314	+	ORF	---
8	78855155	78855158	+	78855156	37	58	ENSSSCG00000024314	+	ORF	---
8	78855236	78855236	+	78855236	3	3	ENSSSCG00000024314	+	ORF	---
8	78857330	78857330	+	78857330	3	3	ENSSSCG00000024314	+	ORF	---
8	78857476	78857476	+	78857476	3	3	ENSSSCG00000024314	+	ORF	---
8	78859029	78859029	+	78859029	7	7	ENSSSCG00000024314	+	ORF	---
8	78859081	78859108	+	78859106	26	53	ENSSSCG00000024314	+	intron	---
8	78859187	78859188	+	78859187	4	7	ENSSSCG00000024314	+	intron	---
8	78859285	78859293	+	78859287	13	43	ENSSSCG00000024314	+	intron	---
8	78859336	78859409	+	78859397	993	2292	ENSSSCG00000024314	+	intron	---
8	78859477	78859495	+	78859485	23	99	ENSSSCG00000024314	+	intron	---
8	78859634	78859634	+	78859634	6	6	ENSSSCG00000024314	+	3'UTR	---

8	78859673	78859711	+	78859700	20	58	ENSSSCG00000024314	+	3'UTR	---
8	78859748	78859763	+	78859762	42	80	ENSSSCG00000024314	+	3'UTR	---
8	78861042	78861042	+	78861042	16	16	ENSSSCG00000024314	+	3'UTR	---
8	78861391	78861400	+	78861400	12	19	XLOC_084872	+	predicted	---
8	78872714	78872714	+	78872714	4	4	---	---	intergenic	---
8	78874168	78874168	+	78874168	3	3	ENSSSCG00000008998	+	intron	---
8	78876052	78876063	+	78876063	81	134	ENSSSCG00000008998	+	ORF	---
8	78876635	78876640	+	78876636	11	17	ENSSSCG00000008998	+	ORF	---
8	78876723	78876726	+	78876726	24	34	ENSSSCG00000008998	+	ORF	---
8	78876765	78876765	+	78876765	25	25	ENSSSCG00000008998	+	ORF	---
8	78878130	78878130	+	78878130	3	3	ENSSSCG00000008998	+	ORF	---
8	78878193	78878226	+	78878194	34	46	ENSSSCG00000008998	+	ORF	---
8	78878889	78878910	+	78878908	15	37	ENSSSCG00000008998	+	ORF	---
8	78878954	78878962	+	78878962	4	7	ENSSSCG00000008998	+	ORF	---
8	78936435	78936435	-	78936435	3	3	XLOC_085728	-	predicted	---
8	78936466	78936466	+	78936466	3	3	XLOC_084874	+	predicted	---
8	78936522	78936526	+	78936526	5	9	XLOC_084874	+	predicted	---
8	78936566	78936566	-	78936566	3	3	XLOC_085728	-	predicted	---
8	78936585	78936585	+	78936585	7	7	XLOC_084874	+	predicted	---
8	78936641	78936641	-	78936641	4	4	XLOC_085728	-	predicted	---
8	78936694	78936712	-	78936712	4	7	XLOC_085728	-	predicted	---
8	78936739	78936739	+	78936739	6	6	XLOC_084874	+	predicted	---
8	78936750	78936830	-	78936777	6	19	XLOC_085728	-	predicted	---
8	78936803	78936803	+	78936803	3	3	XLOC_084874	+	predicted	---
8	78936881	78936915	+	78936882	16	45	XLOC_084874	+	predicted	---
8	78936937	78936940	-	78936937	3	6	XLOC_085728	-	predicted	---

8	78936988	78936988	-	78936988	3	3	XLOC_085728	-	predicted	---
8	78937084	78937084	-	78937084	4	4	XLOC_085728	-	predicted	---
8	78937148	78937161	-	78937161	5	8	XLOC_085728	-	predicted	---
8	78937174	78937178	+	78937176	36	94	XLOC_084874	+	predicted	---
8	78937221	78937221	-	78937221	3	3	XLOC_085728	-	predicted	---
8	78937271	78937353	+	78937320	595	2433	XLOC_084874	+	predicted	---
8	78937772	78937772	+	78937772	3	3	XLOC_084874	+	predicted	---
8	78938613	78938613	+	78938613	38	38	ENSSSCG00000028901	+	intron	---
8	78938707	78938716	+	78938714	422	674	ENSSSCG00000028901	+	intron	---
8	79603401	79603401	-	79603401	4	4	---	---	intergenic	---
8	79861970	79861970	+	79861970	6	6	ENSSSCG00000023198	+	3'UTR	---
8	79862053	79862054	+	79862053	7	10	ENSSSCG00000023198	+	3'UTR	---
8	80022200	80022200	+	80022200	3	3	ENSSSCG00000009007	+	intron	---
8	80453275	80453275	-	80453275	4	4	---	---	intergenic	---
8	80611810	80611813	+	80611813	6	14	---	---	intergenic	---
8	80737493	80737500	-	80737497	33	61	ENSSSCG00000009010	-	3'UTR	*ARFIP1*
8	80743698	80743699	-	80743699	15	18	ENSSSCG00000009010	-	intron	*ARFIP1*
8	80931920	80931920	+	80931920	5	5	---	---	intergenic	---
8	80966047	80966048	+	80966047	24	28	XLOC_084894	+	predicted	---
8	80967963	80967963	+	80967963	7	7	XLOC_084894	+	predicted	---
8	81479782	81479782	+	81479782	3	3	XLOC_084900	+	predicted	---
8	81898593	81898593	+	81898593	3	3	ENSSSCG00000009015	+	3'UTR	---
8	82495149	82495149	-	82495149	21	21	---	---	intergenic	---
8	82580651	82580666	+	82580660	4	14	XLOC_084904	+	predicted	---
8	82581031	82581046	+	82581039	24	67	XLOC_084904	+	predicted	---
8	82594428	82594446	-	82594437	761	1627	ENSSSCG00000009019	-	3'UTR	*RPS3A*

8	82597251	82597251	-	82597251	3	3	ENSSSCG00000009019	-	ORF	*RPS3A*
8	82874137	82874137	-	82874137	3	3	---	---	intergenic	---
8	83214707	83214707	+	83214707	5	5	ENSSSCG00000009021	+	intron	---
8	83254681	83254681	+	83254681	3	3	ENSSSCG00000009021	+	intron	---
8	83495746	83495746	-	83495746	4	4	---	---	intergenic	---
8	83846140	83846140	+	83846140	3	3	---	---	intergenic	---
8	84158609	84158610	+	84158610	26	33	---	---	intergenic	---
8	84370238	84370238	+	84370238	4	4	XLOC_084912	+	predicted	---
8	84671897	84671897	+	84671897	3	3	---	---	intergenic	---
8	84930229	84930229	-	84930229	3	3	---	---	intergenic	---
8	84968883	84968883	-	84968883	4	4	---	---	intergenic	---
8	85197502	85197502	-	85197502	14	14	---	---	intergenic	---
8	85217073	85217073	-	85217073	3	3	---	---	intergenic	---
8	85951117	85951118	-	85951117	7	10	ENSSSCG00000009029	-	intron	---
8	85971936	85971936	-	85971936	9	9	ENSSSCG00000009029	-	intron	---
8	86039821	86039821	-	86039821	3	3	ENSSSCG00000009029	-	intron	---
8	86051016	86051016	+	86051016	3	3	---	---	intergenic	---
8	86075596	86075596	+	86075596	3	3	ENSSSCG00000030635	+	ORF	---
8	86291613	86291613	-	86291613	3	3	ENSSSCG00000009031	-	3'UTR	*EDNRA*
8	86354142	86354142	-	86354142	3	3	---	---	intergenic	---
8	86377964	86377964	+	86377964	3	3	---	---	intergenic	---
8	86497112	86497112	+	86497112	6	6	---	---	intergenic	---
8	86593780	86593782	-	86593782	8	13	---	---	intergenic	---
8	87036500	87036512	-	87036504	5	16	---	---	intergenic	---
8	87126144	87126144	-	87126144	13	13	---	---	intergenic	---
8	87277794	87277794	-	87277794	4	4	---	---	intergenic	---

8	87708203	87708203	-	87708203	6	6	XLOC_085782	-	predicted	---
8	87711826	87711826	-	87711826	4	4	XLOC_085782	-	predicted	---
8	87712002	87712033	-	87712027	39	90	ENSSSCG00000009036	-	3'UTR	*LSM6*
8	87716591	87716592	-	87716591	3	6	ENSSSCG00000009036	-	intron	*LSM6*
8	87959899	87959899	+	87959899	5	5	ENSSSCG00000009037	+	intron	*ZNF827*
8	88048588	88048588	+	88048588	3	3	ENSSSCG00000009037	+	intron	*ZNF827*
8	88104057	88104059	+	88104058	21	30	XLOC_084930	+	predicted	---
8	88168927	88168927	+	88168927	6	6	XLOC_084932	+	predicted	---
8	88193865	88193865	-	88193865	8	8	---	---	intergenic	---
8	88270375	88270375	+	88270375	4	4	---	---	intergenic	---
8	88352978	88352982	-	88352978	6	9	---	---	intergenic	---
8	88558469	88558469	+	88558469	3	3	XLOC_084934	+	predicted	---
8	88572102	88572113	-	88572103	27	34	ENSSSCG00000009043	-	3'UTR	*ABCE1*
8	88573039	88573039	-	88573039	5	5	ENSSSCG00000009043	-	3'UTR	*ABCE1*
8	88573230	88573230	-	88573230	5	5	ENSSSCG00000009043	-	3'UTR	*ABCE1*
8	88573539	88573539	-	88573539	3	3	ENSSSCG00000009043	-	3'UTR	*ABCE1*
8	88580798	88580798	-	88580798	4	4	ENSSSCG00000009043	-	ORF	*ABCE1*
8	88581576	88581576	-	88581576	3	3	ENSSSCG00000009043	-	intron	*ABCE1*
8	88617867	88617867	-	88617867	3	3	ENSSSCG00000009043	-	ORF	*ABCE1*
8	88697661	88697661	+	88697661	6	6	XLOC_084935	+	predicted	---
8	88708935	88708935	+	88708935	8	8	---	---	intergenic	---
8	88827277	88827277	+	88827277	3	3	XLOC_084936	+	predicted	---
8	88833746	88833752	+	88833750	11	37	XLOC_084936	+	predicted	---
8	89154729	89154729	+	89154729	3	3	---	---	intergenic	---
8	89163423	89163423	-	89163423	3	3	---	---	intergenic	---
8	89339604	89339605	-	89339604	8	14	XLOC_085795	-	predicted	---

8	89340586	89340586	-	89340586	14	14	XLOC_085795	-	predicted	---
8	89340791	89340791	-	89340791	3	3	XLOC_085795	-	predicted	---
8	89341636	89341636	-	89341636	3	3	ENSSSCG00000009047	-	ORF	*SMARCA5*
8	89344289	89344289	-	89344289	3	3	ENSSSCG00000009047	-	ORF	*SMARCA5*
8	89348187	89348187	-	89348187	3	3	ENSSSCG00000009047	-	intron	*SMARCA5*
8	89395590	89395590	-	89395590	4	4	---	---	intergenic	---
8	89410545	89410545	-	89410545	12	12	ENSSSCG00000009048	-	3'UTR	*GAB1*
8	89415742	89415742	-	89415742	3	3	---	---	intergenic	---
8	89612442	89612442	+	89612442	5	5	---	---	intergenic	---
8	89616738	89616738	-	89616738	7	7	---	---	intergenic	---
8	89716676	89716676	-	89716676	5	5	XLOC_085799	-	predicted	---
8	89716815	89716815	-	89716815	6	6	XLOC_085799	-	predicted	---
8	91340603	91340604	+	91340603	3	6	ENSSSCG00000009052	+	3'UTR	---
8	91340809	91340810	+	91340810	7	10	ENSSSCG00000009052	+	3'UTR	---
8	91426166	91426170	+	91426166	6	10	---	---	intergenic	---
8	91452163	91452164	+	91452164	22	28	---	---	intergenic	---
8	91496561	91496561	-	91496561	4	4	---	---	intergenic	---
8	91833688	91833688	+	91833688	3	3	---	---	intergenic	---
8	91962295	91962296	+	91962296	26	31	---	---	intergenic	---
8	91976401	91976402	+	91976402	4	7	ENSSSCG00000009055	+	ORF	*ELMOD2*
8	91976727	91976727	+	91976727	4	4	XLOC_084956	+	predicted	---
8	91989572	91989572	-	91989572	4	4	XLOC_085812	-	predicted	---
8	92097400	92097400	+	92097400	3	3	---	---	intergenic	---
8	92105630	92105630	+	92105630	5	5	XLOC_084957	+	predicted	---
8	92105753	92105753	+	92105753	5	5	XLOC_084957	+	predicted	---
8	92108437	92108441	+	92108439	51	92	XLOC_084957	+	predicted	---

8	92108734	92108742	+	92108741	8	23	XLOC_084957	+	predicted	---
8	92119925	92119925	+	92119925	6	6	ENSSSCG00000026360	+	intron	*CLGN*
8	92132017	92132019	+	92132018	217	225	ENSSSCG00000026360	+	intron	*CLGN*
8	92132998	92132998	+	92132998	3	3	ENSSSCG00000026360	+	ORF	*CLGN*
8	92158389	92158389	+	92158389	3	3	ENSSSCG00000026360	+	ORF	*CLGN*
8	92162267	92162267	+	92162267	4	4	ENSSSCG00000026360	+	ORF	*CLGN*
8	92163636	92163636	+	92163636	5	5	ENSSSCG00000026360	+	ORF	*CLGN*
8	92166146	92166174	+	92166163	22	87	ENSSSCG00000026360	+	3'UTR	*CLGN*
8	92166495	92166505	+	92166505	19	53	ENSSSCG00000026360	+	3'UTR	*CLGN*
8	92166803	92166803	+	92166803	3	3	ENSSSCG00000026360	+	3'UTR	*CLGN*
8	92167606	92167607	+	92167606	6	9	XLOC_084958	+	predicted	---
8	92172593	92172597	-	92172595	30	56	XLOC_085816	-	predicted	---
8	92172894	92172894	-	92172894	4	4	XLOC_085816	-	predicted	---
8	92173598	92173598	-	92173598	4	4	XLOC_085816	-	predicted	---
8	92173880	92173887	-	92173880	11	19	XLOC_085816	-	predicted	---
8	92173925	92173929	-	92173926	10	24	XLOC_085816	-	predicted	---
8	92193018	92193018	+	92193018	4	4	---	---	intergenic	---
8	92234448	92234448	+	92234448	3	3	---	---	intergenic	---
8	92431278	92431280	+	92431280	4	7	XLOC_084961	+	predicted	---
8	92433169	92433169	-	92433169	3	3	---	---	intergenic	---
8	92486247	92486247	+	92486247	3	3	---	---	intergenic	---
8	92874590	92874599	-	92874598	18	45	XLOC_085818	-	predicted	---
8	92905807	92905808	+	92905808	4	7	---	---	intergenic	---
8	93113166	93113171	+	93113166	5	8	ENSSSCG00000020711	+	3'UTR	*RAB33B*
8	93115437	93115439	+	93115439	10	13	ENSSSCG00000020711	+	3'UTR	*RAB33B*
8	93186872	93186872	-	93186872	4	4	ENSSSCG00000009061	-	3'UTR	*NAA15*

8	93187894	93187894	-	93187894	42	42	ENSSSCG00000009061	-	3'UTR	*NAA15*
8	93188993	93188993	-	93188993	7	7	ENSSSCG00000009061	-	3'UTR	*NAA15*
8	93207673	93207673	-	93207673	3	3	ENSSSCG00000009061	-	ORF	*NAA15*
8	93305837	93305837	-	93305837	5	5	ENSSSCG00000009061	-	intron	*NAA15*
8	93384983	93384992	-	93384983	3	6	ENSSSCG00000009061	-	intron	*NAA15*
8	93410467	93410468	+	93410467	24	27	ENSSSCG00000009063	+	3'UTR	*NDUFC1*
8	93410649	93410649	+	93410649	5	5	XLOC_084969	+	predicted	---
8	93410788	93410791	+	93410790	34	64	XLOC_084969	+	predicted	---
8	93472861	93472861	+	93472861	5	5	ENSSSCG00000029920	+	3'UTR	---
8	93479456	93479456	-	93479456	4	4	ENSSSCG00000024596	-	3'UTR	*CCRN4L*
8	93480619	93480619	-	93480619	4	4	ENSSSCG00000024596	-	3'UTR	*CCRN4L*
8	93535107	93535107	-	93535107	4	4	---	---	intergenic	---
8	93619301	93619301	+	93619301	3	3	---	---	intergenic	---
8	93639358	93639358	+	93639358	23	23	---	---	intergenic	---
8	95284665	95284665	-	95284665	4	4	---	---	intergenic	---
8	95604533	95604533	+	95604533	3	3	---	---	intergenic	---
8	95696032	95696032	-	95696032	3	3	---	---	intergenic	---
8	95886365	95886365	+	95886365	3	3	---	---	intergenic	---
8	95896899	95896900	+	95896900	6	9	---	---	intergenic	---
8	95941362	95941362	+	95941362	6	6	---	---	intergenic	---
8	95968548	95968549	+	95968548	16	20	---	---	intergenic	---
8	96176683	96176683	+	96176683	5	5	---	---	intergenic	---
8	96634547	96634548	+	96634547	9	12	---	---	intergenic	---
8	96755476	96755476	-	96755476	5	5	---	---	intergenic	---
8	96879950	96879950	-	96879950	10	10	---	---	intergenic	---
8	97256792	97256792	+	97256792	4	4	---	---	intergenic	---

8	97880801	97880801	+	97880801	9	9	---	---	intergenic	---
8	98733451	98733451	+	98733451	3	3	---	---	intergenic	---
8	99370582	99370582	+	99370582	3	3	XLOC_084983	+	predicted	---
8	99434112	99434112	-	99434112	5	5	---	---	intergenic	---
8	99733262	99733262	-	99733262	3	3	---	---	intergenic	---
8	99907412	99907412	-	99907412	4	4	---	---	intergenic	---
8	100261137	100261137	+	100261137	5	5	---	---	intergenic	---
8	100279648	100279648	-	100279648	3	3	---	---	intergenic	---
8	100282001	100282001	-	100282001	4	4	---	---	intergenic	---
8	100778004	100778004	-	100778004	8	8	---	---	intergenic	---
8	100930747	100930747	-	100930747	3	3	---	---	intergenic	---
8	101196711	101196711	+	101196711	11	11	---	---	intergenic	---
8	101343136	101343137	+	101343136	5	9	---	---	intergenic	---
8	101536966	101536967	+	101536967	5	8	XLOC_084985	+	predicted	---
8	101746932	101746933	-	101746933	8	11	---	---	intergenic	---
8	102146468	102146468	-	102146468	4	4	---	---	intergenic	---
8	102271286	102271286	+	102271286	5	5	---	---	intergenic	---
8	102397998	102397999	+	102397998	9	16	---	---	intergenic	---
8	102586053	102586060	-	102586054	9	29	XLOC_085839	-	predicted	---
8	102652173	102652176	-	102652173	5	8	XLOC_085839	-	predicted	---
8	102994209	102994209	+	102994209	3	3	ENSSSCG00000009071	+	intron	*JADE1*
8	103053784	103053784	+	103053784	4	4	ENSSSCG00000009071	+	3'UTR	*JADE1*
8	103063150	103063150	-	103063150	7	7	ENSSSCG00000029943	-	3'UTR	*SCLT1*
8	103205611	103205611	-	103205611	5	5	---	---	intergenic	---
8	103354042	103354042	+	103354042	3	3	---	---	intergenic	---
8	103518734	103518734	+	103518734	3	3	---	---	intergenic	---

8	103536339	103536339	+	103536339	3	3	---	---	intergenic	---
8	103557944	103557944	-	103557944	10	10	---	---	intergenic	---
8	103578569	103578569	+	103578569	9	9	---	---	intergenic	---
8	103581669	103581670	+	103581670	5	9	ENSSSCG00000009072	+	3'UTR	*PGRMC2*
8	103582566	103582573	+	103582569	15	30	XLOC_084991	+	predicted	---
8	103586818	103586818	-	103586818	9	9	---	---	intergenic	---
8	103620879	103620881	-	103620881	5	9	---	---	intergenic	---
8	103622882	103622882	-	103622882	21	21	XLOC_085843	-	predicted	---
8	103641891	103641891	-	103641891	6	6	---	---	intergenic	---
8	103683138	103683155	-	103683147	11	27	XLOC_085843	-	predicted	---
8	103683725	103683725	-	103683725	5	5	XLOC_085843	-	predicted	---
8	103839335	103839335	+	103839335	3	3	ENSSSCG00000009075	+	3'UTR	*MFSD8*
8	103855365	103855365	-	103855365	3	3	ENSSSCG00000009076	-	3'UTR	*PLK4*
8	103855624	103855626	-	103855626	22	35	ENSSSCG00000009076	-	3'UTR	*PLK4*
8	103895980	103895982	-	103895982	4	7	XLOC_085847	-	predicted	---
8	103899107	103899107	-	103899107	4	4	XLOC_085847	-	predicted	---
8	103899364	103899366	-	103899364	3	6	XLOC_085847	-	predicted	---
8	103899883	103899899	-	103899883	4	10	XLOC_085847	-	predicted	---
8	103900881	103900881	-	103900881	3	3	XLOC_085847	-	predicted	---
8	103900932	103900936	-	103900934	28	51	XLOC_085847	-	predicted	---
8	103900998	103901007	-	103900998	16	19	XLOC_085847	-	predicted	---
8	103908933	103908933	-	103908933	3	3	ENSSSCG00000009077	-	intron	*HSPA4L*
8	103914226	103914226	-	103914226	9	9	ENSSSCG00000009077	-	ORF	*HSPA4L*
8	103958653	103958655	-	103958655	6	9	XLOC_085848	-	predicted	---
8	103958707	103958707	-	103958707	3	3	XLOC_085848	-	predicted	---
8	104085069	104085069	-	104085069	4	4	ENSSSCG00000009079	-	intron	*INTU*

8	104274293	104274293	-	104274293	4	4	---	---	intergenic	---
8	104382241	104382241	-	104382241	3	3	---	---	intergenic	---
8	104462154	104462154	-	104462154	5	5	---	---	intergenic	---
8	104559951	104559957	+	104559953	115	286	---	---	intergenic	---
8	104560975	104560975	+	104560975	4	4	---	---	intergenic	---
8	104561254	104561254	-	104561254	3	3	---	---	intergenic	---
8	104561324	104561324	+	104561324	3	3	---	---	intergenic	---
8	104582632	104582637	+	104582636	16	30	---	---	intergenic	---
8	104610652	104610652	+	104610652	3	3	XLOC_084996	+	predicted	---
8	104637407	104637409	+	104637407	9	19	XLOC_084996	+	predicted	---
8	104900926	104900929	-	104900929	11	15	---	---	intergenic	---
8	104926829	104926829	+	104926829	3	3	---	---	intergenic	---
8	105229706	105229706	+	105229706	9	9	---	---	intergenic	---
8	105266424	105266424	+	105266424	6	6	XLOC_084998	+	predicted	---
8	105281656	105281656	-	105281656	8	8	---	---	intergenic	---
8	105448744	105448747	+	105448747	6	10	---	---	intergenic	---
8	105724192	105724194	-	105724192	6	9	---	---	intergenic	---
8	105724224	105724224	+	105724224	3	3	---	---	intergenic	---
8	105830422	105830422	-	105830422	3	3	---	---	intergenic	---
8	105950360	105950360	+	105950360	3	3	---	---	intergenic	---
8	105950722	105950722	+	105950722	3	3	---	---	intergenic	---
8	106030157	106030157	+	106030157	3	3	---	---	intergenic	---
8	106646421	106646421	+	106646421	4	4	---	---	intergenic	---
8	106679118	106679118	-	106679118	9	9	---	---	intergenic	---
8	107268052	107268052	+	107268052	3	3	---	---	intergenic	---
8	107422148	107422148	-	107422148	7	7	---	---	intergenic	---

8	107695009	107695009	-	107695009	3	3	---	---	intergenic	---
8	107705396	107705396	+	107705396	3	3	---	---	intergenic	---
8	107741540	107741540	+	107741540	4	4	---	---	intergenic	---
8	107826302	107826302	-	107826302	3	3	---	---	intergenic	---
8	107923523	107923523	+	107923523	3	3	---	---	intergenic	---
8	108133442	108133449	+	108133443	15	25	XLOC_085011	+	predicted	---
8	108436769	108436769	-	108436769	3	3	ENSSSCG00000009084	-	intron	*SPATA5*
8	108679343	108679343	-	108679343	27	27	---	---	intergenic	---
8	108822413	108822413	+	108822413	4	4	---	---	intergenic	---
8	108829636	108829636	-	108829636	4	4	---	---	intergenic	---
8	108829992	108829997	-	108829997	24	73	ENSSSCG00000009089	-	3'UTR	*ADAD1*
8	108861868	108861868	-	108861868	5	5	ENSSSCG00000009089	-	intron	*ADAD1*
8	108861911	108861914	-	108861912	84	103	ENSSSCG00000009089	-	intron	*ADAD1*
8	108861969	108861979	-	108861979	6	9	ENSSSCG00000009089	-	intron	*ADAD1*
8	108862024	108862028	-	108862025	19	36	ENSSSCG00000009089	-	intron	*ADAD1*
8	108870320	108870320	-	108870320	4	4	ENSSSCG00000009089	-	ORF	*ADAD1*
8	108893579	108893586	-	108893579	9	13	XLOC_085869	-	predicted	---
8	108894139	108894139	-	108894139	3	3	ENSSSCG00000009090	-	ORF	*KIAA1109*
8	109060450	109060450	-	109060450	3	3	ENSSSCG00000009090	-	intron	*KIAA1109*
8	109097513	109097513	+	109097513	5	5	XLOC_085019	+	predicted	---
8	109421609	109421609	+	109421609	5	5	ENSSSCG00000009093	+	ORF	*BBS7*
8	109454812	109454812	+	109454812	4	4	XLOC_085021	+	predicted	---
8	109461803	109461803	+	109461803	4	4	ENSSSCG00000009094	+	3'UTR	*CCNA2*
8	109462297	109462299	-	109462297	33	53	---	---	intergenic	---
8	109627216	109627216	+	109627216	3	3	ENSSSCG00000009097	+	ORF	*ANXA5*
8	109641804	109641804	+	109641804	3	3	ENSSSCG00000009097	+	3'UTR	*ANXA5*

8	109641859	109641864	+	109641862	163	264	ENSSSCG00000009097	+	3'UTR	*ANXA5*
8	110321394	110321407	-	110321407	8	19	XLOC_085875	-	predicted	---
8	110674484	110674484	-	110674484	6	6	---	---	intergenic	---
8	110851148	110851162	+	110851148	3	6	XLOC_085032	+	predicted	---
8	111063158	111063158	+	111063158	16	16	---	---	intergenic	---
8	111483620	111483620	+	111483620	3	3	---	---	intergenic	---
8	111506079	111506093	+	111506082	36	58	ENSSSCG00000009104	+	3'UTR	*MAD2L1*
8	111539879	111539879	+	111539879	3	3	---	---	intergenic	---
8	111550896	111550896	+	111550896	3	3	XLOC_085037	+	predicted	---
8	111748988	111748988	-	111748988	4	4	---	---	intergenic	---
8	112152595	112152595	-	112152595	3	3	XLOC_085880	-	predicted	---
8	112155289	112155289	-	112155289	3	3	XLOC_085881	-	predicted	---
8	112309149	112309152	-	112309149	11	14	---	---	intergenic	---
8	112710241	112710241	-	112710241	3	3	ENSSSCG00000009113	-	3'UTR	*METTL14*
8	112778264	112778272	-	112778268	41	72	ENSSSCG00000030024	-	3'UTR	---
8	112910459	112910459	+	112910459	3	3	ENSSSCG00000009114	+	3'UTR	*PRSS12*
8	112911816	112911825	-	112911824	27	57	XLOC_085892	-	predicted	---
8	112911888	112911899	-	112911888	6	14	XLOC_085892	-	predicted	---
8	113265214	113265215	+	113265215	9	12	---	---	intergenic	---
8	114236387	114236387	+	114236387	4	4	---	---	intergenic	---
8	114353194	114353195	+	114353195	9	15	---	---	intergenic	---
8	114380936	114380936	-	114380936	3	3	---	---	intergenic	---
8	114667146	114667146	-	114667146	3	3	---	---	intergenic	---
8	114925167	114925167	+	114925167	3	3	---	---	intergenic	---
8	115141472	115141472	+	115141472	3	3	---	---	intergenic	---
8	115165483	115165485	-	115165484	9	18	---	---	intergenic	---

8	115181923	115181923	-	115181923	3	3	---	---	intergenic	---
8	115188814	115188814	-	115188814	4	4	XLOC_085898	-	predicted	---
8	115189079	115189079	-	115189079	6	6	ENSSSCG00000009119	-	3'UTR	*LARP7*
8	115198512	115198512	-	115198512	4	4	ENSSSCG00000009119	-	intron	*LARP7*
8	115201465	115201469	-	115201468	18	36	ENSSSCG00000009119	-	ORF	*LARP7*
8	115754406	115754407	+	115754406	54	62	---	---	intergenic	---
8	115760191	115760198	+	115760197	7	15	XLOC_085062	+	predicted	---
8	116001889	116001889	-	116001889	8	8	---	---	intergenic	---
8	116376157	116376159	+	116376157	3	6	---	---	intergenic	---
8	116524928	116524928	+	116524928	5	5	---	---	intergenic	---
8	117106067	117106067	+	117106067	3	3	ENSSSCG00000009123	+	intron	*CAMK2D*
8	117139750	117139750	+	117139750	3	3	ENSSSCG00000009123	+	intron	*CAMK2D*
8	117242921	117242921	+	117242921	5	5	ENSSSCG00000009123	+	intron	*CAMK2D*
8	117261925	117261925	+	117261925	11	11	XLOC_085068	+	predicted	---
8	117272168	117272168	+	117272168	11	11	---	---	intergenic	---
8	117543853	117543853	-	117543853	6	6	ENSSSCG00000009125	-	intron	*ANK2*
8	117931672	117931672	-	117931672	3	3	---	---	intergenic	---
8	117991878	117991878	+	117991878	6	6	ENSSSCG00000009126	+	ORF	---
8	118183060	118183060	+	118183060	3	3	ENSSSCG00000009127	+	intron	*ALPK1*
8	118184843	118184843	+	118184843	5	5	ENSSSCG00000009127	+	intron	*ALPK1*
8	118509310	118509310	-	118509310	5	5	---	---	intergenic	---
8	118510360	118510360	+	118510360	3	3	---	---	intergenic	---
8	119005718	119005719	+	119005718	5	8	---	---	intergenic	---
8	119421842	119421842	-	119421842	3	3	---	---	intergenic	---
8	119890919	119890921	-	119890920	8	16	---	---	intergenic	---
8	119968867	119968867	-	119968867	3	3	ENSSSCG00000009132	-	3'UTR	*ENPEP*

8	120120401	120120401	-	120120401	10	10	ENSSSCG00000025541	-	intron	*ELOVL6*
8	120251095	120251104	+	120251095	6	11	ENSSSCG00000009133	+	ORF	---
8	120483853	120483854	-	120483853	12	15	ENSSSCG00000020907	-	3'UTR	---
8	120485817	120485817	-	120485817	5	5	ENSSSCG00000020907	-	3'UTR	---
8	120485864	120485864	-	120485864	5	5	ENSSSCG00000020907	-	3'UTR	---
8	120513308	120513317	+	120513317	4	10	---	---	intergenic	---
8	120517393	120517393	+	120517393	3	3	ENSSSCG00000009138	+	intron	*CFI*
8	120527630	120527630	+	120527630	6	6	---	---	intergenic	---
8	120527745	120527745	+	120527745	4	4	---	---	intergenic	---
8	120527801	120527804	+	120527804	115	143	---	---	intergenic	---
8	120529730	120529730	+	120529730	14	14	---	---	intergenic	---
8	120535658	120535699	+	120535661	23	80	---	---	intergenic	---
8	120540619	120540619	+	120540619	3	3	---	---	intergenic	---
8	120543003	120543004	+	120543004	7	13	---	---	intergenic	---
8	120548614	120548614	+	120548614	4	4	---	---	intergenic	---
8	120548658	120548658	+	120548658	19	19	---	---	intergenic	---
8	120548802	120548804	+	120548802	64	77	---	---	intergenic	---
8	120552257	120552257	+	120552257	10	10	---	---	intergenic	---
8	120554048	120554048	+	120554048	5	5	---	---	intergenic	---
8	120554234	120554235	+	120554234	13	22	ENSSSCG00000009138	+	3'UTR	*CFI*
8	120595995	120595998	+	120595997	18	42	ENSSSCG00000009139	+	3'UTR	*PLA2G12A*
8	120678399	120678401	+	120678399	10	20	ENSSSCG00000009140	+	3'UTR	*CASP6*
8	120683276	120683285	-	120683283	82	109	ENSSSCG00000009141	-	3'UTR	*CCDC109B*
8	120687768	120687768	-	120687768	4	4	ENSSSCG00000009141	-	ORF	*CCDC109B*
8	120894729	120894730	-	120894730	10	18	---	---	intergenic	---
8	121254033	121254033	+	121254033	3	3	---	---	intergenic	---

8	121294462	121294462	+	121294462	3	3	---	---	intergenic	---
8	121622419	121622421	+	121622419	14	25	XLOC_085089	+	predicted	---
8	121666887	121666887	-	121666887	4	4	---	---	intergenic	---
8	121674963	121674967	-	121674963	64	113	ENSSSCG00000009145	-	3'UTR	*OSTC*
8	121675189	121675195	-	121675192	9	32	ENSSSCG00000009145	-	3'UTR	*OSTC*
8	121679643	121679652	-	121679643	7	10	ENSSSCG00000009145	-	ORF	*OSTC*
8	121707356	121707365	-	121707365	245	391	ENSSSCG00000009146	-	3'UTR	*RPL34*
8	121765508	121765508	-	121765508	3	3	---	---	intergenic	---
8	121829611	121829617	-	121829611	4	7	---	---	intergenic	---
8	122204407	122204408	+	122204407	14	24	XLOC_085093	+	predicted	---
8	122210369	122210377	-	122210377	5	11	XLOC_085934	-	predicted	---
8	122212872	122212906	-	122212880	97	274	ENSSSCG00000009150	-	3'UTR	*HADH*
8	122213339	122213339	-	122213339	3	3	ENSSSCG00000009150	-	3'UTR	*HADH*
8	122213381	122213401	-	122213381	4	7	ENSSSCG00000009150	-	3'UTR	*HADH*
8	122277258	122277263	-	122277263	7	12	XLOC_085935	-	predicted	---
8	122318458	122318458	-	122318458	4	4	ENSSSCG00000009152	-	3'UTR	*SGMS2*
8	122336086	122336086	-	122336086	4	4	ENSSSCG00000009152	-	ORF	*SGMS2*
8	122549560	122549560	+	122549560	28	28	XLOC_085101	+	predicted	---
8	122682003	122682003	-	122682003	3	3	---	---	intergenic	---
8	123293847	123293847	+	123293847	10	10	---	---	intergenic	---
8	123319912	123319912	+	123319912	3	3	---	---	intergenic	---
8	123380481	123380481	-	123380481	6	6	---	---	intergenic	---
8	123610488	123610488	+	123610488	3	3	---	---	intergenic	---
8	123653111	123653111	+	123653111	3	3	---	---	intergenic	---
8	123840341	123840351	-	123840347	67	166	ENSSSCG00000024168	-	3'UTR	*SCYE1*
8	123840392	123840403	-	123840403	6	10	ENSSSCG00000024168	-	3'UTR	*SCYE1*

8	123889011	123889011	-	123889011	4	4	ENSSSCG00000024168	-	ORF	*SCYE1*
8	123891644	123891644	-	123891644	3	3	ENSSSCG00000024168	-	intron	*SCYE1*
8	124325381	124325387	+	124325387	4	7	ENSSSCG00000023548	+	intron	---
8	124532047	124532051	+	124532047	8	12	ENSSSCG00000030617	+	3'UTR	*INTS12*
8	124714077	124714077	-	124714077	4	4	ENSSSCG00000009156	-	intron	---
8	124855996	124855996	+	124855996	3	3	ENSSSCG00000022365	+	intron	*PPA2*
8	125151693	125151693	+	125151693	3	3	---	---	intergenic	---
8	125545272	125545274	-	125545274	6	11	---	---	intergenic	---
8	126071297	126071297	+	126071297	3	3	---	---	intergenic	---
8	126363637	126363637	+	126363637	3	3	---	---	intergenic	---
8	126800678	126800678	+	126800678	3	3	ENSSSCG00000023296	+	intron	*CENPE*
8	127077622	127077630	+	127077628	7	13	ENSSSCG00000009162	+	intron	*SLC9B1*
8	127103454	127103457	+	127103457	4	7	XLOC_085129	+	predicted	---
8	127103565	127103568	+	127103567	19	40	XLOC_085129	+	predicted	---
8	127126777	127126777	-	127126777	3	3	XLOC_085955	-	predicted	---
8	127126876	127126876	-	127126876	3	3	XLOC_085955	-	predicted	---
8	127238772	127238773	+	127238773	5	8	ENSSSCG00000026525	+	intron	---
8	127514799	127514799	+	127514799	3	3	ENSSSCG00000009166	+	intron	---
8	127563790	127563790	+	127563790	3	3	ENSSSCG00000009166	+	3'UTR	---
8	127753645	127753653	-	127753653	16	22	XLOC_085958	-	predicted	---
8	127843689	127843713	-	127843713	9	12	---	---	intergenic	---
8	127863029	127863030	+	127863029	7	13	---	---	intergenic	---
8	127899814	127899814	+	127899814	4	4	---	---	intergenic	---
8	128231116	128231116	-	128231116	7	7	ENSSSCG00000009171	-	intron	---
8	128459576	128459577	-	128459576	5	8	XLOC_085962	-	predicted	---
8	128632466	128632466	+	128632466	4	4	ENSSSCG00000009172	+	intron	---

8	129214168	129214168	+	129214168	4	4	---	---	intergenic	---
8	129233057	129233057	+	129233057	3	3	---	---	intergenic	---
8	129335742	129335742	+	129335742	3	3	XLOC_085146	+	predicted	---
8	129459686	129459686	-	129459686	4	4	---	---	intergenic	---
8	129493145	129493145	-	129493145	3	3	XLOC_085966	-	predicted	---
8	129553437	129553442	+	129553437	12	23	ENSSSCG00000025240	+	3'UTR	*DDIT4L*
8	129608807	129608807	+	129608807	4	4	---	---	intergenic	---
8	129695523	129695523	+	129695523	3	3	---	---	intergenic	---
8	129745279	129745279	+	129745279	3	3	ENSSSCG00000009178	+	3'UTR	*H2AFZ*
8	129745422	129745429	+	129745423	77	102	ENSSSCG00000009178	+	3'UTR	*H2AFZ*
8	129745627	129745627	+	129745627	4	4	XLOC_085149	+	predicted	---
8	129798205	129798211	+	129798209	5	11	ENSSSCG00000009177	+	3'UTR	*DNAJB14*
8	129802761	129802762	+	129802761	4	7	XLOC_085150	+	predicted	---
8	129821867	129821875	+	129821874	57	93	ENSSSCG00000009176	+	3'UTR	*LAMTOR3*
8	129824348	129824348	-	129824348	5	5	ENSSSCG00000028367	-	3'UTR	*DAPP1*
8	130036471	130036484	-	130036473	65	141	ENSSSCG00000009179	-	3'UTR	*MTTP*
8	130036681	130036681	-	130036681	3	3	ENSSSCG00000009179	-	3'UTR	*MTTP*
8	130037209	130037209	-	130037209	5	5	ENSSSCG00000009179	-	3'UTR	*MTTP*
8	130037359	130037359	-	130037359	3	3	ENSSSCG00000009179	-	3'UTR	*MTTP*
8	130037504	130037504	-	130037504	3	3	ENSSSCG00000009179	-	3'UTR	*MTTP*
8	130078360	130078360	-	130078360	6	6	ENSSSCG00000009179	-	ORF	*MTTP*
8	130079973	130079973	-	130079973	3	3	ENSSSCG00000009179	-	ORF	*MTTP*
8	130133410	130133418	+	130133411	7	20	ENSSSCG00000009180	+	3'UTR	---
8	130136914	130136914	-	130136914	5	5	---	---	intergenic	---
8	130138501	130138501	-	130138501	7	7	XLOC_085974	-	predicted	---
8	130185234	130185237	+	130185234	3	6	XLOC_085158	+	predicted	---

8	130234051	130234052	+	130234052	27	31	ENSSSCG00000030522	+	ORF	---
8	130236389	130236389	+	130236389	9	9	ENSSSCG00000030522	+	ORF	---
8	130236466	130236466	+	130236466	3	3	ENSSSCG00000030522	+	ORF	---
8	130240613	130240613	+	130240613	7	7	ENSSSCG00000030522	+	ORF	---
8	130242137	130242138	+	130242138	7	12	ENSSSCG00000030522	+	3'UTR	---
8	130242263	130242263	+	130242263	5	5	ENSSSCG00000030522	+	3'UTR	---
8	130242314	130242334	+	130242324	953	2766	ENSSSCG00000030522	+	3'UTR	---
8	130242546	130242551	+	130242551	6	11	ENSSSCG00000030522	+	3'UTR	---
8	130242838	130242838	+	130242838	6	6	XLOC_085158	+	predicted	---
8	130242904	130242904	+	130242904	3	3	XLOC_085158	+	predicted	---
8	130305381	130305381	-	130305381	5	5	---	---	intergenic	---
8	130305431	130305434	+	130305431	5	12	---	---	intergenic	---
8	130381341	130381341	-	130381341	6	6	---	---	intergenic	---
8	130434469	130434483	+	130434472	19	79	XLOC_085161	+	predicted	---
8	130434602	130434604	+	130434604	17	32	XLOC_085161	+	predicted	---
8	130446259	130446259	+	130446259	3	3	XLOC_085161	+	predicted	---
8	130466467	130466467	+	130466467	6	6	ENSSSCG00000009185	+	ORF	*ADH5*
8	130466901	130466906	+	130466901	25	88	ENSSSCG00000009185	+	3'UTR	*ADH5*
8	130476753	130476753	-	130476753	3	3	XLOC_085978	-	predicted	---
8	130646598	130646634	+	130646633	34	139	ENSSSCG00000028420	+	3'UTR	*EIF4E*
8	130647441	130647457	+	130647452	22	41	ENSSSCG00000028420	+	3'UTR	*EIF4E*
8	130649616	130649616	+	130649616	3	3	ENSSSCG00000028420	+	3'UTR	*EIF4E*
8	130667868	130667868	-	130667868	5	5	---	---	intergenic	---
8	130830714	130830714	-	130830714	3	3	---	---	intergenic	---
8	131052411	131052411	-	131052411	3	3	XLOC_085981	-	predicted	---
8	131053515	131053516	-	131053516	8	15	ENSSSCG00000022353	-	3'UTR	*RAP1GDS1*

8	131054633	131054644	-	131054635	96	194	ENSSSCG00000022353	-	3'UTR	*RAP1GDS1*
8	131054976	131055021	-	131055019	13	67	ENSSSCG00000022353	-	3'UTR	*RAP1GDS1*
8	131390557	131390557	-	131390557	5	5	---	---	intergenic	---
8	131430241	131430245	-	131430243	9	30	---	---	intergenic	---
8	131430261	131430261	+	131430261	5	5	---	---	intergenic	---
8	131698910	131698911	+	131698910	8	11	---	---	intergenic	---
8	131767857	131767857	-	131767857	10	10	---	---	intergenic	---
8	131824538	131824538	-	131824538	4	4	---	---	intergenic	---
8	132426466	132426466	+	132426466	7	7	---	---	intergenic	---
8	132721619	132721619	-	132721619	3	3	---	---	intergenic	---
8	132721753	132721754	+	132721753	3	6	---	---	intergenic	---
8	132788111	132788111	+	132788111	5	5	---	---	intergenic	---
8	132840313	132840313	-	132840313	5	5	---	---	intergenic	---
8	133092323	133092323	-	133092323	4	4	---	---	intergenic	---
8	133227798	133227798	-	133227798	6	6	---	---	intergenic	---
8	133370092	133370092	+	133370092	11	11	---	---	intergenic	---
8	133731819	133731819	+	133731819	3	3	---	---	intergenic	---
8	133845191	133845191	-	133845191	3	3	ENSSSCG00000029621	-	intron	*BMPR1B*
8	134280256	134280256	+	134280256	3	3	---	---	intergenic	---
8	134331214	134331222	-	134331217	8	25	ENSSSCG00000009192	-	3'UTR	*PDLIM5*
8	134333946	134333946	-	134333946	3	3	ENSSSCG00000009192	-	3'UTR	*PDLIM5*
8	134334405	134334405	-	134334405	4	4	ENSSSCG00000009192	-	3'UTR	*PDLIM5*
8	134406735	134406735	-	134406735	9	9	ENSSSCG00000009192	-	intron	*PDLIM5*
8	134706830	134706830	-	134706830	3	3	ENSSSCG00000009193	-	ORF	*SMARCAD1*
8	134756152	134756152	-	134756152	9	9	ENSSSCG00000009193	-	intron	*SMARCAD1*
8	134914422	134914422	-	134914422	3	3	XLOC_085995	-	predicted	---

8	135362317	135362327	+	135362323	4	10	---	---	intergenic	---
8	135407164	135407164	-	135407164	4	4	---	---	intergenic	---
8	135649653	135649653	-	135649653	3	3	---	---	intergenic	---
8	136703377	136703378	+	136703377	5	8	---	---	intergenic	---
8	136874425	136874425	+	136874425	3	3	---	---	intergenic	---
8	137251190	137251190	+	137251190	3	3	---	---	intergenic	---
8	137578089	137578089	-	137578089	3	3	---	---	intergenic	---
8	137812695	137812695	-	137812695	3	3	---	---	intergenic	---
8	138104609	138104609	-	138104609	3	3	ENSSSCG00000022986	-	intron	---
8	138282970	138282997	+	138282972	5	20	XLOC_085189	+	predicted	---
8	138333109	138333109	+	138333109	3	3	---	---	intergenic	---
8	138500976	138500977	-	138500976	3	6	ENSSSCG00000009202	-	3'UTR	*MMRN1*
8	139261381	139261381	-	139261381	9	9	---	---	intergenic	---
8	139307761	139307761	+	139307761	5	5	---	---	intergenic	---
8	139458375	139458375	-	139458375	9	9	---	---	intergenic	---
8	139551220	139551220	+	139551220	3	3	---	---	intergenic	---
8	139729863	139729863	+	139729863	16	16	ENSSSCG00000026196	+	intron	*FAM13A*
8	139735555	139735559	+	139735555	6	12	ENSSSCG00000026196	+	3'UTR	*FAM13A*
8	139756473	139756473	-	139756473	8	8	ENSSSCG00000009208	-	3'UTR	*HERC3*
8	139757934	139757934	-	139757934	3	3	ENSSSCG00000009208	-	3'UTR	*HERC3*
8	139845090	139845090	-	139845090	3	3	ENSSSCG00000009208	-	intron	*HERC3*
8	139943193	139943206	+	139943205	49	89	ENSSSCG00000009211	+	3'UTR	*PIGY*
8	139943284	139943287	+	139943287	28	38	ENSSSCG00000009211	+	3'UTR	*PIGY*
8	139947188	139947201	-	139947201	4	7	ENSSSCG00000030548	-	3'UTR	*HERC5*
8	139947302	139947310	-	139947303	6	15	ENSSSCG00000030548	-	3'UTR	*HERC5*
8	140136826	140136826	+	140136826	8	8	ENSSSCG00000009213	+	intron	*ABCG2*

8	140237551	140237552	-	140237552	106	113	ENSSSCG00000009217	-	intron	*PKD2*
8	140307933	140307939	-	140307939	51	60	ENSSSCG00000009216	-	3'UTR	*SPP1*
8	140628841	140628841	+	140628841	3	3	ENSSSCG00000009222	+	ORF	*SPARCL1*
8	140629046	140629046	+	140629046	5	5	ENSSSCG00000009222	+	ORF	*SPARCL1*
8	140643504	140643512	+	140643504	6	9	ENSSSCG00000009222	+	3'UTR	*SPARCL1*
8	140656078	140656082	-	140656079	26	61	ENSSSCG00000009223	-	3'UTR	*NUDT9*
8	140749330	140749341	+	140749339	132	279	XLOC_085207	+	predicted	---
8	140767776	140767777	+	140767777	8	13	ENSSSCG00000009225	+	intron	*HSD17B13*
8	140769085	140769104	+	140769104	4	7	ENSSSCG00000009225	+	ORF	*HSD17B13*
8	140776275	140776275	+	140776275	3	3	ENSSSCG00000009225	+	intron	*HSD17B13*
8	140777164	140777188	+	140777180	5	20	ENSSSCG00000009225	+	3'UTR	*HSD17B13*
8	140778223	140778230	+	140778223	13	20	ENSSSCG00000009225	+	3'UTR	*HSD17B13*
8	140868838	140868838	-	140868838	3	3	---	---	intergenic	---
8	141270197	141270197	-	141270197	12	12	---	---	intergenic	---
8	141298105	141298105	+	141298105	6	6	---	---	intergenic	---
8	141418293	141418293	-	141418293	4	4	ENSSSCG00000026655	-	ORF	*PTPN13*
8	141538730	141538730	-	141538730	9	9	ENSSSCG00000026655	-	intron	*PTPN13*
8	141940634	141940634	-	141940634	7	7	---	---	intergenic	---
8	142031405	142031405	+	142031405	3	3	ENSSSCG00000009228	+	intron	*MAPK10*
8	142055029	142055030	+	142055030	31	37	ENSSSCG00000009228	+	intron	*MAPK10*
8	142129695	142129695	+	142129695	3	3	ENSSSCG00000009228	+	intron	*MAPK10*
8	142142533	142142533	-	142142533	5	5	XLOC_086038	-	predicted	---
8	142360050	142360050	-	142360050	5	5	ENSSSCG00000009229	-	intron	*ARHGAP24*
8	143388427	143388428	+	143388427	5	8	ENSSSCG00000009230	+	intron	*WDFY3*
8	143417888	143417888	+	143417888	3	3	XLOC_085217	+	predicted	---
8	143433514	143433514	-	143433514	3	3	---	---	intergenic	---

8	143437502	143437502	-	143437502	4	4	XLOC_086041	-	predicted	---
8	143439807	143439810	-	143439807	3	6	ENSSSCG00000009231	-	3'UTR	*CDS1*
8	143969891	143969891	+	143969891	5	5	---	---	intergenic	---
8	144088042	144088042	-	144088042	3	3	---	---	intergenic	---
8	144151227	144151227	-	144151227	4	4	---	---	intergenic	---
8	144210527	144210530	-	144210529	22	38	ENSSSCG00000009233	-	3'UTR	*AGPAT9*
8	144211399	144211400	-	144211399	19	26	ENSSSCG00000009233	-	3'UTR	*AGPAT9*
8	144312313	144312313	+	144312313	4	4	ENSSSCG00000009234	+	intron	*FAM175A*
8	144316520	144316541	-	144316521	53	124	XLOC_086046	-	predicted	---
8	144417258	144417258	+	144417258	5	5	ENSSSCG00000009237	+	3'UTR	*HPSE*
8	144446382	144446403	+	144446384	10	23	XLOC_085223	+	predicted	---
8	144446515	144446515	+	144446515	3	3	XLOC_085223	+	predicted	---
8	144531004	144531005	+	144531004	14	18	ENSSSCG00000009239	+	3'UTR	---
8	144536764	144536765	+	144536765	39	44	---	---	intergenic	---
8	144573882	144573889	+	144573888	26	43	ENSSSCG00000009240	+	3'UTR	*PLAC8*
8	144573937	144573937	+	144573937	6	6	---	---	intergenic	---
8	144575310	144575340	-	144575318	19	30	ENSSSCG00000009241	-	3'UTR	*COPS4*
8	144581168	144581168	-	144581168	7	7	ENSSSCG00000009241	-	intron	*COPS4*
8	144596299	144596299	-	144596299	3	3	ENSSSCG00000009241	-	ORF	*COPS4*
8	144691547	144691547	+	144691547	3	3	ENSSSCG00000009242	+	3'UTR	*LIN54*
8	144711638	144711638	-	144711638	4	4	---	---	intergenic	---
8	144723594	144723599	+	144723594	7	11	XLOC_085228	+	predicted	---
8	144723637	144723642	+	144723637	14	23	XLOC_085228	+	predicted	---
8	144855573	144855581	+	144855579	79	112	ENSSSCG00000009244	+	3'UTR	*SEC31A*
8	144855817	144855824	+	144855824	14	30	XLOC_085228	+	predicted	---
8	145004014	145004014	+	145004014	4	4	ENSSSCG00000009245	+	3'UTR	*SCD5*

8	145136002	145136002	+	145136002	3	3	ENSSSCG00000026729	+	3'UTR	---
8	145145808	145145812	-	145145812	11	17	ENSSSCG00000009246	-	3'UTR	*ENOPH1*
8	145146722	145146722	-	145146722	3	3	ENSSSCG00000009246	-	3'UTR	*ENOPH1*
8	145172807	145172807	+	145172807	4	4	---	---	intergenic	---
8	145172890	145172890	+	145172890	4	4	ENSSSCG00000009247	+	intron	*HNRNPDL*
8	145176278	145176278	+	145176278	6	6	XLOC_085232	+	predicted	---
8	145177135	145177135	+	145177135	3	3	XLOC_085232	+	predicted	---
8	145178664	145178664	+	145178664	12	12	XLOC_085232	+	predicted	---
8	145195482	145195482	-	145195482	3	3	---	---	intergenic	---
8	145207819	145207819	-	145207819	3	3	XLOC_086060	-	predicted	---
8	145214995	145214995	+	145214995	4	4	ENSSSCG00000009249	+	ORF	*HNRNPD*
8	145218274	145218276	+	145218275	27	38	XLOC_085234	+	predicted	---
8	145218431	145218435	+	145218434	30	81	XLOC_085234	+	predicted	---
8	145227259	145227259	+	145227259	5	5	XLOC_085234	+	predicted	---
8	145463572	145463572	-	145463572	5	5	---	---	intergenic	---
8	145849572	145849572	+	145849572	5	5	ENSSSCG00000029507	+	intron	*RASGEF1B*
8	145859406	145859406	+	145859406	4	4	ENSSSCG00000029507	+	intron	*RASGEF1B*
8	145887684	145887684	+	145887684	9	9	ENSSSCG00000029507	+	3'UTR	*RASGEF1B*
8	145888768	145888771	+	145888771	24	30	ENSSSCG00000029507	+	3'UTR	*RASGEF1B*
8	145897864	145897864	-	145897864	3	3	---	---	intergenic	---
8	145965471	145965471	-	145965471	4	4	XLOC_086065	-	predicted	---
8	145982353	145982353	+	145982353	5	5	---	---	intergenic	---
8	146342137	146342137	+	146342137	4	4	---	---	intergenic	---
8	146372697	146372697	-	146372697	5	5	---	---	intergenic	---
8	146391746	146391746	+	146391746	4	4	XLOC_085244	+	predicted	---
8	146624576	146624576	+	146624576	10	10	ENSSSCG00000027817	+	intron	*C4orf22*

8	147020216	147020219	+	147020217	27	48	XLOC_085251	+	predicted	---
8	147411677	147411677	+	147411677	4	4	ENSSSCG00000009257	+	ORF	---
8	147412024	147412024	+	147412024	3	3	ENSSSCG00000009257	+	ORF	---
8	147412174	147412174	+	147412174	3	3	ENSSSCG00000009257	+	ORF	---
8	147412348	147412370	+	147412350	133	497	ENSSSCG00000009257	+	3'UTR	*GK2*
							ENSSSCG00000009257	+	ORF	---
8	147546603	147546603	+	147546603	6	6	---	---	intergenic	---
8	147664468	147664476	-	147664468	4	7	XLOC_086080	-	predicted	---
8	147697177	147697177	-	147697177	3	3	---	---	intergenic	---
8	147810237	147810237	+	147810237	4	4	ENSSSCG00000009259	+	3'UTR	*PAQR3*
8	147812301	147812301	+	147812301	3	3	ENSSSCG00000009259	+	3'UTR	*PAQR3*
8	147812552	147812552	+	147812552	4	4	ENSSSCG00000009259	+	3'UTR	*PAQR3*
8	147819079	147819079	-	147819079	3	3	XLOC_086081	-	predicted	---
8	147934614	147934614	-	147934614	4	4	---	---	intergenic	---
8	147975237	147975237	-	147975237	5	5	---	---	intergenic	---
8	148030991	148030991	+	148030991	8	8	ENSSSCG00000027722	+	intron	*BMP2K*
8	148222497	148222499	+	148222499	6	9	ENSSSCG00000009262	+	3'UTR	*CSN1S1*
8	148228435	148228435	-	148228435	10	10	ENSSSCG00000009261	-	3'UTR	*CSN2*
8	148326956	148326956	+	148326956	5	5	ENSSSCG00000009263	+	3'UTR	*CSN1S2*
8	148343351	148343351	+	148343351	3	3	ENSSSCG00000009264	+	ORF	*PRR27*
8	148347337	148347337	+	148347337	3	3	---	---	intergenic	---
9	1395	1395	+	1395	3	3	---	---	intergenic	---
9	47661	47665	+	47665	12	15	ENSSSCG00000028886	+	3'UTR	*IPO7*
9	114426	114427	+	114426	4	7	ENSSSCG00000022070	+	3'UTR	*ZNF143*
9	114984	114984	+	114984	3	3	XLOC_089166	+	predicted	---
9	265504	265504	+	265504	4	4	ENSSSCG00000014579	+	3'UTR	---

9	265715	265718	+	265715	4	8	ENSSSCG00000014579	+	3'UTR	---
9	295134	295136	-	295134	14	26	---	---	intergenic	---
9	349792	349793	+	349793	12	16	---	---	intergenic	---
9	400832	400832	+	400832	11	11	ENSSSCG00000014578	+	intron	*DENND5A*
9	426207	426208	+	426208	27	31	XLOC_089171	+	predicted	---
9	551773	551774	+	551773	3	6	ENSSSCG00000014570	+	3'UTR	*NRIP3*
9	554438	554438	+	554438	3	3	ENSSSCG00000014570	+	3'UTR	*NRIP3*
9	578709	578709	+	578709	4	4	ENSSSCG00000014577	+	3'UTR	*TMEM9B*
9	579597	579602	+	579598	70	121	ENSSSCG00000014577	+	3'UTR	*TMEM9B*
9	599972	599977	-	599977	12	21	ENSSSCG00000014574	-	3'UTR	*AKIP1*
9	873500	873500	+	873500	4	4	---	---	intergenic	---
9	904457	904457	-	904457	5	5	XLOC_090389	-	predicted	---
9	909113	909113	-	909113	4	4	XLOC_090389	-	predicted	---
9	1137475	1137475	-	1137475	3	3	XLOC_090393	-	predicted	---
9	1143471	1143492	+	1143477	32	86	XLOC_089181	+	predicted	---
9	1522118	1522118	+	1522118	4	4	XLOC_089185	+	predicted	---
9	1651003	1651003	-	1651003	3	3	XLOC_090397	-	predicted	---
9	1654820	1654820	-	1654820	6	6	---	---	intergenic	---
9	2059288	2059288	-	2059288	4	4	---	---	intergenic	---
9	2269304	2269304	+	2269304	6	6	---	---	intergenic	---
9	2587266	2587272	-	2587267	6	25	XLOC_090409	-	predicted	---
9	2674966	2674967	-	2674966	6	9	XLOC_090416	-	predicted	---
9	2930790	2930790	+	2930790	3	3	---	---	intergenic	---
9	3041732	3041752	+	3041752	6	12	XLOC_089202	+	predicted	---
9	3041815	3041846	+	3041843	25	79	XLOC_089202	+	predicted	---
9	3042806	3042806	+	3042806	13	13	XLOC_089202	+	predicted	---

9	3090081	3090098	-	3090098	4	7	---	---	intergenic	---
9	3393012	3393012	-	3393012	23	23	---	---	intergenic	---
9	3735085	3735114	+	3735106	98	237	ENSSSCG00000024693	+	3'UTR	*MRPL17*
9	3735185	3735214	+	3735213	27	64	ENSSSCG00000024693	+	3'UTR	*MRPL17*
9	3799103	3799103	+	3799103	4	4	ENSSSCG00000023080	+	3'UTR	*TPP1*
9	3799207	3799207	+	3799207	3	3	ENSSSCG00000023080	+	3'UTR	*TPP1*
9	3800627	3800637	+	3800629	28	70	ENSSSCG00000023080	+	3'UTR	*TPP1*
9	3803437	3803439	-	3803438	60	70	ENSSSCG00000023272	-	3'UTR	*ILK*
9	3803458	3803486	+	3803462	35	54	ENSSSCG00000029630	+	3'UTR	*TAF10*
9	3814829	3814831	+	3814831	4	7	ENSSSCG00000023880	+	3'UTR	*RRP8*
9	3830430	3830431	-	3830431	5	8	XLOC_090431	-	predicted	---
9	3907123	3907123	-	3907123	3	3	XLOC_090431	-	predicted	---
9	3907484	3907500	-	3907494	45	149	XLOC_090431	-	predicted	---
9	3913408	3913408	+	3913408	6	6	ENSSSCG00000014624	+	3'UTR	*ARFIP2*
9	3946811	3946811	+	3946811	3	3	ENSSSCG00000014626	+	ORF	*HPX*
9	3947462	3947462	+	3947462	10	10	ENSSSCG00000014626	+	ORF	*HPX*
9	3949164	3949164	+	3949164	10	10	ENSSSCG00000014626	+	ORF	*HPX*
9	3949932	3949932	+	3949932	4	4	ENSSSCG00000014626	+	ORF	*HPX*
9	3954643	3954643	+	3954643	3	3	ENSSSCG00000014626	+	ORF	*HPX*
9	3954965	3954965	+	3954965	11	11	ENSSSCG00000014626	+	ORF	*HPX*
9	3955207	3955278	+	3955256	2670	4207	XLOC_089222	+	predicted	---
9	3964415	3964421	+	3964415	38	53	ENSSSCG00000014627	+	3'UTR	*SMPD1*
9	3964690	3964692	-	3964692	4	7	ENSSSCG00000014628	-	3'UTR	*APBB1*
9	3991704	3991704	-	3991704	17	17	ENSSSCG00000014633	-	3'UTR	*PRKCDBP*
9	3991886	3991886	-	3991886	3	3	ENSSSCG00000014633	-	3'UTR	*PRKCDBP*
9	4079421	4079421	+	4079421	3	3	XLOC_089226	+	predicted	---

9	4088267	4088272	+	4088270	32	62	XLOC_089226	+	predicted	---
9	4138934	4138934	+	4138934	4	4	XLOC_089228	+	predicted	---
9	4359867	4359867	-	4359867	3	3	XLOC_090452	-	predicted	---
9	4369120	4369120	-	4369120	3	3	---	---	intergenic	---
9	4375199	4375199	-	4375199	3	3	ENSSSCG00000014650	-	ORF	*OR52E4*
9	4375480	4375491	-	4375486	29	61	ENSSSCG00000014650	-	ORF	*OR52E4*
9	4720185	4720191	+	4720191	6	10	ENSSSCG00000014672	+	3'UTR	*TRIM6*
9	4737187	4737189	+	4737188	23	35	ENSSSCG00000014670	+	3'UTR	*TRIM5*
9	4897147	4897161	+	4897154	116	363	XLOC_089251	+	predicted	---
9	4902090	4902095	+	4902093	209	408	XLOC_089251	+	predicted	---
9	5422968	5422968	+	5422968	3	3	---	---	intergenic	---
9	5426461	5426474	+	5426470	4	11	---	---	intergenic	---
9	5426487	5426500	-	5426493	4	10	---	---	intergenic	---
9	5426536	5426536	+	5426536	7	7	---	---	intergenic	---
9	5426564	5426564	-	5426564	5	5	---	---	intergenic	---
9	5426592	5426605	+	5426592	3	6	---	---	intergenic	---
9	5426603	5426604	-	5426603	3	6	---	---	intergenic	---
9	5426671	5426742	+	5426725	1517	2129	---	---	intergenic	---
9	5426872	5426872	-	5426872	3	3	---	---	intergenic	---
9	5426979	5426981	-	5426981	4	7	---	---	intergenic	---
9	5427103	5427109	+	5427108	183	190	---	---	intergenic	---
9	5427179	5427204	-	5427179	5	15	---	---	intergenic	---
9	5427213	5427213	+	5427213	3	3	---	---	intergenic	---
9	5427252	5427286	+	5427286	18	33	---	---	intergenic	---
9	5632712	5632719	-	5632715	135	150	ENSSSCG00000014725	-	3'UTR	*HBB*
9	5632877	5632877	-	5632877	3	3	ENSSSCG00000014725	-	3'UTR	*HBB*

9	5632970	5633065	-	5632982	12980	18830	ENSSSCG00000014725	-	3'UTR	*HBB*
9	5650511	5650512	-	5650511	18	21	---	---	intergenic	---
9	6146006	6146006	+	6146006	4	4	XLOC_089286	+	predicted	---
9	6818977	6818983	-	6818982	33	51	ENSSSCG00000014791	-	3'UTR	---
9	6848913	6848919	-	6848919	7	13	ENSSSCG00000014792	-	3'UTR	*STIM1*
9	6854470	6854472	-	6854472	4	7	ENSSSCG00000014792	-	intron	*STIM1*
9	6859415	6859415	-	6859415	3	3	ENSSSCG00000014792	-	intron	*STIM1*
9	6956129	6956129	-	6956129	5	5	ENSSSCG00000014792	-	intron	*STIM1*
9	7151212	7151212	+	7151212	6	6	ENSSSCG00000014794	+	intron	*NUP98*
9	7179719	7179719	+	7179719	3	3	ENSSSCG00000014794	+	3'UTR	*NUP98*
9	7180629	7180630	+	7180629	7	12	ENSSSCG00000014794	+	3'UTR	*NUP98*
9	7323654	7323654	+	7323654	3	3	ENSSSCG00000014800	+	intron	*RNF121*
9	7347960	7347960	+	7347960	6	6	ENSSSCG00000014800	+	3'UTR	*RNF121*
9	7353200	7353200	+	7353200	7	7	ENSSSCG00000023796	+	3'UTR	*IL18BP*
							ENSSSCG00000023796	+	intron	*IL18BP*
							ENSSSCG00000023796	+	ORF	*IL18BP*
9	7353401	7353404	-	7353401	30	54	XLOC_090547	-	predicted	---
9	7426945	7426945	+	7426945	4	4	ENSSSCG00000014803	+	intron	---
9	7428339	7428352	+	7428349	17	100	ENSSSCG00000014803	+	intron	---
9	7434739	7434768	+	7434763	249	702	ENSSSCG00000014803	+	3'UTR	---
9	7435534	7435541	-	7435538	20	53	ENSSSCG00000014804	-	3'UTR	*LAMTOR1*
9	7447425	7447438	-	7447427	33	135	ENSSSCG00000014806	-	3'UTR	*ANAPC15*
9	7465154	7465154	-	7465154	3	3	XLOC_090550	-	predicted	---
9	7495579	7495581	+	7495581	53	64	---	---	intergenic	---
9	7499704	7499704	-	7499704	4	4	XLOC_090552	-	predicted	---
9	7511898	7511924	+	7511917	177	669	XLOC_089304	+	predicted	---

9	7551953	7551963	+	7551953	90	347	XLOC_089306	+	predicted	---
9	7570170	7570176	+	7570170	7	19	ENSSSCG00000014811	+	3'UTR	*INPPL1*
9	7599253	7599256	-	7599254	15	40	ENSSSCG00000025600	-	3'UTR	---
9	7601827	7601837	-	7601831	55	117	ENSSSCG00000025600	-	3'UTR	---
9	7998475	7998475	-	7998475	14	14	ENSSSCG00000014817	-	3'UTR	*ARAP1*
9	8064135	8064139	-	8064137	33	53	ENSSSCG00000014818	-	3'UTR	*STARD10*
9	8101198	8101199	+	8101198	8	11	XLOC_089313	+	predicted	---
9	8122145	8122145	+	8122145	5	5	ENSSSCG00000014819	+	3'UTR	*ATG16L2*
9	8128490	8128495	-	8128491	5	12	XLOC_090562	-	predicted	---
9	8506210	8506210	+	8506210	11	11	ENSSSCG00000014825	+	intron	*RELT*
9	8591693	8591693	+	8591693	4	4	ENSSSCG00000014822	+	intron	---
9	8856603	8856603	-	8856603	8	8	ENSSSCG00000014827	-	3'UTR	*PLEKHB1*
9	8857385	8857385	-	8857385	7	7	ENSSSCG00000014827	-	3'UTR	*PLEKHB1*
9	8885636	8885636	+	8885636	4	4	---	---	intergenic	---
9	8897354	8897356	+	8897354	4	8	XLOC_089320	+	predicted	---
9	8926789	8926798	-	8926791	21	71	ENSSSCG00000014828	-	3'UTR	---
9	9031029	9031032	+	9031032	16	22	ENSSSCG00000014829	+	intron	*MRPL48*
9	9056078	9056082	+	9056080	38	84	ENSSSCG00000014829	+	3'UTR	*MRPL48*
9	9067599	9067606	-	9067606	8	12	XLOC_090575	-	predicted	---
9	9123967	9123980	+	9123974	13	37	ENSSSCG00000014831	+	3'UTR	*PAAF1*
9	9144855	9144865	+	9144855	12	24	ENSSSCG00000014832	+	3'UTR	*DNAJB13*
9	9147995	9147996	-	9147995	48	54	ENSSSCG00000014833	-	3'UTR	*UCP2*
9	9167480	9167487	-	9167487	4	7	ENSSSCG00000014834	-	3'UTR	*UCP3*
9	9203037	9203037	-	9203037	4	4	ENSSSCG00000014835	-	intron	*C2CD3*
9	9300216	9300218	-	9300216	3	6	ENSSSCG00000014835	-	intron	*C2CD3*
9	9316273	9316273	-	9316273	3	3	ENSSSCG00000014835	-	intron	*C2CD3*

9	9341467	9341467	-	9341467	3	3	ENSSSCG00000014835	-	intron	*C2CD3*
9	9442710	9442712	+	9442711	18	25	ENSSSCG00000020856	+	3'UTR	*PPME1*
9	9443415	9443426	+	9443422	20	52	ENSSSCG00000020856	+	3'UTR	*PPME1*
9	9531683	9531683	-	9531683	4	4	XLOC_090579	-	predicted	---
9	10070170	10070175	-	10070172	55	93	XLOC_090584	-	predicted	---
9	10396535	10396535	+	10396535	3	3	ENSSSCG00000014850	+	3'UTR	*SPCS2*
9	10397048	10397048	+	10397048	3	3	ENSSSCG00000014850	+	3'UTR	*SPCS2*
9	10397107	10397114	+	10397108	31	47	ENSSSCG00000014850	+	3'UTR	*SPCS2*
9	10397237	10397237	+	10397237	6	6	ENSSSCG00000014850	+	3'UTR	*SPCS2*
9	10401948	10401950	-	10401948	8	15	XLOC_090587	-	predicted	---
9	10692648	10692671	-	10692671	23	68	ENSSSCG00000014851	-	3'UTR	*SLCO2B1*
9	10765288	10765291	+	10765288	4	11	XLOC_089347	+	predicted	---
9	10818034	10818034	+	10818034	16	16	ENSSSCG00000014855	+	ORF	*RPS3*
9	10818612	10818621	+	10818613	444	581	XLOC_089351	+	predicted	---
9	10842154	10842155	-	10842154	3	6	ENSSSCG00000014853	-	3'UTR	*GDPD5*
9	10935532	10935532	-	10935532	3	3	XLOC_090598	-	predicted	---
9	10938393	10938393	-	10938393	4	4	XLOC_090598	-	predicted	---
9	10940777	10940800	+	10940784	16	35	---	---	intergenic	---
9	10977245	10977248	+	10977247	27	47	XLOC_089355	+	predicted	---
9	10993231	10993231	-	10993231	3	3	XLOC_090600	-	predicted	---
9	11120965	11120965	+	11120965	13	13	ENSSSCG00000014861	+	intron	*MOGAT2*
9	11155520	11155520	-	11155520	4	4	---	---	intergenic	---
9	11405171	11405171	+	11405171	3	3	ENSSSCG00000028979	+	intron	*UVRAG*
9	11651436	11651436	-	11651436	12	12	XLOC_090604	-	predicted	---
9	11651780	11651780	-	11651780	3	3	XLOC_090604	-	predicted	---
9	11674109	11674109	+	11674109	60	60	---	---	intergenic	---

9	11730924	11730924	-	11730924	3	3	---	---	intergenic	---
9	11826497	11826497	+	11826497	3	3	XLOC_089368	+	predicted	---
9	11913109	11913112	-	11913109	3	6	ENSSSCG00000014869	-	3'UTR	*LRRC32*
9	12013505	12013508	+	12013506	153	179	---	---	intergenic	---
9	12050295	12050314	+	12050314	10	19	XLOC_089376	+	predicted	---
9	12064719	12064719	-	12064719	4	4	---	---	intergenic	---
9	12187258	12187259	+	12187259	8	11	XLOC_089377	+	predicted	---
9	12187417	12187425	+	12187425	7	18	XLOC_089377	+	predicted	---
9	12189202	12189202	+	12189202	3	3	XLOC_089377	+	predicted	---
9	12190485	12190485	+	12190485	3	3	XLOC_089377	+	predicted	---
9	12195455	12195455	-	12195455	4	4	---	---	intergenic	---
9	12341060	12341060	+	12341060	4	4	ENSSSCG00000014875	+	3'UTR	*CAPN5*
9	12373439	12373439	+	12373439	3	3	ENSSSCG00000014876	+	ORF	*MYO7A*
9	12431451	12431456	+	12431453	4	14	XLOC_089382	+	predicted	---
9	12572278	12572278	-	12572278	11	11	XLOC_090614	-	predicted	---
9	12578199	12578207	-	12578199	7	13	---	---	intergenic	---
9	12578534	12578534	-	12578534	5	5	XLOC_090613	-	predicted	---
9	12754190	12754190	+	12754190	5	5	XLOC_089383	+	predicted	---
9	12979554	12979554	-	12979554	18	18	ENSSSCG00000014878	-	3'UTR	*PAK1*
9	12980718	12980718	-	12980718	6	6	ENSSSCG00000014878	-	3'UTR	*PAK1*
9	13158311	13158311	+	13158311	3	3	---	---	intergenic	---
9	13228451	13228489	+	13228483	20	55	ENSSSCG00000014880	+	3'UTR	*AQP11*
9	13245175	13245186	-	13245183	21	66	ENSSSCG00000014881	-	3'UTR	*CLNS1A*
9	13270083	13270086	+	13270083	7	10	---	---	intergenic	---
9	13336154	13336164	-	13336164	4	7	ENSSSCG00000014882	-	ORF	*RSF1*
9	13643438	13643450	+	13643438	53	141	ENSSSCG00000014883	+	3'UTR	*AAMDC*

9	13796326	13796326	-	13796326	4	4	XLOC_090624	-	predicted	---
9	13821219	13821226	-	13821224	14	25	ENSSSCG00000014889	-	3'UTR	*NDUFC2*
9	13934121	13934125	+	13934121	4	8	---	---	intergenic	---
9	14221679	14221679	-	14221679	3	3	ENSSSCG00000014893	-	intron	*NARS2*
9	14324410	14324410	-	14324410	3	3	XLOC_090633	-	predicted	---
9	14839943	14839943	-	14839943	3	3	---	---	intergenic	---
9	14893727	14893727	-	14893727	3	3	---	---	intergenic	---
9	15435227	15435227	-	15435227	3	3	XLOC_090636	-	predicted	---
9	15558467	15558469	-	15558469	9	12	---	---	intergenic	---
9	16068432	16068432	-	16068432	14	14	---	---	intergenic	---
9	16522838	16522838	+	16522838	4	4	---	---	intergenic	---
9	16793909	16793909	+	16793909	3	3	XLOC_089399	+	predicted	---
9	16943353	16943353	+	16943353	4	4	---	---	intergenic	---
9	17194718	17194718	+	17194718	4	4	---	---	intergenic	---
9	17797083	17797083	-	17797083	7	7	---	---	intergenic	---
9	18410095	18410095	+	18410095	4	4	---	---	intergenic	---
9	18706506	18706506	-	18706506	4	4	---	---	intergenic	---
9	18857829	18857829	+	18857829	8	8	---	---	intergenic	---
9	18972507	18972510	+	18972509	59	120	ENSSSCG00000014899	+	3'UTR	*PRCP*
9	18984768	18984768	-	18984768	4	4	---	---	intergenic	---
9	19106182	19106182	+	19106182	6	6	---	---	intergenic	---
9	19238051	19238051	+	19238051	3	3	ENSSSCG00000014901	+	3'UTR	*PCF11*
9	19238637	19238638	+	19238637	7	12	ENSSSCG00000014901	+	3'UTR	*PCF11*
9	19300147	19300147	+	19300147	3	3	ENSSSCG00000014902	+	intron	*ANKRD42*
9	19320157	19320157	+	19320157	3	3	ENSSSCG00000014902	+	3'UTR	*ANKRD42*
9	19320305	19320305	+	19320305	3	3	XLOC_089407	+	predicted	---

9	19328883	19328883	-	19328883	3	3	ENSSSCG00000014903	-	3'UTR	*CCDC90B*
9	19330629	19330629	-	19330629	3	3	ENSSSCG00000014903	-	3'UTR	*CCDC90B*
9	19338367	19338367	-	19338367	6	6	ENSSSCG00000014903	-	ORF	*CCDC90B*
9	20027965	20027965	-	20027965	3	3	---	---	intergenic	---
9	20443481	20443481	-	20443481	3	3	---	---	intergenic	---
9	20487554	20487555	+	20487555	10	13	---	---	intergenic	---
9	20579838	20579839	-	20579839	75	100	---	---	intergenic	---
9	21520014	21520014	+	21520014	40	40	---	---	intergenic	---
9	21855408	21855408	+	21855408	37	37	ENSSSCG00000025187	+	3'UTR	*TMEM126A*
9	21861146	21861146	-	21861146	3	3	XLOC_090660	-	predicted	---
9	21861823	21861823	-	21861823	3	3	XLOC_090660	-	predicted	---
9	21877772	21877780	-	21877779	49	83	XLOC_090661	-	predicted	---
9	21877867	21877867	-	21877867	4	4	XLOC_090661	-	predicted	---
9	22007916	22007919	-	22007916	3	6	---	---	intergenic	---
9	22034350	22034350	-	22034350	3	3	---	---	intergenic	---
9	22034446	22034446	-	22034446	13	13	---	---	intergenic	---
9	22044877	22044880	-	22044880	33	40	ENSSSCG00000014910	-	ORF	---
9	22045242	22045244	+	22045242	5	10	XLOC_089418	+	predicted	---
9	22060951	22060951	+	22060951	6	6	ENSSSCG00000014912	+	ORF	*CCDC83*
9	22087320	22087338	+	22087328	100	176	XLOC_089419	+	predicted	---
9	22107104	22107104	-	22107104	9	9	XLOC_090666	-	predicted	---
9	22107616	22107636	-	22107634	82	151	ENSSSCG00000014913	-	3'UTR	*PICALM*
9	22108741	22108792	-	22108785	13	35	ENSSSCG00000014913	-	3'UTR	*PICALM*
9	22108911	22108911	-	22108911	3	3	ENSSSCG00000014913	-	3'UTR	*PICALM*
9	22124718	22124719	-	22124719	22	34	ENSSSCG00000014913	-	intron	*PICALM*
9	22163916	22163933	-	22163933	17	20	ENSSSCG00000014913	-	intron	*PICALM*

9	22268766	22268767	+	22268766	5	8	XLOC_089422	+	predicted	---
9	22378038	22378038	-	22378038	3	3	---	---	intergenic	---
9	22382627	22382627	-	22382627	7	7	ENSSSCG00000014915	-	3'UTR	---
9	22493491	22493501	-	22493501	9	17	ENSSSCG00000021210	-	3'UTR	---
9	22493753	22493764	-	22493759	11	25	ENSSSCG00000021210	-	3'UTR	---
9	22571528	22571528	+	22571528	3	3	ENSSSCG00000014917	+	3'UTR	*CCDC81*
9	22571639	22571640	+	22571639	16	20	ENSSSCG00000014917	+	3'UTR	*CCDC81*
9	22583343	22583371	+	22583345	84	201	XLOC_089424	+	predicted	---
9	22583620	22583631	+	22583620	7	14	XLOC_089424	+	predicted	---
9	22583827	22583827	+	22583827	3	3	XLOC_089424	+	predicted	---
9	22589632	22589633	-	22589632	18	23	ENSSSCG00000014919	-	3'UTR	*ME3*
9	22912464	22912464	+	22912464	3	3	ENSSSCG00000014921	+	ORF	---
9	22913015	22913016	+	22913016	16	20	ENSSSCG00000014921	+	3'UTR	*PRSS23*
							ENSSSCG00000014921	+	ORF	---
9	22997809	22997812	-	22997810	21	37	ENSSSCG00000014920	-	3'UTR	*FZD4*
9	23385502	23385503	-	23385502	19	30	---	---	intergenic	---
9	23522249	23522249	-	23522249	3	3	---	---	intergenic	---
9	23717698	23717698	-	23717698	4	4	---	---	intergenic	---
9	23739367	23739369	+	23739367	10	21	---	---	intergenic	---
9	24007973	24007973	-	24007973	3	3	---	---	intergenic	---
9	24081768	24081777	-	24081776	89	110	ENSSSCG00000014924	-	3'UTR	*CTSC*
9	24104341	24104357	-	24104351	23	56	ENSSSCG00000014924	-	intron	*CTSC*
9	24116308	24116311	-	24116309	9	21	ENSSSCG00000014924	-	intron	*CTSC*
9	24182249	24182253	+	24182251	26	50	---	---	intergenic	---
9	24291626	24291626	+	24291626	3	3	ENSSSCG00000029865	+	intron	---
9	24521657	24521657	-	24521657	3	3	ENSSSCG00000021425	-	intron	---

9	24813489	24813489	-	24813489	5	5	---	---	intergenic	---
9	25073250	25073250	-	25073250	4	4	---	---	intergenic	---
9	25214565	25214565	+	25214565	9	9	XLOC_089439	+	predicted	---
9	25214655	25214660	+	25214660	7	11	XLOC_089439	+	predicted	---
9	25239052	25239060	-	25239054	16	29	ENSSSCG00000014927	-	3'UTR	*NOX4*
9	25239467	25239467	-	25239467	3	3	ENSSSCG00000014927	-	ORF	---
9	25687721	25687721	+	25687721	3	3	ENSSSCG00000027093	+	3'UTR	*FOLH1*
9	25772071	25772071	-	25772071	3	3	---	---	intergenic	---
9	25963587	25963587	-	25963587	5	5	---	---	intergenic	---
9	25963649	25963649	-	25963649	3	3	---	---	intergenic	---
9	25966340	25966340	-	25966340	6	6	---	---	intergenic	---
9	25967180	25967186	-	25967180	22	28	---	---	intergenic	---
9	26075015	26075015	-	26075015	3	3	---	---	intergenic	---
9	26122526	26122526	+	26122526	3	3	---	---	intergenic	---
9	26367147	26367159	-	26367158	7	23	---	---	intergenic	---
9	26433603	26433603	-	26433603	4	4	---	---	intergenic	---
9	26481022	26481022	-	26481022	5	5	---	---	intergenic	---
9	27142268	27142268	+	27142268	3	3	---	---	intergenic	---
9	27454807	27454807	+	27454807	3	3	---	---	intergenic	---
9	27601621	27601621	+	27601621	4	4	---	---	intergenic	---
9	28554797	28554797	+	28554797	4	4	ENSSSCG00000014935	+	intron	*FAT3*
9	28891073	28891073	-	28891073	3	3	XLOC_090694	-	predicted	---
9	29289357	29289359	+	29289358	9	17	---	---	intergenic	---
9	29373293	29373293	+	29373293	4	4	XLOC_089463	+	predicted	---
9	29519236	29519241	-	29519238	41	84	XLOC_090696	-	predicted	---
9	29519575	29519586	-	29519579	7	28	XLOC_090696	-	predicted	---

9	29666357	29666376	-	29666357	11	17	---	---	intergenic	---
9	29666571	29666580	-	29666579	14	20	---	---	intergenic	---
9	29683485	29683485	+	29683485	5	5	XLOC_089468	+	predicted	---
9	29689105	29689105	-	29689105	5	5	---	---	intergenic	---
9	29689256	29689260	+	29689260	11	23	XLOC_089468	+	predicted	---
9	29820981	29820981	+	29820981	3	3	ENSSSCG00000014948	+	3'UTR	*C11orf54*
9	29821063	29821063	+	29821063	5	5	ENSSSCG00000014948	+	3'UTR	*C11orf54*
9	29821178	29821186	+	29821180	7	21	ENSSSCG00000014948	+	3'UTR	*C11orf54*
9	29847750	29847750	+	29847750	3	3	ENSSSCG00000014949	+	3'UTR	*MED17*
9	29855300	29855300	-	29855300	5	5	XLOC_090701	-	predicted	---
9	30186891	30186891	+	30186891	4	4	XLOC_089477	+	predicted	---
9	30305059	30305059	-	30305059	3	3	ENSSSCG00000022490	-	3'UTR	*GPR83*
9	30328979	30328979	-	30328979	5	5	XLOC_090705	-	predicted	---
9	30331998	30331998	-	30331998	4	4	ENSSSCG00000014955	-	3'UTR	*MRE11A*
9	30395684	30395684	+	30395684	4	4	ENSSSCG00000014956	+	3'UTR	*ANKRD49*
9	30603834	30603834	+	30603834	5	5	---	---	intergenic	---
9	30604006	30604007	+	30604006	22	30	---	---	intergenic	---
9	30645248	30645249	+	30645249	9	13	---	---	intergenic	---
9	30727223	30727224	+	30727223	4	8	XLOC_089483	+	predicted	---
9	30732223	30732223	+	30732223	6	6	XLOC_089483	+	predicted	---
9	30760476	30760477	+	30760476	3	6	XLOC_089484	+	predicted	---
9	30788882	30788890	-	30788883	20	34	ENSSSCG00000014961	-	3'UTR	*CWC15*
9	30789305	30789305	-	30789305	3	3	ENSSSCG00000014961	-	3'UTR	*CWC15*
9	30789364	30789373	-	30789373	11	20	ENSSSCG00000014961	-	3'UTR	*CWC15*
9	30789610	30789617	-	30789610	15	20	ENSSSCG00000014961	-	3'UTR	*CWC15*
9	31023164	31023165	+	31023164	3	6	---	---	intergenic	---

9	31076104	31076104	+	31076104	5	5	XLOC_089488	+	predicted	---
9	31117161	31117168	+	31117168	7	10	---	---	intergenic	---
9	31339369	31339369	+	31339369	3	3	---	---	intergenic	---
9	31821166	31821166	-	31821166	3	3	ENSSSCG00000014969	-	ORF	---
9	31840088	31840091	+	31840088	5	8	XLOC_089492	+	predicted	---
9	31840380	31840390	+	31840388	9	32	XLOC_089492	+	predicted	---
9	31840564	31840571	+	31840565	8	24	XLOC_089492	+	predicted	---
9	31841060	31841060	+	31841060	3	3	XLOC_089492	+	predicted	---
9	31847126	31847135	-	31847127	25	48	ENSSSCG00000014970	-	3'UTR	*MTMR2*
9	31946238	31946239	+	31946239	20	29	---	---	intergenic	---
9	32156603	32156603	-	32156603	6	6	---	---	intergenic	---
9	32213404	32213417	+	32213404	7	26	XLOC_089496	+	predicted	---
9	32328364	32328388	-	32328365	11	30	XLOC_090724	-	predicted	---
9	32410762	32410771	-	32410762	6	17	ENSSSCG00000014972	-	3'UTR	*CCDC82*
9	32414631	32414631	-	32414631	4	4	ENSSSCG00000014972	-	ORF	*CCDC82*
9	32468142	32468142	-	32468142	3	3	---	---	intergenic	---
9	32577167	32577167	+	32577167	3	3	---	---	intergenic	---
9	32665213	32665213	-	32665213	5	5	---	---	intergenic	---
9	32705437	32705437	-	32705437	3	3	---	---	intergenic	---
9	33403424	33403424	+	33403424	3	3	---	---	intergenic	---
9	34574506	34574506	-	34574506	3	3	---	---	intergenic	---
9	34592968	34592968	+	34592968	15	15	---	---	intergenic	---
9	35261393	35261393	+	35261393	21	21	ENSSSCG00000014975	+	intron	*CNTN5*
9	35437406	35437406	+	35437406	6	6	ENSSSCG00000014975	+	intron	*CNTN5*
9	36008357	36008357	+	36008357	3	3	XLOC_089504	+	predicted	---
9	36165014	36165015	-	36165014	17	21	---	---	intergenic	---

9	36273407	36273407	-	36273407	3	3	ENSSSCG00000024022	-	ORF	*TRPC6*
9	36379710	36379710	-	36379710	4	4	---	---	intergenic	---
9	36403253	36403253	-	36403253	3	3	---	---	intergenic	---
9	36559029	36559029	-	36559029	3	3	XLOC_090735	-	predicted	---
9	36624078	36624078	+	36624078	5	5	ENSSSCG00000014979	+	intron	*CEP126*
9	36663856	36663856	+	36663856	3	3	XLOC_089507	+	predicted	---
9	36666000	36666001	-	36666000	6	10	---	---	intergenic	---
9	36693431	36693431	+	36693431	3	3	---	---	intergenic	---
9	36725781	36725781	+	36725781	4	4	ENSSSCG00000014980	+	intron	*C11orf70*
9	36744683	36744707	+	36744688	33	132	ENSSSCG00000014980	+	3'UTR	*C11orf70*
9	36858774	36858774	+	36858774	3	3	ENSSSCG00000025396	+	intron	*YAP1*
9	36870301	36870301	+	36870301	3	3	ENSSSCG00000025396	+	3'UTR	*YAP1*
9	36872903	36872903	+	36872903	4	4	ENSSSCG00000025396	+	3'UTR	*YAP1*
9	36873586	36873586	+	36873586	3	3	XLOC_089508	+	predicted	---
9	36913321	36913321	+	36913321	4	4	---	---	intergenic	---
9	36956660	36956660	+	36956660	3	3	XLOC_089511	+	predicted	---
9	36969101	36969101	+	36969101	28	28	XLOC_089511	+	predicted	---
9	36992651	36992658	+	36992658	8	12	---	---	intergenic	---
9	37009463	37009473	+	37009465	15	30	ENSSSCG00000027529	+	3'UTR	*BIRC2*
9	37031464	37031464	-	37031464	3	3	XLOC_090738	-	predicted	---
9	37038382	37038382	-	37038382	7	7	XLOC_090738	-	predicted	---
9	37038578	37038578	-	37038578	3	3	XLOC_090738	-	predicted	---
9	37133040	37133041	-	37133041	10	15	ENSSSCG00000014982	-	3'UTR	*MMP7*
9	37330746	37330746	-	37330746	5	5	XLOC_090743	-	predicted	---
9	37610514	37610514	-	37610514	10	10	---	---	intergenic	---
9	37622038	37622038	-	37622038	3	3	ENSSSCG00000014989	-	3'UTR	*DCUN1D5*

9	37622294	37622303	-	37622294	8	20	ENSSSCG00000014989	-	3'UTR	*DCUN1D5*
9	37651813	37651820	+	37651820	6	15	XLOC_089517	+	predicted	---
9	37943862	37943863	+	37943862	3	6	ENSSSCG00000014993	+	intron	---
9	38033509	38033519	+	38033509	6	19	XLOC_089519	+	predicted	---
9	38099372	38099372	-	38099372	3	3	---	---	intergenic	---
9	38127663	38127663	+	38127663	10	10	XLOC_089523	+	predicted	---
9	38282042	38282042	-	38282042	3	3	---	---	intergenic	---
9	38402399	38402399	+	38402399	11	11	XLOC_089524	+	predicted	---
9	38491455	38491455	-	38491455	3	3	ENSSSCG00000014994	-	intron	*PDGFD*
9	38498719	38498738	+	38498724	16	35	XLOC_089526	+	predicted	---
9	39078929	39078929	-	39078929	4	4	---	---	intergenic	---
9	39189533	39189536	-	39189533	4	8	ENSSSCG00000014997	-	3'UTR	---
9	39190179	39190179	-	39190179	18	18	ENSSSCG00000014997	-	3'UTR	---
9	39192465	39192465	-	39192465	3	3	ENSSSCG00000014997	-	ORF	---
9	39199847	39199850	-	39199847	12	16	ENSSSCG00000014997	-	intron	---
9	39207618	39207621	-	39207618	14	17	ENSSSCG00000014997	-	intron	---
9	39209734	39209734	-	39209734	13	13	ENSSSCG00000028711	-	3'UTR	*CASP1*
9	39448466	39448469	-	39448468	167	303	---	---	intergenic	---
9	39681313	39681313	+	39681313	3	3	ENSSSCG00000030565	+	intron	*GRIA4*
9	39701029	39701029	+	39701029	40	40	ENSSSCG00000030565	+	intron	*GRIA4*
9	39865364	39865364	-	39865364	5	5	ENSSSCG00000026833	-	3'UTR	*MSANTD4*
9	39866956	39866956	-	39866956	3	3	ENSSSCG00000026833	-	3'UTR	*MSANTD4*
9	39914932	39914936	-	39914933	18	51	ENSSSCG00000014999	-	ORF	*KBTBD3*
9	39984296	39984306	+	39984302	10	22	ENSSSCG00000014998	+	3'UTR	*AASDHPPT*
9	40006369	40006369	-	40006369	6	6	ENSSSCG00000028201	-	3'UTR	*CWF19L2*
9	40124449	40124449	-	40124449	3	3	ENSSSCG00000024628	-	3'UTR	*ALKBH8*

9	40178200	40178200	-	40178200	3	3	---	---	intergenic	---
9	40284053	40284053	+	40284053	3	3	XLOC_089539	+	predicted	---
9	40309466	40309468	-	40309466	77	80	ENSSSCG00000029651	-	3'UTR	*SLN*
9	40309686	40309686	+	40309686	6	6	---	---	intergenic	---
9	40386579	40386579	-	40386579	11	11	ENSSSCG00000015001	-	intron	*SLC35F2*
9	40458661	40458661	+	40458661	3	3	ENSSSCG00000021212	+	intron	*RAB39A*
9	40464582	40464582	-	40464582	4	4	ENSSSCG00000015006	-	ORF	---
9	40465308	40465308	+	40465308	3	3	ENSSSCG00000021212	+	intron	*RAB39A*
9	40532633	40532635	+	40532633	3	9	ENSSSCG00000029198	+	intron	*CUL5*
9	40581093	40581093	+	40581093	5	5	XLOC_089542	+	predicted	---
9	40609245	40609245	+	40609245	7	7	ENSSSCG00000026153	+	intron	*ACAT1*
9	40614261	40614261	+	40614261	4	4	ENSSSCG00000026153	+	ORF	*ACAT1*
9	40622001	40622001	+	40622001	3	3	ENSSSCG00000026153	+	3'UTR	*ACAT1*
9	40622044	40622085	+	40622083	46	137	ENSSSCG00000026153	+	3'UTR	*ACAT1*
9	40622136	40622146	+	40622137	33	82	ENSSSCG00000026153	+	3'UTR	*ACAT1*
9	40631179	40631179	-	40631179	3	3	XLOC_090768	-	predicted	---
9	40643367	40643367	-	40643367	3	3	---	---	intergenic	---
9	40761249	40761249	-	40761249	4	4	ENSSSCG00000025606	-	ORF	*ATM*
9	40834599	40834599	-	40834599	4	4	---	---	intergenic	---
9	40945436	40945436	+	40945436	3	3	ENSSSCG00000015007	+	3'UTR	*ATM*
9	41043249	41043249	-	41043249	4	4	XLOC_090773	-	predicted	---
9	41069989	41069995	-	41069995	4	7	XLOC_090773	-	predicted	---
9	41743058	41743058	-	41743058	3	3	---	---	intergenic	---
9	42434095	42434095	+	42434095	3	3	---	---	intergenic	---
9	42620872	42620872	-	42620872	3	3	---	---	intergenic	---
9	42951153	42951162	-	42951161	10	23	XLOC_090779	-	predicted	---

9	42953296	42953296	-	42953296	5	5	ENSSSCG00000015013	-	3'UTR	*RDX*
9	42980480	42980481	-	42980481	4	7	ENSSSCG00000015013	-	intron	*RDX*
9	42986757	42986757	-	42986757	5	5	ENSSSCG00000015013	-	intron	*RDX*
9	43027192	43027195	-	43027193	23	43	XLOC_090779	-	predicted	---
9	43051774	43051774	+	43051774	3	3	---	---	intergenic	---
9	43226392	43226392	+	43226392	3	3	---	---	intergenic	---
9	43236412	43236414	+	43236412	9	20	---	---	intergenic	---
9	43237415	43237417	+	43237417	6	13	XLOC_089558	+	predicted	---
9	43262102	43262103	+	43262102	8	12	XLOC_089559	+	predicted	---
9	43262159	43262185	+	43262162	25	102	XLOC_089559	+	predicted	---
9	43262318	43262318	+	43262318	3	3	XLOC_089559	+	predicted	---
9	43262778	43262778	+	43262778	3	3	XLOC_089559	+	predicted	---
9	43263068	43263068	+	43263068	4	4	XLOC_089559	+	predicted	---
9	43365909	43365915	+	43365909	12	18	---	---	intergenic	---
9	43440044	43440044	-	43440044	8	8	---	---	intergenic	---
9	43497340	43497340	+	43497340	16	16	---	---	intergenic	---
9	43701796	43701796	-	43701796	4	4	---	---	intergenic	---
9	43916019	43916019	+	43916019	3	3	XLOC_089563	+	predicted	---
9	43976618	43976618	+	43976618	4	4	---	---	intergenic	---
9	43990442	43990443	-	43990443	9	12	---	---	intergenic	---
9	44142392	44142404	-	44142404	55	92	XLOC_090788	-	predicted	---
9	44178006	44178006	-	44178006	4	4	---	---	intergenic	---
9	44269793	44269796	-	44269795	24	37	XLOC_090792	-	predicted	---
9	44280542	44280554	-	44280554	18	43	ENSSSCG00000029538	-	intron	*PPP2R1B*
9	44320749	44320749	-	44320749	3	3	ENSSSCG00000021820	-	3'UTR	---
9	44324277	44324281	-	44324278	34	45	ENSSSCG00000021820	-	intron	---

9	44379311	44379311	-	44379311	4	4	---	---	intergenic	---
9	44425457	44425465	+	44425464	21	71	XLOC_089571	+	predicted	---
9	44428978	44428989	-	44428985	139	399	ENSSSCG00000015025	-	3'UTR	*CRYAB*
9	44434631	44434637	+	44434636	9	21	XLOC_089571	+	predicted	---
9	44521229	44521229	+	44521229	7	7	ENSSSCG00000015036	+	intron	*DIXDC1*
9	44604883	44604883	+	44604883	3	3	ENSSSCG00000015030	+	3'UTR	*DLAT*
9	44605025	44605025	+	44605025	4	4	ENSSSCG00000015030	+	3'UTR	*DLAT*
9	44609301	44609316	-	44609316	10	40	XLOC_090797	-	predicted	---
9	44609372	44609393	-	44609393	9	23	XLOC_090797	-	predicted	---
9	44612643	44612647	-	44612645	21	45	ENSSSCG00000015031	-	ORF	*PIH1D2*
9	44612744	44612744	-	44612744	3	3	ENSSSCG00000015031	-	ORF	*PIH1D2*
9	44615934	44615940	-	44615940	6	15	---	---	intergenic	---
9	44629574	44629574	+	44629574	5	5	XLOC_089574	+	predicted	---
9	44629773	44629781	-	44629780	246	445	ENSSSCG00000023599	-	3'UTR	*TIMM8B*
9	44629860	44629860	+	44629860	3	3	XLOC_089574	+	predicted	---
9	44645551	44645616	+	44645586	24	69	ENSSSCG00000015034	+	3'UTR	---
9	44692183	44692186	-	44692185	77	117	ENSSSCG00000015037	-	3'UTR	*IL18*
9	44721684	44721684	+	44721684	8	8	XLOC_089575	+	predicted	---
9	44721730	44721732	+	44721732	5	13	---	---	intergenic	---
9	44765715	44765715	+	44765715	4	4	ENSSSCG00000015039	+	ORF	*BCO2*
9	44766400	44766400	+	44766400	3	3	ENSSSCG00000015039	+	ORF	*BCO2*
9	44767958	44767961	+	44767960	34	74	XLOC_089575	+	predicted	---
9	44779246	44779246	+	44779246	11	11	XLOC_089575	+	predicted	---
9	44779300	44779305	+	44779302	20	47	XLOC_089575	+	predicted	---
9	44815854	44815854	-	44815854	5	5	---	---	intergenic	---
9	44879129	44879129	-	44879129	7	7	---	---	intergenic	---

9	44886723	44886723	+	44886723	3	3	XLOC_089578	+	predicted	---
9	45216596	45216596	+	45216596	3	3	---	---	intergenic	---
9	45692354	45692354	-	45692354	5	5	---	---	intergenic	---
9	45693206	45693206	+	45693206	3	3	---	---	intergenic	---
9	46178500	46178507	-	46178505	8	22	ENSSSCG00000015050	-	3'UTR	*ZW10*
9	46235274	46235274	-	46235274	8	8	XLOC_090807	-	predicted	---
9	46235373	46235373	-	46235373	3	3	XLOC_090807	-	predicted	---
9	46258505	46258524	-	46258509	12	40	ENSSSCG00000015052	-	intron	*USP28*
9	46332136	46332136	+	46332136	3	3	ENSSSCG00000015053	+	intron	*HTR3B*
9	46368068	46368068	+	46368068	4	4	XLOC_089599	+	predicted	---
9	46368163	46368172	+	46368171	6	12	XLOC_089599	+	predicted	---
9	46368322	46368322	+	46368322	3	3	XLOC_089599	+	predicted	---
9	46371070	46371072	+	46371070	18	23	XLOC_089599	+	predicted	---
9	46443078	46443078	-	46443078	9	9	---	---	intergenic	---
9	46448157	46448157	-	46448157	7	7	XLOC_090808	-	predicted	---
9	46840659	46840659	-	46840659	6	6	---	---	intergenic	---
9	46840687	46840688	+	46840687	7	10	---	---	intergenic	---
9	46890872	46890881	-	46890874	73	116	XLOC_090811	-	predicted	---
9	46891035	46891035	-	46891035	3	3	XLOC_090811	-	predicted	---
9	46891276	46891276	-	46891276	5	5	XLOC_090811	-	predicted	---
9	46920778	46920778	-	46920778	5	5	---	---	intergenic	---
9	47005684	47005684	+	47005684	3	3	XLOC_089609	+	predicted	---
9	47076990	47076990	+	47076990	4	4	---	---	intergenic	---
9	47077778	47077778	+	47077778	3	3	---	---	intergenic	---
9	47096275	47096275	+	47096275	3	3	---	---	intergenic	---
9	47166257	47166257	+	47166257	3	3	ENSSSCG00000025102	+	3'UTR	---

9	47347614	47347614	-	47347614	3	3	---	---	intergenic	---
9	47729163	47729163	-	47729163	4	4	---	---	intergenic	---
9	47769394	47769396	+	47769396	12	25	XLOC_089611	+	predicted	---
9	47791885	47791887	+	47791887	7	10	XLOC_089613	+	predicted	---
9	48000249	48000249	-	48000249	4	4	---	---	intergenic	---
9	48031779	48031780	-	48031780	10	13	XLOC_090817	-	predicted	---
9	48301389	48301392	-	48301392	8	15	---	---	intergenic	---
9	48584576	48584576	-	48584576	3	3	---	---	intergenic	---
9	48781740	48781740	-	48781740	5	5	---	---	intergenic	---
9	48991166	48991166	+	48991166	6	6	---	---	intergenic	---
9	49198621	49198624	+	49198621	4	8	---	---	intergenic	---
9	49208940	49208942	-	49208941	13	21	ENSSSCG00000015065	-	3'UTR	*BUD13*
9	49241410	49241419	-	49241412	137	244	ENSSSCG00000015066	-	3'UTR	*ZPR1*
9	49251670	49251679	-	49251672	88	211	ENSSSCG00000015067	-	3'UTR	*APOA5*
9	49251785	49251818	-	49251805	84	317	ENSSSCG00000015067	-	3'UTR	*APOA5*
9	49252095	49252096	-	49252096	4	7	ENSSSCG00000015067	-	3'UTR	*APOA5*
9	49252386	49252399	-	49252394	348	704	ENSSSCG00000015067	-	3'UTR	*APOA5*
9	49252962	49252962	-	49252962	3	3	ENSSSCG00000015067	-	ORF	*APOA5*
9	49256882	49256884	+	49256884	6	9	XLOC_089619	+	predicted	---
9	49273923	49273923	-	49273923	4	4	ENSSSCG00000015068	-	3'UTR	*APOA4*
9	49274776	49274778	-	49274778	16	20	ENSSSCG00000015068	-	ORF	*APOA4*
9	49284422	49284441	+	49284422	3	6	ENSSSCG00000015069	+	ORF	*APOC3*
9	49285696	49285803	+	49285784	3380	12051	ENSSSCG00000015069	+	3'UTR	*APOC3*
9	49288637	49288645	-	49288640	540	909	ENSSSCG00000015070	-	3'UTR	*APOA1*
							ENSSSCG00000030921	-	3'UTR	*APOA1*

9	49289174	49289186	-	49289174	5	14	ENSSSCG00000030921	-	3'UTR	*APOA1*
							ENSSSCG00000015070	-	ORF	*APOA1*
9	49289232	49289287	-	49289250	58	203	ENSSSCG00000030921	-	3'UTR	*APOA1*
							ENSSSCG00000015070	-	ORF	*APOA1*
9	49290036	49290036	-	49290036	15	15	ENSSSCG00000030921	-	3'UTR	*APOA1*
9	49290089	49290089	-	49290089	7	7	ENSSSCG00000030921	-	3'UTR	*APOA1*
9	49295478	49295478	-	49295478	3	3	XLOC_090826	-	predicted	---
9	49297466	49297473	-	49297468	36	66	ENSSSCG00000015071	-	3'UTR	*SIK3*
9	49485894	49485894	-	49485894	9	9	---	---	intergenic	---
9	49499218	49499218	+	49499218	4	4	---	---	intergenic	---
9	49558756	49558756	-	49558756	5	5	---	---	intergenic	---
9	49602946	49602946	+	49602946	3	3	ENSSSCG00000024027	+	3'UTR	*PAFAH1B2*
9	49603902	49603904	+	49603902	13	22	XLOC_089624	+	predicted	---
9	49627984	49627984	+	49627984	3	3	ENSSSCG00000015074	+	3'UTR	*SIDT2*
9	49629033	49629038	+	49629037	17	43	ENSSSCG00000015074	+	3'UTR	*SIDT2*
9	49636692	49636703	+	49636696	34	102	XLOC_089626	+	predicted	---
9	49636893	49636894	-	49636894	9	12	ENSSSCG00000015072	-	3'UTR	*PCSK7*
9	49679247	49679247	+	49679247	3	3	ENSSSCG00000015077	+	intron	*RNF214*
9	49680588	49680588	+	49680588	9	9	ENSSSCG00000015077	+	intron	*RNF214*
							ENSSSCG00000015078	+	ORF	*PTMAP*
9	49682068	49682068	+	49682068	3	3	ENSSSCG00000015077	+	intron	*RNF214*
9	49714555	49714555	+	49714555	4	4	XLOC_089627	+	predicted	---
9	49714563	49714563	-	49714563	4	4	ENSSSCG00000015079	-	3'UTR	*BACE1*
9	49717884	49717884	-	49717884	3	3	ENSSSCG00000015079	-	3'UTR	*BACE1*
9	49825459	49825468	+	49825462	10	36	ENSSSCG00000015080	+	3'UTR	*CEP164*
9	50194906	50194906	-	50194906	3	3	ENSSSCG00000015082	-	intron	---

9	50244579	50244592	-	50244589	10	20	XLOC_090840	-	predicted	---
9	50261909	50261916	-	50261914	45	132	XLOC_090841	-	predicted	---
9	50368572	50368572	+	50368572	3	3	ENSSSCG00000015085	+	intron	*IL10RA*
9	50378866	50378876	+	50378866	8	18	ENSSSCG00000015085	+	3'UTR	*IL10RA*
9	50547543	50547544	-	50547543	17	20	ENSSSCG00000015089	-	3'UTR	*AMICA1*
9	50614270	50614270	-	50614270	3	3	ENSSSCG00000015091	-	ORF	*MPZL2*
9	50675095	50675095	+	50675095	13	13	ENSSSCG00000015092	+	3'UTR	*CD3E*
9	50678221	50678232	-	50678221	10	15	---	---	intergenic	---
9	50680923	50680923	-	50680923	5	5	XLOC_090847	-	predicted	---
9	50681154	50681154	-	50681154	3	3	ENSSSCG00000015093	-	3'UTR	*CD3D*
9	50697787	50697787	+	50697787	11	11	XLOC_089635	+	predicted	---
9	50743041	50743041	+	50743041	3	3	ENSSSCG00000015095	+	3'UTR	*UBE4A*
9	50745874	50745876	+	50745876	14	23	ENSSSCG00000015095	+	3'UTR	*UBE4A*
9	50754754	50754754	+	50754754	8	8	ENSSSCG00000023777	+	intron	---
9	50755647	50755669	+	50755648	74	96	ENSSSCG00000023777	+	3'UTR	---
9	50755722	50755738	+	50755727	34	129	ENSSSCG00000023777	+	3'UTR	---
9	50755805	50755805	+	50755805	13	13	ENSSSCG00000023777	+	3'UTR	---
9	50755913	50755913	+	50755913	6	6	ENSSSCG00000023777	+	3'UTR	---
9	50757676	50757676	+	50757676	7	7	ENSSSCG00000023777	+	3'UTR	---
9	50814122	50814128	-	50814122	41	46	XLOC_090849	-	predicted	---
9	50815866	50815866	-	50815866	7	7	ENSSSCG00000030053	-	3'UTR	*ARCN1*
9	50815923	50815923	-	50815923	3	3	ENSSSCG00000030053	-	3'UTR	*ARCN1*
9	50832811	50832811	-	50832811	4	4	ENSSSCG00000030053	-	ORF	*ARCN1*
9	50861085	50861089	+	50861087	22	41	ENSSSCG00000029649	+	3'UTR	*IFT46*
9	50861229	50861232	+	50861230	36	61	ENSSSCG00000029649	+	3'UTR	*IFT46*
9	50865551	50865551	-	50865551	5	5	XLOC_090850	-	predicted	---

9	50875972	50875980	-	50875972	6	13	XLOC_090850	-	predicted	---
9	50877090	50877090	+	50877090	3	3	XLOC_089637	+	predicted	---
9	50881899	50881899	-	50881899	3	3	ENSSSCG00000023324	-	intron	*KMT2A*
9	51060182	51060191	-	51060185	131	195	ENSSSCG00000015097	-	3'UTR	*DDX6*
9	51060387	51060388	-	51060387	9	12	ENSSSCG00000015097	-	3'UTR	*DDX6*
9	51062226	51062226	-	51062226	4	4	ENSSSCG00000015097	-	3'UTR	*DDX6*
9	51062469	51062469	-	51062469	8	8	ENSSSCG00000015097	-	3'UTR	*DDX6*
9	51063841	51063841	-	51063841	3	3	ENSSSCG00000015097	-	3'UTR	*DDX6*
9	51094618	51094618	-	51094618	3	3	ENSSSCG00000015097	-	ORF	*DDX6*
9	51255141	51255141	+	51255141	4	4	XLOC_089646	+	predicted	---
9	51274497	51274502	-	51274502	50	92	XLOC_090854	-	predicted	---
9	51274517	51274517	+	51274517	3	3	ENSSSCG00000015102	+	3'UTR	*CCDC84*
9	51281433	51281442	+	51281439	127	181	ENSSSCG00000015104	+	3'UTR	*TRAPPC4*
9	51282375	51282378	-	51282377	50	70	ENSSSCG00000015105	-	3'UTR	*SLC37A4*
9	51282813	51282813	+	51282813	3	3	XLOC_089648	+	predicted	---
9	51282942	51282942	-	51282942	3	3	ENSSSCG00000015105	-	3'UTR	*SLC37A4*
9	51283171	51283171	-	51283171	3	3	ENSSSCG00000015105	-	ORF	*SLC37A4*
9	51300060	51300067	-	51300064	68	122	ENSSSCG00000015106	-	3'UTR	*HYOU1*
9	51332594	51332600	+	51332594	6	16	ENSSSCG00000015107	+	3'UTR	*VPS11*
9	51341267	51341267	+	51341267	12	12	ENSSSCG00000015108	+	intron	*HMBS*
9	51343652	51343658	+	51343658	7	11	XLOC_089650	+	predicted	---
9	51343811	51343818	+	51343811	7	13	XLOC_089650	+	predicted	---
9	51343963	51343963	-	51343963	7	7	XLOC_090857	-	predicted	---
9	51346010	51346015	-	51346010	7	14	ENSSSCG00000015110	-	3'UTR	*DPAGT1*
9	51366224	51366225	+	51366224	7	11	ENSSSCG00000015111	+	3'UTR	*C2CD2L*
9	51383068	51383068	+	51383068	5	5	ENSSSCG00000015112	+	3'UTR	*HINFP*

9	51435893	51435897	+	51435893	6	21	XLOC_089654	+	predicted	---
9	51580812	51580817	+	51580814	21	37	XLOC_089658	+	predicted	---
9	51582421	51582421	-	51582421	4	4	ENSSSCG00000024936	-	3'UTR	*C1QTNF5*
9	51595416	51595427	-	51595417	35	70	ENSSSCG00000015120	-	3'UTR	*USP2*
9	51596472	51596472	-	51596472	5	5	ENSSSCG00000015120	-	3'UTR	*USP2*
9	51639473	51639473	-	51639473	4	4	ENSSSCG00000015122	-	3'UTR	*CD90*
9	51670387	51670393	+	51670392	13	19	XLOC_089663	+	predicted	---
9	51716937	51716937	+	51716937	3	3	XLOC_089664	+	predicted	---
9	51877512	51877512	-	51877512	10	10	XLOC_090867	-	predicted	---
9	52345361	52345368	-	52345362	39	116	XLOC_090871	-	predicted	---
9	52374314	52374316	+	52374316	22	26	ENSSSCG00000015126	+	3'UTR	*TRIM29*
9	52382329	52382329	+	52382329	3	3	---	---	intergenic	---
9	52431283	52431296	+	52431284	10	41	ENSSSCG00000026647	+	3'UTR	*OAF*
9	52432142	52432142	+	52432142	3	3	ENSSSCG00000026647	+	3'UTR	*OAF*
9	52489517	52489517	+	52489517	4	4	---	---	intergenic	---
9	52728718	52728718	+	52728718	3	3	ENSSSCG00000015129	+	3'UTR	*ARHGEF12*
9	52729243	52729253	+	52729244	15	23	ENSSSCG00000015129	+	3'UTR	*ARHGEF12*
9	52753359	52753359	+	52753359	3	3	---	---	intergenic	---
9	52806756	52806756	-	52806756	3	3	---	---	intergenic	---
9	53198613	53198613	+	53198613	3	3	ENSSSCG00000015131	+	intron	*GRIK4*
9	53210953	53210953	+	53210953	3	3	ENSSSCG00000015131	+	intron	*GRIK4*
9	53405307	53405316	+	53405315	4	11	ENSSSCG00000023728	+	3'UTR	*TBCEL*
9	53407754	53407760	+	53407754	8	11	ENSSSCG00000023728	+	3'UTR	*TBCEL*
9	53415667	53415668	+	53415668	4	7	---	---	intergenic	---
9	53480794	53480794	+	53480794	4	4	ENSSSCG00000024215	+	intron	*TECTA*
9	53580334	53580340	+	53580338	88	199	ENSSSCG00000028517	+	3'UTR	*SC5D*

9	53580769	53580769	+	53580769	5	5	ENSSSCG00000028517	+	3'UTR	*SC5D*
9	53677146	53677155	+	53677155	15	24	XLOC_089682	+	predicted	---
9	53799753	53799753	+	53799753	5	5	ENSSSCG00000026007	+	intron	*SORL1*
9	53830380	53830380	-	53830380	3	3	XLOC_090881	-	predicted	---
9	53910430	53910457	+	53910433	11	33	---	---	intergenic	---
9	53985158	53985158	+	53985158	3	3	---	---	intergenic	---
9	54146097	54146099	-	54146097	3	6	---	---	intergenic	---
9	54262559	54262559	+	54262559	3	3	---	---	intergenic	---
9	54324151	54324151	-	54324151	3	3	XLOC_090883	-	predicted	---
9	54379828	54379833	+	54379833	7	12	XLOC_089685	+	predicted	---
9	55118032	55118053	+	55118053	5	11	ENSSSCG00000015136	+	3'UTR	*UBASH3B*
9	55233151	55233153	+	55233152	4	10	ENSSSCG00000015138	+	3'UTR	*C11orf63*
9	55303747	55303747	+	55303747	6	6	ENSSSCG00000015142	+	intron	---
9	55406867	55406867	+	55406867	4	4	ENSSSCG00000015140	+	ORF	---
9	55559854	55559854	-	55559854	9	9	---	---	intergenic	---
9	55790944	55790947	+	55790947	6	10	ENSSSCG00000015144	+	3'UTR	---
9	55795665	55795665	+	55795665	5	5	XLOC_089704	+	predicted	---
9	55873839	55873839	-	55873839	4	4	XLOC_090894	-	predicted	---
9	55981304	55981304	-	55981304	6	6	---	---	intergenic	---
9	55981337	55981340	+	55981339	7	16	---	---	intergenic	---
9	56018871	56018871	-	56018871	4	4	---	---	intergenic	---
9	56022207	56022226	+	56022211	4	14	XLOC_089708	+	predicted	---
9	56045277	56045277	+	56045277	3	3	---	---	intergenic	---
9	56554575	56554575	+	56554575	7	7	ENSSSCG00000015175	+	3'UTR	*VWA5A*
9	57211441	57211441	-	57211441	3	3	---	---	intergenic	---
9	57234573	57234573	+	57234573	7	7	---	---	intergenic	---

9	57393599	57393606	+	57393599	4	8	ENSSSCG00000015195	+	3'UTR	---
9	57397133	57397133	-	57397133	10	10	XLOC_090934	-	predicted	---
9	57398377	57398377	-	57398377	6	6	ENSSSCG00000015196	-	ORF	---
9	57437704	57437705	+	57437705	6	10	ENSSSCG00000015197	+	ORF	*SPA17*
9	57448698	57448700	+	57448698	8	11	ENSSSCG00000015197	+	ORF	*SPA17*
9	57448804	57448814	+	57448812	39	71	ENSSSCG00000015197	+	3'UTR	*SPA17*
9	57448881	57448939	+	57448923	216	1072	ENSSSCG00000015197	+	3'UTR	*SPA17*
9	57498523	57498524	-	57498523	8	14	ENSSSCG00000015200	-	3'UTR	---
9	57509431	57509431	+	57509431	3	3	XLOC_089738	+	predicted	---
9	57575900	57575900	+	57575900	3	3	---	---	intergenic	---
9	57597133	57597133	+	57597133	3	3	XLOC_089740	+	predicted	---
9	57599264	57599264	-	57599264	5	5	ENSSSCG00000015203	-	3'UTR	*ROBO4*
9	57602551	57602551	-	57602551	3	3	ENSSSCG00000015203	-	intron	*ROBO4*
9	57666754	57666754	+	57666754	3	3	ENSSSCG00000015206	+	intron	*CCDC15*
9	57678344	57678344	+	57678344	11	11	ENSSSCG00000015206	+	ORF	*CCDC15*
9	57731499	57731500	+	57731499	7	10	ENSSSCG00000015206	+	3'UTR	*CCDC15*
9	57787190	57787194	+	57787190	3	6	ENSSSCG00000022536	+	3'UTR	*SLC37A2*
9	57844890	57844900	-	57844891	4	14	---	---	intergenic	---
9	57905820	57905820	-	57905820	7	7	---	---	intergenic	---
9	58195476	58195476	+	58195476	3	3	XLOC_089747	+	predicted	---
9	58374162	58374165	+	58374165	12	36	ENSSSCG00000015213	+	3'UTR	*EI24*
9	58378733	58378744	-	58378741	8	18	---	---	intergenic	---
9	58378817	58378833	-	58378817	5	8	---	---	intergenic	---
9	58378970	58379011	-	58378978	103	164	---	---	intergenic	---
9	58379068	58379068	-	58379068	4	4	---	---	intergenic	---
9	58379725	58379725	-	58379725	3	3	---	---	intergenic	---

9	58406192	58406224	+	58406217	40	92	ENSSSCG00000015214	+	3'UTR	*STT3A*
9	58406502	58406502	+	58406502	4	4	XLOC_089750	+	predicted	---
9	58407887	58407897	+	58407896	7	27	XLOC_089750	+	predicted	---
9	58456599	58456640	-	58456600	167	233	ENSSSCG00000015216	-	3'UTR	*ACRV1*
9	58456821	58456821	-	58456821	3	3	ENSSSCG00000015216	-	3'UTR	*ACRV1*
9	58627605	58627615	-	58627608	14	42	XLOC_090946	-	predicted	---
9	58635131	58635135	+	58635135	5	12	ENSSSCG00000015222	+	3'UTR	---
9	58667144	58667144	+	58667144	6	6	XLOC_089754	+	predicted	---
9	58668384	58668384	+	58668384	7	7	XLOC_089754	+	predicted	---
9	58714171	58714171	+	58714171	4	4	---	---	intergenic	---
9	58763537	58763542	+	58763537	6	11	ENSSSCG00000015223	+	ORF	---
9	58771808	58771855	+	58771831	34	69	ENSSSCG00000015223	+	3'UTR	---
9	58771903	58771920	+	58771906	50	126	---	---	intergenic	---
9	58772216	58772216	+	58772216	4	4	---	---	intergenic	---
9	58968326	58968336	+	58968326	6	13	ENSSSCG00000025806	+	3'UTR	---
9	58968390	58968401	+	58968394	30	72	ENSSSCG00000025806	+	3'UTR	---
9	58968921	58968936	-	58968934	37	60	ENSSSCG00000015227	-	3'UTR	*SRPR*
9	58969832	58969832	-	58969832	4	4	ENSSSCG00000015227	-	3'UTR	*SRPR*
9	59036371	59036378	+	59036373	53	119	ENSSSCG00000030017	+	3'UTR	*FOXRED1*
9	59062708	59062708	-	59062708	3	3	---	---	intergenic	---
9	59100506	59100509	+	59100506	13	23	ENSSSCG00000015231	+	3'UTR	*DCPS*
9	59100756	59100774	+	59100756	4	7	ENSSSCG00000015231	+	3'UTR	*DCPS*
9	59323658	59323658	+	59323658	3	3	ENSSSCG00000015232	+	3'UTR	*ST3GAL4*
9	59590451	59590451	+	59590451	3	3	XLOC_089763	+	predicted	---
9	60069544	60069544	+	60069544	3	3	---	---	intergenic	---
9	60298686	60298686	-	60298686	4	4	---	---	intergenic	---

9	60926197	60926197	+	60926197	3	3	XLOC_089766	+	predicted	---
9	60970179	60970180	+	60970180	8	14	---	---	intergenic	---
9	61052504	61052504	-	61052504	5	5	XLOC_090957	-	predicted	---
9	61500242	61500242	+	61500242	3	3	---	---	intergenic	---
9	61532398	61532398	+	61532398	3	3	ENSSSCG00000015235	+	intron	*ETS1*
9	61540104	61540104	+	61540104	5	5	XLOC_089771	+	predicted	---
9	61543110	61543112	+	61543112	11	17	XLOC_089771	+	predicted	---
9	61713867	61713874	-	61713874	9	13	XLOC_090962	-	predicted	---
9	61840825	61840833	+	61840826	10	24	---	---	intergenic	---
9	61997193	61997193	-	61997193	3	3	ENSSSCG00000015238	-	3'UTR	*ARHGAP32*
9	62330671	62330671	+	62330671	5	5	---	---	intergenic	---
9	62633120	62633120	-	62633120	3	3	---	---	intergenic	---
9	62806434	62806434	-	62806434	5	5	ENSSSCG00000015242	-	3'UTR	---
9	62824000	62824000	-	62824000	4	4	XLOC_090970	-	predicted	---
9	62880308	62880308	-	62880308	4	4	---	---	intergenic	---
9	63000659	63000659	+	63000659	3	3	ENSSSCG00000015244	+	ORF	*APLP2*
9	63013062	63013065	+	63013062	3	6	ENSSSCG00000015244	+	ORF	*APLP2*
9	63132698	63132698	+	63132698	3	3	ENSSSCG00000015247	+	intron	*ZBTB44*
9	63136709	63136717	+	63136715	30	68	XLOC_089782	+	predicted	---
9	63142839	63142844	+	63142843	5	12	XLOC_089782	+	predicted	---
9	63158989	63158994	-	63158990	14	20	XLOC_090974	-	predicted	---
9	63214653	63214657	-	63214656	44	51	XLOC_090976	-	predicted	---
9	63214901	63214901	+	63214901	3	3	---	---	intergenic	---
9	63275498	63275498	-	63275498	4	4	---	---	intergenic	---
9	63543491	63543491	-	63543491	3	3	---	---	intergenic	---
9	63787756	63787756	-	63787756	3	3	XLOC_090982	-	predicted	---

9	63915875	63915876	+	63915876	5	8	---	---	intergenic	---
9	64225453	64225454	+	64225454	6	9	---	---	intergenic	---
9	64787465	64787465	+	64787465	77	77	---	---	intergenic	---
9	64844771	64844771	+	64844771	3	3	XLOC_089794	+	predicted	---
9	64969445	64969445	+	64969445	3	3	---	---	intergenic	---
9	65040835	65040835	-	65040835	3	3	---	---	intergenic	---
9	65173477	65173477	-	65173477	5	5	---	---	intergenic	---
9	65530758	65530758	+	65530758	3	3	---	---	intergenic	---
9	66160210	66160210	-	66160210	3	3	---	---	intergenic	---
9	66173222	66173224	-	66173222	7	17	---	---	intergenic	---
9	66317080	66317081	-	66317080	3	6	---	---	intergenic	---
9	66478234	66478239	+	66478234	13	35	XLOC_089798	+	predicted	---
9	66589510	66589510	-	66589510	6	6	---	---	intergenic	---
9	66636540	66636540	-	66636540	3	3	---	---	intergenic	---
9	67438998	67438998	+	67438998	3	3	XLOC_089808	+	predicted	---
9	67560792	67560799	+	67560797	76	117	ENSSSCG00000015257	+	3'UTR	*GLB1L3*
9	67614252	67614264	+	67614258	14	41	ENSSSCG00000015258	+	3'UTR	*GLB1L2*
9	67668481	67668481	-	67668481	3	3	---	---	intergenic	---
9	67668502	67668503	+	67668503	6	9	---	---	intergenic	---
9	67790875	67790875	+	67790875	3	3	---	---	intergenic	---
9	67812865	67812865	-	67812865	4	4	XLOC_091002	-	predicted	---
9	68030956	68030956	+	68030956	3	3	---	---	intergenic	---
9	68075178	68075180	-	68075180	8	15	---	---	intergenic	---
9	68847717	68847719	+	68847718	26	49	---	---	intergenic	---
9	69255884	69255884	+	69255884	3	3	ENSSSCG00000024613	+	intron	*GUCY1A2*
9	69301361	69301361	+	69301361	4	4	ENSSSCG00000024613	+	intron	*GUCY1A2*

9	70043603	70043603	+	70043603	4	4	ENSSSCG00000023472	+	ORF	*FMO3*
9	70044641	70044641	+	70044641	23	23	ENSSSCG00000023472	+	ORF	*FMO3*
9	70053077	70053077	+	70053077	3	3	ENSSSCG00000023472	+	3'UTR	*FMO3*
9	70053130	70053147	+	70053147	13	51	ENSSSCG00000023472	+	3'UTR	*FMO3*
9	70053795	70053801	+	70053801	10	17	ENSSSCG00000023472	+	3'UTR	*FMO3*
9	70156184	70156213	+	70156196	17	31	ENSSSCG00000015268	+	ORF	*FMO1*
9	70159280	70159280	+	70159280	4	4	ENSSSCG00000015268	+	ORF	*FMO1*
9	70165403	70165423	+	70165403	3	6	ENSSSCG00000015268	+	ORF	*FMO1*
9	70167669	70167673	+	70167673	4	7	ENSSSCG00000015268	+	ORF	*FMO1*
9	70171167	70171181	+	70171167	13	20	ENSSSCG00000015268	+	ORF	*FMO1*
9	70171555	70171555	+	70171555	16	16	ENSSSCG00000015268	+	3'UTR	*FMO1*
9	70171692	70171701	+	70171693	178	454	ENSSSCG00000015268	+	3'UTR	*FMO1*
9	70216687	70216687	+	70216687	3	3	ENSSSCG00000015266	+	intron	*FMO4*
9	70277428	70277428	-	70277428	4	4	---	---	intergenic	---
9	70279243	70279243	-	70279243	5	5	---	---	intergenic	---
9	70329076	70329076	+	70329076	4	4	ENSSSCG00000028322	+	ORF	*BTG2*
9	70329423	70329423	+	70329423	4	4	ENSSSCG00000028322	+	3'UTR	*BTG2*
9	70331393	70331410	+	70331398	122	179	ENSSSCG00000028322	+	3'UTR	*BTG2*
9	70356598	70356604	-	70356598	10	23	ENSSSCG00000015270	-	3'UTR	*FMOD*
9	70500610	70500612	+	70500611	31	38	XLOC_089826	+	predicted	---
9	70514565	70514590	+	70514590	8	17	ENSSSCG00000015272	+	3'UTR	*OPTC*
9	70784179	70784179	-	70784179	23	23	ENSSSCG00000028069	-	3'UTR	---
9	70896933	70896934	+	70896933	8	12	ENSSSCG00000028035	+	ORF	*ZC3H11A*
9	70897279	70897281	+	70897279	4	7	ENSSSCG00000028035	+	intron	*ZC3H11A*
9	70900500	70900500	+	70900500	3	3	ENSSSCG00000028035	+	ORF	*ZC3H11A*
9	70903376	70903385	+	70903376	11	28	ENSSSCG00000028035	+	3'UTR	*ZC3H11A*

9	70915524	70915528	+	70915526	86	123	ENSSSCG00000023562	+	3'UTR	*SNRPE*
9	71080644	71080657	+	71080652	110	346	ENSSSCG00000015276	+	3'UTR	*ETNK2*
9	71085279	71085280	-	71085280	8	11	---	---	intergenic	---
9	71106076	71106077	-	71106076	3	6	---	---	intergenic	---
9	71106112	71106112	+	71106112	3	3	---	---	intergenic	---
9	71134586	71134586	-	71134586	4	4	---	---	intergenic	---
9	71175507	71175514	-	71175507	6	21	XLOC_091022	-	predicted	---
9	71199414	71199414	-	71199414	3	3	XLOC_091023	-	predicted	---
9	71379873	71379873	+	71379873	8	8	---	---	intergenic	---
9	71392199	71392205	-	71392205	9	16	ENSSSCG00000015282	-	3'UTR	*PPP1R15B*
9	71395206	71395206	-	71395206	4	4	ENSSSCG00000015282	-	ORF	*PPP1R15B*
9	71553379	71553382	+	71553379	7	11	XLOC_089839	+	predicted	---
9	71564580	71564580	-	71564580	19	19	---	---	intergenic	---
9	71698892	71698892	-	71698892	4	4	---	---	intergenic	---
9	71913078	71913078	-	71913078	5	5	XLOC_091030	-	predicted	---
9	72008657	72008657	-	72008657	7	7	XLOC_091031	-	predicted	---
9	72008698	72008698	+	72008698	3	3	XLOC_089843	+	predicted	---
9	72010450	72010462	-	72010450	3	9	XLOC_091031	-	predicted	---
9	72010499	72010499	-	72010499	4	4	XLOC_091031	-	predicted	---
9	72173632	72173632	+	72173632	4	4	ENSSSCG00000015287	+	3'UTR	*TMCC2*
9	72201236	72201241	-	72201236	6	11	ENSSSCG00000027243	-	3'UTR	*NUAK2*
9	72278805	72278805	-	72278805	17	17	XLOC_091037	-	predicted	---
9	72278861	72278863	-	72278861	4	8	XLOC_091037	-	predicted	---
9	72279082	72279082	-	72279082	3	3	ENSSSCG00000015291	-	ORF	*LEMD1*
9	72309043	72309043	+	72309043	3	3	---	---	intergenic	---
9	72449031	72449031	+	72449031	10	10	XLOC_089850	+	predicted	---

9	72605342	72605345	+	72605342	8	17	---	---	intergenic	---
9	72609621	72609621	-	72609621	4	4	ENSSSCG00000026547	-	3'UTR	*SLC45A3*
9	72663392	72663399	-	72663394	60	100	XLOC_091043	-	predicted	---
9	72667692	72667699	-	72667699	7	13	XLOC_091043	-	predicted	---
9	72668357	72668357	-	72668357	3	3	XLOC_091043	-	predicted	---
9	72668450	72668450	-	72668450	8	8	XLOC_091043	-	predicted	---
9	72668586	72668586	-	72668586	7	7	XLOC_091043	-	predicted	---
9	72672047	72672047	-	72672047	4	4	ENSSSCG00000022398	-	ORF	*NUCKS1*
9	72714984	72714984	-	72714984	5	5	XLOC_091044	-	predicted	---
9	72732594	72732594	-	72732594	5	5	---	---	intergenic	---
9	72805964	72805964	-	72805964	5	5	ENSSSCG00000021041	-	intron	*PM20D1*
9	72931423	72931423	+	72931423	5	5	ENSSSCG00000022307	+	intron	*RAB7B*
9	72946413	72946413	+	72946413	6	6	ENSSSCG00000022307	+	3'UTR	*RAB7B*
9	72972402	72972404	+	72972404	4	7	XLOC_089856	+	predicted	---
9	72973124	72973124	+	72973124	5	5	XLOC_089856	+	predicted	---
9	73007279	73007279	+	73007279	3	3	ENSSSCG00000015638	+	3'UTR	*C1orf186*
9	73032897	73032897	-	73032897	4	4	---	---	intergenic	---
9	73106826	73106826	+	73106826	5	5	ENSSSCG00000015645	+	intron	*SRGAP2*
9	73344685	73344685	+	73344685	3	3	ENSSSCG00000015645	+	intron	*SRGAP2*
9	73553584	73553584	+	73553584	3	3	ENSSSCG00000015647	+	intron	*RASSF5*
9	73588710	73588710	+	73588710	3	3	ENSSSCG00000015647	+	3'UTR	*RASSF5*
9	73590596	73590604	+	73590604	11	20	ENSSSCG00000015647	+	3'UTR	*RASSF5*
9	73593839	73593841	-	73593840	16	24	XLOC_091053	-	predicted	---
9	73652010	73652018	-	73652011	45	69	XLOC_091054	-	predicted	---
9	73659482	73659482	+	73659482	5	5	---	---	intergenic	---
9	73717080	73717080	+	73717080	11	11	ENSSSCG00000015650	+	3'UTR	*MAPKAPK2*

9	73787027	73787028	+	73787027	5	10	---	---	intergenic	---
9	74018104	74018104	+	74018104	4	4	---	---	intergenic	---
9	74051915	74051915	-	74051915	9	9	XLOC_091059	-	predicted	---
9	74057172	74057183	-	74057176	76	175	ENSSSCG00000015659	-	3'UTR	*YOD1*
9	74084460	74084460	-	74084460	8	8	---	---	intergenic	---
9	74087284	74087289	+	74087284	7	11	XLOC_089877	+	predicted	---
9	74087375	74087382	+	74087376	6	14	XLOC_089877	+	predicted	---
9	74105683	74105687	+	74105685	104	206	ENSSSCG00000015661	+	3'UTR	*C4BPB*
9	74122355	74122356	+	74122356	32	36	ENSSSCG00000015662	+	ORF	*C4BPA*
9	74126406	74126406	+	74126406	3	3	ENSSSCG00000015662	+	ORF	*C4BPA*
9	74136429	74136429	+	74136429	8	8	ENSSSCG00000015662	+	ORF	*C4BPA*
9	74154157	74154157	+	74154157	3	3	ENSSSCG00000015662	+	ORF	*C4BPA*
9	74154462	74154501	+	74154498	60	139	XLOC_089878	+	predicted	---
9	74207943	74207954	+	74207947	119	215	ENSSSCG00000015663	+	3'UTR	---
9	74235523	74235523	+	74235523	5	5	ENSSSCG00000022701	+	3'UTR	---
9	74250579	74250579	-	74250579	3	3	---	---	intergenic	---
9	74596245	74596245	-	74596245	3	3	---	---	intergenic	---
9	74616610	74616612	+	74616610	14	24	XLOC_089887	+	predicted	---
9	74661960	74661960	+	74661960	3	3	---	---	intergenic	---
9	74669900	74669901	+	74669900	5	9	---	---	intergenic	---
9	74675720	74675720	+	74675720	3	3	XLOC_089888	+	predicted	---
9	74676006	74676006	-	74676006	4	4	---	---	intergenic	---
9	74702961	74702962	-	74702962	9	12	---	---	intergenic	---
9	74716177	74716177	+	74716177	9	9	---	---	intergenic	---
9	74928753	74928759	-	74928754	48	90	ENSSSCG00000015298	-	3'UTR	*SRI*
9	74928919	74928923	-	74928922	17	39	ENSSSCG00000015298	-	3'UTR	*SRI*

9	74933282	74933282	-	74933282	3	3	ENSSSCG00000015298	-	intron	*SRI*
9	74979877	74979877	+	74979877	18	18	---	---	intergenic	---
9	74981679	74981679	-	74981679	7	7	XLOC_091071	-	predicted	---
9	74982788	74982794	-	74982788	20	32	XLOC_091071	-	predicted	---
9	74983888	74983888	-	74983888	5	5	ENSSSCG00000015299	-	3'UTR	*STEAP4*
9	74984204	74984204	-	74984204	3	3	ENSSSCG00000015299	-	3'UTR	*STEAP4*
9	74987928	74987928	-	74987928	4	4	ENSSSCG00000015299	-	intron	*STEAP4*
9	75000881	75000881	-	75000881	4	4	ENSSSCG00000015299	-	intron	*STEAP4*
9	75398420	75398421	-	75398421	18	27	XLOC_091072	-	predicted	---
9	75409923	75409925	+	75409924	34	52	XLOC_089890	+	predicted	---
9	75412359	75412359	-	75412359	5	5	XLOC_091072	-	predicted	---
9	75412940	75412949	-	75412946	53	97	XLOC_091072	-	predicted	---
9	75413063	75413071	-	75413064	28	86	ENSSSCG00000021276	-	ORF	*C7orf62*
9	75413126	75413134	-	75413127	30	55	ENSSSCG00000021276	-	ORF	*C7orf62*
9	75413259	75413259	-	75413259	3	3	ENSSSCG00000021276	-	ORF	*C7orf62*
9	75413313	75413323	-	75413319	17	44	ENSSSCG00000021276	-	ORF	*C7orf62*
9	75444055	75444055	+	75444055	11	11	---	---	intergenic	---
9	75462627	75462628	+	75462627	4	7	---	---	intergenic	---
9	75639611	75639614	+	75639612	10	21	---	---	intergenic	---
9	76969145	76969146	-	76969145	7	11	---	---	intergenic	---
9	76988865	76988865	+	76988865	8	8	XLOC_089896	+	predicted	---
9	76998243	76998244	-	76998244	5	9	XLOC_091073	-	predicted	---
9	77019188	77019191	-	77019188	13	26	ENSSSCG00000015305	-	3'UTR	*GTPBP10*
9	77051428	77051428	-	77051428	4	4	---	---	intergenic	---
9	77055010	77055010	-	77055010	6	6	---	---	intergenic	---
9	77067871	77067871	+	77067871	4	4	---	---	intergenic	---

9	77083275	77083275	-	77083275	5	5	---	---	intergenic	---
9	77138421	77138421	-	77138421	3	3	---	---	intergenic	---
9	77180131	77180132	+	77180132	5	9	---	---	intergenic	---
9	77213288	77213297	+	77213291	33	70	XLOC_089899	+	predicted	---
9	77914438	77914438	-	77914438	3	3	XLOC_091077	-	predicted	---
9	78302976	78302976	+	78302976	8	8	---	---	intergenic	---
9	78371151	78371151	-	78371151	3	3	---	---	intergenic	---
9	78581678	78581678	-	78581678	3	3	XLOC_091080	-	predicted	---
9	78583764	78583764	-	78583764	3	3	ENSSSCG00000015309	-	ORF	*MTERF1*
9	78790290	78790290	+	78790290	4	4	ENSSSCG00000015310	+	3'UTR	*AKAP9*
							ENSSSCG00000015310	+	ORF	*AKAP9*
9	78792894	78792926	-	78792907	84	255	ENSSSCG00000015311	-	3'UTR	*CYP51*
9	78792994	78793005	-	78792998	30	74	ENSSSCG00000015311	-	3'UTR	*CYP51*
9	78793469	78793478	-	78793477	27	56	ENSSSCG00000015311	-	3'UTR	*CYP51*
9	78793573	78793588	-	78793582	124	260	ENSSSCG00000015311	-	3'UTR	*CYP51*
9	78793637	78793637	-	78793637	3	3	ENSSSCG00000015311	-	3'UTR	*CYP51*
9	78801052	78801052	-	78801052	3	3	ENSSSCG00000015311	-	ORF	*CYP51*
9	78812250	78812250	-	78812250	3	3	ENSSSCG00000015311	-	ORF	*CYP51*
9	78826572	78826573	-	78826573	29	42	XLOC_091082	-	predicted	---
9	78854957	78854957	-	78854957	3	3	ENSSSCG00000029092	-	ORF	*LRRD1*
9	78855026	78855026	-	78855026	4	4	ENSSSCG00000029092	-	ORF	*LRRD1*
9	78855503	78855503	-	78855503	5	5	XLOC_091082	-	predicted	---
9	78866485	78866485	-	78866485	3	3	XLOC_091082	-	predicted	---
9	78870046	78870046	-	78870046	13	13	---	---	intergenic	---
9	78874199	78874202	-	78874202	5	8	ENSSSCG00000015313	-	3'UTR	*KRIT1*
9	79058053	79058053	+	79058053	4	4	XLOC_089905	+	predicted	---

9	79064890	79064915	-	79064891	84	252	XLOC_091090	-	predicted	---
9	79098720	79098726	+	79098725	13	23	ENSSSCG00000025577	+	3'UTR	*GATAD1*
9	79114426	79114426	-	79114426	3	3	XLOC_091093	-	predicted	---
9	79121834	79121834	-	79121834	7	7	ENSSSCG00000029967	-	intron	*PEX1*
9	79123701	79123701	-	79123701	3	3	ENSSSCG00000029967	-	intron	*PEX1*
9	79174344	79174344	+	79174344	3	3	ENSSSCG00000015315	+	intron	*RBM48*
9	79181535	79181561	-	79181560	78	154	XLOC_091094	-	predicted	---
9	79212003	79212003	-	79212003	5	5	ENSSSCG00000015317	-	intron	*FAM133B*
9	79219404	79219405	-	79219404	36	40	ENSSSCG00000015317	-	intron	*FAM133B*
9	79220107	79220107	-	79220107	3	3	ENSSSCG00000015317	-	intron	*FAM133B*
9	79221079	79221079	-	79221079	7	7	ENSSSCG00000015317	-	intron	*FAM133B*
9	79239881	79239888	-	79239881	7	11	XLOC_091095	-	predicted	---
9	79249875	79249875	-	79249875	3	3	ENSSSCG00000015318	-	3'UTR	*CDK6*
9	79249958	79249958	-	79249958	7	7	ENSSSCG00000015318	-	3'UTR	*CDK6*
9	79679758	79679767	-	79679758	3	6	XLOC_091098	-	predicted	---
9	79728329	79728329	-	79728329	3	3	---	---	intergenic	---
9	79735227	79735227	-	79735227	3	3	ENSSSCG00000025870	-	3'UTR	*HEPACAM2*
9	79855491	79855491	+	79855491	4	4	ENSSSCG00000023293	+	intron	---
9	80646431	80646431	-	80646431	4	4	ENSSSCG00000015322	-	3'UTR	*TFPI2*
9	80646485	80646492	-	80646485	6	11	ENSSSCG00000015322	-	3'UTR	*TFPI2*
9	80649099	80649099	-	80649099	3	3	ENSSSCG00000015322	-	ORF	*TFPI2*
9	80763110	80763110	+	80763110	3	3	ENSSSCG00000015324	+	ORF	*GNG11*
9	80765344	80765345	+	80765344	27	45	ENSSSCG00000015324	+	intron	*GNG11*
9	80767238	80767238	+	80767238	3	3	ENSSSCG00000015324	+	3'UTR	*GNG11*
9	80767389	80767400	+	80767392	115	207	ENSSSCG00000015324	+	3'UTR	*GNG11*
9	80823676	80823676	-	80823676	3	3	ENSSSCG00000015325	-	3'UTR	*BET1*

9	80824371	80824378	-	80824376	38	73	ENSSSCG00000015325	-	3'UTR	*BET1*
9	80854299	80854299	-	80854299	13	13	---	---	intergenic	---
9	81390454	81390454	+	81390454	4	4	ENSSSCG00000015326	+	ORF	*COL1A2*
9	81391194	81391194	+	81391194	13	13	ENSSSCG00000015326	+	ORF	*COL1A2*
9	81391351	81391353	+	81391352	173	253	ENSSSCG00000015326	+	ORF	*COL1A2*
9	81392308	81392308	+	81392308	9	9	ENSSSCG00000015326	+	3'UTR	*COL1A2*
9	81392479	81392479	+	81392479	17	17	ENSSSCG00000015326	+	3'UTR	*COL1A2*
9	81392582	81392595	+	81392592	39	104	ENSSSCG00000015326	+	3'UTR	*COL1A2*
9	81392921	81392921	+	81392921	9	9	ENSSSCG00000015326	+	3'UTR	*COL1A2*
9	81486544	81486544	+	81486544	4	4	ENSSSCG00000015327	+	intron	*CASD1*
9	81549759	81549760	+	81549759	5	10	ENSSSCG00000015327	+	3'UTR	*CASD1*
9	81654196	81654196	-	81654196	4	4	---	---	intergenic	---
9	81655361	81655362	+	81655362	8	11	XLOC_089925	+	predicted	---
9	81866186	81866186	-	81866186	7	7	---	---	intergenic	---
9	81911132	81911132	-	81911132	3	3	---	---	intergenic	---
9	81929610	81929610	-	81929610	9	9	---	---	intergenic	---
9	82193773	82193773	+	82193773	3	3	ENSSSCG00000029515	+	3'UTR	*PON3*
9	82194454	82194458	+	82194457	5	11	XLOC_089926	+	predicted	---
9	82195534	82195535	+	82195534	10	13	XLOC_089926	+	predicted	---
9	82219032	82219032	+	82219032	3	3	ENSSSCG00000015331	+	intron	*PON2*
9	82224093	82224093	+	82224093	4	4	ENSSSCG00000015331	+	intron	*PON2*
9	82227688	82227688	+	82227688	3	3	ENSSSCG00000015331	+	intron	*PON2*
9	82248064	82248064	-	82248064	3	3	ENSSSCG00000023414	-	3'UTR	---
9	82248654	82248710	-	82248657	120	349	ENSSSCG00000023414	-	3'UTR	---
9	82334686	82334688	+	82334686	3	9	ENSSSCG00000015332	+	ORF	*PON1*
9	82405444	82405452	-	82405446	44	84	ENSSSCG00000027073	-	3'UTR	*PON2*

9	82625076	82625084	-	82625076	7	14	ENSSSCG00000015334	-	3'UTR	*PDK4*
9	82625837	82625837	-	82625837	4	4	ENSSSCG00000015334	-	3'UTR	*PDK4*
9	82914167	82914167	+	82914167	3	3	ENSSSCG00000015335	+	intron	---
9	83173002	83173002	+	83173002	9	9	XLOC_089934	+	predicted	---
9	83193939	83193946	-	83193940	19	31	XLOC_091124	-	predicted	---
9	83194151	83194151	-	83194151	10	10	XLOC_091124	-	predicted	---
9	83962667	83962686	-	83962685	182	311	ENSSSCG00000015337	-	3'UTR	---
9	83998490	83998490	-	83998490	3	3	---	---	intergenic	---
9	84048908	84048908	-	84048908	3	3	---	---	intergenic	---
9	84296963	84296963	-	84296963	4	4	---	---	intergenic	---
9	84433647	84433647	-	84433647	7	7	---	---	intergenic	---
9	84537234	84537247	+	84537246	17	46	XLOC_089938	+	predicted	---
9	84623668	84623668	-	84623668	6	6	---	---	intergenic	---
9	84880368	84880375	-	84880368	12	24	ENSSSCG00000015340	-	3'UTR	*ASNS*
9	84971072	84971073	+	84971073	11	14	---	---	intergenic	---
9	85114373	85114380	+	85114373	9	14	XLOC_089944	+	predicted	---
9	85133810	85133810	-	85133810	4	4	---	---	intergenic	---
9	85224545	85224545	-	85224545	7	7	ENSSSCG00000015342	-	intron	*COL28A1*
9	85523834	85523834	+	85523834	3	3	ENSSSCG00000015343	+	ORF	*MIOS*
9	85562062	85562072	-	85562064	45	90	---	---	intergenic	---
9	85562335	85562359	-	85562338	15	41	XLOC_091132	-	predicted	---
9	85710967	85710968	-	85710967	6	11	---	---	intergenic	---
9	85940487	85940487	+	85940487	5	5	XLOC_089948	+	predicted	---
9	85942743	85942743	+	85942743	16	16	XLOC_089948	+	predicted	---
9	85969142	85969167	-	85969145	40	220	ENSSSCG00000021298	-	3'UTR	---
9	86172570	86172570	-	86172570	3	3	XLOC_091137	-	predicted	---

9	86210060	86210061	+	86210060	3	6	ENSSSCG00000015346	+	intron	---
9	86297473	86297473	+	86297473	3	3	---	---	intergenic	---
9	86940620	86940621	+	86940621	30	33	---	---	intergenic	---
9	87671246	87671246	+	87671246	3	3	---	---	intergenic	---
9	88555978	88555988	-	88555980	97	204	ENSSSCG00000024313	-	3'UTR	*NDUFA4*
9	88608237	88608237	+	88608237	3	3	---	---	intergenic	---
9	88772949	88772949	+	88772949	4	4	ENSSSCG00000026722	+	intron	*PHF14*
9	88853158	88853159	+	88853158	8	12	---	---	intergenic	---
9	89096574	89096575	-	89096574	7	11	ENSSSCG00000015350	-	intron	*THSD7A*
9	89146745	89146745	-	89146745	4	4	ENSSSCG00000015350	-	intron	*THSD7A*
9	89905864	89905864	-	89905864	4	4	---	---	intergenic	---
9	89966974	89966975	-	89966975	6	11	---	---	intergenic	---
9	90022195	90022195	+	90022195	6	6	XLOC_089962	+	predicted	---
9	90022626	90022626	+	90022626	3	3	XLOC_089962	+	predicted	---
9	90023520	90023520	+	90023520	4	4	XLOC_089962	+	predicted	---
9	90027441	90027446	+	90027444	13	35	XLOC_089962	+	predicted	---
9	90348505	90348505	+	90348505	6	6	XLOC_089963	+	predicted	---
9	90367872	90367872	+	90367872	3	3	ENSSSCG00000015352	+	ORF	---
9	90368599	90368644	+	90368642	62	181	XLOC_089964	+	predicted	---
9	90496518	90496520	-	90496520	18	26	---	---	intergenic	---
9	90584985	90584985	+	90584985	57	57	---	---	intergenic	---
9	90652962	90652962	+	90652962	5	5	---	---	intergenic	---
9	90767407	90767407	+	90767407	3	3	---	---	intergenic	---
9	91391306	91391306	-	91391306	15	15	---	---	intergenic	---
9	91502616	91502616	-	91502616	5	5	ENSSSCG00000024591	-	3'UTR	*ETV1*
9	92262597	92262599	-	92262597	16	37	---	---	intergenic	---

9	92262630	92262632	+	92262631	6	14	---	---	intergenic	---
9	92369133	92369133	-	92369133	3	3	ENSSSCG00000015355	-	intron	*DGKB*
9	93083981	93083996	-	93083996	10	13	XLOC_091153	-	predicted	---
9	93129020	93129020	+	93129020	3	3	---	---	intergenic	---
9	93596096	93596096	-	93596096	7	7	XLOC_091155	-	predicted	---
9	93938501	93938501	+	93938501	3	3	XLOC_089970	+	predicted	---
9	94235302	94235302	-	94235302	3	3	---	---	intergenic	---
9	94254527	94254531	-	94254531	14	28	ENSSSCG00000029648	-	intron	---
9	94260130	94260130	-	94260130	4	4	ENSSSCG00000029648	-	intron	---
9	94600201	94600201	-	94600201	3	3	---	---	intergenic	---
9	94658944	94658951	+	94658951	6	9	ENSSSCG00000015361	+	ORF	---
9	94673127	94673136	+	94673128	31	91	---	---	intergenic	---
9	94817944	94817944	+	94817944	6	6	XLOC_089977	+	predicted	---
9	94864176	94864176	-	94864176	3	3	ENSSSCG00000015362	-	3'UTR	*TSPAN13*
9	94864480	94864480	-	94864480	4	4	ENSSSCG00000015362	-	3'UTR	*TSPAN13*
9	94864604	94864604	-	94864604	3	3	ENSSSCG00000015362	-	3'UTR	*TSPAN13*
9	94970160	94970160	-	94970160	3	3	---	---	intergenic	---
9	95070005	95070007	-	95070006	19	31	---	---	intergenic	---
9	95542787	95542787	+	95542787	4	4	XLOC_089980	+	predicted	---
9	95996493	95996493	-	95996493	6	6	ENSSSCG00000024761	-	3'UTR	*SNX13*
9	95999606	95999606	-	95999606	3	3	ENSSSCG00000024761	-	3'UTR	*SNX13*
9	96255844	96255845	-	96255844	7	11	---	---	intergenic	---
9	96258720	96258725	-	96258723	11	30	XLOC_091164	-	predicted	---
9	96684498	96684498	+	96684498	3	3	---	---	intergenic	---
9	98404982	98404982	+	98404982	5	5	---	---	intergenic	---
9	98771858	98771858	+	98771858	3	3	XLOC_089994	+	predicted	---

9	98783503	98783503	+	98783503	3	3	---	---	intergenic	---
9	98802206	98802206	+	98802206	5	5	---	---	intergenic	---
9	98859173	98859174	+	98859174	8	13	---	---	intergenic	---
9	99000154	99000154	+	99000154	37	37	ENSSSCG00000015377	+	intron	*ABCB5*
9	99327823	99327823	+	99327823	3	3	---	---	intergenic	---
9	99342430	99342430	-	99342430	5	5	---	---	intergenic	---
9	99465939	99465950	-	99465939	7	23	XLOC_091178	-	predicted	---
9	99466082	99466082	-	99466082	3	3	XLOC_091178	-	predicted	---
9	99678082	99678082	-	99678082	3	3	---	---	intergenic	---
9	100217610	100217613	-	100217611	8	22	ENSSSCG00000015380	-	3'UTR	*CDCA7L*
9	100361142	100361142	-	100361142	6	6	---	---	intergenic	---
9	100401678	100401684	-	100401684	6	10	XLOC_091181	-	predicted	---
9	100431610	100431610	-	100431610	3	3	ENSSSCG00000015383	-	intron	*RAPGEF5*
9	100886781	100886786	-	100886785	12	27	XLOC_091183	-	predicted	---
9	100891356	100891356	+	100891356	3	3	---	---	intergenic	---
9	100959699	100959699	-	100959699	5	5	---	---	intergenic	---
9	100968624	100968635	+	100968633	120	246	XLOC_090004	+	predicted	---
9	101172617	101172617	+	101172617	4	4	---	---	intergenic	---
9	101466855	101466855	-	101466855	4	4	---	---	intergenic	---
9	101624693	101624693	-	101624693	7	7	---	---	intergenic	---
9	101625999	101625999	-	101625999	3	3	---	---	intergenic	---
9	101650612	101650617	+	101650617	6	12	ENSSSCG00000015387	+	3'UTR	*TRA2A*
9	101651041	101651041	+	101651041	5	5	ENSSSCG00000015387	+	3'UTR	*TRA2A*
9	101651247	101651248	+	101651247	13	18	ENSSSCG00000015387	+	3'UTR	*TRA2A*
9	101862742	101862742	+	101862742	4	4	ENSSSCG00000020870	+	ORF	*DBF4*
9	101871577	101871577	+	101871577	3	3	ENSSSCG00000020870	+	3'UTR	*DBF4*

9	101902051	101902052	-	101902052	6	11	---	---	intergenic	---
9	101902342	101902342	-	101902342	3	3	XLOC_091193	-	predicted	---
9	102338710	102338720	-	102338719	82	140	---	---	intergenic	---
9	102338758	102338758	-	102338758	4	4	XLOC_091196	-	predicted	---
9	102426552	102426552	-	102426552	4	4	XLOC_091197	-	predicted	---
9	102497120	102497120	+	102497120	3	3	---	---	intergenic	---
9	102532997	102532997	-	102532997	14	14	---	---	intergenic	---
9	102626038	102626038	+	102626038	3	3	ENSSSCG00000015390	+	3'UTR	*PGP1A*
9	102626349	102626354	+	102626349	8	15	ENSSSCG00000015390	+	3'UTR	*PGP1A*
9	102740660	102740660	+	102740660	3	3	ENSSSCG00000024520	+	3'UTR	*PGP3*
9	102741976	102741976	+	102741976	3	3	XLOC_090018	+	predicted	---
9	102763201	102763201	-	102763201	3	3	ENSSSCG00000015391	-	ORF	*CROT*
9	102772824	102772824	-	102772824	3	3	ENSSSCG00000015391	-	intron	*CROT*
9	102779236	102779236	-	102779236	19	19	ENSSSCG00000015391	-	ORF	*CROT*
9	102779288	102779288	-	102779288	3	3	ENSSSCG00000015391	-	ORF	*CROT*
9	102781903	102781903	-	102781903	12	12	ENSSSCG00000015391	-	intron	*CROT*
9	102894423	102894423	+	102894423	3	3	ENSSSCG00000025326	+	ORF	*TMEM243*
9	102896729	102896729	+	102896729	6	6	ENSSSCG00000025326	+	3'UTR	*TMEM243*
9	102937770	102937778	-	102937770	23	56	XLOC_091204	-	predicted	---
9	103551770	103551770	+	103551770	3	3	---	---	intergenic	---
9	103677920	103677920	+	103677920	3	3	---	---	intergenic	---
9	103716436	103716436	+	103716436	21	21	---	---	intergenic	---
9	105525522	105525529	+	105525522	3	6	XLOC_090033	+	predicted	---
9	106138599	106138599	+	106138599	8	8	---	---	intergenic	---
9	106209418	106209420	+	106209419	6	15	---	---	intergenic	---
9	106211416	106211423	-	106211416	11	23	XLOC_091211	-	predicted	---

9	106366187	106366187	+	106366187	3	3	---	---	intergenic	---
9	108057305	108057306	-	108057305	12	17	---	---	intergenic	---
9	108471620	108471620	+	108471620	9	9	ENSSSCG00000015402	+	intron	*CACNA2D1*
9	108472251	108472253	+	108472252	9	20	ENSSSCG00000015402	+	intron	*CACNA2D1*
9	108519804	108519804	+	108519804	3	3	ENSSSCG00000015402	+	intron	*CACNA2D1*
9	108521970	108521970	+	108521970	6	6	ENSSSCG00000015402	+	intron	*CACNA2D1*
9	108554990	108554996	+	108554996	14	17	XLOC_090039	+	predicted	---
9	109007328	109007328	-	109007328	13	13	---	---	intergenic	---
9	109391816	109391816	+	109391816	5	5	---	---	intergenic	---
9	109537688	109537688	+	109537688	3	3	---	---	intergenic	---
9	109603091	109603091	+	109603091	9	9	ENSSSCG00000015404	+	intron	---
9	109760685	109760685	-	109760685	3	3	---	---	intergenic	---
9	109983261	109983261	-	109983261	3	3	---	---	intergenic	---
9	109997222	109997222	-	109997222	4	4	---	---	intergenic	---
9	110000823	110000823	-	110000823	4	4	ENSSSCG00000030638	-	ORF	*CH242-168I5.2*
9	110037591	110037591	-	110037591	3	3	XLOC_091217	-	predicted	---
9	110040079	110040079	-	110040079	17	17	ENSSSCG00000015405	-	3'UTR	*CD36*
9	110215622	110215622	-	110215622	4	4	---	---	intergenic	---
9	110430859	110430859	+	110430859	7	7	---	---	intergenic	---
9	110961846	110961847	+	110961846	9	12	---	---	intergenic	---
9	111056196	111056196	+	111056196	3	3	---	---	intergenic	---
9	111461492	111461494	-	111461493	60	104	---	---	intergenic	---
9	111817733	111817733	-	111817733	5	5	---	---	intergenic	---
9	112494264	112494264	+	112494264	6	6	---	---	intergenic	---
9	112741466	112741466	-	112741466	3	3	XLOC_091223	-	predicted	---

9	112788976	112788976	-	112788976	5	5	ENSSSCG00000015410	-	intron	*PHTF2*
9	112812070	112812070	-	112812070	4	4	---	---	intergenic	---
9	112816486	112816486	+	112816486	6	6	---	---	intergenic	---
9	112860269	112860270	+	112860269	3	6	---	---	intergenic	---
9	113019022	113019022	-	113019022	4	4	---	---	intergenic	---
9	113073566	113073567	-	113073567	34	48	---	---	intergenic	---
9	113096373	113096373	+	113096373	3	3	---	---	intergenic	---
9	113110924	113110924	-	113110924	85	85	ENSSSCG00000015413	-	3'UTR	*FGL2*
9	113111961	113111976	-	113111961	21	34	ENSSSCG00000015413	-	3'UTR	*FGL2*
9	113124068	113124085	+	113124069	12	37	ENSSSCG00000015412	+	ORF	*CCDC146*
9	113129880	113129880	+	113129880	3	3	ENSSSCG00000015412	+	ORF	*CCDC146*
9	113130658	113130658	+	113130658	3	3	ENSSSCG00000015412	+	intron	*CCDC146*
9	113148238	113148238	+	113148238	5	5	ENSSSCG00000015412	+	3'UTR	*CCDC146*
9	113148329	113148339	+	113148329	12	21	ENSSSCG00000015412	+	3'UTR	*CCDC146*
9	113150671	113150671	-	113150671	3	3	ENSSSCG00000025166	-	3'UTR	---
9	113845145	113845145	-	113845145	3	3	ENSSSCG00000022903	-	intron	---
9	113930902	113930902	+	113930902	3	3	---	---	intergenic	---
9	113938151	113938161	+	113938152	32	127	ENSSSCG00000015422	+	3'UTR	---
							ENSSSCG00000015422	+	intron	---
9	113938402	113938404	-	113938404	11	15	ENSSSCG00000015423	-	3'UTR	*DNAJC2*
9	113940071	113940071	-	113940071	3	3	ENSSSCG00000015423	-	intron	*DNAJC2*
9	113944761	113944761	-	113944761	3	3	ENSSSCG00000015423	-	ORF	*DNAJC2*
9	113949734	113949735	-	113949734	11	16	ENSSSCG00000015423	-	intron	*DNAJC2*
9	113983871	113983883	+	113983880	57	153	ENSSSCG00000015424	+	3'UTR	*PSMC2*
9	113984282	113984282	+	113984282	6	6	ENSSSCG00000015424	+	3'UTR	*PSMC2*
9	113984555	113984555	+	113984555	16	16	ENSSSCG00000015424	+	3'UTR	*PSMC2*

9	114069880	114069888	-	114069888	13	28	XLOC_091235	-	predicted	---
9	114159035	114159035	-	114159035	4	4	ENSSSCG00000015426	-	intron	*RELN*
9	114477365	114477365	-	114477365	6	6	---	---	intergenic	---
9	114629304	114629304	+	114629304	7	7	---	---	intergenic	---
9	115176684	115176684	-	115176684	4	4	---	---	intergenic	---
9	115405613	115405613	-	115405613	6	6	---	---	intergenic	---
9	115531398	115531400	+	115531398	6	9	XLOC_090073	+	predicted	---
9	115727003	115727003	+	115727003	5	5	XLOC_090076	+	predicted	---
9	115727226	115727226	-	115727226	4	4	XLOC_091240	-	predicted	---
9	115729524	115729524	-	115729524	6	6	---	---	intergenic	---
9	115730727	115730737	-	115730730	19	63	---	---	intergenic	---
9	115752054	115752054	-	115752054	4	4	ENSSSCG00000026110	-	intron	*SRPK2*
9	115839397	115839397	-	115839397	4	4	---	---	intergenic	---
9	115946639	115946639	+	115946639	7	7	---	---	intergenic	---
9	116095213	116095213	-	116095213	8	8	ENSSSCG00000015428	-	3'UTR	*PUS7*
9	116194186	116194194	-	116194191	52	93	XLOC_091243	-	predicted	---
9	116194383	116194383	-	116194383	3	3	ENSSSCG00000029078	-	3'UTR	*EFCAB10*
9	116194633	116194637	-	116194637	31	56	ENSSSCG00000029078	-	3'UTR	*EFCAB10*
9	116390381	116390385	-	116390385	5	8	ENSSSCG00000015432	-	intron	*ATXN7L1*
9	116676909	116676934	+	116676909	36	84	ENSSSCG00000015434	+	3'UTR	---
9	116858943	116858943	+	116858943	4	4	---	---	intergenic	---
9	116890801	116890801	-	116890801	9	9	ENSSSCG00000015435	-	3'UTR	*PBEF1*
9	116891315	116891318	-	116891318	14	18	ENSSSCG00000015435	-	3'UTR	*PBEF1*
9	117029385	117029385	-	117029385	3	3	---	---	intergenic	---
9	117338870	117338873	-	117338873	23	30	XLOC_091251	-	predicted	---
9	117731066	117731068	+	117731067	23	40	---	---	intergenic	---

9	118047381	118047381	+	118047381	3	3	ENSSSCG00000022869	+	intron	*BCAP29*
9	118062512	118062512	+	118062512	4	4	ENSSSCG00000022869	+	intron	*BCAP29*
9	118067520	118067520	+	118067520	3	3	ENSSSCG00000022869	+	intron	*BCAP29*
9	118089516	118089550	+	118089544	16	37	XLOC_090094	+	predicted	---
9	118089650	118089659	+	118089652	88	146	XLOC_090094	+	predicted	---
9	118089789	118089790	+	118089789	4	7	XLOC_090094	+	predicted	---
9	118089962	118089962	+	118089962	3	3	XLOC_090094	+	predicted	---
9	118090042	118090051	+	118090050	11	34	XLOC_090094	+	predicted	---
9	118090483	118090492	+	118090484	15	39	XLOC_090094	+	predicted	---
9	118174044	118174044	+	118174044	10	10	ENSSSCG00000028969	+	intron	*SLC26A4*
9	118233631	118233631	+	118233631	4	4	ENSSSCG00000028585	+	3'UTR	---
							ENSSSCG00000021813	+	intron	*SLC26A3*
9	118235956	118235956	+	118235956	8	8	ENSSSCG00000028585	+	3'UTR	---
							ENSSSCG00000021813	+	intron	*SLC26A3*
9	118437310	118437349	+	118437348	67	98	ENSSSCG00000015443	+	3'UTR	*DLD*
9	118440049	118440056	-	118440049	6	9	ENSSSCG00000015444	-	3'UTR	*LAMB1*
9	118440533	118440533	-	118440533	3	3	ENSSSCG00000015444	-	intron	*LAMB1*
9	118449134	118449134	-	118449134	3	3	ENSSSCG00000015444	-	intron	*LAMB1*
9	118453345	118453345	-	118453345	4	4	ENSSSCG00000015444	-	intron	*LAMB1*
9	118601494	118601494	+	118601494	6	6	---	---	intergenic	---
9	118822272	118822273	-	118822272	3	6	---	---	intergenic	---
9	119034237	119034238	+	119034238	7	10	ENSSSCG00000027699	+	ORF	---
9	119035064	119035078	+	119035078	8	12	ENSSSCG00000027699	+	3'UTR	---
9	119105762	119105771	-	119105763	30	84	ENSSSCG00000015448	-	3'UTR	*NME8*
9	119118836	119118836	-	119118836	3	3	ENSSSCG00000015448	-	intron	*NME8*
9	119118935	119118941	-	119118938	9	30	ENSSSCG00000015448	-	ORF	*NME8*

9	119196951	119196960	+	119196958	87	200	XLOC_090107	+	predicted	---
9	119468549	119468549	+	119468549	7	7	ENSSSCG00000020963	+	3'UTR	*EPDR1*
9	119542538	119542538	-	119542538	6	6	ENSSSCG00000029596	-	3'UTR	---
9	119567513	119567519	-	119567518	8	30	ENSSSCG00000029596	-	intron	---
9	119577348	119577349	+	119577348	19	23	XLOC_090112	+	predicted	---
9	119580479	119580480	-	119580480	12	15	ENSSSCG00000023584	-	3'UTR	---
							ENSSSCG00000029596	-	intron	---
9	119611753	119611753	-	119611753	8	8	XLOC_091275	-	predicted	---
9	119967503	119967504	-	119967504	6	10	---	---	intergenic	---
9	120091436	120091437	+	120091436	26	31	---	---	intergenic	---
9	120110745	120110745	-	120110745	5	5	ENSSSCG00000030294	-	3'UTR	*ZNF398*
9	120190191	120190192	+	120190191	4	7	---	---	intergenic	---
9	120208124	120208124	+	120208124	3	3	ENSSSCG00000015453	+	ORF	*PDIA4*
9	120210543	120210543	+	120210543	3	3	ENSSSCG00000015453	+	ORF	*PDIA4*
9	120216770	120216770	+	120216770	5	5	ENSSSCG00000015453	+	ORF	*PDIA4*
9	120217171	120217181	+	120217178	6	19	ENSSSCG00000015453	+	3'UTR	*PDIA4*
9	120217247	120217249	+	120217248	17	24	ENSSSCG00000015453	+	3'UTR	*PDIA4*
9	120217652	120217660	+	120217653	96	137	ENSSSCG00000015453	+	3'UTR	*PDIA4*
9	120220425	120220425	-	120220425	3	3	---	---	intergenic	---
9	120222952	120222952	+	120222952	3	3	---	---	intergenic	---
9	120225482	120225482	+	120225482	3	3	ENSSSCG00000019375	+	ORF	*Y_RNA*
9	120228856	120228856	-	120228856	34	34	ENSSSCG00000018101	-	ORF	*Y_RNA*
9	120249031	120249031	+	120249031	5	5	XLOC_090120	+	predicted	---
9	120251554	120251554	-	120251554	19	19	ENSSSCG00000018715	-	ORF	*Y_RNA*
9	120265517	120265517	-	120265517	3	3	---	---	intergenic	---
9	120382632	120382632	+	120382632	3	3	ENSSSCG00000028840	+	ORF	*EZH2*

9	120388748	120388756	+	120388754	28	77	ENSSSCG00000028840	+	3'UTR	*EZH2*
9	120392278	120392278	-	120392278	3	3	ENSSSCG00000015455	-	3'UTR	*CUL1*
9	120392339	120392348	-	120392348	9	26	ENSSSCG00000015455	-	3'UTR	*CUL1*
9	120479145	120479145	-	120479145	3	3	---	---	intergenic	---
9	120497070	120497079	+	120497072	6	13	XLOC_090134	+	predicted	---
9	120497186	120497191	+	120497186	7	17	XLOC_090134	+	predicted	---
9	120504067	120504069	-	120504068	6	14	---	---	intergenic	---
9	120552772	120552772	+	120552772	3	3	---	---	intergenic	---
9	120686292	120686292	-	120686292	5	5	ENSSSCG00000022338	-	3'UTR	---
9	121104588	121104613	-	121104589	11	36	ENSSSCG00000015458	-	intron	---
9	121885980	121885980	-	121885980	6	6	---	---	intergenic	---
9	123033589	123033589	-	123033589	3	3	---	---	intergenic	---
9	123894036	123894037	-	123894036	9	12	---	---	intergenic	---
9	124136658	124136660	-	124136659	13	22	---	---	intergenic	---
9	124214697	124214702	-	124214700	15	32	XLOC_091293	-	predicted	---
9	124214831	124214831	-	124214831	4	4	XLOC_091293	-	predicted	---
9	124553211	124553211	+	124553211	6	6	ENSSSCG00000015462	+	intron	*TPK1*
9	124629453	124629453	+	124629453	8	8	ENSSSCG00000015462	+	3'UTR	*TPK1*
9	125095906	125095906	-	125095906	3	3	ENSSSCG00000015472	-	intron	---
9	125185522	125185524	+	125185522	3	6	ENSSSCG00000015473	+	3'UTR	*TMEM183A*
9	125185761	125185771	+	125185763	28	65	ENSSSCG00000015473	+	3'UTR	*TMEM183A*
9	125185837	125185846	+	125185838	11	26	ENSSSCG00000015473	+	3'UTR	*TMEM183A*
9	125186362	125186362	+	125186362	156	156	XLOC_090149	+	predicted	---
9	125187464	125187464	+	125187464	3	3	XLOC_090149	+	predicted	---
9	125331575	125331580	+	125331576	9	34	ENSSSCG00000021059	+	3'UTR	*ADORA1*
9	125331946	125331949	-	125331946	10	15	XLOC_091304	-	predicted	---

9	125344019	125344022	-	125344019	59	69	ENSSSCG00000015476	-	3'UTR	*CHI3L1*
9	125386210	125386210	-	125386210	5	5	XLOC_091306	-	predicted	---
9	125471952	125471952	+	125471952	3	3	ENSSSCG00000015480	+	ORF	*PRRC2C*
9	125485218	125485218	+	125485218	4	4	ENSSSCG00000015480	+	ORF	*PRRC2C*
9	125606177	125606186	-	125606182	40	92	ENSSSCG00000015482	-	3'UTR	*VAMP4*
9	125606700	125606729	-	125606706	17	35	ENSSSCG00000015482	-	3'UTR	*VAMP4*
9	125682291	125682309	+	125682292	12	61	ENSSSCG00000015483	+	3'UTR	*METTL13*
9	126012137	126012137	-	126012137	7	7	---	---	intergenic	---
9	126088517	126088517	+	126088517	14	14	---	---	intergenic	---
9	126241898	126241899	-	126241898	15	20	---	---	intergenic	---
9	126405090	126405091	+	126405091	5	8	---	---	intergenic	---
9	126405277	126405277	+	126405277	3	3	---	---	intergenic	---
9	126420133	126420145	-	126420140	32	104	ENSSSCG00000015486	-	3'UTR	*PIGC*
9	126445639	126445647	+	126445640	44	60	XLOC_090157	+	predicted	---
9	126545747	126545747	+	126545747	4	4	ENSSSCG00000015485	+	ORF	---
9	126577358	126577358	+	126577358	3	3	ENSSSCG00000015485	+	intron	---
9	126602958	126602965	+	126602964	16	34	ENSSSCG00000015485	+	3'UTR	---
9	126604563	126604568	+	126604566	64	92	ENSSSCG00000015485	+	3'UTR	---
9	126650376	126650376	-	126650376	4	4	---	---	intergenic	---
9	127054197	127054199	-	127054197	34	61	---	---	intergenic	---
9	127447689	127447689	-	127447689	3	3	---	---	intergenic	---
9	127460572	127460572	-	127460572	3	3	---	---	intergenic	---
9	127502391	127502396	+	127502395	66	111	ENSSSCG00000022742	+	3'UTR	*PRDX6*
9	127503129	127503136	+	127503129	127	209	ENSSSCG00000022742	+	3'UTR	*PRDX6*
9	127583035	127583035	-	127583035	3	3	XLOC_091321	-	predicted	---
9	127587067	127587098	-	127587067	15	41	XLOC_091321	-	predicted	---

9	127779849	127779849	-	127779849	8	8	XLOC_091323	-	predicted	---
9	127809842	127809842	-	127809842	5	5	XLOC_091323	-	predicted	---
9	127811379	127811379	-	127811379	3	3	XLOC_091323	-	predicted	---
9	127814575	127814576	-	127814575	3	6	XLOC_091323	-	predicted	---
9	127816692	127816692	-	127816692	4	4	ENSSSCG00000015493	-	3'UTR	*SERPINC1*
9	127816734	127816734	-	127816734	15	15	ENSSSCG00000015493	-	3'UTR	*SERPINC1*
9	127816793	127816801	-	127816801	58	93	ENSSSCG00000015493	-	3'UTR	*SERPINC1*
9	127817098	127817122	-	127817115	436	1666	ENSSSCG00000015493	-	3'UTR	*SERPINC1*
9	127817172	127817200	-	127817172	3	6	ENSSSCG00000015493	-	3'UTR	*SERPINC1*
9	127822151	127822151	-	127822151	10	10	ENSSSCG00000015493	-	ORF	*SERPINC1*
9	127825022	127825026	-	127825022	3	6	ENSSSCG00000015493	-	intron	*SERPINC1*
							ENSSSCG00000015493	-	ORF	*SERPINC1*
9	127825076	127825076	-	127825076	3	3	ENSSSCG00000015493	-	ORF	*SERPINC1*
9	127825149	127825179	-	127825156	44	72	ENSSSCG00000015493	-	ORF	*SERPINC1*
9	127825656	127825656	-	127825656	9	9	ENSSSCG00000015493	-	ORF	*SERPINC1*
9	127825786	127825786	-	127825786	6	6	ENSSSCG00000015493	-	ORF	*SERPINC1*
9	127839471	127839471	-	127839471	5	5	XLOC_091326	-	predicted	---
9	127843990	127843990	-	127843990	5	5	XLOC_091326	-	predicted	---
9	127846540	127846541	-	127846541	12	20	XLOC_091326	-	predicted	---
9	127961486	127961495	+	127961489	15	38	---	---	intergenic	---
9	127964128	127964133	+	127964129	14	43	---	---	intergenic	---
9	127964271	127964271	+	127964271	3	3	---	---	intergenic	---
9	128025805	128025805	+	128025805	18	18	XLOC_090168	+	predicted	---
9	128080577	128080577	+	128080577	13	13	ENSSSCG00000015499	+	intron	---
9	128401114	128401114	+	128401114	8	8	---	---	intergenic	---
9	128625842	128625842	+	128625842	3	3	---	---	intergenic	---

9	128715485	128715494	+	128715488	19	25	ENSSSCG00000015503	+	3'UTR	*MRPS14*
9	128738085	128738088	-	128738086	46	95	ENSSSCG00000015504	-	3'UTR	*CACYBP*
9	128738322	128738324	-	128738323	12	25	ENSSSCG00000015504	-	3'UTR	*CACYBP*
9	128738387	128738387	-	128738387	3	3	ENSSSCG00000015504	-	3'UTR	*CACYBP*
9	128742030	128742030	-	128742030	6	6	ENSSSCG00000015504	-	ORF	*CACYBP*
9	128743651	128743651	-	128743651	5	5	ENSSSCG00000015504	-	ORF	*CACYBP*
9	128746733	128746733	-	128746733	3	3	ENSSSCG00000015504	-	ORF	*CACYBP*
9	128773003	128773007	+	128773006	4	11	---	---	intergenic	---
9	128775246	128775253	-	128775253	10	17	ENSSSCG00000023253	-	3'UTR	---
9	128778442	128778442	-	128778442	3	3	ENSSSCG00000023253	-	3'UTR	---
9	128780043	128780043	-	128780043	8	8	ENSSSCG00000023253	-	3'UTR	---
9	128823932	128823936	+	128823932	5	8	XLOC_090171	+	predicted	---
9	128979370	128979370	+	128979370	4	4	ENSSSCG00000015506	+	intron	*TNN*
9	129035796	129035796	-	129035796	3	3	XLOC_091332	-	predicted	---
9	129860478	129860478	-	129860478	3	3	---	---	intergenic	---
9	129968144	129968144	+	129968144	3	3	---	---	intergenic	---
9	130096528	130096530	+	130096530	6	9	ENSSSCG00000015512	+	intron	*PAPPA2*
9	130528814	130528814	-	130528814	4	4	ENSSSCG00000023997	-	intron	---
9	130961268	130961268	+	130961268	3	3	---	---	intergenic	---
9	131369873	131369878	-	131369875	24	47	---	---	intergenic	---
9	131879015	131879015	+	131879015	10	10	ENSSSCG00000015519	+	intron	*RASAL2*
9	131895625	131895625	+	131895625	3	3	ENSSSCG00000015519	+	intron	*RASAL2*
9	132012848	132012848	+	132012848	6	6	XLOC_090192	+	predicted	---
9	132017529	132017536	+	132017529	6	9	XLOC_090192	+	predicted	---
9	132295517	132295517	+	132295517	3	3	---	---	intergenic	---
9	132386997	132386997	+	132386997	4	4	ENSSSCG00000015523	+	intron	*RALGPS2*

9	132494277	132494284	+	132494277	8	14	XLOC_090197	+	predicted	---
9	132497022	132497029	+	132497022	15	25	XLOC_090197	+	predicted	---
9	132499667	132499667	+	132499667	4	4	XLOC_090197	+	predicted	---
9	132628159	132628161	+	132628161	4	7	ENSSSCG00000015524	+	3'UTR	*FAM20B*
9	132629517	132629521	+	132629521	6	11	ENSSSCG00000015524	+	3'UTR	*FAM20B*
9	132660666	132660666	+	132660666	3	3	ENSSSCG00000015525	+	3'UTR	*TOR3A*
9	132663638	132663638	-	132663638	3	3	XLOC_091358	-	predicted	---
9	132663694	132663694	-	132663694	3	3	XLOC_091358	-	predicted	---
9	132762679	132762680	-	132762680	6	9	ENSSSCG00000024674	-	intron	*ABL2*
9	132887472	132887478	-	132887472	15	27	---	---	intergenic	---
9	132888926	132888926	+	132888926	4	4	---	---	intergenic	---
9	132896060	132896060	-	132896060	3	3	ENSSSCG00000021464	-	3'UTR	*SOAT1*
9	132896510	132896510	-	132896510	4	4	ENSSSCG00000021464	-	3'UTR	*SOAT1*
9	133025262	133025263	+	133025262	17	20	XLOC_090204	+	predicted	---
9	133162997	133162998	-	133162997	10	13	---	---	intergenic	---
9	133168473	133168473	-	133168473	4	4	---	---	intergenic	---
9	133195961	133195980	-	133195961	7	15	XLOC_091365	-	predicted	---
9	133196113	133196122	-	133196118	5	14	XLOC_091365	-	predicted	---
9	133200957	133201001	-	133200975	44	113	XLOC_091365	-	predicted	---
9	133351484	133351484	-	133351484	3	3	ENSSSCG00000015531	-	ORF	---
9	133358165	133358165	-	133358165	3	3	ENSSSCG00000015531	-	ORF	---
9	133379303	133379303	-	133379303	3	3	ENSSSCG00000026518	-	intron	---
9	133415438	133415438	+	133415438	4	4	---	---	intergenic	---
9	133415931	133415931	+	133415931	3	3	---	---	intergenic	---
9	133418165	133418167	+	133418166	46	94	ENSSSCG00000029261	+	3'UTR	---
							ENSSSCG00000029261	+	ORF	*protein_codin*

g

9	133430244	133430268	+	133430264	7	33	XLOC_090208	+	predicted	---
9	133430327	133430327	+	133430327	4	4	XLOC_090208	+	predicted	---
9	133430375	133430393	+	133430390	25	110	XLOC_090208	+	predicted	---
9	133431396	133431396	+	133431396	3	3	XLOC_090208	+	predicted	---
9	133431771	133431771	+	133431771	15	15	XLOC_090208	+	predicted	---
9	133470726	133470726	+	133470726	3	3	---	---	intergenic	---
9	133470893	133470893	+	133470893	3	3	---	---	intergenic	---
9	133472339	133472339	+	133472339	3	3	---	---	intergenic	---
9	133489769	133489769	+	133489769	3	3	XLOC_090210	+	predicted	---
9	133496511	133496511	-	133496511	3	3	---	---	intergenic	---
9	133556648	133556655	+	133556649	21	54	ENSSSCG00000030296	+	3'UTR	---
9	133560347	133560353	+	133560347	10	16	XLOC_090210	+	predicted	---
9	133629650	133629650	+	133629650	5	5	ENSSSCG00000015535	+	ORF	*QSOX1*
9	133635518	133635518	+	133635518	3	3	ENSSSCG00000015535	+	intron	*QSOX1*
9	133638603	133638610	+	133638608	110	197	XLOC_090212	+	predicted	---
9	133712062	133712062	+	133712062	4	4	XLOC_090213	+	predicted	---
9	133728814	133728824	-	133728815	23	69	ENSSSCG00000015534	-	3'UTR	*ACBD6*
9	133845594	133845594	-	133845594	4	4	ENSSSCG00000015534	-	ORF	*ACBD6*
9	133924817	133924817	-	133924817	6	6	ENSSSCG00000015534	-	intron	*ACBD6*
9	133928595	133928595	-	133928595	3	3	ENSSSCG00000015534	-	intron	*ACBD6*
9	134332487	134332487	-	134332487	3	3	---	---	intergenic	---
9	134558458	134558460	+	134558459	8	16	ENSSSCG00000015538	+	3'UTR	*KIAA1614*
9	134631218	134631222	-	134631219	18	54	XLOC_091379	-	predicted	---
9	134703871	134703886	-	134703871	31	73	---	---	intergenic	---

9	134703908	134703908	+	134703908	3	3	---	---	intergenic	---
9	134731675	134731682	-	134731682	9	29	XLOC_091381	-	predicted	---
9	134802804	134802804	-	134802804	10	10	---	---	intergenic	---
9	134824290	134824290	+	134824290	3	3	XLOC_090222	+	predicted	---
9	134886820	134886820	+	134886820	3	3	---	---	intergenic	---
9	134911759	134911759	+	134911759	3	3	---	---	intergenic	---
9	134999526	134999526	+	134999526	16	16	---	---	intergenic	---
9	135217310	135217310	+	135217310	3	3	ENSSSCG00000015543	+	intron	*CACNA1E*
9	135633320	135633321	+	135633321	4	7	XLOC_090225	+	predicted	---
9	135634083	135634088	+	135634088	11	16	XLOC_090225	+	predicted	---
9	135639903	135639903	+	135639903	3	3	---	---	intergenic	---
9	135685848	135685863	-	135685849	9	28	XLOC_091384	-	predicted	---
9	135685884	135685887	+	135685885	13	43	---	---	intergenic	---
9	135775152	135775152	-	135775152	3	3	---	---	intergenic	---
9	135821269	135821269	+	135821269	3	3	---	---	intergenic	---
9	135907448	135907463	-	135907461	36	43	XLOC_091387	-	predicted	---
9	135907651	135907655	-	135907654	58	107	XLOC_091387	-	predicted	---
9	135908538	135908540	-	135908538	3	6	XLOC_091387	-	predicted	---
9	135909153	135909178	-	135909153	8	19	ENSSSCG00000015545	-	3'UTR	*GLUL*
9	136133218	136133218	+	136133218	3	3	XLOC_090228	+	predicted	---
9	136208330	136208330	-	136208330	12	12	XLOC_091390	-	predicted	---
9	136227205	136227212	-	136227205	9	19	---	---	intergenic	---
9	136261506	136261506	+	136261506	3	3	XLOC_090230	+	predicted	---
9	136355596	136355596	+	136355596	3	3	---	---	intergenic	---
9	136391895	136391897	-	136391896	99	107	---	---	intergenic	---
9	136414451	136414451	-	136414451	4	4	ENSSSCG00000015553	-	ORF	---

9	136415377	136415377	-	136415377	3	3	ENSSSCG00000015553	-	ORF	---
9	136441768	136441768	+	136441768	4	4	---	---	intergenic	---
9	136444040	136444047	+	136444040	6	9	---	---	intergenic	---
9	136545711	136545711	-	136545711	6	6	XLOC_091394	-	predicted	---
9	136548245	136548245	-	136548245	3	3	XLOC_091394	-	predicted	---
9	136553012	136553012	-	136553012	3	3	ENSSSCG00000015555	-	ORF	*LAMC1*
9	136557886	136557897	-	136557886	14	20	ENSSSCG00000015555	-	intron	*LAMC1*
9	136686181	136686181	-	136686181	6	6	---	---	intergenic	---
9	136809815	136809818	-	136809815	3	6	XLOC_091396	-	predicted	---
9	136813443	136813443	-	136813443	4	4	XLOC_091396	-	predicted	---
9	136816185	136816185	-	136816185	6	6	ENSSSCG00000015557	-	ORF	*NMNAT2*
9	137103823	137103826	+	137103826	7	15	XLOC_090239	+	predicted	---
9	137106507	137106516	-	137106516	7	18	XLOC_091397	-	predicted	---
9	137168062	137168072	-	137168067	75	169	ENSSSCG00000015560	-	3'UTR	*ARPC5*
9	137168315	137168316	-	137168316	10	13	ENSSSCG00000015560	-	3'UTR	*ARPC5*
9	137168358	137168358	-	137168358	4	4	ENSSSCG00000015560	-	3'UTR	*ARPC5*
9	137240097	137240097	-	137240097	3	3	---	---	intergenic	---
9	137240124	137240124	+	137240124	4	4	---	---	intergenic	---
9	137338476	137338486	-	137338486	15	45	---	---	intergenic	---
9	137342276	137342276	+	137342276	5	5	---	---	intergenic	---
9	137425355	137425355	+	137425355	4	4	ENSSSCG00000015563	+	intron	*RGL1*
9	137470900	137470900	+	137470900	3	3	ENSSSCG00000015563	+	ORF	*RGL1*
9	137471187	137471187	+	137471187	5	5	XLOC_090241	+	predicted	---
9	137471274	137471281	+	137471279	12	47	XLOC_090241	+	predicted	---
9	137472485	137472492	+	137472492	8	15	XLOC_090241	+	predicted	---
9	137842452	137842452	+	137842452	3	3	---	---	intergenic	---

9	138217177	138217181	+	138217181	6	9	ENSSSCG00000015565	+	3'UTR	*C1orf21*
9	138358620	138358620	-	138358620	7	7	---	---	intergenic	---
9	138452136	138452136	-	138452136	20	20	XLOC_091406	-	predicted	---
9	138728720	138728736	+	138728721	25	132	ENSSSCG00000015568	+	3'UTR	*RNF2*
9	138748273	138748273	-	138748273	5	5	XLOC_091407	-	predicted	---
9	138749126	138749128	-	138749128	4	7	XLOC_091407	-	predicted	---
9	138900697	138900697	+	138900697	3	3	XLOC_090250	+	predicted	---
9	138906522	138906522	-	138906522	3	3	XLOC_091408	-	predicted	---
9	138906569	138906581	-	138906569	3	6	XLOC_091408	-	predicted	---
9	138907219	138907219	-	138907219	4	4	XLOC_091408	-	predicted	---
9	138907444	138907454	-	138907449	12	23	XLOC_091408	-	predicted	---
9	138907581	138907588	-	138907584	34	73	ENSSSCG00000015570	-	3'UTR	*IVNS1ABP*
9	138907789	138907789	-	138907789	3	3	ENSSSCG00000015570	-	ORF	*IVNS1ABP*
9	138979780	138979782	-	138979781	5	12	XLOC_091409	-	predicted	---
9	139163986	139163986	-	139163986	5	5	---	---	intergenic	---
9	139286488	139286488	+	139286488	3	3	---	---	intergenic	---
9	139695428	139695436	+	139695428	5	26	XLOC_090256	+	predicted	---
9	139933091	139933099	-	139933098	20	38	ENSSSCG00000015576	-	3'UTR	*TPR*
9	139965655	139965655	-	139965655	3	3	ENSSSCG00000015576	-	intron	*TPR*
9	139974272	139974280	-	139974274	11	24	ENSSSCG00000015576	-	intron	*TPR*
9	139981932	139981932	-	139981932	3	3	ENSSSCG00000015576	-	ORF	*TPR*
9	140033092	140033092	+	140033092	9	9	ENSSSCG00000015577	+	3'UTR	*C1orf27*
9	140586899	140586899	+	140586899	3	3	ENSSSCG00000023351	+	intron	*PLA2G4A*
9	140623411	140623411	+	140623411	3	3	ENSSSCG00000023351	+	3'UTR	*PLA2G4A*
9	141418614	141418635	-	141418618	37	262	---	---	intergenic	---
9	141418698	141418699	+	141418698	17	27	---	---	intergenic	---

9	141647101	141647106	+	141647106	15	27	XLOC_090266	+	predicted	---
9	141658339	141658341	+	141658339	5	8	---	---	intergenic	---
9	141728650	141728650	-	141728650	3	3	ENSSSCG00000015581	-	intron	*CENPF*
9	141780514	141780514	-	141780514	3	3	---	---	intergenic	---
9	141952122	141952122	-	141952122	3	3	---	---	intergenic	---
9	142151833	142151835	+	142151834	41	56	---	---	intergenic	---
9	142158967	142158968	-	142158968	8	12	ENSSSCG00000015583	-	3'UTR	*SMYD2*
9	142424390	142424391	-	142424391	9	14	XLOC_091431	-	predicted	---
9	142424602	142424602	-	142424602	7	7	XLOC_091431	-	predicted	---
9	142558676	142558676	+	142558676	3	3	---	---	intergenic	---
9	142693668	142693668	+	142693668	3	3	---	---	intergenic	---
9	143096063	143096063	-	143096063	3	3	---	---	intergenic	---
9	143279840	143279840	+	143279840	10	10	ENSSSCG00000015586	+	3'UTR	*RPS6KC1*
9	143398319	143398319	+	143398319	13	13	ENSSSCG00000015588	+	3'UTR	*ANGEL2*
9	143400799	143400806	-	143400800	16	26	XLOC_091434	-	predicted	---
9	143403496	143403498	-	143403497	5	13	XLOC_091434	-	predicted	---
9	143443143	143443143	-	143443143	3	3	---	---	intergenic	---
9	143468208	143468208	-	143468208	5	5	ENSSSCG00000015590	-	3'UTR	*FLVCR1*
9	143523180	143523182	+	143523181	25	33	ENSSSCG00000015591	+	ORF	*SPATA45*
9	143523988	143523995	+	143523991	38	153	XLOC_090283	+	predicted	---
9	143539884	143539885	-	143539884	9	16	ENSSSCG00000015592	-	intron	*TATDN3*
9	143593031	143593031	+	143593031	3	3	ENSSSCG00000015593	+	3'UTR	*NSL1*
9	143635812	143635821	+	143635821	8	14	ENSSSCG00000015594	+	3'UTR	---
9	143647158	143647168	-	143647167	29	93	XLOC_091440	-	predicted	---
9	143703864	143703884	+	143703879	11	25	XLOC_090285	+	predicted	---
9	143713782	143713782	-	143713782	5	5	---	---	intergenic	---

9	143996002	143996002	-	143996002	25	25	ENSSSCG00000015597	-	3'UTR	*NENF*
9	144051577	144051588	-	144051587	35	102	ENSSSCG00000015599	-	3'UTR	*PPP2R5A*
9	144052438	144052438	-	144052438	7	7	ENSSSCG00000015599	-	3'UTR	*PPP2R5A*
9	144052606	144052607	-	144052606	11	18	ENSSSCG00000015599	-	3'UTR	*PPP2R5A*
9	144053837	144053839	-	144053837	3	6	ENSSSCG00000015599	-	ORF	*PPP2R5A*
9	144272746	144272746	-	144272746	3	3	ENSSSCG00000015600	-	3'UTR	*DTL*
9	144424083	144424083	+	144424083	3	3	---	---	intergenic	---
9	144453994	144453994	+	144453994	4	4	---	---	intergenic	---
9	144510501	144510539	+	144510501	7	10	ENSSSCG00000015603	+	ORF	*LPGAT1*
9	144538851	144538851	+	144538851	6	6	XLOC_090295	+	predicted	---
9	144540916	144540922	+	144540922	13	19	XLOC_090295	+	predicted	---
9	144543173	144543179	+	144543173	19	51	XLOC_090295	+	predicted	---
9	144543664	144543664	+	144543664	3	3	XLOC_090295	+	predicted	---
9	144543736	144543736	+	144543736	5	5	XLOC_090295	+	predicted	---
9	144544672	144544680	+	144544672	3	6	XLOC_090295	+	predicted	---
9	144576942	144576942	-	144576942	7	7	XLOC_091454	-	predicted	---
9	144587782	144587782	+	144587782	3	3	ENSSSCG00000015604	+	ORF	*NEK2*
9	144592699	144592699	+	144592699	3	3	ENSSSCG00000015604	+	intron	*NEK2*
9	144599138	144599150	+	144599144	89	263	ENSSSCG00000015604	+	3'UTR	*NEK2*
9	144677619	144677624	+	144677619	20	32	XLOC_090302	+	predicted	---
9	144749866	144749866	+	144749866	3	3	ENSSSCG00000024619	+	intron	*RD3*
9	144770503	144770503	-	144770503	3	3	XLOC_091460	-	predicted	---
9	144770610	144770628	-	144770628	69	107	XLOC_091460	-	predicted	---
9	144786264	144786264	-	144786264	19	19	XLOC_091460	-	predicted	---
9	144798884	144798892	-	144798889	17	35	---	---	intergenic	---
9	144894517	144894517	-	144894517	189	189	ENSSSCG00000021068	-	intron	*TRAF5*

9	144950977	144950978	-	144950978	8	11	ENSSSCG00000028293	-	intron	*RCOR3*
9	144998581	144998581	-	144998581	3	3	---	---	intergenic	---
9	144999523	144999524	-	144999524	5	8	---	---	intergenic	---
9	145000499	145000499	-	145000499	4	4	---	---	intergenic	---
9	145114490	145114490	-	145114490	5	5	---	---	intergenic	---
9	145183564	145183564	-	145183564	3	3	---	---	intergenic	---
9	145272833	145272834	-	145272834	8	12	---	---	intergenic	---
9	145380749	145380757	-	145380757	7	26	ENSSSCG00000015607	-	3'UTR	*HHAT*
9	145528767	145528767	-	145528767	5	5	ENSSSCG00000015607	-	intron	*HHAT*
9	146259064	146259064	+	146259064	5	5	ENSSSCG00000015612	+	3'UTR	*IRF6*
9	146362687	146362687	-	146362687	3	3	---	---	intergenic	---
9	146382511	146382518	-	146382511	7	21	ENSSSCG00000015611	-	3'UTR	---
9	146482285	146482285	-	146482285	3	3	---	---	intergenic	---
9	146494452	146494462	+	146494457	67	128	ENSSSCG00000015617	+	3'UTR	*G0S2*
							ENSSSCG00000015617	+	ORF	---
9	146675835	146675835	-	146675835	4	4	---	---	intergenic	---
9	146840402	146840402	-	146840402	3	3	---	---	intergenic	---
9	146886160	146886160	-	146886160	11	11	---	---	intergenic	---
9	146971796	146971796	+	146971796	8	8	---	---	intergenic	---
9	147250134	147250142	-	147250136	160	268	---	---	intergenic	---
9	147283363	147283363	+	147283363	18	18	---	---	intergenic	---
9	147588904	147588904	-	147588904	3	3	---	---	intergenic	---
9	147800035	147800036	-	147800036	6	9	XLOC_091480	-	predicted	---
9	148015533	148015533	+	148015533	9	9	---	---	intergenic	---
9	148328932	148328932	+	148328932	3	3	ENSSSCG00000026412	+	3'UTR	---
9	148347665	148347666	-	148347666	9	13	XLOC_091484	-	predicted	---

9	148470632	148470634	+	148470633	22	29	ENSSSCG00000015622	+	3'UTR	*CD34*
9	148472976	148472977	-	148472977	23	44	---	---	intergenic	---
9	148558801	148558807	-	148558802	66	95	XLOC_091489	-	predicted	---
9	148787295	148787295	+	148787295	4	4	ENSSSCG00000029907	+	intron	*ABCA13*
9	149047266	149047266	-	149047266	3	3	---	---	intergenic	---
9	149631288	149631291	-	149631290	31	61	ENSSSCG00000015627	-	3'UTR	*ZPBP*
9	149724583	149724583	+	149724583	9	9	XLOC_090345	+	predicted	---
9	150052630	150052632	+	150052630	3	9	XLOC_090351	+	predicted	---
9	150067400	150067400	-	150067400	3	3	ENSSSCG00000023303	-	ORF	*FIGNL1*
9	150076902	150076915	-	150076909	44	81	ENSSSCG00000022009	-	3'UTR	*AADC*
9	150155389	150155389	-	150155389	3	3	ENSSSCG00000022009	-	intron	*AADC*
9	150268562	150268569	-	150268569	6	17	ENSSSCG00000015631	-	3'UTR	*GRB10*
9	150426534	150426535	+	150426534	4	7	XLOC_090354	+	predicted	---
9	150646379	150646379	-	150646379	3	3	XLOC_091502	-	predicted	---
9	151035370	151035370	-	151035370	3	3	---	---	intergenic	---
9	151035394	151035395	+	151035394	4	7	---	---	intergenic	---
9	151216067	151216067	-	151216067	3	3	---	---	intergenic	---
9	151360548	151360552	-	151360549	57	73	---	---	intergenic	---
9	151360577	151360583	+	151360577	3	6	---	---	intergenic	---
9	151365493	151365500	-	151365500	14	24	XLOC_091507	-	predicted	---
9	151371739	151371739	-	151371739	3	3	XLOC_091507	-	predicted	---
9	151561202	151561202	-	151561202	7	7	---	---	intergenic	---
9	151614344	151614344	-	151614344	3	3	XLOC_091510	-	predicted	---
9	152171804	152171804	-	152171804	3	3	---	---	intergenic	---
9	152176061	152176061	-	152176061	5	5	XLOC_091511	-	predicted	---
9	152393586	152393586	+	152393586	3	3	---	---	intergenic	---

9	152590720	152590720	-	152590720	4	4	XLOC_091515	-	predicted	---
9	152604426	152604426	-	152604426	15	15	---	---	intergenic	---
9	152823510	152823510	-	152823510	3	3	---	---	intergenic	---
9	153348321	153348329	-	153348328	69	100	ENSSSCG00000015637	-	3'UTR	*SEC61G*
9	153384379	153384379	-	153384379	4	4	---	---	intergenic	---
10	43985	43985	+	43985	8	8	XLOC_009980	+	predicted	---
10	59214	59214	+	59214	8	8	ENSSSCG00000010795	+	ORF	*NDUFAB1*
10	62590	62612	+	62597	146	348	ENSSSCG00000010795	+	3'UTR	*NDUFAB1*
10	71014	71018	-	71014	4	7	XLOC_010543	-	predicted	---
10	74438	74439	-	74438	10	15	ENSSSCG00000010796	-	3'UTR	*UBFD1*
10	115540	115546	+	115546	4	16	ENSSSCG00000010797	+	3'UTR	*EARS2*
10	149068	149070	+	149068	45	50	ENSSSCG00000010798	+	intron	*GGA2*
10	159170	159170	+	159170	3	3	ENSSSCG00000010798	+	ORF	*GGA2*
10	165438	165460	+	165458	15	48	XLOC_009983	+	predicted	---
10	210063	210063	+	210063	8	8	ENSSSCG00000010799	+	intron	*COG7*
10	216014	216014	+	216014	4	4	ENSSSCG00000010799	+	intron	*COG7*
10	289215	289215	-	289215	3	3	---	---	intergenic	---
10	605970	605976	-	605971	14	21	XLOC_010546	-	predicted	---
10	607771	607772	+	607771	21	28	XLOC_009987	+	predicted	---
10	1040884	1040885	+	1040884	4	7	---	---	intergenic	---
10	1572295	1572295	+	1572295	3	3	---	---	intergenic	---
10	2418432	2418432	-	2418432	4	4	---	---	intergenic	---
10	2543426	2543433	-	2543433	15	28	ENSSSCG00000010806	-	3'UTR	*RGS2*
10	2678127	2678127	-	2678127	4	4	---	---	intergenic	---
10	3526686	3526686	-	3526686	5	5	---	---	intergenic	---
10	3665365	3665365	+	3665365	5	5	---	---	intergenic	---

10	4168340	4168340	-	4168340	6	6	---	---	intergenic	---
10	4295566	4295566	-	4295566	32	32	---	---	intergenic	---
10	4920306	4920306	-	4920306	3	3	---	---	intergenic	---
10	4976154	4976154	-	4976154	3	3	---	---	intergenic	---
10	5014634	5014634	+	5014634	3	3	ENSSSCG00000010809	+	intron	---
10	5244241	5244246	-	5244242	38	106	---	---	intergenic	---
10	5244264	5244281	+	5244275	9	53	---	---	intergenic	---
10	5612647	5612650	-	5612648	11	31	---	---	intergenic	---
10	5612687	5612689	+	5612687	6	16	---	---	intergenic	---
10	5730463	5730463	+	5730463	4	4	---	---	intergenic	---
10	6590647	6590647	+	6590647	6	6	---	---	intergenic	---
10	7022041	7022041	-	7022041	3	3	---	---	intergenic	---
10	7340851	7340851	-	7340851	3	3	---	---	intergenic	---
10	7370022	7370022	-	7370022	8	8	---	---	intergenic	---
10	7428642	7428642	-	7428642	3	3	XLOC_010574	-	predicted	---
10	8218772	8218772	+	8218772	4	4	---	---	intergenic	---
10	8513635	8513637	-	8513635	5	8	---	---	intergenic	---
10	8811573	8811573	-	8811573	4	4	ENSSSCG00000010814	-	intron	*ESRRG*
10	9074926	9074926	+	9074926	3	3	---	---	intergenic	---
10	9651054	9651056	-	9651054	4	7	ENSSSCG00000027207	-	3'UTR	*GPATCH2*
10	9685292	9685292	-	9685292	5	5	ENSSSCG00000027207	-	intron	*GPATCH2*
10	9909007	9909007	+	9909007	5	5	ENSSSCG00000029778	+	intron	*SPATA17*
10	9994103	9994103	-	9994103	3	3	---	---	intergenic	---
10	10035765	10035765	+	10035765	4	4	XLOC_010013	+	predicted	---
10	10083326	10083326	-	10083326	3	3	---	---	intergenic	---
10	10083390	10083394	-	10083390	4	7	---	---	intergenic	---

10	10083768	10083772	-	10083768	4	8	---	---	intergenic	---
10	10089008	10089009	-	10089008	17	27	---	---	intergenic	---
10	10119699	10119699	+	10119699	3	3	---	---	intergenic	---
10	10338080	10338084	+	10338083	7	20	ENSSSCG00000027846	+	3'UTR	*RRP15*
10	10338966	10338966	+	10338966	5	5	ENSSSCG00000027846	+	3'UTR	*RRP15*
10	10515364	10515364	-	10515364	4	4	---	---	intergenic	---
10	10515469	10515469	-	10515469	3	3	---	---	intergenic	---
10	10599301	10599301	-	10599301	3	3	---	---	intergenic	---
10	10602901	10602901	-	10602901	4	4	XLOC_010590	-	predicted	---
10	10626186	10626186	-	10626186	3	3	---	---	intergenic	---
10	11061014	11061022	+	11061014	8	17	XLOC_010021	+	predicted	---
10	11287742	11287742	+	11287742	5	5	---	---	intergenic	---
10	11440441	11440443	-	11440442	9	18	---	---	intergenic	---
10	11611255	11611261	-	11611261	9	12	XLOC_010596	-	predicted	---
10	11663039	11663046	-	11663041	13	29	XLOC_010596	-	predicted	---
10	11680852	11680852	-	11680852	6	6	ENSSSCG00000010821	-	ORF	*EPRS*
10	11730780	11730780	+	11730780	3	3	XLOC_010025	+	predicted	---
10	11740269	11740277	-	11740277	7	17	---	---	intergenic	---
10	11741885	11741885	-	11741885	6	6	ENSSSCG00000010822	-	3'UTR	*BPNT1*
10	11759547	11759547	-	11759547	3	3	ENSSSCG00000010822	-	intron	*BPNT1*
10	11933314	11933324	-	11933324	36	54	ENSSSCG00000027748	-	3'UTR	---
10	12257861	12257889	-	12257868	86	212	XLOC_010604	-	predicted	---
10	12258309	12258310	-	12258309	5	9	XLOC_010604	-	predicted	---
10	12258419	12258419	-	12258419	5	5	XLOC_010604	-	predicted	---
10	12275719	12275744	-	12275719	46	58	ENSSSCG00000010829	-	intron	*MARCH1*
							ENSSSCG00000010826	-	ORF	*MARC2*

10	12317346	12317354	-	12317352	28	56	ENSSSCG00000027882	-	3'UTR	*C1orf115*
							ENSSSCG00000010829	-	intron	*42064*
10	12319547	12319547	-	12319547	5	5	ENSSSCG00000027882	-	3'UTR	*C1orf115*
							ENSSSCG00000010829	-	intron	*42064*
10	12338741	12338741	-	12338741	3	3	ENSSSCG00000010829	-	intron	*MARCH1*
10	12340203	12340203	-	12340203	3	3	ENSSSCG00000010829	-	intron	*MARCH1*
10	12363632	12363640	-	12363634	260	445	XLOC_010608	-	predicted	---
10	12363778	12363778	+	12363778	3	3	XLOC_010035	+	predicted	---
10	12364247	12364257	-	12364254	25	45	XLOC_010608	-	predicted	---
10	12368035	12368036	-	12368035	26	31	XLOC_010608	-	predicted	---
10	12446924	12446924	+	12446924	5	5	---	---	intergenic	---
10	12643732	12643732	+	12643732	4	4	---	---	intergenic	---
10	12796563	12796571	+	12796570	16	34	---	---	intergenic	---
10	12933586	12933586	+	12933586	3	3	---	---	intergenic	---
10	12978112	12978123	-	12978114	12	44	ENSSSCG00000010831	-	3'UTR	*DUSP10*
10	13424920	13424920	-	13424920	10	10	XLOC_010616	-	predicted	---
10	13449700	13449701	-	13449700	9	12	XLOC_010616	-	predicted	---
10	13570758	13570758	+	13570758	6	6	ENSSSCG00000010834	+	intron	*MIA3*
10	13576792	13576793	+	13576792	3	6	ENSSSCG00000010834	+	intron	*MIA3*
10	13583063	13583067	+	13583063	5	9	ENSSSCG00000010834	+	3'UTR	*MIA3*
10	13583276	13583276	+	13583276	7	7	ENSSSCG00000010834	+	3'UTR	*MIA3*
10	13583332	13583335	+	13583335	41	77	ENSSSCG00000010834	+	3'UTR	*MIA3*
10	13585250	13585258	-	13585251	10	22	XLOC_010619	-	predicted	---
10	13680969	13680974	+	13680969	13	17	---	---	intergenic	---
10	13681019	13681059	+	13681054	4	17	---	---	intergenic	---
10	13777002	13777008	-	13777008	6	10	XLOC_010623	-	predicted	---

10	13866745	13866752	+	13866745	7	11	---	---	intergenic	---
10	13906573	13906573	+	13906573	4	4	XLOC_010046	+	predicted	---
10	13937855	13937860	-	13937860	4	7	---	---	intergenic	---
10	14046931	14046931	-	14046931	3	3	---	---	intergenic	---
10	14153869	14153869	+	14153869	9	9	---	---	intergenic	---
10	14352319	14352319	-	14352319	5	5	---	---	intergenic	---
10	14692400	14692400	-	14692400	3	3	---	---	intergenic	---
10	14850138	14850138	-	14850138	4	4	XLOC_010636	-	predicted	---
10	14852880	14852880	-	14852880	5	5	ENSSSCG00000028178	-	3'UTR	*FH*
10	14853001	14853028	-	14853028	31	110	ENSSSCG00000028178	-	3'UTR	*FH*
10	14880877	14880879	+	14880878	10	17	---	---	intergenic	---
10	14906104	14906104	+	14906104	3	3	ENSSSCG00000025686	+	ORF	*KMO*
10	14936651	14936664	+	14936651	4	14	ENSSSCG00000025686	+	3'UTR	*KMO*
10	14936716	14936728	+	14936728	21	59	ENSSSCG00000025686	+	3'UTR	*KMO*
10	14937345	14937355	+	14937345	30	46	ENSSSCG00000025686	+	3'UTR	*KMO*
10	14958857	14958869	-	14958862	8	23	ENSSSCG00000025290	-	intron	*ZNF596*
10	14987168	14987168	-	14987168	5	5	---	---	intergenic	---
10	15053163	15053163	+	15053163	3	3	ENSSSCG00000024484	+	3'UTR	*DEGS1*
10	15053237	15053244	+	15053243	43	93	ENSSSCG00000024484	+	3'UTR	*DEGS1*
10	15128426	15128427	+	15128426	10	13	ENSSSCG00000027103	+	3'UTR	*CNIH4*
10	15136569	15136569	-	15136569	8	8	ENSSSCG00000010849	-	3'UTR	*WDR26*
10	15136805	15136806	-	15136806	5	8	ENSSSCG00000010849	-	3'UTR	*WDR26*
10	15139290	15139291	-	15139290	5	8	ENSSSCG00000010849	-	3'UTR	*WDR26*
10	15140589	15140589	-	15140589	11	11	ENSSSCG00000010849	-	3'UTR	*WDR26*
10	15140915	15140915	-	15140915	4	4	ENSSSCG00000010849	-	3'UTR	*WDR26*
10	15169327	15169327	-	15169327	3	3	ENSSSCG00000010849	-	intron	*WDR26*

10	15211387	15211387	+	15211387	3	3	---	---	intergenic	---
10	15476272	15476276	-	15476276	6	15	---	---	intergenic	---
10	15476298	15476305	+	15476304	37	74	---	---	intergenic	---
10	15555114	15555114	+	15555114	4	4	XLOC_010065	+	predicted	---
10	15645131	15645131	+	15645131	3	3	ENSSSCG00000021310	+	intron	---
10	15663178	15663178	+	15663178	3	3	XLOC_010066	+	predicted	---
10	15687694	15687694	+	15687694	3	3	---	---	intergenic	---
10	15837205	15837205	-	15837205	5	5	---	---	intergenic	---
10	15839070	15839100	-	15839093	14	29	XLOC_010648	-	predicted	---
10	15839246	15839251	-	15839250	83	91	XLOC_010648	-	predicted	---
10	15839300	15839300	-	15839300	4	4	XLOC_010648	-	predicted	---
10	15839549	15839549	-	15839549	4	4	XLOC_010648	-	predicted	---
10	15839596	15839600	-	15839600	6	9	XLOC_010648	-	predicted	---
10	15908273	15908281	+	15908273	5	11	ENSSSCG00000010858	+	3'UTR	*SDE2*
10	15908407	15908410	+	15908409	9	19	ENSSSCG00000010858	+	3'UTR	*SDE2*
10	15941059	15941060	+	15941059	11	19	XLOC_010072	+	predicted	---
10	15947497	15947506	+	15947500	6	13	ENSSSCG00000010855	+	3'UTR	---
10	16015678	16015699	+	16015691	5	12	ENSSSCG00000010854	+	3'UTR	*TMEM63A*
10	16016581	16016625	-	16016587	612	1067	ENSSSCG00000010853	-	3'UTR	*EPHX1*
10	16016979	16016979	-	16016979	11	11	ENSSSCG00000010853	-	ORF	*EPHX1*
10	16018039	16018041	+	16018039	3	6	ENSSSCG00000010854	+	3'UTR	*TMEM63A*
10	16029417	16029418	-	16029417	29	34	ENSSSCG00000010853	-	ORF	*EPHX1*
10	16042973	16042973	-	16042973	8	8	---	---	intergenic	---
10	16065546	16065552	-	16065549	60	140	ENSSSCG00000028491	-	3'UTR	*SRP9*
10	16065647	16065654	-	16065649	52	93	ENSSSCG00000028491	-	3'UTR	*SRP9*
10	16065699	16065700	-	16065699	9	13	ENSSSCG00000028491	-	3'UTR	*SRP9*

10	16066351	16066351	-	16066351	7	7	ENSSSCG00000028491	-	3'UTR	*SRP9*
10	16307253	16307262	-	16307262	8	19	---	---	intergenic	---
10	16307278	16307283	+	16307283	36	68	---	---	intergenic	---
10	16564576	16564576	-	16564576	3	3	ENSSSCG00000020704	-	intron	*LIN9*
10	16598169	16598170	-	16598170	17	20	ENSSSCG00000023660	-	3'UTR	*PARP1*
10	16641982	16641982	+	16641982	19	19	---	---	intergenic	---
10	16754514	16754516	+	16754515	15	42	---	---	intergenic	---
10	16758349	16758350	-	16758349	16	19	ENSSSCG00000022888	-	3'UTR	*ITPKB*
10	16874211	16874211	-	16874211	9	9	XLOC_010656	-	predicted	---
10	16891770	16891777	+	16891772	50	139	ENSSSCG00000010860	+	3'UTR	*PSEN2*
10	16930460	16930461	-	16930460	11	16	ENSSSCG00000010862	-	3'UTR	*CDC42BPA*
10	17114948	17114950	+	17114949	19	36	ENSSSCG00000010861	+	3'UTR	*ADCK3*
10	17356908	17356908	-	17356908	3	3	XLOC_010658	-	predicted	---
10	17432966	17432966	+	17432966	3	3	ENSSSCG00000010863	+	ORF	*AHCTF1*
10	17433779	17433779	+	17433779	3	3	ENSSSCG00000010863	+	ORF	*AHCTF1*
10	17434075	17434075	+	17434075	5	5	ENSSSCG00000010863	+	ORF	*AHCTF1*
10	17440140	17440144	+	17440143	16	26	ENSSSCG00000010863	+	3'UTR	*AHCTF1*
10	17441407	17441407	+	17441407	5	5	XLOC_010090	+	predicted	---
10	17521582	17521582	+	17521582	3	3	XLOC_010092	+	predicted	---
10	17524007	17524019	-	17524019	58	128	ENSSSCG00000010866	-	3'UTR	*SCCPDH*
10	17524471	17524486	-	17524486	8	24	ENSSSCG00000010866	-	3'UTR	*SCCPDH*
10	17945976	17945979	-	17945979	6	9	---	---	intergenic	---
10	18263720	18263720	+	18263720	3	3	ENSSSCG00000010872	+	3'UTR	*AKT3*
							ENSSSCG00000010872	+	intron	*AKT3*
							ENSSSCG00000010872	+	ORF	*AKT3*

10	18264453	18264456	+	18264453	5	8	ENSSSCG00000010872	+	3'UTR	*AKT3*
							ENSSSCG00000010872	+	intron	*AKT3*
							ENSSSCG00000010872	+	ORF	*AKT3*
10	18264559	18264559	+	18264559	5	5	ENSSSCG00000010872	+	3'UTR	*AKT3*
							ENSSSCG00000010872	+	intron	*AKT3*
							ENSSSCG00000010872	+	ORF	*AKT3*
10	18264787	18264787	+	18264787	3	3	ENSSSCG00000010872	+	3'UTR	*AKT3*
							ENSSSCG00000010872	+	intron	*AKT3*
							ENSSSCG00000010872	+	ORF	*AKT3*
10	18266865	18266867	+	18266865	7	15	ENSSSCG00000010872	+	3'UTR	*AKT3*
							ENSSSCG00000010872	+	intron	*AKT3*
							ENSSSCG00000010872	+	ORF	*AKT3*
10	18268463	18268474	-	18268466	39	163	XLOC_010664	-	predicted	---
10	18273108	18273108	-	18273108	3	3	ENSSSCG00000028096	-	intron	---
10	18351211	18351219	-	18351219	6	9	ENSSSCG00000028096	-	intron	---
10	18364182	18364182	-	18364182	3	3	ENSSSCG00000028096	-	ORF	---
10	18367379	18367379	-	18367379	3	3	ENSSSCG00000028096	-	intron	---
10	18398773	18398777	-	18398773	5	15	ENSSSCG00000028096	-	intron	---
10	18464741	18464742	-	18464741	6	9	ENSSSCG00000028096	-	intron	---
10	18690862	18690863	+	18690862	8	14	XLOC_010100	+	predicted	---
10	18708890	18708890	+	18708890	3	3	ENSSSCG00000030502	+	intron	---
10	18753528	18753536	+	18753534	53	109	ENSSSCG00000030502	+	3'UTR	---
10	18755473	18755479	+	18755479	11	14	XLOC_010100	+	predicted	---
10	19058552	19058552	+	19058552	3	3	---	---	intergenic	---
10	19305906	19305906	-	19305906	5	5	---	---	intergenic	---
10	19359699	19359707	+	19359704	16	44	XLOC_010101	+	predicted	---

10	19433952	19433952	-	19433952	6	6	XLOC_010671	-	predicted	---
10	19459477	19459477	+	19459477	3	3	---	---	intergenic	---
10	19578932	19578951	+	19578951	16	23	---	---	intergenic	---
10	19583685	19583686	-	19583686	12	15	ENSSSCG00000010874	-	3'UTR	*ADSS*
10	19657967	19657969	-	19657968	20	29	XLOC_010674	-	predicted	---
10	19674704	19674710	+	19674706	15	50	XLOC_010104	+	predicted	---
10	19696106	19696122	-	19696107	5	12	ENSSSCG00000010876	-	3'UTR	*PNAS-4*
10	19697784	19697804	-	19697784	4	7	ENSSSCG00000010876	-	3'UTR	*PNAS-4*
10	19697883	19697885	-	19697885	12	16	ENSSSCG00000010876	-	3'UTR	*PNAS-4*
10	19699286	19699300	-	19699297	17	46	ENSSSCG00000010876	-	3'UTR	*PNAS-4*
10	19706087	19706088	-	19706087	3	6	ENSSSCG00000010876	-	intron	*PNAS-4*
10	19748540	19748540	-	19748540	4	4	---	---	intergenic	---
10	19797053	19797053	-	19797053	3	3	XLOC_010676	-	predicted	---
10	19835014	19835015	-	19835014	50	60	---	---	intergenic	---
10	19918660	19918661	+	19918661	9	17	ENSSSCG00000010877	+	ORF	*HNRNPU*
10	19919279	19919279	+	19919279	3	3	ENSSSCG00000010877	+	intron	*HNRNPU*
10	19926760	19926760	+	19926760	5	5	ENSSSCG00000010877	+	ORF	*HNRNPU*
10	19942792	19942808	+	19942799	33	118	XLOC_010109	+	predicted	---
10	19950616	19950616	-	19950616	14	14	XLOC_010679	-	predicted	---
10	19950882	19950890	-	19950888	59	90	XLOC_010679	-	predicted	---
10	19954311	19954311	-	19954311	4	4	---	---	intergenic	---
10	19955336	19955338	+	19955337	7	18	---	---	intergenic	---
10	19962809	19962809	-	19962809	5	5	---	---	intergenic	---
10	20007511	20007511	+	20007511	3	3	---	---	intergenic	---
10	20066744	20066744	+	20066744	3	3	ENSSSCG00000010878	+	intron	*EFCAB2*
10	20137095	20137121	+	20137121	14	70	ENSSSCG00000010878	+	3'UTR	*EFCAB2*

10	20217283	20217283	-	20217283	3	3	---	---	intergenic	---
10	20388893	20388897	-	20388893	4	8	---	---	intergenic	---
10	20666712	20666712	+	20666712	6	6	ENSSSCG00000020956	+	3'UTR	---
							ENSSSCG00000010879	+	intron	*KIF26B*
10	20669910	20669910	+	20669910	3	3	ENSSSCG00000010879	+	intron	*KIF26B*
10	20705025	20705025	+	20705025	3	3	ENSSSCG00000010879	+	intron	*KIF26B*
10	21074783	21074784	-	21074783	5	10	---	---	intergenic	---
10	21154585	21154585	-	21154585	4	4	---	---	intergenic	---
10	21471001	21471001	+	21471001	3	3	ENSSSCG00000023775	+	intron	---
10	21539319	21539319	+	21539319	12	12	ENSSSCG00000023775	+	intron	---
10	21563000	21563000	+	21563000	5	5	XLOC_010117	+	predicted	---
10	21700897	21700899	-	21700898	21	34	XLOC_010688	-	predicted	---
10	21739508	21739508	+	21739508	4	4	---	---	intergenic	---
10	21767655	21767655	-	21767655	3	3	XLOC_010691	-	predicted	---
10	21777951	21777952	+	21777951	3	6	---	---	intergenic	---
10	21821759	21821759	+	21821759	99	99	---	---	intergenic	---
10	21849780	21849780	+	21849780	3	3	XLOC_010123	+	predicted	---
10	21915401	21915401	-	21915401	3	3	ENSSSCG00000010886	-	3'UTR	---
10	22084613	22084622	+	22084621	35	81	XLOC_010129	+	predicted	---
10	22103025	22103031	+	22103027	27	59	XLOC_010130	+	predicted	---
10	22128617	22128619	-	22128617	15	29	---	---	intergenic	---
10	22163271	22163271	+	22163271	8	8	---	---	intergenic	---
10	22270325	22270325	-	22270325	13	13	---	---	intergenic	---
10	22600243	22600244	+	22600243	33	37	XLOC_010133	+	predicted	---
10	22602386	22602387	+	22602386	14	17	XLOC_010133	+	predicted	---
10	22602814	22602816	+	22602814	3	6	XLOC_010133	+	predicted	---

10	22821952	22821952	+	22821952	3	3	---	---	intergenic	---
10	22940132	22940141	+	22940132	4	11	---	---	intergenic	---
10	22943157	22943163	-	22943157	21	35	XLOC_010700	-	predicted	---
10	23338204	23338204	-	23338204	4	4	XLOC_010703	-	predicted	---
10	23443629	23443629	-	23443629	3	3	---	---	intergenic	---
10	24034757	24034757	-	24034757	11	11	ENSSSCG00000010892	-	intron	*KCNT2*
10	24355672	24355673	+	24355673	28	34	---	---	intergenic	---
10	24377628	24377628	+	24377628	8	8	ENSSSCG00000010893	+	intron	*CFH*
10	24387654	24387657	+	24387657	16	23	---	---	intergenic	---
10	24399195	24399222	+	24399222	4	7	---	---	intergenic	---
10	24399274	24399275	+	24399275	23	27	---	---	intergenic	---
10	24406851	24406888	+	24406851	10	14	---	---	intergenic	---
10	24461167	24461169	-	24461168	8	14	ENSSSCG00000010894	-	3'UTR	*TP53BP2*
10	24604231	24604231	+	24604231	8	8	ENSSSCG00000024618	+	3'UTR	*FBXO28*
10	24606620	24606624	+	24606623	11	20	ENSSSCG00000024618	+	3'UTR	*FBXO28*
10	24612798	24612798	-	24612798	7	7	XLOC_010708	-	predicted	---
10	24752421	24752422	+	24752421	11	21	---	---	intergenic	---
10	24904380	24904385	+	24904385	8	12	XLOC_010150	+	predicted	---
10	24958441	24958441	-	24958441	4	4	---	---	intergenic	---
10	24969561	24969562	-	24969561	4	7	---	---	intergenic	---
10	25347621	25347624	+	25347623	13	31	ENSSSCG00000010902	+	3'UTR	*C1orf53*
10	25435805	25435805	+	25435805	4	4	---	---	intergenic	---
10	25478950	25478950	+	25478950	4	4	---	---	intergenic	---
10	25491682	25491682	+	25491682	3	3	---	---	intergenic	---
10	25556595	25556595	-	25556595	8	8	---	---	intergenic	---
10	26084056	26084056	+	26084056	3	3	---	---	intergenic	---

10	26158692	26158692	+	26158692	12	12	XLOC_010161	+	predicted	---
10	26281053	26281081	-	26281081	22	65	ENSSSCG00000010907	-	intron	---
10	26314719	26314725	+	26314725	41	53	ENSSSCG00000010908	+	intron	*PTPRC*
10	26331809	26331809	+	26331809	80	80	ENSSSCG00000010908	+	3'UTR	*PTPRC*
							ENSSSCG00000010908	+	ORF	---
10	26332080	26332084	+	26332080	3	6	ENSSSCG00000010908	+	3'UTR	*PTPRC*
							ENSSSCG00000010908	+	ORF	---
10	26332283	26332283	+	26332283	3	3	ENSSSCG00000010908	+	3'UTR	*PTPRC*
							ENSSSCG00000010908	+	ORF	---
10	26334640	26334640	+	26334640	3	3	---	---	intergenic	---
10	26355594	26355594	-	26355594	3	3	---	---	intergenic	---
10	26671185	26671185	-	26671185	3	3	---	---	intergenic	---
10	26833375	26833376	+	26833375	3	6	---	---	intergenic	---
10	26861369	26861369	+	26861369	3	3	---	---	intergenic	---
10	26908031	26908031	+	26908031	3	3	---	---	intergenic	---
10	26959179	26959180	+	26959179	3	6	---	---	intergenic	---
10	27053649	27053649	-	27053649	3	3	---	---	intergenic	---
10	27183870	27183870	+	27183870	3	3	---	---	intergenic	---
10	27311215	27311216	+	27311215	6	10	ENSSSCG00000024975	+	intron	*NR5A2*
10	27424557	27424557	+	27424557	4	4	ENSSSCG00000024975	+	3'UTR	*NR5A2*
10	27426327	27426333	+	27426327	7	14	ENSSSCG00000024975	+	3'UTR	*NR5A2*
10	27490614	27490614	-	27490614	3	3	---	---	intergenic	---
10	27494637	27494637	+	27494637	3	3	---	---	intergenic	---
10	27510761	27510761	-	27510761	4	4	XLOC_010725	-	predicted	---
10	27512776	27512776	-	27512776	3	3	XLOC_010725	-	predicted	---
10	27734060	27734060	-	27734060	3	3	---	---	intergenic	---

10	27746412	27746412	-	27746412	3	3	ENSSSCG00000027314	-	3'UTR	*CAMSAP2*
10	27924524	27924533	-	27924524	8	16	---	---	intergenic	---
10	27934780	27934780	-	27934780	8	8	XLOC_010732	-	predicted	---
10	27957217	27957217	-	27957217	3	3	ENSSSCG00000024784	-	intron	*CACNA1S*
10	28018120	28018120	-	28018120	5	5	ENSSSCG00000027928	-	3'UTR	*TMEM9*
10	28084490	28084490	-	28084490	4	4	XLOC_010734	-	predicted	---
10	28159010	28159013	-	28159012	4	10	XLOC_010735	-	predicted	---
10	28176636	28176665	-	28176665	4	7	ENSSSCG00000021624	-	3'UTR	*LAD1*
10	28197737	28197746	-	28197737	13	25	ENSSSCG00000024061	-	3'UTR	*TNNI1*
10	28232723	28232723	-	28232723	5	5	XLOC_010738	-	predicted	---
10	28233392	28233393	-	28233393	6	9	ENSSSCG00000029359	-	3'UTR	*PHLDA3*
10	28305022	28305025	-	28305025	14	24	ENSSSCG00000030134	-	3'UTR	*IGFN1*
10	28319701	28319701	-	28319701	5	5	---	---	intergenic	---
10	28398051	28398059	+	28398058	20	35	ENSSSCG00000020976	+	3'UTR	*CSRP1*
10	28407284	28407284	-	28407284	6	6	---	---	intergenic	---
10	28639783	28639784	+	28639784	13	20	XLOC_010174	+	predicted	---
10	28699355	28699367	-	28699361	29	59	---	---	intergenic	---
10	28715506	28715516	-	28715516	8	18	XLOC_010747	-	predicted	---
10	28744137	28744138	-	28744137	4	8	XLOC_010750	-	predicted	---
10	28755069	28755069	+	28755069	5	5	XLOC_010177	+	predicted	---
10	28850301	28850304	-	28850301	14	23	ENSSSCG00000010924	-	3'UTR	*UBE2T*
10	28860175	28860176	-	28860176	6	10	---	---	intergenic	---
10	29087664	29087664	-	29087664	5	5	ENSSSCG00000010926	-	3'UTR	*SYT2*
10	29186614	29186622	-	29186622	13	21	XLOC_010753	-	predicted	---
10	29187400	29187401	-	29187400	6	12	ENSSSCG00000022827	-	3'UTR	---
10	29196153	29196153	-	29196153	3	3	---	---	intergenic	---

10	29201690	29201690	+	29201690	3	3	ENSSSCG00000021179	+	intron	---
10	29219319	29219319	-	29219319	3	3	ENSSSCG00000022787	-	3'UTR	*RABIF*
10	29219620	29219635	-	29219634	14	49	ENSSSCG00000022787	-	3'UTR	*RABIF*
10	29227964	29227966	-	29227966	17	27	XLOC_010755	-	predicted	---
10	29265680	29265696	-	29265681	31	45	ENSSSCG00000010930	-	3'UTR	*ADIPOR1*
10	29266346	29266351	-	29266346	11	14	ENSSSCG00000010930	-	3'UTR	*ADIPOR1*
10	29284347	29284357	-	29284350	144	313	ENSSSCG00000010929	-	3'UTR	*CYB5R1*
10	29286231	29286231	-	29286231	4	4	ENSSSCG00000010929	-	intron	*CYB5R1*
10	29289428	29289428	-	29289428	3	3	ENSSSCG00000010929	-	ORF	*CYB5R1*
10	29434306	29434306	-	29434306	3	3	---	---	intergenic	---
10	29514562	29514562	+	29514562	7	7	ENSSSCG00000010934	+	ORF	*HABP4*
10	29531461	29531473	+	29531473	6	10	ENSSSCG00000010934	+	intron	*HABP4*
10	29538318	29538329	+	29538322	98	200	XLOC_010189	+	predicted	---
10	29539562	29539564	+	29539563	23	37	XLOC_010189	+	predicted	---
10	29543473	29543488	-	29543475	6	22	---	---	intergenic	---
10	29574662	29574662	-	29574662	4	4	ENSSSCG00000027557	-	intron	*CDC14B*
10	29639311	29639311	+	29639311	15	15	---	---	intergenic	---
10	29833324	29833324	+	29833324	4	4	---	---	intergenic	---
10	29993350	29993358	+	29993351	21	109	ENSSSCG00000010937	+	intron	*HSD17B3*
10	30167411	30167411	-	30167411	5	5	---	---	intergenic	---
10	30779430	30779430	-	30779430	3	3	---	---	intergenic	---
10	30892547	30892547	+	30892547	4	4	ENSSSCG00000027312	+	intron	*PTCH1*
10	30915227	30915227	+	30915227	3	3	ENSSSCG00000027312	+	3'UTR	*PTCH1*
10	30929320	30929320	+	30929320	4	4	---	---	intergenic	---
10	31339030	31339034	-	31339032	12	34	XLOC_010774	-	predicted	---
10	31410672	31410672	+	31410672	125	125	---	---	intergenic	---

10	31815590	31815590	+	31815590	3	3	ENSSSCG00000010944	+	ORF	*FBP1*
10	31825545	31825545	+	31825545	3	3	ENSSSCG00000010944	+	ORF	*FBP1*
10	31827451	31827451	+	31827451	17	17	ENSSSCG00000010944	+	ORF	*FBP1*
10	31829318	31829318	+	31829318	23	23	ENSSSCG00000010944	+	3'UTR	*FBP1*
10	31829428	31829435	+	31829432	482	762	ENSSSCG00000010944	+	3'UTR	*FBP1*
10	31829755	31829756	+	31829755	12	18	ENSSSCG00000010944	+	3'UTR	*FBP1*
10	31877480	31877480	-	31877480	7	7	ENSSSCG00000026279	-	intron	*DAPK1*
10	31900681	31900681	+	31900681	15	15	ENSSSCG00000010947	+	3'UTR	*FBP2*
10	31907421	31907427	-	31907427	22	54	ENSSSCG00000010948	-	3'UTR	*CTSL*
							ENSSSCG00000026279	-	intron	*DAPK1*
10	31963589	31963593	-	31963592	8	15	ENSSSCG00000026279	-	intron	*DAPK1*
10	32107699	32107699	+	32107699	8	8	---	---	intergenic	---
10	32210551	32210552	-	32210551	5	10	---	---	intergenic	---
10	32360998	32361017	+	32361016	20	28	XLOC_010210	+	predicted	---
10	32613227	32613227	+	32613227	4	4	XLOC_010211	+	predicted	---
10	32613307	32613307	+	32613307	3	3	XLOC_010211	+	predicted	---
10	32614618	32614618	-	32614618	3	3	---	---	intergenic	---
10	32761607	32761607	-	32761607	4	4	XLOC_010786	-	predicted	---
10	32761626	32761626	+	32761626	4	4	---	---	intergenic	---
10	32789371	32789371	-	32789371	6	6	---	---	intergenic	---
10	33103368	33103368	+	33103368	6	6	ENSSSCG00000010952	+	3'UTR	*ZCCHC6*
10	33103430	33103430	+	33103430	4	4	ENSSSCG00000010952	+	3'UTR	*ZCCHC6*
10	33103745	33103745	+	33103745	3	3	ENSSSCG00000010952	+	3'UTR	*ZCCHC6*
10	33103956	33103963	+	33103963	6	10	ENSSSCG00000010952	+	3'UTR	*ZCCHC6*
10	33123596	33123610	+	33123608	312	705	ENSSSCG00000010953	+	3'UTR	*ISCA1*
10	33125500	33125500	+	33125500	3	3	XLOC_010214	+	predicted	---

10	33137575	33137575	+	33137575	3	3	ENSSSCG00000010954	+	ORF	*C9orf153*
10	33141543	33141569	+	33141567	28	69	XLOC_010214	+	predicted	---
10	33144100	33144100	-	33144100	5	5	---	---	intergenic	---
10	33145091	33145091	+	33145091	6	6	XLOC_010214	+	predicted	---
10	33194502	33194502	+	33194502	3	3	ENSSSCG00000010955	+	intron	*GOLM1*
10	33261236	33261236	+	33261236	5	5	XLOC_010215	+	predicted	---
10	33265600	33265600	-	33265600	3	3	ENSSSCG00000010956	-	3'UTR	*NAA35*
10	33267123	33267132	-	33267123	5	12	ENSSSCG00000010956	-	3'UTR	*NAA35*
10	33267255	33267259	-	33267255	3	6	ENSSSCG00000010956	-	3'UTR	*NAA35*
10	33365125	33365127	-	33365127	58	73	---	---	intergenic	---
10	33554388	33554388	+	33554388	4	4	ENSSSCG00000010957	+	ORF	---
10	33666280	33666280	+	33666280	3	3	ENSSSCG00000010957	+	3'UTR	---
10	33666385	33666385	+	33666385	7	7	ENSSSCG00000010957	+	3'UTR	---
10	33944882	33944882	+	33944882	3	3	---	---	intergenic	---
10	33954782	33954782	-	33954782	13	13	XLOC_010796	-	predicted	---
10	34025453	34025454	-	34025453	3	6	ENSSSCG00000022856	-	intron	*NTRK2*
10	34187174	34187174	+	34187174	5	5	XLOC_010218	+	predicted	---
10	34190886	34190886	+	34190886	5	5	XLOC_010218	+	predicted	---
10	34736669	34736669	+	34736669	3	3	ENSSSCG00000010960	+	intron	*SLC28A3*
10	34805523	34805523	+	34805523	4	4	---	---	intergenic	---
10	34851253	34851253	-	34851253	7	7	XLOC_010802	-	predicted	---
10	34901530	34901530	+	34901530	4	4	---	---	intergenic	---
10	34999119	34999126	-	34999119	4	7	ENSSSCG00000021571	-	intron	*KIF27*
10	34999380	34999380	-	34999380	3	3	ENSSSCG00000021571	-	ORF	*KIF27*
10	35025756	35025764	-	35025756	4	7	ENSSSCG00000021571	-	ORF	*KIF27*
10	35071356	35071362	-	35071356	4	7	ENSSSCG00000026291	-	intron	*C9orf64*

10	35114057	35114057	+	35114057	3	3	ENSSSCG00000022760	+	3'UTR	*HNRNPK*
10	35115118	35115118	+	35115118	5	5	XLOC_010222	+	predicted	---
10	35260737	35260737	+	35260737	3	3	ENSSSCG00000026087	+	ORF	*GKAP1*
10	35312090	35312092	+	35312090	5	8	ENSSSCG00000026087	+	intron	*GKAP1*
10	35317627	35317632	+	35317628	28	47	ENSSSCG00000026087	+	3'UTR	*GKAP1*
10	35402431	35402431	+	35402431	4	4	ENSSSCG00000021005	+	3'UTR	*UBQLN1*
10	35402756	35402756	+	35402756	3	3	ENSSSCG00000021005	+	3'UTR	*UBQLN1*
10	35403396	35403396	+	35403396	3	3	ENSSSCG00000021005	+	3'UTR	*UBQLN1*
10	35404191	35404198	+	35404195	37	60	ENSSSCG00000021005	+	3'UTR	*UBQLN1*
10	35458123	35458123	+	35458123	3	3	---	---	intergenic	---
10	35851442	35851442	-	35851442	3	3	---	---	intergenic	---
10	35988901	35988901	+	35988901	3	3	---	---	intergenic	---
10	36194811	36194811	+	36194811	5	5	---	---	intergenic	---
10	36234348	36234380	+	36234376	217	482	ENSSSCG00000010965	+	3'UTR	*CCL21*
10	36252378	36252382	+	36252381	32	63	ENSSSCG00000010966	+	3'UTR	*CCL19*
10	36274925	36274932	+	36274931	14	30	XLOC_010234	+	predicted	---
10	36278894	36278907	-	36278905	20	48	ENSSSCG00000010968	-	3'UTR	---
10	36291030	36291037	-	36291030	27	46	ENSSSCG00000010969	-	3'UTR	*GALT*
10	36303066	36303077	-	36303070	60	209	ENSSSCG00000029899	-	3'UTR	*SIGMAR1*
10	36320931	36320931	+	36320931	7	7	---	---	intergenic	---
10	36328152	36328158	+	36328156	74	189	ENSSSCG00000010972	+	3'UTR	*DCTN3*
10	36331795	36331801	+	36331800	19	35	ENSSSCG00000010973	+	3'UTR	*RPP25L*
							ENSSSCG00000010973	+	ORF	---
10	36332342	36332342	+	36332342	4	4	XLOC_010235	+	predicted	---
10	36433581	36433581	+	36433581	8	8	XLOC_010237	+	predicted	---
10	36433640	36433641	-	36433641	14	17	XLOC_010820	-	predicted	---

10	36588826	36588835	+	36588826	26	34	XLOC_010239	+	predicted	---
10	36604319	36604342	+	36604340	152	298	XLOC_010239	+	predicted	---
10	36609420	36609420	+	36609420	3	3	---	---	intergenic	---
10	36611343	36611349	+	36611349	11	21	---	---	intergenic	---
10	36618565	36618586	-	36618576	34	74	XLOC_010821	-	predicted	---
10	36734202	36734202	+	36734202	4	4	---	---	intergenic	---
10	36740001	36740009	-	36740002	10	21	ENSSSCG00000021761	-	3'UTR	*UBAP1*
							ENSSSCG00000021761	-	intron	*UBAP1*
10	36859640	36859640	-	36859640	4	4	---	---	intergenic	---
10	37089453	37089456	+	37089456	14	33	XLOC_010245	+	predicted	---
10	37090110	37090117	+	37090114	7	21	XLOC_010245	+	predicted	---
10	37093934	37093943	-	37093935	33	69	ENSSSCG00000010987	-	3'UTR	*UBAP2*
10	37297445	37297445	+	37297445	14	14	ENSSSCG00000010990	+	3'UTR	*NOL6*
10	37376594	37376629	+	37376616	222	439	ENSSSCG00000010992	+	3'UTR	*AQP7*
10	37378606	37378606	+	37378606	11	11	XLOC_010250	+	predicted	---
10	37378655	37378655	+	37378655	4	4	XLOC_010250	+	predicted	---
10	37389041	37389058	-	37389054	17	69	ENSSSCG00000010993	-	3'UTR	---
10	37441086	37441087	-	37441087	30	37	XLOC_010836	-	predicted	---
10	37441654	37441664	-	37441664	8	20	XLOC_010836	-	predicted	---
10	37488294	37488309	+	37488309	6	13	ENSSSCG00000010996	+	intron	*BAG1*
10	37497088	37497106	+	37497088	5	8	ENSSSCG00000010996	+	3'UTR	*BAG1*
10	37497839	37497845	+	37497839	19	61	ENSSSCG00000010996	+	3'UTR	*BAG1*
10	37499084	37499084	+	37499084	3	3	ENSSSCG00000010996	+	3'UTR	*BAG1*
10	37503365	37503365	-	37503365	14	14	ENSSSCG00000010997	-	3'UTR	*SPINK4*
10	37513576	37513578	+	37513577	82	94	---	---	intergenic	---
10	37558040	37558052	-	37558040	3	9	XLOC_010839	-	predicted	---

10	37570945	37570949	+	37570947	22	37	XLOC_010252	+	predicted	---
10	37586428	37586428	+	37586428	3	3	ENSSSCG00000010998	+	intron	*B4GALT1*
10	37632706	37632706	+	37632706	3	3	ENSSSCG00000010998	+	3'UTR	*B4GALT1*
10	37635281	37635288	+	37635282	44	76	ENSSSCG00000010998	+	3'UTR	*B4GALT1*
10	37680327	37680333	+	37680331	7	21	ENSSSCG00000010999	+	3'UTR	*SMU1*
10	37682905	37682905	+	37682905	3	3	ENSSSCG00000010999	+	3'UTR	*SMU1*
10	37686861	37686872	-	37686870	47	116	ENSSSCG00000011000	-	3'UTR	*DNAJA1*
10	37687666	37687666	-	37687666	3	3	ENSSSCG00000011000	-	3'UTR	*DNAJA1*
10	37687707	37687735	-	37687721	68	165	ENSSSCG00000011000	-	3'UTR	*DNAJA1*
10	37687945	37687945	-	37687945	4	4	ENSSSCG00000011000	-	ORF	*DNAJA1*
10	37695130	37695139	-	37695130	10	19	ENSSSCG00000011000	-	ORF	*DNAJA1*
10	37696041	37696041	-	37696041	3	3	ENSSSCG00000011000	-	ORF	*DNAJA1*
10	37697933	37697968	-	37697933	13	52	ENSSSCG00000011000	-	intron	*DNAJA1*
10	37698033	37698052	-	37698033	16	26	ENSSSCG00000011000	-	ORF	*DNAJA1*
10	37698296	37698296	-	37698296	4	4	ENSSSCG00000011000	-	ORF	*DNAJA1*
10	37700431	37700432	+	37700431	3	6	---	---	intergenic	---
10	37733073	37733073	+	37733073	3	3	ENSSSCG00000011001	+	intron	*APTX*
10	37823736	37823736	+	37823736	6	6	ENSSSCG00000011001	+	ORF	*APTX*
10	37837107	37837143	+	37837142	28	40	ENSSSCG00000011001	+	3'UTR	*APTX*
10	38102162	38102163	-	38102163	8	11	---	---	intergenic	---
10	38102357	38102357	-	38102357	4	4	---	---	intergenic	---
10	38118367	38118367	+	38118367	7	7	ENSSSCG00000011003	+	3'UTR	*NDUFB6*
10	38118409	38118412	+	38118409	28	62	ENSSSCG00000011003	+	3'UTR	*NDUFB6*
10	38118484	38118495	+	38118489	30	70	ENSSSCG00000011003	+	3'UTR	*NDUFB6*
10	38131104	38131104	+	38131104	3	3	ENSSSCG00000011004	+	3'UTR	*TOPORS*
10	38153614	38153614	+	38153614	4	4	ENSSSCG00000030408	+	ORF	*DDX58*

10	38154754	38154754	+	38154754	3	3	ENSSSCG00000030408	+	intron	*DDX58*
10	38190595	38190600	+	38190600	6	11	ENSSSCG00000030408	+	3'UTR	*DDX58*
10	38194981	38194985	-	38194985	25	34	ENSSSCG00000023807	-	3'UTR	*ACO1*
10	38235708	38235708	-	38235708	11	11	ENSSSCG00000023807	-	ORF	*ACO1*
10	38239714	38239714	-	38239714	5	5	ENSSSCG00000023807	-	ORF	*ACO1*
10	39015888	39015890	+	39015889	13	21	---	---	intergenic	---
10	39067188	39067188	+	39067188	3	3	---	---	intergenic	---
10	39141334	39141334	-	39141334	8	8	---	---	intergenic	---
10	39141604	39141604	-	39141604	3	3	---	---	intergenic	---
10	39142390	39142394	-	39142394	4	7	---	---	intergenic	---
10	39415613	39415613	+	39415613	6	6	---	---	intergenic	---
10	39485072	39485074	+	39485074	18	31	XLOC_010262	+	predicted	---
10	40057653	40057653	-	40057653	4	4	---	---	intergenic	---
10	40259364	40259364	-	40259364	3	3	---	---	intergenic	---
10	40724487	40724487	-	40724487	3	3	---	---	intergenic	---
10	41032832	41032832	-	41032832	3	3	---	---	intergenic	---
10	41833956	41833956	-	41833956	26	26	---	---	intergenic	---
10	42296209	42296213	+	42296212	15	42	XLOC_010272	+	predicted	---
10	42442051	42442051	+	42442051	3	3	---	---	intergenic	---
10	42548193	42548193	+	42548193	5	5	---	---	intergenic	---
10	42653145	42653145	-	42653145	3	3	---	---	intergenic	---
10	42897035	42897036	-	42897036	18	26	---	---	intergenic	---
10	42985062	42985062	+	42985062	3	3	---	---	intergenic	---
10	43181548	43181548	+	43181548	3	3	---	---	intergenic	---
10	43558679	43558682	+	43558681	4	10	ENSSSCG00000011010	+	3'UTR	*C9orf72*
10	43563594	43563594	-	43563594	17	17	---	---	intergenic	---

10	43833976	43833976	-	43833976	7	7	XLOC_010858	-	predicted	---
10	44013057	44013057	-	44013057	4	4	---	---	intergenic	---
10	44060972	44060972	+	44060972	3	3	---	---	intergenic	---
10	44075016	44075016	+	44075016	7	7	XLOC_010282	+	predicted	---
10	44089184	44089184	-	44089184	7	7	---	---	intergenic	---
10	44183671	44183671	+	44183671	3	3	ENSSSCG00000011013	+	intron	*WAC*
							ENSSSCG00000011013	+	ORF	*WAC*
10	44210089	44210092	+	44210089	9	23	ENSSSCG00000011013	+	3'UTR	*WAC*
10	44211864	44211864	+	44211864	8	8	XLOC_010283	+	predicted	---
10	44350266	44350266	-	44350266	4	4	---	---	intergenic	---
10	44425143	44425143	-	44425143	4	4	---	---	intergenic	---
10	44573522	44573529	-	44573527	14	25	XLOC_010863	-	predicted	---
10	44631994	44631994	+	44631994	3	3	---	---	intergenic	---
10	44791634	44791634	+	44791634	3	3	---	---	intergenic	---
10	44928719	44928738	+	44928736	55	95	XLOC_010286	+	predicted	---
10	44976597	44976597	-	44976597	3	3	XLOC_010864	-	predicted	---
10	45058097	45058097	+	45058097	3	3	XLOC_010288	+	predicted	---
10	45197639	45197642	-	45197640	22	39	---	---	intergenic	---
10	45222789	45222789	+	45222789	7	7	ENSSSCG00000011018	+	3'UTR	---
10	45223009	45223009	+	45223009	3	3	ENSSSCG00000011018	+	3'UTR	---
10	45428631	45428631	+	45428631	3	3	XLOC_010290	+	predicted	---
10	45430123	45430129	+	45430128	6	12	XLOC_010290	+	predicted	---
10	45704081	45704081	-	45704081	3	3	XLOC_010870	-	predicted	---
10	45744526	45744533	+	45744526	4	13	---	---	intergenic	---
10	45876766	45876776	-	45876776	31	54	ENSSSCG00000011022	-	3'UTR	*SVIL*
10	45878543	45878543	-	45878543	3	3	ENSSSCG00000011022	-	intron	*SVIL*

10	45950481	45950481	-	45950481	3	3	---	---	intergenic	---
10	45990270	45990270	-	45990270	11	11	ENSSSCG00000011022	-	intron	*SVIL*
10	45996651	45996651	-	45996651	3	3	ENSSSCG00000011022	-	intron	*SVIL*
10	46000623	46000625	-	46000624	29	67	---	---	intergenic	---
10	46122442	46122442	-	46122442	3	3	XLOC_010873	-	predicted	---
10	46149127	46149127	-	46149127	3	3	XLOC_010874	-	predicted	---
10	46171231	46171231	+	46171231	6	6	---	---	intergenic	---
10	46177758	46177758	-	46177758	3	3	---	---	intergenic	---
10	46219111	46219114	-	46219114	6	13	---	---	intergenic	---
10	46823793	46823804	-	46823793	3	9	ENSSSCG00000020757	-	3'UTR	---
10	46916268	46916269	+	46916269	16	20	---	---	intergenic	---
10	46929009	46929009	+	46929009	4	4	---	---	intergenic	---
10	47019393	47019393	-	47019393	5	5	XLOC_010880	-	predicted	---
10	47055063	47055063	-	47055063	7	7	ENSSSCG00000011026	-	3'UTR	*ARHGAP12*
10	47233048	47233055	-	47233054	15	33	ENSSSCG00000011027	-	3'UTR	*KIF5B*
10	47233439	47233441	-	47233439	3	6	ENSSSCG00000011027	-	3'UTR	*KIF5B*
10	47235387	47235387	-	47235387	8	8	ENSSSCG00000011027	-	3'UTR	*KIF5B*
10	47246074	47246074	-	47246074	4	4	ENSSSCG00000011027	-	intron	*KIF5B*
10	47261238	47261238	-	47261238	5	5	ENSSSCG00000011027	-	ORF	*KIF5B*
10	47431323	47431325	-	47431323	12	19	ENSSSCG00000011028	-	3'UTR	*EPC1*
10	47485826	47485834	+	47485827	16	33	ENSSSCG00000022045	+	3'UTR	*PTCHD3*
10	47572543	47572543	+	47572543	4	4	---	---	intergenic	---
10	48074819	48074819	+	48074819	3	3	---	---	intergenic	---
10	48232809	48232809	+	48232809	3	3	ENSSSCG00000011033	+	ORF	*VIM*
10	48235414	48235414	+	48235414	5	5	ENSSSCG00000011033	+	3'UTR	*VIM*
10	48235621	48235623	+	48235622	20	29	ENSSSCG00000011033	+	3'UTR	*VIM*

10	48235662	48235673	+	48235670	489	755	ENSSSCG00000011033	+	3'UTR	*VIM*
10	48337849	48337849	+	48337849	3	3	XLOC_010312	+	predicted	---
10	48504268	48504268	+	48504268	3	3	---	---	intergenic	---
10	48560331	48560344	+	48560335	15	52	---	---	intergenic	---
10	48592801	48592801	-	48592801	4	4	XLOC_010894	-	predicted	---
10	48594458	48594461	-	48594458	10	16	XLOC_010894	-	predicted	---
10	48699709	48699715	+	48699709	5	8	---	---	intergenic	---
10	48913932	48913932	+	48913932	12	12	ENSSSCG00000011038	+	intron	*MRC1*
10	48925888	48925888	+	48925888	3	3	ENSSSCG00000011038	+	intron	*MRC1*
10	48936457	48936457	+	48936457	4	4	ENSSSCG00000011038	+	intron	*MRC1*
10	48960123	48960123	+	48960123	3	3	---	---	intergenic	---
10	49185907	49185907	+	49185907	5	5	---	---	intergenic	---
10	49569445	49569445	+	49569445	4	4	ENSSSCG00000011040	+	intron	*CACNB2*
10	49623605	49623607	+	49623605	3	6	ENSSSCG00000011040	+	3'UTR	*CACNB2*
10	49629042	49629042	-	49629042	3	3	XLOC_010898	-	predicted	---
10	49758697	49758707	-	49758703	47	119	XLOC_010900	-	predicted	---
10	49761933	49761933	-	49761933	4	4	XLOC_010900	-	predicted	---
10	49842762	49842762	-	49842762	4	4	ENSSSCG00000011042	-	intron	---
10	49848638	49848660	-	49848659	18	50	---	---	intergenic	---
10	49996876	49996912	+	49996878	10	19	XLOC_010332	+	predicted	---
10	50123117	50123123	-	50123123	6	12	ENSSSCG00000022953	-	intron	---
10	50163230	50163230	-	50163230	7	7	ENSSSCG00000022953	-	intron	---
10	50194209	50194209	+	50194209	10	10	---	---	intergenic	---
10	50196828	50196829	-	50196828	3	6	---	---	intergenic	---
10	50196843	50196847	+	50196843	4	7	---	---	intergenic	---
10	50758105	50758105	+	50758105	3	3	XLOC_010335	+	predicted	---

10	51104613	51104613	+	51104613	14	14	---	---	intergenic	---
10	51142436	51142439	+	51142437	11	23	---	---	intergenic	---
10	51147953	51147953	+	51147953	3	3	XLOC_010337	+	predicted	---
10	51307775	51307775	-	51307775	3	3	---	---	intergenic	---
10	51424284	51424284	+	51424284	3	3	ENSSSCG00000026499	+	intron	*NMT2*
10	51454238	51454240	-	51454238	9	12	ENSSSCG00000027110	-	3'UTR	*RPP38*
10	51462649	51462649	-	51462649	3	3	---	---	intergenic	---
10	51523887	51523887	-	51523887	5	5	XLOC_010910	-	predicted	---
10	51524884	51524920	-	51524890	667	1613	ENSSSCG00000011050	-	3'UTR	*MEIG1*
10	51524999	51525035	-	51524999	8	17	ENSSSCG00000011050	-	3'UTR	*MEIG1*
10	51529777	51529793	-	51529777	6	14	ENSSSCG00000011050	-	ORF	*MEIG1*
10	51607834	51607834	-	51607834	5	5	XLOC_010911	-	predicted	---
10	51640170	51640180	-	51640180	14	36	ENSSSCG00000011051	-	3'UTR	---
10	51942236	51942236	+	51942236	3	3	XLOC_010346	+	predicted	---
10	51944682	51944682	+	51944682	4	4	XLOC_010346	+	predicted	---
10	52006476	52006476	-	52006476	5	5	---	---	intergenic	---
10	52161786	52161787	+	52161787	5	8	XLOC_010351	+	predicted	---
10	52458773	52458773	+	52458773	5	5	---	---	intergenic	---
10	52805725	52805725	-	52805725	8	8	ENSSSCG00000011057	-	ORF	---
10	52809212	52809212	-	52809212	3	3	ENSSSCG00000011057	-	intron	---
10	52821303	52821305	-	52821303	19	33	ENSSSCG00000011057	-	intron	---
							ENSSSCG00000011057	-	ORF	---
10	52884753	52884759	+	52884758	30	47	ENSSSCG00000026354	+	3'UTR	*PRPF18*
10	52884809	52884816	+	52884816	4	7	ENSSSCG00000026354	+	3'UTR	*PRPF18*
10	52884938	52884941	+	52884939	16	44	ENSSSCG00000026354	+	3'UTR	*PRPF18*
10	52903244	52903244	-	52903244	4	4	XLOC_010923	-	predicted	---

10	53002035	53002036	+	53002036	20	23	ENSSSCG00000011070	+	intron	*MPP7*
10	53015048	53015051	+	53015050	6	18	ENSSSCG00000011070	+	intron	*MPP7*
10	53117974	53117974	+	53117974	11	11	ENSSSCG00000011070	+	intron	*MPP7*
10	53328285	53328285	-	53328285	3	3	---	---	intergenic	---
10	53346652	53346652	-	53346652	3	3	---	---	intergenic	---
10	53371158	53371158	+	53371158	3	3	ENSSSCG00000021311	+	ORF	*ARMC4*
10	53480997	53480997	+	53480997	3	3	ENSSSCG00000011069	+	intron	---
10	53520462	53520462	+	53520462	5	5	ENSSSCG00000011069	+	3'UTR	---
10	53520633	53520633	+	53520633	4	4	XLOC_010358	+	predicted	---
10	53520991	53520991	+	53520991	5	5	XLOC_010358	+	predicted	---
10	53866895	53866912	+	53866900	32	52	ENSSSCG00000028655	+	3'UTR	*RAB18*
10	53867555	53867558	+	53867555	45	95	ENSSSCG00000028655	+	3'UTR	*RAB18*
10	53886982	53886982	+	53886982	4	4	---	---	intergenic	---
10	54023903	54023903	+	54023903	3	3	ENSSSCG00000011066	+	ORF	*YME1L1*
10	54057001	54057001	+	54057001	4	4	ENSSSCG00000011066	+	ORF	*YME1L1*
10	54057063	54057063	+	54057063	5	5	ENSSSCG00000011066	+	intron	*YME1L1*
10	54062271	54062275	+	54062273	56	96	ENSSSCG00000011066	+	3'UTR	*YME1L1*
10	54062940	54062940	+	54062940	3	3	XLOC_010364	+	predicted	---
10	54063144	54063144	+	54063144	6	6	XLOC_010364	+	predicted	---
10	54066324	54066329	+	54066324	3	6	XLOC_010364	+	predicted	---
10	54070893	54070896	-	54070893	6	15	---	---	intergenic	---
10	54130743	54130743	-	54130743	11	11	ENSSSCG00000011068	-	intron	---
10	54189572	54189579	-	54189573	11	26	XLOC_010930	-	predicted	---
10	54199625	54199625	-	54199625	7	7	ENSSSCG00000011064	-	ORF	---
10	54213784	54213784	-	54213784	3	3	ENSSSCG00000011064	-	ORF	---
10	54350125	54350125	+	54350125	7	7	ENSSSCG00000011062	+	intron	*ABI1*

10	54376721	54376721	+	54376721	3	3	ENSSSCG00000030156	+	ORF	*APBB1IP*
10	54430062	54430062	+	54430062	51	51	XLOC_010368	+	predicted	---
10	54485328	54485328	-	54485328	3	3	ENSSSCG00000024127	-	3'UTR	---
10	54485508	54485508	+	54485508	5	5	ENSSSCG00000011061	+	3'UTR	---
10	54486498	54486498	-	54486498	5	5	ENSSSCG00000024127	-	3'UTR	---
10	54486878	54486878	-	54486878	4	4	ENSSSCG00000024127	-	3'UTR	---
10	54487245	54487245	-	54487245	3	3	ENSSSCG00000024127	-	3'UTR	---
10	54548623	54548623	-	54548623	3	3	---	---	intergenic	---
10	54728555	54728555	-	54728555	8	8	---	---	intergenic	---
10	54983426	54983426	-	54983426	110	110	---	---	intergenic	---
10	55025570	55025570	+	55025570	3	3	---	---	intergenic	---
10	55653485	55653485	-	55653485	3	3	---	---	intergenic	---
10	55741717	55741718	+	55741717	4	7	XLOC_010375	+	predicted	---
10	55751300	55751300	+	55751300	3	3	XLOC_010376	+	predicted	---
10	55758596	55758596	+	55758596	5	5	XLOC_010376	+	predicted	---
10	55758939	55758940	+	55758940	4	7	XLOC_010376	+	predicted	---
10	55759022	55759030	+	55759030	6	13	XLOC_010376	+	predicted	---
10	55759097	55759097	+	55759097	6	6	XLOC_010376	+	predicted	---
10	55759561	55759561	+	55759561	3	3	XLOC_010376	+	predicted	---
10	55760216	55760216	+	55760216	4	4	XLOC_010376	+	predicted	---
10	55923998	55923998	-	55923998	5	5	---	---	intergenic	---
10	55936068	55936068	-	55936068	3	3	---	---	intergenic	---
10	56091418	56091418	+	56091418	3	3	---	---	intergenic	---
10	56121075	56121075	+	56121075	5	5	ENSSSCG00000011074	+	intron	*ARHGAP21*
10	56132235	56132239	+	56132235	5	13	XLOC_010381	+	predicted	---
10	56132989	56132996	+	56132989	15	19	XLOC_010381	+	predicted	---

10	56157810	56157810	-	56157810	5	5	XLOC_010948	-	predicted	---
10	56158150	56158159	-	56158159	8	11	XLOC_010948	-	predicted	---
10	56336966	56336969	+	56336966	3	6	---	---	intergenic	---
10	56420189	56420189	+	56420189	8	8	XLOC_010383	+	predicted	---
10	57013524	57013524	+	57013524	4	4	---	---	intergenic	---
10	57069540	57069540	-	57069540	3	3	---	---	intergenic	---
10	57215154	57215154	+	57215154	3	3	---	---	intergenic	---
10	57272983	57272983	-	57272983	5	5	XLOC_010953	-	predicted	---
10	57409137	57409137	+	57409137	4	4	ENSSSCG00000021093	+	intron	*C10orf67*
10	57463933	57463933	+	57463933	4	4	---	---	intergenic	---
10	57512329	57512329	-	57512329	3	3	XLOC_010956	-	predicted	---
10	57514667	57514667	+	57514667	3	3	XLOC_010391	+	predicted	---
10	57541809	57541809	+	57541809	3	3	---	---	intergenic	---
10	57544140	57544141	-	57544141	10	13	ENSSSCG00000011077	-	3'UTR	*MSRB2*
10	57615064	57615075	-	57615074	6	36	XLOC_010958	-	predicted	---
10	57615163	57615163	-	57615163	3	3	XLOC_010958	-	predicted	---
10	57615223	57615223	-	57615223	4	4	XLOC_010958	-	predicted	---
10	57676840	57676841	-	57676840	3	6	ENSSSCG00000011078	-	intron	*ARMC3*
10	58063397	58063404	+	58063397	5	8	ENSSSCG00000011079	+	3'UTR	*PIP4K2A*
10	58064467	58064467	+	58064467	3	3	ENSSSCG00000011079	+	3'UTR	*PIP4K2A*
10	58064681	58064684	+	58064683	12	22	ENSSSCG00000011079	+	3'UTR	*PIP4K2A*
10	58101803	58101816	-	58101814	37	86	XLOC_010962	-	predicted	---
10	58101919	58101922	-	58101921	26	54	XLOC_010962	-	predicted	---
10	58102911	58102922	+	58102912	30	199	XLOC_010394	+	predicted	---
10	58103315	58103316	+	58103315	3	6	XLOC_010394	+	predicted	---
10	58137907	58137930	-	58137922	61	200	XLOC_010962	-	predicted	---

10	58151762	58151780	-	58151774	48	147	ENSSSCG00000011080	-	3'UTR	*SPAG6*
10	58240743	58240745	-	58240743	5	12	ENSSSCG00000011081	-	3'UTR	*BMI1*
10	58240940	58240940	-	58240940	3	3	ENSSSCG00000011081	-	3'UTR	*BMI1*
10	58241161	58241162	-	58241161	4	7	ENSSSCG00000011081	-	3'UTR	*BMI1*
10	58251900	58251903	-	58251901	29	60	XLOC_010965	-	predicted	---
10	58252073	58252088	-	58252075	18	34	ENSSSCG00000011082	-	3'UTR	*COMMD3*
10	58296713	58296715	-	58296714	43	62	---	---	intergenic	---
10	58361426	58361426	+	58361426	4	4	---	---	intergenic	---
10	58596108	58596108	+	58596108	3	3	ENSSSCG00000026001	+	intron	---
10	58612588	58612588	+	58612588	4	4	ENSSSCG00000026001	+	ORF	---
10	58721779	58721779	+	58721779	7	7	ENSSSCG00000026001	+	3'UTR	---
10	58733136	58733140	-	58733136	5	8	XLOC_010971	-	predicted	---
10	58743104	58743104	-	58743104	4	4	ENSSSCG00000011085	-	intron	*MLLT10*
10	58921539	58921539	+	58921539	4	4	ENSSSCG00000029019	+	ORF	---
10	58971721	58971721	-	58971721	3	3	---	---	intergenic	---
10	59085623	59085623	-	59085623	6	6	XLOC_010972	-	predicted	---
10	59367835	59367835	-	59367835	5	5	---	---	intergenic	---
10	59590179	59590179	+	59590179	4	4	---	---	intergenic	---
10	59713966	59713966	+	59713966	3	3	XLOC_010403	+	predicted	---
10	59719060	59719078	+	59719060	4	11	XLOC_010403	+	predicted	---
10	59740653	59740653	+	59740653	3	3	---	---	intergenic	---
10	59742130	59742130	+	59742130	4	4	---	---	intergenic	---
10	59807695	59807695	-	59807695	3	3	---	---	intergenic	---
10	59833334	59833334	-	59833334	6	6	XLOC_010974	-	predicted	---
10	60032853	60032853	-	60032853	3	3	XLOC_010976	-	predicted	---
10	60033247	60033247	-	60033247	6	6	XLOC_010976	-	predicted	---

10	60336241	60336241	+	60336241	3	3	---	---	intergenic	---
10	60840986	60840986	-	60840986	3	3	---	---	intergenic	---
10	60852400	60852400	+	60852400	7	7	---	---	intergenic	---
10	60960111	60960111	-	60960111	3	3	---	---	intergenic	---
10	61070383	61070383	-	61070383	5	5	---	---	intergenic	---
10	61414321	61414332	+	61414321	29	106	XLOC_010410	+	predicted	---
10	61450696	61450713	-	61450703	45	75	ENSSSCG00000011101	-	3'UTR	*ITGB1*
10	61451175	61451176	-	61451175	21	29	ENSSSCG00000011101	-	3'UTR	*ITGB1*
10	61451290	61451290	-	61451290	5	5	ENSSSCG00000011101	-	3'UTR	*ITGB1*
10	61462398	61462398	-	61462398	3	3	ENSSSCG00000011101	-	3'UTR	*ITGB1*
10	61651754	61651764	-	61651764	29	42	ENSSSCG00000011102	-	3'UTR	*NRP1*
10	61718733	61718733	-	61718733	4	4	ENSSSCG00000011102	-	intron	*NRP1*
10	62516139	62516140	-	62516139	3	6	XLOC_010987	-	predicted	---
10	62540783	62540784	-	62540783	3	6	---	---	intergenic	---
10	62591677	62591685	+	62591682	9	40	---	---	intergenic	---
10	62683716	62683716	-	62683716	4	4	ENSSSCG00000011103	-	intron	---
10	62717522	62717522	-	62717522	5	5	ENSSSCG00000011103	-	intron	---
10	62751709	62751709	-	62751709	7	7	ENSSSCG00000011103	-	intron	---
10	62870908	62870909	-	62870909	4	7	ENSSSCG00000011103	-	intron	---
10	62967420	62967423	-	62967420	3	6	ENSSSCG00000011103	-	intron	---
10	63092279	63092279	-	63092279	8	8	---	---	intergenic	---
10	63144177	63144177	-	63144177	3	3	---	---	intergenic	---
10	63270582	63270582	+	63270582	4	4	---	---	intergenic	---
10	63302296	63302298	+	63302296	3	6	ENSSSCG00000011104	+	3'UTR	*CUL2*
10	63475934	63475940	+	63475940	15	33	ENSSSCG00000011106	+	intron	*CREB*
10	63480853	63480853	+	63480853	4	4	ENSSSCG00000011106	+	intron	*CREB*

10	63500116	63500116	+	63500116	8	8	---	---	intergenic	---
10	63503963	63503969	+	63503966	76	137	ENSSSCG00000011106	+	3'UTR	*CREB*
10	63504026	63504026	+	63504026	3	3	ENSSSCG00000011106	+	3'UTR	*CREB*
10	63504229	63504236	+	63504230	14	22	ENSSSCG00000011106	+	3'UTR	*CREB*
10	63504492	63504502	+	63504497	11	33	ENSSSCG00000011106	+	3'UTR	*CREB*
10	63544244	63544244	-	63544244	5	5	---	---	intergenic	---
10	63619942	63619942	-	63619942	4	4	---	---	intergenic	---
10	63659658	63659658	+	63659658	3	3	ENSSSCG00000011107	+	3'UTR	*CCNY*
10	63659729	63659771	+	63659762	97	368	ENSSSCG00000011107	+	3'UTR	*CCNY*
10	63660256	63660256	+	63660256	4	4	ENSSSCG00000011107	+	3'UTR	*CCNY*
10	63661223	63661223	+	63661223	15	15	ENSSSCG00000011107	+	3'UTR	*CCNY*
10	64069094	64069100	+	64069100	6	9	---	---	intergenic	---
10	64652337	64652337	+	64652337	3	3	---	---	intergenic	---
10	64680928	64680929	-	64680928	12	15	---	---	intergenic	---
10	64893449	64893450	-	64893450	11	14	---	---	intergenic	---
10	65115113	65115113	+	65115113	3	3	---	---	intergenic	---
10	65151585	65151591	+	65151591	4	7	---	---	intergenic	---
10	65152444	65152444	+	65152444	5	5	---	---	intergenic	---
10	65482555	65482556	+	65482556	8	12	---	---	intergenic	---
10	65505121	65505150	-	65505140	12	39	ENSSSCG00000011112	-	3'UTR	---
10	65574094	65574094	+	65574094	4	4	---	---	intergenic	---
10	65574253	65574262	+	65574260	100	152	ENSSSCG00000011113	+	3'UTR	---
10	65576996	65576996	-	65576996	8	8	ENSSSCG00000011114	-	3'UTR	*SEC61A2*
10	65577052	65577055	-	65577055	14	18	ENSSSCG00000011114	-	3'UTR	*SEC61A2*
10	65577874	65577875	-	65577875	4	7	ENSSSCG00000011114	-	3'UTR	*SEC61A2*
10	65607339	65607346	-	65607345	6	15	---	---	intergenic	---

10	65693810	65693810	+	65693810	4	4	XLOC_010431	+	predicted	---
10	65694693	65694693	+	65694693	9	9	XLOC_010431	+	predicted	---
10	65735109	65735109	-	65735109	4	4	ENSSSCG00000022247	-	3'UTR	*PROSER2*
10	65778995	65778997	+	65778997	83	155	---	---	intergenic	---
10	65788470	65788470	+	65788470	9	9	XLOC_010433	+	predicted	---
10	65800701	65800701	+	65800701	4	4	ENSSSCG00000011119	+	intron	*ECHDC3*
10	65802105	65802105	+	65802105	3	3	ENSSSCG00000011119	+	intron	*ECHDC3*
10	65809766	65809766	+	65809766	9	9	ENSSSCG00000011119	+	3'UTR	*ECHDC3*
10	65810362	65810363	+	65810363	5	8	ENSSSCG00000011119	+	3'UTR	*ECHDC3*
10	65810791	65810797	+	65810791	42	63	ENSSSCG00000011119	+	3'UTR	*ECHDC3*
10	65938416	65938417	-	65938416	5	8	---	---	intergenic	---
10	66240975	66240975	+	66240975	3	3	XLOC_010437	+	predicted	---
10	66241168	66241168	+	66241168	3	3	XLOC_010437	+	predicted	---
10	66358584	66358584	-	66358584	4	4	---	---	intergenic	---
10	66363056	66363056	-	66363056	3	3	XLOC_011010	-	predicted	---
10	66914809	66914809	-	66914809	3	3	---	---	intergenic	---
10	67218908	67218919	-	67218909	17	29	XLOC_011014	-	predicted	---
10	67726477	67726477	-	67726477	10	10	---	---	intergenic	---
10	68323540	68323558	+	68323540	7	18	XLOC_010444	+	predicted	---
10	69117950	69117952	-	69117951	15	23	XLOC_011018	-	predicted	---
10	69139658	69139658	-	69139658	5	5	---	---	intergenic	---
10	69191531	69191531	+	69191531	5	5	XLOC_010447	+	predicted	---
10	69195551	69195551	+	69195551	3	3	XLOC_010447	+	predicted	---
10	69277212	69277212	-	69277212	3	3	XLOC_011020	-	predicted	---
10	69280524	69280524	+	69280524	5	5	---	---	intergenic	---
10	69461825	69461825	+	69461825	3	3	---	---	intergenic	---

10	69669516	69669516	-	69669516	23	23	ENSSSCG00000011127	-	ORF	*TAF3*
10	69733474	69733474	-	69733474	11	11	ENSSSCG00000011128	-	ORF	*ITIH2*
10	69736083	69736084	-	69736084	47	51	ENSSSCG00000011128	-	ORF	*ITIH2*
10	69738085	69738086	-	69738085	3	6	ENSSSCG00000011128	-	intron	*ITIH2*
10	69842743	69842791	+	69842761	12	41	ENSSSCG00000011129	+	3'UTR	---
10	69887384	69887384	+	69887384	3	3	XLOC_010452	+	predicted	---
10	70126590	70126590	+	70126590	3	3	---	---	intergenic	---
10	70250379	70250379	+	70250379	4	4	---	---	intergenic	---
10	70309001	70309001	+	70309001	3	3	---	---	intergenic	---
10	70465455	70465455	-	70465455	3	3	---	---	intergenic	---
10	70658945	70658945	+	70658945	3	3	XLOC_010457	+	predicted	---
10	70659128	70659135	+	70659134	27	81	XLOC_010457	+	predicted	---
10	70681332	70681332	-	70681332	3	3	---	---	intergenic	---
10	70742354	70742355	-	70742355	9	15	---	---	intergenic	---
10	70774578	70774579	-	70774578	5	8	---	---	intergenic	---
10	70857732	70857732	+	70857732	3	3	XLOC_010458	+	predicted	---
10	70864854	70864854	-	70864854	3	3	---	---	intergenic	---
10	70943098	70943099	-	70943099	11	14	XLOC_011039	-	predicted	---
10	70943164	70943169	-	70943165	39	63	ENSSSCG00000011134	-	3'UTR	*RBM17*
10	70987694	70987697	+	70987694	7	11	---	---	intergenic	---
10	71085478	71085486	+	71085484	61	113	XLOC_010461	+	predicted	---
10	71237927	71237927	+	71237927	5	5	ENSSSCG00000028790	+	3'UTR	*ANKRD16*
10	71290310	71290321	+	71290321	12	18	ENSSSCG00000011137	+	intron	*GDI2*
10	71307595	71307595	+	71307595	4	4	ENSSSCG00000011137	+	ORF	*GDI2*
10	71311340	71311340	+	71311340	4	4	ENSSSCG00000011137	+	ORF	*GDI2*
10	71312183	71312195	+	71312186	205	334	ENSSSCG00000011137	+	3'UTR	*GDI2*

10	71313322	71313323	-	71313323	20	25	XLOC_011049	-	predicted	---
10	71359757	71359770	+	71359760	9	30	XLOC_010467	+	predicted	---
10	71362193	71362197	+	71362195	37	54	XLOC_010467	+	predicted	---
10	71416918	71416927	+	71416919	7	28	ENSSSCG00000011145	+	3'UTR	*ASB13*
10	71541975	71541978	-	71541977	27	48	XLOC_011056	-	predicted	---
10	71542460	71542460	-	71542460	4	4	XLOC_011056	-	predicted	---
10	71542739	71542743	-	71542739	14	20	XLOC_011056	-	predicted	---
10	71684061	71684061	-	71684061	3	3	---	---	intergenic	---
10	71835676	71835676	+	71835676	8	8	ENSSSCG00000011147	+	ORF	*AKR1C1*
10	71847738	71847762	+	71847754	117	229	ENSSSCG00000011147	+	3'UTR	*AKR1C1*
10	71989823	71989827	-	71989827	12	19	---	---	intergenic	---
10	72341169	72341170	-	72341170	10	13	---	---	intergenic	---
10	72356309	72356309	-	72356309	3	3	---	---	intergenic	---
10	72393394	72393398	-	72393395	20	31	---	---	intergenic	---
10	72405285	72405285	-	72405285	4	4	XLOC_011065	-	predicted	---
10	72495017	72495017	-	72495017	4	4	---	---	intergenic	---
10	72902767	72902768	+	72902767	29	33	---	---	intergenic	---
10	72937896	72937903	-	72937903	18	43	XLOC_011071	-	predicted	---
10	73001800	73001801	+	73001800	14	18	ENSSSCG00000028828	+	3'UTR	*KLF6*
10	73121699	73121708	-	73121706	46	80	---	---	intergenic	---
10	73166052	73166060	-	73166058	11	18	XLOC_011076	-	predicted	---
10	73300086	73300086	+	73300086	3	3	---	---	intergenic	---
10	73794302	73794302	-	73794302	4	4	---	---	intergenic	---
10	74087436	74087436	+	74087436	3	3	XLOC_010504	+	predicted	---
10	74117712	74117712	+	74117712	5	5	---	---	intergenic	---
10	74128822	74128822	-	74128822	3	3	---	---	intergenic	---

10	74576832	74576832	+	74576832	3	3	---	---	intergenic	---
10	75051406	75051406	+	75051406	4	4	---	---	intergenic	---
10	75205667	75205667	+	75205667	6	6	ENSSSCG00000011160	+	intron	*ADARB2*
10	75286298	75286298	+	75286298	3	3	ENSSSCG00000011160	+	intron	*ADARB2*
10	75370777	75370807	+	75370782	89	349	ENSSSCG00000029066	+	3'UTR	---
10	75588541	75588541	-	75588541	4	4	---	---	intergenic	---
10	75711017	75711020	+	75711019	10	29	ENSSSCG00000011162	+	3'UTR	*LARP4B*
10	76004740	76004740	+	76004740	5	5	---	---	intergenic	---
10	76212773	76212773	+	76212773	3	3	XLOC_010520	+	predicted	---
10	76230380	76230380	-	76230380	9	9	XLOC_011105	-	predicted	---
10	76246368	76246369	-	76246368	8	11	ENSSSCG00000011166	-	intron	*ZYMND11*
10	76246458	76246458	-	76246458	3	3	ENSSSCG00000011166	-	ORF	*ZYMND11*
10	76934589	76934595	-	76934594	14	35	XLOC_011111	-	predicted	---
10	76999149	76999149	+	76999149	3	3	ENSSSCG00000011171	+	ORF	*CTSL*
10	76999205	76999208	+	76999208	9	13	ENSSSCG00000011171	+	ORF	*CTSL*
10	77003487	77003507	+	77003505	86	395	ENSSSCG00000011171	+	3'UTR	*CTSL*
10	77145925	77145931	-	77145931	11	21	XLOC_011112	-	predicted	---
10	77220826	77220826	-	77220826	3	3	---	---	intergenic	---
10	77453611	77453611	-	77453611	4	4	---	---	intergenic	---
10	77689149	77689149	-	77689149	10	10	XLOC_011116	-	predicted	---
10	77690693	77690727	-	77690727	82	167	ENSSSCG00000023247	-	3'UTR	*OPTN*
10	77710876	77710876	-	77710876	12	12	ENSSSCG00000023247	-	ORF	*OPTN*
10	77720008	77720008	-	77720008	3	3	ENSSSCG00000023247	-	ORF	*OPTN*
11	5727	5730	-	5729	16	50	ENSSSCG00000009268	-	3'UTR	*GULDH*
11	116447	116450	-	116447	3	6	ENSSSCG00000009270	-	intron	---
11	117758	117758	-	117758	4	4	ENSSSCG00000009270	-	intron	---

11	191972	191972	+	191972	5	5	ENSSSCG00000025308	+	3'UTR	*IL17D*
							ENSSSCG00000025308	+	ORF	*IL17D*
11	230255	230262	-	230261	12	21	ENSSSCG00000009276	-	3'UTR	*XPO4*
11	304687	304694	-	304693	17	26	XLOC_013980	-	predicted	---
11	429001	429001	+	429001	4	4	ENSSSCG00000028865	+	intron	*SAP18*
11	433165	433179	+	433170	617	1763	ENSSSCG00000028865	+	3'UTR	*SAP18*
11	434543	434543	+	434543	3	3	ENSSSCG00000028865	+	3'UTR	*SAP18*
11	439297	439297	-	439297	3	3	XLOC_013982	-	predicted	---
11	452780	452788	+	452780	12	21	---	---	intergenic	---
11	458971	458978	-	458971	6	13	ENSSSCG00000026744	-	3'UTR	*ZDHHC20*
11	459306	459306	-	459306	4	4	ENSSSCG00000026744	-	3'UTR	*ZDHHC20*
11	532621	532627	-	532622	48	83	XLOC_013984	-	predicted	---
11	532969	532969	-	532969	12	12	XLOC_013984	-	predicted	---
11	533023	533047	-	533023	8	20	XLOC_013984	-	predicted	---
11	533107	533107	-	533107	3	3	XLOC_013984	-	predicted	---
11	596102	596102	+	596102	3	3	---	---	intergenic	---
11	1858978	1858984	-	1858984	4	7	XLOC_013998	-	predicted	---
11	2039125	2039132	-	2039126	46	110	ENSSSCG00000009290	-	3'UTR	---
11	2160031	2160033	-	2160032	10	19	XLOC_014003	-	predicted	---
11	2486730	2486731	+	2486731	9	13	---	---	intergenic	---
11	2489590	2489590	+	2489590	9	9	XLOC_013529	+	predicted	---
11	2489868	2489868	+	2489868	3	3	XLOC_013529	+	predicted	---
11	2490640	2490640	+	2490640	3	3	XLOC_013529	+	predicted	---
11	2491002	2491028	+	2491027	10	40	XLOC_013529	+	predicted	---
11	2508949	2508949	+	2508949	4	4	---	---	intergenic	---
11	2524843	2524843	-	2524843	3	3	---	---	intergenic	---

11	2587661	2587661	+	2587661	3	3	---	---	intergenic	---
11	2616446	2616446	-	2616446	5	5	XLOC_014010	-	predicted	---
11	2911855	2911856	+	2911855	52	62	ENSSSCG00000025583	+	intron	---
11	3021889	3021892	+	3021891	10	17	XLOC_013532	+	predicted	---
11	3042203	3042203	-	3042203	4	4	ENSSSCG00000030113	-	intron	*SHISA2*
11	3046764	3046764	+	3046764	7	7	XLOC_013533	+	predicted	---
11	3177047	3177047	+	3177047	3	3	---	---	intergenic	---
11	3239762	3239766	+	3239763	8	18	---	---	intergenic	---
11	3239805	3239805	+	3239805	3	3	---	---	intergenic	---
11	3239901	3239901	+	3239901	5	5	---	---	intergenic	---
11	3250312	3250312	-	3250312	3	3	---	---	intergenic	---
11	3252258	3252258	-	3252258	3	3	---	---	intergenic	---
11	3458581	3458582	+	3458582	7	10	---	---	intergenic	---
11	3459795	3459806	-	3459805	16	55	XLOC_014018	-	predicted	---
11	3460777	3460777	-	3460777	11	11	XLOC_014018	-	predicted	---
11	3465776	3465776	-	3465776	3	3	ENSSSCG00000024064	-	intron	*RNF6*
11	3467269	3467269	-	3467269	3	3	ENSSSCG00000024064	-	ORF	*RNF6*
11	3467391	3467391	-	3467391	3	3	ENSSSCG00000024064	-	ORF	*RNF6*
11	3601960	3601961	+	3601961	7	10	XLOC_013536	+	predicted	---
11	3734822	3734822	+	3734822	4	4	---	---	intergenic	---
11	3795321	3795321	+	3795321	11	11	XLOC_013541	+	predicted	---
11	4029964	4029965	-	4029964	4	8	ENSSSCG00000009302	-	3'UTR	*USP12*
11	4031997	4032006	-	4032006	17	59	ENSSSCG00000009302	-	3'UTR	*USP12*
11	4032827	4032828	-	4032828	4	7	ENSSSCG00000009302	-	3'UTR	*USP12*
11	4193160	4193160	+	4193160	8	8	---	---	intergenic	---
11	4201642	4201681	+	4201650	5	11	ENSSSCG00000009303	+	ORF	*RPL21*

11	4201946	4201968	+	4201953	325	445	ENSSSCG00000009303	+	3'UTR	*RPL21*
11	4202027	4202028	+	4202027	20	26	ENSSSCG00000009303	+	3'UTR	*RPL21*
11	4211541	4211549	+	4211541	12	44	XLOC_013547	+	predicted	---
11	4496674	4496679	-	4496679	76	110	XLOC_014032	-	predicted	---
11	4564771	4564771	+	4564771	3	3	---	---	intergenic	---
11	4593036	4593036	-	4593036	5	5	---	---	intergenic	---
11	4679279	4679279	+	4679279	4	4	ENSSSCG00000009310	+	intron	*POLR1D*
11	4680704	4680714	+	4680706	104	223	XLOC_013553	+	predicted	---
11	4719584	4719584	+	4719584	3	3	ENSSSCG00000009309	+	intron	---
11	4724631	4724644	+	4724644	13	24	XLOC_013553	+	predicted	---
11	4725200	4725202	+	4725200	6	9	XLOC_013553	+	predicted	---
11	5165012	5165012	-	5165012	7	7	XLOC_014038	-	predicted	---
11	5176673	5176673	+	5176673	9	9	ENSSSCG00000009319	+	intron	*PAN3*
11	5216752	5216752	+	5216752	3	3	ENSSSCG00000009319	+	intron	*PAN3*
11	5301062	5301062	+	5301062	6	6	ENSSSCG00000009319	+	3'UTR	*PAN3*
11	5304874	5304874	-	5304874	3	3	---	---	intergenic	---
11	5307314	5307314	-	5307314	9	9	XLOC_014040	-	predicted	---
11	5309594	5309598	-	5309595	12	25	XLOC_014040	-	predicted	---
11	5692168	5692168	+	5692168	4	4	ENSSSCG00000009322	+	3'UTR	*SLC46A3*
11	5692585	5692585	+	5692585	6	6	XLOC_013570	+	predicted	---
11	5693462	5693462	+	5693462	6	6	XLOC_013570	+	predicted	---
11	5707363	5707363	+	5707363	4	4	XLOC_013572	+	predicted	---
11	5707414	5707434	+	5707419	228	580	XLOC_013572	+	predicted	---
11	5707559	5707559	+	5707559	8	8	XLOC_013572	+	predicted	---
11	5707710	5707710	+	5707710	3	3	XLOC_013572	+	predicted	---
11	5709844	5709846	+	5709844	5	9	XLOC_013572	+	predicted	---

11	5758122	5758122	-	5758122	4	4	ENSSSCG00000029470	-	ORF	*POMP*
11	6118242	6118242	+	6118242	4	4	---	---	intergenic	---
11	6151007	6151007	+	6151007	3	3	ENSSSCG00000009323	+	intron	*MTUS2*
11	6179730	6179731	-	6179730	24	31	XLOC_014048	-	predicted	---
11	6521275	6521275	+	6521275	5	5	XLOC_013579	+	predicted	---
11	6656036	6656036	+	6656036	3	3	---	---	intergenic	---
11	6753438	6753446	-	6753439	28	37	XLOC_014059	-	predicted	---
11	6756671	6756671	-	6756671	3	3	XLOC_014059	-	predicted	---
11	6761121	6761127	-	6761121	5	11	ENSSSCG00000009326	-	3'UTR	*KATNAL1*
11	6772275	6772275	-	6772275	4	4	ENSSSCG00000009326	-	intron	*KATNAL1*
							ENSSSCG00000009326	-	ORF	*KATNAL1*
11	6826045	6826045	-	6826045	9	9	XLOC_014059	-	predicted	---
11	6826662	6826662	-	6826662	3	3	XLOC_014059	-	predicted	---
11	6864876	6864876	-	6864876	3	3	---	---	intergenic	---
11	6914825	6914825	+	6914825	3	3	XLOC_013586	+	predicted	---
11	7125296	7125296	+	7125296	4	4	XLOC_013588	+	predicted	---
11	7125865	7125871	+	7125869	11	22	---	---	intergenic	---
11	7129143	7129157	+	7129157	26	51	ENSSSCG00000009327	+	ORF	*HMGB1*
11	7130330	7130340	+	7130340	12	28	ENSSSCG00000009327	+	ORF	*HMGB1*
11	7130501	7130508	+	7130507	25	33	ENSSSCG00000009327	+	3'UTR	*HMGB1*
11	7130661	7130695	+	7130661	5	8	ENSSSCG00000009327	+	3'UTR	*HMGB1*
11	7130872	7130879	+	7130875	66	129	ENSSSCG00000009327	+	3'UTR	*HMGB1*
11	7131054	7131060	+	7131059	4	10	ENSSSCG00000009327	+	3'UTR	*HMGB1*
11	7131344	7131344	+	7131344	3	3	ENSSSCG00000009327	+	3'UTR	*HMGB1*
11	7131935	7131951	+	7131944	58	83	ENSSSCG00000009327	+	3'UTR	*HMGB1*
11	7213808	7213808	+	7213808	6	6	XLOC_013590	+	predicted	---

11	7287554	7287571	+	7287562	95	432	ENSSSCG00000009330	+	3'UTR	*ALOX5AP*
11	7287658	7287658	+	7287658	4	4	XLOC_013591	+	predicted	---
11	7414361	7414373	+	7414373	7	11	ENSSSCG00000009331	+	3'UTR	*MEDAG*
11	7424481	7424481	+	7424481	3	3	ENSSSCG00000009332	+	intron	*TEX26*
11	7444752	7444762	+	7444753	40	89	ENSSSCG00000009332	+	intron	*TEX26*
11	7444909	7444909	+	7444909	5	5	ENSSSCG00000009332	+	intron	*TEX26*
11	7445176	7445194	+	7445194	19	25	ENSSSCG00000009332	+	intron	*TEX26*
11	7585520	7585530	-	7585528	31	100	ENSSSCG00000009334	-	3'UTR	*HSPH1*
11	7644476	7644476	-	7644476	3	3	ENSSSCG00000009334	-	intron	*HSPH1*
11	7658210	7658210	-	7658210	3	3	ENSSSCG00000009334	-	ORF	*HSPH1*
11	7873635	7873635	+	7873635	3	3	ENSSSCG00000009335	+	3'UTR	*B3GLCT*
11	7887904	7887904	+	7887904	3	3	---	---	intergenic	---
11	7980560	7980560	-	7980560	3	3	---	---	intergenic	---
11	7980801	7980801	+	7980801	3	3	XLOC_013601	+	predicted	---
11	8135754	8135754	-	8135754	4	4	---	---	intergenic	---
11	8169589	8169589	-	8169589	3	3	---	---	intergenic	---
11	8737431	8737431	+	8737431	4	4	ENSSSCG00000009346	+	3'UTR	---
11	8737495	8737495	+	8737495	7	7	ENSSSCG00000009346	+	3'UTR	---
11	8740855	8740855	+	8740855	3	3	XLOC_013610	+	predicted	---
11	8840423	8840423	-	8840423	6	6	---	---	intergenic	---
11	9043051	9043051	+	9043051	3	3	ENSSSCG00000009345	+	3'UTR	---
11	9043994	9043994	+	9043994	3	3	ENSSSCG00000009345	+	3'UTR	---
11	9533309	9533309	+	9533309	4	4	---	---	intergenic	---
11	9634628	9634628	-	9634628	3	3	---	---	intergenic	---
11	9657214	9657214	-	9657214	4	4	---	---	intergenic	---
11	9665398	9665399	-	9665398	9	12	XLOC_014083	-	predicted	---

11	9874437	9874437	+	9874437	6	6	XLOC_013618	+	predicted	---
11	9930221	9930221	-	9930221	3	3	---	---	intergenic	---
11	10069612	10069613	+	10069613	15	23	ENSSSCG00000009349	+	3'UTR	*RFC3*
11	10317557	10317557	+	10317557	3	3	---	---	intergenic	---
11	10427504	10427505	-	10427505	5	9	---	---	intergenic	---
11	11338488	11338488	+	11338488	5	5	---	---	intergenic	---
11	11435244	11435244	+	11435244	5	5	---	---	intergenic	---
11	12153274	12153277	-	12153274	11	23	---	---	intergenic	---
11	12280320	12280326	+	12280326	8	11	ENSSSCG00000024635	+	3'UTR	---
11	12281255	12281269	+	12281255	8	16	ENSSSCG00000024635	+	3'UTR	---
11	12281374	12281384	+	12281374	8	16	ENSSSCG00000024635	+	3'UTR	---
11	12364735	12364743	+	12364737	9	49	ENSSSCG00000009354	+	3'UTR	---
11	12501672	12501672	-	12501672	4	4	---	---	intergenic	---
11	12641855	12641855	+	12641855	3	3	XLOC_013630	+	predicted	---
11	12642540	12642544	+	12642544	11	19	XLOC_013630	+	predicted	---
11	12690919	12690920	-	12690919	21	26	ENSSSCG00000009357	-	intron	*SMAD9*
11	12724530	12724541	-	12724540	24	41	ENSSSCG00000025521	-	3'UTR	---
11	12738219	12738220	-	12738220	6	10	---	---	intergenic	---
11	12761424	12761424	-	12761424	4	4	---	---	intergenic	---
11	12767895	12767895	+	12767895	3	3	---	---	intergenic	---
11	12775504	12775505	-	12775505	14	17	ENSSSCG00000026613	-	3'UTR	---
11	12793669	12793669	+	12793669	3	3	ENSSSCG00000009360	+	ORF	*ALG5*
11	12821405	12821405	-	12821405	3	3	ENSSSCG00000009359	-	intron	*SUPT20H*
11	12886458	12886458	+	12886458	6	6	---	---	intergenic	---
11	13024522	13024522	+	13024522	3	3	---	---	intergenic	---
11	13200041	13200061	-	13200046	49	117	ENSSSCG00000009361	-	3'UTR	*POSTN*

11	13200177	13200178	-	13200177	5	8	ENSSSCG00000009361	-	3'UTR	*POSTN*
11	13200268	13200268	-	13200268	5	5	ENSSSCG00000009361	-	3'UTR	*POSTN*
11	13200588	13200588	-	13200588	3	3	ENSSSCG00000009361	-	3'UTR	*POSTN*
11	13200666	13200666	-	13200666	4	4	ENSSSCG00000009361	-	3'UTR	*POSTN*
11	13207739	13207739	-	13207739	3	3	ENSSSCG00000009361	-	intron	*POSTN*
11	13208781	13208788	-	13208782	19	40	ENSSSCG00000009361	-	intron	*POSTN*
11	13216213	13216213	-	13216213	3	3	---	---	intergenic	---
11	13667367	13667367	+	13667367	3	3	---	---	intergenic	---
11	13679173	13679173	-	13679173	4	4	---	---	intergenic	---
11	13830242	13830246	+	13830242	4	10	XLOC_013635	+	predicted	---
11	13830586	13830588	+	13830587	14	26	XLOC_013635	+	predicted	---
11	13831850	13831852	+	13831852	6	9	XLOC_013635	+	predicted	---
11	13835053	13835053	+	13835053	3	3	---	---	intergenic	---
11	14424645	14424645	-	14424645	6	6	---	---	intergenic	---
11	14464123	14464126	-	14464126	16	25	XLOC_014118	-	predicted	---
11	14664823	14664823	-	14664823	3	3	---	---	intergenic	---
11	14906101	14906101	+	14906101	3	3	---	---	intergenic	---
11	14940346	14940346	-	14940346	47	47	---	---	intergenic	---
11	14982112	14982112	+	14982112	11	11	---	---	intergenic	---
11	15549602	15549602	-	15549602	3	3	ENSSSCG00000009370	-	ORF	*FOXO1*
11	15704047	15704051	-	15704047	4	11	---	---	intergenic	---
11	15738167	15738171	-	15738169	24	39	ENSSSCG00000009372	-	3'UTR	*MRPS31*
11	15841890	15841890	+	15841890	3	3	ENSSSCG00000009373	+	3'UTR	*SLC25A15*
11	15841945	15841947	+	15841946	9	17	ENSSSCG00000009373	+	3'UTR	*SLC25A15*
11	15841987	15841987	+	15841987	4	4	ENSSSCG00000009373	+	3'UTR	*SLC25A15*
11	15842785	15842785	+	15842785	3	3	ENSSSCG00000009373	+	3'UTR	*SLC25A15*

11	15843545	15843595	+	15843585	15	72	XLOC_013649	+	predicted	---
11	15843889	15843889	+	15843889	3	3	XLOC_013649	+	predicted	---
11	15958142	15958142	+	15958142	7	7	XLOC_013650	+	predicted	---
11	16002727	16002727	+	16002727	3	3	ENSSSCG00000009376	+	3'UTR	*VPS36*
11	16003058	16003059	+	16003059	7	11	ENSSSCG00000009376	+	3'UTR	*VPS36*
11	16035635	16035635	+	16035635	5	5	XLOC_013652	+	predicted	---
11	16217555	16217566	-	16217556	8	33	---	---	intergenic	---
11	16217582	16217582	+	16217582	3	3	---	---	intergenic	---
11	16306821	16306821	-	16306821	6	6	---	---	intergenic	---
11	16343176	16343200	+	16343178	19	25	ENSSSCG00000009383	+	ORF	*CCDC70*
11	16343304	16343304	+	16343304	7	7	ENSSSCG00000009383	+	ORF	*CCDC70*
11	16343347	16343347	+	16343347	4	4	ENSSSCG00000009383	+	ORF	*CCDC70*
11	16343704	16343717	+	16343707	34	126	XLOC_013658	+	predicted	---
11	16391941	16391941	-	16391941	7	7	XLOC_014141	-	predicted	---
11	16400192	16400203	-	16400192	6	9	XLOC_014142	-	predicted	---
11	16400249	16400255	-	16400250	28	45	XLOC_014142	-	predicted	---
11	16402468	16402475	-	16402471	27	66	XLOC_014142	-	predicted	---
11	16403104	16403104	-	16403104	5	5	XLOC_014142	-	predicted	---
11	16469899	16469899	-	16469899	3	3	---	---	intergenic	---
11	16523271	16523271	+	16523271	4	4	---	---	intergenic	---
11	16651027	16651027	+	16651027	19	19	---	---	intergenic	---
11	16800719	16800725	+	16800719	3	6	ENSSSCG00000009384	+	ORF	*INTS6*
11	16804188	16804188	+	16804188	4	4	ENSSSCG00000009384	+	3'UTR	*INTS6*
11	16804469	16804471	+	16804469	27	34	ENSSSCG00000009384	+	3'UTR	*INTS6*
11	16807076	16807076	+	16807076	4	4	ENSSSCG00000009384	+	3'UTR	*INTS6*
11	17099850	17099850	+	17099850	4	4	---	---	intergenic	---

11	17212757	17212761	-	17212757	3	9	XLOC_014147	-	predicted	---
11	17229112	17229112	+	17229112	4	4	---	---	intergenic	---
11	17292916	17292916	+	17292916	4	4	---	---	intergenic	---
11	17603673	17603673	+	17603673	3	3	---	---	intergenic	---
11	17734914	17734914	-	17734914	3	3	---	---	intergenic	---
11	17905075	17905075	-	17905075	3	3	XLOC_014148	-	predicted	---
11	18002215	18002215	-	18002215	7	7	---	---	intergenic	---
11	18021479	18021479	-	18021479	4	4	XLOC_014151	-	predicted	---
11	18085207	18085208	-	18085207	6	10	ENSSSCG00000009391	-	intron	*KPNA3*
11	18208102	18208102	+	18208102	3	3	XLOC_013668	+	predicted	---
11	18208250	18208254	+	18208253	12	24	XLOC_013668	+	predicted	---
11	18291331	18291331	+	18291331	3	3	ENSSSCG00000024125	+	3'UTR	---
11	18307738	18307738	+	18307738	4	4	ENSSSCG00000009393	+	intron	*EBPL*
11	18330214	18330221	+	18330214	9	21	XLOC_013670	+	predicted	---
11	18331235	18331236	+	18331235	10	13	XLOC_013670	+	predicted	---
11	18512520	18512529	+	18512529	19	35	ENSSSCG00000021060	+	3'UTR	*RCBTB1*
11	18519924	18519924	-	18519924	21	21	ENSSSCG00000009396	-	3'UTR	*PHF11*
11	18603511	18603511	-	18603511	6	6	XLOC_014157	-	predicted	---
11	18715189	18715197	+	18715190	8	14	ENSSSCG00000009397	+	3'UTR	*CAB39L*
11	18740911	18740915	-	18740915	4	7	XLOC_014158	-	predicted	---
11	18741944	18741944	+	18741944	5	5	---	---	intergenic	---
11	18763194	18763200	+	18763195	9	24	XLOC_013672	+	predicted	---
11	18763295	18763295	+	18763295	4	4	XLOC_013672	+	predicted	---
11	18866093	18866093	-	18866093	7	7	ENSSSCG00000022623	-	3'UTR	*CDADC1*
11	18870003	18870009	-	18870003	7	11	ENSSSCG00000022623	-	3'UTR	*CDADC1*
11	18870270	18870270	-	18870270	3	3	ENSSSCG00000022623	-	3'UTR	*CDADC1*

11	18885164	18885192	-	18885191	6	13	ENSSSCG00000022623	-	ORF	*CDADC1*
11	18894445	18894452	-	18894445	6	10	ENSSSCG00000022623	-	ORF	*CDADC1*
11	18961858	18961868	-	18961860	18	90	---	---	intergenic	---
11	18962037	18962037	-	18962037	6	6	---	---	intergenic	---
11	18962342	18962342	-	18962342	6	6	---	---	intergenic	---
11	18964060	18964060	-	18964060	4	4	---	---	intergenic	---
11	18971285	18971319	+	18971319	6	12	ENSSSCG00000022159	+	ORF	---
11	18979929	18979929	+	18979929	4	4	ENSSSCG00000022159	+	ORF	---
11	19074343	19074343	-	19074343	3	3	ENSSSCG00000029933	-	intron	---
11	19080498	19080498	-	19080498	21	21	ENSSSCG00000029933	-	intron	---
11	19378735	19378735	+	19378735	3	3	XLOC_013677	+	predicted	---
11	19486698	19486698	+	19486698	4	4	ENSSSCG00000009399	+	ORF	*CYSLTR2*
11	19535458	19535458	-	19535458	9	9	---	---	intergenic	---
11	19644070	19644070	+	19644070	3	3	ENSSSCG00000009400	+	3'UTR	*RCBTB2*
11	19647249	19647249	-	19647249	3	3	---	---	intergenic	---
11	19648362	19648362	-	19648362	5	5	ENSSSCG00000009401	-	3'UTR	*RB1*
11	19682289	19682290	+	19682289	4	7	XLOC_013685	+	predicted	---
11	19802319	19802319	-	19802319	4	4	---	---	intergenic	---
11	19802596	19802669	-	19802655	150	349	XLOC_014170	-	predicted	---
11	19803128	19803142	-	19803138	103	151	ENSSSCG00000009403	-	3'UTR	*ITM2B*
11	19803249	19803250	-	19803250	9	13	ENSSSCG00000009403	-	3'UTR	*ITM2B*
11	19805309	19805309	-	19805309	3	3	ENSSSCG00000009403	-	ORF	*ITM2B*
11	19807373	19807373	-	19807373	3	3	ENSSSCG00000009403	-	ORF	*ITM2B*
11	19903213	19903217	-	19903217	4	7	---	---	intergenic	---
11	19998275	19998275	-	19998275	3	3	---	---	intergenic	---
11	20019427	20019447	+	20019434	56	90	ENSSSCG00000009404	+	3'UTR	*MED4*

11	20025639	20025639	-	20025639	3	3	ENSSSCG00000027551	-	3'UTR	*NUDT15*
11	20025822	20025823	-	20025822	7	13	ENSSSCG00000027551	-	3'UTR	*NUDT15*
11	20123454	20123462	+	20123460	43	64	ENSSSCG00000009405	+	3'UTR	*SUCLA2*
11	20260402	20260402	+	20260402	4	4	---	---	intergenic	---
11	20301543	20301543	-	20301543	23	23	---	---	intergenic	---
11	20326407	20326417	+	20326408	12	27	XLOC_013689	+	predicted	---
11	20623433	20623433	+	20623433	7	7	---	---	intergenic	---
11	20671289	20671289	+	20671289	3	3	---	---	intergenic	---
11	20827872	20827872	+	20827872	7	7	---	---	intergenic	---
11	20969107	20969108	-	20969107	15	19	---	---	intergenic	---
11	21007546	21007546	+	21007546	6	6	ENSSSCG00000009407	+	ORF	*ESD*
11	21011662	21011679	+	21011669	271	506	ENSSSCG00000009407	+	3'UTR	*ESD*
11	21488417	21488425	-	21488417	10	31	XLOC_014177	-	predicted	---
11	21525732	21525732	-	21525732	3	3	---	---	intergenic	---
11	21533799	21533799	-	21533799	3	3	---	---	intergenic	---
11	21618712	21618712	+	21618712	3	3	ENSSSCG00000009412	+	intron	*LCP1*
11	21685237	21685306	+	21685237	98	133	ENSSSCG00000009412	+	3'UTR	*LCP1*
11	21725065	21725065	+	21725065	3	3	ENSSSCG00000009413	+	ORF	*CPB2*
11	21725126	21725126	+	21725126	8	8	ENSSSCG00000009413	+	intron	*CPB2*
11	21735321	21735324	+	21735324	205	231	ENSSSCG00000009413	+	ORF	*CPB2*
11	21747737	21747740	+	21747740	4	7	ENSSSCG00000009413	+	intron	*CPB2*
11	21757554	21757578	+	21757560	6	15	ENSSSCG00000009413	+	3'UTR	*CPB2*
11	21757673	21757690	+	21757685	327	713	ENSSSCG00000009413	+	3'UTR	*CPB2*
11	21757980	21757981	+	21757980	9	12	XLOC_013699	+	predicted	---
11	22057080	22057080	-	22057080	8	8	XLOC_014184	-	predicted	---
11	22077324	22077324	+	22077324	4	4	---	---	intergenic	---

11	22133842	22133842	-	22133842	3	3	---	---	intergenic	---
11	22159876	22159877	+	22159876	8	16	---	---	intergenic	---
11	22160935	22160941	+	22160940	16	26	---	---	intergenic	---
11	22214124	22214124	-	22214124	4	4	---	---	intergenic	---
11	22320060	22320060	-	22320060	5	5	---	---	intergenic	---
11	22375597	22375597	+	22375597	10	10	---	---	intergenic	---
11	22377115	22377121	+	22377121	15	24	---	---	intergenic	---
11	22535221	22535221	-	22535221	5	5	ENSSSCG00000009419	-	3'UTR	*GPALPP1*
11	22538577	22538577	-	22538577	5	5	ENSSSCG00000009419	-	3'UTR	*GPALPP1*
11	22562462	22562462	-	22562462	5	5	ENSSSCG00000009419	-	ORF	*GPALPP1*
11	22605342	22605342	+	22605342	4	4	ENSSSCG00000009420	+	3'UTR	*NUFIP1*
11	22651970	22651971	-	22651970	6	9	---	---	intergenic	---
11	22875338	22875338	+	22875338	4	4	ENSSSCG00000009422	+	intron	*TSC22D1*
11	22977325	22977325	+	22977325	4	4	ENSSSCG00000009422	+	intron	*TSC22D1*
11	23017149	23017149	-	23017149	5	5	XLOC_014199	-	predicted	---
11	23247265	23247265	-	23247265	3	3	---	---	intergenic	---
11	23325308	23325308	+	23325308	3	3	---	---	intergenic	---
11	23328813	23328841	+	23328831	24	69	XLOC_013712	+	predicted	---
11	23597039	23597039	-	23597039	3	3	ENSSSCG00000009425	-	3'UTR	---
11	23653404	23653404	-	23653404	3	3	---	---	intergenic	---
11	24477058	24477058	+	24477058	3	3	XLOC_013716	+	predicted	---
11	24591325	24591326	-	24591325	3	6	XLOC_014205	-	predicted	---
11	24591411	24591436	-	24591436	85	124	XLOC_014205	-	predicted	---
11	24756431	24756431	-	24756431	4	4	---	---	intergenic	---
11	24789704	24789704	+	24789704	3	3	XLOC_013721	+	predicted	---
11	24791783	24791784	+	24791784	11	14	---	---	intergenic	---

11	24865228	24865229	+	24865229	13	16	XLOC_013721	+	predicted	---
11	25177020	25177020	+	25177020	4	4	---	---	intergenic	---
11	25404623	25404623	-	25404623	6	6	ENSSSCG00000009430	-	3'UTR	*AKAP11*
11	25405713	25405713	-	25405713	3	3	ENSSSCG00000009430	-	3'UTR	*AKAP11*
11	25407480	25407494	-	25407480	6	11	ENSSSCG00000009430	-	3'UTR	*AKAP11*
11	25408177	25408177	-	25408177	3	3	ENSSSCG00000009430	-	3'UTR	*AKAP11*
11	25415862	25415862	-	25415862	3	3	ENSSSCG00000009430	-	intron	*AKAP11*
11	25421346	25421346	-	25421346	3	3	ENSSSCG00000009430	-	intron	*AKAP11*
11	25474649	25474649	-	25474649	3	3	---	---	intergenic	---
11	25487874	25487874	-	25487874	4	4	XLOC_014210	-	predicted	---
11	25488017	25488022	-	25488018	5	11	XLOC_014210	-	predicted	---
11	25488101	25488109	-	25488102	9	16	ENSSSCG00000009431	-	ORF	*DGKH*
11	25524114	25524114	-	25524114	3	3	ENSSSCG00000009431	-	ORF	*DGKH*
11	25986986	25986986	-	25986986	4	4	---	---	intergenic	---
11	25987023	25987023	+	25987023	4	4	---	---	intergenic	---
11	25998931	25998931	+	25998931	3	3	---	---	intergenic	---
11	26114828	26114828	-	26114828	3	3	ENSSSCG00000024707	-	intron	---
11	26202650	26202668	-	26202662	22	46	XLOC_014215	-	predicted	---
11	26246513	26246514	-	26246513	8	15	XLOC_014216	-	predicted	---
11	26380161	26380161	+	26380161	8	8	ENSSSCG00000009440	+	intron	*ELF1*
11	26385611	26385611	+	26385611	5	5	XLOC_013732	+	predicted	---
11	26402231	26402247	+	26402247	4	7	ENSSSCG00000009441	+	intron	*SUGT1*
11	26412741	26412742	+	26412742	20	25	ENSSSCG00000009441	+	intron	*SUGT1*
11	26425803	26425809	+	26425809	14	18	ENSSSCG00000009441	+	intron	*SUGT1*
11	26437282	26437306	+	26437283	21	78	ENSSSCG00000009441	+	3'UTR	*SUGT1*
11	26437370	26437382	+	26437380	8	17	ENSSSCG00000009441	+	intron	*SUGT1*

11	26437714	26437714	+	26437714	3	3	ENSSSCG00000009441	+	intron	*SUGT1*
11	26442078	26442078	+	26442078	3	3	ENSSSCG00000009441	+	intron	*SUGT1*
11	26521139	26521139	-	26521139	9	9	---	---	intergenic	---
11	26569437	26569437	-	26569437	3	3	XLOC_014218	-	predicted	---
11	26782695	26782695	+	26782695	3	3	---	---	intergenic	---
11	27046824	27046825	+	27046825	74	84	ENSSSCG00000009444	+	intron	*OLFM4*
11	27181768	27181768	-	27181768	7	7	---	---	intergenic	---
11	27306195	27306195	-	27306195	48	48	---	---	intergenic	---
11	27801327	27801327	-	27801327	3	3	---	---	intergenic	---
11	28162446	28162446	-	28162446	3	3	---	---	intergenic	---
11	28426747	28426748	+	28426747	3	6	---	---	intergenic	---
11	28526333	28526333	-	28526333	3	3	---	---	intergenic	---
11	28744310	28744310	-	28744310	3	3	---	---	intergenic	---
11	28756190	28756190	-	28756190	30	30	---	---	intergenic	---
11	28800772	28800776	+	28800775	8	20	---	---	intergenic	---
11	28820352	28820352	+	28820352	10	10	---	---	intergenic	---
11	29178063	29178063	-	29178063	3	3	---	---	intergenic	---
11	29286815	29286815	+	29286815	3	3	---	---	intergenic	---
11	30201877	30201877	+	30201877	3	3	---	---	intergenic	---
11	30302454	30302456	-	30302455	9	16	---	---	intergenic	---
11	31481642	31481642	-	31481642	4	4	---	---	intergenic	---
11	32155290	32155290	+	32155290	6	6	---	---	intergenic	---
11	32709376	32709376	+	32709376	16	16	---	---	intergenic	---
11	32791280	32791280	-	32791280	6	6	---	---	intergenic	---
11	32793984	32793984	+	32793984	3	3	---	---	intergenic	---
11	32827554	32827555	+	32827554	3	6	---	---	intergenic	---

11	33003427	33003427	-	33003427	3	3	---	---	intergenic	---
11	33870854	33870854	-	33870854	3	3	---	---	intergenic	---
11	34322336	34322338	+	34322337	4	11	ENSSSCG00000021189	+	intron	---
11	34475403	34475417	+	34475403	36	120	---	---	intergenic	---
11	34987643	34987651	-	34987651	11	19	XLOC_014239	-	predicted	---
11	35013305	35013305	+	35013305	7	7	ENSSSCG00000009449	+	intron	---
11	35135528	35135528	+	35135528	4	4	ENSSSCG00000009449	+	intron	---
11	35171248	35171249	+	35171249	9	14	ENSSSCG00000009449	+	3'UTR	---
11	35676214	35676214	-	35676214	3	3	---	---	intergenic	---
11	36397539	36397539	+	36397539	6	6	---	---	intergenic	---
11	37202910	37202910	+	37202910	3	3	---	---	intergenic	---
11	37592729	37592729	-	37592729	5	5	---	---	intergenic	---
11	37688050	37688050	+	37688050	38	38	---	---	intergenic	---
11	37997600	37997600	-	37997600	5	5	---	---	intergenic	---
11	37997988	37997988	+	37997988	3	3	---	---	intergenic	---
11	39201856	39201856	+	39201856	3	3	---	---	intergenic	---
11	39871836	39871836	-	39871836	3	3	---	---	intergenic	---
11	40154516	40154516	-	40154516	3	3	---	---	intergenic	---
11	41504696	41504705	+	41504696	14	21	---	---	intergenic	---
11	41692444	41692444	+	41692444	3	3	---	---	intergenic	---
11	41949673	41949673	+	41949673	3	3	---	---	intergenic	---
11	43106901	43106901	-	43106901	4	4	---	---	intergenic	---
11	43107324	43107324	-	43107324	3	3	---	---	intergenic	---
11	43551236	43551236	-	43551236	10	10	---	---	intergenic	---
11	43781252	43781253	-	43781252	3	6	---	---	intergenic	---
11	44270416	44270416	+	44270416	4	4	---	---	intergenic	---

11	44314996	44314996	+	44314996	7	7	---	---	intergenic	---
11	45086579	45086579	-	45086579	6	6	---	---	intergenic	---
11	45551522	45551522	-	45551522	8	8	---	---	intergenic	---
11	45869778	45869781	+	45869778	8	16	---	---	intergenic	---
11	45988998	45988998	-	45988998	5	5	XLOC_014248	-	predicted	---
11	45989330	45989330	-	45989330	10	10	XLOC_014248	-	predicted	---
11	46105158	46105160	-	46105160	9	16	---	---	intergenic	---
11	46558631	46558638	+	46558638	5	9	---	---	intergenic	---
11	46974601	46974602	+	46974602	5	9	---	---	intergenic	---
11	47139450	47139450	+	47139450	6	6	---	---	intergenic	---
11	47357837	47357859	+	47357858	215	304	---	---	intergenic	---
11	47414896	47414896	-	47414896	3	3	---	---	intergenic	---
11	48304760	48304761	-	48304760	5	8	ENSSSCG00000009457	-	intron	---
11	48614816	48614816	+	48614816	5	5	---	---	intergenic	---
11	49100963	49100963	+	49100963	4	4	---	---	intergenic	---
11	49189154	49189154	-	49189154	4	4	---	---	intergenic	---
11	49401328	49401328	+	49401328	8	8	---	---	intergenic	---
11	49419463	49419463	+	49419463	5	5	---	---	intergenic	---
11	49543493	49543493	+	49543493	3	3	---	---	intergenic	---
11	49548381	49548387	-	49548381	3	6	XLOC_014256	-	predicted	---
11	49594719	49594721	-	49594720	9	17	ENSSSCG00000009460	-	3'UTR	---
11	49594790	49594790	-	49594790	4	4	ENSSSCG00000009460	-	3'UTR	---
11	49741804	49741804	-	49741804	3	3	---	---	intergenic	---
11	50016195	50016195	-	50016195	4	4	XLOC_014260	-	predicted	---
11	50017664	50017664	-	50017664	20	20	XLOC_014260	-	predicted	---
11	50385127	50385127	+	50385127	14	14	---	---	intergenic	---

11	50451919	50451919	+	50451919	3	3	---	---	intergenic	---
11	51106624	51106624	-	51106624	4	4	---	---	intergenic	---
11	51257681	51257681	-	51257681	10	10	---	---	intergenic	---
11	51334783	51334783	+	51334783	5	5	---	---	intergenic	---
11	51833336	51833336	-	51833336	3	3	---	---	intergenic	---
11	51848205	51848205	-	51848205	3	3	---	---	intergenic	---
11	51904332	51904332	-	51904332	4	4	---	---	intergenic	---
11	51964568	51964580	-	51964578	88	433	---	---	intergenic	---
11	51964612	51964624	+	51964616	9	38	---	---	intergenic	---
11	51998063	51998063	-	51998063	4	4	---	---	intergenic	---
11	52004860	52004860	+	52004860	7	7	---	---	intergenic	---
11	52125819	52125819	+	52125819	15	15	---	---	intergenic	---
11	52159652	52159655	-	52159652	3	9	ENSSSCG00000009465	-	3'UTR	---
11	52401920	52401920	+	52401920	3	3	---	---	intergenic	---
11	52430748	52430748	+	52430748	3	3	ENSSSCG00000009466	+	intron	*UCHL3*
11	52477123	52477128	+	52477123	8	19	ENSSSCG00000009466	+	3'UTR	*UCHL3*
11	52477173	52477190	+	52477174	110	222	XLOC_013788	+	predicted	---
11	52477244	52477244	+	52477244	3	3	XLOC_013788	+	predicted	---
11	52555900	52555904	-	52555902	16	44	---	---	intergenic	---
11	52555927	52555927	+	52555927	5	5	---	---	intergenic	---
11	52709989	52709994	+	52709993	17	26	---	---	intergenic	---
11	52721912	52721913	-	52721912	3	6	---	---	intergenic	---
11	52756558	52756558	+	52756558	3	3	---	---	intergenic	---
11	52839699	52839699	-	52839699	5	5	---	---	intergenic	---
11	52955940	52955945	-	52955945	8	23	---	---	intergenic	---
11	53130954	53130957	-	53130957	5	8	---	---	intergenic	---

11	53238032	53238032	+	53238032	5	5	---	---	intergenic	---
11	53453640	53453640	+	53453640	6	6	---	---	intergenic	---
11	53454727	53454727	+	53454727	3	3	---	---	intergenic	---
11	53527234	53527234	-	53527234	3	3	---	---	intergenic	---
11	53614084	53614085	+	53614084	3	6	XLOC_013799	+	predicted	---
11	53664573	53664573	+	53664573	4	4	XLOC_013800	+	predicted	---
11	53666249	53666249	-	53666249	6	6	XLOC_014277	-	predicted	---
11	53666493	53666493	-	53666493	3	3	XLOC_014277	-	predicted	---
11	53725776	53725776	+	53725776	8	8	ENSSSCG00000009469	+	3'UTR	---
11	53766557	53766579	+	53766561	5	21	XLOC_013802	+	predicted	---
11	53769561	53769582	-	53769582	17	24	XLOC_014279	-	predicted	---
11	53770553	53770553	-	53770553	5	5	XLOC_014279	-	predicted	---
11	53770665	53770665	-	53770665	3	3	XLOC_014279	-	predicted	---
11	53801866	53801867	-	53801867	6	11	---	---	intergenic	---
11	53833402	53833407	-	53833402	3	6	ENSSSCG00000009473	-	intron	*MYCBP2*
11	54362281	54362282	+	54362281	48	73	---	---	intergenic	---
11	54365764	54365764	-	54365764	3	3	---	---	intergenic	---
11	54503965	54503966	+	54503966	5	9	ENSSSCG00000009475	+	3'UTR	---
11	54504070	54504070	+	54504070	8	8	ENSSSCG00000009475	+	3'UTR	---
11	54589171	54589171	+	54589171	4	4	---	---	intergenic	---
11	54692305	54692305	-	54692305	3	3	ENSSSCG00000009477	-	ORF	*EDNRB*
11	54743864	54743864	+	54743864	3	3	---	---	intergenic	---
11	55430775	55430776	-	55430775	4	8	XLOC_014287	-	predicted	---
11	56041181	56041185	+	56041181	4	8	---	---	intergenic	---
11	56059598	56059598	+	56059598	3	3	---	---	intergenic	---
11	56096950	56096950	-	56096950	3	3	XLOC_014291	-	predicted	---

11	56309224	56309227	+	56309225	18	36	XLOC_013814	+	predicted	---
11	56309371	56309371	+	56309371	3	3	XLOC_013814	+	predicted	---
11	56309774	56309780	+	56309774	3	6	XLOC_013814	+	predicted	---
11	56310345	56310348	+	56310347	48	69	XLOC_013814	+	predicted	---
11	56822401	56822401	-	56822401	3	3	---	---	intergenic	---
11	56886683	56886683	+	56886683	4	4	---	---	intergenic	---
11	57007261	57007261	+	57007261	6	6	---	---	intergenic	---
11	57054363	57054363	+	57054363	3	3	---	---	intergenic	---
11	57578145	57578145	+	57578145	3	3	---	---	intergenic	---
11	57772039	57772039	+	57772039	3	3	---	---	intergenic	---
11	57780520	57780520	+	57780520	6	6	---	---	intergenic	---
11	58180805	58180805	+	58180805	4	4	---	---	intergenic	---
11	58388857	58388857	-	58388857	4	4	XLOC_014297	-	predicted	---
11	58390117	58390117	-	58390117	4	4	---	---	intergenic	---
11	59103582	59103582	-	59103582	7	7	---	---	intergenic	---
11	59135052	59135052	+	59135052	13	13	---	---	intergenic	---
11	59539640	59539640	+	59539640	7	7	---	---	intergenic	---
11	59948875	59948875	-	59948875	4	4	---	---	intergenic	---
11	60104738	60104740	-	60104738	4	8	---	---	intergenic	---
11	60306608	60306608	+	60306608	115	115	---	---	intergenic	---
11	61026226	61026226	+	61026226	3	3	---	---	intergenic	---
11	61496340	61496341	+	61496340	14	19	---	---	intergenic	---
11	61725605	61725605	-	61725605	3	3	---	---	intergenic	---
11	62010989	62010989	+	62010989	6	6	---	---	intergenic	---
11	62035033	62035033	-	62035033	3	3	---	---	intergenic	---
11	62068730	62068730	+	62068730	6	6	---	---	intergenic	---

11	62150630	62150630	+	62150630	5	5	---	---	intergenic	---
11	62223345	62223346	+	62223345	9	16	---	---	intergenic	---
11	62917502	62917502	-	62917502	8	8	---	---	intergenic	---
11	63392535	63392535	+	63392535	4	4	---	---	intergenic	---
11	63670007	63670007	-	63670007	3	3	XLOC_014305	-	predicted	---
11	63711463	63711463	+	63711463	3	3	---	---	intergenic	---
11	63741662	63741662	+	63741662	6	6	XLOC_013830	+	predicted	---
11	63747991	63747991	+	63747991	3	3	---	---	intergenic	---
11	64712122	64712122	+	64712122	3	3	---	---	intergenic	---
11	64896065	64896065	-	64896065	5	5	---	---	intergenic	---
11	65098172	65098172	-	65098172	7	7	---	---	intergenic	---
11	65110367	65110368	+	65110367	17	22	XLOC_013831	+	predicted	---
11	65362781	65362783	-	65362781	4	7	---	---	intergenic	---
11	65532780	65532780	-	65532780	4	4	---	---	intergenic	---
11	65591623	65591623	+	65591623	4	4	---	---	intergenic	---
11	66016723	66016724	+	66016724	8	12	---	---	intergenic	---
11	66909688	66909688	-	66909688	5	5	---	---	intergenic	---
11	66954432	66954432	+	66954432	3	3	---	---	intergenic	---
11	67174904	67174904	-	67174904	3	3	---	---	intergenic	---
11	67271331	67271331	-	67271331	6	6	---	---	intergenic	---
11	67756866	67756867	+	67756867	4	7	---	---	intergenic	---
11	67852847	67852847	+	67852847	3	3	---	---	intergenic	---
11	68299280	68299280	+	68299280	4	4	---	---	intergenic	---
11	68392723	68392725	-	68392724	5	12	---	---	intergenic	---
11	68492098	68492099	-	68492098	3	6	---	---	intergenic	---
11	69064019	69064030	-	69064029	14	32	---	---	intergenic	---

11	69070294	69070294	+	69070294	10	10	---	---	intergenic	---
11	69451913	69451913	-	69451913	4	4	---	---	intergenic	---
11	69518639	69518639	-	69518639	17	17	---	---	intergenic	---
11	70022634	70022634	+	70022634	3	3	---	---	intergenic	---
11	70225536	70225536	+	70225536	6	6	XLOC_013851	+	predicted	---
11	70225819	70225823	+	70225823	17	21	XLOC_013851	+	predicted	---
11	70226595	70226595	+	70226595	3	3	---	---	intergenic	---
11	70226778	70226778	+	70226778	5	5	---	---	intergenic	---
11	70405839	70405839	-	70405839	3	3	ENSSSCG00000009495	-	intron	---
11	70573883	70573883	-	70573883	3	3	---	---	intergenic	---
11	70751594	70751594	+	70751594	4	4	XLOC_013853	+	predicted	---
11	71611389	71611391	+	71611390	13	21	XLOC_013858	+	predicted	---
11	71611454	71611457	+	71611457	5	8	---	---	intergenic	---
11	71964113	71964113	+	71964113	5	5	---	---	intergenic	---
11	71981558	71981558	+	71981558	4	4	---	---	intergenic	---
11	72006704	72006704	+	72006704	5	5	ENSSSCG00000009500	+	3'UTR	*CLDN10*
11	72006862	72006862	+	72006862	3	3	XLOC_013860	+	predicted	---
11	72008826	72008832	-	72008827	20	52	XLOC_014329	-	predicted	---
11	72009577	72009584	-	72009578	15	22	XLOC_014329	-	predicted	---
11	72016720	72016720	-	72016720	3	3	ENSSSCG00000009501	-	ORF	*DZIP1*
11	72056689	72056689	-	72056689	4	4	ENSSSCG00000009501	-	intron	*DZIP1*
11	72131065	72131065	+	72131065	3	3	ENSSSCG00000026082	+	intron	*DNAJC3*
11	72149341	72149341	+	72149341	3	3	ENSSSCG00000026082	+	ORF	*DNAJC3*
11	72158614	72158615	+	72158614	6	9	ENSSSCG00000026082	+	ORF	*DNAJC3*
11	72161755	72161772	+	72161767	25	46	ENSSSCG00000026082	+	3'UTR	*DNAJC3*
11	72162425	72162437	+	72162425	7	16	XLOC_013861	+	predicted	---

11	72165288	72165296	+	72165296	18	33	XLOC_013861	+	predicted	---
11	72185135	72185135	-	72185135	3	3	---	---	intergenic	---
11	72431941	72431941	-	72431941	6	6	---	---	intergenic	---
11	72543207	72543207	+	72543207	3	3	---	---	intergenic	---
11	72681582	72681594	+	72681587	21	32	XLOC_013865	+	predicted	---
11	72837800	72837800	-	72837800	4	4	---	---	intergenic	---
11	72913032	72913032	+	72913032	3	3	---	---	intergenic	---
11	73076889	73076889	+	73076889	8	8	---	---	intergenic	---
11	73227769	73227770	+	73227769	4	8	---	---	intergenic	---
11	73714755	73714755	+	73714755	9	9	---	---	intergenic	---
11	73788152	73788156	-	73788152	5	8	XLOC_014342	-	predicted	---
11	74024699	74024699	+	74024699	47	47	---	---	intergenic	---
11	74079047	74079075	+	74079075	12	19	ENSSSCG00000009508	+	ORF	*CISD1*
11	74138237	74138237	+	74138237	4	4	ENSSSCG00000009509	+	ORF	*IPO5*
11	74141344	74141344	+	74141344	3	3	ENSSSCG00000009509	+	ORF	*IPO5*
11	74158633	74158633	+	74158633	8	8	ENSSSCG00000009509	+	ORF	*IPO5*
11	74158730	74158730	+	74158730	3	3	ENSSSCG00000009509	+	ORF	*IPO5*
11	74166405	74166405	+	74166405	3	3	ENSSSCG00000009509	+	ORF	*IPO5*
11	74168228	74168228	+	74168228	4	4	ENSSSCG00000009509	+	ORF	*IPO5*
11	74173270	74173299	+	74173294	49	170	ENSSSCG00000009509	+	3'UTR	*IPO5*
11	74173883	74173888	+	74173883	7	18	ENSSSCG00000009509	+	3'UTR	*IPO5*
11	74174473	74174473	+	74174473	8	8	ENSSSCG00000009509	+	3'UTR	*IPO5*
11	74200347	74200347	-	74200347	4	4	---	---	intergenic	---
11	74304651	74304651	-	74304651	5	5	---	---	intergenic	---
11	74602089	74602089	+	74602089	3	3	ENSSSCG00000026863	+	intron	---
11	74749759	74749759	+	74749759	3	3	---	---	intergenic	---

11	74946191	74946192	-	74946191	93	99	---	---	intergenic	---
11	74973148	74973148	+	74973148	3	3	---	---	intergenic	---
11	74997793	74997797	-	74997797	7	10	ENSSSCG00000022112	-	3'UTR	*DOCK9*
11	75083020	75083021	-	75083021	5	8	ENSSSCG00000022112	-	intron	*DOCK9*
11	75359757	75359757	+	75359757	9	9	---	---	intergenic	---
11	75527994	75527996	-	75527994	4	7	ENSSSCG00000009517	-	3'UTR	*GPR183*
11	75619365	75619390	+	75619384	19	45	XLOC_013880	+	predicted	---
11	75619483	75619486	+	75619485	4	14	XLOC_013880	+	predicted	---
11	75620318	75620322	+	75620321	20	26	XLOC_013880	+	predicted	---
11	75620492	75620492	+	75620492	3	3	XLOC_013880	+	predicted	---
11	75761036	75761036	+	75761036	7	7	---	---	intergenic	---
11	75769609	75769609	+	75769609	4	4	---	---	intergenic	---
11	75776177	75776180	-	75776180	39	45	---	---	intergenic	---
11	76023920	76023920	+	76023920	3	3	ENSSSCG00000009519	+	ORF	*CLYBL*
11	76025085	76025086	+	76025085	16	32	ENSSSCG00000009519	+	3'UTR	*CLYBL*
11	76072418	76072418	+	76072418	3	3	---	---	intergenic	---
11	76094826	76094827	+	76094826	11	16	---	---	intergenic	---
11	76387074	76387074	+	76387074	7	7	---	---	intergenic	---
11	76547553	76547557	+	76547556	69	117	XLOC_013893	+	predicted	---
11	76598169	76598169	-	76598169	3	3	XLOC_014362	-	predicted	---
11	76623518	76623519	-	76623519	6	10	XLOC_014363	-	predicted	---
11	76835965	76835967	-	76835967	4	7	---	---	intergenic	---
11	76836722	76836722	+	76836722	3	3	---	---	intergenic	---
11	76871118	76871118	-	76871118	11	11	---	---	intergenic	---
11	76913833	76913834	+	76913833	13	21	---	---	intergenic	---
11	77129667	77129668	-	77129667	7	13	---	---	intergenic	---

11	77129705	77129708	+	77129706	21	45	---	---	intergenic	---
11	77551285	77551287	-	77551285	4	7	ENSSSCG00000009527	-	3'UTR	*FGF14*
11	77624135	77624135	-	77624135	9	9	ENSSSCG00000009527	-	intron	*FGF14*
11	77800303	77800303	-	77800303	3	3	ENSSSCG00000009527	-	intron	*FGF14*
11	77910917	77910917	+	77910917	3	3	---	---	intergenic	---
11	77951528	77951529	-	77951528	8	14	---	---	intergenic	---
11	78157600	78157600	-	78157600	4	4	---	---	intergenic	---
11	78157723	78157732	-	78157724	9	31	---	---	intergenic	---
11	78255718	78255718	+	78255718	4	4	ENSSSCG00000009528	+	ORF	---
11	78255920	78255921	+	78255920	3	6	ENSSSCG00000009528	+	intron	---
11	78276112	78276117	+	78276116	10	30	ENSSSCG00000009528	+	intron	---
11	78322299	78322301	+	78322299	8	16	XLOC_013906	+	predicted	---
11	78323058	78323058	+	78323058	6	6	XLOC_013906	+	predicted	---
11	78350504	78350504	-	78350504	5	5	---	---	intergenic	---
11	78361286	78361288	-	78361288	4	7	XLOC_014373	-	predicted	---
11	78369212	78369218	-	78369215	71	146	ENSSSCG00000009531	-	3'UTR	---
11	78369283	78369291	-	78369291	6	12	ENSSSCG00000009531	-	3'UTR	---
11	78386613	78386613	-	78386613	8	8	XLOC_014375	-	predicted	---
11	78421427	78421434	-	78421427	6	16	ENSSSCG00000009532	-	intron	---
11	78430013	78430013	-	78430013	3	3	ENSSSCG00000009532	-	intron	---
11	78449032	78449032	-	78449032	3	3	ENSSSCG00000009532	-	intron	---
11	78484669	78484669	-	78484669	5	5	ENSSSCG00000009532	-	intron	---
11	78538743	78538744	-	78538744	11	14	---	---	intergenic	---
11	78538760	78538761	+	78538761	15	21	---	---	intergenic	---
11	78699425	78699425	-	78699425	3	3	---	---	intergenic	---
11	78717331	78717331	+	78717331	5	5	---	---	intergenic	---

11	78763156	78763163	+	78763156	15	23	XLOC_013909	+	predicted	---
11	78919516	78919516	-	78919516	3	3	---	---	intergenic	---
11	79600937	79600937	+	79600937	6	6	---	---	intergenic	---
11	79709992	79709992	-	79709992	18	18	---	---	intergenic	---
11	79765528	79765528	-	79765528	3	3	---	---	intergenic	---
11	80185183	80185183	-	80185183	3	3	---	---	intergenic	---
11	80522095	80522095	+	80522095	3	3	---	---	intergenic	---
11	80998377	80998377	-	80998377	3	3	---	---	intergenic	---
11	81134462	81134463	-	81134462	4	7	---	---	intergenic	---
11	81561730	81561730	-	81561730	6	6	XLOC_014387	-	predicted	---
11	81616166	81616166	-	81616166	4	4	ENSSSCG00000009536	-	3'UTR	*ARGLU1*
11	81617835	81617835	-	81617835	26	26	ENSSSCG00000009536	-	3'UTR	*ARGLU1*
11	81617939	81617939	-	81617939	6	6	ENSSSCG00000009536	-	3'UTR	*ARGLU1*
11	81634958	81634958	-	81634958	3	3	ENSSSCG00000009536	-	intron	*ARGLU1*
11	81635283	81635306	-	81635306	12	16	ENSSSCG00000009536	-	ORF	*ARGLU1*
11	81643518	81643521	-	81643521	13	20	ENSSSCG00000009536	-	intron	*ARGLU1*
11	81728762	81728762	+	81728762	3	3	---	---	intergenic	---
11	81766068	81766068	-	81766068	3	3	---	---	intergenic	---
11	81859522	81859522	-	81859522	6	6	---	---	intergenic	---
11	81860758	81860761	+	81860758	4	7	XLOC_013920	+	predicted	---
11	81865843	81865843	-	81865843	5	5	---	---	intergenic	---
11	81870703	81870703	-	81870703	4	4	XLOC_014394	-	predicted	---
11	81871318	81871320	-	81871318	7	10	XLOC_014394	-	predicted	---
11	82359616	82359616	-	82359616	4	4	---	---	intergenic	---
11	82519093	82519093	+	82519093	9	9	---	---	intergenic	---
11	82596440	82596446	+	82596440	3	6	XLOC_013924	+	predicted	---

11	82672151	82672151	-	82672151	3	3	---	---	intergenic	---
11	82692380	82692386	+	82692380	11	20	---	---	intergenic	---
11	82782144	82782144	+	82782144	3	3	---	---	intergenic	---
11	82853212	82853212	+	82853212	3	3	---	---	intergenic	---
11	82909887	82909890	-	82909888	21	27	---	---	intergenic	---
11	82983577	82983577	+	82983577	3	3	XLOC_013927	+	predicted	---
11	83048062	83048067	+	83048062	5	14	XLOC_013928	+	predicted	---
11	83116492	83116492	-	83116492	4	4	---	---	intergenic	---
11	83725943	83725943	-	83725943	18	18	---	---	intergenic	---
11	83816772	83816772	-	83816772	4	4	XLOC_014409	-	predicted	---
11	83816855	83816855	-	83816855	3	3	XLOC_014409	-	predicted	---
11	83819616	83819616	-	83819616	4	4	XLOC_014409	-	predicted	---
11	83827202	83827204	+	83827203	6	14	XLOC_013931	+	predicted	---
11	83969020	83969043	-	83969041	168	257	XLOC_014410	-	predicted	---
11	84091592	84091597	-	84091592	3	6	---	---	intergenic	---
11	84093439	84093439	-	84093439	13	13	---	---	intergenic	---
11	84155457	84155460	-	84155460	7	10	---	---	intergenic	---
11	84155487	84155488	+	84155488	9	12	---	---	intergenic	---
11	84172978	84172978	-	84172978	32	32	---	---	intergenic	---
11	84372294	84372294	-	84372294	3	3	XLOC_014415	-	predicted	---
11	84372396	84372399	-	84372397	6	15	XLOC_014415	-	predicted	---
11	84372936	84372938	-	84372936	7	18	XLOC_014415	-	predicted	---
11	84373393	84373399	-	84373397	17	31	XLOC_014415	-	predicted	---
11	84627679	84627679	+	84627679	4	4	ENSSSCG00000009545	+	intron	*COL4A2*
11	84675814	84675830	+	84675825	27	78	ENSSSCG00000009545	+	3'UTR	*COL4A2*
11	84681264	84681266	-	84681264	9	13	ENSSSCG00000022719	-	3'UTR	*RAB20*

11	84745326	84745329	+	84745327	125	158	ENSSSCG00000029502	+	3'UTR	*CARKD*
11	84746513	84746514	-	84746514	18	21	ENSSSCG00000023363	-	3'UTR	---
11	84841271	84841283	-	84841272	43	64	---	---	intergenic	---
11	84848103	84848103	-	84848103	4	4	---	---	intergenic	---
11	84863059	84863059	+	84863059	4	4	---	---	intergenic	---
11	84890687	84890687	+	84890687	3	3	---	---	intergenic	---
11	84951068	84951068	+	84951068	3	3	ENSSSCG00000009551	+	intron	---
11	85021723	85021733	+	85021731	65	189	XLOC_013946	+	predicted	---
11	85630235	85630235	-	85630235	4	4	XLOC_014426	-	predicted	---
11	85777611	85777617	-	85777611	3	6	XLOC_014429	-	predicted	---
11	85820849	85820849	+	85820849	6	6	ENSSSCG00000009553	+	3'UTR	*TUBGCP3*
11	85824893	85824918	-	85824917	90	206	XLOC_014430	-	predicted	---
11	85834129	85834139	-	85834129	24	61	XLOC_014430	-	predicted	---
11	85835472	85835479	-	85835478	10	20	XLOC_014430	-	predicted	---
11	86036479	86036479	+	86036479	3	3	XLOC_013951	+	predicted	---
11	86036656	86036664	+	86036664	20	41	XLOC_013951	+	predicted	---
11	86116842	86116843	+	86116842	6	12	ENSSSCG00000009554	+	3'UTR	*GRTP1*
11	86117928	86117938	-	86117933	286	501	ENSSSCG00000009555	-	3'UTR	*LAMP1*
11	86118480	86118481	-	86118481	5	8	ENSSSCG00000009555	-	3'UTR	*LAMP1*
11	86118530	86118544	-	86118530	9	24	ENSSSCG00000009555	-	3'UTR	*LAMP1*
11	86146365	86146365	-	86146365	3	3	ENSSSCG00000009556	-	3'UTR	*CUL4A*
11	86146597	86146600	-	86146599	12	19	ENSSSCG00000009556	-	3'UTR	*CUL4A*
11	86146691	86146710	-	86146710	8	15	ENSSSCG00000009556	-	3'UTR	*CUL4A*
11	86177034	86177034	-	86177034	3	3	ENSSSCG00000009556	-	intron	*CUL4A*
11	86194241	86194260	+	86194254	27	63	XLOC_013956	+	predicted	---
11	86196561	86196561	-	86196561	4	4	XLOC_014436	-	predicted	---

11	86197298	86197303	-	86197301	43	120	ENSSSCG00000009558	-	3'UTR	*F10*
11	86197651	86197651	-	86197651	3	3	ENSSSCG00000009558	-	3'UTR	*F10*
11	86211843	86211844	-	86211843	5	9	ENSSSCG00000009558	-	intron	*F10*
							ENSSSCG00000009558	-	ORF	*F10*
11	86220716	86220716	-	86220716	3	3	ENSSSCG00000029503	-	ORF	*F7*
11	86248296	86248296	+	86248296	4	4	ENSSSCG00000023218	+	intron	---
11	86374294	86374294	+	86374294	3	3	XLOC_013960	+	predicted	---
11	86392441	86392448	+	86392448	4	7	ENSSSCG00000024103	+	3'UTR	*ADPRHL1*
							ENSSSCG00000024103	+	intron	*ADPRHL1*
							ENSSSCG00000024103	+	ORF	*ADPRHL1*
11	86476511	86476526	+	86476519	34	88	ENSSSCG00000009563	+	3'UTR	*TMCO3*
11	86511641	86511641	+	86511641	8	8	XLOC_013961	+	predicted	---
11	86582222	86582232	-	86582231	13	43	ENSSSCG00000009565	-	3'UTR	*GAS6*
11	86644370	86644370	+	86644370	14	14	---	---	intergenic	---
11	86745573	86745573	+	86745573	5	5	ENSSSCG00000009567	+	intron	*RASA3*
11	86763490	86763491	+	86763491	9	13	ENSSSCG00000009567	+	3'UTR	*RASA3*
11	86853729	86853729	+	86853729	3	3	ENSSSCG00000023633	+	3'UTR	---
11	86869988	86869988	+	86869988	3	3	ENSSSCG00000009570	+	ORF	*MPHOSPH8*
11	86870756	86870756	+	86870756	3	3	ENSSSCG00000009570	+	intron	*MPHOSPH8*
11	86890359	86890365	+	86890359	31	40	ENSSSCG00000009570	+	3'UTR	*MPHOSPH8*
11	86891407	86891409	+	86891407	3	6	ENSSSCG00000009570	+	3'UTR	*MPHOSPH8*
11	86976056	86976057	-	86976056	7	10	XLOC_014447	-	predicted	---
11	86985167	86985173	-	86985168	5	11	ENSSSCG00000009568	-	ORF	*ZMYM5*
11	87121213	87121213	-	87121213	3	3	ENSSSCG00000022957	-	intron	---
11	87284453	87284455	-	87284455	13	16	ENSSSCG00000009572	-	intron	*RNF17*
12	3830	3834	+	3833	9	16	---	---	intergenic	---

12	4104	4104	+	4104	6	6	---	---	intergenic	---
12	16454	16456	-	16456	9	16	---	---	intergenic	---
12	32840	32844	-	32843	21	37	ENSSSCG00000028156	-	3'UTR	*OGFOD3*
12	33588	33588	-	33588	3	3	ENSSSCG00000028156	-	intron	*OGFOD3*
12	60084	60085	+	60084	9	12	---	---	intergenic	---
12	105351	105358	+	105351	13	17	ENSSSCG00000017126	+	3'UTR	*NARF*
12	175640	175640	+	175640	5	5	ENSSSCG00000017125	+	3'UTR	*FOXK2*
							ENSSSCG00000017125	+	intron	*FOXK2*
12	176865	176865	+	176865	5	5	ENSSSCG00000017125	+	3'UTR	*FOXK2*
12	190402	190407	-	190402	19	29	ENSSSCG00000017133	-	3'UTR	*WDR45L*
12	221086	221089	-	221086	4	7	XLOC_017240	-	predicted	---
12	269278	269280	+	269279	8	15	ENSSSCG00000017134	+	3'UTR	*FN3KRP*
12	286971	286979	+	286979	15	49	ENSSSCG00000017131	+	3'UTR	*FN3K*
12	410744	410747	+	410747	13	19	ENSSSCG00000017136	+	3'UTR	*TBCD*
12	411339	411339	-	411339	6	6	ENSSSCG00000027229	-	3'UTR	*B3GNTL1*
12	562177	562181	+	562177	8	11	XLOC_016367	+	predicted	---
12	562455	562458	+	562455	9	13	XLOC_016367	+	predicted	---
12	967556	967556	+	967556	9	9	ENSSSCG00000024018	+	3'UTR	*SLC16A3*
12	969945	969959	-	969959	6	13	XLOC_017256	-	predicted	---
12	971008	971008	-	971008	18	18	XLOC_017256	-	predicted	---
12	1048579	1048583	-	1048582	55	118	ENSSSCG00000023044	-	3'UTR	---
12	1127291	1127305	+	1127305	11	21	XLOC_016381	+	predicted	---
12	1128423	1128423	+	1128423	3	3	XLOC_016381	+	predicted	---
12	1128794	1128796	+	1128794	5	9	XLOC_016381	+	predicted	---
12	1131472	1131488	-	1131482	17	82	XLOC_017260	-	predicted	---
12	1131550	1131550	+	1131550	3	3	---	---	intergenic	---

12	1140192	1140201	+	1140196	5	21	ENSSSCG00000029616	+	3'UTR	*FAAP100*
12	1165058	1165058	+	1165058	3	3	ENSSSCG00000028355	+	ORF	*BETA-ACTIN*
12	1165491	1165491	+	1165491	4	4	ENSSSCG00000028355	+	3'UTR	*BETA-ACTIN*
12	1165894	1165894	+	1165894	4	4	ENSSSCG00000028355	+	3'UTR	*BETA-ACTIN*
12	1166007	1166035	+	1166032	787	2978	XLOC_016385	+	predicted	---
12	1424135	1424141	+	1424135	20	27	ENSSSCG00000022317	+	3'UTR	*SLC38A10*
12	1439239	1439239	+	1439239	6	6	ENSSSCG00000028593	+	3'UTR	*ENTHD2*
12	1515857	1515857	-	1515857	5	5	XLOC_017272	-	predicted	---
12	1597556	1597558	-	1597556	6	12	ENSSSCG00000017140	-	3'UTR	*CHMP6*
12	1598115	1598117	-	1598115	6	9	ENSSSCG00000017140	-	3'UTR	*CHMP6*
12	1614076	1614076	-	1614076	7	7	ENSSSCG00000017139	-	3'UTR	---
12	1798891	1798891	-	1798891	3	3	XLOC_017275	-	predicted	---
12	1963141	1963141	-	1963141	8	8	ENSSSCG00000028685	-	intron	---
12	2150665	2150665	-	2150665	3	3	ENSSSCG00000017148	-	intron	---
12	2199560	2199567	+	2199560	12	18	XLOC_016397	+	predicted	---
12	2293204	2293227	+	2293224	42	143	ENSSSCG00000017155	+	3'UTR	*EIF4A3*
12	2297282	2297291	-	2297289	58	105	XLOC_017281	-	predicted	---
12	2412097	2412099	-	2412097	6	12	---	---	intergenic	---
12	2457926	2457926	+	2457926	6	6	ENSSSCG00000017159	+	intron	*TBC1D16*
12	2531086	2531086	+	2531086	3	3	XLOC_016403	+	predicted	---
12	2597523	2597523	+	2597523	6	6	ENSSSCG00000017160	+	3'UTR	*CBX4*
12	2640304	2640304	-	2640304	3	3	ENSSSCG00000024261	-	3'UTR	*CBX2*
12	2766069	2766069	-	2766069	37	37	---	---	intergenic	---
12	3314289	3314302	+	3314295	12	42	ENSSSCG00000017164	+	intron	*TIMP-2*
12	3337901	3337927	+	3337917	124	247	ENSSSCG00000017164	+	3'UTR	*TIMP-2*
12	3340439	3340446	+	3340445	31	79	XLOC_016411	+	predicted	---

12	3385144	3385144	+	3385144	5	5	ENSSSCG00000017165	+	intron	*USP36*
12	3386802	3386802	+	3386802	7	7	ENSSSCG00000017165	+	3'UTR	*USP36*
12	3388370	3388370	+	3388370	11	11	ENSSSCG00000017165	+	3'UTR	*USP36*
12	3522716	3522718	+	3522717	13	20	XLOC_016413	+	predicted	---
12	3700547	3700550	-	3700549	10	21	---	---	intergenic	---
12	3700574	3700578	+	3700576	12	36	---	---	intergenic	---
12	3701684	3701684	+	3701684	3	3	XLOC_016415	+	predicted	---
12	3906811	3906811	-	3906811	16	16	---	---	intergenic	---
12	4002061	4002069	-	4002061	38	62	XLOC_017301	-	predicted	---
12	4177229	4177229	+	4177229	6	6	---	---	intergenic	---
12	4186761	4186762	-	4186762	8	11	ENSSSCG00000017169	-	3'UTR	*SEC14L1*
12	4276506	4276508	+	4276506	21	32	XLOC_016427	+	predicted	---
12	4288312	4288312	-	4288312	3	3	XLOC_017308	-	predicted	---
12	4289747	4289747	+	4289747	3	3	XLOC_016428	+	predicted	---
12	4629773	4629779	-	4629773	13	19	ENSSSCG00000029343	-	3'UTR	*MFSD11*
12	4654906	4654907	+	4654906	10	15	ENSSSCG00000023148	+	3'UTR	*SRSF2*
12	4655627	4655642	+	4655629	42	166	ENSSSCG00000023148	+	3'UTR	*SRSF2*
12	4655902	4655905	-	4655905	4	7	XLOC_017311	-	predicted	---
12	4656262	4656262	+	4656262	5	5	ENSSSCG00000023148	+	3'UTR	*SRSF2*
12	4666790	4666791	+	4666791	9	12	ENSSSCG00000022373	+	intron	*JMJD6*
12	4708864	4708865	+	4708865	14	17	XLOC_016433	+	predicted	---
12	4711988	4711988	+	4711988	3	3	XLOC_016433	+	predicted	---
12	4716292	4716292	+	4716292	3	3	XLOC_016433	+	predicted	---
12	4724505	4724505	-	4724505	3	3	---	---	intergenic	---
12	4805939	4805960	+	4805959	68	165	XLOC_016435	+	predicted	---
12	4807004	4807010	+	4807010	5	9	XLOC_016435	+	predicted	---

12	4807031	4807032	-	4807032	6	9	XLOC_017315	-	predicted	---
12	4840001	4840001	+	4840001	5	5	ENSSSCG00000017181	+	3'UTR	*CYGB*
12	4847412	4847412	+	4847412	3	3	---	---	intergenic	---
12	4887992	4887999	+	4887999	6	10	ENSSSCG00000023362	+	3'UTR	*RHBDF2*
12	4902697	4902697	+	4902697	3	3	---	---	intergenic	---
12	5017799	5017799	+	5017799	8	8	XLOC_016439	+	predicted	---
12	5017870	5017870	+	5017870	4	4	XLOC_016439	+	predicted	---
12	5066186	5066186	+	5066186	5	5	ENSSSCG00000017184	+	intron	*PRPSAP1*
12	5070062	5070062	+	5070062	3	3	ENSSSCG00000017184	+	intron	*PRPSAP1*
12	5078200	5078207	+	5078202	44	127	ENSSSCG00000017184	+	3'UTR	*PRPSAP1*
12	5104522	5104523	+	5104523	4	7	XLOC_016441	+	predicted	---
12	5106926	5106934	-	5106933	34	99	ENSSSCG00000023423	-	3'UTR	*UBALD2*
12	5128918	5128918	-	5128918	7	7	---	---	intergenic	---
12	5253040	5253040	+	5253040	4	4	ENSSSCG00000017190	+	ORF	*EXOC7*
12	5253889	5253889	+	5253889	8	8	ENSSSCG00000017190	+	intron	*EXOC7*
12	5258802	5258811	+	5258809	44	76	ENSSSCG00000017190	+	3'UTR	*EXOC7*
12	5357760	5357766	+	5357762	20	70	ENSSSCG00000017191	+	3'UTR	*SRP68*
12	5386231	5386233	-	5386232	27	48	XLOC_017328	-	predicted	---
12	5428281	5428289	-	5428287	46	124	XLOC_017329	-	predicted	---
12	5438984	5438988	-	5438985	30	57	XLOC_017330	-	predicted	---
12	5465671	5465671	-	5465671	5	5	ENSSSCG00000017197	-	intron	---
12	5468050	5468052	-	5468050	8	19	XLOC_017331	-	predicted	---
12	5470357	5470357	-	5470357	3	3	ENSSSCG00000017198	-	3'UTR	*ACOX1*
12	5470408	5470420	-	5470410	165	324	ENSSSCG00000017198	-	3'UTR	*ACOX1*
12	5470775	5470775	-	5470775	3	3	ENSSSCG00000017198	-	3'UTR	*ACOX1*
12	5471436	5471436	-	5471436	3	3	ENSSSCG00000017198	-	3'UTR	*ACOX1*

12	5472206	5472242	-	5472206	4	8	ENSSSCG00000017198	-	3'UTR	*ACOX1*
12	5472301	5472329	-	5472308	6	20	ENSSSCG00000017198	-	3'UTR	*ACOX1*
12	5477861	5477862	-	5477861	13	16	ENSSSCG00000017198	-	ORF	*ACOX1*
12	5480014	5480014	-	5480014	3	3	ENSSSCG00000017198	-	ORF	*ACOX1*
12	5489527	5489527	-	5489527	3	3	XLOC_017331	-	predicted	---
12	5496458	5496458	+	5496458	3	3	ENSSSCG00000017199	+	3'UTR	*TRIM47*
12	5519942	5519949	+	5519948	63	144	ENSSSCG00000021672	+	3'UTR	*WBP2*
12	5519990	5519995	+	5519992	77	124	ENSSSCG00000021672	+	3'UTR	*WBP2*
12	5535816	5535816	+	5535816	7	7	XLOC_016453	+	predicted	---
12	5539232	5539232	-	5539232	10	10	XLOC_017334	-	predicted	---
12	5563042	5563043	-	5563042	5	9	ENSSSCG00000017201	-	intron	*UNK*
12	5575579	5575587	+	5575579	23	56	XLOC_016454	+	predicted	---
12	5578854	5578855	+	5578855	11	20	ENSSSCG00000017202	+	intron	*H3F3B*
12	5579906	5579906	+	5579906	4	4	ENSSSCG00000017202	+	ORF	*H3F3B*
12	5580091	5580091	+	5580091	4	4	ENSSSCG00000017202	+	3'UTR	*H3F3B*
12	5580292	5580307	+	5580295	290	594	ENSSSCG00000017202	+	3'UTR	*H3F3B*
12	5580530	5580543	+	5580536	139	284	ENSSSCG00000017202	+	3'UTR	*H3F3B*
12	5580645	5580648	+	5580648	14	17	ENSSSCG00000017202	+	3'UTR	*H3F3B*
12	5581106	5581126	+	5581108	16	72	ENSSSCG00000017202	+	3'UTR	*H3F3B*
12	5581354	5581366	+	5581354	33	57	ENSSSCG00000017202	+	3'UTR	*H3F3B*
12	5582152	5582160	+	5582152	11	25	ENSSSCG00000017202	+	3'UTR	*H3F3B*
12	5597561	5597564	+	5597564	20	34	ENSSSCG00000017203	+	3'UTR	*GALK1*
12	5641656	5641666	-	5641665	8	19	ENSSSCG00000017205	-	3'UTR	*SAP30BP*
12	5817163	5817184	+	5817169	44	187	ENSSSCG00000017210	+	3'UTR	*MRPS7*
12	5817352	5817355	-	5817354	26	43	ENSSSCG00000017209	-	3'UTR	*MIF4GD*
12	5824121	5824130	-	5824122	19	53	ENSSSCG00000017208	-	3'UTR	*SLC25A19*

12	5858722	5858723	-	5858722	16	22	XLOC_017340	-	predicted	---
12	5963335	5963338	-	5963335	6	10	XLOC_017341	-	predicted	---
12	6034857	6034858	+	6034857	11	15	ENSSSCG00000017211	+	3'UTR	*GGA3*
12	6035180	6035186	-	6035186	9	18	ENSSSCG00000017207	-	3'UTR	*NUP85*
12	6092354	6092358	+	6092357	22	54	ENSSSCG00000017212	+	3'UTR	*SUMO2*
12	6120130	6120139	+	6120133	227	488	ENSSSCG00000017213	+	3'UTR	*HN1*
12	6120909	6120911	+	6120910	22	41	XLOC_016467	+	predicted	---
12	6126602	6126602	-	6126602	3	3	ENSSSCG00000017214	-	3'UTR	*ARMC7*
12	6126603	6126606	+	6126605	7	16	ENSSSCG00000028210	+	3'UTR	*NT5C*
12	6147985	6147985	-	6147985	6	6	ENSSSCG00000017215	-	3'UTR	*SLC16A5*
12	6148494	6148494	-	6148494	3	3	ENSSSCG00000017215	-	3'UTR	*SLC16A5*
12	6227129	6227129	-	6227129	3	3	XLOC_017345	-	predicted	---
12	6238850	6238871	-	6238871	13	16	XLOC_017345	-	predicted	---
12	6238905	6238905	-	6238905	3	3	XLOC_017345	-	predicted	---
12	6357120	6357130	+	6357129	30	60	XLOC_016475	+	predicted	---
12	6359167	6359167	+	6359167	3	3	XLOC_016475	+	predicted	---
12	6359443	6359452	+	6359451	12	21	XLOC_016475	+	predicted	---
12	6373241	6373244	+	6373241	24	32	ENSSSCG00000017227	+	3'UTR	*FDXR*
12	6453203	6453204	+	6453204	23	28	ENSSSCG00000017231	+	3'UTR	*NAT9*
12	6455089	6455096	-	6455096	64	160	ENSSSCG00000017232	-	3'UTR	*SLC9A3R1*
12	6473507	6473507	-	6473507	4	4	XLOC_017355	-	predicted	---
12	6505275	6505275	+	6505275	19	19	ENSSSCG00000017234	+	3'UTR	*CD300LF*
							ENSSSCG00000017234	+	intron	*CD300LF*
12	6508420	6508420	+	6508420	3	3	ENSSSCG00000017234	+	intron	*CD300LF*
12	6520002	6520012	+	6520002	11	21	XLOC_016480	+	predicted	---
12	6619807	6619807	+	6619807	3	3	---	---	intergenic	---

12	6624937	6624937	+	6624937	13	13	ENSSSCG00000017236	+	3'UTR	*CD300C*
							ENSSSCG00000017236	+	ORF	---
12	6735206	6735219	-	6735213	11	18	---	---	intergenic	---
12	6741979	6741992	-	6741979	3	9	XLOC_017359	-	predicted	---
12	6785204	6785204	-	6785204	3	3	ENSSSCG00000024357	-	ORF	*DNAI2*
12	6787740	6787740	-	6787740	4	4	ENSSSCG00000024357	-	intron	*DNAI2*
12	6865499	6865503	-	6865500	15	26	ENSSSCG00000017238	-	3'UTR	*TTYH2*
12	6866312	6866312	-	6866312	3	3	ENSSSCG00000017238	-	3'UTR	*TTYH2*
12	6911836	6911842	-	6911839	31	126	ENSSSCG00000017239	-	3'UTR	*RPL38*
12	6911974	6911995	-	6911977	280	876	ENSSSCG00000017239	-	3'UTR	*RPL38*
12	6916453	6916457	-	6916456	16	27	ENSSSCG00000017239	-	intron	*RPL38*
12	7089747	7089747	-	7089747	3	3	---	---	intergenic	---
12	7653806	7653806	+	7653806	5	5	---	---	intergenic	---
12	7940479	7940489	+	7940484	31	118	XLOC_016491	+	predicted	---
12	7986959	7986959	-	7986959	16	16	XLOC_017369	-	predicted	---
12	7990705	7990713	-	7990708	42	59	ENSSSCG00000017246	-	3'UTR	*C17orf80*
12	8025468	8025481	+	8025470	22	36	XLOC_016494	+	predicted	---
12	8025523	8025531	+	8025523	11	32	XLOC_016494	+	predicted	---
12	8025780	8025792	+	8025786	47	94	XLOC_016494	+	predicted	---
12	8026369	8026371	-	8026370	20	27	XLOC_017370	-	predicted	---
12	8027204	8027204	-	8027204	3	3	ENSSSCG00000017248	-	intron	*COG1*
12	8215751	8215751	+	8215751	9	9	ENSSSCG00000017250	+	intron	*SLC39A11*
12	8328864	8328864	+	8328864	6	6	ENSSSCG00000017250	+	intron	*SLC39A11*
12	8768264	8768264	-	8768264	6	6	---	---	intergenic	---
12	8768725	8768725	-	8768725	7	7	---	---	intergenic	---
12	8942167	8942167	-	8942167	3	3	---	---	intergenic	---

12	9573778	9573778	+	9573778	3	3	---	---	intergenic	---
12	10370297	10370297	+	10370297	3	3	---	---	intergenic	---
12	10710709	10710713	+	10710712	49	130	XLOC_016509	+	predicted	---
12	10977547	10977549	+	10977549	6	9	---	---	intergenic	---
12	11009163	11009163	+	11009163	8	8	---	---	intergenic	---
12	11018240	11018240	+	11018240	105	105	---	---	intergenic	---
12	11071308	11071308	-	11071308	3	3	---	---	intergenic	---
12	11072533	11072544	-	11072533	3	9	---	---	intergenic	---
12	11102851	11102851	+	11102851	4	4	---	---	intergenic	---
12	11139497	11139497	-	11139497	3	3	ENSSSCG00000017254	-	3'UTR	*MKK6*
12	11150199	11150201	-	11150199	6	10	ENSSSCG00000017254	-	3'UTR	*MKK6*
							ENSSSCG00000017254	-	intron	*MKK6*
12	11150360	11150360	-	11150360	6	6	ENSSSCG00000017254	-	3'UTR	*MKK6*
							ENSSSCG00000017254	-	intron	*MKK6*
12	11212477	11212477	-	11212477	6	6	ENSSSCG00000017254	-	intron	*MKK6*
12	11359770	11359770	+	11359770	3	3	---	---	intergenic	---
12	11750737	11750737	-	11750737	4	4	ENSSSCG00000017257	-	ORF	---
12	11909478	11909478	+	11909478	4	4	---	---	intergenic	---
12	11943706	11943708	-	11943707	87	114	---	---	intergenic	---
12	12023089	12023089	+	12023089	3	3	---	---	intergenic	---
12	12083451	12083451	+	12083451	4	4	XLOC_016523	+	predicted	---
12	12124095	12124101	+	12124095	18	25	ENSSSCG00000017258	+	3'UTR	*FAM20A*
12	12128694	12128711	-	12128702	11	19	XLOC_017396	-	predicted	---
12	12129208	12129208	-	12129208	3	3	XLOC_017396	-	predicted	---
12	12130797	12130805	-	12130800	52	177	ENSSSCG00000017259	-	3'UTR	*PRKAR1A*
12	12130861	12130862	-	12130862	7	10	ENSSSCG00000017259	-	3'UTR	*PRKAR1A*

12	12138720	12138736	-	12138723	54	107	ENSSSCG00000017259	-	intron	*PRKAR1A*
12	12148263	12148268	+	12148268	6	10	---	---	intergenic	---
12	12151296	12151296	+	12151296	8	8	---	---	intergenic	---
12	12185002	12185002	+	12185002	5	5	ENSSSCG00000017260	+	intron	*WIPI1*
12	12218282	12218282	-	12218282	8	8	XLOC_017398	-	predicted	---
12	12218355	12218356	+	12218355	14	23	ENSSSCG00000017260	+	3'UTR	*WIPI1*
12	12219399	12219400	-	12219399	5	9	XLOC_017398	-	predicted	---
12	12341472	12341478	+	12341476	34	41	ENSSSCG00000017262	+	3'UTR	*SLC16A6*
12	12361867	12361887	-	12361887	62	110	ENSSSCG00000017264	-	3'UTR	*AMZ2*
12	12361911	12361939	-	12361936	33	89	ENSSSCG00000017264	-	3'UTR	*AMZ2*
12	12398823	12398837	-	12398827	4	11	XLOC_017402	-	predicted	---
12	12400218	12400218	-	12400218	8	8	XLOC_017402	-	predicted	---
12	12569583	12569583	+	12569583	4	4	---	---	intergenic	---
12	12706088	12706103	-	12706102	19	39	XLOC_017405	-	predicted	---
12	12745854	12745854	+	12745854	3	3	---	---	intergenic	---
12	13024550	13024550	-	13024550	4	4	XLOC_017405	-	predicted	---
12	13122158	13122158	+	13122158	3	3	ENSSSCG00000023992	+	intron	---
12	13143902	13143937	-	13143902	13	19	ENSSSCG00000030997	-	3'UTR	*APOH*
12	13143991	13143991	-	13143991	43	43	ENSSSCG00000030997	-	3'UTR	*APOH*
12	13144037	13144037	-	13144037	3	3	ENSSSCG00000030997	-	3'UTR	*APOH*
12	13146061	13146072	-	13146072	7	10	ENSSSCG00000030997	-	3'UTR	*APOH*
12	13146121	13146121	-	13146121	3	3	ENSSSCG00000030997	-	3'UTR	*APOH*
12	13149672	13149675	-	13149675	6	9	ENSSSCG00000030997	-	3'UTR	*APOH*
12	13150838	13150838	-	13150838	3	3	ENSSSCG00000030997	-	3'UTR	*APOH*
12	13153039	13153039	-	13153039	4	4	ENSSSCG00000030997	-	3'UTR	*APOH*
12	13201285	13201286	-	13201285	3	6	---	---	intergenic	---

12	13612856	13612860	+	13612856	3	6	XLOC_016535	+	predicted	---
12	13717011	13717011	-	13717011	3	3	XLOC_017409	-	predicted	---
12	13865358	13865358	-	13865358	3	3	ENSSSCG00000017272	-	3'UTR	*HELZ*
12	13865737	13865737	-	13865737	3	3	ENSSSCG00000017272	-	3'UTR	*HELZ*
12	14069042	14069042	-	14069042	16	16	ENSSSCG00000017273	-	3'UTR	*PSMD12*
12	14070956	14070958	-	14070956	11	19	ENSSSCG00000017273	-	3'UTR	*PSMD12*
12	14071031	14071043	-	14071037	31	66	ENSSSCG00000017273	-	3'UTR	*PSMD12*
12	14380359	14380359	+	14380359	4	4	XLOC_016544	+	predicted	---
12	14494926	14494926	+	14494926	3	3	XLOC_016551	+	predicted	---
12	14699364	14699364	+	14699364	3	3	ENSSSCG00000017277	+	intron	*PECAM1*
12	14717149	14717153	+	14717150	28	55	ENSSSCG00000017277	+	3'UTR	*PECAM1*
12	14856390	14856390	+	14856390	5	5	ENSSSCG00000017278	+	intron	*TEX2*
12	14861094	14861095	+	14861095	5	9	XLOC_016554	+	predicted	---
12	14861329	14861339	+	14861337	54	80	XLOC_016554	+	predicted	---
12	14862360	14862360	-	14862360	6	6	XLOC_017419	-	predicted	---
12	14862540	14862540	-	14862540	5	5	ENSSSCG00000019194	-	ORF	*SNORND104*
12	14869463	14869470	+	14869463	5	11	XLOC_016554	+	predicted	---
12	14954851	14954854	+	14954853	26	42	ENSSSCG00000017279	+	3'UTR	*ERN1*
12	14982964	14982964	+	14982964	23	23	ENSSSCG00000017280	+	3'UTR	*ICAM2*
							ENSSSCG00000017280	+	ORF	---
12	14985144	14985145	+	14985145	12	15	XLOC_016558	+	predicted	---
12	14988766	14988766	+	14988766	8	8	XLOC_016558	+	predicted	---
12	15058093	15058096	+	15058094	1101	1115	ENSSSCG00000017284	+	3'UTR	*GH*
12	15064775	15064783	-	15064776	22	60	---	---	intergenic	---
12	15092171	15092171	-	15092171	5	5	ENSSSCG00000017287	-	3'UTR	*PSMC5*
12	15092196	15092205	+	15092204	19	61	ENSSSCG00000017286	+	3'UTR	*SMARCD2*

12	15092249	15092280	-	15092260	258	676	ENSSSCG00000017287	-	3'UTR	*PSMC5*
12	15092692	15092693	-	15092692	6	9	ENSSSCG00000017287	-	ORF	*PSMC5*
12	15092864	15092866	-	15092866	5	8	ENSSSCG00000017287	-	ORF	*PSMC5*
12	15093033	15093033	-	15093033	7	7	ENSSSCG00000017287	-	ORF	*PSMC5*
12	15094300	15094300	-	15094300	6	6	ENSSSCG00000017287	-	ORF	*PSMC5*
12	15104411	15104415	+	15104415	6	9	XLOC_016564	+	predicted	---
12	15104458	15104462	+	15104462	5	12	XLOC_016564	+	predicted	---
12	15104509	15104513	-	15104510	24	43	ENSSSCG00000017289	-	3'UTR	*DDX42*
12	15104579	15104588	-	15104588	8	11	ENSSSCG00000017289	-	3'UTR	*DDX42*
12	15175616	15175621	+	15175620	9	32	---	---	intergenic	---
12	15210367	15210370	+	15210369	28	58	XLOC_016566	+	predicted	---
12	15214385	15214397	+	15214391	41	111	ENSSSCG00000017291	+	3'UTR	*LIMD2*
12	15219014	15219034	-	15219016	43	129	ENSSSCG00000024813	-	intron	*MAP3K3*
12	15290653	15290653	-	15290653	3	3	---	---	intergenic	---
12	15295167	15295177	-	15295167	4	18	ENSSSCG00000017293	-	3'UTR	*TACO1*
12	15306442	15306450	-	15306450	21	30	XLOC_017433	-	predicted	---
12	15310866	15310868	-	15310867	28	40	XLOC_017433	-	predicted	---
12	15362297	15362297	-	15362297	3	3	XLOC_017435	-	predicted	---
12	15362557	15362565	-	15362557	7	17	---	---	intergenic	---
12	15381356	15381358	-	15381358	16	25	ENSSSCG00000017296	-	ORF	---
12	15389989	15389989	-	15389989	8	8	---	---	intergenic	---
12	15425203	15425205	+	15425204	4	10	ENSSSCG00000017297	+	intron	*CYB561*
12	15433122	15433122	+	15433122	4	4	ENSSSCG00000017297	+	3'UTR	*CYB561*
12	15433700	15433700	+	15433700	4	4	ENSSSCG00000017297	+	3'UTR	*CYB561*
12	15585385	15585385	-	15585385	3	3	ENSSSCG00000017298	-	intron	*TANC2*
12	15835087	15835087	-	15835087	3	3	---	---	intergenic	---

12	15896877	15896877	+	15896877	6	6	XLOC_016571	+	predicted	---
12	15906197	15906197	+	15906197	5	5	ENSSSCG00000017299	+	intron	*MARCH10*
12	15940996	15940996	+	15940996	3	3	ENSSSCG00000017299	+	intron	*MARCH10*
12	15944619	15944621	+	15944619	11	15	---	---	intergenic	---
12	16019890	16019898	-	16019890	8	14	ENSSSCG00000017301	-	3'UTR	*TLK2*
12	16021550	16021557	-	16021552	27	64	ENSSSCG00000017301	-	3'UTR	*TLK2*
12	16021968	16021968	-	16021968	4	4	ENSSSCG00000017301	-	3'UTR	*TLK2*
12	16022019	16022019	-	16022019	4	4	ENSSSCG00000017301	-	3'UTR	*TLK2*
12	16113529	16113529	-	16113529	4	4	---	---	intergenic	---
12	16717962	16717962	-	16717962	48	48	ENSSSCG00000017306	-	3'UTR	*CD61*
12	16791745	16791745	-	16791745	9	9	---	---	intergenic	---
12	16798625	16798625	-	16798625	5	5	XLOC_017449	-	predicted	---
12	16876199	16876216	+	16876215	11	26	ENSSSCG00000017308	+	intron	*CDC27*
12	16905442	16905442	+	16905442	5	5	ENSSSCG00000017308	+	3'UTR	*CDC27*
12	17120649	17120650	+	17120649	8	11	ENSSSCG00000017310	+	3'UTR	*KANSL1*
12	17122141	17122141	+	17122141	4	4	ENSSSCG00000017310	+	3'UTR	*KANSL1*
12	17123497	17123497	-	17123497	6	6	ENSSSCG00000017311	-	3'UTR	*MAPT*
12	17127041	17127041	-	17127041	4	4	ENSSSCG00000017311	-	3'UTR	*MAPT*
12	17354773	17354780	-	17354779	17	50	XLOC_017451	-	predicted	---
12	17361687	17361687	+	17361687	4	4	XLOC_016580	+	predicted	---
12	17403476	17403478	-	17403476	4	7	ENSSSCG00000017313	-	intron	---
12	17610426	17610430	+	17610426	5	8	ENSSSCG00000017316	+	intron	---
12	17655337	17655337	+	17655337	5	5	ENSSSCG00000017316	+	3'UTR	---
12	17656807	17656807	+	17656807	9	9	---	---	intergenic	---
12	17662991	17662991	-	17662991	25	25	XLOC_017455	-	predicted	---
12	17742261	17742290	+	17742261	5	8	XLOC_016586	+	predicted	---

12	17745158	17745158	+	17745158	4	4	XLOC_016586	+	predicted	---
12	17873066	17873073	+	17873072	19	42	ENSSSCG00000030183	+	3'UTR	---
12	17874359	17874360	+	17874360	5	8	ENSSSCG00000030183	+	3'UTR	---
12	17874949	17874949	+	17874949	4	4	ENSSSCG00000030183	+	3'UTR	---
12	17875504	17875504	+	17875504	3	3	ENSSSCG00000030183	+	3'UTR	---
12	17911663	17911664	-	17911663	5	8	---	---	intergenic	---
12	17924820	17924823	+	17924820	16	38	XLOC_016590	+	predicted	---
12	17924960	17924961	+	17924960	24	34	XLOC_016590	+	predicted	---
12	17989524	17989524	+	17989524	7	7	---	---	intergenic	---
12	18129897	18129902	+	18129897	10	16	XLOC_016593	+	predicted	---
12	18202111	18202111	+	18202111	4	4	ENSSSCG00000017325	+	3'UTR	*PLEKHM1*
12	18311006	18311006	+	18311006	3	3	XLOC_016595	+	predicted	---
12	18371561	18371561	-	18371561	3	3	XLOC_017465	-	predicted	---
12	18431634	18431634	+	18431634	3	3	XLOC_016601	+	predicted	---
12	18441626	18441635	+	18441630	42	151	XLOC_016602	+	predicted	---
12	18447479	18447479	-	18447479	17	17	XLOC_017467	-	predicted	---
12	18447517	18447520	+	18447519	16	25	XLOC_016604	+	predicted	---
12	18500140	18500145	-	18500140	16	19	XLOC_017468	-	predicted	---
12	18515739	18515745	-	18515739	9	14	XLOC_017468	-	predicted	---
12	18551167	18551167	+	18551167	3	3	ENSSSCG00000017338	+	3'UTR	*PLCD3*
12	18552092	18552095	-	18552095	14	20	ENSSSCG00000017339	-	3'UTR	*NMT1*
12	18579474	18579474	-	18579474	3	3	ENSSSCG00000017339	-	intron	*NMT1*
12	18588820	18588820	+	18588820	3	3	ENSSSCG00000017340	+	intron	*DCAKD*
12	18611122	18611125	+	18611124	7	18	ENSSSCG00000017340	+	3'UTR	*DCAKD*
12	18710730	18710730	-	18710730	3	3	XLOC_017472	-	predicted	---
12	18711294	18711303	+	18711301	7	21	ENSSSCG00000017343	+	3'UTR	*GFAP*

12	18729447	18729447	+	18729447	4	4	ENSSSCG00000017346	+	intron	*EFTUD2*
12	18759594	18759634	+	18759613	10	71	ENSSSCG00000017346	+	3'UTR	*EFTUD2*
12	18760071	18760071	-	18760071	3	3	XLOC_017473	-	predicted	---
12	18791351	18791351	+	18791351	3	3	XLOC_016614	+	predicted	---
12	18932937	18932937	+	18932937	4	4	ENSSSCG00000017351	+	ORF	*C17orf104*
12	18942859	18942860	+	18942860	8	13	ENSSSCG00000017351	+	3'UTR	*C17orf104*
12	18945660	18945660	-	18945660	4	4	ENSSSCG00000017352	-	3'UTR	---
12	19179151	19179151	-	19179151	3	3	ENSSSCG00000017358	-	3'UTR	*SLC4A1*
12	19216276	19216285	+	19216281	61	251	ENSSSCG00000017355	+	3'UTR	*GRN*
12	19231695	19231697	-	19231696	7	14	ENSSSCG00000017357	-	intron	*ITGA2B*
12	19256377	19256378	-	19256377	20	29	---	---	intergenic	---
12	19263189	19263195	-	19263189	13	20	XLOC_017482	-	predicted	---
12	19274498	19274500	+	19274499	20	41	XLOC_016626	+	predicted	---
12	19275098	19275114	-	19275104	158	332	ENSSSCG00000030167	-	3'UTR	*SLC25A39*
12	19299561	19299563	+	19299563	6	13	ENSSSCG00000017360	+	ORF	---
12	19361979	19361988	+	19361986	36	82	ENSSSCG00000017365	+	3'UTR	*G6PC3*
12	19364065	19364065	-	19364065	5	5	XLOC_017489	-	predicted	---
12	19386550	19386550	+	19386550	3	3	ENSSSCG00000028150	+	intron	*LSM12*
12	19388990	19388994	+	19388992	67	120	ENSSSCG00000028150	+	intron	*LSM12*
12	19389793	19389798	+	19389798	4	7	ENSSSCG00000028150	+	3'UTR	*LSM12*
12	19390227	19390227	+	19390227	3	3	XLOC_016637	+	predicted	---
12	19391213	19391213	+	19391213	4	4	XLOC_016637	+	predicted	---
12	19405633	19405637	-	19405636	11	18	ENSSSCG00000024179	-	3'UTR	*NAGS*
12	19405658	19405658	+	19405658	3	3	ENSSSCG00000017371	+	3'UTR	*TMEM101*
12	19406604	19406606	-	19406605	29	56	ENSSSCG00000024179	-	3'UTR	*NAGS*
12	19444940	19444941	+	19444941	75	80	ENSSSCG00000025414	+	3'UTR	*PYY*

12	19517737	19517737	-	19517737	5	5	ENSSSCG00000023014	-	intron	*CD300LG*
12	19563985	19564008	-	19563996	79	219	XLOC_017495	-	predicted	---
12	19567420	19567433	-	19567421	11	20	ENSSSCG00000017373	-	intron	*C17orf105*
12	19579997	19580008	+	19579998	13	26	ENSSSCG00000020744	+	3'UTR	*DUSP3*
12	19580680	19580680	+	19580680	10	10	ENSSSCG00000020744	+	3'UTR	*DUSP3*
12	19583370	19583370	+	19583370	6	6	ENSSSCG00000020744	+	3'UTR	*DUSP3*
12	19665157	19665157	-	19665157	11	11	---	---	intergenic	---
12	19665183	19665194	+	19665192	30	57	XLOC_016645	+	predicted	---
12	19792425	19792428	+	19792427	13	25	ENSSSCG00000017380	+	3'UTR	*ARL4D*
							ENSSSCG00000017380	+	ORF	---
12	19792755	19792762	+	19792761	15	29	ENSSSCG00000017380	+	3'UTR	*ARL4D*
							ENSSSCG00000017380	+	ORF	---
12	19890624	19890624	+	19890624	6	6	ENSSSCG00000017378	+	3'UTR	*DHX8*
12	19892858	19892858	+	19892858	5	5	ENSSSCG00000017378	+	3'UTR	*DHX8*
12	19989403	19989403	+	19989403	3	3	ENSSSCG00000025507	+	3'UTR	*RPL27*
12	20002216	20002225	+	20002224	32	64	ENSSSCG00000029763	+	3'UTR	*IFI35*
12	20003138	20003148	-	20003146	90	143	ENSSSCG00000024059	-	3'UTR	*VAT1*
12	20015774	20015778	+	20015778	8	15	ENSSSCG00000029460	+	3'UTR	*RND2*
12	20029734	20029734	-	20029734	14	14	XLOC_017501	-	predicted	---
12	20108335	20108335	+	20108335	3	3	ENSSSCG00000030211	+	ORF	*NBR1*
12	20114979	20114979	+	20114979	3	3	ENSSSCG00000030211	+	ORF	*NBR1*
12	20124824	20124833	+	20124830	64	161	ENSSSCG00000030211	+	3'UTR	*NBR1*
12	20126399	20126409	+	20126406	22	79	ENSSSCG00000030211	+	3'UTR	*NBR1*
12	20172983	20172995	+	20172995	7	15	ENSSSCG00000024536	+	3'UTR	*RUNDC1*
12	20242733	20242734	-	20242733	6	9	XLOC_017507	-	predicted	---
12	20243257	20243267	-	20243257	8	14	XLOC_017507	-	predicted	---

12	20244719	20244729	-	20244720	18	67	XLOC_017507	-	predicted	---
12	20263184	20263184	+	20263184	4	4	ENSSSCG00000017386	+	ORF	*BECN1*
12	20263268	20263268	+	20263268	9	9	ENSSSCG00000017386	+	intron	*BECN1*
12	20268372	20268394	+	20268394	4	7	ENSSSCG00000017386	+	3'UTR	*BECN1*
12	20268430	20268434	-	20268432	32	75	XLOC_017508	-	predicted	---
12	20268548	20268548	-	20268548	3	3	XLOC_017508	-	predicted	---
12	20268909	20268928	+	20268919	25	104	ENSSSCG00000017386	+	3'UTR	*BECN1*
12	20269176	20269176	+	20269176	4	4	XLOC_016658	+	predicted	---
12	20269294	20269295	+	20269294	3	6	XLOC_016658	+	predicted	---
12	20269402	20269402	-	20269402	5	5	XLOC_017508	-	predicted	---
12	20272394	20272394	-	20272394	3	3	ENSSSCG00000017387	-	intron	*WNK4*
12	20272796	20272805	-	20272796	11	16	ENSSSCG00000017387	-	ORF	*WNK4*
12	20273280	20273280	-	20273280	3	3	ENSSSCG00000017387	-	intron	*WNK4*
12	20278910	20278917	+	20278912	75	146	ENSSSCG00000017388	+	3'UTR	*COA3*
12	20304799	20304807	-	20304807	6	9	ENSSSCG00000017387	-	intron	*WNK4*
12	20346940	20346973	-	20346948	7	26	ENSSSCG00000017389	-	3'UTR	*RAMP2*
12	20364501	20364501	+	20364501	3	3	ENSSSCG00000022199	+	3'UTR	*TUBG2*
12	20427180	20427187	-	20427186	89	106	ENSSSCG00000017394	-	3'UTR	---
12	20438311	20438311	+	20438311	90	90	ENSSSCG00000017395	+	intron	*FAM134C*
12	20441894	20441895	+	20441894	11	20	ENSSSCG00000017395	+	intron	*FAM134C*
12	20455995	20455998	+	20455995	8	11	ENSSSCG00000017395	+	3'UTR	*FAM134C*
12	20462462	20462470	-	20462463	23	56	ENSSSCG00000017397	-	3'UTR	*MLX*
12	20462861	20462862	+	20462862	10	13	XLOC_016666	+	predicted	---
12	20471069	20471079	-	20471076	35	102	ENSSSCG00000017398	-	3'UTR	*COASY*
12	20485025	20485027	-	20485025	9	18	---	---	intergenic	---
12	20501314	20501315	-	20501314	17	24	ENSSSCG00000017401	-	3'UTR	---

12	20692083	20692083	+	20692083	7	7	ENSSSCG00000017402	+	intron	*STAT5A*
12	20708609	20708609	+	20708609	3	3	XLOC_016669	+	predicted	---
12	20709838	20709847	-	20709840	99	259	XLOC_017515	-	predicted	---
12	20711582	20711582	-	20711582	4	4	XLOC_017515	-	predicted	---
12	20718928	20718928	-	20718928	3	3	ENSSSCG00000017403	-	ORF	*STAT3*
12	20862155	20862161	+	20862155	5	8	ENSSSCG00000017406	+	3'UTR	*STAT5B*
12	20864509	20864517	+	20864509	7	11	XLOC_016669	+	predicted	---
12	20925516	20925516	+	20925516	7	7	ENSSSCG00000017412	+	3'UTR	*RAB5C*
12	20926105	20926111	+	20926107	22	43	ENSSSCG00000017412	+	3'UTR	*RAB5C*
12	20926887	20926913	-	20926896	1379	2263	---	---	intergenic	---
12	20927341	20927341	+	20927341	3	3	---	---	intergenic	---
12	20927416	20927416	+	20927416	5	5	---	---	intergenic	---
12	20937621	20937624	+	20937622	9	23	ENSSSCG00000017414	+	3'UTR	*KAT2A*
12	20946826	20946828	+	20946828	10	14	ENSSSCG00000017416	+	3'UTR	*DHX58*
12	21007688	21007688	-	21007688	17	17	ENSSSCG00000017418	-	3'UTR	*NKIRAS2*
12	21015252	21015252	-	21015252	3	3	---	---	intergenic	---
12	21046513	21046515	+	21046514	22	31	ENSSSCG00000017419	+	3'UTR	*DNAJC7*
12	21047622	21047625	+	21047622	3	6	---	---	intergenic	---
12	21048291	21048294	-	21048294	15	28	ENSSSCG00000017420	-	3'UTR	*CNP*
12	21057563	21057566	-	21057564	17	35	XLOC_017519	-	predicted	---
12	21057790	21057818	-	21057790	4	7	ENSSSCG00000017415	-	ORF	*TTC25*
12	21123390	21123390	+	21123390	6	6	ENSSSCG00000021910	+	3'UTR	*GAST*
12	21145387	21145391	-	21145387	4	7	XLOC_017522	-	predicted	---
12	21185705	21185708	-	21185708	17	20	ENSSSCG00000017429	-	3'UTR	*P3H4*
12	21278752	21278753	+	21278752	3	6	---	---	intergenic	---
12	21280515	21280515	+	21280515	3	3	ENSSSCG00000017430	+	3'UTR	*EIF1*

12	21280559	21280572	+	21280561	325	1270	ENSSSCG00000017430	+	3'UTR	*EIF1*
12	21281183	21281195	+	21281186	186	492	ENSSSCG00000017430	+	3'UTR	*EIF1*
12	21298239	21298241	+	21298239	7	16	---	---	intergenic	---
12	21307932	21307932	+	21307932	14	14	---	---	intergenic	---
12	21433464	21433478	-	21433471	9	26	XLOC_017526	-	predicted	---
12	21506539	21506539	-	21506539	3	3	---	---	intergenic	---
12	21540273	21540273	+	21540273	3	3	ENSSSCG00000017448	+	3'UTR	*KRT19*
12	21750851	21750851	+	21750851	4	4	---	---	intergenic	---
12	21778906	21778906	-	21778906	3	3	ENSSSCG00000017464	-	3'UTR	*KRT25*
12	21842084	21842084	-	21842084	35	35	XLOC_017543	-	predicted	---
12	21962928	21962928	+	21962928	3	3	---	---	intergenic	---
12	22090759	22090759	-	22090759	4	4	XLOC_017548	-	predicted	---
12	22091765	22091772	-	22091767	49	108	XLOC_017548	-	predicted	---
12	22139671	22139671	-	22139671	3	3	---	---	intergenic	---
12	22174142	22174142	+	22174142	4	4	ENSSSCG00000017468	+	intron	*SMARCE1*
12	22270940	22270942	-	22270940	6	13	XLOC_017552	-	predicted	---
12	22273601	22273601	-	22273601	4	4	XLOC_017552	-	predicted	---
12	22275059	22275101	-	22275061	20	38	XLOC_017552	-	predicted	---
12	22275823	22275826	-	22275823	8	14	ENSSSCG00000017472	-	3'UTR	*IGFBP4*
12	22310375	22310375	+	22310375	8	8	ENSSSCG00000017473	+	intron	*TOP2A*
12	22312111	22312112	+	22312112	9	16	ENSSSCG00000017473	+	intron	*TOP2A*
12	22319491	22319491	+	22319491	3	3	ENSSSCG00000017473	+	intron	*TOP2A*
12	22319943	22319943	+	22319943	12	12	ENSSSCG00000017473	+	intron	*TOP2A*
12	22329193	22329193	+	22329193	5	5	ENSSSCG00000017473	+	intron	*TOP2A*
12	22329400	22329400	+	22329400	8	8	ENSSSCG00000017473	+	intron	*TOP2A*
12	22330939	22330939	+	22330939	3	3	ENSSSCG00000017473	+	intron	*TOP2A*

12	22331324	22331324	+	22331324	3	3	ENSSSCG00000017473	+	intron	*TOP2A*
12	22337484	22337487	+	22337485	72	101	ENSSSCG00000017473	+	3'UTR	*TOP2A*
12	22356039	22356039	-	22356039	7	7	XLOC_017553	-	predicted	---
12	22356828	22356830	-	22356828	4	7	XLOC_017553	-	predicted	---
12	22384717	22384718	+	22384717	4	8	---	---	intergenic	---
12	22425010	22425011	+	22425010	3	6	XLOC_016712	+	predicted	---
12	22501775	22501775	+	22501775	5	5	XLOC_016713	+	predicted	---
12	22508561	22508561	+	22508561	4	4	ENSSSCG00000017476	+	intron	*MSL1*
12	22512855	22512855	+	22512855	4	4	ENSSSCG00000017476	+	3'UTR	*MSL1*
12	22515228	22515230	+	22515230	19	30	ENSSSCG00000017476	+	3'UTR	*MSL1*
12	22531338	22531350	+	22531338	3	6	---	---	intergenic	---
12	22565028	22565036	-	22565036	24	53	ENSSSCG00000017477	-	3'UTR	*CASC3*
12	22617185	22617185	+	22617185	3	3	ENSSSCG00000017479	+	3'UTR	*WIPF2*
12	22618326	22618326	+	22618326	5	5	ENSSSCG00000017479	+	3'UTR	*WIPF2*
12	22619608	22619608	+	22619608	3	3	ENSSSCG00000017479	+	3'UTR	*WIPF2*
12	22644036	22644036	+	22644036	4	4	---	---	intergenic	---
12	22802757	22802766	-	22802765	31	77	ENSSSCG00000017489	-	3'UTR	---
12	22875976	22876007	+	22875998	51	150	ENSSSCG00000017492	+	3'UTR	---
12	22897135	22897136	-	22897135	3	6	---	---	intergenic	---
12	22908909	22908930	-	22908925	6	19	XLOC_017565	-	predicted	---
12	22912459	22912461	-	22912459	43	54	XLOC_017565	-	predicted	---
12	22913241	22913255	-	22913244	252	600	ENSSSCG00000017493	-	3'UTR	*ZPBP2*
12	22913596	22913598	-	22913598	6	10	ENSSSCG00000017493	-	3'UTR	*ZPBP2*
12	22915360	22915360	-	22915360	12	12	---	---	intergenic	---
12	23019487	23019487	+	23019487	4	4	XLOC_016724	+	predicted	---
12	23047398	23047406	+	23047399	127	151	XLOC_016727	+	predicted	---

12	23049129	23049129	-	23049129	5	5	ENSSSCG00000017497	-	3'UTR	*ERBB2*
12	23079029	23079038	+	23079029	7	15	ENSSSCG00000017498	+	3'UTR	*PPP1R1B*
12	23105355	23105356	+	23105356	14	21	ENSSSCG00000017499	+	3'UTR	*STARD3*
12	23108618	23108619	+	23108619	25	28	XLOC_016729	+	predicted	---
12	23111890	23111892	+	23111891	5	12	XLOC_016730	+	predicted	---
12	23112473	23112473	-	23112473	9	9	ENSSSCG00000017502	-	3'UTR	*PGAP3*
12	23294232	23294232	+	23294232	5	5	---	---	intergenic	---
12	23325347	23325347	+	23325347	16	16	XLOC_016736	+	predicted	---
12	23347902	23347902	+	23347902	3	3	ENSSSCG00000017506	+	intron	*FBXL20*
12	23419406	23419406	+	23419406	3	3	XLOC_016737	+	predicted	---
12	23482215	23482301	-	23482222	1142	2081	ENSSSCG00000017509	-	3'UTR	*RPL19*
12	23482336	23482346	-	23482346	18	24	ENSSSCG00000017509	-	ORF	*RPL19*
12	23484595	23484595	-	23484595	7	7	ENSSSCG00000017509	-	ORF	*RPL19*
12	23486047	23486047	-	23486047	6	6	ENSSSCG00000017509	-	ORF	*RPL19*
12	23504608	23504608	+	23504608	3	3	ENSSSCG00000017510	+	intron	*CACNLB1*
12	23740748	23740749	+	23740748	8	12	ENSSSCG00000017513	+	intron	*NPEPPS*
12	23760607	23760618	+	23760607	3	6	XLOC_016743	+	predicted	---
12	23761703	23761709	+	23761708	19	30	XLOC_016743	+	predicted	---
12	23813218	23813218	+	23813218	4	4	ENSSSCG00000017514	+	3'UTR	*KPNB1*
12	23813392	23813400	+	23813399	24	47	ENSSSCG00000017514	+	3'UTR	*KPNB1*
12	23814435	23814435	+	23814435	4	4	ENSSSCG00000017514	+	3'UTR	*KPNB1*
12	23815246	23815252	+	23815252	6	9	ENSSSCG00000017514	+	3'UTR	*KPNB1*
12	23880236	23880236	+	23880236	4	4	XLOC_016746	+	predicted	---
12	23911156	23911156	-	23911156	3	3	---	---	intergenic	---
12	23930646	23930646	-	23930646	3	3	XLOC_017578	-	predicted	---
12	23954336	23954340	-	23954340	38	47	ENSSSCG00000017519	-	3'UTR	*MRPL10*

12	23955002	23955014	-	23955004	13	43	ENSSSCG00000017519	-	3'UTR	*MRPL10*
12	23967251	23967251	+	23967251	4	4	ENSSSCG00000017520	+	ORF	*LRRC46*
12	23967522	23967527	+	23967524	85	192	ENSSSCG00000017520	+	3'UTR	*LRRC46*
12	23968549	23968549	-	23968549	4	4	ENSSSCG00000017521	-	3'UTR	*SCRN2*
12	23977362	23977362	-	23977362	9	9	---	---	intergenic	---
12	23996977	23996977	+	23996977	6	6	ENSSSCG00000017523	+	intron	*PNPO*
12	23998367	23998371	+	23998367	4	8	XLOC_016750	+	predicted	---
12	24000019	24000022	+	24000022	5	12	---	---	intergenic	---
12	24179141	24179141	+	24179141	3	3	ENSSSCG00000017527	+	intron	*SKAP1*
12	24199131	24199132	+	24199132	8	11	ENSSSCG00000017527	+	3'UTR	*SKAP1*
12	24209914	24209914	-	24209914	11	11	ENSSSCG00000017528	-	3'UTR	*SNX11*
12	24223631	24223638	-	24223634	10	23	---	---	intergenic	---
12	24223661	24223661	+	24223661	5	5	---	---	intergenic	---
12	24242519	24242519	+	24242519	3	3	ENSSSCG00000017529	+	ORF	*CBX1*
12	24248109	24248110	+	24248109	9	12	ENSSSCG00000017529	+	3'UTR	*CBX1*
12	24277926	24277931	-	24277928	32	91	ENSSSCG00000017526	-	3'UTR	*COPZ2*
12	24758390	24758390	+	24758390	14	14	XLOC_016766	+	predicted	---
12	25004714	25004719	-	25004717	91	158	XLOC_017595	-	predicted	---
12	25023024	25023029	+	25023024	4	7	ENSSSCG00000017543	+	ORF	*CALCOCO2*
12	25030476	25030484	+	25030476	24	49	ENSSSCG00000017543	+	intron	*CALCOCO2*
12	25035246	25035246	+	25035246	3	3	ENSSSCG00000017543	+	ORF	*CALCOCO2*
12	25035354	25035354	+	25035354	5	5	ENSSSCG00000017543	+	3'UTR	*CALCOCO2*
12	25036122	25036174	+	25036127	28	78	ENSSSCG00000017543	+	3'UTR	*CALCOCO2*
12	25069485	25069487	+	25069486	49	64	ENSSSCG00000017544	+	3'UTR	*ATP5G1*
12	25097164	25097164	+	25097164	6	6	ENSSSCG00000025638	+	3'UTR	*UBE2Z*
12	25104173	25104173	-	25104173	4	4	---	---	intergenic	---

12	25245012	25245012	+	25245012	3	3	---	---	intergenic	---
12	25321970	25321970	-	25321970	6	6	ENSSSCG00000030269	-	intron	*B4GALNT2*
12	25383602	25383610	+	25383605	21	36	ENSSSCG00000027677	+	3'UTR	*ABI3*
12	25384671	25384672	-	25384672	23	27	XLOC_017601	-	predicted	---
12	25394530	25394530	+	25394530	3	3	---	---	intergenic	---
12	25403266	25403267	+	25403266	4	7	---	---	intergenic	---
12	25446943	25446943	-	25446943	4	4	ENSSSCG00000017546	-	ORF	*ZNF652*
12	25535908	25535913	-	25535913	13	34	ENSSSCG00000017549	-	3'UTR	---
12	25536650	25536670	-	25536662	53	142	ENSSSCG00000017549	-	3'UTR	---
12	25777213	25777213	+	25777213	3	3	ENSSSCG00000020796	+	3'UTR	*SPOP*
12	25777617	25777617	+	25777617	3	3	XLOC_016784	+	predicted	---
12	25778006	25778006	+	25778006	3	3	---	---	intergenic	---
12	25888584	25888611	-	25888591	29	73	ENSSSCG00000017550	-	3'UTR	---
12	25897369	25897372	-	25897369	23	29	XLOC_017608	-	predicted	---
12	25982135	25982135	+	25982135	7	7	ENSSSCG00000017553	+	ORF	*KAT7*
12	26009286	26009289	+	26009287	71	100	ENSSSCG00000017553	+	3'UTR	*KAT7*
12	26075484	26075484	-	26075484	3	3	---	---	intergenic	---
12	26335213	26335219	+	26335213	15	19	ENSSSCG00000027198	+	3'UTR	*PDK2*
12	26358245	26358251	-	26358251	6	9	ENSSSCG00000017584	-	3'UTR	*PPP1R9B*
12	26375538	26375538	+	26375538	3	3	---	---	intergenic	---
12	26388767	26388767	+	26388767	3	3	---	---	intergenic	---
12	26400475	26400502	-	26400477	166	341	ENSSSCG00000028776	-	3'UTR	*HILS1*
12	26407113	26407113	+	26407113	4	4	ENSSSCG00000017583	+	3'UTR	*SGCA*
12	26417210	26417211	-	26417210	36	41	ENSSSCG00000021015	-	ORF	---
12	26418960	26418960	-	26418960	3	3	ENSSSCG00000021015	-	ORF	---
12	26469604	26469604	-	26469604	3	3	ENSSSCG00000017581	-	ORF	*COL1A1*

12	26497395	26497395	-	26497395	4	4	ENSSSCG00000017580	-	3'UTR	*TMEM92*
12	26602107	26602108	+	26602107	7	10	ENSSSCG00000028019	+	3'UTR	*LRRC59*
12	26602307	26602314	+	26602309	8	23	ENSSSCG00000028019	+	3'UTR	*LRRC59*
12	26603465	26603466	-	26603465	7	11	XLOC_017623	-	predicted	---
12	26603710	26603720	+	26603718	54	152	ENSSSCG00000028019	+	3'UTR	*LRRC59*
12	26667375	26667375	+	26667375	4	4	XLOC_016802	+	predicted	---
12	26683287	26683287	-	26683287	3	3	XLOC_017625	-	predicted	---
12	26793748	26793754	-	26793748	4	12	---	---	intergenic	---
12	26841143	26841148	+	26841147	74	134	ENSSSCG00000017566	+	3'UTR	*ACSF2*
12	26952335	26952337	+	26952335	21	27	XLOC_016809	+	predicted	---
12	27116871	27116871	+	27116871	4	4	XLOC_016813	+	predicted	---
12	27117430	27117435	+	27117430	11	18	XLOC_016813	+	predicted	---
12	27117799	27117799	+	27117799	4	4	XLOC_016813	+	predicted	---
12	27118392	27118392	-	27118392	3	3	XLOC_017631	-	predicted	---
12	27150154	27150154	+	27150154	12	12	ENSSSCG00000017555	+	intron	*LUC7L3*
12	27170097	27170101	+	27170099	17	37	ENSSSCG00000017555	+	3'UTR	*LUC7L3*
12	27170164	27170166	+	27170165	11	18	XLOC_016814	+	predicted	---
12	27177010	27177010	-	27177010	3	3	XLOC_017632	-	predicted	---
12	27208419	27208419	+	27208419	3	3	XLOC_016815	+	predicted	---
12	27288907	27288907	+	27288907	4	4	ENSSSCG00000017557	+	3'UTR	*WFIKKN2*
12	27305670	27305670	+	27305670	3	3	---	---	intergenic	---
12	27309224	27309231	-	27309231	9	12	XLOC_017635	-	predicted	---
12	27310022	27310029	-	27310022	8	12	ENSSSCG00000017558	-	ORF	*TOB1*
12	27502903	27502921	+	27502905	69	117	ENSSSCG00000024673	+	3'UTR	*NME1*
12	27513372	27513385	+	27513382	368	910	ENSSSCG00000017591	+	3'UTR	*NME2*
12	27519114	27519120	-	27519114	11	16	ENSSSCG00000017592	-	3'UTR	*MBTD1*

12	27633679	27633681	+	27633680	9	16	ENSSSCG00000017593	+	3'UTR	*UTP18*
12	27769238	27769243	-	27769239	5	12	---	---	intergenic	---
12	28158931	28158932	-	28158931	6	10	---	---	intergenic	---
12	28158970	28158970	+	28158970	3	3	---	---	intergenic	---
12	28556309	28556309	-	28556309	3	3	---	---	intergenic	---
12	28556353	28556353	+	28556353	6	6	---	---	intergenic	---
12	28964305	28964305	-	28964305	8	8	---	---	intergenic	---
12	29178477	29178478	+	29178477	3	6	---	---	intergenic	---
12	29178646	29178646	+	29178646	5	5	---	---	intergenic	---
12	29902755	29902781	-	29902780	6	23	---	---	intergenic	---
12	29902808	29902813	+	29902812	15	40	---	---	intergenic	---
12	29918660	29918660	+	29918660	3	3	---	---	intergenic	---
12	30678789	30678814	+	30678802	25	97	XLOC_016826	+	predicted	---
12	31328412	31328412	-	31328412	4	4	---	---	intergenic	---
12	31496562	31496563	-	31496562	4	7	---	---	intergenic	---
12	31702060	31702060	+	31702060	6	6	ENSSSCG00000017601	+	intron	*TOM1L1*
12	32306865	32306865	+	32306865	3	3	XLOC_016833	+	predicted	---
12	32308893	32308893	+	32308893	6	6	XLOC_016833	+	predicted	---
12	32330387	32330387	+	32330387	4	4	---	---	intergenic	---
12	32361326	32361336	-	32361333	17	57	XLOC_017651	-	predicted	---
12	32410584	32410584	+	32410584	4	4	---	---	intergenic	---
12	32558139	32558139	+	32558139	3	3	---	---	intergenic	---
12	32675135	32675144	-	32675144	7	16	ENSSSCG00000017607	-	3'UTR	*TMEM100*
12	32771712	32771719	-	32771712	5	12	ENSSSCG00000017608	-	ORF	---
12	32812330	32812330	+	32812330	4	4	XLOC_016836	+	predicted	---
12	32812484	32812496	+	32812495	53	135	XLOC_016836	+	predicted	---

12	32904293	32904294	-	32904293	7	13	---	---	intergenic	---
12	33168985	33168985	-	33168985	5	5	---	---	intergenic	---
12	33319411	33319411	+	33319411	3	3	---	---	intergenic	---
12	33336408	33336408	+	33336408	3	3	---	---	intergenic	---
12	33634874	33634874	+	33634874	3	3	XLOC_016840	+	predicted	---
12	33634922	33634922	+	33634922	3	3	XLOC_016840	+	predicted	---
12	33668749	33668759	-	33668751	27	59	---	---	intergenic	---
12	33803857	33803857	+	33803857	5	5	---	---	intergenic	---
12	33931048	33931048	-	33931048	3	3	---	---	intergenic	---
12	33995016	33995028	-	33995023	94	164	ENSSSCG00000017617	-	3'UTR	*SCPEP1*
12	34061190	34061196	+	34061195	38	67	XLOC_016843	+	predicted	---
12	34078611	34078611	-	34078611	10	10	XLOC_017662	-	predicted	---
12	34122544	34122544	+	34122544	3	3	XLOC_016845	+	predicted	---
12	34134362	34134362	-	34134362	12	12	XLOC_017663	-	predicted	---
12	34147862	34147863	-	34147862	8	13	XLOC_017663	-	predicted	---
12	34328422	34328422	+	34328422	4	4	---	---	intergenic	---
12	34393229	34393229	+	34393229	5	5	---	---	intergenic	---
12	34574889	34574890	+	34574889	3	6	---	---	intergenic	---
12	34591635	34591635	+	34591635	4	4	---	---	intergenic	---
12	34597883	34597892	+	34597883	3	6	---	---	intergenic	---
12	34748523	34748592	+	34748541	29	109	---	---	intergenic	---
12	34793231	34793231	+	34793231	3	3	---	---	intergenic	---
12	34830765	34830765	+	34830765	3	3	XLOC_016849	+	predicted	---
12	34834493	34834494	+	34834493	5	9	XLOC_016849	+	predicted	---
12	34914602	34914602	-	34914602	7	7	---	---	intergenic	---
12	35030202	35030202	+	35030202	7	7	---	---	intergenic	---

12	35278976	35278976	-	35278976	3	3	XLOC_017674	-	predicted	---
12	35281517	35281517	+	35281517	3	3	ENSSSCG00000017626	+	ORF	*SRSF1*
12	35283901	35283901	+	35283901	47	47	ENSSSCG00000017626	+	3'UTR	*SRSF1*
12	35383173	35383178	-	35383177	41	66	ENSSSCG00000020962	-	3'UTR	*DYNLL2*
12	35384934	35384955	-	35384955	32	40	ENSSSCG00000020962	-	3'UTR	*DYNLL2*
12	35387187	35387187	-	35387187	4	4	ENSSSCG00000020962	-	ORF	*DYNLL2*
12	35722004	35722004	-	35722004	3	3	ENSSSCG00000028335	-	ORF	*U3*
12	35848353	35848354	+	35848353	9	13	---	---	intergenic	---
12	36000281	36000302	+	36000281	8	11	ENSSSCG00000017645	+	3'UTR	*TEX14*
12	36000363	36000402	+	36000401	20	35	ENSSSCG00000017645	+	3'UTR	*TEX14*
							ENSSSCG00000017645	+	ORF	---
12	36043151	36043161	+	36043160	23	34	ENSSSCG00000017643	+	3'UTR	*SEPT4*
12	36069940	36069945	+	36069940	16	25	ENSSSCG00000017642	+	3'UTR	*MTMR4*
12	36094129	36094129	+	36094129	4	4	ENSSSCG00000017641	+	intron	*HSF5*
12	36115777	36115777	+	36115777	3	3	ENSSSCG00000017641	+	3'UTR	*HSF5*
12	36117775	36117782	+	36117775	23	69	ENSSSCG00000017641	+	3'UTR	*HSF5*
12	36189991	36190000	+	36189999	18	34	ENSSSCG00000017639	+	3'UTR	*SUPT4H1*
							ENSSSCG00000017639	+	ORF	---
12	36190564	36190566	+	36190566	8	12	ENSSSCG00000017639	+	3'UTR	*SUPT4H1*
							ENSSSCG00000017639	+	ORF	---
12	36193620	36193628	+	36193620	4	10	XLOC_016870	+	predicted	---
12	36204377	36204377	+	36204377	7	7	XLOC_016871	+	predicted	---
12	36259327	36259327	-	36259327	4	4	XLOC_017696	-	predicted	---
12	36312760	36312761	+	36312760	3	6	XLOC_016874	+	predicted	---
12	36477588	36477588	+	36477588	5	5	ENSSSCG00000017647	+	3'UTR	*PPM1E*
12	36478920	36478929	-	36478929	5	12	ENSSSCG00000023727	-	3'UTR	*TRIM37*

12	36493332	36493333	-	36493333	7	10	ENSSSCG00000023727	-	intron	*TRIM37*
12	36623492	36623504	-	36623504	4	7	ENSSSCG00000017665	-	3'UTR	*DHX40*
12	36624278	36624279	-	36624279	6	10	ENSSSCG00000017665	-	3'UTR	*DHX40*
12	36671249	36671249	+	36671249	6	6	XLOC_016876	+	predicted	---
12	36671735	36671735	+	36671735	6	6	XLOC_016876	+	predicted	---
12	36705228	36705228	-	36705228	17	17	---	---	intergenic	---
12	36750496	36750496	+	36750496	9	9	---	---	intergenic	---
12	36811807	36811807	-	36811807	3	3	XLOC_017708	-	predicted	---
12	36827212	36827212	-	36827212	3	3	---	---	intergenic	---
12	36852055	36852061	-	36852061	7	23	---	---	intergenic	---
12	36852115	36852121	+	36852117	31	59	---	---	intergenic	---
12	36875246	36875246	-	36875246	7	7	---	---	intergenic	---
12	36930213	36930214	-	36930213	4	8	ENSSSCG00000030262	-	3'UTR	*GDPD1*
12	37193830	37193830	+	37193830	3	3	ENSSSCG00000017666	+	3'UTR	*CLTC*
12	37193903	37193903	+	37193903	4	4	ENSSSCG00000017666	+	3'UTR	*CLTC*
12	37199134	37199157	-	37199156	30	65	ENSSSCG00000017667	-	3'UTR	*PTRH2*
12	37339282	37339282	+	37339282	3	3	ENSSSCG00000017668	+	3'UTR	*VMP1*
12	37339365	37339365	+	37339365	4	4	ENSSSCG00000017668	+	3'UTR	*VMP1*
12	37339598	37339598	+	37339598	4	4	ENSSSCG00000017668	+	3'UTR	*VMP1*
12	37339760	37339762	+	37339761	23	31	ENSSSCG00000017668	+	3'UTR	*VMP1*
12	37341360	37341360	+	37341360	17	17	ENSSSCG00000017668	+	3'UTR	*VMP1*
12	37431478	37431478	+	37431478	6	6	ENSSSCG00000017670	+	3'UTR	*RPS6KB1*
12	37432184	37432184	+	37432184	4	4	ENSSSCG00000017670	+	3'UTR	*RPS6KB1*
12	37437558	37437564	-	37437559	39	83	XLOC_017716	-	predicted	---
12	37536377	37536377	+	37536377	3	3	ENSSSCG00000017671	+	ORF	*RNFT1*
12	37592558	37592558	+	37592558	3	3	---	---	intergenic	---

12	37679367	37679367	-	37679367	3	3	ENSSSCG00000017672	-	intron	*MED13*
12	37680498	37680498	-	37680498	3	3	ENSSSCG00000017672	-	intron	*MED13*
12	37719290	37719290	-	37719290	4	4	---	---	intergenic	---
12	37749883	37749883	+	37749883	4	4	ENSSSCG00000017674	+	3'UTR	---
12	37749974	37749974	+	37749974	3	3	ENSSSCG00000017674	+	3'UTR	---
12	37750227	37750227	+	37750227	4	4	ENSSSCG00000017674	+	3'UTR	---
12	37752831	37752831	+	37752831	4	4	ENSSSCG00000017674	+	3'UTR	---
12	37753478	37753478	+	37753478	3	3	XLOC_016888	+	predicted	---
12	37753556	37753557	+	37753557	12	16	XLOC_016888	+	predicted	---
12	38037584	38037584	+	38037584	3	3	---	---	intergenic	---
12	38195274	38195274	-	38195274	4	4	---	---	intergenic	---
12	38334431	38334431	-	38334431	6	6	ENSSSCG00000027060	-	intron	*TBX2*
12	38345068	38345068	-	38345068	4	4	XLOC_017723	-	predicted	---
12	38358895	38358895	-	38358895	5	5	---	---	intergenic	---
12	38370675	38370675	-	38370675	8	8	---	---	intergenic	---
12	38467603	38467603	-	38467603	5	5	---	---	intergenic	---
12	38493877	38493877	+	38493877	5	5	XLOC_016894	+	predicted	---
12	38544503	38544503	+	38544503	3	3	---	---	intergenic	---
12	38583086	38583086	+	38583086	6	6	---	---	intergenic	---
12	38759864	38759865	-	38759865	15	25	ENSSSCG00000017676	-	intron	*BCAS3*
12	38765896	38765896	-	38765896	4	4	ENSSSCG00000017676	-	intron	*BCAS3*
12	38921795	38921795	+	38921795	3	3	ENSSSCG00000026665	+	intron	---
12	38933621	38933621	+	38933621	3	3	ENSSSCG00000026665	+	ORF	---
12	38934133	38934141	+	38934141	14	29	ENSSSCG00000026665	+	3'UTR	---
12	38956622	38956622	-	38956622	18	18	ENSSSCG00000017676	-	intron	*BCAS3*
12	39003409	39003409	-	39003409	4	4	ENSSSCG00000017676	-	intron	*BCAS3*

12	39163541	39163541	-	39163541	3	3	---	---	intergenic	---
12	39181604	39181616	+	39181614	67	148	XLOC_016897	+	predicted	---
12	39188231	39188232	-	39188232	9	14	ENSSSCG00000017678	-	3'UTR	*APPBP2*
12	39192566	39192568	-	39192566	8	12	ENSSSCG00000017678	-	3'UTR	*APPBP2*
12	39242238	39242248	+	39242238	3	6	---	---	intergenic	---
12	39581230	39581231	-	39581231	7	11	XLOC_017734	-	predicted	---
12	39591084	39591084	+	39591084	3	3	---	---	intergenic	---
12	39729799	39729800	+	39729799	9	15	ENSSSCG00000017688	+	3'UTR	*GGNBP2*
12	39730206	39730207	+	39730206	6	10	ENSSSCG00000017688	+	3'UTR	*GGNBP2*
12	39741845	39741853	+	39741848	6	13	ENSSSCG00000017690	+	3'UTR	*DHRS11*
12	39748441	39748445	+	39748445	14	18	ENSSSCG00000017691	+	3'UTR	*MRM1*
12	39773646	39773646	-	39773646	3	3	---	---	intergenic	---
12	40079339	40079339	+	40079339	8	8	ENSSSCG00000017693	+	intron	*AATF*
12	40126465	40126465	+	40126465	3	3	ENSSSCG00000017693	+	intron	*AATF*
12	40180471	40180471	+	40180471	4	4	ENSSSCG00000017693	+	3'UTR	*AATF*
12	40198391	40198393	-	40198391	20	31	XLOC_017743	-	predicted	---
12	40412895	40412895	+	40412895	11	11	ENSSSCG00000017696	+	intron	*C17orf78*
12	40549424	40549426	+	40549426	20	35	XLOC_016915	+	predicted	---
12	40555636	40555636	-	40555636	5	5	ENSSSCG00000025775	-	intron	*TADA2A*
12	40620315	40620326	-	40620318	12	29	XLOC_017746	-	predicted	---
12	40621795	40621795	-	40621795	3	3	ENSSSCG00000026596	-	intron	*SYNRG*
12	40649580	40649580	-	40649580	3	3	ENSSSCG00000026596	-	intron	*SYNRG*
12	40671677	40671690	-	40671677	3	6	ENSSSCG00000026596	-	intron	*SYNRG*
12	40713954	40713954	-	40713954	11	11	ENSSSCG00000021275	-	3'UTR	*DDX52*
12	40739461	40739461	-	40739461	3	3	ENSSSCG00000021275	-	intron	*DDX52*
12	40895831	40895838	-	40895831	7	13	ENSSSCG00000025952	-	3'UTR	---

12	41032450	41032450	+	41032450	3	3	---	---	intergenic	---
12	41063234	41063246	+	41063234	23	27	ENSSSCG00000017700	+	3'UTR	*CCL3L1*
12	41091306	41091306	+	41091306	3	3	---	---	intergenic	---
12	41091971	41091971	-	41091971	161	161	---	---	intergenic	---
12	41098889	41098889	-	41098889	3	3	---	---	intergenic	---
12	41126805	41126811	+	41126809	13	58	XLOC_016925	+	predicted	---
12	41164921	41164968	+	41164922	209	319	ENSSSCG00000017702	+	3'UTR	*CCL23*
12	41165052	41165052	+	41165052	3	3	ENSSSCG00000017702	+	3'UTR	*CCL23*
12	41165119	41165133	+	41165123	28	51	ENSSSCG00000017702	+	3'UTR	*CCL23*
12	41185159	41185160	+	41185160	6	9	XLOC_016927	+	predicted	---
12	41187634	41187634	-	41187634	5	5	XLOC_017752	-	predicted	---
12	41190103	41190103	+	41190103	4	4	ENSSSCG00000017704	+	intron	*CCL16*
12	41190813	41190844	+	41190817	624	1511	ENSSSCG00000017704	+	3'UTR	*CCL16*
12	41200953	41200955	+	41200954	11	18	---	---	intergenic	---
12	41228595	41228602	+	41228601	163	181	ENSSSCG00000017705	+	3'UTR	*CCL5*
12	41256373	41256382	-	41256381	22	31	ENSSSCG00000017707	-	3'UTR	*TAF15*
12	41348711	41348711	+	41348711	4	4	---	---	intergenic	---
12	41398190	41398190	-	41398190	7	7	ENSSSCG00000017712	-	ORF	---
12	41446857	41446870	+	41446858	14	25	ENSSSCG00000017713	+	ORF	*PEX12*
12	41447894	41447895	-	41447894	4	7	XLOC_017756	-	predicted	---
12	41449261	41449261	-	41449261	6	6	XLOC_017756	-	predicted	---
12	41472944	41472944	+	41472944	7	7	ENSSSCG00000024115	+	intron	---
12	41476425	41476425	+	41476425	5	5	ENSSSCG00000024115	+	intron	---
12	41507326	41507327	+	41507327	7	11	ENSSSCG00000024115	+	3'UTR	---
12	41555514	41555535	+	41555520	17	74	ENSSSCG00000025796	+	3'UTR	*NLE1*
12	41556919	41556934	-	41556930	100	357	XLOC_017760	-	predicted	---

12	41560477	41560477	-	41560477	4	4	ENSSSCG00000021617	-	ORF	*FNDC8*
12	41579482	41579482	+	41579482	3	3	XLOC_016936	+	predicted	---
12	41710096	41710107	+	41710107	11	45	ENSSSCG00000026646	+	3'UTR	*RFFL*
12	41711707	41711707	+	41711707	17	17	ENSSSCG00000026646	+	3'UTR	*RFFL*
12	41712001	41712001	+	41712001	4	4	ENSSSCG00000026646	+	3'UTR	*RFFL*
12	41712134	41712134	+	41712134	4	4	ENSSSCG00000026646	+	3'UTR	*RFFL*
12	41715999	41716001	-	41716001	7	11	ENSSSCG00000024770	-	intron	---
12	41734148	41734149	+	41734148	3	6	ENSSSCG00000030491	+	3'UTR	*ZNF830*
							ENSSSCG00000030491	+	ORF	---
12	41771174	41771203	-	41771174	4	8	ENSSSCG00000021313	-	intron	*LIG3*
12	41776904	41776904	+	41776904	4	4	ENSSSCG00000022196	+	ORF	---
12	41789584	41789595	+	41789590	44	119	XLOC_016939	+	predicted	---
12	41907209	41907210	+	41907210	4	7	---	---	intergenic	---
12	41989963	41989963	-	41989963	3	3	---	---	intergenic	---
12	41999397	41999397	-	41999397	17	17	---	---	intergenic	---
12	42058281	42058281	+	42058281	5	5	---	---	intergenic	---
12	42458849	42458849	+	42458849	12	12	ENSSSCG00000017721	+	3'UTR	*CCL8*
12	42473936	42473936	-	42473936	3	3	---	---	intergenic	---
12	42516103	42516104	+	42516103	5	10	ENSSSCG00000017723	+	3'UTR	*CCL2*
12	42657197	42657198	+	42657198	5	8	---	---	intergenic	---
12	42689775	42689775	-	42689775	3	3	XLOC_017774	-	predicted	---
12	43003627	43003627	-	43003627	3	3	---	---	intergenic	---
12	43269457	43269457	+	43269457	6	6	---	---	intergenic	---
12	43408595	43408595	-	43408595	7	7	---	---	intergenic	---
12	43434199	43434199	+	43434199	12	12	---	---	intergenic	---
12	43539155	43539155	-	43539155	6	6	---	---	intergenic	---

12	43574007	43574007	+	43574007	3	3	---	---	intergenic	---
12	43717670	43717676	-	43717676	45	127	XLOC_017777	-	predicted	---
12	43758507	43758516	-	43758508	34	104	XLOC_017778	-	predicted	---
12	43788023	43788023	-	43788023	3	3	---	---	intergenic	---
12	44248453	44248453	+	44248453	3	3	XLOC_016955	+	predicted	---
12	44257984	44257985	-	44257985	11	14	XLOC_017784	-	predicted	---
12	44259322	44259322	-	44259322	3	3	XLOC_017784	-	predicted	---
12	44332448	44332448	+	44332448	4	4	XLOC_016960	+	predicted	---
12	44344042	44344042	-	44344042	3	3	XLOC_017785	-	predicted	---
12	44345018	44345023	-	44345018	6	9	XLOC_017785	-	predicted	---
12	44345121	44345130	-	44345123	21	36	ENSSSCG00000017732	-	3'UTR	*ZNF207*
12	44345501	44345514	-	44345502	17	34	ENSSSCG00000017732	-	3'UTR	*ZNF207*
12	44362853	44362866	+	44362853	4	8	---	---	intergenic	---
12	44387596	44387596	-	44387596	3	3	ENSSSCG00000022850	-	3'UTR	---
12	44408953	44408953	+	44408953	3	3	---	---	intergenic	---
12	44431459	44431460	+	44431460	15	19	XLOC_016962	+	predicted	---
12	44433110	44433110	+	44433110	4	4	XLOC_016962	+	predicted	---
12	44466129	44466129	+	44466129	3	3	ENSSSCG00000017735	+	intron	*RHOT1*
12	44515342	44515342	+	44515342	3	3	XLOC_016963	+	predicted	---
12	44515570	44515570	+	44515570	7	7	XLOC_016963	+	predicted	---
12	44674427	44674427	+	44674427	3	3	XLOC_016965	+	predicted	---
12	44714866	44714866	+	44714866	3	3	---	---	intergenic	---
12	44764159	44764159	+	44764159	4	4	ENSSSCG00000017743	+	3'UTR	*CRLF3*
12	44765118	44765119	+	44765119	28	32	ENSSSCG00000017743	+	3'UTR	*CRLF3*
12	44767884	44767884	+	44767884	3	3	XLOC_016966	+	predicted	---
12	44877990	44877990	+	44877990	5	5	ENSSSCG00000017745	+	3'UTR	*UTP6*

12	44886770	44886779	+	44886778	33	55	ENSSSCG00000017746	+	3'UTR	*COPRS*
12	45147640	45147640	-	45147640	4	4	XLOC_017796	-	predicted	---
12	45188880	45188880	-	45188880	5	5	---	---	intergenic	---
12	45195241	45195244	-	45195241	7	16	ENSSSCG00000017747	-	3'UTR	*RAB11FIP4*
12	45199334	45199334	-	45199334	5	5	ENSSSCG00000017747	-	intron	*RAB11FIP4*
12	45245521	45245522	-	45245521	21	29	ENSSSCG00000017747	-	intron	*RAB11FIP4*
12	45305042	45305042	-	45305042	3	3	---	---	intergenic	---
12	45314405	45314405	-	45314405	3	3	ENSSSCG00000017748	-	3'UTR	*NF1*
12	45387439	45387439	+	45387439	10	10	XLOC_016970	+	predicted	---
12	45667736	45667740	-	45667738	50	93	XLOC_017803	-	predicted	---
12	45683973	45683973	-	45683973	3	3	ENSSSCG00000017752	-	intron	*WSB1*
12	45688860	45688860	+	45688860	4	4	XLOC_016976	+	predicted	---
12	45719227	45719227	-	45719227	4	4	---	---	intergenic	---
12	45982747	45982747	+	45982747	4	4	ENSSSCG00000017753	+	3'UTR	*KSR1*
12	46021344	46021355	+	46021344	5	10	ENSSSCG00000017754	+	3'UTR	---
12	46118528	46118528	-	46118528	4	4	XLOC_017808	-	predicted	---
12	46121972	46121972	-	46121972	4	4	XLOC_017808	-	predicted	---
12	46208469	46208469	-	46208469	4	4	---	---	intergenic	---
12	46522079	46522088	-	46522087	23	52	ENSSSCG00000023719	-	3'UTR	*IFT20*
							ENSSSCG00000027801	-	intron	---
12	46522398	46522398	-	46522398	4	4	ENSSSCG00000023719	-	3'UTR	*IFT20*
							ENSSSCG00000027801	-	intron	---
12	46538731	46538744	+	46538739	18	69	ENSSSCG00000024626	+	3'UTR	*TNFAIP1*
12	46538914	46538920	-	46538916	76	130	ENSSSCG00000028920	-	3'UTR	*POLDIP2*
							ENSSSCG00000027801	-	intron	---

12	46539491	46539506	-	46539503	34	120	ENSSSCG00000028920	-	3'UTR	*POLDIP2*
							ENSSSCG00000027801	-	intron	---
12	46554002	46554003	+	46554003	7	10	XLOC_016986	+	predicted	---
12	46637976	46637976	+	46637976	3	3	---	---	intergenic	---
12	46807788	46807789	-	46807788	48	51	ENSSSCG00000017759	-	3'UTR	*ALDOC*
12	46812355	46812361	-	46812356	30	50	ENSSSCG00000017758	-	3'UTR	*SPAG5*
12	46881805	46881818	-	46881807	6	16	XLOC_017825	-	predicted	---
12	46882040	46882051	-	46882045	52	103	ENSSSCG00000017772	-	3'UTR	*SDF2*
12	46902854	46902864	+	46902854	4	7	ENSSSCG00000017773	+	ORF	---
12	46925632	46925639	+	46925633	13	36	XLOC_016992	+	predicted	---
12	46926710	46926719	-	46926714	34	74	XLOC_017826	-	predicted	---
12	46927051	46927081	-	46927058	228	579	XLOC_017826	-	predicted	---
12	46927145	46927145	-	46927145	6	6	ENSSSCG00000017770	-	ORF	---
12	46927400	46927409	-	46927400	10	13	ENSSSCG00000017770	-	ORF	---
12	46927499	46927502	-	46927499	8	15	ENSSSCG00000017770	-	ORF	---
12	46936923	46936924	-	46936924	9	12	ENSSSCG00000017769	-	3'UTR	*RAB34*
12	46942899	46942899	+	46942899	5	5	ENSSSCG00000017768	+	ORF	---
12	46974130	46974130	-	46974130	3	3	---	---	intergenic	---
12	46995373	46995375	+	46995375	4	7	---	---	intergenic	---
12	46999983	46999984	+	46999984	4	7	ENSSSCG00000017775	+	3'UTR	*TRAF4*
12	47004512	47004512	-	47004512	5	5	ENSSSCG00000017774	-	3'UTR	*FAM222B*
12	47120232	47120232	+	47120232	5	5	ENSSSCG00000017779	+	intron	*PHF12*
12	47137412	47137412	+	47137412	4	4	ENSSSCG00000017779	+	3'UTR	*PHF12*
12	47137903	47137903	+	47137903	6	6	ENSSSCG00000017779	+	3'UTR	*PHF12*
12	47163742	47163744	+	47163742	22	43	XLOC_017000	+	predicted	---
12	47177310	47177310	+	47177310	9	9	XLOC_017001	+	predicted	---

12	47177312	47177324	-	47177324	5	12	ENSSSCG00000017776	-	3'UTR	*ERAL1*
12	47244234	47244239	+	47244235	20	55	XLOC_017005	+	predicted	---
12	47257288	47257291	-	47257288	5	8	XLOC_017834	-	predicted	---
12	47288083	47288084	-	47288084	8	11	XLOC_017835	-	predicted	---
12	47301843	47301843	-	47301843	4	4	---	---	intergenic	---
12	47460308	47460308	+	47460308	3	3	---	---	intergenic	---
12	47519545	47519545	+	47519545	4	4	---	---	intergenic	---
12	47694322	47694322	+	47694322	3	3	XLOC_017010	+	predicted	---
12	47695434	47695434	+	47695434	3	3	XLOC_017010	+	predicted	---
12	47696391	47696392	+	47696391	4	7	XLOC_017010	+	predicted	---
12	47696858	47696859	+	47696858	10	13	XLOC_017010	+	predicted	---
12	47702608	47702608	+	47702608	4	4	XLOC_017010	+	predicted	---
12	47719220	47719224	-	47719221	72	144	XLOC_017841	-	predicted	---
12	47719858	47719858	-	47719858	10	10	XLOC_017841	-	predicted	---
12	47864911	47864911	+	47864911	4	4	---	---	intergenic	---
12	47870425	47870425	+	47870425	3	3	ENSSSCG00000017791	+	ORF	*SSH2*
12	47871709	47871716	+	47871716	12	23	XLOC_017013	+	predicted	---
12	47899005	47899005	+	47899005	3	3	---	---	intergenic	---
12	47946686	47946687	+	47946686	7	13	---	---	intergenic	---
12	48172607	48172609	+	48172607	3	6	ENSSSCG00000017796	+	3'UTR	---
12	48173248	48173256	+	48173249	45	63	ENSSSCG00000017796	+	3'UTR	---
12	48228740	48228740	-	48228740	9	9	ENSSSCG00000017795	-	3'UTR	*NSRP1*
12	48229676	48229676	-	48229676	4	4	ENSSSCG00000017795	-	ORF	*NSRP1*
12	48234310	48234310	-	48234310	4	4	ENSSSCG00000017795	-	ORF	*NSRP1*
12	48287126	48287126	-	48287126	3	3	ENSSSCG00000017795	-	intron	*NSRP1*
12	48293566	48293566	-	48293566	3	3	XLOC_017851	-	predicted	---

12	48293816	48293825	-	48293816	3	6	XLOC_017851	-	predicted	---
12	48338400	48338400	-	48338400	3	3	ENSSSCG00000017794	-	intron	*EFCAB5*
12	48350594	48350595	-	48350595	8	12	ENSSSCG00000017794	-	intron	*EFCAB5*
12	48442287	48442288	-	48442288	6	9	ENSSSCG00000017794	-	ORF	*EFCAB5*
12	48457130	48457131	+	48457131	7	10	---	---	intergenic	---
12	48621510	48621514	+	48621514	6	11	ENSSSCG00000017799	+	3'UTR	*CPD*
12	48622821	48622824	+	48622821	29	38	ENSSSCG00000017799	+	3'UTR	*CPD*
12	48682965	48682972	+	48682971	6	12	ENSSSCG00000029262	+	3'UTR	*GOSR1*
12	48683120	48683132	+	48683126	36	78	ENSSSCG00000029262	+	3'UTR	*GOSR1*
12	48686620	48686620	+	48686620	4	4	XLOC_017020	+	predicted	---
12	48710768	48710768	-	48710768	5	5	XLOC_017855	-	predicted	---
12	48837698	48837698	+	48837698	14	14	---	---	intergenic	---
12	48885871	48885875	-	48885871	5	13	---	---	intergenic	---
12	49018018	49018018	-	49018018	3	3	ENSSSCG00000017804	-	intron	*ABR*
12	49040957	49040957	-	49040957	8	8	XLOC_017859	-	predicted	---
12	49084865	49084865	-	49084865	7	7	---	---	intergenic	---
12	49128157	49128172	-	49128157	7	16	---	---	intergenic	---
12	49128192	49128199	+	49128192	5	15	---	---	intergenic	---
12	49258641	49258641	+	49258641	4	4	ENSSSCG00000017807	+	intron	*GLOD4*
12	49260689	49260692	+	49260692	9	16	ENSSSCG00000017807	+	ORF	*GLOD4*
12	49262082	49262082	+	49262082	4	4	ENSSSCG00000017807	+	intron	*GLOD4*
12	49267508	49267508	+	49267508	5	5	ENSSSCG00000017807	+	intron	*GLOD4*
12	49267834	49267847	+	49267834	4	11	ENSSSCG00000017807	+	intron	*GLOD4*
12	49267890	49267898	+	49267895	15	27	ENSSSCG00000017807	+	3'UTR	*GLOD4*
12	49272528	49272542	-	49272535	401	801	XLOC_017861	-	predicted	---
12	49272710	49272711	-	49272710	3	6	ENSSSCG00000017808	-	ORF	---

12	49272872	49272872	-	49272872	3	3	ENSSSCG00000017808	-	ORF	---
12	49279422	49279422	+	49279422	8	8	---	---	intergenic	---
12	49279507	49279509	+	49279508	29	59	---	---	intergenic	---
12	49280820	49280820	-	49280820	5	5	ENSSSCG00000017810	-	3'UTR	*CT120*
12	49280962	49280969	-	49280968	5	13	ENSSSCG00000017810	-	3'UTR	*CT120*
12	49282026	49282026	-	49282026	5	5	ENSSSCG00000017810	-	3'UTR	*CT120*
12	49471791	49471791	-	49471791	4	4	---	---	intergenic	---
12	49503429	49503429	+	49503429	3	3	ENSSSCG00000017814	+	ORF	*C17orf97*
12	49506224	49506232	+	49506224	5	14	XLOC_017030	+	predicted	---
12	49602565	49602565	-	49602565	7	7	ENSSSCG00000030707	-	3'UTR	*SKIP*
12	49603817	49603828	-	49603828	19	30	ENSSSCG00000030707	-	3'UTR	*SKIP*
12	49626509	49626512	-	49626511	6	15	ENSSSCG00000025248	-	3'UTR	---
12	49626576	49626603	-	49626603	16	19	ENSSSCG00000025248	-	3'UTR	---
12	49640909	49640909	-	49640909	5	5	ENSSSCG00000025248	-	intron	---
12	49676762	49676765	-	49676762	3	6	XLOC_017869	-	predicted	---
12	49679967	49679967	-	49679967	3	3	XLOC_017869	-	predicted	---
12	49680479	49680503	-	49680503	11	21	XLOC_017869	-	predicted	---
12	49731410	49731411	-	49731410	3	6	XLOC_017869	-	predicted	---
12	49742025	49742027	-	49742025	8	20	ENSSSCG00000017816	-	3'UTR	*RILP*
12	49746646	49746657	-	49746655	30	70	XLOC_017870	-	predicted	---
12	49768907	49768907	-	49768907	5	5	ENSSSCG00000017817	-	ORF	*PRPF8*
12	49773384	49773384	-	49773384	7	7	ENSSSCG00000017817	-	ORF	*PRPF8*
12	49773452	49773452	-	49773452	3	3	ENSSSCG00000017817	-	ORF	*PRPF8*
12	49785601	49785602	-	49785601	10	14	ENSSSCG00000021821	-	3'UTR	*MYO1C*
12	49901710	49901716	+	49901711	10	21	ENSSSCG00000017822	+	3'UTR	---
12	49927357	49927357	-	49927357	5	5	XLOC_017874	-	predicted	---

12	50011245	50011256	+	50011247	25	45	ENSSSCG00000028078	+	3'UTR	---
12	50027253	50027253	-	50027253	4	4	XLOC_017878	-	predicted	---
12	50266692	50266692	+	50266692	3	3	XLOC_017039	+	predicted	---
12	50408473	50408473	-	50408473	4	4	---	---	intergenic	---
12	50480786	50480786	+	50480786	7	7	ENSSSCG00000017836	+	intron	*PAFAH1B1*
12	50494940	50494940	+	50494940	5	5	ENSSSCG00000017836	+	intron	*PAFAH1B1*
12	50501449	50501459	+	50501453	165	248	ENSSSCG00000017836	+	3'UTR	*PAFAH1B1*
12	50501496	50501502	+	50501502	6	11	ENSSSCG00000017836	+	3'UTR	*PAFAH1B1*
12	50501643	50501649	+	50501643	7	19	ENSSSCG00000017836	+	3'UTR	*PAFAH1B1*
12	50502596	50502596	+	50502596	5	5	XLOC_017041	+	predicted	---
12	50505185	50505185	+	50505185	7	7	XLOC_017041	+	predicted	---
12	50506580	50506580	-	50506580	3	3	XLOC_017883	-	predicted	---
12	50509471	50509481	-	50509473	25	50	XLOC_017884	-	predicted	---
12	50510567	50510568	-	50510568	5	8	XLOC_017884	-	predicted	---
12	51323020	51323020	-	51323020	5	5	---	---	intergenic	---
12	51572900	51572902	-	51572900	3	6	ENSSSCG00000017864	-	3'UTR	*SHPK*
12	51573672	51573672	-	51573672	5	5	ENSSSCG00000017864	-	3'UTR	*SHPK*
12	51611639	51611639	+	51611639	6	6	ENSSSCG00000017865	+	intron	*CTNS*
12	51620151	51620161	+	51620159	114	261	ENSSSCG00000017867	+	3'UTR	*EMC6*
							ENSSSCG00000017867	+	ORF	---
12	51638892	51638892	-	51638892	5	5	XLOC_017906	-	predicted	---
12	51642089	51642090	-	51642090	5	8	XLOC_017906	-	predicted	---
12	51720277	51720278	-	51720278	17	21	ENSSSCG00000017869	-	3'UTR	*ITGAE*
12	52024219	52024219	+	52024219	23	23	---	---	intergenic	---
12	52031810	52031817	-	52031814	14	30	ENSSSCG00000017874	-	3'UTR	*ATP2A3*
12	52120891	52120891	+	52120891	15	15	ENSSSCG00000017876	+	3'UTR	*CYB5D2*

12	52123891	52123891	-	52123891	5	5	XLOC_017918	-	predicted	---
12	52123971	52123971	-	52123971	4	4	XLOC_017918	-	predicted	---
12	52227109	52227109	-	52227109	5	5	---	---	intergenic	---
12	52277205	52277223	+	52277223	11	22	ENSSSCG00000017878	+	3'UTR	*UBE2G1*
12	52277267	52277267	+	52277267	6	6	ENSSSCG00000017878	+	3'UTR	*UBE2G1*
12	52277430	52277430	+	52277430	5	5	ENSSSCG00000017878	+	3'UTR	*UBE2G1*
12	52278043	52278043	+	52278043	6	6	ENSSSCG00000017878	+	3'UTR	*UBE2G1*
12	52279946	52279949	+	52279949	5	8	XLOC_017073	+	predicted	---
12	52410253	52410255	+	52410254	7	17	ENSSSCG00000017880	+	intron	*SPNS3*
12	52489616	52489627	-	52489621	16	31	XLOC_017922	-	predicted	---
12	52524661	52524661	-	52524661	4	4	XLOC_017924	-	predicted	---
12	52525540	52525549	-	52525541	35	82	XLOC_017924	-	predicted	---
12	52616492	52616492	-	52616492	4	4	---	---	intergenic	---
12	52635022	52635022	-	52635022	3	3	---	---	intergenic	---
12	52636190	52636197	-	52636197	22	80	XLOC_017927	-	predicted	---
12	52643903	52643906	-	52643903	51	63	ENSSSCG00000017887	-	3'UTR	*XAF1*
12	52709931	52709939	+	52709938	7	19	XLOC_017080	+	predicted	---
12	52732666	52732691	+	52732683	51	109	ENSSSCG00000027100	+	3'UTR	*MED31*
12	52733569	52733569	+	52733569	4	4	ENSSSCG00000027100	+	3'UTR	*MED31*
12	52733719	52733719	-	52733719	3	3	XLOC_017927	-	predicted	---
12	52733732	52733732	+	52733732	4	4	XLOC_017081	+	predicted	---
12	52746654	52746654	+	52746654	4	4	ENSSSCG00000017890	+	ORF	*KIAA0753*
12	53052726	53052726	-	53052726	4	4	XLOC_017932	-	predicted	---
12	53087228	53087228	-	53087228	7	7	---	---	intergenic	---
12	53487844	53487844	+	53487844	3	3	---	---	intergenic	---
12	53667893	53667893	+	53667893	3	3	ENSSSCG00000017895	+	intron	*RABEP1*

12	53724650	53724650	+	53724650	6	6	ENSSSCG00000017895	+	ORF	*RABEP1*
12	53738691	53738691	+	53738691	3	3	ENSSSCG00000017895	+	ORF	*RABEP1*
12	53805257	53805257	-	53805257	3	3	XLOC_017937	-	predicted	---
12	53805598	53805606	-	53805606	44	83	XLOC_017937	-	predicted	---
12	53905601	53905601	+	53905601	3	3	ENSSSCG00000022999	+	3'UTR	*ZNF232*
12	53968352	53968362	-	53968362	6	13	ENSSSCG00000017900	-	3'UTR	*KIF1C*
12	54000557	54000557	+	54000557	3	3	XLOC_017087	+	predicted	---
12	54017400	54017400	+	54017400	13	13	XLOC_017088	+	predicted	---
12	54022293	54022296	+	54022293	99	149	ENSSSCG00000017903	+	3'UTR	*SPAG7*
12	54023317	54023324	-	54023320	307	423	ENSSSCG00000017904	-	3'UTR	*ENO3*
12	54034131	54034131	+	54034131	5	5	ENSSSCG00000017905	+	3'UTR	*PFN1*
12	54034273	54034281	+	54034281	61	110	ENSSSCG00000017905	+	3'UTR	*PFN1*
12	54034740	54034747	-	54034744	40	72	ENSSSCG00000017906	-	3'UTR	*RNF167*
12	54042604	54042610	+	54042604	19	31	ENSSSCG00000017907	+	3'UTR	*OMC*
12	54059540	54059540	-	54059540	3	3	XLOC_017944	-	predicted	---
12	54085256	54085264	-	54085257	14	26	XLOC_017946	-	predicted	---
12	54258511	54258511	-	54258511	7	7	XLOC_017949	-	predicted	---
12	54269463	54269485	+	54269477	50	127	ENSSSCG00000017920	+	3'UTR	*CXCL16*
							ENSSSCG00000017920	+	ORF	---
12	54269575	54269575	+	54269575	3	3	ENSSSCG00000017920	+	3'UTR	*CXCL16*
							ENSSSCG00000017920	+	ORF	---
12	54269868	54269868	-	54269868	11	11	ENSSSCG00000017919	-	3'UTR	*MED11*
12	54270685	54270685	+	54270685	4	4	XLOC_017093	+	predicted	---
12	54280939	54280940	-	54280939	11	14	XLOC_017950	-	predicted	---
12	54317056	54317064	-	54317060	155	474	ENSSSCG00000017913	-	3'UTR	*PSMB6*
12	54330888	54330891	+	54330888	3	6	ENSSSCG00000017915	+	3'UTR	*VMO1*

12	54331636	54331636	-	54331636	3	3	XLOC_017954	-	predicted	---
12	54331882	54331882	-	54331882	3	3	XLOC_017954	-	predicted	---
12	54332010	54332011	-	54332010	5	8	XLOC_017954	-	predicted	---
12	54447905	54447911	-	54447905	15	19	ENSSSCG00000017924	-	3'UTR	---
12	54614664	54614665	-	54614664	19	22	---	---	intergenic	---
12	54687403	54687403	-	54687403	3	3	---	---	intergenic	---
12	54699428	54699428	-	54699428	5	5	ENSSSCG00000017926	-	3'UTR	---
12	54704450	54704450	+	54704450	4	4	XLOC_017102	+	predicted	---
12	54717493	54717497	-	54717496	32	48	XLOC_017961	-	predicted	---
12	54720561	54720568	-	54720562	68	163	ENSSSCG00000017929	-	3'UTR	*RNASEK*
12	54726393	54726393	-	54726393	3	3	ENSSSCG00000017930	-	intron	*ALOX12*
12	54768746	54768747	-	54768746	13	20	XLOC_017964	-	predicted	---
12	54769352	54769369	-	54769360	119	374	ENSSSCG00000017932	-	3'UTR	*ASGR2*
12	54916269	54916271	+	54916269	4	11	XLOC_017115	+	predicted	---
12	54921997	54921998	+	54921997	7	10	XLOC_017115	+	predicted	---
12	54937795	54937795	-	54937795	4	4	ENSSSCG00000029391	-	3'UTR	*TMEM256-PLSCR3*
12	54951702	54951724	-	54951724	38	46	ENSSSCG00000025782	-	3'UTR	---
12	54973318	54973327	-	54973320	41	72	XLOC_017974	-	predicted	---
12	55006870	55006870	-	55006870	4	4	ENSSSCG00000024862	-	intron	*FGF11*
12	55017020	55017031	-	55017021	15	69	XLOC_017976	-	predicted	---
12	55021620	55021623	-	55021620	23	43	XLOC_017976	-	predicted	---
12	55025427	55025429	-	55025428	16	28	---	---	intergenic	---
12	55034712	55034713	+	55034712	19	23	ENSSSCG00000027897	+	intron	---
12	55198071	55198071	-	55198071	3	3	ENSSSCG00000017948	-	3'UTR	*EFNB3*
12	55205964	55205964	-	55205964	4	4	ENSSSCG00000017949	-	3'UTR	*WRAP53*

12	55241663	55241664	-	55241664	4	7	XLOC_017982	-	predicted	---
12	55262601	55262601	+	55262601	8	8	ENSSSCG00000017952	+	3'UTR	*SAT2*
12	55288304	55288305	+	55288304	15	25	ENSSSCG00000017953	+	3'UTR	*FXR2*
12	55291373	55291385	-	55291375	29	66	ENSSSCG00000017955	-	3'UTR	---
12	55296985	55296988	-	55296987	73	87	ENSSSCG00000017956	-	3'UTR	*CD68*
12	55299996	55300003	-	55299997	16	45	XLOC_017983	-	predicted	---
12	55300344	55300363	-	55300345	28	46	ENSSSCG00000017957	-	3'UTR	*EIF4A1*
12	55302008	55302008	-	55302008	3	3	ENSSSCG00000017957	-	intron	*EIF4A1*
12	55306674	55306674	-	55306674	4	4	---	---	intergenic	---
12	55306973	55306973	-	55306973	4	4	XLOC_017988	-	predicted	---
12	55307052	55307052	-	55307052	6	6	XLOC_017988	-	predicted	---
12	55452261	55452262	+	55452261	22	29	ENSSSCG00000017963	+	3'UTR	---
12	55483255	55483255	+	55483255	6	6	XLOC_017138	+	predicted	---
12	55529015	55529016	+	55529016	5	8	XLOC_017138	+	predicted	---
12	55529558	55529559	+	55529558	7	11	XLOC_017138	+	predicted	---
12	55545613	55545619	-	55545615	88	199	ENSSSCG00000029679	-	3'UTR	*TRAPPC1*
12	55545751	55545751	-	55545751	4	4	ENSSSCG00000029679	-	3'UTR	*TRAPPC1*
12	55567769	55567769	+	55567769	4	4	ENSSSCG00000017968	+	3'UTR	*CNTROB*
12	55567829	55567829	+	55567829	4	4	ENSSSCG00000017968	+	3'UTR	*CNTROB*
12	55769664	55769664	-	55769664	3	3	ENSSSCG00000017980	-	intron	*ALOX12B*
12	55853619	55853619	-	55853619	12	12	XLOC_018002	-	predicted	---
12	55869746	55869746	-	55869746	6	6	ENSSSCG00000027528	-	3'UTR	*TMEM107*
12	55869833	55869833	-	55869833	11	11	ENSSSCG00000027528	-	3'UTR	*TMEM107*
							ENSSSCG00000023729	-	ORF	*U8*
12	55870282	55870284	-	55870282	4	7	ENSSSCG00000027528	-	3'UTR	*TMEM107*
12	55879568	55879570	-	55879569	20	34	XLOC_018004	-	predicted	---

12	55882046	55882063	+	55882052	4	21	XLOC_017146	+	predicted	---
12	55886061	55886068	-	55886067	65	108	ENSSSCG00000028924	-	3'UTR	*AURKB*
12	55903626	55903626	-	55903626	12	12	XLOC_018006	-	predicted	---
12	55962189	55962191	-	55962191	5	8	ENSSSCG00000022395	-	3'UTR	*SLC25A35*
12	55962209	55962218	+	55962209	39	68	ENSSSCG00000030431	+	3'UTR	*RANGRF*
12	56001068	56001073	+	56001070	29	99	ENSSSCG00000028166	+	3'UTR	*ODF4*
12	56024032	56024047	-	56024047	507	656	ENSSSCG00000028850	-	3'UTR	*RPL26*
12	56024097	56024135	-	56024135	4	7	ENSSSCG00000028850	-	ORF	*RPL26*
12	56026605	56026605	-	56026605	3	3	ENSSSCG00000028850	-	intron	*RPL26*
12	56390572	56390572	-	56390572	3	3	---	---	intergenic	---
12	56419617	56419617	+	56419617	5	5	---	---	intergenic	---
12	56569409	56569409	-	56569409	3	3	---	---	intergenic	---
12	56619323	56619358	-	56619325	44	93	ENSSSCG00000017988	-	3'UTR	---
12	56661529	56661529	-	56661529	3	3	XLOC_018018	-	predicted	---
12	56726692	56726692	-	56726692	3	3	---	---	intergenic	---
12	56731154	56731154	-	56731154	16	16	XLOC_018019	-	predicted	---
12	56975436	56975438	-	56975436	8	19	XLOC_018021	-	predicted	---
12	57058043	57058043	-	57058043	3	3	---	---	intergenic	---
12	57133241	57133241	-	57133241	4	4	---	---	intergenic	---
12	57339664	57339670	+	57339666	36	59	ENSSSCG00000017994	+	3'UTR	*CFAP52*
12	57405731	57405747	-	57405744	98	247	XLOC_018023	-	predicted	---
12	57493500	57493500	+	57493500	3	3	XLOC_017161	+	predicted	---
12	57510520	57510520	-	57510520	3	3	XLOC_018025	-	predicted	---
12	57514375	57514375	-	57514375	4	4	XLOC_018025	-	predicted	---
12	57573209	57573209	+	57573209	3	3	---	---	intergenic	---
12	57589650	57589653	+	57589651	15	40	---	---	intergenic	---

12	57791234	57791234	+	57791234	3	3	---	---	intergenic	---
12	57797874	57797875	+	57797874	4	7	XLOC_017167	+	predicted	---
12	57805631	57805632	+	57805631	5	8	XLOC_017167	+	predicted	---
12	57862748	57862749	+	57862748	5	10	---	---	intergenic	---
12	57883557	57883557	-	57883557	4	4	ENSSSCG00000024416	-	intron	*MYH13*
12	57910293	57910293	-	57910293	4	4	ENSSSCG00000024416	-	intron	*MYH13*
12	57970988	57970989	+	57970988	4	7	ENSSSCG00000018005	+	ORF	*MYH1*
12	57974313	57974315	+	57974315	10	14	ENSSSCG00000018005	+	ORF	*MYH1*
12	57974384	57974386	+	57974385	6	15	ENSSSCG00000018005	+	intron	*MYH1*
12	57979844	57979844	+	57979844	3	3	ENSSSCG00000018005	+	ORF	*MYH1*
12	57982407	57982407	+	57982407	6	6	ENSSSCG00000018005	+	ORF	*MYH1*
12	57984858	57984858	+	57984858	55	55	XLOC_017169	+	predicted	---
12	58034895	58034896	+	58034896	8	11	---	---	intergenic	---
12	58039028	58039035	-	58039031	7	15	ENSSSCG00000018008	-	3'UTR	*SCO1*
12	58583041	58583041	-	58583041	7	7	ENSSSCG00000024467	-	intron	---
12	58897124	58897124	-	58897124	4	4	XLOC_018036	-	predicted	---
12	58929155	58929155	+	58929155	3	3	---	---	intergenic	---
12	58939776	58939776	+	58939776	3	3	XLOC_017177	+	predicted	---
12	59397304	59397304	+	59397304	4	4	ENSSSCG00000018016	+	intron	*MAP2K4*
12	59409492	59409492	+	59409492	5	5	ENSSSCG00000018016	+	3'UTR	*MAP2K4*
12	59558205	59558205	+	59558205	3	3	---	---	intergenic	---
12	59745620	59745629	-	59745620	8	18	XLOC_018043	-	predicted	---
12	59753772	59753772	-	59753772	7	7	---	---	intergenic	---
12	59763041	59763041	+	59763041	5	5	---	---	intergenic	---
12	60230284	60230284	-	60230284	5	5	---	---	intergenic	---
12	60394518	60394518	+	60394518	5	5	---	---	intergenic	---

12	60549588	60549591	-	60549588	5	9	ENSSSCG00000030589	-	3'UTR	*HS3ST3A1*
12	60697751	60697751	-	60697751	4	4	---	---	intergenic	---
12	60856488	60856488	+	60856488	4	4	---	---	intergenic	---
12	60937216	60937216	-	60937216	3	3	ENSSSCG00000025046	-	ORF	---
12	61116874	61116874	+	61116874	3	3	ENSSSCG00000030136	+	intron	*HS3ST3B1*
12	61301824	61301829	-	61301824	3	6	---	---	intergenic	---
12	61403518	61403522	+	61403518	6	14	---	---	intergenic	---
12	61751931	61751935	+	61751931	11	14	XLOC_017198	+	predicted	---
12	61752103	61752104	+	61752103	9	12	XLOC_017198	+	predicted	---
12	61777527	61777527	+	61777527	5	5	XLOC_017199	+	predicted	---
12	61893407	61893410	+	61893410	4	7	ENSSSCG00000018026	+	ORF	---
12	61893501	61893524	+	61893503	203	335	XLOC_017202	+	predicted	---
12	61893815	61893821	+	61893815	7	11	XLOC_017202	+	predicted	---
12	61907658	61907660	+	61907660	16	22	ENSSSCG00000018027	+	3'UTR	---
12	61907733	61907741	+	61907735	8	18	ENSSSCG00000018027	+	3'UTR	---
12	61941275	61941275	-	61941275	5	5	XLOC_018072	-	predicted	---
12	61971016	61971026	+	61971024	21	61	XLOC_017203	+	predicted	---
12	61973678	61973679	+	61973678	6	9	---	---	intergenic	---
12	62012607	62012607	-	62012607	5	5	XLOC_018073	-	predicted	---
12	62019844	62019844	+	62019844	5	5	ENSSSCG00000018028	+	intron	*TEKT3*
12	62127338	62127339	+	62127338	5	9	ENSSSCG00000025254	+	intron	---
12	62153840	62153840	-	62153840	3	3	XLOC_018076	-	predicted	---
12	62200254	62200254	+	62200254	4	4	---	---	intergenic	---
12	62259222	62259222	+	62259222	3	3	ENSSSCG00000021252	+	3'UTR	*LRRC75A*
12	62259235	62259235	-	62259235	3	3	XLOC_018078	-	predicted	---
12	62260461	62260461	-	62260461	4	4	ENSSSCG00000018456	-	ORF	*SNORD49*

12	62262599	62262605	-	62262605	10	13	ENSSSCG00000018032	-	3'UTR	*TRPV2*
12	62297864	62297885	-	62297871	4	10	XLOC_018079	-	predicted	---
12	62297978	62297991	-	62297984	1134	2411	ENSSSCG00000018033	-	3'UTR	*UBB*
12	62298050	62298079	-	62298061	20	27	ENSSSCG00000018033	-	3'UTR	*UBB*
12	62298403	62298403	-	62298403	3	3	ENSSSCG00000018033	-	ORF	*UBB*
12	62307868	62307872	+	62307868	6	16	XLOC_017211	+	predicted	---
12	62315205	62315205	+	62315205	3	3	---	---	intergenic	---
12	62334757	62334769	+	62334763	76	123	XLOC_017211	+	predicted	---
12	62458833	62458833	-	62458833	3	3	ENSSSCG00000018039	-	ORF	*NCOR1*
12	62502489	62502493	-	62502490	16	25	ENSSSCG00000023747	-	3'UTR	---
							ENSSSCG00000018039	-	intron	*NCOR1*
12	62639846	62639846	-	62639846	3	3	ENSSSCG00000018044	-	intron	*ALDH3A1*
12	62666984	62666992	-	62666989	19	54	ENSSSCG00000018045	-	3'UTR	*ULK2*
12	62667662	62667662	-	62667662	5	5	ENSSSCG00000018045	-	3'UTR	*ULK2*
12	62895038	62895038	+	62895038	7	7	ENSSSCG00000018049	+	intron	---
12	62915940	62915940	+	62915940	7	7	ENSSSCG00000018049	+	intron	---
12	62923324	62923326	+	62923324	16	26	XLOC_017220	+	predicted	---
12	62923660	62923660	+	62923660	4	4	XLOC_017220	+	predicted	---
12	62928022	62928023	-	62928023	8	11	---	---	intergenic	---
12	62944511	62944511	-	62944511	4	4	ENSSSCG00000018047	-	3'UTR	*FAM83G*
12	62974577	62974577	-	62974577	3	3	XLOC_018092	-	predicted	---
12	62974580	62974580	+	62974580	5	5	XLOC_017221	+	predicted	---
12	63034588	63034609	-	63034606	223	489	ENSSSCG00000018051	-	3'UTR	*COPS3*
12	63079010	63079019	+	63079019	6	9	ENSSSCG00000018052	+	3'UTR	*NT5M*
12	63133346	63133352	+	63133347	10	47	ENSSSCG00000018053	+	3'UTR	*MED9*
12	63134408	63134408	+	63134408	3	3	XLOC_017225	+	predicted	---

12	63139409	63139418	-	63139412	82	105	ENSSSCG00000018055	-	3'UTR	*PEMT*
12	63177644	63177644	-	63177644	16	16	---	---	intergenic	---
12	63192009	63192010	-	63192010	4	7	XLOC_018099	-	predicted	---
12	63303344	63303344	+	63303344	4	4	---	---	intergenic	---
13	83674	83674	-	83674	15	15	---	---	intergenic	---
13	141739	141739	+	141739	3	3	---	---	intergenic	---
13	159877	159877	+	159877	6	6	---	---	intergenic	---
13	802790	802790	+	802790	4	4	ENSSSCG00000011179	+	3'UTR	*MRPL3*
13	803023	803024	+	803023	15	20	ENSSSCG00000011179	+	3'UTR	*MRPL3*
13	988708	988708	-	988708	4	4	---	---	intergenic	---
13	1118621	1118621	-	1118621	7	7	ENSSSCG00000011183	-	3'UTR	*ATP2C1*
13	1118901	1118912	-	1118911	10	22	ENSSSCG00000011183	-	3'UTR	*ATP2C1*
13	1119128	1119129	-	1119128	5	10	ENSSSCG00000011183	-	3'UTR	*ATP2C1*
13	1224234	1224234	-	1224234	3	3	ENSSSCG00000011183	-	intron	*ATP2C1*
13	1238439	1238439	+	1238439	6	6	---	---	intergenic	---
13	1598190	1598190	+	1598190	3	3	---	---	intergenic	---
13	1775265	1775274	-	1775265	7	14	XLOC_021383	-	predicted	---
13	1928886	1928886	+	1928886	3	3	---	---	intergenic	---
13	2093505	2093505	+	2093505	3	3	XLOC_020062	+	predicted	---
13	2300315	2300315	+	2300315	3	3	---	---	intergenic	---
13	2432344	2432361	-	2432347	13	22	XLOC_021386	-	predicted	---
13	2565967	2565971	-	2565969	4	11	ENSSSCG00000011190	-	3'UTR	*METTL6*
13	2597134	2597140	+	2597135	11	35	ENSSSCG00000026622	+	3'UTR	*EAF1*
13	2602111	2602119	+	2602119	5	16	XLOC_020066	+	predicted	---
13	2685547	2685547	-	2685547	3	3	---	---	intergenic	---
13	2714440	2714447	-	2714440	6	9	XLOC_021391	-	predicted	---

13	3073563	3073563	-	3073563	3	3	ENSSSCG00000011194	-	3'UTR	*ANKRD28*
13	3299035	3299035	+	3299035	3	3	---	---	intergenic	---
13	3463565	3463571	-	3463568	354	672	---	---	intergenic	---
13	3463590	3463600	+	3463595	6	34	---	---	intergenic	---
13	3489219	3489219	+	3489219	3	3	---	---	intergenic	---
13	3581949	3581949	-	3581949	3	3	XLOC_021397	-	predicted	---
13	3582110	3582110	-	3582110	3	3	XLOC_021397	-	predicted	---
13	3582586	3582586	-	3582586	10	10	XLOC_021397	-	predicted	---
13	3584000	3584007	-	3584007	34	45	ENSSSCG00000011196	-	3'UTR	*DPH3*
13	3626472	3626477	+	3626477	8	12	ENSSSCG00000011197	+	intron	*OXNAD1*
13	3627106	3627108	+	3627106	10	16	ENSSSCG00000011197	+	3'UTR	*OXNAD1*
13	3644443	3644443	-	3644443	9	9	XLOC_021398	-	predicted	---
13	3919304	3919305	-	3919305	112	127	---	---	intergenic	---
13	4033222	4033222	-	4033222	9	9	---	---	intergenic	---
13	4138795	4138796	-	4138796	4	7	---	---	intergenic	---
13	4191155	4191158	-	4191155	4	7	---	---	intergenic	---
13	4252420	4252420	+	4252420	8	8	---	---	intergenic	---
13	4509973	4509983	+	4509973	4	8	XLOC_020082	+	predicted	---
13	4510126	4510126	-	4510126	7	7	XLOC_021401	-	predicted	---
13	4510447	4510456	+	4510447	6	9	XLOC_020082	+	predicted	---
13	4598702	4598702	-	4598702	3	3	ENSSSCG00000011199	-	intron	*TBC1D5*
13	5031361	5031362	+	5031362	28	33	---	---	intergenic	---
13	5132296	5132296	+	5132296	11	11	---	---	intergenic	---
13	5534517	5534517	-	5534517	3	3	---	---	intergenic	---
13	5534554	5534554	+	5534554	4	4	---	---	intergenic	---
13	5789475	5789475	+	5789475	6	6	---	---	intergenic	---

13	5990439	5990439	-	5990439	3	3	---	---	intergenic	---
13	6411823	6411823	+	6411823	3	3	XLOC_020087	+	predicted	---
13	6597874	6597874	-	6597874	3	3	---	---	intergenic	---
13	6909312	6909312	+	6909312	3	3	---	---	intergenic	---
13	7101259	7101259	+	7101259	3	3	---	---	intergenic	---
13	7798290	7798295	+	7798295	5	8	XLOC_020092	+	predicted	---
13	7919286	7919286	-	7919286	6	6	XLOC_021411	-	predicted	---
13	7936139	7936139	-	7936139	4	4	ENSSSCG00000027327	-	intron	*PP2D1*
13	7936317	7936317	-	7936317	3	3	---	---	intergenic	---
13	7936675	7936675	-	7936675	3	3	---	---	intergenic	---
13	7953251	7953258	-	7953257	21	49	---	---	intergenic	---
13	8016175	8016175	+	8016175	6	6	ENSSSCG00000024621	+	intron	*KAT2B*
13	8051508	8051508	+	8051508	13	13	XLOC_020094	+	predicted	---
13	8146309	8146312	+	8146309	5	12	---	---	intergenic	---
13	8789671	8789672	-	8789672	30	40	---	---	intergenic	---
13	9409155	9409155	+	9409155	3	3	---	---	intergenic	---
13	9508223	9508223	-	9508223	3	3	ENSSSCG00000011208	-	intron	*ZNF385D*
13	9739057	9739057	-	9739057	3	3	ENSSSCG00000011208	-	intron	*ZNF385D*
13	10226568	10226568	+	10226568	4	4	---	---	intergenic	---
13	10705957	10705957	+	10705957	5	5	---	---	intergenic	---
13	10820942	10820942	+	10820942	3	3	---	---	intergenic	---
13	11009575	11009575	-	11009575	3	3	---	---	intergenic	---
13	11098946	11098946	+	11098946	3	3	---	---	intergenic	---
13	11215238	11215238	-	11215238	3	3	XLOC_021422	-	predicted	---
13	11413556	11413556	-	11413556	3	3	---	---	intergenic	---
13	11420764	11420764	-	11420764	13	13	---	---	intergenic	---

13	11779375	11779377	+	11779375	5	10	ENSSSCG00000011209	+	3'UTR	*UBE2E2*
13	11834290	11834295	+	11834295	4	10	---	---	intergenic	---
13	11907643	11907643	+	11907643	3	3	---	---	intergenic	---
13	12131838	12131852	+	12131838	9	19	ENSSSCG00000026488	+	3'UTR	*UBE2E1*
13	12134531	12134533	-	12134533	14	17	ENSSSCG00000024296	-	3'UTR	*NKIRAS1*
13	12166911	12166923	+	12166912	66	131	XLOC_020109	+	predicted	---
13	12168310	12168310	+	12168310	3	3	XLOC_020109	+	predicted	---
13	12182843	12182843	-	12182843	3	3	---	---	intergenic	---
13	12201854	12201854	-	12201854	5	5	---	---	intergenic	---
13	12219577	12219577	+	12219577	3	3	ENSSSCG00000027017	+	3'UTR	*NR1D2*
13	12222711	12222718	+	12222711	11	22	ENSSSCG00000027017	+	3'UTR	*NR1D2*
13	12238238	12238238	+	12238238	3	3	---	---	intergenic	---
13	12257345	12257355	+	12257345	59	91	ENSSSCG00000028079	+	3'UTR	*RPL15*
13	12339655	12339656	-	12339656	8	12	---	---	intergenic	---
13	12383642	12383642	-	12383642	15	15	---	---	intergenic	---
13	12400739	12400739	-	12400739	3	3	ENSSSCG00000011211	-	3'UTR	*THRB*
13	12514784	12514784	+	12514784	5	5	---	---	intergenic	---
13	12835308	12835308	-	12835308	3	3	XLOC_021433	-	predicted	---
13	12905772	12905772	-	12905772	4	4	---	---	intergenic	---
13	13849634	13849634	-	13849634	4	4	---	---	intergenic	---
13	14004072	14004083	+	14004081	87	332	---	---	intergenic	---
13	14119707	14119722	-	14119707	13	34	XLOC_021436	-	predicted	---
13	14138849	14138849	-	14138849	3	3	ENSSSCG00000011213	-	intron	*TOP2B*
13	14229515	14229515	-	14229515	3	3	ENSSSCG00000011214	-	3'UTR	*NGLY1*
13	14229823	14229866	-	14229824	8	27	ENSSSCG00000011214	-	3'UTR	*NGLY1*
13	14296304	14296304	+	14296304	5	5	ENSSSCG00000011215	+	3'UTR	*OXSM*

13	14326700	14326700	-	14326700	3	3	---	---	intergenic	---
13	14547869	14547869	-	14547869	4	4	---	---	intergenic	---
13	14637033	14637033	-	14637033	6	6	---	---	intergenic	---
13	15135262	15135262	-	15135262	3	3	---	---	intergenic	---
13	15193642	15193642	+	15193642	5	5	---	---	intergenic	---
13	15464397	15464397	-	15464397	4	4	ENSSSCG00000011217	-	intron	*NEK10*
13	15479012	15479016	-	15479016	6	10	XLOC_021442	-	predicted	---
13	15670165	15670165	-	15670165	3	3	---	---	intergenic	---
13	15725389	15725392	+	15725390	5	11	---	---	intergenic	---
13	15934260	15934260	-	15934260	4	4	---	---	intergenic	---
13	16075764	16075774	+	16075764	9	14	---	---	intergenic	---
13	16214098	16214100	-	16214098	7	10	XLOC_021447	-	predicted	---
13	16338003	16338003	+	16338003	6	6	---	---	intergenic	---
13	16458278	16458287	-	16458287	18	31	ENSSSCG00000028258	-	3'UTR	*AZI2*
13	16458592	16458592	-	16458592	6	6	ENSSSCG00000028258	-	ORF	*AZI2*
13	16472323	16472323	-	16472323	3	3	ENSSSCG00000028258	-	ORF	*AZI2*
13	16474547	16474553	-	16474553	4	7	ENSSSCG00000028258	-	ORF	*AZI2*
13	16533150	16533154	-	16533154	5	8	ENSSSCG00000028258	-	intron	*AZI2*
13	16533542	16533543	-	16533542	4	7	ENSSSCG00000028258	-	intron	*AZI2*
13	16640442	16640442	+	16640442	3	3	---	---	intergenic	---
13	16645179	16645181	+	16645179	22	25	---	---	intergenic	---
13	16670918	16670918	+	16670918	3	3	XLOC_020134	+	predicted	---
13	16671415	16671417	+	16671415	4	8	XLOC_020134	+	predicted	---
13	17410709	17410709	+	17410709	3	3	---	---	intergenic	---
13	17527719	17527719	-	17527719	3	3	---	---	intergenic	---
13	17591281	17591291	+	17591281	14	22	---	---	intergenic	---

13	18323240	18323240	+	18323240	3	3	XLOC_020139	+	predicted	---
13	18323491	18323492	+	18323491	4	7	XLOC_020139	+	predicted	---
13	18805624	18805624	-	18805624	3	3	---	---	intergenic	---
13	18869960	18869960	+	18869960	6	6	---	---	intergenic	---
13	18871539	18871545	+	18871544	28	39	---	---	intergenic	---
13	19550910	19550911	-	19550910	3	6	---	---	intergenic	---
13	19563987	19563987	+	19563987	3	3	---	---	intergenic	---
13	19593556	19593557	-	19593556	4	8	---	---	intergenic	---
13	19774234	19774235	+	19774235	21	24	ENSSSCG00000011229	+	intron	*STT3B*
13	19777504	19777510	+	19777505	39	111	ENSSSCG00000011229	+	3'UTR	*STT3B*
13	19778790	19778791	+	19778791	8	12	ENSSSCG00000011229	+	3'UTR	*STT3B*
13	19778898	19778903	+	19778902	46	90	ENSSSCG00000011229	+	3'UTR	*STT3B*
13	19795950	19795967	-	19795964	30	143	ENSSSCG00000011230	-	3'UTR	*OSBPL10*
13	19815320	19815320	-	19815320	3	3	ENSSSCG00000011230	-	intron	*OSBPL10*
13	20117137	20117137	-	20117137	4	4	ENSSSCG00000011230	-	intron	*OSBPL10*
13	20174959	20174959	-	20174959	5	5	---	---	intergenic	---
13	20254563	20254564	-	20254564	4	7	---	---	intergenic	---
13	20262461	20262465	-	20262462	17	46	ENSSSCG00000021223	-	3'UTR	*CMTM6*
13	20309383	20309393	-	20309383	4	7	ENSSSCG00000011233	-	3'UTR	*DYNC1LI1*
13	20310179	20310181	-	20310179	6	10	ENSSSCG00000011233	-	3'UTR	*DYNC1LI1*
13	20352692	20352695	+	20352692	3	6	---	---	intergenic	---
13	20399739	20399739	-	20399739	6	6	ENSSSCG00000011234	-	3'UTR	*CMTM7*
13	20616343	20616350	-	20616344	35	78	ENSSSCG00000021977	-	3'UTR	*GPD1L*
13	20617343	20617343	-	20617343	6	6	ENSSSCG00000021977	-	3'UTR	*GPD1L*
13	20618840	20618840	-	20618840	11	11	ENSSSCG00000021977	-	3'UTR	*GPD1L*
13	20655818	20655818	-	20655818	4	4	ENSSSCG00000021977	-	ORF	*GPD1L*

13	20831156	20831156	+	20831156	3	3	XLOC_020152	+	predicted	---
13	20831365	20831366	+	20831365	10	18	XLOC_020152	+	predicted	---
13	21240620	21240623	+	21240623	21	27	XLOC_020156	+	predicted	---
13	21243504	21243505	+	21243505	6	10	XLOC_020156	+	predicted	---
13	21506210	21506210	-	21506210	3	3	---	---	intergenic	---
13	21531432	21531432	+	21531432	5	5	---	---	intergenic	---
13	21807904	21807904	+	21807904	3	3	---	---	intergenic	---
13	22129576	22129576	+	22129576	8	8	---	---	intergenic	---
13	22510306	22510311	-	22510309	5	11	---	---	intergenic	---
13	23044045	23044045	+	23044045	8	8	XLOC_020162	+	predicted	---
13	23425189	23425189	+	23425189	3	3	XLOC_020164	+	predicted	---
13	23519010	23519010	+	23519010	5	5	---	---	intergenic	---
13	23691633	23691633	-	23691633	3	3	XLOC_021477	-	predicted	---
13	23691685	23691686	-	23691685	5	8	XLOC_021477	-	predicted	---
13	23692360	23692368	+	23692362	31	54	ENSSSCG00000024222	+	3'UTR	*MLH1*
13	23693690	23693695	-	23693692	19	40	ENSSSCG00000027738	-	3'UTR	*LRRFIP2*
13	23693987	23693987	-	23693987	4	4	ENSSSCG00000027738	-	3'UTR	*LRRFIP2*
13	23694330	23694361	-	23694354	47	128	ENSSSCG00000027738	-	3'UTR	*LRRFIP2*
13	23695347	23695347	-	23695347	4	4	---	---	intergenic	---
13	23771297	23771297	+	23771297	3	3	---	---	intergenic	---
13	23787315	23787321	+	23787315	3	6	XLOC_020168	+	predicted	---
13	23843179	23843187	+	23843179	4	11	ENSSSCG00000011239	+	3'UTR	---
13	23883767	23883767	+	23883767	16	16	---	---	intergenic	---
13	24091987	24091987	+	24091987	6	6	---	---	intergenic	---
13	24097831	24097831	+	24097831	4	4	---	---	intergenic	---
13	24100498	24100498	+	24100498	3	3	---	---	intergenic	---

13	24116978	24116978	+	24116978	11	11	ENSSSCG00000011243	+	intron	*GOLGA4*
13	24143171	24143173	+	24143173	5	12	XLOC_020177	+	predicted	---
13	24633194	24633194	-	24633194	4	4	---	---	intergenic	---
13	24724449	24724457	+	24724451	18	47	ENSSSCG00000030516	+	3'UTR	---
13	24725218	24725218	+	24725218	3	3	XLOC_020178	+	predicted	---
13	24847844	24847853	+	24847844	11	23	ENSSSCG00000027550	+	3'UTR	*PLCD1*
13	24871071	24871073	-	24871072	11	18	ENSSSCG00000028740	-	3'UTR	*CTDSPL*
13	24875219	24875219	-	24875219	4	4	ENSSSCG00000028740	-	intron	*CTDSPL*
13	25168694	25168696	+	25168696	7	12	XLOC_020185	+	predicted	---
13	25168695	25168707	-	25168695	5	17	XLOC_021490	-	predicted	---
13	25168858	25168896	-	25168890	283	871	ENSSSCG00000011250	-	3'UTR	*ACAA*
13	25169034	25169039	-	25169034	45	78	ENSSSCG00000011250	-	3'UTR	*ACAA*
13	25185353	25185356	+	25185354	14	37	XLOC_020186	+	predicted	---
13	25187638	25187638	+	25187638	6	6	---	---	intergenic	---
13	25302486	25302502	-	25302497	13	54	XLOC_021491	-	predicted	---
13	25363456	25363462	-	25363456	6	13	XLOC_021492	-	predicted	---
13	25487913	25487913	+	25487913	3	3	ENSSSCG00000011258	+	3'UTR	*EXOG*
13	26030431	26030431	+	26030431	4	4	ENSSSCG00000011261	+	ORF	*WDR48*
13	26044030	26044033	+	26044031	17	42	ENSSSCG00000011261	+	3'UTR	*WDR48*
13	26044519	26044519	+	26044519	5	5	XLOC_020197	+	predicted	---
13	26045843	26045853	+	26045844	20	32	XLOC_020197	+	predicted	---
13	26046725	26046725	-	26046725	3	3	ENSSSCG00000021169	-	3'UTR	*GORASP1*
13	26047498	26047498	-	26047498	3	3	ENSSSCG00000021169	-	3'UTR	*GORASP1*
13	26090202	26090203	+	26090202	12	15	---	---	intergenic	---
13	26093001	26093001	-	26093001	9	9	ENSSSCG00000011264	-	3'UTR	*CSRNP1*
13	26196566	26196566	+	26196566	30	30	---	---	intergenic	---

13	26204858	26204875	-	26204875	5	15	ENSSSCG00000024024	-	3'UTR	*CX3CR1*
13	26300123	26300123	+	26300123	3	3	ENSSSCG00000029708	+	ORF	*SLC25A38*
13	26304476	26304479	+	26304478	20	50	ENSSSCG00000029708	+	3'UTR	*SLC25A38*
13	26323211	26323212	-	26323211	7	10	XLOC_021505	-	predicted	---
13	26393294	26393322	+	26393318	552	2284	XLOC_020204	+	predicted	---
13	26450384	26450385	+	26450384	8	13	ENSSSCG00000011267	+	intron	*MOBP*
13	26583784	26583784	+	26583784	3	3	---	---	intergenic	---
13	26909495	26909495	+	26909495	6	6	XLOC_020207	+	predicted	---
13	26931377	26931377	+	26931377	5	5	ENSSSCG00000011270	+	ORF	---
13	26932551	26932557	+	26932557	25	83	ENSSSCG00000011270	+	3'UTR	*EIF1B*
							ENSSSCG00000011270	+	ORF	---
13	26932637	26932637	+	26932637	4	4	ENSSSCG00000011270	+	3'UTR	*EIF1B*
							ENSSSCG00000011270	+	ORF	---
13	27049618	27049638	-	27049628	84	394	ENSSSCG00000024107	-	3'UTR	---
13	27106530	27106579	+	27106531	66	356	ENSSSCG00000011272	+	3'UTR	*RPL14*
13	27107226	27107235	+	27107226	5	8	XLOC_020212	+	predicted	---
13	27615393	27615393	-	27615393	3	3	---	---	intergenic	---
13	27667254	27667260	+	27667257	61	158	ENSSSCG00000011274	+	3'UTR	*CTNNB1*
13	27671835	27671838	-	27671838	6	15	XLOC_021515	-	predicted	---
13	27736080	27736080	+	27736080	3	3	XLOC_020216	+	predicted	---
13	28220583	28220583	-	28220583	16	16	ENSSSCG00000024220	-	intron	---
							ENSSSCG00000024220	-	ORF	---
13	28311313	28311313	-	28311313	3	3	XLOC_021516	-	predicted	---
13	28378845	28378845	-	28378845	3	3	XLOC_021517	-	predicted	---
13	28584941	28584941	+	28584941	3	3	ENSSSCG00000011278	+	intron	*TRAK1*
13	28591953	28591953	+	28591953	5	5	ENSSSCG00000011278	+	intron	*TRAK1*

13	28604306	28604306	+	28604306	5	5	ENSSSCG00000011278	+	3'UTR	*TRAK1*
13	28610211	28610213	-	28610213	10	26	ENSSSCG00000011277	-	3'UTR	*CCK*
13	28610261	28610262	-	28610261	3	6	ENSSSCG00000011277	-	3'UTR	*CCK*
13	28656764	28656774	-	28656767	137	345	XLOC_021521	-	predicted	---
13	28687671	28687671	-	28687671	3	3	---	---	intergenic	---
13	28740357	28740357	+	28740357	3	3	ENSSSCG00000011280	+	3'UTR	*VPAC1*
13	28749776	28749777	-	28749777	9	13	XLOC_021522	-	predicted	---
13	28779069	28779081	+	28779074	4	10	ENSSSCG00000027711	+	3'UTR	*SS18L2*
13	28779120	28779131	+	28779121	36	65	ENSSSCG00000027711	+	3'UTR	*SS18L2*
13	28779380	28779382	+	28779380	30	50	ENSSSCG00000027711	+	3'UTR	*SS18L2*
13	28823169	28823171	+	28823171	9	13	ENSSSCG00000011284	+	intron	*NKTR*
13	28832984	28832984	+	28832984	3	3	ENSSSCG00000011284	+	ORF	*NKTR*
13	28841167	28841167	+	28841167	5	5	ENSSSCG00000011284	+	3'UTR	*NKTR*
13	28844105	28844105	+	28844105	3	3	XLOC_020230	+	predicted	---
13	28861475	28861475	+	28861475	3	3	ENSSSCG00000030065	+	3'UTR	*ZBTB47*
13	28877131	28877131	-	28877131	5	5	XLOC_021525	-	predicted	---
13	28892531	28892531	-	28892531	3	3	XLOC_021525	-	predicted	---
13	28946330	28946340	-	28946332	43	119	ENSSSCG00000011290	-	3'UTR	---
13	28946498	28946506	-	28946498	9	17	ENSSSCG00000011290	-	3'UTR	---
13	28947182	28947195	-	28947190	14	43	ENSSSCG00000011290	-	3'UTR	---
13	28955389	28955389	+	28955389	14	14	---	---	intergenic	---
13	28996497	28996497	-	28996497	3	3	---	---	intergenic	---
13	29037959	29037959	+	29037959	5	5	---	---	intergenic	---
13	29156079	29156079	-	29156079	3	3	---	---	intergenic	---
13	29397661	29397661	+	29397661	4	4	ENSSSCG00000011295	+	3'UTR	*SNRK*
13	29400034	29400034	+	29400034	14	14	XLOC_020237	+	predicted	---

13	29409669	29409678	-	29409676	9	33	XLOC_021533	-	predicted	---
13	29881687	29881701	+	29881691	20	62	ENSSSCG00000011297	+	3'UTR	*ABHD5*
13	29882736	29882740	+	29882739	18	30	ENSSSCG00000011297	+	3'UTR	*ABHD5*
13	29883556	29883562	+	29883559	48	91	ENSSSCG00000011297	+	3'UTR	*ABHD5*
13	29887073	29887073	+	29887073	3	3	---	---	intergenic	---
13	30157474	30157474	-	30157474	3	3	---	---	intergenic	---
13	30494125	30494127	+	30494125	4	8	XLOC_020242	+	predicted	---
13	30621024	30621024	+	30621024	4	4	XLOC_020245	+	predicted	---
13	30630743	30630748	-	30630746	36	62	XLOC_021538	-	predicted	---
13	30637509	30637516	+	30637512	19	61	XLOC_020247	+	predicted	---
13	30802960	30802961	-	30802960	12	15	ENSSSCG00000011308	-	3'UTR	*KIAA1143*
13	30835124	30835124	+	30835124	5	5	ENSSSCG00000011309	+	intron	*KIF15*
13	30844703	30844703	+	30844703	3	3	ENSSSCG00000011309	+	ORF	*KIF15*
13	30845619	30845619	+	30845619	3	3	ENSSSCG00000011309	+	ORF	*KIF15*
13	30854249	30854249	+	30854249	3	3	ENSSSCG00000011309	+	ORF	*KIF15*
13	30867488	30867488	+	30867488	24	24	ENSSSCG00000011309	+	intron	*KIF15*
13	30932629	30932629	+	30932629	3	3	ENSSSCG00000011309	+	ORF	*KIF15*
13	30933480	30933480	+	30933480	8	8	ENSSSCG00000011309	+	3'UTR	*KIF15*
13	30933692	30933706	+	30933699	16	105	ENSSSCG00000011309	+	3'UTR	*KIF15*
13	30933948	30933948	+	30933948	8	8	XLOC_020252	+	predicted	---
13	30941899	30941904	+	30941899	4	8	ENSSSCG00000011302	+	3'UTR	*TMEM42*
13	30960794	30960796	+	30960796	5	8	XLOC_020254	+	predicted	---
13	30963163	30963172	+	30963163	5	9	XLOC_020254	+	predicted	---
13	30991119	30991127	-	30991127	16	22	XLOC_021545	-	predicted	---
13	30992492	30992492	-	30992492	5	5	XLOC_021545	-	predicted	---

13	31084133	31084144	+	31084135	9	31	ENSSSCG00000011300	+	3'UTR	*EXOSC7*
							ENSSSCG00000011300	+	ORF	---
13	31085856	31085856	+	31085856	3	3	ENSSSCG00000011300	+	3'UTR	*EXOSC7*
							ENSSSCG00000011300	+	ORF	---
13	31086640	31086641	+	31086640	7	10	ENSSSCG00000011300	+	3'UTR	*EXOSC7*
							ENSSSCG00000011300	+	ORF	---
13	31093366	31093386	-	31093367	6	18	XLOC_021546	-	predicted	---
13	31097310	31097310	+	31097310	3	3	ENSSSCG00000011299	+	ORF	*CLEC3B*
13	31106757	31106759	+	31106759	15	18	ENSSSCG00000011299	+	3'UTR	*CLEC3B*
13	31447084	31447086	+	31447086	14	24	XLOC_020258	+	predicted	---
13	31641130	31641130	+	31641130	3	3	ENSSSCG00000011312	+	3'UTR	---
13	31794654	31794654	+	31794654	3	3	ENSSSCG00000026885	+	intron	---
13	31969285	31969286	+	31969286	4	7	XLOC_020264	+	predicted	---
13	31969421	31969421	+	31969421	3	3	XLOC_020264	+	predicted	---
13	32073763	32073788	-	32073770	135	166	ENSSSCG00000011314	-	3'UTR	*LZTFL1*
13	32073848	32073859	-	32073850	57	135	ENSSSCG00000011314	-	3'UTR	*LZTFL1*
13	32073939	32073939	-	32073939	20	20	ENSSSCG00000011314	-	3'UTR	*LZTFL1*
13	32075259	32075287	-	32075287	9	13	---	---	intergenic	---
13	32076093	32076099	-	32076093	16	36	ENSSSCG00000011314	-	intron	*LZTFL1*
13	32076664	32076664	-	32076664	4	4	---	---	intergenic	---
13	32079298	32079307	-	32079307	5	8	ENSSSCG00000011314	-	intron	*LZTFL1*
13	32080859	32080880	-	32080880	19	31	---	---	intergenic	---
13	32323639	32323650	-	32323650	26	39	ENSSSCG00000011322	-	3'UTR	*CCR1*
13	32351722	32351725	+	32351723	11	22	ENSSSCG00000024408	+	intron	*CCR3*
13	32540174	32540175	+	32540175	7	12	XLOC_020270	+	predicted	---

13	32551727	32551727	+	32551727	4	4	ENSSSCG00000024344	+	3'UTR	*CCR5*
							ENSSSCG00000024344	+	ORF	---
13	32568453	32568453	+	32568453	8	8	ENSSSCG00000023557	+	3'UTR	*CCRL2*
							ENSSSCG00000023557	+	ORF	---
13	32577727	32577740	-	32577740	4	7	ENSSSCG00000029879	-	3'UTR	*LTF*
13	32699284	32699286	-	32699286	9	17	ENSSSCG00000028839	-	intron	*TMEM7*
13	32699764	32699764	-	32699764	3	3	ENSSSCG00000028839	-	intron	*TMEM7*
13	32720285	32720286	+	32720285	4	7	---	---	intergenic	---
13	32905218	32905219	+	32905218	11	15	XLOC_020278	+	predicted	---
13	32955745	32955750	-	32955745	6	9	XLOC_021575	-	predicted	---
13	32978262	32978262	-	32978262	15	15	XLOC_021575	-	predicted	---
13	33030971	33030973	-	33030973	11	19	XLOC_021576	-	predicted	---
13	33037855	33037866	-	33037864	22	86	---	---	intergenic	---
13	33056812	33056812	+	33056812	4	4	---	---	intergenic	---
13	33067124	33067124	-	33067124	5	5	ENSSSCG00000011329	-	3'UTR	---
13	33068009	33068013	-	33068011	13	32	ENSSSCG00000011329	-	3'UTR	---
13	33143414	33143424	-	33143414	3	6	ENSSSCG00000011332	-	3'UTR	*SETD2*
13	33210247	33210247	-	33210247	3	3	ENSSSCG00000011332	-	ORF	*SETD2*
13	33225487	33225487	-	33225487	3	3	ENSSSCG00000011332	-	ORF	*SETD2*
13	33229089	33229089	-	33229089	3	3	ENSSSCG00000011332	-	ORF	*SETD2*
13	33373726	33373726	+	33373726	3	3	ENSSSCG00000024513	+	3'UTR	*KLHL18*
13	33483930	33483940	-	33483933	22	58	ENSSSCG00000026281	-	3'UTR	*SCAP*
13	33559683	33559683	-	33559683	3	3	ENSSSCG00000011336	-	3'UTR	*ELP6*
13	33560675	33560683	-	33560683	28	49	ENSSSCG00000011336	-	3'UTR	*ELP6*
13	33594809	33594809	+	33594809	3	3	---	---	intergenic	---
13	33623686	33623687	+	33623687	11	18	---	---	intergenic	---

13	33625617	33625619	+	33625617	3	6	---	---	intergenic	---
13	33625734	33625737	+	33625736	18	35	---	---	intergenic	---
13	33891239	33891252	+	33891241	24	62	XLOC_020290	+	predicted	---
13	33895894	33895897	+	33895896	34	62	ENSSSCG00000011342	+	ORF	*DHX30*
13	33908840	33908846	+	33908845	35	112	ENSSSCG00000011342	+	3'UTR	*DHX30*
13	33909389	33909392	-	33909390	37	80	XLOC_021588	-	predicted	---
13	33910083	33910091	-	33910091	10	13	XLOC_021588	-	predicted	---
13	33921606	33921606	-	33921606	3	3	ENSSSCG00000011343	-	ORF	*MAP4*
13	33922660	33922660	-	33922660	3	3	ENSSSCG00000011343	-	ORF	*MAP4*
13	33928602	33928602	-	33928602	4	4	ENSSSCG00000011343	-	intron	*MAP4*
13	33954366	33954366	-	33954366	7	7	---	---	intergenic	---
13	33993035	33993035	+	33993035	3	3	---	---	intergenic	---
13	33993647	33993648	+	33993648	11	16	---	---	intergenic	---
13	34035879	34035879	-	34035879	6	6	---	---	intergenic	---
13	34125331	34125331	-	34125331	9	9	---	---	intergenic	---
13	34234574	34234574	-	34234574	3	3	---	---	intergenic	---
13	34295024	34295026	-	34295025	15	34	ENSSSCG00000011347	-	3'UTR	*NME6*
13	34307581	34307581	-	34307581	6	6	---	---	intergenic	---
13	34347079	34347079	+	34347079	6	6	---	---	intergenic	---
13	34407786	34407786	-	34407786	5	5	ENSSSCG00000011350	-	3'UTR	*CCDC51*
13	34420791	34420801	+	34420799	37	81	ENSSSCG00000011351	+	3'UTR	*TMA7*
13	34457006	34457006	-	34457006	4	4	XLOC_021602	-	predicted	---
13	34542912	34542932	-	34542913	163	387	ENSSSCG00000011356	-	3'UTR	*UQCRC1*
13	34553591	34553591	+	34553591	3	3	---	---	intergenic	---
13	34561737	34561741	+	34561739	48	73	ENSSSCG00000011357	+	3'UTR	*SHISA5*
13	34561821	34561821	-	34561821	3	3	---	---	intergenic	---

13	34561988	34561992	-	34561988	8	18	---	---	intergenic	---
13	34563866	34563872	-	34563872	14	37	XLOC_021605	-	predicted	---
13	34579355	34579372	-	34579363	19	111	XLOC_021606	-	predicted	---
13	34582927	34582928	-	34582927	10	13	ENSSSCG00000011361	-	3'UTR	---
13	34646757	34646769	-	34646761	24	77	ENSSSCG00000011360	-	intron	*CELSR3*
13	34653098	34653098	-	34653098	3	3	ENSSSCG00000011360	-	intron	*CELSR3*
13	34653350	34653350	-	34653350	3	3	ENSSSCG00000011360	-	intron	*CELSR3*
13	34687796	34687802	-	34687797	10	16	XLOC_021607	-	predicted	---
13	34687843	34687851	-	34687845	32	62	XLOC_021607	-	predicted	---
13	34777392	34777402	-	34777401	19	48	ENSSSCG00000011366	-	3'UTR	*SLC25A20*
13	34859651	34859651	+	34859651	6	6	ENSSSCG00000011367	+	intron	*ARIH2*
13	34871776	34871787	+	34871780	26	54	XLOC_020306	+	predicted	---
13	34873593	34873599	+	34873598	10	19	XLOC_020306	+	predicted	---
13	34885562	34885562	+	34885562	3	3	ENSSSCG00000011368	+	3'UTR	*P4HTM*
13	34893986	34894001	-	34893991	22	61	XLOC_021611	-	predicted	---
13	34894479	34894485	+	34894482	22	69	XLOC_020307	+	predicted	---
13	34901087	34901090	+	34901087	80	178	ENSSSCG00000011371	+	3'UTR	*NDUFAF3*
13	34901653	34901653	-	34901653	19	19	XLOC_021613	-	predicted	---
13	34902226	34902226	+	34902226	4	4	XLOC_020307	+	predicted	---
13	34903913	34903913	-	34903913	3	3	ENSSSCG00000011372	-	ORF	*IMPDH2*
13	34906820	34906820	-	34906820	14	14	ENSSSCG00000011373	-	3'UTR	*QRICH1*
13	34958842	34958842	-	34958842	4	4	ENSSSCG00000011375	-	3'UTR	*USP19*
13	34959489	34959492	-	34959490	7	13	ENSSSCG00000011375	-	intron	*USP19*
13	34998038	34998038	-	34998038	5	5	ENSSSCG00000011378	-	3'UTR	*CCDC71*
13	35011028	35011029	+	35011029	6	10	ENSSSCG00000011379	+	3'UTR	*KLHDC8B*
13	35011822	35011826	-	35011824	68	142	ENSSSCG00000011380	-	ORF	*C3orf84*

13	35029060	35029060	+	35029060	3	3	XLOC_020310	+	predicted	---
13	35035229	35035259	-	35035231	273	814	ENSSSCG00000026739	-	3'UTR	---
13	35035402	35035411	-	35035411	7	12	ENSSSCG00000026739	-	3'UTR	---
13	35035539	35035561	-	35035560	32	64	ENSSSCG00000026739	-	ORF	---
13	35061769	35061769	+	35061769	8	8	ENSSSCG00000011376	+	intron	---
13	35075783	35075812	-	35075785	54	215	XLOC_021621	-	predicted	---
13	35079137	35079137	-	35079137	4	4	XLOC_021621	-	predicted	---
13	35079937	35079937	-	35079937	3	3	XLOC_021621	-	predicted	---
13	35080287	35080287	-	35080287	5	5	ENSSSCG00000011382	-	3'UTR	*USP4*
13	35080355	35080355	-	35080355	4	4	ENSSSCG00000011382	-	3'UTR	*USP4*
13	35080407	35080434	-	35080434	12	28	ENSSSCG00000011382	-	3'UTR	*USP4*
13	35080707	35080707	-	35080707	3	3	ENSSSCG00000011382	-	3'UTR	*USP4*
13	35088162	35088162	-	35088162	3	3	ENSSSCG00000011382	-	ORF	*USP4*
13	35140034	35140037	-	35140036	7	20	---	---	intergenic	---
13	35147251	35147257	-	35147254	559	1338	XLOC_021622	-	predicted	---
13	35148078	35148082	-	35148082	7	13	XLOC_021622	-	predicted	---
13	35148163	35148163	-	35148163	4	4	XLOC_021622	-	predicted	---
13	35149486	35149486	-	35149486	5	5	XLOC_021622	-	predicted	---
13	35149846	35149846	-	35149846	8	8	ENSSSCG00000022080	-	3'UTR	*RHOA*
13	35149993	35149999	-	35149997	79	180	ENSSSCG00000022080	-	3'UTR	*RHOA*
13	35150690	35150690	-	35150690	3	3	ENSSSCG00000022080	-	ORF	*RHOA*
13	35154722	35154722	-	35154722	4	4	ENSSSCG00000022080	-	ORF	*RHOA*
13	35319572	35319573	+	35319572	27	42	---	---	intergenic	---
13	35322474	35322476	-	35322474	5	9	---	---	intergenic	---
13	35329491	35329492	+	35329491	4	7	---	---	intergenic	---
13	35393714	35393717	+	35393716	12	28	ENSSSCG00000011385	+	3'UTR	---

13	35394133	35394138	-	35394135	68	147	ENSSSCG00000011386	-	3'UTR	*MST1*
13	35425675	35425675	-	35425675	6	6	XLOC_021625	-	predicted	---
13	35428667	35428667	-	35428667	19	19	XLOC_021625	-	predicted	---
13	35429913	35429919	-	35429919	7	13	ENSSSCG00000011389	-	3'UTR	*GMPPB*
13	35432716	35432723	-	35432717	33	57	ENSSSCG00000011390	-	3'UTR	*IP6K1*
13	35435456	35435502	-	35435462	7	33	ENSSSCG00000011390	-	3'UTR	*IP6K1*
13	35438585	35438585	-	35438585	3	3	ENSSSCG00000011390	-	intron	*IP6K1*
13	35501289	35501289	-	35501289	3	3	XLOC_021626	-	predicted	---
13	35511416	35511417	+	35511417	10	13	---	---	intergenic	---
13	35511589	35511593	-	35511589	12	22	ENSSSCG00000011393	-	intron	*UBA7*
13	35558558	35558558	-	35558558	4	4	ENSSSCG00000026977	-	intron	*TRAIP*
13	35561869	35561869	-	35561869	3	3	ENSSSCG00000025416	-	3'UTR	*CAMKV*
13	35576938	35576944	-	35576942	40	125	---	---	intergenic	---
13	35578524	35578524	-	35578524	15	15	ENSSSCG00000024930	-	ORF	---
13	35579522	35579522	-	35579522	3	3	ENSSSCG00000024930	-	ORF	---
13	35607468	35607474	-	35607468	7	24	ENSSSCG00000025791	-	3'UTR	*MON1A*
13	35734676	35734677	+	35734676	4	7	ENSSSCG00000011394	+	ORF	*RBM6*
13	35742589	35742589	+	35742589	3	3	ENSSSCG00000011394	+	intron	*RBM6*
13	35749583	35749588	+	35749588	6	10	ENSSSCG00000011394	+	3'UTR	*RBM6*
13	35749684	35749684	+	35749684	14	14	ENSSSCG00000011394	+	3'UTR	*RBM6*
13	35759957	35759958	+	35759958	52	63	---	---	intergenic	---
13	35791843	35791852	+	35791843	41	45	ENSSSCG00000011395	+	3'UTR	*RBM5*
13	35877719	35877724	+	35877723	21	58	ENSSSCG00000011397	+	3'UTR	*SLC38A3*
13	35878394	35878405	+	35878404	10	35	ENSSSCG00000011397	+	3'UTR	*SLC38A3*
13	35878448	35878448	+	35878448	3	3	XLOC_020327	+	predicted	---
13	35911758	35911758	+	35911758	3	3	XLOC_020329	+	predicted	---

13	35911829	35911829	+	35911829	4	4	XLOC_020329	+	predicted	---
13	35912186	35912186	+	35912186	8	8	XLOC_020329	+	predicted	---
13	35935210	35935219	-	35935219	22	59	ENSSSCG00000024363	-	3'UTR	*IFRD2*
13	35979323	35979323	+	35979323	4	4	---	---	intergenic	---
13	35983753	35983755	-	35983754	7	16	XLOC_021633	-	predicted	---
13	35990721	35990723	-	35990722	15	26	ENSSSCG00000011406	-	3'UTR	*TUSC2*
13	35991790	35991790	-	35991790	4	4	ENSSSCG00000011406	-	3'UTR	*TUSC2*
13	35994680	35994697	-	35994690	32	77	XLOC_021634	-	predicted	---
13	36003899	36003908	-	36003905	73	158	XLOC_021634	-	predicted	---
13	36009112	36009119	-	36009114	9	17	XLOC_021635	-	predicted	---
13	36015878	36015887	-	36015881	18	48	ENSSSCG00000011411	-	3'UTR	*TMEM115*
13	36262268	36262269	+	36262269	11	14	XLOC_020333	+	predicted	---
13	36318931	36318937	-	36318932	9	26	ENSSSCG00000011415	-	3'UTR	*MAPKAPK3*
13	36418859	36418859	-	36418859	3	3	---	---	intergenic	---
13	36451527	36451527	+	36451527	6	6	---	---	intergenic	---
13	36822944	36822946	+	36822945	5	12	ENSSSCG00000011416	+	intron	*DOCK3*
13	36885950	36885951	+	36885951	4	7	XLOC_020338	+	predicted	---
13	36886513	36886513	-	36886513	10	10	XLOC_021643	-	predicted	---
13	36886573	36886574	+	36886573	4	8	XLOC_020338	+	predicted	---
13	36890084	36890087	-	36890084	5	9	XLOC_021643	-	predicted	---
13	36895622	36895622	-	36895622	4	4	ENSSSCG00000011421	-	ORF	*MANF*
13	36959170	36959171	-	36959171	5	9	ENSSSCG00000011417	-	intron	*VPRBP*
13	36969251	36969263	+	36969262	21	70	XLOC_020339	+	predicted	---
13	36972273	36972273	+	36972273	6	6	---	---	intergenic	---
13	37127963	37127976	+	37127971	55	103	XLOC_020341	+	predicted	---
13	37170753	37170759	-	37170756	10	22	XLOC_021651	-	predicted	---

13	37188927	37188930	-	37188928	9	25	XLOC_021651	-	predicted	---
13	37204138	37204174	+	37204174	4	7	XLOC_020342	+	predicted	---
13	37220494	37220502	+	37220500	70	168	XLOC_020342	+	predicted	---
13	37227651	37227664	+	37227662	84	204	XLOC_020342	+	predicted	---
13	37232654	37232666	+	37232654	4	8	---	---	intergenic	---
13	37234153	37234163	+	37234159	30	59	XLOC_020342	+	predicted	---
13	37243518	37243527	-	37243523	700	1297	XLOC_021653	-	predicted	---
13	37243566	37243566	-	37243566	9	9	ENSSSCG00000025367	-	ORF	*IQCF5*
13	37243612	37243612	-	37243612	3	3	ENSSSCG00000025367	-	ORF	*IQCF5*
13	37254230	37254247	-	37254239	217	1002	XLOC_021654	-	predicted	---
13	37264059	37264062	-	37264061	13	35	ENSSSCG00000023177	-	3'UTR	*RRP9*
13	37278881	37278891	+	37278885	7	22	ENSSSCG00000023033	+	3'UTR	*PARP3*
13	37286566	37286566	-	37286566	3	3	ENSSSCG00000028446	-	3'UTR	*PCBP4*
13	37297687	37297694	-	37297688	43	103	ENSSSCG00000026847	-	3'UTR	*ABHD14B*
13	37315578	37315598	+	37315582	46	119	ENSSSCG00000023325	+	3'UTR	*ACY1*
13	37316231	37316242	-	37316235	518	824	ENSSSCG00000027573	-	3'UTR	*RPL29*
13	37316363	37316363	-	37316363	4	4	ENSSSCG00000027573	-	ORF	*RPL29*
13	37372645	37372646	-	37372646	37	52	---	---	intergenic	---
13	37615110	37615110	+	37615110	4	4	ENSSSCG00000011433	+	3'UTR	*WDR82*
13	37617093	37617093	+	37617093	3	3	ENSSSCG00000011433	+	3'UTR	*WDR82*
13	37617522	37617538	+	37617523	8	26	ENSSSCG00000011433	+	3'UTR	*WDR82*
13	37619741	37619744	-	37619744	9	17	ENSSSCG00000011434	-	3'UTR	*PPM1M*
13	37657861	37657921	-	37657874	134	492	ENSSSCG00000011437	-	3'UTR	*ALAS1*
13	37658085	37658085	-	37658085	6	6	ENSSSCG00000011437	-	ORF	*ALAS1*
13	37665718	37665718	-	37665718	20	20	ENSSSCG00000011437	-	ORF	*ALAS1*
13	37671057	37671057	-	37671057	4	4	XLOC_021668	-	predicted	---

13	37757888	37757888	+	37757888	3	3	XLOC_020357	+	predicted	---
13	37758313	37758327	-	37758326	22	42	XLOC_021669	-	predicted	---
13	37759223	37759223	-	37759223	3	3	XLOC_021669	-	predicted	---
13	37778961	37778961	+	37778961	3	3	ENSSSCG00000011439	+	ORF	*PHF7*
13	37779386	37779390	+	37779387	489	845	ENSSSCG00000011439	+	3'UTR	*PHF7*
13	37787903	37787903	-	37787903	3	3	XLOC_021670	-	predicted	---
13	37805135	37805136	-	37805135	47	50	XLOC_021671	-	predicted	---
13	37831283	37831283	+	37831283	4	4	ENSSSCG00000011442	+	intron	*NISCH*
13	37844039	37844045	+	37844043	29	57	ENSSSCG00000011442	+	3'UTR	*NISCH*
13	37872006	37872008	-	37872007	9	16	ENSSSCG00000011444	-	3'UTR	*NT5DC2*
13	37872090	37872124	+	37872109	29	60	ENSSSCG00000011443	+	3'UTR	*STAB1*
13	37894049	37894056	+	37894049	12	24	---	---	intergenic	---
13	37897741	37897747	-	37897741	15	19	XLOC_021673	-	predicted	---
13	37918762	37918762	+	37918762	3	3	---	---	intergenic	---
13	38003386	38003387	-	38003387	20	23	---	---	intergenic	---
13	38028552	38028552	+	38028552	20	20	ENSSSCG00000011449	+	3'UTR	*GNL3*
13	38028637	38028640	-	38028640	27	40	ENSSSCG00000011448	-	3'UTR	*GLT8D1*
13	38041365	38041391	+	38041365	12	18	ENSSSCG00000011447	+	3'UTR	*SPCS1*
13	38041556	38041561	+	38041561	6	11	ENSSSCG00000011447	+	3'UTR	*SPCS1*
13	38041618	38041640	+	38041634	412	996	ENSSSCG00000011447	+	3'UTR	*SPCS1*
13	38045068	38045073	-	38045069	12	19	XLOC_021677	-	predicted	---
13	38056647	38056647	-	38056647	4	4	ENSSSCG00000011446	-	intron	*NEK4*
13	38088560	38088560	+	38088560	3	3	ENSSSCG00000011450	+	ORF	*ITIH1*
13	38089269	38089269	+	38089269	12	12	ENSSSCG00000011450	+	intron	*ITIH1*
13	38089734	38089734	+	38089734	14	14	ENSSSCG00000011450	+	ORF	*ITIH1*
13	38092215	38092215	+	38092215	3	3	ENSSSCG00000011450	+	intron	*ITIH1*

13	38101150	38101150	+	38101150	23	23	ENSSSCG00000011450	+	intron	*ITIH1*
13	38101569	38101580	+	38101575	1258	2049	ENSSSCG00000011450	+	3'UTR	*ITIH1*
13	38110993	38110993	+	38110993	3	3	ENSSSCG00000011451	+	ORF	*ITIH3*
13	38112371	38112382	+	38112371	44	57	ENSSSCG00000011451	+	intron	*ITIH3*
13	38119527	38119527	+	38119527	3	3	ENSSSCG00000011451	+	intron	*ITIH3*
13	38120161	38120182	+	38120168	318	676	ENSSSCG00000011451	+	3'UTR	*ITIH3*
13	38120234	38120234	+	38120234	10	10	XLOC_020365	+	predicted	---
13	38123764	38123865	-	38123825	1436	3176	XLOC_021679	-	predicted	---
13	38124848	38124848	+	38124848	3	3	XLOC_020365	+	predicted	---
13	38191798	38191798	+	38191798	5	5	ENSSSCG00000011452	+	intron	---
13	38195108	38195121	+	38195116	20	63	ENSSSCG00000011452	+	3'UTR	---
13	38198943	38198943	+	38198943	4	4	ENSSSCG00000011453	+	ORF	*ITIH4*
13	38202099	38202099	+	38202099	14	14	ENSSSCG00000011453	+	ORF	*ITIH4*
13	38203453	38203453	+	38203453	4	4	ENSSSCG00000011453	+	ORF	*ITIH4*
13	38206865	38206866	+	38206865	8	11	ENSSSCG00000011453	+	ORF	*ITIH4*
13	38213290	38213296	-	38213295	16	33	ENSSSCG00000011454	-	3'UTR	*SFMBT1*
13	38238693	38238705	-	38238705	7	10	ENSSSCG00000011454	-	intron	*SFMBT1*
13	38390188	38390206	+	38390205	18	34	XLOC_020368	+	predicted	---
13	38390507	38390507	+	38390507	3	3	XLOC_020368	+	predicted	---
13	38418411	38418418	-	38418418	7	11	XLOC_021684	-	predicted	---
13	38422814	38422814	-	38422814	4	4	ENSSSCG00000021408	-	ORF	*TKT*
13	38423695	38423695	-	38423695	4	4	ENSSSCG00000021408	-	ORF	*TKT*
13	38449071	38449071	+	38449071	4	4	---	---	intergenic	---
13	38462232	38462232	-	38462232	6	6	ENSSSCG00000022616	-	3'UTR	*DCP1A*
13	38466510	38466510	-	38466510	5	5	ENSSSCG00000022616	-	ORF	*DCP1A*
13	38517241	38517241	-	38517241	3	3	ENSSSCG00000022616	-	intron	*DCP1A*

13	38984220	38984245	-	38984235	7	15	XLOC_021687	-	predicted	---
13	39033825	39033825	-	39033825	7	7	XLOC_021688	-	predicted	---
13	39034623	39034630	-	39034623	4	11	XLOC_021688	-	predicted	---
13	39043762	39043762	-	39043762	6	6	ENSSSCG00000011458	-	intron	*ACTR8*
13	39060204	39060221	-	39060220	36	118	XLOC_021689	-	predicted	---
13	39060303	39060309	-	39060305	39	121	ENSSSCG00000011459	-	3'UTR	*SELK*
13	39060444	39060465	-	39060446	42	105	ENSSSCG00000011459	-	3'UTR	*SELK*
13	39406949	39406957	+	39406957	4	7	XLOC_020374	+	predicted	---
13	39422005	39422005	+	39422005	4	4	---	---	intergenic	---
13	39729140	39729140	+	39729140	3	3	ENSSSCG00000022522	+	intron	---
13	41052871	41052872	-	41052872	7	11	---	---	intergenic	---
13	41395311	41395313	-	41395311	3	6	ENSSSCG00000025776	-	intron	---
13	41841894	41841894	+	41841894	6	6	---	---	intergenic	---
13	41844570	41844571	+	41844570	8	14	---	---	intergenic	---
13	41864518	41864518	-	41864518	6	6	---	---	intergenic	---
13	42022324	42022324	-	42022324	42	42	---	---	intergenic	---
13	42314331	42314331	+	42314331	8	8	ENSSSCG00000021439	+	intron	*CCDC66*
13	42349684	42349684	-	42349684	4	4	ENSSSCG00000024201	-	ORF	*FAM208A*
13	42362635	42362635	-	42362635	9	9	ENSSSCG00000024201	-	ORF	*FAM208A*
13	42429459	42429460	-	42429460	9	16	ENSSSCG00000030359	-	3'UTR	*ARHGEF3*
13	42616987	42616998	-	42616996	41	125	---	---	intergenic	---
13	43310736	43310736	-	43310736	10	10	---	---	intergenic	---
13	43330611	43330611	+	43330611	8	8	ENSSSCG00000011465	+	3'UTR	*PDE12*
13	43333737	43333737	+	43333737	3	3	XLOC_020386	+	predicted	---
13	43341770	43341782	-	43341773	193	406	ENSSSCG00000011466	-	3'UTR	*ARF4*
13	43342195	43342195	-	43342195	39	39	ENSSSCG00000011466	-	3'UTR	*ARF4*

13	43342316	43342330	-	43342329	5	11	ENSSSCG00000011466	-	3'UTR	*ARF4*
13	43342503	43342540	-	43342524	11	44	ENSSSCG00000011466	-	3'UTR	*ARF4*
13	43342621	43342621	-	43342621	5	5	ENSSSCG00000011466	-	3'UTR	*ARF4*
13	43351535	43351535	-	43351535	7	7	ENSSSCG00000011466	-	ORF	*ARF4*
13	43386686	43386686	-	43386686	5	5	ENSSSCG00000011467	-	3'UTR	*DENND6A*
13	43748153	43748158	-	43748158	10	16	XLOC_021707	-	predicted	---
13	43853576	43853576	+	43853576	3	3	---	---	intergenic	---
13	43872875	43872876	-	43872876	15	22	ENSSSCG00000011471	-	3'UTR	*FLNB*
13	43874303	43874303	-	43874303	3	3	ENSSSCG00000011471	-	3'UTR	*FLNB*
13	43927679	43927681	-	43927679	16	24	XLOC_021710	-	predicted	---
13	43927796	43927796	-	43927796	4	4	XLOC_021710	-	predicted	---
13	44015519	44015526	+	44015519	11	33	XLOC_020392	+	predicted	---
13	44038485	44038489	+	44038488	12	24	XLOC_020392	+	predicted	---
13	44063128	44063129	+	44063129	36	44	ENSSSCG00000011474	+	intron	*PXK*
13	44120681	44120685	+	44120682	9	17	ENSSSCG00000011474	+	3'UTR	*PXK*
13	44121782	44121782	-	44121782	3	3	ENSSSCG00000025881	-	3'UTR	*PDHB*
13	44121825	44121828	-	44121827	35	60	ENSSSCG00000025881	-	3'UTR	*PDHB*
13	44121875	44121875	-	44121875	3	3	ENSSSCG00000025881	-	3'UTR	*PDHB*
13	44122210	44122218	-	44122210	4	8	ENSSSCG00000025881	-	ORF	*PDHB*
13	44232255	44232262	-	44232259	69	111	ENSSSCG00000011477	-	3'UTR	*ACOX2*
13	44232435	44232447	-	44232441	9	21	ENSSSCG00000011477	-	3'UTR	*ACOX2*
13	44285779	44285781	-	44285779	15	21	XLOC_021714	-	predicted	---
13	44581081	44581083	-	44581083	5	8	XLOC_021717	-	predicted	---
13	44945668	44945668	+	44945668	3	3	---	---	intergenic	---
13	45307026	45307026	+	45307026	3	3	---	---	intergenic	---
13	45494918	45494918	-	45494918	3	3	---	---	intergenic	---

13	45517587	45517587	+	45517587	3	3	---	---	intergenic	---
13	45527354	45527354	+	45527354	3	3	---	---	intergenic	---
13	45630809	45630810	+	45630809	3	6	XLOC_020400	+	predicted	---
13	45684351	45684351	+	45684351	7	7	---	---	intergenic	---
13	46066322	46066323	-	46066322	9	14	---	---	intergenic	---
13	46562130	46562130	+	46562130	8	8	---	---	intergenic	---
13	46781133	46781133	+	46781133	5	5	---	---	intergenic	---
13	47180471	47180482	-	47180472	8	64	---	---	intergenic	---
13	47290893	47290893	-	47290893	5	5	---	---	intergenic	---
13	47374657	47374657	+	47374657	4	4	---	---	intergenic	---
13	47869614	47869614	+	47869614	5	5	XLOC_020404	+	predicted	---
13	47873300	47873301	+	47873301	9	12	XLOC_020404	+	predicted	---
13	47918042	47918049	+	47918042	8	24	XLOC_020405	+	predicted	---
13	47929593	47929593	-	47929593	27	27	---	---	intergenic	---
13	47929613	47929614	+	47929613	14	20	---	---	intergenic	---
13	47983325	47983326	-	47983326	32	46	---	---	intergenic	---
13	47994718	47994719	-	47994718	18	21	---	---	intergenic	---
13	48032734	48032734	+	48032734	4	4	---	---	intergenic	---
13	48054032	48054032	-	48054032	10	10	---	---	intergenic	---
13	48129409	48129409	-	48129409	3	3	ENSSSCG00000011489	-	intron	---
13	48642532	48642533	-	48642533	5	8	---	---	intergenic	---
13	49623075	49623082	+	49623079	37	65	---	---	intergenic	---
13	49623306	49623306	+	49623306	9	9	---	---	intergenic	---
13	49647938	49647938	-	49647938	113	113	---	---	intergenic	---
13	49648019	49648020	+	49648019	12	15	---	---	intergenic	---
13	49648191	49648191	+	49648191	4	4	---	---	intergenic	---

13	49778438	49778438	-	49778438	19	19	---	---	intergenic	---
13	49795027	49795027	-	49795027	4	4	---	---	intergenic	---
13	49812963	49812971	-	49812967	169	432	ENSSSCG00000011491	-	3'UTR	*THOC7*
13	49818690	49818691	-	49818691	35	38	ENSSSCG00000011491	-	ORF	*THOC7*
13	49819103	49819103	-	49819103	11	11	ENSSSCG00000011491	-	ORF	*THOC7*
13	49948215	49948215	-	49948215	3	3	ENSSSCG00000011493	-	intron	*ATXN7*
13	49995125	49995141	-	49995134	97	222	ENSSSCG00000025202	-	3'UTR	---
13	50517745	50517745	-	50517745	4	4	XLOC_021739	-	predicted	---
13	50840631	50840637	+	50840632	13	24	---	---	intergenic	---
13	50840806	50840806	+	50840806	9	9	---	---	intergenic	---
13	50847287	50847296	-	50847287	3	9	---	---	intergenic	---
13	50847300	50847308	+	50847308	4	10	---	---	intergenic	---
13	50847448	50847463	-	50847454	11	83	---	---	intergenic	---
13	50847492	50847511	+	50847495	23	152	---	---	intergenic	---
13	51389794	51389794	-	51389794	4	4	XLOC_021742	-	predicted	---
13	51805814	51805814	-	51805814	5	5	ENSSSCG00000011497	-	intron	*MAGI1*
13	51835438	51835438	-	51835438	3	3	---	---	intergenic	---
13	52089139	52089139	+	52089139	5	5	---	---	intergenic	---
13	52290058	52290058	-	52290058	3	3	XLOC_021745	-	predicted	---
13	52484757	52484757	+	52484757	3	3	XLOC_020423	+	predicted	---
13	52650489	52650492	+	52650491	14	31	ENSSSCG00000011498	+	3'UTR	*SLC25A26*
13	52725925	52725925	-	52725925	3	3	ENSSSCG00000011499	-	intron	*LRIG1*
13	52923802	52923802	+	52923802	3	3	---	---	intergenic	---
13	53329940	53329940	+	53329940	3	3	ENSSSCG00000011500	+	3'UTR	---
13	53672082	53672091	-	53672084	72	140	ENSSSCG00000011501	-	3'UTR	*SUCLG2*
13	53672393	53672393	-	53672393	8	8	ENSSSCG00000011501	-	3'UTR	*SUCLG2*

13	53672820	53672820	-	53672820	3	3	ENSSSCG00000011501	-	3'UTR	*SUCLG2*
13	53673001	53673019	-	53673001	9	19	ENSSSCG00000011501	-	3'UTR	*SUCLG2*
13	53892700	53892700	-	53892700	7	7	ENSSSCG00000011501	-	intron	*SUCLG2*
13	53900517	53900523	-	53900517	4	7	ENSSSCG00000011501	-	ORF	*SUCLG2*
13	53975538	53975544	-	53975538	49	52	ENSSSCG00000011501	-	intron	*SUCLG2*
13	54130301	54130301	-	54130301	3	3	XLOC_021753	-	predicted	---
13	54136467	54136479	-	54136468	53	81	---	---	intergenic	---
13	54577868	54577868	-	54577868	4	4	---	---	intergenic	---
13	54617307	54617307	+	54617307	3	3	---	---	intergenic	---
13	54675797	54675797	+	54675797	14	14	---	---	intergenic	---
13	55317490	55317490	-	55317490	3	3	---	---	intergenic	---
13	55478586	55478586	-	55478586	4	4	ENSSSCG00000011504	-	3'UTR	*EOGT*
13	55528715	55528715	-	55528715	5	5	ENSSSCG00000011510	-	3'UTR	*TMF1*
13	55545093	55545093	-	55545093	3	3	ENSSSCG00000011510	-	ORF	*TMF1*
13	55549975	55549975	-	55549975	3	3	ENSSSCG00000011510	-	ORF	*TMF1*
13	55554548	55554585	-	55554548	4	7	ENSSSCG00000011510	-	ORF	*TMF1*
13	55554836	55554836	-	55554836	3	3	ENSSSCG00000011510	-	ORF	*TMF1*
13	55560771	55560771	-	55560771	4	4	ENSSSCG00000011506	-	3'UTR	*UBA3*
13	55668851	55668851	+	55668851	8	8	ENSSSCG00000011507	+	3'UTR	*ARL6IP5*
13	55672248	55672248	-	55672248	9	9	XLOC_021758	-	predicted	---
13	55735646	55735646	-	55735646	4	4	ENSSSCG00000011511	-	3'UTR	*FRMD4B*
13	56219181	56219181	-	56219181	5	5	---	---	intergenic	---
13	56313108	56313108	-	56313108	4	4	---	---	intergenic	---
13	56491925	56491925	-	56491925	5	5	---	---	intergenic	---
13	56590673	56590674	-	56590674	9	12	XLOC_021764	-	predicted	---
13	57367639	57367639	+	57367639	5	5	---	---	intergenic	---

13	57861730	57861733	-	57861733	5	8	ENSSSCG00000027675	-	3'UTR	---
13	57864077	57864077	-	57864077	6	6	ENSSSCG00000027675	-	3'UTR	---
13	58268453	58268453	+	58268453	3	3	---	---	intergenic	---
13	58523555	58523555	-	58523555	4	4	---	---	intergenic	---
13	58682147	58682147	+	58682147	3	3	---	---	intergenic	---
13	58774495	58774495	-	58774495	3	3	XLOC_021774	-	predicted	---
13	58816048	58816048	-	58816048	49	49	ENSSSCG00000011516	-	intron	*EIF4E3*
13	58842653	58842653	+	58842653	4	4	---	---	intergenic	---
13	58843618	58843618	-	58843618	3	3	---	---	intergenic	---
13	58857151	58857151	-	58857151	4	4	XLOC_021775	-	predicted	---
13	58857546	58857548	-	58857548	11	20	XLOC_021775	-	predicted	---
13	58858551	58858551	-	58858551	4	4	XLOC_021775	-	predicted	---
13	59045886	59045886	+	59045886	15	15	---	---	intergenic	---
13	59424977	59424985	-	59424984	7	14	---	---	intergenic	---
13	59462865	59462872	-	59462865	5	8	ENSSSCG00000025053	-	3'UTR	*RYBP*
13	59576842	59576842	-	59576842	5	5	XLOC_021782	-	predicted	---
13	59584712	59584718	+	59584717	12	21	---	---	intergenic	---
13	59616751	59616751	-	59616751	13	13	---	---	intergenic	---
13	59958699	59958699	+	59958699	3	3	ENSSSCG00000011519	+	intron	*GXYLT2*
13	59977809	59977812	+	59977811	22	42	ENSSSCG00000011519	+	intron	*GXYLT2*
13	59984265	59984265	+	59984265	8	8	ENSSSCG00000011519	+	intron	*GXYLT2*
13	60046157	60046161	-	60046161	20	75	---	---	intergenic	---
13	60105408	60105409	-	60105408	15	23	---	---	intergenic	---
13	60140939	60140939	-	60140939	7	7	---	---	intergenic	---
13	60161339	60161339	-	60161339	4	4	---	---	intergenic	---
13	60377638	60377638	+	60377638	5	5	---	---	intergenic	---

13	60394149	60394149	-	60394149	4	4	---	---	intergenic	---
13	61496517	61496517	-	61496517	8	8	---	---	intergenic	---
13	62538635	62538635	+	62538635	6	6	---	---	intergenic	---
13	62818838	62818838	+	62818838	6	6	ENSSSCG00000011524	+	intron	*CHL1*
13	62968647	62968653	+	62968647	4	10	ENSSSCG00000011524	+	intron	*CHL1*
13	62985992	62985992	+	62985992	3	3	ENSSSCG00000011524	+	3'UTR	*CHL1*
13	63706834	63706835	+	63706834	98	159	---	---	intergenic	---
13	63829750	63829766	+	63829750	3	6	---	---	intergenic	---
13	64035703	64035703	-	64035703	5	5	---	---	intergenic	---
13	64691566	64691566	+	64691566	15	15	---	---	intergenic	---
13	64848784	64848784	+	64848784	4	4	---	---	intergenic	---
13	65034162	65034163	-	65034163	13	17	---	---	intergenic	---
13	65036243	65036243	+	65036243	16	16	---	---	intergenic	---
13	65401952	65401952	+	65401952	3	3	---	---	intergenic	---
13	65748592	65748592	+	65748592	12	12	---	---	intergenic	---
13	65789689	65789689	+	65789689	4	4	---	---	intergenic	---
13	66015154	66015154	+	66015154	4	4	ENSSSCG00000011529	+	intron	*TRNT1*
13	66078354	66078355	+	66078354	7	13	ENSSSCG00000011529	+	3'UTR	*TRNT1*
13	66224246	66224252	+	66224246	32	50	---	---	intergenic	---
13	66503741	66503741	-	66503741	3	3	---	---	intergenic	---
13	66883540	66883540	+	66883540	4	4	XLOC_020467	+	predicted	---
13	67255363	67255366	-	67255364	41	64	ENSSSCG00000011532	-	3'UTR	*SUMF1*
13	67751797	67751797	-	67751797	3	3	---	---	intergenic	---
13	67837577	67837579	+	67837579	5	8	XLOC_020473	+	predicted	---
13	67976060	67976060	+	67976060	11	11	---	---	intergenic	---
13	68045546	68045548	+	68045546	36	53	XLOC_020474	+	predicted	---

13	68045640	68045685	+	68045657	23	40	XLOC_020474	+	predicted	---
13	68045812	68045819	+	68045819	7	10	XLOC_020474	+	predicted	---
13	68046308	68046319	+	68046310	10	25	XLOC_020474	+	predicted	---
13	68046369	68046372	+	68046369	4	7	XLOC_020474	+	predicted	---
13	68046462	68046465	+	68046464	15	29	XLOC_020474	+	predicted	---
13	68047450	68047453	+	68047453	7	19	XLOC_020474	+	predicted	---
13	68047633	68047633	+	68047633	3	3	XLOC_020474	+	predicted	---
13	68134895	68134896	+	68134896	9	12	XLOC_020476	+	predicted	---
13	68138461	68138466	+	68138464	32	57	XLOC_020476	+	predicted	---
13	68138573	68138573	+	68138573	6	6	XLOC_020476	+	predicted	---
13	68769079	68769079	+	68769079	4	4	---	---	intergenic	---
13	70681015	70681015	+	70681015	6	6	ENSSSCG00000023891	+	intron	*GRM7*
13	70762387	70762387	+	70762387	9	9	ENSSSCG00000023891	+	intron	*GRM7*
13	72140190	72140190	+	72140190	5	5	---	---	intergenic	---
13	72811890	72811890	-	72811890	4	4	ENSSSCG00000030273	-	3'UTR	---
13	72814714	72814717	-	72814715	13	25	ENSSSCG00000030273	-	3'UTR	---
13	72815313	72815330	-	72815313	3	6	ENSSSCG00000030273	-	3'UTR	---
13	72870310	72870310	+	72870310	6	6	ENSSSCG00000011542	+	intron	---
13	72876943	72876943	+	72876943	3	3	ENSSSCG00000011542	+	ORF	---
13	72923982	72923982	+	72923982	3	3	ENSSSCG00000011540	+	ORF	*SETD5*
13	73076679	73076679	+	73076679	5	5	ENSSSCG00000011551	+	ORF	*TADA3*
13	73079774	73079783	+	73079774	46	74	ENSSSCG00000011551	+	3'UTR	*TADA3*
13	73102658	73102658	-	73102658	3	3	XLOC_021828	-	predicted	---
13	73110031	73110031	-	73110031	5	5	ENSSSCG00000011548	-	3'UTR	*BRPF1*
13	73110136	73110136	-	73110136	3	3	ENSSSCG00000011548	-	3'UTR	*BRPF1*
13	73149545	73149546	-	73149545	15	21	XLOC_021831	-	predicted	---

13	73167277	73167291	+	73167291	10	45	XLOC_020494	+	predicted	---
13	73167344	73167348	+	73167344	5	8	XLOC_020494	+	predicted	---
13	73167876	73167882	+	73167879	270	383	XLOC_020494	+	predicted	---
13	73168685	73168685	+	73168685	6	6	XLOC_020494	+	predicted	---
13	73202080	73202080	-	73202080	5	5	XLOC_021832	-	predicted	---
13	73216902	73216905	-	73216902	26	42	ENSSSCG00000011557	-	3'UTR	*CIDE-C*
13	73233412	73233422	+	73233414	47	125	ENSSSCG00000011558	+	3'UTR	*JAGN1*
13	73279254	73279256	+	73279256	19	27	XLOC_020498	+	predicted	---
13	73293303	73293304	-	73293303	3	6	ENSSSCG00000011562	-	3'UTR	*EMC3*
							ENSSSCG00000011561	-	intron	*PRRT3*
13	73293945	73293952	-	73293946	22	37	ENSSSCG00000011562	-	3'UTR	*EMC3*
							ENSSSCG00000011561	-	intron	*PRRT3*
13	73294006	73294013	-	73294007	21	44	ENSSSCG00000011562	-	3'UTR	*EMC3*
							ENSSSCG00000011561	-	intron	*PRRT3*
13	73294225	73294252	-	73294249	94	221	ENSSSCG00000011562	-	3'UTR	*EMC3*
							ENSSSCG00000011561	-	intron	*PRRT3*
13	73318684	73318684	+	73318684	3	3	ENSSSCG00000011563	+	ORF	*FANCD2*
13	73380588	73380591	+	73380589	5	18	XLOC_020499	+	predicted	---
13	73380804	73380810	+	73380805	25	47	XLOC_020499	+	predicted	---
13	73381089	73381096	-	73381096	36	66	ENSSSCG00000011561	-	intron	*PRRT3*
13	73381991	73381991	-	73381991	3	3	ENSSSCG00000011561	-	intron	*PRRT3*
13	73396153	73396156	+	73396155	7	19	XLOC_020501	+	predicted	---
13	73396656	73396667	+	73396664	352	739	XLOC_020501	+	predicted	---
13	73396919	73396927	+	73396927	52	136	XLOC_020501	+	predicted	---
13	73414680	73414686	+	73414682	30	64	ENSSSCG00000011567	+	3'UTR	*VHL*
13	73436154	73436154	-	73436154	5	5	---	---	intergenic	---

13	73509353	73509353	-	73509353	3	3	XLOC_021839	-	predicted	---
13	73647788	73647793	-	73647789	40	113	ENSSSCG00000023084	-	intron	---
13	73810838	73810838	-	73810838	6	6	---	---	intergenic	---
13	74915738	74915738	+	74915738	4	4	ENSSSCG00000011575	+	3'UTR	*ATG7*
13	75062036	75062036	+	75062036	5	5	---	---	intergenic	---
13	75098318	75098319	-	75098318	24	33	---	---	intergenic	---
13	75098355	75098359	+	75098358	64	138	---	---	intergenic	---
13	75136050	75136052	-	75136052	10	17	ENSSSCG00000011578	-	3'UTR	*TAMM41*
13	75208645	75208645	-	75208645	3	3	---	---	intergenic	---
13	75720496	75720496	+	75720496	4	4	ENSSSCG00000011580	+	intron	*TSEN2*
13	75741419	75741419	-	75741419	3	3	XLOC_021856	-	predicted	---
13	75754533	75754540	+	75754540	4	10	---	---	intergenic	---
13	75838260	75838267	+	75838260	3	6	---	---	intergenic	---
13	75847252	75847272	+	75847252	45	82	ENSSSCG00000029877	+	3'UTR	*MKRN2*
13	75910732	75910732	+	75910732	3	3	---	---	intergenic	---
13	76056452	76056452	-	76056452	3	3	ENSSSCG00000027637	-	3'UTR	*RPL32*
13	76056775	76056775	+	76056775	3	3	ENSSSCG00000011582	+	3'UTR	---
13	76057442	76057446	-	76057443	17	29	ENSSSCG00000027637	-	3'UTR	*RPL32*
13	76057708	76057735	-	76057726	795	1627	ENSSSCG00000027637	-	3'UTR	*RPL32*
13	76061485	76061485	-	76061485	3	3	ENSSSCG00000027637	-	ORF	*RPL32*
13	76116224	76116239	+	76116224	3	6	ENSSSCG00000011589	+	intron	*IFT122*
13	76129328	76129329	+	76129328	11	14	ENSSSCG00000011589	+	3'UTR	*IFT122*
13	76175562	76175562	+	76175562	3	3	XLOC_020530	+	predicted	---
13	76179390	76179390	-	76179390	10	10	ENSSSCG00000011592	-	3'UTR	*PLXND1*
13	76266103	76266103	-	76266103	4	4	XLOC_021865	-	predicted	---
13	76268848	76268848	-	76268848	4	4	XLOC_021865	-	predicted	---

13	76353280	76353280	-	76353280	11	11	ENSSSCG00000011593	-	intron	*TMCC1*
13	76509490	76509490	-	76509490	3	3	---	---	intergenic	---
13	76745733	76745733	-	76745733	3	3	ENSSSCG00000029810	-	3'UTR	---
13	76748453	76748453	-	76748453	8	8	ENSSSCG00000029810	-	3'UTR	---
13	76826752	76826753	-	76826753	10	14	ENSSSCG00000011594	-	3'UTR	*NR2C2*
13	76847064	76847071	-	76847069	37	79	ENSSSCG00000011594	-	intron	*NR2C2*
13	76944232	76944232	-	76944232	6	6	XLOC_021872	-	predicted	---
13	77181254	77181256	+	77181255	29	39	XLOC_020535	+	predicted	---
13	77553403	77553403	-	77553403	5	5	XLOC_021877	-	predicted	---
13	77556344	77556344	-	77556344	4	4	---	---	intergenic	---
13	77556407	77556416	-	77556415	20	54	---	---	intergenic	---
13	77606249	77606257	+	77606252	11	23	XLOC_020538	+	predicted	---
13	77606486	77606496	+	77606488	8	18	XLOC_020538	+	predicted	---
13	77606603	77606603	+	77606603	3	3	XLOC_020538	+	predicted	---
13	77610039	77610048	+	77610048	5	8	XLOC_020538	+	predicted	---
13	77663932	77663940	+	77663932	4	8	---	---	intergenic	---
13	77723253	77723253	+	77723253	4	4	---	---	intergenic	---
13	77737212	77737217	-	77737212	13	25	XLOC_021880	-	predicted	---
13	77737331	77737340	-	77737332	7	24	ENSSSCG00000011601	-	3'UTR	*LSM3*
13	77794862	77794863	+	77794862	5	8	ENSSSCG00000011602	+	3'UTR	*XPC*
13	77795483	77795483	-	77795483	5	5	XLOC_021881	-	predicted	---
13	77887015	77887015	+	77887015	3	3	ENSSSCG00000011604	+	3'UTR	*CHCHD4*
13	77887495	77887497	+	77887496	30	42	ENSSSCG00000011604	+	3'UTR	*CHCHD4*
13	78256451	78256451	-	78256451	7	7	ENSSSCG00000011609	-	3'UTR	---
13	78331542	78331542	-	78331542	5	5	ENSSSCG00000011609	-	intron	---
13	78339590	78339590	-	78339590	3	3	ENSSSCG00000011609	-	intron	---

13	78557475	78557484	-	78557482	42	130	ENSSSCG00000011608	-	ORF	---
13	78558340	78558340	-	78558340	4	4	ENSSSCG00000011608	-	intron	---
13	78597873	78597875	-	78597873	13	17	---	---	intergenic	---
13	78704934	78704934	+	78704934	10	10	ENSSSCG00000011610	+	3'UTR	*NUP210*
13	78945022	78945027	-	78945022	5	9	ENSSSCG00000027812	-	3'UTR	*IQSEC1*
13	78945817	78945817	-	78945817	4	4	ENSSSCG00000027812	-	3'UTR	*IQSEC1*
13	79018692	79018697	+	79018697	33	37	ENSSSCG00000023354	+	3'UTR	*ACAD9*
13	79147011	79147011	-	79147011	4	4	---	---	intergenic	---
13	79155703	79155710	-	79155703	13	35	ENSSSCG00000029341	-	3'UTR	---
13	79155911	79155911	-	79155911	11	11	ENSSSCG00000029341	-	3'UTR	---
13	79273120	79273143	-	79273140	171	365	ENSSSCG00000011612	-	3'UTR	*RPN1*
13	79274374	79274374	-	79274374	3	3	ENSSSCG00000011612	-	ORF	*RPN1*
13	79410093	79410097	-	79410096	36	65	ENSSSCG00000011616	-	3'UTR	*COPG1*
13	79424938	79424979	+	79424979	6	19	XLOC_020558	+	predicted	---
13	79433153	79433157	-	79433154	32	69	XLOC_021895	-	predicted	---
13	79450128	79450137	-	79450131	23	50	ENSSSCG00000011615	-	3'UTR	*RAB7A*
13	79450544	79450551	-	79450546	10	20	ENSSSCG00000011615	-	3'UTR	*RAB7A*
13	79450819	79450823	-	79450822	13	19	ENSSSCG00000011615	-	3'UTR	*RAB7A*
13	79560459	79560475	+	79560470	241	634	XLOC_020560	+	predicted	---
13	79579437	79579440	-	79579437	21	27	---	---	intergenic	---
13	79587594	79587598	-	79587596	11	33	---	---	intergenic	---
13	79659058	79659063	-	79659062	23	46	XLOC_021902	-	predicted	---
13	79660818	79660825	-	79660819	4	10	---	---	intergenic	---
13	79727907	79727915	+	79727909	88	348	ENSSSCG00000021063	+	3'UTR	---
13	79735853	79735870	-	79735861	101	164	XLOC_021904	-	predicted	---
13	79737401	79737401	-	79737401	4	4	ENSSSCG00000011621	-	3'UTR	*SEC61A1*

13	79984369	79984375	-	79984370	19	43	ENSSSCG00000011623	-	3'UTR	*TPRA1*
13	80059176	80059176	+	80059176	4	4	ENSSSCG00000024134	+	intron	*MGLL*
13	80086911	80086914	+	80086912	9	21	ENSSSCG00000024134	+	3'UTR	*MGLL*
13	80091285	80091285	-	80091285	3	3	XLOC_021909	-	predicted	---
13	80148556	80148556	-	80148556	6	6	ENSSSCG00000011624	-	3'UTR	*MCM2*
13	80200889	80200890	+	80200890	8	11	XLOC_020569	+	predicted	---
13	80911724	80911761	+	80911730	24	55	XLOC_020573	+	predicted	---
13	80923396	80923419	+	80923401	136	482	XLOC_020574	+	predicted	---
13	80923468	80923477	+	80923470	45	132	XLOC_020574	+	predicted	---
13	80923532	80923554	+	80923553	12	38	XLOC_020574	+	predicted	---
13	80925716	80925722	-	80925717	16	32	ENSSSCG00000011625	-	3'UTR	*CHST13*
13	80955095	80955126	-	80955124	9	46	XLOC_021915	-	predicted	---
13	81038210	81038210	-	81038210	4	4	---	---	intergenic	---
13	81048275	81048275	-	81048275	3	3	---	---	intergenic	---
13	81091324	81091324	-	81091324	3	3	---	---	intergenic	---
13	81144386	81144387	-	81144386	104	112	---	---	intergenic	---
13	81191201	81191201	+	81191201	4	4	ENSSSCG00000011628	+	intron	*DNAJC13*
13	81278914	81278914	+	81278914	3	3	XLOC_020579	+	predicted	---
13	81279027	81279027	+	81279027	4	4	XLOC_020579	+	predicted	---
13	81279630	81279637	+	81279631	9	18	XLOC_020579	+	predicted	---
13	81305201	81305225	-	81305210	32	102	ENSSSCG00000011629	-	3'UTR	*ACAD11*
13	81349252	81349252	+	81349252	7	7	ENSSSCG00000011630	+	3'UTR	*CCRL1*
							ENSSSCG00000011630	+	ORF	---
13	81350290	81350290	+	81350290	4	4	XLOC_020582	+	predicted	---
13	81460962	81460962	+	81460962	16	16	---	---	intergenic	---
13	81539734	81539734	+	81539734	3	3	XLOC_020581	+	predicted	---

13	81539902	81539902	+	81539902	3	3	XLOC_020581	+	predicted	---
13	81544130	81544130	-	81544130	7	7	ENSSSCG00000011634	-	intron	---
13	81683768	81683768	-	81683768	4	4	---	---	intergenic	---
13	81684661	81684662	-	81684662	5	9	---	---	intergenic	---
13	82171499	82171508	+	82171508	7	13	ENSSSCG00000011637	+	intron	---
13	82229566	82229566	+	82229566	3	3	ENSSSCG00000021966	+	3'UTR	---
13	82238954	82238954	-	82238954	5	5	---	---	intergenic	---
13	82248929	82248929	-	82248929	3	3	---	---	intergenic	---
13	82277388	82277388	-	82277388	5	5	ENSSSCG00000027294	-	3'UTR	---
13	82278913	82278913	-	82278913	5	5	ENSSSCG00000027294	-	3'UTR	---
13	82279004	82279031	-	82279031	9	17	ENSSSCG00000027294	-	3'UTR	---
13	82279422	82279443	-	82279429	9	26	ENSSSCG00000027294	-	3'UTR	---
13	82370851	82370852	+	82370852	4	7	ENSSSCG00000028203	+	ORF	*ICA*
13	82385013	82385013	+	82385013	9	9	ENSSSCG00000028203	+	intron	*ICA*
13	82386447	82386447	+	82386447	3	3	ENSSSCG00000028203	+	ORF	*ICA*
13	82403090	82403090	+	82403090	3	3	ENSSSCG00000028203	+	ORF	*ICA*
13	82404019	82404019	+	82404019	3	3	ENSSSCG00000028203	+	ORF	*ICA*
13	82405390	82405395	+	82405392	176	382	ENSSSCG00000028203	+	3'UTR	*ICA*
13	82405506	82405507	+	82405506	3	6	XLOC_020590	+	predicted	---
13	82406471	82406471	+	82406471	10	10	XLOC_020590	+	predicted	---
13	82431154	82431158	+	82431154	14	17	ENSSSCG00000011640	+	ORF	*TF*
13	82431203	82431204	+	82431204	41	57	ENSSSCG00000011640	+	ORF	*TF*
13	82438283	82438299	+	82438286	15	41	ENSSSCG00000011640	+	ORF	*TF*
13	82440221	82440232	+	82440221	5	15	ENSSSCG00000011640	+	ORF	*TF*
13	82443890	82443890	+	82443890	3	3	ENSSSCG00000011640	+	ORF	*TF*
13	82444121	82444122	+	82444122	10	14	ENSSSCG00000011640	+	intron	*TF*

13	82448358	82448368	+	82448368	6	9	ENSSSCG00000011640	+	ORF	*TF*
13	82451099	82451099	+	82451099	8	8	ENSSSCG00000011640	+	ORF	*TF*
13	82455244	82455244	+	82455244	7	7	ENSSSCG00000011640	+	ORF	*TF*
13	82456783	82456800	+	82456799	1454	2666	ENSSSCG00000011640	+	intron	*TF*
13	82464174	82464178	+	82464174	4	10	ENSSSCG00000011640	+	ORF	*TF*
13	82464233	82464247	+	82464234	26	66	ENSSSCG00000011640	+	ORF	*TF*
13	82464290	82464308	+	82464291	16	38	ENSSSCG00000011640	+	ORF	*TF*
13	82468886	82468886	+	82468886	3	3	ENSSSCG00000011640	+	3'UTR	*TF*
13	82468985	82469018	+	82468999	1431	2540	ENSSSCG00000011640	+	3'UTR	*TF*
13	82469081	82469085	+	82469083	47	75	---	---	intergenic	---
13	82507469	82507469	+	82507469	3	3	XLOC_020592	+	predicted	---
13	82507813	82507813	-	82507813	3	3	XLOC_021923	-	predicted	---
13	82507934	82507935	+	82507934	18	21	XLOC_020592	+	predicted	---
13	82508610	82508621	+	82508614	28	55	XLOC_020592	+	predicted	---
13	82605343	82605348	-	82605347	4	14	XLOC_021926	-	predicted	---
13	82691892	82691892	+	82691892	4	4	---	---	intergenic	---
13	82780289	82780289	-	82780289	3	3	---	---	intergenic	---
13	82780313	82780314	+	82780313	5	8	---	---	intergenic	---
13	82824710	82824710	-	82824710	4	4	ENSSSCG00000011642	-	3'UTR	*RYK*
13	82825415	82825415	-	82825415	3	3	ENSSSCG00000011642	-	3'UTR	*RYK*
13	82989946	82989946	+	82989946	3	3	---	---	intergenic	---
13	83023026	83023026	-	83023026	3	3	---	---	intergenic	---
13	83051079	83051079	-	83051079	4	4	---	---	intergenic	---
13	83107309	83107309	-	83107309	3	3	---	---	intergenic	---
13	83126784	83126796	-	83126792	43	141	XLOC_021931	-	predicted	---
13	83171424	83171425	+	83171424	3	6	ENSSSCG00000011645	+	intron	*CEP63*

13	83172366	83172366	+	83172366	3	3	---	---	intergenic	---
13	83200725	83200725	+	83200725	3	3	---	---	intergenic	---
13	83234169	83234173	+	83234170	9	20	XLOC_020599	+	predicted	---
13	83234301	83234301	+	83234301	4	4	XLOC_020599	+	predicted	---
13	83321144	83321145	-	83321145	5	8	---	---	intergenic	---
13	83923781	83923781	+	83923781	3	3	---	---	intergenic	---
13	83934690	83934690	-	83934690	3	3	ENSSSCG00000028410	-	3'UTR	---
13	84052652	84052652	+	84052652	4	4	---	---	intergenic	---
13	84138710	84138710	+	84138710	7	7	XLOC_020605	+	predicted	---
13	84514963	84514963	+	84514963	9	9	XLOC_020607	+	predicted	---
13	84516767	84516767	+	84516767	6	6	XLOC_020607	+	predicted	---
13	84516944	84516952	+	84516944	3	6	XLOC_020607	+	predicted	---
13	84517741	84517741	+	84517741	7	7	XLOC_020607	+	predicted	---
13	84517840	84517840	-	84517840	3	3	XLOC_021940	-	predicted	---
13	84518369	84518377	-	84518377	7	10	XLOC_021940	-	predicted	---
13	84540926	84540926	-	84540926	14	14	ENSSSCG00000011650	-	intron	*MSL2*
13	84684629	84684642	+	84684635	54	98	ENSSSCG00000011653	+	3'UTR	*PCCB*
13	85374728	85374728	+	85374728	3	3	---	---	intergenic	---
13	85560447	85560452	+	85560452	11	14	ENSSSCG00000025701	+	intron	*NCK1*
13	85580441	85580441	+	85580441	7	7	ENSSSCG00000025701	+	3'UTR	*NCK1*
13	85580484	85580485	+	85580484	19	22	ENSSSCG00000025701	+	3'UTR	*NCK1*
13	85835627	85835627	-	85835627	4	4	---	---	intergenic	---
13	85970229	85970236	+	85970229	9	12	---	---	intergenic	---
13	86150720	86150720	+	86150720	3	3	XLOC_020618	+	predicted	---
13	86172700	86172700	-	86172700	4	4	---	---	intergenic	---
13	86443021	86443021	-	86443021	4	4	---	---	intergenic	---

13	86481366	86481367	+	86481366	3	6	ENSSSCG00000025785	+	intron	---
13	86502009	86502009	-	86502009	3	3	ENSSSCG00000024961	-	3'UTR	---
13	86560955	86560955	-	86560955	3	3	XLOC_021953	-	predicted	---
13	86594958	86594958	-	86594958	3	3	---	---	intergenic	---
13	86694110	86694110	+	86694110	4	4	ENSSSCG00000011658	+	3'UTR	---
13	86756004	86756015	-	86756004	14	17	XLOC_021955	-	predicted	---
13	86953944	86953950	-	86953950	8	11	ENSSSCG00000026746	-	intron	*CEP70*
13	86974083	86974090	-	86974090	15	19	ENSSSCG00000026746	-	ORF	*CEP70*
13	87000050	87000050	-	87000050	12	12	ENSSSCG00000026746	-	ORF	*CEP70*
13	87089447	87089447	-	87089447	3	3	ENSSSCG00000023814	-	ORF	*FAIM*
13	87096009	87096012	-	87096010	129	172	ENSSSCG00000021203	-	intron	---
13	87120888	87120888	-	87120888	3	3	ENSSSCG00000021203	-	intron	---
13	87239362	87239362	+	87239362	5	5	---	---	intergenic	---
13	87249977	87249977	+	87249977	3	3	---	---	intergenic	---
13	87584642	87584649	-	87584642	7	14	XLOC_021962	-	predicted	---
13	87584912	87584912	-	87584912	3	3	XLOC_021962	-	predicted	---
13	87893806	87893806	-	87893806	3	3	XLOC_021965	-	predicted	---
13	87894345	87894352	+	87894352	8	12	ENSSSCG00000011661	+	3'UTR	*MRPS22*
13	87894949	87894958	-	87894958	22	38	XLOC_021965	-	predicted	---
13	87895183	87895183	-	87895183	4	4	XLOC_021965	-	predicted	---
13	87896030	87896030	-	87896030	12	12	ENSSSCG00000011662	-	intron	*COPB2*
13	87897995	87897995	+	87897995	5	5	ENSSSCG00000011661	+	3'UTR	*MRPS22*
13	88029900	88029900	-	88029900	3	3	---	---	intergenic	---
13	88047844	88047845	-	88047845	16	23	ENSSSCG00000011664	-	3'UTR	*RBP1*
13	88261684	88261684	-	88261684	3	3	---	---	intergenic	---
13	88916417	88916421	+	88916417	3	6	---	---	intergenic	---

13	89005194	89005194	-	89005194	11	11	XLOC_021970	-	predicted	---
13	89377674	89377675	+	89377675	6	9	---	---	intergenic	---
13	89407574	89407575	+	89407575	12	15	XLOC_020645	+	predicted	---
13	89512413	89512413	+	89512413	3	3	---	---	intergenic	---
13	89548102	89548102	+	89548102	12	12	---	---	intergenic	---
13	89567593	89567593	-	89567593	3	3	---	---	intergenic	---
13	89597557	89597557	-	89597557	3	3	---	---	intergenic	---
13	89634060	89634060	+	89634060	8	8	XLOC_020646	+	predicted	---
13	89667351	89667351	-	89667351	3	3	---	---	intergenic	---
13	89769846	89769847	+	89769846	4	8	ENSSSCG00000028303	+	3'UTR	*SPSB4*
13	89884930	89884930	+	89884930	4	4	ENSSSCG00000011670	+	3'UTR	*PXYLP1*
13	89924926	89924931	+	89924931	8	18	XLOC_020650	+	predicted	---
13	89979791	89979792	+	89979791	15	20	---	---	intergenic	---
13	90001825	90001825	+	90001825	7	7	---	---	intergenic	---
13	90036845	90036845	+	90036845	3	3	XLOC_020650	+	predicted	---
13	90036902	90036903	+	90036902	5	8	XLOC_020650	+	predicted	---
13	90229796	90229796	-	90229796	3	3	---	---	intergenic	---
13	90284369	90284369	-	90284369	5	5	---	---	intergenic	---
13	90384535	90384535	+	90384535	3	3	ENSSSCG00000029774	+	3'UTR	*RNF7*
13	90384741	90384743	+	90384742	20	43	ENSSSCG00000029774	+	3'UTR	*RNF7*
13	90385387	90385389	+	90385389	11	22	ENSSSCG00000029774	+	3'UTR	*RNF7*
13	90527610	90527610	-	90527610	3	3	---	---	intergenic	---
13	90553459	90553462	-	90553462	6	10	XLOC_021982	-	predicted	---
13	90554318	90554329	-	90554329	22	37	XLOC_021982	-	predicted	---
13	90585043	90585043	+	90585043	3	3	XLOC_020659	+	predicted	---
13	90666088	90666088	+	90666088	3	3	---	---	intergenic	---

13	90704078	90704081	-	90704078	5	8	---	---	intergenic	---
13	90749588	90749588	-	90749588	4	4	XLOC_021984	-	predicted	---
13	90866761	90866761	-	90866761	6	6	XLOC_021985	-	predicted	---
13	90875493	90875494	-	90875493	14	21	ENSSSCG00000011678	-	intron	*XRN1*
13	90909958	90909958	-	90909958	7	7	ENSSSCG00000011678	-	ORF	*XRN1*
13	90946355	90946355	-	90946355	4	4	ENSSSCG00000011678	-	intron	*XRN1*
13	90949143	90949143	-	90949143	3	3	ENSSSCG00000011678	-	intron	*XRN1*
13	90958799	90958799	-	90958799	4	4	ENSSSCG00000011678	-	intron	*XRN1*
13	90966561	90966561	-	90966561	3	3	ENSSSCG00000011678	-	intron	*XRN1*
13	90978662	90978662	-	90978662	4	4	XLOC_021986	-	predicted	---
13	91080329	91080334	-	91080334	4	7	ENSSSCG00000011679	-	ORF	*ATR*
13	91094802	91094802	+	91094802	5	5	---	---	intergenic	---
13	91246256	91246258	+	91246256	4	8	ENSSSCG00000011680	+	3'UTR	*PLS1*
							ENSSSCG00000011681	+	intron	*TRPC1*
13	91433917	91433917	-	91433917	3	3	XLOC_021990	-	predicted	---
13	91440942	91440944	-	91440944	5	8	XLOC_021990	-	predicted	---
13	91471193	91471193	+	91471193	3	3	---	---	intergenic	---
13	91495760	91495760	+	91495760	18	18	ENSSSCG00000011684	+	intron	*U2SURP*
13	91524105	91524105	+	91524105	3	3	ENSSSCG00000011684	+	intron	*U2SURP*
13	91530721	91530721	+	91530721	3	3	ENSSSCG00000011684	+	3'UTR	*U2SURP*
13	91534375	91534375	+	91534375	3	3	ENSSSCG00000011684	+	3'UTR	*U2SURP*
13	91734517	91734517	-	91734517	3	3	XLOC_021993	-	predicted	---
13	91841976	91841976	-	91841976	5	5	ENSSSCG00000022733	-	intron	---
13	91998995	91998995	+	91998995	3	3	---	---	intergenic	---
13	92361170	92361170	-	92361170	4	4	---	---	intergenic	---
13	92370410	92370411	-	92370410	6	9	XLOC_021995	-	predicted	---

13	92991209	92991210	-	92991210	12	20	---	---	intergenic	---
13	93082800	93082800	-	93082800	3	3	---	---	intergenic	---
13	93858174	93858174	+	93858174	4	4	---	---	intergenic	---
13	93978176	93978176	+	93978176	7	7	---	---	intergenic	---
13	94057412	94057412	+	94057412	3	3	ENSSSCG00000011688	+	ORF	---
13	94125972	94125972	-	94125972	3	3	---	---	intergenic	---
13	94148555	94148555	+	94148555	3	3	---	---	intergenic	---
13	94320714	94320714	-	94320714	3	3	XLOC_022001	-	predicted	---
13	94547472	94547472	+	94547472	3	3	---	---	intergenic	---
13	94749707	94749710	+	94749707	8	14	---	---	intergenic	---
13	95866148	95866177	-	95866148	3	6	---	---	intergenic	---
13	96400588	96400588	+	96400588	3	3	---	---	intergenic	---
13	96635571	96635571	+	96635571	3	3	---	---	intergenic	---
13	96642460	96642460	+	96642460	17	17	---	---	intergenic	---
13	96942934	96942934	+	96942934	4	4	ENSSSCG00000011695	+	intron	*AGTR1*
13	96977041	96977041	+	96977041	3	3	ENSSSCG00000011695	+	ORF	---
13	96977873	96977874	+	96977874	4	7	ENSSSCG00000011695	+	3'UTR	*AGTR1*
							ENSSSCG00000011695	+	ORF	---
13	96978195	96978195	+	96978195	3	3	ENSSSCG00000011695	+	3'UTR	*AGTR1*
							ENSSSCG00000011695	+	ORF	---
13	96978418	96978420	+	96978418	12	19	XLOC_020680	+	predicted	---
13	97171843	97171843	-	97171843	4	4	---	---	intergenic	---
13	97171850	97171864	+	97171855	18	51	---	---	intergenic	---
13	97174892	97174892	-	97174892	4	4	---	---	intergenic	---
13	97233279	97233279	+	97233279	5	5	ENSSSCG00000011698	+	intron	*GYG1*
13	97243870	97243870	+	97243870	3	3	ENSSSCG00000011698	+	intron	*GYG1*

13	97253057	97253063	+	97253062	35	61	ENSSSCG00000011698	+	3'UTR	*GYG1*
13	97256458	97256460	-	97256460	8	17	ENSSSCG00000025293	-	3'UTR	*HLTF*
13	97260810	97260818	-	97260818	13	20	ENSSSCG00000025293	-	intron	*HLTF*
13	97302625	97302625	-	97302625	7	7	ENSSSCG00000025293	-	ORF	*HLTF*
13	97307296	97307296	-	97307296	6	6	ENSSSCG00000025293	-	ORF	*HLTF*
13	97312432	97312432	-	97312432	3	3	ENSSSCG00000025293	-	intron	*HLTF*
13	97400066	97400067	-	97400067	5	9	ENSSSCG00000011700	-	intron	*CP*
13	97401328	97401354	-	97401351	127	295	ENSSSCG00000011700	-	intron	*CP*
13	97401419	97401419	-	97401419	6	6	ENSSSCG00000011700	-	intron	*CP*
13	97401478	97401482	-	97401482	4	10	ENSSSCG00000011700	-	3'UTR	*CP*
							ENSSSCG00000011700	-	intron	*CP*
13	97402169	97402173	-	97402173	10	19	ENSSSCG00000011700	-	3'UTR	*CP*
							ENSSSCG00000011700	-	intron	*CP*
13	97402363	97402369	-	97402363	13	32	ENSSSCG00000011700	-	3'UTR	*CP*
							ENSSSCG00000011700	-	intron	*CP*
13	97402470	97402522	-	97402470	15	41	ENSSSCG00000011700	-	3'UTR	*CP*
							ENSSSCG00000011700	-	intron	*CP*
13	97402508	97402508	+	97402508	10	10	XLOC_020684	+	predicted	---
13	97405878	97405878	-	97405878	16	16	ENSSSCG00000011700	-	intron	*CP*
13	97405962	97405962	-	97405962	5	5	ENSSSCG00000011700	-	intron	*CP*
13	97407699	97407699	-	97407699	5	5	---	---	intergenic	---
13	97417156	97417156	-	97417156	4	4	ENSSSCG00000011700	-	intron	*CP*
							ENSSSCG00000011700	-	ORF	*CP*
13	97431991	97431991	-	97431991	5	5	ENSSSCG00000011700	-	intron	*CP*
13	97435222	97435228	-	97435222	3	6	ENSSSCG00000011700	-	intron	*CP*
13	97439543	97439577	-	97439543	4	7	ENSSSCG00000011700	-	intron	*CP*

13	97444277	97444296	-	97444280	83	270	ENSSSCG00000011700	-	intron	*CP*
13	97444337	97444339	-	97444338	39	52	ENSSSCG00000011700	-	intron	*CP*
13	97455397	97455397	-	97455397	12	12	---	---	intergenic	---
13	97521866	97521866	-	97521866	3	3	XLOC_022013	-	predicted	---
13	97594583	97594590	-	97594583	5	8	XLOC_022015	-	predicted	---
13	97595655	97595657	-	97595656	30	41	XLOC_022015	-	predicted	---
13	97686564	97686564	+	97686564	3	3	XLOC_020687	+	predicted	---
13	97733853	97733853	+	97733853	4	4	XLOC_020688	+	predicted	---
13	97733916	97733916	+	97733916	4	4	XLOC_020688	+	predicted	---
13	97734003	97734015	+	97734008	21	48	XLOC_020688	+	predicted	---
13	97734191	97734195	+	97734191	3	6	XLOC_020688	+	predicted	---
13	97746663	97746663	-	97746663	6	6	XLOC_022016	-	predicted	---
13	97768848	97768848	-	97768848	9	9	ENSSSCG00000011704	-	intron	*WWTR1*
13	97773050	97773050	-	97773050	3	3	ENSSSCG00000011704	-	intron	*WWTR1*
13	97892315	97892315	-	97892315	5	5	ENSSSCG00000011704	-	intron	*WWTR1*
13	98017390	98017404	-	98017397	17	32	ENSSSCG00000011705	-	3'UTR	*COMMD2*
13	98017590	98017591	-	98017590	7	11	ENSSSCG00000011705	-	3'UTR	*COMMD2*
13	98113432	98113432	+	98113432	4	4	ENSSSCG00000027459	+	intron	*RNF13*
13	98749113	98749113	+	98749113	3	3	ENSSSCG00000011708	+	intron	*TSC22D2*
13	98761482	98761482	+	98761482	9	9	XLOC_020696	+	predicted	---
13	98825369	98825377	-	98825376	53	100	XLOC_022023	-	predicted	---
13	98825908	98825925	-	98825918	12	41	XLOC_022023	-	predicted	---
13	98826818	98826824	-	98826822	11	21	XLOC_022023	-	predicted	---
13	98827633	98827644	-	98827634	35	98	XLOC_022023	-	predicted	---
13	98827762	98827768	-	98827768	12	36	XLOC_022023	-	predicted	---

13	98848272	98848272	-	98848272	3	3	ENSSSCG00000011710	-	intron	*EIF2A*
							ENSSSCG00000011710	-	ORF	*EIF2A*
13	98859099	98859099	-	98859099	12	12	ENSSSCG00000011710	-	ORF	*EIF2A*
13	98969333	98969333	+	98969333	4	4	ENSSSCG00000022706	+	intron	*ERICH6*
13	98992858	98992858	+	98992858	3	3	ENSSSCG00000022706	+	ORF	*ERICH6*
13	99019946	99019953	-	99019946	5	14	ENSSSCG00000027598	-	3'UTR	*SELT*
13	99020659	99020659	-	99020659	3	3	ENSSSCG00000027598	-	3'UTR	*SELT*
13	99021171	99021172	-	99021171	7	11	ENSSSCG00000027598	-	3'UTR	*SELT*
13	99022619	99022629	-	99022625	22	61	ENSSSCG00000027598	-	3'UTR	*SELT*
13	99023014	99023014	-	99023014	12	12	ENSSSCG00000027598	-	3'UTR	*SELT*
13	99023087	99023087	-	99023087	11	11	ENSSSCG00000027598	-	3'UTR	*SELT*
13	99058686	99058692	-	99058686	16	25	XLOC_022026	-	predicted	---
13	99076465	99076465	-	99076465	8	8	ENSSSCG00000024295	-	3'UTR	*SIAH2*
13	99206141	99206142	+	99206142	20	23	---	---	intergenic	---
13	99536784	99536790	+	99536784	17	20	---	---	intergenic	---
13	99604551	99604551	-	99604551	3	3	XLOC_022031	-	predicted	---
13	100027861	100027863	-	100027861	8	16	XLOC_022036	-	predicted	---
13	100212892	100212892	-	100212892	3	3	---	---	intergenic	---
13	100501268	100501268	+	100501268	3	3	---	---	intergenic	---
13	100741200	100741225	+	100741223	30	72	ENSSSCG00000011720	+	3'UTR	*MBNL1*
13	100890743	100890758	-	100890750	31	150	---	---	intergenic	---
13	101034542	101034542	+	101034542	3	3	XLOC_020714	+	predicted	---
13	101038359	101038359	+	101038359	4	4	XLOC_020714	+	predicted	---
13	101150443	101150443	+	101150443	3	3	---	---	intergenic	---
13	101209590	101209590	+	101209590	3	3	---	---	intergenic	---
13	101281069	101281069	-	101281069	6	6	---	---	intergenic	---

13	101371667	101371667	-	101371667	3	3	---	---	intergenic	---
13	101411897	101411897	-	101411897	4	4	XLOC_022041	-	predicted	---
13	101723275	101723276	+	101723275	34	37	---	---	intergenic	---
13	101798654	101798654	-	101798654	16	16	---	---	intergenic	---
13	102369891	102369892	-	102369892	10	14	ENSSSCG00000024916	-	intron	*DHX36*
13	102437930	102437930	+	102437930	7	7	---	---	intergenic	---
13	102619350	102619350	+	102619350	3	3	---	---	intergenic	---
13	102738244	102738244	+	102738244	3	3	---	---	intergenic	---
13	103128283	103128283	+	103128283	8	8	ENSSSCG00000011723	+	3'UTR	*MME*
							ENSSSCG00000011723	+	ORF	*MME*
13	103174385	103174385	-	103174385	3	3	---	---	intergenic	---
13	103254406	103254406	-	103254406	31	31	XLOC_022047	-	predicted	---
13	103285142	103285142	+	103285142	4	4	---	---	intergenic	---
13	103294425	103294425	+	103294425	3	3	---	---	intergenic	---
13	103616136	103616141	-	103616141	4	7	---	---	intergenic	---
13	103665494	103665494	+	103665494	3	3	XLOC_020726	+	predicted	---
13	103667886	103667886	+	103667886	4	4	---	---	intergenic	---
13	103676158	103676158	+	103676158	4	4	XLOC_020726	+	predicted	---
13	103681364	103681368	-	103681364	8	13	ENSSSCG00000021495	-	3'UTR	*C3orf33*
13	103718457	103718457	-	103718457	9	9	ENSSSCG00000025316	-	3'UTR	*SLC33A1*
13	103795468	103795468	+	103795468	3	3	ENSSSCG00000028855	+	intron	---
13	103799018	103799024	+	103799024	22	31	ENSSSCG00000028855	+	intron	---
13	103845255	103845255	-	103845255	4	4	---	---	intergenic	---
13	103856561	103856561	+	103856561	5	5	---	---	intergenic	---
13	103869557	103869562	-	103869559	97	169	XLOC_022050	-	predicted	---
13	104028192	104028199	-	104028199	8	21	---	---	intergenic	---

13	104402071	104402071	+	104402071	6	6	ENSSSCG00000025810	+	intron	*KCNAB1*
13	104483984	104484011	-	104484009	42	142	ENSSSCG00000030567	-	3'UTR	*SSR3*
13	104484277	104484277	-	104484277	3	3	ENSSSCG00000030567	-	3'UTR	*SSR3*
13	104484318	104484323	-	104484320	222	252	ENSSSCG00000030567	-	3'UTR	*SSR3*
13	104484352	104484396	-	104484381	132	308	ENSSSCG00000030567	-	3'UTR	*SSR3*
13	104484473	104484473	-	104484473	12	12	ENSSSCG00000030567	-	3'UTR	*SSR3*
13	104491399	104491399	-	104491399	5	5	ENSSSCG00000030567	-	ORF	*SSR3*
13	104497760	104497760	-	104497760	3	3	XLOC_022053	-	predicted	---
13	105083567	105083567	+	105083567	5	5	XLOC_020738	+	predicted	---
13	105144753	105144753	-	105144753	14	14	ENSSSCG00000029352	-	3'UTR	*CCNL1*
13	105148429	105148429	-	105148429	5	5	ENSSSCG00000029352	-	intron	*CCNL1*
13	105153880	105153880	-	105153880	3	3	ENSSSCG00000029352	-	ORF	*CCNL1*
13	105166282	105166282	-	105166282	4	4	---	---	intergenic	---
13	105354088	105354088	-	105354088	4	4	ENSSSCG00000011726	-	intron	*VEPH1*
13	105604654	105604654	+	105604654	4	4	---	---	intergenic	---
13	106687086	106687086	+	106687086	3	3	ENSSSCG00000020919	+	ORF	*MLF1*
13	106688346	106688349	+	106688349	14	25	ENSSSCG00000020919	+	ORF	*MLF1*
13	106696729	106696741	+	106696739	241	536	ENSSSCG00000020919	+	3'UTR	*MLF1*
13	106696918	106696927	+	106696919	171	283	ENSSSCG00000020919	+	3'UTR	*MLF1*
13	106709363	106709363	+	106709363	10	10	ENSSSCG00000025651	+	intron	*GFM1*
13	106728206	106728209	-	106728208	44	66	ENSSSCG00000020984	-	3'UTR	*LXN*
13	106728270	106728270	-	106728270	10	10	ENSSSCG00000020984	-	3'UTR	*LXN*
13	106748619	106748621	+	106748620	8	17	ENSSSCG00000025651	+	3'UTR	*GFM1*
13	106754585	106754586	-	106754586	11	15	XLOC_022064	-	predicted	---
13	106755370	106755382	-	106755375	4	10	ENSSSCG00000028623	-	3'UTR	*RARRES1*
13	107049828	107049834	+	107049834	32	39	XLOC_020751	+	predicted	---

13	107334429	107334429	+	107334429	5	5	---	---	intergenic	---
13	107480355	107480355	-	107480355	3	3	---	---	intergenic	---
13	107535722	107535722	-	107535722	3	3	---	---	intergenic	---
13	107966175	107966177	+	107966175	3	6	ENSSSCG00000011729	+	3'UTR	*IQCJ-SCHIP1*
13	108078864	108078867	-	108078866	7	19	ENSSSCG00000027790	-	3'UTR	---
13	108173099	108173099	+	108173099	4	4	---	---	intergenic	---
13	108326291	108326291	-	108326291	3	3	---	---	intergenic	---
13	108359824	108359824	-	108359824	4	4	ENSSSCG00000030666	-	intron	---
13	108417570	108417570	+	108417570	8	8	ENSSSCG00000011731	+	intron	*SMC4*
13	108419573	108419573	+	108419573	3	3	ENSSSCG00000011731	+	ORF	*SMC4*
13	108421282	108421282	+	108421282	44	44	ENSSSCG00000011731	+	intron	*SMC4*
13	108422084	108422084	+	108422084	4	4	XLOC_020759	+	predicted	---
13	108422230	108422236	+	108422233	17	33	XLOC_020759	+	predicted	---
13	108425889	108425889	-	108425889	6	6	ENSSSCG00000011732	-	3'UTR	---
13	108426262	108426262	-	108426262	3	3	ENSSSCG00000011732	-	ORF	---
13	108437899	108437899	-	108437899	3	3	---	---	intergenic	---
13	108491455	108491455	+	108491455	3	3	ENSSSCG00000011733	+	ORF	*IFT80*
13	108497966	108497966	+	108497966	3	3	ENSSSCG00000011733	+	intron	*IFT80*
13	108541372	108541372	-	108541372	6	6	---	---	intergenic	---
13	108541965	108541965	-	108541965	3	3	---	---	intergenic	---
13	108754217	108754217	+	108754217	3	3	---	---	intergenic	---
13	108851454	108851454	-	108851454	15	15	XLOC_022076	-	predicted	---
13	109042305	109042305	+	109042305	6	6	ENSSSCG00000023434	+	intron	*PPM1L*
13	109148491	109148491	+	109148491	3	3	XLOC_020764	+	predicted	---
13	109153091	109153093	+	109153092	7	15	XLOC_020764	+	predicted	---
13	109157527	109157527	+	109157527	6	6	XLOC_020764	+	predicted	---

13	109334344	109334345	+	109334344	3	6	ENSSSCG00000022952	+	intron	*NMD3*
13	109338753	109338757	+	109338755	17	36	ENSSSCG00000022952	+	intron	*NMD3*
13	109400158	109400158	-	109400158	5	5	ENSSSCG00000022248	-	intron	*OTOL1*
13	109582410	109582411	-	109582410	46	50	---	---	intergenic	---
13	109590367	109590367	-	109590367	3	3	---	---	intergenic	---
13	109591613	109591613	+	109591613	6	6	---	---	intergenic	---
13	109711633	109711633	+	109711633	3	3	---	---	intergenic	---
13	109877113	109877114	-	109877114	12	16	---	---	intergenic	---
13	110799436	110799436	+	110799436	4	4	---	---	intergenic	---
13	110871274	110871274	+	110871274	3	3	---	---	intergenic	---
13	110959634	110959634	+	110959634	3	3	---	---	intergenic	---
13	111050480	111050498	+	111050492	25	88	XLOC_020770	+	predicted	---
13	111111044	111111047	+	111111045	44	81	---	---	intergenic	---
13	111251305	111251306	+	111251306	13	20	---	---	intergenic	---
13	111505239	111505239	-	111505239	4	4	---	---	intergenic	---
13	112171525	112171525	-	112171525	7	7	---	---	intergenic	---
13	112171555	112171555	+	112171555	3	3	---	---	intergenic	---
13	112265172	112265172	+	112265172	3	3	---	---	intergenic	---
13	112599006	112599006	-	112599006	8	8	---	---	intergenic	---
13	112743562	112743562	+	112743562	3	3	ENSSSCG00000021108	+	intron	---
13	114359264	114359264	+	114359264	3	3	---	---	intergenic	---
13	114708584	114708592	-	114708585	7	17	XLOC_022089	-	predicted	---
13	114719152	114719154	-	114719153	11	21	ENSSSCG00000023393	-	ORF	*ZBBX*
13	114768544	114768544	-	114768544	5	5	ENSSSCG00000023393	-	ORF	*ZBBX*
13	115057316	115057320	+	115057318	31	61	---	---	intergenic	---
13	115067293	115067293	-	115067293	12	12	---	---	intergenic	---

13	115084116	115084134	-	115084116	3	6	ENSSSCG00000011739	-	intron	*PDCD10*
13	115199341	115199341	+	115199341	5	5	ENSSSCG00000011740	+	3'UTR	*SERPINI1*
13	115241865	115241868	-	115241865	5	13	XLOC_022092	-	predicted	---
13	115300313	115300313	+	115300313	7	7	XLOC_020777	+	predicted	---
13	115346088	115346088	-	115346088	4	4	---	---	intergenic	---
13	115370018	115370018	-	115370018	7	7	---	---	intergenic	---
13	115375510	115375525	-	115375511	27	71	ENSSSCG00000011741	-	3'UTR	*GOLIM4*
13	115377007	115377014	-	115377013	14	26	ENSSSCG00000011741	-	3'UTR	*GOLIM4*
13	115377543	115377546	-	115377545	8	19	ENSSSCG00000011741	-	intron	*GOLIM4*
13	115390561	115390564	-	115390561	14	19	ENSSSCG00000011741	-	ORF	*GOLIM4*
13	115391029	115391029	-	115391029	3	3	ENSSSCG00000011741	-	ORF	*GOLIM4*
13	115401104	115401104	-	115401104	7	7	ENSSSCG00000011741	-	ORF	*GOLIM4*
13	116403972	116403973	+	116403972	41	73	---	---	intergenic	---
13	116434571	116434571	-	116434571	3	3	---	---	intergenic	---
13	116692746	116692747	+	116692746	8	16	---	---	intergenic	---
13	116855047	116855047	-	116855047	3	3	---	---	intergenic	---
13	117156393	117156498	-	117156468	25	188	XLOC_022100	-	predicted	---
13	117156556	117156589	-	117156556	25	37	XLOC_022100	-	predicted	---
13	117156833	117156837	-	117156833	4	7	ENSSSCG00000024585	-	ORF	*ACTRT3*
13	117176432	117176433	+	117176433	11	14	ENSSSCG00000026829	+	3'UTR	*MYNN*
13	117176482	117176487	+	117176485	5	11	ENSSSCG00000026829	+	3'UTR	*MYNN*
13	117186299	117186301	-	117186300	13	20	XLOC_022101	-	predicted	---
13	117186356	117186362	-	117186362	13	16	XLOC_022101	-	predicted	---
13	117186439	117186439	-	117186439	4	4	XLOC_022101	-	predicted	---
13	117349494	117349494	+	117349494	3	3	---	---	intergenic	---
13	117524264	117524264	-	117524264	6	6	---	---	intergenic	---

13	117587321	117587321	-	117587321	3	3	---	---	intergenic	---
13	117814133	117814135	-	117814133	6	10	---	---	intergenic	---
13	117937085	117937085	+	117937085	3	3	ENSSSCG00000011746	+	3'UTR	*SKIL*
13	117940405	117940409	+	117940405	21	26	ENSSSCG00000011746	+	3'UTR	*SKIL*
13	117971350	117971355	+	117971353	81	158	ENSSSCG00000011747	+	3'UTR	*CLDN11*
13	118185770	118185770	+	118185770	3	3	---	---	intergenic	---
13	118185825	118185825	+	118185825	3	3	---	---	intergenic	---
13	118530693	118530693	-	118530693	8	8	XLOC_022112	-	predicted	---
13	118532256	118532259	-	118532257	35	46	XLOC_022112	-	predicted	---
13	118532501	118532508	-	118532508	10	28	XLOC_022112	-	predicted	---
13	118532662	118532662	-	118532662	3	3	XLOC_022112	-	predicted	---
13	118534662	118534662	-	118534662	3	3	XLOC_022112	-	predicted	---
13	118534997	118534998	-	118534997	9	12	XLOC_022112	-	predicted	---
13	118535244	118535244	-	118535244	3	3	XLOC_022112	-	predicted	---
13	118553740	118553740	-	118553740	3	3	ENSSSCG00000020927	-	ORF	*SLC2A2*
13	118585749	118585749	-	118585749	3	3	XLOC_022114	-	predicted	---
13	118817882	118817882	-	118817882	4	4	---	---	intergenic	---
13	118911219	118911219	-	118911219	5	5	---	---	intergenic	---
13	119150145	119150147	-	119150145	3	9	ENSSSCG00000011750	-	intron	*PLD1*
13	119489515	119489515	+	119489515	3	3	---	---	intergenic	---
13	119538022	119538022	+	119538022	3	3	---	---	intergenic	---
13	119627536	119627536	-	119627536	14	14	---	---	intergenic	---
13	119627763	119627763	-	119627763	3	3	---	---	intergenic	---
13	119778271	119778295	-	119778272	18	29	---	---	intergenic	---
13	119779502	119779502	-	119779502	3	3	ENSSSCG00000011752	-	3'UTR	---
13	119782554	119782554	-	119782554	3	3	ENSSSCG00000011752	-	3'UTR	---

13	119782716	119782719	-	119782719	50	73	ENSSSCG00000011752	-	3'UTR	---
13	119844284	119844288	+	119844284	3	6	---	---	intergenic	---
13	119878940	119878940	+	119878940	3	3	ENSSSCG00000020906	+	intron	*TNFSF10*
13	119881464	119881465	+	119881465	13	19	ENSSSCG00000020906	+	3'UTR	*TNFSF10*
							ENSSSCG00000020906	+	ORF	---
13	119883883	119883884	+	119883884	7	10	ENSSSCG00000020906	+	3'UTR	*TNFSF10*
							ENSSSCG00000020906	+	ORF	---
13	119883962	119883962	+	119883962	25	25	XLOC_020802	+	predicted	---
13	120071204	120071205	-	120071204	11	18	---	---	intergenic	---
13	120421195	120421195	+	120421195	4	4	---	---	intergenic	---
13	121572870	121572870	+	121572870	3	3	---	---	intergenic	---
13	122131162	122131162	-	122131162	5	5	---	---	intergenic	---
13	122131414	122131414	+	122131414	3	3	---	---	intergenic	---
13	122131524	122131525	+	122131525	48	58	---	---	intergenic	---
13	122140495	122140495	+	122140495	4	4	---	---	intergenic	---
13	122281603	122281603	+	122281603	3	3	---	---	intergenic	---
13	122400417	122400417	-	122400417	3	3	---	---	intergenic	---
13	123043583	123043584	-	123043584	12	15	---	---	intergenic	---
13	123269123	123269123	+	123269123	4	4	---	---	intergenic	---
13	123357100	123357100	-	123357100	4	4	---	---	intergenic	---
13	123843703	123843703	+	123843703	4	4	---	---	intergenic	---
13	124133031	124133031	-	124133031	3	3	---	---	intergenic	---
13	124258144	124258144	-	124258144	16	16	XLOC_022126	-	predicted	---
13	124571459	124571459	-	124571459	3	3	---	---	intergenic	---
13	124728085	124728085	+	124728085	7	7	---	---	intergenic	---
13	124738423	124738423	+	124738423	3	3	---	---	intergenic	---

13	124788284	124788284	+	124788284	31	31	---	---	intergenic	---
13	124803089	124803089	-	124803089	5	5	---	---	intergenic	---
13	124806295	124806295	-	124806295	10	10	---	---	intergenic	---
13	124895714	124895714	+	124895714	3	3	---	---	intergenic	---
13	125447021	125447021	-	125447021	3	3	XLOC_022128	-	predicted	---
13	125466983	125466983	-	125466983	5	5	---	---	intergenic	---
13	125725763	125725763	+	125725763	9	9	---	---	intergenic	---
13	125731292	125731292	+	125731292	3	3	---	---	intergenic	---
13	126183187	126183189	+	126183188	9	15	---	---	intergenic	---
13	126280509	126280510	+	126280509	3	6	XLOC_020824	+	predicted	---
13	126430854	126430854	-	126430854	3	3	ENSSSCG00000030413	-	intron	*PIK3CA*
13	126464851	126464851	-	126464851	3	3	---	---	intergenic	---
13	126464917	126464917	-	126464917	3	3	---	---	intergenic	---
13	126497388	126497388	+	126497388	4	4	---	---	intergenic	---
13	126596431	126596431	-	126596431	6	6	---	---	intergenic	---
13	126617724	126617724	-	126617724	4	4	---	---	intergenic	---
13	126721729	126721732	+	126721731	23	48	ENSSSCG00000011762	+	3'UTR	*ACTL6A*
13	126721987	126721987	-	126721987	11	11	ENSSSCG00000011763	-	3'UTR	*MRPL47*
13	126722102	126722102	-	126722102	4	4	ENSSSCG00000011763	-	3'UTR	*MRPL47*
13	126765641	126765647	+	126765647	8	11	ENSSSCG00000011764	+	3'UTR	*NDUFB5*
13	126765694	126765701	+	126765697	96	168	ENSSSCG00000011764	+	3'UTR	*NDUFB5*
13	126913119	126913119	+	126913119	3	3	ENSSSCG00000011765	+	3'UTR	---
13	126913212	126913212	+	126913212	3	3	ENSSSCG00000011765	+	3'UTR	---
13	126917846	126917846	+	126917846	4	4	ENSSSCG00000011765	+	3'UTR	---
13	127277045	127277045	+	127277045	3	3	---	---	intergenic	---
13	127543651	127543651	-	127543651	7	7	---	---	intergenic	---

13	127566852	127566854	+	127566852	8	14	---	---	intergenic	---
13	127576424	127576424	-	127576424	3	3	---	---	intergenic	---
13	127802840	127802840	-	127802840	3	3	XLOC_022141	-	predicted	---
13	127811682	127811682	+	127811682	7	7	XLOC_020835	+	predicted	---
13	127812737	127812738	+	127812737	4	7	---	---	intergenic	---
13	127823664	127823664	+	127823664	4	4	ENSSSCG00000011767	+	intron	*TTC14*
13	127826010	127826010	+	127826010	5	5	XLOC_020836	+	predicted	---
13	127828112	127828112	-	127828112	3	3	XLOC_022142	-	predicted	---
13	127837468	127837468	-	127837468	4	4	ENSSSCG00000011768	-	intron	*CCDC39*
13	127867728	127867728	-	127867728	3	3	ENSSSCG00000011768	-	ORF	*CCDC39*
13	127871267	127871267	-	127871267	4	4	ENSSSCG00000011768	-	intron	*CCDC39*
13	128122071	128122071	+	128122071	3	3	ENSSSCG00000011769	+	intron	*FXR1*
13	128123532	128123532	+	128123532	4	4	ENSSSCG00000011769	+	intron	*FXR1*
13	128168644	128168644	+	128168644	4	4	ENSSSCG00000011769	+	intron	*FXR1*
13	128174453	128174462	+	128174456	91	226	ENSSSCG00000011769	+	3'UTR	*FXR1*
13	128175101	128175108	+	128175101	10	27	ENSSSCG00000011769	+	3'UTR	*FXR1*
13	128249037	128249046	-	128249038	20	41	ENSSSCG00000011770	-	3'UTR	*DNAJC19*
13	128249483	128249492	-	128249486	28	63	ENSSSCG00000011770	-	3'UTR	*DNAJC19*
13	128569812	128569814	-	128569814	8	11	---	---	intergenic	---
13	129081541	129081541	-	129081541	3	3	---	---	intergenic	---
13	129081566	129081567	+	129081566	8	11	---	---	intergenic	---
13	129102836	129102864	+	129102836	3	9	XLOC_020841	+	predicted	---
13	129154140	129154140	+	129154140	6	6	---	---	intergenic	---
13	129189594	129189594	+	129189594	5	5	XLOC_020843	+	predicted	---
13	129671211	129671211	-	129671211	5	5	---	---	intergenic	---
13	130160182	130160183	-	130160183	10	15	XLOC_022153	-	predicted	---

13	130293539	130293539	+	130293539	3	3	ENSSSCG00000025942	+	ORF	*MCCC1*
13	130308732	130308732	+	130308732	8	8	XLOC_020847	+	predicted	---
13	130356722	130356723	+	130356722	13	18	ENSSSCG00000022783	+	intron	*DCUN1D1*
13	130372442	130372444	+	130372442	7	10	ENSSSCG00000022783	+	intron	*DCUN1D1*
13	130375167	130375188	+	130375186	221	550	ENSSSCG00000022783	+	3'UTR	*DCUN1D1*
13	130375348	130375370	+	130375368	20	54	ENSSSCG00000022783	+	3'UTR	*DCUN1D1*
13	130375473	130375474	+	130375473	5	8	ENSSSCG00000022783	+	3'UTR	*DCUN1D1*
13	130377127	130377127	+	130377127	3	3	ENSSSCG00000022783	+	3'UTR	*DCUN1D1*
13	130377834	130377848	+	130377834	6	22	ENSSSCG00000022783	+	3'UTR	*DCUN1D1*
13	130934989	130934991	-	130934989	4	7	---	---	intergenic	---
13	130987031	130987031	-	130987031	4	4	---	---	intergenic	---
13	131003741	131003741	+	131003741	3	3	XLOC_020857	+	predicted	---
13	131040226	131040235	+	131040226	8	12	ENSSSCG00000011775	+	3'UTR	*KLHL24*
13	131044958	131044994	+	131044959	5	11	XLOC_020857	+	predicted	---
13	131359183	131359183	-	131359183	3	3	ENSSSCG00000011782	-	intron	*ABCC5*
13	131532743	131532750	+	131532750	6	13	ENSSSCG00000025374	+	3'UTR	*DVL3*
13	131540240	131540240	+	131540240	3	3	ENSSSCG00000024437	+	intron	*AP2M1*
13	131543159	131543180	+	131543178	147	333	ENSSSCG00000024437	+	3'UTR	*AP2M1*
13	131543798	131543798	+	131543798	4	4	XLOC_020864	+	predicted	---
13	131552501	131552503	+	131552501	17	36	ENSSSCG00000028856	+	3'UTR	*ABCF3*
13	131552549	131552549	+	131552549	3	3	ENSSSCG00000028856	+	3'UTR	*ABCF3*
13	131552665	131552668	+	131552666	8	19	ENSSSCG00000028856	+	3'UTR	*ABCF3*
13	131564222	131564222	+	131564222	5	5	---	---	intergenic	---
13	131581813	131581814	-	131581814	28	32	ENSSSCG00000021319	-	3'UTR	*ALG3*
13	131595849	131595849	-	131595849	4	4	XLOC_022167	-	predicted	---
13	131628467	131628467	+	131628467	5	5	ENSSSCG00000023998	+	ORF	*PSMD2*

13	131639131	131639174	+	131639169	151	456	ENSSSCG00000023998	+	3'UTR	*PSMD2*
13	131653020	131653020	+	131653020	3	3	ENSSSCG00000030255	+	ORF	*EIF4G1*
13	131664844	131664846	+	131664845	18	31	ENSSSCG00000030255	+	3'UTR	*EIF4G1*
13	131665120	131665136	+	131665136	41	79	ENSSSCG00000030255	+	3'UTR	*EIF4G1*
13	131666912	131666912	+	131666912	4	4	---	---	intergenic	---
13	131674585	131674585	+	131674585	3	3	ENSSSCG00000028372	+	3'UTR	*FAM131A*
13	131745469	131745482	+	131745475	5	11	ENSSSCG00000022507	+	3'UTR	*POLR2H*
13	131745972	131745978	+	131745972	6	11	ENSSSCG00000022507	+	3'UTR	*POLR2H*
13	131750636	131750645	-	131750636	3	6	XLOC_022169	-	predicted	---
13	131767005	131767010	+	131767009	10	24	ENSSSCG00000025270	+	3'UTR	*CHRD*
13	131938418	131938418	+	131938418	3	3	XLOC_020874	+	predicted	---
13	131983288	131983289	+	131983288	17	22	---	---	intergenic	---
13	132132374	132132375	-	132132374	8	11	XLOC_022173	-	predicted	---
13	132563206	132563216	+	132563215	18	45	XLOC_020878	+	predicted	---
13	132563264	132563264	+	132563264	5	5	XLOC_020878	+	predicted	---
13	132566412	132566412	+	132566412	3	3	XLOC_020878	+	predicted	---
13	132625218	132625218	-	132625218	9	9	---	---	intergenic	---
13	132785176	132785181	-	132785176	4	7	ENSSSCG00000028398	-	intron	---
13	132868803	132868803	+	132868803	3	3	ENSSSCG00000011791	+	intron	*MAP3K13*
13	132882878	132882878	+	132882878	3	3	ENSSSCG00000011791	+	intron	*MAP3K13*
13	132907673	132907673	-	132907673	3	3	ENSSSCG00000025921	-	3'UTR	*TMEM41A*
13	132980129	132980129	+	132980129	6	6	ENSSSCG00000011794	+	intron	*SENP2*
13	133006620	133006629	+	133006628	10	17	ENSSSCG00000011794	+	3'UTR	*SENP2*
13	133022555	133022555	-	133022555	10	10	ENSSSCG00000011795	-	3'UTR	*IGF2BP2*
13	133286757	133286757	-	133286757	3	3	---	---	intergenic	---
13	133286802	133286803	+	133286802	9	12	---	---	intergenic	---

13	133406583	133406583	-	133406583	13	13	---	---	intergenic	---
13	133748215	133748215	-	133748215	5	5	XLOC_022190	-	predicted	---
13	133756728	133756728	-	133756728	3	3	XLOC_022190	-	predicted	---
13	133801104	133801105	+	133801104	29	36	ENSSSCG00000011798	+	3'UTR	*DNAJB11*
13	133801469	133801471	+	133801470	16	24	ENSSSCG00000011798	+	3'UTR	*DNAJB11*
13	133821567	133821579	-	133821573	63	165	XLOC_022192	-	predicted	---
13	133821738	133821800	+	133821738	5	14	ENSSSCG00000011799	+	ORF	*AHSG*
13	133824455	133824462	+	133824457	71	139	ENSSSCG00000011799	+	ORF	*AHSG*
13	133825197	133825197	+	133825197	3	3	ENSSSCG00000011799	+	ORF	*AHSG*
13	133826452	133826475	+	133826475	19	37	ENSSSCG00000011799	+	ORF	*AHSG*
13	133826926	133826926	+	133826926	3	3	ENSSSCG00000011799	+	intron	*AHSG*
13	133827298	133827305	+	133827305	10	13	ENSSSCG00000011799	+	ORF	*AHSG*
13	133828147	133828147	+	133828147	4	4	ENSSSCG00000011799	+	ORF	*AHSG*
13	133828411	133828411	+	133828411	3	3	ENSSSCG00000011799	+	3'UTR	*AHSG*
13	133828531	133828556	+	133828531	20	34	ENSSSCG00000011799	+	3'UTR	*AHSG*
13	133828604	133828640	+	133828637	1900	5265	XLOC_020890	+	predicted	---
13	133884126	133884135	+	133884133	142	338	ENSSSCG00000011800	+	3'UTR	*FETUB*
13	133895510	133895512	+	133895510	4	7	ENSSSCG00000011801	+	ORF	*HRG*
13	133900782	133900783	+	133900782	8	11	ENSSSCG00000011801	+	ORF	*HRG*
13	133900838	133900850	+	133900845	8	24	ENSSSCG00000011801	+	ORF	*HRG*
13	133905927	133905933	+	133905933	8	11	ENSSSCG00000011801	+	ORF	*HRG*
13	133906060	133906060	+	133906060	5	5	ENSSSCG00000011801	+	ORF	*HRG*
13	133906311	133906341	+	133906326	441	1098	ENSSSCG00000011801	+	3'UTR	*HRG*
13	133906649	133906650	+	133906650	5	8	XLOC_020892	+	predicted	---
13	133911534	133911534	+	133911534	7	7	ENSSSCG00000011802	+	ORF	*KNG1*
13	133911637	133911653	+	133911653	19	24	ENSSSCG00000011802	+	ORF	*KNG1*

13	133913574	133913574	+	133913574	3	3	ENSSSCG00000011802	+	intron	*KNG1*
13	133935990	133935991	+	133935991	26	32	ENSSSCG00000011802	+	intron	*KNG1*
13	133945795	133945795	+	133945795	11	11	ENSSSCG00000011802	+	intron	*KNG1*
13	133945919	133945920	+	133945920	20	23	ENSSSCG00000011802	+	intron	*KNG1*
13	133946422	133946422	+	133946422	4	4	ENSSSCG00000011802	+	intron	*KNG1*
13	133946539	133946539	+	133946539	3	3	ENSSSCG00000011802	+	intron	*KNG1*
13	133946844	133946844	+	133946844	4	4	ENSSSCG00000011802	+	intron	*KNG1*
13	133947388	133947388	+	133947388	5	5	ENSSSCG00000011802	+	intron	*KNG1*
13	133947427	133947451	+	133947431	245	617	ENSSSCG00000011802	+	intron	*KNG1*
13	133947672	133947681	+	133947681	4	7	ENSSSCG00000011802	+	intron	*KNG1*
13	133947748	133947760	+	133947750	86	320	ENSSSCG00000011802	+	intron	*KNG1*
13	133948330	133948332	+	133948332	10	21	ENSSSCG00000011802	+	intron	*KNG1*
13	133976541	133976541	+	133976541	5	5	ENSSSCG00000011803	+	ORF	*EIF4A2*
13	133978171	133978171	+	133978171	3	3	ENSSSCG00000011803	+	intron	*EIF4A2*
13	133979535	133979539	+	133979539	5	9	ENSSSCG00000011803	+	3'UTR	*EIF4A2*
13	133979666	133979671	+	133979669	195	352	ENSSSCG00000011803	+	3'UTR	*EIF4A2*
13	133979671	133979681	-	133979671	28	82	ENSSSCG00000011804	-	3'UTR	*RFC4*
13	134015115	134015115	-	134015115	5	5	---	---	intergenic	---
13	134015933	134015933	-	134015933	4	4	---	---	intergenic	---
13	134076770	134076770	-	134076770	5	5	---	---	intergenic	---
13	134077608	134077609	+	134077608	29	37	---	---	intergenic	---
13	134140118	134140118	+	134140118	7	7	XLOC_020898	+	predicted	---
13	134197399	134197399	-	134197399	12	12	XLOC_022194	-	predicted	---
13	134350842	134350849	+	134350842	6	14	ENSSSCG00000011806	+	intron	*MASP1*
13	134374549	134374549	+	134374549	3	3	XLOC_020899	+	predicted	---
13	134416789	134416789	+	134416789	9	9	---	---	intergenic	---

13	134436773	134436773	+	134436773	3	3	XLOC_020901	+	predicted	---
13	134436950	134436950	+	134436950	6	6	XLOC_020901	+	predicted	---
13	134451926	134451931	+	134451926	4	7	XLOC_020901	+	predicted	---
13	134660906	134660913	-	134660906	13	16	ENSSSCG00000011810	-	3'UTR	---
13	134661884	134661884	-	134661884	3	3	ENSSSCG00000011810	-	3'UTR	---
13	135532833	135532833	-	135532833	4	4	XLOC_022206	-	predicted	---
13	135882871	135882871	+	135882871	3	3	---	---	intergenic	---
13	136665661	136665661	-	136665661	3	3	ENSSSCG00000011813	-	3'UTR	*P3H2*
13	137364118	137364121	+	137364121	14	26	ENSSSCG00000021206	+	intron	*IL1RAP*
13	137429188	137429188	-	137429188	7	7	---	---	intergenic	---
13	138080708	138080708	+	138080708	3	3	XLOC_020920	+	predicted	---
13	138086597	138086597	+	138086597	9	9	XLOC_020920	+	predicted	---
13	138088100	138088100	+	138088100	3	3	---	---	intergenic	---
13	138500613	138500613	-	138500613	3	3	---	---	intergenic	---
13	139332875	139332875	+	139332875	6	6	---	---	intergenic	---
13	139544439	139544440	+	139544439	4	7	---	---	intergenic	---
13	139825588	139825588	+	139825588	12	12	ENSSSCG00000011820	+	ORF	*HRASLS*
13	139825746	139825757	+	139825752	95	215	XLOC_020926	+	predicted	---
13	140375853	140375854	-	140375854	9	13	---	---	intergenic	---
13	140465637	140465637	+	140465637	9	9	---	---	intergenic	---
13	140545953	140545953	-	140545953	3	3	---	---	intergenic	---
[truncated: 400,854 more chars]
